# The TolC and Lipopolysaccharide-Specific *Escherichia coli* Bacteriophage TLS—the *Tlsvirus* Archetype Virus

**DOI:** 10.1089/phage.2023.0041

**Published:** 2024-09-16

**Authors:** Gregory J. German, James V. DeGiulio, Jolene Ramsey, Andrew M. Kropinski, Rajeev Misra

**Affiliations:** ^1^ St. Joseph’s Health Centre, Unity Health Toronto, Toronto, Canada.; ^2^ Department of Laboratory Medicine & Pathobiology, University of Toronto, Toronto, Canada.; ^3^ Lathrop GPM, Chicago, IL USA.; ^4^ Texas A&M University, Biology Department, College Station, TX USA.; ^5^ Department of Pathobiology, Ontario Veterinary College, University of Guelph, Guelph, Canada.; ^6^ School of Life Sciences, Arizona State University, Tempe, Arizona, USA.

**Keywords:** genomics, bacteriophages, *Drexlerviridae*, *Tempevirinae*, host-receptors, proteome

## Abstract

**Introduction:**

TLS is a virulent bacteriophage of *Escherichia coli* that utilizes TolC and lipopolysaccharide as its cell surface receptors.

**Methods:**

The genome was reannotated using the latest online resources and compared to other T1-like phages.

**Results:**

The TLS genome consists of 49,902 base pairs, encoding 86 coding sequences that display considerable sequence similarity with the T1 phage genome. It also contains 18 intergenic 21-base long repeats, each of them upstream of a predicted start codon and in the direction of transcription. Data revealed that DNA packaging occurs through the *pac* site-mediated headful mechanism.

**Conclusions:**

Based on sequence analysis of its genome, TLS belongs to the *Drexlerviridae* family and represents the type member of the *Tlsvirus* genus.

## Background

*Escherichia* phage T1^1^ was one of the set of viruses identified by Max Delbrück for enhanced study by the “Phage Group,”^2^ but it was the least studied phage in this group. This was probably due to the fact that T1 has a history of lysing *E.coli* cultures and is resistant to drying.^3,4^ An early identified T1-like virus was TLS (TolC and Lipopolysaccharide Specific) because it was the only known phage at the time to utilize TolC, an antibiotic and toxin secretor channel protein, as a coreceptor.^5^ TLS is the archetypal and founding member of the genus *Tlsvirus* that is one of five genera of the subfamily *Tempevirinae*, which is one of at least four subfamilies in the family *Drexlerviridae*. This taxon of T1-like phages was named in honor of the American phage T1 pioneering researcher Henry Drexler (b. 1927, d. 2020). This work provides the updated annotation and characterization of the phage TLS genome that is appropriate for an archetypal siphovirus.

## Materials and Methods

### Bacterial strains and culture conditions

Bacteriophage TLS was obtained from Carl Schnaitman (Arizona State University) that was mistakenly named U3, whereas phage T1 was provided from the Félix d’Hérelle Reference Center for Bacterial Viruses, Université Laval (Québec, Canada). The host was *E. coli* MC4100 (F^-^ *araD139* Δ[*argFlac*] *U139 rpsL150 relA1 flbB5301 ptsF25 deoC1 thi-1 rbsR*) (6). Transformation competent cells used were either JM109 (*endA1 gyrA96 thi hsdR17 supE44 relA1* Δ(*lac-proAB*) *recA1* F'[*traD36 proAB*^+^ *lacIq lacZ*Δ*M15*]) from CGSC or NovaBlue Singles (*endA1 gyrA96 hsdR17*(r_K12_^−^ − m_K12_^−^) *supE44 thi recA1 relA1 lac* F′[*proAB*^+^ *lacI*^q^ M15::Tn*10*]) from Novagen (MilliporeSigma). All bacterial strains were grown at 37°C in Lysogeny Broth or Lysogeny Broth agar.

Large-scale yields of TLS were obtained by adding 10^10^ pfu to 500 mL of an early log phase culture of MC4100 in Lysogeny Broth supplemented with 5 mM CaCl_2_ and letting it grow with rapid shaking for 8–12 h at 37°C. Cultures were then treated with chloroform (2% final concentration), and unlysed cells were removed by centrifugation. The phage-containing supernatants were pooled and were further purified by polyethylene glycol precipitation followed by CsCl density gradients to a concentration of 2 × 10^14^ pfu/mL.^6^

### Electron microscopy

All samples were stained with 2% uranyl acetate and examined using a Philips CM-12S scanning transmission electron microscope in bright-field TEM mode at 100 kV using a 30 nm objective aperture. Digital images were captured using a Gatan model 689 retractable slow-scan camera and processed using Gatan DigitalMicrograph 2.5 software. Phage dimensions were measured on by averaging phages from an electron micrograph at 150,000× magnification.

For thin sectioning, TLS was mixed with susceptible cells in soft agar overlays supported on nitrocellulose and allowed to grow overnight. Portions of phage plaques were excised and fixed in 0.1 M cacodylate buffer with 2% glutaraldehyde for 2 h at 4°C. After washing in buffer, specimens were treated with 2% OsO_4_ for 2 h at 4°C. Next, after three 20 min washes in buffer, specimens were *en bloc* stained in 0.05% aqueous uranyl acetate for 48 h at 4°C. Specimens were dehydrated through progressively higher concentrations of ethanol up to 100% and washed finally with 100% acetone. They were then slowly impregnated with Spurr’s resin in a stepwise manner over a 2-day period. After thin sectioning, samples were poststained with 2% uranyl acetate in 50% methanol for 15 min and then with Reynolds’ lead citrate for 5 min (John Wertz, personal communication).

### DNA extraction, enzymatic digestion, and cloning

Virion genomic DNA was extracted using SDS-EDTA-protease K treatment followed by multiple phenol–chloroform extractions.^6^ Phage and DNA preparations were dialyzed using the Slide-A-Lyzer MINI Dialysis Kit (Pierce Chemical Co, Dallas, TX) against either 10 mM Tris-HCl pH 7.0, 1 mM MgCl_2_ for phage or 50 mM Tris-HCl pH 8.0 for DNA. All restriction enzymes were obtained from Promega Corp (Madison, WI) or New England Biolabs (Ipswich, MA) and used as per manufacturer’s instructions.

TLS DNA was randomly sheared using DNase I at 0.044 units/mL in a manganese (II) buffer to generate near blunt to blunt ended fragments.^7^ The digestion samples were run in a TAE agarose gel, and fragments ranging from 500 to 2000 base pair were excised and purified with the Gel Extraction Kit from Qiagen (Valencia, CA). Fragments were then polished with the Perfectly Blunt Kit Cloning Kit from Novagen (Madison, WI) and were ligated to the pST-1Blue phosphatase treated linear blue/white screening vector.

### Genome sequencing and assembly

Recombinant plasmid DNA was recovered and subjected to Sanger sequencing in the DNA Laboratory, School of Life Sciences, Arizona State University using universal vector primers.^8^ The resulting sequences were trimmed of poor quality and vector sequences and assembled using Sequencher 5.0 software (Gene Codes, Ann Arbor, MI) into contigs. After shotgun cloning reached about a 75% level of redundancy, multiple techniques, including polymerase chain reaction amplification of TLS DNA, were used to fill in gaps.

### Annotation techniques

The TLS genome was recently reannotated initially using DFAST^9,10^ at https://dfast.ddbj.nig.ac.jp/dfc/ with a minimal coding sequence (CDS) length of 75. The resulting gbk file was imported into Kodon 3.0 (Applied Maths, Inc., Austin, TX, USA; now bioMérieux SA) and visually proofread. The updated gbk file was submitted to the Genome2D webserver^11^ to generate a FASTA-formatted proteins file. The protein molecular weights and isoelectric points were calculated using Isoelectric Point Calculator 2.0^12^ at https://ipc2.mimuw.edu.pl/. Transmembrane domains were discovered using Deep^TM^HMM,^13^ whereas Pfam^14^ and NCBI-CDD^15^ motifs were discovered using the GenomeNet Database Resource “MOTIF Search” at https://www.genome.jp/tools/motif/ with an E-value cutoff of 0.00001.

Possible rho-independent terminators were screened for using ARNold at http://rssf.i2bc.paris-saclay.fr/toolbox/arnold/index.php^16^ and as a backup we also used Transcription Terminator Prediction at Genome2D (http://genome2d.molgenrug.nl/g2d_pepper_transterm.php).^11^ In each case the initial ΔG values were recalculated using mfold.^17^ Potential host RNA polymerase-dependent promoter sites were identified by visual inspection of the sequence in Kodon for sequence similarity to TTGACA[N15-19]TATAAT^18^ allowing for a 2 nt mismatch. In addition, ProPr: Prokaryote Promoter Prediction v2.0^19^ at http://ppp.molgenrug.nl/ was used with the results being screened for high probability sequences using SAPPHIRE.CNN^20^ at https://sapphire.biw.kuleuven.be/index.php.

Repeats in the genome sequence were identified using REPuter^21^ and the Phage In silico Regulatory Elements program (PHIRE).^22^ A WebLogo for the direct repeats found in the TLS sequence was constructed using the online tool at http://weblogo.berkeley.edu/logo.cgi.^23^

### Comparative genomics and proteomics

VIRIDIC^24^ was used to generate a heatmap illustrating the DNA sequence similarity between phage genomes with the similarity results file exported to Microsoft Excel. In addition, DiGAlign (https://www.genome.jp/digalign) with output in BLASTN and TBLASTX format and clinker, which is part of Comparative Gene Cluster Analysis Toolbox,^25^ at https://cagecat.bioinformatics.nl were used to generate gene cluster comparison figures.

The *Tlsvirus* and *Tunavirus* tail fiber protein sequences were aligned using AlphaFold2^26,27^ at https://neurosnap.ai/service/AlphaFold2, COBALT,^28^ FALCON2,^25^ Phyre,^2,29^ TopModel,^30^ and trRosetta.^31,32^ The structures were visualized using Mol*3D Viewer^33^ at https://www.rcsb.org/3d-view.

### Proteomic analysis

For whole virion protein analysis phage were solubilized in SDS sample buffer (final concentration: 1% SDS, 50 mM Tris-HCl pH 7.5, 5% 2-mercaptoethanol, 5% glycerol) by boiling for 5 min. The proteins (1 × 10^10^ pfu per lane) were resolved by denaturing 10% polyacrylamide gel electrophoresis along with Bio-Rad’s Precision Plus Protein Unstained Standards and visualized by staining with Coomassie brilliant blue (Bio-Rad Laboratories, Hercules, CA, USA). The major capsid protein was excised from the gel and subjected to Edman degradation in the Proteomics Core Laboratory (Department of Chemistry and Biochemistry, Arizona State University) giving rise to the N-terminal amino acid sequence.

### Phylogenetic analysis

The large subunit terminase (TerL) proteins of TLS-related phages were analyzed using NGPhylogeny.fr^34^ with the tree exported to iTol.^35^ The TerL protein from *Escherichia* phage JakobBernoulli, a member of the *Hanrivervirus* genus, was used as the outlier.

### GenBank submission

The reannotated TLS genome is available under GenBank accession number AY308796.

## Results

### Electron microscopy

**FIG. 1.**
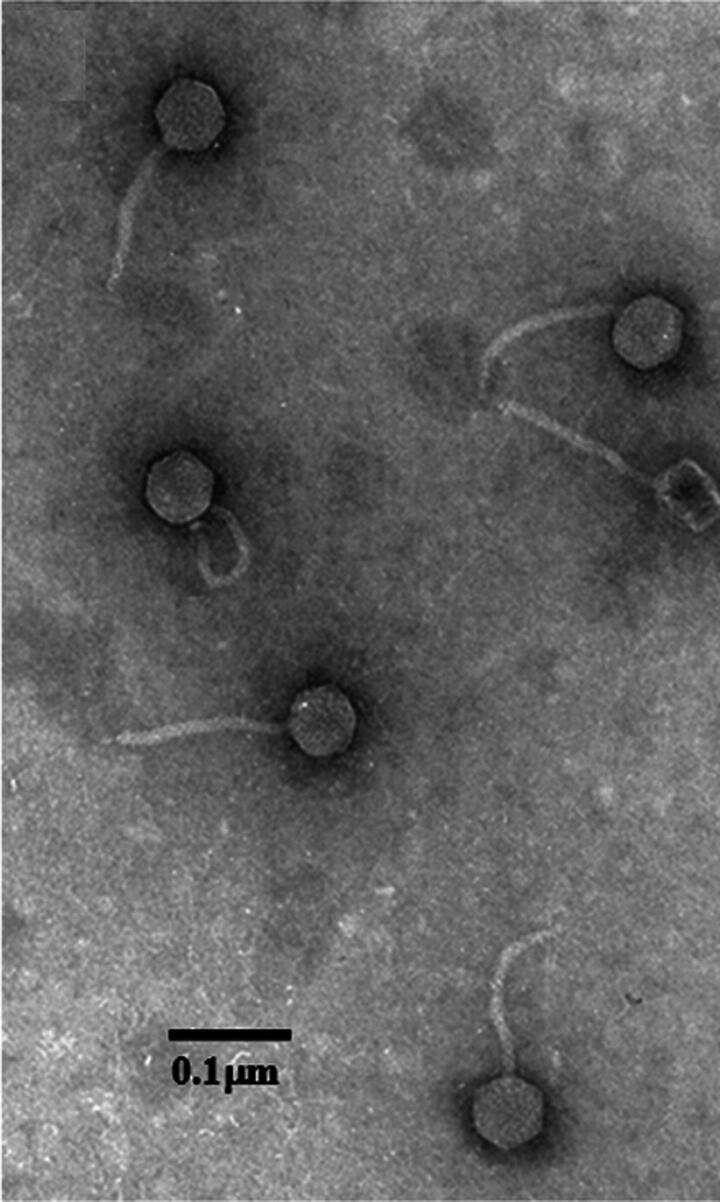
Bacteriophage TLS particles negatively stained with 2% uranyl acetate. The scale bar represents 100 nm.

TLS has a head diameter of 61.4 nm, a tail length of 155 nm, and a tail diameter of 9.8 nm. This matches well with what has been characterized for T1 (Fig. 1).^36^

**FIG. 2.**
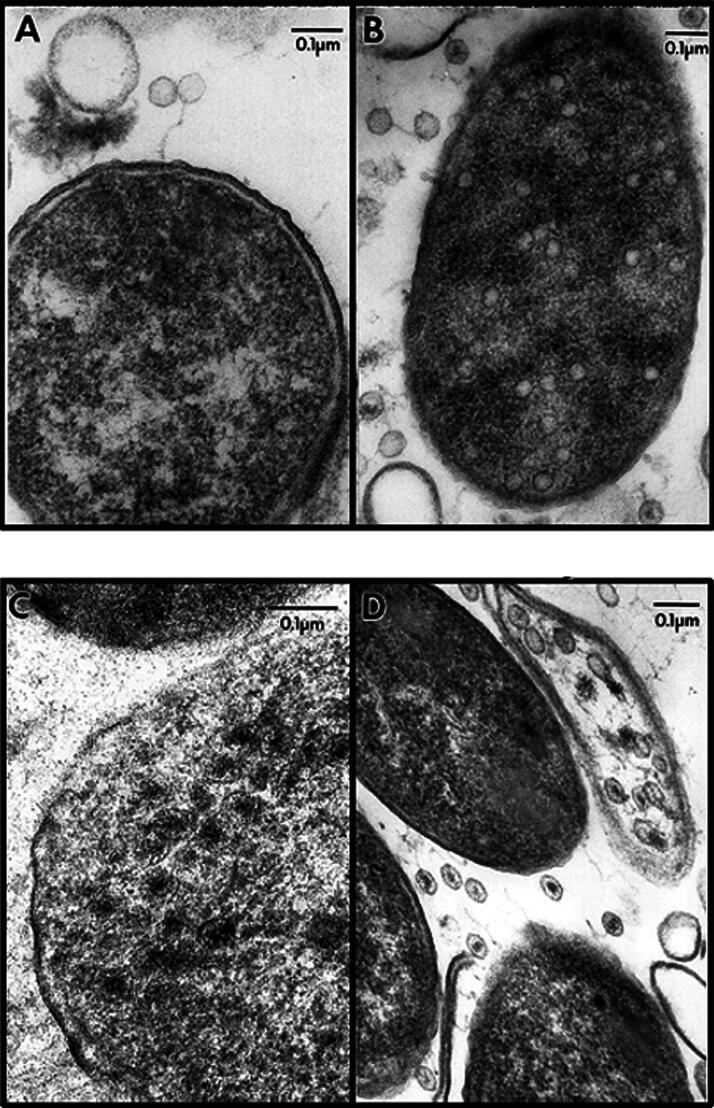
Negatively stained cross-sections of *E. coli* infected with phage TLS. **A**. adsorption/injection phase showing empty particle adsorbed to cells; **B**. capsid morphogenesis revealing empty proheads; **C**. capsids containing DNA; and, **D**. lysis.

In order to investigate the life cycle of TLS, we developed a novel approach involving examination of phage plaque edge by thin sectioning, heavy metal staining, and electron microscopy. In Figure 2 (A–D) we could recognize the following four distinct stages in the development of TLS. First, attachment and DNA injection as typified by the presence of empty capsids on the cell surface. Second, procapsid formation occurred in a nonorganized or localized manner. Third, the *de novo* synthesized DNA is packaged creating full capsids at high concentration. Finally, lysis occurred releasing new phage particles.

### Basic characteristics of the TLS genome and its packaging

The TLS phage has a terminally redundant genome of 49,902 unique base pairs with a GC content of 42.68%. Like the genome of coliphage T1, TLS phage DNA contains over 200 GATC sequences that are adenine methylated, as determined by sensitivity/resistance to the GATC-specific restriction enzymes, Sau3A, MboI, and DpnI.^37^

In addition, as with T1, TLS DNA appears to undergo a headful packaging mechanism (reviewed in References^38,39^) that creates three or four variants of virus particles, each with a genome that has a slightly modified linear gene order (i.e., partial circular permutation) and different terminally redundant ends. Diagnostic for headful packaging is the presence of submolar,^38,40^ concentrations of a restriction fragment encompassing the *pac* site present in only the first variant of concatemeric DNA comprising three to four genomic lengths (Supplementary Fig. S1).

The length of the terminal repeat and the site for the first packaging cut (called the *pac* site) were determined by analyzing HindIII-restricted virion DNA. HindIII digestion of the TLS genome creates a sharp but submolar band of approximately 6 kb belonging to the *pac*–HindIII fragment of the genome (Supplementary Fig. S1). In addition, there are two heterogenous DNA populations of approximately 5 kb and 4 kb in length. Extraction of these populations and digestion with a second restriction enzyme, EcoRV, yielded sharp homogenous DNA fragments that were also present when the 6 kb fragment was restricted (data not shown), thus demonstrating that DNA molecules producing the diffuse heterogeneous populations are variants of each other. An interpretation of these results is that each packaging event leaves a genome with an additional 1 kb of replicated DNA and changes the starting point of the next virion genome by 1 kb. To further narrow down the *pac* site location, the 6 kb *pac*–HindIII fragment was digested with multiple restriction enzymes. From this, the *pac* site was determined to be approximately 5,882 bp from the HindIII site. The final *pac* sequence assignment was deduced by sequence similarity to the P22 *pac*, which in 2002 was proposed to be AAGATTTATCTG.^41^ In 2013 this was extended to GAAGATTTATCTGAAGTCGTTA.^42^ An EcoRV fragment of about 2.7 kb was generated from the pac–HindIII DNA, and this was used to narrow the search for the pac site. Analysis of the region revealed the sequence AGATTT for TLS, which closely resembles the P22 consensus sequence.

### Transcriptional elements

**FIG. 3.**
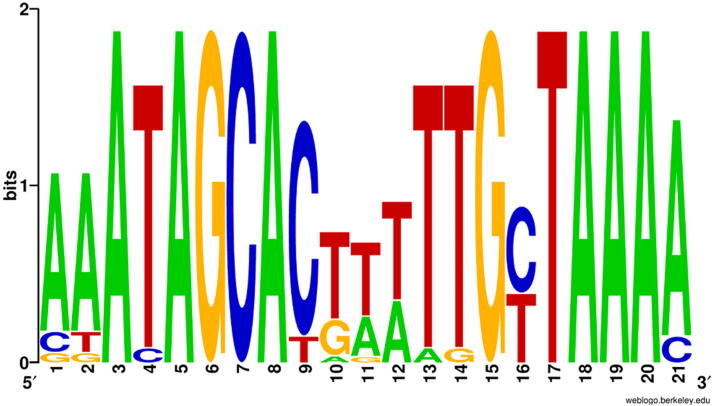
WebLogo of the direct repeats found in the sequence of TLS.

Since our analysis of the TLS genome revealed no evidence for a gene encoding an RNA polymerase or sigma subunit, TLS is expected to use the host transcriptional apparatus. Conservative *in silico* analysis for promoter-like sequences using a variety of tools unexpectedly revealed only three intergenic σ^70^-like promoters (Supplementary Table S1). With the aid of REPuter and PHIRE a 21 nt direct repeat element was found in 21 locations within the TLS genome. Ignoring repeats found wholly within genes, the other 18 repeats are listed in Supplementary Table S1. The consensus sequence is aaATAGCACnnnTTGnTAAAA with its WebLogo presented in Figure 3. The TLS repeats are found in the direction of transcription and are approximately 76 bases upstream from predicted translation start codons. All these repeats have maximum conservation near their 5′ (-ATAGCAC-) and 3′ ends (-TAAAA-). The repeats can be further subdivided into three groups, labeled “A”, “T”, and “G”, based on the intervening consensus sequence AAA, TTT, and GAA, respectively (Supplementary Table S1). Similar repeats are also found in the genomes of phages T1^1^, Rtp, and vB_EcoS_Rogue1 (unpublished observations). The presence of the repeats in the direction of transcription and between genes is reminiscent of the inhibitory transcriptional elements called “stoperators” discovered in the mycobacteriophage L5.^43^ Alternatively, they could represent unidentified enhancer-binding sequences. Finally, these regions resemble the “UP enhancer elements” that are found at 45 bases upstream of the start of transcription and significantly increase the activity of T5^44^ and T4 promoters.^45^

The phage T1 late region appears to be made up of a series of transcriptional modules (transcriptons) flanked by rho-independent terminators and containing RpoD-dependent promoters and perhaps enhancers.^1^ In the case of phage TLS 21 putative rho-independent terminators were identified using ARNold or online software at Genome2D. In every case these were located in intergenic regions. We have used a far more rigorous approach to the *in silico* analysis of promoters and cannot definitively state that TLS contains transcriptons. The only clear situation is P51-gp51-DR9-gp52-T52 (Supplementary Table S1).

### Characterization of the TLS genes

Eighty-six CDSs ranging from 27 (Gp05) to 1259 (Gp50) codons in length were identified using the gene prediction program DFAST, the majority of these beginning with ATG. Most of the genome, including the typical late structural genes, is transcribed in one direction. In the following sections we will discuss the gene products (Supplementary Table S2).

### Nucleotide metabolism and DNA replication

One of the characteristics of coliphages T1 and TLS is that the infection cycle results in enzymatic degradation of the host genome and reutilization of the nucleotides for phage DNA synthesis.^46,47^ We have thus far been unable to identify which of the presumptive early gene products of either phage participate in this feature of the replicative cycle.

TLS Gp09 and Gp14 encode polynucleotide kinase and deoxynucleoside monophosphate kinase, respectively, which can terminally phosphorylate oligonucleotides and monophosphates.^48^ In the latter protein at the amino terminal is the conserved domain “GXXGXGK,” which corresponds to the active site of the kinase.^49^

Unlike the other T-type phages, TLS and T1 do not contain any apparent DNA polymerase genes and most likely utilize host enzymes for DNA replication. A primase (TLS Gp56) and a helicase (TLS Gp58) are located in the replication region of the genome, which might initiate replication, much like the primase (GpO) and helicase (GpP) of the temperate phage λ. The gene organization of these proteins in TLS is unique compared to other phages, because they are located in the opposite orientation with respect to each other. Other potential gene products that may be involved in DNA replication or recombination are exodeoxyribonuclease (Gp51), recombinase (Gp53), single-stranded DNA binding protein (Gp54), Holiday junction recombinase (Gp59), and proof-reading exonuclease (gp66).

The origin of replication (*OriC*) in *E. coli* is characterized as possessing 5′-GATC-3′ sites,^50^ AT-rich repeats, and sites for binding the initiator protein DnaA (consensus: TTA/TTNCACA)^51^ and IHF (consensus: GTTGnnGnnnWnnAAAnnCRnnnnTTTnWnAACnnA).^52^ The latter two proteins induce bending of the sequence.^53^ The origin of replication for phage λ is found within the helicase gene as a set of four tandem repeats (iterons). This is also true for Shiga toxin-converting bacteriophages,^54^ where these sites function as binding sites for replication O protein. No such repeats are found inside TLS gp58 or anywhere else in the genome. A further characteristic of many replication origins is the presence of a high number of GATC sequences, which are often methylated by Dam. For instance, *E. coli oriC* contains 11 GATC sequences in a span of 256 bp, where statistically only one such tetranucleotide is expected. TLS contains 254 GATC sequences; the greatest concentration of these is found in the helicase gene, including three adjacent sites near the 3′-end of the gene.

### Head and tail morphogenesis

**FIG. 4.**
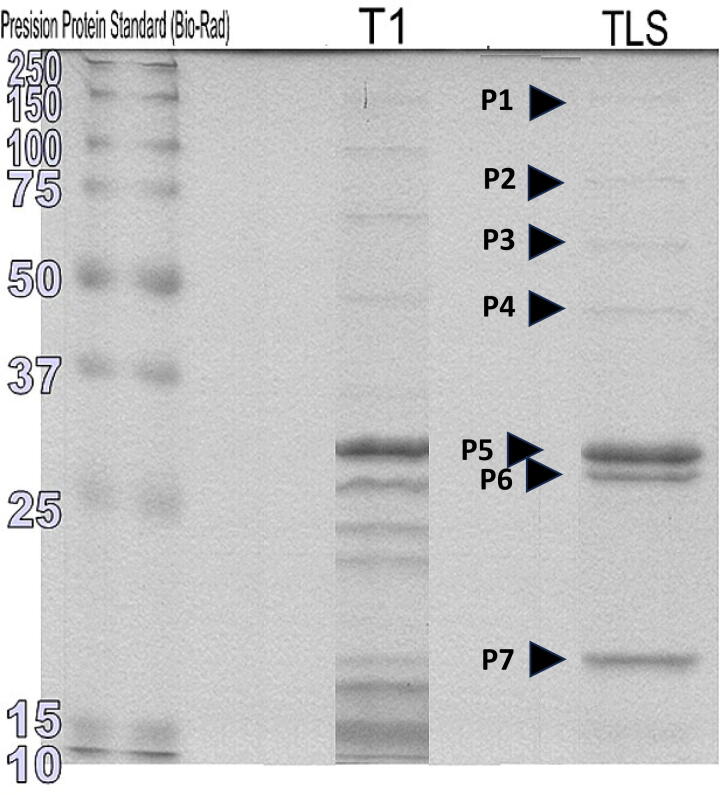
SDS-PAGE resolution of the structural proteins of phages T1 and TLS as revealed by Coomassie brilliant blue staining. The protein molecular weight marker is shown in the left lane.

SDS-PAGE reveals a total of seven distinct protein bands (Fig. 4). The most abundant virion protein (P5) exhibited a molecular weight of approximately 29 kDa, which is significantly less than the predicted mass (35.9 kDa) for the major capsid protein (MCP; gp36). N-terminal sequencing of this protein revealed a sequence of “ASDMGIW.” The smaller size of the head subunit suggests that it has been cleaved removing the putative scaffolding portion of the protein. This finding is congruent with the T1 data^1,55^ and typified by the protease processing in phage HK97 development. Proteomic analysis of the MCPs of coliphage vB_EcoS_Rogue1^56^ reveals that the N-terminus is Ala;^43^ and Clustal alignment of the TLS and Rogue1 MCPs reveal the conserved triplet AYE from which we surmise that, in the case of TLS, the prohead protease removes 45 amino acid residues (5.195 kDa) from the N-terminus.

It is likely that P6 (27.5 kDa) represents the major tail protein (Gp42, 24.3 kDa) and that P7 (18.5 kDa) may represent the capsid decoration protein (Gp34, 16.6 kDa). HHpred analysis^57^ of the latter reveals ≥95.2% probability of sequence relatedness to proteins from four phages—Φ29, XM1, TW1, and Pam3. The lowest E-value is to a capsid asymmetrical subunit (8HDT) from a marine myovirus.^58^ We believe that Gp34 corresponds to a capsid decoration protein like λ GpD rather than a minor capsid or capsid fiber protein. Furthermore, our analyses would suggest that P1 is the tail fiber protein (Gp50) and P4 is the portal protein (Gp31). In GenBank we have described Gp55 as a hypothetical protein, but there is a 78 kDa structural protein. Analysis of the sequence of this protein using HHpred suggests a structural relationship to bacteriophage T5 L-shaped tail fiber (RCSB PDB 4UW8; probability 98.07, E-value 0.00001).^59,60^

### Receptor binding

When this research was initiated phage TLS was the only virus known to utilize the TolC receptor for adsorption. Viruses with known TolC specificity now include members of the *Autographiviridae* such as *Vibrio* phage VP3^61^ and *Salmonella* phages ST29 and ST35,^62^ as well as *Tempevirinae* members—*Salmonella* phage GSP032,^63^ and coliphages U136B,^64,65^ LL5,^66,67^ and TLS.^68^ By comparison T1 binds to the ferric heme uptake protein (FhuA, TonA^69^) a property it shares with phi2013,^70^ *Hanrivervirus* JLBYU41,^71^ *Dhillonviruses* JLBYU37 and JLBYU60,^71^ *Aguilavirus* mEp213,^72^ and *Tequintavirus* T5.^73^ The average TLS-like fiber consists of 1258 amino acid residues, whereas the T1-like fibers are shorter at 1168 residues. Their alignment using COBALT (Supplementary Fig. S2) reveals that the N-terminal region shows considerable sequence similarity and presumably represents the portion of the mature protein, which associates with the tail structure, whereas the variable C-terminal domain is involved in receptor binding. The protein 3D modeling programs Phyre2 indicated a close structural relationship between 327 residues near the N-terminus of TLS and T1 Gp50 and a baseplate protein of the gene transfer agent (6TEH).

**FIG. 5.**
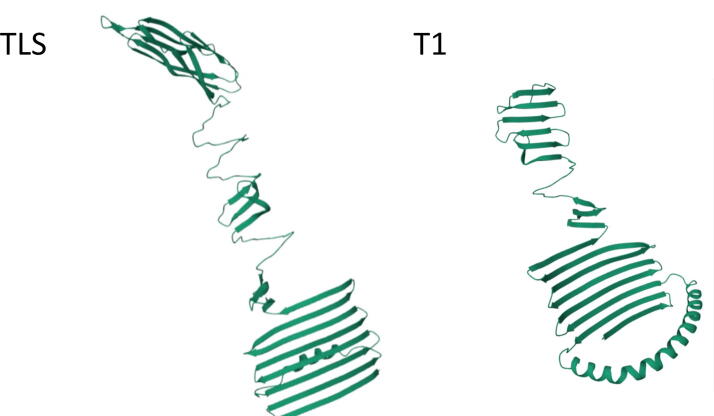
AlphaFold2 proposed 3D structures of the C-terminal sequence of the receptor-binding proteins of TLS and T1 as visualized using Mol*3D Viewer (N.B. the complete 3D structures for these proteins are provided as Supplementary Data 1 and 2).

One critical question to be answered is which amino acid residues are important for host specificity. To gain some insights into this and to potentially stimulate further research, the full length Gp55 proteins of TLS and T1 were modeled using AlphaFold2.^26,27^ The resulting pdb files were trimmed to Tyr796 (T1) and Tyr800 (TLS) and examined using Mol*3D Viewer.^33^ The results (Fig. 5) reveal considerable theoretical structural differences between the C-termini of the two phages, which presumably influence receptor recognition. Although both are rich in β-strands, the upstream region of TLS Gp55 is uniquely rich in α-helices. Each protein possesses an unstructured C-terminus.

## Lysis

Lysis of the cell to release phage progeny is carried out by three classes of lysis proteins. In TLS, these are encoded in a single cluster of genes with high similarity to the syntenic T1 cluster. Lysis is initiated by the holins, which form holes in the cell membrane.^74,75^ Their presence effectively ends all cellular processes that depend on ATP generation and serve to release the next lysis protein. The holin protein in TLS, Gp67, and phage T1 holin protein, T1 Gp67, share 55% amino acid identity. Both have two putative transmembrane segments and are therefore proposed to be class II pinholins. The TLS holin gene has two putative translational start sites (M_1_RDFLLM_2_) with separate RBS sites and an inverted repeat upstream, invoking a potential for a dual-start regulatory mechanism.^76^ Downstream of the holin gene is a signal-anchor-release (SAR) endolysin, Gp68, a cell wall degrading enzyme that degrades the peptidoglycan in the periplasm. Gp68 would be classified as a muramidase that breaks the 1,4 glycosidic bond between *N*-acetylmuramic acid and *N*-acetylglucosamine residues of peptidoglycan. Furthermore, TLS Gp68 would belong to the T4L subgroup because both share similar catalytic residues.^77,78^ It shares 61% amino acid identity with T1 Gp12, also a predicted SAR endolysin. The last gene in the lysis cluster is a unimolecular spanin, Gp69, with 57% similarity to the characterized T1 spanin, Gp11.^79,80^ Both contain identical lipobox signals near the N-terminus that will be lipoylated and anchored in the outer membrane as predicted by LipoP.^81^ A transmembrane domain segment recognized by TMHMM from residues 101–123 will be anchored in the inner membrane.^82^ The periplasmic region of Gp69 is dominated by predicted beta-sheet folds in the periplasmic region, similar to T1 Gp11, with an additional disordered region.^83^ This, therefore, classifies it as part of the T1 u-spanin family.^84^

### Comparative genomics and proteomics

**FIG. 6.**
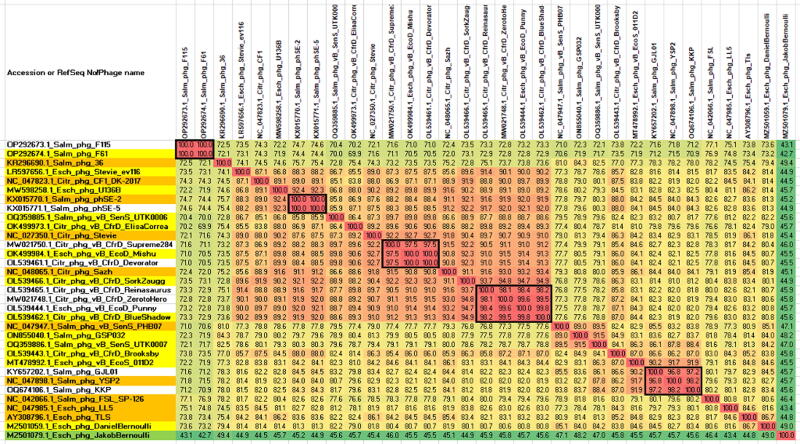
VIRIDIC heatmap showing the DNA sequence similarity for all recent members of the *Tlsvirus* genus, with *Escherichia* phage JakobBernoulli a member of the *Hanrivervirus* as the outlier. The names highlighted in gold represent current species, those in yellow, newly proposed species, and those in white represent strains of new or previously described species. Esch_phg_, *Escherichia* phage; Salm_phg_, *Salmonella* phage; Citr_phg_, *Citrobacter* phage.

**FIG. 7.**
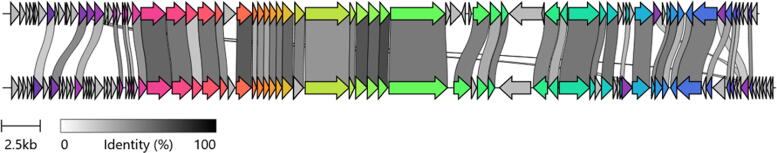
Clinker sequence comparison of the proteomes of TLS (bottom) and T1 (top).

The genus *Tlsvirus* currently consists of ten species of viruses, the exemplars of which infect *Citrobacter, Escherichia*, and *Salmonella* strains. In 2023, a proposal, based upon VIRIDIC analysis (Fig. 6) of related species in GenBank, was presented to ICTV (2023.065B.N.v1.Tempevirinae_4ng_33ns) to increase the number of species to 23. In addition, nine new strains were identified. This proposal has now become official taxonomy. The assignment to this genus is also supported by phylogenetic analysis of the large subunit terminase proteins, which clearly indicate that they are monophyletic (Supplementary Fig. S3). A comparison to TLS and T1 at the protein level is shown in Figure 7.

## Discussion

**FIG. 8.**
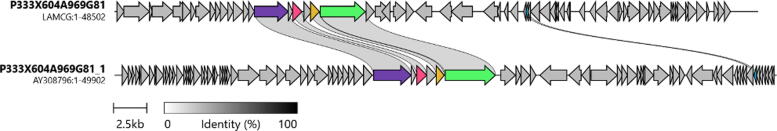
Clinker sequence comparison of phage λ (top) and TLS (bottom) reveals the conserved tail tip assembly module.

One of the unique properties of the T1 and TLS genomes is that they both contain a module which is most closely related to the archetype temperate virus, coliphage lambda, lending credence to the opinion that all phages are mosaic in nature. In 2003 one of us noted that part of the T1 genome was closely related to that of coliphages N15 (*Ravinvirus*) and λ (*Lambdavirus*).^85^ We have revisited this and can now state that T1 and TLS possess a module, which extends from the tail tape measure protein (λ GpH) to host specificity protein (GpJ). In the genome of phage lambda, the baseplate-tail spike assembly genes are arranged *H-M-L-K-I-J*, which is identical to that in TLS (Fig. 8).

To the upcoming generation of phage scientists, we suggest the following unanswered questions about T1-like phages: (a) How do they shut off host syntheses? (b) How do they degrade the host genome? (c) What is the function of the intergenic 21-base long repeat sequenced? (d) Can one model the docking between the tail fiber and its cellular receptors?

## Conclusions

Our ability to sequence bacteriophage genomes has led to initiatives like SEA-Phages, Phage Hunters Integrating Science & Education, and Citizen Phage Library (https://www.citizenphage.com/), as well as a complete reassessment of phage taxonomy.^86,87^ As it relates to this work, recently 22 T1-like bacteriophages were identified and sequenced as part of an educational endeavor.^88^ The ability to go into greater detail for a type virus is a hallmark of this work, including 3D structural analysis, headful packaging, methylation, and electron microscopy. While the initial TLS genome sequence was posted almost 20 years ago, this reanalysis highlighted the broad mosaic nature of phage genomes and placed further attention on the host receptor components and clears up previous loose associations and mischaracterizations. Furthermore, as phage therapy^89^ is becoming a more mainstream realization, considerations on phage therapeutics place more importance on receptors for binding (and with that the evolutionary trade-offs), stability (as was known for T1), and minimum packaging capsid size for engineering and cell-free production.^90^
