# Supplementary material for: The TolC and Lipopolysaccharide-Specific Escherichia coli Bacteriophage TLS—the Tlsvirus Archetype Virus
Source: Phage (New Rochelle). 2024 Sep 16;5(3):173–83. doi: 10.1089/phage.2023.0041 (PMC11447400; doi:10.1089/phage.2023.0041)
Supplement: Supplementary Figure S1 [file phage.2023.0041_suppl_figs1.pdf]

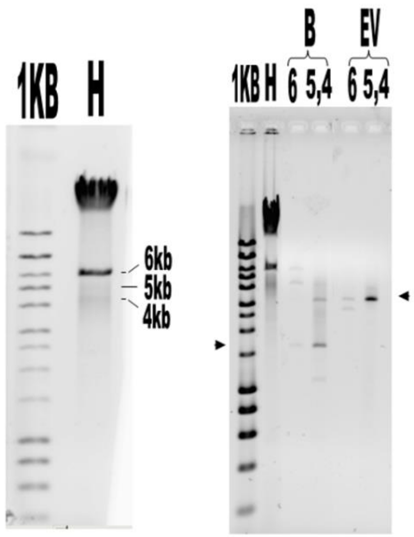

Supplementary Figure 1: TLS packaging Site (pac) determination. Left: TLS HindII-treated DNA creates three bands below 7 kb. The 6kb band is created by the pac site cut and HindIII cut, while the 5 kb and 4 kb are created by a headful cut and the Hind II cut. Right DNA collected by gel excision from the HindIII treatment were then subjected with a second enzyme- (B)spHI or (E)coRV. Arrows indicated found in each of the BspHI or EcoRV preparations.
