# Supplementary material for: The TolC and Lipopolysaccharide-Specific Escherichia coli Bacteriophage TLS—the Tlsvirus Archetype Virus
Source: Phage (New Rochelle). 2024 Sep 16;5(3):173–83. doi: 10.1089/phage.2023.0041 (PMC11447400; doi:10.1089/phage.2023.0041)
Supplement: Supplementary Figure S2 [file phage.2023.0041_suppl_figs2.pdf]

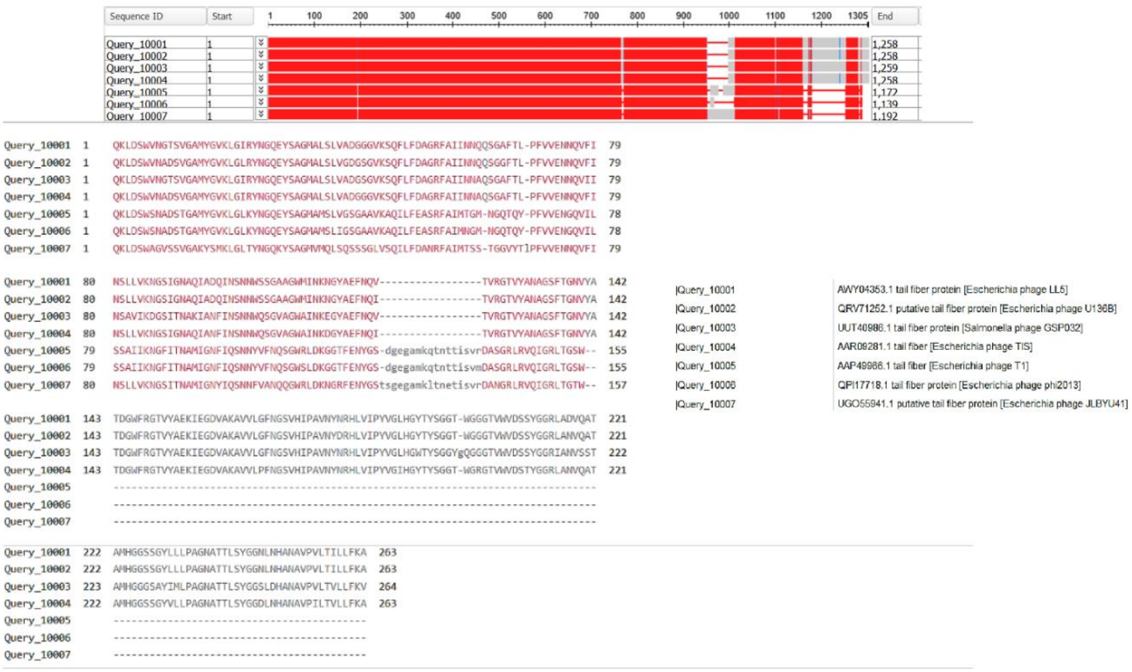


**Supplementary Figure 2:** COBALT alignment of the tail fiber proteins of T1-like and TLS-like phages with details on the alignment of the terminal amino acid residues which reveals the differences between the TolC and FhuA-specific viruses.
