# Supplementary material for: The TolC and Lipopolysaccharide-Specific Escherichia coli Bacteriophage TLS—the Tlsvirus Archetype Virus
Source: Phage (New Rochelle). 2024 Sep 16;5(3):173–83. doi: 10.1089/phage.2023.0041 (PMC11447400; doi:10.1089/phage.2023.0041)
Supplement: Supplementary Figure S3 [file phage.2023.0041_suppl_figs3.pdf]

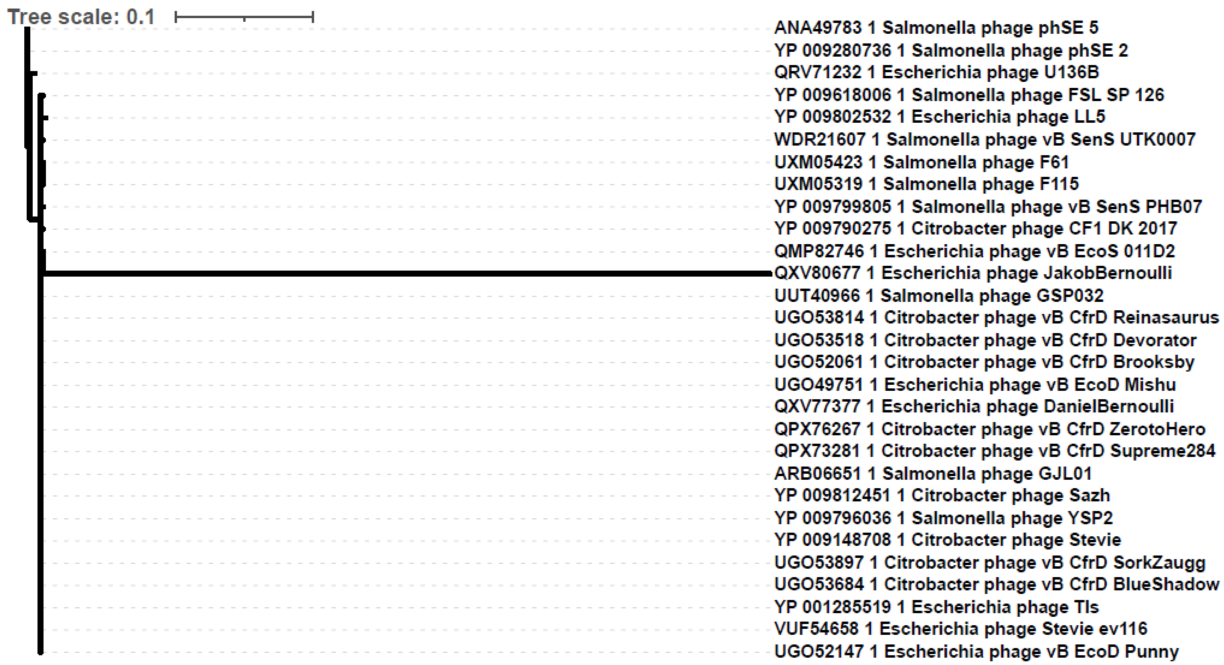

Supplementary Figure 3: This phylogenetic tree was constructed using the large subunit terminase proteins from phages belonging to the *Tlsgenus* using NGPhylogeny.fr[34] with the tree exported to iTol[35]. The TerL protein from *Escherichia* phage JakobBernoulli, a member of the *Hanrivervirus* genus, was used as the outlier.
