# Supplementary material for: The TolC and Lipopolysaccharide-Specific Escherichia coli Bacteriophage TLS—the Tlsvirus Archetype Virus
Source: Phage (New Rochelle). 2024 Sep 16;5(3):173–83. doi: 10.1089/phage.2023.0041 (PMC11447400; doi:10.1089/phage.2023.0041)
Supplement: Supplementary Table S1 [file phage.2023.0041_suppl_tables1.pdf]

Supplementary Table 1: Evidence for the presence of promoters, rho-independent terminators and unique 21 nt direct repeats in the genome of phage TLS.

terminator	strand	position	sequence	ΔG kcal/mol
T28	pos	8723..8744	cccgccttgcgcgggttttttt	-8.3
T30	pos	10914..10941	ggctggaatattccggcctttttattat	-11.8
T34	pos	14520..14547	ggccggattattccggccttttatatat	-14.8
T35	pos	15049..15069	gccgttattatgacggctttt	-8.7
T36A	pos	16078..16107	gggaaccgttaaaggttcccttttttatat	-13.4
T36B	pos	16402..16430	gggcgctcatgtagcgcctttttttattat	-10.7
T42	pos	18714..18740	ggggcgcattgcgtcccttttttattt	-11.9
T44	pos	19418..19440	gcgcctacgggcgctttttttat	-12.4
T46	pos	22718..22742	gccgccattgtgcggcttttttatt	-9.1
T49	pos	24858..24879	cccgccgttgcgcgggtttttt	-8.5
T50	pos	28723..28754	gccctggcttaacggtcggggcattttgtttt	-15.8
T52	pos	30597..30618	ggcggcgcaagccgccattatt	-17.1
T55A	neg	31760..31782	ggggcgcaagcccctttattttt	-14.1
T54	pos	31769..31786	ggggcttgcgcccctttt	-10.3
T55B	neg	32025..32053	gggaggcgcaacgcctcctttttttattt	-12.9
T56	neg	34149..34179	ccccgctaaggcggggtttttatt	-12.5
T57	neg	35178..35214	cctggcatggtttatcatcgtgccaggttttttattt	-17.1
T62	pos	39643..39660	cccgctacggcgggtttt	-11.7
T66	pos	41775..41806	gctagctaaatgctagccttttttttgttttt	-13.2
T69/T70	pos/neg	42963..42987	ggcggcataagccgccttttctttt	-12.9
**promoter**	**strand**	**position**	**sequence**	
P01	pos	523..549	ttgacgagaacaaacggcggcgataat	

P51	pos	29110..29137	ttgactacatgaaacaaagacatataat	
P57	neg	35881..35909	ttgactaaatgccatttgcggcatatcat	
**direct repeat**	**strand**	**position**	**sequence**	
DR1	pos	1521..1541	aaatagcactttttgttaaaa	
DR2	pos	1927..1947	aaatagcacgaattgctaaaa	
DR3	pos	2555..2575	aaacagcacgaattgctaaaa	
DR4	pos	4059..4079	aaatagcactttttgctaaaa	
DR5	pos	5134..5154	aaatagcacgaattgctaaaa	
DR6	pos	6476..6496	aaatagcactttttgttaaaa	
DR7	neg	15044..15024	atatagcatttttttgttaaac	
DR8	pos	24906..24926	aaatagcacaaaaggttaaaa	
DR9	pos	30220..30240	aaatagcactttttgctaaaa	
DR10	neg	35242..35222	cgatagcactgattgctaaaa	
DR11	pos	35841..35861	ctatagcactttttgctaaaa	
DR12	pos	39622..39642	aaatagcactttttgttaaaa	
DR13	neg	45155..45135	aaatagcacgaattgctaaaa	
DR14	neg	45832..45812	aaatagcactttttgttaaaa	
DR15	neg	47245..47225	aaatagcacgaattgctaaaa	
DR16	neg	48197..48177	aaatagcactttttgttaaaa	
DR17	neg	48921..48901	aaatagcactttttgttaaac	
DR18	neg	49784..49764	gaatagcatttttttgctaaaa	
