# Supplementary material for: The TolC and Lipopolysaccharide-Specific Escherichia coli Bacteriophage TLS—the Tlsvirus Archetype Virus
Source: Phage (New Rochelle). 2024 Sep 16;5(3):173–83. doi: 10.1089/phage.2023.0041 (PMC11447400; doi:10.1089/phage.2023.0041)
Supplement: Supplementary Table S2 [file phage.2023.0041_suppl_tables2.pdf]

Supplementary Table 2: The genes and proteins of *Escherichia* phage TLS showing motifs and their sequence similarity to those of phage T1.

Gene	Product	Coordinates	Strand	Amino acid residues	Molecular weight	pI	Phage T1 homolog - locus tag	Phage T1 homolog - Accession No.	E value	Motifs
gp01	hypothetical protein	589..1038	+	149	16724	5.2				
gp02	hypothetical protein	1042..1290	+	82	9449	5.3				
gp03	hypothetical protein	1321..1500	+	59	6766	9.1				
gp04	hypothetical protein	1598..1855	+	85	9596	4.0				
gp05	hypothetical protein	1865..1945	+	26	3279	10.0				
gp06	hypothetical protein	2007..2573	+	188	21049	7.3	CPTT1_008	AAP49930.1	2.00E-18	
gp07	hypothetical protein	2634..2948	+	104	12150	9.1				
gp08	hypothetical protein	3021..3128	+	35	3780	8.5				1 TMD
gp09	polynucleotide kinase	3132..3695	+	187	21420	4.7	CPTT1_014	AAP49936.1	1.00E-68	cl21460, HAD_like superfamily
gp10	hypothetical protein	3676..3864	+	62	7265	5.5				
gp11	hypothetical protein	3875..4060	+	61	7101	4.5				2 TMD
gp12	hypothetical protein	4138..4392	+	84	10049	7.6				1 TMD
gp13	hypothetical protein	4389..4571	+	60	6896	4.4				
gp14	deoxynucleotide kinase	4681..5139	+	191	17684	7.9	CPTT1_016	AAP49939.1	4.00E-51	
gp15	hypothetical protein	5220..5417	+	65	7149	4.7				
gp16	hypothetical protein	5419..5583	+	54	6218	5.5				
gp17	hypothetical protein	5576..5719	+	47	5163	6.0				2 TMD
gp18	hypothetical protein	5712..5957	+	81	9519	4.7	CPTT1_013	AAP49935.1	1.00E-08	
gp19	hypothetical protein	5969..6481	+	170	19443	5.4				
gp20	hypothetical protein	6556..6765	+	69	8185	3.6				
gp21	hypothetical protein	6762..7013	+	83	9377	5.7	CPTT1_018	AAP49941.1	5.00E-13	
gp22	hypothetical protein	7010..7348	+	112	12788	4.4	CPTT1_076	AAP49995.1	3.00E-06	
gp23	hypothetical protein	7408..7536	+	42	5063	4.9				
gp24	hypothetical protein	7538..7765	+	75	8469	9.0				

gp25	hypothetical protein	7948..8157	+	69	7985	5.2				
gp26	hypothetical protein	8147..8308	+	53	6065	10.0	CPTT1_021	AAP49944.1	3.00E-13	
gp27	hypothetical protein	8325..8474	+	49	5434	5.5	CPTT1_022	AAP49945.2	6.00E-12	
gp28	hypothetical membrane protein	8474..8704	+	76	8787	7.7				1 TMD
gp29	terminase, small subunit	8792..9316	+	174	19476	7.8	CPTT1_024	AY216660.1	1.00E-58	cl25037, DNA-packaging protein gp3 (IPR032066)
gp30	terminase, large subunit	9328..10899	+	523	60130	6.2	CPTT1_025	AAP49948.1	0.00E+00	cl40794, COG5410 superfamily
gp31	portal protein	10952..12247	+	431	48987	5.0	CPTT1_026	AAP49949.1	0.00E+00	cl21602, DUF1073 superfamily
gp32	capsid morphogenesis protein	12247..12915	+	222	26121	8.7	CPTT1_027	AAP49999.1	1.00E-52	cl26983, COG2369 superfamily
gp33	capsid maturation protease	12912..14021	+	369	39947	4.9	CPTT1_028	AAP49950.1	3.00E-166	cl44587, DUF2213 superfamily
gp34	capsid decoration protein	14034..14513	+	159	16615	4.7	CPTT1_029	AAP49951.1	3.00E-50	
gp35	IgG-domain-containing decoration protein	14557..14997	+	146	16355	4.7	CPTT1_030	AAP49952.1	2.00E-12	cl41931, DUF2184 superfamily
gp36	major capsid protein	15087..16061	+	324	35964	5.8	CPTT1_031	AAP49953.1	2.00E-179	
gp37	hypothetical protein	16122..16394	+	90	9899	5.1	CPTT1_032	AAP49954.1	2.00E-19	cl16194, DUF4054 superfamily
gp38	head-to-tail connector complex protein	16443..16847	+	134	15381	8.1	CPTT1_033	AAP49955.1	1.00E-42	
gp39	head-to-tail connector complex protein II	16844..17215	+	123	13713	5.8	CPTT1_034	AAP49956.1	3.00E-32	
gp40	hypothetical protein	17208..17648	+	146	16213	8.7	CPTT1_035	AAP49957.1	9.00E-61	
gp41	minor tail protein	17638..18030	+	130	14908	5.9	CPTT1_036	AAP49958.1	7.00E-55	cl16303, DUF4128 superfamily

gp42	major tail protein	18045..18707	+	220	24280	4.5	CPTT1_037	AAP49959.1	7.00E-118	cl07426, Phage_tail_3 superfamily
gp43A	tape measure chaperone	18779..19089, 19089..19416	+	212	24227	4.6	CPTT1_039	AAP50000.2	4.00E-85	cl07424, DUF1799 superfamily
gp43	tape measure chaperone	18779..19099	+	103	11957	5.0	CPTT1_038	AAP49960.1	1.00E-30	cl07386, Phage_TAC_4 superfamily
gp44	hypothetical protein	19219..19416	+	65	7604	4.4	CPTT1_039	AAP50000.2	2.00E-32	cl07424, DUF1799 superfamily
gp45	tape measure protein	19527..22364	+	945	103112	8.5	CPTT1_040	AAP49961.1	0.00E+00	1 TMD, cl06039, TMP_2 superfamily
gp46	minor tail protein	22364..22711	+	115	12948	7.9	CPTT1_041	AAP49962.1	6.00E-49	cl01940, Phage_min_tail superfamily
gp47	minor tail protein	22779..23537	+	252	28102	6.6	CPTT1_042	AAP49963.1	6.00E-152	Phage_tail_L superfamily
gp48	minor tail protein	23534..24256	+	240	27696	5.4	CPTT1_043	AAP49964.1	2.00E-129	cl13996, MPN superfamily
gp49	tail assembly protein	24249..24848	+	199	20989	8.6	CPTT1_044	AAP49965.1	3.00E-115	1 TMD, cl01945, Lambda_tail_I superfamily
gp50	tail fiber	24930..28706	+	1258	138931	4.8	CPTT1_045	AAP49966.1	0.00E+00	cl34810, COG4733 superfamily
gp51	exodeoxyribonuclease VIII	29166..30221	+	351	39915	5.1	CPTT1_050	AAP49970.1	3.00E-167	
gp52	hypothetical protein	30251..30589	+	112	13225	4.7				
gp53	recombinase	30636..31298	+	220	24817	7.0	CPTT1_051	AAP49971.1	1.00E-64	cl04500, ERF superfamily
gp54	single-stranded DNA-binding protein	31336..31761	+	141	16365	5.0	CPTT1_052	AAP49972.1	2.00E-46	
gp55	hypothetical protein	32060..34084	-	674	74104	4.5	CPTT1_053	AAP49973.1	3.00E-66	

gp56	DNA primase	34239..35165	-	308	34977	5.6	CPTT1_055	AAP49975.2	7.00E-131	cl26703, COG4643 superfamily
gp57	transcriptional regulator	35224..35832	-	202	23139	10.3	CPTT1_056	AAP49976.1	2.00E-53	
gp58	DNA helicase	35921..37894	+	657	74576	7.3	CPTT1_057	AAP49977.1	0.00E+00	cl34083, SSL2 superfamily
gp59	Holliday junction resolvase	37896..38303	+	135	15354	7.5	CPTT1_058	AAP49978.1	2.00E-56	cl22959, VRR_NUC superfamily
gp60	hypothetical protein	38231..38653	+	140	15866	9.4				
gp61	DNA adenine methyltransferase	38655..39395	+	246	28257	5.9	CPTT1_059	AAP49979.1	4.00E-118	cl22959, VRR_NUC superfamily
gp62	hypothetical protein	39397..39627	+	76	9250	8.0	CPTT1_060	AAP49980.2	3.00E-06	
gp63	hypothetical protein	39666..39863	+	65	7829	9.7				
gp64	hypothetical protein	39844..40086	+	80	9244	9.6	CPTT1_062	WBY66054.1	2.00E-20	
gp65	HNH endonuclease	39965..40636	+	223	26046	10.2	CPTT1_015	AAP49937.1	1.00E-36	cl00083, HNHc superfamily
gp66	proof-reading exonuclease	40640..41755	+	371	41861	6.5	CPTT1_064	AAP49983.1	8.00E-166	HHpred - similarity to 6KNB
gp67	pinholin class 2	41868..42092	+	74	8286	9.2	CPTT1_067	AAP49986.1	6.00E-25	1 TMD
gp68	SAR endolysin	42092..42586	+	164	18206	9.4	CPTT1_068	AAP49987.1	3.00E-71	1 TMD, cl00222, Lyz-like superfamily
gp69	u-spanin	42583..42972	+	129	13613	9.6	CPTT1_069	AAP49988.1	4.00E-28	
gp70	HTH domain-containing protein	43107..43499	-	130	15713	8.3	CPTT1_070	AAP49989.1	1.00E-41	
gp71	putative ATPase	43502..45082	-	526	58216	6.3	CPTT1_071	AAP49990.1	0.00E+00	cl20483, DUF3987 superfamily; HHpred - similarity to 6RAW
gp72	hypothetical protein	45150..45395	-	81	9418	9.8				
gp73	hypothetical protein	45392..45754	-	120	13754	9.5	CPTT1_073	AAP49992.1	4.00E-29	
gp74	DNA cytosine methyltransferase	45827..46606	-	259	29087	8.0				

gp75	hypothetical protein	46721..46945	-	74	8207	4.0	CPTT1_074	AAP49993.1	2.00E-15	
gp76	hypothetical protein	46947..47168	-	73	8258	9.8	CPTT1_075	AAP49994.1	2.00E-17	
gp77	hypothetical protein	47227..47394	-	55	6348	10.6				
gp78	hypothetical protein	47391..47636	-	81	9307	7.7				
gp79	HNH endonuclease	47608..48120	-	170	19705	9.8	CPTT1_015	AAP49937.1	7.00E-32	cl00083, HNHc superfamily
gp80	hypothetical protein	48196..48390	-	64	7446	7.6	CPTT1_078	AAP49997.1	1.00E-17	
gp81	hypothetical protein	48387..48623	-	78	9069	7.0				
gp82	hypothetical protein	48624..48845	-	73	8292	5.1				
gp83	hypothetical protein	48921..49064	-	47	5377	8.0				
gp84	hypothetical protein	49061..49279	-	72	8117	7.3				
gp85	hypothetical protein	49281..49565	-	94	10806	9.3				
gp86	hypothetical protein	49549..49707	-	52	5964	4.8				
