# Supplementary material for: The TolC and Lipopolysaccharide-Specific Escherichia coli Bacteriophage TLS—the Tlsvirus Archetype Virus
Source: Phage (New Rochelle). 2024 Sep 16;5(3):173–83. doi: 10.1089/phage.2023.0041 (PMC11447400; doi:10.1089/phage.2023.0041)
Supplement: Supplementary Data S1 [file phage.2023.0041_supp_datas1.pdf]

```
MODEL     1
HEADER    PHAGE PROTEIN                                   15-NOV-23
TITLE     ALPHAFOLD2 PREDICTED STRUCTURE OF PHAGE TLS TAIL FIBRE

ATOM      1  N   MET A   1      54.094  -0.114 -33.438  1.00 32.16           N
ATOM      2  CA  MET A   1      55.000  -0.940 -34.219  1.00 32.16           C
ATOM      3  C   MET A   1      54.562  -1.089 -35.656  1.00 32.16           C
ATOM      4  CB  MET A   1      55.156  -2.318 -33.562  1.00 32.16           C
ATOM      5  O   MET A   1      53.531  -1.723 -35.906  1.00 32.16           O
ATOM      6  CG  MET A   1      56.531  -2.551 -32.938  1.00 32.16           C
ATOM      7  SD  MET A   1      56.719  -4.266 -32.312  1.00 32.16           S
ATOM      8  CE  MET A   1      58.406  -4.164 -31.625  1.00 32.16           C
ATOM      9  N   ILE A   2      54.531   0.038 -36.312  1.00 39.12           N
ATOM     10  CA  ILE A   2      54.656   0.371 -37.719  1.00 39.12           C
ATOM     11  C   ILE A   2      55.938  -0.284 -38.281  1.00 39.12           C
ATOM     12  CB  ILE A   2      54.719   1.900 -37.938  1.00 39.12           C
ATOM     13  O   ILE A   2      57.031  -0.052 -37.781  1.00 39.12           O
ATOM     14  CG1 ILE A   2      53.438   2.553 -37.438  1.00 39.12           C
ATOM     15  CG2 ILE A   2      54.938   2.229 -39.438  1.00 39.12           C
ATOM     16  CD1 ILE A   2      53.469   4.074 -37.438  1.00 39.12           C
ATOM     17  N   LYS A   3      55.969  -1.574 -38.625  1.00 38.41           N
ATOM     18  CA  LYS A   3      56.938  -2.221 -39.531  1.00 38.41           C
ATOM     19  C   LYS A   3      57.062  -1.441 -40.844  1.00 38.41           C
ATOM     20  CB  LYS A   3      56.500  -3.658 -39.812  1.00 38.41           C
ATOM     21  O   LYS A   3      56.094  -1.240 -41.562  1.00 38.41           O
ATOM     22  CG  LYS A   3      57.406  -4.711 -39.188  1.00 38.41           C
ATOM     23  CD  LYS A   3      56.969  -6.121 -39.562  1.00 38.41           C
ATOM     24  CE  LYS A   3      57.875  -7.176 -38.969  1.00 38.41           C
ATOM     25  NZ  LYS A   3      57.406  -8.562 -39.281  1.00 38.41           N
ATOM     26  N   ASN A   4      58.000  -0.498 -40.875  1.00 35.16           N
ATOM     27  CA  ASN A   4      59.125  -0.096 -41.719  1.00 35.16           C
ATOM     28  C   ASN A   4      59.406  -1.135 -42.812  1.00 35.16           C
ATOM     29  CB  ASN A   4      60.375   0.153 -40.906  1.00 35.16           C
ATOM     30  O   ASN A   4      59.594  -2.312 -42.500  1.00 35.16           O
ATOM     31  CG  ASN A   4      60.344   1.487 -40.188  1.00 35.16           C
ATOM     32  ND2 ASN A   4      61.188   1.637 -39.156  1.00 35.16           N
ATOM     33  OD1 ASN A   4      59.562   2.375 -40.531  1.00 35.16           O
ATOM     34  N   MET A   5      59.312  -0.828 -44.156  1.00 40.00           N
ATOM     35  CA  MET A   5      60.500  -0.995 -44.938  1.00 40.00           C
ATOM     36  C   MET A   5      60.188  -1.354 -46.406  1.00 40.00           C
ATOM     37  CB  MET A   5      61.406  -2.072 -44.344  1.00 40.00           C
ATOM     38  O   MET A   5      59.406  -2.295 -46.625  1.00 40.00           O
ATOM     39  CG  MET A   5      62.875  -1.935 -44.719  1.00 40.00           C
ATOM     40  SD  MET A   5      63.969  -3.047 -43.750  1.00 40.00           S
ATOM     41  CE  MET A   5      63.469  -4.656 -44.438  1.00 40.00           C
ATOM     42  N   ILE A   6      60.625  -0.537 -47.344  1.00 36.97           N
ATOM     43  CA  ILE A   6      61.688  -0.321 -48.312  1.00 36.97           C
ATOM     44  C   ILE A   6      61.094  -0.372 -49.750  1.00 36.97           C
ATOM     45  CB  ILE A   6      62.812  -1.363 -48.156  1.00 36.97           C
ATOM     46  O   ILE A   6      60.406  -1.329 -50.094  1.00 36.97           O
ATOM     47  CG1 ILE A   6      63.406  -1.296 -46.750  1.00 36.97           C
ATOM     48  CG2 ILE A   6      63.906  -1.151 -49.219  1.00 36.97           C
ATOM     49  CD1 ILE A   6      64.625  -2.182 -46.562  1.00 36.97           C
ATOM     50  N   THR A   7      61.031   0.739 -50.531  1.00 39.03           N
ATOM     51  CA  THR A   7      61.375   1.372 -51.812  1.00 39.03           C
ATOM     52  C   THR A   7      62.438   0.578 -52.531  1.00 39.03           C
ATOM     53  CB  THR A   7      61.844   2.820 -51.594  1.00 39.03           C
ATOM     54  O   THR A   7      63.406   0.113 -51.938  1.00 39.03           O
ATOM     55  CG2 THR A   7      60.719   3.713 -51.094  1.00 39.03           C
ATOM     56  OG1 THR A   7      62.906   2.838 -50.625  1.00 39.03           O
ATOM     57  N   GLY A   8      62.188   0.046 -53.781  1.00 38.09           N
ATOM     58  CA  GLY A   8      63.062  -0.157 -54.938  1.00 38.09           C
ATOM     59  C   GLY A   8      62.406   0.145 -56.250  1.00 38.09           C
ATOM     60  O   GLY A   8      61.250  -0.200 -56.469  1.00 38.09           O
```

ATOM	61	N	SER	A	9	62.656	1.282	-57.000	1.00	39.72	N
ATOM	62	CA	SER	A	9	62.938	1.779	-58.344	1.00	39.72	C
ATOM	63	C	SER	A	9	63.812	0.798	-59.125	1.00	39.72	C
ATOM	64	CB	SER	A	9	63.625	3.143	-58.281	1.00	39.72	C
ATOM	65	O	SER	A	9	64.750	0.252	-58.594	1.00	39.72	O
ATOM	66	OG	SER	A	9	63.062	3.941	-57.250	1.00	39.72	O
ATOM	67	N	LYS	A	10	63.312	0.073	-60.344	1.00	34.94	N
ATOM	68	CA	LYS	A	10	64.062	-0.204	-61.562	1.00	34.94	C
ATOM	69	C	LYS	A	10	63.406	-1.270	-62.406	1.00	34.94	C
ATOM	70	CB	LYS	A	10	65.500	-0.648	-61.219	1.00	34.94	C
ATOM	71	O	LYS	A	10	62.906	-2.273	-61.875	1.00	34.94	O
ATOM	72	CG	LYS	A	10	66.562	0.419	-61.469	1.00	34.94	C
ATOM	73	CD	LYS	A	10	68.000	-0.141	-61.312	1.00	34.94	C
ATOM	74	CE	LYS	A	10	69.000	0.908	-61.594	1.00	34.94	C
ATOM	75	NZ	LYS	A	10	70.438	0.367	-61.438	1.00	34.94	N
ATOM	76	N	GLY	A	11	62.625	-1.061	-63.656	1.00	35.50	N
ATOM	77	CA	GLY	A	11	62.844	-1.522	-65.000	1.00	35.50	C
ATOM	78	C	GLY	A	11	63.250	-2.980	-65.125	1.00	35.50	C
ATOM	79	O	GLY	A	11	64.312	-3.369	-64.562	1.00	35.50	O
ATOM	80	N	GLY	A	12	62.281	-4.125	-65.062	1.00	37.97	N
ATOM	81	CA	GLY	A	12	62.500	-5.516	-65.438	1.00	37.97	C
ATOM	82	C	GLY	A	12	61.406	-6.445	-64.938	1.00	37.97	C
ATOM	83	O	GLY	A	12	60.938	-6.320	-63.781	1.00	37.97	O
ATOM	84	N	SER	A	13	60.219	-6.832	-65.750	1.00	42.44	N
ATOM	85	CA	SER	A	13	59.469	-7.926	-66.312	1.00	42.44	C
ATOM	86	C	SER	A	13	58.031	-7.949	-65.812	1.00	42.44	C
ATOM	87	CB	SER	A	13	60.156	-9.266	-66.062	1.00	42.44	C
ATOM	88	O	SER	A	13	57.812	-7.754	-64.562	1.00	42.44	O
ATOM	89	OG	SER	A	13	60.000	-9.633	-64.688	1.00	42.44	O
ATOM	90	N	SER	A	14	56.969	-7.406	-66.438	1.00	50.28	N
ATOM	91	CA	SER	A	14	55.562	-7.688	-66.375	1.00	50.28	C
ATOM	92	C	SER	A	14	55.281	-9.078	-65.750	1.00	50.28	C
ATOM	93	CB	SER	A	14	54.875	-7.578	-67.750	1.00	50.28	C
ATOM	94	O	SER	A	14	55.938	-10.047	-66.188	1.00	50.28	O
ATOM	95	OG	SER	A	14	54.469	-8.852	-68.188	1.00	50.28	O
ATOM	96	N	LYS	A	15	55.188	-9.289	-64.500	1.00	52.41	N
ATOM	97	CA	LYS	A	15	54.688	-10.484	-63.812	1.00	52.41	C
ATOM	98	C	LYS	A	15	53.500	-11.062	-64.562	1.00	52.41	C
ATOM	99	CB	LYS	A	15	54.281	-10.172	-62.375	1.00	52.41	C
ATOM	100	O	LYS	A	15	52.594	-10.336	-65.000	1.00	52.41	O
ATOM	101	CG	LYS	A	15	55.406	-10.172	-61.375	1.00	52.41	C
ATOM	102	CD	LYS	A	15	54.938	-9.953	-59.969	1.00	52.41	C
ATOM	103	CE	LYS	A	15	56.094	-9.867	-58.969	1.00	52.41	C
ATOM	104	NZ	LYS	A	15	55.625	-9.750	-57.562	1.00	52.41	N
ATOM	105	N	PRO	A	16	53.625	-12.109	-65.500	1.00	64.44	N
ATOM	106	CA	PRO	A	16	52.469	-12.742	-66.125	1.00	64.44	C
ATOM	107	C	PRO	A	16	51.250	-12.758	-65.188	1.00	64.44	C
ATOM	108	CB	PRO	A	16	52.938	-14.172	-66.375	1.00	64.44	C
ATOM	109	O	PRO	A	16	51.438	-12.859	-63.938	1.00	64.44	O
ATOM	110	CG	PRO	A	16	54.312	-14.234	-65.812	1.00	64.44	C
ATOM	111	CD	PRO	A	16	54.656	-12.898	-65.250	1.00	64.44	C
ATOM	112	N	HIS	A	17	50.062	-12.023	-65.500	1.00	77.38	N
ATOM	113	CA	HIS	A	17	48.750	-12.156	-64.875	1.00	77.38	C
ATOM	114	C	HIS	A	17	48.469	-13.609	-64.500	1.00	77.38	C
ATOM	115	CB	HIS	A	17	47.656	-11.617	-65.750	1.00	77.38	C
ATOM	116	O	HIS	A	17	48.625	-14.500	-65.312	1.00	77.38	O
ATOM	117	CG	HIS	A	17	46.281	-11.797	-65.188	1.00	77.38	C
ATOM	118	CD2	HIS	A	17	45.250	-12.531	-65.688	1.00	77.38	C
ATOM	119	ND1	HIS	A	17	45.844	-11.180	-64.062	1.00	77.38	N
ATOM	120	CE1	HIS	A	17	44.594	-11.531	-63.812	1.00	77.38	C
ATOM	121	NE2	HIS	A	17	44.188	-12.352	-64.812	1.00	77.38	N
ATOM	122	N	THR	A	18	48.500	-14.055	-63.188	1.00	79.56	N
ATOM	123	CA	THR	A	18	48.031	-15.336	-62.719	1.00	79.56	C
ATOM	124	C	THR	A	18	46.500	-15.430	-62.812	1.00	79.56	C

ATOM	125	CB	THR	A	18	48.438	-15.578	-61.250	1.00	79.56	C
ATOM	126	O	THR	A	18	45.781	-14.602	-62.250	1.00	79.56	O
ATOM	127	CG2	THR	A	18	48.219	-17.031	-60.844	1.00	79.56	C
ATOM	128	OG1	THR	A	18	49.844	-15.273	-61.125	1.00	79.56	O
ATOM	129	N	PRO	A	19	45.969	-16.031	-63.844	1.00	80.75	N
ATOM	130	CA	PRO	A	19	44.531	-16.234	-64.000	1.00	80.75	C
ATOM	131	C	PRO	A	19	43.812	-16.484	-62.656	1.00	80.75	C
ATOM	132	CB	PRO	A	19	44.438	-17.453	-64.938	1.00	80.75	C
ATOM	133	O	PRO	A	19	44.344	-17.219	-61.812	1.00	80.75	O
ATOM	134	CG	PRO	A	19	45.750	-17.531	-65.562	1.00	80.75	C
ATOM	135	CD	PRO	A	19	46.781	-16.906	-64.688	1.00	80.75	C
ATOM	136	N	VAL	A	20	42.781	-15.719	-62.312	1.00	83.12	N
ATOM	137	CA	VAL	A	20	41.906	-15.836	-61.125	1.00	83.12	C
ATOM	138	C	VAL	A	20	40.562	-16.453	-61.531	1.00	83.12	C
ATOM	139	CB	VAL	A	20	41.688	-14.461	-60.469	1.00	83.12	C
ATOM	140	O	VAL	A	20	39.969	-16.047	-62.531	1.00	83.12	O
ATOM	141	CG1	VAL	A	20	40.750	-14.602	-59.250	1.00	83.12	C
ATOM	142	CG2	VAL	A	20	43.031	-13.844	-60.062	1.00	83.12	C
ATOM	143	N	GLU	A	21	40.312	-17.578	-60.844	1.00	89.62	N
ATOM	144	CA	GLU	A	21	39.062	-18.281	-61.031	1.00	89.62	C
ATOM	145	C	GLU	A	21	38.000	-17.781	-60.062	1.00	89.62	C
ATOM	146	CB	GLU	A	21	39.250	-19.781	-60.844	1.00	89.62	C
ATOM	147	O	GLU	A	21	38.156	-17.844	-58.844	1.00	89.62	O
ATOM	148	CG	GLU	A	21	38.000	-20.609	-61.094	1.00	89.62	C
ATOM	149	CD	GLU	A	21	38.219	-22.109	-61.031	1.00	89.62	C
ATOM	150	OE1	GLU	A	21	37.281	-22.875	-61.250	1.00	89.62	O
ATOM	151	OE2	GLU	A	21	39.375	-22.516	-60.750	1.00	89.62	O
ATOM	152	N	MET	A	22	36.875	-17.047	-60.594	1.00	89.88	N
ATOM	153	CA	MET	A	22	35.719	-16.672	-59.781	1.00	89.88	C
ATOM	154	C	MET	A	22	35.125	-17.906	-59.094	1.00	89.88	C
ATOM	155	CB	MET	A	22	34.656	-16.000	-60.656	1.00	89.88	C
ATOM	156	O	MET	A	22	35.062	-18.984	-59.688	1.00	89.88	O
ATOM	157	CG	MET	A	22	33.438	-15.523	-59.875	1.00	89.88	C
ATOM	158	SD	MET	A	22	32.219	-14.664	-60.938	1.00	89.88	S
ATOM	159	CE	MET	A	22	30.953	-14.258	-59.719	1.00	89.88	C
ATOM	160	N	GLU	A	23	35.031	-17.953	-57.781	1.00	87.19	N
ATOM	161	CA	GLU	A	23	34.375	-19.031	-57.062	1.00	87.19	C
ATOM	162	C	GLU	A	23	33.000	-19.312	-57.594	1.00	87.19	C
ATOM	163	CB	GLU	A	23	34.312	-18.703	-55.562	1.00	87.19	C
ATOM	164	O	GLU	A	23	32.312	-18.406	-58.094	1.00	87.19	O
ATOM	165	CG	GLU	A	23	35.688	-18.703	-54.875	1.00	87.19	C
ATOM	166	CD	GLU	A	23	35.594	-18.453	-53.375	1.00	87.19	C
ATOM	167	OE1	GLU	A	23	36.625	-18.516	-52.688	1.00	87.19	O
ATOM	168	OE2	GLU	A	23	34.469	-18.203	-52.875	1.00	87.19	O
ATOM	169	N	ASP	A	24	32.688	-20.531	-57.625	1.00	86.12	N
ATOM	170	CA	ASP	A	24	31.312	-20.859	-57.969	1.00	86.12	C
ATOM	171	C	ASP	A	24	30.328	-20.031	-57.156	1.00	86.12	C
ATOM	172	CB	ASP	A	24	31.031	-22.344	-57.781	1.00	86.12	C
ATOM	173	O	ASP	A	24	30.344	-20.062	-55.938	1.00	86.12	O
ATOM	174	CG	ASP	A	24	31.641	-23.203	-58.875	1.00	86.12	C
ATOM	175	OD1	ASP	A	24	32.156	-22.656	-59.875	1.00	86.12	O
ATOM	176	OD2	ASP	A	24	31.609	-24.453	-58.750	1.00	86.12	O
ATOM	177	N	ASN	A	25	29.469	-19.172	-57.719	1.00	86.06	N
ATOM	178	CA	ASN	A	25	28.562	-18.328	-56.969	1.00	86.06	C
ATOM	179	C	ASN	A	25	27.094	-18.625	-57.281	1.00	86.06	C
ATOM	180	CB	ASN	A	25	28.859	-16.844	-57.188	1.00	86.06	C
ATOM	181	O	ASN	A	25	26.203	-17.922	-56.844	1.00	86.06	O
ATOM	182	CG	ASN	A	25	28.672	-16.438	-58.625	1.00	86.06	C
ATOM	183	ND2	ASN	A	25	28.656	-15.125	-58.875	1.00	86.06	N
ATOM	184	OD1	ASN	A	25	28.531	-17.281	-59.531	1.00	86.06	O
ATOM	185	N	LEU	A	26	26.891	-19.547	-58.188	1.00	86.75	N
ATOM	186	CA	LEU	A	26	25.516	-20.031	-58.406	1.00	86.75	C
ATOM	187	C	LEU	A	26	25.172	-21.109	-57.375	1.00	86.75	C
ATOM	188	CB	LEU	A	26	25.344	-20.562	-59.812	1.00	86.75	C

ATOM	189	O	LEU	A	26	25.672	-22.234	-57.469	1.00	86.75	O
ATOM	190	CG	LEU	A	26	25.359	-19.516	-60.938	1.00	86.75	C
ATOM	191	CD1	LEU	A	26	25.406	-20.203	-62.281	1.00	86.75	C
ATOM	192	CD2	LEU	A	26	24.125	-18.609	-60.812	1.00	86.75	C
ATOM	193	N	ILE	A	27	24.531	-20.719	-56.344	1.00	78.69	N
ATOM	194	CA	ILE	A	27	24.203	-21.625	-55.250	1.00	78.69	C
ATOM	195	C	ILE	A	27	22.688	-21.859	-55.219	1.00	78.69	C
ATOM	196	CB	ILE	A	27	24.672	-21.047	-53.875	1.00	78.69	C
ATOM	197	O	ILE	A	27	21.906	-20.938	-55.438	1.00	78.69	O
ATOM	198	CG1	ILE	A	27	26.156	-20.656	-53.969	1.00	78.69	C
ATOM	199	CG2	ILE	A	27	24.453	-22.078	-52.750	1.00	78.69	C
ATOM	200	CD1	ILE	A	27	26.641	-19.828	-52.781	1.00	78.69	C
ATOM	201	N	SER	A	28	22.344	-23.062	-55.156	1.00	74.94	N
ATOM	202	CA	SER	A	28	20.938	-23.406	-55.000	1.00	74.94	C
ATOM	203	C	SER	A	28	20.328	-22.766	-53.750	1.00	74.94	C
ATOM	204	CB	SER	A	28	20.766	-24.922	-54.938	1.00	74.94	C
ATOM	205	O	SER	A	28	20.969	-22.734	-52.719	1.00	74.94	O
ATOM	206	OG	SER	A	28	19.406	-25.281	-55.125	1.00	74.94	O
ATOM	207	N	ILE	A	29	19.297	-21.984	-53.906	1.00	76.44	N
ATOM	208	CA	ILE	A	29	18.609	-21.328	-52.812	1.00	76.44	C
ATOM	209	C	ILE	A	29	17.391	-22.141	-52.406	1.00	76.44	C
ATOM	210	CB	ILE	A	29	18.188	-19.891	-53.188	1.00	76.44	C
ATOM	211	O	ILE	A	29	16.672	-22.672	-53.250	1.00	76.44	O
ATOM	212	CG1	ILE	A	29	19.406	-19.078	-53.656	1.00	76.44	C
ATOM	213	CG2	ILE	A	29	17.500	-19.188	-52.000	1.00	76.44	C
ATOM	214	CD1	ILE	A	29	19.047	-17.750	-54.281	1.00	76.44	C
ATOM	215	N	ASN	A	30	17.328	-22.359	-51.156	1.00	80.50	N
ATOM	216	CA	ASN	A	30	16.109	-22.953	-50.594	1.00	80.50	C
ATOM	217	C	ASN	A	30	15.133	-21.891	-50.125	1.00	80.50	C
ATOM	218	CB	ASN	A	30	16.453	-23.922	-49.469	1.00	80.50	C
ATOM	219	O	ASN	A	30	15.547	-20.859	-49.594	1.00	80.50	O
ATOM	220	CG	ASN	A	30	17.172	-25.156	-49.938	1.00	80.50	C
ATOM	221	ND2	ASN	A	30	17.922	-25.797	-49.031	1.00	80.50	N
ATOM	222	OD1	ASN	A	30	17.062	-25.547	-51.094	1.00	80.50	O
ATOM	223	N	ARG	A	31	13.938	-22.078	-50.500	1.00	82.69	N
ATOM	224	CA	ARG	A	31	12.906	-21.141	-50.062	1.00	82.69	C
ATOM	225	C	ARG	A	31	12.047	-21.734	-48.969	1.00	82.69	C
ATOM	226	CB	ARG	A	31	12.031	-20.719	-51.250	1.00	82.69	C
ATOM	227	O	ARG	A	31	11.789	-22.953	-48.969	1.00	82.69	O
ATOM	228	CG	ARG	A	31	12.766	-19.922	-52.312	1.00	82.69	C
ATOM	229	CD	ARG	A	31	11.820	-19.406	-53.406	1.00	82.69	C
ATOM	230	NE	ARG	A	31	12.531	-18.625	-54.406	1.00	82.69	N
ATOM	231	NH1	ARG	A	31	10.695	-18.375	-55.781	1.00	82.69	N
ATOM	232	NH2	ARG	A	31	12.711	-17.438	-56.344	1.00	82.69	N
ATOM	233	CZ	ARG	A	31	11.977	-18.141	-55.500	1.00	82.69	C
ATOM	234	N	ILE	A	32	11.781	-20.875	-48.000	1.00	87.69	N
ATOM	235	CA	ILE	A	32	10.859	-21.250	-46.938	1.00	87.69	C
ATOM	236	C	ILE	A	32	9.445	-20.797	-47.281	1.00	87.69	C
ATOM	237	CB	ILE	A	32	11.289	-20.672	-45.562	1.00	87.69	C
ATOM	238	O	ILE	A	32	9.234	-19.625	-47.625	1.00	87.69	O
ATOM	239	CG1	ILE	A	32	12.648	-21.234	-45.156	1.00	87.69	C
ATOM	240	CG2	ILE	A	32	10.227	-20.953	-44.500	1.00	87.69	C
ATOM	241	CD1	ILE	A	32	13.219	-20.594	-43.875	1.00	87.69	C
ATOM	242	N	ARG	A	33	8.516	-21.781	-47.344	1.00	87.12	N
ATOM	243	CA	ARG	A	33	7.102	-21.484	-47.562	1.00	87.12	C
ATOM	244	C	ARG	A	33	6.270	-21.781	-46.312	1.00	87.12	C
ATOM	245	CB	ARG	A	33	6.574	-22.281	-48.750	1.00	87.12	C
ATOM	246	O	ARG	A	33	6.293	-22.906	-45.812	1.00	87.12	O
ATOM	247	CG	ARG	A	33	7.316	-22.000	-50.062	1.00	87.12	C
ATOM	248	CD	ARG	A	33	6.852	-22.922	-51.188	1.00	87.12	C
ATOM	249	NE	ARG	A	33	7.609	-22.688	-52.406	1.00	87.12	N
ATOM	250	NH1	ARG	A	33	6.590	-24.391	-53.594	1.00	87.12	N
ATOM	251	NH2	ARG	A	33	8.211	-23.109	-54.594	1.00	87.12	N
ATOM	252	CZ	ARG	A	33	7.469	-23.406	-53.531	1.00	87.12	C

ATOM	253	N	ILE	A	34	5.508	-20.750	-45.812	1.00	90.12	N
ATOM	254	CA	ILE	A	34	4.699	-20.891	-44.594	1.00	90.12	C
ATOM	255	C	ILE	A	34	3.264	-20.469	-44.875	1.00	90.12	C
ATOM	256	CB	ILE	A	34	5.285	-20.078	-43.438	1.00	90.12	C
ATOM	257	O	ILE	A	34	3.031	-19.422	-45.500	1.00	90.12	O
ATOM	258	CG1	ILE	A	34	6.719	-20.531	-43.125	1.00	90.12	C
ATOM	259	CG2	ILE	A	34	4.398	-20.219	-42.188	1.00	90.12	C
ATOM	260	CD1	ILE	A	34	7.445	-19.656	-42.125	1.00	90.12	C
ATOM	261	N	LEU	A	35	2.396	-21.359	-44.469	1.00	89.31	N
ATOM	262	CA	LEU	A	35	0.976	-21.031	-44.531	1.00	89.31	C
ATOM	263	C	LEU	A	35	0.398	-20.859	-43.125	1.00	89.31	C
ATOM	264	CB	LEU	A	35	0.206	-22.125	-45.281	1.00	89.31	C
ATOM	265	O	LEU	A	35	0.410	-21.812	-42.312	1.00	89.31	O
ATOM	266	CG	LEU	A	35	-1.283	-21.859	-45.500	1.00	89.31	C
ATOM	267	CD1	LEU	A	35	-1.476	-20.625	-46.375	1.00	89.31	C
ATOM	268	CD2	LEU	A	35	-1.945	-23.078	-46.156	1.00	89.31	C
ATOM	269	N	LEU	A	36	-0.151	-19.594	-42.781	1.00	91.75	N
ATOM	270	CA	LEU	A	36	-0.669	-19.281	-41.469	1.00	91.75	C
ATOM	271	C	LEU	A	36	-2.184	-19.109	-41.500	1.00	91.75	C
ATOM	272	CB	LEU	A	36	-0.013	-18.016	-40.906	1.00	91.75	C
ATOM	273	O	LEU	A	36	-2.717	-18.438	-42.406	1.00	91.75	O
ATOM	274	CG	LEU	A	36	1.511	-18.031	-40.781	1.00	91.75	C
ATOM	275	CD1	LEU	A	36	2.049	-16.625	-40.562	1.00	91.75	C
ATOM	276	CD2	LEU	A	36	1.936	-18.953	-39.656	1.00	91.75	C
ATOM	277	N	ALA	A	37	-2.873	-19.859	-40.562	1.00	91.19	N
ATOM	278	CA	ALA	A	37	-4.262	-19.500	-40.312	1.00	91.19	C
ATOM	279	C	ALA	A	37	-4.344	-18.312	-39.344	1.00	91.19	C
ATOM	280	CB	ALA	A	37	-5.012	-20.703	-39.719	1.00	91.19	C
ATOM	281	O	ALA	A	37	-3.984	-18.438	-38.156	1.00	91.19	O
ATOM	282	N	VAL	A	38	-4.852	-17.156	-39.844	1.00	91.38	N
ATOM	283	CA	VAL	A	38	-4.695	-15.930	-39.062	1.00	91.38	C
ATOM	284	C	VAL	A	38	-6.051	-15.492	-38.500	1.00	91.38	C
ATOM	285	CB	VAL	A	38	-4.086	-14.797	-39.938	1.00	91.38	C
ATOM	286	O	VAL	A	38	-6.113	-14.680	-37.594	1.00	91.38	O
ATOM	287	CG1	VAL	A	38	-2.625	-15.102	-40.250	1.00	91.38	C
ATOM	288	CG2	VAL	A	38	-4.898	-14.594	-41.219	1.00	91.38	C
ATOM	289	N	SER	A	39	-7.133	-16.062	-39.000	1.00	91.81	N
ATOM	290	CA	SER	A	39	-8.438	-15.672	-38.500	1.00	91.81	C
ATOM	291	C	SER	A	39	-9.477	-16.766	-38.719	1.00	91.81	C
ATOM	292	CB	SER	A	39	-8.906	-14.367	-39.156	1.00	91.81	C
ATOM	293	O	SER	A	39	-9.344	-17.578	-39.656	1.00	91.81	O
ATOM	294	OG	SER	A	39	-10.227	-14.055	-38.781	1.00	91.81	O
ATOM	295	N	ASP	A	40	-10.430	-16.828	-37.844	1.00	90.50	N
ATOM	296	CA	ASP	A	40	-11.656	-17.609	-38.000	1.00	90.50	C
ATOM	297	C	ASP	A	40	-12.844	-16.719	-38.312	1.00	90.50	C
ATOM	298	CB	ASP	A	40	-11.938	-18.406	-36.719	1.00	90.50	C
ATOM	299	O	ASP	A	40	-13.320	-15.969	-37.469	1.00	90.50	O
ATOM	300	CG	ASP	A	40	-12.961	-19.500	-36.906	1.00	90.50	C
ATOM	301	OD1	ASP	A	40	-13.508	-19.641	-38.031	1.00	90.50	O
ATOM	302	OD2	ASP	A	40	-13.242	-20.250	-35.938	1.00	90.50	O
ATOM	303	N	GLY	A	41	-13.156	-16.594	-39.625	1.00	90.06	N
ATOM	304	CA	GLY	A	41	-14.164	-15.695	-40.156	1.00	90.06	C
ATOM	305	C	GLY	A	41	-13.609	-14.734	-41.219	1.00	90.06	C
ATOM	306	O	GLY	A	41	-12.391	-14.617	-41.375	1.00	90.06	O
ATOM	307	N	GLU	A	42	-14.531	-14.125	-41.969	1.00	93.38	N
ATOM	308	CA	GLU	A	42	-14.125	-13.188	-43.000	1.00	93.38	C
ATOM	309	C	GLU	A	42	-13.508	-11.930	-42.406	1.00	93.38	C
ATOM	310	CB	GLU	A	42	-15.320	-12.820	-43.906	1.00	93.38	C
ATOM	311	O	GLU	A	42	-14.039	-11.352	-41.469	1.00	93.38	O
ATOM	312	CG	GLU	A	42	-14.969	-11.914	-45.062	1.00	93.38	C
ATOM	313	CD	GLU	A	42	-16.141	-11.648	-46.000	1.00	93.38	C
ATOM	314	OE1	GLU	A	42	-16.047	-10.727	-46.844	1.00	93.38	O
ATOM	315	OE2	GLU	A	42	-17.156	-12.375	-45.875	1.00	93.38	O
ATOM	316	N	VAL	A	43	-12.344	-11.492	-42.906	1.00	94.50	N

ATOM	317	CA	VAL	A	43	-11.656	-10.305	-42.438	1.00	94.50	C
ATOM	318	C	VAL	A	43	-11.539	-9.273	-43.531	1.00	94.50	C
ATOM	319	CB	VAL	A	43	-10.258	-10.648	-41.875	1.00	94.50	C
ATOM	320	O	VAL	A	43	-11.828	-9.570	-44.688	1.00	94.50	O
ATOM	321	CG1	VAL	A	43	-10.367	-11.633	-40.688	1.00	94.50	C
ATOM	322	CG2	VAL	A	43	-9.367	-11.227	-42.969	1.00	94.50	C
ATOM	323	N	ASP	A	44	-11.102	-8.062	-43.188	1.00	94.81	N
ATOM	324	CA	ASP	A	44	-10.852	-7.012	-44.188	1.00	94.81	C
ATOM	325	C	ASP	A	44	-9.898	-7.492	-45.281	1.00	94.81	C
ATOM	326	CB	ASP	A	44	-10.289	-5.766	-43.500	1.00	94.81	C
ATOM	327	O	ASP	A	44	-8.836	-8.039	-44.969	1.00	94.81	O
ATOM	328	CG	ASP	A	44	-10.062	-4.613	-44.469	1.00	94.81	C
ATOM	329	OD1	ASP	A	44	-11.047	-4.012	-44.938	1.00	94.81	O
ATOM	330	OD2	ASP	A	44	-8.883	-4.309	-44.750	1.00	94.81	O
ATOM	331	N	PRO	A	45	-10.305	-7.328	-46.656	1.00	93.62	N
ATOM	332	CA	PRO	A	45	-9.531	-7.922	-47.750	1.00	93.62	C
ATOM	333	C	PRO	A	45	-8.242	-7.164	-48.031	1.00	93.62	C
ATOM	334	CB	PRO	A	45	-10.492	-7.840	-48.938	1.00	93.62	C
ATOM	335	O	PRO	A	45	-7.352	-7.680	-48.719	1.00	93.62	O
ATOM	336	CG	PRO	A	45	-11.430	-6.727	-48.594	1.00	93.62	C
ATOM	337	CD	PRO	A	45	-11.617	-6.699	-47.125	1.00	93.62	C
ATOM	338	N	ASN	A	46	-8.070	-5.922	-47.500	1.00	93.50	N
ATOM	339	CA	ASN	A	46	-6.887	-5.113	-47.781	1.00	93.50	C
ATOM	340	C	ASN	A	46	-5.645	-5.680	-47.094	1.00	93.50	C
ATOM	341	CB	ASN	A	46	-7.113	-3.662	-47.344	1.00	93.50	C
ATOM	342	O	ASN	A	46	-5.637	-5.895	-45.875	1.00	93.50	O
ATOM	343	CG	ASN	A	46	-8.148	-2.953	-48.188	1.00	93.50	C
ATOM	344	ND2	ASN	A	46	-8.727	-1.886	-47.656	1.00	93.50	N
ATOM	345	OD1	ASN	A	46	-8.422	-3.361	-49.312	1.00	93.50	O
ATOM	346	N	PHE	A	47	-4.609	-6.109	-47.844	1.00	94.38	N
ATOM	347	CA	PHE	A	47	-3.350	-6.664	-47.344	1.00	94.38	C
ATOM	348	C	PHE	A	47	-2.203	-6.309	-48.281	1.00	94.38	C
ATOM	349	CB	PHE	A	47	-3.453	-8.188	-47.219	1.00	94.38	C
ATOM	350	O	PHE	A	47	-2.348	-6.391	-49.531	1.00	94.38	O
ATOM	351	CG	PHE	A	47	-2.160	-8.844	-46.812	1.00	94.38	C
ATOM	352	CD1	PHE	A	47	-1.321	-9.391	-47.781	1.00	94.38	C
ATOM	353	CD2	PHE	A	47	-1.783	-8.922	-45.469	1.00	94.38	C
ATOM	354	CE1	PHE	A	47	-0.124	-10.008	-47.406	1.00	94.38	C
ATOM	355	CE2	PHE	A	47	-0.588	-9.539	-45.125	1.00	94.38	C
ATOM	356	CZ	PHE	A	47	0.240	-10.078	-46.094	1.00	94.38	C
ATOM	357	N	SER	A	48	-1.133	-5.824	-47.688	1.00	94.44	N
ATOM	358	CA	SER	A	48	0.090	-5.582	-48.469	1.00	94.44	C
ATOM	359	C	SER	A	48	1.324	-5.992	-47.656	1.00	94.44	C
ATOM	360	CB	SER	A	48	0.192	-4.109	-48.844	1.00	94.44	C
ATOM	361	O	SER	A	48	1.216	-6.398	-46.500	1.00	94.44	O
ATOM	362	OG	SER	A	48	0.604	-3.320	-47.750	1.00	94.44	O
ATOM	363	N	LEU	A	49	2.484	-5.879	-48.250	1.00	94.25	N
ATOM	364	CA	LEU	A	49	3.727	-6.250	-47.594	1.00	94.25	C
ATOM	365	C	LEU	A	49	4.062	-5.258	-46.469	1.00	94.25	C
ATOM	366	CB	LEU	A	49	4.879	-6.316	-48.594	1.00	94.25	C
ATOM	367	O	LEU	A	49	4.883	-5.551	-45.594	1.00	94.25	O
ATOM	368	CG	LEU	A	49	4.859	-7.492	-49.562	1.00	94.25	C
ATOM	369	CD1	LEU	A	49	6.062	-7.426	-50.500	1.00	94.25	C
ATOM	370	CD2	LEU	A	49	4.836	-8.812	-48.812	1.00	94.25	C
ATOM	371	N	LYS	A	50	3.336	-4.121	-46.406	1.00	94.00	N
ATOM	372	CA	LYS	A	50	3.512	-3.139	-45.344	1.00	94.00	C
ATOM	373	C	LYS	A	50	2.928	-3.646	-44.031	1.00	94.00	C
ATOM	374	CB	LYS	A	50	2.863	-1.808	-45.719	1.00	94.00	C
ATOM	375	O	LYS	A	50	3.305	-3.174	-42.938	1.00	94.00	O
ATOM	376	CG	LYS	A	50	3.502	-1.132	-46.938	1.00	94.00	C
ATOM	377	CD	LYS	A	50	2.752	0.134	-47.312	1.00	94.00	C
ATOM	378	CE	LYS	A	50	3.320	0.750	-48.594	1.00	94.00	C
ATOM	379	NZ	LYS	A	50	2.531	1.940	-49.031	1.00	94.00	N
ATOM	380	N	ASP	A	51	2.076	-4.609	-44.125	1.00	94.12	N

ATOM	381	CA	ASP	A	51	1.358	-5.172	-42.969	1.00	94.12	C
ATOM	382	C	ASP	A	51	2.104	-6.371	-42.406	1.00	94.12	C
ATOM	383	CB	ASP	A	51	-0.060	-5.578	-43.375	1.00	94.12	C
ATOM	384	O	ASP	A	51	1.752	-6.871	-41.312	1.00	94.12	O
ATOM	385	CG	ASP	A	51	-0.883	-4.410	-43.906	1.00	94.12	C
ATOM	386	OD1	ASP	A	51	-0.822	-3.311	-43.312	1.00	94.12	O
ATOM	387	OD2	ASP	A	51	-1.601	-4.598	-44.906	1.00	94.12	O
ATOM	388	N	LEU	A	52	3.156	-6.875	-43.094	1.00	95.31	N
ATOM	389	CA	LEU	A	52	3.883	-8.086	-42.719	1.00	95.31	C
ATOM	390	C	LEU	A	52	5.219	-7.746	-42.062	1.00	95.31	C
ATOM	391	CB	LEU	A	52	4.113	-8.969	-43.969	1.00	95.31	C
ATOM	392	O	LEU	A	52	6.016	-6.996	-42.625	1.00	95.31	O
ATOM	393	CG	LEU	A	52	4.852	-10.281	-43.719	1.00	95.31	C
ATOM	394	CD1	LEU	A	52	4.043	-11.188	-42.781	1.00	95.31	C
ATOM	395	CD2	LEU	A	52	5.129	-10.984	-45.031	1.00	95.31	C
ATOM	396	N	TYR	A	53	5.473	-8.312	-40.844	1.00	94.31	N
ATOM	397	CA	TYR	A	53	6.688	-8.047	-40.094	1.00	94.31	C
ATOM	398	C	TYR	A	53	7.465	-9.328	-39.844	1.00	94.31	C
ATOM	399	CB	TYR	A	53	6.352	-7.363	-38.750	1.00	94.31	C
ATOM	400	O	TYR	A	53	6.883	-10.352	-39.469	1.00	94.31	O
ATOM	401	CG	TYR	A	53	5.781	-5.977	-38.906	1.00	94.31	C
ATOM	402	CD1	TYR	A	53	6.508	-4.855	-38.531	1.00	94.31	C
ATOM	403	CD2	TYR	A	53	4.512	-5.781	-39.469	1.00	94.31	C
ATOM	404	CE1	TYR	A	53	5.984	-3.572	-38.656	1.00	94.31	C
ATOM	405	CE2	TYR	A	53	3.980	-4.504	-39.594	1.00	94.31	C
ATOM	406	OH	TYR	A	53	4.199	-2.141	-39.344	1.00	94.31	O
ATOM	407	CZ	TYR	A	53	4.723	-3.408	-39.188	1.00	94.31	C
ATOM	408	N	PHE	A	54	8.820	-9.219	-40.062	1.00	93.62	N
ATOM	409	CA	PHE	A	54	9.766	-10.242	-39.625	1.00	93.62	C
ATOM	410	C	PHE	A	54	10.578	-9.750	-38.438	1.00	93.62	C
ATOM	411	CB	PHE	A	54	10.695	-10.641	-40.781	1.00	93.62	C
ATOM	412	O	PHE	A	54	11.312	-8.766	-38.562	1.00	93.62	O
ATOM	413	CG	PHE	A	54	9.992	-11.344	-41.906	1.00	93.62	C
ATOM	414	CD1	PHE	A	54	8.758	-11.953	-41.719	1.00	93.62	C
ATOM	415	CD2	PHE	A	54	10.578	-11.406	-43.188	1.00	93.62	C
ATOM	416	CE1	PHE	A	54	8.109	-12.602	-42.750	1.00	93.62	C
ATOM	417	CE2	PHE	A	54	9.930	-12.055	-44.219	1.00	93.62	C
ATOM	418	CZ	PHE	A	54	8.703	-12.656	-44.000	1.00	93.62	C
ATOM	419	N	ASP	A	55	10.398	-10.453	-37.250	1.00	90.62	N
ATOM	420	CA	ASP	A	55	11.086	-10.055	-36.031	1.00	90.62	C
ATOM	421	C	ASP	A	55	10.898	-8.570	-35.750	1.00	90.62	C
ATOM	422	CB	ASP	A	55	12.578	-10.391	-36.125	1.00	90.62	C
ATOM	423	O	ASP	A	55	11.859	-7.859	-35.438	1.00	90.62	O
ATOM	424	CG	ASP	A	55	12.859	-11.875	-36.031	1.00	90.62	C
ATOM	425	OD1	ASP	A	55	12.078	-12.617	-35.406	1.00	90.62	O
ATOM	426	OD2	ASP	A	55	13.883	-12.320	-36.625	1.00	90.62	O
ATOM	427	N	ASP	A	56	9.586	-8.062	-36.062	1.00	89.88	N
ATOM	428	CA	ASP	A	56	9.148	-6.727	-35.688	1.00	89.88	C
ATOM	429	C	ASP	A	56	9.617	-5.680	-36.688	1.00	89.88	C
ATOM	430	CB	ASP	A	56	9.648	-6.375	-34.281	1.00	89.88	C
ATOM	431	O	ASP	A	56	9.562	-4.477	-36.406	1.00	89.88	O
ATOM	432	CG	ASP	A	56	9.070	-7.270	-33.188	1.00	89.88	C
ATOM	433	OD1	ASP	A	56	7.898	-7.691	-33.312	1.00	89.88	O
ATOM	434	OD2	ASP	A	56	9.789	-7.555	-32.219	1.00	89.88	O
ATOM	435	N	VAL	A	57	10.250	-6.102	-37.844	1.00	92.69	N
ATOM	436	CA	VAL	A	57	10.625	-5.191	-38.938	1.00	92.69	C
ATOM	437	C	VAL	A	57	9.688	-5.379	-40.125	1.00	92.69	C
ATOM	438	CB	VAL	A	57	12.094	-5.414	-39.375	1.00	92.69	C
ATOM	439	O	VAL	A	57	9.508	-6.496	-40.594	1.00	92.69	O
ATOM	440	CG1	VAL	A	57	12.469	-4.461	-40.500	1.00	92.69	C
ATOM	441	CG2	VAL	A	57	13.039	-5.238	-38.188	1.00	92.69	C
ATOM	442	N	PRO	A	58	8.984	-4.246	-40.469	1.00	94.00	N
ATOM	443	CA	PRO	A	58	8.109	-4.391	-41.656	1.00	94.00	C
ATOM	444	C	PRO	A	58	8.859	-4.828	-42.906	1.00	94.00	C

```
ATOM    445  CB  PRO A  58       7.523  -2.986 -41.812  1.00 94.00           C
ATOM    446  O   PRO A  58       9.984  -4.375 -43.156  1.00 94.00           O
ATOM    447  CG  PRO A  58       8.516  -2.076 -41.188  1.00 94.00           C
ATOM    448  CD  PRO A  58       9.188  -2.816 -40.062  1.00 94.00           C
ATOM    449  N   VAL A  59       8.281  -5.789 -43.625  1.00 94.94           N
ATOM    450  CA  VAL A  59       8.875  -6.262 -44.875  1.00 94.94           C
ATOM    451  C   VAL A  59       8.992  -5.102 -45.844  1.00 94.94           C
ATOM    452  CB  VAL A  59       8.062  -7.418 -45.469  1.00 94.94           C
ATOM    453  O   VAL A  59      10.008  -4.953 -46.531  1.00 94.94           O
ATOM    454  CG1 VAL A  59       8.602  -7.773 -46.875  1.00 94.94           C
ATOM    455  CG2 VAL A  59       8.086  -8.633 -44.562  1.00 94.94           C
ATOM    456  N   MET A  60       7.984  -4.262 -45.906  1.00 95.56           N
ATOM    457  CA  MET A  60       7.961  -3.051 -46.719  1.00 95.56           C
ATOM    458  C   MET A  60       7.613  -1.830 -45.875  1.00 95.56           C
ATOM    459  CB  MET A  60       6.965  -3.197 -47.875  1.00 95.56           C
ATOM    460  O   MET A  60       6.621  -1.839 -45.125  1.00 95.56           O
ATOM    461  CG  MET A  60       7.000  -2.047 -48.844  1.00 95.56           C
ATOM    462  SD  MET A  60       5.816  -2.275 -50.250  1.00 95.56           S
ATOM    463  CE  MET A  60       6.957  -2.770 -51.562  1.00 95.56           C
ATOM    464  N   ASN A  61       8.453  -0.821 -45.906  1.00 92.69           N
ATOM    465  CA  ASN A  61       8.203   0.408 -45.156  1.00 92.69           C
ATOM    466  C   ASN A  61       6.996   1.164 -45.719  1.00 92.69           C
ATOM    467  CB  ASN A  61       9.438   1.305 -45.188  1.00 92.69           C
ATOM    468  O   ASN A  61       6.527   0.874 -46.812  1.00 92.69           O
ATOM    469  CG  ASN A  61      10.602   0.704 -44.406  1.00 92.69           C
ATOM    470  ND2 ASN A  61      11.797   0.802 -44.969  1.00 92.69           N
ATOM    471  OD1 ASN A  61      10.430   0.154 -43.312  1.00 92.69           O
ATOM    472  N   GLN A  62       6.523   2.168 -44.906  1.00 90.06           N
ATOM    473  CA  GLN A  62       5.367   2.961 -45.312  1.00 90.06           C
ATOM    474  C   GLN A  62       5.645   3.740 -46.594  1.00 90.06           C
ATOM    475  CB  GLN A  62       4.961   3.920 -44.188  1.00 90.06           C
ATOM    476  O   GLN A  62       4.727   4.020 -47.375  1.00 90.06           O
ATOM    477  CG  GLN A  62       4.176   3.256 -43.062  1.00 90.06           C
ATOM    478  CD  GLN A  62       2.846   2.697 -43.531  1.00 90.06           C
ATOM    479  NE2 GLN A  62       2.303   1.749 -42.781  1.00 90.06           N
ATOM    480  OE1 GLN A  62       2.309   3.113 -44.562  1.00 90.06           O
ATOM    481  N   ASP A  63       6.953   4.051 -46.844  1.00 92.19           N
ATOM    482  CA  ASP A  63       7.352   4.828 -48.000  1.00 92.19           C
ATOM    483  C   ASP A  63       7.504   3.936 -49.219  1.00 92.19           C
ATOM    484  CB  ASP A  63       8.656   5.578 -47.719  1.00 92.19           C
ATOM    485  O   ASP A  63       7.816   4.418 -50.312  1.00 92.19           O
ATOM    486  CG  ASP A  63       9.828   4.645 -47.469  1.00 92.19           C
ATOM    487  OD1 ASP A  63       9.648   3.410 -47.500  1.00 92.19           O
ATOM    488  OD2 ASP A  63      10.953   5.152 -47.250  1.00 92.19           O
ATOM    489  N   GLY A  64       7.348   2.525 -49.125  1.00 90.31           N
ATOM    490  CA  GLY A  64       7.371   1.574 -50.219  1.00 90.31           C
ATOM    491  C   GLY A  64       8.695   0.845 -50.344  1.00 90.31           C
ATOM    492  O   GLY A  64       8.805  -0.118 -51.125  1.00 90.31           O
ATOM    493  N   SER A  65       9.750   1.246 -49.656  1.00 93.44           N
ATOM    494  CA  SER A  65      11.055   0.586 -49.688  1.00 93.44           C
ATOM    495  C   SER A  65      11.008  -0.757 -48.969  1.00 93.44           C
ATOM    496  CB  SER A  65      12.125   1.478 -49.062  1.00 93.44           C
ATOM    497  O   SER A  65      10.297  -0.910 -47.969  1.00 93.44           O
ATOM    498  OG  SER A  65      11.852   1.694 -47.688  1.00 93.44           O
ATOM    499  N   LEU A  66      11.781  -1.787 -49.531  1.00 93.56           N
ATOM    500  CA  LEU A  66      11.758  -3.145 -49.000  1.00 93.56           C
ATOM    501  C   LEU A  66      12.922  -3.363 -48.062  1.00 93.56           C
ATOM    502  CB  LEU A  66      11.820  -4.168 -50.156  1.00 93.56           C
ATOM    503  O   LEU A  66      14.070  -3.041 -48.375  1.00 93.56           O
ATOM    504  CG  LEU A  66      10.570  -4.289 -51.031  1.00 93.56           C
ATOM    505  CD1 LEU A  66      10.891  -5.074 -52.281  1.00 93.56           C
ATOM    506  CD2 LEU A  66       9.438  -4.949 -50.250  1.00 93.56           C
ATOM    507  N   ASN A  67      12.602  -3.799 -46.750  1.00 93.31           N
ATOM    508  CA  ASN A  67      13.633  -4.184 -45.812  1.00 93.31           C
```

ATOM	509	C	ASN	A	67	14.203	-5.566	-46.125	1.00	93.31	C
ATOM	510	CB	ASN	A	67	13.094	-4.145	-44.375	1.00	93.31	C
ATOM	511	O	ASN	A	67	15.352	-5.863	-45.781	1.00	93.31	O
ATOM	512	CG	ASN	A	67	12.891	-2.732	-43.875	1.00	93.31	C
ATOM	513	ND2	ASN	A	67	11.805	-2.518	-43.125	1.00	93.31	N
ATOM	514	OD1	ASN	A	67	13.703	-1.841	-44.125	1.00	93.31	O
ATOM	515	N	PHE	A	68	13.344	-6.422	-46.750	1.00	92.00	N
ATOM	516	CA	PHE	A	68	13.703	-7.777	-47.156	1.00	92.00	C
ATOM	517	C	PHE	A	68	13.398	-8.000	-48.625	1.00	92.00	C
ATOM	518	CB	PHE	A	68	12.953	-8.812	-46.312	1.00	92.00	C
ATOM	519	O	PHE	A	68	12.273	-7.770	-49.094	1.00	92.00	O
ATOM	520	CG	PHE	A	68	13.164	-8.641	-44.812	1.00	92.00	C
ATOM	521	CD1	PHE	A	68	14.281	-9.188	-44.188	1.00	92.00	C
ATOM	522	CD2	PHE	A	68	12.242	-7.934	-44.062	1.00	92.00	C
ATOM	523	CE1	PHE	A	68	14.477	-9.031	-42.812	1.00	92.00	C
ATOM	524	CE2	PHE	A	68	12.430	-7.773	-42.688	1.00	92.00	C
ATOM	525	CZ	PHE	A	68	13.547	-8.320	-42.062	1.00	92.00	C
ATOM	526	N	GLN	A	69	14.500	-8.422	-49.375	1.00	88.50	N
ATOM	527	CA	GLN	A	69	14.320	-8.680	-50.812	1.00	88.50	C
ATOM	528	C	GLN	A	69	13.859	-10.117	-51.062	1.00	88.50	C
ATOM	529	CB	GLN	A	69	15.617	-8.406	-51.562	1.00	88.50	C
ATOM	530	O	GLN	A	69	14.055	-10.992	-50.188	1.00	88.50	O
ATOM	531	CG	GLN	A	69	16.109	-6.977	-51.438	1.00	88.50	C
ATOM	532	CD	GLN	A	69	15.266	-5.984	-52.219	1.00	88.50	C
ATOM	533	NE2	GLN	A	69	15.281	-4.730	-51.781	1.00	88.50	N
ATOM	534	OE1	GLN	A	69	14.602	-6.340	-53.188	1.00	88.50	O
ATOM	535	N	ASN	A	70	13.031	-10.336	-52.125	1.00	87.06	N
ATOM	536	CA	ASN	A	70	12.594	-11.648	-52.562	1.00	87.06	C
ATOM	537	C	ASN	A	70	11.586	-12.273	-51.625	1.00	87.06	C
ATOM	538	CB	ASN	A	70	13.797	-12.578	-52.750	1.00	87.06	C
ATOM	539	O	ASN	A	70	11.664	-13.469	-51.312	1.00	87.06	O
ATOM	540	CG	ASN	A	70	14.758	-12.078	-53.812	1.00	87.06	C
ATOM	541	ND2	ASN	A	70	16.047	-12.266	-53.562	1.00	87.06	N
ATOM	542	OD1	ASN	A	70	14.344	-11.539	-54.844	1.00	87.06	O
ATOM	543	N	VAL	A	71	10.789	-11.461	-51.000	1.00	91.81	N
ATOM	544	CA	VAL	A	71	9.688	-11.914	-50.156	1.00	91.81	C
ATOM	545	C	VAL	A	71	8.375	-11.859	-50.938	1.00	91.81	C
ATOM	546	CB	VAL	A	71	9.570	-11.062	-48.875	1.00	91.81	C
ATOM	547	O	VAL	A	71	8.078	-10.852	-51.594	1.00	91.81	O
ATOM	548	CG1	VAL	A	71	8.359	-11.500	-48.031	1.00	91.81	C
ATOM	549	CG2	VAL	A	71	10.852	-11.172	-48.031	1.00	91.81	C
ATOM	550	N	LYS	A	72	7.691	-12.969	-51.000	1.00	90.88	N
ATOM	551	CA	LYS	A	72	6.367	-13.062	-51.594	1.00	90.88	C
ATOM	552	C	LYS	A	72	5.312	-13.453	-50.562	1.00	90.88	C
ATOM	553	CB	LYS	A	72	6.371	-14.078	-52.750	1.00	90.88	C
ATOM	554	O	LYS	A	72	5.543	-14.336	-49.750	1.00	90.88	O
ATOM	555	CG	LYS	A	72	5.043	-14.188	-53.469	1.00	90.88	C
ATOM	556	CD	LYS	A	72	5.098	-15.242	-54.562	1.00	90.88	C
ATOM	557	CE	LYS	A	72	3.744	-15.414	-55.250	1.00	90.88	C
ATOM	558	NZ	LYS	A	72	3.783	-16.469	-56.312	1.00	90.88	N
ATOM	559	N	ALA	A	73	4.125	-12.750	-50.531	1.00	93.44	N
ATOM	560	CA	ALA	A	73	3.021	-13.039	-49.625	1.00	93.44	C
ATOM	561	C	ALA	A	73	1.679	-12.953	-50.344	1.00	93.44	C
ATOM	562	CB	ALA	A	73	3.047	-12.086	-48.438	1.00	93.44	C
ATOM	563	O	ALA	A	73	1.482	-12.094	-51.188	1.00	93.44	O
ATOM	564	N	GLU	A	74	0.828	-13.922	-50.094	1.00	92.62	N
ATOM	565	CA	GLU	A	74	-0.556	-13.945	-50.562	1.00	92.62	C
ATOM	566	C	GLU	A	74	-1.532	-13.992	-49.375	1.00	92.62	C
ATOM	567	CB	GLU	A	74	-0.792	-15.133	-51.469	1.00	92.62	C
ATOM	568	O	GLU	A	74	-1.257	-14.625	-48.375	1.00	92.62	O
ATOM	569	CG	GLU	A	74	0.052	-15.102	-52.750	1.00	92.62	C
ATOM	570	CD	GLU	A	74	-0.217	-16.281	-53.688	1.00	92.62	C
ATOM	571	OE1	GLU	A	74	0.698	-16.672	-54.438	1.00	92.62	O
ATOM	572	OE2	GLU	A	74	-1.353	-16.797	-53.656	1.00	92.62	O

ATOM	573	N	PHE	A	75	-2.658	-13.312	-49.531	1.00	94.44	N
ATOM	574	CA	PHE	A	75	-3.635	-13.234	-48.438	1.00	94.44	C
ATOM	575	C	PHE	A	75	-5.012	-13.672	-48.938	1.00	94.44	C
ATOM	576	CB	PHE	A	75	-3.703	-11.805	-47.906	1.00	94.44	C
ATOM	577	O	PHE	A	75	-5.453	-13.273	-50.000	1.00	94.44	O
ATOM	578	CG	PHE	A	75	-4.699	-11.641	-46.781	1.00	94.44	C
ATOM	579	CD1	PHE	A	75	-5.836	-10.859	-46.969	1.00	94.44	C
ATOM	580	CD2	PHE	A	75	-4.504	-12.258	-45.562	1.00	94.44	C
ATOM	581	CE1	PHE	A	75	-6.762	-10.703	-45.938	1.00	94.44	C
ATOM	582	CE2	PHE	A	75	-5.422	-12.109	-44.531	1.00	94.44	C
ATOM	583	CZ	PHE	A	75	-6.551	-11.328	-44.719	1.00	94.44	C
ATOM	584	N	ARG	A	76	-5.602	-14.617	-48.250	1.00	94.75	N
ATOM	585	CA	ARG	A	76	-7.012	-14.953	-48.406	1.00	94.75	C
ATOM	586	C	ARG	A	76	-7.836	-14.461	-47.219	1.00	94.75	C
ATOM	587	CB	ARG	A	76	-7.188	-16.469	-48.562	1.00	94.75	C
ATOM	588	O	ARG	A	76	-7.574	-14.820	-46.094	1.00	94.75	O
ATOM	589	CG	ARG	A	76	-6.449	-17.047	-49.750	1.00	94.75	C
ATOM	590	CD	ARG	A	76	-7.035	-16.562	-51.094	1.00	94.75	C
ATOM	591	NE	ARG	A	76	-6.355	-17.156	-52.219	1.00	94.75	N
ATOM	592	NH1	ARG	A	76	-4.680	-15.562	-52.312	1.00	94.75	N
ATOM	593	NH2	ARG	A	76	-4.719	-17.281	-53.844	1.00	94.75	N
ATOM	594	CZ	ARG	A	76	-5.254	-16.672	-52.781	1.00	94.75	C
ATOM	595	N	PRO	A	77	-8.781	-13.602	-47.438	1.00	94.69	N
ATOM	596	CA	PRO	A	77	-9.516	-12.922	-46.375	1.00	94.69	C
ATOM	597	C	PRO	A	77	-10.508	-13.844	-45.656	1.00	94.69	C
ATOM	598	CB	PRO	A	77	-10.242	-11.797	-47.125	1.00	94.69	C
ATOM	599	O	PRO	A	77	-11.094	-13.461	-44.656	1.00	94.69	O
ATOM	600	CG	PRO	A	77	-10.406	-12.289	-48.500	1.00	94.69	C
ATOM	601	CD	PRO	A	77	-9.250	-13.195	-48.844	1.00	94.69	C
ATOM	602	N	GLY	A	78	-10.664	-15.086	-46.125	1.00	93.38	N
ATOM	603	CA	GLY	A	78	-11.547	-16.031	-45.438	1.00	93.38	C
ATOM	604	C	GLY	A	78	-12.984	-15.938	-45.906	1.00	93.38	C
ATOM	605	O	GLY	A	78	-13.914	-16.141	-45.125	1.00	93.38	O
ATOM	606	N	THR	A	79	-13.273	-15.562	-47.125	1.00	93.56	N
ATOM	607	CA	THR	A	79	-14.617	-15.578	-47.719	1.00	93.56	C
ATOM	608	C	THR	A	79	-15.070	-17.016	-47.969	1.00	93.56	C
ATOM	609	CB	THR	A	79	-14.680	-14.766	-49.000	1.00	93.56	C
ATOM	610	O	THR	A	79	-14.250	-17.922	-48.031	1.00	93.56	O
ATOM	611	CG2	THR	A	79	-14.164	-13.344	-48.812	1.00	93.56	C
ATOM	612	OG1	THR	A	79	-13.867	-15.414	-50.000	1.00	93.56	O
ATOM	613	N	GLN	A	80	-16.391	-17.156	-48.000	1.00	90.88	N
ATOM	614	CA	GLN	A	80	-16.969	-18.469	-48.250	1.00	90.88	C
ATOM	615	C	GLN	A	80	-16.469	-19.047	-49.562	1.00	90.88	C
ATOM	616	CB	GLN	A	80	-18.500	-18.406	-48.250	1.00	90.88	C
ATOM	617	O	GLN	A	80	-16.234	-20.250	-49.688	1.00	90.88	O
ATOM	618	CG	GLN	A	80	-19.172	-19.766	-48.375	1.00	90.88	C
ATOM	619	CD	GLN	A	80	-18.969	-20.641	-47.156	1.00	90.88	C
ATOM	620	NE2	GLN	A	80	-19.297	-21.922	-47.281	1.00	90.88	N
ATOM	621	OE1	GLN	A	80	-18.516	-20.172	-46.094	1.00	90.88	O
ATOM	622	N	THR	A	81	-16.188	-18.203	-50.562	1.00	91.12	N
ATOM	623	CA	THR	A	81	-15.859	-18.656	-51.906	1.00	91.12	C
ATOM	624	C	THR	A	81	-14.438	-18.250	-52.281	1.00	91.12	C
ATOM	625	CB	THR	A	81	-16.844	-18.078	-52.938	1.00	91.12	C
ATOM	626	O	THR	A	81	-14.133	-18.047	-53.469	1.00	91.12	O
ATOM	627	CG2	THR	A	81	-18.266	-18.609	-52.719	1.00	91.12	C
ATOM	628	OG1	THR	A	81	-16.859	-16.656	-52.844	1.00	91.12	O
ATOM	629	N	GLN	A	82	-13.492	-18.078	-51.250	1.00	92.06	N
ATOM	630	CA	GLN	A	82	-12.133	-17.656	-51.562	1.00	92.06	C
ATOM	631	C	GLN	A	82	-11.359	-18.766	-52.281	1.00	92.06	C
ATOM	632	CB	GLN	A	82	-11.398	-17.250	-50.281	1.00	92.06	C
ATOM	633	O	GLN	A	82	-11.664	-19.953	-52.094	1.00	92.06	O
ATOM	634	CG	GLN	A	82	-11.219	-18.391	-49.281	1.00	92.06	C
ATOM	635	CD	GLN	A	82	-10.641	-17.938	-47.938	1.00	92.06	C
ATOM	636	NE2	GLN	A	82	-10.789	-18.750	-46.906	1.00	92.06	N

ATOM	637	OE1	GLN	A	82	-10.055	-16.859	-47.844	1.00	92.06	O
ATOM	638	N	ASP	A	83	-10.414	-18.375	-53.156	1.00	87.56	N
ATOM	639	CA	ASP	A	83	-9.633	-19.328	-53.938	1.00	87.56	C
ATOM	640	C	ASP	A	83	-8.562	-20.000	-53.062	1.00	87.56	C
ATOM	641	CB	ASP	A	83	-9.000	-18.641	-55.125	1.00	87.56	C
ATOM	642	O	ASP	A	83	-8.219	-19.500	-52.000	1.00	87.56	O
ATOM	643	CG	ASP	A	83	-10.008	-18.234	-56.188	1.00	87.56	C
ATOM	644	OD1	ASP	A	83	-11.055	-18.906	-56.312	1.00	87.56	O
ATOM	645	OD2	ASP	A	83	-9.758	-17.250	-56.906	1.00	87.56	O
ATOM	646	N	TYR	A	84	-8.078	-21.234	-53.438	1.00	87.25	N
ATOM	647	CA	TYR	A	84	-6.988	-21.906	-52.719	1.00	87.25	C
ATOM	648	C	TYR	A	84	-5.652	-21.234	-53.031	1.00	87.25	C
ATOM	649	CB	TYR	A	84	-6.926	-23.375	-53.125	1.00	87.25	C
ATOM	650	O	TYR	A	84	-5.539	-20.438	-53.969	1.00	87.25	O
ATOM	651	CG	TYR	A	84	-6.312	-23.625	-54.469	1.00	87.25	C
ATOM	652	CD1	TYR	A	84	-7.051	-23.438	-55.625	1.00	87.25	C
ATOM	653	CD2	TYR	A	84	-4.988	-24.031	-54.594	1.00	87.25	C
ATOM	654	CE1	TYR	A	84	-6.488	-23.656	-56.906	1.00	87.25	C
ATOM	655	CE2	TYR	A	84	-4.418	-24.266	-55.844	1.00	87.25	C
ATOM	656	OH	TYR	A	84	-4.613	-24.297	-58.219	1.00	87.25	O
ATOM	657	CZ	TYR	A	84	-5.176	-24.078	-56.969	1.00	87.25	C
ATOM	658	N	ILE	A	85	-4.703	-21.312	-52.125	1.00	87.50	N
ATOM	659	CA	ILE	A	85	-3.352	-20.797	-52.344	1.00	87.50	C
ATOM	660	C	ILE	A	85	-2.572	-21.750	-53.219	1.00	87.50	C
ATOM	661	CB	ILE	A	85	-2.607	-20.594	-51.000	1.00	87.50	C
ATOM	662	O	ILE	A	85	-2.385	-22.922	-52.906	1.00	87.50	O
ATOM	663	CG1	ILE	A	85	-3.295	-19.516	-50.156	1.00	87.50	C
ATOM	664	CG2	ILE	A	85	-1.136	-20.250	-51.250	1.00	87.50	C
ATOM	665	CD1	ILE	A	85	-2.492	-19.062	-48.938	1.00	87.50	C
ATOM	666	N	GLN	A	86	-2.176	-21.234	-54.469	1.00	84.81	N
ATOM	667	CA	GLN	A	86	-1.466	-22.062	-55.438	1.00	84.81	C
ATOM	668	C	GLN	A	86	-0.025	-22.312	-55.000	1.00	84.81	C
ATOM	669	CB	GLN	A	86	-1.489	-21.406	-56.812	1.00	84.81	C
ATOM	670	O	GLN	A	86	0.617	-21.422	-54.438	1.00	84.81	O
ATOM	671	CG	GLN	A	86	-2.881	-21.312	-57.438	1.00	84.81	C
ATOM	672	CD	GLN	A	86	-2.883	-20.625	-58.781	1.00	84.81	C
ATOM	673	NE2	GLN	A	86	-3.998	-20.719	-59.500	1.00	84.81	N
ATOM	674	OE1	GLN	A	86	-1.889	-20.000	-59.188	1.00	84.81	O
ATOM	675	N	GLY	A	87	0.453	-23.516	-55.031	1.00	77.69	N
ATOM	676	CA	GLY	A	87	1.866	-23.781	-54.812	1.00	77.69	C
ATOM	677	C	GLY	A	87	2.170	-24.266	-53.406	1.00	77.69	C
ATOM	678	O	GLY	A	87	3.334	-24.438	-53.062	1.00	77.69	O
ATOM	679	N	PHE	A	88	1.160	-24.469	-52.469	1.00	74.12	N
ATOM	680	CA	PHE	A	88	1.355	-24.891	-51.062	1.00	74.12	C
ATOM	681	C	PHE	A	88	0.805	-26.297	-50.844	1.00	74.12	C
ATOM	682	CB	PHE	A	88	0.686	-23.906	-50.125	1.00	74.12	C
ATOM	683	O	PHE	A	88	-0.367	-26.562	-51.125	1.00	74.12	O
ATOM	684	CG	PHE	A	88	1.115	-24.062	-48.688	1.00	74.12	C
ATOM	685	CD1	PHE	A	88	0.299	-24.719	-47.750	1.00	74.12	C
ATOM	686	CD2	PHE	A	88	2.338	-23.562	-48.250	1.00	74.12	C
ATOM	687	CE1	PHE	A	88	0.695	-24.859	-46.438	1.00	74.12	C
ATOM	688	CE2	PHE	A	88	2.740	-23.703	-46.938	1.00	74.12	C
ATOM	689	CZ	PHE	A	88	1.917	-24.359	-46.031	1.00	74.12	C
ATOM	690	N	THR	A	89	1.673	-27.359	-50.688	1.00	68.56	N
ATOM	691	CA	THR	A	89	1.389	-28.781	-50.562	1.00	68.56	C
ATOM	692	C	THR	A	89	0.885	-29.375	-51.875	1.00	68.56	C
ATOM	693	CB	THR	A	89	0.349	-29.031	-49.438	1.00	68.56	C
ATOM	694	O	THR	A	89	-0.187	-29.000	-52.344	1.00	68.56	O
ATOM	695	CG2	THR	A	89	0.491	-30.438	-48.875	1.00	68.56	C
ATOM	696	OG1	THR	A	89	0.537	-28.078	-48.406	1.00	68.56	O
ATOM	697	N	ASP	A	90	1.809	-30.141	-52.625	1.00	76.69	N
ATOM	698	CA	ASP	A	90	1.508	-30.703	-53.938	1.00	76.69	C
ATOM	699	C	ASP	A	90	2.035	-32.125	-54.062	1.00	76.69	C
ATOM	700	CB	ASP	A	90	2.102	-29.812	-55.062	1.00	76.69	C

ATOM	701	O	ASP	A	90	2.719	-32.625	-53.188	1.00	76.69	O
ATOM	702	CG	ASP	A	90	3.619	-29.766	-55.031	1.00	76.69	C
ATOM	703	OD1	ASP	A	90	4.266	-30.828	-54.906	1.00	76.69	O
ATOM	704	OD2	ASP	A	90	4.172	-28.641	-55.094	1.00	76.69	O
ATOM	705	N	THR	A	91	1.431	-32.875	-54.906	1.00	70.88	N
ATOM	706	CA	THR	A	91	1.965	-34.125	-55.438	1.00	70.88	C
ATOM	707	C	THR	A	91	2.428	-33.938	-56.875	1.00	70.88	C
ATOM	708	CB	THR	A	91	0.920	-35.250	-55.375	1.00	70.88	C
ATOM	709	O	THR	A	91	1.785	-33.219	-57.656	1.00	70.88	O
ATOM	710	CG2	THR	A	91	1.522	-36.562	-55.812	1.00	70.88	C
ATOM	711	OG1	THR	A	91	0.440	-35.375	-54.031	1.00	70.88	O
ATOM	712	N	ALA	A	92	3.678	-34.281	-57.125	1.00	79.38	N
ATOM	713	CA	ALA	A	92	4.227	-34.031	-58.438	1.00	79.38	C
ATOM	714	C	ALA	A	92	4.750	-35.312	-59.062	1.00	79.38	C
ATOM	715	CB	ALA	A	92	5.336	-32.969	-58.375	1.00	79.38	C
ATOM	716	O	ALA	A	92	5.145	-36.250	-58.375	1.00	79.38	O
ATOM	717	N	SER	A	93	4.523	-35.562	-60.312	1.00	80.06	N
ATOM	718	CA	SER	A	93	5.094	-36.594	-61.156	1.00	80.06	C
ATOM	719	C	SER	A	93	6.078	-36.031	-62.156	1.00	80.06	C
ATOM	720	CB	SER	A	93	3.990	-37.375	-61.875	1.00	80.06	C
ATOM	721	O	SER	A	93	5.727	-35.125	-62.938	1.00	80.06	O
ATOM	722	OG	SER	A	93	4.539	-38.375	-62.688	1.00	80.06	O
ATOM	723	N	GLU	A	94	7.324	-36.375	-62.000	1.00	84.50	N
ATOM	724	CA	GLU	A	94	8.398	-35.812	-62.812	1.00	84.50	C
ATOM	725	C	GLU	A	94	8.570	-36.531	-64.125	1.00	84.50	C
ATOM	726	CB	GLU	A	94	9.719	-35.812	-62.031	1.00	84.50	C
ATOM	727	O	GLU	A	94	8.547	-37.781	-64.188	1.00	84.50	O
ATOM	728	CG	GLU	A	94	10.797	-34.938	-62.625	1.00	84.50	C
ATOM	729	CD	GLU	A	94	11.969	-34.688	-61.688	1.00	84.50	C
ATOM	730	OE1	GLU	A	94	12.734	-33.719	-61.906	1.00	84.50	O
ATOM	731	OE2	GLU	A	94	12.133	-35.469	-60.750	1.00	84.50	O
ATOM	732	N	VAL	A	95	8.641	-35.875	-65.188	1.00	83.44	N
ATOM	733	CA	VAL	A	95	8.992	-36.375	-66.500	1.00	83.44	C
ATOM	734	C	VAL	A	95	10.391	-35.875	-66.875	1.00	83.44	C
ATOM	735	CB	VAL	A	95	7.961	-35.906	-67.562	1.00	83.44	C
ATOM	736	O	VAL	A	95	10.641	-34.656	-66.938	1.00	83.44	O
ATOM	737	CG1	VAL	A	95	8.352	-36.406	-68.938	1.00	83.44	C
ATOM	738	CG2	VAL	A	95	6.562	-36.375	-67.188	1.00	83.44	C
ATOM	739	N	THR	A	96	11.336	-36.875	-67.000	1.00	84.44	N
ATOM	740	CA	THR	A	96	12.711	-36.562	-67.312	1.00	84.44	C
ATOM	741	C	THR	A	96	12.828	-36.281	-68.812	1.00	84.44	C
ATOM	742	CB	THR	A	96	13.680	-37.688	-66.938	1.00	84.44	C
ATOM	743	O	THR	A	96	12.352	-37.062	-69.625	1.00	84.44	O
ATOM	744	CG2	THR	A	96	15.125	-37.312	-67.250	1.00	84.44	C
ATOM	745	OG1	THR	A	96	13.531	-38.000	-65.562	1.00	84.44	O
ATOM	746	N	VAL	A	97	13.359	-35.188	-69.312	1.00	86.75	N
ATOM	747	CA	VAL	A	97	13.469	-34.750	-70.688	1.00	86.75	C
ATOM	748	C	VAL	A	97	14.930	-34.781	-71.125	1.00	86.75	C
ATOM	749	CB	VAL	A	97	12.891	-33.312	-70.875	1.00	86.75	C
ATOM	750	O	VAL	A	97	15.305	-35.562	-72.000	1.00	86.75	O
ATOM	751	CG1	VAL	A	97	13.000	-32.875	-72.312	1.00	86.75	C
ATOM	752	CG2	VAL	A	97	11.438	-33.281	-70.375	1.00	86.75	C
ATOM	753	N	ALA	A	98	15.922	-33.938	-70.438	1.00	86.00	N
ATOM	754	CA	ALA	A	98	17.359	-33.812	-70.625	1.00	86.00	C
ATOM	755	C	ALA	A	98	17.656	-33.281	-72.062	1.00	86.00	C
ATOM	756	CB	ALA	A	98	18.047	-35.156	-70.438	1.00	86.00	C
ATOM	757	O	ALA	A	98	18.531	-33.812	-72.750	1.00	86.00	O
ATOM	758	N	ARG	A	99	16.891	-32.188	-72.562	1.00	89.62	N
ATOM	759	CA	ARG	A	99	17.047	-31.609	-73.938	1.00	89.62	C
ATOM	760	C	ARG	A	99	17.812	-30.281	-73.812	1.00	89.62	C
ATOM	761	CB	ARG	A	99	15.688	-31.391	-74.562	1.00	89.62	C
ATOM	762	O	ARG	A	99	17.500	-29.438	-73.000	1.00	89.62	O
ATOM	763	CG	ARG	A	99	15.758	-31.078	-76.062	1.00	89.62	C
ATOM	764	CD	ARG	A	99	14.477	-31.484	-76.750	1.00	89.62	C

```
ATOM    765  NE  ARG A  99      14.500 -31.078 -78.188  1.00 89.62           N
ATOM    766  NH1 ARG A  99      12.398 -31.891 -78.688  1.00 89.62           N
ATOM    767  NH2 ARG A  99      13.641 -30.859 -80.312  1.00 89.62           N
ATOM    768  CZ  ARG A  99      13.516 -31.281 -79.062  1.00 89.62           C
ATOM    769  N   ASP A 100      18.797 -30.000 -74.750  1.00 90.38           N
ATOM    770  CA  ASP A 100      19.531 -28.734 -74.875  1.00 90.38           C
ATOM    771  C   ASP A 100      18.641 -27.641 -75.438  1.00 90.38           C
ATOM    772  CB  ASP A 100      20.781 -28.906 -75.750  1.00 90.38           C
ATOM    773  O   ASP A 100      17.922 -27.844 -76.375  1.00 90.38           O
ATOM    774  CG  ASP A 100      21.812 -29.828 -75.125  1.00 90.38           C
ATOM    775  OD1 ASP A 100      21.656 -30.219 -73.938  1.00 90.38           O
ATOM    776  OD2 ASP A 100      22.812 -30.156 -75.812  1.00 90.38           O
ATOM    777  N   LEU A 101      18.672 -26.594 -74.688  1.00 90.69           N
ATOM    778  CA  LEU A 101      17.906 -25.438 -75.125  1.00 90.69           C
ATOM    779  C   LEU A 101      18.828 -24.438 -75.812  1.00 90.69           C
ATOM    780  CB  LEU A 101      17.188 -24.766 -74.000  1.00 90.69           C
ATOM    781  O   LEU A 101      19.844 -24.016 -75.250  1.00 90.69           O
ATOM    782  CG  LEU A 101      16.062 -25.578 -73.312  1.00 90.69           C
ATOM    783  CD1 LEU A 101      15.586 -24.875 -72.062  1.00 90.69           C
ATOM    784  CD2 LEU A 101      14.906 -25.781 -74.312  1.00 90.69           C
ATOM    785  N   THR A 102      18.547 -24.109 -77.188  1.00 86.88           N
ATOM    786  CA  THR A 102      19.172 -23.016 -77.938  1.00 86.88           C
ATOM    787  C   THR A 102      18.125 -22.000 -78.375  1.00 86.88           C
ATOM    788  CB  THR A 102      19.938 -23.562 -79.188  1.00 86.88           C
ATOM    789  O   THR A 102      16.922 -22.281 -78.312  1.00 86.88           O
ATOM    790  CG2 THR A 102      20.938 -24.641 -78.750  1.00 86.88           C
ATOM    791  OG1 THR A 102      19.000 -24.109 -80.125  1.00 86.88           O
ATOM    792  N   ALA A 103      18.656 -20.797 -78.625  1.00 84.06           N
ATOM    793  CA  ALA A 103      17.734 -19.766 -79.062  1.00 84.06           C
ATOM    794  C   ALA A 103      16.875 -20.281 -80.250  1.00 84.06           C
ATOM    795  CB  ALA A 103      18.500 -18.516 -79.500  1.00 84.06           C
ATOM    796  O   ALA A 103      15.703 -19.906 -80.312  1.00 84.06           O
ATOM    797  N   ALA A 104      17.281 -21.266 -81.062  1.00 85.00           N
ATOM    798  CA  ALA A 104      16.594 -21.781 -82.250  1.00 85.00           C
ATOM    799  C   ALA A 104      15.750 -23.000 -81.938  1.00 85.00           C
ATOM    800  CB  ALA A 104      17.594 -22.125 -83.375  1.00 85.00           C
ATOM    801  O   ALA A 104      14.805 -23.328 -82.625  1.00 85.00           O
ATOM    802  N   THR A 105      16.047 -23.641 -80.750  1.00 88.69           N
ATOM    803  CA  THR A 105      15.391 -24.922 -80.500  1.00 88.69           C
ATOM    804  C   THR A 105      14.703 -24.875 -79.125  1.00 88.69           C
ATOM    805  CB  THR A 105      16.406 -26.078 -80.500  1.00 88.69           C
ATOM    806  O   THR A 105      15.289 -25.281 -78.125  1.00 88.69           O
ATOM    807  CG2 THR A 105      16.938 -26.297 -81.938  1.00 88.69           C
ATOM    808  OG1 THR A 105      17.484 -25.797 -79.625  1.00 88.69           O
ATOM    809  N   PRO A 106      13.453 -24.328 -79.062  1.00 92.25           N
ATOM    810  CA  PRO A 106      12.695 -24.359 -77.812  1.00 92.25           C
ATOM    811  C   PRO A 106      12.109 -25.734 -77.500  1.00 92.25           C
ATOM    812  CB  PRO A 106      11.578 -23.328 -78.062  1.00 92.25           C
ATOM    813  O   PRO A 106      12.070 -26.594 -78.375  1.00 92.25           O
ATOM    814  CG  PRO A 106      11.305 -23.406 -79.500  1.00 92.25           C
ATOM    815  CD  PRO A 106      12.594 -23.672 -80.250  1.00 92.25           C
ATOM    816  N   TYR A 107      11.734 -26.062 -76.312  1.00 93.69           N
ATOM    817  CA  TYR A 107      11.023 -27.266 -75.875  1.00 93.69           C
ATOM    818  C   TYR A 107       9.578 -26.969 -75.500  1.00 93.69           C
ATOM    819  CB  TYR A 107      11.734 -27.938 -74.688  1.00 93.69           C
ATOM    820  O   TYR A 107       9.312 -26.047 -74.750  1.00 93.69           O
ATOM    821  CG  TYR A 107      11.156 -29.281 -74.312  1.00 93.69           C
ATOM    822  CD1 TYR A 107      10.352 -29.422 -73.188  1.00 93.69           C
ATOM    823  CD2 TYR A 107      11.422 -30.406 -75.062  1.00 93.69           C
ATOM    824  CE1 TYR A 107       9.812 -30.656 -72.875  1.00 93.69           C
ATOM    825  CE2 TYR A 107      10.891 -31.641 -74.750  1.00 93.69           C
ATOM    826  OH  TYR A 107       9.570 -33.000 -73.312  1.00 93.69           O
ATOM    827  CZ  TYR A 107      10.094 -31.766 -73.625  1.00 93.69           C
ATOM    828  N   ILE A 108       8.609 -27.719 -76.188  1.00 93.69           N
```

ATOM	829	CA	ILE	A	108	7.191	-27.453	-75.938	1.00	93.69	C
ATOM	830	C	ILE	A	108	6.590	-28.562	-75.062	1.00	93.69	C
ATOM	831	CB	ILE	A	108	6.430	-27.344	-77.312	1.00	93.69	C
ATOM	832	O	ILE	A	108	6.734	-29.750	-75.375	1.00	93.69	O
ATOM	833	CG1	ILE	A	108	6.984	-26.188	-78.125	1.00	93.69	C
ATOM	834	CG2	ILE	A	108	4.926	-27.172	-77.062	1.00	93.69	C
ATOM	835	CD1	ILE	A	108	6.406	-26.109	-79.500	1.00	93.69	C
ATOM	836	N	ILE	A	109	6.031	-28.203	-73.938	1.00	93.62	N
ATOM	837	CA	ILE	A	109	5.277	-29.094	-73.062	1.00	93.62	C
ATOM	838	C	ILE	A	109	3.785	-28.953	-73.375	1.00	93.62	C
ATOM	839	CB	ILE	A	109	5.566	-28.812	-71.562	1.00	93.62	C
ATOM	840	O	ILE	A	109	3.225	-27.859	-73.250	1.00	93.62	O
ATOM	841	CG1	ILE	A	109	7.074	-28.891	-71.312	1.00	93.62	C
ATOM	842	CG2	ILE	A	109	4.793	-29.781	-70.688	1.00	93.62	C
ATOM	843	CD1	ILE	A	109	7.492	-28.281	-70.000	1.00	93.62	C
ATOM	844	N	SER	A	110	3.121	-30.047	-73.812	1.00	92.69	N
ATOM	845	CA	SER	A	110	1.682	-30.078	-74.062	1.00	92.69	C
ATOM	846	C	SER	A	110	0.937	-30.672	-72.812	1.00	92.69	C
ATOM	847	CB	SER	A	110	1.358	-30.875	-75.312	1.00	92.69	C
ATOM	848	O	SER	A	110	1.184	-31.812	-72.438	1.00	92.69	O
ATOM	849	OG	SER	A	110	-0.040	-30.922	-75.500	1.00	92.69	O
ATOM	850	N	VAL	A	111	0.101	-29.844	-72.250	1.00	92.44	N
ATOM	851	CA	VAL	A	111	-0.683	-30.281	-71.125	1.00	92.44	C
ATOM	852	C	VAL	A	111	-2.148	-30.438	-71.500	1.00	92.44	C
ATOM	853	CB	VAL	A	111	-0.554	-29.281	-69.938	1.00	92.44	C
ATOM	854	O	VAL	A	111	-2.797	-29.469	-71.875	1.00	92.44	O
ATOM	855	CG1	VAL	A	111	-1.337	-29.781	-68.688	1.00	92.44	C
ATOM	856	CG2	VAL	A	111	0.916	-29.078	-69.562	1.00	92.44	C
ATOM	857	N	THR	A	112	-2.709	-31.719	-71.375	1.00	90.19	N
ATOM	858	CA	THR	A	112	-4.105	-31.984	-71.688	1.00	90.19	C
ATOM	859	C	THR	A	112	-4.895	-32.344	-70.438	1.00	90.19	C
ATOM	860	CB	THR	A	112	-4.230	-33.094	-72.750	1.00	90.19	C
ATOM	861	O	THR	A	112	-6.125	-32.281	-70.438	1.00	90.19	O
ATOM	862	CG2	THR	A	112	-3.623	-32.688	-74.062	1.00	90.19	C
ATOM	863	OG1	THR	A	112	-3.551	-34.281	-72.250	1.00	90.19	O
ATOM	864	N	ASN	A	113	-4.133	-32.750	-69.375	1.00	88.81	N
ATOM	865	CA	ASN	A	113	-4.746	-33.125	-68.062	1.00	88.81	C
ATOM	866	C	ASN	A	113	-5.262	-31.906	-67.312	1.00	88.81	C
ATOM	867	CB	ASN	A	113	-3.756	-33.906	-67.188	1.00	88.81	C
ATOM	868	O	ASN	A	113	-4.473	-31.094	-66.875	1.00	88.81	O
ATOM	869	CG	ASN	A	113	-4.391	-34.469	-65.938	1.00	88.81	C
ATOM	870	ND2	ASN	A	113	-3.709	-35.406	-65.312	1.00	88.81	N
ATOM	871	OD1	ASN	A	113	-5.488	-34.062	-65.562	1.00	88.81	O
ATOM	872	N	LYS	A	114	-6.547	-31.719	-67.188	1.00	87.62	N
ATOM	873	CA	LYS	A	114	-7.195	-30.562	-66.562	1.00	87.62	C
ATOM	874	C	LYS	A	114	-7.020	-30.547	-65.062	1.00	87.62	C
ATOM	875	CB	LYS	A	114	-8.680	-30.531	-66.938	1.00	87.62	C
ATOM	876	O	LYS	A	114	-7.332	-29.562	-64.375	1.00	87.62	O
ATOM	877	CG	LYS	A	114	-8.953	-30.328	-68.438	1.00	87.62	C
ATOM	878	CD	LYS	A	114	-10.453	-30.297	-68.688	1.00	87.62	C
ATOM	879	CE	LYS	A	114	-10.727	-30.281	-70.188	1.00	87.62	C
ATOM	880	NZ	LYS	A	114	-12.195	-30.312	-70.500	1.00	87.62	N
ATOM	881	N	ASN	A	115	-6.539	-31.719	-64.500	1.00	87.00	N
ATOM	882	CA	ASN	A	115	-6.371	-31.844	-63.094	1.00	87.00	C
ATOM	883	C	ASN	A	115	-5.043	-31.234	-62.625	1.00	87.00	C
ATOM	884	CB	ASN	A	115	-6.480	-33.312	-62.656	1.00	87.00	C
ATOM	885	O	ASN	A	115	-4.816	-31.078	-61.438	1.00	87.00	O
ATOM	886	CG	ASN	A	115	-7.875	-33.875	-62.844	1.00	87.00	C
ATOM	887	ND2	ASN	A	115	-7.957	-35.156	-63.094	1.00	87.00	N
ATOM	888	OD1	ASN	A	115	-8.867	-33.125	-62.750	1.00	87.00	O
ATOM	889	N	LEU	A	116	-4.137	-30.922	-63.562	1.00	89.25	N
ATOM	890	CA	LEU	A	116	-2.824	-30.375	-63.219	1.00	89.25	C
ATOM	891	C	LEU	A	116	-2.930	-28.906	-62.812	1.00	89.25	C
ATOM	892	CB	LEU	A	116	-1.859	-30.531	-64.438	1.00	89.25	C

ATOM	893	O	LEU	A	116	-3.652	-28.141	-63.469	1.00	89.25	O
ATOM	894	CG	LEU	A	116	-1.551	-31.953	-64.875	1.00	89.25	C
ATOM	895	CD1	LEU	A	116	-0.597	-31.938	-66.062	1.00	89.25	C
ATOM	896	CD2	LEU	A	116	-0.964	-32.750	-63.719	1.00	89.25	C
ATOM	897	N	SER	A	117	-2.291	-28.594	-61.750	1.00	90.69	N
ATOM	898	CA	SER	A	117	-2.314	-27.219	-61.219	1.00	90.69	C
ATOM	899	C	SER	A	117	-1.173	-26.391	-61.812	1.00	90.69	C
ATOM	900	CB	SER	A	117	-2.230	-27.234	-59.688	1.00	90.69	C
ATOM	901	O	SER	A	117	-1.318	-25.188	-62.000	1.00	90.69	O
ATOM	902	OG	SER	A	117	-3.291	-27.984	-59.156	1.00	90.69	O
ATOM	903	N	ALA	A	118	-0.126	-27.016	-62.094	1.00	92.44	N
ATOM	904	CA	ALA	A	118	1.055	-26.297	-62.594	1.00	92.44	C
ATOM	905	C	ALA	A	118	2.049	-27.266	-63.219	1.00	92.44	C
ATOM	906	CB	ALA	A	118	1.720	-25.531	-61.469	1.00	92.44	C
ATOM	907	O	ALA	A	118	1.919	-28.484	-63.094	1.00	92.44	O
ATOM	908	N	ILE	A	119	2.912	-26.672	-63.969	1.00	93.25	N
ATOM	909	CA	ILE	A	119	4.102	-27.344	-64.500	1.00	93.25	C
ATOM	910	C	ILE	A	119	5.352	-26.734	-63.844	1.00	93.25	C
ATOM	911	CB	ILE	A	119	4.172	-27.281	-66.000	1.00	93.25	C
ATOM	912	O	ILE	A	119	5.523	-25.516	-63.875	1.00	93.25	O
ATOM	913	CG1	ILE	A	119	2.992	-28.031	-66.625	1.00	93.25	C
ATOM	914	CG2	ILE	A	119	5.508	-27.828	-66.500	1.00	93.25	C
ATOM	915	CD1	ILE	A	119	3.020	-29.531	-66.375	1.00	93.25	C
ATOM	916	N	ARG	A	120	6.133	-27.656	-63.188	1.00	93.31	N
ATOM	917	CA	ARG	A	120	7.422	-27.203	-62.656	1.00	93.31	C
ATOM	918	C	ARG	A	120	8.562	-27.625	-63.594	1.00	93.31	C
ATOM	919	CB	ARG	A	120	7.656	-27.750	-61.250	1.00	93.31	C
ATOM	920	O	ARG	A	120	8.773	-28.828	-63.781	1.00	93.31	O
ATOM	921	CG	ARG	A	120	8.891	-27.172	-60.562	1.00	93.31	C
ATOM	922	CD	ARG	A	120	9.102	-27.781	-59.188	1.00	93.31	C
ATOM	923	NE	ARG	A	120	7.984	-27.516	-58.281	1.00	93.31	N
ATOM	924	NH1	ARG	A	120	8.453	-29.234	-56.844	1.00	93.31	N
ATOM	925	NH2	ARG	A	120	6.660	-27.859	-56.438	1.00	93.31	N
ATOM	926	CZ	ARG	A	120	7.703	-28.203	-57.188	1.00	93.31	C
ATOM	927	N	ILE	A	121	9.211	-26.547	-64.125	1.00	93.56	N
ATOM	928	CA	ILE	A	121	10.297	-26.859	-65.062	1.00	93.56	C
ATOM	929	C	ILE	A	121	11.633	-26.781	-64.312	1.00	93.56	C
ATOM	930	CB	ILE	A	121	10.289	-25.906	-66.250	1.00	93.56	C
ATOM	931	O	ILE	A	121	11.828	-25.906	-63.469	1.00	93.56	O
ATOM	932	CG1	ILE	A	121	10.477	-24.453	-65.812	1.00	93.56	C
ATOM	933	CG2	ILE	A	121	9.000	-26.078	-67.062	1.00	93.56	C
ATOM	934	CD1	ILE	A	121	10.742	-23.484	-66.938	1.00	93.56	C
ATOM	935	N	LYS	A	122	12.492	-27.734	-64.562	1.00	93.25	N
ATOM	936	CA	LYS	A	122	13.852	-27.766	-64.062	1.00	93.25	C
ATOM	937	C	LYS	A	122	14.883	-27.500	-65.125	1.00	93.25	C
ATOM	938	CB	LYS	A	122	14.141	-29.094	-63.344	1.00	93.25	C
ATOM	939	O	LYS	A	122	14.992	-28.281	-66.062	1.00	93.25	O
ATOM	940	CG	LYS	A	122	15.445	-29.125	-62.594	1.00	93.25	C
ATOM	941	CD	LYS	A	122	15.688	-30.500	-61.938	1.00	93.25	C
ATOM	942	CE	LYS	A	122	14.961	-30.625	-60.625	1.00	93.25	C
ATOM	943	NZ	LYS	A	122	15.211	-31.953	-59.969	1.00	93.25	N
ATOM	944	N	ILE	A	123	15.523	-26.344	-65.000	1.00	93.25	N
ATOM	945	CA	ILE	A	123	16.578	-25.953	-65.938	1.00	93.25	C
ATOM	946	C	ILE	A	123	17.938	-26.266	-65.375	1.00	93.25	C
ATOM	947	CB	ILE	A	123	16.484	-24.453	-66.312	1.00	93.25	C
ATOM	948	O	ILE	A	123	18.219	-25.922	-64.188	1.00	93.25	O
ATOM	949	CG1	ILE	A	123	15.117	-24.125	-66.938	1.00	93.25	C
ATOM	950	CG2	ILE	A	123	17.609	-24.062	-67.312	1.00	93.25	C
ATOM	951	CD1	ILE	A	123	14.844	-24.828	-68.250	1.00	93.25	C
ATOM	952	N	LEU	A	124	18.766	-27.047	-66.062	1.00	93.06	N
ATOM	953	CA	LEU	A	124	20.078	-27.422	-65.562	1.00	93.06	C
ATOM	954	C	LEU	A	124	21.172	-26.750	-66.375	1.00	93.06	C
ATOM	955	CB	LEU	A	124	20.250	-28.953	-65.625	1.00	93.06	C
ATOM	956	O	LEU	A	124	21.125	-26.781	-67.625	1.00	93.06	O

ATOM	957	CG	LEU	A	124	21.656	-29.484	-65.312	1.00	93.06	C
ATOM	958	CD1	LEU	A	124	21.984	-29.281	-63.844	1.00	93.06	C
ATOM	959	CD2	LEU	A	124	21.766	-30.953	-65.688	1.00	93.06	C
ATOM	960	N	MET	A	125	21.984	-26.016	-65.688	1.00	93.00	N
ATOM	961	CA	MET	A	125	23.250	-25.562	-66.250	1.00	93.00	C
ATOM	962	C	MET	A	125	24.406	-26.422	-65.750	1.00	93.00	C
ATOM	963	CB	MET	A	125	23.500	-24.094	-65.938	1.00	93.00	C
ATOM	964	O	MET	A	125	24.891	-26.234	-64.625	1.00	93.00	O
ATOM	965	CG	MET	A	125	24.766	-23.531	-66.500	1.00	93.00	C
ATOM	966	SD	MET	A	125	25.031	-21.766	-66.125	1.00	93.00	S
ATOM	967	CE	MET	A	125	23.969	-20.984	-67.375	1.00	93.00	C
ATOM	968	N	PRO	A	126	24.828	-27.281	-66.688	1.00	91.19	N
ATOM	969	CA	PRO	A	126	25.906	-28.188	-66.250	1.00	91.19	C
ATOM	970	C	PRO	A	126	27.203	-27.453	-65.938	1.00	91.19	C
ATOM	971	CB	PRO	A	126	26.094	-29.109	-67.438	1.00	91.19	C
ATOM	972	O	PRO	A	126	27.875	-27.781	-64.938	1.00	91.19	O
ATOM	973	CG	PRO	A	126	24.812	-29.016	-68.250	1.00	91.19	C
ATOM	974	CD	PRO	A	126	24.203	-27.672	-67.938	1.00	91.19	C
ATOM	975	N	ARG	A	127	27.531	-26.453	-66.812	1.00	90.19	N
ATOM	976	CA	ARG	A	127	28.703	-25.609	-66.625	1.00	90.19	C
ATOM	977	C	ARG	A	127	28.438	-24.188	-67.125	1.00	90.19	C
ATOM	978	CB	ARG	A	127	29.922	-26.188	-67.375	1.00	90.19	C
ATOM	979	O	ARG	A	127	27.859	-24.016	-68.250	1.00	90.19	O
ATOM	980	CG	ARG	A	127	30.406	-27.516	-66.750	1.00	90.19	C
ATOM	981	CD	ARG	A	127	31.656	-28.016	-67.438	1.00	90.19	C
ATOM	982	NE	ARG	A	127	32.812	-27.188	-67.125	1.00	90.19	N
ATOM	983	NH1	ARG	A	127	34.125	-28.031	-68.875	1.00	90.19	N
ATOM	984	NH2	ARG	A	127	34.969	-26.422	-67.438	1.00	90.19	N
ATOM	985	CZ	ARG	A	127	33.969	-27.219	-67.812	1.00	90.19	C
ATOM	986	N	GLY	A	128	28.734	-23.156	-66.375	1.00	92.69	N
ATOM	987	CA	GLY	A	128	28.641	-21.766	-66.812	1.00	92.69	C
ATOM	988	C	GLY	A	128	29.906	-20.984	-66.500	1.00	92.69	C
ATOM	989	O	GLY	A	128	30.078	-20.469	-65.438	1.00	92.69	O
ATOM	990	N	VAL	A	129	30.734	-20.828	-67.562	1.00	93.69	N
ATOM	991	CA	VAL	A	129	32.000	-20.172	-67.312	1.00	93.69	C
ATOM	992	C	VAL	A	129	32.469	-19.453	-68.625	1.00	93.69	C
ATOM	993	CB	VAL	A	129	33.094	-21.172	-66.875	1.00	93.69	C
ATOM	994	O	VAL	A	129	32.219	-19.938	-69.688	1.00	93.69	O
ATOM	995	CG1	VAL	A	129	33.312	-22.234	-67.938	1.00	93.69	C
ATOM	996	CG2	VAL	A	129	34.406	-20.438	-66.562	1.00	93.69	C
ATOM	997	N	THR	A	130	32.906	-18.266	-68.375	1.00	93.44	N
ATOM	998	CA	THR	A	130	33.562	-17.531	-69.438	1.00	93.44	C
ATOM	999	C	THR	A	130	35.062	-17.422	-69.188	1.00	93.44	C
ATOM	1000	CB	THR	A	130	32.969	-16.109	-69.562	1.00	93.44	C
ATOM	1001	O	THR	A	130	35.500	-17.016	-68.125	1.00	93.44	O
ATOM	1002	CG2	THR	A	130	33.656	-15.336	-70.688	1.00	93.44	C
ATOM	1003	OG1	THR	A	130	31.562	-16.219	-69.875	1.00	93.44	O
ATOM	1004	N	GLN	A	131	35.875	-17.891	-70.125	1.00	93.12	N
ATOM	1005	CA	GLN	A	131	37.344	-17.672	-70.062	1.00	93.12	C
ATOM	1006	C	GLN	A	131	37.719	-16.344	-70.688	1.00	93.12	C
ATOM	1007	CB	GLN	A	131	38.062	-18.812	-70.750	1.00	93.12	C
ATOM	1008	O	GLN	A	131	37.531	-16.172	-71.938	1.00	93.12	O
ATOM	1009	CG	GLN	A	131	39.594	-18.750	-70.625	1.00	93.12	C
ATOM	1010	CD	GLN	A	131	40.281	-19.953	-71.188	1.00	93.12	C
ATOM	1011	NE2	GLN	A	131	41.562	-20.109	-70.875	1.00	93.12	N
ATOM	1012	OE1	GLN	A	131	39.688	-20.719	-71.938	1.00	93.12	O
ATOM	1013	N	GLU	A	132	38.125	-15.367	-69.875	1.00	90.12	N
ATOM	1014	CA	GLU	A	132	38.500	-14.055	-70.375	1.00	90.12	C
ATOM	1015	C	GLU	A	132	39.844	-14.109	-71.062	1.00	90.12	C
ATOM	1016	CB	GLU	A	132	38.562	-13.039	-69.188	1.00	90.12	C
ATOM	1017	O	GLU	A	132	40.594	-15.086	-70.938	1.00	90.12	O
ATOM	1018	CG	GLU	A	132	37.250	-12.875	-68.438	1.00	90.12	C
ATOM	1019	CD	GLU	A	132	36.156	-12.180	-69.312	1.00	90.12	C
ATOM	1020	OE1	GLU	A	132	35.000	-12.141	-68.875	1.00	90.12	O

```
ATOM   1021  OE2 GLU A 132      36.500 -11.680 -70.375  1.00 90.12           O
ATOM   1022  N   ASP A 133      40.250 -13.141 -71.875  1.00 84.19           N
ATOM   1023  CA  ASP A 133      41.500 -13.039 -72.625  1.00 84.19           C
ATOM   1024  C   ASP A 133      42.719 -13.211 -71.750  1.00 84.19           C
ATOM   1025  CB  ASP A 133      41.562 -11.695 -73.375  1.00 84.19           C
ATOM   1026  O   ASP A 133      43.750 -13.742 -72.188  1.00 84.19           O
ATOM   1027  CG  ASP A 133      40.594 -11.594 -74.500  1.00 84.19           C
ATOM   1028  OD1 ASP A 133      40.156 -12.648 -75.062  1.00 84.19           O
ATOM   1029  OD2 ASP A 133      40.250 -10.461 -74.875  1.00 84.19           O
ATOM   1030  N   ASN A 134      42.562 -12.812 -70.375  1.00 84.00           N
ATOM   1031  CA  ASN A 134      43.688 -12.883 -69.500  1.00 84.00           C
ATOM   1032  C   ASN A 134      43.719 -14.227 -68.750  1.00 84.00           C
ATOM   1033  CB  ASN A 134      43.625 -11.734 -68.500  1.00 84.00           C
ATOM   1034  O   ASN A 134      44.531 -14.422 -67.812  1.00 84.00           O
ATOM   1035  CG  ASN A 134      42.375 -11.766 -67.625  1.00 84.00           C
ATOM   1036  ND2 ASN A 134      42.250 -10.789 -66.750  1.00 84.00           N
ATOM   1037  OD1 ASN A 134      41.531 -12.656 -67.750  1.00 84.00           O
ATOM   1038  N   GLY A 135      42.969 -15.258 -69.188  1.00 83.69           N
ATOM   1039  CA  GLY A 135      42.938 -16.578 -68.562  1.00 83.69           C
ATOM   1040  C   GLY A 135      41.969 -16.688 -67.438  1.00 83.69           C
ATOM   1041  O   GLY A 135      41.750 -17.766 -66.875  1.00 83.69           O
ATOM   1042  N   ASP A 136      41.312 -15.539 -66.875  1.00 88.81           N
ATOM   1043  CA  ASP A 136      40.375 -15.539 -65.750  1.00 88.81           C
ATOM   1044  C   ASP A 136      39.094 -16.281 -66.125  1.00 88.81           C
ATOM   1045  CB  ASP A 136      40.062 -14.109 -65.312  1.00 88.81           C
ATOM   1046  O   ASP A 136      38.625 -16.219 -67.312  1.00 88.81           O
ATOM   1047  CG  ASP A 136      41.219 -13.422 -64.688  1.00 88.81           C
ATOM   1048  OD1 ASP A 136      42.250 -14.086 -64.375  1.00 88.81           O
ATOM   1049  OD2 ASP A 136      41.125 -12.203 -64.375  1.00 88.81           O
ATOM   1050  N   LEU A 137      38.656 -17.016 -65.250  1.00 90.94           N
ATOM   1051  CA  LEU A 137      37.375 -17.672 -65.375  1.00 90.94           C
ATOM   1052  C   LEU A 137      36.281 -16.906 -64.625  1.00 90.94           C
ATOM   1053  CB  LEU A 137      37.438 -19.109 -64.875  1.00 90.94           C
ATOM   1054  O   LEU A 137      36.344 -16.781 -63.406  1.00 90.94           O
ATOM   1055  CG  LEU A 137      38.406 -20.047 -65.562  1.00 90.94           C
ATOM   1056  CD1 LEU A 137      38.438 -21.406 -64.938  1.00 90.94           C
ATOM   1057  CD2 LEU A 137      38.031 -20.188 -67.062  1.00 90.94           C
ATOM   1058  N   THR A 138      35.312 -16.328 -65.375  1.00 92.06           N
ATOM   1059  CA  THR A 138      34.219 -15.547 -64.750  1.00 92.06           C
ATOM   1060  C   THR A 138      32.875 -16.172 -65.062  1.00 92.06           C
ATOM   1061  CB  THR A 138      34.250 -14.086 -65.250  1.00 92.06           C
ATOM   1062  O   THR A 138      32.781 -17.203 -65.750  1.00 92.06           O
ATOM   1063  CG2 THR A 138      35.594 -13.422 -64.875  1.00 92.06           C
ATOM   1064  OG1 THR A 138      34.094 -14.062 -66.688  1.00 92.06           O
ATOM   1065  N   GLY A 139      31.828 -15.688 -64.625  1.00 91.38           N
ATOM   1066  CA  GLY A 139      30.469 -16.219 -64.750  1.00 91.38           C
ATOM   1067  C   GLY A 139      29.828 -15.891 -66.062  1.00 91.38           C
ATOM   1068  O   GLY A 139      30.359 -15.086 -66.812  1.00 91.38           O
ATOM   1069  N   VAL A 140      28.766 -16.500 -66.438  1.00 92.06           N
ATOM   1070  CA  VAL A 140      27.969 -16.328 -67.625  1.00 92.06           C
ATOM   1071  C   VAL A 140      26.609 -15.742 -67.312  1.00 92.06           C
ATOM   1072  CB  VAL A 140      27.797 -17.656 -68.438  1.00 92.06           C
ATOM   1073  O   VAL A 140      26.219 -15.727 -66.125  1.00 92.06           O
ATOM   1074  CG1 VAL A 140      29.156 -18.188 -68.875  1.00 92.06           C
ATOM   1075  CG2 VAL A 140      27.109 -18.688 -67.500  1.00 92.06           C
ATOM   1076  N   ARG A 141      26.062 -15.195 -68.250  1.00 92.44           N
ATOM   1077  CA  ARG A 141      24.688 -14.703 -68.188  1.00 92.44           C
ATOM   1078  C   ARG A 141      23.812 -15.305 -69.250  1.00 92.44           C
ATOM   1079  CB  ARG A 141      24.656 -13.180 -68.250  1.00 92.44           C
ATOM   1080  O   ARG A 141      24.062 -15.109 -70.438  1.00 92.44           O
ATOM   1081  CG  ARG A 141      23.266 -12.586 -68.062  1.00 92.44           C
ATOM   1082  CD  ARG A 141      23.297 -11.062 -68.062  1.00 92.44           C
ATOM   1083  NE  ARG A 141      21.969 -10.500 -67.875  1.00 92.44           N
ATOM   1084  NH1 ARG A 141      21.922  -9.391 -69.875  1.00 92.44           N
```

ATOM	1085	NH2	ARG	A	141	20.125	-9.258	-68.500	1.00	92.44	N
ATOM	1086	CZ	ARG	A	141	21.344	-9.719	-68.750	1.00	92.44	C
ATOM	1087	N	VAL	A	142	22.859	-16.203	-68.938	1.00	91.69	N
ATOM	1088	CA	VAL	A	142	21.938	-16.859	-69.875	1.00	91.69	C
ATOM	1089	C	VAL	A	142	20.500	-16.469	-69.500	1.00	91.69	C
ATOM	1090	CB	VAL	A	142	22.094	-18.391	-69.812	1.00	91.69	C
ATOM	1091	O	VAL	A	142	20.078	-16.578	-68.375	1.00	91.69	O
ATOM	1092	CG1	VAL	A	142	21.094	-19.047	-70.750	1.00	91.69	C
ATOM	1093	CG2	VAL	A	142	23.516	-18.781	-70.188	1.00	91.69	C
ATOM	1094	N	GLU	A	143	19.766	-15.953	-70.500	1.00	94.06	N
ATOM	1095	CA	GLU	A	143	18.359	-15.609	-70.312	1.00	94.06	C
ATOM	1096	C	GLU	A	143	17.453	-16.641	-71.000	1.00	94.06	C
ATOM	1097	CB	GLU	A	143	18.062	-14.219	-70.875	1.00	94.06	C
ATOM	1098	O	GLU	A	143	17.750	-17.125	-72.062	1.00	94.06	O
ATOM	1099	CG	GLU	A	143	18.859	-13.102	-70.188	1.00	94.06	C
ATOM	1100	CD	GLU	A	143	18.516	-11.719	-70.750	1.00	94.06	C
ATOM	1101	OE1	GLU	A	143	19.391	-10.828	-70.750	1.00	94.06	O
ATOM	1102	OE2	GLU	A	143	17.344	-11.523	-71.188	1.00	94.06	O
ATOM	1103	N	TYR	A	144	16.375	-17.078	-70.312	1.00	93.88	N
ATOM	1104	CA	TYR	A	144	15.391	-18.000	-70.875	1.00	93.88	C
ATOM	1105	C	TYR	A	144	13.977	-17.562	-70.500	1.00	93.88	C
ATOM	1106	CB	TYR	A	144	15.641	-19.422	-70.375	1.00	93.88	C
ATOM	1107	O	TYR	A	144	13.789	-16.734	-69.562	1.00	93.88	O
ATOM	1108	CG	TYR	A	144	15.352	-19.609	-68.875	1.00	93.88	C
ATOM	1109	CD1	TYR	A	144	16.312	-19.281	-67.938	1.00	93.88	C
ATOM	1110	CD2	TYR	A	144	14.133	-20.109	-68.438	1.00	93.88	C
ATOM	1111	CE1	TYR	A	144	16.047	-19.453	-66.562	1.00	93.88	C
ATOM	1112	CE2	TYR	A	144	13.859	-20.281	-67.125	1.00	93.88	C
ATOM	1113	OH	TYR	A	144	14.570	-20.141	-64.812	1.00	93.88	O
ATOM	1114	CZ	TYR	A	144	14.828	-19.953	-66.188	1.00	93.88	C
ATOM	1115	N	ALA	A	145	13.047	-18.000	-71.312	1.00	94.62	N
ATOM	1116	CA	ALA	A	145	11.656	-17.609	-71.125	1.00	94.62	C
ATOM	1117	C	ALA	A	145	10.719	-18.797	-71.312	1.00	94.62	C
ATOM	1118	CB	ALA	A	145	11.273	-16.469	-72.062	1.00	94.62	C
ATOM	1119	O	ALA	A	145	11.078	-19.766	-72.000	1.00	94.62	O
ATOM	1120	N	VAL	A	146	9.562	-18.781	-70.625	1.00	95.50	N
ATOM	1121	CA	VAL	A	146	8.484	-19.734	-70.812	1.00	95.50	C
ATOM	1122	C	VAL	A	146	7.246	-19.016	-71.375	1.00	95.50	C
ATOM	1123	CB	VAL	A	146	8.133	-20.484	-69.500	1.00	95.50	C
ATOM	1124	O	VAL	A	146	6.797	-18.031	-70.812	1.00	95.50	O
ATOM	1125	CG1	VAL	A	146	7.047	-21.531	-69.750	1.00	95.50	C
ATOM	1126	CG2	VAL	A	146	9.383	-21.141	-68.938	1.00	95.50	C
ATOM	1127	N	ASP	A	147	6.801	-19.438	-72.500	1.00	94.81	N
ATOM	1128	CA	ASP	A	147	5.586	-18.906	-73.125	1.00	94.81	C
ATOM	1129	C	ASP	A	147	4.438	-19.906	-73.000	1.00	94.81	C
ATOM	1130	CB	ASP	A	147	5.832	-18.578	-74.625	1.00	94.81	C
ATOM	1131	O	ASP	A	147	4.656	-21.125	-73.062	1.00	94.81	O
ATOM	1132	CG	ASP	A	147	7.043	-17.688	-74.812	1.00	94.81	C
ATOM	1133	OD1	ASP	A	147	7.242	-16.734	-74.062	1.00	94.81	O
ATOM	1134	OD2	ASP	A	147	7.801	-17.922	-75.812	1.00	94.81	O
ATOM	1135	N	MET	A	148	3.207	-19.391	-72.750	1.00	96.00	N
ATOM	1136	CA	MET	A	148	2.043	-20.250	-72.562	1.00	96.00	C
ATOM	1137	C	MET	A	148	0.969	-19.922	-73.625	1.00	96.00	C
ATOM	1138	CB	MET	A	148	1.470	-20.094	-71.188	1.00	96.00	C
ATOM	1139	O	MET	A	148	0.670	-18.766	-73.875	1.00	96.00	O
ATOM	1140	CG	MET	A	148	0.272	-20.984	-70.875	1.00	96.00	C
ATOM	1141	SD	MET	A	148	-0.235	-20.969	-69.125	1.00	96.00	S
ATOM	1142	CE	MET	A	148	-0.732	-19.234	-68.938	1.00	96.00	C
ATOM	1143	N	ALA	A	149	0.448	-20.953	-74.250	1.00	94.31	N
ATOM	1144	CA	ALA	A	149	-0.714	-20.875	-75.125	1.00	94.31	C
ATOM	1145	C	ALA	A	149	-1.859	-21.734	-74.625	1.00	94.31	C
ATOM	1146	CB	ALA	A	149	-0.335	-21.281	-76.562	1.00	94.31	C
ATOM	1147	O	ALA	A	149	-1.689	-22.938	-74.438	1.00	94.31	O
ATOM	1148	N	VAL	A	150	-3.010	-21.141	-74.438	1.00	93.44	N

ATOM	1149	CA	VAL	A	150	-4.156	-21.844	-73.812	1.00	93.44	C
ATOM	1150	C	VAL	A	150	-5.215	-22.062	-74.938	1.00	93.44	C
ATOM	1151	CB	VAL	A	150	-4.762	-21.062	-72.625	1.00	93.44	C
ATOM	1152	O	VAL	A	150	-5.656	-21.109	-75.562	1.00	93.44	O
ATOM	1153	CG1	VAL	A	150	-5.926	-21.828	-72.062	1.00	93.44	C
ATOM	1154	CG2	VAL	A	150	-3.689	-20.781	-71.625	1.00	93.44	C
ATOM	1155	N	ASP	A	151	-5.574	-23.281	-75.312	1.00	91.25	N
ATOM	1156	CA	ASP	A	151	-6.668	-23.688	-76.188	1.00	91.25	C
ATOM	1157	C	ASP	A	151	-6.562	-23.000	-77.562	1.00	91.25	C
ATOM	1158	CB	ASP	A	151	-8.016	-23.359	-75.500	1.00	91.25	C
ATOM	1159	O	ASP	A	151	-7.535	-22.422	-78.000	1.00	91.25	O
ATOM	1160	CG	ASP	A	151	-8.312	-24.234	-74.312	1.00	91.25	C
ATOM	1161	OD1	ASP	A	151	-7.906	-25.406	-74.312	1.00	91.25	O
ATOM	1162	OD2	ASP	A	151	-8.969	-23.734	-73.375	1.00	91.25	O
ATOM	1163	N	GLY	A	152	-5.273	-22.938	-78.125	1.00	86.19	N
ATOM	1164	CA	GLY	A	152	-5.074	-22.438	-79.500	1.00	86.19	C
ATOM	1165	C	GLY	A	152	-4.750	-20.969	-79.562	1.00	86.19	C
ATOM	1166	O	GLY	A	152	-4.582	-20.406	-80.625	1.00	86.19	O
ATOM	1167	N	ALA	A	153	-4.727	-20.250	-78.312	1.00	91.25	N
ATOM	1168	CA	ALA	A	153	-4.348	-18.844	-78.312	1.00	91.25	C
ATOM	1169	C	ALA	A	153	-2.859	-18.672	-78.562	1.00	91.25	C
ATOM	1170	CB	ALA	A	153	-4.691	-18.234	-76.938	1.00	91.25	C
ATOM	1171	O	ALA	A	153	-2.111	-19.641	-78.625	1.00	91.25	O
ATOM	1172	N	GLU	A	154	-2.455	-17.391	-78.938	1.00	93.31	N
ATOM	1173	CA	GLU	A	154	-1.055	-17.047	-79.188	1.00	93.31	C
ATOM	1174	C	GLU	A	154	-0.227	-17.234	-77.875	1.00	93.31	C
ATOM	1175	CB	GLU	A	154	-0.925	-15.609	-79.688	1.00	93.31	C
ATOM	1176	O	GLU	A	154	-0.754	-17.141	-76.812	1.00	93.31	O
ATOM	1177	CG	GLU	A	154	-1.505	-15.398	-81.062	1.00	93.31	C
ATOM	1178	CD	GLU	A	154	-1.277	-13.992	-81.625	1.00	93.31	C
ATOM	1179	OE1	GLU	A	154	-1.588	-13.734	-82.812	1.00	93.31	O
ATOM	1180	OE2	GLU	A	154	-0.785	-13.141	-80.812	1.00	93.31	O
ATOM	1181	N	TYR	A	155	1.066	-17.625	-78.125	1.00	94.25	N
ATOM	1182	CA	TYR	A	155	1.974	-17.750	-76.938	1.00	94.25	C
ATOM	1183	C	TYR	A	155	2.156	-16.391	-76.250	1.00	94.25	C
ATOM	1184	CB	TYR	A	155	3.334	-18.297	-77.438	1.00	94.25	C
ATOM	1185	O	TYR	A	155	2.410	-15.383	-76.938	1.00	94.25	O
ATOM	1186	CG	TYR	A	155	3.336	-19.781	-77.688	1.00	94.25	C
ATOM	1187	CD1	TYR	A	155	3.393	-20.688	-76.625	1.00	94.25	C
ATOM	1188	CD2	TYR	A	155	3.285	-20.281	-79.000	1.00	94.25	C
ATOM	1189	CE1	TYR	A	155	3.398	-22.062	-76.875	1.00	94.25	C
ATOM	1190	CE2	TYR	A	155	3.289	-21.641	-79.188	1.00	94.25	C
ATOM	1191	OH	TYR	A	155	3.352	-23.875	-78.375	1.00	94.25	O
ATOM	1192	CZ	TYR	A	155	3.346	-22.531	-78.125	1.00	94.25	C
ATOM	1193	N	LYS	A	156	1.907	-16.406	-74.938	1.00	94.94	N
ATOM	1194	CA	LYS	A	156	2.191	-15.266	-74.062	1.00	94.94	C
ATOM	1195	C	LYS	A	156	3.275	-15.594	-73.062	1.00	94.94	C
ATOM	1196	CB	LYS	A	156	0.920	-14.805	-73.375	1.00	94.94	C
ATOM	1197	O	LYS	A	156	3.268	-16.672	-72.500	1.00	94.94	O
ATOM	1198	CG	LYS	A	156	-0.111	-14.164	-74.312	1.00	94.94	C
ATOM	1199	CD	LYS	A	156	-1.306	-13.633	-73.500	1.00	94.94	C
ATOM	1200	CE	LYS	A	156	-2.361	-13.039	-74.438	1.00	94.94	C
ATOM	1201	NZ	LYS	A	156	-3.541	-12.531	-73.688	1.00	94.94	N
ATOM	1202	N	GLU	A	157	4.309	-14.734	-73.000	1.00	95.00	N
ATOM	1203	CA	GLU	A	157	5.391	-14.945	-72.062	1.00	95.00	C
ATOM	1204	C	GLU	A	157	4.867	-14.945	-70.625	1.00	95.00	C
ATOM	1205	CB	GLU	A	157	6.473	-13.883	-72.188	1.00	95.00	C
ATOM	1206	O	GLU	A	157	4.258	-13.969	-70.188	1.00	95.00	O
ATOM	1207	CG	GLU	A	157	7.691	-14.094	-71.312	1.00	95.00	C
ATOM	1208	CD	GLU	A	157	8.781	-13.055	-71.500	1.00	95.00	C
ATOM	1209	OE1	GLU	A	157	8.906	-12.133	-70.688	1.00	95.00	O
ATOM	1210	OE2	GLU	A	157	9.508	-13.156	-72.562	1.00	95.00	O
ATOM	1211	N	VAL	A	158	5.035	-16.094	-69.875	1.00	93.38	N
ATOM	1212	CA	VAL	A	158	4.512	-16.219	-68.500	1.00	93.38	C

ATOM	1213	C	VAL	A	158	5.668	-16.234	-67.500	1.00	93.38	C
ATOM	1214	CB	VAL	A	158	3.664	-17.500	-68.375	1.00	93.38	C
ATOM	1215	O	VAL	A	158	5.457	-16.047	-66.312	1.00	93.38	O
ATOM	1216	CG1	VAL	A	158	2.354	-17.391	-69.125	1.00	93.38	C
ATOM	1217	CG2	VAL	A	158	4.453	-18.719	-68.812	1.00	93.38	C
ATOM	1218	N	LEU	A	159	6.941	-16.438	-68.000	1.00	93.69	N
ATOM	1219	CA	LEU	A	159	8.141	-16.438	-67.188	1.00	93.69	C
ATOM	1220	C	LEU	A	159	9.359	-15.969	-67.938	1.00	93.69	C
ATOM	1221	CB	LEU	A	159	8.391	-17.828	-66.562	1.00	93.69	C
ATOM	1222	O	LEU	A	159	9.578	-16.422	-69.062	1.00	93.69	O
ATOM	1223	CG	LEU	A	159	9.672	-18.000	-65.750	1.00	93.69	C
ATOM	1224	CD1	LEU	A	159	9.477	-17.469	-64.375	1.00	93.69	C
ATOM	1225	CD2	LEU	A	159	10.094	-19.469	-65.750	1.00	93.69	C
ATOM	1226	N	HIS	A	160	10.039	-14.969	-67.500	1.00	94.19	N
ATOM	1227	CA	HIS	A	160	11.352	-14.523	-67.938	1.00	94.19	C
ATOM	1228	C	HIS	A	160	12.383	-14.578	-66.812	1.00	94.19	C
ATOM	1229	CB	HIS	A	160	11.266	-13.102	-68.500	1.00	94.19	C
ATOM	1230	O	HIS	A	160	12.188	-13.969	-65.750	1.00	94.19	O
ATOM	1231	CG	HIS	A	160	12.531	-12.641	-69.125	1.00	94.19	C
ATOM	1232	CD2	HIS	A	160	13.766	-13.195	-69.188	1.00	94.19	C
ATOM	1233	ND1	HIS	A	160	12.617	-11.461	-69.875	1.00	94.19	N
ATOM	1234	CE1	HIS	A	160	13.852	-11.312	-70.312	1.00	94.19	C
ATOM	1235	NE2	HIS	A	160	14.570	-12.352	-69.938	1.00	94.19	N
ATOM	1236	N	ASP	A	161	13.445	-15.359	-67.000	1.00	91.56	N
ATOM	1237	CA	ASP	A	161	14.414	-15.539	-65.938	1.00	91.56	C
ATOM	1238	C	ASP	A	161	15.844	-15.516	-66.500	1.00	91.56	C
ATOM	1239	CB	ASP	A	161	14.164	-16.859	-65.188	1.00	91.56	C
ATOM	1240	O	ASP	A	161	16.062	-15.672	-67.688	1.00	91.56	O
ATOM	1241	CG	ASP	A	161	14.648	-16.828	-63.781	1.00	91.56	C
ATOM	1242	OD1	ASP	A	161	15.328	-15.859	-63.375	1.00	91.56	O
ATOM	1243	OD2	ASP	A	161	14.352	-17.797	-63.031	1.00	91.56	O
ATOM	1244	N	VAL	A	162	16.781	-15.172	-65.500	1.00	91.62	N
ATOM	1245	CA	VAL	A	162	18.188	-15.047	-65.875	1.00	91.62	C
ATOM	1246	C	VAL	A	162	19.047	-15.891	-64.938	1.00	91.62	C
ATOM	1247	CB	VAL	A	162	18.656	-13.578	-65.875	1.00	91.62	C
ATOM	1248	O	VAL	A	162	18.875	-15.844	-63.719	1.00	91.62	O
ATOM	1249	CG1	VAL	A	162	20.141	-13.469	-66.188	1.00	91.62	C
ATOM	1250	CG2	VAL	A	162	17.812	-12.727	-66.812	1.00	91.62	C
ATOM	1251	N	ILE	A	163	19.906	-16.781	-65.562	1.00	90.50	N
ATOM	1252	CA	ILE	A	163	20.969	-17.453	-64.812	1.00	90.50	C
ATOM	1253	C	ILE	A	163	22.297	-16.734	-65.062	1.00	90.50	C
ATOM	1254	CB	ILE	A	163	21.094	-18.938	-65.188	1.00	90.50	C
ATOM	1255	O	ILE	A	163	22.812	-16.703	-66.125	1.00	90.50	O
ATOM	1256	CG1	ILE	A	163	19.750	-19.656	-65.000	1.00	90.50	C
ATOM	1257	CG2	ILE	A	163	22.203	-19.625	-64.438	1.00	90.50	C
ATOM	1258	CD1	ILE	A	163	19.734	-21.094	-65.562	1.00	90.50	C
ATOM	1259	N	GLU	A	164	22.797	-16.016	-63.906	1.00	90.81	N
ATOM	1260	CA	GLU	A	164	24.031	-15.234	-64.000	1.00	90.81	C
ATOM	1261	C	GLU	A	164	25.016	-15.625	-62.906	1.00	90.81	C
ATOM	1262	CB	GLU	A	164	23.719	-13.742	-63.906	1.00	90.81	C
ATOM	1263	O	GLU	A	164	24.688	-15.578	-61.719	1.00	90.81	O
ATOM	1264	CG	GLU	A	164	24.922	-12.844	-64.125	1.00	90.81	C
ATOM	1265	CD	GLU	A	164	24.578	-11.359	-64.125	1.00	90.81	C
ATOM	1266	OE1	GLU	A	164	25.469	-10.531	-64.438	1.00	90.81	O
ATOM	1267	OE2	GLU	A	164	23.422	-11.031	-63.812	1.00	90.81	O
ATOM	1268	N	GLY	A	165	26.078	-16.031	-63.312	1.00	91.94	N
ATOM	1269	CA	GLY	A	165	27.156	-16.406	-62.406	1.00	91.94	C
ATOM	1270	C	GLY	A	165	28.031	-17.516	-62.969	1.00	91.94	C
ATOM	1271	O	GLY	A	165	27.953	-17.844	-64.125	1.00	91.94	O
ATOM	1272	N	LYS	A	166	28.953	-18.047	-62.156	1.00	92.62	N
ATOM	1273	CA	LYS	A	166	29.875	-19.125	-62.562	1.00	92.62	C
ATOM	1274	C	LYS	A	166	29.531	-20.422	-61.812	1.00	92.62	C
ATOM	1275	CB	LYS	A	166	31.328	-18.719	-62.281	1.00	92.62	C
ATOM	1276	O	LYS	A	166	29.297	-20.406	-60.625	1.00	92.62	O

ATOM	1277	CG	LYS	A	166	32.344	-19.766	-62.688	1.00	92.62	C
ATOM	1278	CD	LYS	A	166	33.688	-19.531	-62.031	1.00	92.62	C
ATOM	1279	CE	LYS	A	166	34.625	-20.734	-62.188	1.00	92.62	C
ATOM	1280	NZ	LYS	A	166	34.875	-21.375	-60.844	1.00	92.62	N
ATOM	1281	N	THR	A	167	29.453	-21.547	-62.531	1.00	92.00	N
ATOM	1282	CA	THR	A	167	29.312	-22.859	-61.938	1.00	92.00	C
ATOM	1283	C	THR	A	167	30.109	-23.906	-62.719	1.00	92.00	C
ATOM	1284	CB	THR	A	167	27.828	-23.281	-61.875	1.00	92.00	C
ATOM	1285	O	THR	A	167	30.094	-23.906	-63.938	1.00	92.00	O
ATOM	1286	CG2	THR	A	167	27.250	-23.406	-63.281	1.00	92.00	C
ATOM	1287	OG1	THR	A	167	27.734	-24.547	-61.219	1.00	92.00	O
ATOM	1288	N	MET	A	168	30.953	-24.812	-62.031	1.00	88.31	N
ATOM	1289	CA	MET	A	168	31.688	-25.922	-62.625	1.00	88.31	C
ATOM	1290	C	MET	A	168	31.062	-27.266	-62.250	1.00	88.31	C
ATOM	1291	CB	MET	A	168	33.156	-25.891	-62.188	1.00	88.31	C
ATOM	1292	O	MET	A	168	31.328	-28.281	-62.875	1.00	88.31	O
ATOM	1293	CG	MET	A	168	33.906	-24.656	-62.656	1.00	88.31	C
ATOM	1294	SD	MET	A	168	34.125	-24.609	-64.438	1.00	88.31	S
ATOM	1295	CE	MET	A	168	35.312	-25.969	-64.688	1.00	88.31	C
ATOM	1296	N	SER	A	169	30.250	-27.297	-61.281	1.00	85.44	N
ATOM	1297	CA	SER	A	169	29.672	-28.516	-60.688	1.00	85.44	C
ATOM	1298	C	SER	A	169	28.219	-28.672	-61.062	1.00	85.44	C
ATOM	1299	CB	SER	A	169	29.828	-28.484	-59.156	1.00	85.44	C
ATOM	1300	O	SER	A	169	27.594	-29.688	-60.750	1.00	85.44	O
ATOM	1301	OG	SER	A	169	29.219	-27.328	-58.625	1.00	85.44	O
ATOM	1302	N	GLY	A	170	27.656	-27.812	-61.969	1.00	87.00	N
ATOM	1303	CA	GLY	A	170	26.266	-27.906	-62.375	1.00	87.00	C
ATOM	1304	C	GLY	A	170	25.312	-27.156	-61.469	1.00	87.00	C
ATOM	1305	O	GLY	A	170	25.516	-27.125	-60.250	1.00	87.00	O
ATOM	1306	N	TYR	A	171	24.422	-26.422	-61.938	1.00	91.00	N
ATOM	1307	CA	TYR	A	171	23.406	-25.609	-61.250	1.00	91.00	C
ATOM	1308	C	TYR	A	171	22.031	-25.859	-61.844	1.00	91.00	C
ATOM	1309	CB	TYR	A	171	23.766	-24.125	-61.375	1.00	91.00	C
ATOM	1310	O	TYR	A	171	21.844	-25.812	-63.062	1.00	91.00	O
ATOM	1311	CG	TYR	A	171	22.688	-23.203	-60.812	1.00	91.00	C
ATOM	1312	CD1	TYR	A	171	21.891	-22.453	-61.656	1.00	91.00	C
ATOM	1313	CD2	TYR	A	171	22.484	-23.109	-59.438	1.00	91.00	C
ATOM	1314	CE1	TYR	A	171	20.906	-21.609	-61.156	1.00	91.00	C
ATOM	1315	CE2	TYR	A	171	21.516	-22.266	-58.906	1.00	91.00	C
ATOM	1316	OH	TYR	A	171	19.750	-20.688	-59.281	1.00	91.00	O
ATOM	1317	CZ	TYR	A	171	20.719	-21.516	-59.781	1.00	91.00	C
ATOM	1318	N	ASP	A	172	21.125	-26.344	-61.031	1.00	89.56	N
ATOM	1319	CA	ASP	A	172	19.750	-26.516	-61.500	1.00	89.56	C
ATOM	1320	C	ASP	A	172	18.812	-25.531	-60.781	1.00	89.56	C
ATOM	1321	CB	ASP	A	172	19.281	-27.953	-61.250	1.00	89.56	C
ATOM	1322	O	ASP	A	172	19.094	-25.094	-59.688	1.00	89.56	O
ATOM	1323	CG	ASP	A	172	19.266	-28.328	-59.781	1.00	89.56	C
ATOM	1324	OD1	ASP	A	172	19.469	-27.453	-58.938	1.00	89.56	O
ATOM	1325	OD2	ASP	A	172	19.047	-29.531	-59.500	1.00	89.56	O
ATOM	1326	N	ARG	A	173	17.875	-25.109	-61.469	1.00	88.62	N
ATOM	1327	CA	ARG	A	173	16.859	-24.188	-61.000	1.00	88.62	C
ATOM	1328	C	ARG	A	173	15.461	-24.594	-61.469	1.00	88.62	C
ATOM	1329	CB	ARG	A	173	17.156	-22.766	-61.469	1.00	88.62	C
ATOM	1330	O	ARG	A	173	15.258	-24.859	-62.656	1.00	88.62	O
ATOM	1331	CG	ARG	A	173	16.297	-21.703	-60.781	1.00	88.62	C
ATOM	1332	CD	ARG	A	173	16.625	-20.312	-61.312	1.00	88.62	C
ATOM	1333	NE	ARG	A	173	17.719	-19.688	-60.562	1.00	88.62	N
ATOM	1334	NH1	ARG	A	173	17.625	-17.672	-61.719	1.00	88.62	N
ATOM	1335	NH2	ARG	A	173	19.156	-17.984	-60.062	1.00	88.62	N
ATOM	1336	CZ	ARG	A	173	18.156	-18.453	-60.781	1.00	88.62	C
ATOM	1337	N	SER	A	174	14.562	-24.750	-60.562	1.00	90.12	N
ATOM	1338	CA	SER	A	174	13.188	-25.125	-60.875	1.00	90.12	C
ATOM	1339	C	SER	A	174	12.250	-23.922	-60.812	1.00	90.12	C
ATOM	1340	CB	SER	A	174	12.703	-26.219	-59.906	1.00	90.12	C

ATOM	1341	O	SER	A	174	12.383	-23.078	-59.938	1.00	90.12	O
ATOM	1342	OG	SER	A	174	13.500	-27.375	-60.031	1.00	90.12	O
ATOM	1343	N	ARG	A	175	11.422	-23.844	-61.812	1.00	90.50	N
ATOM	1344	CA	ARG	A	175	10.398	-22.797	-61.875	1.00	90.50	C
ATOM	1345	C	ARG	A	175	9.008	-23.391	-62.062	1.00	90.50	C
ATOM	1346	CB	ARG	A	175	10.703	-21.797	-63.000	1.00	90.50	C
ATOM	1347	O	ARG	A	175	8.805	-24.219	-62.938	1.00	90.50	O
ATOM	1348	CG	ARG	A	175	11.961	-20.969	-62.750	1.00	90.50	C
ATOM	1349	CD	ARG	A	175	11.789	-20.000	-61.594	1.00	90.50	C
ATOM	1350	NE	ARG	A	175	12.977	-19.156	-61.406	1.00	90.50	N
ATOM	1351	NH1	ARG	A	175	12.891	-19.172	-59.094	1.00	90.50	N
ATOM	1352	NH2	ARG	A	175	14.547	-18.016	-60.188	1.00	90.50	N
ATOM	1353	CZ	ARG	A	175	13.469	-18.781	-60.250	1.00	90.50	C
ATOM	1354	N	ARG	A	176	8.109	-22.922	-61.125	1.00	91.00	N
ATOM	1355	CA	ARG	A	176	6.719	-23.375	-61.188	1.00	91.00	C
ATOM	1356	C	ARG	A	176	5.898	-22.453	-62.094	1.00	91.00	C
ATOM	1357	CB	ARG	A	176	6.094	-23.453	-59.812	1.00	91.00	C
ATOM	1358	O	ARG	A	176	5.926	-21.234	-61.938	1.00	91.00	O
ATOM	1359	CG	ARG	A	176	4.617	-23.797	-59.812	1.00	91.00	C
ATOM	1360	CD	ARG	A	176	4.055	-23.906	-58.406	1.00	91.00	C
ATOM	1361	NE	ARG	A	176	2.604	-24.047	-58.406	1.00	91.00	N
ATOM	1362	NH1	ARG	A	176	2.490	-25.125	-56.375	1.00	91.00	N
ATOM	1363	NH2	ARG	A	176	0.573	-24.688	-57.531	1.00	91.00	N
ATOM	1364	CZ	ARG	A	176	1.892	-24.625	-57.438	1.00	91.00	C
ATOM	1365	N	ILE	A	177	5.195	-22.984	-63.125	1.00	92.81	N
ATOM	1366	CA	ILE	A	177	4.289	-22.250	-64.000	1.00	92.81	C
ATOM	1367	C	ILE	A	177	2.852	-22.703	-63.750	1.00	92.81	C
ATOM	1368	CB	ILE	A	177	4.664	-22.422	-65.500	1.00	92.81	C
ATOM	1369	O	ILE	A	177	2.492	-23.828	-64.062	1.00	92.81	O
ATOM	1370	CG1	ILE	A	177	6.129	-22.047	-65.750	1.00	92.81	C
ATOM	1371	CG2	ILE	A	177	3.732	-21.609	-66.375	1.00	92.81	C
ATOM	1372	CD1	ILE	A	177	6.809	-21.422	-64.500	1.00	92.81	C
ATOM	1373	N	ASP	A	178	2.016	-21.812	-63.125	1.00	91.62	N
ATOM	1374	CA	ASP	A	178	0.625	-22.141	-62.812	1.00	91.62	C
ATOM	1375	C	ASP	A	178	-0.213	-22.188	-64.125	1.00	91.62	C
ATOM	1376	CB	ASP	A	178	0.034	-21.125	-61.844	1.00	91.62	C
ATOM	1377	O	ASP	A	178	-0.130	-21.281	-64.938	1.00	91.62	O
ATOM	1378	CG	ASP	A	178	0.636	-21.219	-60.469	1.00	91.62	C
ATOM	1379	OD1	ASP	A	178	0.945	-22.344	-60.000	1.00	91.62	O
ATOM	1380	OD2	ASP	A	178	0.801	-20.172	-59.812	1.00	91.62	O
ATOM	1381	N	LEU	A	179	-0.941	-23.266	-64.188	1.00	91.06	N
ATOM	1382	CA	LEU	A	179	-1.790	-23.438	-65.375	1.00	91.06	C
ATOM	1383	C	LEU	A	179	-3.127	-22.734	-65.188	1.00	91.06	C
ATOM	1384	CB	LEU	A	179	-2.020	-24.922	-65.625	1.00	91.06	C
ATOM	1385	O	LEU	A	179	-3.719	-22.781	-64.062	1.00	91.06	O
ATOM	1386	CG	LEU	A	179	-0.777	-25.750	-65.938	1.00	91.06	C
ATOM	1387	CD1	LEU	A	179	-1.126	-27.234	-66.000	1.00	91.06	C
ATOM	1388	CD2	LEU	A	179	-0.169	-25.297	-67.312	1.00	91.06	C
ATOM	1389	N	PRO	A	180	-3.619	-22.016	-66.125	1.00	89.75	N
ATOM	1390	CA	PRO	A	180	-4.980	-21.469	-66.062	1.00	89.75	C
ATOM	1391	C	PRO	A	180	-6.047	-22.531	-66.312	1.00	89.75	C
ATOM	1392	CB	PRO	A	180	-4.973	-20.406	-67.188	1.00	89.75	C
ATOM	1393	O	PRO	A	180	-5.719	-23.672	-66.688	1.00	89.75	O
ATOM	1394	CG	PRO	A	180	-4.031	-20.938	-68.188	1.00	89.75	C
ATOM	1395	CD	PRO	A	180	-2.934	-21.688	-67.500	1.00	89.75	C
ATOM	1396	N	ALA	A	181	-7.285	-22.188	-65.938	1.00	86.62	N
ATOM	1397	CA	ALA	A	181	-8.359	-23.078	-66.375	1.00	86.62	C
ATOM	1398	C	ALA	A	181	-8.391	-23.203	-67.938	1.00	86.62	C
ATOM	1399	CB	ALA	A	181	-9.703	-22.578	-65.875	1.00	86.62	C
ATOM	1400	O	ALA	A	181	-8.195	-22.219	-68.625	1.00	86.62	O
ATOM	1401	N	PHE	A	182	-8.344	-24.422	-68.562	1.00	90.19	N
ATOM	1402	CA	PHE	A	182	-8.383	-24.609	-70.000	1.00	90.19	C
ATOM	1403	C	PHE	A	182	-9.344	-25.734	-70.375	1.00	90.19	C
ATOM	1404	CB	PHE	A	182	-6.984	-24.906	-70.562	1.00	90.19	C

ATOM	1405	O	PHE	A	182	-9.672	-26.578	-69.562	1.00	90.19	O
ATOM	1406	CG	PHE	A	182	-6.379	-26.172	-70.000	1.00	90.19	C
ATOM	1407	CD1	PHE	A	182	-5.715	-26.172	-68.750	1.00	90.19	C
ATOM	1408	CD2	PHE	A	182	-6.469	-27.359	-70.688	1.00	90.19	C
ATOM	1409	CE1	PHE	A	182	-5.152	-27.344	-68.250	1.00	90.19	C
ATOM	1410	CE2	PHE	A	182	-5.906	-28.531	-70.188	1.00	90.19	C
ATOM	1411	CZ	PHE	A	182	-5.250	-28.516	-69.000	1.00	90.19	C
ATOM	1412	N	ASN	A	183	-9.883	-25.672	-71.688	1.00	89.00	N
ATOM	1413	CA	ASN	A	183	-10.867	-26.641	-72.188	1.00	89.00	C
ATOM	1414	C	ASN	A	183	-10.195	-27.812	-72.875	1.00	89.00	C
ATOM	1415	CB	ASN	A	183	-11.867	-25.953	-73.062	1.00	89.00	C
ATOM	1416	O	ASN	A	183	-10.523	-28.969	-72.625	1.00	89.00	O
ATOM	1417	CG	ASN	A	183	-12.750	-24.953	-72.375	1.00	89.00	C
ATOM	1418	ND2	ASN	A	183	-13.062	-23.859	-73.062	1.00	89.00	N
ATOM	1419	OD1	ASN	A	183	-13.148	-25.172	-71.250	1.00	89.00	O
ATOM	1420	N	GLU	A	184	-9.180	-27.594	-73.812	1.00	89.94	N
ATOM	1421	CA	GLU	A	184	-8.562	-28.625	-74.625	1.00	89.94	C
ATOM	1422	C	GLU	A	184	-7.129	-28.906	-74.188	1.00	89.94	C
ATOM	1423	CB	GLU	A	184	-8.594	-28.219	-76.125	1.00	89.94	C
ATOM	1424	O	GLU	A	184	-6.809	-30.000	-73.750	1.00	89.94	O
ATOM	1425	CG	GLU	A	184	-10.000	-28.188	-76.688	1.00	89.94	C
ATOM	1426	CD	GLU	A	184	-10.016	-27.781	-78.188	1.00	89.94	C
ATOM	1427	OE1	GLU	A	184	-11.117	-27.656	-78.750	1.00	89.94	O
ATOM	1428	OE2	GLU	A	184	-8.922	-27.562	-78.750	1.00	89.94	O
ATOM	1429	N	ARG	A	185	-6.223	-27.891	-74.312	1.00	93.25	N
ATOM	1430	CA	ARG	A	185	-4.816	-28.109	-74.000	1.00	93.25	C
ATOM	1431	C	ARG	A	185	-4.109	-26.781	-73.688	1.00	93.25	C
ATOM	1432	CB	ARG	A	185	-4.105	-28.859	-75.125	1.00	93.25	C
ATOM	1433	O	ARG	A	185	-4.574	-25.719	-74.125	1.00	93.25	O
ATOM	1434	CG	ARG	A	185	-3.986	-28.047	-76.438	1.00	93.25	C
ATOM	1435	CD	ARG	A	185	-3.258	-28.844	-77.500	1.00	93.25	C
ATOM	1436	NE	ARG	A	185	-4.191	-29.484	-78.438	1.00	93.25	N
ATOM	1437	NH1	ARG	A	185	-2.561	-30.531	-79.688	1.00	93.25	N
ATOM	1438	NH2	ARG	A	185	-4.773	-30.797	-80.188	1.00	93.25	N
ATOM	1439	CZ	ARG	A	185	-3.840	-30.266	-79.438	1.00	93.25	C
ATOM	1440	N	VAL	A	186	-3.094	-26.812	-72.875	1.00	95.12	N
ATOM	1441	CA	VAL	A	186	-2.133	-25.750	-72.625	1.00	95.12	C
ATOM	1442	C	VAL	A	186	-0.763	-26.156	-73.188	1.00	95.12	C
ATOM	1443	CB	VAL	A	186	-2.035	-25.375	-71.125	1.00	95.12	C
ATOM	1444	O	VAL	A	186	-0.312	-27.281	-72.938	1.00	95.12	O
ATOM	1445	CG1	VAL	A	186	-0.956	-24.312	-70.938	1.00	95.12	C
ATOM	1446	CG2	VAL	A	186	-3.387	-24.875	-70.625	1.00	95.12	C
ATOM	1447	N	LEU	A	187	-0.131	-25.266	-74.000	1.00	95.44	N
ATOM	1448	CA	LEU	A	187	1.221	-25.484	-74.500	1.00	95.44	C
ATOM	1449	C	LEU	A	187	2.213	-24.547	-73.812	1.00	95.44	C
ATOM	1450	CB	LEU	A	187	1.268	-25.281	-76.000	1.00	95.44	C
ATOM	1451	O	LEU	A	187	2.000	-23.344	-73.750	1.00	95.44	O
ATOM	1452	CG	LEU	A	187	0.396	-26.219	-76.875	1.00	95.44	C
ATOM	1453	CD1	LEU	A	187	0.366	-25.766	-78.312	1.00	95.44	C
ATOM	1454	CD2	LEU	A	187	0.903	-27.656	-76.750	1.00	95.44	C
ATOM	1455	N	LEU	A	188	3.199	-25.156	-73.188	1.00	95.62	N
ATOM	1456	CA	LEU	A	188	4.305	-24.391	-72.562	1.00	95.62	C
ATOM	1457	C	LEU	A	188	5.559	-24.531	-73.438	1.00	95.62	C
ATOM	1458	CB	LEU	A	188	4.594	-24.859	-71.125	1.00	95.62	C
ATOM	1459	O	LEU	A	188	6.051	-25.641	-73.625	1.00	95.62	O
ATOM	1460	CG	LEU	A	188	3.469	-24.656	-70.125	1.00	95.62	C
ATOM	1461	CD1	LEU	A	188	3.795	-25.391	-68.812	1.00	95.62	C
ATOM	1462	CD2	LEU	A	188	3.238	-23.172	-69.875	1.00	95.62	C
ATOM	1463	N	ARG	A	189	5.977	-23.344	-73.938	1.00	94.88	N
ATOM	1464	CA	ARG	A	189	7.195	-23.297	-74.750	1.00	94.88	C
ATOM	1465	C	ARG	A	189	8.352	-22.703	-73.938	1.00	94.88	C
ATOM	1466	CB	ARG	A	189	6.973	-22.484	-76.062	1.00	94.88	C
ATOM	1467	O	ARG	A	189	8.336	-21.516	-73.625	1.00	94.88	O
ATOM	1468	CG	ARG	A	189	8.172	-22.469	-77.000	1.00	94.88	C

ATOM	1469	CD	ARG	A	189	7.824	-21.844	-78.312	1.00 94.88	C
ATOM	1470	NE	ARG	A	189	7.527	-20.422	-78.188	1.00 94.88	N
ATOM	1471	NH1	ARG	A	189	6.836	-20.172	-80.375	1.00 94.88	N
ATOM	1472	NH2	ARG	A	189	6.824	-18.375	-78.938	1.00 94.88	N
ATOM	1473	CZ	ARG	A	189	7.062	-19.656	-79.188	1.00 94.88	C
ATOM	1474	N	VAL	A	190	9.359	-23.641	-73.625	1.00 95.12	N
ATOM	1475	CA	VAL	A	190	10.570	-23.234	-72.875	1.00 95.12	C
ATOM	1476	C	VAL	A	190	11.664	-22.891	-73.875	1.00 95.12	C
ATOM	1477	CB	VAL	A	190	11.055	-24.328	-71.938	1.00 95.12	C
ATOM	1478	O	VAL	A	190	12.102	-23.750	-74.688	1.00 95.12	O
ATOM	1479	CG1	VAL	A	190	12.211	-23.812	-71.062	1.00 95.12	C
ATOM	1480	CG2	VAL	A	190	9.906	-24.812	-71.062	1.00 95.12	C
ATOM	1481	N	ARG	A	191	12.109	-21.562	-73.875	1.00 93.69	N
ATOM	1482	CA	ARG	A	191	13.070	-21.172	-74.938	1.00 93.69	C
ATOM	1483	C	ARG	A	191	14.219	-20.359	-74.312	1.00 93.69	C
ATOM	1484	CB	ARG	A	191	12.383	-20.359	-76.000	1.00 93.69	C
ATOM	1485	O	ARG	A	191	14.023	-19.609	-73.375	1.00 93.69	O
ATOM	1486	CG	ARG	A	191	11.594	-19.172	-75.500	1.00 93.69	C
ATOM	1487	CD	ARG	A	191	10.914	-18.406	-76.625	1.00 93.69	C
ATOM	1488	NE	ARG	A	191	10.219	-17.219	-76.125	1.00 93.69	N
ATOM	1489	NH1	ARG	A	191	12.070	-15.844	-76.250	1.00 93.69	N
ATOM	1490	NH2	ARG	A	191	10.070	-15.008	-75.500	1.00 93.69	N
ATOM	1491	CZ	ARG	A	191	10.789	-16.031	-76.000	1.00 93.69	C
ATOM	1492	N	ARG	A	192	15.367	-20.609	-74.750	1.00 93.44	N
ATOM	1493	CA	ARG	A	192	16.531	-19.781	-74.438	1.00 93.44	C
ATOM	1494	C	ARG	A	192	16.531	-18.500	-75.250	1.00 93.44	C
ATOM	1495	CB	ARG	A	192	17.828	-20.562	-74.688	1.00 93.44	C
ATOM	1496	O	ARG	A	192	16.234	-18.531	-76.438	1.00 93.44	O
ATOM	1497	CG	ARG	A	192	19.078	-19.781	-74.312	1.00 93.44	C
ATOM	1498	CD	ARG	A	192	20.328	-20.625	-74.500	1.00 93.44	C
ATOM	1499	NE	ARG	A	192	21.547	-19.859	-74.188	1.00 93.44	N
ATOM	1500	NH1	ARG	A	192	23.000	-21.609	-74.625	1.00 93.44	N
ATOM	1501	NH2	ARG	A	192	23.812	-19.562	-74.000	1.00 93.44	N
ATOM	1502	CZ	ARG	A	192	22.781	-20.344	-74.312	1.00 93.44	C
ATOM	1503	N	LEU	A	193	16.844	-17.344	-74.688	1.00 90.31	N
ATOM	1504	CA	LEU	A	193	16.766	-16.062	-75.312	1.00 90.31	C
ATOM	1505	C	LEU	A	193	18.141	-15.602	-75.812	1.00 90.31	C
ATOM	1506	CB	LEU	A	193	16.141	-15.008	-74.438	1.00 90.31	C
ATOM	1507	O	LEU	A	193	18.266	-14.914	-76.812	1.00 90.31	O
ATOM	1508	CG	LEU	A	193	14.711	-15.266	-73.938	1.00 90.31	C
ATOM	1509	CD1	LEU	A	193	14.281	-14.227	-72.938	1.00 90.31	C
ATOM	1510	CD2	LEU	A	193	13.758	-15.273	-75.188	1.00 90.31	C
ATOM	1511	N	THR	A	194	19.234	-16.000	-75.125	1.00 84.19	N
ATOM	1512	CA	THR	A	194	20.578	-15.523	-75.438	1.00 84.19	C
ATOM	1513	C	THR	A	194	21.516	-16.688	-75.688	1.00 84.19	C
ATOM	1514	CB	THR	A	194	21.141	-14.656	-74.312	1.00 84.19	C
ATOM	1515	O	THR	A	194	21.625	-17.609	-74.875	1.00 84.19	O
ATOM	1516	CG2	THR	A	194	20.344	-13.375	-74.125	1.00 84.19	C
ATOM	1517	OG1	THR	A	194	21.094	-15.398	-73.062	1.00 84.19	O
ATOM	1518	N	ASP	A	195	22.031	-16.844	-77.062	1.00 76.88	N
ATOM	1519	CA	ASP	A	195	23.062	-17.828	-77.375	1.00 76.88	C
ATOM	1520	C	ASP	A	195	24.422	-17.172	-77.562	1.00 76.88	C
ATOM	1521	CB	ASP	A	195	22.688	-18.547	-78.688	1.00 76.88	C
ATOM	1522	O	ASP	A	195	24.547	-16.062	-78.062	1.00 76.88	O
ATOM	1523	CG	ASP	A	195	21.641	-19.641	-78.500	1.00 76.88	C
ATOM	1524	OD1	ASP	A	195	21.516	-20.156	-77.375	1.00 76.88	O
ATOM	1525	OD2	ASP	A	195	20.953	-19.969	-79.500	1.00 76.88	O
ATOM	1526	N	SER	A	196	25.391	-17.734	-76.812	1.00 81.38	N
ATOM	1527	CA	SER	A	196	26.734	-17.219	-77.000	1.00 81.38	C
ATOM	1528	C	SER	A	196	27.406	-17.828	-78.250	1.00 81.38	C
ATOM	1529	CB	SER	A	196	27.594	-17.469	-75.750	1.00 81.38	C
ATOM	1530	O	SER	A	196	27.234	-19.016	-78.500	1.00 81.38	O
ATOM	1531	OG	SER	A	196	28.922	-17.016	-75.938	1.00 81.38	O
ATOM	1532	N	THR	A	197	28.078	-17.031	-79.125	1.00 79.06	N

```
ATOM   1533  CA  THR A 197      28.781 -17.484 -80.375  1.00 79.06           C
ATOM   1534  C   THR A 197      30.250 -17.719 -80.062  1.00 79.06           C
ATOM   1535  CB  THR A 197      28.641 -16.453 -81.500  1.00 79.06           C
ATOM   1536  O   THR A 197      30.984 -18.250 -80.875  1.00 79.06           O
ATOM   1537  CG2 THR A 197      27.188 -16.328 -81.938  1.00 79.06           C
ATOM   1538  OG1 THR A 197      29.094 -15.180 -81.062  1.00 79.06           O
ATOM   1539  N   SER A 198      30.703 -17.453 -78.688  1.00 85.19           N
ATOM   1540  CA  SER A 198      32.125 -17.547 -78.375  1.00 85.19           C
ATOM   1541  C   SER A 198      32.469 -18.938 -77.812  1.00 85.19           C
ATOM   1542  CB  SER A 198      32.469 -16.484 -77.312  1.00 85.19           C
ATOM   1543  O   SER A 198      31.703 -19.516 -77.062  1.00 85.19           O
ATOM   1544  OG  SER A 198      33.781 -16.703 -76.812  1.00 85.19           O
ATOM   1545  N   ALA A 199      33.562 -19.562 -78.375  1.00 83.44           N
ATOM   1546  CA  ALA A 199      34.062 -20.859 -77.938  1.00 83.44           C
ATOM   1547  C   ALA A 199      34.562 -20.750 -76.500  1.00 83.44           C
ATOM   1548  CB  ALA A 199      35.156 -21.375 -78.812  1.00 83.44           C
ATOM   1549  O   ALA A 199      34.781 -21.766 -75.812  1.00 83.44           O
ATOM   1550  N   ARG A 200      34.812 -19.469 -76.000  1.00 88.50           N
ATOM   1551  CA  ARG A 200      35.375 -19.203 -74.688  1.00 88.50           C
ATOM   1552  C   ARG A 200      34.312 -19.172 -73.625  1.00 88.50           C
ATOM   1553  CB  ARG A 200      36.156 -17.891 -74.688  1.00 88.50           C
ATOM   1554  O   ARG A 200      34.594 -19.047 -72.438  1.00 88.50           O
ATOM   1555  CG  ARG A 200      37.406 -17.891 -75.562  1.00 88.50           C
ATOM   1556  CD  ARG A 200      38.250 -16.641 -75.375  1.00 88.50           C
ATOM   1557  NE  ARG A 200      39.438 -16.688 -76.188  1.00 88.50           N
ATOM   1558  NH1 ARG A 200      40.125 -14.547 -75.688  1.00 88.50           N
ATOM   1559  NH2 ARG A 200      41.406 -15.844 -77.062  1.00 88.50           N
ATOM   1560  CZ  ARG A 200      40.312 -15.695 -76.312  1.00 88.50           C
ATOM   1561  N   VAL A 201      33.062 -19.203 -74.000  1.00 90.62           N
ATOM   1562  CA  VAL A 201      31.891 -19.172 -73.125  1.00 90.62           C
ATOM   1563  C   VAL A 201      31.188 -20.531 -73.125  1.00 90.62           C
ATOM   1564  CB  VAL A 201      30.906 -18.047 -73.500  1.00 90.62           C
ATOM   1565  O   VAL A 201      30.875 -21.062 -74.188  1.00 90.62           O
ATOM   1566  CG1 VAL A 201      29.688 -18.062 -72.625  1.00 90.62           C
ATOM   1567  CG2 VAL A 201      31.594 -16.688 -73.500  1.00 90.62           C
ATOM   1568  N   THR A 202      31.172 -21.156 -72.062  1.00 91.31           N
ATOM   1569  CA  THR A 202      30.359 -22.359 -71.812  1.00 91.31           C
ATOM   1570  C   THR A 202      29.109 -22.016 -71.000  1.00 91.31           C
ATOM   1571  CB  THR A 202      31.156 -23.453 -71.125  1.00 91.31           C
ATOM   1572  O   THR A 202      29.188 -21.750 -69.812  1.00 91.31           O
ATOM   1573  CG2 THR A 202      30.375 -24.766 -71.062  1.00 91.31           C
ATOM   1574  OG1 THR A 202      32.406 -23.656 -71.812  1.00 91.31           O
ATOM   1575  N   ASP A 203      27.906 -21.984 -71.688  1.00 91.88           N
ATOM   1576  CA  ASP A 203      26.703 -21.531 -71.000  1.00 91.88           C
ATOM   1577  C   ASP A 203      25.484 -22.359 -71.438  1.00 91.88           C
ATOM   1578  CB  ASP A 203      26.453 -20.047 -71.250  1.00 91.88           C
ATOM   1579  O   ASP A 203      24.375 -21.828 -71.562  1.00 91.88           O
ATOM   1580  CG  ASP A 203      26.203 -19.750 -72.750  1.00 91.88           C
ATOM   1581  OD1 ASP A 203      26.422 -20.641 -73.562  1.00 91.88           O
ATOM   1582  OD2 ASP A 203      25.781 -18.609 -73.062  1.00 91.88           O
ATOM   1583  N   LEU A 204      25.656 -23.625 -71.750  1.00 91.06           N
ATOM   1584  CA  LEU A 204      24.578 -24.516 -72.125  1.00 91.06           C
ATOM   1585  C   LEU A 204      23.547 -24.641 -71.000  1.00 91.06           C
ATOM   1586  CB  LEU A 204      25.109 -25.891 -72.500  1.00 91.06           C
ATOM   1587  O   LEU A 204      23.906 -24.812 -69.875  1.00 91.06           O
ATOM   1588  CG  LEU A 204      24.078 -26.969 -72.812  1.00 91.06           C
ATOM   1589  CD1 LEU A 204      23.422 -26.703 -74.188  1.00 91.06           C
ATOM   1590  CD2 LEU A 204      24.703 -28.359 -72.812  1.00 91.06           C
ATOM   1591  N   ILE A 205      22.250 -24.500 -71.312  1.00 92.69           N
ATOM   1592  CA  ILE A 205      21.188 -24.844 -70.375  1.00 92.69           C
ATOM   1593  C   ILE A 205      20.297 -25.938 -71.000  1.00 92.69           C
ATOM   1594  CB  ILE A 205      20.344 -23.609 -70.062  1.00 92.69           C
ATOM   1595  O   ILE A 205      20.109 -25.984 -72.188  1.00 92.69           O
ATOM   1596  CG1 ILE A 205      19.719 -23.000 -71.312  1.00 92.69           C
```

ATOM	1597	CG2	ILE	A	205	21.188	-22.578	-69.312	1.00	92.69	C
ATOM	1598	CD1	ILE	A	205	18.703	-21.891	-71.000	1.00	92.69	C
ATOM	1599	N	LYS	A	206	19.906	-26.844	-70.125	1.00	92.75	N
ATOM	1600	CA	LYS	A	206	19.047	-27.969	-70.500	1.00	92.75	C
ATOM	1601	C	LYS	A	206	17.703	-27.891	-69.750	1.00	92.75	C
ATOM	1602	CB	LYS	A	206	19.734	-29.297	-70.188	1.00	92.75	C
ATOM	1603	O	LYS	A	206	17.641	-27.438	-68.625	1.00	92.75	O
ATOM	1604	CG	LYS	A	206	21.000	-29.547	-71.000	1.00	92.75	C
ATOM	1605	CD	LYS	A	206	21.656	-30.875	-70.625	1.00	92.75	C
ATOM	1606	CE	LYS	A	206	22.938	-31.109	-71.375	1.00	92.75	C
ATOM	1607	NZ	LYS	A	206	23.375	-32.531	-71.312	1.00	92.75	N
ATOM	1608	N	MET	A	207	16.672	-28.141	-70.625	1.00	93.50	N
ATOM	1609	CA	MET	A	207	15.453	-28.562	-69.938	1.00	93.50	C
ATOM	1610	C	MET	A	207	15.617	-29.969	-69.375	1.00	93.50	C
ATOM	1611	CB	MET	A	207	14.258	-28.516	-70.875	1.00	93.50	C
ATOM	1612	O	MET	A	207	15.516	-30.953	-70.125	1.00	93.50	O
ATOM	1613	CG	MET	A	207	12.945	-28.922	-70.250	1.00	93.50	C
ATOM	1614	SD	MET	A	207	12.438	-27.750	-68.938	1.00	93.50	S
ATOM	1615	CE	MET	A	207	10.922	-28.562	-68.312	1.00	93.50	C
ATOM	1616	N	GLN	A	208	15.938	-30.062	-68.125	1.00	92.56	N
ATOM	1617	CA	GLN	A	208	16.281	-31.312	-67.438	1.00	92.56	C
ATOM	1618	C	GLN	A	208	15.047	-32.188	-67.250	1.00	92.56	C
ATOM	1619	CB	GLN	A	208	16.984	-31.047	-66.125	1.00	92.56	C
ATOM	1620	O	GLN	A	208	15.031	-33.344	-67.625	1.00	92.56	O
ATOM	1621	CG	GLN	A	208	17.484	-32.312	-65.438	1.00	92.56	C
ATOM	1622	CD	GLN	A	208	18.656	-32.938	-66.125	1.00	92.56	C
ATOM	1623	NE2	GLN	A	208	19.000	-34.156	-65.688	1.00	92.56	N
ATOM	1624	OE1	GLN	A	208	19.250	-32.344	-67.062	1.00	92.56	O
ATOM	1625	N	SER	A	209	14.055	-31.641	-66.688	1.00	91.56	N
ATOM	1626	CA	SER	A	209	12.805	-32.344	-66.375	1.00	91.56	C
ATOM	1627	C	SER	A	209	11.672	-31.359	-66.062	1.00	91.56	C
ATOM	1628	CB	SER	A	209	12.992	-33.281	-65.188	1.00	91.56	C
ATOM	1629	O	SER	A	209	11.906	-30.156	-65.938	1.00	91.56	O
ATOM	1630	OG	SER	A	209	13.234	-32.531	-63.969	1.00	91.56	O
ATOM	1631	N	TYR	A	210	10.484	-31.812	-66.250	1.00	92.44	N
ATOM	1632	CA	TYR	A	210	9.328	-31.078	-65.750	1.00	92.44	C
ATOM	1633	C	TYR	A	210	8.430	-31.969	-64.938	1.00	92.44	C
ATOM	1634	CB	TYR	A	210	8.531	-30.484	-66.938	1.00	92.44	C
ATOM	1635	O	TYR	A	210	8.414	-33.188	-65.062	1.00	92.44	O
ATOM	1636	CG	TYR	A	210	7.863	-31.516	-67.812	1.00	92.44	C
ATOM	1637	CD1	TYR	A	210	8.516	-32.062	-68.875	1.00	92.44	C
ATOM	1638	CD2	TYR	A	210	6.578	-31.969	-67.500	1.00	92.44	C
ATOM	1639	CE1	TYR	A	210	7.910	-33.000	-69.688	1.00	92.44	C
ATOM	1640	CE2	TYR	A	210	5.957	-32.906	-68.312	1.00	92.44	C
ATOM	1641	OH	TYR	A	210	6.020	-34.375	-70.188	1.00	92.44	O
ATOM	1642	CZ	TYR	A	210	6.629	-33.438	-69.375	1.00	92.44	C
ATOM	1643	N	ALA	A	211	7.883	-31.359	-63.906	1.00	90.50	N
ATOM	1644	CA	ALA	A	211	6.965	-32.094	-63.000	1.00	90.50	C
ATOM	1645	C	ALA	A	211	5.531	-31.594	-63.188	1.00	90.50	C
ATOM	1646	CB	ALA	A	211	7.395	-31.922	-61.562	1.00	90.50	C
ATOM	1647	O	ALA	A	211	5.285	-30.391	-63.281	1.00	90.50	O
ATOM	1648	N	GLU	A	212	4.707	-32.562	-63.469	1.00	90.69	N
ATOM	1649	CA	GLU	A	212	3.277	-32.250	-63.438	1.00	90.69	C
ATOM	1650	C	GLU	A	212	2.766	-32.156	-62.000	1.00	90.69	C
ATOM	1651	CB	GLU	A	212	2.492	-33.344	-64.188	1.00	90.69	C
ATOM	1652	O	GLU	A	212	2.826	-33.125	-61.250	1.00	90.69	O
ATOM	1653	CG	GLU	A	212	2.838	-33.406	-65.688	1.00	90.69	C
ATOM	1654	CD	GLU	A	212	2.098	-34.531	-66.375	1.00	90.69	C
ATOM	1655	OE1	GLU	A	212	2.162	-34.594	-67.625	1.00	90.69	O
ATOM	1656	OE2	GLU	A	212	1.446	-35.344	-65.688	1.00	90.69	O
ATOM	1657	N	VAL	A	213	2.309	-30.984	-61.625	1.00	89.94	N
ATOM	1658	CA	VAL	A	213	2.031	-30.688	-60.219	1.00	89.94	C
ATOM	1659	C	VAL	A	213	0.522	-30.594	-60.000	1.00	89.94	C
ATOM	1660	CB	VAL	A	213	2.717	-29.375	-59.781	1.00	89.94	C

```
ATOM   1661  O   VAL A 213      -0.182 -29.938 -60.781  1.00 89.94           O
ATOM   1662  CG1 VAL A 213       2.404 -29.078 -58.312  1.00 89.94           C
ATOM   1663  CG2 VAL A 213       4.227 -29.469 -60.000  1.00 89.94           C
ATOM   1664  N   VAL A 214       0.071 -31.406 -59.094  1.00 87.81           N
ATOM   1665  CA  VAL A 214      -1.300 -31.266 -58.625  1.00 87.81           C
ATOM   1666  C   VAL A 214      -1.296 -30.688 -57.219  1.00 87.81           C
ATOM   1667  CB  VAL A 214      -2.047 -32.625 -58.625  1.00 87.81           C
ATOM   1668  O   VAL A 214      -0.761 -31.281 -56.281  1.00 87.81           O
ATOM   1669  CG1 VAL A 214      -3.484 -32.438 -58.156  1.00 87.81           C
ATOM   1670  CG2 VAL A 214      -2.025 -33.219 -60.031  1.00 87.81           C
ATOM   1671  N   ASP A 215      -1.861 -29.438 -57.094  1.00 85.94           N
ATOM   1672  CA  ASP A 215      -1.936 -28.812 -55.781  1.00 85.94           C
ATOM   1673  C   ASP A 215      -3.047 -29.438 -54.938  1.00 85.94           C
ATOM   1674  CB  ASP A 215      -2.160 -27.312 -55.906  1.00 85.94           C
ATOM   1675  O   ASP A 215      -4.074 -29.859 -55.469  1.00 85.94           O
ATOM   1676  CG  ASP A 215      -0.941 -26.578 -56.438  1.00 85.94           C
ATOM   1677  OD1 ASP A 215       0.188 -27.094 -56.344  1.00 85.94           O
ATOM   1678  OD2 ASP A 215      -1.111 -25.469 -57.000  1.00 85.94           O
ATOM   1679  N   ALA A 216      -2.758 -29.734 -53.656  1.00 84.50           N
ATOM   1680  CA  ALA A 216      -3.889 -29.906 -52.750  1.00 84.50           C
ATOM   1681  C   ALA A 216      -4.723 -28.625 -52.688  1.00 84.50           C
ATOM   1682  CB  ALA A 216      -3.402 -30.297 -51.375  1.00 84.50           C
ATOM   1683  O   ALA A 216      -4.211 -27.562 -52.312  1.00 84.50           O
ATOM   1684  N   LYS A 217      -6.051 -28.672 -53.188  1.00 84.88           N
ATOM   1685  CA  LYS A 217      -6.867 -27.484 -53.344  1.00 84.88           C
ATOM   1686  C   LYS A 217      -7.809 -27.297 -52.156  1.00 84.88           C
ATOM   1687  CB  LYS A 217      -7.672 -27.531 -54.656  1.00 84.88           C
ATOM   1688  O   LYS A 217      -8.992 -27.625 -52.250  1.00 84.88           O
ATOM   1689  CG  LYS A 217      -6.812 -27.547 -55.906  1.00 84.88           C
ATOM   1690  CD  LYS A 217      -7.668 -27.594 -57.156  1.00 84.88           C
ATOM   1691  CE  LYS A 217      -6.812 -27.609 -58.406  1.00 84.88           C
ATOM   1692  NZ  LYS A 217      -7.645 -27.641 -59.656  1.00 84.88           N
ATOM   1693  N   PHE A 218      -7.203 -26.828 -51.000  1.00 84.94           N
ATOM   1694  CA  PHE A 218      -8.008 -26.500 -49.844  1.00 84.94           C
ATOM   1695  C   PHE A 218      -8.312 -25.000 -49.781  1.00 84.94           C
ATOM   1696  CB  PHE A 218      -7.289 -26.938 -48.562  1.00 84.94           C
ATOM   1697  O   PHE A 218      -7.391 -24.188 -49.750  1.00 84.94           O
ATOM   1698  CG  PHE A 218      -7.094 -28.422 -48.438  1.00 84.94           C
ATOM   1699  CD1 PHE A 218      -8.188 -29.266 -48.375  1.00 84.94           C
ATOM   1700  CD2 PHE A 218      -5.816 -28.969 -48.406  1.00 84.94           C
ATOM   1701  CE1 PHE A 218      -8.016 -30.656 -48.281  1.00 84.94           C
ATOM   1702  CE2 PHE A 218      -5.637 -30.344 -48.281  1.00 84.94           C
ATOM   1703  CZ  PHE A 218      -6.734 -31.188 -48.219  1.00 84.94           C
ATOM   1704  N   ARG A 219      -9.594 -24.672 -49.938  1.00 87.25           N
ATOM   1705  CA  ARG A 219      -9.984 -23.266 -49.906  1.00 87.25           C
ATOM   1706  C   ARG A 219     -10.094 -22.766 -48.469  1.00 87.25           C
ATOM   1707  CB  ARG A 219     -11.312 -23.062 -50.625  1.00 87.25           C
ATOM   1708  O   ARG A 219      -9.961 -21.562 -48.219  1.00 87.25           O
ATOM   1709  CG  ARG A 219     -12.484 -23.781 -49.969  1.00 87.25           C
ATOM   1710  CD  ARG A 219     -13.766 -23.609 -50.781  1.00 87.25           C
ATOM   1711  NE  ARG A 219     -14.930 -24.109 -50.062  1.00 87.25           N
ATOM   1712  NH1 ARG A 219     -16.453 -23.594 -51.750  1.00 87.25           N
ATOM   1713  NH2 ARG A 219     -17.172 -24.578 -49.781  1.00 87.25           N
ATOM   1714  CZ  ARG A 219     -16.172 -24.094 -50.531  1.00 87.25           C
ATOM   1715  N   TYR A 220     -10.289 -23.609 -47.469  1.00 89.12           N
ATOM   1716  CA  TYR A 220     -10.445 -23.234 -46.062  1.00 89.12           C
ATOM   1717  C   TYR A 220     -11.469 -22.125 -45.906  1.00 89.12           C
ATOM   1718  CB  TYR A 220      -9.102 -22.812 -45.469  1.00 89.12           C
ATOM   1719  O   TYR A 220     -11.133 -21.016 -45.500  1.00 89.12           O
ATOM   1720  CG  TYR A 220      -8.047 -23.891 -45.500  1.00 89.12           C
ATOM   1721  CD1 TYR A 220      -8.094 -24.953 -44.625  1.00 89.12           C
ATOM   1722  CD2 TYR A 220      -7.000 -23.828 -46.438  1.00 89.12           C
ATOM   1723  CE1 TYR A 220      -7.121 -25.953 -44.656  1.00 89.12           C
ATOM   1724  CE2 TYR A 220      -6.023 -24.812 -46.469  1.00 89.12           C
```

```
ATOM   1725  OH  TYR A 220      -5.125 -26.859 -45.594  1.00 89.12           O
ATOM   1726  CZ  TYR A 220      -6.090 -25.875 -45.562  1.00 89.12           C
ATOM   1727  N   PRO A 221     -12.781 -22.516 -46.281  1.00 91.38           N
ATOM   1728  CA  PRO A 221     -13.820 -21.484 -46.188  1.00 91.38           C
ATOM   1729  C   PRO A 221     -13.898 -20.844 -44.812  1.00 91.38           C
ATOM   1730  CB  PRO A 221     -15.109 -22.250 -46.500  1.00 91.38           C
ATOM   1731  O   PRO A 221     -13.734 -21.531 -43.781  1.00 91.38           O
ATOM   1732  CG  PRO A 221     -14.797 -23.672 -46.188  1.00 91.38           C
ATOM   1733  CD  PRO A 221     -13.328 -23.891 -46.375  1.00 91.38           C
ATOM   1734  N   LEU A 222     -13.961 -19.391 -44.750  1.00 92.00           N
ATOM   1735  CA  LEU A 222     -14.172 -18.594 -43.562  1.00 92.00           C
ATOM   1736  C   LEU A 222     -12.953 -18.641 -42.625  1.00 92.00           C
ATOM   1737  CB  LEU A 222     -15.414 -19.078 -42.812  1.00 92.00           C
ATOM   1738  O   LEU A 222     -13.078 -18.516 -41.406  1.00 92.00           O
ATOM   1739  CG  LEU A 222     -16.750 -18.953 -43.531  1.00 92.00           C
ATOM   1740  CD1 LEU A 222     -17.875 -19.578 -42.719  1.00 92.00           C
ATOM   1741  CD2 LEU A 222     -17.062 -17.500 -43.844  1.00 92.00           C
ATOM   1742  N   THR A 223     -11.852 -19.078 -43.188  1.00 93.12           N
ATOM   1743  CA  THR A 223     -10.562 -19.031 -42.500  1.00 93.12           C
ATOM   1744  C   THR A 223      -9.602 -18.109 -43.250  1.00 93.12           C
ATOM   1745  CB  THR A 223      -9.945 -20.438 -42.375  1.00 93.12           C
ATOM   1746  O   THR A 223      -9.297 -18.344 -44.406  1.00 93.12           O
ATOM   1747  CG2 THR A 223      -8.617 -20.391 -41.625  1.00 93.12           C
ATOM   1748  OG1 THR A 223     -10.852 -21.297 -41.656  1.00 93.12           O
ATOM   1749  N   GLY A 224      -9.172 -16.984 -42.625  1.00 92.75           N
ATOM   1750  CA  GLY A 224      -8.164 -16.125 -43.219  1.00 92.75           C
ATOM   1751  C   GLY A 224      -6.793 -16.781 -43.281  1.00 92.75           C
ATOM   1752  O   GLY A 224      -6.336 -17.391 -42.312  1.00 92.75           O
ATOM   1753  N   LEU A 225      -6.125 -16.656 -44.438  1.00 93.06           N
ATOM   1754  CA  LEU A 225      -4.840 -17.312 -44.688  1.00 93.06           C
ATOM   1755  C   LEU A 225      -3.799 -16.297 -45.156  1.00 93.06           C
ATOM   1756  CB  LEU A 225      -4.977 -18.438 -45.688  1.00 93.06           C
ATOM   1757  O   LEU A 225      -4.109 -15.414 -45.938  1.00 93.06           O
ATOM   1758  CG  LEU A 225      -6.000 -19.531 -45.375  1.00 93.06           C
ATOM   1759  CD1 LEU A 225      -6.199 -20.453 -46.562  1.00 93.06           C
ATOM   1760  CD2 LEU A 225      -5.555 -20.328 -44.156  1.00 93.06           C
ATOM   1761  N   VAL A 226      -2.609 -16.469 -44.562  1.00 93.62           N
ATOM   1762  CA  VAL A 226      -1.479 -15.711 -45.062  1.00 93.62           C
ATOM   1763  C   VAL A 226      -0.365 -16.656 -45.500  1.00 93.62           C
ATOM   1764  CB  VAL A 226      -0.949 -14.695 -44.031  1.00 93.62           C
ATOM   1765  O   VAL A 226       0.108 -17.469 -44.688  1.00 93.62           O
ATOM   1766  CG1 VAL A 226       0.328 -14.023 -44.531  1.00 93.62           C
ATOM   1767  CG2 VAL A 226      -2.016 -13.656 -43.719  1.00 93.62           C
ATOM   1768  N   TYR A 227      -0.017 -16.578 -46.781  1.00 92.75           N
ATOM   1769  CA  TYR A 227       1.090 -17.344 -47.375  1.00 92.75           C
ATOM   1770  C   TYR A 227       2.328 -16.453 -47.500  1.00 92.75           C
ATOM   1771  CB  TYR A 227       0.695 -17.906 -48.750  1.00 92.75           C
ATOM   1772  O   TYR A 227       2.264 -15.375 -48.094  1.00 92.75           O
ATOM   1773  CG  TYR A 227       1.841 -18.547 -49.469  1.00 92.75           C
ATOM   1774  CD1 TYR A 227       2.518 -17.844 -50.500  1.00 92.75           C
ATOM   1775  CD2 TYR A 227       2.248 -19.844 -49.188  1.00 92.75           C
ATOM   1776  CE1 TYR A 227       3.572 -18.438 -51.188  1.00 92.75           C
ATOM   1777  CE2 TYR A 227       3.303 -20.438 -49.875  1.00 92.75           C
ATOM   1778  OH  TYR A 227       5.000 -20.312 -51.531  1.00 92.75           O
ATOM   1779  CZ  TYR A 227       3.957 -19.734 -50.875  1.00 92.75           C
ATOM   1780  N   VAL A 228       3.400 -16.891 -46.844  1.00 92.62           N
ATOM   1781  CA  VAL A 228       4.645 -16.141 -46.906  1.00 92.62           C
ATOM   1782  C   VAL A 228       5.766 -17.031 -47.438  1.00 92.62           C
ATOM   1783  CB  VAL A 228       5.027 -15.562 -45.500  1.00 92.62           C
ATOM   1784  O   VAL A 228       5.922 -18.172 -47.000  1.00 92.62           O
ATOM   1785  CG1 VAL A 228       6.305 -14.734 -45.594  1.00 92.62           C
ATOM   1786  CG2 VAL A 228       3.883 -14.727 -44.938  1.00 92.62           C
ATOM   1787  N   GLU A 229       6.535 -16.422 -48.438  1.00 90.94           N
ATOM   1788  CA  GLU A 229       7.684 -17.094 -49.062  1.00 90.94           C
```

ATOM	1789	C	GLU	A	229	8.906	-16.188	-49.062	1.00	90.94	C
ATOM	1790	CB	GLU	A	229	7.355	-17.562	-50.469	1.00	90.94	C
ATOM	1791	O	GLU	A	229	8.812	-15.016	-49.438	1.00	90.94	O
ATOM	1792	CG	GLU	A	229	8.508	-18.250	-51.188	1.00	90.94	C
ATOM	1793	CD	GLU	A	229	8.180	-18.672	-52.594	1.00	90.94	C
ATOM	1794	OE1	GLU	A	229	9.117	-18.812	-53.438	1.00	90.94	O
ATOM	1795	OE2	GLU	A	229	6.980	-18.859	-52.906	1.00	90.94	O
ATOM	1796	N	PHE	A	230	10.008	-16.703	-48.531	1.00	90.75	N
ATOM	1797	CA	PHE	A	230	11.242	-15.930	-48.562	1.00	90.75	C
ATOM	1798	C	PHE	A	230	12.461	-16.844	-48.625	1.00	90.75	C
ATOM	1799	CB	PHE	A	230	11.352	-15.023	-47.312	1.00	90.75	C
ATOM	1800	O	PHE	A	230	12.352	-18.047	-48.375	1.00	90.75	O
ATOM	1801	CG	PHE	A	230	11.227	-15.758	-46.031	1.00	90.75	C
ATOM	1802	CD1	PHE	A	230	9.977	-15.953	-45.438	1.00	90.75	C
ATOM	1803	CD2	PHE	A	230	12.352	-16.250	-45.375	1.00	90.75	C
ATOM	1804	CE1	PHE	A	230	9.859	-16.641	-44.219	1.00	90.75	C
ATOM	1805	CE2	PHE	A	230	12.242	-16.922	-44.156	1.00	90.75	C
ATOM	1806	CZ	PHE	A	230	10.992	-17.125	-43.594	1.00	90.75	C
ATOM	1807	N	ASP	A	231	13.672	-16.250	-49.000	1.00	88.31	N
ATOM	1808	CA	ASP	A	231	14.930	-16.984	-49.125	1.00	88.31	C
ATOM	1809	C	ASP	A	231	15.461	-17.438	-47.781	1.00	88.31	C
ATOM	1810	CB	ASP	A	231	15.977	-16.125	-49.844	1.00	88.31	C
ATOM	1811	O	ASP	A	231	15.484	-16.656	-46.812	1.00	88.31	O
ATOM	1812	CG	ASP	A	231	15.625	-15.859	-51.312	1.00	88.31	C
ATOM	1813	OD1	ASP	A	231	14.609	-16.391	-51.812	1.00	88.31	O
ATOM	1814	OD2	ASP	A	231	16.375	-15.102	-51.969	1.00	88.31	O
ATOM	1815	N	SER	A	232	15.859	-18.734	-47.625	1.00	83.19	N
ATOM	1816	CA	SER	A	232	16.391	-19.250	-46.375	1.00	83.19	C
ATOM	1817	C	SER	A	232	17.641	-18.484	-45.938	1.00	83.19	C
ATOM	1818	CB	SER	A	232	16.703	-20.750	-46.500	1.00	83.19	C
ATOM	1819	O	SER	A	232	17.984	-18.469	-44.750	1.00	83.19	O
ATOM	1820	OG	SER	A	232	17.703	-20.969	-47.469	1.00	83.19	O
ATOM	1821	N	GLU	A	233	18.344	-17.797	-46.875	1.00	84.38	N
ATOM	1822	CA	GLU	A	233	19.547	-17.031	-46.562	1.00	84.38	C
ATOM	1823	C	GLU	A	233	19.250	-15.844	-45.656	1.00	84.38	C
ATOM	1824	CB	GLU	A	233	20.203	-16.547	-47.875	1.00	84.38	C
ATOM	1825	O	GLU	A	233	20.125	-15.375	-44.938	1.00	84.38	O
ATOM	1826	CG	GLU	A	233	21.562	-15.883	-47.656	1.00	84.38	C
ATOM	1827	CD	GLU	A	233	22.250	-15.492	-48.969	1.00	84.38	C
ATOM	1828	OE1	GLU	A	233	23.359	-14.922	-48.906	1.00	84.38	O
ATOM	1829	OE2	GLU	A	233	21.656	-15.758	-50.031	1.00	84.38	O
ATOM	1830	N	LEU	A	234	17.906	-15.320	-45.719	1.00	85.38	N
ATOM	1831	CA	LEU	A	234	17.547	-14.141	-44.938	1.00	85.38	C
ATOM	1832	C	LEU	A	234	17.578	-14.453	-43.469	1.00	85.38	C
ATOM	1833	CB	LEU	A	234	16.156	-13.633	-45.344	1.00	85.38	C
ATOM	1834	O	LEU	A	234	17.953	-13.602	-42.656	1.00	85.38	O
ATOM	1835	CG	LEU	A	234	16.047	-13.031	-46.750	1.00	85.38	C
ATOM	1836	CD1	LEU	A	234	14.586	-12.688	-47.062	1.00	85.38	C
ATOM	1837	CD2	LEU	A	234	16.922	-11.797	-46.875	1.00	85.38	C
ATOM	1838	N	PHE	A	235	17.234	-15.742	-43.125	1.00	86.06	N
ATOM	1839	CA	PHE	A	235	17.203	-16.203	-41.719	1.00	86.06	C
ATOM	1840	C	PHE	A	235	17.781	-17.594	-41.594	1.00	86.06	C
ATOM	1841	CB	PHE	A	235	15.766	-16.172	-41.188	1.00	86.06	C
ATOM	1842	O	PHE	A	235	17.047	-18.578	-41.500	1.00	86.06	O
ATOM	1843	CG	PHE	A	235	15.094	-14.836	-41.281	1.00	86.06	C
ATOM	1844	CD1	PHE	A	235	15.391	-13.820	-40.375	1.00	86.06	C
ATOM	1845	CD2	PHE	A	235	14.180	-14.578	-42.312	1.00	86.06	C
ATOM	1846	CE1	PHE	A	235	14.766	-12.578	-40.469	1.00	86.06	C
ATOM	1847	CE2	PHE	A	235	13.555	-13.336	-42.406	1.00	86.06	C
ATOM	1848	CZ	PHE	A	235	13.852	-12.336	-41.500	1.00	86.06	C
ATOM	1849	N	PRO	A	236	19.266	-17.766	-41.531	1.00	80.25	N
ATOM	1850	CA	PRO	A	236	19.969	-19.047	-41.594	1.00	80.25	C
ATOM	1851	C	PRO	A	236	19.781	-19.875	-40.312	1.00	80.25	C
ATOM	1852	CB	PRO	A	236	21.422	-18.641	-41.781	1.00	80.25	C

ATOM	1853	O	PRO	A	236	19.812	-21.109	-40.375	1.00	80.25	O
ATOM	1854	CG	PRO	A	236	21.469	-17.188	-41.406	1.00	80.25	C
ATOM	1855	CD	PRO	A	236	20.078	-16.641	-41.500	1.00	80.25	C
ATOM	1856	N	ASN	A	237	19.453	-19.312	-39.156	1.00	83.62	N
ATOM	1857	CA	ASN	A	237	19.578	-20.016	-37.875	1.00	83.62	C
ATOM	1858	C	ASN	A	237	18.203	-20.359	-37.312	1.00	83.62	C
ATOM	1859	CB	ASN	A	237	20.375	-19.172	-36.875	1.00	83.62	C
ATOM	1860	O	ASN	A	237	18.078	-21.266	-36.469	1.00	83.62	O
ATOM	1861	CG	ASN	A	237	21.812	-18.969	-37.312	1.00	83.62	C
ATOM	1862	ND2	ASN	A	237	22.391	-17.828	-36.938	1.00	83.62	N
ATOM	1863	OD1	ASN	A	237	22.406	-19.828	-37.969	1.00	83.62	O
ATOM	1864	N	ALA	A	238	17.125	-19.609	-37.719	1.00	85.12	N
ATOM	1865	CA	ALA	A	238	15.789	-19.859	-37.219	1.00	85.12	C
ATOM	1866	C	ALA	A	238	14.742	-19.062	-38.000	1.00	85.12	C
ATOM	1867	CB	ALA	A	238	15.727	-19.531	-35.719	1.00	85.12	C
ATOM	1868	O	ALA	A	238	15.062	-18.062	-38.625	1.00	85.12	O
ATOM	1869	N	LEU	A	239	13.531	-19.688	-37.938	1.00	86.12	N
ATOM	1870	CA	LEU	A	239	12.414	-18.984	-38.531	1.00	86.12	C
ATOM	1871	C	LEU	A	239	12.086	-17.719	-37.781	1.00	86.12	C
ATOM	1872	CB	LEU	A	239	11.188	-19.891	-38.625	1.00	86.12	C
ATOM	1873	O	LEU	A	239	12.047	-17.719	-36.531	1.00	86.12	O
ATOM	1874	CG	LEU	A	239	11.250	-21.047	-39.625	1.00	86.12	C
ATOM	1875	CD1	LEU	A	239	10.047	-21.969	-39.438	1.00	86.12	C
ATOM	1876	CD2	LEU	A	239	11.320	-20.516	-41.031	1.00	86.12	C
ATOM	1877	N	PRO	A	240	12.055	-16.562	-38.469	1.00	91.38	N
ATOM	1878	CA	PRO	A	240	11.688	-15.320	-37.781	1.00	91.38	C
ATOM	1879	C	PRO	A	240	10.258	-15.336	-37.250	1.00	91.38	C
ATOM	1880	CB	PRO	A	240	11.852	-14.258	-38.875	1.00	91.38	C
ATOM	1881	O	PRO	A	240	9.438	-16.141	-37.688	1.00	91.38	O
ATOM	1882	CG	PRO	A	240	11.523	-14.961	-40.156	1.00	91.38	C
ATOM	1883	CD	PRO	A	240	12.031	-16.375	-40.062	1.00	91.38	C
ATOM	1884	N	ASN	A	241	10.000	-14.523	-36.188	1.00	90.81	N
ATOM	1885	CA	ASN	A	241	8.625	-14.273	-35.781	1.00	90.81	C
ATOM	1886	C	ASN	A	241	7.855	-13.492	-36.844	1.00	90.81	C
ATOM	1887	CB	ASN	A	241	8.594	-13.523	-34.438	1.00	90.81	C
ATOM	1888	O	ASN	A	241	8.312	-12.438	-37.281	1.00	90.81	O
ATOM	1889	CG	ASN	A	241	7.223	-13.539	-33.812	1.00	90.81	C
ATOM	1890	ND2	ASN	A	241	6.977	-12.594	-32.906	1.00	90.81	N
ATOM	1891	OD1	ASN	A	241	6.391	-14.398	-34.094	1.00	90.81	O
ATOM	1892	N	ILE	A	242	6.785	-14.148	-37.312	1.00	91.56	N
ATOM	1893	CA	ILE	A	242	5.957	-13.523	-38.344	1.00	91.56	C
ATOM	1894	C	ILE	A	242	4.734	-12.883	-37.688	1.00	91.56	C
ATOM	1895	CB	ILE	A	242	5.527	-14.547	-39.406	1.00	91.56	C
ATOM	1896	O	ILE	A	242	3.932	-13.562	-37.031	1.00	91.56	O
ATOM	1897	CG1	ILE	A	242	6.754	-15.172	-40.062	1.00	91.56	C
ATOM	1898	CG2	ILE	A	242	4.605	-13.891	-40.438	1.00	91.56	C
ATOM	1899	CD1	ILE	A	242	6.430	-16.297	-41.062	1.00	91.56	C
ATOM	1900	N	SER	A	243	4.637	-11.562	-37.844	1.00	92.62	N
ATOM	1901	CA	SER	A	243	3.492	-10.844	-37.312	1.00	92.62	C
ATOM	1902	C	SER	A	243	2.822	-9.977	-38.375	1.00	92.62	C
ATOM	1903	CB	SER	A	243	3.924	-9.969	-36.125	1.00	92.62	C
ATOM	1904	O	SER	A	243	3.473	-9.539	-39.312	1.00	92.62	O
ATOM	1905	OG	SER	A	243	4.957	-9.078	-36.500	1.00	92.62	O
ATOM	1906	N	ILE	A	244	1.474	-9.805	-38.188	1.00	92.69	N
ATOM	1907	CA	ILE	A	244	0.668	-9.055	-39.156	1.00	92.69	C
ATOM	1908	C	ILE	A	244	-0.116	-7.969	-38.438	1.00	92.69	C
ATOM	1909	CB	ILE	A	244	-0.295	-9.984	-39.938	1.00	92.69	C
ATOM	1910	O	ILE	A	244	-0.781	-8.234	-37.438	1.00	92.69	O
ATOM	1911	CG1	ILE	A	244	0.483	-11.117	-40.594	1.00	92.69	C
ATOM	1912	CG2	ILE	A	244	-1.094	-9.180	-40.969	1.00	92.69	C
ATOM	1913	CD1	ILE	A	244	-0.398	-12.242	-41.125	1.00	92.69	C
ATOM	1914	N	LYS	A	245	0.049	-6.766	-38.969	1.00	91.94	N
ATOM	1915	CA	LYS	A	245	-0.727	-5.633	-38.469	1.00	91.94	C
ATOM	1916	C	LYS	A	245	-1.716	-5.133	-39.531	1.00	91.94	C

```
ATOM   1917  CB  LYS A 245       0.200  -4.496 -38.031  1.00 91.94           C
ATOM   1918  O   LYS A 245      -1.315  -4.535 -40.531  1.00 91.94           O
ATOM   1919  CG  LYS A 245      -0.521  -3.322 -37.406  1.00 91.94           C
ATOM   1920  CD  LYS A 245       0.447  -2.201 -37.031  1.00 91.94           C
ATOM   1921  CE  LYS A 245      -0.283  -0.993 -36.469  1.00 91.94           C
ATOM   1922  NZ  LYS A 245       0.656   0.126 -36.156  1.00 91.94           N
ATOM   1923  N   LYS A 246      -3.074  -5.406 -39.344  1.00 92.00           N
ATOM   1924  CA  LYS A 246      -4.121  -5.004 -40.281  1.00 92.00           C
ATOM   1925  C   LYS A 246      -5.488  -4.996 -39.594  1.00 92.00           C
ATOM   1926  CB  LYS A 246      -4.141  -5.934 -41.469  1.00 92.00           C
ATOM   1927  O   LYS A 246      -5.602  -5.281 -38.406  1.00 92.00           O
ATOM   1928  CG  LYS A 246      -4.684  -7.324 -41.188  1.00 92.00           C
ATOM   1929  CD  LYS A 246      -4.723  -8.188 -42.438  1.00 92.00           C
ATOM   1930  CE  LYS A 246      -5.680  -7.621 -43.469  1.00 92.00           C
ATOM   1931  NZ  LYS A 246      -6.457  -6.465 -42.938  1.00 92.00           N
ATOM   1932  N   LYS A 247      -6.430  -4.480 -40.344  1.00 93.12           N
ATOM   1933  CA  LYS A 247      -7.824  -4.617 -39.938  1.00 93.12           C
ATOM   1934  C   LYS A 247      -8.328  -6.039 -40.188  1.00 93.12           C
ATOM   1935  CB  LYS A 247      -8.703  -3.623 -40.688  1.00 93.12           C
ATOM   1936  O   LYS A 247      -8.242  -6.562 -41.281  1.00 93.12           O
ATOM   1937  CG  LYS A 247      -8.367  -2.166 -40.438  1.00 93.12           C
ATOM   1938  CD  LYS A 247      -9.188  -1.228 -41.312  1.00 93.12           C
ATOM   1939  CE  LYS A 247      -8.742   0.220 -41.156  1.00 93.12           C
ATOM   1940  NZ  LYS A 247      -9.477   1.131 -42.062  1.00 93.12           N
ATOM   1941  N   TRP A 248      -8.906  -6.672 -39.125  1.00 93.19           N
ATOM   1942  CA  TRP A 248      -9.156  -8.109 -39.219  1.00 93.19           C
ATOM   1943  C   TRP A 248     -10.633  -8.391 -39.438  1.00 93.19           C
ATOM   1944  CB  TRP A 248      -8.688  -8.805 -37.938  1.00 93.19           C
ATOM   1945  O   TRP A 248     -11.109  -8.328 -40.562  1.00 93.19           O
ATOM   1946  CG  TRP A 248      -7.195  -8.867 -37.781  1.00 93.19           C
ATOM   1947  CD1 TRP A 248      -6.418  -8.109 -36.938  1.00 93.19           C
ATOM   1948  CD2 TRP A 248      -6.301  -9.727 -38.500  1.00 93.19           C
ATOM   1949  CE2 TRP A 248      -4.996  -9.438 -38.031  1.00 93.19           C
ATOM   1950  CE3 TRP A 248      -6.477 -10.711 -39.469  1.00 93.19           C
ATOM   1951  NE1 TRP A 248      -5.094  -8.453 -37.094  1.00 93.19           N
ATOM   1952  CH2 TRP A 248      -4.066 -11.062 -39.469  1.00 93.19           C
ATOM   1953  CZ2 TRP A 248      -3.869 -10.102 -38.531  1.00 93.19           C
ATOM   1954  CZ3 TRP A 248      -5.352 -11.375 -39.938  1.00 93.19           C
ATOM   1955  N   ASN A 249     -11.375  -8.797 -38.469  1.00 93.12           N
ATOM   1956  CA  ASN A 249     -12.703  -9.391 -38.625  1.00 93.12           C
ATOM   1957  C   ASN A 249     -13.719  -8.359 -39.094  1.00 93.12           C
ATOM   1958  CB  ASN A 249     -13.164 -10.039 -37.344  1.00 93.12           C
ATOM   1959  O   ASN A 249     -13.727  -7.223 -38.625  1.00 93.12           O
ATOM   1960  CG  ASN A 249     -12.523 -11.391 -37.094  1.00 93.12           C
ATOM   1961  ND2 ASN A 249     -12.805 -11.984 -35.938  1.00 93.12           N
ATOM   1962  OD1 ASN A 249     -11.773 -11.891 -37.938  1.00 93.12           O
ATOM   1963  N   ILE A 250     -14.508  -8.742 -40.125  1.00 94.44           N
ATOM   1964  CA  ILE A 250     -15.734  -8.078 -40.562  1.00 94.44           C
ATOM   1965  C   ILE A 250     -16.906  -8.578 -39.719  1.00 94.44           C
ATOM   1966  CB  ILE A 250     -16.000  -8.305 -42.062  1.00 94.44           C
ATOM   1967  O   ILE A 250     -17.188  -9.773 -39.688  1.00 94.44           O
ATOM   1968  CG1 ILE A 250     -14.859  -7.715 -42.906  1.00 94.44           C
ATOM   1969  CG2 ILE A 250     -17.344  -7.699 -42.469  1.00 94.44           C
ATOM   1970  CD1 ILE A 250     -14.969  -8.031 -44.375  1.00 94.44           C
ATOM   1971  N   ILE A 251     -17.562  -7.586 -39.000  1.00 94.50           N
ATOM   1972  CA  ILE A 251     -18.594  -7.930 -38.031  1.00 94.50           C
ATOM   1973  C   ILE A 251     -19.875  -7.156 -38.344  1.00 94.50           C
ATOM   1974  CB  ILE A 251     -18.125  -7.641 -36.594  1.00 94.50           C
ATOM   1975  O   ILE A 251     -19.891  -6.297 -39.219  1.00 94.50           O
ATOM   1976  CG1 ILE A 251     -17.766  -6.160 -36.438  1.00 94.50           C
ATOM   1977  CG2 ILE A 251     -16.938  -8.539 -36.219  1.00 94.50           C
ATOM   1978  CD1 ILE A 251     -17.312  -5.777 -35.031  1.00 94.50           C
ATOM   1979  N   ASN A 252     -20.938  -7.578 -37.594  1.00 94.00           N
ATOM   1980  CA  ASN A 252     -22.203  -6.871 -37.750  1.00 94.00           C
```

ATOM	1981	C	ASN	A	252	-22.172	-5.504	-37.062	1.00	94.00	C
ATOM	1982	CB	ASN	A	252	-23.359	-7.707	-37.188	1.00	94.00	C
ATOM	1983	O	ASN	A	252	-21.797	-5.398	-35.875	1.00	94.00	O
ATOM	1984	CG	ASN	A	252	-23.703	-8.891	-38.062	1.00	94.00	C
ATOM	1985	ND2	ASN	A	252	-24.062	-10.008	-37.438	1.00	94.00	N
ATOM	1986	OD1	ASN	A	252	-23.656	-8.797	-39.281	1.00	94.00	O
ATOM	1987	N	VAL	A	253	-22.438	-4.504	-37.906	1.00	95.75	N
ATOM	1988	CA	VAL	A	253	-22.609	-3.152	-37.375	1.00	95.75	C
ATOM	1989	C	VAL	A	253	-23.984	-2.607	-37.812	1.00	95.75	C
ATOM	1990	CB	VAL	A	253	-21.500	-2.211	-37.875	1.00	95.75	C
ATOM	1991	O	VAL	A	253	-24.625	-3.162	-38.688	1.00	95.75	O
ATOM	1992	CG1	VAL	A	253	-20.125	-2.721	-37.406	1.00	95.75	C
ATOM	1993	CG2	VAL	A	253	-21.531	-2.070	-39.375	1.00	95.75	C
ATOM	1994	N	THR	A	254	-24.422	-1.566	-37.156	1.00	96.88	N
ATOM	1995	CA	THR	A	254	-25.703	-0.997	-37.500	1.00	96.88	C
ATOM	1996	C	THR	A	254	-25.672	-0.378	-38.906	1.00	96.88	C
ATOM	1997	CB	THR	A	254	-26.156	0.066	-36.500	1.00	96.88	C
ATOM	1998	O	THR	A	254	-24.625	0.134	-39.344	1.00	96.88	O
ATOM	1999	CG2	THR	A	254	-26.250	-0.525	-35.094	1.00	96.88	C
ATOM	2000	OG1	THR	A	254	-25.203	1.139	-36.500	1.00	96.88	O
ATOM	2001	N	SER	A	255	-26.797	-0.399	-39.625	1.00	95.81	N
ATOM	2002	CA	SER	A	255	-26.875	0.015	-41.000	1.00	95.81	C
ATOM	2003	C	SER	A	255	-26.594	1.507	-41.156	1.00	95.81	C
ATOM	2004	CB	SER	A	255	-28.234	-0.321	-41.594	1.00	95.81	C
ATOM	2005	O	SER	A	255	-26.156	1.960	-42.219	1.00	95.81	O
ATOM	2006	OG	SER	A	255	-29.281	0.269	-40.844	1.00	95.81	O
ATOM	2007	N	ASN	A	256	-26.797	2.330	-40.125	1.00	96.19	N
ATOM	2008	CA	ASN	A	256	-26.609	3.775	-40.188	1.00	96.19	C
ATOM	2009	C	ASN	A	256	-25.219	4.184	-39.688	1.00	96.19	C
ATOM	2010	CB	ASN	A	256	-27.703	4.500	-39.375	1.00	96.19	C
ATOM	2011	O	ASN	A	256	-24.938	5.375	-39.531	1.00	96.19	O
ATOM	2012	CG	ASN	A	256	-27.609	4.238	-37.906	1.00	96.19	C
ATOM	2013	ND2	ASN	A	256	-28.156	5.148	-37.094	1.00	96.19	N
ATOM	2014	OD1	ASN	A	256	-27.047	3.227	-37.469	1.00	96.19	O
ATOM	2015	N	TYR	A	257	-24.344	3.205	-39.312	1.00	97.00	N
ATOM	2016	CA	TYR	A	257	-23.047	3.432	-38.688	1.00	97.00	C
ATOM	2017	C	TYR	A	257	-21.922	3.357	-39.750	1.00	97.00	C
ATOM	2018	CB	TYR	A	257	-22.797	2.410	-37.594	1.00	97.00	C
ATOM	2019	O	TYR	A	257	-21.891	2.441	-40.562	1.00	97.00	O
ATOM	2020	CG	TYR	A	257	-21.406	2.506	-36.969	1.00	97.00	C
ATOM	2021	CD1	TYR	A	257	-20.547	1.411	-36.969	1.00	97.00	C
ATOM	2022	CD2	TYR	A	257	-20.969	3.693	-36.406	1.00	97.00	C
ATOM	2023	CE1	TYR	A	257	-19.281	1.494	-36.406	1.00	97.00	C
ATOM	2024	CE2	TYR	A	257	-19.703	3.787	-35.844	1.00	97.00	C
ATOM	2025	OH	TYR	A	257	-17.609	2.773	-35.281	1.00	97.00	O
ATOM	2026	CZ	TYR	A	257	-18.859	2.686	-35.844	1.00	97.00	C
ATOM	2027	N	ASP	A	258	-21.047	4.383	-39.750	1.00	95.75	N
ATOM	2028	CA	ASP	A	258	-19.797	4.363	-40.500	1.00	95.75	C
ATOM	2029	C	ASP	A	258	-18.609	4.109	-39.562	1.00	95.75	C
ATOM	2030	CB	ASP	A	258	-19.594	5.680	-41.250	1.00	95.75	C
ATOM	2031	O	ASP	A	258	-18.219	4.988	-38.781	1.00	95.75	O
ATOM	2032	CG	ASP	A	258	-18.312	5.699	-42.062	1.00	95.75	C
ATOM	2033	OD1	ASP	A	258	-17.547	4.719	-42.031	1.00	95.75	O
ATOM	2034	OD2	ASP	A	258	-18.078	6.711	-42.781	1.00	95.75	O
ATOM	2035	N	PRO	A	259	-17.938	2.922	-39.688	1.00	94.50	N
ATOM	2036	CA	PRO	A	259	-16.938	2.490	-38.719	1.00	94.50	C
ATOM	2037	C	PRO	A	259	-15.633	3.283	-38.812	1.00	94.50	C
ATOM	2038	CB	PRO	A	259	-16.703	1.020	-39.094	1.00	94.50	C
ATOM	2039	O	PRO	A	259	-14.867	3.354	-37.844	1.00	94.50	O
ATOM	2040	CG	PRO	A	259	-17.109	0.910	-40.500	1.00	94.50	C
ATOM	2041	CD	PRO	A	259	-18.234	1.872	-40.781	1.00	94.50	C
ATOM	2042	N	ILE	A	260	-15.383	3.887	-40.031	1.00	93.94	N
ATOM	2043	CA	ILE	A	260	-14.125	4.605	-40.250	1.00	93.94	C
ATOM	2044	C	ILE	A	260	-14.227	6.000	-39.625	1.00	93.94	C

ATOM	2045	CB	ILE	A	260	-13.758	4.695	-41.750	1.00	93.94	C
ATOM	2046	O	ILE	A	260	-13.367	6.410	-38.844	1.00	93.94	O
ATOM	2047	CG1	ILE	A	260	-13.641	3.293	-42.344	1.00	93.94	C
ATOM	2048	CG2	ILE	A	260	-12.453	5.480	-41.938	1.00	93.94	C
ATOM	2049	CD1	ILE	A	260	-12.680	2.373	-41.625	1.00	93.94	C
ATOM	2050	N	SER	A	261	-15.305	6.695	-40.000	1.00	94.12	N
ATOM	2051	CA	SER	A	261	-15.492	8.039	-39.469	1.00	94.12	C
ATOM	2052	C	SER	A	261	-16.156	8.008	-38.094	1.00	94.12	C
ATOM	2053	CB	SER	A	261	-16.328	8.883	-40.406	1.00	94.12	C
ATOM	2054	O	SER	A	261	-16.188	9.023	-37.375	1.00	94.12	O
ATOM	2055	OG	SER	A	261	-17.625	8.328	-40.562	1.00	94.12	O
ATOM	2056	N	ARG	A	262	-16.828	6.848	-37.719	1.00	94.75	N
ATOM	2057	CA	ARG	A	262	-17.484	6.617	-36.438	1.00	94.75	C
ATOM	2058	C	ARG	A	262	-18.688	7.539	-36.250	1.00	94.75	C
ATOM	2059	CB	ARG	A	262	-16.500	6.812	-35.281	1.00	94.75	C
ATOM	2060	O	ARG	A	262	-18.891	8.109	-35.188	1.00	94.75	O
ATOM	2061	CG	ARG	A	262	-15.289	5.891	-35.344	1.00	94.75	C
ATOM	2062	CD	ARG	A	262	-14.461	5.980	-34.062	1.00	94.75	C
ATOM	2063	NE	ARG	A	262	-13.406	4.969	-34.031	1.00	94.75	N
ATOM	2064	NH1	ARG	A	262	-14.586	3.469	-32.750	1.00	94.75	N
ATOM	2065	NH2	ARG	A	262	-12.469	2.953	-33.438	1.00	94.75	N
ATOM	2066	CZ	ARG	A	262	-13.492	3.799	-33.406	1.00	94.75	C
ATOM	2067	N	THR	A	263	-19.438	7.746	-37.250	1.00	94.88	N
ATOM	2068	CA	THR	A	263	-20.609	8.609	-37.281	1.00	94.88	C
ATOM	2069	C	THR	A	263	-21.875	7.785	-37.531	1.00	94.88	C
ATOM	2070	CB	THR	A	263	-20.484	9.688	-38.375	1.00	94.88	C
ATOM	2071	O	THR	A	263	-21.828	6.738	-38.188	1.00	94.88	O
ATOM	2072	CG2	THR	A	263	-19.312	10.617	-38.094	1.00	94.88	C
ATOM	2073	OG1	THR	A	263	-20.266	9.047	-39.656	1.00	94.88	O
ATOM	2074	N	TYR	A	264	-23.016	8.234	-36.938	1.00	95.94	N
ATOM	2075	CA	TYR	A	264	-24.328	7.621	-37.062	1.00	95.94	C
ATOM	2076	C	TYR	A	264	-25.266	8.531	-37.844	1.00	95.94	C
ATOM	2077	CB	TYR	A	264	-24.922	7.301	-35.688	1.00	95.94	C
ATOM	2078	O	TYR	A	264	-25.594	9.633	-37.406	1.00	95.94	O
ATOM	2079	CG	TYR	A	264	-24.094	6.336	-34.875	1.00	95.94	C
ATOM	2080	CD1	TYR	A	264	-24.469	5.004	-34.750	1.00	95.94	C
ATOM	2081	CD2	TYR	A	264	-22.922	6.758	-34.250	1.00	95.94	C
ATOM	2082	CE1	TYR	A	264	-23.703	4.109	-34.000	1.00	95.94	C
ATOM	2083	CE2	TYR	A	264	-22.156	5.871	-33.500	1.00	95.94	C
ATOM	2084	OH	TYR	A	264	-21.797	3.674	-32.625	1.00	95.94	O
ATOM	2085	CZ	TYR	A	264	-22.547	4.555	-33.375	1.00	95.94	C
ATOM	2086	N	SER	A	265	-25.656	8.141	-39.000	1.00	94.19	N
ATOM	2087	CA	SER	A	265	-26.500	8.984	-39.844	1.00	94.19	C
ATOM	2088	C	SER	A	265	-27.969	8.602	-39.719	1.00	94.19	C
ATOM	2089	CB	SER	A	265	-26.062	8.891	-41.312	1.00	94.19	C
ATOM	2090	O	SER	A	265	-28.391	7.543	-40.188	1.00	94.19	O
ATOM	2091	OG	SER	A	265	-24.719	9.281	-41.469	1.00	94.19	O
ATOM	2092	N	GLY	A	266	-28.812	9.453	-39.000	1.00	92.38	N
ATOM	2093	CA	GLY	A	266	-30.234	9.195	-38.812	1.00	92.38	C
ATOM	2094	C	GLY	A	266	-30.500	8.172	-37.719	1.00	92.38	C
ATOM	2095	O	GLY	A	266	-29.578	7.703	-37.062	1.00	92.38	O
ATOM	2096	N	ALA	A	267	-31.812	8.000	-37.375	1.00	90.94	N
ATOM	2097	CA	ALA	A	267	-32.219	6.977	-36.406	1.00	90.94	C
ATOM	2098	C	ALA	A	267	-32.031	5.578	-36.969	1.00	90.94	C
ATOM	2099	CB	ALA	A	267	-33.688	7.176	-36.031	1.00	90.94	C
ATOM	2100	O	ALA	A	267	-32.188	5.359	-38.188	1.00	90.94	O
ATOM	2101	N	TRP	A	268	-31.438	4.668	-36.281	1.00	94.12	N
ATOM	2102	CA	TRP	A	268	-31.219	3.283	-36.688	1.00	94.12	C
ATOM	2103	C	TRP	A	268	-32.531	2.531	-36.812	1.00	94.12	C
ATOM	2104	CB	TRP	A	268	-30.297	2.570	-35.719	1.00	94.12	C
ATOM	2105	O	TRP	A	268	-33.375	2.562	-35.906	1.00	94.12	O
ATOM	2106	CG	TRP	A	268	-30.031	1.133	-36.031	1.00	94.12	C
ATOM	2107	CD1	TRP	A	268	-29.688	0.620	-37.250	1.00	94.12	C
ATOM	2108	CD2	TRP	A	268	-30.094	0.021	-35.125	1.00	94.12	C

ATOM	2109	CE2	TRP	A	268	-29.781	-1.137	-35.875	1.00	94.12	C
ATOM	2110	CE3	TRP	A	268	-30.391	-0.109	-33.781	1.00	94.12	C
ATOM	2111	NE1	TRP	A	268	-29.547	-0.745	-37.188	1.00	94.12	N
ATOM	2112	CH2	TRP	A	268	-30.031	-2.508	-33.969	1.00	94.12	C
ATOM	2113	CZ2	TRP	A	268	-29.750	-2.410	-35.312	1.00	94.12	C
ATOM	2114	CZ3	TRP	A	268	-30.359	-1.376	-33.219	1.00	94.12	C
ATOM	2115	N	ASP	A	269	-32.812	1.749	-37.812	1.00	91.94	N
ATOM	2116	CA	ASP	A	269	-34.062	1.064	-38.156	1.00	91.94	C
ATOM	2117	C	ASP	A	269	-34.062	-0.366	-37.625	1.00	91.94	C
ATOM	2118	CB	ASP	A	269	-34.281	1.062	-39.656	1.00	91.94	C
ATOM	2119	O	ASP	A	269	-34.969	-1.145	-37.906	1.00	91.94	O
ATOM	2120	CG	ASP	A	269	-33.188	0.335	-40.438	1.00	91.94	C
ATOM	2121	OD1	ASP	A	269	-32.281	-0.230	-39.781	1.00	91.94	O
ATOM	2122	OD2	ASP	A	269	-33.250	0.330	-41.688	1.00	91.94	O
ATOM	2123	N	GLY	A	270	-33.000	-0.773	-36.938	1.00	91.25	N
ATOM	2124	CA	GLY	A	270	-32.938	-2.084	-36.312	1.00	91.25	C
ATOM	2125	C	GLY	A	270	-32.250	-3.121	-37.156	1.00	91.25	C
ATOM	2126	O	GLY	A	270	-32.125	-4.285	-36.781	1.00	91.25	O
ATOM	2127	N	THR	A	271	-31.734	-2.756	-38.375	1.00	93.75	N
ATOM	2128	CA	THR	A	271	-31.078	-3.686	-39.312	1.00	93.75	C
ATOM	2129	C	THR	A	271	-29.562	-3.576	-39.188	1.00	93.75	C
ATOM	2130	CB	THR	A	271	-31.516	-3.430	-40.750	1.00	93.75	C
ATOM	2131	O	THR	A	271	-29.031	-2.549	-38.750	1.00	93.75	O
ATOM	2132	CG2	THR	A	271	-33.000	-3.611	-40.938	1.00	93.75	C
ATOM	2133	OG1	THR	A	271	-31.156	-2.090	-41.125	1.00	93.75	O
ATOM	2134	N	TRP	A	272	-28.844	-4.695	-39.562	1.00	94.06	N
ATOM	2135	CA	TRP	A	272	-27.391	-4.793	-39.469	1.00	94.06	C
ATOM	2136	C	TRP	A	272	-26.734	-4.836	-40.844	1.00	94.06	C
ATOM	2137	CB	TRP	A	272	-27.000	-6.035	-38.656	1.00	94.06	C
ATOM	2138	O	TRP	A	272	-27.359	-5.246	-41.812	1.00	94.06	O
ATOM	2139	CG	TRP	A	272	-27.547	-6.055	-37.250	1.00	94.06	C
ATOM	2140	CD1	TRP	A	272	-28.750	-6.547	-36.844	1.00	94.06	C
ATOM	2141	CD2	TRP	A	272	-26.891	-5.547	-36.094	1.00	94.06	C
ATOM	2142	CE2	TRP	A	272	-27.766	-5.770	-35.000	1.00	94.06	C
ATOM	2143	CE3	TRP	A	272	-25.656	-4.930	-35.844	1.00	94.06	C
ATOM	2144	NE1	TRP	A	272	-28.891	-6.379	-35.500	1.00	94.06	N
ATOM	2145	CH2	TRP	A	272	-26.234	-4.789	-33.500	1.00	94.06	C
ATOM	2146	CZ2	TRP	A	272	-27.453	-5.395	-33.688	1.00	94.06	C
ATOM	2147	CZ3	TRP	A	272	-25.344	-4.555	-34.562	1.00	94.06	C
ATOM	2148	N	LYS	A	273	-25.500	-4.355	-41.031	1.00	95.12	N
ATOM	2149	CA	LYS	A	273	-24.625	-4.543	-42.188	1.00	95.12	C
ATOM	2150	C	LYS	A	273	-23.266	-5.086	-41.750	1.00	95.12	C
ATOM	2151	CB	LYS	A	273	-24.453	-3.229	-42.938	1.00	95.12	C
ATOM	2152	O	LYS	A	273	-22.906	-5.023	-40.562	1.00	95.12	O
ATOM	2153	CG	LYS	A	273	-23.656	-2.176	-42.188	1.00	95.12	C
ATOM	2154	CD	LYS	A	273	-23.484	-0.908	-43.000	1.00	95.12	C
ATOM	2155	CE	LYS	A	273	-22.672	0.139	-42.250	1.00	95.12	C
ATOM	2156	NZ	LYS	A	273	-22.500	1.384	-43.062	1.00	95.12	N
ATOM	2157	N	LYS	A	274	-22.469	-5.695	-42.656	1.00	93.56	N
ATOM	2158	CA	LYS	A	274	-21.141	-6.234	-42.375	1.00	93.56	C
ATOM	2159	C	LYS	A	274	-20.062	-5.219	-42.688	1.00	93.56	C
ATOM	2160	CB	LYS	A	274	-20.906	-7.523	-43.156	1.00	93.56	C
ATOM	2161	O	LYS	A	274	-20.000	-4.676	-43.781	1.00	93.56	O
ATOM	2162	CG	LYS	A	274	-21.781	-8.688	-42.719	1.00	93.56	C
ATOM	2163	CD	LYS	A	274	-21.266	-9.352	-41.469	1.00	93.56	C
ATOM	2164	CE	LYS	A	274	-21.953	-10.680	-41.188	1.00	93.56	C
ATOM	2165	NZ	LYS	A	274	-21.406	-11.352	-39.969	1.00	93.56	N
ATOM	2166	N	ALA	A	275	-19.250	-4.934	-41.656	1.00	96.00	N
ATOM	2167	CA	ALA	A	275	-18.141	-4.008	-41.812	1.00	96.00	C
ATOM	2168	C	ALA	A	275	-17.094	-4.219	-40.750	1.00	96.00	C
ATOM	2169	CB	ALA	A	275	-18.641	-2.564	-41.812	1.00	96.00	C
ATOM	2170	O	ALA	A	275	-17.375	-4.781	-39.688	1.00	96.00	O
ATOM	2171	N	TRP	A	276	-15.867	-3.811	-41.125	1.00	94.75	N
ATOM	2172	CA	TRP	A	276	-14.844	-3.801	-40.094	1.00	94.75	C

ATOM	2173	C	TRP	A	276	-15.156	-2.754	-39.031	1.00	94.75	C
ATOM	2174	CB	TRP	A	276	-13.469	-3.531	-40.719	1.00	94.75	C
ATOM	2175	O	TRP	A	276	-15.594	-1.646	-39.344	1.00	94.75	O
ATOM	2176	CG	TRP	A	276	-12.375	-3.379	-39.688	1.00	94.75	C
ATOM	2177	CD1	TRP	A	276	-11.617	-4.375	-39.125	1.00	94.75	C
ATOM	2178	CD2	TRP	A	276	-11.914	-2.156	-39.094	1.00	94.75	C
ATOM	2179	CE2	TRP	A	276	-10.883	-2.486	-38.188	1.00	94.75	C
ATOM	2180	CE3	TRP	A	276	-12.289	-0.813	-39.250	1.00	94.75	C
ATOM	2181	NE1	TRP	A	276	-10.711	-3.846	-38.250	1.00	94.75	N
ATOM	2182	CH2	TRP	A	276	-10.586	-0.213	-37.625	1.00	94.75	C
ATOM	2183	CZ2	TRP	A	276	-10.203	-1.521	-37.438	1.00	94.75	C
ATOM	2184	CZ3	TRP	A	276	-11.617	0.147	-38.500	1.00	94.75	C
ATOM	2185	N	SER	A	277	-14.953	-3.184	-37.750	1.00	95.69	N
ATOM	2186	CA	SER	A	277	-15.094	-2.219	-36.656	1.00	95.69	C
ATOM	2187	C	SER	A	277	-14.328	-2.662	-35.406	1.00	95.69	C
ATOM	2188	CB	SER	A	277	-16.578	-2.021	-36.312	1.00	95.69	C
ATOM	2189	O	SER	A	277	-14.211	-3.859	-35.156	1.00	95.69	O
ATOM	2190	OG	SER	A	277	-16.719	-1.095	-35.250	1.00	95.69	O
ATOM	2191	N	ASN	A	278	-13.758	-1.769	-34.812	1.00	95.50	N
ATOM	2192	CA	ASN	A	278	-13.148	-2.074	-33.500	1.00	95.50	C
ATOM	2193	C	ASN	A	278	-13.883	-1.381	-32.375	1.00	95.50	C
ATOM	2194	CB	ASN	A	278	-11.672	-1.683	-33.500	1.00	95.50	C
ATOM	2195	O	ASN	A	278	-13.281	-1.042	-31.344	1.00	95.50	O
ATOM	2196	CG	ASN	A	278	-11.461	-0.199	-33.719	1.00	95.50	C
ATOM	2197	ND2	ASN	A	278	-10.203	0.238	-33.656	1.00	95.50	N
ATOM	2198	OD1	ASN	A	278	-12.414	0.550	-33.969	1.00	95.50	O
ATOM	2199	N	ASN	A	279	-15.062	-1.027	-32.562	1.00	96.38	N
ATOM	2200	CA	ASN	A	279	-15.977	-0.610	-31.516	1.00	96.38	C
ATOM	2201	C	ASN	A	279	-16.375	-1.781	-30.625	1.00	96.38	C
ATOM	2202	CB	ASN	A	279	-17.234	0.043	-32.125	1.00	96.38	C
ATOM	2203	O	ASN	A	279	-16.938	-2.766	-31.094	1.00	96.38	O
ATOM	2204	CG	ASN	A	279	-18.109	0.679	-31.062	1.00	96.38	C
ATOM	2205	ND2	ASN	A	279	-18.594	1.885	-31.344	1.00	96.38	N
ATOM	2206	OD1	ASN	A	279	-18.359	0.093	-30.000	1.00	96.38	O
ATOM	2207	N	PRO	A	280	-15.992	-1.697	-29.312	1.00	95.94	N
ATOM	2208	CA	PRO	A	280	-16.172	-2.834	-28.406	1.00	95.94	C
ATOM	2209	C	PRO	A	280	-17.625	-3.301	-28.328	1.00	95.94	C
ATOM	2210	CB	PRO	A	280	-15.695	-2.285	-27.062	1.00	95.94	C
ATOM	2211	O	PRO	A	280	-17.891	-4.477	-28.062	1.00	95.94	O
ATOM	2212	CG	PRO	A	280	-15.781	-0.800	-27.203	1.00	95.94	C
ATOM	2213	CD	PRO	A	280	-15.562	-0.443	-28.641	1.00	95.94	C
ATOM	2214	N	ALA	A	281	-18.656	-2.420	-28.500	1.00	95.81	N
ATOM	2215	CA	ALA	A	281	-20.062	-2.801	-28.453	1.00	95.81	C
ATOM	2216	C	ALA	A	281	-20.422	-3.754	-29.594	1.00	95.81	C
ATOM	2217	CB	ALA	A	281	-20.953	-1.559	-28.516	1.00	95.81	C
ATOM	2218	O	ALA	A	281	-21.047	-4.789	-29.375	1.00	95.81	O
ATOM	2219	N	PHE	A	282	-19.953	-3.467	-30.734	1.00	95.62	N
ATOM	2220	CA	PHE	A	282	-20.234	-4.293	-31.906	1.00	95.62	C
ATOM	2221	C	PHE	A	282	-19.406	-5.578	-31.875	1.00	95.62	C
ATOM	2222	CB	PHE	A	282	-19.938	-3.523	-33.188	1.00	95.62	C
ATOM	2223	O	PHE	A	282	-19.859	-6.621	-32.344	1.00	95.62	O
ATOM	2224	CG	PHE	A	282	-20.875	-2.367	-33.438	1.00	95.62	C
ATOM	2225	CD1	PHE	A	282	-22.250	-2.512	-33.250	1.00	95.62	C
ATOM	2226	CD2	PHE	A	282	-20.375	-1.135	-33.844	1.00	95.62	C
ATOM	2227	CE1	PHE	A	282	-23.109	-1.443	-33.469	1.00	95.62	C
ATOM	2228	CE2	PHE	A	282	-21.234	-0.062	-34.062	1.00	95.62	C
ATOM	2229	CZ	PHE	A	282	-22.609	-0.219	-33.875	1.00	95.62	C
ATOM	2230	N	VAL	A	283	-18.188	-5.480	-31.328	1.00	94.94	N
ATOM	2231	CA	VAL	A	283	-17.391	-6.680	-31.125	1.00	94.94	C
ATOM	2232	C	VAL	A	283	-18.109	-7.621	-30.156	1.00	94.94	C
ATOM	2233	CB	VAL	A	283	-15.977	-6.332	-30.578	1.00	94.94	C
ATOM	2234	O	VAL	A	283	-18.156	-8.828	-30.391	1.00	94.94	O
ATOM	2235	CG1	VAL	A	283	-15.211	-7.602	-30.219	1.00	94.94	C
ATOM	2236	CG2	VAL	A	283	-15.203	-5.516	-31.609	1.00	94.94	C

ATOM	2237	N	LEU	A	284	-18.625	-7.078	-29.078	1.00	93.88	N
ATOM	2238	CA	LEU	A	284	-19.406	-7.855	-28.125	1.00	93.88	C
ATOM	2239	C	LEU	A	284	-20.594	-8.516	-28.797	1.00	93.88	C
ATOM	2240	CB	LEU	A	284	-19.875	-6.965	-26.969	1.00	93.88	C
ATOM	2241	O	LEU	A	284	-20.844	-9.711	-28.594	1.00	93.88	O
ATOM	2242	CG	LEU	A	284	-20.688	-7.648	-25.875	1.00	93.88	C
ATOM	2243	CD1	LEU	A	284	-19.859	-8.727	-25.188	1.00	93.88	C
ATOM	2244	CD2	LEU	A	284	-21.188	-6.625	-24.859	1.00	93.88	C
ATOM	2245	N	TYR	A	285	-21.328	-7.742	-29.547	1.00	92.62	N
ATOM	2246	CA	TYR	A	285	-22.484	-8.266	-30.266	1.00	92.62	C
ATOM	2247	C	TYR	A	285	-22.094	-9.438	-31.156	1.00	92.62	C
ATOM	2248	CB	TYR	A	285	-23.141	-7.172	-31.125	1.00	92.62	C
ATOM	2249	O	TYR	A	285	-22.766	-10.469	-31.188	1.00	92.62	O
ATOM	2250	CG	TYR	A	285	-24.344	-7.645	-31.906	1.00	92.62	C
ATOM	2251	CD1	TYR	A	285	-24.234	-7.941	-33.250	1.00	92.62	C
ATOM	2252	CD2	TYR	A	285	-25.578	-7.789	-31.297	1.00	92.62	C
ATOM	2253	CE1	TYR	A	285	-25.344	-8.375	-34.000	1.00	92.62	C
ATOM	2254	CE2	TYR	A	285	-26.688	-8.219	-32.000	1.00	92.62	C
ATOM	2255	OH	TYR	A	285	-27.656	-8.938	-34.062	1.00	92.62	O
ATOM	2256	CZ	TYR	A	285	-26.562	-8.516	-33.344	1.00	92.62	C
ATOM	2257	N	ASP	A	286	-21.062	-9.297	-31.859	1.00	92.00	N
ATOM	2258	CA	ASP	A	286	-20.578	-10.328	-32.781	1.00	92.00	C
ATOM	2259	C	ASP	A	286	-20.219	-11.609	-32.031	1.00	92.00	C
ATOM	2260	CB	ASP	A	286	-19.391	-9.828	-33.562	1.00	92.00	C
ATOM	2261	O	ASP	A	286	-20.547	-12.711	-32.469	1.00	92.00	O
ATOM	2262	CG	ASP	A	286	-18.984	-10.773	-34.688	1.00	92.00	C
ATOM	2263	OD1	ASP	A	286	-19.781	-11.016	-35.625	1.00	92.00	O
ATOM	2264	OD2	ASP	A	286	-17.844	-11.281	-34.656	1.00	92.00	O
ATOM	2265	N	LEU	A	287	-19.500	-11.492	-30.906	1.00	90.56	N
ATOM	2266	CA	LEU	A	287	-19.078	-12.633	-30.109	1.00	90.56	C
ATOM	2267	C	LEU	A	287	-20.281	-13.398	-29.562	1.00	90.56	C
ATOM	2268	CB	LEU	A	287	-18.188	-12.172	-28.953	1.00	90.56	C
ATOM	2269	O	LEU	A	287	-20.266	-14.633	-29.516	1.00	90.56	O
ATOM	2270	CG	LEU	A	287	-16.734	-11.828	-29.312	1.00	90.56	C
ATOM	2271	CD1	LEU	A	287	-16.031	-11.195	-28.125	1.00	90.56	C
ATOM	2272	CD2	LEU	A	287	-15.984	-13.070	-29.781	1.00	90.56	C
ATOM	2273	N	ILE	A	288	-21.297	-12.656	-29.234	1.00	89.69	N
ATOM	2274	CA	ILE	A	288	-22.484	-13.258	-28.641	1.00	89.69	C
ATOM	2275	C	ILE	A	288	-23.328	-13.945	-29.719	1.00	89.69	C
ATOM	2276	CB	ILE	A	288	-23.344	-12.211	-27.891	1.00	89.69	C
ATOM	2277	O	ILE	A	288	-23.844	-15.039	-29.516	1.00	89.69	O
ATOM	2278	CG1	ILE	A	288	-22.594	-11.680	-26.672	1.00	89.69	C
ATOM	2279	CG2	ILE	A	288	-24.703	-12.797	-27.484	1.00	89.69	C
ATOM	2280	CD1	ILE	A	288	-23.250	-10.484	-26.000	1.00	89.69	C
ATOM	2281	N	THR	A	289	-23.391	-13.398	-30.938	1.00	88.75	N
ATOM	2282	CA	THR	A	289	-24.359	-13.844	-31.938	1.00	88.75	C
ATOM	2283	C	THR	A	289	-23.672	-14.758	-32.969	1.00	88.75	C
ATOM	2284	CB	THR	A	289	-25.000	-12.648	-32.656	1.00	88.75	C
ATOM	2285	O	THR	A	289	-24.344	-15.578	-33.594	1.00	88.75	O
ATOM	2286	CG2	THR	A	289	-25.844	-11.820	-31.703	1.00	88.75	C
ATOM	2287	OG1	THR	A	289	-23.984	-11.820	-33.219	1.00	88.75	O
ATOM	2288	N	ASN	A	290	-22.359	-14.695	-33.094	1.00	87.69	N
ATOM	2289	CA	ASN	A	290	-21.703	-15.461	-34.156	1.00	87.69	C
ATOM	2290	C	ASN	A	290	-21.719	-16.953	-33.875	1.00	87.69	C
ATOM	2291	CB	ASN	A	290	-20.250	-14.977	-34.344	1.00	87.69	C
ATOM	2292	O	ASN	A	290	-21.281	-17.391	-32.781	1.00	87.69	O
ATOM	2293	CG	ASN	A	290	-19.625	-15.500	-35.594	1.00	87.69	C
ATOM	2294	ND2	ASN	A	290	-19.234	-14.586	-36.500	1.00	87.69	N
ATOM	2295	OD1	ASN	A	290	-19.469	-16.703	-35.781	1.00	87.69	O
ATOM	2296	N	GLN	A	291	-22.062	-17.828	-34.750	1.00	83.75	N
ATOM	2297	CA	GLN	A	291	-22.250	-19.281	-34.594	1.00	83.75	C
ATOM	2298	C	GLN	A	291	-20.922	-20.016	-34.719	1.00	83.75	C
ATOM	2299	CB	GLN	A	291	-23.250	-19.797	-35.656	1.00	83.75	C
ATOM	2300	O	GLN	A	291	-20.781	-21.141	-34.250	1.00	83.75	O

ATOM	2301	CG	GLN	A	291	-24.672	-19.344	-35.406	1.00	83.75	C
ATOM	2302	CD	GLN	A	291	-25.641	-19.859	-36.469	1.00	83.75	C
ATOM	2303	NE2	GLN	A	291	-26.938	-19.797	-36.156	1.00	83.75	N
ATOM	2304	OE1	GLN	A	291	-25.219	-20.312	-37.531	1.00	83.75	O
ATOM	2305	N	ARG	A	292	-19.906	-19.391	-35.188	1.00	82.56	N
ATOM	2306	CA	ARG	A	292	-18.656	-20.062	-35.531	1.00	82.56	C
ATOM	2307	C	ARG	A	292	-17.656	-19.969	-34.375	1.00	82.56	C
ATOM	2308	CB	ARG	A	292	-18.062	-19.469	-36.781	1.00	82.56	C
ATOM	2309	O	ARG	A	292	-17.250	-21.000	-33.812	1.00	82.56	O
ATOM	2310	CG	ARG	A	292	-16.875	-20.250	-37.344	1.00	82.56	C
ATOM	2311	CD	ARG	A	292	-16.594	-19.891	-38.781	1.00	82.56	C
ATOM	2312	NE	ARG	A	292	-15.523	-20.719	-39.344	1.00	82.56	N
ATOM	2313	NH1	ARG	A	292	-16.625	-21.188	-41.312	1.00	82.56	N
ATOM	2314	NH2	ARG	A	292	-14.539	-22.047	-40.938	1.00	82.56	N
ATOM	2315	CZ	ARG	A	292	-15.562	-21.312	-40.531	1.00	82.56	C
ATOM	2316	N	TYR	A	293	-17.469	-18.797	-34.031	1.00	84.94	N
ATOM	2317	CA	TYR	A	293	-16.453	-18.672	-32.969	1.00	84.94	C
ATOM	2318	C	TYR	A	293	-17.047	-18.078	-31.703	1.00	84.94	C
ATOM	2319	CB	TYR	A	293	-15.289	-17.812	-33.469	1.00	84.94	C
ATOM	2320	O	TYR	A	293	-16.375	-18.000	-30.672	1.00	84.94	O
ATOM	2321	CG	TYR	A	293	-15.719	-16.484	-34.062	1.00	84.94	C
ATOM	2322	CD1	TYR	A	293	-15.781	-16.297	-35.438	1.00	84.94	C
ATOM	2323	CD2	TYR	A	293	-16.062	-15.422	-33.219	1.00	84.94	C
ATOM	2324	CE1	TYR	A	293	-16.172	-15.078	-35.969	1.00	84.94	C
ATOM	2325	CE2	TYR	A	293	-16.453	-14.195	-33.750	1.00	84.94	C
ATOM	2326	OH	TYR	A	293	-16.906	-12.828	-35.656	1.00	84.94	O
ATOM	2327	CZ	TYR	A	293	-16.516	-14.031	-35.125	1.00	84.94	C
ATOM	2328	N	GLY	A	294	-18.250	-17.625	-31.781	1.00	81.69	N
ATOM	2329	CA	GLY	A	294	-18.922	-17.016	-30.641	1.00	81.69	C
ATOM	2330	C	GLY	A	294	-19.859	-17.953	-29.906	1.00	81.69	C
ATOM	2331	O	GLY	A	294	-19.703	-19.172	-29.984	1.00	81.69	O
ATOM	2332	N	LEU	A	295	-20.875	-17.438	-29.188	1.00	82.12	N
ATOM	2333	CA	LEU	A	295	-21.734	-18.203	-28.281	1.00	82.12	C
ATOM	2334	C	LEU	A	295	-22.953	-18.734	-29.031	1.00	82.12	C
ATOM	2335	CB	LEU	A	295	-22.172	-17.328	-27.109	1.00	82.12	C
ATOM	2336	O	LEU	A	295	-23.625	-19.656	-28.547	1.00	82.12	O
ATOM	2337	CG	LEU	A	295	-21.078	-16.891	-26.141	1.00	82.12	C
ATOM	2338	CD1	LEU	A	295	-21.609	-15.836	-25.172	1.00	82.12	C
ATOM	2339	CD2	LEU	A	295	-20.531	-18.094	-25.375	1.00	82.12	C
ATOM	2340	N	ASP	A	296	-23.172	-18.297	-30.266	1.00	75.44	N
ATOM	2341	CA	ASP	A	296	-24.312	-18.719	-31.062	1.00	75.44	C
ATOM	2342	C	ASP	A	296	-25.625	-18.578	-30.281	1.00	75.44	C
ATOM	2343	CB	ASP	A	296	-24.141	-20.172	-31.531	1.00	75.44	C
ATOM	2344	O	ASP	A	296	-26.453	-19.500	-30.281	1.00	75.44	O
ATOM	2345	CG	ASP	A	296	-25.094	-20.562	-32.656	1.00	75.44	C
ATOM	2346	OD1	ASP	A	296	-25.672	-19.672	-33.281	1.00	75.44	O
ATOM	2347	OD2	ASP	A	296	-25.266	-21.781	-32.875	1.00	75.44	O
ATOM	2348	N	GLN	A	297	-25.797	-17.453	-29.453	1.00	64.44	N
ATOM	2349	CA	GLN	A	297	-26.906	-17.328	-28.516	1.00	64.44	C
ATOM	2350	C	GLN	A	297	-28.234	-17.234	-29.266	1.00	64.44	C
ATOM	2351	CB	GLN	A	297	-26.734	-16.094	-27.625	1.00	64.44	C
ATOM	2352	O	GLN	A	297	-29.281	-17.625	-28.734	1.00	64.44	O
ATOM	2353	CG	GLN	A	297	-25.750	-16.312	-26.469	1.00	64.44	C
ATOM	2354	CD	GLN	A	297	-25.938	-15.320	-25.344	1.00	64.44	C
ATOM	2355	NE2	GLN	A	297	-25.359	-15.625	-24.188	1.00	64.44	N
ATOM	2356	OE1	GLN	A	297	-26.594	-14.289	-25.516	1.00	64.44	O
ATOM	2357	N	ARG	A	298	-28.328	-16.688	-30.562	1.00	55.84	N
ATOM	2358	CA	ARG	A	298	-29.609	-16.656	-31.250	1.00	55.84	C
ATOM	2359	C	ARG	A	298	-30.188	-18.062	-31.406	1.00	55.84	C
ATOM	2360	CB	ARG	A	298	-29.469	-16.000	-32.625	1.00	55.84	C
ATOM	2361	O	ARG	A	298	-31.391	-18.266	-31.219	1.00	55.84	O
ATOM	2362	CG	ARG	A	298	-30.734	-15.336	-33.125	1.00	55.84	C
ATOM	2363	CD	ARG	A	298	-30.547	-14.711	-34.500	1.00	55.84	C
ATOM	2364	NE	ARG	A	298	-31.797	-14.148	-35.031	1.00	55.84	N

ATOM	2365	NH1	ARG	A	298	-30.922	-13.602	-37.094	1.00	55.84	N
ATOM	2366	NH2	ARG	A	298	-33.125	-13.156	-36.625	1.00	55.84	N
ATOM	2367	CZ	ARG	A	298	-31.938	-13.633	-36.250	1.00	55.84	C
ATOM	2368	N	GLU	A	299	-29.359	-19.094	-31.516	1.00	53.12	N
ATOM	2369	CA	GLU	A	299	-29.828	-20.469	-31.641	1.00	53.12	C
ATOM	2370	C	GLU	A	299	-30.219	-21.047	-30.281	1.00	53.12	C
ATOM	2371	CB	GLU	A	299	-28.766	-21.344	-32.281	1.00	53.12	C
ATOM	2372	O	GLU	A	299	-31.078	-21.922	-30.188	1.00	53.12	O
ATOM	2373	CG	GLU	A	299	-28.641	-21.141	-33.812	1.00	53.12	C
ATOM	2374	CD	GLU	A	299	-29.812	-21.703	-34.594	1.00	53.12	C
ATOM	2375	OE1	GLU	A	299	-30.047	-21.266	-35.719	1.00	53.12	O
ATOM	2376	OE2	GLU	A	299	-30.500	-22.594	-34.031	1.00	53.12	O
ATOM	2377	N	LEU	A	300	-29.703	-20.375	-29.109	1.00	56.88	N
ATOM	2378	CA	LEU	A	300	-30.047	-20.875	-27.797	1.00	56.88	C
ATOM	2379	C	LEU	A	300	-31.312	-20.188	-27.281	1.00	56.88	C
ATOM	2380	CB	LEU	A	300	-28.906	-20.641	-26.812	1.00	56.88	C
ATOM	2381	O	LEU	A	300	-31.797	-20.500	-26.188	1.00	56.88	O
ATOM	2382	CG	LEU	A	300	-27.656	-21.500	-27.016	1.00	56.88	C
ATOM	2383	CD1	LEU	A	300	-26.469	-20.938	-26.234	1.00	56.88	C
ATOM	2384	CD2	LEU	A	300	-27.922	-22.953	-26.609	1.00	56.88	C
ATOM	2385	N	GLY	A	301	-31.922	-19.219	-28.078	1.00	59.75	N
ATOM	2386	CA	GLY	A	301	-33.188	-18.594	-27.734	1.00	59.75	C
ATOM	2387	C	GLY	A	301	-33.031	-17.484	-26.703	1.00	59.75	C
ATOM	2388	O	GLY	A	301	-33.969	-17.156	-25.984	1.00	59.75	O
ATOM	2389	N	ILE	A	302	-31.812	-17.047	-26.406	1.00	70.12	N
ATOM	2390	CA	ILE	A	302	-31.641	-15.953	-25.438	1.00	70.12	C
ATOM	2391	C	ILE	A	302	-31.625	-14.617	-26.172	1.00	70.12	C
ATOM	2392	CB	ILE	A	302	-30.344	-16.125	-24.609	1.00	70.12	C
ATOM	2393	O	ILE	A	302	-30.766	-14.375	-27.016	1.00	70.12	O
ATOM	2394	CG1	ILE	A	302	-30.359	-17.484	-23.891	1.00	70.12	C
ATOM	2395	CG2	ILE	A	302	-30.172	-14.977	-23.625	1.00	70.12	C
ATOM	2396	CD1	ILE	A	302	-29.047	-17.812	-23.172	1.00	70.12	C
ATOM	2397	N	ALA	A	303	-32.656	-13.812	-26.078	1.00	81.25	N
ATOM	2398	CA	ALA	A	303	-32.812	-12.516	-26.734	1.00	81.25	C
ATOM	2399	C	ALA	A	303	-31.812	-11.492	-26.203	1.00	81.25	C
ATOM	2400	CB	ALA	A	303	-34.250	-12.008	-26.547	1.00	81.25	C
ATOM	2401	O	ALA	A	303	-31.500	-11.492	-25.016	1.00	81.25	O
ATOM	2402	N	LEU	A	304	-31.047	-10.883	-27.156	1.00	86.25	N
ATOM	2403	CA	LEU	A	304	-30.109	-9.797	-26.859	1.00	86.25	C
ATOM	2404	C	LEU	A	304	-30.781	-8.445	-27.094	1.00	86.25	C
ATOM	2405	CB	LEU	A	304	-28.859	-9.914	-27.719	1.00	86.25	C
ATOM	2406	O	LEU	A	304	-31.438	-8.234	-28.109	1.00	86.25	O
ATOM	2407	CG	LEU	A	304	-27.781	-8.852	-27.500	1.00	86.25	C
ATOM	2408	CD1	LEU	A	304	-27.156	-9.008	-26.109	1.00	86.25	C
ATOM	2409	CD2	LEU	A	304	-26.703	-8.945	-28.578	1.00	86.25	C
ATOM	2410	N	ASP	A	305	-30.719	-7.555	-26.125	1.00	90.00	N
ATOM	2411	CA	ASP	A	305	-31.203	-6.191	-26.250	1.00	90.00	C
ATOM	2412	C	ASP	A	305	-30.312	-5.367	-27.188	1.00	90.00	C
ATOM	2413	CB	ASP	A	305	-31.312	-5.516	-24.891	1.00	90.00	C
ATOM	2414	O	ASP	A	305	-29.281	-4.840	-26.750	1.00	90.00	O
ATOM	2415	CG	ASP	A	305	-31.891	-4.113	-24.953	1.00	90.00	C
ATOM	2416	OD1	ASP	A	305	-32.188	-3.635	-26.062	1.00	90.00	O
ATOM	2417	OD2	ASP	A	305	-32.031	-3.484	-23.875	1.00	90.00	O
ATOM	2418	N	LYS	A	306	-30.656	-5.293	-28.438	1.00	90.56	N
ATOM	2419	CA	LYS	A	306	-29.812	-4.645	-29.453	1.00	90.56	C
ATOM	2420	C	LYS	A	306	-29.797	-3.131	-29.250	1.00	90.56	C
ATOM	2421	CB	LYS	A	306	-30.312	-4.980	-30.859	1.00	90.56	C
ATOM	2422	O	LYS	A	306	-28.828	-2.461	-29.625	1.00	90.56	O
ATOM	2423	CG	LYS	A	306	-31.750	-4.559	-31.125	1.00	90.56	C
ATOM	2424	CD	LYS	A	306	-32.219	-4.984	-32.500	1.00	90.56	C
ATOM	2425	CE	LYS	A	306	-33.656	-4.609	-32.750	1.00	90.56	C
ATOM	2426	NZ	LYS	A	306	-34.125	-5.086	-34.094	1.00	90.56	N
ATOM	2427	N	TRP	A	307	-30.828	-2.621	-28.656	1.00	91.50	N
ATOM	2428	CA	TRP	A	307	-30.891	-1.179	-28.438	1.00	91.50	C

ATOM	2429	C	TRP	A	307	-29.891	-0.728	-27.391	1.00	91.50	C
ATOM	2430	CB	TRP	A	307	-32.312	-0.766	-28.016	1.00	91.50	C
ATOM	2431	O	TRP	A	307	-29.312	0.360	-27.500	1.00	91.50	O
ATOM	2432	CG	TRP	A	307	-33.375	-1.155	-29.000	1.00	91.50	C
ATOM	2433	CD1	TRP	A	307	-34.312	-2.127	-28.828	1.00	91.50	C
ATOM	2434	CD2	TRP	A	307	-33.594	-0.583	-30.281	1.00	91.50	C
ATOM	2435	CE2	TRP	A	307	-34.719	-1.257	-30.859	1.00	91.50	C
ATOM	2436	CE3	TRP	A	307	-32.969	0.436	-31.016	1.00	91.50	C
ATOM	2437	NE1	TRP	A	307	-35.125	-2.193	-29.953	1.00	91.50	N
ATOM	2438	CH2	TRP	A	307	-34.562	0.057	-32.812	1.00	91.50	C
ATOM	2439	CZ2	TRP	A	307	-35.188	-0.944	-32.125	1.00	91.50	C
ATOM	2440	CZ3	TRP	A	307	-33.469	0.746	-32.281	1.00	91.50	C
ATOM	2441	N	SER	A	308	-29.688	-1.555	-26.359	1.00	90.88	N
ATOM	2442	CA	SER	A	308	-28.656	-1.248	-25.375	1.00	90.88	C
ATOM	2443	C	SER	A	308	-27.266	-1.308	-25.984	1.00	90.88	C
ATOM	2444	CB	SER	A	308	-28.750	-2.215	-24.188	1.00	90.88	C
ATOM	2445	O	SER	A	308	-26.391	-0.516	-25.625	1.00	90.88	O
ATOM	2446	OG	SER	A	308	-28.359	-3.520	-24.578	1.00	90.88	O
ATOM	2447	N	ILE	A	309	-27.016	-2.215	-26.938	1.00	92.81	N
ATOM	2448	CA	ILE	A	309	-25.750	-2.320	-27.641	1.00	92.81	C
ATOM	2449	C	ILE	A	309	-25.500	-1.058	-28.469	1.00	92.81	C
ATOM	2450	CB	ILE	A	309	-25.703	-3.568	-28.547	1.00	92.81	C
ATOM	2451	O	ILE	A	309	-24.391	-0.519	-28.484	1.00	92.81	O
ATOM	2452	CG1	ILE	A	309	-25.797	-4.848	-27.703	1.00	92.81	C
ATOM	2453	CG2	ILE	A	309	-24.453	-3.566	-29.422	1.00	92.81	C
ATOM	2454	CD1	ILE	A	309	-24.578	-5.082	-26.812	1.00	92.81	C
ATOM	2455	N	TYR	A	310	-26.562	-0.611	-29.141	1.00	94.94	N
ATOM	2456	CA	TYR	A	310	-26.500	0.588	-29.969	1.00	94.94	C
ATOM	2457	C	TYR	A	310	-26.125	1.811	-29.125	1.00	94.94	C
ATOM	2458	CB	TYR	A	310	-27.844	0.820	-30.672	1.00	94.94	C
ATOM	2459	O	TYR	A	310	-25.266	2.594	-29.516	1.00	94.94	O
ATOM	2460	CG	TYR	A	310	-27.844	2.029	-31.578	1.00	94.94	C
ATOM	2461	CD1	TYR	A	310	-28.703	3.104	-31.312	1.00	94.94	C
ATOM	2462	CD2	TYR	A	310	-27.016	2.102	-32.688	1.00	94.94	C
ATOM	2463	CE1	TYR	A	310	-28.719	4.223	-32.156	1.00	94.94	C
ATOM	2464	CE2	TYR	A	310	-27.016	3.213	-33.500	1.00	94.94	C
ATOM	2465	OH	TYR	A	310	-27.875	5.371	-34.062	1.00	94.94	O
ATOM	2466	CZ	TYR	A	310	-27.875	4.266	-33.250	1.00	94.94	C
ATOM	2467	N	GLU	A	311	-26.750	1.993	-27.969	1.00	93.25	N
ATOM	2468	CA	GLU	A	311	-26.469	3.107	-27.078	1.00	93.25	C
ATOM	2469	C	GLU	A	311	-25.016	3.057	-26.578	1.00	93.25	C
ATOM	2470	CB	GLU	A	311	-27.422	3.107	-25.875	1.00	93.25	C
ATOM	2471	O	GLU	A	311	-24.344	4.086	-26.516	1.00	93.25	O
ATOM	2472	CG	GLU	A	311	-27.281	4.324	-24.984	1.00	93.25	C
ATOM	2473	CD	GLU	A	311	-28.250	4.316	-23.812	1.00	93.25	C
ATOM	2474	OE1	GLU	A	311	-28.344	5.340	-23.094	1.00	93.25	O
ATOM	2475	OE2	GLU	A	311	-28.906	3.273	-23.594	1.00	93.25	O
ATOM	2476	N	CYS	A	312	-24.594	1.885	-26.250	1.00	94.75	N
ATOM	2477	CA	CYS	A	312	-23.203	1.684	-25.828	1.00	94.75	C
ATOM	2478	C	CYS	A	312	-22.234	2.037	-26.938	1.00	94.75	C
ATOM	2479	CB	CYS	A	312	-22.984	0.236	-25.375	1.00	94.75	C
ATOM	2480	O	CYS	A	312	-21.219	2.695	-26.703	1.00	94.75	O
ATOM	2481	SG	CYS	A	312	-21.312	-0.104	-24.812	1.00	94.75	S
ATOM	2482	N	ALA	A	313	-22.609	1.643	-28.156	1.00	95.75	N
ATOM	2483	CA	ALA	A	313	-21.750	1.922	-29.312	1.00	95.75	C
ATOM	2484	C	ALA	A	313	-21.609	3.426	-29.531	1.00	95.75	C
ATOM	2485	CB	ALA	A	313	-22.328	1.264	-30.562	1.00	95.75	C
ATOM	2486	O	ALA	A	313	-20.500	3.912	-29.781	1.00	95.75	O
ATOM	2487	N	GLN	A	314	-22.625	4.141	-29.406	1.00	95.06	N
ATOM	2488	CA	GLN	A	314	-22.562	5.590	-29.562	1.00	95.06	C
ATOM	2489	C	GLN	A	314	-21.688	6.219	-28.484	1.00	95.06	C
ATOM	2490	CB	GLN	A	314	-23.969	6.195	-29.500	1.00	95.06	C
ATOM	2491	O	GLN	A	314	-20.906	7.129	-28.766	1.00	95.06	O
ATOM	2492	CG	GLN	A	314	-24.828	5.883	-30.719	1.00	95.06	C

ATOM	2493	CD	GLN	A	314	-26.219	6.473	-30.625	1.00	95.06	C
ATOM	2494	NE2	GLN	A	314	-26.531	7.395	-31.516	1.00	95.06	N
ATOM	2495	OE1	GLN	A	314	-27.016	6.102	-29.750	1.00	95.06	O
ATOM	2496	N	TYR	A	315	-21.828	5.676	-27.297	1.00	95.06	N
ATOM	2497	CA	TYR	A	315	-21.016	6.172	-26.188	1.00	95.06	C
ATOM	2498	C	TYR	A	315	-19.531	5.898	-26.438	1.00	95.06	C
ATOM	2499	CB	TYR	A	315	-21.453	5.523	-24.875	1.00	95.06	C
ATOM	2500	O	TYR	A	315	-18.688	6.770	-26.203	1.00	95.06	O
ATOM	2501	CG	TYR	A	315	-20.797	6.117	-23.656	1.00	95.06	C
ATOM	2502	CD1	TYR	A	315	-19.938	5.355	-22.875	1.00	95.06	C
ATOM	2503	CD2	TYR	A	315	-21.016	7.441	-23.297	1.00	95.06	C
ATOM	2504	CE1	TYR	A	315	-19.328	5.898	-21.734	1.00	95.06	C
ATOM	2505	CE2	TYR	A	315	-20.406	7.992	-22.172	1.00	95.06	C
ATOM	2506	OH	TYR	A	315	-18.969	7.758	-20.297	1.00	95.06	O
ATOM	2507	CZ	TYR	A	315	-19.562	7.215	-21.406	1.00	95.06	C
ATOM	2508	N	CYS	A	316	-19.172	4.727	-26.844	1.00	95.62	N
ATOM	2509	CA	CYS	A	316	-17.797	4.348	-27.141	1.00	95.62	C
ATOM	2510	C	CYS	A	316	-17.203	5.219	-28.250	1.00	95.62	C
ATOM	2511	CB	CYS	A	316	-17.719	2.875	-27.547	1.00	95.62	C
ATOM	2512	O	CYS	A	316	-16.016	5.535	-28.234	1.00	95.62	O
ATOM	2513	SG	CYS	A	316	-18.062	1.730	-26.188	1.00	95.62	S
ATOM	2514	N	ASP	A	317	-18.016	5.754	-29.156	1.00	96.69	N
ATOM	2515	CA	ASP	A	317	-17.562	6.500	-30.328	1.00	96.69	C
ATOM	2516	C	ASP	A	317	-17.547	8.000	-30.047	1.00	96.69	C
ATOM	2517	CB	ASP	A	317	-18.438	6.195	-31.547	1.00	96.69	C
ATOM	2518	O	ASP	A	317	-17.156	8.789	-30.906	1.00	96.69	O
ATOM	2519	CG	ASP	A	317	-18.047	4.906	-32.250	1.00	96.69	C
ATOM	2520	OD1	ASP	A	317	-16.875	4.465	-32.125	1.00	96.69	O
ATOM	2521	OD2	ASP	A	317	-18.906	4.324	-32.938	1.00	96.69	O
ATOM	2522	N	GLN	A	318	-17.891	8.422	-28.938	1.00	95.12	N
ATOM	2523	CA	GLN	A	318	-17.797	9.836	-28.594	1.00	95.12	C
ATOM	2524	C	GLN	A	318	-16.344	10.305	-28.547	1.00	95.12	C
ATOM	2525	CB	GLN	A	318	-18.500	10.125	-27.266	1.00	95.12	C
ATOM	2526	O	GLN	A	318	-15.508	9.648	-27.922	1.00	95.12	O
ATOM	2527	CG	GLN	A	318	-20.016	10.055	-27.328	1.00	95.12	C
ATOM	2528	CD	GLN	A	318	-20.672	10.305	-25.984	1.00	95.12	C
ATOM	2529	NE2	GLN	A	318	-21.984	10.508	-26.000	1.00	95.12	N
ATOM	2530	OE1	GLN	A	318	-20.000	10.320	-24.953	1.00	95.12	O
ATOM	2531	N	MET	A	319	-16.078	11.438	-29.281	1.00	94.56	N
ATOM	2532	CA	MET	A	319	-14.711	11.945	-29.328	1.00	94.56	C
ATOM	2533	C	MET	A	319	-14.336	12.609	-28.000	1.00	94.56	C
ATOM	2534	CB	MET	A	319	-14.547	12.945	-30.469	1.00	94.56	C
ATOM	2535	O	MET	A	319	-15.047	13.492	-27.516	1.00	94.56	O
ATOM	2536	CG	MET	A	319	-14.789	12.344	-31.844	1.00	94.56	C
ATOM	2537	SD	MET	A	319	-13.672	10.938	-32.188	1.00	94.56	S
ATOM	2538	CE	MET	A	319	-12.203	11.836	-32.781	1.00	94.56	C
ATOM	2539	N	VAL	A	320	-13.234	12.094	-27.375	1.00	95.06	N
ATOM	2540	CA	VAL	A	320	-12.703	12.648	-26.141	1.00	95.06	C
ATOM	2541	C	VAL	A	320	-11.250	13.086	-26.344	1.00	95.06	C
ATOM	2542	CB	VAL	A	320	-12.781	11.633	-24.984	1.00	95.06	C
ATOM	2543	O	VAL	A	320	-10.586	12.633	-27.281	1.00	95.06	O
ATOM	2544	CG1	VAL	A	320	-14.242	11.273	-24.672	1.00	95.06	C
ATOM	2545	CG2	VAL	A	320	-11.984	10.375	-25.312	1.00	95.06	C
ATOM	2546	N	PRO	A	321	-10.789	14.062	-25.562	1.00	94.44	N
ATOM	2547	CA	PRO	A	321	-9.414	14.555	-25.719	1.00	94.44	C
ATOM	2548	C	PRO	A	321	-8.375	13.453	-25.547	1.00	94.44	C
ATOM	2549	CB	PRO	A	321	-9.289	15.602	-24.609	1.00	94.44	C
ATOM	2550	O	PRO	A	321	-8.492	12.617	-24.641	1.00	94.44	O
ATOM	2551	CG	PRO	A	321	-10.688	16.062	-24.359	1.00	94.44	C
ATOM	2552	CD	PRO	A	321	-11.617	14.906	-24.562	1.00	94.44	C
ATOM	2553	N	ASP	A	322	-7.352	13.344	-26.484	1.00	91.56	N
ATOM	2554	CA	ASP	A	322	-6.328	12.305	-26.453	1.00	91.56	C
ATOM	2555	C	ASP	A	322	-5.176	12.695	-25.531	1.00	91.56	C
ATOM	2556	CB	ASP	A	322	-5.805	12.031	-27.859	1.00	91.56	C

ATOM	2557	O	ASP	A	322	-4.207	11.945	-25.375	1.00	91.56	O
ATOM	2558	CG	ASP	A	322	-5.102	13.227	-28.484	1.00	91.56	C
ATOM	2559	OD1	ASP	A	322	-4.992	14.281	-27.812	1.00	91.56	O
ATOM	2560	OD2	ASP	A	322	-4.656	13.117	-29.641	1.00	91.56	O
ATOM	2561	N	GLY	A	323	-5.184	13.891	-24.859	1.00	85.75	N
ATOM	2562	CA	GLY	A	323	-4.129	14.344	-23.969	1.00	85.75	C
ATOM	2563	C	GLY	A	323	-2.957	14.977	-24.688	1.00	85.75	C
ATOM	2564	O	GLY	A	323	-2.000	15.430	-24.062	1.00	85.75	O
ATOM	2565	N	LYS	A	324	-2.988	14.992	-26.047	1.00	87.44	N
ATOM	2566	CA	LYS	A	324	-1.919	15.555	-26.859	1.00	87.44	C
ATOM	2567	C	LYS	A	324	-2.432	16.703	-27.719	1.00	87.44	C
ATOM	2568	CB	LYS	A	324	-1.298	14.477	-27.750	1.00	87.44	C
ATOM	2569	O	LYS	A	324	-1.812	17.062	-28.719	1.00	87.44	O
ATOM	2570	CG	LYS	A	324	-0.692	13.312	-26.984	1.00	87.44	C
ATOM	2571	CD	LYS	A	324	-0.180	12.227	-27.922	1.00	87.44	C
ATOM	2572	CE	LYS	A	324	0.335	11.016	-27.156	1.00	87.44	C
ATOM	2573	NZ	LYS	A	324	0.763	9.922	-28.062	1.00	87.44	N
ATOM	2574	N	GLY	A	325	-3.617	17.250	-27.375	1.00	85.00	N
ATOM	2575	CA	GLY	A	325	-4.164	18.375	-28.109	1.00	85.00	C
ATOM	2576	C	GLY	A	325	-5.152	17.953	-29.188	1.00	85.00	C
ATOM	2577	O	GLY	A	325	-5.738	18.812	-29.859	1.00	85.00	O
ATOM	2578	N	GLY	A	326	-5.523	16.734	-29.422	1.00	93.75	N
ATOM	2579	CA	GLY	A	326	-6.508	16.188	-30.344	1.00	93.75	C
ATOM	2580	C	GLY	A	326	-7.598	15.398	-29.641	1.00	93.75	C
ATOM	2581	O	GLY	A	326	-7.738	15.469	-28.422	1.00	93.75	O
ATOM	2582	N	THR	A	327	-8.484	14.750	-30.484	1.00	94.81	N
ATOM	2583	CA	THR	A	327	-9.555	13.922	-29.938	1.00	94.81	C
ATOM	2584	C	THR	A	327	-9.430	12.484	-30.438	1.00	94.81	C
ATOM	2585	CB	THR	A	327	-10.945	14.477	-30.328	1.00	94.81	C
ATOM	2586	O	THR	A	327	-8.711	12.211	-31.406	1.00	94.81	O
ATOM	2587	CG2	THR	A	327	-11.133	15.891	-29.797	1.00	94.81	C
ATOM	2588	OG1	THR	A	327	-11.070	14.484	-31.750	1.00	94.81	O
ATOM	2589	N	GLU	A	328	-9.914	11.617	-29.703	1.00	94.69	N
ATOM	2590	CA	GLU	A	328	-9.984	10.195	-30.047	1.00	94.69	C
ATOM	2591	C	GLU	A	328	-11.289	9.578	-29.562	1.00	94.69	C
ATOM	2592	CB	GLU	A	328	-8.789	9.438	-29.453	1.00	94.69	C
ATOM	2593	O	GLU	A	328	-11.945	10.109	-28.672	1.00	94.69	O
ATOM	2594	CG	GLU	A	328	-8.781	9.414	-27.938	1.00	94.69	C
ATOM	2595	CD	GLU	A	328	-7.555	8.719	-27.359	1.00	94.69	C
ATOM	2596	OE1	GLU	A	328	-7.387	8.711	-26.125	1.00	94.69	O
ATOM	2597	OE2	GLU	A	328	-6.754	8.180	-28.156	1.00	94.69	O
ATOM	2598	N	PRO	A	329	-11.703	8.477	-30.234	1.00	95.62	N
ATOM	2599	CA	PRO	A	329	-12.867	7.781	-29.672	1.00	95.62	C
ATOM	2600	C	PRO	A	329	-12.633	7.320	-28.234	1.00	95.62	C
ATOM	2601	CB	PRO	A	329	-13.055	6.586	-30.609	1.00	95.62	C
ATOM	2602	O	PRO	A	329	-11.500	7.039	-27.844	1.00	95.62	O
ATOM	2603	CG	PRO	A	329	-12.305	6.953	-31.859	1.00	95.62	C
ATOM	2604	CD	PRO	A	329	-11.188	7.887	-31.484	1.00	95.62	C
ATOM	2605	N	ARG	A	330	-13.602	7.273	-27.500	1.00	95.38	N
ATOM	2606	CA	ARG	A	330	-13.508	7.020	-26.078	1.00	95.38	C
ATOM	2607	C	ARG	A	330	-12.930	5.637	-25.797	1.00	95.38	C
ATOM	2608	CB	ARG	A	330	-14.875	7.160	-25.406	1.00	95.38	C
ATOM	2609	O	ARG	A	330	-12.016	5.488	-24.984	1.00	95.38	O
ATOM	2610	CG	ARG	A	330	-14.828	7.125	-23.891	1.00	95.38	C
ATOM	2611	CD	ARG	A	330	-16.203	7.375	-23.266	1.00	95.38	C
ATOM	2612	NE	ARG	A	330	-16.641	8.758	-23.469	1.00	95.38	N
ATOM	2613	NH1	ARG	A	330	-17.484	9.016	-21.344	1.00	95.38	N
ATOM	2614	NH2	ARG	A	330	-17.594	10.742	-22.844	1.00	95.38	N
ATOM	2615	CZ	ARG	A	330	-17.234	9.500	-22.547	1.00	95.38	C
ATOM	2616	N	TYR	A	331	-13.617	4.551	-26.422	1.00	95.88	N
ATOM	2617	CA	TYR	A	331	-13.156	3.182	-26.203	1.00	95.88	C
ATOM	2618	C	TYR	A	331	-12.945	2.463	-27.531	1.00	95.88	C
ATOM	2619	CB	TYR	A	331	-14.164	2.406	-25.344	1.00	95.88	C
ATOM	2620	O	TYR	A	331	-13.758	2.592	-28.453	1.00	95.88	O

```
ATOM   2621  CG  TYR A 331     -14.336   2.975 -23.953  1.00 95.88           C
ATOM   2622  CD1 TYR A 331     -13.312   2.898 -23.016  1.00 95.88           C
ATOM   2623  CD2 TYR A 331     -15.531   3.588 -23.578  1.00 95.88           C
ATOM   2624  CE1 TYR A 331     -13.477   3.420 -21.734  1.00 95.88           C
ATOM   2625  CE2 TYR A 331     -15.703   4.109 -22.312  1.00 95.88           C
ATOM   2626  OH  TYR A 331     -14.836   4.539 -20.125  1.00 95.88           O
ATOM   2627  CZ  TYR A 331     -14.672   4.023 -21.391  1.00 95.88           C
ATOM   2628  N   LEU A 332     -11.852   1.692 -27.609  1.00 94.94           N
ATOM   2629  CA  LEU A 332     -11.586   0.785 -28.719  1.00 94.94           C
ATOM   2630  C   LEU A 332     -11.391  -0.643 -28.219  1.00 94.94           C
ATOM   2631  CB  LEU A 332     -10.344   1.238 -29.500  1.00 94.94           C
ATOM   2632  O   LEU A 332     -11.102  -0.860 -27.047  1.00 94.94           O
ATOM   2633  CG  LEU A 332     -10.414   2.629 -30.125  1.00 94.94           C
ATOM   2634  CD1 LEU A 332      -9.109   2.947 -30.844  1.00 94.94           C
ATOM   2635  CD2 LEU A 332     -11.594   2.721 -31.094  1.00 94.94           C
ATOM   2636  N   CYS A 333     -11.555  -1.603 -29.109  1.00 95.31           N
ATOM   2637  CA  CYS A 333     -11.305  -3.002 -28.781  1.00 95.31           C
ATOM   2638  C   CYS A 333     -10.367  -3.641 -29.797  1.00 95.31           C
ATOM   2639  CB  CYS A 333     -12.617  -3.781 -28.719  1.00 95.31           C
ATOM   2640  O   CYS A 333     -10.797  -4.020 -30.891  1.00 95.31           O
ATOM   2641  SG  CYS A 333     -12.414  -5.508 -28.234  1.00 95.31           S
ATOM   2642  N   ASP A 334      -9.125  -3.746 -29.406  1.00 94.12           N
ATOM   2643  CA  ASP A 334      -8.109  -4.406 -30.234  1.00 94.12           C
ATOM   2644  C   ASP A 334      -7.598  -5.672 -29.547  1.00 94.12           C
ATOM   2645  CB  ASP A 334      -6.949  -3.453 -30.516  1.00 94.12           C
ATOM   2646  O   ASP A 334      -6.559  -5.648 -28.875  1.00 94.12           O
ATOM   2647  CG  ASP A 334      -7.359  -2.250 -31.344  1.00 94.12           C
ATOM   2648  OD1 ASP A 334      -8.156  -2.408 -32.312  1.00 94.12           O
ATOM   2649  OD2 ASP A 334      -6.887  -1.133 -31.047  1.00 94.12           O
ATOM   2650  N   VAL A 335      -8.359  -6.703 -29.750  1.00 92.19           N
ATOM   2651  CA  VAL A 335      -8.039  -7.938 -29.047  1.00 92.19           C
ATOM   2652  C   VAL A 335      -7.809  -9.062 -30.047  1.00 92.19           C
ATOM   2653  CB  VAL A 335      -9.148  -8.328 -28.047  1.00 92.19           C
ATOM   2654  O   VAL A 335      -8.406  -9.070 -31.125  1.00 92.19           O
ATOM   2655  CG1 VAL A 335     -10.414  -8.758 -28.797  1.00 92.19           C
ATOM   2656  CG2 VAL A 335      -8.672  -9.445 -27.125  1.00 92.19           C
ATOM   2657  N   VAL A 336      -6.863  -9.953 -29.656  1.00 90.44           N
ATOM   2658  CA  VAL A 336      -6.664 -11.227 -30.344  1.00 90.44           C
ATOM   2659  C   VAL A 336      -6.945 -12.375 -29.391  1.00 90.44           C
ATOM   2660  CB  VAL A 336      -5.230 -11.344 -30.922  1.00 90.44           C
ATOM   2661  O   VAL A 336      -6.273 -12.516 -28.359  1.00 90.44           O
ATOM   2662  CG1 VAL A 336      -5.035 -12.688 -31.609  1.00 90.44           C
ATOM   2663  CG2 VAL A 336      -4.945 -10.195 -31.875  1.00 90.44           C
ATOM   2664  N   ILE A 337      -7.977 -13.102 -29.688  1.00 89.62           N
ATOM   2665  CA  ILE A 337      -8.336 -14.250 -28.859  1.00 89.62           C
ATOM   2666  C   ILE A 337      -7.723 -15.516 -29.438  1.00 89.62           C
ATOM   2667  CB  ILE A 337      -9.875 -14.398 -28.750  1.00 89.62           C
ATOM   2668  O   ILE A 337      -8.211 -16.047 -30.438  1.00 89.62           O
ATOM   2669  CG1 ILE A 337     -10.500 -13.102 -28.219  1.00 89.62           C
ATOM   2670  CG2 ILE A 337     -10.234 -15.586 -27.844  1.00 89.62           C
ATOM   2671  CD1 ILE A 337     -12.016 -13.055 -28.359  1.00 89.62           C
ATOM   2672  N   GLN A 338      -6.648 -16.078 -28.844  1.00 86.31           N
ATOM   2673  CA  GLN A 338      -5.828 -17.125 -29.422  1.00 86.31           C
ATOM   2674  C   GLN A 338      -6.129 -18.484 -28.766  1.00 86.31           C
ATOM   2675  CB  GLN A 338      -4.344 -16.797 -29.281  1.00 86.31           C
ATOM   2676  O   GLN A 338      -6.039 -19.516 -29.422  1.00 86.31           O
ATOM   2677  CG  GLN A 338      -3.881 -15.641 -30.156  1.00 86.31           C
ATOM   2678  CD  GLN A 338      -2.393 -15.375 -30.031  1.00 86.31           C
ATOM   2679  NE2 GLN A 338      -1.878 -14.500 -30.891  1.00 86.31           N
ATOM   2680  OE1 GLN A 338      -1.708 -15.953 -29.188  1.00 86.31           O
ATOM   2681  N   SER A 339      -6.496 -18.328 -27.422  1.00 87.19           N
ATOM   2682  CA  SER A 339      -6.738 -19.578 -26.688  1.00 87.19           C
ATOM   2683  C   SER A 339      -8.195 -19.688 -26.266  1.00 87.19           C
ATOM   2684  CB  SER A 339      -5.832 -19.656 -25.453  1.00 87.19           C
```

ATOM	2685	O	SER	A	339	-8.898	-18.688	-26.141	1.00	87.19	O
ATOM	2686	OG	SER	A	339	-5.949	-18.484	-24.672	1.00	87.19	O
ATOM	2687	N	GLN	A	340	-8.672	-20.922	-26.125	1.00	83.94	N
ATOM	2688	CA	GLN	A	340	-10.031	-21.203	-25.672	1.00	83.94	C
ATOM	2689	C	GLN	A	340	-10.273	-20.656	-24.266	1.00	83.94	C
ATOM	2690	CB	GLN	A	340	-10.320	-22.703	-25.703	1.00	83.94	C
ATOM	2691	O	GLN	A	340	-9.477	-20.891	-23.359	1.00	83.94	O
ATOM	2692	CG	GLN	A	340	-11.773	-23.062	-25.438	1.00	83.94	C
ATOM	2693	CD	GLN	A	340	-12.039	-24.547	-25.547	1.00	83.94	C
ATOM	2694	NE2	GLN	A	340	-13.312	-24.922	-25.609	1.00	83.94	N
ATOM	2695	OE1	GLN	A	340	-11.109	-25.359	-25.578	1.00	83.94	O
ATOM	2696	N	VAL	A	341	-11.273	-19.672	-24.188	1.00	85.94	N
ATOM	2697	CA	VAL	A	341	-11.656	-19.094	-22.891	1.00	85.94	C
ATOM	2698	C	VAL	A	341	-13.148	-19.297	-22.656	1.00	85.94	C
ATOM	2699	CB	VAL	A	341	-11.305	-17.594	-22.828	1.00	85.94	C
ATOM	2700	O	VAL	A	341	-13.922	-19.438	-23.609	1.00	85.94	O
ATOM	2701	CG1	VAL	A	341	-10.836	-17.203	-21.438	1.00	85.94	C
ATOM	2702	CG2	VAL	A	341	-10.242	-17.250	-23.875	1.00	85.94	C
ATOM	2703	N	GLU	A	342	-13.477	-19.453	-21.359	1.00	88.81	N
ATOM	2704	CA	GLU	A	342	-14.891	-19.531	-21.031	1.00	88.81	C
ATOM	2705	C	GLU	A	342	-15.672	-18.359	-21.609	1.00	88.81	C
ATOM	2706	CB	GLU	A	342	-15.086	-19.578	-19.500	1.00	88.81	C
ATOM	2707	O	GLU	A	342	-15.258	-17.203	-21.469	1.00	88.81	O
ATOM	2708	CG	GLU	A	342	-16.516	-19.859	-19.078	1.00	88.81	C
ATOM	2709	CD	GLU	A	342	-16.734	-19.703	-17.578	1.00	88.81	C
ATOM	2710	OE1	GLU	A	342	-17.891	-19.859	-17.109	1.00	88.81	O
ATOM	2711	OE2	GLU	A	342	-15.742	-19.406	-16.859	1.00	88.81	O
ATOM	2712	N	ALA	A	343	-16.734	-18.688	-22.250	1.00	82.06	N
ATOM	2713	CA	ALA	A	343	-17.500	-17.734	-23.031	1.00	82.06	C
ATOM	2714	C	ALA	A	343	-18.000	-16.578	-22.156	1.00	82.06	C
ATOM	2715	CB	ALA	A	343	-18.672	-18.422	-23.719	1.00	82.06	C
ATOM	2716	O	ALA	A	343	-17.875	-15.414	-22.531	1.00	82.06	O
ATOM	2717	N	TYR	A	344	-18.578	-16.891	-21.000	1.00	83.62	N
ATOM	2718	CA	TYR	A	344	-19.109	-15.852	-20.125	1.00	83.62	C
ATOM	2719	C	TYR	A	344	-18.016	-14.914	-19.641	1.00	83.62	C
ATOM	2720	CB	TYR	A	344	-19.828	-16.484	-18.922	1.00	83.62	C
ATOM	2721	O	TYR	A	344	-18.203	-13.703	-19.578	1.00	83.62	O
ATOM	2722	CG	TYR	A	344	-20.516	-15.477	-18.031	1.00	83.62	C
ATOM	2723	CD1	TYR	A	344	-20.000	-15.172	-16.766	1.00	83.62	C
ATOM	2724	CD2	TYR	A	344	-21.672	-14.828	-18.438	1.00	83.62	C
ATOM	2725	CE1	TYR	A	344	-20.625	-14.242	-15.945	1.00	83.62	C
ATOM	2726	CE2	TYR	A	344	-22.312	-13.906	-17.625	1.00	83.62	C
ATOM	2727	OH	TYR	A	344	-22.406	-12.703	-15.562	1.00	83.62	O
ATOM	2728	CZ	TYR	A	344	-21.781	-13.617	-16.375	1.00	83.62	C
ATOM	2729	N	GLN	A	345	-16.875	-15.430	-19.297	1.00	89.44	N
ATOM	2730	CA	GLN	A	345	-15.727	-14.617	-18.891	1.00	89.44	C
ATOM	2731	C	GLN	A	345	-15.289	-13.680	-20.016	1.00	89.44	C
ATOM	2732	CB	GLN	A	345	-14.562	-15.508	-18.453	1.00	89.44	C
ATOM	2733	O	GLN	A	345	-14.961	-12.516	-19.766	1.00	89.44	O
ATOM	2734	CG	GLN	A	345	-13.375	-14.734	-17.891	1.00	89.44	C
ATOM	2735	CD	GLN	A	345	-13.711	-13.984	-16.625	1.00	89.44	C
ATOM	2736	NE2	GLN	A	345	-13.031	-12.867	-16.391	1.00	89.44	N
ATOM	2737	OE1	GLN	A	345	-14.578	-14.406	-15.844	1.00	89.44	O
ATOM	2738	N	LEU	A	346	-15.219	-14.211	-21.250	1.00	90.06	N
ATOM	2739	CA	LEU	A	346	-14.844	-13.391	-22.406	1.00	90.06	C
ATOM	2740	C	LEU	A	346	-15.828	-12.242	-22.594	1.00	90.06	C
ATOM	2741	CB	LEU	A	346	-14.789	-14.250	-23.672	1.00	90.06	C
ATOM	2742	O	LEU	A	346	-15.422	-11.109	-22.844	1.00	90.06	O
ATOM	2743	CG	LEU	A	346	-14.375	-13.531	-24.953	1.00	90.06	C
ATOM	2744	CD1	LEU	A	346	-12.992	-12.891	-24.781	1.00	90.06	C
ATOM	2745	CD2	LEU	A	346	-14.383	-14.492	-26.141	1.00	90.06	C
ATOM	2746	N	VAL	A	347	-17.109	-12.531	-22.438	1.00	90.25	N
ATOM	2747	CA	VAL	A	347	-18.141	-11.516	-22.547	1.00	90.25	C
ATOM	2748	C	VAL	A	347	-17.938	-10.445	-21.484	1.00	90.25	C

```
ATOM   2749  CB  VAL A 347     -19.562 -12.117 -22.438  1.00 90.25           C
ATOM   2750  O   VAL A 347     -17.984  -9.250 -21.766  1.00 90.25           O
ATOM   2751  CG1 VAL A 347     -20.609 -11.023 -22.328  1.00 90.25           C
ATOM   2752  CG2 VAL A 347     -19.844 -13.023 -23.641  1.00 90.25           C
ATOM   2753  N   ARG A 348     -17.641 -10.844 -20.266  1.00 89.94           N
ATOM   2754  CA  ARG A 348     -17.406  -9.906 -19.172  1.00 89.94           C
ATOM   2755  C   ARG A 348     -16.156  -9.070 -19.422  1.00 89.94           C
ATOM   2756  CB  ARG A 348     -17.281 -10.656 -17.844  1.00 89.94           C
ATOM   2757  O   ARG A 348     -16.125  -7.871 -19.141  1.00 89.94           O
ATOM   2758  CG  ARG A 348     -18.609 -11.078 -17.234  1.00 89.94           C
ATOM   2759  CD  ARG A 348     -18.438 -11.633 -15.828  1.00 89.94           C
ATOM   2760  NE  ARG A 348     -19.719 -11.945 -15.211  1.00 89.94           N
ATOM   2761  NH1 ARG A 348     -18.812 -12.711 -13.227  1.00 89.94           N
ATOM   2762  NH2 ARG A 348     -21.094 -12.688 -13.531  1.00 89.94           N
ATOM   2763  CZ  ARG A 348     -19.875 -12.445 -13.992  1.00 89.94           C
ATOM   2764  N   ASP A 349     -15.141  -9.711 -20.000  1.00 92.50           N
ATOM   2765  CA  ASP A 349     -13.891  -9.023 -20.281  1.00 92.50           C
ATOM   2766  C   ASP A 349     -14.094  -7.926 -21.328  1.00 92.50           C
ATOM   2767  CB  ASP A 349     -12.820 -10.008 -20.766  1.00 92.50           C
ATOM   2768  O   ASP A 349     -13.570  -6.820 -21.188  1.00 92.50           O
ATOM   2769  CG  ASP A 349     -12.273 -10.867 -19.641  1.00 92.50           C
ATOM   2770  OD1 ASP A 349     -12.492 -10.547 -18.453  1.00 92.50           O
ATOM   2771  OD2 ASP A 349     -11.602 -11.883 -19.938  1.00 92.50           O
ATOM   2772  N   ILE A 350     -14.930  -8.250 -22.359  1.00 93.94           N
ATOM   2773  CA  ILE A 350     -15.188  -7.270 -23.406  1.00 93.94           C
ATOM   2774  C   ILE A 350     -16.031  -6.133 -22.844  1.00 93.94           C
ATOM   2775  CB  ILE A 350     -15.883  -7.914 -24.625  1.00 93.94           C
ATOM   2776  O   ILE A 350     -15.805  -4.965 -23.156  1.00 93.94           O
ATOM   2777  CG1 ILE A 350     -14.984  -8.984 -25.250  1.00 93.94           C
ATOM   2778  CG2 ILE A 350     -16.266  -6.844 -25.656  1.00 93.94           C
ATOM   2779  CD1 ILE A 350     -13.633  -8.453 -25.719  1.00 93.94           C
ATOM   2780  N   CYS A 351     -17.031  -6.484 -21.953  1.00 92.75           N
ATOM   2781  CA  CYS A 351     -17.875  -5.465 -21.344  1.00 92.75           C
ATOM   2782  C   CYS A 351     -17.047  -4.531 -20.453  1.00 92.75           C
ATOM   2783  CB  CYS A 351     -18.969  -6.117 -20.500  1.00 92.75           C
ATOM   2784  O   CYS A 351     -17.328  -3.334 -20.375  1.00 92.75           O
ATOM   2785  SG  CYS A 351     -20.328  -6.797 -21.500  1.00 92.75           S
ATOM   2786  N   SER A 352     -16.016  -5.012 -19.828  1.00 93.00           N
ATOM   2787  CA  SER A 352     -15.172  -4.227 -18.922  1.00 93.00           C
ATOM   2788  C   SER A 352     -14.430  -3.127 -19.672  1.00 93.00           C
ATOM   2789  CB  SER A 352     -14.172  -5.129 -18.203  1.00 93.00           C
ATOM   2790  O   SER A 352     -14.039  -2.119 -19.078  1.00 93.00           O
ATOM   2791  OG  SER A 352     -13.164  -5.574 -19.078  1.00 93.00           O
ATOM   2792  N   ILE A 353     -14.250  -3.268 -21.078  1.00 94.75           N
ATOM   2793  CA  ILE A 353     -13.555  -2.277 -21.891  1.00 94.75           C
ATOM   2794  C   ILE A 353     -14.289  -0.941 -21.812  1.00 94.75           C
ATOM   2795  CB  ILE A 353     -13.430  -2.742 -23.359  1.00 94.75           C
ATOM   2796  O   ILE A 353     -13.656   0.114 -21.703  1.00 94.75           O
ATOM   2797  CG1 ILE A 353     -12.477  -3.938 -23.469  1.00 94.75           C
ATOM   2798  CG2 ILE A 353     -12.969  -1.589 -24.250  1.00 94.75           C
ATOM   2799  CD1 ILE A 353     -12.445  -4.578 -24.844  1.00 94.75           C
ATOM   2800  N   PHE A 354     -15.609  -0.915 -21.812  1.00 92.44           N
ATOM   2801  CA  PHE A 354     -16.375   0.324 -21.797  1.00 92.44           C
ATOM   2802  C   PHE A 354     -17.062   0.531 -20.453  1.00 92.44           C
ATOM   2803  CB  PHE A 354     -17.422   0.317 -22.922  1.00 92.44           C
ATOM   2804  O   PHE A 354     -18.141   1.140 -20.375  1.00 92.44           O
ATOM   2805  CG  PHE A 354     -18.156  -0.993 -23.062  1.00 92.44           C
ATOM   2806  CD1 PHE A 354     -17.766  -1.911 -24.031  1.00 92.44           C
ATOM   2807  CD2 PHE A 354     -19.219  -1.305 -22.234  1.00 92.44           C
ATOM   2808  CE1 PHE A 354     -18.438  -3.125 -24.172  1.00 92.44           C
ATOM   2809  CE2 PHE A 354     -19.891  -2.516 -22.359  1.00 92.44           C
ATOM   2810  CZ  PHE A 354     -19.500  -3.422 -23.328  1.00 92.44           C
ATOM   2811  N   ARG A 355     -16.484  -0.020 -19.391  1.00 90.69           N
ATOM   2812  CA  ARG A 355     -16.984   0.125 -18.016  1.00 90.69           C
```

ATOM	2813	C	ARG	A	355	-18.391	-0.434	-17.891	1.00	90.69	C
ATOM	2814	CB	ARG	A	355	-16.953	1.594	-17.594	1.00	90.69	C
ATOM	2815	O	ARG	A	355	-19.250	0.164	-17.219	1.00	90.69	O
ATOM	2816	CG	ARG	A	355	-15.594	2.246	-17.703	1.00	90.69	C
ATOM	2817	CD	ARG	A	355	-15.672	3.760	-17.578	1.00	90.69	C
ATOM	2818	NE	ARG	A	355	-14.359	4.387	-17.688	1.00	90.69	N
ATOM	2819	NH1	ARG	A	355	-15.172	6.512	-18.078	1.00	90.69	N
ATOM	2820	NH2	ARG	A	355	-12.914	6.141	-18.016	1.00	90.69	N
ATOM	2821	CZ	ARG	A	355	-14.156	5.680	-17.922	1.00	90.69	C
ATOM	2822	N	GLY	A	356	-18.672	-1.513	-18.547	1.00	88.25	N
ATOM	2823	CA	GLY	A	356	-20.016	-2.061	-18.547	1.00	88.25	C
ATOM	2824	C	GLY	A	356	-20.094	-3.469	-17.984	1.00	88.25	C
ATOM	2825	O	GLY	A	356	-19.062	-4.051	-17.625	1.00	88.25	O
ATOM	2826	N	MET	A	357	-21.203	-3.844	-17.719	1.00	87.94	N
ATOM	2827	CA	MET	A	357	-21.516	-5.199	-17.266	1.00	87.94	C
ATOM	2828	C	MET	A	357	-22.688	-5.773	-18.047	1.00	87.94	C
ATOM	2829	CB	MET	A	357	-21.828	-5.207	-15.773	1.00	87.94	C
ATOM	2830	O	MET	A	357	-23.578	-5.035	-18.469	1.00	87.94	O
ATOM	2831	CG	MET	A	357	-23.078	-4.430	-15.398	1.00	87.94	C
ATOM	2832	SD	MET	A	357	-23.328	-4.355	-13.578	1.00	87.94	S
ATOM	2833	CE	MET	A	357	-22.781	-6.016	-13.109	1.00	87.94	C
ATOM	2834	N	SER	A	358	-22.531	-7.113	-18.359	1.00	86.12	N
ATOM	2835	CA	SER	A	358	-23.656	-7.820	-18.953	1.00	86.12	C
ATOM	2836	C	SER	A	358	-24.547	-8.438	-17.875	1.00	86.12	C
ATOM	2837	CB	SER	A	358	-23.156	-8.914	-19.906	1.00	86.12	C
ATOM	2838	O	SER	A	358	-24.078	-8.805	-16.797	1.00	86.12	O
ATOM	2839	OG	SER	A	358	-22.484	-9.938	-19.188	1.00	86.12	O
ATOM	2840	N	PHE	A	359	-25.828	-8.320	-18.000	1.00	82.88	N
ATOM	2841	CA	PHE	A	359	-26.781	-8.922	-17.062	1.00	82.88	C
ATOM	2842	C	PHE	A	359	-28.047	-9.367	-17.797	1.00	82.88	C
ATOM	2843	CB	PHE	A	359	-27.141	-7.945	-15.945	1.00	82.88	C
ATOM	2844	O	PHE	A	359	-28.297	-8.945	-18.922	1.00	82.88	O
ATOM	2845	CG	PHE	A	359	-28.016	-6.805	-16.391	1.00	82.88	C
ATOM	2846	CD1	PHE	A	359	-27.469	-5.707	-17.031	1.00	82.88	C
ATOM	2847	CD2	PHE	A	359	-29.375	-6.836	-16.156	1.00	82.88	C
ATOM	2848	CE1	PHE	A	359	-28.281	-4.648	-17.453	1.00	82.88	C
ATOM	2849	CE2	PHE	A	359	-30.203	-5.785	-16.562	1.00	82.88	C
ATOM	2850	CZ	PHE	A	359	-29.641	-4.691	-17.203	1.00	82.88	C
ATOM	2851	N	TRP	A	360	-28.781	-10.367	-17.125	1.00	80.94	N
ATOM	2852	CA	TRP	A	360	-30.094	-10.812	-17.609	1.00	80.94	C
ATOM	2853	C	TRP	A	360	-31.203	-9.969	-17.016	1.00	80.94	C
ATOM	2854	CB	TRP	A	360	-30.312	-12.289	-17.266	1.00	80.94	C
ATOM	2855	O	TRP	A	360	-31.391	-9.930	-15.789	1.00	80.94	O
ATOM	2856	CG	TRP	A	360	-31.578	-12.859	-17.844	1.00	80.94	C
ATOM	2857	CD1	TRP	A	360	-32.719	-13.148	-17.172	1.00	80.94	C
ATOM	2858	CD2	TRP	A	360	-31.812	-13.219	-19.219	1.00	80.94	C
ATOM	2859	CE2	TRP	A	360	-33.125	-13.711	-19.297	1.00	80.94	C
ATOM	2860	CE3	TRP	A	360	-31.031	-13.156	-20.375	1.00	80.94	C
ATOM	2861	NE1	TRP	A	360	-33.656	-13.664	-18.047	1.00	80.94	N
ATOM	2862	CH2	TRP	A	360	-32.906	-14.086	-21.625	1.00	80.94	C
ATOM	2863	CZ2	TRP	A	360	-33.688	-14.156	-20.500	1.00	80.94	C
ATOM	2864	CZ3	TRP	A	360	-31.578	-13.594	-21.578	1.00	80.94	C
ATOM	2865	N	ASN	A	361	-31.922	-9.188	-17.812	1.00	74.38	N
ATOM	2866	CA	ASN	A	361	-32.938	-8.273	-17.312	1.00	74.38	C
ATOM	2867	C	ASN	A	361	-34.312	-8.953	-17.188	1.00	74.38	C
ATOM	2868	CB	ASN	A	361	-33.062	-7.047	-18.234	1.00	74.38	C
ATOM	2869	O	ASN	A	361	-35.312	-8.312	-16.844	1.00	74.38	O
ATOM	2870	CG	ASN	A	361	-33.562	-7.391	-19.609	1.00	74.38	C
ATOM	2871	ND2	ASN	A	361	-33.531	-6.426	-20.516	1.00	74.38	N
ATOM	2872	OD1	ASN	A	361	-34.000	-8.516	-19.859	1.00	74.38	O
ATOM	2873	N	GLY	A	362	-34.344	-10.266	-17.312	1.00	75.44	N
ATOM	2874	CA	GLY	A	362	-35.562	-11.039	-17.234	1.00	75.44	C
ATOM	2875	C	GLY	A	362	-36.094	-11.469	-18.594	1.00	75.44	C
ATOM	2876	O	GLY	A	362	-36.750	-12.508	-18.703	1.00	75.44	O

ATOM	2877	N	GLU	A	363	-35.750	-10.711	-19.625	1.00	82.75	N
ATOM	2878	CA	GLU	A	363	-36.250	-10.977	-20.984	1.00	82.75	C
ATOM	2879	C	GLU	A	363	-35.094	-11.219	-21.938	1.00	82.75	C
ATOM	2880	CB	GLU	A	363	-37.125	-9.812	-21.484	1.00	82.75	C
ATOM	2881	O	GLU	A	363	-35.156	-12.094	-22.812	1.00	82.75	O
ATOM	2882	CG	GLU	A	363	-38.469	-9.695	-20.766	1.00	82.75	C
ATOM	2883	CD	GLU	A	363	-39.281	-8.523	-21.266	1.00	82.75	C
ATOM	2884	OE1	GLU	A	363	-40.406	-8.328	-20.750	1.00	82.75	O
ATOM	2885	OE2	GLU	A	363	-38.812	-7.801	-22.156	1.00	82.75	O
ATOM	2886	N	SER	A	364	-34.094	-10.477	-21.859	1.00	84.44	N
ATOM	2887	CA	SER	A	364	-32.969	-10.523	-22.781	1.00	84.44	C
ATOM	2888	C	SER	A	364	-31.656	-10.219	-22.078	1.00	84.44	C
ATOM	2889	CB	SER	A	364	-33.156	-9.547	-23.938	1.00	84.44	C
ATOM	2890	O	SER	A	364	-31.656	-9.734	-20.938	1.00	84.44	O
ATOM	2891	OG	SER	A	364	-33.250	-8.211	-23.453	1.00	84.44	O
ATOM	2892	N	LEU	A	365	-30.578	-10.773	-22.656	1.00	85.19	N
ATOM	2893	CA	LEU	A	365	-29.266	-10.344	-22.203	1.00	85.19	C
ATOM	2894	C	LEU	A	365	-29.031	-8.867	-22.516	1.00	85.19	C
ATOM	2895	CB	LEU	A	365	-28.172	-11.188	-22.875	1.00	85.19	C
ATOM	2896	O	LEU	A	365	-29.125	-8.445	-23.672	1.00	85.19	O
ATOM	2897	CG	LEU	A	365	-26.734	-10.914	-22.438	1.00	85.19	C
ATOM	2898	CD1	LEU	A	365	-26.531	-11.336	-20.984	1.00	85.19	C
ATOM	2899	CD2	LEU	A	365	-25.750	-11.633	-23.344	1.00	85.19	C
ATOM	2900	N	SER	A	366	-28.812	-8.023	-21.578	1.00	86.56	N
ATOM	2901	CA	SER	A	366	-28.656	-6.578	-21.719	1.00	86.56	C
ATOM	2902	C	SER	A	366	-27.312	-6.105	-21.172	1.00	86.56	C
ATOM	2903	CB	SER	A	366	-29.797	-5.840	-21.031	1.00	86.56	C
ATOM	2904	O	SER	A	366	-26.625	-6.855	-20.469	1.00	86.56	O
ATOM	2905	OG	SER	A	366	-29.812	-4.469	-21.391	1.00	86.56	O
ATOM	2906	N	ILE	A	367	-26.891	-4.926	-21.594	1.00	87.19	N
ATOM	2907	CA	ILE	A	367	-25.641	-4.340	-21.109	1.00	87.19	C
ATOM	2908	C	ILE	A	367	-25.938	-2.992	-20.453	1.00	87.19	C
ATOM	2909	CB	ILE	A	367	-24.609	-4.176	-22.250	1.00	87.19	C
ATOM	2910	O	ILE	A	367	-26.844	-2.279	-20.859	1.00	87.19	O
ATOM	2911	CG1	ILE	A	367	-25.156	-3.256	-23.344	1.00	87.19	C
ATOM	2912	CG2	ILE	A	367	-24.219	-5.539	-22.828	1.00	87.19	C
ATOM	2913	CD1	ILE	A	367	-24.109	-2.773	-24.328	1.00	87.19	C
ATOM	2914	N	VAL	A	368	-25.203	-2.787	-19.453	1.00	85.81	N
ATOM	2915	CA	VAL	A	368	-25.297	-1.482	-18.797	1.00	85.81	C
ATOM	2916	C	VAL	A	368	-23.906	-0.853	-18.719	1.00	85.81	C
ATOM	2917	CB	VAL	A	368	-25.906	-1.590	-17.391	1.00	85.81	C
ATOM	2918	O	VAL	A	368	-22.906	-1.551	-18.500	1.00	85.81	O
ATOM	2919	CG1	VAL	A	368	-25.000	-2.383	-16.453	1.00	85.81	C
ATOM	2920	CG2	VAL	A	368	-26.188	-0.199	-16.828	1.00	85.81	C
ATOM	2921	N	ILE	A	369	-23.844	0.447	-19.062	1.00	89.25	N
ATOM	2922	CA	ILE	A	369	-22.578	1.152	-19.047	1.00	89.25	C
ATOM	2923	C	ILE	A	369	-22.594	2.242	-17.969	1.00	89.25	C
ATOM	2924	CB	ILE	A	369	-22.250	1.764	-20.422	1.00	89.25	C
ATOM	2925	O	ILE	A	369	-23.656	2.760	-17.625	1.00	89.25	O
ATOM	2926	CG1	ILE	A	369	-23.375	2.727	-20.859	1.00	89.25	C
ATOM	2927	CG2	ILE	A	369	-22.047	0.663	-21.469	1.00	89.25	C
ATOM	2928	CD1	ILE	A	369	-23.078	3.459	-22.156	1.00	89.25	C
ATOM	2929	N	ASP	A	370	-21.391	2.488	-17.328	1.00	89.81	N
ATOM	2930	CA	ASP	A	370	-21.234	3.564	-16.359	1.00	89.81	C
ATOM	2931	C	ASP	A	370	-21.125	4.922	-17.047	1.00	89.81	C
ATOM	2932	CB	ASP	A	370	-19.984	3.322	-15.492	1.00	89.81	C
ATOM	2933	O	ASP	A	370	-20.031	5.355	-17.422	1.00	89.81	O
ATOM	2934	CG	ASP	A	370	-19.891	4.285	-14.320	1.00	89.81	C
ATOM	2935	OD1	ASP	A	370	-20.891	4.965	-14.000	1.00	89.81	O
ATOM	2936	OD2	ASP	A	370	-18.797	4.363	-13.711	1.00	89.81	O
ATOM	2937	N	LYS	A	371	-22.203	5.605	-17.250	1.00	89.56	N
ATOM	2938	CA	LYS	A	371	-22.328	6.910	-17.891	1.00	89.56	C
ATOM	2939	C	LYS	A	371	-23.203	7.848	-17.062	1.00	89.56	C
ATOM	2940	CB	LYS	A	371	-22.922	6.754	-19.297	1.00	89.56	C

ATOM	2941	O	LYS	A	371	-23.875	7.414	-16.125	1.00 89.56	O
ATOM	2942	CG	LYS	A	371	-24.359	6.246	-19.312	1.00 89.56	C
ATOM	2943	CD	LYS	A	371	-24.906	6.176	-20.734	1.00 89.56	C
ATOM	2944	CE	LYS	A	371	-26.312	5.598	-20.766	1.00 89.56	C
ATOM	2945	NZ	LYS	A	371	-27.344	6.660	-20.953	1.00 89.56	N
ATOM	2946	N	PRO	A	372	-23.016	9.156	-17.344	1.00 89.69	N
ATOM	2947	CA	PRO	A	372	-23.891	10.062	-16.594	1.00 89.69	C
ATOM	2948	C	PRO	A	372	-25.359	9.672	-16.672	1.00 89.69	C
ATOM	2949	CB	PRO	A	372	-23.641	11.414	-17.250	1.00 89.69	C
ATOM	2950	O	PRO	A	372	-25.875	9.422	-17.766	1.00 89.69	O
ATOM	2951	CG	PRO	A	372	-22.266	11.320	-17.828	1.00 89.69	C
ATOM	2952	CD	PRO	A	372	-22.047	9.906	-18.281	1.00 89.69	C
ATOM	2953	N	ARG	A	373	-25.922	9.539	-15.445	1.00 89.19	N
ATOM	2954	CA	ARG	A	373	-27.328	9.125	-15.344	1.00 89.19	C
ATOM	2955	C	ARG	A	373	-28.125	10.109	-14.500	1.00 89.19	C
ATOM	2956	CB	ARG	A	373	-27.438	7.723	-14.750	1.00 89.19	C
ATOM	2957	O	ARG	A	373	-27.594	10.727	-13.578	1.00 89.19	O
ATOM	2958	CG	ARG	A	373	-26.766	6.645	-15.594	1.00 89.19	C
ATOM	2959	CD	ARG	A	373	-26.812	5.285	-14.906	1.00 89.19	C
ATOM	2960	NE	ARG	A	373	-25.750	5.168	-13.898	1.00 89.19	N
ATOM	2961	NH1	ARG	A	373	-26.266	2.990	-13.320	1.00 89.19	N
ATOM	2962	NH2	ARG	A	373	-24.547	4.078	-12.281	1.00 89.19	N
ATOM	2963	CZ	ARG	A	373	-25.531	4.078	-13.172	1.00 89.19	C
ATOM	2964	N	ASP	A	374	-29.375	10.102	-14.828	1.00 89.00	N
ATOM	2965	CA	ASP	A	374	-30.281	10.906	-14.016	1.00 89.00	C
ATOM	2966	C	ASP	A	374	-30.906	10.070	-12.898	1.00 89.00	C
ATOM	2967	CB	ASP	A	374	-31.375	11.523	-14.883	1.00 89.00	C
ATOM	2968	O	ASP	A	374	-30.953	8.844	-12.984	1.00 89.00	O
ATOM	2969	CG	ASP	A	374	-30.844	12.531	-15.883	1.00 89.00	C
ATOM	2970	OD1	ASP	A	374	-29.859	13.234	-15.570	1.00 89.00	O
ATOM	2971	OD2	ASP	A	374	-31.422	12.633	-16.984	1.00 89.00	O
ATOM	2972	N	ALA	A	375	-31.281	10.727	-11.805	1.00 91.38	N
ATOM	2973	CA	ALA	A	375	-31.875	10.047	-10.656	1.00 91.38	C
ATOM	2974	C	ALA	A	375	-33.219	9.430	-11.031	1.00 91.38	C
ATOM	2975	CB	ALA	A	375	-32.031	11.008	-9.492	1.00 91.38	C
ATOM	2976	O	ALA	A	375	-34.000	10.016	-11.797	1.00 91.38	O
ATOM	2977	N	SER	A	376	-33.500	8.242	-10.484	1.00 91.62	N
ATOM	2978	CA	SER	A	376	-34.719	7.500	-10.773	1.00 91.62	C
ATOM	2979	C	SER	A	376	-35.781	7.746	-9.711	1.00 91.62	C
ATOM	2980	CB	SER	A	376	-34.438	6.004	-10.883	1.00 91.62	C
ATOM	2981	O	SER	A	376	-36.969	7.613	-9.977	1.00 91.62	O
ATOM	2982	OG	SER	A	376	-33.875	5.512	-9.680	1.00 91.62	O
ATOM	2983	N	TYR	A	377	-35.344	8.008	-8.523	1.00 94.19	N
ATOM	2984	CA	TYR	A	377	-36.312	8.172	-7.438	1.00 94.19	C
ATOM	2985	C	TYR	A	377	-35.656	8.844	-6.234	1.00 94.19	C
ATOM	2986	CB	TYR	A	377	-36.906	6.820	-7.023	1.00 94.19	C
ATOM	2987	O	TYR	A	377	-34.469	8.719	-6.023	1.00 94.19	O
ATOM	2988	CG	TYR	A	377	-38.281	6.918	-6.387	1.00 94.19	C
ATOM	2989	CD1	TYR	A	377	-38.438	6.770	-5.012	1.00 94.19	C
ATOM	2990	CD2	TYR	A	377	-39.406	7.160	-7.156	1.00 94.19	C
ATOM	2991	CE1	TYR	A	377	-39.688	6.859	-4.418	1.00 94.19	C
ATOM	2992	CE2	TYR	A	377	-40.656	7.254	-6.574	1.00 94.19	C
ATOM	2993	OH	TYR	A	377	-42.031	7.191	-4.625	1.00 94.19	O
ATOM	2994	CZ	TYR	A	377	-40.781	7.102	-5.207	1.00 94.19	C
ATOM	2995	N	ILE	A	378	-36.531	9.531	-5.387	1.00 94.62	N
ATOM	2996	CA	ILE	A	378	-36.062	10.227	-4.191	1.00 94.62	C
ATOM	2997	C	ILE	A	378	-36.469	9.445	-2.947	1.00 94.62	C
ATOM	2998	CB	ILE	A	378	-36.594	11.672	-4.129	1.00 94.62	C
ATOM	2999	O	ILE	A	378	-37.625	9.031	-2.820	1.00 94.62	O
ATOM	3000	CG1	ILE	A	378	-36.219	12.445	-5.391	1.00 94.62	C
ATOM	3001	CG2	ILE	A	378	-36.094	12.383	-2.869	1.00 94.62	C
ATOM	3002	CD1	ILE	A	378	-37.000	13.727	-5.602	1.00 94.62	C
ATOM	3003	N	PHE	A	379	-35.500	9.180	-2.068	1.00 95.25	N
ATOM	3004	CA	PHE	A	379	-35.781	8.594	-0.765	1.00 95.25	C

ATOM	3005	C	PHE	A	379	-35.469	9.586	0.354	1.00	95.25	C
ATOM	3006	CB	PHE	A	379	-34.969	7.309	-0.564	1.00	95.25	C
ATOM	3007	O	PHE	A	379	-34.375	10.148	0.403	1.00	95.25	O
ATOM	3008	CG	PHE	A	379	-35.438	6.156	-1.414	1.00	95.25	C
ATOM	3009	CD1	PHE	A	379	-36.531	5.391	-1.024	1.00	95.25	C
ATOM	3010	CD2	PHE	A	379	-34.781	5.844	-2.602	1.00	95.25	C
ATOM	3011	CE1	PHE	A	379	-36.969	4.324	-1.808	1.00	95.25	C
ATOM	3012	CE2	PHE	A	379	-35.219	4.781	-3.391	1.00	95.25	C
ATOM	3013	CZ	PHE	A	379	-36.312	4.023	-2.992	1.00	95.25	C
ATOM	3014	N	THR	A	380	-36.438	9.766	1.225	1.00	94.69	N
ATOM	3015	CA	THR	A	380	-36.344	10.633	2.396	1.00	94.69	C
ATOM	3016	C	THR	A	380	-36.656	9.852	3.672	1.00	94.69	C
ATOM	3017	CB	THR	A	380	-37.281	11.836	2.287	1.00	94.69	C
ATOM	3018	O	THR	A	380	-37.000	8.672	3.615	1.00	94.69	O
ATOM	3019	CG2	THR	A	380	-37.125	12.547	0.946	1.00	94.69	C
ATOM	3020	OG1	THR	A	380	-38.656	11.383	2.420	1.00	94.69	O
ATOM	3021	N	ASN	A	381	-36.438	10.500	4.844	1.00	94.12	N
ATOM	3022	CA	ASN	A	381	-36.719	9.844	6.113	1.00	94.12	C
ATOM	3023	C	ASN	A	381	-38.188	9.422	6.215	1.00	94.12	C
ATOM	3024	CB	ASN	A	381	-36.344	10.758	7.285	1.00	94.12	C
ATOM	3025	O	ASN	A	381	-38.500	8.492	6.953	1.00	94.12	O
ATOM	3026	CG	ASN	A	381	-34.875	11.078	7.332	1.00	94.12	C
ATOM	3027	ND2	ASN	A	381	-34.125	10.289	8.086	1.00	94.12	N
ATOM	3028	OD1	ASN	A	381	-34.406	12.031	6.695	1.00	94.12	O
ATOM	3029	N	ASP	A	382	-39.188	9.891	5.387	1.00	92.81	N
ATOM	3030	CA	ASP	A	382	-40.625	9.656	5.473	1.00	92.81	C
ATOM	3031	C	ASP	A	382	-41.000	8.398	4.699	1.00	92.81	C
ATOM	3032	CB	ASP	A	382	-41.406	10.867	4.949	1.00	92.81	C
ATOM	3033	O	ASP	A	382	-42.031	7.785	4.988	1.00	92.81	O
ATOM	3034	CG	ASP	A	382	-41.625	11.922	6.012	1.00	92.81	C
ATOM	3035	OD1	ASP	A	382	-41.125	11.781	7.141	1.00	92.81	O
ATOM	3036	OD2	ASP	A	382	-42.344	12.914	5.719	1.00	92.81	O
ATOM	3037	N	ASN	A	383	-40.188	8.070	3.662	1.00	93.19	N
ATOM	3038	CA	ASN	A	383	-40.562	6.875	2.906	1.00	93.19	C
ATOM	3039	C	ASN	A	383	-39.594	5.723	3.178	1.00	93.19	C
ATOM	3040	CB	ASN	A	383	-40.625	7.184	1.407	1.00	93.19	C
ATOM	3041	O	ASN	A	383	-39.594	4.738	2.438	1.00	93.19	O
ATOM	3042	CG	ASN	A	383	-39.250	7.539	0.841	1.00	93.19	C
ATOM	3043	ND2	ASN	A	383	-39.156	7.527	-0.482	1.00	93.19	N
ATOM	3044	OD1	ASN	A	383	-38.312	7.824	1.586	1.00	93.19	O
ATOM	3045	N	VAL	A	384	-38.812	5.867	4.125	1.00	94.88	N
ATOM	3046	CA	VAL	A	384	-37.938	4.816	4.598	1.00	94.88	C
ATOM	3047	C	VAL	A	384	-38.438	4.277	5.938	1.00	94.88	C
ATOM	3048	CB	VAL	A	384	-36.469	5.324	4.734	1.00	94.88	C
ATOM	3049	O	VAL	A	384	-38.875	5.043	6.789	1.00	94.88	O
ATOM	3050	CG1	VAL	A	384	-35.625	4.312	5.496	1.00	94.88	C
ATOM	3051	CG2	VAL	A	384	-35.875	5.613	3.359	1.00	94.88	C
ATOM	3052	N	VAL	A	385	-38.500	2.982	6.086	1.00	92.44	N
ATOM	3053	CA	VAL	A	385	-39.000	2.328	7.289	1.00	92.44	C
ATOM	3054	C	VAL	A	385	-38.281	2.900	8.516	1.00	92.44	C
ATOM	3055	CB	VAL	A	385	-38.781	0.797	7.227	1.00	92.44	C
ATOM	3056	O	VAL	A	385	-37.062	2.914	8.586	1.00	92.44	O
ATOM	3057	CG1	VAL	A	385	-39.250	0.141	8.531	1.00	92.44	C
ATOM	3058	CG2	VAL	A	385	-39.562	0.209	6.039	1.00	92.44	C
ATOM	3059	N	ASN	A	386	-38.969	3.582	9.461	1.00	91.31	N
ATOM	3060	CA	ASN	A	386	-38.500	4.203	10.695	1.00	91.31	C
ATOM	3061	C	ASN	A	386	-37.625	5.430	10.406	1.00	91.31	C
ATOM	3062	CB	ASN	A	386	-37.719	3.191	11.539	1.00	91.31	C
ATOM	3063	O	ASN	A	386	-36.906	5.906	11.289	1.00	91.31	O
ATOM	3064	CG	ASN	A	386	-38.594	2.053	12.031	1.00	91.31	C
ATOM	3065	ND2	ASN	A	386	-37.969	0.878	12.195	1.00	91.31	N
ATOM	3066	OD1	ASN	A	386	-39.781	2.227	12.266	1.00	91.31	O
ATOM	3067	N	GLY	A	387	-37.500	5.758	9.094	1.00	91.00	N
ATOM	3068	CA	GLY	A	387	-36.719	6.910	8.703	1.00	91.00	C

ATOM	3069	C	GLY	A	387	-35.219	6.699	8.891	1.00	91.00	C
ATOM	3070	O	GLY	A	387	-34.469	7.664	9.039	1.00	91.00	O
ATOM	3071	N	GLU	A	388	-34.719	5.480	8.844	1.00	94.19	N
ATOM	3072	CA	GLU	A	388	-33.344	5.113	9.211	1.00	94.19	C
ATOM	3073	C	GLU	A	388	-32.531	4.762	7.980	1.00	94.19	C
ATOM	3074	CB	GLU	A	388	-33.375	3.938	10.195	1.00	94.19	C
ATOM	3075	O	GLU	A	388	-32.844	3.812	7.262	1.00	94.19	O
ATOM	3076	CG	GLU	A	388	-32.000	3.623	10.789	1.00	94.19	C
ATOM	3077	CD	GLU	A	388	-32.031	2.461	11.766	1.00	94.19	C
ATOM	3078	OE1	GLU	A	388	-30.953	2.084	12.289	1.00	94.19	O
ATOM	3079	OE2	GLU	A	388	-33.125	1.924	12.016	1.00	94.19	O
ATOM	3080	N	PHE	A	389	-31.516	5.527	7.832	1.00	95.94	N
ATOM	3081	CA	PHE	A	389	-30.453	5.227	6.871	1.00	95.94	C
ATOM	3082	C	PHE	A	389	-29.203	4.750	7.582	1.00	95.94	C
ATOM	3083	CB	PHE	A	389	-30.141	6.453	6.012	1.00	95.94	C
ATOM	3084	O	PHE	A	389	-28.766	5.363	8.562	1.00	95.94	O
ATOM	3085	CG	PHE	A	389	-31.266	6.852	5.090	1.00	95.94	C
ATOM	3086	CD1	PHE	A	389	-31.453	6.203	3.875	1.00	95.94	C
ATOM	3087	CD2	PHE	A	389	-32.125	7.875	5.438	1.00	95.94	C
ATOM	3088	CE1	PHE	A	389	-32.469	6.566	3.018	1.00	95.94	C
ATOM	3089	CE2	PHE	A	389	-33.188	8.242	4.586	1.00	95.94	C
ATOM	3090	CZ	PHE	A	389	-33.344	7.590	3.375	1.00	95.94	C
ATOM	3091	N	THR	A	390	-28.609	3.734	7.125	1.00	95.50	N
ATOM	3092	CA	THR	A	390	-27.359	3.256	7.688	1.00	95.50	C
ATOM	3093	C	THR	A	390	-26.219	3.398	6.680	1.00	95.50	C
ATOM	3094	CB	THR	A	390	-27.469	1.787	8.133	1.00	95.50	C
ATOM	3095	O	THR	A	390	-26.328	2.941	5.539	1.00	95.50	O
ATOM	3096	CG2	THR	A	390	-26.203	1.340	8.875	1.00	95.50	C
ATOM	3097	OG1	THR	A	390	-28.594	1.643	9.008	1.00	95.50	O
ATOM	3098	N	TYR	A	391	-25.125	4.059	7.121	1.00	94.62	N
ATOM	3099	CA	TYR	A	391	-24.000	4.332	6.223	1.00	94.62	C
ATOM	3100	C	TYR	A	391	-22.781	3.504	6.609	1.00	94.62	C
ATOM	3101	CB	TYR	A	391	-23.656	5.820	6.242	1.00	94.62	C
ATOM	3102	O	TYR	A	391	-22.500	3.322	7.793	1.00	94.62	O
ATOM	3103	CG	TYR	A	391	-24.781	6.719	5.793	1.00	94.62	C
ATOM	3104	CD1	TYR	A	391	-24.984	7.000	4.441	1.00	94.62	C
ATOM	3105	CD2	TYR	A	391	-25.656	7.285	6.715	1.00	94.62	C
ATOM	3106	CE1	TYR	A	391	-26.016	7.824	4.023	1.00	94.62	C
ATOM	3107	CE2	TYR	A	391	-26.688	8.109	6.309	1.00	94.62	C
ATOM	3108	OH	TYR	A	391	-27.891	9.195	4.555	1.00	94.62	O
ATOM	3109	CZ	TYR	A	391	-26.875	8.375	4.961	1.00	94.62	C
ATOM	3110	N	THR	A	392	-22.156	2.990	5.602	1.00	93.31	N
ATOM	3111	CA	THR	A	392	-20.906	2.258	5.746	1.00	93.31	C
ATOM	3112	C	THR	A	392	-19.797	2.906	4.918	1.00	93.31	C
ATOM	3113	CB	THR	A	392	-21.078	0.785	5.324	1.00	93.31	C
ATOM	3114	O	THR	A	392	-20.000	3.195	3.734	1.00	93.31	O
ATOM	3115	CG2	THR	A	392	-19.797	-0.012	5.613	1.00	93.31	C
ATOM	3116	OG1	THR	A	392	-22.156	0.204	6.055	1.00	93.31	O
ATOM	3117	N	PHE	A	393	-18.719	3.230	5.605	1.00	89.00	N
ATOM	3118	CA	PHE	A	393	-17.594	3.857	4.930	1.00	89.00	C
ATOM	3119	C	PHE	A	393	-16.453	2.857	4.730	1.00	89.00	C
ATOM	3120	CB	PHE	A	393	-17.094	5.066	5.727	1.00	89.00	C
ATOM	3121	O	PHE	A	393	-16.266	1.950	5.543	1.00	89.00	O
ATOM	3122	CG	PHE	A	393	-18.156	6.086	6.016	1.00	89.00	C
ATOM	3123	CD1	PHE	A	393	-18.453	7.086	5.098	1.00	89.00	C
ATOM	3124	CD2	PHE	A	393	-18.859	6.043	7.211	1.00	89.00	C
ATOM	3125	CE1	PHE	A	393	-19.438	8.031	5.363	1.00	89.00	C
ATOM	3126	CE2	PHE	A	393	-19.859	6.984	7.484	1.00	89.00	C
ATOM	3127	CZ	PHE	A	393	-20.141	7.980	6.559	1.00	89.00	C
ATOM	3128	N	ALA	A	394	-15.836	3.049	3.547	1.00	87.00	N
ATOM	3129	CA	ALA	A	394	-14.734	2.145	3.232	1.00	87.00	C
ATOM	3130	C	ALA	A	394	-13.531	2.418	4.129	1.00	87.00	C
ATOM	3131	CB	ALA	A	394	-14.344	2.275	1.763	1.00	87.00	C
ATOM	3132	O	ALA	A	394	-13.359	3.531	4.629	1.00	87.00	O

ATOM	3133	N	SER	A	395	-12.781	1.407	4.406	1.00	88.12	N
ATOM	3134	CA	SER	A	395	-11.555	1.532	5.191	1.00	88.12	C
ATOM	3135	C	SER	A	395	-10.539	2.422	4.488	1.00	88.12	C
ATOM	3136	CB	SER	A	395	-10.945	0.156	5.457	1.00	88.12	C
ATOM	3137	O	SER	A	395	-10.477	2.453	3.258	1.00	88.12	O
ATOM	3138	OG	SER	A	395	-9.656	0.279	6.027	1.00	88.12	O
ATOM	3139	N	GLU	A	396	-9.828	3.193	5.289	1.00	86.19	N
ATOM	3140	CA	GLU	A	396	-8.773	4.055	4.750	1.00	86.19	C
ATOM	3141	C	GLU	A	396	-7.797	3.260	3.893	1.00	86.19	C
ATOM	3142	CB	GLU	A	396	-8.031	4.766	5.879	1.00	86.19	C
ATOM	3143	O	GLU	A	396	-7.312	3.754	2.871	1.00	86.19	O
ATOM	3144	CG	GLU	A	396	-7.043	5.820	5.398	1.00	86.19	C
ATOM	3145	CD	GLU	A	396	-6.336	6.539	6.535	1.00	86.19	C
ATOM	3146	OE1	GLU	A	396	-5.465	7.398	6.262	1.00	86.19	O
ATOM	3147	OE2	GLU	A	396	-6.648	6.238	7.711	1.00	86.19	O
ATOM	3148	N	LYS	A	397	-7.531	2.059	4.082	1.00	86.69	N
ATOM	3149	CA	LYS	A	397	-6.586	1.207	3.365	1.00	86.69	C
ATOM	3150	C	LYS	A	397	-7.137	0.807	2.000	1.00	86.69	C
ATOM	3151	CB	LYS	A	397	-6.258	-0.042	4.188	1.00	86.69	C
ATOM	3152	O	LYS	A	397	-6.371	0.484	1.088	1.00	86.69	O
ATOM	3153	CG	LYS	A	397	-5.430	0.235	5.430	1.00	86.69	C
ATOM	3154	CD	LYS	A	397	-5.016	-1.057	6.125	1.00	86.69	C
ATOM	3155	CE	LYS	A	397	-4.180	-0.781	7.367	1.00	86.69	C
ATOM	3156	NZ	LYS	A	397	-3.793	-2.043	8.070	1.00	86.69	N
ATOM	3157	N	SER	A	398	-8.375	0.786	1.899	1.00	87.31	N
ATOM	3158	CA	SER	A	398	-9.000	0.383	0.642	1.00	87.31	C
ATOM	3159	C	SER	A	398	-9.266	1.588	-0.254	1.00	87.31	C
ATOM	3160	CB	SER	A	398	-10.305	-0.367	0.905	1.00	87.31	C
ATOM	3161	O	SER	A	398	-9.812	1.443	-1.351	1.00	87.31	O
ATOM	3162	OG	SER	A	398	-10.055	-1.578	1.601	1.00	87.31	O
ATOM	3163	N	MET	A	399	-8.875	2.770	0.316	1.00	91.19	N
ATOM	3164	CA	MET	A	399	-8.977	3.969	-0.512	1.00	91.19	C
ATOM	3165	C	MET	A	399	-7.730	4.133	-1.382	1.00	91.19	C
ATOM	3166	CB	MET	A	399	-9.172	5.211	0.359	1.00	91.19	C
ATOM	3167	O	MET	A	399	-6.734	4.715	-0.946	1.00	91.19	O
ATOM	3168	CG	MET	A	399	-10.484	5.215	1.129	1.00	91.19	C
ATOM	3169	SD	MET	A	399	-11.945	5.270	0.024	1.00	91.19	S
ATOM	3170	CE	MET	A	399	-12.914	6.570	0.838	1.00	91.19	C
ATOM	3171	N	TYR	A	400	-7.836	3.676	-2.668	1.00	94.44	N
ATOM	3172	CA	TYR	A	400	-6.707	3.670	-3.594	1.00	94.44	C
ATOM	3173	C	TYR	A	400	-6.410	5.078	-4.098	1.00	94.44	C
ATOM	3174	CB	TYR	A	400	-6.988	2.740	-4.777	1.00	94.44	C
ATOM	3175	O	TYR	A	400	-7.324	5.828	-4.441	1.00	94.44	O
ATOM	3176	CG	TYR	A	400	-7.281	1.316	-4.375	1.00	94.44	C
ATOM	3177	CD1	TYR	A	400	-6.387	0.592	-3.592	1.00	94.44	C
ATOM	3178	CD2	TYR	A	400	-8.461	0.691	-4.773	1.00	94.44	C
ATOM	3179	CE1	TYR	A	400	-6.652	-0.721	-3.221	1.00	94.44	C
ATOM	3180	CE2	TYR	A	400	-8.734	-0.621	-4.406	1.00	94.44	C
ATOM	3181	OH	TYR	A	400	-8.102	-2.617	-3.266	1.00	94.44	O
ATOM	3182	CZ	TYR	A	400	-7.828	-1.318	-3.631	1.00	94.44	C
ATOM	3183	N	THR	A	401	-5.152	5.434	-4.113	1.00	94.69	N
ATOM	3184	CA	THR	A	401	-4.719	6.773	-4.500	1.00	94.69	C
ATOM	3185	C	THR	A	401	-3.863	6.719	-5.762	1.00	94.69	C
ATOM	3186	CB	THR	A	401	-3.922	7.449	-3.365	1.00	94.69	C
ATOM	3187	O	THR	A	401	-3.533	7.758	-6.336	1.00	94.69	O
ATOM	3188	CG2	THR	A	401	-4.793	7.660	-2.133	1.00	94.69	C
ATOM	3189	OG1	THR	A	401	-2.809	6.617	-3.012	1.00	94.69	O
ATOM	3190	N	GLN	A	402	-3.531	5.531	-6.195	1.00	95.38	N
ATOM	3191	CA	GLN	A	402	-2.768	5.305	-7.418	1.00	95.38	C
ATOM	3192	C	GLN	A	402	-3.199	4.012	-8.102	1.00	95.38	C
ATOM	3193	CB	GLN	A	402	-1.270	5.262	-7.113	1.00	95.38	C
ATOM	3194	O	GLN	A	402	-3.506	3.021	-7.434	1.00	95.38	O
ATOM	3195	CG	GLN	A	402	-0.409	4.910	-8.320	1.00	95.38	C
ATOM	3196	CD	GLN	A	402	1.062	4.773	-7.973	1.00	95.38	C

ATOM	3197	NE2	GLN	A	402	1.927	5.000	-8.953	1.00	95.38	N
ATOM	3198	OE1	GLN	A	402	1.417	4.469	-6.828	1.00	95.38	O
ATOM	3199	N	CYS	A	403	-3.244	4.035	-9.477	1.00	97.00	N
ATOM	3200	CA	CYS	A	403	-3.588	2.846	-10.250	1.00	97.00	C
ATOM	3201	C	CYS	A	403	-2.590	2.623	-11.375	1.00	97.00	C
ATOM	3202	CB	CYS	A	403	-5.000	2.969	-10.820	1.00	97.00	C
ATOM	3203	O	CYS	A	403	-2.373	3.514	-12.203	1.00	97.00	O
ATOM	3204	SG	CYS	A	403	-5.559	1.495	-11.695	1.00	97.00	S
ATOM	3205	N	ASN	A	404	-1.991	1.421	-11.406	1.00	97.62	N
ATOM	3206	CA	ASN	A	404	-1.132	0.999	-12.508	1.00	97.62	C
ATOM	3207	C	ASN	A	404	-1.913	0.218	-13.562	1.00	97.62	C
ATOM	3208	CB	ASN	A	404	0.038	0.162	-11.984	1.00	97.62	C
ATOM	3209	O	ASN	A	404	-2.436	-0.861	-13.273	1.00	97.62	O
ATOM	3210	CG	ASN	A	404	0.946	0.944	-11.055	1.00	97.62	C
ATOM	3211	ND2	ASN	A	404	1.400	0.298	-9.984	1.00	97.62	N
ATOM	3212	OD1	ASN	A	404	1.236	2.119	-11.297	1.00	97.62	O
ATOM	3213	N	VAL	A	405	-2.023	0.835	-14.781	1.00	97.81	N
ATOM	3214	CA	VAL	A	405	-2.830	0.229	-15.836	1.00	97.81	C
ATOM	3215	C	VAL	A	405	-1.918	-0.334	-16.922	1.00	97.81	C
ATOM	3216	CB	VAL	A	405	-3.824	1.243	-16.453	1.00	97.81	C
ATOM	3217	O	VAL	A	405	-1.225	0.419	-17.609	1.00	97.81	O
ATOM	3218	CG1	VAL	A	405	-4.746	0.558	-17.453	1.00	97.81	C
ATOM	3219	CG2	VAL	A	405	-4.637	1.922	-15.344	1.00	97.81	C
ATOM	3220	N	THR	A	406	-1.920	-1.710	-17.016	1.00	97.69	N
ATOM	3221	CA	THR	A	406	-1.150	-2.371	-18.062	1.00	97.69	C
ATOM	3222	C	THR	A	406	-1.923	-2.377	-19.375	1.00	97.69	C
ATOM	3223	CB	THR	A	406	-0.792	-3.814	-17.672	1.00	97.69	C
ATOM	3224	O	THR	A	406	-3.117	-2.684	-19.406	1.00	97.69	O
ATOM	3225	CG2	THR	A	406	0.060	-4.484	-18.750	1.00	97.69	C
ATOM	3226	OG1	THR	A	406	-0.063	-3.801	-16.438	1.00	97.69	O
ATOM	3227	N	PHE	A	407	-1.276	-1.902	-20.516	1.00	96.81	N
ATOM	3228	CA	PHE	A	407	-1.859	-1.875	-21.859	1.00	96.81	C
ATOM	3229	C	PHE	A	407	-0.830	-2.275	-22.906	1.00	96.81	C
ATOM	3230	CB	PHE	A	407	-2.418	-0.484	-22.172	1.00	96.81	C
ATOM	3231	O	PHE	A	407	0.362	-2.377	-22.594	1.00	96.81	O
ATOM	3232	CG	PHE	A	407	-1.356	0.547	-22.438	1.00	96.81	C
ATOM	3233	CD1	PHE	A	407	-0.551	1.028	-21.422	1.00	96.81	C
ATOM	3234	CD2	PHE	A	407	-1.165	1.034	-23.734	1.00	96.81	C
ATOM	3235	CE1	PHE	A	407	0.432	1.982	-21.672	1.00	96.81	C
ATOM	3236	CE2	PHE	A	407	-0.185	1.987	-24.000	1.00	96.81	C
ATOM	3237	CZ	PHE	A	407	0.612	2.461	-22.969	1.00	96.81	C
ATOM	3238	N	ASP	A	408	-1.303	-2.605	-24.078	1.00	95.81	N
ATOM	3239	CA	ASP	A	408	-0.417	-2.875	-25.203	1.00	95.81	C
ATOM	3240	C	ASP	A	408	-0.102	-1.595	-25.984	1.00	95.81	C
ATOM	3241	CB	ASP	A	408	-1.043	-3.910	-26.141	1.00	95.81	C
ATOM	3242	O	ASP	A	408	-0.996	-0.977	-26.562	1.00	95.81	O
ATOM	3243	CG	ASP	A	408	-1.162	-5.285	-25.516	1.00	95.81	C
ATOM	3244	OD1	ASP	A	408	-0.432	-5.578	-24.547	1.00	95.81	O
ATOM	3245	OD2	ASP	A	408	-1.992	-6.090	-26.000	1.00	95.81	O
ATOM	3246	N	ASP	A	409	1.187	-1.217	-25.938	1.00	94.88	N
ATOM	3247	CA	ASP	A	409	1.644	0.077	-26.438	1.00	94.88	C
ATOM	3248	C	ASP	A	409	1.932	0.019	-27.938	1.00	94.88	C
ATOM	3249	CB	ASP	A	409	2.893	0.532	-25.672	1.00	94.88	C
ATOM	3250	O	ASP	A	409	2.881	-0.641	-28.359	1.00	94.88	O
ATOM	3251	CG	ASP	A	409	3.297	1.960	-26.000	1.00	94.88	C
ATOM	3252	OD1	ASP	A	409	2.688	2.576	-26.906	1.00	94.88	O
ATOM	3253	OD2	ASP	A	409	4.234	2.473	-25.359	1.00	94.88	O
ATOM	3254	N	GLU	A	410	1.143	0.705	-28.641	1.00	89.88	N
ATOM	3255	CA	GLU	A	410	1.286	0.727	-30.094	1.00	89.88	C
ATOM	3256	C	GLU	A	410	2.605	1.373	-30.516	1.00	89.88	C
ATOM	3257	CB	GLU	A	410	0.112	1.466	-30.734	1.00	89.88	C
ATOM	3258	O	GLU	A	410	3.186	1.007	-31.531	1.00	89.88	O
ATOM	3259	CG	GLU	A	410	0.098	2.961	-30.453	1.00	89.88	C
ATOM	3260	CD	GLU	A	410	-1.026	3.695	-31.172	1.00	89.88	C

ATOM	3261	OE1	GLU	A	410	-1.090	4.941	-31.078	1.00	89.88	O
ATOM	3262	OE2	GLU	A	410	-1.851	3.018	-31.828	1.00	89.88	O
ATOM	3263	N	GLN	A	411	3.119	2.334	-29.781	1.00	89.56	N
ATOM	3264	CA	GLN	A	411	4.363	3.035	-30.078	1.00	89.56	C
ATOM	3265	C	GLN	A	411	5.574	2.143	-29.828	1.00	89.56	C
ATOM	3266	CB	GLN	A	411	4.473	4.316	-29.250	1.00	89.56	C
ATOM	3267	O	GLN	A	411	6.672	2.424	-30.312	1.00	89.56	O
ATOM	3268	CG	GLN	A	411	3.436	5.371	-29.609	1.00	89.56	C
ATOM	3269	CD	GLN	A	411	3.568	6.633	-28.781	1.00	89.56	C
ATOM	3270	NE2	GLN	A	411	3.062	7.746	-29.297	1.00	89.56	N
ATOM	3271	OE1	GLN	A	411	4.117	6.602	-27.672	1.00	89.56	O
ATOM	3272	N	ASN	A	412	5.277	1.059	-29.047	1.00	91.06	N
ATOM	3273	CA	ASN	A	412	6.316	0.076	-28.750	1.00	91.06	C
ATOM	3274	C	ASN	A	412	5.973	-1.293	-29.328	1.00	91.06	C
ATOM	3275	CB	ASN	A	412	6.539	-0.031	-27.234	1.00	91.06	C
ATOM	3276	O	ASN	A	412	6.145	-2.316	-28.672	1.00	91.06	O
ATOM	3277	CG	ASN	A	412	7.832	-0.747	-26.891	1.00	91.06	C
ATOM	3278	ND2	ASN	A	412	7.906	-1.277	-25.672	1.00	91.06	N
ATOM	3279	OD1	ASN	A	412	8.750	-0.821	-27.703	1.00	91.06	O
ATOM	3280	N	MET	A	413	5.422	-1.319	-30.484	1.00	89.00	N
ATOM	3281	CA	MET	A	413	5.109	-2.514	-31.266	1.00	89.00	C
ATOM	3282	C	MET	A	413	4.172	-3.438	-30.500	1.00	89.00	C
ATOM	3283	CB	MET	A	413	6.391	-3.260	-31.641	1.00	89.00	C
ATOM	3284	O	MET	A	413	4.352	-4.656	-30.516	1.00	89.00	O
ATOM	3285	CG	MET	A	413	7.332	-2.457	-32.531	1.00	89.00	C
ATOM	3286	SD	MET	A	413	6.551	-1.956	-34.125	1.00	89.00	S
ATOM	3287	CE	MET	A	413	6.859	-3.443	-35.094	1.00	89.00	C
ATOM	3288	N	TYR	A	414	3.283	-2.883	-29.594	1.00	90.75	N
ATOM	3289	CA	TYR	A	414	2.201	-3.559	-28.875	1.00	90.75	C
ATOM	3290	C	TYR	A	414	2.746	-4.434	-27.750	1.00	90.75	C
ATOM	3291	CB	TYR	A	414	1.372	-4.406	-29.844	1.00	90.75	C
ATOM	3292	O	TYR	A	414	2.146	-5.457	-27.406	1.00	90.75	O
ATOM	3293	CG	TYR	A	414	0.618	-3.596	-30.875	1.00	90.75	C
ATOM	3294	CD1	TYR	A	414	-0.632	-3.055	-30.578	1.00	90.75	C
ATOM	3295	CD2	TYR	A	414	1.153	-3.367	-32.125	1.00	90.75	C
ATOM	3296	CE1	TYR	A	414	-1.331	-2.307	-31.516	1.00	90.75	C
ATOM	3297	CE2	TYR	A	414	0.463	-2.621	-33.094	1.00	90.75	C
ATOM	3298	OH	TYR	A	414	-1.465	-1.357	-33.719	1.00	90.75	O
ATOM	3299	CZ	TYR	A	414	-0.776	-2.096	-32.781	1.00	90.75	C
ATOM	3300	N	GLN	A	415	3.951	-4.141	-27.344	1.00	92.81	N
ATOM	3301	CA	GLN	A	415	4.457	-4.734	-26.109	1.00	92.81	C
ATOM	3302	C	GLN	A	415	3.740	-4.168	-24.891	1.00	92.81	C
ATOM	3303	CB	GLN	A	415	5.965	-4.512	-25.984	1.00	92.81	C
ATOM	3304	O	GLN	A	415	3.289	-3.023	-24.906	1.00	92.81	O
ATOM	3305	CG	GLN	A	415	6.793	-5.312	-26.984	1.00	92.81	C
ATOM	3306	CD	GLN	A	415	8.281	-5.129	-26.797	1.00	92.81	C
ATOM	3307	NE2	GLN	A	415	9.078	-5.879	-27.547	1.00	92.81	N
ATOM	3308	OE1	GLN	A	415	8.719	-4.324	-25.969	1.00	92.81	O
ATOM	3309	N	GLN	A	416	3.641	-5.066	-23.781	1.00	94.88	N
ATOM	3310	CA	GLN	A	416	2.920	-4.625	-22.594	1.00	94.88	C
ATOM	3311	C	GLN	A	416	3.660	-3.490	-21.891	1.00	94.88	C
ATOM	3312	CB	GLN	A	416	2.711	-5.793	-21.625	1.00	94.88	C
ATOM	3313	O	GLN	A	416	4.879	-3.551	-21.719	1.00	94.88	O
ATOM	3314	CG	GLN	A	416	1.724	-6.836	-22.125	1.00	94.88	C
ATOM	3315	CD	GLN	A	416	1.513	-7.969	-21.141	1.00	94.88	C
ATOM	3316	NE2	GLN	A	416	0.459	-8.750	-21.344	1.00	94.88	N
ATOM	3317	OE1	GLN	A	416	2.291	-8.133	-20.188	1.00	94.88	O
ATOM	3318	N	ASP	A	417	3.016	-2.391	-21.672	1.00	97.62	N
ATOM	3319	CA	ASP	A	417	3.500	-1.234	-20.938	1.00	97.62	C
ATOM	3320	C	ASP	A	417	2.512	-0.833	-19.844	1.00	97.62	C
ATOM	3321	CB	ASP	A	417	3.750	-0.054	-21.875	1.00	97.62	C
ATOM	3322	O	ASP	A	417	1.353	-1.253	-19.859	1.00	97.62	O
ATOM	3323	CG	ASP	A	417	4.688	0.985	-21.281	1.00	97.62	C
ATOM	3324	OD1	ASP	A	417	5.211	0.773	-20.172	1.00	97.62	O

ATOM	3325	OD2	ASP	A	417	4.906	2.025	-21.938	1.00	97.62	O
ATOM	3326	N	VAL	A	418	3.018	-0.111	-18.766	1.00	97.25	N
ATOM	3327	CA	VAL	A	418	2.182	0.270	-17.641	1.00	97.25	C
ATOM	3328	C	VAL	A	418	2.039	1.790	-17.594	1.00	97.25	C
ATOM	3329	CB	VAL	A	418	2.758	-0.255	-16.312	1.00	97.25	C
ATOM	3330	O	VAL	A	418	3.035	2.516	-17.672	1.00	97.25	O
ATOM	3331	CG1	VAL	A	418	1.891	0.190	-15.133	1.00	97.25	C
ATOM	3332	CG2	VAL	A	418	2.879	-1.777	-16.344	1.00	97.25	C
ATOM	3333	N	GLU	A	419	0.734	2.252	-17.516	1.00	96.25	N
ATOM	3334	CA	GLU	A	419	0.436	3.664	-17.297	1.00	96.25	C
ATOM	3335	C	GLU	A	419	0.073	3.932	-15.836	1.00	96.25	C
ATOM	3336	CB	GLU	A	419	-0.701	4.125	-18.219	1.00	96.25	C
ATOM	3337	O	GLU	A	419	-0.904	3.381	-15.328	1.00	96.25	O
ATOM	3338	CG	GLU	A	419	-1.126	5.566	-17.984	1.00	96.25	C
ATOM	3339	CD	GLU	A	419	-0.024	6.570	-18.297	1.00	96.25	C
ATOM	3340	OE1	GLU	A	419	-0.127	7.738	-17.844	1.00	96.25	O
ATOM	3341	OE2	GLU	A	419	0.949	6.188	-18.984	1.00	96.25	O
ATOM	3342	N	GLY	A	420	0.905	4.715	-15.062	1.00	96.38	N
ATOM	3343	CA	GLY	A	420	0.613	5.082	-13.688	1.00	96.38	C
ATOM	3344	C	GLY	A	420	-0.312	6.277	-13.570	1.00	96.38	C
ATOM	3345	O	GLY	A	420	-0.042	7.336	-14.148	1.00	96.38	O
ATOM	3346	N	VAL	A	421	-1.436	6.121	-12.922	1.00	95.44	N
ATOM	3347	CA	VAL	A	421	-2.424	7.176	-12.727	1.00	95.44	C
ATOM	3348	C	VAL	A	421	-2.520	7.523	-11.242	1.00	95.44	C
ATOM	3349	CB	VAL	A	421	-3.811	6.762	-13.266	1.00	95.44	C
ATOM	3350	O	VAL	A	421	-2.648	6.633	-10.398	1.00	95.44	O
ATOM	3351	CG1	VAL	A	421	-4.812	7.902	-13.109	1.00	95.44	C
ATOM	3352	CG2	VAL	A	421	-3.707	6.336	-14.734	1.00	95.44	C
ATOM	3353	N	PHE	A	422	-2.492	8.875	-10.914	1.00	93.31	N
ATOM	3354	CA	PHE	A	422	-2.492	9.328	-9.531	1.00	93.31	C
ATOM	3355	C	PHE	A	422	-3.643	10.289	-9.273	1.00	93.31	C
ATOM	3356	CB	PHE	A	422	-1.158	9.992	-9.180	1.00	93.31	C
ATOM	3357	O	PHE	A	422	-3.990	11.094	-10.141	1.00	93.31	O
ATOM	3358	CG	PHE	A	422	0.043	9.133	-9.461	1.00	93.31	C
ATOM	3359	CD1	PHE	A	422	0.547	8.273	-8.484	1.00	93.31	C
ATOM	3360	CD2	PHE	A	422	0.668	9.180	-10.695	1.00	93.31	C
ATOM	3361	CE1	PHE	A	422	1.658	7.477	-8.750	1.00	93.31	C
ATOM	3362	CE2	PHE	A	422	1.779	8.383	-10.969	1.00	93.31	C
ATOM	3363	CZ	PHE	A	422	2.273	7.531	-9.992	1.00	93.31	C
ATOM	3364	N	GLU	A	423	-4.270	10.133	-8.070	1.00	91.31	N
ATOM	3365	CA	GLU	A	423	-5.211	11.125	-7.543	1.00	91.31	C
ATOM	3366	C	GLU	A	423	-4.617	11.883	-6.359	1.00	91.31	C
ATOM	3367	CB	GLU	A	423	-6.523	10.445	-7.129	1.00	91.31	C
ATOM	3368	O	GLU	A	423	-4.598	11.375	-5.238	1.00	91.31	O
ATOM	3369	CG	GLU	A	423	-7.566	11.414	-6.590	1.00	91.31	C
ATOM	3370	CD	GLU	A	423	-7.914	12.531	-7.562	1.00	91.31	C
ATOM	3371	OE1	GLU	A	423	-7.750	13.719	-7.203	1.00	91.31	O
ATOM	3372	OE2	GLU	A	423	-8.344	12.211	-8.688	1.00	91.31	O
ATOM	3373	N	THR	A	424	-4.152	13.039	-6.594	1.00	88.88	N
ATOM	3374	CA	THR	A	424	-3.350	13.812	-5.656	1.00	88.88	C
ATOM	3375	C	THR	A	424	-4.172	14.188	-4.426	1.00	88.88	C
ATOM	3376	CB	THR	A	424	-2.795	15.086	-6.316	1.00	88.88	C
ATOM	3377	O	THR	A	424	-3.672	14.148	-3.301	1.00	88.88	O
ATOM	3378	CG2	THR	A	424	-1.745	14.750	-7.367	1.00	88.88	C
ATOM	3379	OG1	THR	A	424	-3.869	15.805	-6.938	1.00	88.88	O
ATOM	3380	N	GLU	A	425	-5.430	14.648	-4.656	1.00	84.69	N
ATOM	3381	CA	GLU	A	425	-6.285	15.039	-3.539	1.00	84.69	C
ATOM	3382	C	GLU	A	425	-6.516	13.867	-2.588	1.00	84.69	C
ATOM	3383	CB	GLU	A	425	-7.625	15.570	-4.047	1.00	84.69	C
ATOM	3384	O	GLU	A	425	-6.457	14.031	-1.367	1.00	84.69	O
ATOM	3385	CG	GLU	A	425	-8.508	16.156	-2.957	1.00	84.69	C
ATOM	3386	CD	GLU	A	425	-9.750	16.859	-3.496	1.00	84.69	C
ATOM	3387	OE1	GLU	A	425	-10.516	17.438	-2.695	1.00	84.69	O
ATOM	3388	OE2	GLU	A	425	-9.961	16.812	-4.730	1.00	84.69	O

ATOM	3389	N	ALA	A	426	-6.758	12.750	-3.123	1.00	87.62	N
ATOM	3390	CA	ALA	A	426	-6.961	11.555	-2.314	1.00	87.62	C
ATOM	3391	C	ALA	A	426	-5.664	11.117	-1.641	1.00	87.62	C
ATOM	3392	CB	ALA	A	426	-7.516	10.422	-3.176	1.00	87.62	C
ATOM	3393	O	ALA	A	426	-5.672	10.672	-0.492	1.00	87.62	O
ATOM	3394	N	ALA	A	427	-4.559	11.258	-2.289	1.00	90.94	N
ATOM	3395	CA	ALA	A	427	-3.254	10.898	-1.741	1.00	90.94	C
ATOM	3396	C	ALA	A	427	-2.906	11.766	-0.535	1.00	90.94	C
ATOM	3397	CB	ALA	A	427	-2.174	11.023	-2.812	1.00	90.94	C
ATOM	3398	O	ALA	A	427	-2.344	11.273	0.448	1.00	90.94	O
ATOM	3399	N	LEU	A	428	-3.250	13.062	-0.602	1.00	90.44	N
ATOM	3400	CA	LEU	A	428	-2.994	13.977	0.508	1.00	90.44	C
ATOM	3401	C	LEU	A	428	-3.799	13.570	1.739	1.00	90.44	C
ATOM	3402	CB	LEU	A	428	-3.334	15.414	0.109	1.00	90.44	C
ATOM	3403	O	LEU	A	428	-3.307	13.664	2.865	1.00	90.44	O
ATOM	3404	CG	LEU	A	428	-2.340	16.109	-0.822	1.00	90.44	C
ATOM	3405	CD1	LEU	A	428	-2.928	17.422	-1.337	1.00	90.44	C
ATOM	3406	CD2	LEU	A	428	-1.018	16.359	-0.105	1.00	90.44	C
ATOM	3407	N	ARG	A	429	-4.961	13.086	1.487	1.00	87.25	N
ATOM	3408	CA	ARG	A	429	-5.871	12.734	2.570	1.00	87.25	C
ATOM	3409	C	ARG	A	429	-5.559	11.344	3.113	1.00	87.25	C
ATOM	3410	CB	ARG	A	429	-7.324	12.789	2.094	1.00	87.25	C
ATOM	3411	O	ARG	A	429	-5.516	11.141	4.328	1.00	87.25	O
ATOM	3412	CG	ARG	A	429	-8.344	12.492	3.182	1.00	87.25	C
ATOM	3413	CD	ARG	A	429	-9.766	12.727	2.701	1.00	87.25	C
ATOM	3414	NE	ARG	A	429	-10.750	12.359	3.719	1.00	87.25	N
ATOM	3415	NH1	ARG	A	429	-12.578	13.094	2.516	1.00	87.25	N
ATOM	3416	NH2	ARG	A	429	-12.859	12.172	4.598	1.00	87.25	N
ATOM	3417	CZ	ARG	A	429	-12.062	12.539	3.607	1.00	87.25	C
ATOM	3418	N	PHE	A	430	-5.312	10.336	2.240	1.00	89.88	N
ATOM	3419	CA	PHE	A	430	-5.273	8.938	2.666	1.00	89.88	C
ATOM	3420	C	PHE	A	430	-3.861	8.375	2.553	1.00	89.88	C
ATOM	3421	CB	PHE	A	430	-6.246	8.094	1.836	1.00	89.88	C
ATOM	3422	O	PHE	A	430	-3.602	7.250	2.971	1.00	89.88	O
ATOM	3423	CG	PHE	A	430	-7.672	8.570	1.894	1.00	89.88	C
ATOM	3424	CD1	PHE	A	430	-8.375	8.562	3.092	1.00	89.88	C
ATOM	3425	CD2	PHE	A	430	-8.305	9.031	0.749	1.00	89.88	C
ATOM	3426	CE1	PHE	A	430	-9.695	9.008	3.148	1.00	89.88	C
ATOM	3427	CE2	PHE	A	430	-9.625	9.477	0.798	1.00	89.88	C
ATOM	3428	CZ	PHE	A	430	-10.320	9.461	1.998	1.00	89.88	C
ATOM	3429	N	GLY	A	431	-2.936	9.109	2.109	1.00	90.56	N
ATOM	3430	CA	GLY	A	431	-1.613	8.578	1.829	1.00	90.56	C
ATOM	3431	C	GLY	A	431	-1.546	7.809	0.521	1.00	90.56	C
ATOM	3432	O	GLY	A	431	-2.400	7.980	-0.349	1.00	90.56	O
ATOM	3433	N	TYR	A	432	-0.447	6.945	0.386	1.00	91.31	N
ATOM	3434	CA	TYR	A	432	-0.199	6.258	-0.878	1.00	91.31	C
ATOM	3435	C	TYR	A	432	-0.683	4.812	-0.819	1.00	91.31	C
ATOM	3436	CB	TYR	A	432	1.293	6.293	-1.226	1.00	91.31	C
ATOM	3437	O	TYR	A	432	-0.090	3.980	-0.130	1.00	91.31	O
ATOM	3438	CG	TYR	A	432	1.806	7.676	-1.555	1.00	91.31	C
ATOM	3439	CD1	TYR	A	432	1.665	8.203	-2.836	1.00	91.31	C
ATOM	3440	CD2	TYR	A	432	2.432	8.453	-0.586	1.00	91.31	C
ATOM	3441	CE1	TYR	A	432	2.139	9.477	-3.145	1.00	91.31	C
ATOM	3442	CE2	TYR	A	432	2.908	9.727	-0.884	1.00	91.31	C
ATOM	3443	OH	TYR	A	432	3.227	11.484	-2.463	1.00	91.31	O
ATOM	3444	CZ	TYR	A	432	2.756	10.227	-2.164	1.00	91.31	C
ATOM	3445	N	ASN	A	433	-1.822	4.512	-1.436	1.00	93.31	N
ATOM	3446	CA	ASN	A	433	-2.400	3.184	-1.602	1.00	93.31	C
ATOM	3447	C	ASN	A	433	-2.594	2.834	-3.074	1.00	93.31	C
ATOM	3448	CB	ASN	A	433	-3.732	3.080	-0.854	1.00	93.31	C
ATOM	3449	O	ASN	A	433	-3.385	3.473	-3.770	1.00	93.31	O
ATOM	3450	CG	ASN	A	433	-3.572	3.225	0.647	1.00	93.31	C
ATOM	3451	ND2	ASN	A	433	-4.621	3.697	1.312	1.00	93.31	N
ATOM	3452	OD1	ASN	A	433	-2.516	2.916	1.203	1.00	93.31	O

ATOM	3453	N	SER	A	434	-1.911	1.740	-3.621	1.00	94.44	N
ATOM	3454	CA	SER	A	434	-1.860	1.451	-5.051	1.00	94.44	C
ATOM	3455	C	SER	A	434	-2.729	0.247	-5.402	1.00	94.44	C
ATOM	3456	CB	SER	A	434	-0.419	1.197	-5.496	1.00	94.44	C
ATOM	3457	O	SER	A	434	-2.908	-0.656	-4.582	1.00	94.44	O
ATOM	3458	OG	SER	A	434	-0.354	0.995	-6.895	1.00	94.44	O
ATOM	3459	N	THR	A	435	-3.318	0.278	-6.617	1.00	95.44	N
ATOM	3460	CA	THR	A	435	-3.996	-0.846	-7.254	1.00	95.44	C
ATOM	3461	C	THR	A	435	-3.562	-0.986	-8.711	1.00	95.44	C
ATOM	3462	CB	THR	A	435	-5.527	-0.687	-7.184	1.00	95.44	C
ATOM	3463	O	THR	A	435	-3.021	-0.045	-9.297	1.00	95.44	O
ATOM	3464	CG2	THR	A	435	-6.000	0.457	-8.070	1.00	95.44	C
ATOM	3465	OG1	THR	A	435	-6.148	-1.903	-7.617	1.00	95.44	O
ATOM	3466	N	SER	A	436	-3.688	-2.256	-9.250	1.00	96.88	N
ATOM	3467	CA	SER	A	436	-3.260	-2.490	-10.625	1.00	96.88	C
ATOM	3468	C	SER	A	436	-4.340	-3.211	-11.430	1.00	96.88	C
ATOM	3469	CB	SER	A	436	-1.965	-3.305	-10.656	1.00	96.88	C
ATOM	3470	O	SER	A	436	-5.043	-4.070	-10.891	1.00	96.88	O
ATOM	3471	OG	SER	A	436	-0.926	-2.619	-9.977	1.00	96.88	O
ATOM	3472	N	ILE	A	437	-4.535	-2.742	-12.719	1.00	95.44	N
ATOM	3473	CA	ILE	A	437	-5.434	-3.438	-13.641	1.00	95.44	C
ATOM	3474	C	ILE	A	437	-4.746	-3.613	-14.992	1.00	95.44	C
ATOM	3475	CB	ILE	A	437	-6.766	-2.676	-13.805	1.00	95.44	C
ATOM	3476	O	ILE	A	437	-3.795	-2.896	-15.312	1.00	95.44	O
ATOM	3477	CG1	ILE	A	437	-6.523	-1.306	-14.453	1.00	95.44	C
ATOM	3478	CG2	ILE	A	437	-7.480	-2.531	-12.461	1.00	95.44	C
ATOM	3479	CD1	ILE	A	437	-7.785	-0.634	-14.977	1.00	95.44	C
ATOM	3480	N	THR	A	438	-5.188	-4.727	-15.695	1.00	95.12	N
ATOM	3481	CA	THR	A	438	-4.840	-4.930	-17.094	1.00	95.12	C
ATOM	3482	C	THR	A	438	-5.992	-4.512	-18.000	1.00	95.12	C
ATOM	3483	CB	THR	A	438	-4.473	-6.398	-17.375	1.00	95.12	C
ATOM	3484	O	THR	A	438	-7.070	-5.105	-17.969	1.00	95.12	O
ATOM	3485	CG2	THR	A	438	-4.102	-6.605	-18.844	1.00	95.12	C
ATOM	3486	OG1	THR	A	438	-3.357	-6.770	-16.547	1.00	95.12	O
ATOM	3487	N	ALA	A	439	-5.727	-3.447	-18.750	1.00	95.44	N
ATOM	3488	CA	ALA	A	439	-6.750	-2.932	-19.656	1.00	95.44	C
ATOM	3489	C	ALA	A	439	-6.844	-3.779	-20.922	1.00	95.44	C
ATOM	3490	CB	ALA	A	439	-6.457	-1.476	-20.016	1.00	95.44	C
ATOM	3491	O	ALA	A	439	-6.039	-3.621	-21.844	1.00	95.44	O
ATOM	3492	N	ILE	A	440	-7.812	-4.652	-20.938	1.00	93.31	N
ATOM	3493	CA	ILE	A	440	-8.008	-5.578	-22.047	1.00	93.31	C
ATOM	3494	C	ILE	A	440	-8.328	-4.793	-23.328	1.00	93.31	C
ATOM	3495	CB	ILE	A	440	-9.125	-6.594	-21.750	1.00	93.31	C
ATOM	3496	O	ILE	A	440	-9.164	-3.889	-23.312	1.00	93.31	O
ATOM	3497	CG1	ILE	A	440	-8.758	-7.457	-20.547	1.00	93.31	C
ATOM	3498	CG2	ILE	A	440	-9.406	-7.469	-22.984	1.00	93.31	C
ATOM	3499	CD1	ILE	A	440	-9.883	-8.359	-20.047	1.00	93.31	C
ATOM	3500	N	GLY	A	441	-7.727	-5.145	-24.469	1.00	92.94	N
ATOM	3501	CA	GLY	A	441	-7.973	-4.566	-25.781	1.00	92.94	C
ATOM	3502	C	GLY	A	441	-7.535	-3.121	-25.891	1.00	92.94	C
ATOM	3503	O	GLY	A	441	-7.891	-2.428	-26.844	1.00	92.94	O
ATOM	3504	N	CYS	A	442	-6.762	-2.572	-24.906	1.00	95.44	N
ATOM	3505	CA	CYS	A	442	-6.336	-1.177	-24.875	1.00	95.44	C
ATOM	3506	C	CYS	A	442	-4.957	-1.020	-25.516	1.00	95.44	C
ATOM	3507	CB	CYS	A	442	-6.316	-0.645	-23.453	1.00	95.44	C
ATOM	3508	O	CYS	A	442	-3.992	-1.646	-25.062	1.00	95.44	O
ATOM	3509	SG	CYS	A	442	-5.789	1.078	-23.328	1.00	95.44	S
ATOM	3510	N	THR	A	443	-4.766	-0.258	-26.531	1.00	94.81	N
ATOM	3511	CA	THR	A	443	-3.504	-0.064	-27.234	1.00	94.81	C
ATOM	3512	C	THR	A	443	-3.059	1.394	-27.156	1.00	94.81	C
ATOM	3513	CB	THR	A	443	-3.615	-0.491	-28.719	1.00	94.81	C
ATOM	3514	O	THR	A	443	-1.987	1.749	-27.656	1.00	94.81	O
ATOM	3515	CG2	THR	A	443	-3.852	-1.993	-28.828	1.00	94.81	C
ATOM	3516	OG1	THR	A	443	-4.707	0.205	-29.328	1.00	94.81	O

ATOM	3517	N	ARG	A	444	-3.941	2.213	-26.516	1.00	94.06	N
ATOM	3518	CA	ARG	A	444	-3.629	3.633	-26.391	1.00	94.06	C
ATOM	3519	C	ARG	A	444	-3.359	4.012	-24.938	1.00	94.06	C
ATOM	3520	CB	ARG	A	444	-4.770	4.484	-26.953	1.00	94.06	C
ATOM	3521	O	ARG	A	444	-4.102	3.615	-24.031	1.00	94.06	O
ATOM	3522	CG	ARG	A	444	-5.066	4.230	-28.422	1.00	94.06	C
ATOM	3523	CD	ARG	A	444	-6.258	5.043	-28.906	1.00	94.06	C
ATOM	3524	NE	ARG	A	444	-7.488	4.656	-28.219	1.00	94.06	N
ATOM	3525	NH1	ARG	A	444	-8.766	6.359	-29.125	1.00	94.06	N
ATOM	3526	NH2	ARG	A	444	-9.711	4.852	-27.672	1.00	94.06	N
ATOM	3527	CZ	ARG	A	444	-8.648	5.289	-28.344	1.00	94.06	C
ATOM	3528	N	ARG	A	445	-2.293	4.770	-24.734	1.00	94.56	N
ATOM	3529	CA	ARG	A	445	-1.903	5.188	-23.391	1.00	94.56	C
ATOM	3530	C	ARG	A	445	-2.955	6.105	-22.781	1.00	94.56	C
ATOM	3531	CB	ARG	A	445	-0.545	5.891	-23.422	1.00	94.56	C
ATOM	3532	O	ARG	A	445	-3.248	6.008	-21.578	1.00	94.56	O
ATOM	3533	CG	ARG	A	445	-0.014	6.262	-22.047	1.00	94.56	C
ATOM	3534	CD	ARG	A	445	1.379	6.871	-22.125	1.00	94.56	C
ATOM	3535	NE	ARG	A	445	2.391	5.867	-22.438	1.00	94.56	N
ATOM	3536	NH1	ARG	A	445	4.156	7.352	-22.469	1.00	94.56	N
ATOM	3537	NH2	ARG	A	445	4.520	5.129	-22.875	1.00	94.56	N
ATOM	3538	CZ	ARG	A	445	3.688	6.117	-22.594	1.00	94.56	C
ATOM	3539	N	SER	A	446	-3.502	7.031	-23.516	1.00	94.25	N
ATOM	3540	CA	SER	A	446	-4.508	7.984	-23.047	1.00	94.25	C
ATOM	3541	C	SER	A	446	-5.762	7.266	-22.562	1.00	94.25	C
ATOM	3542	CB	SER	A	446	-4.879	8.961	-24.172	1.00	94.25	C
ATOM	3543	O	SER	A	446	-6.348	7.656	-21.547	1.00	94.25	O
ATOM	3544	OG	SER	A	446	-5.324	8.258	-25.312	1.00	94.25	O
ATOM	3545	N	GLU	A	447	-6.227	6.254	-23.297	1.00	95.00	N
ATOM	3546	CA	GLU	A	447	-7.367	5.453	-22.859	1.00	95.00	C
ATOM	3547	C	GLU	A	447	-7.059	4.719	-21.562	1.00	95.00	C
ATOM	3548	CB	GLU	A	447	-7.770	4.453	-23.938	1.00	95.00	C
ATOM	3549	O	GLU	A	447	-7.910	4.645	-20.656	1.00	95.00	O
ATOM	3550	CG	GLU	A	447	-8.961	3.584	-23.562	1.00	95.00	C
ATOM	3551	CD	GLU	A	447	-9.375	2.621	-24.672	1.00	95.00	C
ATOM	3552	OE1	GLU	A	447	-10.055	1.614	-24.375	1.00	95.00	O
ATOM	3553	OE2	GLU	A	447	-9.008	2.873	-25.844	1.00	95.00	O
ATOM	3554	N	ALA	A	448	-5.746	4.137	-21.406	1.00	95.62	N
ATOM	3555	CA	ALA	A	448	-5.297	3.488	-20.188	1.00	95.62	C
ATOM	3556	C	ALA	A	448	-5.348	4.453	-19.000	1.00	95.62	C
ATOM	3557	CB	ALA	A	448	-3.883	2.938	-20.359	1.00	95.62	C
ATOM	3558	O	ALA	A	448	-5.750	4.074	-17.906	1.00	95.62	O
ATOM	3559	N	ASN	A	449	-4.953	5.684	-19.188	1.00	94.88	N
ATOM	3560	CA	ASN	A	449	-5.008	6.723	-18.172	1.00	94.88	C
ATOM	3561	C	ASN	A	449	-6.441	6.984	-17.719	1.00	94.88	C
ATOM	3562	CB	ASN	A	449	-4.371	8.016	-18.688	1.00	94.88	C
ATOM	3563	O	ASN	A	449	-6.707	7.086	-16.516	1.00	94.88	O
ATOM	3564	CG	ASN	A	449	-4.289	9.094	-17.625	1.00	94.88	C
ATOM	3565	ND2	ASN	A	449	-3.188	9.109	-16.875	1.00	94.88	N
ATOM	3566	OD1	ASN	A	449	-5.211	9.898	-17.453	1.00	94.88	O
ATOM	3567	N	ARG	A	450	-7.418	7.102	-18.656	1.00	94.88	N
ATOM	3568	CA	ARG	A	450	-8.820	7.336	-18.328	1.00	94.88	C
ATOM	3569	C	ARG	A	450	-9.398	6.168	-17.531	1.00	94.88	C
ATOM	3570	CB	ARG	A	450	-9.641	7.566	-19.594	1.00	94.88	C
ATOM	3571	O	ARG	A	450	-10.180	6.367	-16.609	1.00	94.88	O
ATOM	3572	CG	ARG	A	450	-9.414	8.922	-20.234	1.00	94.88	C
ATOM	3573	CD	ARG	A	450	-10.352	9.156	-21.406	1.00	94.88	C
ATOM	3574	NE	ARG	A	450	-10.047	8.273	-22.531	1.00	94.88	N
ATOM	3575	NH1	ARG	A	450	-12.234	8.078	-23.250	1.00	94.88	N
ATOM	3576	NH2	ARG	A	450	-10.555	6.984	-24.359	1.00	94.88	N
ATOM	3577	CZ	ARG	A	450	-10.945	7.781	-23.375	1.00	94.88	C
ATOM	3578	N	ARG	A	451	-8.977	4.941	-17.859	1.00	94.69	N
ATOM	3579	CA	ARG	A	451	-9.430	3.771	-17.125	1.00	94.69	C
ATOM	3580	C	ARG	A	451	-8.914	3.805	-15.680	1.00	94.69	C

```
ATOM   3581  CB  ARG A 451      -8.977   2.486 -17.812  1.00 94.69           C
ATOM   3582  O   ARG A 451      -9.648   3.477 -14.750  1.00 94.69           O
ATOM   3583  CG  ARG A 451      -9.680   2.211 -19.125  1.00 94.69           C
ATOM   3584  CD  ARG A 451      -9.383   0.812 -19.641  1.00 94.69           C
ATOM   3585  NE  ARG A 451     -10.039   0.562 -20.922  1.00 94.69           N
ATOM   3586  NH1 ARG A 451     -10.094  -1.735 -20.688  1.00 94.69           N
ATOM   3587  NH2 ARG A 451     -10.953  -0.756 -22.578  1.00 94.69           N
ATOM   3588  CZ  ARG A 451     -10.359  -0.643 -21.391  1.00 94.69           C
ATOM   3589  N   GLY A 452      -7.621   4.191 -15.508  1.00 95.38           N
ATOM   3590  CA  GLY A 452      -7.055   4.348 -14.180  1.00 95.38           C
ATOM   3591  C   GLY A 452      -7.750   5.414 -13.359  1.00 95.38           C
ATOM   3592  O   GLY A 452      -8.047   5.203 -12.180  1.00 95.38           O
ATOM   3593  N   ARG A 453      -8.031   6.539 -13.969  1.00 93.94           N
ATOM   3594  CA  ARG A 453      -8.727   7.637 -13.297  1.00 93.94           C
ATOM   3595  C   ARG A 453     -10.133   7.227 -12.891  1.00 93.94           C
ATOM   3596  CB  ARG A 453      -8.781   8.867 -14.211  1.00 93.94           C
ATOM   3597  O   ARG A 453     -10.602   7.594 -11.812  1.00 93.94           O
ATOM   3598  CG  ARG A 453      -7.457   9.609 -14.320  1.00 93.94           C
ATOM   3599  CD  ARG A 453      -7.594  10.875 -15.156  1.00 93.94           C
ATOM   3600  NE  ARG A 453      -6.320  11.586 -15.266  1.00 93.94           N
ATOM   3601  NH1 ARG A 453      -7.168  13.328 -16.516  1.00 93.94           N
ATOM   3602  NH2 ARG A 453      -4.949  13.297 -15.953  1.00 93.94           N
ATOM   3603  CZ  ARG A 453      -6.148  12.734 -15.914  1.00 93.94           C
ATOM   3604  N   TRP A 454     -10.727   6.445 -13.758  1.00 93.62           N
ATOM   3605  CA  TRP A 454     -12.070   5.961 -13.453  1.00 93.62           C
ATOM   3606  C   TRP A 454     -12.070   5.109 -12.188  1.00 93.62           C
ATOM   3607  CB  TRP A 454     -12.625   5.152 -14.633  1.00 93.62           C
ATOM   3608  O   TRP A 454     -12.930   5.281 -11.320  1.00 93.62           O
ATOM   3609  CG  TRP A 454     -13.930   4.469 -14.344  1.00 93.62           C
ATOM   3610  CD1 TRP A 454     -15.164   5.051 -14.250  1.00 93.62           C
ATOM   3611  CD2 TRP A 454     -14.125   3.072 -14.094  1.00 93.62           C
ATOM   3612  CE2 TRP A 454     -15.500   2.877 -13.867  1.00 93.62           C
ATOM   3613  CE3 TRP A 454     -13.266   1.964 -14.055  1.00 93.62           C
ATOM   3614  NE1 TRP A 454     -16.109   4.098 -13.961  1.00 93.62           N
ATOM   3615  CH2 TRP A 454     -15.188   0.553 -13.555  1.00 93.62           C
ATOM   3616  CZ2 TRP A 454     -16.047   1.619 -13.594  1.00 93.62           C
ATOM   3617  CZ3 TRP A 454     -13.805   0.713 -13.781  1.00 93.62           C
ATOM   3618  N   ILE A 455     -11.172   4.266 -12.047  1.00 93.38           N
ATOM   3619  CA  ILE A 455     -11.070   3.391 -10.883  1.00 93.38           C
ATOM   3620  C   ILE A 455     -10.844   4.230  -9.625  1.00 93.38           C
ATOM   3621  CB  ILE A 455      -9.930   2.357 -11.047  1.00 93.38           C
ATOM   3622  O   ILE A 455     -11.492   4.004  -8.594  1.00 93.38           O
ATOM   3623  CG1 ILE A 455     -10.258   1.385 -12.188  1.00 93.38           C
ATOM   3624  CG2 ILE A 455      -9.688   1.606  -9.734  1.00 93.38           C
ATOM   3625  CD1 ILE A 455     -11.469   0.505 -11.922  1.00 93.38           C
ATOM   3626  N   LEU A 456      -9.961   5.172  -9.703  1.00 93.06           N
ATOM   3627  CA  LEU A 456      -9.602   5.992  -8.555  1.00 93.06           C
ATOM   3628  C   LEU A 456     -10.758   6.902  -8.156  1.00 93.06           C
ATOM   3629  CB  LEU A 456      -8.359   6.832  -8.859  1.00 93.06           C
ATOM   3630  O   LEU A 456     -11.078   7.027  -6.969  1.00 93.06           O
ATOM   3631  CG  LEU A 456      -7.059   6.062  -9.078  1.00 93.06           C
ATOM   3632  CD1 LEU A 456      -5.926   7.023  -9.422  1.00 93.06           C
ATOM   3633  CD2 LEU A 456      -6.715   5.238  -7.844  1.00 93.06           C
ATOM   3634  N   LYS A 457     -11.445   7.508  -9.141  1.00 91.38           N
ATOM   3635  CA  LYS A 457     -12.523   8.453  -8.852  1.00 91.38           C
ATOM   3636  C   LYS A 457     -13.758   7.727  -8.336  1.00 91.38           C
ATOM   3637  CB  LYS A 457     -12.875   9.266 -10.102  1.00 91.38           C
ATOM   3638  O   LYS A 457     -14.477   8.242  -7.477  1.00 91.38           O
ATOM   3639  CG  LYS A 457     -11.797  10.242 -10.531  1.00 91.38           C
ATOM   3640  CD  LYS A 457     -11.664  11.398  -9.547  1.00 91.38           C
ATOM   3641  CE  LYS A 457     -10.656  12.430 -10.031  1.00 91.38           C
ATOM   3642  NZ  LYS A 457     -10.438  13.508  -9.016  1.00 91.38           N
ATOM   3643  N   THR A 458     -13.961   6.570  -8.867  1.00 90.56           N
ATOM   3644  CA  THR A 458     -15.070   5.754  -8.375  1.00 90.56           C
```

ATOM	3645	C	THR	A	458	-14.797	5.270	-6.957	1.00	90.56	C
ATOM	3646	CB	THR	A	458	-15.312	4.543	-9.297	1.00	90.56	C
ATOM	3647	O	THR	A	458	-15.711	5.219	-6.125	1.00	90.56	O
ATOM	3648	CG2	THR	A	458	-16.469	3.693	-8.789	1.00	90.56	C
ATOM	3649	OG1	THR	A	458	-15.625	5.008	-10.617	1.00	90.56	O
ATOM	3650	N	ASN	A	459	-13.547	5.016	-6.633	1.00	89.81	N
ATOM	3651	CA	ASN	A	459	-13.125	4.496	-5.340	1.00	89.81	C
ATOM	3652	C	ASN	A	459	-13.297	5.531	-4.230	1.00	89.81	C
ATOM	3653	CB	ASN	A	459	-11.664	4.027	-5.398	1.00	89.81	C
ATOM	3654	O	ASN	A	459	-13.688	5.188	-3.111	1.00	89.81	O
ATOM	3655	CG	ASN	A	459	-11.195	3.420	-4.094	1.00	89.81	C
ATOM	3656	ND2	ASN	A	459	-11.719	2.248	-3.758	1.00	89.81	N
ATOM	3657	OD1	ASN	A	459	-10.359	3.996	-3.391	1.00	89.81	O
ATOM	3658	N	VAL	A	460	-13.039	6.816	-4.566	1.00	86.56	N
ATOM	3659	CA	VAL	A	460	-13.094	7.879	-3.570	1.00	86.56	C
ATOM	3660	C	VAL	A	460	-14.539	8.086	-3.115	1.00	86.56	C
ATOM	3661	CB	VAL	A	460	-12.508	9.203	-4.121	1.00	86.56	C
ATOM	3662	O	VAL	A	460	-14.781	8.555	-2.000	1.00	86.56	O
ATOM	3663	CG1	VAL	A	460	-12.695	10.336	-3.109	1.00	86.56	C
ATOM	3664	CG2	VAL	A	460	-11.031	9.031	-4.469	1.00	86.56	C
ATOM	3665	N	LYS	A	461	-15.469	7.660	-3.959	1.00	86.75	N
ATOM	3666	CA	LYS	A	461	-16.891	7.777	-3.645	1.00	86.75	C
ATOM	3667	C	LYS	A	461	-17.484	6.418	-3.295	1.00	86.75	C
ATOM	3668	CB	LYS	A	461	-17.641	8.398	-4.816	1.00	86.75	C
ATOM	3669	O	LYS	A	461	-18.344	5.906	-4.023	1.00	86.75	O
ATOM	3670	CG	LYS	A	461	-17.094	9.742	-5.266	1.00	86.75	C
ATOM	3671	CD	LYS	A	461	-17.188	10.789	-4.156	1.00	86.75	C
ATOM	3672	CE	LYS	A	461	-16.656	12.141	-4.617	1.00	86.75	C
ATOM	3673	NZ	LYS	A	461	-16.578	13.117	-3.488	1.00	86.75	N
ATOM	3674	N	SER	A	462	-17.125	5.777	-2.096	1.00	86.25	N
ATOM	3675	CA	SER	A	462	-17.438	4.383	-1.788	1.00	86.25	C
ATOM	3676	C	SER	A	462	-18.406	4.270	-0.626	1.00	86.25	C
ATOM	3677	CB	SER	A	462	-16.141	3.619	-1.458	1.00	86.25	C
ATOM	3678	O	SER	A	462	-18.609	3.184	-0.072	1.00	86.25	O
ATOM	3679	OG	SER	A	462	-15.430	4.262	-0.417	1.00	86.25	O
ATOM	3680	N	THR	A	463	-18.938	5.402	-0.198	1.00	92.69	N
ATOM	3681	CA	THR	A	463	-19.906	5.348	0.892	1.00	92.69	C
ATOM	3682	C	THR	A	463	-21.141	4.535	0.485	1.00	92.69	C
ATOM	3683	CB	THR	A	463	-20.344	6.758	1.322	1.00	92.69	C
ATOM	3684	O	THR	A	463	-21.734	4.785	-0.562	1.00	92.69	O
ATOM	3685	CG2	THR	A	463	-21.297	6.703	2.512	1.00	92.69	C
ATOM	3686	OG1	THR	A	463	-19.188	7.520	1.688	1.00	92.69	O
ATOM	3687	N	THR	A	464	-21.438	3.529	1.304	1.00	95.06	N
ATOM	3688	CA	THR	A	464	-22.594	2.666	1.062	1.00	95.06	C
ATOM	3689	C	THR	A	464	-23.750	3.029	1.994	1.00	95.06	C
ATOM	3690	CB	THR	A	464	-22.234	1.182	1.247	1.00	95.06	C
ATOM	3691	O	THR	A	464	-23.531	3.260	3.188	1.00	95.06	O
ATOM	3692	CG2	THR	A	464	-23.422	0.282	0.975	1.00	95.06	C
ATOM	3693	OG1	THR	A	464	-21.172	0.839	0.341	1.00	95.06	O
ATOM	3694	N	VAL	A	465	-24.969	3.135	1.430	1.00	96.38	N
ATOM	3695	CA	VAL	A	465	-26.156	3.404	2.223	1.00	96.38	C
ATOM	3696	C	VAL	A	465	-27.094	2.203	2.164	1.00	96.38	C
ATOM	3697	CB	VAL	A	465	-26.891	4.676	1.735	1.00	96.38	C
ATOM	3698	O	VAL	A	465	-27.297	1.610	1.100	1.00	96.38	O
ATOM	3699	CG1	VAL	A	465	-27.375	4.504	0.298	1.00	96.38	C
ATOM	3700	CG2	VAL	A	465	-28.062	5.012	2.662	1.00	96.38	C
ATOM	3701	N	ASN	A	466	-27.562	1.800	3.363	1.00	95.94	N
ATOM	3702	CA	ASN	A	466	-28.547	0.730	3.502	1.00	95.94	C
ATOM	3703	C	ASN	A	466	-29.828	1.226	4.172	1.00	95.94	C
ATOM	3704	CB	ASN	A	466	-27.969	-0.442	4.289	1.00	95.94	C
ATOM	3705	O	ASN	A	466	-29.781	1.906	5.199	1.00	95.94	O
ATOM	3706	CG	ASN	A	466	-26.734	-1.038	3.619	1.00	95.94	C
ATOM	3707	ND2	ASN	A	466	-25.578	-0.841	4.223	1.00	95.94	N
ATOM	3708	OD1	ASN	A	466	-26.844	-1.671	2.564	1.00	95.94	O

ATOM	3709	N	PHE	A	467	-30.938	0.929	3.555	1.00	96.25	N
ATOM	3710	CA	PHE	A	467	-32.219	1.292	4.160	1.00	96.25	C
ATOM	3711	C	PHE	A	467	-33.344	0.386	3.656	1.00	96.25	C
ATOM	3712	CB	PHE	A	467	-32.562	2.756	3.867	1.00	96.25	C
ATOM	3713	O	PHE	A	467	-33.188	-0.295	2.641	1.00	96.25	O
ATOM	3714	CG	PHE	A	467	-32.719	3.059	2.402	1.00	96.25	C
ATOM	3715	CD1	PHE	A	467	-31.625	3.383	1.617	1.00	96.25	C
ATOM	3716	CD2	PHE	A	467	-33.969	3.018	1.812	1.00	96.25	C
ATOM	3717	CE1	PHE	A	467	-31.766	3.664	0.260	1.00	96.25	C
ATOM	3718	CE2	PHE	A	467	-34.125	3.297	0.457	1.00	96.25	C
ATOM	3719	CZ	PHE	A	467	-33.031	3.621	-0.317	1.00	96.25	C
ATOM	3720	N	ALA	A	468	-34.438	0.304	4.438	1.00	94.94	N
ATOM	3721	CA	ALA	A	468	-35.625	-0.451	4.082	1.00	94.94	C
ATOM	3722	C	ALA	A	468	-36.781	0.483	3.771	1.00	94.94	C
ATOM	3723	CB	ALA	A	468	-36.000	-1.412	5.207	1.00	94.94	C
ATOM	3724	O	ALA	A	468	-37.000	1.480	4.465	1.00	94.94	O
ATOM	3725	N	THR	A	469	-37.438	0.168	2.646	1.00	94.31	N
ATOM	3726	CA	THR	A	469	-38.594	0.970	2.234	1.00	94.31	C
ATOM	3727	C	THR	A	469	-39.781	0.078	1.899	1.00	94.31	C
ATOM	3728	CB	THR	A	469	-38.250	1.853	1.020	1.00	94.31	C
ATOM	3729	O	THR	A	469	-39.625	-1.147	1.847	1.00	94.31	O
ATOM	3730	CG2	THR	A	469	-38.000	1.005	-0.227	1.00	94.31	C
ATOM	3731	OG1	THR	A	469	-39.312	2.756	0.771	1.00	94.31	O
ATOM	3732	N	GLY	A	470	-40.938	0.685	1.839	1.00	92.94	N
ATOM	3733	CA	GLY	A	470	-42.156	-0.053	1.472	1.00	92.94	C
ATOM	3734	C	GLY	A	470	-42.188	-0.412	-0.001	1.00	92.94	C
ATOM	3735	O	GLY	A	470	-41.188	-0.772	-0.595	1.00	92.94	O
ATOM	3736	N	LEU	A	471	-43.312	-0.294	-0.641	1.00	89.88	N
ATOM	3737	CA	LEU	A	471	-43.562	-0.709	-2.018	1.00	89.88	C
ATOM	3738	C	LEU	A	471	-42.812	0.170	-2.996	1.00	89.88	C
ATOM	3739	CB	LEU	A	471	-45.062	-0.654	-2.334	1.00	89.88	C
ATOM	3740	O	LEU	A	471	-42.625	-0.204	-4.156	1.00	89.88	O
ATOM	3741	CG	LEU	A	471	-45.938	-1.726	-1.678	1.00	89.88	C
ATOM	3742	CD1	LEU	A	471	-47.406	-1.437	-1.931	1.00	89.88	C
ATOM	3743	CD2	LEU	A	471	-45.562	-3.111	-2.193	1.00	89.88	C
ATOM	3744	N	GLU	A	472	-42.344	1.262	-2.439	1.00	89.88	N
ATOM	3745	CA	GLU	A	472	-41.562	2.156	-3.293	1.00	89.88	C
ATOM	3746	C	GLU	A	472	-40.219	1.521	-3.693	1.00	89.88	C
ATOM	3747	CB	GLU	A	472	-41.312	3.492	-2.588	1.00	89.88	C
ATOM	3748	O	GLU	A	472	-39.594	1.940	-4.668	1.00	89.88	O
ATOM	3749	CG	GLU	A	472	-42.562	4.309	-2.355	1.00	89.88	C
ATOM	3750	CD	GLU	A	472	-43.250	4.016	-1.018	1.00	89.88	C
ATOM	3751	OE1	GLU	A	472	-44.188	4.734	-0.646	1.00	89.88	O
ATOM	3752	OE2	GLU	A	472	-42.781	3.064	-0.336	1.00	89.88	O
ATOM	3753	N	GLY	A	473	-39.750	0.540	-2.945	1.00	89.94	N
ATOM	3754	CA	GLY	A	473	-38.531	-0.182	-3.242	1.00	89.94	C
ATOM	3755	C	GLY	A	473	-38.562	-0.935	-4.559	1.00	89.94	C
ATOM	3756	O	GLY	A	473	-37.531	-1.355	-5.082	1.00	89.94	O
ATOM	3757	N	MET	A	474	-39.812	-1.055	-5.230	1.00	87.81	N
ATOM	3758	CA	MET	A	474	-40.000	-1.777	-6.488	1.00	87.81	C
ATOM	3759	C	MET	A	474	-39.719	-0.870	-7.680	1.00	87.81	C
ATOM	3760	CB	MET	A	474	-41.406	-2.348	-6.578	1.00	87.81	C
ATOM	3761	O	MET	A	474	-39.625	-1.344	-8.812	1.00	87.81	O
ATOM	3762	CG	MET	A	474	-41.688	-3.486	-5.609	1.00	87.81	C
ATOM	3763	SD	MET	A	474	-43.406	-4.070	-5.672	1.00	87.81	S
ATOM	3764	CE	MET	A	474	-43.406	-4.914	-7.277	1.00	87.81	C
ATOM	3765	N	ILE	A	475	-39.531	0.400	-7.484	1.00	89.50	N
ATOM	3766	CA	ILE	A	475	-39.406	1.365	-8.570	1.00	89.50	C
ATOM	3767	C	ILE	A	475	-37.969	1.354	-9.117	1.00	89.50	C
ATOM	3768	CB	ILE	A	475	-39.781	2.789	-8.109	1.00	89.50	C
ATOM	3769	O	ILE	A	475	-37.750	1.066	-10.289	1.00	89.50	O
ATOM	3770	CG1	ILE	A	475	-41.250	2.834	-7.688	1.00	89.50	C
ATOM	3771	CG2	ILE	A	475	-39.500	3.811	-9.211	1.00	89.50	C
ATOM	3772	CD1	ILE	A	475	-41.688	4.164	-7.086	1.00	89.50	C

ATOM	3773	N	PRO	A	476	-36.969	1.552	-8.117	1.00	89.94	N
ATOM	3774	CA	PRO	A	476	-35.625	1.496	-8.656	1.00	89.94	C
ATOM	3775	C	PRO	A	476	-35.125	0.068	-8.914	1.00	89.94	C
ATOM	3776	CB	PRO	A	476	-34.781	2.160	-7.562	1.00	89.94	C
ATOM	3777	O	PRO	A	476	-35.625	-0.870	-8.266	1.00	89.94	O
ATOM	3778	CG	PRO	A	476	-35.531	1.920	-6.301	1.00	89.94	C
ATOM	3779	CD	PRO	A	476	-37.000	1.873	-6.621	1.00	89.94	C
ATOM	3780	N	THR	A	477	-34.375	-0.110	-9.938	1.00	86.75	N
ATOM	3781	CA	THR	A	477	-33.688	-1.370	-10.211	1.00	86.75	C
ATOM	3782	C	THR	A	477	-32.188	-1.235	-9.953	1.00	86.75	C
ATOM	3783	CB	THR	A	477	-33.906	-1.836	-11.656	1.00	86.75	C
ATOM	3784	O	THR	A	477	-31.672	-0.128	-9.766	1.00	86.75	O
ATOM	3785	CG2	THR	A	477	-35.406	-2.029	-11.930	1.00	86.75	C
ATOM	3786	OG1	THR	A	477	-33.406	-0.853	-12.562	1.00	86.75	O
ATOM	3787	N	VAL	A	478	-31.516	-2.404	-9.773	1.00	84.88	N
ATOM	3788	CA	VAL	A	478	-30.062	-2.396	-9.586	1.00	84.88	C
ATOM	3789	C	VAL	A	478	-29.406	-1.643	-10.734	1.00	84.88	C
ATOM	3790	CB	VAL	A	478	-29.500	-3.830	-9.484	1.00	84.88	C
ATOM	3791	O	VAL	A	478	-29.734	-1.847	-11.898	1.00	84.88	O
ATOM	3792	CG1	VAL	A	478	-27.969	-3.807	-9.469	1.00	84.88	C
ATOM	3793	CG2	VAL	A	478	-30.031	-4.535	-8.250	1.00	84.88	C
ATOM	3794	N	GLY	A	479	-28.594	-0.606	-10.422	1.00	82.88	N
ATOM	3795	CA	GLY	A	479	-27.906	0.218	-11.406	1.00	82.88	C
ATOM	3796	C	GLY	A	479	-28.484	1.616	-11.516	1.00	82.88	C
ATOM	3797	O	GLY	A	479	-27.828	2.529	-12.016	1.00	82.88	O
ATOM	3798	N	ASP	A	480	-29.672	1.833	-11.023	1.00	89.00	N
ATOM	3799	CA	ASP	A	480	-30.328	3.139	-11.055	1.00	89.00	C
ATOM	3800	C	ASP	A	480	-29.703	4.090	-10.031	1.00	89.00	C
ATOM	3801	CB	ASP	A	480	-31.828	2.998	-10.805	1.00	89.00	C
ATOM	3802	O	ASP	A	480	-29.234	3.652	-8.977	1.00	89.00	O
ATOM	3803	CG	ASP	A	480	-32.594	2.461	-12.008	1.00	89.00	C
ATOM	3804	OD1	ASP	A	480	-31.984	2.336	-13.102	1.00	89.00	O
ATOM	3805	OD2	ASP	A	480	-33.781	2.160	-11.867	1.00	89.00	O
ATOM	3806	N	VAL	A	481	-29.656	5.363	-10.391	1.00	93.06	N
ATOM	3807	CA	VAL	A	481	-29.219	6.406	-9.469	1.00	93.06	C
ATOM	3808	C	VAL	A	481	-30.406	6.938	-8.688	1.00	93.06	C
ATOM	3809	CB	VAL	A	481	-28.516	7.562	-10.211	1.00	93.06	C
ATOM	3810	O	VAL	A	481	-31.453	7.273	-9.273	1.00	93.06	O
ATOM	3811	CG1	VAL	A	481	-28.125	8.672	-9.234	1.00	93.06	C
ATOM	3812	CG2	VAL	A	481	-27.281	7.047	-10.961	1.00	93.06	C
ATOM	3813	N	ILE	A	482	-30.297	6.918	-7.340	1.00	94.88	N
ATOM	3814	CA	ILE	A	482	-31.375	7.414	-6.484	1.00	94.88	C
ATOM	3815	C	ILE	A	482	-30.875	8.617	-5.680	1.00	94.88	C
ATOM	3816	CB	ILE	A	482	-31.891	6.312	-5.535	1.00	94.88	C
ATOM	3817	O	ILE	A	482	-29.672	8.773	-5.469	1.00	94.88	O
ATOM	3818	CG1	ILE	A	482	-30.766	5.805	-4.637	1.00	94.88	C
ATOM	3819	CG2	ILE	A	482	-32.531	5.168	-6.336	1.00	94.88	C
ATOM	3820	CD1	ILE	A	482	-31.234	4.898	-3.504	1.00	94.88	C
ATOM	3821	N	VAL	A	483	-31.812	9.383	-5.320	1.00	94.94	N
ATOM	3822	CA	VAL	A	483	-31.516	10.523	-4.461	1.00	94.94	C
ATOM	3823	C	VAL	A	483	-31.891	10.195	-3.018	1.00	94.94	C
ATOM	3824	CB	VAL	A	483	-32.250	11.789	-4.930	1.00	94.94	C
ATOM	3825	O	VAL	A	483	-33.000	9.711	-2.750	1.00	94.94	O
ATOM	3826	CG1	VAL	A	483	-32.062	12.938	-3.943	1.00	94.94	C
ATOM	3827	CG2	VAL	A	483	-31.797	12.195	-6.328	1.00	94.94	C
ATOM	3828	N	VAL	A	484	-30.922	10.367	-2.156	1.00	95.00	N
ATOM	3829	CA	VAL	A	484	-31.172	10.148	-0.735	1.00	95.00	C
ATOM	3830	C	VAL	A	484	-31.109	11.484	0.009	1.00	95.00	C
ATOM	3831	CB	VAL	A	484	-30.172	9.148	-0.124	1.00	95.00	C
ATOM	3832	O	VAL	A	484	-30.078	12.148	0.014	1.00	95.00	O
ATOM	3833	CG1	VAL	A	484	-30.359	9.055	1.390	1.00	95.00	C
ATOM	3834	CG2	VAL	A	484	-30.328	7.773	-0.772	1.00	95.00	C
ATOM	3835	N	SER	A	485	-32.219	11.867	0.506	1.00	94.31	N
ATOM	3836	CA	SER	A	485	-32.281	13.070	1.320	1.00	94.31	C

ATOM	3837	C	SER	A	485	-32.531	12.734	2.791	1.00	94.31	C
ATOM	3838	CB	SER	A	485	-33.406	13.977	0.822	1.00	94.31	C
ATOM	3839	O	SER	A	485	-33.656	12.562	3.229	1.00	94.31	O
ATOM	3840	OG	SER	A	485	-33.406	15.227	1.495	1.00	94.31	O
ATOM	3841	N	ASP	A	486	-31.453	12.586	3.521	1.00	94.25	N
ATOM	3842	CA	ASP	A	486	-31.453	12.227	4.938	1.00	94.25	C
ATOM	3843	C	ASP	A	486	-31.250	13.453	5.816	1.00	94.25	C
ATOM	3844	CB	ASP	A	486	-30.391	11.180	5.227	1.00	94.25	C
ATOM	3845	O	ASP	A	486	-30.141	14.000	5.875	1.00	94.25	O
ATOM	3846	CG	ASP	A	486	-30.406	10.695	6.664	1.00	94.25	C
ATOM	3847	OD1	ASP	A	486	-31.281	11.109	7.441	1.00	94.25	O
ATOM	3848	OD2	ASP	A	486	-29.516	9.891	7.027	1.00	94.25	O
ATOM	3849	N	ASN	A	487	-32.281	13.852	6.578	1.00	90.50	N
ATOM	3850	CA	ASN	A	487	-32.219	15.055	7.402	1.00	90.50	C
ATOM	3851	C	ASN	A	487	-31.266	14.875	8.586	1.00	90.50	C
ATOM	3852	CB	ASN	A	487	-33.594	15.438	7.895	1.00	90.50	C
ATOM	3853	O	ASN	A	487	-30.703	15.852	9.094	1.00	90.50	O
ATOM	3854	CG	ASN	A	487	-34.500	15.961	6.781	1.00	90.50	C
ATOM	3855	ND2	ASN	A	487	-35.812	15.914	6.996	1.00	90.50	N
ATOM	3856	OD1	ASN	A	487	-34.000	16.406	5.738	1.00	90.50	O
ATOM	3857	N	PHE	A	488	-31.047	13.633	9.023	1.00	89.88	N
ATOM	3858	CA	PHE	A	488	-30.078	13.359	10.078	1.00	89.88	C
ATOM	3859	C	PHE	A	488	-28.656	13.664	9.609	1.00	89.88	C
ATOM	3860	CB	PHE	A	488	-30.172	11.906	10.539	1.00	89.88	C
ATOM	3861	O	PHE	A	488	-27.859	14.234	10.352	1.00	89.88	O
ATOM	3862	CG	PHE	A	488	-31.391	11.617	11.391	1.00	89.88	C
ATOM	3863	CD1	PHE	A	488	-31.375	11.898	12.750	1.00	89.88	C
ATOM	3864	CD2	PHE	A	488	-32.531	11.062	10.820	1.00	89.88	C
ATOM	3865	CE1	PHE	A	488	-32.500	11.641	13.531	1.00	89.88	C
ATOM	3866	CE2	PHE	A	488	-33.656	10.797	11.602	1.00	89.88	C
ATOM	3867	CZ	PHE	A	488	-33.625	11.086	12.961	1.00	89.88	C
ATOM	3868	N	TRP	A	489	-28.375	13.312	8.352	1.00	89.19	N
ATOM	3869	CA	TRP	A	489	-27.062	13.500	7.742	1.00	89.19	C
ATOM	3870	C	TRP	A	489	-26.844	14.953	7.332	1.00	89.19	C
ATOM	3871	CB	TRP	A	489	-26.891	12.578	6.527	1.00	89.19	C
ATOM	3872	O	TRP	A	489	-25.828	15.555	7.668	1.00	89.19	O
ATOM	3873	CG	TRP	A	489	-25.531	12.648	5.895	1.00	89.19	C
ATOM	3874	CD1	TRP	A	489	-25.109	13.547	4.961	1.00	89.19	C
ATOM	3875	CD2	TRP	A	489	-24.438	11.773	6.148	1.00	89.19	C
ATOM	3876	CE2	TRP	A	489	-23.359	12.195	5.332	1.00	89.19	C
ATOM	3877	CE3	TRP	A	489	-24.250	10.664	6.988	1.00	89.19	C
ATOM	3878	NE1	TRP	A	489	-23.797	13.281	4.617	1.00	89.19	N
ATOM	3879	CH2	TRP	A	489	-21.969	10.477	6.160	1.00	89.19	C
ATOM	3880	CZ2	TRP	A	489	-22.125	11.555	5.332	1.00	89.19	C
ATOM	3881	CZ3	TRP	A	489	-23.016	10.031	6.984	1.00	89.19	C
ATOM	3882	N	SER	A	490	-27.781	15.516	6.641	1.00	90.88	N
ATOM	3883	CA	SER	A	490	-27.609	16.812	5.984	1.00	90.88	C
ATOM	3884	C	SER	A	490	-27.578	17.938	7.004	1.00	90.88	C
ATOM	3885	CB	SER	A	490	-28.719	17.062	4.973	1.00	90.88	C
ATOM	3886	O	SER	A	490	-26.812	18.906	6.848	1.00	90.88	O
ATOM	3887	OG	SER	A	490	-29.984	17.094	5.617	1.00	90.88	O
ATOM	3888	N	SER	A	491	-28.344	17.797	8.195	1.00	89.81	N
ATOM	3889	CA	SER	A	491	-28.500	18.953	9.078	1.00	89.81	C
ATOM	3890	C	SER	A	491	-28.594	18.516	10.539	1.00	89.81	C
ATOM	3891	CB	SER	A	491	-29.734	19.766	8.695	1.00	89.81	C
ATOM	3892	O	SER	A	491	-28.797	19.359	11.422	1.00	89.81	O
ATOM	3893	OG	SER	A	491	-30.922	19.047	8.984	1.00	89.81	O
ATOM	3894	N	ALA	A	492	-28.516	17.234	10.828	1.00	86.62	N
ATOM	3895	CA	ALA	A	492	-28.734	16.719	12.180	1.00	86.62	C
ATOM	3896	C	ALA	A	492	-30.109	17.094	12.703	1.00	86.62	C
ATOM	3897	CB	ALA	A	492	-27.641	17.234	13.125	1.00	86.62	C
ATOM	3898	O	ALA	A	492	-30.250	17.516	13.852	1.00	86.62	O
ATOM	3899	N	LEU	A	493	-31.078	17.219	11.828	1.00	87.12	N
ATOM	3900	CA	LEU	A	493	-32.500	17.422	12.086	1.00	87.12	C

ATOM	3901	C	LEU	A	493	-32.781	18.875	12.422	1.00	87.12	C
ATOM	3902	CB	LEU	A	493	-32.969	16.516	13.234	1.00	87.12	C
ATOM	3903	O	LEU	A	493	-33.781	19.188	13.070	1.00	87.12	O
ATOM	3904	CG	LEU	A	493	-32.844	15.016	13.000	1.00	87.12	C
ATOM	3905	CD1	LEU	A	493	-33.219	14.250	14.273	1.00	87.12	C
ATOM	3906	CD2	LEU	A	493	-33.750	14.586	11.852	1.00	87.12	C
ATOM	3907	N	THR	A	494	-31.812	19.844	12.141	1.00	87.38	N
ATOM	3908	CA	THR	A	494	-32.031	21.250	12.398	1.00	87.38	C
ATOM	3909	C	THR	A	494	-32.812	21.891	11.258	1.00	87.38	C
ATOM	3910	CB	THR	A	494	-30.703	22.000	12.594	1.00	87.38	C
ATOM	3911	O	THR	A	494	-33.469	22.938	11.445	1.00	87.38	O
ATOM	3912	CG2	THR	A	494	-29.969	21.516	13.836	1.00	87.38	C
ATOM	3913	OG1	THR	A	494	-29.875	21.797	11.445	1.00	87.38	O
ATOM	3914	N	LEU	A	495	-32.781	21.234	10.109	1.00	90.12	N
ATOM	3915	CA	LEU	A	495	-33.500	21.719	8.953	1.00	90.12	C
ATOM	3916	C	LEU	A	495	-34.500	20.672	8.461	1.00	90.12	C
ATOM	3917	CB	LEU	A	495	-32.562	22.094	7.820	1.00	90.12	C
ATOM	3918	O	LEU	A	495	-34.219	19.469	8.477	1.00	90.12	O
ATOM	3919	CG	LEU	A	495	-31.531	23.188	8.133	1.00	90.12	C
ATOM	3920	CD1	LEU	A	495	-30.562	23.344	6.973	1.00	90.12	C
ATOM	3921	CD2	LEU	A	495	-32.250	24.516	8.430	1.00	90.12	C
ATOM	3922	N	ASN	A	496	-35.750	21.125	8.062	1.00	91.69	N
ATOM	3923	CA	ASN	A	496	-36.750	20.281	7.418	1.00	91.69	C
ATOM	3924	C	ASN	A	496	-36.812	20.531	5.914	1.00	91.69	C
ATOM	3925	CB	ASN	A	496	-38.125	20.500	8.047	1.00	91.69	C
ATOM	3926	O	ASN	A	496	-37.500	21.438	5.453	1.00	91.69	O
ATOM	3927	CG	ASN	A	496	-38.188	20.062	9.492	1.00	91.69	C
ATOM	3928	ND2	ASN	A	496	-38.969	20.766	10.297	1.00	91.69	N
ATOM	3929	OD1	ASN	A	496	-37.531	19.078	9.883	1.00	91.69	O
ATOM	3930	N	LEU	A	497	-36.094	19.703	5.098	1.00	93.25	N
ATOM	3931	CA	LEU	A	497	-35.969	19.969	3.668	1.00	93.25	C
ATOM	3932	C	LEU	A	497	-36.531	18.828	2.846	1.00	93.25	C
ATOM	3933	CB	LEU	A	497	-34.500	20.203	3.289	1.00	93.25	C
ATOM	3934	O	LEU	A	497	-36.469	18.828	1.616	1.00	93.25	O
ATOM	3935	CG	LEU	A	497	-33.812	21.438	3.900	1.00	93.25	C
ATOM	3936	CD1	LEU	A	497	-32.344	21.453	3.557	1.00	93.25	C
ATOM	3937	CD2	LEU	A	497	-34.500	22.719	3.418	1.00	93.25	C
ATOM	3938	N	SER	A	498	-37.062	17.891	3.461	1.00	94.06	N
ATOM	3939	CA	SER	A	498	-37.688	16.781	2.744	1.00	94.06	C
ATOM	3940	C	SER	A	498	-38.906	16.234	3.486	1.00	94.06	C
ATOM	3941	CB	SER	A	498	-36.656	15.656	2.541	1.00	94.06	C
ATOM	3942	O	SER	A	498	-39.062	16.484	4.684	1.00	94.06	O
ATOM	3943	OG	SER	A	498	-36.250	15.117	3.787	1.00	94.06	O
ATOM	3944	N	GLY	A	499	-39.812	15.594	2.723	1.00	93.44	N
ATOM	3945	CA	GLY	A	499	-41.000	15.000	3.273	1.00	93.44	C
ATOM	3946	C	GLY	A	499	-42.062	14.695	2.219	1.00	93.44	C
ATOM	3947	O	GLY	A	499	-41.719	14.461	1.055	1.00	93.44	O
ATOM	3948	N	ARG	A	500	-43.281	14.625	2.752	1.00	93.25	N
ATOM	3949	CA	ARG	A	500	-44.406	14.383	1.849	1.00	93.25	C
ATOM	3950	C	ARG	A	500	-45.250	15.641	1.685	1.00	93.25	C
ATOM	3951	CB	ARG	A	500	-45.250	13.227	2.357	1.00	93.25	C
ATOM	3952	O	ARG	A	500	-45.438	16.391	2.641	1.00	93.25	O
ATOM	3953	CG	ARG	A	500	-44.562	11.875	2.301	1.00	93.25	C
ATOM	3954	CD	ARG	A	500	-45.500	10.758	2.785	1.00	93.25	C
ATOM	3955	NE	ARG	A	500	-44.812	9.461	2.750	1.00	93.25	N
ATOM	3956	NH1	ARG	A	500	-45.469	8.961	0.586	1.00	93.25	N
ATOM	3957	NH2	ARG	A	500	-44.188	7.477	1.781	1.00	93.25	N
ATOM	3958	CZ	ARG	A	500	-44.812	8.633	1.705	1.00	93.25	C
ATOM	3959	N	LEU	A	501	-45.625	15.766	0.418	1.00	93.81	N
ATOM	3960	CA	LEU	A	501	-46.500	16.906	0.124	1.00	93.81	C
ATOM	3961	C	LEU	A	501	-47.906	16.656	0.601	1.00	93.81	C
ATOM	3962	CB	LEU	A	501	-46.500	17.203	-1.378	1.00	93.81	C
ATOM	3963	O	LEU	A	501	-48.375	15.516	0.610	1.00	93.81	O
ATOM	3964	CG	LEU	A	501	-45.156	17.609	-1.984	1.00	93.81	C

ATOM	3965	CD1	LEU	A	501	-45.250	17.672	-3.506	1.00	93.81	C
ATOM	3966	CD2	LEU	A	501	-44.688	18.953	-1.421	1.00	93.81	C
ATOM	3967	N	MET	A	502	-48.625	17.734	0.964	1.00	94.12	N
ATOM	3968	CA	MET	A	502	-50.062	17.656	1.180	1.00	94.12	C
ATOM	3969	C	MET	A	502	-50.812	17.719	-0.146	1.00	94.12	C
ATOM	3970	CB	MET	A	502	-50.531	18.797	2.098	1.00	94.12	C
ATOM	3971	O	MET	A	502	-51.688	16.891	-0.409	1.00	94.12	O
ATOM	3972	CG	MET	A	502	-50.219	18.562	3.566	1.00	94.12	C
ATOM	3973	SD	MET	A	502	-51.656	18.938	4.652	1.00	94.12	S
ATOM	3974	CE	MET	A	502	-51.812	20.719	4.387	1.00	94.12	C
ATOM	3975	N	GLU	A	503	-50.469	18.672	-0.942	1.00	93.62	N
ATOM	3976	CA	GLU	A	503	-51.062	18.875	-2.258	1.00	93.62	C
ATOM	3977	C	GLU	A	503	-50.344	19.969	-3.037	1.00	93.62	C
ATOM	3978	CB	GLU	A	503	-52.562	19.203	-2.125	1.00	93.62	C
ATOM	3979	O	GLU	A	503	-49.656	20.797	-2.451	1.00	93.62	O
ATOM	3980	CG	GLU	A	503	-53.250	19.438	-3.455	1.00	93.62	C
ATOM	3981	CD	GLU	A	503	-54.688	19.922	-3.303	1.00	93.62	C
ATOM	3982	OE1	GLU	A	503	-55.375	20.125	-4.332	1.00	93.62	O
ATOM	3983	OE2	GLU	A	503	-55.125	20.094	-2.146	1.00	93.62	O
ATOM	3984	N	VAL	A	504	-50.406	19.922	-4.359	1.00	93.50	N
ATOM	3985	CA	VAL	A	504	-49.938	20.969	-5.250	1.00	93.50	C
ATOM	3986	C	VAL	A	504	-51.094	21.547	-6.043	1.00	93.50	C
ATOM	3987	CB	VAL	A	504	-48.844	20.438	-6.215	1.00	93.50	C
ATOM	3988	O	VAL	A	504	-51.844	20.812	-6.688	1.00	93.50	O
ATOM	3989	CG1	VAL	A	504	-48.375	21.531	-7.164	1.00	93.50	C
ATOM	3990	CG2	VAL	A	504	-47.656	19.859	-5.426	1.00	93.50	C
ATOM	3991	N	SER	A	505	-51.375	22.875	-5.848	1.00	92.56	N
ATOM	3992	CA	SER	A	505	-52.375	23.594	-6.609	1.00	92.56	C
ATOM	3993	C	SER	A	505	-51.750	24.734	-7.414	1.00	92.56	C
ATOM	3994	CB	SER	A	505	-53.469	24.156	-5.676	1.00	92.56	C
ATOM	3995	O	SER	A	505	-51.344	25.750	-6.848	1.00	92.56	O
ATOM	3996	OG	SER	A	505	-54.469	24.797	-6.418	1.00	92.56	O
ATOM	3997	N	GLY	A	506	-51.719	24.500	-8.789	1.00	91.19	N
ATOM	3998	CA	GLY	A	506	-51.062	25.469	-9.648	1.00	91.19	C
ATOM	3999	C	GLY	A	506	-49.562	25.609	-9.336	1.00	91.19	C
ATOM	4000	O	GLY	A	506	-48.812	24.641	-9.359	1.00	91.19	O
ATOM	4001	N	LEU	A	507	-49.125	26.781	-8.930	1.00	94.38	N
ATOM	4002	CA	LEU	A	507	-47.719	27.078	-8.633	1.00	94.38	C
ATOM	4003	C	LEU	A	507	-47.438	26.938	-7.145	1.00	94.38	C
ATOM	4004	CB	LEU	A	507	-47.375	28.500	-9.094	1.00	94.38	C
ATOM	4005	O	LEU	A	507	-46.281	27.078	-6.711	1.00	94.38	O
ATOM	4006	CG	LEU	A	507	-47.438	28.766	-10.602	1.00	94.38	C
ATOM	4007	CD1	LEU	A	507	-47.188	30.234	-10.891	1.00	94.38	C
ATOM	4008	CD2	LEU	A	507	-46.406	27.891	-11.328	1.00	94.38	C
ATOM	4009	N	GLN	A	508	-48.531	26.672	-6.410	1.00	95.38	N
ATOM	4010	CA	GLN	A	508	-48.406	26.594	-4.957	1.00	95.38	C
ATOM	4011	C	GLN	A	508	-48.281	25.156	-4.492	1.00	95.38	C
ATOM	4012	CB	GLN	A	508	-49.594	27.266	-4.270	1.00	95.38	C
ATOM	4013	O	GLN	A	508	-49.062	24.297	-4.871	1.00	95.38	O
ATOM	4014	CG	GLN	A	508	-49.688	28.766	-4.547	1.00	95.38	C
ATOM	4015	CD	GLN	A	508	-50.812	29.422	-3.756	1.00	95.38	C
ATOM	4016	NE2	GLN	A	508	-50.625	30.703	-3.420	1.00	95.38	N
ATOM	4017	OE1	GLN	A	508	-51.812	28.797	-3.449	1.00	95.38	O
ATOM	4018	N	VAL	A	509	-47.250	24.891	-3.736	1.00	96.12	N
ATOM	4019	CA	VAL	A	509	-47.000	23.578	-3.129	1.00	96.12	C
ATOM	4020	C	VAL	A	509	-47.281	23.656	-1.627	1.00	96.12	C
ATOM	4021	CB	VAL	A	509	-45.562	23.094	-3.395	1.00	96.12	C
ATOM	4022	O	VAL	A	509	-46.688	24.469	-0.923	1.00	96.12	O
ATOM	4023	CG1	VAL	A	509	-45.344	21.734	-2.762	1.00	96.12	C
ATOM	4024	CG2	VAL	A	509	-45.281	23.047	-4.898	1.00	96.12	C
ATOM	4025	N	PHE	A	510	-48.156	22.797	-1.104	1.00	96.06	N
ATOM	4026	CA	PHE	A	510	-48.531	22.781	0.307	1.00	96.06	C
ATOM	4027	C	PHE	A	510	-47.844	21.625	1.035	1.00	96.06	C
ATOM	4028	CB	PHE	A	510	-50.062	22.688	0.466	1.00	96.06	C

ATOM	4029	O	PHE	A	510	-47.938	20.484	0.592	1.00	96.06	O
ATOM	4030	CG	PHE	A	510	-50.812	23.844	-0.148	1.00	96.06	C
ATOM	4031	CD1	PHE	A	510	-51.062	24.984	0.592	1.00	96.06	C
ATOM	4032	CD2	PHE	A	510	-51.219	23.781	-1.466	1.00	96.06	C
ATOM	4033	CE1	PHE	A	510	-51.750	26.047	0.027	1.00	96.06	C
ATOM	4034	CE2	PHE	A	510	-51.938	24.844	-2.039	1.00	96.06	C
ATOM	4035	CZ	PHE	A	510	-52.188	25.969	-1.290	1.00	96.06	C
ATOM	4036	N	THR	A	511	-47.156	21.953	2.152	1.00	95.19	N
ATOM	4037	CA	THR	A	511	-46.500	20.953	2.998	1.00	95.19	C
ATOM	4038	C	THR	A	511	-47.156	20.891	4.371	1.00	95.19	C
ATOM	4039	CB	THR	A	511	-45.000	21.266	3.164	1.00	95.19	C
ATOM	4040	O	THR	A	511	-47.750	21.859	4.820	1.00	95.19	O
ATOM	4041	CG2	THR	A	511	-44.281	21.312	1.812	1.00	95.19	C
ATOM	4042	OG1	THR	A	511	-44.844	22.531	3.814	1.00	95.19	O
ATOM	4043	N	PRO	A	512	-47.219	19.734	5.094	1.00	93.38	N
ATOM	4044	CA	PRO	A	512	-47.906	19.562	6.375	1.00	93.38	C
ATOM	4045	C	PRO	A	512	-47.156	20.234	7.531	1.00	93.38	C
ATOM	4046	CB	PRO	A	512	-47.938	18.047	6.555	1.00	93.38	C
ATOM	4047	O	PRO	A	512	-47.719	20.406	8.617	1.00	93.38	O
ATOM	4048	CG	PRO	A	512	-46.781	17.547	5.781	1.00	93.38	C
ATOM	4049	CD	PRO	A	512	-46.531	18.453	4.609	1.00	93.38	C
ATOM	4050	N	PHE	A	513	-45.938	20.609	7.332	1.00	91.69	N
ATOM	4051	CA	PHE	A	513	-45.125	21.219	8.367	1.00	91.69	C
ATOM	4052	C	PHE	A	513	-44.250	22.328	7.781	1.00	91.69	C
ATOM	4053	CB	PHE	A	513	-44.250	20.172	9.055	1.00	91.69	C
ATOM	4054	O	PHE	A	513	-44.125	22.438	6.562	1.00	91.69	O
ATOM	4055	CG	PHE	A	513	-43.406	19.359	8.102	1.00	91.69	C
ATOM	4056	CD1	PHE	A	513	-43.875	18.156	7.590	1.00	91.69	C
ATOM	4057	CD2	PHE	A	513	-42.156	19.797	7.715	1.00	91.69	C
ATOM	4058	CE1	PHE	A	513	-43.125	17.406	6.707	1.00	91.69	C
ATOM	4059	CE2	PHE	A	513	-41.375	19.047	6.832	1.00	91.69	C
ATOM	4060	CZ	PHE	A	513	-41.875	17.859	6.328	1.00	91.69	C
ATOM	4061	N	LYS	A	514	-43.719	23.172	8.641	1.00	93.88	N
ATOM	4062	CA	LYS	A	514	-42.844	24.250	8.203	1.00	93.88	C
ATOM	4063	C	LYS	A	514	-41.562	23.688	7.570	1.00	93.88	C
ATOM	4064	CB	LYS	A	514	-42.500	25.156	9.375	1.00	93.88	C
ATOM	4065	O	LYS	A	514	-40.875	22.859	8.180	1.00	93.88	O
ATOM	4066	CG	LYS	A	514	-41.594	26.328	9.000	1.00	93.88	C
ATOM	4067	CD	LYS	A	514	-41.250	27.188	10.219	1.00	93.88	C
ATOM	4068	CE	LYS	A	514	-40.281	28.297	9.859	1.00	93.88	C
ATOM	4069	NZ	LYS	A	514	-39.969	29.172	11.031	1.00	93.88	N
ATOM	4070	N	VAL	A	515	-41.312	24.109	6.215	1.00	93.38	N
ATOM	4071	CA	VAL	A	515	-40.125	23.688	5.512	1.00	93.38	C
ATOM	4072	C	VAL	A	515	-39.094	24.828	5.492	1.00	93.38	C
ATOM	4073	CB	VAL	A	515	-40.438	23.234	4.070	1.00	93.38	C
ATOM	4074	O	VAL	A	515	-39.469	26.000	5.336	1.00	93.38	O
ATOM	4075	CG1	VAL	A	515	-39.188	22.703	3.383	1.00	93.38	C
ATOM	4076	CG2	VAL	A	515	-41.531	22.172	4.074	1.00	93.38	C
ATOM	4077	N	ASP	A	516	-37.844	24.562	5.820	1.00	93.31	N
ATOM	4078	CA	ASP	A	516	-36.781	25.547	5.887	1.00	93.31	C
ATOM	4079	C	ASP	A	516	-36.250	25.891	4.492	1.00	93.31	C
ATOM	4080	CB	ASP	A	516	-35.625	25.047	6.770	1.00	93.31	C
ATOM	4081	O	ASP	A	516	-35.031	26.047	4.301	1.00	93.31	O
ATOM	4082	CG	ASP	A	516	-36.031	24.875	8.227	1.00	93.31	C
ATOM	4083	OD1	ASP	A	516	-36.406	25.875	8.875	1.00	93.31	O
ATOM	4084	OD2	ASP	A	516	-35.969	23.734	8.719	1.00	93.31	O
ATOM	4085	N	ALA	A	517	-37.031	26.094	3.443	1.00	93.19	N
ATOM	4086	CA	ALA	A	517	-36.688	26.453	2.072	1.00	93.19	C
ATOM	4087	C	ALA	A	517	-36.594	27.969	1.917	1.00	93.19	C
ATOM	4088	CB	ALA	A	517	-37.719	25.891	1.100	1.00	93.19	C
ATOM	4089	O	ALA	A	517	-37.188	28.719	2.686	1.00	93.19	O
ATOM	4090	N	ARG	A	518	-35.750	28.453	0.983	1.00	94.06	N
ATOM	4091	CA	ARG	A	518	-35.594	29.859	0.615	1.00	94.06	C
ATOM	4092	C	ARG	A	518	-35.969	30.078	-0.847	1.00	94.06	C

ATOM	4093	CB	ARG	A	518	-34.156	30.328	0.871	1.00	94.06	C
ATOM	4094	O	ARG	A	518	-35.875	29.156	-1.661	1.00	94.06	O
ATOM	4095	CG	ARG	A	518	-33.688	30.094	2.295	1.00	94.06	C
ATOM	4096	CD	ARG	A	518	-32.188	30.312	2.430	1.00	94.06	C
ATOM	4097	NE	ARG	A	518	-31.688	29.922	3.744	1.00	94.06	N
ATOM	4098	NH1	ARG	A	518	-29.453	30.016	3.172	1.00	94.06	N
ATOM	4099	NH2	ARG	A	518	-30.078	29.406	5.297	1.00	94.06	N
ATOM	4100	CZ	ARG	A	518	-30.406	29.781	4.066	1.00	94.06	C
ATOM	4101	N	ALA	A	519	-36.375	31.328	-1.115	1.00	93.75	N
ATOM	4102	CA	ALA	A	519	-36.562	31.688	-2.523	1.00	93.75	C
ATOM	4103	C	ALA	A	519	-35.281	31.422	-3.318	1.00	93.75	C
ATOM	4104	CB	ALA	A	519	-36.969	33.156	-2.660	1.00	93.75	C
ATOM	4105	O	ALA	A	519	-34.188	31.750	-2.865	1.00	93.75	O
ATOM	4106	N	GLY	A	520	-35.344	30.688	-4.523	1.00	93.00	N
ATOM	4107	CA	GLY	A	520	-34.219	30.328	-5.344	1.00	93.00	C
ATOM	4108	C	GLY	A	520	-33.812	28.875	-5.207	1.00	93.00	C
ATOM	4109	O	GLY	A	520	-33.125	28.328	-6.082	1.00	93.00	O
ATOM	4110	N	ASP	A	521	-34.219	28.266	-4.105	1.00	95.12	N
ATOM	4111	CA	ASP	A	521	-33.969	26.844	-3.900	1.00	95.12	C
ATOM	4112	C	ASP	A	521	-34.750	25.984	-4.887	1.00	95.12	C
ATOM	4113	CB	ASP	A	521	-34.312	26.438	-2.465	1.00	95.12	C
ATOM	4114	O	ASP	A	521	-35.719	26.484	-5.500	1.00	95.12	O
ATOM	4115	CG	ASP	A	521	-33.375	27.047	-1.439	1.00	95.12	C
ATOM	4116	OD1	ASP	A	521	-32.281	27.562	-1.824	1.00	95.12	O
ATOM	4117	OD2	ASP	A	521	-33.688	27.000	-0.235	1.00	95.12	O
ATOM	4118	N	ARG	A	522	-34.344	24.688	-5.070	1.00	94.31	N
ATOM	4119	CA	ARG	A	522	-35.031	23.797	-6.004	1.00	94.31	C
ATOM	4120	C	ARG	A	522	-35.750	22.672	-5.262	1.00	94.31	C
ATOM	4121	CB	ARG	A	522	-34.031	23.219	-7.008	1.00	94.31	C
ATOM	4122	O	ARG	A	522	-35.125	21.953	-4.473	1.00	94.31	O
ATOM	4123	CG	ARG	A	522	-33.562	24.203	-8.062	1.00	94.31	C
ATOM	4124	CD	ARG	A	522	-32.812	23.531	-9.188	1.00	94.31	C
ATOM	4125	NE	ARG	A	522	-33.688	22.688	-10.000	1.00	94.31	N
ATOM	4126	NH1	ARG	A	522	-31.984	21.875	-11.344	1.00	94.31	N
ATOM	4127	NH2	ARG	A	522	-34.156	21.203	-11.688	1.00	94.31	N
ATOM	4128	CZ	ARG	A	522	-33.281	21.922	-11.008	1.00	94.31	C
ATOM	4129	N	ILE	A	523	-37.062	22.531	-5.496	1.00	94.94	N
ATOM	4130	CA	ILE	A	523	-37.844	21.438	-4.930	1.00	94.94	C
ATOM	4131	C	ILE	A	523	-38.000	20.312	-5.957	1.00	94.94	C
ATOM	4132	CB	ILE	A	523	-39.250	21.922	-4.477	1.00	94.94	C
ATOM	4133	O	ILE	A	523	-38.312	20.562	-7.117	1.00	94.94	O
ATOM	4134	CG1	ILE	A	523	-40.031	20.781	-3.834	1.00	94.94	C
ATOM	4135	CG2	ILE	A	523	-40.031	22.516	-5.660	1.00	94.94	C
ATOM	4136	CD1	ILE	A	523	-41.312	21.219	-3.125	1.00	94.94	C
ATOM	4137	N	LEU	A	524	-37.656	19.109	-5.527	1.00	94.75	N
ATOM	4138	CA	LEU	A	524	-37.750	17.906	-6.359	1.00	94.75	C
ATOM	4139	C	LEU	A	524	-38.969	17.062	-5.941	1.00	94.75	C
ATOM	4140	CB	LEU	A	524	-36.469	17.094	-6.273	1.00	94.75	C
ATOM	4141	O	LEU	A	524	-39.156	16.797	-4.754	1.00	94.75	O
ATOM	4142	CG	LEU	A	524	-35.219	17.719	-6.883	1.00	94.75	C
ATOM	4143	CD1	LEU	A	524	-34.500	18.594	-5.852	1.00	94.75	C
ATOM	4144	CD2	LEU	A	524	-34.281	16.641	-7.414	1.00	94.75	C
ATOM	4145	N	VAL	A	525	-39.719	16.641	-7.008	1.00	94.38	N
ATOM	4146	CA	VAL	A	525	-40.844	15.734	-6.762	1.00	94.38	C
ATOM	4147	C	VAL	A	525	-40.781	14.570	-7.750	1.00	94.38	C
ATOM	4148	CB	VAL	A	525	-42.188	16.469	-6.875	1.00	94.38	C
ATOM	4149	O	VAL	A	525	-40.281	14.711	-8.867	1.00	94.38	O
ATOM	4150	CG1	VAL	A	525	-42.312	17.531	-5.789	1.00	94.38	C
ATOM	4151	CG2	VAL	A	525	-42.344	17.094	-8.258	1.00	94.38	C
ATOM	4152	N	ASN	A	526	-41.281	13.469	-7.273	1.00	91.31	N
ATOM	4153	CA	ASN	A	526	-41.312	12.289	-8.133	1.00	91.31	C
ATOM	4154	C	ASN	A	526	-42.469	12.352	-9.125	1.00	91.31	C
ATOM	4155	CB	ASN	A	526	-41.375	11.016	-7.293	1.00	91.31	C
ATOM	4156	O	ASN	A	526	-43.625	12.570	-8.727	1.00	91.31	O

ATOM	4157	CG	ASN	A	526	-40.125	10.742	-6.520	1.00	91.31	C
ATOM	4158	ND2	ASN	A	526	-40.250	10.094	-5.367	1.00	91.31	N
ATOM	4159	OD1	ASN	A	526	-39.031	11.117	-6.945	1.00	91.31	O
ATOM	4160	N	LYS	A	527	-42.125	12.180	-10.461	1.00	88.81	N
ATOM	4161	CA	LYS	A	527	-43.156	11.969	-11.477	1.00	88.81	C
ATOM	4162	C	LYS	A	527	-43.812	10.609	-11.305	1.00	88.81	C
ATOM	4163	CB	LYS	A	527	-42.562	12.086	-12.883	1.00	88.81	C
ATOM	4164	O	LYS	A	527	-43.281	9.727	-10.625	1.00	88.81	O
ATOM	4165	CG	LYS	A	527	-42.094	13.484	-13.242	1.00	88.81	C
ATOM	4166	CD	LYS	A	527	-41.531	13.547	-14.664	1.00	88.81	C
ATOM	4167	CE	LYS	A	527	-40.969	14.914	-14.984	1.00	88.81	C
ATOM	4168	NZ	LYS	A	527	-40.406	14.977	-16.375	1.00	88.81	N
ATOM	4169	N	PRO	A	528	-45.000	10.422	-11.883	1.00	83.44	N
ATOM	4170	CA	PRO	A	528	-45.688	9.133	-11.773	1.00	83.44	C
ATOM	4171	C	PRO	A	528	-44.844	7.961	-12.227	1.00	83.44	C
ATOM	4172	CB	PRO	A	528	-46.906	9.297	-12.688	1.00	83.44	C
ATOM	4173	O	PRO	A	528	-45.000	6.840	-11.734	1.00	83.44	O
ATOM	4174	CG	PRO	A	528	-47.219	10.758	-12.664	1.00	83.44	C
ATOM	4175	CD	PRO	A	528	-45.906	11.508	-12.555	1.00	83.44	C
ATOM	4176	N	ASP	A	529	-43.844	8.242	-13.102	1.00	82.94	N
ATOM	4177	CA	ASP	A	529	-42.969	7.168	-13.555	1.00	82.94	C
ATOM	4178	C	ASP	A	529	-41.781	6.969	-12.594	1.00	82.94	C
ATOM	4179	CB	ASP	A	529	-42.438	7.465	-14.961	1.00	82.94	C
ATOM	4180	O	ASP	A	529	-40.969	6.066	-12.773	1.00	82.94	O
ATOM	4181	CG	ASP	A	529	-41.594	8.719	-15.023	1.00	82.94	C
ATOM	4182	OD1	ASP	A	529	-41.344	9.359	-13.969	1.00	82.94	O
ATOM	4183	OD2	ASP	A	529	-41.125	9.078	-16.141	1.00	82.94	O
ATOM	4184	N	GLY	A	530	-41.781	7.840	-11.531	1.00	81.50	N
ATOM	4185	CA	GLY	A	530	-40.750	7.680	-10.508	1.00	81.50	C
ATOM	4186	C	GLY	A	530	-39.594	8.656	-10.656	1.00	81.50	C
ATOM	4187	O	GLY	A	530	-38.969	9.039	-9.672	1.00	81.50	O
ATOM	4188	N	LYS	A	531	-39.312	9.250	-11.852	1.00	88.19	N
ATOM	4189	CA	LYS	A	531	-38.188	10.148	-12.094	1.00	88.19	C
ATOM	4190	C	LYS	A	531	-38.438	11.516	-11.445	1.00	88.19	C
ATOM	4191	CB	LYS	A	531	-37.938	10.320	-13.594	1.00	88.19	C
ATOM	4192	O	LYS	A	531	-39.469	12.133	-11.648	1.00	88.19	O
ATOM	4193	CG	LYS	A	531	-37.406	9.070	-14.281	1.00	88.19	C
ATOM	4194	CD	LYS	A	531	-37.125	9.328	-15.750	1.00	88.19	C
ATOM	4195	CE	LYS	A	531	-36.625	8.062	-16.453	1.00	88.19	C
ATOM	4196	NZ	LYS	A	531	-36.375	8.297	-17.906	1.00	88.19	N
ATOM	4197	N	PRO	A	532	-37.438	12.008	-10.672	1.00	91.88	N
ATOM	4198	CA	PRO	A	532	-37.625	13.289	-9.984	1.00	91.88	C
ATOM	4199	C	PRO	A	532	-37.562	14.477	-10.938	1.00	91.88	C
ATOM	4200	CB	PRO	A	532	-36.438	13.328	-9.008	1.00	91.88	C
ATOM	4201	O	PRO	A	532	-36.812	14.469	-11.906	1.00	91.88	O
ATOM	4202	CG	PRO	A	532	-35.906	11.930	-8.977	1.00	91.88	C
ATOM	4203	CD	PRO	A	532	-36.312	11.242	-10.242	1.00	91.88	C
ATOM	4204	N	VAL	A	533	-38.406	15.430	-10.750	1.00	93.12	N
ATOM	4205	CA	VAL	A	533	-38.406	16.688	-11.500	1.00	93.12	C
ATOM	4206	C	VAL	A	533	-38.281	17.859	-10.531	1.00	93.12	C
ATOM	4207	CB	VAL	A	533	-39.688	16.828	-12.336	1.00	93.12	C
ATOM	4208	O	VAL	A	533	-39.000	17.938	-9.531	1.00	93.12	O
ATOM	4209	CG1	VAL	A	533	-39.719	18.172	-13.078	1.00	93.12	C
ATOM	4210	CG2	VAL	A	533	-39.812	15.672	-13.328	1.00	93.12	C
ATOM	4211	N	GLY	A	534	-37.281	18.734	-10.812	1.00	92.94	N
ATOM	4212	CA	GLY	A	534	-37.031	19.875	-9.938	1.00	92.94	C
ATOM	4213	C	GLY	A	534	-37.594	21.172	-10.477	1.00	92.94	C
ATOM	4214	O	GLY	A	534	-37.656	21.375	-11.688	1.00	92.94	O
ATOM	4215	N	ARG	A	535	-38.094	22.031	-9.633	1.00	94.94	N
ATOM	4216	CA	ARG	A	535	-38.531	23.391	-9.922	1.00	94.94	C
ATOM	4217	C	ARG	A	535	-37.938	24.375	-8.930	1.00	94.94	C
ATOM	4218	CB	ARG	A	535	-40.062	23.469	-9.898	1.00	94.94	C
ATOM	4219	O	ARG	A	535	-37.719	24.047	-7.758	1.00	94.94	O
ATOM	4220	CG	ARG	A	535	-40.750	22.656	-10.977	1.00	94.94	C

ATOM	4221	CD	ARG	A	535	-40.531	23.250	-12.359	1.00	94.94	C
ATOM	4222	NE	ARG	A	535	-41.250	22.516	-13.383	1.00	94.94	N
ATOM	4223	NH1	ARG	A	535	-39.594	20.953	-13.766	1.00	94.94	N
ATOM	4224	NH2	ARG	A	535	-41.562	20.859	-14.945	1.00	94.94	N
ATOM	4225	CZ	ARG	A	535	-40.812	21.438	-14.031	1.00	94.94	C
ATOM	4226	N	THR	A	536	-37.656	25.578	-9.445	1.00	96.00	N
ATOM	4227	CA	THR	A	536	-37.094	26.641	-8.602	1.00	96.00	C
ATOM	4228	C	THR	A	536	-38.188	27.297	-7.785	1.00	96.00	C
ATOM	4229	CB	THR	A	536	-36.375	27.703	-9.445	1.00	96.00	C
ATOM	4230	O	THR	A	536	-39.250	27.641	-8.320	1.00	96.00	O
ATOM	4231	CG2	THR	A	536	-35.719	28.750	-8.562	1.00	96.00	C
ATOM	4232	OG1	THR	A	536	-35.344	27.062	-10.227	1.00	96.00	O
ATOM	4233	N	ILE	A	537	-38.031	27.531	-6.496	1.00	96.69	N
ATOM	4234	CA	ILE	A	537	-38.969	28.141	-5.570	1.00	96.69	C
ATOM	4235	C	ILE	A	537	-38.938	29.656	-5.715	1.00	96.69	C
ATOM	4236	CB	ILE	A	537	-38.688	27.734	-4.109	1.00	96.69	C
ATOM	4237	O	ILE	A	537	-37.875	30.266	-5.586	1.00	96.69	O
ATOM	4238	CG1	ILE	A	537	-38.844	26.219	-3.947	1.00	96.69	C
ATOM	4239	CG2	ILE	A	537	-39.625	28.469	-3.148	1.00	96.69	C
ATOM	4240	CD1	ILE	A	537	-38.312	25.688	-2.617	1.00	96.69	C
ATOM	4241	N	ALA	A	538	-40.000	30.250	-6.039	1.00	96.44	N
ATOM	4242	CA	ALA	A	538	-40.094	31.688	-6.203	1.00	96.44	C
ATOM	4243	C	ALA	A	538	-40.250	32.375	-4.852	1.00	96.44	C
ATOM	4244	CB	ALA	A	538	-41.250	32.031	-7.117	1.00	96.44	C
ATOM	4245	O	ALA	A	538	-39.625	33.406	-4.598	1.00	96.44	O
ATOM	4246	N	ARG	A	539	-41.125	31.906	-4.027	1.00	95.69	N
ATOM	4247	CA	ARG	A	539	-41.406	32.531	-2.727	1.00	95.69	C
ATOM	4248	C	ARG	A	539	-41.844	31.469	-1.716	1.00	95.69	C
ATOM	4249	CB	ARG	A	539	-42.469	33.594	-2.852	1.00	95.69	C
ATOM	4250	O	ARG	A	539	-42.438	30.453	-2.082	1.00	95.69	O
ATOM	4251	CG	ARG	A	539	-42.719	34.375	-1.562	1.00	95.69	C
ATOM	4252	CD	ARG	A	539	-43.719	35.500	-1.765	1.00	95.69	C
ATOM	4253	NE	ARG	A	539	-45.094	35.000	-1.958	1.00	95.69	N
ATOM	4254	NH1	ARG	A	539	-46.000	36.219	-0.240	1.00	95.69	N
ATOM	4255	NH2	ARG	A	539	-47.312	34.812	-1.491	1.00	95.69	N
ATOM	4256	CZ	ARG	A	539	-46.125	35.344	-1.229	1.00	95.69	C
ATOM	4257	N	VAL	A	540	-41.500	31.719	-0.381	1.00	95.69	N
ATOM	4258	CA	VAL	A	540	-41.906	30.875	0.732	1.00	95.69	C
ATOM	4259	C	VAL	A	540	-42.844	31.672	1.654	1.00	95.69	C
ATOM	4260	CB	VAL	A	540	-40.719	30.312	1.527	1.00	95.69	C
ATOM	4261	O	VAL	A	540	-42.500	32.812	2.039	1.00	95.69	O
ATOM	4262	CG1	VAL	A	540	-41.188	29.391	2.643	1.00	95.69	C
ATOM	4263	CG2	VAL	A	540	-39.750	29.594	0.593	1.00	95.69	C
ATOM	4264	N	SER	A	541	-44.094	31.172	1.982	1.00	95.31	N
ATOM	4265	CA	SER	A	541	-45.031	31.859	2.877	1.00	95.31	C
ATOM	4266	C	SER	A	541	-44.375	32.125	4.230	1.00	95.31	C
ATOM	4267	CB	SER	A	541	-46.281	31.031	3.072	1.00	95.31	C
ATOM	4268	O	SER	A	541	-43.375	31.516	4.590	1.00	95.31	O
ATOM	4269	OG	SER	A	541	-46.000	29.844	3.803	1.00	95.31	O
ATOM	4270	N	ASP	A	542	-44.906	33.031	5.051	1.00	91.00	N
ATOM	4271	CA	ASP	A	542	-44.406	33.438	6.355	1.00	91.00	C
ATOM	4272	C	ASP	A	542	-44.375	32.281	7.332	1.00	91.00	C
ATOM	4273	CB	ASP	A	542	-45.250	34.594	6.922	1.00	91.00	C
ATOM	4274	O	ASP	A	542	-43.469	32.125	8.141	1.00	91.00	O
ATOM	4275	CG	ASP	A	542	-45.031	35.906	6.191	1.00	91.00	C
ATOM	4276	OD1	ASP	A	542	-44.000	36.031	5.480	1.00	91.00	O
ATOM	4277	OD2	ASP	A	542	-45.875	36.812	6.328	1.00	91.00	O
ATOM	4278	N	ASP	A	543	-45.469	31.375	7.230	1.00	91.94	N
ATOM	4279	CA	ASP	A	543	-45.531	30.250	8.156	1.00	91.94	C
ATOM	4280	C	ASP	A	543	-44.625	29.109	7.707	1.00	91.94	C
ATOM	4281	CB	ASP	A	543	-47.000	29.766	8.289	1.00	91.94	C
ATOM	4282	O	ASP	A	543	-44.438	28.141	8.438	1.00	91.94	O
ATOM	4283	CG	ASP	A	543	-47.594	29.312	6.973	1.00	91.94	C
ATOM	4284	OD1	ASP	A	543	-46.875	29.312	5.945	1.00	91.94	O

ATOM	4285	OD2	ASP	A	543	-48.781	28.969	6.953	1.00	91.94	O
ATOM	4286	N	GLY	A	544	-44.031	29.234	6.363	1.00	92.44	N
ATOM	4287	CA	GLY	A	544	-43.094	28.266	5.840	1.00	92.44	C
ATOM	4288	C	GLY	A	544	-43.750	26.984	5.367	1.00	92.44	C
ATOM	4289	O	GLY	A	544	-43.094	26.000	5.070	1.00	92.44	O
ATOM	4290	N	LYS	A	545	-45.062	26.891	5.188	1.00	94.25	N
ATOM	4291	CA	LYS	A	545	-45.812	25.672	4.867	1.00	94.25	C
ATOM	4292	C	LYS	A	545	-46.312	25.703	3.422	1.00	94.25	C
ATOM	4293	CB	LYS	A	545	-47.000	25.500	5.820	1.00	94.25	C
ATOM	4294	O	LYS	A	545	-46.812	24.703	2.912	1.00	94.25	O
ATOM	4295	CG	LYS	A	545	-46.594	25.188	7.254	1.00	94.25	C
ATOM	4296	CD	LYS	A	545	-47.812	24.953	8.141	1.00	94.25	C
ATOM	4297	CE	LYS	A	545	-47.406	24.625	9.570	1.00	94.25	C
ATOM	4298	NZ	LYS	A	545	-48.594	24.406	10.445	1.00	94.25	N
ATOM	4299	N	THR	A	546	-46.219	26.859	2.750	1.00	96.44	N
ATOM	4300	CA	THR	A	546	-46.594	27.000	1.353	1.00	96.44	C
ATOM	4301	C	THR	A	546	-45.438	27.547	0.519	1.00	96.44	C
ATOM	4302	CB	THR	A	546	-47.812	27.938	1.208	1.00	96.44	C
ATOM	4303	O	THR	A	546	-44.875	28.594	0.848	1.00	96.44	O
ATOM	4304	CG2	THR	A	546	-48.312	27.969	-0.233	1.00	96.44	C
ATOM	4305	OG1	THR	A	546	-48.875	27.453	2.055	1.00	96.44	O
ATOM	4306	N	LEU	A	547	-45.125	26.766	-0.538	1.00	96.56	N
ATOM	4307	CA	LEU	A	547	-44.094	27.188	-1.481	1.00	96.56	C
ATOM	4308	C	LEU	A	547	-44.719	27.578	-2.822	1.00	96.56	C
ATOM	4309	CB	LEU	A	547	-43.062	26.078	-1.684	1.00	96.56	C
ATOM	4310	O	LEU	A	547	-45.531	26.828	-3.367	1.00	96.56	O
ATOM	4311	CG	LEU	A	547	-42.500	25.422	-0.414	1.00	96.56	C
ATOM	4312	CD1	LEU	A	547	-41.625	24.250	-0.771	1.00	96.56	C
ATOM	4313	CD2	LEU	A	547	-41.750	26.453	0.429	1.00	96.56	C
ATOM	4314	N	THR	A	548	-44.312	28.750	-3.352	1.00	96.69	N
ATOM	4315	CA	THR	A	548	-44.750	29.188	-4.680	1.00	96.69	C
ATOM	4316	C	THR	A	548	-43.594	29.031	-5.684	1.00	96.69	C
ATOM	4317	CB	THR	A	548	-45.219	30.641	-4.664	1.00	96.69	C
ATOM	4318	O	THR	A	548	-42.531	29.562	-5.484	1.00	96.69	O
ATOM	4319	CG2	THR	A	548	-45.781	31.062	-6.027	1.00	96.69	C
ATOM	4320	OG1	THR	A	548	-46.250	30.797	-3.682	1.00	96.69	O
ATOM	4321	N	LEU	A	549	-43.844	28.266	-6.816	1.00	96.50	N
ATOM	4322	CA	LEU	A	549	-42.812	27.938	-7.812	1.00	96.50	C
ATOM	4323	C	LEU	A	549	-42.844	28.953	-8.953	1.00	96.50	C
ATOM	4324	CB	LEU	A	549	-43.062	26.516	-8.367	1.00	96.50	C
ATOM	4325	O	LEU	A	549	-43.812	29.656	-9.156	1.00	96.50	O
ATOM	4326	CG	LEU	A	549	-43.250	25.406	-7.336	1.00	96.50	C
ATOM	4327	CD1	LEU	A	549	-43.531	24.078	-8.031	1.00	96.50	C
ATOM	4328	CD2	LEU	A	549	-42.000	25.297	-6.453	1.00	96.50	C
ATOM	4329	N	ASN	A	550	-41.719	28.984	-9.758	1.00	94.44	N
ATOM	4330	CA	ASN	A	550	-41.594	29.859	-10.922	1.00	94.44	C
ATOM	4331	C	ASN	A	550	-42.375	29.297	-12.117	1.00	94.44	C
ATOM	4332	CB	ASN	A	550	-40.125	30.047	-11.297	1.00	94.44	C
ATOM	4333	O	ASN	A	550	-42.812	30.062	-12.977	1.00	94.44	O
ATOM	4334	CG	ASN	A	550	-39.438	31.031	-10.383	1.00	94.44	C
ATOM	4335	ND2	ASN	A	550	-38.094	30.922	-10.289	1.00	94.44	N
ATOM	4336	OD1	ASN	A	550	-40.062	31.891	-9.766	1.00	94.44	O
ATOM	4337	N	THR	A	551	-42.406	27.953	-12.156	1.00	94.62	N
ATOM	4338	CA	THR	A	551	-43.125	27.250	-13.219	1.00	94.62	C
ATOM	4339	C	THR	A	551	-43.750	25.953	-12.695	1.00	94.62	C
ATOM	4340	CB	THR	A	551	-42.219	26.953	-14.406	1.00	94.62	C
ATOM	4341	O	THR	A	551	-43.250	25.391	-11.703	1.00	94.62	O
ATOM	4342	CG2	THR	A	551	-41.156	25.922	-14.039	1.00	94.62	C
ATOM	4343	OG1	THR	A	551	-43.000	26.453	-15.500	1.00	94.62	O
ATOM	4344	N	THR	A	552	-44.938	25.531	-13.352	1.00	91.81	N
ATOM	4345	CA	THR	A	552	-45.594	24.297	-12.922	1.00	91.81	C
ATOM	4346	C	THR	A	552	-44.719	23.094	-13.250	1.00	91.81	C
ATOM	4347	CB	THR	A	552	-46.969	24.125	-13.570	1.00	91.81	C
ATOM	4348	O	THR	A	552	-43.781	23.188	-14.055	1.00	91.81	O

ATOM	4349	CG2	THR	A	552	-47.781	25.406	-13.445	1.00	91.81	C
ATOM	4350	OG1	THR	A	552	-46.812	23.812	-14.961	1.00	91.81	O
ATOM	4351	N	PHE	A	553	-44.938	21.891	-12.609	1.00	90.62	N
ATOM	4352	CA	PHE	A	553	-44.188	20.656	-12.836	1.00	90.62	C
ATOM	4353	C	PHE	A	553	-44.531	20.047	-14.188	1.00	90.62	C
ATOM	4354	CB	PHE	A	553	-44.469	19.641	-11.719	1.00	90.62	C
ATOM	4355	O	PHE	A	553	-43.781	19.250	-14.734	1.00	90.62	O
ATOM	4356	CG	PHE	A	553	-43.750	19.938	-10.430	1.00	90.62	C
ATOM	4357	CD1	PHE	A	553	-42.406	19.719	-10.312	1.00	90.62	C
ATOM	4358	CD2	PHE	A	553	-44.469	20.438	-9.336	1.00	90.62	C
ATOM	4359	CE1	PHE	A	553	-41.719	20.000	-9.117	1.00	90.62	C
ATOM	4360	CE2	PHE	A	553	-43.812	20.719	-8.141	1.00	90.62	C
ATOM	4361	CZ	PHE	A	553	-42.438	20.500	-8.031	1.00	90.62	C
ATOM	4362	N	GLY	A	554	-45.625	20.484	-14.914	1.00	88.50	N
ATOM	4363	CA	GLY	A	554	-46.031	19.984	-16.203	1.00	88.50	C
ATOM	4364	C	GLY	A	554	-46.844	18.703	-16.109	1.00	88.50	C
ATOM	4365	O	GLY	A	554	-47.125	18.047	-17.125	1.00	88.50	O
ATOM	4366	N	PHE	A	555	-47.156	18.156	-14.891	1.00	90.25	N
ATOM	4367	CA	PHE	A	555	-48.031	17.016	-14.617	1.00	90.25	C
ATOM	4368	C	PHE	A	555	-48.750	17.203	-13.297	1.00	90.25	C
ATOM	4369	CB	PHE	A	555	-47.219	15.719	-14.602	1.00	90.25	C
ATOM	4370	O	PHE	A	555	-48.375	18.062	-12.492	1.00	90.25	O
ATOM	4371	CG	PHE	A	555	-46.156	15.664	-13.523	1.00	90.25	C
ATOM	4372	CD1	PHE	A	555	-44.906	16.188	-13.742	1.00	90.25	C
ATOM	4373	CD2	PHE	A	555	-46.438	15.086	-12.297	1.00	90.25	C
ATOM	4374	CE1	PHE	A	555	-43.938	16.156	-12.750	1.00	90.25	C
ATOM	4375	CE2	PHE	A	555	-45.469	15.039	-11.297	1.00	90.25	C
ATOM	4376	CZ	PHE	A	555	-44.219	15.570	-11.523	1.00	90.25	C
ATOM	4377	N	ASP	A	556	-49.812	16.453	-13.086	1.00	90.19	N
ATOM	4378	CA	ASP	A	556	-50.594	16.531	-11.844	1.00	90.19	C
ATOM	4379	C	ASP	A	556	-49.875	15.789	-10.711	1.00	90.19	C
ATOM	4380	CB	ASP	A	556	-52.000	15.977	-12.039	1.00	90.19	C
ATOM	4381	O	ASP	A	556	-49.781	14.562	-10.734	1.00	90.19	O
ATOM	4382	CG	ASP	A	556	-52.844	16.812	-12.984	1.00	90.19	C
ATOM	4383	OD1	ASP	A	556	-52.656	18.047	-13.023	1.00	90.19	O
ATOM	4384	OD2	ASP	A	556	-53.688	16.234	-13.688	1.00	90.19	O
ATOM	4385	N	VAL	A	557	-49.281	16.562	-9.734	1.00	92.25	N
ATOM	4386	CA	VAL	A	557	-48.594	15.969	-8.586	1.00	92.25	C
ATOM	4387	C	VAL	A	557	-49.625	15.438	-7.590	1.00	92.25	C
ATOM	4388	CB	VAL	A	557	-47.656	17.000	-7.898	1.00	92.25	C
ATOM	4389	O	VAL	A	557	-50.500	16.156	-7.160	1.00	92.25	O
ATOM	4390	CG1	VAL	A	557	-46.906	16.344	-6.734	1.00	92.25	C
ATOM	4391	CG2	VAL	A	557	-46.688	17.578	-8.906	1.00	92.25	C
ATOM	4392	N	GLN	A	558	-49.562	14.117	-7.203	1.00	91.19	N
ATOM	4393	CA	GLN	A	558	-50.500	13.477	-6.289	1.00	91.19	C
ATOM	4394	C	GLN	A	558	-50.188	13.844	-4.840	1.00	91.19	C
ATOM	4395	CB	GLN	A	558	-50.469	11.953	-6.465	1.00	91.19	C
ATOM	4396	O	GLN	A	558	-49.031	14.078	-4.488	1.00	91.19	O
ATOM	4397	CG	GLN	A	558	-50.906	11.492	-7.840	1.00	91.19	C
ATOM	4398	CD	GLN	A	558	-52.344	11.836	-8.133	1.00	91.19	C
ATOM	4399	NE2	GLN	A	558	-52.625	12.273	-9.359	1.00	91.19	N
ATOM	4400	OE1	GLN	A	558	-53.219	11.703	-7.270	1.00	91.19	O
ATOM	4401	N	PRO	A	559	-51.344	13.969	-4.012	1.00	91.56	N
ATOM	4402	CA	PRO	A	559	-51.094	14.188	-2.584	1.00	91.56	C
ATOM	4403	C	PRO	A	559	-50.219	13.109	-1.968	1.00	91.56	C
ATOM	4404	CB	PRO	A	559	-52.500	14.148	-1.979	1.00	91.56	C
ATOM	4405	O	PRO	A	559	-50.219	11.961	-2.430	1.00	91.56	O
ATOM	4406	CG	PRO	A	559	-53.438	14.492	-3.111	1.00	91.56	C
ATOM	4407	CD	PRO	A	559	-52.812	13.992	-4.387	1.00	91.56	C
ATOM	4408	N	ASP	A	560	-49.281	13.438	-1.062	1.00	90.62	N
ATOM	4409	CA	ASP	A	560	-48.406	12.562	-0.278	1.00	90.62	C
ATOM	4410	C	ASP	A	560	-47.188	12.133	-1.086	1.00	90.62	C
ATOM	4411	CB	ASP	A	560	-49.188	11.328	0.203	1.00	90.62	C
ATOM	4412	O	ASP	A	560	-46.500	11.188	-0.713	1.00	90.62	O

ATOM	4413	CG	ASP	A	560	-50.156	11.641	1.326	1.00	90.62	C
ATOM	4414	OD1	ASP	A	560	-49.906	12.570	2.117	1.00	90.62	O
ATOM	4415	OD2	ASP	A	560	-51.188	10.945	1.424	1.00	90.62	O
ATOM	4416	N	THR	A	561	-46.969	12.797	-2.164	1.00	92.69	N
ATOM	4417	CA	THR	A	561	-45.750	12.578	-2.955	1.00	92.69	C
ATOM	4418	C	THR	A	561	-44.500	13.016	-2.180	1.00	92.69	C
ATOM	4419	CB	THR	A	561	-45.812	13.336	-4.293	1.00	92.69	C
ATOM	4420	O	THR	A	561	-44.531	14.008	-1.455	1.00	92.69	O
ATOM	4421	CG2	THR	A	561	-44.531	13.117	-5.109	1.00	92.69	C
ATOM	4422	OG1	THR	A	561	-46.938	12.859	-5.051	1.00	92.69	O
ATOM	4423	N	ILE	A	562	-43.500	12.297	-2.373	1.00	93.75	N
ATOM	4424	CA	ILE	A	562	-42.250	12.594	-1.679	1.00	93.75	C
ATOM	4425	C	ILE	A	562	-41.594	13.805	-2.324	1.00	93.75	C
ATOM	4426	CB	ILE	A	562	-41.312	11.383	-1.687	1.00	93.75	C
ATOM	4427	O	ILE	A	562	-41.562	13.930	-3.551	1.00	93.75	O
ATOM	4428	CG1	ILE	A	562	-41.906	10.227	-0.873	1.00	93.75	C
ATOM	4429	CG2	ILE	A	562	-39.938	11.789	-1.149	1.00	93.75	C
ATOM	4430	CD1	ILE	A	562	-42.062	10.539	0.609	1.00	93.75	C
ATOM	4431	N	PHE	A	563	-41.094	14.672	-1.468	1.00	94.25	N
ATOM	4432	CA	PHE	A	563	-40.344	15.797	-2.012	1.00	94.25	C
ATOM	4433	C	PHE	A	563	-39.000	15.945	-1.306	1.00	94.25	C
ATOM	4434	CB	PHE	A	563	-41.156	17.094	-1.885	1.00	94.25	C
ATOM	4435	O	PHE	A	563	-38.844	15.469	-0.182	1.00	94.25	O
ATOM	4436	CG	PHE	A	563	-41.125	17.688	-0.503	1.00	94.25	C
ATOM	4437	CD1	PHE	A	563	-42.062	17.297	0.456	1.00	94.25	C
ATOM	4438	CD2	PHE	A	563	-40.188	18.641	-0.165	1.00	94.25	C
ATOM	4439	CE1	PHE	A	563	-42.031	17.844	1.735	1.00	94.25	C
ATOM	4440	CE2	PHE	A	563	-40.156	19.203	1.111	1.00	94.25	C
ATOM	4441	CZ	PHE	A	563	-41.094	18.797	2.061	1.00	94.25	C
ATOM	4442	N	ALA	A	564	-38.094	16.562	-1.949	1.00	95.12	N
ATOM	4443	CA	ALA	A	564	-36.812	16.984	-1.410	1.00	95.12	C
ATOM	4444	C	ALA	A	564	-36.406	18.375	-1.928	1.00	95.12	C
ATOM	4445	CB	ALA	A	564	-35.719	15.969	-1.761	1.00	95.12	C
ATOM	4446	O	ALA	A	564	-36.688	18.703	-3.086	1.00	95.12	O
ATOM	4447	N	ILE	A	565	-35.781	19.141	-1.115	1.00	94.38	N
ATOM	4448	CA	ILE	A	565	-35.406	20.500	-1.490	1.00	94.38	C
ATOM	4449	C	ILE	A	565	-33.875	20.594	-1.570	1.00	94.38	C
ATOM	4450	CB	ILE	A	565	-35.969	21.547	-0.493	1.00	94.38	C
ATOM	4451	O	ILE	A	565	-33.188	20.219	-0.628	1.00	94.38	O
ATOM	4452	CG1	ILE	A	565	-37.500	21.562	-0.535	1.00	94.38	C
ATOM	4453	CG2	ILE	A	565	-35.375	22.922	-0.790	1.00	94.38	C
ATOM	4454	CD1	ILE	A	565	-38.125	22.422	0.542	1.00	94.38	C
ATOM	4455	N	GLU	A	566	-33.406	21.047	-2.725	1.00	91.88	N
ATOM	4456	CA	GLU	A	566	-31.984	21.297	-2.949	1.00	91.88	C
ATOM	4457	C	GLU	A	566	-31.656	22.781	-2.725	1.00	91.88	C
ATOM	4458	CB	GLU	A	566	-31.578	20.875	-4.363	1.00	91.88	C
ATOM	4459	O	GLU	A	566	-32.281	23.656	-3.324	1.00	91.88	O
ATOM	4460	CG	GLU	A	566	-30.078	20.750	-4.555	1.00	91.88	C
ATOM	4461	CD	GLU	A	566	-29.688	20.234	-5.930	1.00	91.88	C
ATOM	4462	OE1	GLU	A	566	-28.484	20.078	-6.203	1.00	91.88	O
ATOM	4463	OE2	GLU	A	566	-30.609	19.969	-6.742	1.00	91.88	O
ATOM	4464	N	ARG	A	567	-30.641	23.047	-1.927	1.00	90.44	N
ATOM	4465	CA	ARG	A	567	-30.172	24.391	-1.640	1.00	90.44	C
ATOM	4466	C	ARG	A	567	-28.766	24.625	-2.223	1.00	90.44	C
ATOM	4467	CB	ARG	A	567	-30.156	24.641	-0.131	1.00	90.44	C
ATOM	4468	O	ARG	A	567	-28.125	23.672	-2.680	1.00	90.44	O
ATOM	4469	CG	ARG	A	567	-31.531	24.625	0.512	1.00	90.44	C
ATOM	4470	CD	ARG	A	567	-31.453	24.906	2.006	1.00	90.44	C
ATOM	4471	NE	ARG	A	567	-30.922	26.250	2.279	1.00	90.44	N
ATOM	4472	NH1	ARG	A	567	-30.766	25.938	4.562	1.00	90.44	N
ATOM	4473	NH2	ARG	A	567	-30.141	27.938	3.623	1.00	90.44	N
ATOM	4474	CZ	ARG	A	567	-30.609	26.703	3.488	1.00	90.44	C
ATOM	4475	N	THR	A	568	-28.359	25.875	-2.230	1.00	85.81	N
ATOM	4476	CA	THR	A	568	-27.031	26.219	-2.717	1.00	85.81	C

ATOM	4477	C	THR	A	568	-25.953	25.688	-1.771	1.00	85.81	C
ATOM	4478	CB	THR	A	568	-26.859	27.734	-2.883	1.00	85.81	C
ATOM	4479	O	THR	A	568	-24.812	25.438	-2.189	1.00	85.81	O
ATOM	4480	CG2	THR	A	568	-27.703	28.266	-4.039	1.00	85.81	C
ATOM	4481	OG1	THR	A	568	-27.281	28.391	-1.674	1.00	85.81	O
ATOM	4482	N	ASP	A	569	-26.266	25.500	-0.458	1.00	86.94	N
ATOM	4483	CA	ASP	A	569	-25.266	25.078	0.532	1.00	86.94	C
ATOM	4484	C	ASP	A	569	-25.453	23.609	0.898	1.00	86.94	C
ATOM	4485	CB	ASP	A	569	-25.359	25.953	1.786	1.00	86.94	C
ATOM	4486	O	ASP	A	569	-24.688	23.062	1.695	1.00	86.94	O
ATOM	4487	CG	ASP	A	569	-26.766	26.031	2.350	1.00	86.94	C
ATOM	4488	OD1	ASP	A	569	-27.719	25.547	1.692	1.00	86.94	O
ATOM	4489	OD2	ASP	A	569	-26.938	26.594	3.457	1.00	86.94	O
ATOM	4490	N	ILE	A	570	-26.578	22.922	0.387	1.00	89.69	N
ATOM	4491	CA	ILE	A	570	-26.812	21.500	0.628	1.00	89.69	C
ATOM	4492	C	ILE	A	570	-27.266	20.828	-0.663	1.00	89.69	C
ATOM	4493	CB	ILE	A	570	-27.859	21.281	1.746	1.00	89.69	C
ATOM	4494	O	ILE	A	570	-28.391	21.047	-1.120	1.00	89.69	O
ATOM	4495	CG1	ILE	A	570	-27.375	21.922	3.055	1.00	89.69	C
ATOM	4496	CG2	ILE	A	570	-28.141	19.781	1.938	1.00	89.69	C
ATOM	4497	CD1	ILE	A	570	-28.422	21.922	4.160	1.00	89.69	C
ATOM	4498	N	ALA	A	571	-26.344	20.062	-1.172	1.00	89.56	N
ATOM	4499	CA	ALA	A	571	-26.703	19.281	-2.355	1.00	89.56	C
ATOM	4500	C	ALA	A	571	-27.281	17.938	-1.968	1.00	89.56	C
ATOM	4501	CB	ALA	A	571	-25.484	19.094	-3.262	1.00	89.56	C
ATOM	4502	O	ALA	A	571	-26.953	17.391	-0.910	1.00	89.56	O
ATOM	4503	N	GLN	A	572	-28.172	17.422	-2.828	1.00	90.19	N
ATOM	4504	CA	GLN	A	572	-28.734	16.094	-2.570	1.00	90.19	C
ATOM	4505	C	GLN	A	572	-27.703	15.008	-2.828	1.00	90.19	C
ATOM	4506	CB	GLN	A	572	-29.984	15.867	-3.432	1.00	90.19	C
ATOM	4507	O	GLN	A	572	-26.891	15.117	-3.746	1.00	90.19	O
ATOM	4508	CG	GLN	A	572	-31.109	16.859	-3.172	1.00	90.19	C
ATOM	4509	CD	GLN	A	572	-31.672	16.734	-1.772	1.00	90.19	C
ATOM	4510	NE2	GLN	A	572	-32.031	17.875	-1.187	1.00	90.19	N
ATOM	4511	OE1	GLN	A	572	-31.781	15.641	-1.223	1.00	90.19	O
ATOM	4512	N	GLN	A	573	-27.781	13.969	-1.932	1.00	93.00	N
ATOM	4513	CA	GLN	A	573	-26.891	12.820	-2.098	1.00	93.00	C
ATOM	4514	C	GLN	A	573	-27.422	11.859	-3.154	1.00	93.00	C
ATOM	4515	CB	GLN	A	573	-26.703	12.086	-0.768	1.00	93.00	C
ATOM	4516	O	GLN	A	573	-28.609	11.531	-3.156	1.00	93.00	O
ATOM	4517	CG	GLN	A	573	-26.047	12.938	0.314	1.00	93.00	C
ATOM	4518	CD	GLN	A	573	-25.906	12.195	1.634	1.00	93.00	C
ATOM	4519	NE2	GLN	A	573	-24.688	12.070	2.113	1.00	93.00	N
ATOM	4520	OE1	GLN	A	573	-26.906	11.750	2.219	1.00	93.00	O
ATOM	4521	N	ARG	A	574	-26.547	11.508	-4.020	1.00	93.88	N
ATOM	4522	CA	ARG	A	574	-26.906	10.562	-5.066	1.00	93.88	C
ATOM	4523	C	ARG	A	574	-26.172	9.234	-4.895	1.00	93.88	C
ATOM	4524	CB	ARG	A	574	-26.609	11.148	-6.449	1.00	93.88	C
ATOM	4525	O	ARG	A	574	-24.969	9.227	-4.598	1.00	93.88	O
ATOM	4526	CG	ARG	A	574	-27.406	12.406	-6.762	1.00	93.88	C
ATOM	4527	CD	ARG	A	574	-27.094	12.930	-8.156	1.00	93.88	C
ATOM	4528	NE	ARG	A	574	-27.828	14.164	-8.445	1.00	93.88	N
ATOM	4529	NH1	ARG	A	574	-27.062	14.359	-10.617	1.00	93.88	N
ATOM	4530	NH2	ARG	A	574	-28.500	15.930	-9.750	1.00	93.88	N
ATOM	4531	CZ	ARG	A	574	-27.797	14.812	-9.602	1.00	93.88	C
ATOM	4532	N	TYR	A	575	-26.953	8.164	-5.055	1.00	95.19	N
ATOM	4533	CA	TYR	A	575	-26.422	6.816	-4.891	1.00	95.19	C
ATOM	4534	C	TYR	A	575	-26.844	5.914	-6.043	1.00	95.19	C
ATOM	4535	CB	TYR	A	575	-26.875	6.215	-3.559	1.00	95.19	C
ATOM	4536	O	TYR	A	575	-27.922	6.109	-6.625	1.00	95.19	O
ATOM	4537	CG	TYR	A	575	-26.234	6.848	-2.352	1.00	95.19	C
ATOM	4538	CD1	TYR	A	575	-25.094	6.277	-1.767	1.00	95.19	C
ATOM	4539	CD2	TYR	A	575	-26.734	8.016	-1.796	1.00	95.19	C
ATOM	4540	CE1	TYR	A	575	-24.500	6.855	-0.653	1.00	95.19	C

ATOM	4541	CE2	TYR	A	575	-26.141	8.602	-0.684	1.00	95.19	C
ATOM	4542	OH	TYR	A	575	-24.438	8.594	0.981	1.00	95.19	O
ATOM	4543	CZ	TYR	A	575	-25.031	8.016	-0.120	1.00	95.19	C
ATOM	4544	N	VAL	A	576	-25.922	4.996	-6.402	1.00	93.12	N
ATOM	4545	CA	VAL	A	576	-26.281	3.967	-7.375	1.00	93.12	C
ATOM	4546	C	VAL	A	576	-26.656	2.676	-6.652	1.00	93.12	C
ATOM	4547	CB	VAL	A	576	-25.125	3.707	-8.367	1.00	93.12	C
ATOM	4548	O	VAL	A	576	-25.906	2.195	-5.801	1.00	93.12	O
ATOM	4549	CG1	VAL	A	576	-25.453	2.529	-9.281	1.00	93.12	C
ATOM	4550	CG2	VAL	A	576	-24.828	4.961	-9.188	1.00	93.12	C
ATOM	4551	N	VAL	A	577	-27.781	2.119	-6.969	1.00	92.12	N
ATOM	4552	CA	VAL	A	577	-28.312	0.931	-6.320	1.00	92.12	C
ATOM	4553	C	VAL	A	577	-27.516	-0.298	-6.730	1.00	92.12	C
ATOM	4554	CB	VAL	A	577	-29.812	0.727	-6.660	1.00	92.12	C
ATOM	4555	O	VAL	A	577	-27.344	-0.564	-7.926	1.00	92.12	O
ATOM	4556	CG1	VAL	A	577	-30.328	-0.561	-6.027	1.00	92.12	C
ATOM	4557	CG2	VAL	A	577	-30.625	1.928	-6.195	1.00	92.12	C
ATOM	4558	N	THR	A	578	-27.000	-1.055	-5.750	1.00	90.88	N
ATOM	4559	CA	THR	A	578	-26.188	-2.232	-6.035	1.00	90.88	C
ATOM	4560	C	THR	A	578	-26.938	-3.510	-5.668	1.00	90.88	C
ATOM	4561	CB	THR	A	578	-24.844	-2.186	-5.281	1.00	90.88	C
ATOM	4562	O	THR	A	578	-26.625	-4.586	-6.188	1.00	90.88	O
ATOM	4563	CG2	THR	A	578	-23.969	-1.036	-5.773	1.00	90.88	C
ATOM	4564	OG1	THR	A	578	-25.094	-2.010	-3.881	1.00	90.88	O
ATOM	4565	N	GLY	A	579	-27.969	-3.363	-4.805	1.00	90.75	N
ATOM	4566	CA	GLY	A	579	-28.703	-4.543	-4.391	1.00	90.75	C
ATOM	4567	C	GLY	A	579	-30.094	-4.223	-3.848	1.00	90.75	C
ATOM	4568	O	GLY	A	579	-30.266	-3.223	-3.146	1.00	90.75	O
ATOM	4569	N	ILE	A	580	-31.047	-5.023	-4.227	1.00	91.38	N
ATOM	4570	CA	ILE	A	580	-32.406	-4.930	-3.686	1.00	91.38	C
ATOM	4571	C	ILE	A	580	-32.875	-6.309	-3.229	1.00	91.38	C
ATOM	4572	CB	ILE	A	580	-33.375	-4.344	-4.723	1.00	91.38	C
ATOM	4573	O	ILE	A	580	-32.844	-7.270	-4.000	1.00	91.38	O
ATOM	4574	CG1	ILE	A	580	-32.906	-2.973	-5.207	1.00	91.38	C
ATOM	4575	CG2	ILE	A	580	-34.812	-4.250	-4.137	1.00	91.38	C
ATOM	4576	CD1	ILE	A	580	-33.688	-2.408	-6.371	1.00	91.38	C
ATOM	4577	N	THR	A	581	-33.188	-6.363	-1.983	1.00	92.38	N
ATOM	4578	CA	THR	A	581	-33.688	-7.621	-1.449	1.00	92.38	C
ATOM	4579	C	THR	A	581	-35.094	-7.430	-0.847	1.00	92.38	C
ATOM	4580	CB	THR	A	581	-32.750	-8.195	-0.384	1.00	92.38	C
ATOM	4581	O	THR	A	581	-35.312	-6.500	-0.069	1.00	92.38	O
ATOM	4582	CG2	THR	A	581	-33.156	-9.617	-0.003	1.00	92.38	C
ATOM	4583	OG1	THR	A	581	-31.406	-8.219	-0.902	1.00	92.38	O
ATOM	4584	N	LYS	A	582	-36.000	-8.328	-1.302	1.00	90.69	N
ATOM	4585	CA	LYS	A	582	-37.344	-8.320	-0.751	1.00	90.69	C
ATOM	4586	C	LYS	A	582	-37.406	-9.102	0.558	1.00	90.69	C
ATOM	4587	CB	LYS	A	582	-38.344	-8.898	-1.759	1.00	90.69	C
ATOM	4588	O	LYS	A	582	-36.812	-10.180	0.672	1.00	90.69	O
ATOM	4589	CG	LYS	A	582	-39.781	-8.805	-1.319	1.00	90.69	C
ATOM	4590	CD	LYS	A	582	-40.719	-9.414	-2.352	1.00	90.69	C
ATOM	4591	CE	LYS	A	582	-42.188	-9.406	-1.872	1.00	90.69	C
ATOM	4592	NZ	LYS	A	582	-43.094	-10.008	-2.881	1.00	90.69	N
ATOM	4593	N	GLY	A	583	-37.969	-8.430	1.648	1.00	84.38	N
ATOM	4594	CA	GLY	A	583	-38.125	-9.117	2.924	1.00	84.38	C
ATOM	4595	C	GLY	A	583	-39.031	-10.328	2.846	1.00	84.38	C
ATOM	4596	O	GLY	A	583	-39.750	-10.516	1.857	1.00	84.38	O
ATOM	4597	N	ASP	A	584	-38.812	-11.305	3.645	1.00	80.50	N
ATOM	4598	CA	ASP	A	584	-39.625	-12.508	3.785	1.00	80.50	C
ATOM	4599	C	ASP	A	584	-40.594	-12.391	4.969	1.00	80.50	C
ATOM	4600	CB	ASP	A	584	-38.750	-13.742	3.953	1.00	80.50	C
ATOM	4601	O	ASP	A	584	-40.469	-11.492	5.797	1.00	80.50	O
ATOM	4602	CG	ASP	A	584	-37.875	-14.008	2.742	1.00	80.50	C
ATOM	4603	OD1	ASP	A	584	-38.219	-13.555	1.629	1.00	80.50	O
ATOM	4604	OD2	ASP	A	584	-36.812	-14.680	2.900	1.00	80.50	O

ATOM	4605	N	GLY	A	585	-41.719	-13.148	4.906	1.00	83.19	N
ATOM	4606	CA	GLY	A	585	-42.688	-13.250	5.996	1.00	83.19	C
ATOM	4607	C	GLY	A	585	-43.438	-11.969	6.227	1.00	83.19	C
ATOM	4608	O	GLY	A	585	-44.125	-11.477	5.320	1.00	83.19	O
ATOM	4609	N	ASP	A	586	-43.344	-11.383	7.508	1.00	78.31	N
ATOM	4610	CA	ASP	A	586	-44.062	-10.195	7.965	1.00	78.31	C
ATOM	4611	C	ASP	A	586	-43.500	-8.930	7.328	1.00	78.31	C
ATOM	4612	CB	ASP	A	586	-44.031	-10.086	9.492	1.00	78.31	C
ATOM	4613	O	ASP	A	586	-44.156	-7.867	7.387	1.00	78.31	O
ATOM	4614	CG	ASP	A	586	-44.781	-11.203	10.188	1.00	78.31	C
ATOM	4615	OD1	ASP	A	586	-45.719	-11.773	9.594	1.00	78.31	O
ATOM	4616	OD2	ASP	A	586	-44.438	-11.516	11.352	1.00	78.31	O
ATOM	4617	N	GLU	A	587	-42.344	-8.961	6.629	1.00	80.94	N
ATOM	4618	CA	GLU	A	587	-41.719	-7.832	5.953	1.00	80.94	C
ATOM	4619	C	GLU	A	587	-41.844	-7.961	4.438	1.00	80.94	C
ATOM	4620	CB	GLU	A	587	-40.250	-7.707	6.359	1.00	80.94	C
ATOM	4621	O	GLU	A	587	-40.969	-7.500	3.699	1.00	80.94	O
ATOM	4622	CG	GLU	A	587	-40.062	-7.344	7.824	1.00	80.94	C
ATOM	4623	CD	GLU	A	587	-38.594	-7.102	8.180	1.00	80.94	C
ATOM	4624	OE1	GLU	A	587	-38.312	-6.723	9.344	1.00	80.94	O
ATOM	4625	OE2	GLU	A	587	-37.719	-7.289	7.297	1.00	80.94	O
ATOM	4626	N	GLU	A	588	-42.906	-8.844	3.957	1.00	83.50	N
ATOM	4627	CA	GLU	A	588	-43.062	-9.133	2.533	1.00	83.50	C
ATOM	4628	C	GLU	A	588	-43.188	-7.848	1.719	1.00	83.50	C
ATOM	4629	CB	GLU	A	588	-44.281	-10.023	2.287	1.00	83.50	C
ATOM	4630	O	GLU	A	588	-42.812	-7.824	0.537	1.00	83.50	O
ATOM	4631	CG	GLU	A	588	-44.469	-10.430	0.831	1.00	83.50	C
ATOM	4632	CD	GLU	A	588	-45.656	-11.328	0.603	1.00	83.50	C
ATOM	4633	OE1	GLU	A	588	-45.875	-11.781	-0.544	1.00	83.50	O
ATOM	4634	OE2	GLU	A	588	-46.406	-11.578	1.580	1.00	83.50	O
ATOM	4635	N	PHE	A	589	-43.594	-6.699	2.281	1.00	86.62	N
ATOM	4636	CA	PHE	A	589	-43.750	-5.465	1.522	1.00	86.62	C
ATOM	4637	C	PHE	A	589	-42.625	-4.477	1.850	1.00	86.62	C
ATOM	4638	CB	PHE	A	589	-45.125	-4.832	1.810	1.00	86.62	C
ATOM	4639	O	PHE	A	589	-42.781	-3.275	1.608	1.00	86.62	O
ATOM	4640	CG	PHE	A	589	-46.281	-5.695	1.413	1.00	86.62	C
ATOM	4641	CD1	PHE	A	589	-46.531	-5.977	0.076	1.00	86.62	C
ATOM	4642	CD2	PHE	A	589	-47.125	-6.227	2.377	1.00	86.62	C
ATOM	4643	CE1	PHE	A	589	-47.625	-6.777	-0.295	1.00	86.62	C
ATOM	4644	CE2	PHE	A	589	-48.219	-7.027	2.016	1.00	86.62	C
ATOM	4645	CZ	PHE	A	589	-48.438	-7.301	0.679	1.00	86.62	C
ATOM	4646	N	THR	A	590	-41.594	-5.000	2.465	1.00	92.88	N
ATOM	4647	CA	THR	A	590	-40.406	-4.215	2.768	1.00	92.88	C
ATOM	4648	C	THR	A	590	-39.219	-4.629	1.876	1.00	92.88	C
ATOM	4649	CB	THR	A	590	-40.000	-4.359	4.246	1.00	92.88	C
ATOM	4650	O	THR	A	590	-38.969	-5.824	1.695	1.00	92.88	O
ATOM	4651	CG2	THR	A	590	-38.812	-3.455	4.586	1.00	92.88	C
ATOM	4652	OG1	THR	A	590	-41.094	-4.004	5.078	1.00	92.88	O
ATOM	4653	N	TYR	A	591	-38.656	-3.648	1.368	1.00	94.19	N
ATOM	4654	CA	TYR	A	591	-37.500	-3.877	0.480	1.00	94.19	C
ATOM	4655	C	TYR	A	591	-36.250	-3.256	1.047	1.00	94.19	C
ATOM	4656	CB	TYR	A	591	-37.781	-3.305	-0.913	1.00	94.19	C
ATOM	4657	O	TYR	A	591	-36.219	-2.072	1.389	1.00	94.19	O
ATOM	4658	CG	TYR	A	591	-38.938	-3.971	-1.618	1.00	94.19	C
ATOM	4659	CD1	TYR	A	591	-38.719	-4.961	-2.574	1.00	94.19	C
ATOM	4660	CD2	TYR	A	591	-40.250	-3.611	-1.332	1.00	94.19	C
ATOM	4661	CE1	TYR	A	591	-39.781	-5.574	-3.229	1.00	94.19	C
ATOM	4662	CE2	TYR	A	591	-41.344	-4.219	-1.980	1.00	94.19	C
ATOM	4663	OH	TYR	A	591	-42.156	-5.805	-3.570	1.00	94.19	O
ATOM	4664	CZ	TYR	A	591	-41.094	-5.199	-2.926	1.00	94.19	C
ATOM	4665	N	ASN	A	592	-35.188	-4.023	1.191	1.00	94.38	N
ATOM	4666	CA	ASN	A	592	-33.906	-3.539	1.625	1.00	94.38	C
ATOM	4667	C	ASN	A	592	-33.031	-3.105	0.440	1.00	94.38	C
ATOM	4668	CB	ASN	A	592	-33.156	-4.609	2.443	1.00	94.38	C

ATOM	4669	O	ASN	A	592	-32.750	-3.902	-0.457	1.00	94.38	O
ATOM	4670	CG	ASN	A	592	-33.844	-4.879	3.777	1.00	94.38	C
ATOM	4671	ND2	ASN	A	592	-33.844	-6.137	4.203	1.00	94.38	N
ATOM	4672	OD1	ASN	A	592	-34.375	-3.963	4.418	1.00	94.38	O
ATOM	4673	N	ILE	A	593	-32.688	-1.855	0.487	1.00	95.19	N
ATOM	4674	CA	ILE	A	593	-31.922	-1.305	-0.625	1.00	95.19	C
ATOM	4675	C	ILE	A	593	-30.484	-1.013	-0.170	1.00	95.19	C
ATOM	4676	CB	ILE	A	593	-32.562	-0.023	-1.185	1.00	95.19	C
ATOM	4677	O	ILE	A	593	-30.281	-0.405	0.881	1.00	95.19	O
ATOM	4678	CG1	ILE	A	593	-33.969	-0.336	-1.742	1.00	95.19	C
ATOM	4679	CG2	ILE	A	593	-31.688	0.614	-2.258	1.00	95.19	C
ATOM	4680	CD1	ILE	A	593	-34.688	0.874	-2.320	1.00	95.19	C
ATOM	4681	N	THR	A	594	-29.531	-1.477	-0.914	1.00	95.88	N
ATOM	4682	CA	THR	A	594	-28.109	-1.149	-0.761	1.00	95.88	C
ATOM	4683	C	THR	A	594	-27.609	-0.359	-1.965	1.00	95.88	C
ATOM	4684	CB	THR	A	594	-27.266	-2.422	-0.585	1.00	95.88	C
ATOM	4685	O	THR	A	594	-27.844	-0.751	-3.111	1.00	95.88	O
ATOM	4686	CG2	THR	A	594	-25.797	-2.072	-0.330	1.00	95.88	C
ATOM	4687	OG1	THR	A	594	-27.766	-3.174	0.526	1.00	95.88	O
ATOM	4688	N	ALA	A	595	-26.984	0.786	-1.621	1.00	95.31	N
ATOM	4689	CA	ALA	A	595	-26.516	1.647	-2.707	1.00	95.31	C
ATOM	4690	C	ALA	A	595	-25.203	2.312	-2.352	1.00	95.31	C
ATOM	4691	CB	ALA	A	595	-27.578	2.705	-3.029	1.00	95.31	C
ATOM	4692	O	ALA	A	595	-24.875	2.477	-1.174	1.00	95.31	O
ATOM	4693	N	VAL	A	596	-24.391	2.684	-3.434	1.00	94.50	N
ATOM	4694	CA	VAL	A	596	-23.094	3.303	-3.250	1.00	94.50	C
ATOM	4695	C	VAL	A	596	-23.109	4.723	-3.816	1.00	94.50	C
ATOM	4696	CB	VAL	A	596	-21.969	2.475	-3.918	1.00	94.50	C
ATOM	4697	O	VAL	A	596	-23.812	5.000	-4.789	1.00	94.50	O
ATOM	4698	CG1	VAL	A	596	-20.625	3.201	-3.822	1.00	94.50	C
ATOM	4699	CG2	VAL	A	596	-21.875	1.090	-3.277	1.00	94.50	C
ATOM	4700	N	GLU	A	597	-22.375	5.559	-3.172	1.00	94.50	N
ATOM	4701	CA	GLU	A	597	-22.344	6.977	-3.531	1.00	94.50	C
ATOM	4702	C	GLU	A	597	-22.031	7.164	-5.012	1.00	94.50	C
ATOM	4703	CB	GLU	A	597	-21.328	7.730	-2.670	1.00	94.50	C
ATOM	4704	O	GLU	A	597	-21.125	6.504	-5.547	1.00	94.50	O
ATOM	4705	CG	GLU	A	597	-21.391	9.242	-2.828	1.00	94.50	C
ATOM	4706	CD	GLU	A	597	-20.469	9.984	-1.876	1.00	94.50	C
ATOM	4707	OE1	GLU	A	597	-20.547	11.227	-1.793	1.00	94.50	O
ATOM	4708	OE2	GLU	A	597	-19.656	9.312	-1.207	1.00	94.50	O
ATOM	4709	N	TYR	A	598	-22.781	8.062	-5.652	1.00	93.06	N
ATOM	4710	CA	TYR	A	598	-22.672	8.359	-7.078	1.00	93.06	C
ATOM	4711	C	TYR	A	598	-22.266	9.812	-7.305	1.00	93.06	C
ATOM	4712	CB	TYR	A	598	-24.000	8.070	-7.789	1.00	93.06	C
ATOM	4713	O	TYR	A	598	-22.859	10.727	-6.723	1.00	93.06	O
ATOM	4714	CG	TYR	A	598	-24.000	8.453	-9.250	1.00	93.06	C
ATOM	4715	CD1	TYR	A	598	-24.734	9.547	-9.695	1.00	93.06	C
ATOM	4716	CD2	TYR	A	598	-23.266	7.727	-10.180	1.00	93.06	C
ATOM	4717	CE1	TYR	A	598	-24.750	9.906	-11.039	1.00	93.06	C
ATOM	4718	CE2	TYR	A	598	-23.281	8.078	-11.523	1.00	93.06	C
ATOM	4719	OH	TYR	A	598	-24.016	9.516	-13.273	1.00	93.06	O
ATOM	4720	CZ	TYR	A	598	-24.016	9.164	-11.945	1.00	93.06	C
ATOM	4721	N	ASP	A	599	-21.266	10.008	-8.133	1.00	90.75	N
ATOM	4722	CA	ASP	A	599	-20.812	11.344	-8.508	1.00	90.75	C
ATOM	4723	C	ASP	A	599	-20.984	11.578	-10.008	1.00	90.75	C
ATOM	4724	CB	ASP	A	599	-19.344	11.547	-8.117	1.00	90.75	C
ATOM	4725	O	ASP	A	599	-20.234	11.039	-10.820	1.00	90.75	O
ATOM	4726	CG	ASP	A	599	-18.844	12.953	-8.406	1.00	90.75	C
ATOM	4727	OD1	ASP	A	599	-19.656	13.820	-8.805	1.00	90.75	O
ATOM	4728	OD2	ASP	A	599	-17.625	13.195	-8.234	1.00	90.75	O
ATOM	4729	N	PRO	A	600	-21.922	12.438	-10.359	1.00	89.19	N
ATOM	4730	CA	PRO	A	600	-22.172	12.680	-11.781	1.00	89.19	C
ATOM	4731	C	PRO	A	600	-21.062	13.477	-12.445	1.00	89.19	C
ATOM	4732	CB	PRO	A	600	-23.484	13.469	-11.781	1.00	89.19	C

ATOM	4733	O	PRO	A	600	-20.906	13.438	-13.672	1.00	89.19	O
ATOM	4734	CG	PRO	A	600	-23.562	14.078	-10.414	1.00	89.19	C
ATOM	4735	CD	PRO	A	600	-22.906	13.141	-9.438	1.00	89.19	C
ATOM	4736	N	ASN	A	601	-20.250	14.180	-11.641	1.00	89.50	N
ATOM	4737	CA	ASN	A	601	-19.203	15.055	-12.164	1.00	89.50	C
ATOM	4738	C	ASN	A	601	-17.938	14.266	-12.500	1.00	89.50	C
ATOM	4739	CB	ASN	A	601	-18.891	16.172	-11.164	1.00	89.50	C
ATOM	4740	O	ASN	A	601	-17.031	14.797	-13.133	1.00	89.50	O
ATOM	4741	CG	ASN	A	601	-20.047	17.141	-10.992	1.00	89.50	C
ATOM	4742	ND2	ASN	A	601	-20.141	17.734	-9.805	1.00	89.50	N
ATOM	4743	OD1	ASN	A	601	-20.844	17.344	-11.906	1.00	89.50	O
ATOM	4744	N	LYS	A	602	-17.812	13.023	-12.086	1.00	91.06	N
ATOM	4745	CA	LYS	A	602	-16.594	12.242	-12.266	1.00	91.06	C
ATOM	4746	C	LYS	A	602	-16.297	12.016	-13.742	1.00	91.06	C
ATOM	4747	CB	LYS	A	602	-16.719	10.898	-11.539	1.00	91.06	C
ATOM	4748	O	LYS	A	602	-15.141	11.914	-14.141	1.00	91.06	O
ATOM	4749	CG	LYS	A	602	-17.734	9.953	-12.156	1.00	91.06	C
ATOM	4750	CD	LYS	A	602	-17.797	8.625	-11.406	1.00	91.06	C
ATOM	4751	CE	LYS	A	602	-18.875	7.707	-11.969	1.00	91.06	C
ATOM	4752	NZ	LYS	A	602	-19.016	6.461	-11.164	1.00	91.06	N
ATOM	4753	N	TYR	A	603	-17.281	11.906	-14.594	1.00	91.75	N
ATOM	4754	CA	TYR	A	603	-17.109	11.586	-16.000	1.00	91.75	C
ATOM	4755	C	TYR	A	603	-16.375	12.703	-16.734	1.00	91.75	C
ATOM	4756	CB	TYR	A	603	-18.469	11.336	-16.672	1.00	91.75	C
ATOM	4757	O	TYR	A	603	-15.453	12.453	-17.516	1.00	91.75	O
ATOM	4758	CG	TYR	A	603	-19.266	10.242	-16.016	1.00	91.75	C
ATOM	4759	CD1	TYR	A	603	-18.953	8.898	-16.203	1.00	91.75	C
ATOM	4760	CD2	TYR	A	603	-20.359	10.547	-15.203	1.00	91.75	C
ATOM	4761	CE1	TYR	A	603	-19.688	7.891	-15.609	1.00	91.75	C
ATOM	4762	CE2	TYR	A	603	-21.109	9.547	-14.602	1.00	91.75	C
ATOM	4763	OH	TYR	A	603	-21.500	7.223	-14.211	1.00	91.75	O
ATOM	4764	CZ	TYR	A	603	-20.766	8.219	-14.805	1.00	91.75	C
ATOM	4765	N	ASP	A	604	-16.719	13.961	-16.469	1.00	90.69	N
ATOM	4766	CA	ASP	A	604	-16.000	15.094	-17.062	1.00	90.69	C
ATOM	4767	C	ASP	A	604	-14.578	15.172	-16.531	1.00	90.69	C
ATOM	4768	CB	ASP	A	604	-16.750	16.406	-16.781	1.00	90.69	C
ATOM	4769	O	ASP	A	604	-13.656	15.531	-17.266	1.00	90.69	O
ATOM	4770	CG	ASP	A	604	-17.984	16.578	-17.641	1.00	90.69	C
ATOM	4771	OD1	ASP	A	604	-18.156	15.820	-18.625	1.00	90.69	O
ATOM	4772	OD2	ASP	A	604	-18.797	17.484	-17.328	1.00	90.69	O
ATOM	4773	N	GLU	A	605	-14.414	14.773	-15.305	1.00	89.50	N
ATOM	4774	CA	GLU	A	605	-13.078	14.742	-14.719	1.00	89.50	C
ATOM	4775	C	GLU	A	605	-12.203	13.680	-15.383	1.00	89.50	C
ATOM	4776	CB	GLU	A	605	-13.156	14.492	-13.211	1.00	89.50	C
ATOM	4777	O	GLU	A	605	-11.023	13.914	-15.633	1.00	89.50	O
ATOM	4778	CG	GLU	A	605	-13.758	15.641	-12.422	1.00	89.50	C
ATOM	4779	CD	GLU	A	605	-13.797	15.391	-10.922	1.00	89.50	C
ATOM	4780	OE1	GLU	A	605	-14.242	16.281	-10.164	1.00	89.50	O
ATOM	4781	OE2	GLU	A	605	-13.383	14.289	-10.500	1.00	89.50	O
ATOM	4782	N	ILE	A	606	-12.789	12.602	-15.633	1.00	92.25	N
ATOM	4783	CA	ILE	A	606	-12.086	11.484	-16.250	1.00	92.25	C
ATOM	4784	C	ILE	A	606	-11.766	11.805	-17.703	1.00	92.25	C
ATOM	4785	CB	ILE	A	606	-12.914	10.180	-16.156	1.00	92.25	C
ATOM	4786	O	ILE	A	606	-10.625	11.656	-18.141	1.00	92.25	O
ATOM	4787	CG1	ILE	A	606	-13.047	9.727	-14.703	1.00	92.25	C
ATOM	4788	CG2	ILE	A	606	-12.289	9.086	-17.016	1.00	92.25	C
ATOM	4789	CD1	ILE	A	606	-14.133	8.688	-14.469	1.00	92.25	C
ATOM	4790	N	ASP	A	607	-12.695	12.312	-18.453	1.00	90.94	N
ATOM	4791	CA	ASP	A	607	-12.562	12.492	-19.891	1.00	90.94	C
ATOM	4792	C	ASP	A	607	-11.852	13.805	-20.219	1.00	90.94	C
ATOM	4793	CB	ASP	A	607	-13.938	12.453	-20.562	1.00	90.94	C
ATOM	4794	O	ASP	A	607	-11.008	13.852	-21.109	1.00	90.94	O
ATOM	4795	CG	ASP	A	607	-14.547	11.062	-20.594	1.00	90.94	C
ATOM	4796	OD1	ASP	A	607	-13.812	10.070	-20.391	1.00	90.94	O

ATOM	4797	OD2	ASP	A	607	-15.773	10.961	-20.812	1.00	90.94	O
ATOM	4798	N	TYR	A	608	-12.078	14.883	-19.391	1.00	88.56	N
ATOM	4799	CA	TYR	A	608	-11.648	16.219	-19.797	1.00	88.56	C
ATOM	4800	C	TYR	A	608	-10.672	16.812	-18.781	1.00	88.56	C
ATOM	4801	CB	TYR	A	608	-12.852	17.141	-19.953	1.00	88.56	C
ATOM	4802	O	TYR	A	608	-10.094	17.875	-19.016	1.00	88.56	O
ATOM	4803	CG	TYR	A	608	-13.875	16.641	-20.953	1.00	88.56	C
ATOM	4804	CD1	TYR	A	608	-13.641	16.719	-22.328	1.00	88.56	C
ATOM	4805	CD2	TYR	A	608	-15.078	16.094	-20.516	1.00	88.56	C
ATOM	4806	CE1	TYR	A	608	-14.578	16.266	-23.234	1.00	88.56	C
ATOM	4807	CE2	TYR	A	608	-16.016	15.633	-21.438	1.00	88.56	C
ATOM	4808	OH	TYR	A	608	-16.703	15.266	-23.688	1.00	88.56	O
ATOM	4809	CZ	TYR	A	608	-15.766	15.727	-22.781	1.00	88.56	C
ATOM	4810	N	GLY	A	609	-10.523	16.047	-17.641	1.00	80.56	N
ATOM	4811	CA	GLY	A	609	-9.594	16.531	-16.625	1.00	80.56	C
ATOM	4812	C	GLY	A	609	-10.078	17.766	-15.906	1.00	80.56	C
ATOM	4813	O	GLY	A	609	-9.273	18.594	-15.461	1.00	80.56	O
ATOM	4814	N	VAL	A	610	-11.430	18.031	-15.953	1.00	82.12	N
ATOM	4815	CA	VAL	A	610	-12.070	19.188	-15.320	1.00	82.12	C
ATOM	4816	C	VAL	A	610	-12.258	18.922	-13.828	1.00	82.12	C
ATOM	4817	CB	VAL	A	610	-13.430	19.500	-15.977	1.00	82.12	C
ATOM	4818	O	VAL	A	610	-12.594	17.797	-13.430	1.00	82.12	O
ATOM	4819	CG1	VAL	A	610	-14.086	20.719	-15.297	1.00	82.12	C
ATOM	4820	CG2	VAL	A	610	-13.258	19.766	-17.469	1.00	82.12	C
ATOM	4821	N	ASN	A	611	-11.750	19.828	-13.031	1.00	78.62	N
ATOM	4822	CA	ASN	A	611	-11.961	19.750	-11.594	1.00	78.62	C
ATOM	4823	C	ASN	A	611	-13.188	20.547	-11.164	1.00	78.62	C
ATOM	4824	CB	ASN	A	611	-10.719	20.234	-10.836	1.00	78.62	C
ATOM	4825	O	ASN	A	611	-13.188	21.781	-11.234	1.00	78.62	O
ATOM	4826	CG	ASN	A	611	-10.758	19.891	-9.359	1.00	78.62	C
ATOM	4827	ND2	ASN	A	611	-9.695	20.234	-8.648	1.00	78.62	N
ATOM	4828	OD1	ASN	A	611	-11.734	19.328	-8.867	1.00	78.62	O
ATOM	4829	N	ILE	A	612	-14.258	19.828	-10.844	1.00	81.12	N
ATOM	4830	CA	ILE	A	612	-15.508	20.453	-10.414	1.00	81.12	C
ATOM	4831	C	ILE	A	612	-15.672	20.297	-8.906	1.00	81.12	C
ATOM	4832	CB	ILE	A	612	-16.719	19.844	-11.148	1.00	81.12	C
ATOM	4833	O	ILE	A	612	-15.602	19.172	-8.383	1.00	81.12	O
ATOM	4834	CG1	ILE	A	612	-16.547	19.969	-12.664	1.00	81.12	C
ATOM	4835	CG2	ILE	A	612	-18.016	20.531	-10.695	1.00	81.12	C
ATOM	4836	CD1	ILE	A	612	-17.594	19.219	-13.477	1.00	81.12	C
ATOM	4837	N	ASP	A	613	-15.680	21.406	-8.219	1.00	80.62	N
ATOM	4838	CA	ASP	A	613	-15.875	21.375	-6.770	1.00	80.62	C
ATOM	4839	C	ASP	A	613	-17.281	20.891	-6.422	1.00	80.62	C
ATOM	4840	CB	ASP	A	613	-15.633	22.750	-6.160	1.00	80.62	C
ATOM	4841	O	ASP	A	613	-18.266	21.375	-6.988	1.00	80.62	O
ATOM	4842	CG	ASP	A	613	-14.172	23.188	-6.227	1.00	80.62	C
ATOM	4843	OD1	ASP	A	613	-13.281	22.312	-6.207	1.00	80.62	O
ATOM	4844	OD2	ASP	A	613	-13.922	24.406	-6.297	1.00	80.62	O
ATOM	4845	N	ASP	A	614	-17.266	19.750	-5.633	1.00	82.19	N
ATOM	4846	CA	ASP	A	614	-18.562	19.219	-5.230	1.00	82.19	C
ATOM	4847	C	ASP	A	614	-19.250	20.141	-4.234	1.00	82.19	C
ATOM	4848	CB	ASP	A	614	-18.406	17.828	-4.621	1.00	82.19	C
ATOM	4849	O	ASP	A	614	-18.594	20.766	-3.393	1.00	82.19	O
ATOM	4850	CG	ASP	A	614	-17.938	16.781	-5.629	1.00	82.19	C
ATOM	4851	OD1	ASP	A	614	-18.391	16.828	-6.793	1.00	82.19	O
ATOM	4852	OD2	ASP	A	614	-17.125	15.922	-5.254	1.00	82.19	O
ATOM	4853	N	ARG	A	615	-20.578	20.359	-4.391	1.00	84.50	N
ATOM	4854	CA	ARG	A	615	-21.375	21.094	-3.414	1.00	84.50	C
ATOM	4855	C	ARG	A	615	-21.531	20.297	-2.123	1.00	84.50	C
ATOM	4856	CB	ARG	A	615	-22.766	21.422	-3.986	1.00	84.50	C
ATOM	4857	O	ARG	A	615	-21.594	19.062	-2.152	1.00	84.50	O
ATOM	4858	CG	ARG	A	615	-22.719	22.359	-5.176	1.00	84.50	C
ATOM	4859	CD	ARG	A	615	-24.078	22.438	-5.875	1.00	84.50	C
ATOM	4860	NE	ARG	A	615	-25.125	22.875	-4.961	1.00	84.50	N

ATOM	4861	NH1	ARG	A	615	-26.828	22.672	-6.508	1.00	84.50	N
ATOM	4862	NH2	ARG	A	615	-27.297	23.375	-4.375	1.00	84.50	N
ATOM	4863	CZ	ARG	A	615	-26.422	22.969	-5.285	1.00	84.50	C
ATOM	4864	N	PRO	A	616	-21.391	20.953	-0.993	1.00	89.00	N
ATOM	4865	CA	PRO	A	616	-21.500	20.219	0.269	1.00	89.00	C
ATOM	4866	C	PRO	A	616	-22.828	19.516	0.429	1.00	89.00	C
ATOM	4867	CB	PRO	A	616	-21.328	21.312	1.329	1.00	89.00	C
ATOM	4868	O	PRO	A	616	-23.859	20.000	-0.034	1.00	89.00	O
ATOM	4869	CG	PRO	A	616	-21.750	22.578	0.646	1.00	89.00	C
ATOM	4870	CD	PRO	A	616	-21.484	22.438	-0.826	1.00	89.00	C
ATOM	4871	N	THR	A	617	-22.812	18.250	0.958	1.00	89.25	N
ATOM	4872	CA	THR	A	617	-24.031	17.500	1.182	1.00	89.25	C
ATOM	4873	C	THR	A	617	-24.547	17.703	2.604	1.00	89.25	C
ATOM	4874	CB	THR	A	617	-23.828	15.992	0.925	1.00	89.25	C
ATOM	4875	O	THR	A	617	-25.656	17.281	2.939	1.00	89.25	O
ATOM	4876	CG2	THR	A	617	-23.469	15.727	-0.533	1.00	89.25	C
ATOM	4877	OG1	THR	A	617	-22.766	15.516	1.763	1.00	89.25	O
ATOM	4878	N	SER	A	618	-23.672	18.328	3.457	1.00	88.56	N
ATOM	4879	CA	SER	A	618	-24.094	18.531	4.840	1.00	88.56	C
ATOM	4880	C	SER	A	618	-23.641	19.906	5.344	1.00	88.56	C
ATOM	4881	CB	SER	A	618	-23.531	17.438	5.746	1.00	88.56	C
ATOM	4882	O	SER	A	618	-22.562	20.391	4.977	1.00	88.56	O
ATOM	4883	OG	SER	A	618	-23.812	17.719	7.109	1.00	88.56	O
ATOM	4884	N	ILE	A	619	-24.453	20.594	6.094	1.00	85.56	N
ATOM	4885	CA	ILE	A	619	-24.078	21.859	6.715	1.00	85.56	C
ATOM	4886	C	ILE	A	619	-23.516	21.609	8.109	1.00	85.56	C
ATOM	4887	CB	ILE	A	619	-25.281	22.844	6.793	1.00	85.56	C
ATOM	4888	O	ILE	A	619	-23.094	22.531	8.797	1.00	85.56	O
ATOM	4889	CG1	ILE	A	619	-26.422	22.203	7.582	1.00	85.56	C
ATOM	4890	CG2	ILE	A	619	-25.734	23.250	5.391	1.00	85.56	C
ATOM	4891	CD1	ILE	A	619	-27.500	23.203	8.023	1.00	85.56	C
ATOM	4892	N	VAL	A	620	-23.516	20.219	8.367	1.00	79.38	N
ATOM	4893	CA	VAL	A	620	-23.000	19.828	9.664	1.00	79.38	C
ATOM	4894	C	VAL	A	620	-21.469	19.766	9.617	1.00	79.38	C
ATOM	4895	CB	VAL	A	620	-23.562	18.453	10.117	1.00	79.38	C
ATOM	4896	O	VAL	A	620	-20.891	19.047	8.797	1.00	79.38	O
ATOM	4897	CG1	VAL	A	620	-23.016	18.062	11.484	1.00	79.38	C
ATOM	4898	CG2	VAL	A	620	-25.094	18.484	10.133	1.00	79.38	C
ATOM	4899	N	GLN	A	621	-20.844	20.641	10.148	1.00	72.88	N
ATOM	4900	CA	GLN	A	621	-19.391	20.719	10.203	1.00	72.88	C
ATOM	4901	C	GLN	A	621	-18.875	20.438	11.609	1.00	72.88	C
ATOM	4902	CB	GLN	A	621	-18.906	22.094	9.734	1.00	72.88	C
ATOM	4903	O	GLN	A	621	-18.562	21.359	12.367	1.00	72.88	O
ATOM	4904	CG	GLN	A	621	-19.172	22.375	8.266	1.00	72.88	C
ATOM	4905	CD	GLN	A	621	-18.734	23.766	7.840	1.00	72.88	C
ATOM	4906	NE2	GLN	A	621	-18.594	23.969	6.535	1.00	72.88	N
ATOM	4907	OE1	GLN	A	621	-18.516	24.641	8.680	1.00	72.88	O
ATOM	4908	N	PRO	A	622	-18.750	19.125	11.875	1.00	67.31	N
ATOM	4909	CA	PRO	A	622	-18.312	18.891	13.250	1.00	67.31	C
ATOM	4910	C	PRO	A	622	-16.891	19.375	13.500	1.00	67.31	C
ATOM	4911	CB	PRO	A	622	-18.406	17.359	13.391	1.00	67.31	C
ATOM	4912	O	PRO	A	622	-16.500	19.609	14.648	1.00	67.31	O
ATOM	4913	CG	PRO	A	622	-18.469	16.859	11.984	1.00	67.31	C
ATOM	4914	CD	PRO	A	622	-18.969	17.969	11.102	1.00	67.31	C
ATOM	4915	N	ASP	A	623	-16.172	19.609	12.328	1.00	68.94	N
ATOM	4916	CA	ASP	A	623	-14.758	19.922	12.516	1.00	68.94	C
ATOM	4917	C	ASP	A	623	-14.555	21.422	12.750	1.00	68.94	C
ATOM	4918	CB	ASP	A	623	-13.938	19.469	11.305	1.00	68.94	C
ATOM	4919	O	ASP	A	623	-13.484	21.844	13.195	1.00	68.94	O
ATOM	4920	CG	ASP	A	623	-13.953	17.953	11.125	1.00	68.94	C
ATOM	4921	OD1	ASP	A	623	-14.055	17.219	12.125	1.00	68.94	O
ATOM	4922	OD2	ASP	A	623	-13.852	17.500	9.961	1.00	68.94	O
ATOM	4923	N	ILE	A	624	-15.617	22.234	12.375	1.00	75.62	N
ATOM	4924	CA	ILE	A	624	-15.531	23.672	12.586	1.00	75.62	C

ATOM	4925	C	ILE	A	624	-16.453	24.078	13.727	1.00	75.62	C
ATOM	4926	CB	ILE	A	624	-15.883	24.469	11.305	1.00	75.62	C
ATOM	4927	O	ILE	A	624	-17.688	24.000	13.602	1.00	75.62	O
ATOM	4928	CG1	ILE	A	624	-14.969	24.031	10.148	1.00	75.62	C
ATOM	4929	CG2	ILE	A	624	-15.781	25.969	11.555	1.00	75.62	C
ATOM	4930	CD1	ILE	A	624	-15.367	24.609	8.797	1.00	75.62	C
ATOM	4931	N	LEU	A	625	-15.945	24.234	14.930	1.00	84.06	N
ATOM	4932	CA	LEU	A	625	-16.688	24.562	16.141	1.00	84.06	C
ATOM	4933	C	LEU	A	625	-16.641	26.062	16.422	1.00	84.06	C
ATOM	4934	CB	LEU	A	625	-16.141	23.781	17.344	1.00	84.06	C
ATOM	4935	O	LEU	A	625	-15.570	26.672	16.328	1.00	84.06	O
ATOM	4936	CG	LEU	A	625	-16.328	22.266	17.297	1.00	84.06	C
ATOM	4937	CD1	LEU	A	625	-15.305	21.578	18.203	1.00	84.06	C
ATOM	4938	CD2	LEU	A	625	-17.750	21.891	17.719	1.00	84.06	C
ATOM	4939	N	PRO	A	626	-17.859	26.750	16.531	1.00	86.69	N
ATOM	4940	CA	PRO	A	626	-17.797	28.141	16.984	1.00	86.69	C
ATOM	4941	C	PRO	A	626	-17.266	28.297	18.406	1.00	86.69	C
ATOM	4942	CB	PRO	A	626	-19.250	28.609	16.891	1.00	86.69	C
ATOM	4943	O	PRO	A	626	-17.281	27.328	19.172	1.00	86.69	O
ATOM	4944	CG	PRO	A	626	-20.062	27.344	16.953	1.00	86.69	C
ATOM	4945	CD	PRO	A	626	-19.188	26.203	16.500	1.00	86.69	C
ATOM	4946	N	ALA	A	627	-16.703	29.406	18.656	1.00	89.94	N
ATOM	4947	CA	ALA	A	627	-16.281	29.672	20.031	1.00	89.94	C
ATOM	4948	C	ALA	A	627	-17.469	29.828	20.953	1.00	89.94	C
ATOM	4949	CB	ALA	A	627	-15.414	30.938	20.078	1.00	89.94	C
ATOM	4950	O	ALA	A	627	-18.516	30.328	20.547	1.00	89.94	O
ATOM	4951	N	PRO	A	628	-17.484	29.250	22.109	1.00	92.94	N
ATOM	4952	CA	PRO	A	628	-18.562	29.406	23.094	1.00	92.94	C
ATOM	4953	C	PRO	A	628	-18.953	30.875	23.312	1.00	92.94	C
ATOM	4954	CB	PRO	A	628	-17.984	28.812	24.375	1.00	92.94	C
ATOM	4955	O	PRO	A	628	-18.094	31.750	23.266	1.00	92.94	O
ATOM	4956	CG	PRO	A	628	-16.906	27.875	23.891	1.00	92.94	C
ATOM	4957	CD	PRO	A	628	-16.328	28.438	22.625	1.00	92.94	C
ATOM	4958	N	GLN	A	629	-20.266	31.234	23.359	1.00	91.81	N
ATOM	4959	CA	GLN	A	629	-20.766	32.594	23.609	1.00	91.81	C
ATOM	4960	C	GLN	A	629	-21.234	32.750	25.062	1.00	91.81	C
ATOM	4961	CB	GLN	A	629	-21.906	32.906	22.656	1.00	91.81	C
ATOM	4962	O	GLN	A	629	-21.453	31.734	25.750	1.00	91.81	O
ATOM	4963	CG	GLN	A	629	-21.469	33.000	21.188	1.00	91.81	C
ATOM	4964	CD	GLN	A	629	-22.625	33.344	20.266	1.00	91.81	C
ATOM	4965	NE2	GLN	A	629	-22.281	33.750	19.031	1.00	91.81	N
ATOM	4966	OE1	GLN	A	629	-23.797	33.250	20.625	1.00	91.81	O
ATOM	4967	N	ASN	A	630	-21.266	34.000	25.609	1.00	90.25	N
ATOM	4968	CA	ASN	A	630	-21.797	34.375	26.906	1.00	90.25	C
ATOM	4969	C	ASN	A	630	-20.969	33.781	28.047	1.00	90.25	C
ATOM	4970	CB	ASN	A	630	-23.266	33.969	27.031	1.00	90.25	C
ATOM	4971	O	ASN	A	630	-21.531	33.219	28.984	1.00	90.25	O
ATOM	4972	CG	ASN	A	630	-24.156	34.656	26.031	1.00	90.25	C
ATOM	4973	ND2	ASN	A	630	-25.094	33.938	25.469	1.00	90.25	N
ATOM	4974	OD1	ASN	A	630	-24.000	35.844	25.781	1.00	90.25	O
ATOM	4975	N	VAL	A	631	-19.672	33.844	27.812	1.00	92.44	N
ATOM	4976	CA	VAL	A	631	-18.781	33.438	28.906	1.00	92.44	C
ATOM	4977	C	VAL	A	631	-18.984	34.375	30.094	1.00	92.44	C
ATOM	4978	CB	VAL	A	631	-17.312	33.438	28.453	1.00	92.44	C
ATOM	4979	O	VAL	A	631	-18.906	35.594	29.953	1.00	92.44	O
ATOM	4980	CG1	VAL	A	631	-16.406	33.000	29.609	1.00	92.44	C
ATOM	4981	CG2	VAL	A	631	-17.109	32.531	27.234	1.00	92.44	C
ATOM	4982	N	LYS	A	632	-19.438	33.875	31.172	1.00	92.69	N
ATOM	4983	CA	LYS	A	632	-19.688	34.625	32.406	1.00	92.69	C
ATOM	4984	C	LYS	A	632	-18.766	34.188	33.531	1.00	92.69	C
ATOM	4985	CB	LYS	A	632	-21.141	34.531	32.812	1.00	92.69	C
ATOM	4986	O	LYS	A	632	-18.562	33.000	33.719	1.00	92.69	O
ATOM	4987	CG	LYS	A	632	-22.125	35.219	31.875	1.00	92.69	C
ATOM	4988	CD	LYS	A	632	-23.562	35.094	32.375	1.00	92.69	C

ATOM	4989	CE	LYS	A	632	-24.547	35.688	31.375	1.00	92.69	C
ATOM	4990	NZ	LYS	A	632	-25.953	35.562	31.859	1.00	92.69	N
ATOM	4991	N	ILE	A	633	-18.156	35.219	34.250	1.00	92.06	N
ATOM	4992	CA	ILE	A	633	-17.266	34.938	35.375	1.00	92.06	C
ATOM	4993	C	ILE	A	633	-17.938	35.375	36.656	1.00	92.06	C
ATOM	4994	CB	ILE	A	633	-15.906	35.625	35.188	1.00	92.06	C
ATOM	4995	O	ILE	A	633	-18.344	36.531	36.812	1.00	92.06	O
ATOM	4996	CG1	ILE	A	633	-15.297	35.312	33.844	1.00	92.06	C
ATOM	4997	CG2	ILE	A	633	-14.953	35.250	36.344	1.00	92.06	C
ATOM	4998	CD1	ILE	A	633	-14.070	36.125	33.469	1.00	92.06	C
ATOM	4999	N	GLU	A	634	-18.344	34.500	37.531	1.00	89.62	N
ATOM	5000	CA	GLU	A	634	-18.828	34.750	38.906	1.00	89.62	C
ATOM	5001	C	GLU	A	634	-17.938	34.094	39.938	1.00	89.62	C
ATOM	5002	CB	GLU	A	634	-20.266	34.250	39.062	1.00	89.62	C
ATOM	5003	O	GLU	A	634	-17.125	33.219	39.625	1.00	89.62	O
ATOM	5004	CG	GLU	A	634	-21.266	34.969	38.125	1.00	89.62	C
ATOM	5005	CD	GLU	A	634	-22.672	34.406	38.281	1.00	89.62	C
ATOM	5006	OE1	GLU	A	634	-23.625	35.062	37.750	1.00	89.62	O
ATOM	5007	OE2	GLU	A	634	-22.844	33.344	38.906	1.00	89.62	O
ATOM	5008	N	SER	A	635	-17.938	34.625	41.094	1.00	86.44	N
ATOM	5009	CA	SER	A	635	-17.219	33.969	42.188	1.00	86.44	C
ATOM	5010	C	SER	A	635	-18.109	33.750	43.406	1.00	86.44	C
ATOM	5011	CB	SER	A	635	-15.992	34.812	42.594	1.00	86.44	C
ATOM	5012	O	SER	A	635	-19.109	34.469	43.594	1.00	86.44	O
ATOM	5013	OG	SER	A	635	-16.391	36.031	43.219	1.00	86.44	O
ATOM	5014	N	TYR	A	636	-18.016	32.750	44.094	1.00	81.38	N
ATOM	5015	CA	TYR	A	636	-18.641	32.531	45.406	1.00	81.38	C
ATOM	5016	C	TYR	A	636	-17.656	31.891	46.375	1.00	81.38	C
ATOM	5017	CB	TYR	A	636	-19.875	31.641	45.250	1.00	81.38	C
ATOM	5018	O	TYR	A	636	-16.641	31.344	45.969	1.00	81.38	O
ATOM	5019	CG	TYR	A	636	-19.594	30.281	44.688	1.00	81.38	C
ATOM	5020	CD1	TYR	A	636	-19.609	30.047	43.312	1.00	81.38	C
ATOM	5021	CD2	TYR	A	636	-19.297	29.203	45.531	1.00	81.38	C
ATOM	5022	CE1	TYR	A	636	-19.344	28.797	42.781	1.00	81.38	C
ATOM	5023	CE2	TYR	A	636	-19.016	27.938	45.000	1.00	81.38	C
ATOM	5024	OH	TYR	A	636	-18.781	26.500	43.125	1.00	81.38	O
ATOM	5025	CZ	TYR	A	636	-19.047	27.750	43.656	1.00	81.38	C
ATOM	5026	N	SER	A	637	-17.859	32.188	47.625	1.00	73.44	N
ATOM	5027	CA	SER	A	637	-17.000	31.672	48.656	1.00	73.44	C
ATOM	5028	C	SER	A	637	-17.703	30.562	49.438	1.00	73.44	C
ATOM	5029	CB	SER	A	637	-16.562	32.781	49.625	1.00	73.44	C
ATOM	5030	O	SER	A	637	-18.922	30.594	49.625	1.00	73.44	O
ATOM	5031	OG	SER	A	637	-17.688	33.312	50.312	1.00	73.44	O
ATOM	5032	N	ARG	A	638	-17.125	29.359	49.469	1.00	68.69	N
ATOM	5033	CA	ARG	A	638	-17.609	28.297	50.344	1.00	68.69	C
ATOM	5034	C	ARG	A	638	-16.562	27.875	51.344	1.00	68.69	C
ATOM	5035	CB	ARG	A	638	-18.031	27.078	49.500	1.00	68.69	C
ATOM	5036	O	ARG	A	638	-15.359	28.062	51.125	1.00	68.69	O
ATOM	5037	CG	ARG	A	638	-16.859	26.219	49.031	1.00	68.69	C
ATOM	5038	CD	ARG	A	638	-17.344	25.000	48.250	1.00	68.69	C
ATOM	5039	NE	ARG	A	638	-16.234	24.125	47.906	1.00	68.69	N
ATOM	5040	NH1	ARG	A	638	-17.516	22.672	46.656	1.00	68.69	N
ATOM	5041	NH2	ARG	A	638	-15.266	22.297	46.906	1.00	68.69	N
ATOM	5042	CZ	ARG	A	638	-16.344	23.031	47.156	1.00	68.69	C
ATOM	5043	N	VAL	A	639	-16.938	27.641	52.656	1.00	62.84	N
ATOM	5044	CA	VAL	A	639	-16.078	27.203	53.750	1.00	62.84	C
ATOM	5045	C	VAL	A	639	-15.805	25.703	53.625	1.00	62.84	C
ATOM	5046	CB	VAL	A	639	-16.703	27.516	55.125	1.00	62.84	C
ATOM	5047	O	VAL	A	639	-16.750	24.891	53.594	1.00	62.84	O
ATOM	5048	CG1	VAL	A	639	-15.797	27.031	56.250	1.00	62.84	C
ATOM	5049	CG2	VAL	A	639	-16.969	29.016	55.250	1.00	62.84	C
ATOM	5050	N	VAL	A	640	-14.688	25.375	53.031	1.00	56.31	N
ATOM	5051	CA	VAL	A	640	-14.289	23.969	52.969	1.00	56.31	C
ATOM	5052	C	VAL	A	640	-13.289	23.656	54.094	1.00	56.31	C

ATOM	5053	CB	VAL	A	640	-13.688	23.609	51.594	1.00	56.31	C
ATOM	5054	O	VAL	A	640	-12.172	24.188	54.094	1.00	56.31	O
ATOM	5055	CG1	VAL	A	640	-13.305	22.125	51.531	1.00	56.31	C
ATOM	5056	CG2	VAL	A	640	-14.664	23.953	50.469	1.00	56.31	C
ATOM	5057	N	GLN	A	641	-13.680	22.953	55.188	1.00	49.69	N
ATOM	5058	CA	GLN	A	641	-12.891	22.531	56.344	1.00	49.69	C
ATOM	5059	C	GLN	A	641	-12.320	23.750	57.094	1.00	49.69	C
ATOM	5060	CB	GLN	A	641	-11.758	21.594	55.938	1.00	49.69	C
ATOM	5061	O	GLN	A	641	-11.125	23.797	57.375	1.00	49.69	O
ATOM	5062	CG	GLN	A	641	-12.242	20.250	55.438	1.00	49.69	C
ATOM	5063	CD	GLN	A	641	-11.102	19.266	55.188	1.00	49.69	C
ATOM	5064	NE2	GLN	A	641	-11.406	17.984	55.219	1.00	49.69	N
ATOM	5065	OE1	GLN	A	641	-9.953	19.672	54.969	1.00	49.69	O
ATOM	5066	N	GLY	A	642	-13.125	24.844	57.312	1.00	52.75	N
ATOM	5067	CA	GLY	A	642	-12.742	25.906	58.219	1.00	52.75	C
ATOM	5068	C	GLY	A	642	-12.008	27.047	57.531	1.00	52.75	C
ATOM	5069	O	GLY	A	642	-11.695	28.062	58.156	1.00	52.75	O
ATOM	5070	N	ALA	A	643	-11.672	27.047	56.188	1.00	54.00	N
ATOM	5071	CA	ALA	A	643	-11.039	28.094	55.375	1.00	54.00	C
ATOM	5072	C	ALA	A	643	-11.938	28.516	54.219	1.00	54.00	C
ATOM	5073	CB	ALA	A	643	-9.688	27.609	54.844	1.00	54.00	C
ATOM	5074	O	ALA	A	643	-12.578	27.672	53.594	1.00	54.00	O
ATOM	5075	N	SER	A	644	-12.336	29.875	54.250	1.00	64.31	N
ATOM	5076	CA	SER	A	644	-13.094	30.484	53.156	1.00	64.31	C
ATOM	5077	C	SER	A	644	-12.320	30.438	51.844	1.00	64.31	C
ATOM	5078	CB	SER	A	644	-13.445	31.938	53.500	1.00	64.31	C
ATOM	5079	O	SER	A	644	-11.195	30.953	51.781	1.00	64.31	O
ATOM	5080	OG	SER	A	644	-14.148	32.562	52.438	1.00	64.31	O
ATOM	5081	N	VAL	A	645	-12.695	29.516	50.906	1.00	68.88	N
ATOM	5082	CA	VAL	A	645	-12.070	29.359	49.594	1.00	68.88	C
ATOM	5083	C	VAL	A	645	-12.930	30.047	48.531	1.00	68.88	C
ATOM	5084	CB	VAL	A	645	-11.867	27.875	49.250	1.00	68.88	C
ATOM	5085	O	VAL	A	645	-14.109	29.719	48.375	1.00	68.88	O
ATOM	5086	CG1	VAL	A	645	-11.195	27.734	47.875	1.00	68.88	C
ATOM	5087	CG2	VAL	A	645	-11.047	27.172	50.312	1.00	68.88	C
ATOM	5088	N	GLU	A	646	-12.438	31.125	48.188	1.00	78.56	N
ATOM	5089	CA	GLU	A	646	-13.102	31.828	47.094	1.00	78.56	C
ATOM	5090	C	GLU	A	646	-12.906	31.094	45.781	1.00	78.56	C
ATOM	5091	CB	GLU	A	646	-12.570	33.250	46.969	1.00	78.56	C
ATOM	5092	O	GLU	A	646	-11.781	30.781	45.375	1.00	78.56	O
ATOM	5093	CG	GLU	A	646	-13.398	34.156	46.062	1.00	78.56	C
ATOM	5094	CD	GLU	A	646	-14.750	34.531	46.688	1.00	78.56	C
ATOM	5095	OE1	GLU	A	646	-15.672	34.906	45.906	1.00	78.56	O
ATOM	5096	OE2	GLU	A	646	-14.883	34.438	47.906	1.00	78.56	O
ATOM	5097	N	THR	A	647	-14.055	30.641	45.031	1.00	85.19	N
ATOM	5098	CA	THR	A	647	-14.102	29.844	43.812	1.00	85.19	C
ATOM	5099	C	THR	A	647	-14.656	30.688	42.656	1.00	85.19	C
ATOM	5100	CB	THR	A	647	-14.961	28.578	44.000	1.00	85.19	C
ATOM	5101	O	THR	A	647	-15.703	31.312	42.781	1.00	85.19	O
ATOM	5102	CG2	THR	A	647	-14.953	27.734	42.719	1.00	85.19	C
ATOM	5103	OG1	THR	A	647	-14.445	27.812	45.094	1.00	85.19	O
ATOM	5104	N	MET	A	648	-13.844	30.766	41.688	1.00	89.25	N
ATOM	5105	CA	MET	A	648	-14.297	31.391	40.438	1.00	89.25	C
ATOM	5106	C	MET	A	648	-15.164	30.438	39.625	1.00	89.25	C
ATOM	5107	CB	MET	A	648	-13.094	31.844	39.594	1.00	89.25	C
ATOM	5108	O	MET	A	648	-14.758	29.297	39.375	1.00	89.25	O
ATOM	5109	CG	MET	A	648	-13.484	32.562	38.312	1.00	89.25	C
ATOM	5110	SD	MET	A	648	-12.023	33.094	37.344	1.00	89.25	S
ATOM	5111	CE	MET	A	648	-11.453	31.469	36.719	1.00	89.25	C
ATOM	5112	N	HIS	A	649	-16.344	30.812	39.375	1.00	92.25	N
ATOM	5113	CA	HIS	A	649	-17.297	30.062	38.562	1.00	92.25	C
ATOM	5114	C	HIS	A	649	-17.469	30.688	37.188	1.00	92.25	C
ATOM	5115	CB	HIS	A	649	-18.656	29.984	39.281	1.00	92.25	C
ATOM	5116	O	HIS	A	649	-17.891	31.828	37.062	1.00	92.25	O

ATOM	5117	CG	HIS	A	649	-19.688	29.219	38.500	1.00	92.25	C
ATOM	5118	CD2	HIS	A	649	-19.781	27.891	38.219	1.00	92.25	C
ATOM	5119	ND1	HIS	A	649	-20.797	29.812	37.969	1.00	92.25	N
ATOM	5120	CE1	HIS	A	649	-21.531	28.875	37.344	1.00	92.25	C
ATOM	5121	NE2	HIS	A	649	-20.922	27.719	37.500	1.00	92.25	N
ATOM	5122	N	VAL	A	650	-17.062	29.938	36.188	1.00	92.94	N
ATOM	5123	CA	VAL	A	650	-17.141	30.375	34.812	1.00	92.94	C
ATOM	5124	C	VAL	A	650	-18.125	29.516	34.031	1.00	92.94	C
ATOM	5125	CB	VAL	A	650	-15.750	30.344	34.125	1.00	92.94	C
ATOM	5126	O	VAL	A	650	-18.031	28.281	34.062	1.00	92.94	O
ATOM	5127	CG1	VAL	A	650	-15.820	30.938	32.719	1.00	92.94	C
ATOM	5128	CG2	VAL	A	650	-14.719	31.094	34.969	1.00	92.94	C
ATOM	5129	N	SER	A	651	-19.062	30.172	33.375	1.00	94.31	N
ATOM	5130	CA	SER	A	651	-20.094	29.469	32.625	1.00	94.31	C
ATOM	5131	C	SER	A	651	-20.266	30.062	31.234	1.00	94.31	C
ATOM	5132	CB	SER	A	651	-21.422	29.500	33.375	1.00	94.31	C
ATOM	5133	O	SER	A	651	-19.938	31.219	31.016	1.00	94.31	O
ATOM	5134	OG	SER	A	651	-21.891	30.828	33.531	1.00	94.31	O
ATOM	5135	N	TRP	A	652	-20.625	29.172	30.344	1.00	94.25	N
ATOM	5136	CA	TRP	A	652	-20.859	29.578	28.953	1.00	94.25	C
ATOM	5137	C	TRP	A	652	-22.016	28.766	28.359	1.00	94.25	C
ATOM	5138	CB	TRP	A	652	-19.594	29.391	28.125	1.00	94.25	C
ATOM	5139	O	TRP	A	652	-22.531	27.844	28.984	1.00	94.25	O
ATOM	5140	CG	TRP	A	652	-19.016	28.000	28.188	1.00	94.25	C
ATOM	5141	CD1	TRP	A	652	-19.328	26.938	27.391	1.00	94.25	C
ATOM	5142	CD2	TRP	A	652	-18.031	27.547	29.109	1.00	94.25	C
ATOM	5143	CE2	TRP	A	652	-17.797	26.188	28.828	1.00	94.25	C
ATOM	5144	CE3	TRP	A	652	-17.328	28.141	30.172	1.00	94.25	C
ATOM	5145	NE1	TRP	A	652	-18.594	25.844	27.766	1.00	94.25	N
ATOM	5146	CH2	TRP	A	652	-16.203	26.031	30.562	1.00	94.25	C
ATOM	5147	CZ2	TRP	A	652	-16.875	25.406	29.547	1.00	94.25	C
ATOM	5148	CZ3	TRP	A	652	-16.422	27.375	30.891	1.00	94.25	C
ATOM	5149	N	ASP	A	653	-22.469	29.188	27.156	1.00	92.19	N
ATOM	5150	CA	ASP	A	653	-23.547	28.500	26.438	1.00	92.19	C
ATOM	5151	C	ASP	A	653	-23.047	27.234	25.766	1.00	92.19	C
ATOM	5152	CB	ASP	A	653	-24.188	29.438	25.406	1.00	92.19	C
ATOM	5153	O	ASP	A	653	-21.875	27.172	25.344	1.00	92.19	O
ATOM	5154	CG	ASP	A	653	-24.969	30.562	26.047	1.00	92.19	C
ATOM	5155	OD1	ASP	A	653	-25.219	30.531	27.266	1.00	92.19	O
ATOM	5156	OD2	ASP	A	653	-25.359	31.500	25.312	1.00	92.19	O
ATOM	5157	N	LYS	A	654	-23.828	26.234	25.625	1.00	87.81	N
ATOM	5158	CA	LYS	A	654	-23.531	25.016	24.875	1.00	87.81	C
ATOM	5159	C	LYS	A	654	-23.312	25.328	23.391	1.00	87.81	C
ATOM	5160	CB	LYS	A	654	-24.672	24.000	25.031	1.00	87.81	C
ATOM	5161	O	LYS	A	654	-24.062	26.094	22.797	1.00	87.81	O
ATOM	5162	CG	LYS	A	654	-24.438	22.703	24.281	1.00	87.81	C
ATOM	5163	CD	LYS	A	654	-25.609	21.750	24.438	1.00	87.81	C
ATOM	5164	CE	LYS	A	654	-25.391	20.438	23.688	1.00	87.81	C
ATOM	5165	NZ	LYS	A	654	-26.531	19.500	23.859	1.00	87.81	N
ATOM	5166	N	VAL	A	655	-22.094	24.859	23.000	1.00	87.75	N
ATOM	5167	CA	VAL	A	655	-21.781	25.000	21.594	1.00	87.75	C
ATOM	5168	C	VAL	A	655	-22.188	23.719	20.844	1.00	87.75	C
ATOM	5169	CB	VAL	A	655	-20.281	25.281	21.375	1.00	87.75	C
ATOM	5170	O	VAL	A	655	-21.875	22.609	21.281	1.00	87.75	O
ATOM	5171	CG1	VAL	A	655	-19.953	25.375	19.891	1.00	87.75	C
ATOM	5172	CG2	VAL	A	655	-19.875	26.578	22.094	1.00	87.75	C
ATOM	5173	N	GLU	A	656	-22.984	23.875	19.875	1.00	82.06	N
ATOM	5174	CA	GLU	A	656	-23.453	22.781	19.031	1.00	82.06	C
ATOM	5175	C	GLU	A	656	-22.297	21.906	18.547	1.00	82.06	C
ATOM	5176	CB	GLU	A	656	-24.250	23.312	17.844	1.00	82.06	C
ATOM	5177	O	GLU	A	656	-21.281	22.438	18.062	1.00	82.06	O
ATOM	5178	CG	GLU	A	656	-25.031	22.234	17.094	1.00	82.06	C
ATOM	5179	CD	GLU	A	656	-25.953	22.812	16.031	1.00	82.06	C
ATOM	5180	OE1	GLU	A	656	-26.734	22.031	15.422	1.00	82.06	O

ATOM	5181	OE2	GLU	A	656	-25.906	24.031	15.797	1.00	82.06	O
ATOM	5182	N	TYR	A	657	-22.281	20.562	18.781	1.00	78.62	N
ATOM	5183	CA	TYR	A	657	-21.375	19.500	18.328	1.00	78.62	C
ATOM	5184	C	TYR	A	657	-20.172	19.391	19.250	1.00	78.62	C
ATOM	5185	CB	TYR	A	657	-20.922	19.766	16.891	1.00	78.62	C
ATOM	5186	O	TYR	A	657	-19.281	18.578	19.031	1.00	78.62	O
ATOM	5187	CG	TYR	A	657	-22.047	19.875	15.906	1.00	78.62	C
ATOM	5188	CD1	TYR	A	657	-22.984	18.859	15.758	1.00	78.62	C
ATOM	5189	CD2	TYR	A	657	-22.188	21.016	15.117	1.00	78.62	C
ATOM	5190	CE1	TYR	A	657	-24.031	18.953	14.844	1.00	78.62	C
ATOM	5191	CE2	TYR	A	657	-23.234	21.125	14.195	1.00	78.62	C
ATOM	5192	OH	TYR	A	657	-25.172	20.203	13.164	1.00	78.62	O
ATOM	5193	CZ	TYR	A	657	-24.141	20.094	14.070	1.00	78.62	C
ATOM	5194	N	ALA	A	658	-20.062	20.375	20.234	1.00	85.38	N
ATOM	5195	CA	ALA	A	658	-18.969	20.297	21.188	1.00	85.38	C
ATOM	5196	C	ALA	A	658	-19.188	19.156	22.172	1.00	85.38	C
ATOM	5197	CB	ALA	A	658	-18.812	21.625	21.938	1.00	85.38	C
ATOM	5198	O	ALA	A	658	-20.266	19.016	22.750	1.00	85.38	O
ATOM	5199	N	SER	A	659	-18.281	18.156	22.141	1.00	86.31	N
ATOM	5200	CA	SER	A	659	-18.312	17.078	23.125	1.00	86.31	C
ATOM	5201	C	SER	A	659	-17.641	17.484	24.422	1.00	86.31	C
ATOM	5202	CB	SER	A	659	-17.609	15.828	22.562	1.00	86.31	C
ATOM	5203	O	SER	A	659	-18.109	17.109	25.516	1.00	86.31	O
ATOM	5204	OG	SER	A	659	-17.734	14.742	23.453	1.00	86.31	O
ATOM	5205	N	LEU	A	660	-16.625	18.344	24.297	1.00	91.81	N
ATOM	5206	CA	LEU	A	660	-15.859	18.812	25.453	1.00	91.81	C
ATOM	5207	C	LEU	A	660	-15.547	20.297	25.344	1.00	91.81	C
ATOM	5208	CB	LEU	A	660	-14.562	18.000	25.594	1.00	91.81	C
ATOM	5209	O	LEU	A	660	-15.648	20.875	24.250	1.00	91.81	O
ATOM	5210	CG	LEU	A	660	-14.719	16.500	25.766	1.00	91.81	C
ATOM	5211	CD1	LEU	A	660	-13.438	15.781	25.344	1.00	91.81	C
ATOM	5212	CD2	LEU	A	660	-15.078	16.156	27.203	1.00	91.81	C
ATOM	5213	N	TYR	A	661	-15.211	21.000	26.484	1.00	94.00	N
ATOM	5214	CA	TYR	A	661	-14.773	22.391	26.500	1.00	94.00	C
ATOM	5215	C	TYR	A	661	-13.430	22.531	27.203	1.00	94.00	C
ATOM	5216	CB	TYR	A	661	-15.820	23.266	27.188	1.00	94.00	C
ATOM	5217	O	TYR	A	661	-13.133	21.781	28.141	1.00	94.00	O
ATOM	5218	CG	TYR	A	661	-17.156	23.297	26.469	1.00	94.00	C
ATOM	5219	CD1	TYR	A	661	-17.422	24.219	25.484	1.00	94.00	C
ATOM	5220	CD2	TYR	A	661	-18.156	22.375	26.812	1.00	94.00	C
ATOM	5221	CE1	TYR	A	661	-18.656	24.250	24.828	1.00	94.00	C
ATOM	5222	CE2	TYR	A	661	-19.391	22.391	26.156	1.00	94.00	C
ATOM	5223	OH	TYR	A	661	-20.844	23.359	24.531	1.00	94.00	O
ATOM	5224	CZ	TYR	A	661	-19.625	23.328	25.172	1.00	94.00	C
ATOM	5225	N	GLU	A	662	-12.688	23.344	26.547	1.00	94.12	N
ATOM	5226	CA	GLU	A	662	-11.422	23.734	27.156	1.00	94.12	C
ATOM	5227	C	GLU	A	662	-11.461	25.188	27.609	1.00	94.12	C
ATOM	5228	CB	GLU	A	662	-10.258	23.500	26.203	1.00	94.12	C
ATOM	5229	O	GLU	A	662	-11.766	26.078	26.812	1.00	94.12	O
ATOM	5230	CG	GLU	A	662	-9.953	22.047	25.938	1.00	94.12	C
ATOM	5231	CD	GLU	A	662	-8.812	21.828	24.953	1.00	94.12	C
ATOM	5232	OE1	GLU	A	662	-8.438	20.672	24.688	1.00	94.12	O
ATOM	5233	OE2	GLU	A	662	-8.289	22.844	24.438	1.00	94.12	O
ATOM	5234	N	MET	A	663	-11.219	25.578	28.891	1.00	93.81	N
ATOM	5235	CA	MET	A	663	-11.234	26.922	29.438	1.00	93.81	C
ATOM	5236	C	MET	A	663	-9.844	27.344	29.906	1.00	93.81	C
ATOM	5237	CB	MET	A	663	-12.227	27.016	30.594	1.00	93.81	C
ATOM	5238	O	MET	A	663	-9.125	26.547	30.500	1.00	93.81	O
ATOM	5239	CG	MET	A	663	-12.281	28.391	31.250	1.00	93.81	C
ATOM	5240	SD	MET	A	663	-12.883	28.328	32.969	1.00	93.81	S
ATOM	5241	CE	MET	A	663	-11.320	28.031	33.844	1.00	93.81	C
ATOM	5242	N	GLN	A	664	-9.508	28.578	29.578	1.00	94.69	N
ATOM	5243	CA	GLN	A	664	-8.281	29.188	30.094	1.00	94.69	C
ATOM	5244	C	GLN	A	664	-8.594	30.453	30.891	1.00	94.69	C

ATOM	5245	CB	GLN	A	664	-7.316	29.516	28.953	1.00 94.69	C
ATOM	5246	O	GLN	A	664	-9.570	31.156	30.609	1.00 94.69	O
ATOM	5247	CG	GLN	A	664	-6.719	28.281	28.281	1.00 94.69	C
ATOM	5248	CD	GLN	A	664	-5.688	28.625	27.234	1.00 94.69	C
ATOM	5249	NE2	GLN	A	664	-4.754	27.719	26.984	1.00 94.69	N
ATOM	5250	OE1	GLN	A	664	-5.734	29.703	26.625	1.00 94.69	O
ATOM	5251	N	TRP	A	665	-7.891	30.734	31.875	1.00 92.06	N
ATOM	5252	CA	TRP	A	665	-8.047	31.953	32.656	1.00 92.06	C
ATOM	5253	C	TRP	A	665	-6.688	32.562	33.000	1.00 92.06	C
ATOM	5254	CB	TRP	A	665	-8.828	31.656	33.938	1.00 92.06	C
ATOM	5255	O	TRP	A	665	-5.668	31.859	33.000	1.00 92.06	O
ATOM	5256	CG	TRP	A	665	-8.133	30.719	34.875	1.00 92.06	C
ATOM	5257	CD1	TRP	A	665	-8.148	29.359	34.844	1.00 92.06	C
ATOM	5258	CD2	TRP	A	665	-7.316	31.078	36.000	1.00 92.06	C
ATOM	5259	CE2	TRP	A	665	-6.871	29.875	36.594	1.00 92.06	C
ATOM	5260	CE3	TRP	A	665	-6.918	32.312	36.562	1.00 92.06	C
ATOM	5261	NE1	TRP	A	665	-7.395	28.844	35.875	1.00 92.06	N
ATOM	5262	CH2	TRP	A	665	-5.672	31.078	38.250	1.00 92.06	C
ATOM	5263	CZ2	TRP	A	665	-6.047	29.875	37.719	1.00 92.06	C
ATOM	5264	CZ3	TRP	A	665	-6.098	32.281	37.688	1.00 92.06	C
ATOM	5265	N	ARG	A	666	-6.629	33.844	33.094	1.00 90.62	N
ATOM	5266	CA	ARG	A	666	-5.430	34.500	33.594	1.00 90.62	C
ATOM	5267	C	ARG	A	666	-5.793	35.656	34.531	1.00 90.62	C
ATOM	5268	CB	ARG	A	666	-4.574	35.000	32.438	1.00 90.62	C
ATOM	5269	O	ARG	A	666	-6.879	36.219	34.438	1.00 90.62	O
ATOM	5270	CG	ARG	A	666	-5.223	36.125	31.656	1.00 90.62	C
ATOM	5271	CD	ARG	A	666	-4.328	36.625	30.531	1.00 90.62	C
ATOM	5272	NE	ARG	A	666	-4.949	37.719	29.781	1.00 90.62	N
ATOM	5273	NH1	ARG	A	666	-3.197	37.969	28.297	1.00 90.62	N
ATOM	5274	NH2	ARG	A	666	-5.051	39.312	28.125	1.00 90.62	N
ATOM	5275	CZ	ARG	A	666	-4.398	38.312	28.734	1.00 90.62	C
ATOM	5276	N	LYS	A	667	-5.070	36.156	35.500	1.00 86.19	N
ATOM	5277	CA	LYS	A	667	-5.195	37.219	36.469	1.00 86.19	C
ATOM	5278	C	LYS	A	667	-4.223	38.375	36.156	1.00 86.19	C
ATOM	5279	CB	LYS	A	667	-4.949	36.719	37.875	1.00 86.19	C
ATOM	5280	O	LYS	A	667	-3.033	38.125	35.938	1.00 86.19	O
ATOM	5281	CG	LYS	A	667	-5.082	37.781	38.969	1.00 86.19	C
ATOM	5282	CD	LYS	A	667	-4.816	37.188	40.344	1.00 86.19	C
ATOM	5283	CE	LYS	A	667	-4.859	38.281	41.438	1.00 86.19	C
ATOM	5284	NZ	LYS	A	667	-3.592	39.062	41.469	1.00 86.19	N
ATOM	5285	N	ASP	A	668	-4.633	39.656	36.031	1.00 83.38	N
ATOM	5286	CA	ASP	A	668	-3.881	40.875	35.938	1.00 83.38	C
ATOM	5287	C	ASP	A	668	-2.885	40.812	34.781	1.00 83.38	C
ATOM	5288	CB	ASP	A	668	-3.146	41.188	37.250	1.00 83.38	C
ATOM	5289	O	ASP	A	668	-1.693	41.094	34.969	1.00 83.38	O
ATOM	5290	CG	ASP	A	668	-4.090	41.438	38.406	1.00 83.38	C
ATOM	5291	OD1	ASP	A	668	-5.180	42.031	38.188	1.00 83.38	O
ATOM	5292	OD2	ASP	A	668	-3.740	41.062	39.531	1.00 83.38	O
ATOM	5293	N	ASN	A	669	-3.299	40.219	33.594	1.00 80.44	N
ATOM	5294	CA	ASN	A	669	-2.537	40.156	32.344	1.00 80.44	C
ATOM	5295	C	ASN	A	669	-1.348	39.219	32.469	1.00 80.44	C
ATOM	5296	CB	ASN	A	669	-2.064	41.562	31.953	1.00 80.44	C
ATOM	5297	O	ASN	A	669	-0.302	39.469	31.859	1.00 80.44	O
ATOM	5298	CG	ASN	A	669	-3.191	42.438	31.438	1.00 80.44	C
ATOM	5299	ND2	ASN	A	669	-3.078	43.750	31.656	1.00 80.44	N
ATOM	5300	OD1	ASN	A	669	-4.156	41.938	30.844	1.00 80.44	O
ATOM	5301	N	GLY	A	670	-1.397	38.156	33.375	1.00 78.75	N
ATOM	5302	CA	GLY	A	670	-0.411	37.094	33.500	1.00 78.75	C
ATOM	5303	C	GLY	A	670	-0.555	36.031	32.438	1.00 78.75	C
ATOM	5304	O	GLY	A	670	-1.127	36.281	31.359	1.00 78.75	O
ATOM	5305	N	ASN	A	671	0.036	34.844	32.562	1.00 85.25	N
ATOM	5306	CA	ASN	A	671	-0.032	33.719	31.641	1.00 85.25	C
ATOM	5307	C	ASN	A	671	-1.403	33.031	31.672	1.00 85.25	C
ATOM	5308	CB	ASN	A	671	1.063	32.688	31.953	1.00 85.25	C

ATOM	5309	O	ASN	A	671	-2.062	33.000	32.719	1.00	85.25	O
ATOM	5310	CG	ASN	A	671	2.451	33.219	31.625	1.00	85.25	C
ATOM	5311	ND2	ASN	A	671	3.459	32.656	32.312	1.00	85.25	N
ATOM	5312	OD1	ASN	A	671	2.617	34.062	30.766	1.00	85.25	O
ATOM	5313	N	TRP	A	672	-1.833	32.531	30.500	1.00	88.38	N
ATOM	5314	CA	TRP	A	672	-3.084	31.797	30.391	1.00	88.38	C
ATOM	5315	C	TRP	A	672	-2.969	30.453	31.078	1.00	88.38	C
ATOM	5316	CB	TRP	A	672	-3.480	31.609	28.922	1.00	88.38	C
ATOM	5317	O	TRP	A	672	-1.979	29.734	30.906	1.00	88.38	O
ATOM	5318	CG	TRP	A	672	-3.955	32.875	28.250	1.00	88.38	C
ATOM	5319	CD1	TRP	A	672	-3.236	33.688	27.422	1.00	88.38	C
ATOM	5320	CD2	TRP	A	672	-5.258	33.469	28.375	1.00	88.38	C
ATOM	5321	CE2	TRP	A	672	-5.258	34.625	27.578	1.00	88.38	C
ATOM	5322	CE3	TRP	A	672	-6.422	33.094	29.062	1.00	88.38	C
ATOM	5323	NE1	TRP	A	672	-4.012	34.750	27.016	1.00	88.38	N
ATOM	5324	CH2	TRP	A	672	-7.508	35.094	28.141	1.00	88.38	C
ATOM	5325	CZ2	TRP	A	672	-6.379	35.438	27.453	1.00	88.38	C
ATOM	5326	CZ3	TRP	A	672	-7.539	33.906	28.938	1.00	88.38	C
ATOM	5327	N	ASN	A	673	-3.908	30.062	31.969	1.00	88.88	N
ATOM	5328	CA	ASN	A	673	-4.004	28.781	32.656	1.00	88.88	C
ATOM	5329	C	ASN	A	673	-5.176	27.953	32.125	1.00	88.88	C
ATOM	5330	CB	ASN	A	673	-4.133	28.984	34.156	1.00	88.88	C
ATOM	5331	O	ASN	A	673	-6.281	28.469	31.969	1.00	88.88	O
ATOM	5332	CG	ASN	A	673	-2.900	29.609	34.781	1.00	88.88	C
ATOM	5333	ND2	ASN	A	673	-3.033	30.859	35.219	1.00	88.88	N
ATOM	5334	OD1	ASN	A	673	-1.835	28.984	34.844	1.00	88.88	O
ATOM	5335	N	ASN	A	674	-4.859	26.531	31.797	1.00	89.62	N
ATOM	5336	CA	ASN	A	674	-5.883	25.641	31.234	1.00	89.62	C
ATOM	5337	C	ASN	A	674	-6.660	24.938	32.344	1.00	89.62	C
ATOM	5338	CB	ASN	A	674	-5.250	24.625	30.281	1.00	89.62	C
ATOM	5339	O	ASN	A	674	-6.086	24.516	33.344	1.00	89.62	O
ATOM	5340	CG	ASN	A	674	-4.680	25.266	29.047	1.00	89.62	C
ATOM	5341	ND2	ASN	A	674	-3.498	24.812	28.625	1.00	89.62	N
ATOM	5342	OD1	ASN	A	674	-5.289	26.156	28.453	1.00	89.62	O
ATOM	5343	N	THR	A	675	-7.910	24.781	32.281	1.00	88.94	N
ATOM	5344	CA	THR	A	675	-8.719	23.906	33.094	1.00	88.94	C
ATOM	5345	C	THR	A	675	-8.930	22.562	32.406	1.00	88.94	C
ATOM	5346	CB	THR	A	675	-10.086	24.531	33.438	1.00	88.94	C
ATOM	5347	O	THR	A	675	-8.812	22.453	31.188	1.00	88.94	O
ATOM	5348	CG2	THR	A	675	-9.914	25.812	34.250	1.00	88.94	C
ATOM	5349	OG1	THR	A	675	-10.758	24.859	32.219	1.00	88.94	O
ATOM	5350	N	PRO	A	676	-9.070	21.344	33.156	1.00	87.75	N
ATOM	5351	CA	PRO	A	676	-9.398	20.078	32.500	1.00	87.75	C
ATOM	5352	C	PRO	A	676	-10.609	20.188	31.562	1.00	87.75	C
ATOM	5353	CB	PRO	A	676	-9.703	19.141	33.688	1.00	87.75	C
ATOM	5354	O	PRO	A	676	-11.523	20.969	31.828	1.00	87.75	O
ATOM	5355	CG	PRO	A	676	-9.055	19.797	34.844	1.00	87.75	C
ATOM	5356	CD	PRO	A	676	-9.008	21.281	34.625	1.00	87.75	C
ATOM	5357	N	ARG	A	677	-10.469	19.453	30.484	1.00	88.25	N
ATOM	5358	CA	ARG	A	677	-11.578	19.422	29.547	1.00	88.25	C
ATOM	5359	C	ARG	A	677	-12.859	18.938	30.219	1.00	88.25	C
ATOM	5360	CB	ARG	A	677	-11.242	18.531	28.344	1.00	88.25	C
ATOM	5361	O	ARG	A	677	-12.820	18.047	31.062	1.00	88.25	O
ATOM	5362	CG	ARG	A	677	-10.148	19.078	27.453	1.00	88.25	C
ATOM	5363	CD	ARG	A	677	-9.836	18.141	26.297	1.00	88.25	C
ATOM	5364	NE	ARG	A	677	-8.773	18.656	25.438	1.00	88.25	N
ATOM	5365	NH1	ARG	A	677	-8.922	16.969	23.875	1.00	88.25	N
ATOM	5366	NH2	ARG	A	677	-7.387	18.656	23.609	1.00	88.25	N
ATOM	5367	CZ	ARG	A	677	-8.367	18.094	24.312	1.00	88.25	C
ATOM	5368	N	THR	A	678	-13.922	19.609	30.000	1.00	91.38	N
ATOM	5369	CA	THR	A	678	-15.180	19.250	30.625	1.00	91.38	C
ATOM	5370	C	THR	A	678	-16.297	19.141	29.594	1.00	91.38	C
ATOM	5371	CB	THR	A	678	-15.578	20.266	31.719	1.00	91.38	C
ATOM	5372	O	THR	A	678	-16.281	19.859	28.594	1.00	91.38	O

ATOM	5373	CG2	THR	A	678	-15.773	21.656	31.125	1.00	91.38	C
ATOM	5374	OG1	THR	A	678	-16.797	19.844	32.344	1.00	91.38	O
ATOM	5375	N	ALA	A	679	-17.172	18.125	29.766	1.00	89.44	N
ATOM	5376	CA	ALA	A	679	-18.359	17.984	28.938	1.00	89.44	C
ATOM	5377	C	ALA	A	679	-19.453	18.953	29.391	1.00	89.44	C
ATOM	5378	CB	ALA	A	679	-18.859	16.547	28.953	1.00	89.44	C
ATOM	5379	O	ALA	A	679	-20.453	19.125	28.688	1.00	89.44	O
ATOM	5380	N	ASN	A	680	-19.188	19.578	30.594	1.00	91.94	N
ATOM	5381	CA	ASN	A	680	-20.172	20.500	31.172	1.00	91.94	C
ATOM	5382	C	ASN	A	680	-19.984	21.922	30.641	1.00	91.94	C
ATOM	5383	CB	ASN	A	680	-20.094	20.484	32.688	1.00	91.94	C
ATOM	5384	O	ASN	A	680	-18.938	22.250	30.078	1.00	91.94	O
ATOM	5385	CG	ASN	A	680	-20.484	19.141	33.281	1.00	91.94	C
ATOM	5386	ND2	ASN	A	680	-19.859	18.797	34.406	1.00	91.94	N
ATOM	5387	OD1	ASN	A	680	-21.328	18.422	32.750	1.00	91.94	O
ATOM	5388	N	LYS	A	681	-21.016	22.844	30.719	1.00	91.06	N
ATOM	5389	CA	LYS	A	681	-21.031	24.219	30.250	1.00	91.06	C
ATOM	5390	C	LYS	A	681	-20.516	25.172	31.328	1.00	91.06	C
ATOM	5391	CB	LYS	A	681	-22.438	24.641	29.812	1.00	91.06	C
ATOM	5392	O	LYS	A	681	-20.828	26.359	31.312	1.00	91.06	O
ATOM	5393	CG	LYS	A	681	-23.031	23.734	28.750	1.00	91.06	C
ATOM	5394	CD	LYS	A	681	-24.469	24.141	28.406	1.00	91.06	C
ATOM	5395	CE	LYS	A	681	-25.453	23.641	29.438	1.00	91.06	C
ATOM	5396	NZ	LYS	A	681	-26.875	23.938	29.047	1.00	91.06	N
ATOM	5397	N	GLU	A	682	-19.781	24.625	32.250	1.00	92.19	N
ATOM	5398	CA	GLU	A	682	-19.203	25.422	33.344	1.00	92.19	C
ATOM	5399	C	GLU	A	682	-17.953	24.750	33.906	1.00	92.19	C
ATOM	5400	CB	GLU	A	682	-20.234	25.656	34.438	1.00	92.19	C
ATOM	5401	O	GLU	A	682	-17.812	23.531	33.844	1.00	92.19	O
ATOM	5402	CG	GLU	A	682	-20.750	24.359	35.094	1.00	92.19	C
ATOM	5403	CD	GLU	A	682	-21.859	24.594	36.094	1.00	92.19	C
ATOM	5404	OE1	GLU	A	682	-22.188	23.672	36.875	1.00	92.19	O
ATOM	5405	OE2	GLU	A	682	-22.406	25.719	36.125	1.00	92.19	O
ATOM	5406	N	THR	A	683	-17.156	25.578	34.375	1.00	91.62	N
ATOM	5407	CA	THR	A	683	-15.969	25.125	35.094	1.00	91.62	C
ATOM	5408	C	THR	A	683	-15.695	26.000	36.312	1.00	91.62	C
ATOM	5409	CB	THR	A	683	-14.734	25.125	34.188	1.00	91.62	C
ATOM	5410	O	THR	A	683	-16.016	27.203	36.312	1.00	91.62	O
ATOM	5411	CG2	THR	A	683	-13.477	24.719	34.938	1.00	91.62	C
ATOM	5412	OG1	THR	A	683	-14.953	24.203	33.094	1.00	91.62	O
ATOM	5413	N	GLU	A	684	-15.273	25.438	37.344	1.00	89.81	N
ATOM	5414	CA	GLU	A	684	-14.953	26.188	38.562	1.00	89.81	C
ATOM	5415	C	GLU	A	684	-13.461	26.109	38.875	1.00	89.81	C
ATOM	5416	CB	GLU	A	684	-15.766	25.672	39.750	1.00	89.81	C
ATOM	5417	O	GLU	A	684	-12.828	25.078	38.688	1.00	89.81	O
ATOM	5418	CG	GLU	A	684	-17.266	25.891	39.625	1.00	89.81	C
ATOM	5419	CD	GLU	A	684	-18.062	25.344	40.781	1.00	89.81	C
ATOM	5420	OE1	GLU	A	684	-19.281	25.625	40.875	1.00	89.81	O
ATOM	5421	OE2	GLU	A	684	-17.469	24.625	41.625	1.00	89.81	O
ATOM	5422	N	VAL	A	685	-12.938	27.297	39.156	1.00	87.06	N
ATOM	5423	CA	VAL	A	685	-11.562	27.391	39.625	1.00	87.06	C
ATOM	5424	C	VAL	A	685	-11.539	27.812	41.094	1.00	87.06	C
ATOM	5425	CB	VAL	A	685	-10.734	28.375	38.781	1.00	87.06	C
ATOM	5426	O	VAL	A	685	-11.930	28.938	41.438	1.00	87.06	O
ATOM	5427	CG1	VAL	A	685	-9.281	28.422	39.250	1.00	87.06	C
ATOM	5428	CG2	VAL	A	685	-10.805	27.984	37.312	1.00	87.06	C
ATOM	5429	N	GLU	A	686	-11.305	26.938	42.000	1.00	83.25	N
ATOM	5430	CA	GLU	A	686	-11.320	27.141	43.438	1.00	83.25	C
ATOM	5431	C	GLU	A	686	-10.039	27.812	43.938	1.00	83.25	C
ATOM	5432	CB	GLU	A	686	-11.500	25.797	44.156	1.00	83.25	C
ATOM	5433	O	GLU	A	686	-8.969	27.609	43.344	1.00	83.25	O
ATOM	5434	CG	GLU	A	686	-12.906	25.234	44.062	1.00	83.25	C
ATOM	5435	CD	GLU	A	686	-13.117	24.000	44.938	1.00	83.25	C
ATOM	5436	OE1	GLU	A	686	-14.266	23.500	45.031	1.00	83.25	O

ATOM	5437	OE2	GLU	A	686	-12.133	23.531	45.531	1.00	83.25	O
ATOM	5438	N	GLY	A	687	-10.180	28.703	44.906	1.00	74.06	N
ATOM	5439	CA	GLY	A	687	-9.023	29.266	45.594	1.00	74.06	C
ATOM	5440	C	GLY	A	687	-8.359	30.391	44.812	1.00	74.06	C
ATOM	5441	O	GLY	A	687	-7.141	30.391	44.656	1.00	74.06	O
ATOM	5442	N	ILE	A	688	-9.133	31.391	44.344	1.00	75.31	N
ATOM	5443	CA	ILE	A	688	-8.602	32.438	43.500	1.00	75.31	C
ATOM	5444	C	ILE	A	688	-8.133	33.625	44.344	1.00	75.31	C
ATOM	5445	CB	ILE	A	688	-9.656	32.938	42.469	1.00	75.31	C
ATOM	5446	O	ILE	A	688	-8.641	33.812	45.469	1.00	75.31	O
ATOM	5447	CG1	ILE	A	688	-10.883	33.500	43.188	1.00	75.31	C
ATOM	5448	CG2	ILE	A	688	-10.055	31.781	41.531	1.00	75.31	C
ATOM	5449	CD1	ILE	A	688	-11.797	34.312	42.281	1.00	75.31	C
ATOM	5450	N	TYR	A	689	-7.004	34.375	44.094	1.00	71.56	N
ATOM	5451	CA	TYR	A	689	-6.520	35.594	44.719	1.00	71.56	C
ATOM	5452	C	TYR	A	689	-7.273	36.844	44.219	1.00	71.56	C
ATOM	5453	CB	TYR	A	689	-5.016	35.781	44.469	1.00	71.56	C
ATOM	5454	O	TYR	A	689	-7.895	36.781	43.156	1.00	71.56	O
ATOM	5455	CG	TYR	A	689	-4.152	34.906	45.344	1.00	71.56	C
ATOM	5456	CD1	TYR	A	689	-3.576	35.375	46.500	1.00	71.56	C
ATOM	5457	CD2	TYR	A	689	-3.908	33.562	45.000	1.00	71.56	C
ATOM	5458	CE1	TYR	A	689	-2.777	34.594	47.312	1.00	71.56	C
ATOM	5459	CE2	TYR	A	689	-3.111	32.750	45.781	1.00	71.56	C
ATOM	5460	OH	TYR	A	689	-1.761	32.469	47.719	1.00	71.56	O
ATOM	5461	CZ	TYR	A	689	-2.551	33.281	46.938	1.00	71.56	C
ATOM	5462	N	ALA	A	690	-7.270	37.781	45.031	1.00	77.44	N
ATOM	5463	CA	ALA	A	690	-7.918	39.031	44.656	1.00	77.44	C
ATOM	5464	C	ALA	A	690	-7.234	39.656	43.438	1.00	77.44	C
ATOM	5465	CB	ALA	A	690	-7.914	40.031	45.812	1.00	77.44	C
ATOM	5466	O	ALA	A	690	-6.004	39.719	43.375	1.00	77.44	O
ATOM	5467	N	GLY	A	691	-7.863	39.844	42.344	1.00	80.62	N
ATOM	5468	CA	GLY	A	691	-7.359	40.500	41.125	1.00	80.62	C
ATOM	5469	C	GLY	A	691	-8.336	40.438	39.969	1.00	80.62	C
ATOM	5470	O	GLY	A	691	-9.500	40.062	40.156	1.00	80.62	O
ATOM	5471	N	ASN	A	692	-7.902	41.062	38.844	1.00	88.31	N
ATOM	5472	CA	ASN	A	692	-8.680	41.094	37.594	1.00	88.31	C
ATOM	5473	C	ASN	A	692	-8.453	39.812	36.781	1.00	88.31	C
ATOM	5474	CB	ASN	A	692	-8.344	42.344	36.781	1.00	88.31	C
ATOM	5475	O	ASN	A	692	-7.320	39.531	36.375	1.00	88.31	O
ATOM	5476	CG	ASN	A	692	-9.469	42.719	35.844	1.00	88.31	C
ATOM	5477	ND2	ASN	A	692	-9.156	43.594	34.875	1.00	88.31	N
ATOM	5478	OD1	ASN	A	692	-10.609	42.281	36.000	1.00	88.31	O
ATOM	5479	N	TYR	A	693	-9.477	38.938	36.594	1.00	89.69	N
ATOM	5480	CA	TYR	A	693	-9.383	37.688	35.875	1.00	89.69	C
ATOM	5481	C	TYR	A	693	-9.938	37.812	34.469	1.00	89.69	C
ATOM	5482	CB	TYR	A	693	-10.133	36.594	36.625	1.00	89.69	C
ATOM	5483	O	TYR	A	693	-10.969	38.438	34.250	1.00	89.69	O
ATOM	5484	CG	TYR	A	693	-9.430	36.125	37.875	1.00	89.69	C
ATOM	5485	CD1	TYR	A	693	-8.664	34.938	37.875	1.00	89.69	C
ATOM	5486	CD2	TYR	A	693	-9.539	36.812	39.062	1.00	89.69	C
ATOM	5487	CE1	TYR	A	693	-8.016	34.500	39.031	1.00	89.69	C
ATOM	5488	CE2	TYR	A	693	-8.898	36.406	40.219	1.00	89.69	C
ATOM	5489	OH	TYR	A	693	-7.500	34.812	41.344	1.00	89.69	O
ATOM	5490	CZ	TYR	A	693	-8.141	35.250	40.188	1.00	89.69	C
ATOM	5491	N	HIS	A	694	-9.125	37.219	33.500	1.00	92.88	N
ATOM	5492	CA	HIS	A	694	-9.523	37.094	32.125	1.00	92.88	C
ATOM	5493	C	HIS	A	694	-9.750	35.625	31.734	1.00	92.88	C
ATOM	5494	CB	HIS	A	694	-8.469	37.719	31.188	1.00	92.88	C
ATOM	5495	O	HIS	A	694	-8.938	34.781	32.094	1.00	92.88	O
ATOM	5496	CG	HIS	A	694	-8.117	39.125	31.547	1.00	92.88	C
ATOM	5497	CD2	HIS	A	694	-7.027	39.625	32.156	1.00	92.88	C
ATOM	5498	ND1	HIS	A	694	-8.945	40.188	31.281	1.00	92.88	N
ATOM	5499	CE1	HIS	A	694	-8.375	41.312	31.703	1.00	92.88	C
ATOM	5500	NE2	HIS	A	694	-7.211	41.000	32.250	1.00	92.88	N

ATOM	5501	N	VAL	A	695	-10.852	35.312	31.188	1.00	94.31	N
ATOM	5502	CA	VAL	A	695	-11.203	33.938	30.844	1.00	94.31	C
ATOM	5503	C	VAL	A	695	-11.562	33.844	29.375	1.00	94.31	C
ATOM	5504	CB	VAL	A	695	-12.375	33.438	31.719	1.00	94.31	C
ATOM	5505	O	VAL	A	695	-12.180	34.781	28.812	1.00	94.31	O
ATOM	5506	CG1	VAL	A	695	-12.844	32.062	31.219	1.00	94.31	C
ATOM	5507	CG2	VAL	A	695	-11.969	33.375	33.188	1.00	94.31	C
ATOM	5508	N	ARG	A	696	-11.125	32.781	28.750	1.00	94.62	N
ATOM	5509	CA	ARG	A	696	-11.609	32.438	27.422	1.00	94.62	C
ATOM	5510	C	ARG	A	696	-11.836	30.922	27.297	1.00	94.62	C
ATOM	5511	CB	ARG	A	696	-10.617	32.906	26.359	1.00	94.62	C
ATOM	5512	O	ARG	A	696	-11.164	30.141	27.969	1.00	94.62	O
ATOM	5513	CG	ARG	A	696	-9.250	32.250	26.453	1.00	94.62	C
ATOM	5514	CD	ARG	A	696	-8.297	32.781	25.391	1.00	94.62	C
ATOM	5515	NE	ARG	A	696	-6.984	32.125	25.484	1.00	94.62	N
ATOM	5516	NH1	ARG	A	696	-6.055	33.344	23.734	1.00	94.62	N
ATOM	5517	NH2	ARG	A	696	-4.809	31.766	24.844	1.00	94.62	N
ATOM	5518	CZ	ARG	A	696	-5.953	32.406	24.688	1.00	94.62	C
ATOM	5519	N	VAL	A	697	-12.766	30.500	26.562	1.00	95.06	N
ATOM	5520	CA	VAL	A	697	-13.203	29.109	26.438	1.00	95.06	C
ATOM	5521	C	VAL	A	697	-13.297	28.734	24.969	1.00	95.06	C
ATOM	5522	CB	VAL	A	697	-14.562	28.875	27.141	1.00	95.06	C
ATOM	5523	O	VAL	A	697	-13.672	29.562	24.125	1.00	95.06	O
ATOM	5524	CG1	VAL	A	697	-14.938	27.406	27.109	1.00	95.06	C
ATOM	5525	CG2	VAL	A	697	-14.516	29.391	28.578	1.00	95.06	C
ATOM	5526	N	ARG	A	698	-12.953	27.500	24.641	1.00	94.88	N
ATOM	5527	CA	ARG	A	698	-13.195	26.984	23.297	1.00	94.88	C
ATOM	5528	C	ARG	A	698	-13.844	25.609	23.344	1.00	94.88	C
ATOM	5529	CB	ARG	A	698	-11.883	26.922	22.516	1.00	94.88	C
ATOM	5530	O	ARG	A	698	-13.719	24.891	24.328	1.00	94.88	O
ATOM	5531	CG	ARG	A	698	-10.930	25.844	22.984	1.00	94.88	C
ATOM	5532	CD	ARG	A	698	-9.648	25.812	22.156	1.00	94.88	C
ATOM	5533	NE	ARG	A	698	-8.727	24.797	22.625	1.00	94.88	N
ATOM	5534	NH1	ARG	A	698	-7.086	25.266	21.078	1.00	94.88	N
ATOM	5535	NH2	ARG	A	698	-6.766	23.594	22.609	1.00	94.88	N
ATOM	5536	CZ	ARG	A	698	-7.527	24.547	22.109	1.00	94.88	C
ATOM	5537	N	SER	A	699	-14.477	25.250	22.359	1.00	92.44	N
ATOM	5538	CA	SER	A	699	-15.195	23.984	22.234	1.00	92.44	C
ATOM	5539	C	SER	A	699	-14.359	22.953	21.500	1.00	92.44	C
ATOM	5540	CB	SER	A	699	-16.531	24.203	21.516	1.00	92.44	C
ATOM	5541	O	SER	A	699	-13.570	23.297	20.609	1.00	92.44	O
ATOM	5542	OG	SER	A	699	-16.312	24.828	20.250	1.00	92.44	O
ATOM	5543	N	VAL	A	700	-14.484	21.641	21.922	1.00	90.19	N
ATOM	5544	CA	VAL	A	700	-13.750	20.531	21.312	1.00	90.19	C
ATOM	5545	C	VAL	A	700	-14.734	19.453	20.859	1.00	90.19	C
ATOM	5546	CB	VAL	A	700	-12.719	19.938	22.297	1.00	90.19	C
ATOM	5547	O	VAL	A	700	-15.609	19.047	21.625	1.00	90.19	O
ATOM	5548	CG1	VAL	A	700	-11.930	18.812	21.641	1.00	90.19	C
ATOM	5549	CG2	VAL	A	700	-11.781	21.031	22.812	1.00	90.19	C
ATOM	5550	N	ALA	A	701	-14.516	19.031	19.609	1.00	83.88	N
ATOM	5551	CA	ALA	A	701	-15.367	17.984	19.078	1.00	83.88	C
ATOM	5552	C	ALA	A	701	-14.906	16.609	19.547	1.00	83.88	C
ATOM	5553	CB	ALA	A	701	-15.375	18.031	17.547	1.00	83.88	C
ATOM	5554	O	ALA	A	701	-13.828	16.469	20.125	1.00	83.88	O
ATOM	5555	N	ALA	A	702	-15.586	15.523	19.344	1.00	77.81	N
ATOM	5556	CA	ALA	A	702	-15.273	14.156	19.750	1.00	77.81	C
ATOM	5557	C	ALA	A	702	-13.992	13.664	19.062	1.00	77.81	C
ATOM	5558	CB	ALA	A	702	-16.438	13.227	19.422	1.00	77.81	C
ATOM	5559	O	ALA	A	702	-13.258	12.852	19.625	1.00	77.81	O
ATOM	5560	N	ASN	A	703	-13.773	14.188	17.812	1.00	75.19	N
ATOM	5561	CA	ASN	A	703	-12.594	13.758	17.062	1.00	75.19	C
ATOM	5562	C	ASN	A	703	-11.359	14.570	17.453	1.00	75.19	C
ATOM	5563	CB	ASN	A	703	-12.844	13.859	15.555	1.00	75.19	C
ATOM	5564	O	ASN	A	703	-10.289	14.391	16.875	1.00	75.19	O

ATOM	5565	CG	ASN	A	703	-13.055	15.289	15.094	1.00	75.19	C
ATOM	5566	ND2	ASN	A	703	-13.305	15.461	13.797	1.00	75.19	N
ATOM	5567	OD1	ASN	A	703	-13.000	16.234	15.891	1.00	75.19	O
ATOM	5568	N	GLY	A	704	-11.555	15.578	18.422	1.00	77.06	N
ATOM	5569	CA	GLY	A	704	-10.422	16.344	18.922	1.00	77.06	C
ATOM	5570	C	GLY	A	704	-10.273	17.703	18.266	1.00	77.06	C
ATOM	5571	O	GLY	A	704	-9.422	18.500	18.656	1.00	77.06	O
ATOM	5572	N	SER	A	705	-10.961	18.125	17.188	1.00	81.19	N
ATOM	5573	CA	SER	A	705	-10.938	19.438	16.547	1.00	81.19	C
ATOM	5574	C	SER	A	705	-11.477	20.516	17.484	1.00	81.19	C
ATOM	5575	CB	SER	A	705	-11.750	19.422	15.250	1.00	81.19	C
ATOM	5576	O	SER	A	705	-12.484	20.297	18.156	1.00	81.19	O
ATOM	5577	OG	SER	A	705	-11.273	18.422	14.367	1.00	81.19	O
ATOM	5578	N	ALA	A	706	-10.727	21.641	17.641	1.00	88.75	N
ATOM	5579	CA	ALA	A	706	-11.094	22.672	18.609	1.00	88.75	C
ATOM	5580	C	ALA	A	706	-11.492	23.969	17.906	1.00	88.75	C
ATOM	5581	CB	ALA	A	706	-9.938	22.922	19.562	1.00	88.75	C
ATOM	5582	O	ALA	A	706	-11.016	24.266	16.797	1.00	88.75	O
ATOM	5583	N	SER	A	707	-12.469	24.734	18.391	1.00	90.62	N
ATOM	5584	CA	SER	A	707	-12.836	26.062	17.906	1.00	90.62	C
ATOM	5585	C	SER	A	707	-11.781	27.094	18.266	1.00	90.62	C
ATOM	5586	CB	SER	A	707	-14.195	26.484	18.453	1.00	90.62	C
ATOM	5587	O	SER	A	707	-10.844	26.797	19.016	1.00	90.62	O
ATOM	5588	OG	SER	A	707	-14.078	26.875	19.812	1.00	90.62	O
ATOM	5589	N	GLY	A	708	-11.859	28.266	17.609	1.00	88.31	N
ATOM	5590	CA	GLY	A	708	-11.125	29.391	18.188	1.00	88.31	C
ATOM	5591	C	GLY	A	708	-11.531	29.703	19.609	1.00	88.31	C
ATOM	5592	O	GLY	A	708	-12.523	29.156	20.109	1.00	88.31	O
ATOM	5593	N	TRP	A	709	-10.797	30.531	20.359	1.00	93.69	N
ATOM	5594	CA	TRP	A	709	-11.109	30.922	21.734	1.00	93.69	C
ATOM	5595	C	TRP	A	709	-12.266	31.922	21.766	1.00	93.69	C
ATOM	5596	CB	TRP	A	709	-9.875	31.516	22.406	1.00	93.69	C
ATOM	5597	O	TRP	A	709	-12.430	32.719	20.828	1.00	93.69	O
ATOM	5598	CG	TRP	A	709	-8.758	30.547	22.609	1.00	93.69	C
ATOM	5599	CD1	TRP	A	709	-7.645	30.391	21.844	1.00	93.69	C
ATOM	5600	CD2	TRP	A	709	-8.656	29.562	23.656	1.00	93.69	C
ATOM	5601	CE2	TRP	A	709	-7.449	28.859	23.469	1.00	93.69	C
ATOM	5602	CE3	TRP	A	709	-9.469	29.219	24.750	1.00	93.69	C
ATOM	5603	NE1	TRP	A	709	-6.852	29.391	22.344	1.00	93.69	N
ATOM	5604	CH2	TRP	A	709	-7.848	27.516	25.375	1.00	93.69	C
ATOM	5605	CZ2	TRP	A	709	-7.035	27.844	24.312	1.00	93.69	C
ATOM	5606	CZ3	TRP	A	709	-9.055	28.203	25.594	1.00	93.69	C
ATOM	5607	N	SER	A	710	-13.148	31.922	22.734	1.00	92.94	N
ATOM	5608	CA	SER	A	710	-14.148	32.969	22.969	1.00	92.94	C
ATOM	5609	C	SER	A	710	-13.484	34.312	23.234	1.00	92.94	C
ATOM	5610	CB	SER	A	710	-15.062	32.594	24.125	1.00	92.94	C
ATOM	5611	O	SER	A	710	-12.273	34.375	23.422	1.00	92.94	O
ATOM	5612	OG	SER	A	710	-14.375	32.719	25.359	1.00	92.94	O
ATOM	5613	N	ALA	A	711	-14.297	35.344	23.125	1.00	90.00	N
ATOM	5614	CA	ALA	A	711	-13.789	36.656	23.562	1.00	90.00	C
ATOM	5615	C	ALA	A	711	-13.305	36.594	25.016	1.00	90.00	C
ATOM	5616	CB	ALA	A	711	-14.867	37.719	23.406	1.00	90.00	C
ATOM	5617	O	ALA	A	711	-13.867	35.875	25.828	1.00	90.00	O
ATOM	5618	N	ILE	A	712	-12.188	37.219	25.281	1.00	92.12	N
ATOM	5619	CA	ILE	A	712	-11.672	37.281	26.656	1.00	92.12	C
ATOM	5620	C	ILE	A	712	-12.648	38.062	27.531	1.00	92.12	C
ATOM	5621	CB	ILE	A	712	-10.273	37.938	26.703	1.00	92.12	C
ATOM	5622	O	ILE	A	712	-13.055	39.188	27.203	1.00	92.12	O
ATOM	5623	CG1	ILE	A	712	-9.266	37.094	25.922	1.00	92.12	C
ATOM	5624	CG2	ILE	A	712	-9.820	38.125	28.156	1.00	92.12	C
ATOM	5625	CD1	ILE	A	712	-7.906	37.750	25.750	1.00	92.12	C
ATOM	5626	N	VAL	A	713	-13.289	37.531	28.562	1.00	91.81	N
ATOM	5627	CA	VAL	A	713	-14.156	38.156	29.547	1.00	91.81	C
ATOM	5628	C	VAL	A	713	-13.383	38.375	30.844	1.00	91.81	C

ATOM	5629	CB	VAL	A	713	-15.414	37.312	29.828	1.00	91.81	C
ATOM	5630	O	VAL	A	713	-12.703	37.469	31.328	1.00	91.81	O
ATOM	5631	CG1	VAL	A	713	-16.328	37.969	30.844	1.00	91.81	C
ATOM	5632	CG2	VAL	A	713	-16.172	37.031	28.516	1.00	91.81	C
ATOM	5633	N	SER	A	714	-13.422	39.656	31.281	1.00	91.94	N
ATOM	5634	CA	SER	A	714	-12.641	40.000	32.469	1.00	91.94	C
ATOM	5635	C	SER	A	714	-13.555	40.312	33.625	1.00	91.94	C
ATOM	5636	CB	SER	A	714	-11.734	41.188	32.156	1.00	91.94	C
ATOM	5637	O	SER	A	714	-14.656	40.844	33.469	1.00	91.94	O
ATOM	5638	OG	SER	A	714	-10.836	40.875	31.109	1.00	91.94	O
ATOM	5639	N	ALA	A	715	-13.328	39.938	34.812	1.00	89.75	N
ATOM	5640	CA	ALA	A	715	-14.023	40.281	36.031	1.00	89.75	C
ATOM	5641	C	ALA	A	715	-13.039	40.438	37.188	1.00	89.75	C
ATOM	5642	CB	ALA	A	715	-15.062	39.219	36.375	1.00	89.75	C
ATOM	5643	O	ALA	A	715	-12.078	39.688	37.312	1.00	89.75	O
ATOM	5644	N	GLY	A	716	-13.164	41.656	37.969	1.00	83.00	N
ATOM	5645	CA	GLY	A	716	-12.438	41.781	39.219	1.00	83.00	C
ATOM	5646	C	GLY	A	716	-13.016	40.906	40.312	1.00	83.00	C
ATOM	5647	O	GLY	A	716	-14.203	41.000	40.625	1.00	83.00	O
ATOM	5648	N	LEU	A	717	-12.336	39.938	40.750	1.00	82.06	N
ATOM	5649	CA	LEU	A	717	-12.812	39.000	41.781	1.00	82.06	C
ATOM	5650	C	LEU	A	717	-12.016	39.156	43.062	1.00	82.06	C
ATOM	5651	CB	LEU	A	717	-12.703	37.562	41.281	1.00	82.06	C
ATOM	5652	O	LEU	A	717	-10.836	39.500	43.031	1.00	82.06	O
ATOM	5653	CG	LEU	A	717	-13.461	37.219	40.000	1.00	82.06	C
ATOM	5654	CD1	LEU	A	717	-13.172	35.812	39.562	1.00	82.06	C
ATOM	5655	CD2	LEU	A	717	-14.961	37.469	40.188	1.00	82.06	C
ATOM	5656	N	THR	A	718	-12.734	39.250	44.250	1.00	71.88	N
ATOM	5657	CA	THR	A	718	-12.195	39.312	45.594	1.00	71.88	C
ATOM	5658	C	THR	A	718	-11.734	37.938	46.062	1.00	71.88	C
ATOM	5659	CB	THR	A	718	-13.227	39.875	46.594	1.00	71.88	C
ATOM	5660	O	THR	A	718	-12.227	36.906	45.562	1.00	71.88	O
ATOM	5661	CG2	THR	A	718	-13.625	41.281	46.219	1.00	71.88	C
ATOM	5662	OG1	THR	A	718	-14.391	39.031	46.562	1.00	71.88	O
ATOM	5663	N	GLY	A	719	-10.383	37.812	46.344	1.00	62.66	N
ATOM	5664	CA	GLY	A	719	-9.789	36.531	46.719	1.00	62.66	C
ATOM	5665	C	GLY	A	719	-8.594	36.688	47.656	1.00	62.66	C
ATOM	5666	O	GLY	A	719	-8.469	37.688	48.344	1.00	62.66	O
ATOM	5667	N	LYS	A	720	-7.832	35.906	48.000	1.00	56.41	N
ATOM	5668	CA	LYS	A	720	-6.691	35.781	48.906	1.00	56.41	C
ATOM	5669	C	LYS	A	720	-5.555	36.719	48.500	1.00	56.41	C
ATOM	5670	CB	LYS	A	720	-6.199	34.344	49.000	1.00	56.41	C
ATOM	5671	O	LYS	A	720	-5.262	36.812	47.281	1.00	56.41	O
ATOM	5672	CG	LYS	A	720	-5.328	34.031	50.188	1.00	56.41	C
ATOM	5673	CD	LYS	A	720	-4.961	32.562	50.281	1.00	56.41	C
ATOM	5674	CE	LYS	A	720	-4.027	32.281	51.438	1.00	56.41	C
ATOM	5675	NZ	LYS	A	720	-3.682	30.828	51.531	1.00	56.41	N
ATOM	5676	N	VAL	A	721	-4.875	37.688	49.250	1.00	54.03	N
ATOM	5677	CA	VAL	A	721	-3.807	38.688	49.188	1.00	54.03	C
ATOM	5678	C	VAL	A	721	-2.512	38.094	49.719	1.00	54.03	C
ATOM	5679	CB	VAL	A	721	-4.168	39.969	49.969	1.00	54.03	C
ATOM	5680	O	VAL	A	721	-2.518	37.406	50.750	1.00	54.03	O
ATOM	5681	CG1	VAL	A	721	-3.064	41.000	49.844	1.00	54.03	C
ATOM	5682	CG2	VAL	A	721	-5.504	40.531	49.500	1.00	54.03	C
ATOM	5683	N	GLY	A	722	-1.220	38.125	48.719	1.00	61.59	N
ATOM	5684	CA	GLY	A	722	0.125	37.719	49.094	1.00	61.59	C
ATOM	5685	C	GLY	A	722	0.767	36.781	48.062	1.00	61.59	C
ATOM	5686	O	GLY	A	722	0.095	36.281	47.188	1.00	61.59	O
ATOM	5687	N	GLU	A	723	2.346	36.844	47.875	1.00	65.31	N
ATOM	5688	CA	GLU	A	723	3.189	36.000	47.031	1.00	65.31	C
ATOM	5689	C	GLU	A	723	3.199	34.562	47.531	1.00	65.31	C
ATOM	5690	CB	GLU	A	723	4.617	36.562	46.969	1.00	65.31	C
ATOM	5691	O	GLU	A	723	3.084	34.344	48.750	1.00	65.31	O
ATOM	5692	CG	GLU	A	723	4.730	37.906	46.250	1.00	65.31	C

ATOM	5693	CD	GLU	A	723	6.152	38.438	46.219	1.00	65.31	C
ATOM	5694	OE1	GLU	A	723	6.391	39.469	45.562	1.00	65.31	O
ATOM	5695	OE2	GLU	A	723	7.035	37.812	46.844	1.00	65.31	O
ATOM	5696	N	PRO	A	724	3.055	33.625	46.594	1.00	74.81	N
ATOM	5697	CA	PRO	A	724	3.080	32.219	46.938	1.00	74.81	C
ATOM	5698	C	PRO	A	724	4.387	31.797	47.625	1.00	74.81	C
ATOM	5699	CB	PRO	A	724	2.912	31.500	45.594	1.00	74.81	C
ATOM	5700	O	PRO	A	724	5.430	32.406	47.375	1.00	74.81	O
ATOM	5701	CG	PRO	A	724	3.164	32.562	44.562	1.00	74.81	C
ATOM	5702	CD	PRO	A	724	3.102	33.906	45.250	1.00	74.81	C
ATOM	5703	N	GLU	A	725	4.355	31.094	48.656	1.00	83.06	N
ATOM	5704	CA	GLU	A	725	5.523	30.516	49.312	1.00	83.06	C
ATOM	5705	C	GLU	A	725	6.273	29.562	48.406	1.00	83.06	C
ATOM	5706	CB	GLU	A	725	5.105	29.797	50.625	1.00	83.06	C
ATOM	5707	O	GLU	A	725	5.680	28.938	47.531	1.00	83.06	O
ATOM	5708	CG	GLU	A	725	4.605	30.734	51.719	1.00	83.06	C
ATOM	5709	CD	GLU	A	725	4.324	30.031	53.031	1.00	83.06	C
ATOM	5710	OE1	GLU	A	725	4.160	30.703	54.062	1.00	83.06	O
ATOM	5711	OE2	GLU	A	725	4.266	28.781	53.000	1.00	83.06	O
ATOM	5712	N	LYS	A	726	7.727	29.656	48.438	1.00	88.62	N
ATOM	5713	CA	LYS	A	726	8.547	28.766	47.625	1.00	88.62	C
ATOM	5714	C	LYS	A	726	8.289	27.312	48.000	1.00	88.62	C
ATOM	5715	CB	LYS	A	726	10.031	29.094	47.812	1.00	88.62	C
ATOM	5716	O	LYS	A	726	7.906	27.000	49.125	1.00	88.62	O
ATOM	5717	CG	LYS	A	726	10.570	28.844	49.188	1.00	88.62	C
ATOM	5718	CD	LYS	A	726	12.047	29.203	49.312	1.00	88.62	C
ATOM	5719	CE	LYS	A	726	12.570	29.031	50.719	1.00	88.62	C
ATOM	5720	NZ	LYS	A	726	13.977	29.500	50.844	1.00	88.62	N
ATOM	5721	N	PRO	A	727	8.344	26.328	47.000	1.00	91.94	N
ATOM	5722	CA	PRO	A	727	8.242	24.891	47.312	1.00	91.94	C
ATOM	5723	C	PRO	A	727	9.164	24.469	48.438	1.00	91.94	C
ATOM	5724	CB	PRO	A	727	8.641	24.219	45.969	1.00	91.94	C
ATOM	5725	O	PRO	A	727	10.211	25.078	48.656	1.00	91.94	O
ATOM	5726	CG	PRO	A	727	8.375	25.250	44.938	1.00	91.94	C
ATOM	5727	CD	PRO	A	727	8.609	26.609	45.531	1.00	91.94	C
ATOM	5728	N	ILE	A	728	8.727	23.578	49.281	1.00	90.94	N
ATOM	5729	CA	ILE	A	728	9.516	23.109	50.438	1.00	90.94	C
ATOM	5730	C	ILE	A	728	9.938	21.656	50.188	1.00	90.94	C
ATOM	5731	CB	ILE	A	728	8.727	23.219	51.750	1.00	90.94	C
ATOM	5732	O	ILE	A	728	9.359	20.969	49.344	1.00	90.94	O
ATOM	5733	CG1	ILE	A	728	7.383	22.484	51.625	1.00	90.94	C
ATOM	5734	CG2	ILE	A	728	8.523	24.688	52.125	1.00	90.94	C
ATOM	5735	CD1	ILE	A	728	6.605	22.422	52.938	1.00	90.94	C
ATOM	5736	N	ASN	A	729	11.016	21.234	50.594	1.00	91.75	N
ATOM	5737	CA	ASN	A	729	11.523	19.875	50.594	1.00	91.75	C
ATOM	5738	C	ASN	A	729	11.883	19.422	49.188	1.00	91.75	C
ATOM	5739	CB	ASN	A	729	10.492	18.922	51.219	1.00	91.75	C
ATOM	5740	O	ASN	A	729	11.547	18.297	48.781	1.00	91.75	O
ATOM	5741	CG	ASN	A	729	10.266	19.188	52.688	1.00	91.75	C
ATOM	5742	ND2	ASN	A	729	9.055	18.922	53.156	1.00	91.75	N
ATOM	5743	OD1	ASN	A	729	11.172	19.625	53.406	1.00	91.75	O
ATOM	5744	N	LEU	A	730	12.469	20.375	48.344	1.00	94.06	N
ATOM	5745	CA	LEU	A	730	12.922	19.969	47.000	1.00	94.06	C
ATOM	5746	C	LEU	A	730	13.992	18.891	47.094	1.00	94.06	C
ATOM	5747	CB	LEU	A	730	13.469	21.188	46.250	1.00	94.06	C
ATOM	5748	O	LEU	A	730	15.000	19.062	47.781	1.00	94.06	O
ATOM	5749	CG	LEU	A	730	13.867	20.953	44.781	1.00	94.06	C
ATOM	5750	CD1	LEU	A	730	12.633	20.703	43.938	1.00	94.06	C
ATOM	5751	CD2	LEU	A	730	14.664	22.125	44.250	1.00	94.06	C
ATOM	5752	N	THR	A	731	13.664	17.719	46.531	1.00	93.88	N
ATOM	5753	CA	THR	A	731	14.625	16.625	46.500	1.00	93.88	C
ATOM	5754	C	THR	A	731	14.828	16.125	45.062	1.00	93.88	C
ATOM	5755	CB	THR	A	731	14.180	15.453	47.375	1.00	93.88	C
ATOM	5756	O	THR	A	731	13.922	16.219	44.219	1.00	93.88	O

ATOM	5757	CG2	THR	A	731	14.109	15.883	48.844	1.00	93.88	C
ATOM	5758	OG1	THR	A	731	12.883	15.008	46.969	1.00	93.88	O
ATOM	5759	N	ALA	A	732	15.961	15.773	44.656	1.00	94.62	N
ATOM	5760	CA	ALA	A	732	16.328	15.109	43.406	1.00	94.62	C
ATOM	5761	C	ALA	A	732	16.938	13.734	43.688	1.00	94.62	C
ATOM	5762	CB	ALA	A	732	17.312	15.977	42.625	1.00	94.62	C
ATOM	5763	O	ALA	A	732	17.797	13.586	44.562	1.00	94.62	O
ATOM	5764	N	SER	A	733	16.391	12.797	42.969	1.00	92.56	N
ATOM	5765	CA	SER	A	733	16.797	11.422	43.219	1.00	92.56	C
ATOM	5766	C	SER	A	733	18.219	11.156	42.781	1.00	92.56	C
ATOM	5767	CB	SER	A	733	15.828	10.445	42.531	1.00	92.56	C
ATOM	5768	O	SER	A	733	18.750	11.891	41.938	1.00	92.56	O
ATOM	5769	OG	SER	A	733	15.930	10.547	41.125	1.00	92.56	O
ATOM	5770	N	ASP	A	734	18.828	10.180	43.344	1.00	88.81	N
ATOM	5771	CA	ASP	A	734	20.125	9.688	42.938	1.00	88.81	C
ATOM	5772	C	ASP	A	734	20.078	8.195	42.594	1.00	88.81	C
ATOM	5773	CB	ASP	A	734	21.188	9.953	44.000	1.00	88.81	C
ATOM	5774	O	ASP	A	734	21.109	7.527	42.562	1.00	88.81	O
ATOM	5775	CG	ASP	A	734	20.875	9.250	45.312	1.00	88.81	C
ATOM	5776	OD1	ASP	A	734	19.844	8.539	45.406	1.00	88.81	O
ATOM	5777	OD2	ASP	A	734	21.672	9.414	46.281	1.00	88.81	O
ATOM	5778	N	ASN	A	735	18.781	7.680	42.438	1.00	84.19	N
ATOM	5779	CA	ASN	A	735	18.688	6.227	42.344	1.00	84.19	C
ATOM	5780	C	ASN	A	735	17.875	5.809	41.125	1.00	84.19	C
ATOM	5781	CB	ASN	A	735	18.094	5.629	43.625	1.00	84.19	C
ATOM	5782	O	ASN	A	735	17.594	4.621	40.938	1.00	84.19	O
ATOM	5783	CG	ASN	A	735	16.703	6.152	43.906	1.00	84.19	C
ATOM	5784	ND2	ASN	A	735	16.047	5.562	44.906	1.00	84.19	N
ATOM	5785	OD1	ASN	A	735	16.234	7.082	43.250	1.00	84.19	O
ATOM	5786	N	GLU	A	736	17.453	6.781	40.281	1.00	87.00	N
ATOM	5787	CA	GLU	A	736	16.594	6.484	39.125	1.00	87.00	C
ATOM	5788	C	GLU	A	736	17.422	6.082	37.906	1.00	87.00	C
ATOM	5789	CB	GLU	A	736	15.711	7.691	38.781	1.00	87.00	C
ATOM	5790	O	GLU	A	736	18.422	6.730	37.594	1.00	87.00	O
ATOM	5791	CG	GLU	A	736	14.766	8.094	39.906	1.00	87.00	C
ATOM	5792	CD	GLU	A	736	13.602	7.133	40.094	1.00	87.00	C
ATOM	5793	OE1	GLU	A	736	12.875	7.242	41.094	1.00	87.00	O
ATOM	5794	OE2	GLU	A	736	13.422	6.258	39.219	1.00	87.00	O
ATOM	5795	N	VAL	A	737	17.062	4.879	37.281	1.00	82.81	N
ATOM	5796	CA	VAL	A	737	17.797	4.340	36.125	1.00	82.81	C
ATOM	5797	C	VAL	A	737	17.609	5.246	34.906	1.00	82.81	C
ATOM	5798	CB	VAL	A	737	17.344	2.900	35.812	1.00	82.81	C
ATOM	5799	O	VAL	A	737	16.484	5.574	34.562	1.00	82.81	O
ATOM	5800	CG1	VAL	A	737	18.078	2.373	34.562	1.00	82.81	C
ATOM	5801	CG2	VAL	A	737	17.562	1.979	37.000	1.00	82.81	C
ATOM	5802	N	PHE	A	738	18.641	5.777	34.375	1.00	86.56	N
ATOM	5803	CA	PHE	A	738	18.719	6.621	33.188	1.00	86.56	C
ATOM	5804	C	PHE	A	738	17.875	7.883	33.375	1.00	86.56	C
ATOM	5805	CB	PHE	A	738	18.266	5.855	31.953	1.00	86.56	C
ATOM	5806	O	PHE	A	738	17.375	8.430	32.375	1.00	86.56	O
ATOM	5807	CG	PHE	A	738	19.109	4.652	31.625	1.00	86.56	C
ATOM	5808	CD1	PHE	A	738	20.500	4.734	31.656	1.00	86.56	C
ATOM	5809	CD2	PHE	A	738	18.516	3.438	31.312	1.00	86.56	C
ATOM	5810	CE1	PHE	A	738	21.281	3.623	31.375	1.00	86.56	C
ATOM	5811	CE2	PHE	A	738	19.297	2.322	31.016	1.00	86.56	C
ATOM	5812	CZ	PHE	A	738	20.688	2.416	31.047	1.00	86.56	C
ATOM	5813	N	GLY	A	739	17.688	8.273	34.656	1.00	91.81	N
ATOM	5814	CA	GLY	A	739	16.906	9.477	34.906	1.00	91.81	C
ATOM	5815	C	GLY	A	739	17.031	10.008	36.312	1.00	91.81	C
ATOM	5816	O	GLY	A	739	17.656	9.375	37.156	1.00	91.81	O
ATOM	5817	N	ILE	A	740	16.531	11.180	36.625	1.00	93.88	N
ATOM	5818	CA	ILE	A	740	16.469	11.852	37.938	1.00	93.88	C
ATOM	5819	C	ILE	A	740	15.023	12.258	38.219	1.00	93.88	C
ATOM	5820	CB	ILE	A	740	17.391	13.094	37.969	1.00	93.88	C

ATOM	5821	O	ILE	A	740	14.352	12.852	37.375	1.00	93.88	O
ATOM	5822	CG1	ILE	A	740	18.844	12.680	37.750	1.00	93.88	C
ATOM	5823	CG2	ILE	A	740	17.203	13.852	39.281	1.00	93.88	C
ATOM	5824	CD1	ILE	A	740	19.781	13.844	37.469	1.00	93.88	C
ATOM	5825	N	ARG	A	741	14.508	11.805	39.375	1.00	93.75	N
ATOM	5826	CA	ARG	A	741	13.188	12.227	39.844	1.00	93.75	C
ATOM	5827	C	ARG	A	741	13.289	13.398	40.812	1.00	93.75	C
ATOM	5828	CB	ARG	A	741	12.453	11.062	40.500	1.00	93.75	C
ATOM	5829	O	ARG	A	741	13.938	13.289	41.844	1.00	93.75	O
ATOM	5830	CG	ARG	A	741	10.953	11.273	40.625	1.00	93.75	C
ATOM	5831	CD	ARG	A	741	10.195	9.953	40.688	1.00	93.75	C
ATOM	5832	NE	ARG	A	741	9.750	9.523	39.375	1.00	93.75	N
ATOM	5833	NH1	ARG	A	741	8.484	7.754	40.156	1.00	93.75	N
ATOM	5834	NH2	ARG	A	741	8.594	8.180	37.906	1.00	93.75	N
ATOM	5835	CZ	ARG	A	741	8.945	8.484	39.156	1.00	93.75	C
ATOM	5836	N	VAL	A	742	12.758	14.477	40.469	1.00	95.31	N
ATOM	5837	CA	VAL	A	742	12.742	15.688	41.281	1.00	95.31	C
ATOM	5838	C	VAL	A	742	11.375	15.859	41.938	1.00	95.31	C
ATOM	5839	CB	VAL	A	742	13.094	16.938	40.438	1.00	95.31	C
ATOM	5840	O	VAL	A	742	10.344	15.859	41.250	1.00	95.31	O
ATOM	5841	CG1	VAL	A	742	13.227	18.172	41.312	1.00	95.31	C
ATOM	5842	CG2	VAL	A	742	14.375	16.703	39.625	1.00	95.31	C
ATOM	5843	N	LYS	A	743	11.359	15.922	43.312	1.00	94.88	N
ATOM	5844	CA	LYS	A	743	10.133	16.062	44.094	1.00	94.88	C
ATOM	5845	C	LYS	A	743	10.188	17.297	44.969	1.00	94.88	C
ATOM	5846	CB	LYS	A	743	9.891	14.820	44.938	1.00	94.88	C
ATOM	5847	O	LYS	A	743	11.266	17.719	45.406	1.00	94.88	O
ATOM	5848	CG	LYS	A	743	9.703	13.547	44.125	1.00	94.88	C
ATOM	5849	CD	LYS	A	743	9.422	12.344	45.031	1.00	94.88	C
ATOM	5850	CE	LYS	A	743	9.258	11.062	44.219	1.00	94.88	C
ATOM	5851	NZ	LYS	A	743	8.977	9.891	45.094	1.00	94.88	N
ATOM	5852	N	TRP	A	744	9.102	17.891	45.219	1.00	94.19	N
ATOM	5853	CA	TRP	A	744	8.992	19.047	46.094	1.00	94.19	C
ATOM	5854	C	TRP	A	744	7.680	19.016	46.875	1.00	94.19	C
ATOM	5855	CB	TRP	A	744	9.094	20.344	45.281	1.00	94.19	C
ATOM	5856	O	TRP	A	744	6.781	18.219	46.562	1.00	94.19	O
ATOM	5857	CG	TRP	A	744	8.008	20.531	44.281	1.00	94.19	C
ATOM	5858	CD1	TRP	A	744	6.809	21.156	44.469	1.00	94.19	C
ATOM	5859	CD2	TRP	A	744	8.023	20.062	42.938	1.00	94.19	C
ATOM	5860	CE2	TRP	A	744	6.801	20.453	42.344	1.00	94.19	C
ATOM	5861	CE3	TRP	A	744	8.961	19.375	42.156	1.00	94.19	C
ATOM	5862	NE1	TRP	A	744	6.078	21.109	43.281	1.00	94.19	N
ATOM	5863	CH2	TRP	A	744	7.414	19.484	40.281	1.00	94.19	C
ATOM	5864	CZ2	TRP	A	744	6.484	20.156	41.000	1.00	94.19	C
ATOM	5865	CZ3	TRP	A	744	8.641	19.078	40.812	1.00	94.19	C
ATOM	5866	N	GLY	A	745	7.668	19.625	48.062	1.00	90.38	N
ATOM	5867	CA	GLY	A	745	6.445	19.844	48.812	1.00	90.38	C
ATOM	5868	C	GLY	A	745	5.895	21.250	48.656	1.00	90.38	C
ATOM	5869	O	GLY	A	745	6.590	22.141	48.188	1.00	90.38	O
ATOM	5870	N	MET	A	746	4.637	21.359	49.062	1.00	87.88	N
ATOM	5871	CA	MET	A	746	4.000	22.672	49.031	1.00	87.88	C
ATOM	5872	C	MET	A	746	3.641	23.109	50.469	1.00	87.88	C
ATOM	5873	CB	MET	A	746	2.746	22.656	48.156	1.00	87.88	C
ATOM	5874	O	MET	A	746	3.098	22.312	51.250	1.00	87.88	O
ATOM	5875	CG	MET	A	746	3.033	22.453	46.688	1.00	87.88	C
ATOM	5876	SD	MET	A	746	3.809	23.922	45.906	1.00	87.88	S
ATOM	5877	CE	MET	A	746	4.082	23.297	44.250	1.00	87.88	C
ATOM	5878	N	PRO	A	747	4.094	24.281	50.906	1.00	83.94	N
ATOM	5879	CA	PRO	A	747	3.699	24.781	52.219	1.00	83.94	C
ATOM	5880	C	PRO	A	747	2.184	24.859	52.406	1.00	83.94	C
ATOM	5881	CB	PRO	A	747	4.324	26.172	52.281	1.00	83.94	C
ATOM	5882	O	PRO	A	747	1.445	24.844	51.406	1.00	83.94	O
ATOM	5883	CG	PRO	A	747	4.566	26.531	50.844	1.00	83.94	C
ATOM	5884	CD	PRO	A	747	4.613	25.266	50.031	1.00	83.94	C

ATOM	5885	N	GLU	A	748	1.707	24.703	53.594	1.00	75.88	N
ATOM	5886	CA	GLU	A	748	0.301	24.906	53.938	1.00	75.88	C
ATOM	5887	C	GLU	A	748	-0.216	26.234	53.406	1.00	75.88	C
ATOM	5888	CB	GLU	A	748	0.101	24.828	55.438	1.00	75.88	C
ATOM	5889	O	GLU	A	748	0.474	27.250	53.469	1.00	75.88	O
ATOM	5890	CG	GLU	A	748	-1.276	24.344	55.875	1.00	75.88	C
ATOM	5891	CD	GLU	A	748	-1.394	24.078	57.344	1.00	75.88	C
ATOM	5892	OE1	GLU	A	748	-2.473	23.625	57.812	1.00	75.88	O
ATOM	5893	OE2	GLU	A	748	-0.398	24.312	58.062	1.00	75.88	O
ATOM	5894	N	GLY	A	749	-1.268	26.188	52.562	1.00	70.81	N
ATOM	5895	CA	GLY	A	749	-1.892	27.359	51.969	1.00	70.81	C
ATOM	5896	C	GLY	A	749	-1.437	27.641	50.562	1.00	70.81	C
ATOM	5897	O	GLY	A	749	-1.659	28.734	50.031	1.00	70.81	O
ATOM	5898	N	SER	A	750	-0.624	26.688	49.938	1.00	75.56	N
ATOM	5899	CA	SER	A	750	-0.075	26.875	48.594	1.00	75.56	C
ATOM	5900	C	SER	A	750	-1.029	26.344	47.531	1.00	75.56	C
ATOM	5901	CB	SER	A	750	1.280	26.172	48.469	1.00	75.56	C
ATOM	5902	O	SER	A	750	-0.620	26.078	46.375	1.00	75.56	O
ATOM	5903	OG	SER	A	750	1.572	25.422	49.625	1.00	75.56	O
ATOM	5904	N	GLY	A	751	-2.273	26.000	47.875	1.00	72.25	N
ATOM	5905	CA	GLY	A	751	-3.275	25.453	46.969	1.00	72.25	C
ATOM	5906	C	GLY	A	751	-3.520	26.328	45.750	1.00	72.25	C
ATOM	5907	O	GLY	A	751	-4.039	25.859	44.750	1.00	72.25	O
ATOM	5908	N	ASP	A	752	-2.938	27.547	45.781	1.00	72.25	N
ATOM	5909	CA	ASP	A	752	-3.174	28.469	44.688	1.00	72.25	C
ATOM	5910	C	ASP	A	752	-2.059	28.391	43.656	1.00	72.25	C
ATOM	5911	CB	ASP	A	752	-3.299	29.906	45.219	1.00	72.25	C
ATOM	5912	O	ASP	A	752	-2.027	29.188	42.719	1.00	72.25	O
ATOM	5913	CG	ASP	A	752	-2.090	30.344	46.031	1.00	72.25	C
ATOM	5914	OD1	ASP	A	752	-1.340	29.484	46.531	1.00	72.25	O
ATOM	5915	OD2	ASP	A	752	-1.885	31.578	46.156	1.00	72.25	O
ATOM	5916	N	THR	A	753	-1.011	27.578	43.812	1.00	83.25	N
ATOM	5917	CA	THR	A	753	0.091	27.391	42.875	1.00	83.25	C
ATOM	5918	C	THR	A	753	-0.418	26.844	41.531	1.00	83.25	C
ATOM	5919	CB	THR	A	753	1.155	26.438	43.438	1.00	83.25	C
ATOM	5920	O	THR	A	753	-1.213	25.906	41.500	1.00	83.25	O
ATOM	5921	CG2	THR	A	753	2.324	26.266	42.469	1.00	83.25	C
ATOM	5922	OG1	THR	A	753	1.644	26.969	44.688	1.00	83.25	O
ATOM	5923	N	ALA	A	754	-0.169	27.625	40.375	1.00	84.50	N
ATOM	5924	CA	ALA	A	754	-0.522	27.156	39.031	1.00	84.50	C
ATOM	5925	C	ALA	A	754	0.449	26.078	38.562	1.00	84.50	C
ATOM	5926	CB	ALA	A	754	-0.549	28.312	38.031	1.00	84.50	C
ATOM	5927	O	ALA	A	754	0.033	24.984	38.156	1.00	84.50	O
ATOM	5928	N	TYR	A	755	1.721	26.438	38.656	1.00	89.88	N
ATOM	5929	CA	TYR	A	755	2.750	25.484	38.250	1.00	89.88	C
ATOM	5930	C	TYR	A	755	4.066	25.766	38.969	1.00	89.88	C
ATOM	5931	CB	TYR	A	755	2.957	25.531	36.750	1.00	89.88	C
ATOM	5932	O	TYR	A	755	4.230	26.828	39.594	1.00	89.88	O
ATOM	5933	CG	TYR	A	755	3.217	26.922	36.219	1.00	89.88	C
ATOM	5934	CD1	TYR	A	755	2.180	27.703	35.688	1.00	89.88	C
ATOM	5935	CD2	TYR	A	755	4.500	27.453	36.188	1.00	89.88	C
ATOM	5936	CE1	TYR	A	755	2.414	28.969	35.188	1.00	89.88	C
ATOM	5937	CE2	TYR	A	755	4.750	28.734	35.719	1.00	89.88	C
ATOM	5938	OH	TYR	A	755	3.939	30.750	34.719	1.00	89.88	O
ATOM	5939	CZ	TYR	A	755	3.699	29.484	35.219	1.00	89.88	C
ATOM	5940	N	ILE	A	756	4.930	24.922	39.031	1.00	94.00	N
ATOM	5941	CA	ILE	A	756	6.297	25.016	39.531	1.00	94.00	C
ATOM	5942	C	ILE	A	756	7.266	25.219	38.375	1.00	94.00	C
ATOM	5943	CB	ILE	A	756	6.676	23.734	40.344	1.00	94.00	C
ATOM	5944	O	ILE	A	756	7.191	24.531	37.375	1.00	94.00	O
ATOM	5945	CG1	ILE	A	756	5.797	23.594	41.594	1.00	94.00	C
ATOM	5946	CG2	ILE	A	756	8.164	23.766	40.719	1.00	94.00	C
ATOM	5947	CD1	ILE	A	756	6.008	24.703	42.625	1.00	94.00	C
ATOM	5948	N	GLU	A	757	8.039	26.297	38.438	1.00	94.94	N

ATOM	5949	CA	GLU	A	757	9.156	26.531	37.531	1.00	94.94	C
ATOM	5950	C	GLU	A	757	10.406	25.781	38.000	1.00	94.94	C
ATOM	5951	CB	GLU	A	757	9.445	28.031	37.406	1.00	94.94	C
ATOM	5952	O	GLU	A	757	10.969	26.109	39.031	1.00	94.94	O
ATOM	5953	CG	GLU	A	757	10.430	28.375	36.281	1.00	94.94	C
ATOM	5954	CD	GLU	A	757	10.617	29.859	36.094	1.00	94.94	C
ATOM	5955	OE1	GLU	A	757	11.641	30.281	35.500	1.00	94.94	O
ATOM	5956	OE2	GLU	A	757	9.727	30.625	36.531	1.00	94.94	O
ATOM	5957	N	LEU	A	758	10.875	24.781	37.188	1.00	96.38	N
ATOM	5958	CA	LEU	A	758	12.031	23.953	37.531	1.00	96.38	C
ATOM	5959	C	LEU	A	758	13.203	24.281	36.625	1.00	96.38	C
ATOM	5960	CB	LEU	A	758	11.672	22.469	37.406	1.00	96.38	C
ATOM	5961	O	LEU	A	758	13.094	24.188	35.375	1.00	96.38	O
ATOM	5962	CG	LEU	A	758	12.727	21.469	37.906	1.00	96.38	C
ATOM	5963	CD1	LEU	A	758	12.938	21.641	39.406	1.00	96.38	C
ATOM	5964	CD2	LEU	A	758	12.312	20.047	37.562	1.00	96.38	C
ATOM	5965	N	HIS	A	759	14.281	24.641	37.156	1.00	96.25	N
ATOM	5966	CA	HIS	A	759	15.516	24.953	36.438	1.00	96.25	C
ATOM	5967	C	HIS	A	759	16.578	23.891	36.688	1.00	96.25	C
ATOM	5968	CB	HIS	A	759	16.031	26.328	36.844	1.00	96.25	C
ATOM	5969	O	HIS	A	759	16.562	23.234	37.719	1.00	96.25	O
ATOM	5970	CG	HIS	A	759	15.281	27.469	36.219	1.00	96.25	C
ATOM	5971	CD2	HIS	A	759	13.977	27.594	35.906	1.00	96.25	C
ATOM	5972	ND1	HIS	A	759	15.891	28.656	35.875	1.00	96.25	N
ATOM	5973	CE1	HIS	A	759	14.984	29.469	35.344	1.00	96.25	C
ATOM	5974	NE2	HIS	A	759	13.812	28.844	35.344	1.00	96.25	N
ATOM	5975	N	GLN	A	760	17.469	23.719	35.656	1.00	95.31	N
ATOM	5976	CA	GLN	A	760	18.594	22.797	35.781	1.00	95.31	C
ATOM	5977	C	GLN	A	760	19.922	23.531	35.594	1.00	95.31	C
ATOM	5978	CB	GLN	A	760	18.469	21.656	34.781	1.00	95.31	C
ATOM	5979	O	GLN	A	760	19.984	24.547	34.906	1.00	95.31	O
ATOM	5980	CG	GLN	A	760	18.609	22.094	33.344	1.00	95.31	C
ATOM	5981	CD	GLN	A	760	18.594	20.922	32.375	1.00	95.31	C
ATOM	5982	NE2	GLN	A	760	18.406	21.219	31.078	1.00	95.31	N
ATOM	5983	OE1	GLN	A	760	18.750	19.766	32.750	1.00	95.31	O
ATOM	5984	N	ALA	A	761	20.891	23.047	36.250	1.00	94.94	N
ATOM	5985	CA	ALA	A	761	22.250	23.562	36.125	1.00	94.94	C
ATOM	5986	C	ALA	A	761	23.266	22.422	36.062	1.00	94.94	C
ATOM	5987	CB	ALA	A	761	22.578	24.500	37.281	1.00	94.94	C
ATOM	5988	O	ALA	A	761	23.094	21.406	36.750	1.00	94.94	O
ATOM	5989	N	PRO	A	762	24.266	22.562	35.219	1.00	92.75	N
ATOM	5990	CA	PRO	A	762	25.312	21.531	35.156	1.00	92.75	C
ATOM	5991	C	PRO	A	762	26.156	21.469	36.438	1.00	92.75	C
ATOM	5992	CB	PRO	A	762	26.172	21.953	33.969	1.00	92.75	C
ATOM	5993	O	PRO	A	762	26.234	22.453	37.188	1.00	92.75	O
ATOM	5994	CG	PRO	A	762	25.875	23.406	33.781	1.00	92.75	C
ATOM	5995	CD	PRO	A	762	24.547	23.719	34.406	1.00	92.75	C
ATOM	5996	N	ASN	A	763	26.781	20.234	36.688	1.00	92.19	N
ATOM	5997	CA	ASN	A	763	27.719	20.031	37.781	1.00	92.19	C
ATOM	5998	C	ASN	A	763	29.078	20.672	37.500	1.00	92.19	C
ATOM	5999	CB	ASN	A	763	27.891	18.547	38.094	1.00	92.19	C
ATOM	6000	O	ASN	A	763	29.688	20.391	36.469	1.00	92.19	O
ATOM	6001	CG	ASN	A	763	28.531	18.297	39.469	1.00	92.19	C
ATOM	6002	ND2	ASN	A	763	29.453	17.344	39.500	1.00	92.19	N
ATOM	6003	OD1	ASN	A	763	28.203	18.969	40.438	1.00	92.19	O
ATOM	6004	N	GLY	A	764	29.516	21.672	38.312	1.00	86.38	N
ATOM	6005	CA	GLY	A	764	30.812	22.281	38.156	1.00	86.38	C
ATOM	6006	C	GLY	A	764	31.953	21.391	38.562	1.00	86.38	C
ATOM	6007	O	GLY	A	764	31.734	20.312	39.125	1.00	86.38	O
ATOM	6008	N	SER	A	765	33.219	21.750	38.219	1.00	82.75	N
ATOM	6009	CA	SER	A	765	34.406	21.031	38.562	1.00	82.75	C
ATOM	6010	C	SER	A	765	34.594	20.922	40.094	1.00	82.75	C
ATOM	6011	CB	SER	A	765	35.656	21.688	37.938	1.00	82.75	C
ATOM	6012	O	SER	A	765	35.250	20.000	40.594	1.00	82.75	O

ATOM	6013	OG	SER	A	765	35.594	23.094	38.125	1.00	82.75	O
ATOM	6014	N	ASP	A	766	33.938	21.797	40.844	1.00	82.75	N
ATOM	6015	CA	ASP	A	766	34.031	21.844	42.312	1.00	82.75	C
ATOM	6016	C	ASP	A	766	32.906	21.047	42.969	1.00	82.75	C
ATOM	6017	CB	ASP	A	766	34.000	23.297	42.812	1.00	82.75	C
ATOM	6018	O	ASP	A	766	32.781	21.047	44.188	1.00	82.75	O
ATOM	6019	CG	ASP	A	766	32.781	24.047	42.375	1.00	82.75	C
ATOM	6020	OD1	ASP	A	766	32.000	23.531	41.531	1.00	82.75	O
ATOM	6021	OD2	ASP	A	766	32.562	25.172	42.875	1.00	82.75	O
ATOM	6022	N	GLY	A	767	32.062	20.344	42.188	1.00	83.06	N
ATOM	6023	CA	GLY	A	767	30.969	19.547	42.719	1.00	83.06	C
ATOM	6024	C	GLY	A	767	29.750	20.391	43.094	1.00	83.06	C
ATOM	6025	O	GLY	A	767	28.875	19.922	43.812	1.00	83.06	O
ATOM	6026	N	HIS	A	768	29.766	21.672	42.688	1.00	89.19	N
ATOM	6027	CA	HIS	A	768	28.656	22.609	42.938	1.00	89.19	C
ATOM	6028	C	HIS	A	768	28.047	23.078	41.625	1.00	89.19	C
ATOM	6029	CB	HIS	A	768	29.141	23.812	43.750	1.00	89.19	C
ATOM	6030	O	HIS	A	768	28.672	22.953	40.562	1.00	89.19	O
ATOM	6031	CG	HIS	A	768	29.641	23.438	45.125	1.00	89.19	C
ATOM	6032	CD2	HIS	A	768	30.891	23.375	45.625	1.00	89.19	C
ATOM	6033	ND1	HIS	A	768	28.781	23.094	46.156	1.00	89.19	N
ATOM	6034	CE1	HIS	A	768	29.500	22.828	47.219	1.00	89.19	C
ATOM	6035	NE2	HIS	A	768	30.781	22.984	46.938	1.00	89.19	N
ATOM	6036	N	PRO	A	769	26.719	23.500	41.625	1.00	92.50	N
ATOM	6037	CA	PRO	A	769	26.078	23.969	40.406	1.00	92.50	C
ATOM	6038	C	PRO	A	769	26.766	25.188	39.812	1.00	92.50	C
ATOM	6039	CB	PRO	A	769	24.656	24.328	40.844	1.00	92.50	C
ATOM	6040	O	PRO	A	769	27.266	26.047	40.531	1.00	92.50	O
ATOM	6041	CG	PRO	A	769	24.797	24.703	42.312	1.00	92.50	C
ATOM	6042	CD	PRO	A	769	25.891	23.859	42.906	1.00	92.50	C
ATOM	6043	N	ILE	A	770	27.000	25.219	38.562	1.00	90.69	N
ATOM	6044	CA	ILE	A	770	27.359	26.438	37.875	1.00	90.69	C
ATOM	6045	C	ILE	A	770	26.141	27.344	37.719	1.00	90.69	C
ATOM	6046	CB	ILE	A	770	27.969	26.156	36.469	1.00	90.69	C
ATOM	6047	O	ILE	A	770	25.359	27.219	36.781	1.00	90.69	O
ATOM	6048	CG1	ILE	A	770	29.109	25.125	36.594	1.00	90.69	C
ATOM	6049	CG2	ILE	A	770	28.453	27.438	35.812	1.00	90.69	C
ATOM	6050	CD1	ILE	A	770	29.672	24.672	35.250	1.00	90.69	C
ATOM	6051	N	VAL	A	771	25.906	28.250	38.688	1.00	90.12	N
ATOM	6052	CA	VAL	A	771	24.688	29.016	38.906	1.00	90.12	C
ATOM	6053	C	VAL	A	771	24.375	29.859	37.656	1.00	90.12	C
ATOM	6054	CB	VAL	A	771	24.781	29.922	40.156	1.00	90.12	C
ATOM	6055	O	VAL	A	771	23.219	29.984	37.250	1.00	90.12	O
ATOM	6056	CG1	VAL	A	771	23.516	30.766	40.312	1.00	90.12	C
ATOM	6057	CG2	VAL	A	771	25.031	29.078	41.406	1.00	90.12	C
ATOM	6058	N	ASP	A	772	25.406	30.359	36.938	1.00	87.38	N
ATOM	6059	CA	ASP	A	772	25.250	31.250	35.781	1.00	87.38	C
ATOM	6060	C	ASP	A	772	24.781	30.469	34.531	1.00	87.38	C
ATOM	6061	CB	ASP	A	772	26.578	31.953	35.469	1.00	87.38	C
ATOM	6062	O	ASP	A	772	24.281	31.047	33.594	1.00	87.38	O
ATOM	6063	CG	ASP	A	772	26.984	32.938	36.562	1.00	87.38	C
ATOM	6064	OD1	ASP	A	772	26.125	33.375	37.344	1.00	87.38	O
ATOM	6065	OD2	ASP	A	772	28.188	33.250	36.625	1.00	87.38	O
ATOM	6066	N	GLU	A	773	24.750	29.156	34.625	1.00	89.38	N
ATOM	6067	CA	GLU	A	773	24.328	28.328	33.500	1.00	89.38	C
ATOM	6068	C	GLU	A	773	23.000	27.625	33.781	1.00	89.38	C
ATOM	6069	CB	GLU	A	773	25.406	27.297	33.156	1.00	89.38	C
ATOM	6070	O	GLU	A	773	22.656	26.656	33.125	1.00	89.38	O
ATOM	6071	CG	GLU	A	773	26.688	27.906	32.594	1.00	89.38	C
ATOM	6072	CD	GLU	A	773	27.703	26.859	32.188	1.00	89.38	C
ATOM	6073	OE1	GLU	A	773	28.828	27.234	31.781	1.00	89.38	O
ATOM	6074	OE2	GLU	A	773	27.391	25.656	32.250	1.00	89.38	O
ATOM	6075	N	ALA	A	774	22.297	28.125	34.875	1.00	92.06	N
ATOM	6076	CA	ALA	A	774	20.984	27.562	35.188	1.00	92.06	C

ATOM	6077	C	ALA	A	774	19.953	27.938	34.125	1.00	92.06	C
ATOM	6078	CB	ALA	A	774	20.516	28.016	36.562	1.00	92.06	C
ATOM	6079	O	ALA	A	774	19.859	29.109	33.750	1.00	92.06	O
ATOM	6080	N	THR	A	775	19.312	26.953	33.531	1.00	93.62	N
ATOM	6081	CA	THR	A	775	18.312	27.172	32.469	1.00	93.62	C
ATOM	6082	C	THR	A	775	17.000	26.500	32.844	1.00	93.62	C
ATOM	6083	CB	THR	A	775	18.797	26.656	31.125	1.00	93.62	C
ATOM	6084	O	THR	A	775	16.969	25.578	33.656	1.00	93.62	O
ATOM	6085	CG2	THR	A	775	20.031	27.422	30.656	1.00	93.62	C
ATOM	6086	OG1	THR	A	775	19.141	25.266	31.234	1.00	93.62	O
ATOM	6087	N	LEU	A	776	15.867	27.047	32.281	1.00	95.38	N
ATOM	6088	CA	LEU	A	776	14.555	26.453	32.469	1.00	95.38	C
ATOM	6089	C	LEU	A	776	14.500	25.031	31.906	1.00	95.38	C
ATOM	6090	CB	LEU	A	776	13.469	27.312	31.812	1.00	95.38	C
ATOM	6091	O	LEU	A	776	14.805	24.828	30.734	1.00	95.38	O
ATOM	6092	CG	LEU	A	776	12.047	26.766	31.859	1.00	95.38	C
ATOM	6093	CD1	LEU	A	776	11.531	26.734	33.281	1.00	95.38	C
ATOM	6094	CD2	LEU	A	776	11.125	27.594	30.969	1.00	95.38	C
ATOM	6095	N	LEU	A	777	14.234	24.016	32.906	1.00	92.94	N
ATOM	6096	CA	LEU	A	777	14.039	22.641	32.438	1.00	92.94	C
ATOM	6097	C	LEU	A	777	12.617	22.438	31.938	1.00	92.94	C
ATOM	6098	CB	LEU	A	777	14.336	21.656	33.594	1.00	92.94	C
ATOM	6099	O	LEU	A	777	12.422	22.000	30.797	1.00	92.94	O
ATOM	6100	CG	LEU	A	777	14.172	20.172	33.250	1.00	92.94	C
ATOM	6101	CD1	LEU	A	777	15.133	19.766	32.125	1.00	92.94	C
ATOM	6102	CD2	LEU	A	777	14.398	19.328	34.500	1.00	92.94	C
ATOM	6103	N	THR	A	778	11.617	22.781	32.844	1.00	95.25	N
ATOM	6104	CA	THR	A	778	10.211	22.609	32.500	1.00	95.25	C
ATOM	6105	C	THR	A	778	9.312	23.359	33.469	1.00	95.25	C
ATOM	6106	CB	THR	A	778	9.820	21.109	32.500	1.00	95.25	C
ATOM	6107	O	THR	A	778	9.773	23.812	34.531	1.00	95.25	O
ATOM	6108	CG2	THR	A	778	9.750	20.562	33.906	1.00	95.25	C
ATOM	6109	OG1	THR	A	778	8.547	20.969	31.859	1.00	95.25	O
ATOM	6110	N	LEU	A	779	8.094	23.625	33.062	1.00	94.00	N
ATOM	6111	CA	LEU	A	779	7.031	24.125	33.906	1.00	94.00	C
ATOM	6112	C	LEU	A	779	6.066	23.016	34.312	1.00	94.00	C
ATOM	6113	CB	LEU	A	779	6.270	25.250	33.219	1.00	94.00	C
ATOM	6114	O	LEU	A	779	5.480	22.359	33.438	1.00	94.00	O
ATOM	6115	CG	LEU	A	779	7.098	26.469	32.781	1.00	94.00	C
ATOM	6116	CD1	LEU	A	779	6.246	27.422	31.953	1.00	94.00	C
ATOM	6117	CD2	LEU	A	779	7.680	27.188	34.000	1.00	94.00	C
ATOM	6118	N	ILE	A	780	5.848	22.781	35.688	1.00	92.75	N
ATOM	6119	CA	ILE	A	780	5.059	21.656	36.156	1.00	92.75	C
ATOM	6120	C	ILE	A	780	3.781	22.172	36.844	1.00	92.75	C
ATOM	6121	CB	ILE	A	780	5.863	20.781	37.156	1.00	92.75	C
ATOM	6122	O	ILE	A	780	3.840	22.906	37.812	1.00	92.75	O
ATOM	6123	CG1	ILE	A	780	7.176	20.312	36.531	1.00	92.75	C
ATOM	6124	CG2	ILE	A	780	5.031	19.594	37.625	1.00	92.75	C
ATOM	6125	CD1	ILE	A	780	8.406	20.578	37.375	1.00	92.75	C
ATOM	6126	N	PRO	A	781	2.578	21.750	36.250	1.00	89.75	N
ATOM	6127	CA	PRO	A	781	1.311	22.234	36.812	1.00	89.75	C
ATOM	6128	C	PRO	A	781	1.039	21.719	38.219	1.00	89.75	C
ATOM	6129	CB	PRO	A	781	0.269	21.703	35.812	1.00	89.75	C
ATOM	6130	O	PRO	A	781	1.364	20.562	38.531	1.00	89.75	O
ATOM	6131	CG	PRO	A	781	0.983	20.641	35.031	1.00	89.75	C
ATOM	6132	CD	PRO	A	781	2.461	20.859	35.188	1.00	89.75	C
ATOM	6133	N	PHE	A	782	0.735	22.453	39.281	1.00	83.50	N
ATOM	6134	CA	PHE	A	782	0.224	22.062	40.594	1.00	83.50	C
ATOM	6135	C	PHE	A	782	-0.988	21.156	40.438	1.00	83.50	C
ATOM	6136	CB	PHE	A	782	-0.144	23.297	41.406	1.00	83.50	C
ATOM	6137	O	PHE	A	782	-1.814	21.344	39.562	1.00	83.50	O
ATOM	6138	CG	PHE	A	782	-0.677	22.984	42.781	1.00	83.50	C
ATOM	6139	CD1	PHE	A	782	-2.043	22.844	43.000	1.00	83.50	C
ATOM	6140	CD2	PHE	A	782	0.188	22.812	43.844	1.00	83.50	C

ATOM	6141	CE1	PHE	A	782	-2.539	22.547	44.281	1.00	83.50	C
ATOM	6142	CE2	PHE	A	782	-0.300	22.516	45.125	1.00	83.50	C
ATOM	6143	CZ	PHE	A	782	-1.664	22.391	45.344	1.00	83.50	C
ATOM	6144	N	PRO	A	783	-1.324	20.219	41.219	1.00	85.00	N
ATOM	6145	CA	PRO	A	783	-0.577	19.938	42.469	1.00	85.00	C
ATOM	6146	C	PRO	A	783	0.550	18.938	42.250	1.00	85.00	C
ATOM	6147	CB	PRO	A	783	-1.646	19.375	43.406	1.00	85.00	C
ATOM	6148	O	PRO	A	783	0.979	18.281	43.219	1.00	85.00	O
ATOM	6149	CG	PRO	A	783	-2.613	18.672	42.500	1.00	85.00	C
ATOM	6150	CD	PRO	A	783	-2.652	19.391	41.188	1.00	85.00	C
ATOM	6151	N	GLN	A	784	0.998	18.891	40.844	1.00	87.56	N
ATOM	6152	CA	GLN	A	784	2.084	17.938	40.656	1.00	87.56	C
ATOM	6153	C	GLN	A	784	3.270	18.266	41.562	1.00	87.56	C
ATOM	6154	CB	GLN	A	784	2.529	17.906	39.188	1.00	87.56	C
ATOM	6155	O	GLN	A	784	3.564	19.438	41.781	1.00	87.56	O
ATOM	6156	CG	GLN	A	784	1.515	17.281	38.250	1.00	87.56	C
ATOM	6157	CD	GLN	A	784	2.043	17.109	36.844	1.00	87.56	C
ATOM	6158	NE2	GLN	A	784	1.229	16.531	35.969	1.00	87.56	N
ATOM	6159	OE1	GLN	A	784	3.172	17.516	36.531	1.00	87.56	O
ATOM	6160	N	TYR	A	785	3.783	17.422	42.156	1.00	89.25	N
ATOM	6161	CA	TYR	A	785	4.844	17.672	43.125	1.00	89.25	C
ATOM	6162	C	TYR	A	785	6.109	16.906	42.781	1.00	89.25	C
ATOM	6163	CB	TYR	A	785	4.383	17.281	44.531	1.00	89.25	C
ATOM	6164	O	TYR	A	785	6.992	16.719	43.625	1.00	89.25	O
ATOM	6165	CG	TYR	A	785	3.863	15.867	44.656	1.00	89.25	C
ATOM	6166	CD1	TYR	A	785	2.518	15.586	44.438	1.00	89.25	C
ATOM	6167	CD2	TYR	A	785	4.719	14.812	44.969	1.00	89.25	C
ATOM	6168	CE1	TYR	A	785	2.033	14.281	44.531	1.00	89.25	C
ATOM	6169	CE2	TYR	A	785	4.246	13.508	45.062	1.00	89.25	C
ATOM	6170	OH	TYR	A	785	2.430	11.969	44.938	1.00	89.25	O
ATOM	6171	CZ	TYR	A	785	2.904	13.258	44.844	1.00	89.25	C
ATOM	6172	N	GLU	A	786	6.148	16.359	41.500	1.00	92.69	N
ATOM	6173	CA	GLU	A	786	7.320	15.594	41.094	1.00	92.69	C
ATOM	6174	C	GLU	A	786	7.539	15.703	39.594	1.00	92.69	C
ATOM	6175	CB	GLU	A	786	7.184	14.125	41.500	1.00	92.69	C
ATOM	6176	O	GLU	A	786	6.598	15.961	38.844	1.00	92.69	O
ATOM	6177	CG	GLU	A	786	6.137	13.359	40.688	1.00	92.69	C
ATOM	6178	CD	GLU	A	786	6.090	11.875	41.031	1.00	92.69	C
ATOM	6179	OE1	GLU	A	786	5.227	11.156	40.469	1.00	92.69	O
ATOM	6180	OE2	GLU	A	786	6.922	11.430	41.844	1.00	92.69	O
ATOM	6181	N	TYR	A	787	8.766	15.680	39.031	1.00	94.00	N
ATOM	6182	CA	TYR	A	787	9.156	15.680	37.625	1.00	94.00	C
ATOM	6183	C	TYR	A	787	10.203	14.609	37.344	1.00	94.00	C
ATOM	6184	CB	TYR	A	787	9.695	17.062	37.219	1.00	94.00	C
ATOM	6185	O	TYR	A	787	11.180	14.484	38.094	1.00	94.00	O
ATOM	6186	CG	TYR	A	787	10.117	17.141	35.781	1.00	94.00	C
ATOM	6187	CD1	TYR	A	787	11.469	17.109	35.438	1.00	94.00	C
ATOM	6188	CD2	TYR	A	787	9.172	17.250	34.781	1.00	94.00	C
ATOM	6189	CE1	TYR	A	787	11.867	17.188	34.094	1.00	94.00	C
ATOM	6190	CE2	TYR	A	787	9.562	17.328	33.438	1.00	94.00	C
ATOM	6191	OH	TYR	A	787	11.297	17.375	31.797	1.00	94.00	O
ATOM	6192	CZ	TYR	A	787	10.906	17.297	33.125	1.00	94.00	C
ATOM	6193	N	TRP	A	788	10.031	13.773	36.188	1.00	92.94	N
ATOM	6194	CA	TRP	A	788	10.992	12.758	35.781	1.00	92.94	C
ATOM	6195	C	TRP	A	788	11.891	13.281	34.688	1.00	92.94	C
ATOM	6196	CB	TRP	A	788	10.266	11.492	35.312	1.00	92.94	C
ATOM	6197	O	TRP	A	788	11.453	13.461	33.531	1.00	92.94	O
ATOM	6198	CG	TRP	A	788	11.180	10.375	34.906	1.00	92.94	C
ATOM	6199	CD1	TRP	A	788	11.219	9.727	33.719	1.00	92.94	C
ATOM	6200	CD2	TRP	A	788	12.195	9.766	35.750	1.00	92.94	C
ATOM	6201	CE2	TRP	A	788	12.805	8.758	34.969	1.00	92.94	C
ATOM	6202	CE3	TRP	A	788	12.641	9.984	37.031	1.00	92.94	C
ATOM	6203	NE1	TRP	A	788	12.195	8.750	33.750	1.00	92.94	N
ATOM	6204	CH2	TRP	A	788	14.266	8.195	36.750	1.00	92.94	C

ATOM	6205	CZ2	TRP	A	788	13.844	7.965	35.469	1.00	92.94	C
ATOM	6206	CZ3	TRP	A	788	13.672	9.195	37.531	1.00	92.94	C
ATOM	6207	N	HIS	A	789	13.070	13.555	34.844	1.00	93.25	N
ATOM	6208	CA	HIS	A	789	14.070	14.008	33.875	1.00	93.25	C
ATOM	6209	C	HIS	A	789	14.867	12.836	33.312	1.00	93.25	C
ATOM	6210	CB	HIS	A	789	15.016	15.016	34.531	1.00	93.25	C
ATOM	6211	O	HIS	A	789	15.703	12.258	34.000	1.00	93.25	O
ATOM	6212	CG	HIS	A	789	15.906	15.727	33.562	1.00	93.25	C
ATOM	6213	CD2	HIS	A	789	16.062	15.578	32.219	1.00	93.25	C
ATOM	6214	ND1	HIS	A	789	16.766	16.734	33.938	1.00	93.25	N
ATOM	6215	CE1	HIS	A	789	17.422	17.172	32.875	1.00	93.25	C
ATOM	6216	NE2	HIS	A	789	17.016	16.484	31.828	1.00	93.25	N
ATOM	6217	N	SER	A	790	14.531	12.281	32.062	1.00	89.31	N
ATOM	6218	CA	SER	A	790	15.109	11.180	31.312	1.00	89.31	C
ATOM	6219	C	SER	A	790	15.102	11.477	29.812	1.00	89.31	C
ATOM	6220	CB	SER	A	790	14.344	9.883	31.578	1.00	89.31	C
ATOM	6221	O	SER	A	790	14.359	12.352	29.344	1.00	89.31	O
ATOM	6222	OG	SER	A	790	13.195	9.789	30.750	1.00	89.31	O
ATOM	6223	N	ILE	A	791	16.156	10.781	28.922	1.00	86.31	N
ATOM	6224	CA	ILE	A	791	17.141	9.734	29.188	1.00	86.31	C
ATOM	6225	C	ILE	A	791	18.469	10.367	29.562	1.00	86.31	C
ATOM	6226	CB	ILE	A	791	17.297	8.789	27.984	1.00	86.31	C
ATOM	6227	O	ILE	A	791	18.953	11.273	28.891	1.00	86.31	O
ATOM	6228	CG1	ILE	A	791	15.969	8.133	27.625	1.00	86.31	C
ATOM	6229	CG2	ILE	A	791	18.375	7.734	28.266	1.00	86.31	C
ATOM	6230	CD1	ILE	A	791	15.969	7.414	26.281	1.00	86.31	C
ATOM	6231	N	LEU	A	792	18.984	10.141	30.781	1.00	87.75	N
ATOM	6232	CA	LEU	A	792	20.297	10.609	31.250	1.00	87.75	C
ATOM	6233	C	LEU	A	792	21.281	9.453	31.344	1.00	87.75	C
ATOM	6234	CB	LEU	A	792	20.156	11.289	32.625	1.00	87.75	C
ATOM	6235	O	LEU	A	792	20.906	8.328	31.656	1.00	87.75	O
ATOM	6236	CG	LEU	A	792	19.312	12.570	32.656	1.00	87.75	C
ATOM	6237	CD1	LEU	A	792	19.016	12.969	34.094	1.00	87.75	C
ATOM	6238	CD2	LEU	A	792	20.047	13.695	31.922	1.00	87.75	C
ATOM	6239	N	PRO	A	793	22.578	9.703	30.906	1.00	84.56	N
ATOM	6240	CA	PRO	A	793	23.578	8.664	31.109	1.00	84.56	C
ATOM	6241	C	PRO	A	793	23.734	8.266	32.562	1.00	84.56	C
ATOM	6242	CB	PRO	A	793	24.859	9.312	30.578	1.00	84.56	C
ATOM	6243	O	PRO	A	793	23.438	9.062	33.469	1.00	84.56	O
ATOM	6244	CG	PRO	A	793	24.406	10.523	29.828	1.00	84.56	C
ATOM	6245	CD	PRO	A	793	23.031	10.906	30.344	1.00	84.56	C
ATOM	6246	N	ALA	A	794	24.094	7.004	32.844	1.00	82.00	N
ATOM	6247	CA	ALA	A	794	24.328	6.531	34.188	1.00	82.00	C
ATOM	6248	C	ALA	A	794	25.375	7.391	34.906	1.00	82.00	C
ATOM	6249	CB	ALA	A	794	24.766	5.070	34.188	1.00	82.00	C
ATOM	6250	O	ALA	A	794	26.406	7.723	34.312	1.00	82.00	O
ATOM	6251	N	GLY	A	795	25.062	7.871	36.094	1.00	85.00	N
ATOM	6252	CA	GLY	A	795	25.984	8.625	36.938	1.00	85.00	C
ATOM	6253	C	GLY	A	795	25.984	10.117	36.625	1.00	85.00	C
ATOM	6254	O	GLY	A	795	26.734	10.875	37.250	1.00	85.00	O
ATOM	6255	N	HIS	A	796	25.125	10.492	35.625	1.00	89.38	N
ATOM	6256	CA	HIS	A	796	25.078	11.898	35.219	1.00	89.38	C
ATOM	6257	C	HIS	A	796	24.469	12.750	36.344	1.00	89.38	C
ATOM	6258	CB	HIS	A	796	24.297	12.078	33.938	1.00	89.38	C
ATOM	6259	O	HIS	A	796	23.391	12.430	36.844	1.00	89.38	O
ATOM	6260	CG	HIS	A	796	24.531	13.398	33.250	1.00	89.38	C
ATOM	6261	CD2	HIS	A	796	25.422	13.750	32.281	1.00	89.38	C
ATOM	6262	ND1	HIS	A	796	23.828	14.531	33.562	1.00	89.38	N
ATOM	6263	CE1	HIS	A	796	24.250	15.531	32.812	1.00	89.38	C
ATOM	6264	NE2	HIS	A	796	25.219	15.086	32.031	1.00	89.38	N
ATOM	6265	N	VAL	A	797	25.172	13.844	36.719	1.00	91.75	N
ATOM	6266	CA	VAL	A	797	24.750	14.719	37.812	1.00	91.75	C
ATOM	6267	C	VAL	A	797	24.172	16.000	37.250	1.00	91.75	C
ATOM	6268	CB	VAL	A	797	25.922	15.047	38.781	1.00	91.75	C

ATOM	6269	O	VAL	A	797	24.750	16.625	36.375	1.00	91.75	O
ATOM	6270	CG1	VAL	A	797	25.484	16.031	39.844	1.00	91.75	C
ATOM	6271	CG2	VAL	A	797	26.469	13.766	39.406	1.00	91.75	C
ATOM	6272	N	VAL	A	798	23.000	16.406	37.750	1.00	94.62	N
ATOM	6273	CA	VAL	A	798	22.328	17.656	37.406	1.00	94.62	C
ATOM	6274	C	VAL	A	798	21.828	18.312	38.688	1.00	94.62	C
ATOM	6275	CB	VAL	A	798	21.141	17.438	36.438	1.00	94.62	C
ATOM	6276	O	VAL	A	798	21.406	17.625	39.625	1.00	94.62	O
ATOM	6277	CG1	VAL	A	798	20.484	18.766	36.094	1.00	94.62	C
ATOM	6278	CG2	VAL	A	798	21.625	16.719	35.188	1.00	94.62	C
ATOM	6279	N	TRP	A	799	21.906	19.625	38.812	1.00	96.06	N
ATOM	6280	CA	TRP	A	799	21.391	20.406	39.938	1.00	96.06	C
ATOM	6281	C	TRP	A	799	20.078	21.078	39.594	1.00	96.06	C
ATOM	6282	CB	TRP	A	799	22.422	21.453	40.375	1.00	96.06	C
ATOM	6283	O	TRP	A	799	19.891	21.531	38.469	1.00	96.06	O
ATOM	6284	CG	TRP	A	799	23.688	20.875	40.938	1.00	96.06	C
ATOM	6285	CD1	TRP	A	799	24.734	20.375	40.250	1.00	96.06	C
ATOM	6286	CD2	TRP	A	799	24.016	20.734	42.312	1.00	96.06	C
ATOM	6287	CE2	TRP	A	799	25.297	20.141	42.375	1.00	96.06	C
ATOM	6288	CE3	TRP	A	799	23.359	21.062	43.531	1.00	96.06	C
ATOM	6289	NE1	TRP	A	799	25.719	19.922	41.094	1.00	96.06	N
ATOM	6290	CH2	TRP	A	799	25.266	20.188	44.750	1.00	96.06	C
ATOM	6291	CZ2	TRP	A	799	25.938	19.859	43.594	1.00	96.06	C
ATOM	6292	CZ3	TRP	A	799	24.000	20.781	44.719	1.00	96.06	C
ATOM	6293	N	TYR	A	800	19.094	21.078	40.594	1.00	96.44	N
ATOM	6294	CA	TYR	A	800	17.750	21.625	40.344	1.00	96.44	C
ATOM	6295	C	TYR	A	800	17.391	22.688	41.344	1.00	96.44	C
ATOM	6296	CB	TYR	A	800	16.719	20.500	40.344	1.00	96.44	C
ATOM	6297	O	TYR	A	800	17.812	22.625	42.500	1.00	96.44	O
ATOM	6298	CG	TYR	A	800	16.938	19.453	39.281	1.00	96.44	C
ATOM	6299	CD1	TYR	A	800	16.422	19.609	38.000	1.00	96.44	C
ATOM	6300	CD2	TYR	A	800	17.656	18.297	39.562	1.00	96.44	C
ATOM	6301	CE1	TYR	A	800	16.609	18.641	37.031	1.00	96.44	C
ATOM	6302	CE2	TYR	A	800	17.859	17.312	38.594	1.00	96.44	C
ATOM	6303	OH	TYR	A	800	17.531	16.531	36.375	1.00	96.44	O
ATOM	6304	CZ	TYR	A	800	17.344	17.500	37.344	1.00	96.44	C
ATOM	6305	N	LYS	A	801	16.672	23.672	41.000	1.00	96.06	N
ATOM	6306	CA	LYS	A	801	15.977	24.641	41.844	1.00	96.06	C
ATOM	6307	C	LYS	A	801	14.570	24.906	41.312	1.00	96.06	C
ATOM	6308	CB	LYS	A	801	16.766	25.953	41.938	1.00	96.06	C
ATOM	6309	O	LYS	A	801	14.305	24.734	40.125	1.00	96.06	O
ATOM	6310	CG	LYS	A	801	16.875	26.703	40.625	1.00	96.06	C
ATOM	6311	CD	LYS	A	801	17.656	28.000	40.781	1.00	96.06	C
ATOM	6312	CE	LYS	A	801	17.750	28.766	39.469	1.00	96.06	C
ATOM	6313	NZ	LYS	A	801	18.484	30.047	39.625	1.00	96.06	N
ATOM	6314	N	ALA	A	802	13.680	25.219	42.188	1.00	95.44	N
ATOM	6315	CA	ALA	A	802	12.289	25.375	41.781	1.00	95.44	C
ATOM	6316	C	ALA	A	802	11.656	26.594	42.438	1.00	95.44	C
ATOM	6317	CB	ALA	A	802	11.492	24.109	42.125	1.00	95.44	C
ATOM	6318	O	ALA	A	802	12.125	27.047	43.500	1.00	95.44	O
ATOM	6319	N	ARG	A	803	10.688	27.188	41.969	1.00	94.50	N
ATOM	6320	CA	ARG	A	803	9.844	28.219	42.562	1.00	94.50	C
ATOM	6321	C	ARG	A	803	8.398	28.062	42.125	1.00	94.50	C
ATOM	6322	CB	ARG	A	803	10.359	29.625	42.188	1.00	94.50	C
ATOM	6323	O	ARG	A	803	8.125	27.500	41.062	1.00	94.50	O
ATOM	6324	CG	ARG	A	803	10.148	29.984	40.719	1.00	94.50	C
ATOM	6325	CD	ARG	A	803	10.641	31.391	40.438	1.00	94.50	C
ATOM	6326	NE	ARG	A	803	10.461	31.734	39.031	1.00	94.50	N
ATOM	6327	NH1	ARG	A	803	11.492	33.812	39.188	1.00	94.50	N
ATOM	6328	NH2	ARG	A	803	10.656	33.094	37.156	1.00	94.50	N
ATOM	6329	CZ	ARG	A	803	10.875	32.875	38.469	1.00	94.50	C
ATOM	6330	N	ALA	A	804	7.480	28.406	42.906	1.00	91.12	N
ATOM	6331	CA	ALA	A	804	6.043	28.297	42.656	1.00	91.12	C
ATOM	6332	C	ALA	A	804	5.516	29.562	41.969	1.00	91.12	C

ATOM	6333	CB	ALA	A	804	5.293	28.047	43.938	1.00	91.12	C
ATOM	6334	O	ALA	A	804	5.922	30.672	42.312	1.00	91.12	O
ATOM	6335	N	VAL	A	805	4.785	29.312	40.938	1.00	88.12	N
ATOM	6336	CA	VAL	A	805	4.062	30.391	40.281	1.00	88.12	C
ATOM	6337	C	VAL	A	805	2.559	30.219	40.500	1.00	88.12	C
ATOM	6338	CB	VAL	A	805	4.387	30.469	38.781	1.00	88.12	C
ATOM	6339	O	VAL	A	805	2.010	29.156	40.188	1.00	88.12	O
ATOM	6340	CG1	VAL	A	805	3.682	31.656	38.125	1.00	88.12	C
ATOM	6341	CG2	VAL	A	805	5.895	30.547	38.562	1.00	88.12	C
ATOM	6342	N	ASP	A	806	1.890	31.000	41.219	1.00	81.75	N
ATOM	6343	CA	ASP	A	806	0.473	30.844	41.531	1.00	81.75	C
ATOM	6344	C	ASP	A	806	-0.395	31.109	40.312	1.00	81.75	C
ATOM	6345	CB	ASP	A	806	0.077	31.797	42.656	1.00	81.75	C
ATOM	6346	O	ASP	A	806	0.120	31.422	39.219	1.00	81.75	O
ATOM	6347	CG	ASP	A	806	0.147	33.250	42.250	1.00	81.75	C
ATOM	6348	OD1	ASP	A	806	0.192	33.562	41.031	1.00	81.75	O
ATOM	6349	OD2	ASP	A	806	0.160	34.125	43.156	1.00	81.75	O
ATOM	6350	N	LYS	A	807	-1.578	30.656	40.219	1.00	71.19	N
ATOM	6351	CA	LYS	A	807	-2.535	30.609	39.094	1.00	71.19	C
ATOM	6352	C	LYS	A	807	-2.709	31.984	38.469	1.00	71.19	C
ATOM	6353	CB	LYS	A	807	-3.887	30.078	39.594	1.00	71.19	C
ATOM	6354	O	LYS	A	807	-3.168	32.094	37.344	1.00	71.19	O
ATOM	6355	CG	LYS	A	807	-3.875	28.609	39.969	1.00	71.19	C
ATOM	6356	CD	LYS	A	807	-5.258	28.125	40.375	1.00	71.19	C
ATOM	6357	CE	LYS	A	807	-5.238	26.672	40.812	1.00	71.19	C
ATOM	6358	NZ	LYS	A	807	-6.578	26.219	41.281	1.00	71.19	N
ATOM	6359	N	ILE	A	808	-2.170	33.062	39.125	1.00	66.25	N
ATOM	6360	CA	ILE	A	808	-2.398	34.344	38.500	1.00	66.25	C
ATOM	6361	C	ILE	A	808	-1.059	35.000	38.156	1.00	66.25	C
ATOM	6362	CB	ILE	A	808	-3.227	35.281	39.438	1.00	66.25	C
ATOM	6363	O	ILE	A	808	-1.002	36.188	37.844	1.00	66.25	O
ATOM	6364	CG1	ILE	A	808	-2.594	35.344	40.812	1.00	66.25	C
ATOM	6365	CG2	ILE	A	808	-4.680	34.781	39.500	1.00	66.25	C
ATOM	6366	CD1	ILE	A	808	-3.201	36.406	41.719	1.00	66.25	C
ATOM	6367	N	GLY	A	809	0.145	34.250	38.219	1.00	68.75	N
ATOM	6368	CA	GLY	A	809	1.431	34.688	37.688	1.00	68.75	C
ATOM	6369	C	GLY	A	809	2.383	35.188	38.719	1.00	68.75	C
ATOM	6370	O	GLY	A	809	3.502	35.625	38.406	1.00	68.75	O
ATOM	6371	N	ASN	A	810	2.004	35.406	40.062	1.00	74.62	N
ATOM	6372	CA	ASN	A	810	2.938	35.750	41.125	1.00	74.62	C
ATOM	6373	C	ASN	A	810	3.947	34.625	41.375	1.00	74.62	C
ATOM	6374	CB	ASN	A	810	2.184	36.062	42.406	1.00	74.62	C
ATOM	6375	O	ASN	A	810	3.594	33.438	41.312	1.00	74.62	O
ATOM	6376	CG	ASN	A	810	1.222	37.250	42.250	1.00	74.62	C
ATOM	6377	ND2	ASN	A	810	0.020	37.094	42.781	1.00	74.62	N
ATOM	6378	OD1	ASN	A	810	1.556	38.250	41.625	1.00	74.62	O
ATOM	6379	N	VAL	A	811	5.234	35.062	41.469	1.00	85.19	N
ATOM	6380	CA	VAL	A	811	6.312	34.062	41.562	1.00	85.19	C
ATOM	6381	C	VAL	A	811	6.867	34.094	43.000	1.00	85.19	C
ATOM	6382	CB	VAL	A	811	7.441	34.344	40.562	1.00	85.19	C
ATOM	6383	O	VAL	A	811	6.977	35.125	43.625	1.00	85.19	O
ATOM	6384	CG1	VAL	A	811	6.938	34.156	39.125	1.00	85.19	C
ATOM	6385	CG2	VAL	A	811	8.008	35.750	40.750	1.00	85.19	C
ATOM	6386	N	SER	A	812	7.047	32.969	43.625	1.00	89.38	N
ATOM	6387	CA	SER	A	812	7.723	32.812	44.906	1.00	89.38	C
ATOM	6388	C	SER	A	812	9.227	33.031	44.781	1.00	89.38	C
ATOM	6389	CB	SER	A	812	7.449	31.422	45.500	1.00	89.38	C
ATOM	6390	O	SER	A	812	9.742	33.156	43.656	1.00	89.38	O
ATOM	6391	OG	SER	A	812	8.203	30.438	44.812	1.00	89.38	O
ATOM	6392	N	ASP	A	813	10.109	33.125	45.906	1.00	90.12	N
ATOM	6393	CA	ASP	A	813	11.562	33.000	45.938	1.00	90.12	C
ATOM	6394	C	ASP	A	813	11.992	31.594	45.469	1.00	90.12	C
ATOM	6395	CB	ASP	A	813	12.102	33.250	47.344	1.00	90.12	C
ATOM	6396	O	ASP	A	813	11.203	30.656	45.469	1.00	90.12	O

ATOM	6397	CG	ASP	A	813	12.008	34.719	47.750	1.00	90.12	C
ATOM	6398	OD1	ASP	A	813	11.953	35.594	46.875	1.00	90.12	O
ATOM	6399	OD2	ASP	A	813	11.984	35.000	48.969	1.00	90.12	O
ATOM	6400	N	TRP	A	814	13.273	31.484	44.938	1.00	93.19	N
ATOM	6401	CA	TRP	A	814	13.820	30.203	44.500	1.00	93.19	C
ATOM	6402	C	TRP	A	814	14.148	29.312	45.719	1.00	93.19	C
ATOM	6403	CB	TRP	A	814	15.086	30.406	43.656	1.00	93.19	C
ATOM	6404	O	TRP	A	814	14.547	29.812	46.750	1.00	93.19	O
ATOM	6405	CG	TRP	A	814	14.812	30.969	42.281	1.00	93.19	C
ATOM	6406	CD1	TRP	A	814	15.000	32.250	41.875	1.00	93.19	C
ATOM	6407	CD2	TRP	A	814	14.297	30.250	41.156	1.00	93.19	C
ATOM	6408	CE2	TRP	A	814	14.203	31.156	40.094	1.00	93.19	C
ATOM	6409	CE3	TRP	A	814	13.914	28.922	40.969	1.00	93.19	C
ATOM	6410	NE1	TRP	A	814	14.633	32.375	40.562	1.00	93.19	N
ATOM	6411	CH2	TRP	A	814	13.367	29.484	38.656	1.00	93.19	C
ATOM	6412	CZ2	TRP	A	814	13.734	30.781	38.844	1.00	93.19	C
ATOM	6413	CZ3	TRP	A	814	13.445	28.547	39.719	1.00	93.19	C
ATOM	6414	N	THR	A	815	14.008	28.031	45.531	1.00	94.12	N
ATOM	6415	CA	THR	A	815	14.578	27.062	46.469	1.00	94.12	C
ATOM	6416	C	THR	A	815	16.094	27.016	46.344	1.00	94.12	C
ATOM	6417	CB	THR	A	815	13.992	25.656	46.250	1.00	94.12	C
ATOM	6418	O	THR	A	815	16.656	27.547	45.375	1.00	94.12	O
ATOM	6419	CG2	THR	A	815	12.469	25.688	46.344	1.00	94.12	C
ATOM	6420	OG1	THR	A	815	14.391	25.172	44.969	1.00	94.12	O
ATOM	6421	N	ASP	A	816	16.781	26.375	47.312	1.00	93.38	N
ATOM	6422	CA	ASP	A	816	18.203	26.031	47.188	1.00	93.38	C
ATOM	6423	C	ASP	A	816	18.391	24.938	46.125	1.00	93.38	C
ATOM	6424	CB	ASP	A	816	18.781	25.578	48.531	1.00	93.38	C
ATOM	6425	O	ASP	A	816	17.484	24.125	45.875	1.00	93.38	O
ATOM	6426	CG	ASP	A	816	18.844	26.703	49.531	1.00	93.38	C
ATOM	6427	OD1	ASP	A	816	18.953	27.875	49.156	1.00	93.38	O
ATOM	6428	OD2	ASP	A	816	18.766	26.406	50.750	1.00	93.38	O
ATOM	6429	N	PHE	A	817	19.531	24.953	45.375	1.00	95.00	N
ATOM	6430	CA	PHE	A	817	19.844	23.891	44.438	1.00	95.00	C
ATOM	6431	C	PHE	A	817	19.891	22.547	45.125	1.00	95.00	C
ATOM	6432	CB	PHE	A	817	21.172	24.172	43.750	1.00	95.00	C
ATOM	6433	O	PHE	A	817	20.375	22.438	46.281	1.00	95.00	O
ATOM	6434	CG	PHE	A	817	21.062	25.125	42.562	1.00	95.00	C
ATOM	6435	CD1	PHE	A	817	20.594	24.688	41.344	1.00	95.00	C
ATOM	6436	CD2	PHE	A	817	21.406	26.469	42.719	1.00	95.00	C
ATOM	6437	CE1	PHE	A	817	20.500	25.562	40.250	1.00	95.00	C
ATOM	6438	CE2	PHE	A	817	21.297	27.344	41.656	1.00	95.00	C
ATOM	6439	CZ	PHE	A	817	20.844	26.891	40.406	1.00	95.00	C
ATOM	6440	N	VAL	A	818	19.281	21.562	44.531	1.00	94.25	N
ATOM	6441	CA	VAL	A	818	19.406	20.188	45.000	1.00	94.25	C
ATOM	6442	C	VAL	A	818	20.094	19.328	43.938	1.00	94.25	C
ATOM	6443	CB	VAL	A	818	18.047	19.578	45.375	1.00	94.25	C
ATOM	6444	O	VAL	A	818	19.875	19.531	42.750	1.00	94.25	O
ATOM	6445	CG1	VAL	A	818	17.453	20.312	46.594	1.00	94.25	C
ATOM	6446	CG2	VAL	A	818	17.094	19.641	44.188	1.00	94.25	C
ATOM	6447	N	ARG	A	819	21.000	18.484	44.281	1.00	94.06	N
ATOM	6448	CA	ARG	A	819	21.812	17.625	43.438	1.00	94.06	C
ATOM	6449	C	ARG	A	819	21.062	16.344	43.062	1.00	94.06	C
ATOM	6450	CB	ARG	A	819	23.141	17.281	44.125	1.00	94.06	C
ATOM	6451	O	ARG	A	819	20.531	15.664	43.938	1.00	94.06	O
ATOM	6452	CG	ARG	A	819	24.062	16.422	43.281	1.00	94.06	C
ATOM	6453	CD	ARG	A	819	25.375	16.125	44.000	1.00	94.06	C
ATOM	6454	NE	ARG	A	819	26.281	15.352	43.156	1.00	94.06	N
ATOM	6455	NH1	ARG	A	819	25.281	13.312	43.594	1.00	94.06	N
ATOM	6456	NH2	ARG	A	819	27.109	13.430	42.188	1.00	94.06	N
ATOM	6457	CZ	ARG	A	819	26.219	14.039	43.000	1.00	94.06	C
ATOM	6458	N	GLY	A	820	20.875	16.062	41.750	1.00	93.50	N
ATOM	6459	CA	GLY	A	820	20.359	14.805	41.219	1.00	93.50	C
ATOM	6460	C	GLY	A	820	21.375	14.039	40.406	1.00	93.50	C

ATOM	6461	O	GLY	A	820	22.219	14.641	39.719	1.00	93.50	O
ATOM	6462	N	MET	A	821	21.328	12.750	40.500	1.00	92.25	N
ATOM	6463	CA	MET	A	821	22.234	11.898	39.719	1.00	92.25	C
ATOM	6464	C	MET	A	821	21.500	10.656	39.219	1.00	92.25	C
ATOM	6465	CB	MET	A	821	23.422	11.492	40.594	1.00	92.25	C
ATOM	6466	O	MET	A	821	20.766	10.016	39.969	1.00	92.25	O
ATOM	6467	CG	MET	A	821	24.469	10.672	39.844	1.00	92.25	C
ATOM	6468	SD	MET	A	821	25.938	10.281	40.875	1.00	92.25	S
ATOM	6469	CE	MET	A	821	25.266	8.930	41.875	1.00	92.25	C
ATOM	6470	N	ALA	A	822	21.547	10.516	37.875	1.00	89.12	N
ATOM	6471	CA	ALA	A	822	21.062	9.234	37.375	1.00	89.12	C
ATOM	6472	C	ALA	A	822	21.781	8.070	38.062	1.00	89.12	C
ATOM	6473	CB	ALA	A	822	21.266	9.156	35.844	1.00	89.12	C
ATOM	6474	O	ALA	A	822	22.969	8.172	38.375	1.00	89.12	O
ATOM	6475	N	SER	A	823	21.031	7.027	38.438	1.00	85.62	N
ATOM	6476	CA	SER	A	823	21.547	5.922	39.219	1.00	85.62	C
ATOM	6477	C	SER	A	823	22.891	5.426	38.656	1.00	85.62	C
ATOM	6478	CB	SER	A	823	20.547	4.770	39.250	1.00	85.62	C
ATOM	6479	O	SER	A	823	23.031	5.277	37.438	1.00	85.62	O
ATOM	6480	OG	SER	A	823	21.062	3.686	40.031	1.00	85.62	O
ATOM	6481	N	ASP	A	824	23.906	5.363	39.375	1.00	80.62	N
ATOM	6482	CA	ASP	A	824	25.125	4.680	38.969	1.00	80.62	C
ATOM	6483	C	ASP	A	824	25.188	3.277	39.562	1.00	80.62	C
ATOM	6484	CB	ASP	A	824	26.359	5.488	39.406	1.00	80.62	C
ATOM	6485	O	ASP	A	824	26.266	2.672	39.625	1.00	80.62	O
ATOM	6486	CG	ASP	A	824	26.438	5.703	40.906	1.00	80.62	C
ATOM	6487	OD1	ASP	A	824	25.453	5.391	41.625	1.00	80.62	O
ATOM	6488	OD2	ASP	A	824	27.484	6.195	41.375	1.00	80.62	O
ATOM	6489	N	ASP	A	825	23.969	2.982	40.219	1.00	78.19	N
ATOM	6490	CA	ASP	A	825	23.859	1.646	40.812	1.00	78.19	C
ATOM	6491	C	ASP	A	825	23.719	0.586	39.719	1.00	78.19	C
ATOM	6492	CB	ASP	A	825	22.672	1.571	41.750	1.00	78.19	C
ATOM	6493	O	ASP	A	825	22.672	0.452	39.094	1.00	78.19	O
ATOM	6494	CG	ASP	A	825	22.641	0.285	42.562	1.00	78.19	C
ATOM	6495	OD1	ASP	A	825	23.234	-0.722	42.125	1.00	78.19	O
ATOM	6496	OD2	ASP	A	825	22.000	0.277	43.656	1.00	78.19	O
ATOM	6497	N	THR	A	826	24.781	-0.076	39.438	1.00	74.44	N
ATOM	6498	CA	THR	A	826	24.859	-1.040	38.344	1.00	74.44	C
ATOM	6499	C	THR	A	826	23.828	-2.143	38.531	1.00	74.44	C
ATOM	6500	CB	THR	A	826	26.266	-1.664	38.250	1.00	74.44	C
ATOM	6501	O	THR	A	826	23.328	-2.697	37.531	1.00	74.44	O
ATOM	6502	CG2	THR	A	826	27.328	-0.605	37.938	1.00	74.44	C
ATOM	6503	OG1	THR	A	826	26.594	-2.287	39.500	1.00	74.44	O
ATOM	6504	N	SER	A	827	23.406	-2.494	39.844	1.00	76.06	N
ATOM	6505	CA	SER	A	827	22.438	-3.562	40.062	1.00	76.06	C
ATOM	6506	C	SER	A	827	21.062	-3.178	39.562	1.00	76.06	C
ATOM	6507	CB	SER	A	827	22.359	-3.902	41.562	1.00	76.06	C
ATOM	6508	O	SER	A	827	20.391	-3.990	38.906	1.00	76.06	O
ATOM	6509	OG	SER	A	827	21.969	-2.771	42.312	1.00	76.06	O
ATOM	6510	N	ILE	A	828	20.594	-1.911	39.750	1.00	76.25	N
ATOM	6511	CA	ILE	A	828	19.281	-1.430	39.312	1.00	76.25	C
ATOM	6512	C	ILE	A	828	19.250	-1.327	37.781	1.00	76.25	C
ATOM	6513	CB	ILE	A	828	18.938	-0.063	39.938	1.00	76.25	C
ATOM	6514	O	ILE	A	828	18.266	-1.697	37.156	1.00	76.25	O
ATOM	6515	CG1	ILE	A	828	18.844	-0.195	41.469	1.00	76.25	C
ATOM	6516	CG2	ILE	A	828	17.656	0.501	39.344	1.00	76.25	C
ATOM	6517	CD1	ILE	A	828	18.688	1.133	42.188	1.00	76.25	C
ATOM	6518	N	ILE	A	829	20.391	-0.817	37.188	1.00	78.12	N
ATOM	6519	CA	ILE	A	829	20.484	-0.626	35.750	1.00	78.12	C
ATOM	6520	C	ILE	A	829	20.422	-1.980	35.031	1.00	78.12	C
ATOM	6521	CB	ILE	A	829	21.766	0.128	35.344	1.00	78.12	C
ATOM	6522	O	ILE	A	829	19.672	-2.160	34.062	1.00	78.12	O
ATOM	6523	CG1	ILE	A	829	21.750	1.552	35.906	1.00	78.12	C
ATOM	6524	CG2	ILE	A	829	21.953	0.149	33.844	1.00	78.12	C

ATOM	6525	CD1	ILE	A	829	23.078	2.285	35.812	1.00	78.12	C
ATOM	6526	N	THR	A	830	21.250	-2.916	35.531	1.00	78.31	N
ATOM	6527	CA	THR	A	830	21.266	-4.246	34.938	1.00	78.31	C
ATOM	6528	C	THR	A	830	19.906	-4.922	35.094	1.00	78.31	C
ATOM	6529	CB	THR	A	830	22.344	-5.133	35.562	1.00	78.31	C
ATOM	6530	O	THR	A	830	19.469	-5.645	34.188	1.00	78.31	O
ATOM	6531	CG2	THR	A	830	23.750	-4.703	35.125	1.00	78.31	C
ATOM	6532	OG1	THR	A	830	22.266	-5.031	37.000	1.00	78.31	O
ATOM	6533	N	ASP	A	831	19.141	-4.723	36.188	1.00	79.44	N
ATOM	6534	CA	ASP	A	831	17.812	-5.297	36.375	1.00	79.44	C
ATOM	6535	C	ASP	A	831	16.812	-4.754	35.375	1.00	79.44	C
ATOM	6536	CB	ASP	A	831	17.312	-5.012	37.812	1.00	79.44	C
ATOM	6537	O	ASP	A	831	15.977	-5.496	34.844	1.00	79.44	O
ATOM	6538	CG	ASP	A	831	17.969	-5.891	38.844	1.00	79.44	C
ATOM	6539	OD1	ASP	A	831	18.641	-6.871	38.500	1.00	79.44	O
ATOM	6540	OD2	ASP	A	831	17.797	-5.602	40.062	1.00	79.44	O
ATOM	6541	N	HIS	A	832	16.812	-3.398	35.125	1.00	78.06	N
ATOM	6542	CA	HIS	A	832	15.945	-2.789	34.125	1.00	78.06	C
ATOM	6543	C	HIS	A	832	16.219	-3.350	32.719	1.00	78.06	C
ATOM	6544	CB	HIS	A	832	16.125	-1.269	34.125	1.00	78.06	C
ATOM	6545	O	HIS	A	832	15.297	-3.658	31.984	1.00	78.06	O
ATOM	6546	CG	HIS	A	832	15.047	-0.545	33.375	1.00	78.06	C
ATOM	6547	CD2	HIS	A	832	13.930	0.090	33.812	1.00	78.06	C
ATOM	6548	ND1	HIS	A	832	15.055	-0.421	32.000	1.00	78.06	N
ATOM	6549	CE1	HIS	A	832	13.992	0.262	31.609	1.00	78.06	C
ATOM	6550	NE2	HIS	A	832	13.289	0.584	32.688	1.00	78.06	N
ATOM	6551	N	ILE	A	833	17.547	-3.383	32.312	1.00	81.44	N
ATOM	6552	CA	ILE	A	833	17.953	-3.965	31.031	1.00	81.44	C
ATOM	6553	C	ILE	A	833	17.531	-5.430	30.969	1.00	81.44	C
ATOM	6554	CB	ILE	A	833	19.484	-3.844	30.797	1.00	81.44	C
ATOM	6555	O	ILE	A	833	17.078	-5.910	29.922	1.00	81.44	O
ATOM	6556	CG1	ILE	A	833	19.891	-2.371	30.656	1.00	81.44	C
ATOM	6557	CG2	ILE	A	833	19.906	-4.652	29.578	1.00	81.44	C
ATOM	6558	CD1	ILE	A	833	21.391	-2.143	30.625	1.00	81.44	C
ATOM	6559	N	LYS	A	834	17.594	-6.125	32.125	1.00	84.06	N
ATOM	6560	CA	LYS	A	834	17.172	-7.516	32.250	1.00	84.06	C
ATOM	6561	C	LYS	A	834	15.695	-7.664	31.891	1.00	84.06	C
ATOM	6562	CB	LYS	A	834	17.438	-8.055	33.656	1.00	84.06	C
ATOM	6563	O	LYS	A	834	15.312	-8.570	31.141	1.00	84.06	O
ATOM	6564	CG	LYS	A	834	17.047	-9.508	33.844	1.00	84.06	C
ATOM	6565	CD	LYS	A	834	17.391	-10.008	35.219	1.00	84.06	C
ATOM	6566	CE	LYS	A	834	17.031	-11.469	35.406	1.00	84.06	C
ATOM	6567	NZ	LYS	A	834	15.594	-11.641	35.781	1.00	84.06	N
ATOM	6568	N	VAL	A	835	14.773	-6.844	32.469	1.00	83.50	N
ATOM	6569	CA	VAL	A	835	13.336	-6.910	32.219	1.00	83.50	C
ATOM	6570	C	VAL	A	835	13.062	-6.723	30.734	1.00	83.50	C
ATOM	6571	CB	VAL	A	835	12.570	-5.848	33.031	1.00	83.50	C
ATOM	6572	O	VAL	A	835	12.258	-7.449	30.156	1.00	83.50	O
ATOM	6573	CG1	VAL	A	835	11.117	-5.758	32.594	1.00	83.50	C
ATOM	6574	CG2	VAL	A	835	12.641	-6.168	34.531	1.00	83.50	C
ATOM	6575	N	ASP	A	836	13.727	-5.770	30.109	1.00	81.81	N
ATOM	6576	CA	ASP	A	836	13.578	-5.496	28.672	1.00	81.81	C
ATOM	6577	C	ASP	A	836	14.008	-6.699	27.844	1.00	81.81	C
ATOM	6578	CB	ASP	A	836	14.383	-4.258	28.281	1.00	81.81	C
ATOM	6579	O	ASP	A	836	13.320	-7.086	26.891	1.00	81.81	O
ATOM	6580	CG	ASP	A	836	13.672	-2.959	28.609	1.00	81.81	C
ATOM	6581	OD1	ASP	A	836	12.523	-2.998	29.109	1.00	81.81	O
ATOM	6582	OD2	ASP	A	836	14.266	-1.883	28.391	1.00	81.81	O
ATOM	6583	N	ILE	A	837	15.133	-7.316	28.172	1.00	85.94	N
ATOM	6584	CA	ILE	A	837	15.664	-8.492	27.500	1.00	85.94	C
ATOM	6585	C	ILE	A	837	14.711	-9.672	27.688	1.00	85.94	C
ATOM	6586	CB	ILE	A	837	17.078	-8.859	28.000	1.00	85.94	C
ATOM	6587	O	ILE	A	837	14.398	-10.391	26.734	1.00	85.94	O
ATOM	6588	CG1	ILE	A	837	18.094	-7.816	27.531	1.00	85.94	C

ATOM	6589	CG2	ILE	A	837	17.469	-10.266	27.547	1.00	85.94	C
ATOM	6590	CD1	ILE	A	837	19.484	-8.000	28.109	1.00	85.94	C
ATOM	6591	N	GLU	A	838	14.195	-9.820	28.953	1.00	87.00	N
ATOM	6592	CA	GLU	A	838	13.312	-10.938	29.281	1.00	87.00	C
ATOM	6593	C	GLU	A	838	12.000	-10.852	28.500	1.00	87.00	C
ATOM	6594	CB	GLU	A	838	13.023	-10.992	30.781	1.00	87.00	C
ATOM	6595	O	GLU	A	838	11.375	-11.875	28.203	1.00	87.00	O
ATOM	6596	CG	GLU	A	838	14.195	-11.500	31.609	1.00	87.00	C
ATOM	6597	CD	GLU	A	838	13.906	-11.508	33.094	1.00	87.00	C
ATOM	6598	OE1	GLU	A	838	14.680	-12.133	33.875	1.00	87.00	O
ATOM	6599	OE2	GLU	A	838	12.906	-10.875	33.500	1.00	87.00	O
ATOM	6600	N	ASN	A	839	11.547	-9.648	28.109	1.00	83.81	N
ATOM	6601	CA	ASN	A	839	10.297	-9.445	27.375	1.00	83.81	C
ATOM	6602	C	ASN	A	839	10.516	-9.500	25.875	1.00	83.81	C
ATOM	6603	CB	ASN	A	839	9.656	-8.117	27.781	1.00	83.81	C
ATOM	6604	O	ASN	A	839	9.562	-9.422	25.094	1.00	83.81	O
ATOM	6605	CG	ASN	A	839	9.047	-8.156	29.156	1.00	83.81	C
ATOM	6606	ND2	ASN	A	839	8.930	-6.992	29.797	1.00	83.81	N
ATOM	6607	OD1	ASN	A	839	8.672	-9.219	29.656	1.00	83.81	O
ATOM	6608	N	SER	A	840	11.812	-9.664	25.344	1.00	86.81	N
ATOM	6609	CA	SER	A	840	12.125	-9.727	23.906	1.00	86.81	C
ATOM	6610	C	SER	A	840	11.859	-11.125	23.359	1.00	86.81	C
ATOM	6611	CB	SER	A	840	13.586	-9.344	23.672	1.00	86.81	C
ATOM	6612	O	SER	A	840	11.898	-12.109	24.094	1.00	86.81	O
ATOM	6613	OG	SER	A	840	14.461	-10.359	24.125	1.00	86.81	O
ATOM	6614	N	ASP	A	841	11.445	-11.211	22.031	1.00	84.94	N
ATOM	6615	CA	ASP	A	841	11.180	-12.477	21.344	1.00	84.94	C
ATOM	6616	C	ASP	A	841	12.398	-13.391	21.391	1.00	84.94	C
ATOM	6617	CB	ASP	A	841	10.758	-12.219	19.891	1.00	84.94	C
ATOM	6618	O	ASP	A	841	12.266	-14.602	21.578	1.00	84.94	O
ATOM	6619	CG	ASP	A	841	9.367	-11.625	19.766	1.00	84.94	C
ATOM	6620	OD1	ASP	A	841	8.531	-11.852	20.672	1.00	84.94	O
ATOM	6621	OD2	ASP	A	841	9.109	-10.930	18.766	1.00	84.94	O
ATOM	6622	N	GLY	A	842	13.578	-12.773	21.312	1.00	87.38	N
ATOM	6623	CA	GLY	A	842	14.820	-13.539	21.359	1.00	87.38	C
ATOM	6624	C	GLY	A	842	15.031	-14.242	22.672	1.00	87.38	C
ATOM	6625	O	GLY	A	842	15.398	-15.422	22.703	1.00	87.38	O
ATOM	6626	N	TYR	A	843	14.742	-13.531	23.781	1.00	90.06	N
ATOM	6627	CA	TYR	A	843	14.898	-14.117	25.109	1.00	90.06	C
ATOM	6628	C	TYR	A	843	13.883	-15.234	25.344	1.00	90.06	C
ATOM	6629	CB	TYR	A	843	14.742	-13.047	26.188	1.00	90.06	C
ATOM	6630	O	TYR	A	843	14.227	-16.281	25.875	1.00	90.06	O
ATOM	6631	CG	TYR	A	843	14.906	-13.570	27.594	1.00	90.06	C
ATOM	6632	CD1	TYR	A	843	13.797	-13.891	28.375	1.00	90.06	C
ATOM	6633	CD2	TYR	A	843	16.172	-13.750	28.141	1.00	90.06	C
ATOM	6634	CE1	TYR	A	843	13.945	-14.375	29.672	1.00	90.06	C
ATOM	6635	CE2	TYR	A	843	16.328	-14.227	29.438	1.00	90.06	C
ATOM	6636	OH	TYR	A	843	15.367	-15.016	31.469	1.00	90.06	O
ATOM	6637	CZ	TYR	A	843	15.219	-14.539	30.188	1.00	90.06	C
ATOM	6638	N	LYS	A	844	12.617	-14.969	24.984	1.00	88.69	N
ATOM	6639	CA	LYS	A	844	11.555	-15.953	25.141	1.00	88.69	C
ATOM	6640	C	LYS	A	844	11.898	-17.250	24.422	1.00	88.69	C
ATOM	6641	CB	LYS	A	844	10.227	-15.406	24.625	1.00	88.69	C
ATOM	6642	O	LYS	A	844	11.633	-18.344	24.938	1.00	88.69	O
ATOM	6643	CG	LYS	A	844	9.625	-14.312	25.500	1.00	88.69	C
ATOM	6644	CD	LYS	A	844	8.320	-13.789	24.922	1.00	88.69	C
ATOM	6645	CE	LYS	A	844	7.777	-12.617	25.734	1.00	88.69	C
ATOM	6646	NZ	LYS	A	844	6.539	-12.047	25.125	1.00	88.69	N
ATOM	6647	N	TRP	A	845	12.500	-17.094	23.203	1.00	88.88	N
ATOM	6648	CA	TRP	A	845	12.930	-18.266	22.438	1.00	88.88	C
ATOM	6649	C	TRP	A	845	14.055	-19.000	23.141	1.00	88.88	C
ATOM	6650	CB	TRP	A	845	13.383	-17.844	21.031	1.00	88.88	C
ATOM	6651	O	TRP	A	845	14.055	-20.234	23.203	1.00	88.88	O
ATOM	6652	CG	TRP	A	845	13.820	-18.984	20.172	1.00	88.88	C

ATOM	6653	CD1	TRP	A	845	13.023	-19.875	19.516	1.00	88.88	C
ATOM	6654	CD2	TRP	A	845	15.172	-19.344	19.859	1.00	88.88	C
ATOM	6655	CE2	TRP	A	845	15.117	-20.469	19.016	1.00	88.88	C
ATOM	6656	CE3	TRP	A	845	16.422	-18.828	20.219	1.00	88.88	C
ATOM	6657	NE1	TRP	A	845	13.797	-20.781	18.828	1.00	88.88	N
ATOM	6658	CH2	TRP	A	845	17.484	-20.562	18.891	1.00	88.88	C
ATOM	6659	CZ2	TRP	A	845	16.266	-21.094	18.531	1.00	88.88	C
ATOM	6660	CZ3	TRP	A	845	17.562	-19.438	19.734	1.00	88.88	C
ATOM	6661	N	LEU	A	846	15.000	-18.281	23.734	1.00	89.25	N
ATOM	6662	CA	LEU	A	846	16.156	-18.859	24.422	1.00	89.25	C
ATOM	6663	C	LEU	A	846	15.727	-19.547	25.719	1.00	89.25	C
ATOM	6664	CB	LEU	A	846	17.188	-17.781	24.734	1.00	89.25	C
ATOM	6665	O	LEU	A	846	16.391	-20.484	26.172	1.00	89.25	O
ATOM	6666	CG	LEU	A	846	18.203	-17.469	23.625	1.00	89.25	C
ATOM	6667	CD1	LEU	A	846	18.969	-16.188	23.938	1.00	89.25	C
ATOM	6668	CD2	LEU	A	846	19.172	-18.641	23.453	1.00	89.25	C
ATOM	6669	N	GLN	A	847	14.586	-19.172	26.344	1.00	88.44	N
ATOM	6670	CA	GLN	A	847	14.102	-19.688	27.625	1.00	88.44	C
ATOM	6671	C	GLN	A	847	13.602	-21.125	27.469	1.00	88.44	C
ATOM	6672	CB	GLN	A	847	12.984	-18.797	28.172	1.00	88.44	C
ATOM	6673	O	GLN	A	847	13.734	-21.922	28.406	1.00	88.44	O
ATOM	6674	CG	GLN	A	847	12.617	-19.109	29.609	1.00	88.44	C
ATOM	6675	CD	GLN	A	847	11.516	-18.203	30.141	1.00	88.44	C
ATOM	6676	NE2	GLN	A	847	11.422	-18.094	31.469	1.00	88.44	N
ATOM	6677	OE1	GLN	A	847	10.750	-17.609	29.375	1.00	88.44	O
ATOM	6678	N	GLU	A	848	13.047	-21.391	26.312	1.00	86.44	N
ATOM	6679	CA	GLU	A	848	12.484	-22.719	26.062	1.00	86.44	C
ATOM	6680	C	GLU	A	848	13.586	-23.750	25.828	1.00	86.44	C
ATOM	6681	CB	GLU	A	848	11.531	-22.688	24.859	1.00	86.44	C
ATOM	6682	O	GLU	A	848	14.555	-23.484	25.125	1.00	86.44	O
ATOM	6683	CG	GLU	A	848	10.516	-23.812	24.859	1.00	86.44	C
ATOM	6684	CD	GLU	A	848	9.367	-23.578	23.891	1.00	86.44	C
ATOM	6685	OE1	GLU	A	848	8.383	-24.359	23.922	1.00	86.44	O
ATOM	6686	OE2	GLU	A	848	9.453	-22.625	23.094	1.00	86.44	O
ATOM	6687	N	ASN	A	849	13.539	-24.875	26.422	1.00	86.81	N
ATOM	6688	CA	ASN	A	849	14.484	-25.984	26.297	1.00	86.81	C
ATOM	6689	C	ASN	A	849	14.516	-26.547	24.875	1.00	86.81	C
ATOM	6690	CB	ASN	A	849	14.141	-27.094	27.297	1.00	86.81	C
ATOM	6691	O	ASN	A	849	13.477	-26.672	24.234	1.00	86.81	O
ATOM	6692	CG	ASN	A	849	15.094	-28.266	27.219	1.00	86.81	C
ATOM	6693	ND2	ASN	A	849	15.891	-28.469	28.266	1.00	86.81	N
ATOM	6694	OD1	ASN	A	849	15.125	-29.000	26.219	1.00	86.81	O
ATOM	6695	N	ALA	A	850	15.742	-26.656	24.234	1.00	89.88	N
ATOM	6696	CA	ALA	A	850	15.898	-27.125	22.859	1.00	89.88	C
ATOM	6697	C	ALA	A	850	15.172	-28.453	22.641	1.00	89.88	C
ATOM	6698	CB	ALA	A	850	17.375	-27.266	22.516	1.00	89.88	C
ATOM	6699	O	ALA	A	850	14.578	-28.672	21.578	1.00	89.88	O
ATOM	6700	N	ILE	A	851	15.203	-29.391	23.641	1.00	89.25	N
ATOM	6701	CA	ILE	A	851	14.547	-30.688	23.547	1.00	89.25	C
ATOM	6702	C	ILE	A	851	13.031	-30.500	23.438	1.00	89.25	C
ATOM	6703	CB	ILE	A	851	14.891	-31.578	24.750	1.00	89.25	C
ATOM	6704	O	ILE	A	851	12.383	-31.094	22.578	1.00	89.25	O
ATOM	6705	CG1	ILE	A	851	16.359	-31.984	24.719	1.00	89.25	C
ATOM	6706	CG2	ILE	A	851	13.984	-32.812	24.781	1.00	89.25	C
ATOM	6707	CD1	ILE	A	851	16.844	-32.688	25.984	1.00	89.25	C
ATOM	6708	N	LYS	A	852	12.453	-29.672	24.266	1.00	88.81	N
ATOM	6709	CA	LYS	A	852	11.031	-29.344	24.250	1.00	88.81	C
ATOM	6710	C	LYS	A	852	10.641	-28.672	22.938	1.00	88.81	C
ATOM	6711	CB	LYS	A	852	10.672	-28.453	25.422	1.00	88.81	C
ATOM	6712	O	LYS	A	852	9.594	-28.984	22.359	1.00	88.81	O
ATOM	6713	CG	LYS	A	852	10.570	-29.188	26.750	1.00	88.81	C
ATOM	6714	CD	LYS	A	852	10.078	-28.266	27.875	1.00	88.81	C
ATOM	6715	CE	LYS	A	852	10.078	-28.969	29.219	1.00	88.81	C
ATOM	6716	NZ	LYS	A	852	9.625	-28.062	30.312	1.00	88.81	N

ATOM	6717	N	THR	A	853	11.477	-27.719	22.531	1.00	88.88	N
ATOM	6718	CA	THR	A	853	11.227	-27.031	21.281	1.00	88.88	C
ATOM	6719	C	THR	A	853	11.234	-28.016	20.109	1.00	88.88	C
ATOM	6720	CB	THR	A	853	12.273	-25.922	21.031	1.00	88.88	C
ATOM	6721	O	THR	A	853	10.352	-27.953	19.250	1.00	88.88	O
ATOM	6722	CG2	THR	A	853	12.000	-25.188	19.719	1.00	88.88	C
ATOM	6723	OG1	THR	A	853	12.242	-24.984	22.109	1.00	88.88	O
ATOM	6724	N	ASN	A	854	12.211	-28.828	19.969	1.00	90.25	N
ATOM	6725	CA	ASN	A	854	12.281	-29.844	18.938	1.00	90.25	C
ATOM	6726	C	ASN	A	854	11.062	-30.766	18.953	1.00	90.25	C
ATOM	6727	CB	ASN	A	854	13.570	-30.672	19.078	1.00	90.25	C
ATOM	6728	O	ASN	A	854	10.555	-31.156	17.906	1.00	90.25	O
ATOM	6729	CG	ASN	A	854	14.742	-30.031	18.359	1.00	90.25	C
ATOM	6730	ND2	ASN	A	854	15.812	-30.797	18.172	1.00	90.25	N
ATOM	6731	OD1	ASN	A	854	14.695	-28.859	17.969	1.00	90.25	O
ATOM	6732	N	ASP	A	855	10.617	-31.156	20.219	1.00	86.62	N
ATOM	6733	CA	ASP	A	855	9.422	-32.000	20.344	1.00	86.62	C
ATOM	6734	C	ASP	A	855	8.211	-31.312	19.734	1.00	86.62	C
ATOM	6735	CB	ASP	A	855	9.156	-32.312	21.828	1.00	86.62	C
ATOM	6736	O	ASP	A	855	7.414	-31.938	19.031	1.00	86.62	O
ATOM	6737	CG	ASP	A	855	10.117	-33.344	22.391	1.00	86.62	C
ATOM	6738	OD1	ASP	A	855	10.828	-34.000	21.609	1.00	86.62	O
ATOM	6739	OD2	ASP	A	855	10.164	-33.500	23.625	1.00	86.62	O
ATOM	6740	N	LYS	A	856	8.078	-30.016	20.031	1.00	85.81	N
ATOM	6741	CA	LYS	A	856	6.980	-29.219	19.484	1.00	85.81	C
ATOM	6742	C	LYS	A	856	7.070	-29.156	17.953	1.00	85.81	C
ATOM	6743	CB	LYS	A	856	6.984	-27.812	20.062	1.00	85.81	C
ATOM	6744	O	LYS	A	856	6.062	-29.328	17.266	1.00	85.81	O
ATOM	6745	CG	LYS	A	856	6.434	-27.734	21.484	1.00	85.81	C
ATOM	6746	CD	LYS	A	856	6.465	-26.312	22.016	1.00	85.81	C
ATOM	6747	CE	LYS	A	856	6.039	-26.250	23.484	1.00	85.81	C
ATOM	6748	NZ	LYS	A	856	6.113	-24.859	24.016	1.00	85.81	N
ATOM	6749	N	ILE	A	857	8.328	-28.922	17.422	1.00	87.62	N
ATOM	6750	CA	ILE	A	857	8.547	-28.797	15.984	1.00	87.62	C
ATOM	6751	C	ILE	A	857	8.258	-30.141	15.305	1.00	87.62	C
ATOM	6752	CB	ILE	A	857	9.992	-28.344	15.672	1.00	87.62	C
ATOM	6753	O	ILE	A	857	7.539	-30.188	14.305	1.00	87.62	O
ATOM	6754	CG1	ILE	A	857	10.195	-26.891	16.109	1.00	87.62	C
ATOM	6755	CG2	ILE	A	857	10.297	-28.516	14.180	1.00	87.62	C
ATOM	6756	CD1	ILE	A	857	11.641	-26.422	16.047	1.00	87.62	C
ATOM	6757	N	HIS	A	858	8.820	-31.203	15.914	1.00	85.69	N
ATOM	6758	CA	HIS	A	858	8.578	-32.531	15.383	1.00	85.69	C
ATOM	6759	C	HIS	A	858	7.086	-32.875	15.398	1.00	85.69	C
ATOM	6760	CB	HIS	A	858	9.359	-33.594	16.172	1.00	85.69	C
ATOM	6761	O	HIS	A	858	6.551	-33.406	14.422	1.00	85.69	O
ATOM	6762	CG	HIS	A	858	10.844	-33.531	15.953	1.00	85.69	C
ATOM	6763	CD2	HIS	A	858	11.867	-33.438	16.828	1.00	85.69	C
ATOM	6764	ND1	HIS	A	858	11.406	-33.531	14.695	1.00	85.69	N
ATOM	6765	CE1	HIS	A	858	12.719	-33.469	14.812	1.00	85.69	C
ATOM	6766	NE2	HIS	A	858	13.023	-33.406	16.094	1.00	85.69	N
ATOM	6767	N	SER	A	859	6.395	-32.656	16.531	1.00	82.56	N
ATOM	6768	CA	SER	A	859	4.988	-32.969	16.719	1.00	82.56	C
ATOM	6769	C	SER	A	859	4.105	-32.219	15.742	1.00	82.56	C
ATOM	6770	CB	SER	A	859	4.551	-32.688	18.156	1.00	82.56	C
ATOM	6771	O	SER	A	859	3.178	-32.781	15.156	1.00	82.56	O
ATOM	6772	OG	SER	A	859	5.203	-33.562	19.062	1.00	82.56	O
ATOM	6773	N	THR	A	860	4.363	-30.969	15.594	1.00	78.81	N
ATOM	6774	CA	THR	A	860	3.605	-30.125	14.672	1.00	78.81	C
ATOM	6775	C	THR	A	860	3.783	-30.594	13.234	1.00	78.81	C
ATOM	6776	CB	THR	A	860	4.035	-28.641	14.789	1.00	78.81	C
ATOM	6777	O	THR	A	860	2.812	-30.688	12.484	1.00	78.81	O
ATOM	6778	CG2	THR	A	860	3.213	-27.766	13.852	1.00	78.81	C
ATOM	6779	OG1	THR	A	860	3.844	-28.203	16.141	1.00	78.81	O
ATOM	6780	N	ALA	A	861	5.031	-30.812	12.789	1.00	80.75	N

```
ATOM   6781  CA  ALA A 861       5.316 -31.312  11.453  1.00 80.75           C
ATOM   6782  C   ALA A 861       4.613 -32.656  11.203  1.00 80.75           C
ATOM   6783  CB  ALA A 861       6.824 -31.469  11.250  1.00 80.75           C
ATOM   6784  O   ALA A 861       4.023 -32.875  10.148  1.00 80.75           O
ATOM   6785  N   GLU A 862       4.656 -33.500  12.273  1.00 78.31           N
ATOM   6786  CA  GLU A 862       4.039 -34.812  12.141  1.00 78.31           C
ATOM   6787  C   GLU A 862       2.525 -34.719  11.977  1.00 78.31           C
ATOM   6788  CB  GLU A 862       4.371 -35.688  13.359  1.00 78.31           C
ATOM   6789  O   GLU A 862       1.930 -35.406  11.164  1.00 78.31           O
ATOM   6790  CG  GLU A 862       5.742 -36.344  13.289  1.00 78.31           C
ATOM   6791  CD  GLU A 862       6.086 -37.156  14.539  1.00 78.31           C
ATOM   6792  OE1 GLU A 862       7.188 -37.750  14.594  1.00 78.31           O
ATOM   6793  OE2 GLU A 862       5.246 -37.188  15.461  1.00 78.31           O
ATOM   6794  N   SER A 863       1.916 -33.906  12.773  1.00 77.88           N
ATOM   6795  CA  SER A 863       0.473 -33.688  12.703  1.00 77.88           C
ATOM   6796  C   SER A 863       0.059 -33.156  11.344  1.00 77.88           C
ATOM   6797  CB  SER A 863       0.017 -32.750  13.812  1.00 77.88           C
ATOM   6798  O   SER A 863      -0.923 -33.594  10.758  1.00 77.88           O
ATOM   6799  OG  SER A 863       0.515 -31.453  13.594  1.00 77.88           O
ATOM   6800  N   VAL A 864       0.827 -32.125  10.766  1.00 73.00           N
ATOM   6801  CA  VAL A 864       0.530 -31.531   9.469  1.00 73.00           C
ATOM   6802  C   VAL A 864       0.743 -32.562   8.359  1.00 73.00           C
ATOM   6803  CB  VAL A 864       1.401 -30.281   9.219  1.00 73.00           C
ATOM   6804  O   VAL A 864      -0.067 -32.656   7.434  1.00 73.00           O
ATOM   6805  CG1 VAL A 864       1.194 -29.766   7.797  1.00 73.00           C
ATOM   6806  CG2 VAL A 864       1.084 -29.203  10.242  1.00 73.00           C
ATOM   6807  N   ILE A 865       1.842 -33.281   8.461  1.00 77.31           N
ATOM   6808  CA  ILE A 865       2.164 -34.312   7.473  1.00 77.31           C
ATOM   6809  C   ILE A 865       1.070 -35.375   7.457  1.00 77.31           C
ATOM   6810  CB  ILE A 865       3.539 -34.969   7.762  1.00 77.31           C
ATOM   6811  O   ILE A 865       0.598 -35.781   6.391  1.00 77.31           O
ATOM   6812  CG1 ILE A 865       4.668 -33.969   7.465  1.00 77.31           C
ATOM   6813  CG2 ILE A 865       3.711 -36.250   6.945  1.00 77.31           C
ATOM   6814  CD1 ILE A 865       6.043 -34.438   7.926  1.00 77.31           C
ATOM   6815  N   GLU A 866       0.606 -35.844   8.734  1.00 76.19           N
ATOM   6816  CA  GLU A 866      -0.418 -36.875   8.844  1.00 76.19           C
ATOM   6817  C   GLU A 866      -1.743 -36.406   8.250  1.00 76.19           C
ATOM   6818  CB  GLU A 866      -0.613 -37.281  10.312  1.00 76.19           C
ATOM   6819  O   GLU A 866      -2.408 -37.156   7.535  1.00 76.19           O
ATOM   6820  CG  GLU A 866       0.296 -38.406  10.758  1.00 76.19           C
ATOM   6821  CD  GLU A 866       0.035 -38.844  12.188  1.00 76.19           C
ATOM   6822  OE1 GLU A 866       0.755 -39.750  12.688  1.00 76.19           O
ATOM   6823  OE2 GLU A 866      -0.898 -38.312  12.812  1.00 76.19           O
ATOM   6824  N   ASN A 867      -2.109 -35.188   8.539  1.00 72.31           N
ATOM   6825  CA  ASN A 867      -3.336 -34.594   8.000  1.00 72.31           C
ATOM   6826  C   ASN A 867      -3.275 -34.469   6.480  1.00 72.31           C
ATOM   6827  CB  ASN A 867      -3.613 -33.250   8.641  1.00 72.31           C
ATOM   6828  O   ASN A 867      -4.250 -34.750   5.789  1.00 72.31           O
ATOM   6829  CG  ASN A 867      -4.461 -33.344   9.898  1.00 72.31           C
ATOM   6830  ND2 ASN A 867      -4.523 -32.281  10.664  1.00 72.31           N
ATOM   6831  OD1 ASN A 867      -5.051 -34.406  10.172  1.00 72.31           O
ATOM   6832  N   ALA A 868      -2.080 -33.938   5.930  1.00 67.62           N
ATOM   6833  CA  ALA A 868      -1.909 -33.781   4.484  1.00 67.62           C
ATOM   6834  C   ALA A 868      -1.962 -35.156   3.779  1.00 67.62           C
ATOM   6835  CB  ALA A 868      -0.592 -33.094   4.180  1.00 67.62           C
ATOM   6836  O   ALA A 868      -2.555 -35.281   2.705  1.00 67.62           O
ATOM   6837  N   LEU A 869      -1.368 -36.219   4.418  1.00 71.00           N
ATOM   6838  CA  LEU A 869      -1.320 -37.531   3.818  1.00 71.00           C
ATOM   6839  C   LEU A 869      -2.688 -38.219   3.883  1.00 71.00           C
ATOM   6840  CB  LEU A 869      -0.276 -38.406   4.523  1.00 71.00           C
ATOM   6841  O   LEU A 869      -3.066 -38.938   2.965  1.00 71.00           O
ATOM   6842  CG  LEU A 869       1.188 -38.031   4.277  1.00 71.00           C
ATOM   6843  CD1 LEU A 869       2.088 -38.750   5.285  1.00 71.00           C
ATOM   6844  CD2 LEU A 869       1.596 -38.406   2.850  1.00 71.00           C
```

ATOM	6845	N	ALA	A	870	-3.436	-38.062	4.984	1.00	71.81	N
ATOM	6846	CA	ALA	A	870	-4.754	-38.656	5.160	1.00	71.81	C
ATOM	6847	C	ALA	A	870	-5.766	-38.062	4.184	1.00	71.81	C
ATOM	6848	CB	ALA	A	870	-5.234	-38.469	6.598	1.00	71.81	C
ATOM	6849	O	ALA	A	870	-6.637	-38.781	3.680	1.00	71.81	O
ATOM	6850	N	ASN	A	871	-5.613	-36.781	3.805	1.00	65.19	N
ATOM	6851	CA	ASN	A	871	-6.621	-36.062	3.023	1.00	65.19	C
ATOM	6852	C	ASN	A	871	-6.266	-36.031	1.540	1.00	65.19	C
ATOM	6853	CB	ASN	A	871	-6.805	-34.625	3.549	1.00	65.19	C
ATOM	6854	O	ASN	A	871	-7.129	-35.781	0.695	1.00	65.19	O
ATOM	6855	CG	ASN	A	871	-7.469	-34.594	4.910	1.00	65.19	C
ATOM	6856	ND2	ASN	A	871	-7.199	-33.531	5.672	1.00	65.19	N
ATOM	6857	OD1	ASN	A	871	-8.219	-35.500	5.277	1.00	65.19	O
ATOM	6858	N	ASP	A	872	-4.953	-36.375	1.192	1.00	66.88	N
ATOM	6859	CA	ASP	A	872	-4.496	-36.250	-0.188	1.00	66.88	C
ATOM	6860	C	ASP	A	872	-4.637	-37.562	-0.939	1.00	66.88	C
ATOM	6861	CB	ASP	A	872	-3.043	-35.750	-0.233	1.00	66.88	C
ATOM	6862	O	ASP	A	872	-4.055	-38.562	-0.542	1.00	66.88	O
ATOM	6863	CG	ASP	A	872	-2.631	-35.250	-1.600	1.00	66.88	C
ATOM	6864	OD1	ASP	A	872	-3.238	-35.656	-2.613	1.00	66.88	O
ATOM	6865	OD2	ASP	A	872	-1.688	-34.438	-1.662	1.00	66.88	O
ATOM	6866	N	LYS	A	873	-5.543	-37.719	-1.914	1.00	67.62	N
ATOM	6867	CA	LYS	A	873	-5.797	-38.938	-2.699	1.00	67.62	C
ATOM	6868	C	LYS	A	873	-4.582	-39.312	-3.537	1.00	67.62	C
ATOM	6869	CB	LYS	A	873	-7.016	-38.719	-3.602	1.00	67.62	C
ATOM	6870	O	LYS	A	873	-4.441	-40.438	-3.957	1.00	67.62	O
ATOM	6871	CG	LYS	A	873	-8.320	-38.531	-2.846	1.00	67.62	C
ATOM	6872	CD	LYS	A	873	-9.508	-38.406	-3.797	1.00	67.62	C
ATOM	6873	CE	LYS	A	873	-10.812	-38.219	-3.043	1.00	67.62	C
ATOM	6874	NZ	LYS	A	873	-11.977	-38.094	-3.971	1.00	67.62	N
ATOM	6875	N	ASP	A	874	-3.502	-38.375	-3.678	1.00	69.62	N
ATOM	6876	CA	ASP	A	874	-2.361	-38.594	-4.562	1.00	69.62	C
ATOM	6877	C	ASP	A	874	-1.066	-38.750	-3.768	1.00	69.62	C
ATOM	6878	CB	ASP	A	874	-2.236	-37.438	-5.570	1.00	69.62	C
ATOM	6879	O	ASP	A	874	-0.029	-38.188	-4.160	1.00	69.62	O
ATOM	6880	CG	ASP	A	874	-3.357	-37.438	-6.594	1.00	69.62	C
ATOM	6881	OD1	ASP	A	874	-3.914	-38.500	-6.906	1.00	69.62	O
ATOM	6882	OD2	ASP	A	874	-3.686	-36.344	-7.098	1.00	69.62	O
ATOM	6883	N	VAL	A	875	-1.082	-39.281	-2.539	1.00	77.88	N
ATOM	6884	CA	VAL	A	875	0.153	-39.500	-1.793	1.00	77.88	C
ATOM	6885	C	VAL	A	875	1.023	-40.500	-2.518	1.00	77.88	C
ATOM	6886	CB	VAL	A	875	-0.132	-39.969	-0.349	1.00	77.88	C
ATOM	6887	O	VAL	A	875	0.530	-41.531	-2.971	1.00	77.88	O
ATOM	6888	CG1	VAL	A	875	1.171	-40.281	0.381	1.00	77.88	C
ATOM	6889	CG2	VAL	A	875	-0.925	-38.906	0.412	1.00	77.88	C
ATOM	6890	N	ARG	A	876	2.279	-40.156	-2.924	1.00	80.25	N
ATOM	6891	CA	ARG	A	876	3.281	-41.062	-3.461	1.00	80.25	C
ATOM	6892	C	ARG	A	876	4.031	-41.781	-2.340	1.00	80.25	C
ATOM	6893	CB	ARG	A	876	4.270	-40.312	-4.359	1.00	80.25	C
ATOM	6894	O	ARG	A	876	4.676	-41.125	-1.510	1.00	80.25	O
ATOM	6895	CG	ARG	A	876	3.680	-39.875	-5.691	1.00	80.25	C
ATOM	6896	CD	ARG	A	876	4.719	-39.219	-6.574	1.00	80.25	C
ATOM	6897	NE	ARG	A	876	4.121	-38.656	-7.785	1.00	80.25	N
ATOM	6898	NH1	ARG	A	876	6.121	-37.938	-8.695	1.00	80.25	N
ATOM	6899	NH2	ARG	A	876	4.156	-37.594	-9.828	1.00	80.25	N
ATOM	6900	CZ	ARG	A	876	4.801	-38.062	-8.766	1.00	80.25	C
ATOM	6901	N	ARG	A	877	3.701	-43.094	-2.172	1.00	84.06	N
ATOM	6902	CA	ARG	A	877	4.293	-43.938	-1.135	1.00	84.06	C
ATOM	6903	C	ARG	A	877	5.250	-44.969	-1.738	1.00	84.06	C
ATOM	6904	CB	ARG	A	877	3.205	-44.656	-0.330	1.00	84.06	C
ATOM	6905	O	ARG	A	877	4.863	-45.719	-2.621	1.00	84.06	O
ATOM	6906	CG	ARG	A	877	3.719	-45.344	0.912	1.00	84.06	C
ATOM	6907	CD	ARG	A	877	2.604	-46.094	1.653	1.00	84.06	C
ATOM	6908	NE	ARG	A	877	1.826	-45.156	2.482	1.00	84.06	N

ATOM	6909	NH1	ARG	A	877	1.267	-46.750	4.070	1.00	84.06	N
ATOM	6910	NH2	ARG	A	877	0.529	-44.594	4.289	1.00	84.06	N
ATOM	6911	CZ	ARG	A	877	1.209	-45.500	3.611	1.00	84.06	C
ATOM	6912	N	MET	A	878	6.566	-44.875	-1.420	1.00	80.88	N
ATOM	6913	CA	MET	A	878	7.570	-45.875	-1.795	1.00	80.88	C
ATOM	6914	C	MET	A	878	8.070	-46.625	-0.570	1.00	80.88	C
ATOM	6915	CB	MET	A	878	8.742	-45.219	-2.518	1.00	80.88	C
ATOM	6916	O	MET	A	878	8.609	-46.031	0.359	1.00	80.88	O
ATOM	6917	CG	MET	A	878	8.359	-44.562	-3.834	1.00	80.88	C
ATOM	6918	SD	MET	A	878	9.773	-43.719	-4.641	1.00	80.88	S
ATOM	6919	CE	MET	A	878	8.945	-43.000	-6.090	1.00	80.88	C
ATOM	6920	N	ARG	A	879	7.715	-47.938	-0.532	1.00	87.12	N
ATOM	6921	CA	ARG	A	879	8.055	-48.750	0.636	1.00	87.12	C
ATOM	6922	C	ARG	A	879	8.812	-50.000	0.229	1.00	87.12	C
ATOM	6923	CB	ARG	A	879	6.797	-49.125	1.422	1.00	87.12	C
ATOM	6924	O	ARG	A	879	8.469	-50.625	-0.769	1.00	87.12	O
ATOM	6925	CG	ARG	A	879	7.070	-49.938	2.672	1.00	87.12	C
ATOM	6926	CD	ARG	A	879	5.793	-50.250	3.438	1.00	87.12	C
ATOM	6927	NE	ARG	A	879	5.195	-51.500	3.008	1.00	87.12	N
ATOM	6928	NH1	ARG	A	879	3.504	-51.469	4.582	1.00	87.12	N
ATOM	6929	NH2	ARG	A	879	3.660	-53.219	3.092	1.00	87.12	N
ATOM	6930	CZ	ARG	A	879	4.121	-52.062	3.562	1.00	87.12	C
ATOM	6931	N	VAL	A	880	9.938	-50.250	0.914	1.00	75.12	N
ATOM	6932	CA	VAL	A	880	10.672	-51.500	0.803	1.00	75.12	C
ATOM	6933	C	VAL	A	880	10.781	-52.188	2.176	1.00	75.12	C
ATOM	6934	CB	VAL	A	880	12.086	-51.281	0.208	1.00	75.12	C
ATOM	6935	O	VAL	A	880	11.164	-51.531	3.154	1.00	75.12	O
ATOM	6936	CG1	VAL	A	880	12.859	-52.594	0.157	1.00	75.12	C
ATOM	6937	CG2	VAL	A	880	11.984	-50.656	-1.185	1.00	75.12	C
ATOM	6938	N	GLU	A	881	10.227	-53.344	2.234	1.00	83.19	N
ATOM	6939	CA	GLU	A	881	10.250	-54.125	3.479	1.00	83.19	C
ATOM	6940	C	GLU	A	881	11.039	-55.406	3.318	1.00	83.19	C
ATOM	6941	CB	GLU	A	881	8.820	-54.406	3.941	1.00	83.19	C
ATOM	6942	O	GLU	A	881	10.867	-56.125	2.330	1.00	83.19	O
ATOM	6943	CG	GLU	A	881	8.742	-55.000	5.348	1.00	83.19	C
ATOM	6944	CD	GLU	A	881	7.312	-55.188	5.840	1.00	83.19	C
ATOM	6945	OE1	GLU	A	881	7.117	-55.781	6.922	1.00	83.19	O
ATOM	6946	OE2	GLU	A	881	6.383	-54.719	5.137	1.00	83.19	O
ATOM	6947	N	ASN	A	882	12.109	-55.625	4.156	1.00	71.44	N
ATOM	6948	CA	ASN	A	882	12.844	-56.875	4.312	1.00	71.44	C
ATOM	6949	C	ASN	A	882	12.844	-57.344	5.766	1.00	71.44	C
ATOM	6950	CB	ASN	A	882	14.281	-56.719	3.807	1.00	71.44	C
ATOM	6951	O	ASN	A	882	13.680	-56.938	6.562	1.00	71.44	O
ATOM	6952	CG	ASN	A	882	15.023	-58.062	3.734	1.00	71.44	C
ATOM	6953	ND2	ASN	A	882	16.328	-58.000	3.535	1.00	71.44	N
ATOM	6954	OD1	ASN	A	882	14.422	-59.125	3.855	1.00	71.44	O
ATOM	6955	N	GLY	A	883	11.875	-58.281	6.184	1.00	78.06	N
ATOM	6956	CA	GLY	A	883	11.773	-58.719	7.570	1.00	78.06	C
ATOM	6957	C	GLY	A	883	11.328	-57.594	8.508	1.00	78.06	C
ATOM	6958	O	GLY	A	883	10.250	-57.031	8.344	1.00	78.06	O
ATOM	6959	N	LYS	A	884	12.141	-57.281	9.539	1.00	73.94	N
ATOM	6960	CA	LYS	A	884	11.906	-56.281	10.578	1.00	73.94	C
ATOM	6961	C	LYS	A	884	12.469	-54.938	10.172	1.00	73.94	C
ATOM	6962	CB	LYS	A	884	12.531	-56.750	11.906	1.00	73.94	C
ATOM	6963	O	LYS	A	884	12.469	-54.000	10.969	1.00	73.94	O
ATOM	6964	CG	LYS	A	884	11.883	-57.969	12.500	1.00	73.94	C
ATOM	6965	CD	LYS	A	884	12.484	-58.312	13.859	1.00	73.94	C
ATOM	6966	CE	LYS	A	884	11.836	-59.562	14.461	1.00	73.94	C
ATOM	6967	NZ	LYS	A	884	12.445	-59.938	15.773	1.00	73.94	N
ATOM	6968	N	ARG	A	885	12.992	-54.812	8.844	1.00	74.44	N
ATOM	6969	CA	ARG	A	885	13.586	-53.594	8.258	1.00	74.44	C
ATOM	6970	C	ARG	A	885	12.617	-52.938	7.293	1.00	74.44	C
ATOM	6971	CB	ARG	A	885	14.891	-53.938	7.539	1.00	74.44	C
ATOM	6972	O	ARG	A	885	12.102	-53.562	6.371	1.00	74.44	O

ATOM	6973	CG	ARG	A	885	15.898	-54.688	8.406	1.00	74.44	C
ATOM	6974	CD	ARG	A	885	17.031	-55.250	7.578	1.00	74.44	C
ATOM	6975	NE	ARG	A	885	17.891	-56.125	8.375	1.00	74.44	N
ATOM	6976	NH1	ARG	A	885	19.234	-56.781	6.613	1.00	74.44	N
ATOM	6977	NH2	ARG	A	885	19.625	-57.594	8.711	1.00	74.44	N
ATOM	6978	CZ	ARG	A	885	18.906	-56.844	7.898	1.00	74.44	C
ATOM	6979	N	LYS	A	886	12.320	-51.625	7.648	1.00	81.69	N
ATOM	6980	CA	LYS	A	886	11.398	-50.875	6.816	1.00	81.69	C
ATOM	6981	C	LYS	A	886	12.023	-49.562	6.375	1.00	81.69	C
ATOM	6982	CB	LYS	A	886	10.094	-50.625	7.566	1.00	81.69	C
ATOM	6983	O	LYS	A	886	12.648	-48.844	7.180	1.00	81.69	O
ATOM	6984	CG	LYS	A	886	9.289	-51.875	7.875	1.00	81.69	C
ATOM	6985	CD	LYS	A	886	8.016	-51.531	8.641	1.00	81.69	C
ATOM	6986	CE	LYS	A	886	7.285	-52.812	9.070	1.00	81.69	C
ATOM	6987	NZ	LYS	A	886	6.066	-52.500	9.875	1.00	81.69	N
ATOM	6988	N	ALA	A	887	12.117	-49.344	4.984	1.00	74.31	N
ATOM	6989	CA	ALA	A	887	12.438	-48.031	4.434	1.00	74.31	C
ATOM	6990	C	ALA	A	887	11.227	-47.438	3.713	1.00	74.31	C
ATOM	6991	CB	ALA	A	887	13.625	-48.125	3.488	1.00	74.31	C
ATOM	6992	O	ALA	A	887	10.609	-48.094	2.879	1.00	74.31	O
ATOM	6993	N	GLU	A	888	10.859	-46.281	4.090	1.00	86.56	N
ATOM	6994	CA	GLU	A	888	9.641	-45.656	3.541	1.00	86.56	C
ATOM	6995	C	GLU	A	888	9.883	-44.219	3.117	1.00	86.56	C
ATOM	6996	CB	GLU	A	888	8.500	-45.750	4.562	1.00	86.56	C
ATOM	6997	O	GLU	A	888	10.562	-43.469	3.816	1.00	86.56	O
ATOM	6998	CG	GLU	A	888	7.148	-45.344	3.998	1.00	86.56	C
ATOM	6999	CD	GLU	A	888	6.016	-45.469	5.004	1.00	86.56	C
ATOM	7000	OE1	GLU	A	888	4.926	-45.938	4.629	1.00	86.56	O
ATOM	7001	OE2	GLU	A	888	6.223	-45.062	6.176	1.00	86.56	O
ATOM	7002	N	PHE	A	889	9.477	-43.844	1.875	1.00	82.62	N
ATOM	7003	CA	PHE	A	889	9.453	-42.469	1.353	1.00	82.62	C
ATOM	7004	C	PHE	A	889	8.023	-42.031	1.059	1.00	82.62	C
ATOM	7005	CB	PHE	A	889	10.305	-42.375	0.085	1.00	82.62	C
ATOM	7006	O	PHE	A	889	7.297	-42.719	0.328	1.00	82.62	O
ATOM	7007	CG	PHE	A	889	10.273	-41.000	-0.553	1.00	82.62	C
ATOM	7008	CD1	PHE	A	889	9.586	-40.812	-1.739	1.00	82.62	C
ATOM	7009	CD2	PHE	A	889	10.945	-39.938	0.033	1.00	82.62	C
ATOM	7010	CE1	PHE	A	889	9.555	-39.531	-2.332	1.00	82.62	C
ATOM	7011	CE2	PHE	A	889	10.922	-38.688	-0.553	1.00	82.62	C
ATOM	7012	CZ	PHE	A	889	10.227	-38.469	-1.736	1.00	82.62	C
ATOM	7013	N	LEU	A	890	7.578	-40.875	1.705	1.00	83.38	N
ATOM	7014	CA	LEU	A	890	6.242	-40.312	1.525	1.00	83.38	C
ATOM	7015	C	LEU	A	890	6.316	-38.875	0.976	1.00	83.38	C
ATOM	7016	CB	LEU	A	890	5.473	-40.312	2.850	1.00	83.38	C
ATOM	7017	O	LEU	A	890	7.137	-38.094	1.428	1.00	83.38	O
ATOM	7018	CG	LEU	A	890	5.230	-41.688	3.482	1.00	83.38	C
ATOM	7019	CD1	LEU	A	890	4.805	-41.531	4.941	1.00	83.38	C
ATOM	7020	CD2	LEU	A	890	4.176	-42.469	2.695	1.00	83.38	C
ATOM	7021	N	GLN	A	891	5.574	-38.656	-0.145	1.00	83.81	N
ATOM	7022	CA	GLN	A	891	5.477	-37.312	-0.741	1.00	83.81	C
ATOM	7023	C	GLN	A	891	4.023	-36.938	-1.014	1.00	83.81	C
ATOM	7024	CB	GLN	A	891	6.289	-37.250	-2.035	1.00	83.81	C
ATOM	7025	O	GLN	A	891	3.268	-37.750	-1.581	1.00	83.81	O
ATOM	7026	CG	GLN	A	891	6.301	-35.875	-2.678	1.00	83.81	C
ATOM	7027	CD	GLN	A	891	7.102	-35.812	-3.965	1.00	83.81	C
ATOM	7028	NE2	GLN	A	891	7.641	-34.656	-4.293	1.00	83.81	N
ATOM	7029	OE1	GLN	A	891	7.238	-36.844	-4.660	1.00	83.81	O
ATOM	7030	N	SER	A	892	3.650	-35.719	-0.476	1.00	82.69	N
ATOM	7031	CA	SER	A	892	2.297	-35.219	-0.707	1.00	82.69	C
ATOM	7032	C	SER	A	892	2.305	-33.750	-1.040	1.00	82.69	C
ATOM	7033	CB	SER	A	892	1.417	-35.469	0.519	1.00	82.69	C
ATOM	7034	O	SER	A	892	3.139	-33.000	-0.532	1.00	82.69	O
ATOM	7035	OG	SER	A	892	0.132	-34.906	0.341	1.00	82.69	O
ATOM	7036	N	LEU	A	893	1.541	-33.312	-2.031	1.00	77.69	N

ATOM	7037	CA	LEU	A	893	1.278	-31.906	-2.398	1.00	77.69	C
ATOM	7038	C	LEU	A	893	-0.208	-31.594	-2.289	1.00	77.69	C
ATOM	7039	CB	LEU	A	893	1.769	-31.641	-3.822	1.00	77.69	C
ATOM	7040	O	LEU	A	893	-1.034	-32.219	-2.955	1.00	77.69	O
ATOM	7041	CG	LEU	A	893	1.667	-30.188	-4.301	1.00	77.69	C
ATOM	7042	CD1	LEU	A	893	2.658	-29.312	-3.545	1.00	77.69	C
ATOM	7043	CD2	LEU	A	893	1.909	-30.094	-5.805	1.00	77.69	C
ATOM	7044	N	LYS	A	894	-0.514	-30.734	-1.315	1.00	75.75	N
ATOM	7045	CA	LYS	A	894	-1.896	-30.312	-1.110	1.00	75.75	C
ATOM	7046	C	LYS	A	894	-2.084	-28.844	-1.469	1.00	75.75	C
ATOM	7047	CB	LYS	A	894	-2.322	-30.562	0.338	1.00	75.75	C
ATOM	7048	O	LYS	A	894	-1.358	-27.984	-0.970	1.00	75.75	O
ATOM	7049	CG	LYS	A	894	-3.797	-30.297	0.604	1.00	75.75	C
ATOM	7050	CD	LYS	A	894	-4.188	-30.672	2.027	1.00	75.75	C
ATOM	7051	CE	LYS	A	894	-5.668	-30.422	2.287	1.00	75.75	C
ATOM	7052	NZ	LYS	A	894	-6.055	-30.797	3.682	1.00	75.75	N
ATOM	7053	N	LEU	A	895	-2.969	-28.547	-2.461	1.00	72.31	N
ATOM	7054	CA	LEU	A	895	-3.316	-27.203	-2.908	1.00	72.31	C
ATOM	7055	C	LEU	A	895	-4.750	-26.859	-2.523	1.00	72.31	C
ATOM	7056	CB	LEU	A	895	-3.139	-27.078	-4.422	1.00	72.31	C
ATOM	7057	O	LEU	A	895	-5.688	-27.578	-2.898	1.00	72.31	O
ATOM	7058	CG	LEU	A	895	-1.744	-27.391	-4.969	1.00	72.31	C
ATOM	7059	CD1	LEU	A	895	-1.812	-27.672	-6.465	1.00	72.31	C
ATOM	7060	CD2	LEU	A	895	-0.786	-26.250	-4.680	1.00	72.31	C
ATOM	7061	N	ILE	A	896	-4.879	-25.969	-1.532	1.00	68.19	N
ATOM	7062	CA	ILE	A	896	-6.195	-25.500	-1.109	1.00	68.19	C
ATOM	7063	C	ILE	A	896	-6.410	-24.062	-1.562	1.00	68.19	C
ATOM	7064	CB	ILE	A	896	-6.367	-25.609	0.423	1.00	68.19	C
ATOM	7065	O	ILE	A	896	-5.590	-23.188	-1.277	1.00	68.19	O
ATOM	7066	CG1	ILE	A	896	-6.203	-27.078	0.875	1.00	68.19	C
ATOM	7067	CG2	ILE	A	896	-7.730	-25.062	0.854	1.00	68.19	C
ATOM	7068	CD1	ILE	A	896	-6.199	-27.250	2.387	1.00	68.19	C
ATOM	7069	N	ALA	A	897	-7.406	-23.922	-2.469	1.00	64.56	N
ATOM	7070	CA	ALA	A	897	-7.844	-22.594	-2.867	1.00	64.56	C
ATOM	7071	C	ALA	A	897	-9.352	-22.438	-2.709	1.00	64.56	C
ATOM	7072	CB	ALA	A	897	-7.434	-22.312	-4.309	1.00	64.56	C
ATOM	7073	O	ALA	A	897	-10.125	-22.953	-3.531	1.00	64.56	O
ATOM	7074	N	ASP	A	898	-9.672	-21.953	-1.450	1.00	65.19	N
ATOM	7075	CA	ASP	A	898	-11.102	-21.734	-1.225	1.00	65.19	C
ATOM	7076	C	ASP	A	898	-11.391	-20.266	-0.932	1.00	65.19	C
ATOM	7077	CB	ASP	A	898	-11.602	-22.609	-0.073	1.00	65.19	C
ATOM	7078	O	ASP	A	898	-10.547	-19.391	-1.182	1.00	65.19	O
ATOM	7079	CG	ASP	A	898	-10.922	-22.297	1.248	1.00	65.19	C
ATOM	7080	OD1	ASP	A	898	-10.484	-21.141	1.452	1.00	65.19	O
ATOM	7081	OD2	ASP	A	898	-10.820	-23.203	2.092	1.00	65.19	O
ATOM	7082	N	GLU	A	899	-12.742	-19.906	-0.543	1.00	54.44	N
ATOM	7083	CA	GLU	A	899	-13.164	-18.516	-0.372	1.00	54.44	C
ATOM	7084	C	GLU	A	899	-12.344	-17.812	0.709	1.00	54.44	C
ATOM	7085	CB	GLU	A	899	-14.656	-18.438	-0.029	1.00	54.44	C
ATOM	7086	O	GLU	A	899	-12.227	-16.594	0.708	1.00	54.44	O
ATOM	7087	CG	GLU	A	899	-15.570	-18.859	-1.171	1.00	54.44	C
ATOM	7088	CD	GLU	A	899	-17.047	-18.766	-0.822	1.00	54.44	C
ATOM	7089	OE1	GLU	A	899	-17.891	-19.047	-1.698	1.00	54.44	O
ATOM	7090	OE2	GLU	A	899	-17.359	-18.406	0.336	1.00	54.44	O
ATOM	7091	N	THR	A	900	-11.547	-18.594	1.598	1.00	53.81	N
ATOM	7092	CA	THR	A	900	-10.945	-18.000	2.789	1.00	53.81	C
ATOM	7093	C	THR	A	900	-9.422	-18.016	2.691	1.00	53.81	C
ATOM	7094	CB	THR	A	900	-11.391	-18.750	4.062	1.00	53.81	C
ATOM	7095	O	THR	A	900	-8.750	-17.188	3.309	1.00	53.81	O
ATOM	7096	CG2	THR	A	900	-12.867	-18.500	4.359	1.00	53.81	C
ATOM	7097	OG1	THR	A	900	-11.172	-20.156	3.891	1.00	53.81	O
ATOM	7098	N	GLU	A	901	-8.844	-18.938	1.756	1.00	61.19	N
ATOM	7099	CA	GLU	A	901	-7.391	-19.094	1.803	1.00	61.19	C
ATOM	7100	C	GLU	A	901	-6.867	-19.781	0.544	1.00	61.19	C

ATOM	7101	CB	GLU	A	901	-6.973	-19.891	3.045	1.00	61.19	C
ATOM	7102	O	GLU	A	901	-7.625	-20.438	-0.172	1.00	61.19	O
ATOM	7103	CG	GLU	A	901	-7.375	-21.359	3.002	1.00	61.19	C
ATOM	7104	CD	GLU	A	901	-6.902	-22.141	4.215	1.00	61.19	C
ATOM	7105	OE1	GLU	A	901	-7.098	-23.375	4.246	1.00	61.19	O
ATOM	7106	OE2	GLU	A	901	-6.336	-21.516	5.137	1.00	61.19	O
ATOM	7107	N	ALA	A	902	-5.703	-19.359	0.152	1.00	59.56	N
ATOM	7108	CA	ALA	A	902	-4.859	-20.094	-0.788	1.00	59.56	C
ATOM	7109	C	ALA	A	902	-3.650	-20.703	-0.081	1.00	59.56	C
ATOM	7110	CB	ALA	A	902	-4.406	-19.188	-1.923	1.00	59.56	C
ATOM	7111	O	ALA	A	902	-2.838	-19.984	0.501	1.00	59.56	O
ATOM	7112	N	ARG	A	903	-3.719	-22.016	0.066	1.00	75.75	N
ATOM	7113	CA	ARG	A	903	-2.705	-22.719	0.852	1.00	75.75	C
ATOM	7114	C	ARG	A	903	-2.053	-23.828	0.042	1.00	75.75	C
ATOM	7115	CB	ARG	A	903	-3.314	-23.281	2.135	1.00	75.75	C
ATOM	7116	O	ARG	A	903	-2.738	-24.562	-0.668	1.00	75.75	O
ATOM	7117	CG	ARG	A	903	-2.324	-24.047	3.004	1.00	75.75	C
ATOM	7118	CD	ARG	A	903	-3.010	-24.719	4.184	1.00	75.75	C
ATOM	7119	NE	ARG	A	903	-3.703	-23.750	5.027	1.00	75.75	N
ATOM	7120	NH1	ARG	A	903	-1.917	-23.391	6.449	1.00	75.75	N
ATOM	7121	NH2	ARG	A	903	-3.891	-22.281	6.785	1.00	75.75	N
ATOM	7122	CZ	ARG	A	903	-3.170	-23.141	6.086	1.00	75.75	C
ATOM	7123	N	VAL	A	904	-0.707	-23.859	-0.010	1.00	73.06	N
ATOM	7124	CA	VAL	A	904	0.080	-24.953	-0.571	1.00	73.06	C
ATOM	7125	C	VAL	A	904	0.870	-25.641	0.538	1.00	73.06	C
ATOM	7126	CB	VAL	A	904	1.037	-24.453	-1.677	1.00	73.06	C
ATOM	7127	O	VAL	A	904	1.594	-24.984	1.293	1.00	73.06	O
ATOM	7128	CG1	VAL	A	904	1.849	-25.609	-2.252	1.00	73.06	C
ATOM	7129	CG2	VAL	A	904	0.253	-23.750	-2.781	1.00	73.06	C
ATOM	7130	N	THR	A	905	0.567	-26.891	0.704	1.00	78.62	N
ATOM	7131	CA	THR	A	905	1.323	-27.688	1.665	1.00	78.62	C
ATOM	7132	C	THR	A	905	2.078	-28.812	0.960	1.00	78.62	C
ATOM	7133	CB	THR	A	905	0.402	-28.281	2.746	1.00	78.62	C
ATOM	7134	O	THR	A	905	1.482	-29.609	0.223	1.00	78.62	O
ATOM	7135	CG2	THR	A	905	1.210	-28.984	3.830	1.00	78.62	C
ATOM	7136	OG1	THR	A	905	-0.365	-27.234	3.344	1.00	78.62	O
ATOM	7137	N	GLN	A	906	3.389	-28.844	0.973	1.00	79.06	N
ATOM	7138	CA	GLN	A	906	4.242	-29.906	0.460	1.00	79.06	C
ATOM	7139	C	GLN	A	906	4.918	-30.672	1.599	1.00	79.06	C
ATOM	7140	CB	GLN	A	906	5.297	-29.344	-0.491	1.00	79.06	C
ATOM	7141	O	GLN	A	906	5.516	-30.062	2.488	1.00	79.06	O
ATOM	7142	CG	GLN	A	906	6.176	-30.406	-1.139	1.00	79.06	C
ATOM	7143	CD	GLN	A	906	7.227	-29.812	-2.061	1.00	79.06	C
ATOM	7144	NE2	GLN	A	906	7.938	-30.672	-2.779	1.00	79.06	N
ATOM	7145	OE1	GLN	A	906	7.398	-28.594	-2.129	1.00	79.06	O
ATOM	7146	N	VAL	A	907	4.762	-31.969	1.537	1.00	81.69	N
ATOM	7147	CA	VAL	A	907	5.324	-32.812	2.588	1.00	81.69	C
ATOM	7148	C	VAL	A	907	6.195	-33.906	1.969	1.00	81.69	C
ATOM	7149	CB	VAL	A	907	4.219	-33.438	3.463	1.00	81.69	C
ATOM	7150	O	VAL	A	907	5.809	-34.531	0.978	1.00	81.69	O
ATOM	7151	CG1	VAL	A	907	4.824	-34.375	4.508	1.00	81.69	C
ATOM	7152	CG2	VAL	A	907	3.383	-32.344	4.133	1.00	81.69	C
ATOM	7153	N	THR	A	908	7.477	-34.062	2.463	1.00	82.75	N
ATOM	7154	CA	THR	A	908	8.359	-35.156	2.168	1.00	82.75	C
ATOM	7155	C	THR	A	908	8.844	-35.844	3.455	1.00	82.75	C
ATOM	7156	CB	THR	A	908	9.578	-34.719	1.333	1.00	82.75	C
ATOM	7157	O	THR	A	908	9.172	-35.156	4.426	1.00	82.75	O
ATOM	7158	CG2	THR	A	908	9.148	-34.281	-0.062	1.00	82.75	C
ATOM	7159	OG1	THR	A	908	10.227	-33.625	1.994	1.00	82.75	O
ATOM	7160	N	GLN	A	909	8.625	-37.125	3.553	1.00	84.12	N
ATOM	7161	CA	GLN	A	909	9.070	-37.875	4.730	1.00	84.12	C
ATOM	7162	C	GLN	A	909	9.844	-39.125	4.328	1.00	84.12	C
ATOM	7163	CB	GLN	A	909	7.879	-38.250	5.609	1.00	84.12	C
ATOM	7164	O	GLN	A	909	9.406	-39.875	3.465	1.00	84.12	O

ATOM	7165	CG	GLN	A	909	8.266	-38.938	6.914	1.00	84.12	C
ATOM	7166	CD	GLN	A	909	7.070	-39.312	7.762	1.00	84.12	C
ATOM	7167	NE2	GLN	A	909	7.305	-39.562	9.039	1.00	84.12	N
ATOM	7168	OE1	GLN	A	909	5.938	-39.344	7.273	1.00	84.12	O
ATOM	7169	N	MET	A	910	11.094	-39.312	4.848	1.00	79.56	N
ATOM	7170	CA	MET	A	910	11.914	-40.500	4.707	1.00	79.56	C
ATOM	7171	C	MET	A	910	12.117	-41.188	6.055	1.00	79.56	C
ATOM	7172	CB	MET	A	910	13.273	-40.156	4.094	1.00	79.56	C
ATOM	7173	O	MET	A	910	12.398	-40.531	7.055	1.00	79.56	O
ATOM	7174	CG	MET	A	910	13.180	-39.531	2.713	1.00	79.56	C
ATOM	7175	SD	MET	A	910	14.820	-39.094	2.021	1.00	79.56	S
ATOM	7176	CE	MET	A	910	15.172	-37.562	2.961	1.00	79.56	C
ATOM	7177	N	SER	A	911	11.750	-42.344	6.070	1.00	83.56	N
ATOM	7178	CA	SER	A	911	11.930	-43.062	7.320	1.00	83.56	C
ATOM	7179	C	SER	A	911	12.586	-44.438	7.082	1.00	83.56	C
ATOM	7180	CB	SER	A	911	10.594	-43.250	8.039	1.00	83.56	C
ATOM	7181	O	SER	A	911	12.414	-45.031	6.020	1.00	83.56	O
ATOM	7182	OG	SER	A	911	9.734	-44.094	7.277	1.00	83.56	O
ATOM	7183	N	ALA	A	912	13.461	-44.844	7.926	1.00	73.06	N
ATOM	7184	CA	ALA	A	912	14.117	-46.156	7.988	1.00	73.06	C
ATOM	7185	C	ALA	A	912	14.039	-46.750	9.391	1.00	73.06	C
ATOM	7186	CB	ALA	A	912	15.578	-46.031	7.547	1.00	73.06	C
ATOM	7187	O	ALA	A	912	14.305	-46.062	10.375	1.00	73.06	O
ATOM	7188	N	GLN	A	913	13.484	-47.875	9.469	1.00	78.88	N
ATOM	7189	CA	GLN	A	913	13.266	-48.500	10.773	1.00	78.88	C
ATOM	7190	C	GLN	A	913	13.805	-49.906	10.820	1.00	78.88	C
ATOM	7191	CB	GLN	A	913	11.773	-48.469	11.133	1.00	78.88	C
ATOM	7192	O	GLN	A	913	13.797	-50.625	9.805	1.00	78.88	O
ATOM	7193	CG	GLN	A	913	10.922	-49.344	10.219	1.00	78.88	C
ATOM	7194	CD	GLN	A	913	9.445	-49.312	10.578	1.00	78.88	C
ATOM	7195	NE2	GLN	A	913	8.625	-50.000	9.797	1.00	78.88	N
ATOM	7196	OE1	GLN	A	913	9.047	-48.625	11.539	1.00	78.88	O
ATOM	7197	N	PHE	A	914	14.312	-50.438	12.000	1.00	62.88	N
ATOM	7198	CA	PHE	A	914	14.711	-51.781	12.344	1.00	62.88	C
ATOM	7199	C	PHE	A	914	13.977	-52.250	13.594	1.00	62.88	C
ATOM	7200	CB	PHE	A	914	16.219	-51.875	12.555	1.00	62.88	C
ATOM	7201	O	PHE	A	914	14.164	-51.719	14.680	1.00	62.88	O
ATOM	7202	CG	PHE	A	914	16.781	-53.250	12.430	1.00	62.88	C
ATOM	7203	CD1	PHE	A	914	16.859	-54.094	13.539	1.00	62.88	C
ATOM	7204	CD2	PHE	A	914	17.219	-53.750	11.203	1.00	62.88	C
ATOM	7205	CE1	PHE	A	914	17.359	-55.375	13.422	1.00	62.88	C
ATOM	7206	CE2	PHE	A	914	17.734	-55.031	11.086	1.00	62.88	C
ATOM	7207	CZ	PHE	A	914	17.797	-55.844	12.195	1.00	62.88	C
ATOM	7208	N	ASP	A	915	13.047	-53.094	13.477	1.00	71.69	N
ATOM	7209	CA	ASP	A	915	12.391	-53.781	14.578	1.00	71.69	C
ATOM	7210	C	ASP	A	915	11.602	-52.844	15.453	1.00	71.69	C
ATOM	7211	CB	ASP	A	915	13.422	-54.562	15.414	1.00	71.69	C
ATOM	7212	O	ASP	A	915	11.547	-53.000	16.672	1.00	71.69	O
ATOM	7213	CG	ASP	A	915	12.805	-55.656	16.250	1.00	71.69	C
ATOM	7214	OD1	ASP	A	915	11.664	-56.094	15.961	1.00	71.69	O
ATOM	7215	OD2	ASP	A	915	13.469	-56.125	17.188	1.00	71.69	O
ATOM	7216	N	GLU	A	916	11.219	-51.625	14.797	1.00	61.56	N
ATOM	7217	CA	GLU	A	916	10.430	-50.625	15.523	1.00	61.56	C
ATOM	7218	C	GLU	A	916	11.219	-50.031	16.688	1.00	61.56	C
ATOM	7219	CB	GLU	A	916	9.125	-51.250	16.031	1.00	61.56	C
ATOM	7220	O	GLU	A	916	10.711	-49.188	17.406	1.00	61.56	O
ATOM	7221	CG	GLU	A	916	8.172	-51.688	14.922	1.00	61.56	C
ATOM	7222	CD	GLU	A	916	6.887	-52.281	15.445	1.00	61.56	C
ATOM	7223	OE1	GLU	A	916	6.035	-52.719	14.625	1.00	61.56	O
ATOM	7224	OE2	GLU	A	916	6.730	-52.375	16.688	1.00	61.56	O
ATOM	7225	N	LYS	A	917	12.469	-50.469	16.844	1.00	55.53	N
ATOM	7226	CA	LYS	A	917	13.312	-50.031	17.953	1.00	55.53	C
ATOM	7227	C	LYS	A	917	14.297	-48.938	17.484	1.00	55.53	C
ATOM	7228	CB	LYS	A	917	14.070	-51.188	18.547	1.00	55.53	C

ATOM	7229	O	LYS	A	917	14.641	-48.031	18.266	1.00	55.53	O
ATOM	7230	CG	LYS	A	917	13.195	-52.188	19.312	1.00	55.53	C
ATOM	7231	CD	LYS	A	917	14.023	-53.219	20.047	1.00	55.53	C
ATOM	7232	CE	LYS	A	917	13.148	-54.219	20.781	1.00	55.53	C
ATOM	7233	NZ	LYS	A	917	13.953	-55.219	21.531	1.00	55.53	N
ATOM	7234	N	LEU	A	918	14.844	-49.062	16.203	1.00	56.72	N
ATOM	7235	CA	LEU	A	918	15.773	-48.156	15.578	1.00	56.72	C
ATOM	7236	C	LEU	A	918	15.094	-47.375	14.453	1.00	56.72	C
ATOM	7237	CB	LEU	A	918	17.000	-48.906	15.039	1.00	56.72	C
ATOM	7238	O	LEU	A	918	14.703	-47.969	13.438	1.00	56.72	O
ATOM	7239	CG	LEU	A	918	18.234	-48.062	14.758	1.00	56.72	C
ATOM	7240	CD1	LEU	A	918	18.891	-47.625	16.078	1.00	56.72	C
ATOM	7241	CD2	LEU	A	918	19.219	-48.812	13.883	1.00	56.72	C
ATOM	7242	N	THR	A	919	14.820	-46.031	14.734	1.00	75.44	N
ATOM	7243	CA	THR	A	919	14.055	-45.219	13.797	1.00	75.44	C
ATOM	7244	C	THR	A	919	14.828	-43.969	13.406	1.00	75.44	C
ATOM	7245	CB	THR	A	919	12.688	-44.844	14.375	1.00	75.44	C
ATOM	7246	O	THR	A	919	15.461	-43.344	14.258	1.00	75.44	O
ATOM	7247	CG2	THR	A	919	11.781	-44.219	13.305	1.00	75.44	C
ATOM	7248	OG1	THR	A	919	12.047	-46.000	14.914	1.00	75.44	O
ATOM	7249	N	ALA	A	920	15.039	-43.844	12.070	1.00	70.44	N
ATOM	7250	CA	ALA	A	920	15.516	-42.594	11.508	1.00	70.44	C
ATOM	7251	C	ALA	A	920	14.445	-41.938	10.633	1.00	70.44	C
ATOM	7252	CB	ALA	A	920	16.797	-42.812	10.711	1.00	70.44	C
ATOM	7253	O	ALA	A	920	13.820	-42.625	9.812	1.00	70.44	O
ATOM	7254	N	GLN	A	921	14.258	-40.719	10.930	1.00	82.69	N
ATOM	7255	CA	GLN	A	921	13.203	-40.031	10.195	1.00	82.69	C
ATOM	7256	C	GLN	A	921	13.648	-38.656	9.758	1.00	82.69	C
ATOM	7257	CB	GLN	A	921	11.938	-39.938	11.047	1.00	82.69	C
ATOM	7258	O	GLN	A	921	14.352	-37.938	10.500	1.00	82.69	O
ATOM	7259	CG	GLN	A	921	10.719	-39.438	10.289	1.00	82.69	C
ATOM	7260	CD	GLN	A	921	9.453	-39.438	11.133	1.00	82.69	C
ATOM	7261	NE2	GLN	A	921	8.570	-38.469	10.875	1.00	82.69	N
ATOM	7262	OE1	GLN	A	921	9.273	-40.281	12.000	1.00	82.69	O
ATOM	7263	N	ASN	A	922	13.453	-38.312	8.500	1.00	76.56	N
ATOM	7264	CA	ASN	A	922	13.641	-36.969	7.941	1.00	76.56	C
ATOM	7265	C	ASN	A	922	12.344	-36.438	7.348	1.00	76.56	C
ATOM	7266	CB	ASN	A	922	14.742	-37.000	6.879	1.00	76.56	C
ATOM	7267	O	ASN	A	922	11.773	-37.031	6.430	1.00	76.56	O
ATOM	7268	CG	ASN	A	922	15.133	-35.594	6.434	1.00	76.56	C
ATOM	7269	ND2	ASN	A	922	16.094	-35.500	5.512	1.00	76.56	N
ATOM	7270	OD1	ASN	A	922	14.586	-34.594	6.914	1.00	76.56	O
ATOM	7271	N	SER	A	923	11.875	-35.344	7.961	1.00	83.12	N
ATOM	7272	CA	SER	A	923	10.625	-34.750	7.492	1.00	83.12	C
ATOM	7273	C	SER	A	923	10.828	-33.312	7.031	1.00	83.12	C
ATOM	7274	CB	SER	A	923	9.562	-34.781	8.594	1.00	83.12	C
ATOM	7275	O	SER	A	923	11.617	-32.562	7.625	1.00	83.12	O
ATOM	7276	OG	SER	A	923	9.328	-36.125	9.008	1.00	83.12	O
ATOM	7277	N	GLU	A	924	10.359	-32.969	5.855	1.00	81.00	N
ATOM	7278	CA	GLU	A	924	10.320	-31.625	5.332	1.00	81.00	C
ATOM	7279	C	GLU	A	924	8.883	-31.172	5.090	1.00	81.00	C
ATOM	7280	CB	GLU	A	924	11.125	-31.516	4.039	1.00	81.00	C
ATOM	7281	O	GLU	A	924	8.109	-31.859	4.414	1.00	81.00	O
ATOM	7282	CG	GLU	A	924	11.312	-30.078	3.545	1.00	81.00	C
ATOM	7283	CD	GLU	A	924	12.164	-29.984	2.289	1.00	81.00	C
ATOM	7284	OE1	GLU	A	924	12.469	-28.859	1.846	1.00	81.00	O
ATOM	7285	OE2	GLU	A	924	12.531	-31.062	1.743	1.00	81.00	O
ATOM	7286	N	LEU	A	925	8.461	-30.062	5.742	1.00	81.94	N
ATOM	7287	CA	LEU	A	925	7.152	-29.438	5.566	1.00	81.94	C
ATOM	7288	C	LEU	A	925	7.297	-28.031	5.008	1.00	81.94	C
ATOM	7289	CB	LEU	A	925	6.391	-29.406	6.891	1.00	81.94	C
ATOM	7290	O	LEU	A	925	8.008	-27.203	5.582	1.00	81.94	O
ATOM	7291	CG	LEU	A	925	5.055	-28.656	6.887	1.00	81.94	C
ATOM	7292	CD1	LEU	A	925	4.012	-29.453	6.105	1.00	81.94	C

ATOM	7293	CD2	LEU	A	925	4.582	-28.391	8.312	1.00	81.94	C
ATOM	7294	N	ARG	A	926	6.812	-27.797	3.754	1.00	76.50	N
ATOM	7295	CA	ARG	A	926	6.750	-26.484	3.129	1.00	76.50	C
ATOM	7296	C	ARG	A	926	5.305	-26.016	2.988	1.00	76.50	C
ATOM	7297	CB	ARG	A	926	7.430	-26.500	1.759	1.00	76.50	C
ATOM	7298	O	ARG	A	926	4.465	-26.734	2.439	1.00	76.50	O
ATOM	7299	CG	ARG	A	926	8.930	-26.734	1.821	1.00	76.50	C
ATOM	7300	CD	ARG	A	926	9.562	-26.703	0.436	1.00	76.50	C
ATOM	7301	NE	ARG	A	926	10.992	-26.984	0.492	1.00	76.50	N
ATOM	7302	NH1	ARG	A	926	11.617	-25.031	-0.593	1.00	76.50	N
ATOM	7303	NH2	ARG	A	926	13.211	-26.516	0.108	1.00	76.50	N
ATOM	7304	CZ	ARG	A	926	11.938	-26.172	0.002	1.00	76.50	C
ATOM	7305	N	GLU	A	927	5.000	-24.859	3.654	1.00	75.19	N
ATOM	7306	CA	GLU	A	927	3.635	-24.344	3.643	1.00	75.19	C
ATOM	7307	C	GLU	A	927	3.600	-22.875	3.238	1.00	75.19	C
ATOM	7308	CB	GLU	A	927	2.980	-24.531	5.016	1.00	75.19	C
ATOM	7309	O	GLU	A	927	4.387	-22.062	3.734	1.00	75.19	O
ATOM	7310	CG	GLU	A	927	1.477	-24.297	5.016	1.00	75.19	C
ATOM	7311	CD	GLU	A	927	0.830	-24.562	6.367	1.00	75.19	C
ATOM	7312	OE1	GLU	A	927	-0.396	-24.812	6.414	1.00	75.19	O
ATOM	7313	OE2	GLU	A	927	1.554	-24.516	7.387	1.00	75.19	O
ATOM	7314	N	VAL	A	928	2.703	-22.547	2.219	1.00	62.22	N
ATOM	7315	CA	VAL	A	928	2.455	-21.188	1.785	1.00	62.22	C
ATOM	7316	C	VAL	A	928	0.976	-20.844	1.953	1.00	62.22	C
ATOM	7317	CB	VAL	A	928	2.889	-20.969	0.318	1.00	62.22	C
ATOM	7318	O	VAL	A	928	0.109	-21.562	1.443	1.00	62.22	O
ATOM	7319	CG1	VAL	A	928	2.615	-19.516	-0.117	1.00	62.22	C
ATOM	7320	CG2	VAL	A	928	4.367	-21.297	0.142	1.00	62.22	C
ATOM	7321	N	ILE	A	929	0.653	-19.891	2.773	1.00	69.25	N
ATOM	7322	CA	ILE	A	929	-0.737	-19.562	3.062	1.00	69.25	C
ATOM	7323	C	ILE	A	929	-1.008	-18.109	2.691	1.00	69.25	C
ATOM	7324	CB	ILE	A	929	-1.080	-19.812	4.551	1.00	69.25	C
ATOM	7325	O	ILE	A	929	-0.269	-17.203	3.102	1.00	69.25	O
ATOM	7326	CG1	ILE	A	929	-0.799	-21.266	4.926	1.00	69.25	C
ATOM	7327	CG2	ILE	A	929	-2.539	-19.438	4.836	1.00	69.25	C
ATOM	7328	CD1	ILE	A	929	-0.866	-21.547	6.422	1.00	69.25	C
ATOM	7329	N	ALA	A	930	-2.031	-17.844	1.834	1.00	58.56	N
ATOM	7330	CA	ALA	A	930	-2.576	-16.531	1.501	1.00	58.56	C
ATOM	7331	C	ALA	A	930	-4.027	-16.406	1.959	1.00	58.56	C
ATOM	7332	CB	ALA	A	930	-2.471	-16.266	-0.000	1.00	58.56	C
ATOM	7333	O	ALA	A	930	-4.895	-17.156	1.503	1.00	58.56	O
ATOM	7334	N	ASN	A	931	-4.309	-15.789	3.104	1.00	54.88	N
ATOM	7335	CA	ASN	A	931	-5.594	-15.789	3.797	1.00	54.88	C
ATOM	7336	C	ASN	A	931	-6.164	-14.375	3.920	1.00	54.88	C
ATOM	7337	CB	ASN	A	931	-5.461	-16.438	5.180	1.00	54.88	C
ATOM	7338	O	ASN	A	931	-5.414	-13.422	4.129	1.00	54.88	O
ATOM	7339	CG	ASN	A	931	-5.418	-17.938	5.121	1.00	54.88	C
ATOM	7340	ND2	ASN	A	931	-5.223	-18.578	6.270	1.00	54.88	N
ATOM	7341	OD1	ASN	A	931	-5.559	-18.547	4.047	1.00	54.88	O
ATOM	7342	N	SER	A	932	-7.508	-14.164	3.641	1.00	51.38	N
ATOM	7343	CA	SER	A	932	-8.289	-12.953	3.865	1.00	51.38	C
ATOM	7344	C	SER	A	932	-9.047	-13.023	5.184	1.00	51.38	C
ATOM	7345	CB	SER	A	932	-9.266	-12.727	2.713	1.00	51.38	C
ATOM	7346	O	SER	A	932	-9.516	-12.000	5.695	1.00	51.38	O
ATOM	7347	OG	SER	A	932	-10.586	-12.562	3.197	1.00	51.38	O
ATOM	7348	N	THR	A	933	-8.414	-13.203	6.461	1.00	44.28	N
ATOM	7349	CA	THR	A	933	-8.953	-13.023	7.805	1.00	44.28	C
ATOM	7350	C	THR	A	933	-8.344	-14.023	8.781	1.00	44.28	C
ATOM	7351	CB	THR	A	933	-10.492	-13.164	7.812	1.00	44.28	C
ATOM	7352	O	THR	A	933	-8.234	-15.211	8.469	1.00	44.28	O
ATOM	7353	CG2	THR	A	933	-11.094	-12.523	9.055	1.00	44.28	C
ATOM	7354	OG1	THR	A	933	-11.031	-12.523	6.648	1.00	44.28	O
ATOM	7355	N	GLU	A	934	-7.453	-13.648	9.781	1.00	41.91	N
ATOM	7356	CA	GLU	A	934	-7.023	-13.914	11.156	1.00	41.91	C

ATOM	7357	C	GLU	A	934	-7.066	-15.414	11.461	1.00	41.91	C
ATOM	7358	CB	GLU	A	934	-7.898	-13.148	12.148	1.00	41.91	C
ATOM	7359	O	GLU	A	934	-8.039	-16.094	11.141	1.00	41.91	O
ATOM	7360	CG	GLU	A	934	-7.109	-12.281	13.117	1.00	41.91	C
ATOM	7361	CD	GLU	A	934	-7.973	-11.266	13.844	1.00	41.91	C
ATOM	7362	OE1	GLU	A	934	-7.410	-10.359	14.516	1.00	41.91	O
ATOM	7363	OE2	GLU	A	934	-9.211	-11.367	13.750	1.00	41.91	O
ATOM	7364	N	THR	A	935	-6.000	-16.125	12.086	1.00	38.19	N
ATOM	7365	CA	THR	A	935	-5.387	-16.656	13.305	1.00	38.19	C
ATOM	7366	C	THR	A	935	-5.492	-18.172	13.344	1.00	38.19	C
ATOM	7367	CB	THR	A	935	-6.039	-16.062	14.562	1.00	38.19	C
ATOM	7368	O	THR	A	935	-6.590	-18.719	13.430	1.00	38.19	O
ATOM	7369	CG2	THR	A	935	-4.992	-15.742	15.625	1.00	38.19	C
ATOM	7370	OG1	THR	A	935	-6.727	-14.852	14.219	1.00	38.19	O
ATOM	7371	N	ILE	A	936	-4.340	-18.984	13.156	1.00	42.75	N
ATOM	7372	CA	ILE	A	936	-4.191	-20.328	13.711	1.00	42.75	C
ATOM	7373	C	ILE	A	936	-2.908	-20.406	14.531	1.00	42.75	C
ATOM	7374	CB	ILE	A	936	-4.188	-21.406	12.594	1.00	42.75	C
ATOM	7375	O	ILE	A	936	-2.598	-21.453	15.109	1.00	42.75	O
ATOM	7376	CG1	ILE	A	936	-5.570	-21.500	11.938	1.00	42.75	C
ATOM	7377	CG2	ILE	A	936	-3.748	-22.766	13.156	1.00	42.75	C
ATOM	7378	CD1	ILE	A	936	-5.617	-22.422	10.727	1.00	42.75	C
ATOM	7379	N	SER	A	937	-2.510	-19.469	15.422	1.00	45.00	N
ATOM	7380	CA	SER	A	937	-1.400	-19.688	16.344	1.00	45.00	C
ATOM	7381	C	SER	A	937	-1.840	-19.500	17.797	1.00	45.00	C
ATOM	7382	CB	SER	A	937	-0.243	-18.734	16.031	1.00	45.00	C
ATOM	7383	O	SER	A	937	-1.361	-20.203	18.688	1.00	45.00	O
ATOM	7384	OG	SER	A	937	0.857	-19.453	15.484	1.00	45.00	O
ATOM	7385	N	GLN	A	938	-3.201	-19.281	18.125	1.00	46.66	N
ATOM	7386	CA	GLN	A	938	-3.564	-19.125	19.531	1.00	46.66	C
ATOM	7387	C	GLN	A	938	-4.484	-20.250	20.000	1.00	46.66	C
ATOM	7388	CB	GLN	A	938	-4.238	-17.766	19.750	1.00	46.66	C
ATOM	7389	O	GLN	A	938	-4.414	-20.672	21.156	1.00	46.66	O
ATOM	7390	CG	GLN	A	938	-3.260	-16.594	19.859	1.00	46.66	C
ATOM	7391	CD	GLN	A	938	-3.951	-15.266	20.062	1.00	46.66	C
ATOM	7392	NE2	GLN	A	938	-3.189	-14.258	20.469	1.00	46.66	N
ATOM	7393	OE1	GLN	A	938	-5.160	-15.141	19.844	1.00	46.66	O
ATOM	7394	N	ARG	A	939	-4.781	-21.266	19.109	1.00	48.00	N
ATOM	7395	CA	ARG	A	939	-5.645	-22.344	19.594	1.00	48.00	C
ATOM	7396	C	ARG	A	939	-4.883	-23.672	19.672	1.00	48.00	C
ATOM	7397	CB	ARG	A	939	-6.863	-22.500	18.688	1.00	48.00	C
ATOM	7398	O	ARG	A	939	-5.137	-24.484	20.562	1.00	48.00	O
ATOM	7399	CG	ARG	A	939	-8.102	-21.781	19.188	1.00	48.00	C
ATOM	7400	CD	ARG	A	939	-9.297	-21.984	18.266	1.00	48.00	C
ATOM	7401	NE	ARG	A	939	-10.438	-21.172	18.656	1.00	48.00	N
ATOM	7402	NH1	ARG	A	939	-11.930	-22.188	17.219	1.00	48.00	N
ATOM	7403	NH2	ARG	A	939	-12.633	-20.500	18.594	1.00	48.00	N
ATOM	7404	CZ	ARG	A	939	-11.664	-21.297	18.156	1.00	48.00	C
ATOM	7405	N	ILE	A	940	-3.459	-23.766	19.328	1.00	49.91	N
ATOM	7406	CA	ILE	A	940	-2.703	-25.000	19.297	1.00	49.91	C
ATOM	7407	C	ILE	A	940	-1.803	-25.094	20.531	1.00	49.91	C
ATOM	7408	CB	ILE	A	940	-1.859	-25.125	18.000	1.00	49.91	C
ATOM	7409	O	ILE	A	940	-1.646	-26.156	21.109	1.00	49.91	O
ATOM	7410	CG1	ILE	A	940	-2.758	-25.453	16.812	1.00	49.91	C
ATOM	7411	CG2	ILE	A	940	-0.763	-26.188	18.172	1.00	49.91	C
ATOM	7412	CD1	ILE	A	940	-2.051	-25.375	15.461	1.00	49.91	C
ATOM	7413	N	ASN	A	941	-1.558	-23.922	21.219	1.00	51.06	N
ATOM	7414	CA	ASN	A	941	-0.754	-24.000	22.438	1.00	51.06	C
ATOM	7415	C	ASN	A	941	-1.608	-24.359	23.641	1.00	51.06	C
ATOM	7416	CB	ASN	A	941	-0.012	-22.688	22.672	1.00	51.06	C
ATOM	7417	O	ASN	A	941	-1.150	-25.078	24.547	1.00	51.06	O
ATOM	7418	CG	ASN	A	941	1.161	-22.500	21.734	1.00	51.06	C
ATOM	7419	ND2	ASN	A	941	1.609	-21.266	21.594	1.00	51.06	N
ATOM	7420	OD1	ASN	A	941	1.661	-23.469	21.141	1.00	51.06	O

ATOM	7421	N	GLN	A	942	-2.951	-24.281	23.531	1.00	48.09	N
ATOM	7422	CA	GLN	A	942	-3.801	-24.625	24.656	1.00	48.09	C
ATOM	7423	C	GLN	A	942	-4.266	-26.078	24.578	1.00	48.09	C
ATOM	7424	CB	GLN	A	942	-5.008	-23.688	24.734	1.00	48.09	C
ATOM	7425	O	GLN	A	942	-4.367	-26.766	25.594	1.00	48.09	O
ATOM	7426	CG	GLN	A	942	-5.059	-22.844	26.000	1.00	48.09	C
ATOM	7427	CD	GLN	A	942	-6.133	-21.781	25.953	1.00	48.09	C
ATOM	7428	NE2	GLN	A	942	-6.234	-20.984	27.016	1.00	48.09	N
ATOM	7429	OE1	GLN	A	942	-6.867	-21.656	24.969	1.00	48.09	O
ATOM	7430	N	LEU	A	943	-4.141	-26.734	23.344	1.00	47.75	N
ATOM	7431	CA	LEU	A	943	-4.566	-28.125	23.141	1.00	47.75	C
ATOM	7432	C	LEU	A	943	-3.408	-29.078	23.375	1.00	47.75	C
ATOM	7433	CB	LEU	A	943	-5.145	-28.312	21.750	1.00	47.75	C
ATOM	7434	O	LEU	A	943	-3.600	-30.172	23.906	1.00	47.75	O
ATOM	7435	CG	LEU	A	943	-6.141	-29.469	21.578	1.00	47.75	C
ATOM	7436	CD1	LEU	A	943	-7.469	-29.125	22.234	1.00	47.75	C
ATOM	7437	CD2	LEU	A	943	-6.336	-29.781	20.094	1.00	47.75	C
ATOM	7438	N	THR	A	944	-2.100	-28.594	23.312	1.00	52.88	N
ATOM	7439	CA	THR	A	944	-0.889	-29.375	23.531	1.00	52.88	C
ATOM	7440	C	THR	A	944	-0.587	-29.516	25.016	1.00	52.88	C
ATOM	7441	CB	THR	A	944	0.320	-28.734	22.812	1.00	52.88	C
ATOM	7442	O	THR	A	944	-0.163	-30.594	25.469	1.00	52.88	O
ATOM	7443	CG2	THR	A	944	1.556	-29.625	22.938	1.00	52.88	C
ATOM	7444	OG1	THR	A	944	0.008	-28.547	21.422	1.00	52.88	O
ATOM	7445	N	ALA	A	945	-1.145	-28.609	25.906	1.00	47.75	N
ATOM	7446	CA	ALA	A	945	-0.901	-28.766	27.344	1.00	47.75	C
ATOM	7447	C	ALA	A	945	-1.880	-29.766	27.953	1.00	47.75	C
ATOM	7448	CB	ALA	A	945	-1.002	-27.422	28.047	1.00	47.75	C
ATOM	7449	O	ALA	A	945	-1.532	-30.484	28.891	1.00	47.75	O
ATOM	7450	N	THR	A	946	-2.928	-30.250	27.234	1.00	42.47	N
ATOM	7451	CA	THR	A	946	-3.885	-31.188	27.812	1.00	42.47	C
ATOM	7452	C	THR	A	946	-3.557	-32.625	27.391	1.00	42.47	C
ATOM	7453	CB	THR	A	946	-5.324	-30.844	27.391	1.00	42.47	C
ATOM	7454	O	THR	A	946	-3.688	-33.531	28.203	1.00	42.47	O
ATOM	7455	CG2	THR	A	946	-6.332	-31.312	28.422	1.00	42.47	C
ATOM	7456	OG1	THR	A	946	-5.449	-29.422	27.219	1.00	42.47	O
ATOM	7457	N	PHE	A	947	-2.600	-32.906	26.391	1.00	43.53	N
ATOM	7458	CA	PHE	A	947	-2.410	-34.281	25.922	1.00	43.53	C
ATOM	7459	C	PHE	A	947	-1.059	-34.844	26.375	1.00	43.53	C
ATOM	7460	CB	PHE	A	947	-2.523	-34.375	24.406	1.00	43.53	C
ATOM	7461	O	PHE	A	947	-0.814	-36.031	26.312	1.00	43.53	O
ATOM	7462	CG	PHE	A	947	-3.910	-34.688	23.906	1.00	43.53	C
ATOM	7463	CD1	PHE	A	947	-4.543	-35.875	24.297	1.00	43.53	C
ATOM	7464	CD2	PHE	A	947	-4.578	-33.812	23.062	1.00	43.53	C
ATOM	7465	CE1	PHE	A	947	-5.828	-36.156	23.844	1.00	43.53	C
ATOM	7466	CE2	PHE	A	947	-5.863	-34.094	22.609	1.00	43.53	C
ATOM	7467	CZ	PHE	A	947	-6.484	-35.281	23.000	1.00	43.53	C
ATOM	7468	N	GLU	A	948	-0.249	-34.031	27.266	1.00	49.47	N
ATOM	7469	CA	GLU	A	948	1.062	-34.562	27.609	1.00	49.47	C
ATOM	7470	C	GLU	A	948	1.017	-35.312	28.938	1.00	49.47	C
ATOM	7471	CB	GLU	A	948	2.104	-33.438	27.672	1.00	49.47	C
ATOM	7472	O	GLU	A	948	1.917	-36.125	29.234	1.00	49.47	O
ATOM	7473	CG	GLU	A	948	2.891	-33.250	26.391	1.00	49.47	C
ATOM	7474	CD	GLU	A	948	3.859	-32.094	26.438	1.00	49.47	C
ATOM	7475	OE1	GLU	A	948	4.570	-31.844	25.438	1.00	49.47	O
ATOM	7476	OE2	GLU	A	948	3.908	-31.422	27.500	1.00	49.47	O
ATOM	7477	N	SER	A	949	-0.176	-35.781	29.438	1.00	42.66	N
ATOM	7478	CA	SER	A	949	-0.086	-36.625	30.609	1.00	42.66	C
ATOM	7479	C	SER	A	949	-0.767	-37.969	30.375	1.00	42.66	C
ATOM	7480	CB	SER	A	949	-0.711	-35.938	31.828	1.00	42.66	C
ATOM	7481	O	SER	A	949	-0.528	-38.938	31.094	1.00	42.66	O
ATOM	7482	OG	SER	A	949	-1.474	-36.875	32.594	1.00	42.66	O
ATOM	7483	N	GLU	A	950	-0.473	-38.625	29.156	1.00	36.03	N
ATOM	7484	CA	GLU	A	950	-0.872	-40.031	29.203	1.00	36.03	C

ATOM	7485	C	GLU	A	950	-0.310	-40.812	28.000	1.00	36.03	C
ATOM	7486	CB	GLU	A	950	-2.396	-40.156	29.234	1.00	36.03	C
ATOM	7487	O	GLU	A	950	-0.984	-41.688	27.438	1.00	36.03	O
ATOM	7488	CG	GLU	A	950	-2.973	-40.281	30.641	1.00	36.03	C
ATOM	7489	CD	GLU	A	950	-4.480	-40.469	30.656	1.00	36.03	C
ATOM	7490	OE1	GLU	A	950	-5.059	-40.625	31.750	1.00	36.03	O
ATOM	7491	OE2	GLU	A	950	-5.086	-40.500	29.562	1.00	36.03	O
ATOM	7492	N	ILE	A	951	1.132	-41.062	27.844	1.00	43.84	N
ATOM	7493	CA	ILE	A	951	1.587	-42.281	27.141	1.00	43.84	C
ATOM	7494	C	ILE	A	951	2.963	-42.688	27.672	1.00	43.84	C
ATOM	7495	CB	ILE	A	951	1.635	-42.062	25.609	1.00	43.84	C
ATOM	7496	O	ILE	A	951	3.916	-41.906	27.594	1.00	43.84	O
ATOM	7497	CG1	ILE	A	951	0.216	-42.000	25.031	1.00	43.84	C
ATOM	7498	CG2	ILE	A	951	2.455	-43.156	24.938	1.00	43.84	C
ATOM	7499	CD1	ILE	A	951	0.161	-41.625	23.562	1.00	43.84	C
ATOM	7500	N	ASP	A	952	3.139	-43.469	28.781	1.00	37.50	N
ATOM	7501	CA	ASP	A	952	3.383	-44.844	29.203	1.00	37.50	C
ATOM	7502	C	ASP	A	952	4.738	-45.344	28.703	1.00	37.50	C
ATOM	7503	CB	ASP	A	952	2.270	-45.781	28.703	1.00	37.50	C
ATOM	7504	O	ASP	A	952	4.996	-45.344	27.484	1.00	37.50	O
ATOM	7505	CG	ASP	A	952	1.303	-46.188	29.797	1.00	37.50	C
ATOM	7506	OD1	ASP	A	952	1.490	-45.781	30.953	1.00	37.50	O
ATOM	7507	OD2	ASP	A	952	0.346	-46.938	29.484	1.00	37.50	O
ATOM	7508	N	GLY	A	953	5.895	-45.688	29.516	1.00	35.59	N
ATOM	7509	CA	GLY	A	953	6.414	-46.938	30.047	1.00	35.59	C
ATOM	7510	C	GLY	A	953	7.176	-47.750	29.031	1.00	35.59	C
ATOM	7511	O	GLY	A	953	6.715	-47.938	27.906	1.00	35.59	O
ATOM	7512	N	VAL	A	954	8.633	-47.719	28.906	1.00	35.59	N
ATOM	7513	CA	VAL	A	954	9.898	-48.438	28.781	1.00	35.59	C
ATOM	7514	C	VAL	A	954	10.062	-48.969	27.359	1.00	35.59	C
ATOM	7515	CB	VAL	A	954	9.969	-49.625	29.781	1.00	35.59	C
ATOM	7516	O	VAL	A	954	9.414	-49.938	26.953	1.00	35.59	O
ATOM	7517	CG1	VAL	A	954	11.320	-50.344	29.688	1.00	35.59	C
ATOM	7518	CG2	VAL	A	954	9.719	-49.125	31.203	1.00	35.59	C
ATOM	7519	N	LYS	A	955	10.328	-48.156	26.266	1.00	42.44	N
ATOM	7520	CA	LYS	A	955	11.070	-48.625	25.109	1.00	42.44	C
ATOM	7521	C	LYS	A	955	12.375	-47.844	24.938	1.00	42.44	C
ATOM	7522	CB	LYS	A	955	10.219	-48.500	23.844	1.00	42.44	C
ATOM	7523	O	LYS	A	955	12.406	-46.625	25.141	1.00	42.44	O
ATOM	7524	CG	LYS	A	955	9.398	-49.719	23.531	1.00	42.44	C
ATOM	7525	CD	LYS	A	955	8.656	-49.594	22.203	1.00	42.44	C
ATOM	7526	CE	LYS	A	955	7.773	-50.812	21.922	1.00	42.44	C
ATOM	7527	NZ	LYS	A	955	7.051	-50.656	20.625	1.00	42.44	N
ATOM	7528	N	GLN	A	956	13.555	-48.406	25.312	1.00	49.16	N
ATOM	7529	CA	GLN	A	956	14.891	-47.969	24.922	1.00	49.16	C
ATOM	7530	C	GLN	A	956	14.945	-47.562	23.453	1.00	49.16	C
ATOM	7531	CB	GLN	A	956	15.930	-49.031	25.219	1.00	49.16	C
ATOM	7532	O	GLN	A	956	14.984	-48.438	22.578	1.00	49.16	O
ATOM	7533	CG	GLN	A	956	16.547	-48.938	26.609	1.00	49.16	C
ATOM	7534	CD	GLN	A	956	17.766	-49.812	26.781	1.00	49.16	C
ATOM	7535	NE2	GLN	A	956	18.344	-49.812	27.969	1.00	49.16	N
ATOM	7536	OE1	GLN	A	956	18.203	-50.500	25.828	1.00	49.16	O
ATOM	7537	N	ASP	A	957	14.133	-46.625	22.969	1.00	61.66	N
ATOM	7538	CA	ASP	A	957	14.125	-46.250	21.547	1.00	61.66	C
ATOM	7539	C	ASP	A	957	15.297	-45.344	21.203	1.00	61.66	C
ATOM	7540	CB	ASP	A	957	12.805	-45.594	21.172	1.00	61.66	C
ATOM	7541	O	ASP	A	957	15.719	-44.531	22.031	1.00	61.66	O
ATOM	7542	CG	ASP	A	957	11.633	-46.531	21.109	1.00	61.66	C
ATOM	7543	OD1	ASP	A	957	11.859	-47.781	20.969	1.00	61.66	O
ATOM	7544	OD2	ASP	A	957	10.477	-46.094	21.172	1.00	61.66	O
ATOM	7545	N	ILE	A	958	16.188	-45.844	20.344	1.00	67.00	N
ATOM	7546	CA	ILE	A	958	17.219	-45.094	19.625	1.00	67.00	C
ATOM	7547	C	ILE	A	958	16.578	-44.344	18.453	1.00	67.00	C
ATOM	7548	CB	ILE	A	958	18.359	-46.000	19.125	1.00	67.00	C

ATOM	7549	O	ILE	A	958	16.047	-44.969	17.531	1.00	67.00	O
ATOM	7550	CG1	ILE	A	958	19.016	-46.750	20.297	1.00	67.00	C
ATOM	7551	CG2	ILE	A	958	19.391	-45.188	18.344	1.00	67.00	C
ATOM	7552	CD1	ILE	A	958	20.047	-47.781	19.891	1.00	67.00	C
ATOM	7553	N	LYS	A	959	16.406	-42.969	18.562	1.00	77.94	N
ATOM	7554	CA	LYS	A	959	15.719	-42.125	17.594	1.00	77.94	C
ATOM	7555	C	LYS	A	959	16.641	-41.031	17.062	1.00	77.94	C
ATOM	7556	CB	LYS	A	959	14.469	-41.500	18.203	1.00	77.94	C
ATOM	7557	O	LYS	A	959	17.438	-40.469	17.828	1.00	77.94	O
ATOM	7558	CG	LYS	A	959	13.383	-42.531	18.547	1.00	77.94	C
ATOM	7559	CD	LYS	A	959	12.156	-41.844	19.156	1.00	77.94	C
ATOM	7560	CE	LYS	A	959	11.102	-42.844	19.562	1.00	77.94	C
ATOM	7561	NZ	LYS	A	959	9.914	-42.188	20.188	1.00	77.94	N
ATOM	7562	N	ALA	A	960	16.781	-40.969	15.773	1.00	75.69	N
ATOM	7563	CA	ALA	A	960	17.359	-39.812	15.102	1.00	75.69	C
ATOM	7564	C	ALA	A	960	16.344	-39.125	14.180	1.00	75.69	C
ATOM	7565	CB	ALA	A	960	18.609	-40.219	14.305	1.00	75.69	C
ATOM	7566	O	ALA	A	960	15.711	-39.812	13.359	1.00	75.69	O
ATOM	7567	N	GLN	A	961	16.125	-37.812	14.453	1.00	84.69	N
ATOM	7568	CA	GLN	A	961	15.094	-37.062	13.703	1.00	84.69	C
ATOM	7569	C	GLN	A	961	15.625	-35.750	13.203	1.00	84.69	C
ATOM	7570	CB	GLN	A	961	13.852	-36.844	14.570	1.00	84.69	C
ATOM	7571	O	GLN	A	961	16.344	-35.031	13.914	1.00	84.69	O
ATOM	7572	CG	GLN	A	961	13.117	-38.156	14.922	1.00	84.69	C
ATOM	7573	CD	GLN	A	961	11.875	-37.906	15.766	1.00	84.69	C
ATOM	7574	NE2	GLN	A	961	10.883	-38.750	15.625	1.00	84.69	N
ATOM	7575	OE1	GLN	A	961	11.820	-36.938	16.547	1.00	84.69	O
ATOM	7576	N	ILE	A	962	15.430	-35.500	12.000	1.00	85.38	N
ATOM	7577	CA	ILE	A	962	15.641	-34.188	11.391	1.00	85.38	C
ATOM	7578	C	ILE	A	962	14.312	-33.656	10.844	1.00	85.38	C
ATOM	7579	CB	ILE	A	962	16.703	-34.250	10.266	1.00	85.38	C
ATOM	7580	O	ILE	A	962	13.641	-34.344	10.070	1.00	85.38	O
ATOM	7581	CG1	ILE	A	962	18.031	-34.781	10.820	1.00	85.38	C
ATOM	7582	CG2	ILE	A	962	16.875	-32.875	9.617	1.00	85.38	C
ATOM	7583	CD1	ILE	A	962	19.078	-35.062	9.750	1.00	85.38	C
ATOM	7584	N	THR	A	963	13.898	-32.406	11.312	1.00	89.12	N
ATOM	7585	CA	THR	A	963	12.656	-31.797	10.859	1.00	89.12	C
ATOM	7586	C	THR	A	963	12.898	-30.359	10.391	1.00	89.12	C
ATOM	7587	CB	THR	A	963	11.586	-31.797	11.969	1.00	89.12	C
ATOM	7588	O	THR	A	963	13.523	-29.578	11.102	1.00	89.12	O
ATOM	7589	CG2	THR	A	963	10.273	-31.219	11.469	1.00	89.12	C
ATOM	7590	OG1	THR	A	963	11.359	-33.156	12.391	1.00	89.12	O
ATOM	7591	N	ASP	A	964	12.562	-30.094	9.156	1.00	89.56	N
ATOM	7592	CA	ASP	A	964	12.570	-28.750	8.609	1.00	89.56	C
ATOM	7593	C	ASP	A	964	11.148	-28.234	8.391	1.00	89.56	C
ATOM	7594	CB	ASP	A	964	13.344	-28.703	7.289	1.00	89.56	C
ATOM	7595	O	ASP	A	964	10.352	-28.875	7.691	1.00	89.56	O
ATOM	7596	CG	ASP	A	964	14.836	-28.906	7.473	1.00	89.56	C
ATOM	7597	OD1	ASP	A	964	15.383	-28.500	8.523	1.00	89.56	O
ATOM	7598	OD2	ASP	A	964	15.484	-29.453	6.559	1.00	89.56	O
ATOM	7599	N	VAL	A	965	10.742	-27.062	9.070	1.00	87.88	N
ATOM	7600	CA	VAL	A	965	9.438	-26.438	8.883	1.00	87.88	C
ATOM	7601	C	VAL	A	965	9.617	-25.031	8.328	1.00	87.88	C
ATOM	7602	CB	VAL	A	965	8.633	-26.391	10.195	1.00	87.88	C
ATOM	7603	O	VAL	A	965	10.148	-24.141	9.016	1.00	87.88	O
ATOM	7604	CG1	VAL	A	965	7.312	-25.641	10.000	1.00	87.88	C
ATOM	7605	CG2	VAL	A	965	8.383	-27.812	10.719	1.00	87.88	C
ATOM	7606	N	ASN	A	966	9.352	-24.906	7.012	1.00	86.81	N
ATOM	7607	CA	ASN	A	966	9.383	-23.594	6.363	1.00	86.81	C
ATOM	7608	C	ASN	A	966	7.973	-23.078	6.094	1.00	86.81	C
ATOM	7609	CB	ASN	A	966	10.180	-23.656	5.059	1.00	86.81	C
ATOM	7610	O	ASN	A	966	7.148	-23.781	5.508	1.00	86.81	O
ATOM	7611	CG	ASN	A	966	11.625	-24.062	5.281	1.00	86.81	C
ATOM	7612	ND2	ASN	A	966	12.203	-24.750	4.301	1.00	86.81	N

```
ATOM   7613  OD1 ASN A 966      12.211 -23.781   6.328  1.00 86.81           O
ATOM   7614  N   GLN A 967       7.691 -21.797   6.676  1.00 86.25           N
ATOM   7615  CA  GLN A 967       6.359 -21.203   6.551  1.00 86.25           C
ATOM   7616  C   GLN A 967       6.438 -19.766   6.031  1.00 86.25           C
ATOM   7617  CB  GLN A 967       5.629 -21.234   7.891  1.00 86.25           C
ATOM   7618  O   GLN A 967       7.297 -19.000   6.461  1.00 86.25           O
ATOM   7619  CG  GLN A 967       5.141 -22.625   8.297  1.00 86.25           C
ATOM   7620  CD  GLN A 967       4.371 -22.609   9.602  1.00 86.25           C
ATOM   7621  NE2 GLN A 967       3.680 -23.719   9.883  1.00 86.25           N
ATOM   7622  OE1 GLN A 967       4.398 -21.641  10.352  1.00 86.25           O
ATOM   7623  N   ALA A 968       5.648 -19.500   5.020  1.00 80.12           N
ATOM   7624  CA  ALA A 968       5.445 -18.141   4.520  1.00 80.12           C
ATOM   7625  C   ALA A 968       3.969 -17.750   4.562  1.00 80.12           C
ATOM   7626  CB  ALA A 968       5.980 -18.016   3.094  1.00 80.12           C
ATOM   7627  O   ALA A 968       3.129 -18.422   3.955  1.00 80.12           O
ATOM   7628  N   ILE A 969       3.678 -16.734   5.441  1.00 80.94           N
ATOM   7629  CA  ILE A 969       2.297 -16.312   5.652  1.00 80.94           C
ATOM   7630  C   ILE A 969       2.123 -14.867   5.191  1.00 80.94           C
ATOM   7631  CB  ILE A 969       1.879 -16.453   7.133  1.00 80.94           C
ATOM   7632  O   ILE A 969       2.926 -14.000   5.539  1.00 80.94           O
ATOM   7633  CG1 ILE A 969       2.029 -17.922   7.590  1.00 80.94           C
ATOM   7634  CG2 ILE A 969       0.445 -15.969   7.340  1.00 80.94           C
ATOM   7635  CD1 ILE A 969       1.882 -18.109   9.094  1.00 80.94           C
ATOM   7636  N   THR A 970       1.081 -14.672   4.367  1.00 67.12           N
ATOM   7637  CA  THR A 970       0.679 -13.328   3.969  1.00 67.12           C
ATOM   7638  C   THR A 970      -0.827 -13.141   4.133  1.00 67.12           C
ATOM   7639  CB  THR A 970       1.082 -13.031   2.514  1.00 67.12           C
ATOM   7640  O   THR A 970      -1.614 -13.930   3.607  1.00 67.12           O
ATOM   7641  CG2 THR A 970       1.100 -11.531   2.242  1.00 67.12           C
ATOM   7642  OG1 THR A 970       2.389 -13.562   2.268  1.00 67.12           O
ATOM   7643  N   ASN A 971      -1.096 -12.195   5.098  1.00 70.06           N
ATOM   7644  CA  ASN A 971      -2.494 -11.789   5.184  1.00 70.06           C
ATOM   7645  C   ASN A 971      -2.633 -10.273   5.254  1.00 70.06           C
ATOM   7646  CB  ASN A 971      -3.170 -12.438   6.391  1.00 70.06           C
ATOM   7647  O   ASN A 971      -1.685  -9.539   4.953  1.00 70.06           O
ATOM   7648  CG  ASN A 971      -2.525 -12.047   7.703  1.00 70.06           C
ATOM   7649  ND2 ASN A 971      -2.316 -13.016   8.578  1.00 70.06           N
ATOM   7650  OD1 ASN A 971      -2.219 -10.867   7.930  1.00 70.06           O
ATOM   7651  N   GLU A 972      -3.887  -9.688   5.602  1.00 60.28           N
ATOM   7652  CA  GLU A 972      -4.137  -8.250   5.551  1.00 60.28           C
ATOM   7653  C   GLU A 972      -3.320  -7.516   6.609  1.00 60.28           C
ATOM   7654  CB  GLU A 972      -5.629  -7.957   5.738  1.00 60.28           C
ATOM   7655  O   GLU A 972      -3.008  -6.332   6.445  1.00 60.28           O
ATOM   7656  CG  GLU A 972      -6.484  -8.352   4.543  1.00 60.28           C
ATOM   7657  CD  GLU A 972      -7.961  -8.055   4.738  1.00 60.28           C
ATOM   7658  OE1 GLU A 972      -8.766  -8.328   3.818  1.00 60.28           O
ATOM   7659  OE2 GLU A 972      -8.320  -7.535   5.824  1.00 60.28           O
ATOM   7660  N   ALA A 973      -2.787  -8.195   7.590  1.00 67.12           N
ATOM   7661  CA  ALA A 973      -2.217  -7.555   8.773  1.00 67.12           C
ATOM   7662  C   ALA A 973      -0.743  -7.914   8.938  1.00 67.12           C
ATOM   7663  CB  ALA A 973      -3.000  -7.949  10.023  1.00 67.12           C
ATOM   7664  O   ALA A 973      -0.005  -7.230   9.648  1.00 67.12           O
ATOM   7665  N   GLU A 974      -0.251  -9.062   8.203  1.00 76.38           N
ATOM   7666  CA  GLU A 974       1.083  -9.539   8.547  1.00 76.38           C
ATOM   7667  C   GLU A 974       1.717 -10.297   7.379  1.00 76.38           C
ATOM   7668  CB  GLU A 974       1.028 -10.445   9.781  1.00 76.38           C
ATOM   7669  O   GLU A 974       1.010 -10.859   6.543  1.00 76.38           O
ATOM   7670  CG  GLU A 974       0.363 -11.789   9.539  1.00 76.38           C
ATOM   7671  CD  GLU A 974       0.443 -12.727  10.727  1.00 76.38           C
ATOM   7672  OE1 GLU A 974       0.011 -13.898  10.609  1.00 76.38           O
ATOM   7673  OE2 GLU A 974       0.942 -12.289  11.789  1.00 76.38           O
ATOM   7674  N   ALA A 975       2.961 -10.039   7.168  1.00 76.56           N
ATOM   7675  CA  ALA A 975       3.848 -10.867   6.348  1.00 76.56           C
ATOM   7676  C   ALA A 975       4.957 -11.484   7.195  1.00 76.56           C
```

ATOM	7677	CB	ALA	A	975	4.445	-10.031	5.215	1.00	76.56	C
ATOM	7678	O	ALA	A	975	5.684	-10.773	7.898	1.00	76.56	O
ATOM	7679	N	ARG	A	976	5.000	-12.797	7.223	1.00	89.81	N
ATOM	7680	CA	ARG	A	976	5.980	-13.453	8.078	1.00	89.81	C
ATOM	7681	C	ARG	A	976	6.602	-14.656	7.383	1.00	89.81	C
ATOM	7682	CB	ARG	A	976	5.332	-13.883	9.398	1.00	89.81	C
ATOM	7683	O	ARG	A	976	5.898	-15.438	6.742	1.00	89.81	O
ATOM	7684	CG	ARG	A	976	6.309	-14.500	10.391	1.00	89.81	C
ATOM	7685	CD	ARG	A	976	5.680	-14.664	11.766	1.00	89.81	C
ATOM	7686	NE	ARG	A	976	6.648	-15.156	12.742	1.00	89.81	N
ATOM	7687	NH1	ARG	A	976	5.156	-15.203	14.508	1.00	89.81	N
ATOM	7688	NH2	ARG	A	976	7.336	-15.844	14.828	1.00	89.81	N
ATOM	7689	CZ	ARG	A	976	6.379	-15.398	14.023	1.00	89.81	C
ATOM	7690	N	ALA	A	977	7.875	-14.758	7.328	1.00	85.81	N
ATOM	7691	CA	ALA	A	977	8.641	-15.914	6.867	1.00	85.81	C
ATOM	7692	C	ALA	A	977	9.438	-16.547	8.008	1.00	85.81	C
ATOM	7693	CB	ALA	A	977	9.570	-15.516	5.727	1.00	85.81	C
ATOM	7694	O	ALA	A	977	10.203	-15.859	8.688	1.00	85.81	O
ATOM	7695	N	SER	A	978	9.117	-17.875	8.227	1.00	91.75	N
ATOM	7696	CA	SER	A	978	9.812	-18.547	9.312	1.00	91.75	C
ATOM	7697	C	SER	A	978	10.375	-19.891	8.859	1.00	91.75	C
ATOM	7698	CB	SER	A	978	8.875	-18.750	10.508	1.00	91.75	C
ATOM	7699	O	SER	A	978	9.805	-20.547	7.984	1.00	91.75	O
ATOM	7700	OG	SER	A	978	7.840	-19.672	10.180	1.00	91.75	O
ATOM	7701	N	ALA	A	979	11.594	-20.141	9.375	1.00	90.81	N
ATOM	7702	CA	ALA	A	979	12.250	-21.422	9.133	1.00	90.81	C
ATOM	7703	C	ALA	A	979	12.773	-22.031	10.438	1.00	90.81	C
ATOM	7704	CB	ALA	A	979	13.398	-21.266	8.133	1.00	90.81	C
ATOM	7705	O	ALA	A	979	13.562	-21.391	11.148	1.00	90.81	O
ATOM	7706	N	ASP	A	980	12.242	-23.234	10.773	1.00	92.69	N
ATOM	7707	CA	ASP	A	980	12.695	-23.969	11.945	1.00	92.69	C
ATOM	7708	C	ASP	A	980	13.391	-25.266	11.539	1.00	92.69	C
ATOM	7709	CB	ASP	A	980	11.516	-24.281	12.875	1.00	92.69	C
ATOM	7710	O	ASP	A	980	12.844	-26.062	10.773	1.00	92.69	O
ATOM	7711	CG	ASP	A	980	10.922	-23.031	13.508	1.00	92.69	C
ATOM	7712	OD1	ASP	A	980	11.688	-22.156	13.953	1.00	92.69	O
ATOM	7713	OD2	ASP	A	980	9.680	-22.938	13.562	1.00	92.69	O
ATOM	7714	N	ARG	A	981	14.578	-25.438	11.922	1.00	93.44	N
ATOM	7715	CA	ARG	A	981	15.320	-26.672	11.742	1.00	93.44	C
ATOM	7716	C	ARG	A	981	15.609	-27.344	13.086	1.00	93.44	C
ATOM	7717	CB	ARG	A	981	16.641	-26.406	11.000	1.00	93.44	C
ATOM	7718	O	ARG	A	981	16.172	-26.719	13.984	1.00	93.44	O
ATOM	7719	CG	ARG	A	981	17.312	-27.672	10.484	1.00	93.44	C
ATOM	7720	CD	ARG	A	981	18.453	-27.344	9.523	1.00	93.44	C
ATOM	7721	NE	ARG	A	981	19.062	-28.547	8.984	1.00	93.44	N
ATOM	7722	NH1	ARG	A	981	20.719	-27.438	7.824	1.00	93.44	N
ATOM	7723	NH2	ARG	A	981	20.609	-29.734	7.750	1.00	93.44	N
ATOM	7724	CZ	ARG	A	981	20.125	-28.562	8.188	1.00	93.44	C
ATOM	7725	N	ALA	A	982	15.078	-28.562	13.219	1.00	93.06	N
ATOM	7726	CA	ALA	A	982	15.227	-29.266	14.492	1.00	93.06	C
ATOM	7727	C	ALA	A	982	15.953	-30.594	14.305	1.00	93.06	C
ATOM	7728	CB	ALA	A	982	13.859	-29.500	15.125	1.00	93.06	C
ATOM	7729	O	ALA	A	982	15.531	-31.422	13.500	1.00	93.06	O
ATOM	7730	N	LEU	A	983	17.078	-30.844	15.023	1.00	92.50	N
ATOM	7731	CA	LEU	A	983	17.844	-32.094	15.070	1.00	92.50	C
ATOM	7732	C	LEU	A	983	17.750	-32.719	16.453	1.00	92.50	C
ATOM	7733	CB	LEU	A	983	19.297	-31.844	14.695	1.00	92.50	C
ATOM	7734	O	LEU	A	983	18.016	-32.062	17.453	1.00	92.50	O
ATOM	7735	CG	LEU	A	983	19.594	-31.594	13.219	1.00	92.50	C
ATOM	7736	CD1	LEU	A	983	18.969	-30.266	12.773	1.00	92.50	C
ATOM	7737	CD2	LEU	A	983	21.094	-31.578	12.961	1.00	92.50	C
ATOM	7738	N	SER	A	984	17.266	-33.938	16.453	1.00	90.62	N
ATOM	7739	CA	SER	A	984	17.141	-34.625	17.750	1.00	90.62	C
ATOM	7740	C	SER	A	984	17.734	-36.031	17.672	1.00	90.62	C

ATOM	7741	CB	SER	A	984	15.680	-34.719	18.188	1.00	90.62	C
ATOM	7742	O	SER	A	984	17.578	-36.750	16.672	1.00	90.62	O
ATOM	7743	OG	SER	A	984	15.125	-33.406	18.281	1.00	90.62	O
ATOM	7744	N	THR	A	985	18.500	-36.469	18.609	1.00	83.38	N
ATOM	7745	CA	THR	A	985	18.984	-37.812	18.812	1.00	83.38	C
ATOM	7746	C	THR	A	985	18.656	-38.312	20.219	1.00	83.38	C
ATOM	7747	CB	THR	A	985	20.500	-37.906	18.578	1.00	83.38	C
ATOM	7748	O	THR	A	985	18.719	-37.531	21.188	1.00	83.38	O
ATOM	7749	CG2	THR	A	985	20.859	-37.594	17.125	1.00	83.38	C
ATOM	7750	OG1	THR	A	985	21.172	-36.969	19.438	1.00	83.38	O
ATOM	7751	N	GLN	A	986	18.109	-39.531	20.344	1.00	84.88	N
ATOM	7752	CA	GLN	A	986	17.703	-40.062	21.641	1.00	84.88	C
ATOM	7753	C	GLN	A	986	18.094	-41.531	21.781	1.00	84.88	C
ATOM	7754	CB	GLN	A	986	16.203	-39.906	21.859	1.00	84.88	C
ATOM	7755	O	GLN	A	986	17.875	-42.344	20.875	1.00	84.88	O
ATOM	7756	CG	GLN	A	986	15.703	-40.406	23.188	1.00	84.88	C
ATOM	7757	CD	GLN	A	986	14.203	-40.250	23.375	1.00	84.88	C
ATOM	7758	NE2	GLN	A	986	13.680	-40.719	24.500	1.00	84.88	N
ATOM	7759	OE1	GLN	A	986	13.523	-39.688	22.516	1.00	84.88	O
ATOM	7760	N	ILE	A	987	18.703	-41.906	22.875	1.00	76.12	N
ATOM	7761	CA	ILE	A	987	18.922	-43.281	23.344	1.00	76.12	C
ATOM	7762	C	ILE	A	987	18.422	-43.438	24.766	1.00	76.12	C
ATOM	7763	CB	ILE	A	987	20.422	-43.688	23.250	1.00	76.12	C
ATOM	7764	O	ILE	A	987	19.016	-42.906	25.703	1.00	76.12	O
ATOM	7765	CG1	ILE	A	987	20.922	-43.531	21.812	1.00	76.12	C
ATOM	7766	CG2	ILE	A	987	20.641	-45.094	23.781	1.00	76.12	C
ATOM	7767	CD1	ILE	A	987	22.406	-43.812	21.656	1.00	76.12	C
ATOM	7768	N	GLY	A	988	17.328	-44.125	24.906	1.00	75.81	N
ATOM	7769	CA	GLY	A	988	16.781	-44.188	26.250	1.00	75.81	C
ATOM	7770	C	GLY	A	988	16.359	-42.844	26.812	1.00	75.81	C
ATOM	7771	O	GLY	A	988	15.531	-42.156	26.219	1.00	75.81	O
ATOM	7772	N	ASP	A	989	17.047	-42.344	27.953	1.00	78.12	N
ATOM	7773	CA	ASP	A	989	16.750	-41.094	28.609	1.00	78.12	C
ATOM	7774	C	ASP	A	989	17.734	-40.000	28.188	1.00	78.12	C
ATOM	7775	CB	ASP	A	989	16.781	-41.250	30.141	1.00	78.12	C
ATOM	7776	O	ASP	A	989	17.562	-38.844	28.547	1.00	78.12	O
ATOM	7777	CG	ASP	A	989	15.648	-42.125	30.672	1.00	78.12	C
ATOM	7778	OD1	ASP	A	989	14.609	-42.219	29.984	1.00	78.12	O
ATOM	7779	OD2	ASP	A	989	15.781	-42.656	31.781	1.00	78.12	O
ATOM	7780	N	THR	A	990	18.750	-40.344	27.422	1.00	85.50	N
ATOM	7781	CA	THR	A	990	19.766	-39.406	26.938	1.00	85.50	C
ATOM	7782	C	THR	A	990	19.359	-38.844	25.578	1.00	85.50	C
ATOM	7783	CB	THR	A	990	21.141	-40.094	26.797	1.00	85.50	C
ATOM	7784	O	THR	A	990	19.078	-39.562	24.641	1.00	85.50	O
ATOM	7785	CG2	THR	A	990	22.219	-39.094	26.406	1.00	85.50	C
ATOM	7786	OG1	THR	A	990	21.500	-40.688	28.047	1.00	85.50	O
ATOM	7787	N	GLN	A	991	19.281	-37.469	25.531	1.00	90.94	N
ATOM	7788	CA	GLN	A	991	18.766	-36.781	24.344	1.00	90.94	C
ATOM	7789	C	GLN	A	991	19.625	-35.594	23.984	1.00	90.94	C
ATOM	7790	CB	GLN	A	991	17.312	-36.344	24.562	1.00	90.94	C
ATOM	7791	O	GLN	A	991	20.156	-34.906	24.875	1.00	90.94	O
ATOM	7792	CG	GLN	A	991	16.641	-35.781	23.328	1.00	90.94	C
ATOM	7793	CD	GLN	A	991	15.172	-35.469	23.531	1.00	90.94	C
ATOM	7794	NE2	GLN	A	991	14.492	-35.094	22.469	1.00	90.94	N
ATOM	7795	OE1	GLN	A	991	14.656	-35.594	24.656	1.00	90.94	O
ATOM	7796	N	SER	A	992	19.906	-35.406	22.734	1.00	91.69	N
ATOM	7797	CA	SER	A	992	20.516	-34.188	22.234	1.00	91.69	C
ATOM	7798	C	SER	A	992	19.609	-33.500	21.203	1.00	91.69	C
ATOM	7799	CB	SER	A	992	21.875	-34.500	21.594	1.00	91.69	C
ATOM	7800	O	SER	A	992	18.891	-34.156	20.453	1.00	91.69	O
ATOM	7801	OG	SER	A	992	21.719	-35.188	20.375	1.00	91.69	O
ATOM	7802	N	ALA	A	993	19.562	-32.219	21.312	1.00	93.62	N
ATOM	7803	CA	ALA	A	993	18.719	-31.453	20.406	1.00	93.62	C
ATOM	7804	C	ALA	A	993	19.438	-30.188	19.938	1.00	93.62	C

```
ATOM   7805  CB  ALA A 993      17.406 -31.094  21.078  1.00 93.62           C
ATOM   7806  O   ALA A 993      20.188 -29.578  20.688  1.00 93.62           O
ATOM   7807  N   VAL A 994      19.391 -29.969  18.672  1.00 94.38           N
ATOM   7808  CA  VAL A 994      19.812 -28.703  18.078  1.00 94.38           C
ATOM   7809  C   VAL A 994      18.656 -28.094  17.297  1.00 94.38           C
ATOM   7810  CB  VAL A 994      21.031 -28.891  17.141  1.00 94.38           C
ATOM   7811  O   VAL A 994      17.984 -28.766  16.516  1.00 94.38           O
ATOM   7812  CG1 VAL A 994      21.438 -27.578  16.500  1.00 94.38           C
ATOM   7813  CG2 VAL A 994      22.203 -29.516  17.906  1.00 94.38           C
ATOM   7814  N   ASN A 995      18.359 -26.781  17.578  1.00 93.00           N
ATOM   7815  CA  ASN A 995      17.359 -26.125  16.734  1.00 93.00           C
ATOM   7816  C   ASN A 995      17.828 -24.750  16.281  1.00 93.00           C
ATOM   7817  CB  ASN A 995      16.031 -26.031  17.469  1.00 93.00           C
ATOM   7818  O   ASN A 995      18.500 -24.031  17.047  1.00 93.00           O
ATOM   7819  CG  ASN A 995      16.062 -25.031  18.609  1.00 93.00           C
ATOM   7820  ND2 ASN A 995      15.016 -25.031  19.438  1.00 93.00           N
ATOM   7821  OD1 ASN A 995      17.016 -24.281  18.766  1.00 93.00           O
ATOM   7822  N   GLN A 996      17.594 -24.469  15.078  1.00 94.38           N
ATOM   7823  CA  GLN A 996      17.875 -23.203  14.406  1.00 94.38           C
ATOM   7824  C   GLN A 996      16.578 -22.531  13.953  1.00 94.38           C
ATOM   7825  CB  GLN A 996      18.797 -23.422  13.203  1.00 94.38           C
ATOM   7826  O   GLN A 996      15.688 -23.172  13.398  1.00 94.38           O
ATOM   7827  CG  GLN A 996      19.266 -22.141  12.547  1.00 94.38           C
ATOM   7828  CD  GLN A 996      20.156 -22.375  11.352  1.00 94.38           C
ATOM   7829  NE2 GLN A 996      21.062 -21.438  11.094  1.00 94.38           N
ATOM   7830  OE1 GLN A 996      20.047 -23.406  10.672  1.00 94.38           O
ATOM   7831  N   LYS A 997      16.453 -21.156  14.219  1.00 93.88           N
ATOM   7832  CA  LYS A 997      15.250 -20.406  13.844  1.00 93.88           C
ATOM   7833  C   LYS A 997      15.609 -19.156  13.055  1.00 93.88           C
ATOM   7834  CB  LYS A 997      14.445 -20.031  15.086  1.00 93.88           C
ATOM   7835  O   LYS A 997      16.469 -18.375  13.469  1.00 93.88           O
ATOM   7836  CG  LYS A 997      13.102 -19.375  14.781  1.00 93.88           C
ATOM   7837  CD  LYS A 997      12.242 -19.250  16.031  1.00 93.88           C
ATOM   7838  CE  LYS A 997      10.867 -18.688  15.711  1.00 93.88           C
ATOM   7839  NZ  LYS A 997       9.859 -19.031  16.766  1.00 93.88           N
ATOM   7840  N   LEU A 998      15.039 -19.078  11.859  1.00 94.88           N
ATOM   7841  CA  LEU A 998      14.992 -17.844  11.086  1.00 94.88           C
ATOM   7842  C   LEU A 998      13.578 -17.297  11.031  1.00 94.88           C
ATOM   7843  CB  LEU A 998      15.516 -18.094   9.664  1.00 94.88           C
ATOM   7844  O   LEU A 998      12.641 -18.016  10.656  1.00 94.88           O
ATOM   7845  CG  LEU A 998      16.938 -18.641   9.547  1.00 94.88           C
ATOM   7846  CD1 LEU A 998      17.188 -19.172   8.141  1.00 94.88           C
ATOM   7847  CD2 LEU A 998      17.953 -17.562   9.906  1.00 94.88           C
ATOM   7848  N   ASP A 999      13.422 -16.062  11.438  1.00 92.50           N
ATOM   7849  CA  ASP A 999      12.102 -15.445  11.531  1.00 92.50           C
ATOM   7850  C   ASP A 999      12.148 -13.992  11.078  1.00 92.50           C
ATOM   7851  CB  ASP A 999      11.570 -15.539  12.961  1.00 92.50           C
ATOM   7852  O   ASP A 999      12.984 -13.211  11.547  1.00 92.50           O
ATOM   7853  CG  ASP A 999      10.055 -15.438  13.039  1.00 92.50           C
ATOM   7854  OD1 ASP A 999       9.391 -15.453  11.977  1.00 92.50           O
ATOM   7855  OD2 ASP A 999       9.523 -15.344  14.164  1.00 92.50           O
ATOM   7856  N   SER A1000      11.430 -13.711  10.055  1.00 90.88           N
ATOM   7857  CA  SER A1000      11.250 -12.352   9.562  1.00 90.88           C
ATOM   7858  C   SER A1000       9.773 -11.984   9.477  1.00 90.88           C
ATOM   7859  CB  SER A1000      11.906 -12.180   8.188  1.00 90.88           C
ATOM   7860  O   SER A1000       8.969 -12.750   8.938  1.00 90.88           O
ATOM   7861  OG  SER A1000      11.750 -10.859   7.715  1.00 90.88           O
ATOM   7862  N   TRP A1001       9.438 -10.797  10.109  1.00 91.56           N
ATOM   7863  CA  TRP A1001       8.016 -10.469  10.094  1.00 91.56           C
ATOM   7864  C   TRP A1001       7.809  -8.961   9.953  1.00 91.56           C
ATOM   7865  CB  TRP A1001       7.336 -10.977  11.375  1.00 91.56           C
ATOM   7866  O   TRP A1001       8.719  -8.180  10.227  1.00 91.56           O
ATOM   7867  CG  TRP A1001       7.758 -10.250  12.617  1.00 91.56           C
ATOM   7868  CD1 TRP A1001       7.137  -9.180  13.195  1.00 91.56           C
```

```
ATOM   7869  CD2 TRP A1001       8.906 -10.539  13.422  1.00 91.56           C
ATOM   7870  CE2 TRP A1001       8.914  -9.602  14.484  1.00 91.56           C
ATOM   7871  CE3 TRP A1001       9.922 -11.500  13.359  1.00 91.56           C
ATOM   7872  NE1 TRP A1001       7.824  -8.789  14.320  1.00 91.56           N
ATOM   7873  CH2 TRP A1001      10.883 -10.555  15.383  1.00 91.56           C
ATOM   7874  CZ2 TRP A1001       9.898  -9.602  15.469  1.00 91.56           C
ATOM   7875  CZ3 TRP A1001      10.906 -11.492  14.344  1.00 91.56           C
ATOM   7876  N   VAL A1002       6.652  -8.609   9.305  1.00 85.31           N
ATOM   7877  CA  VAL A1002       6.152  -7.242   9.242  1.00 85.31           C
ATOM   7878  C   VAL A1002       4.664  -7.219   9.586  1.00 85.31           C
ATOM   7879  CB  VAL A1002       6.391  -6.621   7.848  1.00 85.31           C
ATOM   7880  O   VAL A1002       3.871  -7.941   8.977  1.00 85.31           O
ATOM   7881  CG1 VAL A1002       5.852  -5.191   7.797  1.00 85.31           C
ATOM   7882  CG2 VAL A1002       7.875  -6.648   7.496  1.00 85.31           C
ATOM   7883  N   ASN A1003       4.309  -6.496  10.625  1.00 83.56           N
ATOM   7884  CA  ASN A1003       2.898  -6.305  10.945  1.00 83.56           C
ATOM   7885  C   ASN A1003       2.604  -4.863  11.336  1.00 83.56           C
ATOM   7886  CB  ASN A1003       2.465  -7.262  12.055  1.00 83.56           C
ATOM   7887  O   ASN A1003       3.447  -3.980  11.164  1.00 83.56           O
ATOM   7888  CG  ASN A1003       3.244  -7.055  13.344  1.00 83.56           C
ATOM   7889  ND2 ASN A1003       3.664  -8.148  13.961  1.00 83.56           N
ATOM   7890  OD1 ASN A1003       3.467  -5.918  13.773  1.00 83.56           O
ATOM   7891  N   ALA A1004       1.414  -4.500  11.734  1.00 77.44           N
ATOM   7892  CA  ALA A1004       0.977  -3.133  12.000  1.00 77.44           C
ATOM   7893  C   ALA A1004       1.760  -2.527  13.164  1.00 77.44           C
ATOM   7894  CB  ALA A1004      -0.520  -3.102  12.305  1.00 77.44           C
ATOM   7895  O   ALA A1004       2.002  -1.317  13.195  1.00 77.44           O
ATOM   7896  N   ASP A1005       2.303  -3.432  14.008  1.00 80.44           N
ATOM   7897  CA  ASP A1005       2.846  -2.980  15.281  1.00 80.44           C
ATOM   7898  C   ASP A1005       4.371  -3.027  15.281  1.00 80.44           C
ATOM   7899  CB  ASP A1005       2.297  -3.834  16.438  1.00 80.44           C
ATOM   7900  O   ASP A1005       5.023  -2.271  16.000  1.00 80.44           O
ATOM   7901  CG  ASP A1005       0.789  -3.736  16.578  1.00 80.44           C
ATOM   7902  OD1 ASP A1005       0.234  -2.625  16.453  1.00 80.44           O
ATOM   7903  OD2 ASP A1005       0.148  -4.781  16.812  1.00 80.44           O
ATOM   7904  N   SER A1006       5.000  -4.023  14.391  1.00 85.44           N
ATOM   7905  CA  SER A1006       6.438  -4.230  14.547  1.00 85.44           C
ATOM   7906  C   SER A1006       7.047  -4.836  13.289  1.00 85.44           C
ATOM   7907  CB  SER A1006       6.719  -5.141  15.742  1.00 85.44           C
ATOM   7908  O   SER A1006       6.328  -5.383  12.445  1.00 85.44           O
ATOM   7909  OG  SER A1006       6.316  -6.473  15.477  1.00 85.44           O
ATOM   7910  N   VAL A1007       8.219  -4.496  13.039  1.00 87.56           N
ATOM   7911  CA  VAL A1007       9.062  -5.117  12.023  1.00 87.56           C
ATOM   7912  C   VAL A1007      10.289  -5.738  12.688  1.00 87.56           C
ATOM   7913  CB  VAL A1007       9.492  -4.102  10.945  1.00 87.56           C
ATOM   7914  O   VAL A1007      10.906  -5.125  13.555  1.00 87.56           O
ATOM   7915  CG1 VAL A1007      10.383  -3.021  11.547  1.00 87.56           C
ATOM   7916  CG2 VAL A1007      10.211  -4.816   9.797  1.00 87.56           C
ATOM   7917  N   GLY A1008      10.492  -7.039  12.297  1.00 91.12           N
ATOM   7918  CA  GLY A1008      11.641  -7.621  12.977  1.00 91.12           C
ATOM   7919  C   GLY A1008      12.258  -8.781  12.219  1.00 91.12           C
ATOM   7920  O   GLY A1008      11.641  -9.320  11.297  1.00 91.12           O
ATOM   7921  N   ALA A1009      13.484  -8.984  12.422  1.00 91.38           N
ATOM   7922  CA  ALA A1009      14.242 -10.141  11.945  1.00 91.38           C
ATOM   7923  C   ALA A1009      15.008 -10.805  13.086  1.00 91.38           C
ATOM   7924  CB  ALA A1009      15.211  -9.727  10.836  1.00 91.38           C
ATOM   7925  O   ALA A1009      15.547 -10.125  13.961  1.00 91.38           O
ATOM   7926  N   MET A1010      14.930 -12.195  13.094  1.00 93.81           N
ATOM   7927  CA  MET A1010      15.578 -12.930  14.180  1.00 93.81           C
ATOM   7928  C   MET A1010      16.328 -14.148  13.641  1.00 93.81           C
ATOM   7929  CB  MET A1010      14.555 -13.359  15.227  1.00 93.81           C
ATOM   7930  O   MET A1010      15.883 -14.773  12.672  1.00 93.81           O
ATOM   7931  CG  MET A1010      15.148 -14.195  16.359  1.00 93.81           C
ATOM   7932  SD  MET A1010      13.867 -14.820  17.516  1.00 93.81           S
```

ATOM	7933	CE	MET	A1010	14.117	-16.609	17.312	1.00	93.81	C
ATOM	7934	N	TYR	A1011	17.516	-14.359	14.203	1.00	95.38	N
ATOM	7935	CA	TYR	A1011	18.312	-15.562	14.000	1.00	95.38	C
ATOM	7936	C	TYR	A1011	18.688	-16.203	15.336	1.00	95.38	C
ATOM	7937	CB	TYR	A1011	19.578	-15.234	13.211	1.00	95.38	C
ATOM	7938	O	TYR	A1011	19.219	-15.539	16.219	1.00	95.38	O
ATOM	7939	CG	TYR	A1011	20.547	-16.391	13.094	1.00	95.38	C
ATOM	7940	CD1	TYR	A1011	21.797	-16.359	13.703	1.00	95.38	C
ATOM	7941	CD2	TYR	A1011	20.203	-17.531	12.367	1.00	95.38	C
ATOM	7942	CE1	TYR	A1011	22.688	-17.422	13.594	1.00	95.38	C
ATOM	7943	CE2	TYR	A1011	21.078	-18.609	12.258	1.00	95.38	C
ATOM	7944	OH	TYR	A1011	23.188	-19.594	12.758	1.00	95.38	O
ATOM	7945	CZ	TYR	A1011	22.312	-18.547	12.867	1.00	95.38	C
ATOM	7946	N	GLY	A1012	18.344	-17.469	15.508	1.00	94.31	N
ATOM	7947	CA	GLY	A1012	18.641	-18.141	16.766	1.00	94.31	C
ATOM	7948	C	GLY	A1012	19.156	-19.562	16.578	1.00	94.31	C
ATOM	7949	O	GLY	A1012	18.719	-20.266	15.664	1.00	94.31	O
ATOM	7950	N	VAL	A1013	20.188	-19.922	17.438	1.00	95.25	N
ATOM	7951	CA	VAL	A1013	20.688	-21.297	17.516	1.00	95.25	C
ATOM	7952	C	VAL	A1013	20.672	-21.750	18.984	1.00	95.25	C
ATOM	7953	CB	VAL	A1013	22.125	-21.406	16.938	1.00	95.25	C
ATOM	7954	O	VAL	A1013	21.094	-21.016	19.875	1.00	95.25	O
ATOM	7955	CG1	VAL	A1013	22.625	-22.844	17.047	1.00	95.25	C
ATOM	7956	CG2	VAL	A1013	22.141	-20.938	15.492	1.00	95.25	C
ATOM	7957	N	LYS	A1014	20.109	-22.938	19.172	1.00	94.56	N
ATOM	7958	CA	LYS	A1014	20.047	-23.453	20.531	1.00	94.56	C
ATOM	7959	C	LYS	A1014	20.453	-24.922	20.578	1.00	94.56	C
ATOM	7960	CB	LYS	A1014	18.641	-23.266	21.109	1.00	94.56	C
ATOM	7961	O	LYS	A1014	20.047	-25.719	19.719	1.00	94.56	O
ATOM	7962	CG	LYS	A1014	18.594	-23.344	22.625	1.00	94.56	C
ATOM	7963	CD	LYS	A1014	17.250	-22.844	23.172	1.00	94.56	C
ATOM	7964	CE	LYS	A1014	17.297	-22.672	24.688	1.00	94.56	C
ATOM	7965	NZ	LYS	A1014	15.984	-22.188	25.219	1.00	94.56	N
ATOM	7966	N	LEU	A1015	21.188	-25.266	21.547	1.00	93.69	N
ATOM	7967	CA	LEU	A1015	21.641	-26.625	21.812	1.00	93.69	C
ATOM	7968	C	LEU	A1015	21.125	-27.125	23.156	1.00	93.69	C
ATOM	7969	CB	LEU	A1015	23.172	-26.703	21.797	1.00	93.69	C
ATOM	7970	O	LEU	A1015	21.000	-26.344	24.109	1.00	93.69	O
ATOM	7971	CG	LEU	A1015	23.875	-26.047	20.609	1.00	93.69	C
ATOM	7972	CD1	LEU	A1015	25.359	-25.844	20.906	1.00	93.69	C
ATOM	7973	CD2	LEU	A1015	23.688	-26.875	19.344	1.00	93.69	C
ATOM	7974	N	GLY	A1016	20.672	-28.359	23.156	1.00	93.19	N
ATOM	7975	CA	GLY	A1016	20.203	-28.953	24.406	1.00	93.19	C
ATOM	7976	C	GLY	A1016	20.578	-30.406	24.562	1.00	93.19	C
ATOM	7977	O	GLY	A1016	20.609	-31.156	23.578	1.00	93.19	O
ATOM	7978	N	ILE	A1017	20.984	-30.781	25.766	1.00	92.94	N
ATOM	7979	CA	ILE	A1017	21.281	-32.156	26.094	1.00	92.94	C
ATOM	7980	C	ILE	A1017	20.562	-32.562	27.375	1.00	92.94	C
ATOM	7981	CB	ILE	A1017	22.812	-32.406	26.250	1.00	92.94	C
ATOM	7982	O	ILE	A1017	20.422	-31.750	28.297	1.00	92.94	O
ATOM	7983	CG1	ILE	A1017	23.547	-31.953	24.984	1.00	92.94	C
ATOM	7984	CG2	ILE	A1017	23.109	-33.875	26.562	1.00	92.94	C
ATOM	7985	CD1	ILE	A1017	25.062	-31.875	25.141	1.00	92.94	C
ATOM	7986	N	ARG	A1018	19.859	-33.688	27.359	1.00	90.62	N
ATOM	7987	CA	ARG	A1018	19.391	-34.375	28.578	1.00	90.62	C
ATOM	7988	C	ARG	A1018	20.281	-35.562	28.922	1.00	90.62	C
ATOM	7989	CB	ARG	A1018	17.938	-34.812	28.406	1.00	90.62	C
ATOM	7990	O	ARG	A1018	20.359	-36.500	28.156	1.00	90.62	O
ATOM	7991	CG	ARG	A1018	17.328	-35.406	29.672	1.00	90.62	C
ATOM	7992	CD	ARG	A1018	15.867	-35.781	29.484	1.00	90.62	C
ATOM	7993	NE	ARG	A1018	15.062	-34.594	29.125	1.00	90.62	N
ATOM	7994	NH1	ARG	A1018	13.117	-35.781	28.750	1.00	90.62	N
ATOM	7995	NH2	ARG	A1018	13.141	-33.531	28.484	1.00	90.62	N
ATOM	7996	CZ	ARG	A1018	13.773	-34.625	28.781	1.00	90.62	C

```
ATOM   7997  N   TYR A1019      21.078 -35.438  29.844  1.00 85.00           N
ATOM   7998  CA  TYR A1019      22.016 -36.438  30.281  1.00 85.00           C
ATOM   7999  C   TYR A1019      21.859 -36.719  31.781  1.00 85.00           C
ATOM   8000  CB  TYR A1019      23.453 -36.000  29.984  1.00 85.00           C
ATOM   8001  O   TYR A1019      21.953 -35.781  32.594  1.00 85.00           O
ATOM   8002  CG  TYR A1019      24.500 -37.031  30.328  1.00 85.00           C
ATOM   8003  CD1 TYR A1019      25.344 -36.875  31.422  1.00 85.00           C
ATOM   8004  CD2 TYR A1019      24.625 -38.188  29.562  1.00 85.00           C
ATOM   8005  CE1 TYR A1019      26.312 -37.812  31.734  1.00 85.00           C
ATOM   8006  CE2 TYR A1019      25.578 -39.156  29.859  1.00 85.00           C
ATOM   8007  OH  TYR A1019      27.359 -39.906  31.266  1.00 85.00           O
ATOM   8008  CZ  TYR A1019      26.422 -38.969  30.953  1.00 85.00           C
ATOM   8009  N   ASN A1020      21.469 -37.938  32.156  1.00 82.56           N
ATOM   8010  CA  ASN A1020      21.312 -38.375  33.562  1.00 82.56           C
ATOM   8011  C   ASN A1020      20.188 -37.594  34.250  1.00 82.56           C
ATOM   8012  CB  ASN A1020      22.609 -38.219  34.312  1.00 82.56           C
ATOM   8013  O   ASN A1020      20.328 -37.156  35.375  1.00 82.56           O
ATOM   8014  CG  ASN A1020      23.656 -39.250  33.938  1.00 82.56           C
ATOM   8015  ND2 ASN A1020      24.906 -39.000  34.281  1.00 82.56           N
ATOM   8016  OD1 ASN A1020      23.328 -40.281  33.375  1.00 82.56           O
ATOM   8017  N   GLY A1021      19.188 -37.281  33.469  1.00 80.62           N
ATOM   8018  CA  GLY A1021      18.016 -36.656  34.062  1.00 80.62           C
ATOM   8019  C   GLY A1021      18.125 -35.125  34.125  1.00 80.62           C
ATOM   8020  O   GLY A1021      17.172 -34.469  34.531  1.00 80.62           O
ATOM   8021  N   GLN A1022      19.344 -34.625  33.812  1.00 85.88           N
ATOM   8022  CA  GLN A1022      19.547 -33.156  33.875  1.00 85.88           C
ATOM   8023  C   GLN A1022      19.578 -32.562  32.500  1.00 85.88           C
ATOM   8024  CB  GLN A1022      20.828 -32.844  34.625  1.00 85.88           C
ATOM   8025  O   GLN A1022      20.156 -33.125  31.562  1.00 85.88           O
ATOM   8026  CG  GLN A1022      20.828 -33.250  36.094  1.00 85.88           C
ATOM   8027  CD  GLN A1022      20.016 -32.281  36.969  1.00 85.88           C
ATOM   8028  NE2 GLN A1022      19.453 -32.812  38.062  1.00 85.88           N
ATOM   8029  OE1 GLN A1022      19.922 -31.109  36.656  1.00 85.88           O
ATOM   8030  N   GLU A1023      19.000 -31.438  32.250  1.00 89.00           N
ATOM   8031  CA  GLU A1023      18.922 -30.750  30.969  1.00 89.00           C
ATOM   8032  C   GLU A1023      19.922 -29.594  30.906  1.00 89.00           C
ATOM   8033  CB  GLU A1023      17.500 -30.234  30.719  1.00 89.00           C
ATOM   8034  O   GLU A1023      20.047 -28.828  31.859  1.00 89.00           O
ATOM   8035  CG  GLU A1023      16.484 -31.344  30.484  1.00 89.00           C
ATOM   8036  CD  GLU A1023      15.086 -30.828  30.188  1.00 89.00           C
ATOM   8037  OE1 GLU A1023      14.188 -31.641  29.875  1.00 89.00           O
ATOM   8038  OE2 GLU A1023      14.891 -29.594  30.281  1.00 89.00           O
ATOM   8039  N   TYR A1024      20.766 -29.578  29.938  1.00 89.19           N
ATOM   8040  CA  TYR A1024      21.703 -28.500  29.672  1.00 89.19           C
ATOM   8041  C   TYR A1024      21.359 -27.781  28.375  1.00 89.19           C
ATOM   8042  CB  TYR A1024      23.141 -29.047  29.594  1.00 89.19           C
ATOM   8043  O   TYR A1024      20.891 -28.406  27.406  1.00 89.19           O
ATOM   8044  CG  TYR A1024      23.578 -29.766  30.844  1.00 89.19           C
ATOM   8045  CD1 TYR A1024      24.219 -29.094  31.875  1.00 89.19           C
ATOM   8046  CD2 TYR A1024      23.344 -31.125  31.000  1.00 89.19           C
ATOM   8047  CE1 TYR A1024      24.625 -29.750  33.031  1.00 89.19           C
ATOM   8048  CE2 TYR A1024      23.750 -31.797  32.156  1.00 89.19           C
ATOM   8049  OH  TYR A1024      24.781 -31.766  34.312  1.00 89.19           O
ATOM   8050  CZ  TYR A1024      24.375 -31.109  33.156  1.00 89.19           C
ATOM   8051  N   SER A1025      21.547 -26.438  28.359  1.00 90.38           N
ATOM   8052  CA  SER A1025      21.219 -25.688  27.156  1.00 90.38           C
ATOM   8053  C   SER A1025      22.250 -24.594  26.891  1.00 90.38           C
ATOM   8054  CB  SER A1025      19.828 -25.062  27.281  1.00 90.38           C
ATOM   8055  O   SER A1025      22.906 -24.109  27.828  1.00 90.38           O
ATOM   8056  OG  SER A1025      19.781 -24.141  28.344  1.00 90.38           O
ATOM   8057  N   ALA A1026      22.594 -24.406  25.734  1.00 91.75           N
ATOM   8058  CA  ALA A1026      23.391 -23.281  25.234  1.00 91.75           C
ATOM   8059  C   ALA A1026      22.781 -22.719  23.938  1.00 91.75           C
ATOM   8060  CB  ALA A1026      24.828 -23.703  25.000  1.00 91.75           C
```

```
ATOM   8061  O   ALA A1026      22.297 -23.484  23.094  1.00 91.75           O
ATOM   8062  N   GLY A1027      22.766 -21.375  23.828  1.00 93.50           N
ATOM   8063  CA  GLY A1027      22.172 -20.828  22.609  1.00 93.50           C
ATOM   8064  C   GLY A1027      22.484 -19.375  22.391  1.00 93.50           C
ATOM   8065  O   GLY A1027      23.094 -18.719  23.234  1.00 93.50           O
ATOM   8066  N   MET A1028      22.281 -18.906  21.172  1.00 94.56           N
ATOM   8067  CA  MET A1028      22.391 -17.484  20.812  1.00 94.56           C
ATOM   8068  C   MET A1028      21.172 -17.016  20.047  1.00 94.56           C
ATOM   8069  CB  MET A1028      23.656 -17.250  19.984  1.00 94.56           C
ATOM   8070  O   MET A1028      20.531 -17.812  19.344  1.00 94.56           O
ATOM   8071  CG  MET A1028      23.578 -17.812  18.562  1.00 94.56           C
ATOM   8072  SD  MET A1028      25.031 -17.344  17.547  1.00 94.56           S
ATOM   8073  CE  MET A1028      26.172 -18.688  18.016  1.00 94.56           C
ATOM   8074  N   ALA A1029      20.875 -15.742  20.281  1.00 94.06           N
ATOM   8075  CA  ALA A1029      19.766 -15.102  19.562  1.00 94.06           C
ATOM   8076  C   ALA A1029      20.141 -13.695  19.109  1.00 94.06           C
ATOM   8077  CB  ALA A1029      18.516 -15.055  20.453  1.00 94.06           C
ATOM   8078  O   ALA A1029      20.531 -12.852  19.938  1.00 94.06           O
ATOM   8079  N   LEU A1030      20.094 -13.477  17.766  1.00 94.88           N
ATOM   8080  CA  LEU A1030      20.281 -12.172  17.141  1.00 94.88           C
ATOM   8081  C   LEU A1030      18.953 -11.602  16.656  1.00 94.88           C
ATOM   8082  CB  LEU A1030      21.266 -12.273  15.969  1.00 94.88           C
ATOM   8083  O   LEU A1030      18.250 -12.250  15.867  1.00 94.88           O
ATOM   8084  CG  LEU A1030      21.547 -10.977  15.203  1.00 94.88           C
ATOM   8085  CD1 LEU A1030      22.250  -9.969  16.109  1.00 94.88           C
ATOM   8086  CD2 LEU A1030      22.391 -11.258  13.961  1.00 94.88           C
ATOM   8087  N   SER A1031      18.625 -10.438  17.156  1.00 93.25           N
ATOM   8088  CA  SER A1031      17.312  -9.898  16.781  1.00 93.25           C
ATOM   8089  C   SER A1031      17.391  -8.398  16.516  1.00 93.25           C
ATOM   8090  CB  SER A1031      16.281 -10.172  17.891  1.00 93.25           C
ATOM   8091  O   SER A1031      18.188  -7.691  17.141  1.00 93.25           O
ATOM   8092  OG  SER A1031      16.641  -9.492  19.078  1.00 93.25           O
ATOM   8093  N   LEU A1032      16.688  -7.898  15.430  1.00 91.62           N
ATOM   8094  CA  LEU A1032      16.438  -6.508  15.078  1.00 91.62           C
ATOM   8095  C   LEU A1032      14.930  -6.234  15.023  1.00 91.62           C
ATOM   8096  CB  LEU A1032      17.078  -6.156  13.742  1.00 91.62           C
ATOM   8097  O   LEU A1032      14.242  -6.719  14.125  1.00 91.62           O
ATOM   8098  CG  LEU A1032      17.422  -4.684  13.516  1.00 91.62           C
ATOM   8099  CD1 LEU A1032      18.844  -4.398  14.008  1.00 91.62           C
ATOM   8100  CD2 LEU A1032      17.281  -4.312  12.047  1.00 91.62           C
ATOM   8101  N   VAL A1033      14.469  -5.527  16.047  1.00 88.88           N
ATOM   8102  CA  VAL A1033      13.023  -5.359  16.156  1.00 88.88           C
ATOM   8103  C   VAL A1033      12.680  -3.879  16.297  1.00 88.88           C
ATOM   8104  CB  VAL A1033      12.445  -6.152  17.359  1.00 88.88           C
ATOM   8105  O   VAL A1033      13.258  -3.178  17.141  1.00 88.88           O
ATOM   8106  CG1 VAL A1033      10.930  -6.000  17.438  1.00 88.88           C
ATOM   8107  CG2 VAL A1033      12.836  -7.625  17.266  1.00 88.88           C
ATOM   8108  N   ALA A1034      11.789  -3.365  15.398  1.00 85.69           N
ATOM   8109  CA  ALA A1034      11.273  -2.002  15.484  1.00 85.69           C
ATOM   8110  C   ALA A1034       9.844  -1.994  16.016  1.00 85.69           C
ATOM   8111  CB  ALA A1034      11.328  -1.322  14.117  1.00 85.69           C
ATOM   8112  O   ALA A1034       8.945  -2.590  15.422  1.00 85.69           O
ATOM   8113  N   ASP A1035       9.617  -1.389  17.297  1.00 75.56           N
ATOM   8114  CA  ASP A1035       8.305  -1.255  17.938  1.00 75.56           C
ATOM   8115  C   ASP A1035       8.148   0.119  18.578  1.00 75.56           C
ATOM   8116  CB  ASP A1035       8.102  -2.355  18.984  1.00 75.56           C
ATOM   8117  O   ASP A1035       9.070   0.618  19.219  1.00 75.56           O
ATOM   8118  CG  ASP A1035       8.812  -2.068  20.297  1.00 75.56           C
ATOM   8119  OD1 ASP A1035       9.555  -1.067  20.375  1.00 75.56           O
ATOM   8120  OD2 ASP A1035       8.625  -2.852  21.250  1.00 75.56           O
ATOM   8121  N   GLY A1036       6.984   0.940  18.203  1.00 64.12           N
ATOM   8122  CA  GLY A1036       6.402   2.057  18.922  1.00 64.12           C
ATOM   8123  C   GLY A1036       7.309   3.273  18.984  1.00 64.12           C
ATOM   8124  O   GLY A1036       6.828   4.410  19.016  1.00 64.12           O
```

```
ATOM   8125  N   GLY A1037       8.789   3.170  18.734  1.00 65.19           N
ATOM   8126  CA  GLY A1037       9.516   4.430  18.766  1.00 65.19           C
ATOM   8127  C   GLY A1037      11.008   4.266  18.594  1.00 65.19           C
ATOM   8128  O   GLY A1037      11.734   5.254  18.469  1.00 65.19           O
ATOM   8129  N   GLY A1038      11.539   2.926  18.266  1.00 73.19           N
ATOM   8130  CA  GLY A1038      12.945   2.723  17.953  1.00 73.19           C
ATOM   8131  C   GLY A1038      13.281   1.282  17.609  1.00 73.19           C
ATOM   8132  O   GLY A1038      12.398   0.423  17.594  1.00 73.19           O
ATOM   8133  N   VAL A1039      14.461   1.167  17.031  1.00 82.75           N
ATOM   8134  CA  VAL A1039      15.000  -0.126  16.625  1.00 82.75           C
ATOM   8135  C   VAL A1039      15.891  -0.689  17.719  1.00 82.75           C
ATOM   8136  CB  VAL A1039      15.781  -0.020  15.297  1.00 82.75           C
ATOM   8137  O   VAL A1039      16.750   0.020  18.250  1.00 82.75           O
ATOM   8138  CG1 VAL A1039      16.344  -1.382  14.891  1.00 82.75           C
ATOM   8139  CG2 VAL A1039      14.891   0.549  14.195  1.00 82.75           C
ATOM   8140  N   LYS A1040      15.453  -1.877  18.203  1.00 86.81           N
ATOM   8141  CA  LYS A1040      16.281  -2.570  19.172  1.00 86.81           C
ATOM   8142  C   LYS A1040      17.078  -3.693  18.516  1.00 86.81           C
ATOM   8143  CB  LYS A1040      15.430  -3.129  20.312  1.00 86.81           C
ATOM   8144  O   LYS A1040      16.500  -4.582  17.891  1.00 86.81           O
ATOM   8145  CG  LYS A1040      14.750  -2.062  21.156  1.00 86.81           C
ATOM   8146  CD  LYS A1040      13.891  -2.682  22.266  1.00 86.81           C
ATOM   8147  CE  LYS A1040      13.164  -1.616  23.062  1.00 86.81           C
ATOM   8148  NZ  LYS A1040      12.273  -2.221  24.094  1.00 86.81           N
ATOM   8149  N   SER A1041      18.406  -3.531  18.547  1.00 88.50           N
ATOM   8150  CA  SER A1041      19.328  -4.562  18.094  1.00 88.50           C
ATOM   8151  C   SER A1041      19.891  -5.352  19.281  1.00 88.50           C
ATOM   8152  CB  SER A1041      20.469  -3.945  17.297  1.00 88.50           C
ATOM   8153  O   SER A1041      20.531  -4.781  20.156  1.00 88.50           O
ATOM   8154  OG  SER A1041      21.422  -4.934  16.922  1.00 88.50           O
ATOM   8155  N   GLN A1042      19.531  -6.668  19.344  1.00 90.75           N
ATOM   8156  CA  GLN A1042      19.984  -7.469  20.469  1.00 90.75           C
ATOM   8157  C   GLN A1042      20.766  -8.695  20.000  1.00 90.75           C
ATOM   8158  CB  GLN A1042      18.797  -7.898  21.328  1.00 90.75           C
ATOM   8159  O   GLN A1042      20.391  -9.328  19.016  1.00 90.75           O
ATOM   8160  CG  GLN A1042      18.031  -6.734  21.938  1.00 90.75           C
ATOM   8161  CD  GLN A1042      16.828  -7.184  22.750  1.00 90.75           C
ATOM   8162  NE2 GLN A1042      16.703  -6.672  23.969  1.00 90.75           N
ATOM   8163  OE1 GLN A1042      16.016  -7.988  22.281  1.00 90.75           O
ATOM   8164  N   PHE A1043      21.844  -8.992  20.641  1.00 92.81           N
ATOM   8165  CA  PHE A1043      22.641 -10.203  20.516  1.00 92.81           C
ATOM   8166  C   PHE A1043      22.797 -10.898  21.859  1.00 92.81           C
ATOM   8167  CB  PHE A1043      24.031  -9.867  19.938  1.00 92.81           C
ATOM   8168  O   PHE A1043      23.625 -10.508  22.688  1.00 92.81           O
ATOM   8169  CG  PHE A1043      24.719 -11.039  19.312  1.00 92.81           C
ATOM   8170  CD1 PHE A1043      25.953 -10.883  18.688  1.00 92.81           C
ATOM   8171  CD2 PHE A1043      24.141 -12.305  19.328  1.00 92.81           C
ATOM   8172  CE1 PHE A1043      26.609 -11.961  18.094  1.00 92.81           C
ATOM   8173  CE2 PHE A1043      24.781 -13.391  18.750  1.00 92.81           C
ATOM   8174  CZ  PHE A1043      26.016 -13.219  18.125  1.00 92.81           C
ATOM   8175  N   LEU A1044      22.016 -11.953  22.094  1.00 92.38           N
ATOM   8176  CA  LEU A1044      21.906 -12.609  23.406  1.00 92.38           C
ATOM   8177  C   LEU A1044      22.594 -13.969  23.375  1.00 92.38           C
ATOM   8178  CB  LEU A1044      20.438 -12.758  23.797  1.00 92.38           C
ATOM   8179  O   LEU A1044      22.406 -14.742  22.438  1.00 92.38           O
ATOM   8180  CG  LEU A1044      19.594 -11.484  23.766  1.00 92.38           C
ATOM   8181  CD1 LEU A1044      18.109 -11.828  23.984  1.00 92.38           C
ATOM   8182  CD2 LEU A1044      20.078 -10.492  24.812  1.00 92.38           C
ATOM   8183  N   PHE A1045      23.391 -14.273  24.406  1.00 91.81           N
ATOM   8184  CA  PHE A1045      24.047 -15.570  24.594  1.00 91.81           C
ATOM   8185  C   PHE A1045      23.578 -16.234  25.875  1.00 91.81           C
ATOM   8186  CB  PHE A1045      25.562 -15.398  24.625  1.00 91.81           C
ATOM   8187  O   PHE A1045      23.484 -15.594  26.922  1.00 91.81           O
ATOM   8188  CG  PHE A1045      26.156 -14.945  23.312  1.00 91.81           C
```

ATOM	8189	CD1	PHE	A1045	26.562	-15.875	22.359	1.00	91.81	C
ATOM	8190	CD2	PHE	A1045	26.281	-13.594	23.031	1.00	91.81	C
ATOM	8191	CE1	PHE	A1045	27.109	-15.453	21.141	1.00	91.81	C
ATOM	8192	CE2	PHE	A1045	26.828	-13.172	21.812	1.00	91.81	C
ATOM	8193	CZ	PHE	A1045	27.234	-14.102	20.875	1.00	91.81	C
ATOM	8194	N	ASP	A1046	23.094	-17.531	25.812	1.00	90.56	N
ATOM	8195	CA	ASP	A1046	22.875	-18.422	26.938	1.00	90.56	C
ATOM	8196	C	ASP	A1046	23.953	-19.500	27.000	1.00	90.56	C
ATOM	8197	CB	ASP	A1046	21.484	-19.062	26.844	1.00	90.56	C
ATOM	8198	O	ASP	A1046	23.953	-20.422	26.188	1.00	90.56	O
ATOM	8199	CG	ASP	A1046	21.203	-20.016	28.000	1.00	90.56	C
ATOM	8200	OD1	ASP	A1046	21.828	-19.891	29.078	1.00	90.56	O
ATOM	8201	OD2	ASP	A1046	20.359	-20.922	27.828	1.00	90.56	O
ATOM	8202	N	ALA	A1047	24.859	-19.250	27.891	1.00	89.00	N
ATOM	8203	CA	ALA	A1047	25.953	-20.203	28.000	1.00	89.00	C
ATOM	8204	C	ALA	A1047	26.531	-20.219	29.422	1.00	89.00	C
ATOM	8205	CB	ALA	A1047	27.047	-19.875	26.984	1.00	89.00	C
ATOM	8206	O	ALA	A1047	26.609	-19.172	30.078	1.00	89.00	O
ATOM	8207	N	GLY	A1048	26.797	-21.375	30.000	1.00	86.31	N
ATOM	8208	CA	GLY	A1048	27.516	-21.469	31.266	1.00	86.31	C
ATOM	8209	C	GLY	A1048	28.906	-20.859	31.219	1.00	86.31	C
ATOM	8210	O	GLY	A1048	29.375	-20.297	32.219	1.00	86.31	O
ATOM	8211	N	ARG	A1049	29.609	-20.812	30.109	1.00	90.12	N
ATOM	8212	CA	ARG	A1049	30.922	-20.266	29.812	1.00	90.12	C
ATOM	8213	C	ARG	A1049	30.953	-19.625	28.422	1.00	90.12	C
ATOM	8214	CB	ARG	A1049	32.000	-21.344	29.922	1.00	90.12	C
ATOM	8215	O	ARG	A1049	30.578	-20.266	27.438	1.00	90.12	O
ATOM	8216	CG	ARG	A1049	33.406	-20.828	29.688	1.00	90.12	C
ATOM	8217	CD	ARG	A1049	34.438	-21.953	29.797	1.00	90.12	C
ATOM	8218	NE	ARG	A1049	34.531	-22.469	31.172	1.00	90.12	N
ATOM	8219	NH1	ARG	A1049	36.031	-24.141	30.656	1.00	90.12	N
ATOM	8220	NH2	ARG	A1049	35.281	-23.875	32.812	1.00	90.12	N
ATOM	8221	CZ	ARG	A1049	35.281	-23.500	31.547	1.00	90.12	C
ATOM	8222	N	PHE	A1050	31.156	-18.328	28.391	1.00	92.81	N
ATOM	8223	CA	PHE	A1050	31.344	-17.594	27.141	1.00	92.81	C
ATOM	8224	C	PHE	A1050	32.750	-16.984	27.078	1.00	92.81	C
ATOM	8225	CB	PHE	A1050	30.281	-16.484	27.016	1.00	92.81	C
ATOM	8226	O	PHE	A1050	33.188	-16.359	28.031	1.00	92.81	O
ATOM	8227	CG	PHE	A1050	30.453	-15.617	25.797	1.00	92.81	C
ATOM	8228	CD1	PHE	A1050	31.109	-14.398	25.891	1.00	92.81	C
ATOM	8229	CD2	PHE	A1050	29.953	-16.016	24.562	1.00	92.81	C
ATOM	8230	CE1	PHE	A1050	31.281	-13.594	24.766	1.00	92.81	C
ATOM	8231	CE2	PHE	A1050	30.125	-15.219	23.438	1.00	92.81	C
ATOM	8232	CZ	PHE	A1050	30.781	-14.008	23.547	1.00	92.81	C
ATOM	8233	N	ALA	A1051	33.500	-17.281	25.938	1.00	91.19	N
ATOM	8234	CA	ALA	A1051	34.844	-16.750	25.812	1.00	91.19	C
ATOM	8235	C	ALA	A1051	35.125	-16.297	24.375	1.00	91.19	C
ATOM	8236	CB	ALA	A1051	35.875	-17.797	26.250	1.00	91.19	C
ATOM	8237	O	ALA	A1051	34.594	-16.859	23.422	1.00	91.19	O
ATOM	8238	N	ILE	A1052	35.656	-15.086	24.266	1.00	89.06	N
ATOM	8239	CA	ILE	A1052	36.281	-14.688	23.016	1.00	89.06	C
ATOM	8240	C	ILE	A1052	37.719	-15.227	22.953	1.00	89.06	C
ATOM	8241	CB	ILE	A1052	36.281	-13.148	22.844	1.00	89.06	C
ATOM	8242	O	ILE	A1052	38.531	-14.969	23.844	1.00	89.06	O
ATOM	8243	CG1	ILE	A1052	34.844	-12.617	22.844	1.00	89.06	C
ATOM	8244	CG2	ILE	A1052	37.031	-12.742	21.562	1.00	89.06	C
ATOM	8245	CD1	ILE	A1052	34.750	-11.102	22.734	1.00	89.06	C
ATOM	8246	N	ILE	A1053	38.000	-16.078	21.969	1.00	87.00	N
ATOM	8247	CA	ILE	A1053	39.281	-16.766	21.828	1.00	87.00	C
ATOM	8248	C	ILE	A1053	40.031	-16.188	20.656	1.00	87.00	C
ATOM	8249	CB	ILE	A1053	39.094	-18.297	21.656	1.00	87.00	C
ATOM	8250	O	ILE	A1053	39.469	-16.000	19.562	1.00	87.00	O
ATOM	8251	CG1	ILE	A1053	38.219	-18.859	22.797	1.00	87.00	C
ATOM	8252	CG2	ILE	A1053	40.438	-19.000	21.594	1.00	87.00	C

ATOM	8253	CD1	ILE	A1053	37.812	-20.297	22.594	1.00	87.00	C
ATOM	8254	N	ASN	A1054	41.094	-15.656	20.859	1.00	81.88	N
ATOM	8255	CA	ASN	A1054	42.000	-15.203	19.812	1.00	81.88	C
ATOM	8256	C	ASN	A1054	43.125	-16.188	19.578	1.00	81.88	C
ATOM	8257	CB	ASN	A1054	42.531	-13.820	20.141	1.00	81.88	C
ATOM	8258	O	ASN	A1054	43.781	-16.609	20.531	1.00	81.88	O
ATOM	8259	CG	ASN	A1054	43.375	-13.234	19.000	1.00	81.88	C
ATOM	8260	ND2	ASN	A1054	44.094	-12.164	19.297	1.00	81.88	N
ATOM	8261	OD1	ASN	A1054	43.344	-13.734	17.875	1.00	81.88	O
ATOM	8262	N	ASN	A1055	43.188	-16.656	18.344	1.00	78.75	N
ATOM	8263	CA	ASN	A1055	44.281	-17.547	17.922	1.00	78.75	C
ATOM	8264	C	ASN	A1055	45.469	-16.766	17.391	1.00	78.75	C
ATOM	8265	CB	ASN	A1055	43.781	-18.547	16.875	1.00	78.75	C
ATOM	8266	O	ASN	A1055	45.406	-16.219	16.281	1.00	78.75	O
ATOM	8267	CG	ASN	A1055	44.781	-19.641	16.578	1.00	78.75	C
ATOM	8268	ND2	ASN	A1055	44.469	-20.438	15.562	1.00	78.75	N
ATOM	8269	OD1	ASN	A1055	45.781	-19.766	17.266	1.00	78.75	O
ATOM	8270	N	ALA	A1056	46.375	-16.344	18.266	1.00	63.56	N
ATOM	8271	CA	ALA	A1056	47.625	-15.711	17.812	1.00	63.56	C
ATOM	8272	C	ALA	A1056	48.438	-16.656	16.922	1.00	63.56	C
ATOM	8273	CB	ALA	A1056	48.438	-15.281	19.031	1.00	63.56	C
ATOM	8274	O	ALA	A1056	48.281	-17.875	17.016	1.00	63.56	O
ATOM	8275	N	GLN	A1057	48.750	-16.266	15.633	1.00	66.50	N
ATOM	8276	CA	GLN	A1057	49.656	-16.938	14.688	1.00	66.50	C
ATOM	8277	C	GLN	A1057	50.594	-17.906	15.406	1.00	66.50	C
ATOM	8278	CB	GLN	A1057	50.438	-15.922	13.883	1.00	66.50	C
ATOM	8279	O	GLN	A1057	51.000	-18.922	14.844	1.00	66.50	O
ATOM	8280	CG	GLN	A1057	49.750	-15.461	12.602	1.00	66.50	C
ATOM	8281	CD	GLN	A1057	50.250	-14.125	12.109	1.00	66.50	C
ATOM	8282	NE2	GLN	A1057	49.656	-13.633	11.023	1.00	66.50	N
ATOM	8283	OE1	GLN	A1057	51.188	-13.531	12.695	1.00	66.50	O
ATOM	8284	N	SER	A1058	50.812	-17.969	16.781	1.00	64.44	N
ATOM	8285	CA	SER	A1058	51.750	-18.797	17.516	1.00	64.44	C
ATOM	8286	C	SER	A1058	51.062	-19.953	18.219	1.00	64.44	C
ATOM	8287	CB	SER	A1058	52.531	-17.953	18.531	1.00	64.44	C
ATOM	8288	O	SER	A1058	51.719	-20.766	18.891	1.00	64.44	O
ATOM	8289	OG	SER	A1058	51.625	-17.391	19.484	1.00	64.44	O
ATOM	8290	N	GLY	A1059	49.781	-20.344	17.797	1.00	69.62	N
ATOM	8291	CA	GLY	A1059	49.156	-21.484	18.438	1.00	69.62	C
ATOM	8292	C	GLY	A1059	48.719	-21.203	19.859	1.00	69.62	C
ATOM	8293	O	GLY	A1059	48.062	-22.016	20.484	1.00	69.62	O
ATOM	8294	N	ALA	A1060	49.094	-19.906	20.406	1.00	78.12	N
ATOM	8295	CA	ALA	A1060	48.688	-19.594	21.781	1.00	78.12	C
ATOM	8296	C	ALA	A1060	47.312	-18.906	21.797	1.00	78.12	C
ATOM	8297	CB	ALA	A1060	49.719	-18.719	22.469	1.00	78.12	C
ATOM	8298	O	ALA	A1060	47.094	-17.938	21.094	1.00	78.12	O
ATOM	8299	N	PHE	A1061	46.312	-19.594	22.359	1.00	80.38	N
ATOM	8300	CA	PHE	A1061	45.000	-19.031	22.547	1.00	80.38	C
ATOM	8301	C	PHE	A1061	45.000	-18.000	23.672	1.00	80.38	C
ATOM	8302	CB	PHE	A1061	43.969	-20.141	22.859	1.00	80.38	C
ATOM	8303	O	PHE	A1061	45.688	-18.172	24.672	1.00	80.38	O
ATOM	8304	CG	PHE	A1061	43.688	-21.031	21.688	1.00	80.38	C
ATOM	8305	CD1	PHE	A1061	42.812	-20.641	20.688	1.00	80.38	C
ATOM	8306	CD2	PHE	A1061	44.281	-22.281	21.578	1.00	80.38	C
ATOM	8307	CE1	PHE	A1061	42.531	-21.469	19.594	1.00	80.38	C
ATOM	8308	CE2	PHE	A1061	44.031	-23.109	20.500	1.00	80.38	C
ATOM	8309	CZ	PHE	A1061	43.156	-22.703	19.516	1.00	80.38	C
ATOM	8310	N	THR	A1062	44.688	-16.797	23.391	1.00	83.94	N
ATOM	8311	CA	THR	A1062	44.438	-15.812	24.438	1.00	83.94	C
ATOM	8312	C	THR	A1062	42.938	-15.617	24.656	1.00	83.94	C
ATOM	8313	CB	THR	A1062	45.094	-14.461	24.109	1.00	83.94	C
ATOM	8314	O	THR	A1062	42.156	-15.859	23.734	1.00	83.94	O
ATOM	8315	CG2	THR	A1062	46.625	-14.609	23.969	1.00	83.94	C
ATOM	8316	OG1	THR	A1062	44.562	-13.953	22.891	1.00	83.94	O

ATOM	8317	N	LEU	A1063	42.469	-15.344	25.859	1.00	87.88	N
ATOM	8318	CA	LEU	A1063	41.062	-15.117	26.250	1.00	87.88	C
ATOM	8319	C	LEU	A1063	40.875	-13.688	26.750	1.00	87.88	C
ATOM	8320	CB	LEU	A1063	40.656	-16.109	27.344	1.00	87.88	C
ATOM	8321	O	LEU	A1063	40.875	-13.445	27.953	1.00	87.88	O
ATOM	8322	CG	LEU	A1063	40.688	-17.594	26.953	1.00	87.88	C
ATOM	8323	CD1	LEU	A1063	40.781	-18.469	28.203	1.00	87.88	C
ATOM	8324	CD2	LEU	A1063	39.469	-17.953	26.125	1.00	87.88	C
ATOM	8325	N	PRO	A1064	40.875	-12.836	25.828	1.00	87.56	N
ATOM	8326	CA	PRO	A1064	40.719	-11.445	26.250	1.00	87.56	C
ATOM	8327	C	PRO	A1064	39.438	-11.203	27.047	1.00	87.56	C
ATOM	8328	CB	PRO	A1064	40.719	-10.672	24.938	1.00	87.56	C
ATOM	8329	O	PRO	A1064	39.375	-10.258	27.844	1.00	87.56	O
ATOM	8330	CG	PRO	A1064	40.344	-11.680	23.906	1.00	87.56	C
ATOM	8331	CD	PRO	A1064	40.781	-13.039	24.375	1.00	87.56	C
ATOM	8332	N	PHE	A1065	38.375	-12.102	26.875	1.00	91.44	N
ATOM	8333	CA	PHE	A1065	37.062	-11.906	27.500	1.00	91.44	C
ATOM	8334	C	PHE	A1065	36.406	-13.250	27.828	1.00	91.44	C
ATOM	8335	CB	PHE	A1065	36.156	-11.078	26.594	1.00	91.44	C
ATOM	8336	O	PHE	A1065	36.219	-14.086	26.938	1.00	91.44	O
ATOM	8337	CG	PHE	A1065	34.812	-10.781	27.203	1.00	91.44	C
ATOM	8338	CD1	PHE	A1065	33.750	-11.656	27.047	1.00	91.44	C
ATOM	8339	CD2	PHE	A1065	34.594	-9.617	27.938	1.00	91.44	C
ATOM	8340	CE1	PHE	A1065	32.500	-11.383	27.609	1.00	91.44	C
ATOM	8341	CE2	PHE	A1065	33.375	-9.336	28.500	1.00	91.44	C
ATOM	8342	CZ	PHE	A1065	32.312	-10.219	28.344	1.00	91.44	C
ATOM	8343	N	VAL	A1066	36.250	-13.555	29.109	1.00	93.12	N
ATOM	8344	CA	VAL	A1066	35.594	-14.789	29.547	1.00	93.12	C
ATOM	8345	C	VAL	A1066	34.500	-14.461	30.578	1.00	93.12	C
ATOM	8346	CB	VAL	A1066	36.625	-15.781	30.141	1.00	93.12	C
ATOM	8347	O	VAL	A1066	34.719	-13.656	31.484	1.00	93.12	O
ATOM	8348	CG1	VAL	A1066	35.906	-17.047	30.625	1.00	93.12	C
ATOM	8349	CG2	VAL	A1066	37.688	-16.125	29.125	1.00	93.12	C
ATOM	8350	N	VAL	A1067	33.344	-14.977	30.297	1.00	92.56	N
ATOM	8351	CA	VAL	A1067	32.281	-14.961	31.297	1.00	92.56	C
ATOM	8352	C	VAL	A1067	32.094	-16.375	31.859	1.00	92.56	C
ATOM	8353	CB	VAL	A1067	30.969	-14.422	30.719	1.00	92.56	C
ATOM	8354	O	VAL	A1067	31.844	-17.312	31.109	1.00	92.56	O
ATOM	8355	CG1	VAL	A1067	29.891	-14.383	31.812	1.00	92.56	C
ATOM	8356	CG2	VAL	A1067	31.172	-13.039	30.125	1.00	92.56	C
ATOM	8357	N	GLU	A1068	32.344	-16.562	33.062	1.00	91.56	N
ATOM	8358	CA	GLU	A1068	32.219	-17.844	33.750	1.00	91.56	C
ATOM	8359	C	GLU	A1068	31.797	-17.625	35.219	1.00	91.56	C
ATOM	8360	CB	GLU	A1068	33.500	-18.656	33.688	1.00	91.56	C
ATOM	8361	O	GLU	A1068	32.406	-16.812	35.906	1.00	91.56	O
ATOM	8362	CG	GLU	A1068	33.375	-20.062	34.219	1.00	91.56	C
ATOM	8363	CD	GLU	A1068	34.625	-20.922	34.000	1.00	91.56	C
ATOM	8364	OE1	GLU	A1068	34.500	-22.156	34.031	1.00	91.56	O
ATOM	8365	OE2	GLU	A1068	35.688	-20.328	33.750	1.00	91.56	O
ATOM	8366	N	ASN	A1069	30.812	-18.438	35.719	1.00	86.50	N
ATOM	8367	CA	ASN	A1069	30.312	-18.375	37.094	1.00	86.50	C
ATOM	8368	C	ASN	A1069	29.859	-16.969	37.469	1.00	86.50	C
ATOM	8369	CB	ASN	A1069	31.391	-18.875	38.062	1.00	86.50	C
ATOM	8370	O	ASN	A1069	30.219	-16.469	38.531	1.00	86.50	O
ATOM	8371	CG	ASN	A1069	31.609	-20.375	37.969	1.00	86.50	C
ATOM	8372	ND2	ASN	A1069	32.812	-20.812	38.344	1.00	86.50	N
ATOM	8373	OD1	ASN	A1069	30.719	-21.125	37.562	1.00	86.50	O
ATOM	8374	N	ASN	A1070	29.281	-16.250	36.406	1.00	84.31	N
ATOM	8375	CA	ASN	A1070	28.734	-14.914	36.656	1.00	84.31	C
ATOM	8376	C	ASN	A1070	29.828	-13.883	36.875	1.00	84.31	C
ATOM	8377	CB	ASN	A1070	27.766	-14.930	37.844	1.00	84.31	C
ATOM	8378	O	ASN	A1070	29.609	-12.867	37.531	1.00	84.31	O
ATOM	8379	CG	ASN	A1070	26.438	-15.570	37.500	1.00	84.31	C
ATOM	8380	ND2	ASN	A1070	25.703	-16.016	38.531	1.00	84.31	N

ATOM	8381	OD1	ASN	A1070	26.062	-15.672	36.344	1.00	84.31	O
ATOM	8382	N	GLN	A1071	31.078	-14.312	36.500	1.00	88.62	N
ATOM	8383	CA	GLN	A1071	32.219	-13.406	36.594	1.00	88.62	C
ATOM	8384	C	GLN	A1071	32.812	-13.148	35.188	1.00	88.62	C
ATOM	8385	CB	GLN	A1071	33.312	-13.977	37.500	1.00	88.62	C
ATOM	8386	O	GLN	A1071	32.812	-14.039	34.344	1.00	88.62	O
ATOM	8387	CG	GLN	A1071	32.906	-14.000	38.969	1.00	88.62	C
ATOM	8388	CD	GLN	A1071	34.062	-14.398	39.875	1.00	88.62	C
ATOM	8389	NE2	GLN	A1071	34.062	-13.844	41.094	1.00	88.62	N
ATOM	8390	OE1	GLN	A1071	34.906	-15.195	39.500	1.00	88.62	O
ATOM	8391	N	VAL	A1072	33.156	-11.859	34.938	1.00	92.00	N
ATOM	8392	CA	VAL	A1072	33.844	-11.484	33.719	1.00	92.00	C
ATOM	8393	C	VAL	A1072	35.375	-11.430	33.969	1.00	92.00	C
ATOM	8394	CB	VAL	A1072	33.344	-10.125	33.156	1.00	92.00	C
ATOM	8395	O	VAL	A1072	35.812	-10.789	34.906	1.00	92.00	O
ATOM	8396	CG1	VAL	A1072	34.094	-9.750	31.891	1.00	92.00	C
ATOM	8397	CG2	VAL	A1072	31.844	-10.172	32.938	1.00	92.00	C
ATOM	8398	N	PHE	A1073	36.125	-12.242	33.219	1.00	90.56	N
ATOM	8399	CA	PHE	A1073	37.562	-12.234	33.281	1.00	90.56	C
ATOM	8400	C	PHE	A1073	38.156	-11.438	32.125	1.00	90.56	C
ATOM	8401	CB	PHE	A1073	38.125	-13.664	33.250	1.00	90.56	C
ATOM	8402	O	PHE	A1073	37.875	-11.734	30.953	1.00	90.56	O
ATOM	8403	CG	PHE	A1073	37.625	-14.523	34.375	1.00	90.56	C
ATOM	8404	CD1	PHE	A1073	38.344	-14.656	35.562	1.00	90.56	C
ATOM	8405	CD2	PHE	A1073	36.406	-15.203	34.250	1.00	90.56	C
ATOM	8406	CE1	PHE	A1073	37.875	-15.453	36.594	1.00	90.56	C
ATOM	8407	CE2	PHE	A1073	35.938	-16.000	35.312	1.00	90.56	C
ATOM	8408	CZ	PHE	A1073	36.688	-16.125	36.469	1.00	90.56	C
ATOM	8409	N	ILE	A1074	38.844	-10.258	32.375	1.00	91.44	N
ATOM	8410	CA	ILE	A1074	39.500	-9.422	31.375	1.00	91.44	C
ATOM	8411	C	ILE	A1074	41.000	-9.305	31.719	1.00	91.44	C
ATOM	8412	CB	ILE	A1074	38.875	-8.023	31.297	1.00	91.44	C
ATOM	8413	O	ILE	A1074	41.344	-8.859	32.812	1.00	91.44	O
ATOM	8414	CG1	ILE	A1074	37.344	-8.141	31.031	1.00	91.44	C
ATOM	8415	CG2	ILE	A1074	39.531	-7.176	30.219	1.00	91.44	C
ATOM	8416	CD1	ILE	A1074	36.594	-6.828	31.141	1.00	91.44	C
ATOM	8417	N	ASN	A1075	41.938	-9.766	30.844	1.00	85.00	N
ATOM	8418	CA	ASN	A1075	43.406	-9.703	31.047	1.00	85.00	C
ATOM	8419	C	ASN	A1075	43.906	-8.281	30.938	1.00	85.00	C
ATOM	8420	CB	ASN	A1075	44.125	-10.625	30.062	1.00	85.00	C
ATOM	8421	O	ASN	A1075	44.781	-7.859	31.703	1.00	85.00	O
ATOM	8422	CG	ASN	A1075	44.188	-12.062	30.531	1.00	85.00	C
ATOM	8423	ND2	ASN	A1075	44.500	-12.969	29.625	1.00	85.00	N
ATOM	8424	OD1	ASN	A1075	43.906	-12.359	31.703	1.00	85.00	O
ATOM	8425	N	SER	A1076	43.500	-7.469	29.938	1.00	86.44	N
ATOM	8426	CA	SER	A1076	43.906	-6.086	29.703	1.00	86.44	C
ATOM	8427	C	SER	A1076	42.688	-5.219	29.344	1.00	86.44	C
ATOM	8428	CB	SER	A1076	44.938	-6.008	28.578	1.00	86.44	C
ATOM	8429	O	SER	A1076	41.906	-5.578	28.484	1.00	86.44	O
ATOM	8430	OG	SER	A1076	45.375	-4.672	28.375	1.00	86.44	O
ATOM	8431	N	LEU	A1077	42.469	-4.066	30.125	1.00	87.75	N
ATOM	8432	CA	LEU	A1077	41.344	-3.158	29.922	1.00	87.75	C
ATOM	8433	C	LEU	A1077	41.844	-1.718	29.781	1.00	87.75	C
ATOM	8434	CB	LEU	A1077	40.375	-3.268	31.078	1.00	87.75	C
ATOM	8435	O	LEU	A1077	42.656	-1.262	30.578	1.00	87.75	O
ATOM	8436	CG	LEU	A1077	39.188	-2.281	31.062	1.00	87.75	C
ATOM	8437	CD1	LEU	A1077	38.312	-2.521	29.844	1.00	87.75	C
ATOM	8438	CD2	LEU	A1077	38.375	-2.396	32.344	1.00	87.75	C
ATOM	8439	N	LEU	A1078	41.688	-1.037	28.703	1.00	86.44	N
ATOM	8440	CA	LEU	A1078	41.875	0.399	28.531	1.00	86.44	C
ATOM	8441	C	LEU	A1078	40.562	1.144	28.750	1.00	86.44	C
ATOM	8442	CB	LEU	A1078	42.438	0.703	27.141	1.00	86.44	C
ATOM	8443	O	LEU	A1078	39.594	0.933	28.016	1.00	86.44	O
ATOM	8444	CG	LEU	A1078	42.938	2.131	26.906	1.00	86.44	C

ATOM	8445	CD1	LEU	A1078	44.125	2.430	27.781	1.00	86.44	C
ATOM	8446	CD2	LEU	A1078	43.281	2.338	25.422	1.00	86.44	C
ATOM	8447	N	VAL	A1079	40.531	1.979	29.938	1.00	85.56	N
ATOM	8448	CA	VAL	A1079	39.312	2.705	30.266	1.00	85.56	C
ATOM	8449	C	VAL	A1079	39.594	4.203	30.297	1.00	85.56	C
ATOM	8450	CB	VAL	A1079	38.719	2.240	31.625	1.00	85.56	C
ATOM	8451	O	VAL	A1079	40.406	4.672	31.109	1.00	85.56	O
ATOM	8452	CG1	VAL	A1079	37.406	2.932	31.891	1.00	85.56	C
ATOM	8453	CG2	VAL	A1079	38.562	0.722	31.625	1.00	85.56	C
ATOM	8454	N	LYS	A1080	39.062	4.992	29.234	1.00	82.50	N
ATOM	8455	CA	LYS	A1080	39.219	6.445	29.266	1.00	82.50	C
ATOM	8456	C	LYS	A1080	38.562	7.039	30.500	1.00	82.50	C
ATOM	8457	CB	LYS	A1080	38.594	7.066	28.000	1.00	82.50	C
ATOM	8458	O	LYS	A1080	39.188	7.781	31.250	1.00	82.50	O
ATOM	8459	CG	LYS	A1080	38.875	8.555	27.844	1.00	82.50	C
ATOM	8460	CD	LYS	A1080	38.344	9.086	26.516	1.00	82.50	C
ATOM	8461	CE	LYS	A1080	38.625	10.578	26.375	1.00	82.50	C
ATOM	8462	NZ	LYS	A1080	38.125	11.102	25.062	1.00	82.50	N
ATOM	8463	N	ASN	A1081	37.125	6.691	30.672	1.00	79.56	N
ATOM	8464	CA	ASN	A1081	36.312	7.070	31.844	1.00	79.56	C
ATOM	8465	C	ASN	A1081	35.719	5.852	32.531	1.00	79.56	C
ATOM	8466	CB	ASN	A1081	35.219	8.047	31.438	1.00	79.56	C
ATOM	8467	O	ASN	A1081	35.000	5.082	31.922	1.00	79.56	O
ATOM	8468	CG	ASN	A1081	35.750	9.359	30.906	1.00	79.56	C
ATOM	8469	ND2	ASN	A1081	35.125	9.875	29.859	1.00	79.56	N
ATOM	8470	OD1	ASN	A1081	36.719	9.906	31.438	1.00	79.56	O
ATOM	8471	N	GLY	A1082	36.281	5.453	33.750	1.00	79.19	N
ATOM	8472	CA	GLY	A1082	35.781	4.281	34.438	1.00	79.19	C
ATOM	8473	C	GLY	A1082	35.125	4.609	35.781	1.00	79.19	C
ATOM	8474	O	GLY	A1082	35.531	5.574	36.438	1.00	79.19	O
ATOM	8475	N	SER	A1083	33.844	4.074	36.031	1.00	80.00	N
ATOM	8476	CA	SER	A1083	33.219	4.098	37.312	1.00	80.00	C
ATOM	8477	C	SER	A1083	33.375	2.764	38.031	1.00	80.00	C
ATOM	8478	CB	SER	A1083	31.719	4.430	37.188	1.00	80.00	C
ATOM	8479	O	SER	A1083	32.906	1.730	37.562	1.00	80.00	O
ATOM	8480	OG	SER	A1083	31.094	4.477	38.469	1.00	80.00	O
ATOM	8481	N	ILE	A1084	34.219	2.625	39.125	1.00	83.88	N
ATOM	8482	CA	ILE	A1084	34.500	1.415	39.875	1.00	83.88	C
ATOM	8483	C	ILE	A1084	34.000	1.586	41.312	1.00	83.88	C
ATOM	8484	CB	ILE	A1084	36.000	1.076	39.844	1.00	83.88	C
ATOM	8485	O	ILE	A1084	34.406	2.525	42.000	1.00	83.88	O
ATOM	8486	CG1	ILE	A1084	36.500	0.956	38.406	1.00	83.88	C
ATOM	8487	CG2	ILE	A1084	36.281	-0.210	40.625	1.00	83.88	C
ATOM	8488	CD1	ILE	A1084	38.031	0.861	38.281	1.00	83.88	C
ATOM	8489	N	GLY	A1085	32.875	0.717	41.719	1.00	83.06	N
ATOM	8490	CA	GLY	A1085	32.406	0.747	43.094	1.00	83.06	C
ATOM	8491	C	GLY	A1085	33.438	0.259	44.094	1.00	83.06	C
ATOM	8492	O	GLY	A1085	33.781	0.969	45.062	1.00	83.06	O
ATOM	8493	N	ASN	A1086	33.969	-0.971	43.938	1.00	83.12	N
ATOM	8494	CA	ASN	A1086	35.031	-1.568	44.719	1.00	83.12	C
ATOM	8495	C	ASN	A1086	36.156	-2.102	43.844	1.00	83.12	C
ATOM	8496	CB	ASN	A1086	34.500	-2.684	45.594	1.00	83.12	C
ATOM	8497	O	ASN	A1086	35.906	-2.680	42.781	1.00	83.12	O
ATOM	8498	CG	ASN	A1086	33.375	-2.201	46.531	1.00	83.12	C
ATOM	8499	ND2	ASN	A1086	32.281	-2.943	46.594	1.00	83.12	N
ATOM	8500	OD1	ASN	A1086	33.531	-1.171	47.188	1.00	83.12	O
ATOM	8501	N	ALA	A1087	37.438	-1.654	44.156	1.00	86.06	N
ATOM	8502	CA	ALA	A1087	38.562	-2.172	43.375	1.00	86.06	C
ATOM	8503	C	ALA	A1087	39.562	-2.857	44.281	1.00	86.06	C
ATOM	8504	CB	ALA	A1087	39.219	-1.045	42.594	1.00	86.06	C
ATOM	8505	O	ALA	A1087	39.938	-2.326	45.312	1.00	86.06	O
ATOM	8506	N	GLN	A1088	39.781	-4.141	44.000	1.00	86.38	N
ATOM	8507	CA	GLN	A1088	40.938	-4.785	44.594	1.00	86.38	C
ATOM	8508	C	GLN	A1088	42.156	-4.602	43.688	1.00	86.38	C

ATOM	8509	CB	GLN	A1088	40.688	-6.273	44.812	1.00	86.38	C
ATOM	8510	O	GLN	A1088	42.125	-4.934	42.500	1.00	86.38	O
ATOM	8511	CG	GLN	A1088	39.656	-6.555	45.906	1.00	86.38	C
ATOM	8512	CD	GLN	A1088	39.438	-8.039	46.125	1.00	86.38	C
ATOM	8513	NE2	GLN	A1088	40.031	-8.555	47.219	1.00	86.38	N
ATOM	8514	OE1	GLN	A1088	38.781	-8.711	45.344	1.00	86.38	O
ATOM	8515	N	ILE	A1089	43.156	-3.865	44.188	1.00	87.06	N
ATOM	8516	CA	ILE	A1089	44.375	-3.592	43.438	1.00	87.06	C
ATOM	8517	C	ILE	A1089	45.375	-4.719	43.656	1.00	87.06	C
ATOM	8518	CB	ILE	A1089	45.000	-2.234	43.844	1.00	87.06	C
ATOM	8519	O	ILE	A1089	45.812	-4.953	44.781	1.00	87.06	O
ATOM	8520	CG1	ILE	A1089	43.906	-1.138	43.844	1.00	87.06	C
ATOM	8521	CG2	ILE	A1089	46.125	-1.868	42.875	1.00	87.06	C
ATOM	8522	CD1	ILE	A1089	43.250	-0.924	42.500	1.00	87.06	C
ATOM	8523	N	ALA	A1090	45.656	-5.492	42.500	1.00	86.81	N
ATOM	8524	CA	ALA	A1090	46.625	-6.598	42.594	1.00	86.81	C
ATOM	8525	C	ALA	A1090	48.031	-6.090	42.938	1.00	86.81	C
ATOM	8526	CB	ALA	A1090	46.656	-7.410	41.312	1.00	86.81	C
ATOM	8527	O	ALA	A1090	48.656	-6.609	43.844	1.00	86.81	O
ATOM	8528	N	ASN	A1091	48.469	-5.059	42.188	1.00	91.50	N
ATOM	8529	CA	ASN	A1091	49.812	-4.508	42.406	1.00	91.50	C
ATOM	8530	C	ASN	A1091	49.750	-3.025	42.750	1.00	91.50	C
ATOM	8531	CB	ASN	A1091	50.688	-4.723	41.156	1.00	91.50	C
ATOM	8532	O	ASN	A1091	49.969	-2.652	43.906	1.00	91.50	O
ATOM	8533	CG	ASN	A1091	50.969	-6.188	40.906	1.00	91.50	C
ATOM	8534	ND2	ASN	A1091	50.938	-6.574	39.625	1.00	91.50	N
ATOM	8535	OD1	ASN	A1091	51.219	-6.961	41.812	1.00	91.50	O
ATOM	8536	N	PHE	A1092	49.281	-2.090	41.906	1.00	92.50	N
ATOM	8537	CA	PHE	A1092	49.219	-0.649	42.125	1.00	92.50	C
ATOM	8538	C	PHE	A1092	48.188	-0.004	41.188	1.00	92.50	C
ATOM	8539	CB	PHE	A1092	50.594	-0.011	41.906	1.00	92.50	C
ATOM	8540	O	PHE	A1092	47.781	-0.617	40.219	1.00	92.50	O
ATOM	8541	CG	PHE	A1092	51.219	-0.318	40.562	1.00	92.50	C
ATOM	8542	CD1	PHE	A1092	52.062	-1.405	40.406	1.00	92.50	C
ATOM	8543	CD2	PHE	A1092	50.938	0.482	39.469	1.00	92.50	C
ATOM	8544	CE1	PHE	A1092	52.625	-1.691	39.188	1.00	92.50	C
ATOM	8545	CE2	PHE	A1092	51.500	0.203	38.219	1.00	92.50	C
ATOM	8546	CZ	PHE	A1092	52.344	-0.884	38.094	1.00	92.50	C
ATOM	8547	N	ILE	A1093	47.562	1.115	41.656	1.00	93.75	N
ATOM	8548	CA	ILE	A1093	46.875	2.086	40.812	1.00	93.75	C
ATOM	8549	C	ILE	A1093	47.656	3.398	40.812	1.00	93.75	C
ATOM	8550	CB	ILE	A1093	45.438	2.330	41.281	1.00	93.75	C
ATOM	8551	O	ILE	A1093	48.094	3.875	41.844	1.00	93.75	O
ATOM	8552	CG1	ILE	A1093	44.688	3.219	40.312	1.00	93.75	C
ATOM	8553	CG2	ILE	A1093	45.406	2.947	42.688	1.00	93.75	C
ATOM	8554	CD1	ILE	A1093	43.156	3.256	40.500	1.00	93.75	C
ATOM	8555	N	ASN	A1094	47.969	3.916	39.500	1.00	93.69	N
ATOM	8556	CA	ASN	A1094	48.750	5.137	39.406	1.00	93.69	C
ATOM	8557	C	ASN	A1094	48.250	6.031	38.281	1.00	93.69	C
ATOM	8558	CB	ASN	A1094	50.219	4.801	39.219	1.00	93.69	C
ATOM	8559	O	ASN	A1094	47.500	5.586	37.406	1.00	93.69	O
ATOM	8560	CG	ASN	A1094	50.500	4.004	37.969	1.00	93.69	C
ATOM	8561	ND2	ASN	A1094	51.688	3.418	37.875	1.00	93.69	N
ATOM	8562	OD1	ASN	A1094	49.625	3.920	37.062	1.00	93.69	O
ATOM	8563	N	SER	A1095	48.500	7.324	38.531	1.00	93.06	N
ATOM	8564	CA	SER	A1095	48.250	8.281	37.438	1.00	93.06	C
ATOM	8565	C	SER	A1095	49.125	7.984	36.250	1.00	93.06	C
ATOM	8566	CB	SER	A1095	48.500	9.711	37.938	1.00	93.06	C
ATOM	8567	O	SER	A1095	50.188	7.367	36.375	1.00	93.06	O
ATOM	8568	OG	SER	A1095	49.844	9.875	38.344	1.00	93.06	O
ATOM	8569	N	ASN	A1096	48.688	8.281	34.969	1.00	90.94	N
ATOM	8570	CA	ASN	A1096	49.375	7.988	33.719	1.00	90.94	C
ATOM	8571	C	ASN	A1096	50.750	8.688	33.656	1.00	90.94	C
ATOM	8572	CB	ASN	A1096	48.531	8.375	32.500	1.00	90.94	C

ATOM	8573	O	ASN	A1096	51.625	8.266	32.938	1.00	90.94	O
ATOM	8574	CG	ASN	A1096	48.312	9.875	32.406	1.00	90.94	C
ATOM	8575	ND2	ASN	A1096	47.781	10.320	31.281	1.00	90.94	N
ATOM	8576	OD1	ASN	A1096	48.656	10.625	33.312	1.00	90.94	O
ATOM	8577	N	ASN	A1097	50.969	9.852	34.562	1.00	93.00	N
ATOM	8578	CA	ASN	A1097	52.250	10.578	34.594	1.00	93.00	C
ATOM	8579	C	ASN	A1097	53.062	10.219	35.844	1.00	93.00	C
ATOM	8580	CB	ASN	A1097	52.000	12.086	34.562	1.00	93.00	C
ATOM	8581	O	ASN	A1097	53.969	10.945	36.219	1.00	93.00	O
ATOM	8582	CG	ASN	A1097	51.281	12.609	35.781	1.00	93.00	C
ATOM	8583	ND2	ASN	A1097	51.375	13.906	36.031	1.00	93.00	N
ATOM	8584	OD1	ASN	A1097	50.594	11.852	36.469	1.00	93.00	O
ATOM	8585	N	TRP	A1098	52.625	9.016	36.594	1.00	92.81	N
ATOM	8586	CA	TRP	A1098	53.312	8.664	37.844	1.00	92.81	C
ATOM	8587	C	TRP	A1098	54.781	8.352	37.625	1.00	92.81	C
ATOM	8588	CB	TRP	A1098	52.625	7.465	38.500	1.00	92.81	C
ATOM	8589	O	TRP	A1098	55.125	7.555	36.719	1.00	92.81	O
ATOM	8590	CG	TRP	A1098	53.312	6.969	39.750	1.00	92.81	C
ATOM	8591	CD1	TRP	A1098	53.312	7.559	40.969	1.00	92.81	C
ATOM	8592	CD2	TRP	A1098	54.094	5.781	39.844	1.00	92.81	C
ATOM	8593	CE2	TRP	A1098	54.562	5.711	41.188	1.00	92.81	C
ATOM	8594	CE3	TRP	A1098	54.469	4.762	38.969	1.00	92.81	C
ATOM	8595	NE1	TRP	A1098	54.062	6.809	41.844	1.00	92.81	N
ATOM	8596	CH2	TRP	A1098	55.688	3.684	40.750	1.00	92.81	C
ATOM	8597	CZ2	TRP	A1098	55.344	4.664	41.656	1.00	92.81	C
ATOM	8598	CZ3	TRP	A1098	55.250	3.723	39.438	1.00	92.81	C
ATOM	8599	N	GLN	A1099	55.562	9.023	38.219	1.00	92.44	N
ATOM	8600	CA	GLN	A1099	57.000	8.852	38.406	1.00	92.44	C
ATOM	8601	C	GLN	A1099	57.375	8.969	39.875	1.00	92.44	C
ATOM	8602	CB	GLN	A1099	57.781	9.875	37.562	1.00	92.44	C
ATOM	8603	O	GLN	A1099	57.219	10.023	40.469	1.00	92.44	O
ATOM	8604	CG	GLN	A1099	57.719	9.633	36.062	1.00	92.44	C
ATOM	8605	CD	GLN	A1099	58.531	10.641	35.281	1.00	92.44	C
ATOM	8606	NE2	GLN	A1099	58.562	10.469	33.969	1.00	92.44	N
ATOM	8607	OE1	GLN	A1099	59.125	11.570	35.844	1.00	92.44	O
ATOM	8608	N	SER	A1100	57.844	7.926	40.500	1.00	93.50	N
ATOM	8609	CA	SER	A1100	58.188	7.887	41.938	1.00	93.50	C
ATOM	8610	C	SER	A1100	59.031	9.102	42.312	1.00	93.50	C
ATOM	8611	CB	SER	A1100	58.969	6.602	42.250	1.00	93.50	C
ATOM	8612	O	SER	A1100	60.031	9.398	41.688	1.00	93.50	O
ATOM	8613	OG	SER	A1100	59.219	6.512	43.625	1.00	93.50	O
ATOM	8614	N	GLY	A1101	58.500	9.875	43.281	1.00	90.19	N
ATOM	8615	CA	GLY	A1101	59.250	11.000	43.844	1.00	90.19	C
ATOM	8616	C	GLY	A1101	59.219	12.227	42.938	1.00	90.19	C
ATOM	8617	O	GLY	A1101	59.812	13.250	43.281	1.00	90.19	O
ATOM	8618	N	VAL	A1102	58.688	12.164	41.688	1.00	92.62	N
ATOM	8619	CA	VAL	A1102	58.812	13.242	40.719	1.00	92.62	C
ATOM	8620	C	VAL	A1102	57.406	13.789	40.375	1.00	92.62	C
ATOM	8621	CB	VAL	A1102	59.500	12.781	39.406	1.00	92.62	C
ATOM	8622	O	VAL	A1102	57.156	14.984	40.531	1.00	92.62	O
ATOM	8623	CG1	VAL	A1102	59.594	13.930	38.406	1.00	92.62	C
ATOM	8624	CG2	VAL	A1102	60.875	12.211	39.719	1.00	92.62	C
ATOM	8625	N	ALA	A1103	56.500	13.031	39.969	1.00	93.25	N
ATOM	8626	CA	ALA	A1103	55.188	13.438	39.500	1.00	93.25	C
ATOM	8627	C	ALA	A1103	54.156	12.312	39.656	1.00	93.25	C
ATOM	8628	CB	ALA	A1103	55.250	13.883	38.031	1.00	93.25	C
ATOM	8629	O	ALA	A1103	54.531	11.141	39.719	1.00	93.25	O
ATOM	8630	N	GLY	A1104	52.938	12.758	39.906	1.00	94.44	N
ATOM	8631	CA	GLY	A1104	51.844	11.828	39.906	1.00	94.44	C
ATOM	8632	C	GLY	A1104	51.531	11.234	41.250	1.00	94.44	C
ATOM	8633	O	GLY	A1104	51.969	11.781	42.281	1.00	94.44	O
ATOM	8634	N	TRP	A1105	50.625	10.297	41.281	1.00	94.94	N
ATOM	8635	CA	TRP	A1105	50.281	9.594	42.531	1.00	94.94	C
ATOM	8636	C	TRP	A1105	50.219	8.086	42.281	1.00	94.94	C

ATOM	8637	CB	TRP	A1105	48.938	10.094	43.062	1.00	94.94	C
ATOM	8638	O	TRP	A1105	50.000	7.637	41.156	1.00	94.94	O
ATOM	8639	CG	TRP	A1105	47.812	10.016	42.094	1.00	94.94	C
ATOM	8640	CD1	TRP	A1105	47.406	11.000	41.219	1.00	94.94	C
ATOM	8641	CD2	TRP	A1105	46.969	8.898	41.844	1.00	94.94	C
ATOM	8642	CE2	TRP	A1105	46.031	9.266	40.844	1.00	94.94	C
ATOM	8643	CE3	TRP	A1105	46.875	7.609	42.406	1.00	94.94	C
ATOM	8644	NE1	TRP	A1105	46.312	10.555	40.500	1.00	94.94	N
ATOM	8645	CH2	TRP	A1105	45.000	7.148	40.938	1.00	94.94	C
ATOM	8646	CZ2	TRP	A1105	45.031	8.398	40.375	1.00	94.94	C
ATOM	8647	CZ3	TRP	A1105	45.875	6.746	41.938	1.00	94.94	C
ATOM	8648	N	ALA	A1106	50.594	7.262	43.219	1.00	94.94	N
ATOM	8649	CA	ALA	A1106	50.469	5.809	43.219	1.00	94.94	C
ATOM	8650	C	ALA	A1106	50.000	5.285	44.562	1.00	94.94	C
ATOM	8651	CB	ALA	A1106	51.812	5.172	42.844	1.00	94.94	C
ATOM	8652	O	ALA	A1106	50.438	5.777	45.594	1.00	94.94	O
ATOM	8653	N	ILE	A1107	49.000	4.359	44.531	1.00	94.25	N
ATOM	8654	CA	ILE	A1107	48.562	3.607	45.688	1.00	94.25	C
ATOM	8655	C	ILE	A1107	48.938	2.133	45.531	1.00	94.25	C
ATOM	8656	CB	ILE	A1107	47.062	3.768	45.938	1.00	94.25	C
ATOM	8657	O	ILE	A1107	48.406	1.455	44.656	1.00	94.25	O
ATOM	8658	CG1	ILE	A1107	46.656	5.250	45.969	1.00	94.25	C
ATOM	8659	CG2	ILE	A1107	46.625	3.049	47.219	1.00	94.25	C
ATOM	8660	CD1	ILE	A1107	45.156	5.504	46.031	1.00	94.25	C
ATOM	8661	N	ASN	A1108	49.750	1.637	46.312	1.00	92.44	N
ATOM	8662	CA	ASN	A1108	50.250	0.262	46.219	1.00	92.44	C
ATOM	8663	C	ASN	A1108	49.406	-0.679	47.094	1.00	92.44	C
ATOM	8664	CB	ASN	A1108	51.719	0.182	46.594	1.00	92.44	C
ATOM	8665	O	ASN	A1108	48.812	-0.252	48.062	1.00	92.44	O
ATOM	8666	CG	ASN	A1108	52.625	0.886	45.625	1.00	92.44	C
ATOM	8667	ND2	ASN	A1108	53.062	2.084	45.969	1.00	92.44	N
ATOM	8668	OD1	ASN	A1108	52.906	0.356	44.531	1.00	92.44	O
ATOM	8669	N	LYS	A1109	49.344	-1.973	46.625	1.00	92.38	N
ATOM	8670	CA	LYS	A1109	48.594	-2.980	47.375	1.00	92.38	C
ATOM	8671	C	LYS	A1109	49.125	-3.180	48.781	1.00	92.38	C
ATOM	8672	CB	LYS	A1109	48.594	-4.309	46.594	1.00	92.38	C
ATOM	8673	O	LYS	A1109	48.438	-3.668	49.656	1.00	92.38	O
ATOM	8674	CG	LYS	A1109	49.938	-5.008	46.594	1.00	92.38	C
ATOM	8675	CD	LYS	A1109	49.875	-6.359	45.906	1.00	92.38	C
ATOM	8676	CE	LYS	A1109	51.219	-7.078	45.938	1.00	92.38	C
ATOM	8677	NZ	LYS	A1109	51.031	-8.562	45.969	1.00	92.38	N
ATOM	8678	N	ASP	A1110	50.406	-2.811	49.062	1.00	91.44	N
ATOM	8679	CA	ASP	A1110	51.062	-3.020	50.344	1.00	91.44	C
ATOM	8680	C	ASP	A1110	50.750	-1.884	51.344	1.00	91.44	C
ATOM	8681	CB	ASP	A1110	52.562	-3.146	50.188	1.00	91.44	C
ATOM	8682	O	ASP	A1110	51.250	-1.867	52.469	1.00	91.44	O
ATOM	8683	CG	ASP	A1110	53.188	-1.925	49.531	1.00	91.44	C
ATOM	8684	OD1	ASP	A1110	52.438	-0.988	49.156	1.00	91.44	O
ATOM	8685	OD2	ASP	A1110	54.438	-1.901	49.375	1.00	91.44	O
ATOM	8686	N	GLY	A1111	49.938	-0.890	50.875	1.00	88.25	N
ATOM	8687	CA	GLY	A1111	49.562	0.217	51.719	1.00	88.25	C
ATOM	8688	C	GLY	A1111	50.375	1.473	51.500	1.00	88.25	C
ATOM	8689	O	GLY	A1111	50.062	2.537	52.031	1.00	88.25	O
ATOM	8690	N	TYR	A1112	51.531	1.279	50.750	1.00	90.25	N
ATOM	8691	CA	TYR	A1112	52.344	2.432	50.375	1.00	90.25	C
ATOM	8692	C	TYR	A1112	51.594	3.316	49.375	1.00	90.25	C
ATOM	8693	CB	TYR	A1112	53.656	1.979	49.781	1.00	90.25	C
ATOM	8694	O	TYR	A1112	51.031	2.820	48.375	1.00	90.25	O
ATOM	8695	CG	TYR	A1112	54.594	3.117	49.438	1.00	90.25	C
ATOM	8696	CD1	TYR	A1112	54.719	3.553	48.125	1.00	90.25	C
ATOM	8697	CD2	TYR	A1112	55.344	3.762	50.406	1.00	90.25	C
ATOM	8698	CE1	TYR	A1112	55.562	4.602	47.781	1.00	90.25	C
ATOM	8699	CE2	TYR	A1112	56.188	4.812	50.062	1.00	90.25	C
ATOM	8700	OH	TYR	A1112	57.125	6.262	48.438	1.00	90.25	O

ATOM	8701	CZ	TYR	A1112	56.281	5.223	48.750	1.00	90.25	C
ATOM	8702	N	ALA	A1113	51.469	4.621	49.688	1.00	93.12	N
ATOM	8703	CA	ALA	A1113	50.844	5.582	48.781	1.00	93.12	C
ATOM	8704	C	ALA	A1113	51.719	6.828	48.625	1.00	93.12	C
ATOM	8705	CB	ALA	A1113	49.438	5.965	49.281	1.00	93.12	C
ATOM	8706	O	ALA	A1113	52.344	7.277	49.594	1.00	93.12	O
ATOM	8707	N	GLU	A1114	51.938	7.234	47.312	1.00	94.06	N
ATOM	8708	CA	GLU	A1114	52.656	8.469	47.000	1.00	94.06	C
ATOM	8709	C	GLU	A1114	51.750	9.453	46.281	1.00	94.06	C
ATOM	8710	CB	GLU	A1114	53.906	8.180	46.188	1.00	94.06	C
ATOM	8711	O	GLU	A1114	51.156	9.102	45.250	1.00	94.06	O
ATOM	8712	CG	GLU	A1114	54.750	9.406	45.906	1.00	94.06	C
ATOM	8713	CD	GLU	A1114	56.000	9.102	45.094	1.00	94.06	C
ATOM	8714	OE1	GLU	A1114	56.562	10.031	44.469	1.00	94.06	O
ATOM	8715	OE2	GLU	A1114	56.406	7.918	45.062	1.00	94.06	O
ATOM	8716	N	PHE	A1115	51.562	10.641	46.875	1.00	94.06	N
ATOM	8717	CA	PHE	A1115	50.844	11.742	46.250	1.00	94.06	C
ATOM	8718	C	PHE	A1115	51.750	12.961	46.094	1.00	94.06	C
ATOM	8719	CB	PHE	A1115	49.625	12.094	47.094	1.00	94.06	C
ATOM	8720	O	PHE	A1115	52.250	13.484	47.094	1.00	94.06	O
ATOM	8721	CG	PHE	A1115	48.562	11.016	47.125	1.00	94.06	C
ATOM	8722	CD1	PHE	A1115	47.562	10.977	46.188	1.00	94.06	C
ATOM	8723	CD2	PHE	A1115	48.594	10.031	48.094	1.00	94.06	C
ATOM	8724	CE1	PHE	A1115	46.594	9.977	46.219	1.00	94.06	C
ATOM	8725	CE2	PHE	A1115	47.656	9.023	48.125	1.00	94.06	C
ATOM	8726	CZ	PHE	A1115	46.625	9.000	47.188	1.00	94.06	C
ATOM	8727	N	ASN	A1116	52.031	13.398	44.812	1.00	91.62	N
ATOM	8728	CA	ASN	A1116	52.688	14.688	44.594	1.00	91.62	C
ATOM	8729	C	ASN	A1116	51.688	15.836	44.625	1.00	91.62	C
ATOM	8730	CB	ASN	A1116	53.406	14.672	43.250	1.00	91.62	C
ATOM	8731	O	ASN	A1116	50.719	15.859	43.875	1.00	91.62	O
ATOM	8732	CG	ASN	A1116	54.812	14.062	43.375	1.00	91.62	C
ATOM	8733	ND2	ASN	A1116	55.062	13.031	42.562	1.00	91.62	N
ATOM	8734	OD1	ASN	A1116	55.625	14.516	44.156	1.00	91.62	O
ATOM	8735	N	GLN	A1117	51.625	16.703	45.750	1.00	87.38	N
ATOM	8736	CA	GLN	A1117	50.750	17.859	45.906	1.00	87.38	C
ATOM	8737	C	GLN	A1117	49.406	17.453	46.469	1.00	87.38	C
ATOM	8738	CB	GLN	A1117	50.594	18.578	44.562	1.00	87.38	C
ATOM	8739	O	GLN	A1117	48.375	17.766	45.875	1.00	87.38	O
ATOM	8740	CG	GLN	A1117	51.875	19.141	44.000	1.00	87.38	C
ATOM	8741	CD	GLN	A1117	52.219	20.516	44.562	1.00	87.38	C
ATOM	8742	NE2	GLN	A1117	53.406	21.000	44.250	1.00	87.38	N
ATOM	8743	OE1	GLN	A1117	51.406	21.109	45.281	1.00	87.38	O
ATOM	8744	N	ILE	A1118	49.406	16.781	47.719	1.00	90.19	N
ATOM	8745	CA	ILE	A1118	48.188	16.250	48.344	1.00	90.19	C
ATOM	8746	C	ILE	A1118	47.562	17.297	49.250	1.00	90.19	C
ATOM	8747	CB	ILE	A1118	48.500	14.953	49.125	1.00	90.19	C
ATOM	8748	O	ILE	A1118	48.281	18.078	49.906	1.00	90.19	O
ATOM	8749	CG1	ILE	A1118	47.188	14.375	49.719	1.00	90.19	C
ATOM	8750	CG2	ILE	A1118	49.500	15.234	50.250	1.00	90.19	C
ATOM	8751	CD1	ILE	A1118	47.312	12.945	50.219	1.00	90.19	C
ATOM	8752	N	THR	A1119	46.281	17.578	49.125	1.00	88.19	N
ATOM	8753	CA	THR	A1119	45.438	18.281	50.094	1.00	88.19	C
ATOM	8754	C	THR	A1119	44.656	17.281	50.969	1.00	88.19	C
ATOM	8755	CB	THR	A1119	44.500	19.266	49.438	1.00	88.19	C
ATOM	8756	O	THR	A1119	43.906	16.484	50.438	1.00	88.19	O
ATOM	8757	CG2	THR	A1119	43.688	20.047	50.469	1.00	88.19	C
ATOM	8758	OG1	THR	A1119	45.250	20.188	48.625	1.00	88.19	O
ATOM	8759	N	VAL	A1120	44.938	17.234	52.344	1.00	89.19	N
ATOM	8760	CA	VAL	A1120	44.250	16.344	53.250	1.00	89.19	C
ATOM	8761	C	VAL	A1120	43.312	17.156	54.156	1.00	89.19	C
ATOM	8762	CB	VAL	A1120	45.219	15.500	54.094	1.00	89.19	C
ATOM	8763	O	VAL	A1120	43.781	18.094	54.812	1.00	89.19	O
ATOM	8764	CG1	VAL	A1120	44.469	14.516	54.969	1.00	89.19	C

ATOM	8765	CG2	VAL	A1120	46.188	14.758	53.156	1.00	89.19	C
ATOM	8766	N	ARG	A1121	42.000	16.984	54.156	1.00	89.25	N
ATOM	8767	CA	ARG	A1121	41.031	17.578	55.062	1.00	89.25	C
ATOM	8768	C	ARG	A1121	40.594	16.578	56.125	1.00	89.25	C
ATOM	8769	CB	ARG	A1121	39.812	18.094	54.312	1.00	89.25	C
ATOM	8770	O	ARG	A1121	40.281	15.430	55.812	1.00	89.25	O
ATOM	8771	CG	ARG	A1121	40.125	19.234	53.344	1.00	89.25	C
ATOM	8772	CD	ARG	A1121	38.875	19.781	52.688	1.00	89.25	C
ATOM	8773	NE	ARG	A1121	39.156	20.891	51.781	1.00	89.25	N
ATOM	8774	NH1	ARG	A1121	38.094	19.969	49.969	1.00	89.25	N
ATOM	8775	NH2	ARG	A1121	39.125	22.016	49.781	1.00	89.25	N
ATOM	8776	CZ	ARG	A1121	38.781	20.953	50.531	1.00	89.25	C
ATOM	8777	N	GLY	A1122	40.781	16.906	57.500	1.00	86.44	N
ATOM	8778	CA	GLY	A1122	40.500	16.000	58.594	1.00	86.44	C
ATOM	8779	C	GLY	A1122	41.719	15.734	59.469	1.00	86.44	C
ATOM	8780	O	GLY	A1122	42.656	16.531	59.500	1.00	86.44	O
ATOM	8781	N	THR	A1123	41.438	14.664	60.406	1.00	87.31	N
ATOM	8782	CA	THR	A1123	42.500	14.250	61.344	1.00	87.31	C
ATOM	8783	C	THR	A1123	43.562	13.414	60.625	1.00	87.31	C
ATOM	8784	CB	THR	A1123	41.906	13.445	62.500	1.00	87.31	C
ATOM	8785	O	THR	A1123	43.219	12.500	59.875	1.00	87.31	O
ATOM	8786	CG2	THR	A1123	43.000	13.102	63.531	1.00	87.31	C
ATOM	8787	OG1	THR	A1123	40.906	14.227	63.156	1.00	87.31	O
ATOM	8788	N	VAL	A1124	44.812	13.758	60.719	1.00	87.94	N
ATOM	8789	CA	VAL	A1124	45.938	13.016	60.188	1.00	87.94	C
ATOM	8790	C	VAL	A1124	46.656	12.273	61.281	1.00	87.94	C
ATOM	8791	CB	VAL	A1124	46.906	13.953	59.438	1.00	87.94	C
ATOM	8792	O	VAL	A1124	47.031	12.867	62.312	1.00	87.94	O
ATOM	8793	CG1	VAL	A1124	48.094	13.164	58.906	1.00	87.94	C
ATOM	8794	CG2	VAL	A1124	46.188	14.672	58.281	1.00	87.94	C
ATOM	8795	N	TYR	A1125	46.656	10.797	61.188	1.00	87.19	N
ATOM	8796	CA	TYR	A1125	47.469	9.961	62.062	1.00	87.19	C
ATOM	8797	C	TYR	A1125	48.781	9.570	61.406	1.00	87.19	C
ATOM	8798	CB	TYR	A1125	46.688	8.695	62.469	1.00	87.19	C
ATOM	8799	O	TYR	A1125	48.781	8.945	60.344	1.00	87.19	O
ATOM	8800	CG	TYR	A1125	45.469	8.977	63.344	1.00	87.19	C
ATOM	8801	CD1	TYR	A1125	45.594	9.055	64.750	1.00	87.19	C
ATOM	8802	CD2	TYR	A1125	44.219	9.156	62.781	1.00	87.19	C
ATOM	8803	CE1	TYR	A1125	44.500	9.305	65.500	1.00	87.19	C
ATOM	8804	CE2	TYR	A1125	43.094	9.406	63.562	1.00	87.19	C
ATOM	8805	OH	TYR	A1125	42.156	9.734	65.750	1.00	87.19	O
ATOM	8806	CZ	TYR	A1125	43.250	9.484	64.938	1.00	87.19	C
ATOM	8807	N	ALA	A1126	49.844	10.148	61.812	1.00	85.62	N
ATOM	8808	CA	ALA	A1126	51.188	9.805	61.281	1.00	85.62	C
ATOM	8809	C	ALA	A1126	52.125	9.367	62.406	1.00	85.62	C
ATOM	8810	CB	ALA	A1126	51.781	10.984	60.500	1.00	85.62	C
ATOM	8811	O	ALA	A1126	52.094	9.930	63.500	1.00	85.62	O
ATOM	8812	N	ASN	A1127	52.719	8.055	62.156	1.00	85.88	N
ATOM	8813	CA	ASN	A1127	53.688	7.566	63.125	1.00	85.88	C
ATOM	8814	C	ASN	A1127	54.969	8.406	63.094	1.00	85.88	C
ATOM	8815	CB	ASN	A1127	54.031	6.098	62.844	1.00	85.88	C
ATOM	8816	O	ASN	A1127	55.688	8.492	64.062	1.00	85.88	O
ATOM	8817	CG	ASN	A1127	52.875	5.164	63.188	1.00	85.88	C
ATOM	8818	ND2	ASN	A1127	52.812	4.027	62.500	1.00	85.88	N
ATOM	8819	OD1	ASN	A1127	52.062	5.465	64.062	1.00	85.88	O
ATOM	8820	N	ALA	A1128	55.312	9.070	61.844	1.00	86.44	N
ATOM	8821	CA	ALA	A1128	56.469	9.930	61.594	1.00	86.44	C
ATOM	8822	C	ALA	A1128	56.250	10.812	60.375	1.00	86.44	C
ATOM	8823	CB	ALA	A1128	57.750	9.086	61.438	1.00	86.44	C
ATOM	8824	O	ALA	A1128	55.438	10.484	59.500	1.00	86.44	O
ATOM	8825	N	GLY	A1129	56.719	12.086	60.375	1.00	85.56	N
ATOM	8826	CA	GLY	A1129	56.656	12.969	59.219	1.00	85.56	C
ATOM	8827	C	GLY	A1129	57.531	14.180	59.344	1.00	85.56	C
ATOM	8828	O	GLY	A1129	58.188	14.391	60.375	1.00	85.56	O

ATOM	8829	N	SER	A1130	58.031	14.727	58.125	1.00	83.31	N
ATOM	8830	CA	SER	A1130	58.750	15.992	58.000	1.00	83.31	C
ATOM	8831	C	SER	A1130	57.906	17.047	57.312	1.00	83.31	C
ATOM	8832	CB	SER	A1130	60.062	15.797	57.250	1.00	83.31	C
ATOM	8833	O	SER	A1130	57.250	16.766	56.344	1.00	83.31	O
ATOM	8834	OG	SER	A1130	60.719	17.047	57.062	1.00	83.31	O
ATOM	8835	N	PHE	A1131	57.562	18.141	58.031	1.00	83.12	N
ATOM	8836	CA	PHE	A1131	56.875	19.297	57.469	1.00	83.12	C
ATOM	8837	C	PHE	A1131	57.875	20.391	57.094	1.00	83.12	C
ATOM	8838	CB	PHE	A1131	55.844	19.828	58.469	1.00	83.12	C
ATOM	8839	O	PHE	A1131	58.688	20.797	57.906	1.00	83.12	O
ATOM	8840	CG	PHE	A1131	54.781	18.828	58.812	1.00	83.12	C
ATOM	8841	CD1	PHE	A1131	53.594	18.766	58.062	1.00	83.12	C
ATOM	8842	CD2	PHE	A1131	54.938	17.984	59.906	1.00	83.12	C
ATOM	8843	CE1	PHE	A1131	52.594	17.844	58.406	1.00	83.12	C
ATOM	8844	CE2	PHE	A1131	53.938	17.062	60.219	1.00	83.12	C
ATOM	8845	CZ	PHE	A1131	52.781	17.000	59.469	1.00	83.12	C
ATOM	8846	N	THR	A1132	58.000	20.688	55.719	1.00	83.12	N
ATOM	8847	CA	THR	A1132	58.781	21.828	55.250	1.00	83.12	C
ATOM	8848	C	THR	A1132	57.844	23.000	54.906	1.00	83.12	C
ATOM	8849	CB	THR	A1132	59.625	21.469	54.031	1.00	83.12	C
ATOM	8850	O	THR	A1132	56.844	22.812	54.219	1.00	83.12	O
ATOM	8851	CG2	THR	A1132	60.781	20.562	54.406	1.00	83.12	C
ATOM	8852	OG1	THR	A1132	58.812	20.797	53.062	1.00	83.12	O
ATOM	8853	N	GLY	A1133	57.656	23.969	55.875	1.00	85.00	N
ATOM	8854	CA	GLY	A1133	56.812	25.141	55.688	1.00	85.00	C
ATOM	8855	C	GLY	A1133	56.125	25.594	56.969	1.00	85.00	C
ATOM	8856	O	GLY	A1133	56.719	25.594	58.031	1.00	85.00	O
ATOM	8857	N	ASN	A1134	54.938	26.422	56.750	1.00	83.25	N
ATOM	8858	CA	ASN	A1134	54.188	27.000	57.844	1.00	83.25	C
ATOM	8859	C	ASN	A1134	53.125	26.031	58.375	1.00	83.25	C
ATOM	8860	CB	ASN	A1134	53.531	28.312	57.406	1.00	83.25	C
ATOM	8861	O	ASN	A1134	52.469	25.344	57.594	1.00	83.25	O
ATOM	8862	CG	ASN	A1134	54.531	29.406	57.125	1.00	83.25	C
ATOM	8863	ND2	ASN	A1134	54.062	30.531	56.625	1.00	83.25	N
ATOM	8864	OD1	ASN	A1134	55.750	29.234	57.375	1.00	83.25	O
ATOM	8865	N	VAL	A1135	53.156	25.672	59.594	1.00	85.44	N
ATOM	8866	CA	VAL	A1135	52.094	24.953	60.281	1.00	85.44	C
ATOM	8867	C	VAL	A1135	51.188	25.953	61.031	1.00	85.44	C
ATOM	8868	CB	VAL	A1135	52.656	23.906	61.281	1.00	85.44	C
ATOM	8869	O	VAL	A1135	51.688	26.734	61.844	1.00	85.44	O
ATOM	8870	CG1	VAL	A1135	51.531	23.188	62.000	1.00	85.44	C
ATOM	8871	CG2	VAL	A1135	53.531	22.906	60.531	1.00	85.44	C
ATOM	8872	N	TYR	A1136	49.844	26.172	60.562	1.00	82.56	N
ATOM	8873	CA	TYR	A1136	48.844	26.969	61.281	1.00	82.56	C
ATOM	8874	C	TYR	A1136	47.969	26.094	62.156	1.00	82.56	C
ATOM	8875	CB	TYR	A1136	47.969	27.734	60.281	1.00	82.56	C
ATOM	8876	O	TYR	A1136	47.219	25.250	61.688	1.00	82.56	O
ATOM	8877	CG	TYR	A1136	48.719	28.781	59.469	1.00	82.56	C
ATOM	8878	CD1	TYR	A1136	48.906	30.062	59.969	1.00	82.56	C
ATOM	8879	CD2	TYR	A1136	49.219	28.484	58.219	1.00	82.56	C
ATOM	8880	CE1	TYR	A1136	49.594	31.016	59.250	1.00	82.56	C
ATOM	8881	CE2	TYR	A1136	49.906	29.438	57.469	1.00	82.56	C
ATOM	8882	OH	TYR	A1136	50.781	31.656	57.281	1.00	82.56	O
ATOM	8883	CZ	TYR	A1136	50.094	30.703	58.000	1.00	82.56	C
ATOM	8884	N	ALA	A1137	48.156	26.062	63.469	1.00	83.31	N
ATOM	8885	CA	ALA	A1137	47.406	25.250	64.438	1.00	83.31	C
ATOM	8886	C	ALA	A1137	46.906	26.109	65.562	1.00	83.31	C
ATOM	8887	CB	ALA	A1137	48.250	24.094	64.938	1.00	83.31	C
ATOM	8888	O	ALA	A1137	47.562	27.078	65.938	1.00	83.31	O
ATOM	8889	N	THR	A1138	45.469	25.875	65.938	1.00	84.62	N
ATOM	8890	CA	THR	A1138	44.906	26.562	67.125	1.00	84.62	C
ATOM	8891	C	THR	A1138	45.531	26.078	68.375	1.00	84.62	C
ATOM	8892	CB	THR	A1138	43.375	26.375	67.188	1.00	84.62	C

ATOM	8893	O	THR	A1138	45.781	26.859	69.312	1.00	84.62	O
ATOM	8894	CG2	THR	A1138	42.750	27.391	68.125	1.00	84.62	C
ATOM	8895	OG1	THR	A1138	42.812	26.547	65.875	1.00	84.62	O
ATOM	8896	N	ASP	A1139	45.906	24.641	68.500	1.00	82.06	N
ATOM	8897	CA	ASP	A1139	46.531	23.984	69.688	1.00	82.06	C
ATOM	8898	C	ASP	A1139	47.375	22.797	69.250	1.00	82.06	C
ATOM	8899	CB	ASP	A1139	45.438	23.516	70.625	1.00	82.06	C
ATOM	8900	O	ASP	A1139	47.250	22.312	68.125	1.00	82.06	O
ATOM	8901	CG	ASP	A1139	45.938	23.422	72.062	1.00	82.06	C
ATOM	8902	OD1	ASP	A1139	47.094	23.719	72.312	1.00	82.06	O
ATOM	8903	OD2	ASP	A1139	45.125	23.047	72.938	1.00	82.06	O
ATOM	8904	N	GLY	A1140	48.469	22.609	69.875	1.00	81.75	N
ATOM	8905	CA	GLY	A1140	49.312	21.438	69.625	1.00	81.75	C
ATOM	8906	C	GLY	A1140	49.969	20.922	70.938	1.00	81.75	C
ATOM	8907	O	GLY	A1140	50.156	21.656	71.875	1.00	81.75	O
ATOM	8908	N	TRP	A1141	50.000	19.484	71.188	1.00	80.94	N
ATOM	8909	CA	TRP	A1141	50.781	18.828	72.250	1.00	80.94	C
ATOM	8910	C	TRP	A1141	52.062	18.203	71.625	1.00	80.94	C
ATOM	8911	CB	TRP	A1141	49.969	17.734	72.938	1.00	80.94	C
ATOM	8912	O	TRP	A1141	52.000	17.438	70.688	1.00	80.94	O
ATOM	8913	CG	TRP	A1141	48.812	18.281	73.750	1.00	80.94	C
ATOM	8914	CD1	TRP	A1141	47.688	18.875	73.250	1.00	80.94	C
ATOM	8915	CD2	TRP	A1141	48.719	18.281	75.188	1.00	80.94	C
ATOM	8916	CE2	TRP	A1141	47.469	18.891	75.500	1.00	80.94	C
ATOM	8917	CE3	TRP	A1141	49.531	17.812	76.188	1.00	80.94	C
ATOM	8918	NE1	TRP	A1141	46.875	19.250	74.312	1.00	80.94	N
ATOM	8919	CH2	TRP	A1141	47.875	18.578	77.812	1.00	80.94	C
ATOM	8920	CZ2	TRP	A1141	47.062	19.047	76.812	1.00	80.94	C
ATOM	8921	CZ3	TRP	A1141	49.125	17.969	77.500	1.00	80.94	C
ATOM	8922	N	PHE	A1142	53.281	18.672	72.000	1.00	83.44	N
ATOM	8923	CA	PHE	A1142	54.562	18.125	71.625	1.00	83.44	C
ATOM	8924	C	PHE	A1142	55.219	17.375	72.750	1.00	83.44	C
ATOM	8925	CB	PHE	A1142	55.500	19.234	71.125	1.00	83.44	C
ATOM	8926	O	PHE	A1142	55.562	17.984	73.812	1.00	83.44	O
ATOM	8927	CG	PHE	A1142	54.938	20.016	69.938	1.00	83.44	C
ATOM	8928	CD1	PHE	A1142	55.188	19.609	68.625	1.00	83.44	C
ATOM	8929	CD2	PHE	A1142	54.188	21.156	70.188	1.00	83.44	C
ATOM	8930	CE1	PHE	A1142	54.656	20.328	67.562	1.00	83.44	C
ATOM	8931	CE2	PHE	A1142	53.656	21.891	69.125	1.00	83.44	C
ATOM	8932	CZ	PHE	A1142	53.906	21.469	67.812	1.00	83.44	C
ATOM	8933	N	ARG	A1143	55.094	15.938	72.812	1.00	82.19	N
ATOM	8934	CA	ARG	A1143	55.625	15.078	73.875	1.00	82.19	C
ATOM	8935	C	ARG	A1143	57.094	14.766	73.625	1.00	82.19	C
ATOM	8936	CB	ARG	A1143	54.812	13.781	73.938	1.00	82.19	C
ATOM	8937	O	ARG	A1143	57.719	14.117	74.500	1.00	82.19	O
ATOM	8938	CG	ARG	A1143	53.406	13.969	74.500	1.00	82.19	C
ATOM	8939	CD	ARG	A1143	52.719	12.641	74.750	1.00	82.19	C
ATOM	8940	NE	ARG	A1143	51.469	12.805	75.500	1.00	82.19	N
ATOM	8941	NH1	ARG	A1143	50.906	10.570	75.500	1.00	82.19	N
ATOM	8942	NH2	ARG	A1143	49.562	12.086	76.562	1.00	82.19	N
ATOM	8943	CZ	ARG	A1143	50.656	11.820	75.875	1.00	82.19	C
ATOM	8944	N	GLY	A1144	57.906	15.633	72.812	1.00	82.44	N
ATOM	8945	CA	GLY	A1144	59.312	15.469	72.562	1.00	82.44	C
ATOM	8946	C	GLY	A1144	60.062	16.781	72.562	1.00	82.44	C
ATOM	8947	O	GLY	A1144	59.688	17.734	73.188	1.00	82.44	O
ATOM	8948	N	THR	A1145	61.344	16.719	72.062	1.00	82.81	N
ATOM	8949	CA	THR	A1145	62.250	17.844	71.938	1.00	82.81	C
ATOM	8950	C	THR	A1145	61.844	18.766	70.812	1.00	82.81	C
ATOM	8951	CB	THR	A1145	63.688	17.375	71.750	1.00	82.81	C
ATOM	8952	O	THR	A1145	61.531	18.297	69.688	1.00	82.81	O
ATOM	8953	CG2	THR	A1145	64.688	18.547	71.812	1.00	82.81	C
ATOM	8954	OG1	THR	A1145	64.062	16.438	72.750	1.00	82.81	O
ATOM	8955	N	VAL	A1146	61.562	19.922	71.062	1.00	82.69	N
ATOM	8956	CA	VAL	A1146	61.312	20.969	70.062	1.00	82.69	C

ATOM	8957	C	VAL	A1146	62.594	21.719	69.812	1.00	82.69	C
ATOM	8958	CB	VAL	A1146	60.219	21.938	70.562	1.00	82.69	C
ATOM	8959	O	VAL	A1146	63.250	22.250	70.688	1.00	82.69	O
ATOM	8960	CG1	VAL	A1146	59.969	23.031	69.562	1.00	82.69	C
ATOM	8961	CG2	VAL	A1146	58.906	21.188	70.875	1.00	82.69	C
ATOM	8962	N	TYR	A1147	63.281	21.516	68.562	1.00	81.88	N
ATOM	8963	CA	TYR	A1147	64.375	22.359	68.062	1.00	81.88	C
ATOM	8964	C	TYR	A1147	63.844	23.562	67.312	1.00	81.88	C
ATOM	8965	CB	TYR	A1147	65.312	21.562	67.188	1.00	81.88	C
ATOM	8966	O	TYR	A1147	63.250	23.422	66.250	1.00	81.88	O
ATOM	8967	CG	TYR	A1147	66.125	20.516	67.938	1.00	81.88	C
ATOM	8968	CD1	TYR	A1147	67.312	20.844	68.562	1.00	81.88	C
ATOM	8969	CD2	TYR	A1147	65.688	19.203	68.000	1.00	81.88	C
ATOM	8970	CE1	TYR	A1147	68.062	19.891	69.250	1.00	81.88	C
ATOM	8971	CE2	TYR	A1147	66.375	18.234	68.688	1.00	81.88	C
ATOM	8972	OH	TYR	A1147	68.312	17.641	70.000	1.00	81.88	O
ATOM	8973	CZ	TYR	A1147	67.625	18.594	69.312	1.00	81.88	C
ATOM	8974	N	ALA	A1148	63.688	24.672	68.000	1.00	77.81	N
ATOM	8975	CA	ALA	A1148	63.219	25.922	67.375	1.00	77.81	C
ATOM	8976	C	ALA	A1148	64.312	26.984	67.375	1.00	77.81	C
ATOM	8977	CB	ALA	A1148	62.000	26.422	68.125	1.00	77.81	C
ATOM	8978	O	ALA	A1148	65.062	27.156	68.312	1.00	77.81	O
ATOM	8979	N	GLU	A1149	64.625	27.469	66.062	1.00	78.19	N
ATOM	8980	CA	GLU	A1149	65.562	28.578	66.000	1.00	78.19	C
ATOM	8981	C	GLU	A1149	65.062	29.797	66.750	1.00	78.19	C
ATOM	8982	CB	GLU	A1149	65.812	28.938	64.500	1.00	78.19	C
ATOM	8983	O	GLU	A1149	65.875	30.578	67.250	1.00	78.19	O
ATOM	8984	CG	GLU	A1149	66.688	27.906	63.750	1.00	78.19	C
ATOM	8985	CD	GLU	A1149	67.000	28.328	62.344	1.00	78.19	C
ATOM	8986	OE1	GLU	A1149	67.812	27.625	61.656	1.00	78.19	O
ATOM	8987	OE2	GLU	A1149	66.500	29.375	61.906	1.00	78.19	O
ATOM	8988	N	LYS	A1150	63.719	30.031	66.938	1.00	70.56	N
ATOM	8989	CA	LYS	A1150	63.031	31.172	67.500	1.00	70.56	C
ATOM	8990	C	LYS	A1150	61.656	30.797	68.000	1.00	70.56	C
ATOM	8991	CB	LYS	A1150	62.969	32.344	66.562	1.00	70.56	C
ATOM	8992	O	LYS	A1150	60.875	30.203	67.250	1.00	70.56	O
ATOM	8993	CG	LYS	A1150	62.438	33.625	67.125	1.00	70.56	C
ATOM	8994	CD	LYS	A1150	62.406	34.750	66.125	1.00	70.56	C
ATOM	8995	CE	LYS	A1150	61.844	36.031	66.688	1.00	70.56	C
ATOM	8996	NZ	LYS	A1150	61.781	37.125	65.688	1.00	70.56	N
ATOM	8997	N	ILE	A1151	61.438	30.891	69.312	1.00	72.44	N
ATOM	8998	CA	ILE	A1151	60.094	30.797	69.875	1.00	72.44	C
ATOM	8999	C	ILE	A1151	59.531	32.188	70.125	1.00	72.44	C
ATOM	9000	CB	ILE	A1151	60.125	30.000	71.188	1.00	72.44	C
ATOM	9001	O	ILE	A1151	60.094	33.000	70.875	1.00	72.44	O
ATOM	9002	CG1	ILE	A1151	60.656	28.594	70.938	1.00	72.44	C
ATOM	9003	CG2	ILE	A1151	58.719	29.938	71.812	1.00	72.44	C
ATOM	9004	CD1	ILE	A1151	60.875	27.781	72.250	1.00	72.44	C
ATOM	9005	N	GLU	A1152	58.719	32.781	69.250	1.00	65.25	N
ATOM	9006	CA	GLU	A1152	58.031	34.062	69.375	1.00	65.25	C
ATOM	9007	C	GLU	A1152	56.750	33.938	70.188	1.00	65.25	C
ATOM	9008	CB	GLU	A1152	57.750	34.656	68.000	1.00	65.25	C
ATOM	9009	O	GLU	A1152	55.875	33.156	69.875	1.00	65.25	O
ATOM	9010	CG	GLU	A1152	58.969	35.250	67.312	1.00	65.25	C
ATOM	9011	CD	GLU	A1152	58.594	36.000	66.062	1.00	65.25	C
ATOM	9012	OE1	GLU	A1152	59.531	36.562	65.375	1.00	65.25	O
ATOM	9013	OE2	GLU	A1152	57.406	35.969	65.625	1.00	65.25	O
ATOM	9014	N	GLY	A1153	56.781	34.406	71.562	1.00	60.66	N
ATOM	9015	CA	GLY	A1153	55.750	34.375	72.562	1.00	60.66	C
ATOM	9016	C	GLY	A1153	56.250	33.969	73.938	1.00	60.66	C
ATOM	9017	O	GLY	A1153	57.312	33.344	74.062	1.00	60.66	O
ATOM	9018	N	ASP	A1154	56.344	34.906	74.938	1.00	55.81	N
ATOM	9019	CA	ASP	A1154	57.062	34.906	76.188	1.00	55.81	C
ATOM	9020	C	ASP	A1154	56.594	33.719	77.062	1.00	55.81	C

ATOM	9021	CB	ASP	A1154	56.875	36.219	76.938	1.00	55.81	C
ATOM	9022	O	ASP	A1154	56.000	33.938	78.125	1.00	55.81	O
ATOM	9023	CG	ASP	A1154	57.844	37.312	76.438	1.00	55.81	C
ATOM	9024	OD1	ASP	A1154	58.812	37.000	75.750	1.00	55.81	O
ATOM	9025	OD2	ASP	A1154	57.594	38.500	76.750	1.00	55.81	O
ATOM	9026	N	VAL	A1155	56.469	32.438	76.562	1.00	68.38	N
ATOM	9027	CA	VAL	A1155	56.281	31.359	77.500	1.00	68.38	C
ATOM	9028	C	VAL	A1155	57.562	31.234	78.375	1.00	68.38	C
ATOM	9029	CB	VAL	A1155	56.000	30.016	76.812	1.00	68.38	C
ATOM	9030	O	VAL	A1155	57.469	31.125	79.562	1.00	68.38	O
ATOM	9031	CG1	VAL	A1155	55.781	28.906	77.875	1.00	68.38	C
ATOM	9032	CG2	VAL	A1155	54.750	30.141	75.938	1.00	68.38	C
ATOM	9033	N	ALA	A1156	58.875	31.609	77.875	1.00	72.94	N
ATOM	9034	CA	ALA	A1156	60.125	31.531	78.625	1.00	72.94	C
ATOM	9035	C	ALA	A1156	61.250	32.281	77.875	1.00	72.94	C
ATOM	9036	CB	ALA	A1156	60.531	30.078	78.875	1.00	72.94	C
ATOM	9037	O	ALA	A1156	61.562	31.938	76.688	1.00	72.94	O
ATOM	9038	N	LYS	A1157	61.562	33.312	78.188	1.00	83.44	N
ATOM	9039	CA	LYS	A1157	62.625	34.188	77.688	1.00	83.44	C
ATOM	9040	C	LYS	A1157	63.719	34.375	78.750	1.00	83.44	C
ATOM	9041	CB	LYS	A1157	62.094	35.531	77.250	1.00	83.44	C
ATOM	9042	O	LYS	A1157	63.406	34.562	79.938	1.00	83.44	O
ATOM	9043	CG	LYS	A1157	63.125	36.438	76.625	1.00	83.44	C
ATOM	9044	CD	LYS	A1157	62.500	37.750	76.188	1.00	83.44	C
ATOM	9045	CE	LYS	A1157	63.562	38.688	75.562	1.00	83.44	C
ATOM	9046	NZ	LYS	A1157	62.938	39.938	75.062	1.00	83.44	N
ATOM	9047	N	ALA	A1158	65.125	34.188	78.375	1.00	83.38	N
ATOM	9048	CA	ALA	A1158	66.188	34.500	79.312	1.00	83.38	C
ATOM	9049	C	ALA	A1158	66.875	35.812	78.938	1.00	83.38	C
ATOM	9050	CB	ALA	A1158	67.250	33.344	79.312	1.00	83.38	C
ATOM	9051	O	ALA	A1158	67.188	36.031	77.750	1.00	83.38	O
ATOM	9052	N	VAL	A1159	66.812	36.531	79.750	1.00	86.62	N
ATOM	9053	CA	VAL	A1159	67.500	37.812	79.562	1.00	86.62	C
ATOM	9054	C	VAL	A1159	68.750	37.812	80.438	1.00	86.62	C
ATOM	9055	CB	VAL	A1159	66.625	39.000	79.875	1.00	86.62	C
ATOM	9056	O	VAL	A1159	68.750	37.312	81.562	1.00	86.62	O
ATOM	9057	CG1	VAL	A1159	66.938	40.188	79.000	1.00	86.62	C
ATOM	9058	CG2	VAL	A1159	65.125	38.625	79.812	1.00	86.62	C
ATOM	9059	N	VAL	A1160	69.938	38.219	79.875	1.00	88.50	N
ATOM	9060	CA	VAL	A1160	71.250	38.219	80.625	1.00	88.50	C
ATOM	9061	C	VAL	A1160	71.688	39.656	80.812	1.00	88.50	C
ATOM	9062	CB	VAL	A1160	72.312	37.406	79.875	1.00	88.50	C
ATOM	9063	O	VAL	A1160	71.500	40.531	80.000	1.00	88.50	O
ATOM	9064	CG1	VAL	A1160	72.000	35.938	79.875	1.00	88.50	C
ATOM	9065	CG2	VAL	A1160	72.500	37.938	78.438	1.00	88.50	C
ATOM	9066	N	LEU	A1161	72.062	39.875	81.812	1.00	89.81	N
ATOM	9067	CA	LEU	A1161	72.625	41.188	82.188	1.00	89.81	C
ATOM	9068	C	LEU	A1161	74.062	41.031	82.750	1.00	89.81	C
ATOM	9069	CB	LEU	A1161	71.750	41.875	83.188	1.00	89.81	C
ATOM	9070	O	LEU	A1161	74.250	40.188	83.625	1.00	89.81	O
ATOM	9071	CG	LEU	A1161	70.250	41.969	82.875	1.00	89.81	C
ATOM	9072	CD1	LEU	A1161	69.438	41.031	83.750	1.00	89.81	C
ATOM	9073	CD2	LEU	A1161	69.750	43.375	83.000	1.00	89.81	C
ATOM	9074	N	PRO	A1162	75.062	41.844	82.312	1.00	90.62	N
ATOM	9075	CA	PRO	A1162	76.438	41.844	82.875	1.00	90.62	C
ATOM	9076	C	PRO	A1162	76.438	42.562	84.250	1.00	90.62	C
ATOM	9077	CB	PRO	A1162	77.250	42.594	81.812	1.00	90.62	C
ATOM	9078	O	PRO	A1162	75.562	43.375	84.562	1.00	90.62	O
ATOM	9079	CG	PRO	A1162	76.250	43.469	81.125	1.00	90.62	C
ATOM	9080	CD	PRO	A1162	74.938	42.812	81.125	1.00	90.62	C
ATOM	9081	N	PHE	A1163	77.500	42.188	85.062	1.00	89.69	N
ATOM	9082	CA	PHE	A1163	77.688	42.844	86.375	1.00	89.69	C
ATOM	9083	C	PHE	A1163	77.750	44.375	86.188	1.00	89.69	C
ATOM	9084	CB	PHE	A1163	78.938	42.312	87.062	1.00	89.69	C

ATOM	9085	O	PHE	A1163	78.438	44.844	85.312	1.00	89.69	O
ATOM	9086	CG	PHE	A1163	79.125	42.906	88.438	1.00	89.69	C
ATOM	9087	CD1	PHE	A1163	80.188	43.750	88.688	1.00	89.69	C
ATOM	9088	CD2	PHE	A1163	78.250	42.594	89.500	1.00	89.69	C
ATOM	9089	CE1	PHE	A1163	80.438	44.281	90.000	1.00	89.69	C
ATOM	9090	CE2	PHE	A1163	78.438	43.156	90.750	1.00	89.69	C
ATOM	9091	CZ	PHE	A1163	79.500	43.969	91.000	1.00	89.69	C
ATOM	9092	N	ASN	A1164	77.062	45.250	86.812	1.00	88.88	N
ATOM	9093	CA	ASN	A1164	76.875	46.688	86.750	1.00	88.88	C
ATOM	9094	C	ASN	A1164	76.062	47.125	85.562	1.00	88.88	C
ATOM	9095	CB	ASN	A1164	78.188	47.406	86.812	1.00	88.88	C
ATOM	9096	O	ASN	A1164	76.188	48.250	85.125	1.00	88.88	O
ATOM	9097	CG	ASN	A1164	78.750	47.562	88.188	1.00	88.88	C
ATOM	9098	ND2	ASN	A1164	80.062	47.844	88.312	1.00	88.88	N
ATOM	9099	OD1	ASN	A1164	78.062	47.406	89.188	1.00	88.88	O
ATOM	9100	N	GLY	A1165	75.250	46.188	84.875	1.00	91.00	N
ATOM	9101	CA	GLY	A1165	74.438	46.500	83.750	1.00	91.00	C
ATOM	9102	C	GLY	A1165	72.938	46.500	84.062	1.00	91.00	C
ATOM	9103	O	GLY	A1165	72.562	46.156	85.188	1.00	91.00	O
ATOM	9104	N	SER	A1166	72.062	47.000	83.062	1.00	91.44	N
ATOM	9105	CA	SER	A1166	70.625	47.031	83.188	1.00	91.44	C
ATOM	9106	C	SER	A1166	69.938	46.531	81.938	1.00	91.44	C
ATOM	9107	CB	SER	A1166	70.125	48.438	83.500	1.00	91.44	C
ATOM	9108	O	SER	A1166	70.562	46.562	80.812	1.00	91.44	O
ATOM	9109	OG	SER	A1166	70.375	49.312	82.438	1.00	91.44	O
ATOM	9110	N	VAL	A1167	68.812	45.875	82.062	1.00	91.44	N
ATOM	9111	CA	VAL	A1167	68.000	45.469	80.938	1.00	91.44	C
ATOM	9112	C	VAL	A1167	66.500	45.844	81.188	1.00	91.44	C
ATOM	9113	CB	VAL	A1167	68.062	43.938	80.688	1.00	91.44	C
ATOM	9114	O	VAL	A1167	66.000	45.625	82.250	1.00	91.44	O
ATOM	9115	CG1	VAL	A1167	67.375	43.156	81.812	1.00	91.44	C
ATOM	9116	CG2	VAL	A1167	67.500	43.562	79.312	1.00	91.44	C
ATOM	9117	N	HIS	A1168	65.938	46.500	80.125	1.00	92.19	N
ATOM	9118	CA	HIS	A1168	64.562	46.906	80.125	1.00	92.19	C
ATOM	9119	C	HIS	A1168	63.656	45.750	79.688	1.00	92.19	C
ATOM	9120	CB	HIS	A1168	64.312	48.125	79.250	1.00	92.19	C
ATOM	9121	O	HIS	A1168	63.906	45.125	78.625	1.00	92.19	O
ATOM	9122	CG	HIS	A1168	62.875	48.562	79.250	1.00	92.19	C
ATOM	9123	CD2	HIS	A1168	61.969	48.594	78.188	1.00	92.19	C
ATOM	9124	ND1	HIS	A1168	62.250	49.094	80.312	1.00	92.19	N
ATOM	9125	CE1	HIS	A1168	61.000	49.406	80.000	1.00	92.19	C
ATOM	9126	NE2	HIS	A1168	60.812	49.125	78.750	1.00	92.19	N
ATOM	9127	N	ILE	A1169	62.750	45.406	80.562	1.00	89.25	N
ATOM	9128	CA	ILE	A1169	61.719	44.438	80.188	1.00	89.25	C
ATOM	9129	C	ILE	A1169	60.406	45.156	79.875	1.00	89.25	C
ATOM	9130	CB	ILE	A1169	61.531	43.375	81.312	1.00	89.25	C
ATOM	9131	O	ILE	A1169	59.781	45.719	80.812	1.00	89.25	O
ATOM	9132	CG1	ILE	A1169	62.875	42.750	81.688	1.00	89.25	C
ATOM	9133	CG2	ILE	A1169	60.531	42.312	80.875	1.00	89.25	C
ATOM	9134	CD1	ILE	A1169	62.938	41.250	81.500	1.00	89.25	C
ATOM	9135	N	PRO	A1170	59.875	45.125	78.562	1.00	88.94	N
ATOM	9136	CA	PRO	A1170	58.688	45.875	78.188	1.00	88.94	C
ATOM	9137	C	PRO	A1170	57.406	45.281	78.750	1.00	88.94	C
ATOM	9138	CB	PRO	A1170	58.688	45.812	76.688	1.00	88.94	C
ATOM	9139	O	PRO	A1170	57.375	44.125	79.188	1.00	88.94	O
ATOM	9140	CG	PRO	A1170	59.438	44.562	76.312	1.00	88.94	C
ATOM	9141	CD	PRO	A1170	60.438	44.312	77.438	1.00	88.94	C
ATOM	9142	N	ALA	A1171	56.250	46.125	78.750	1.00	86.94	N
ATOM	9143	CA	ALA	A1171	54.938	45.719	79.125	1.00	86.94	C
ATOM	9144	C	ALA	A1171	54.312	44.781	78.125	1.00	86.94	C
ATOM	9145	CB	ALA	A1171	54.031	46.906	79.375	1.00	86.94	C
ATOM	9146	O	ALA	A1171	54.469	45.000	76.938	1.00	86.94	O
ATOM	9147	N	VAL	A1172	53.719	43.625	78.625	1.00	86.06	N
ATOM	9148	CA	VAL	A1172	52.938	42.719	77.750	1.00	86.06	C

ATOM	9149	C	VAL	A1172	51.562	42.500	78.438	1.00	86.06	C
ATOM	9150	CB	VAL	A1172	53.656	41.375	77.562	1.00	86.06	C
ATOM	9151	O	VAL	A1172	51.344	42.875	79.562	1.00	86.06	O
ATOM	9152	CG1	VAL	A1172	54.938	41.562	76.812	1.00	86.06	C
ATOM	9153	CG2	VAL	A1172	53.938	40.688	78.938	1.00	86.06	C
ATOM	9154	N	ASN	A1173	50.500	42.094	77.625	1.00	87.06	N
ATOM	9155	CA	ASN	A1173	49.125	42.000	78.062	1.00	87.06	C
ATOM	9156	C	ASN	A1173	48.906	40.781	78.938	1.00	87.06	C
ATOM	9157	CB	ASN	A1173	48.156	42.000	76.875	1.00	87.06	C
ATOM	9158	O	ASN	A1173	47.750	40.312	79.062	1.00	87.06	O
ATOM	9159	CG	ASN	A1173	48.375	40.781	76.000	1.00	87.06	C
ATOM	9160	ND2	ASN	A1173	47.531	40.656	74.938	1.00	87.06	N
ATOM	9161	OD1	ASN	A1173	49.250	39.969	76.188	1.00	87.06	O
ATOM	9162	N	TYR	A1174	49.875	40.031	79.375	1.00	86.12	N
ATOM	9163	CA	TYR	A1174	49.719	38.938	80.312	1.00	86.12	C
ATOM	9164	C	TYR	A1174	50.750	39.031	81.438	1.00	86.12	C
ATOM	9165	CB	TYR	A1174	49.875	37.594	79.625	1.00	86.12	C
ATOM	9166	O	TYR	A1174	51.750	39.750	81.375	1.00	86.12	O
ATOM	9167	CG	TYR	A1174	51.062	37.500	78.688	1.00	86.12	C
ATOM	9168	CD1	TYR	A1174	50.938	37.781	77.312	1.00	86.12	C
ATOM	9169	CD2	TYR	A1174	52.312	37.125	79.188	1.00	86.12	C
ATOM	9170	CE1	TYR	A1174	52.062	37.719	76.500	1.00	86.12	C
ATOM	9171	CE2	TYR	A1174	53.406	37.062	78.375	1.00	86.12	C
ATOM	9172	OH	TYR	A1174	54.375	37.281	76.188	1.00	86.12	O
ATOM	9173	CZ	TYR	A1174	53.281	37.344	77.000	1.00	86.12	C
ATOM	9174	N	ASN	A1175	50.500	38.406	82.688	1.00	87.12	N
ATOM	9175	CA	ASN	A1175	51.406	38.375	83.875	1.00	87.12	C
ATOM	9176	C	ASN	A1175	52.656	37.594	83.562	1.00	87.12	C
ATOM	9177	CB	ASN	A1175	50.688	37.812	85.062	1.00	87.12	C
ATOM	9178	O	ASN	A1175	52.625	36.562	82.875	1.00	87.12	O
ATOM	9179	CG	ASN	A1175	49.469	38.656	85.500	1.00	87.12	C
ATOM	9180	ND2	ASN	A1175	48.656	38.094	86.375	1.00	87.12	N
ATOM	9181	OD1	ASN	A1175	49.312	39.781	85.062	1.00	87.12	O
ATOM	9182	N	ARG	A1176	53.844	38.156	83.938	1.00	90.00	N
ATOM	9183	CA	ARG	A1176	55.125	37.469	83.750	1.00	90.00	C
ATOM	9184	C	ARG	A1176	55.781	37.156	85.125	1.00	90.00	C
ATOM	9185	CB	ARG	A1176	56.062	38.312	82.875	1.00	90.00	C
ATOM	9186	O	ARG	A1176	55.688	37.938	86.062	1.00	90.00	O
ATOM	9187	CG	ARG	A1176	55.594	38.500	81.500	1.00	90.00	C
ATOM	9188	CD	ARG	A1176	56.562	39.312	80.625	1.00	90.00	C
ATOM	9189	NE	ARG	A1176	56.469	40.750	80.875	1.00	90.00	N
ATOM	9190	NH1	ARG	A1176	58.375	41.281	79.688	1.00	90.00	N
ATOM	9191	NH2	ARG	A1176	57.125	42.938	80.688	1.00	90.00	N
ATOM	9192	CZ	ARG	A1176	57.312	41.656	80.438	1.00	90.00	C
ATOM	9193	N	HIS	A1177	56.344	35.750	85.188	1.00	89.44	N
ATOM	9194	CA	HIS	A1177	57.281	35.406	86.250	1.00	89.44	C
ATOM	9195	C	HIS	A1177	58.719	35.844	85.875	1.00	89.44	C
ATOM	9196	CB	HIS	A1177	57.250	33.906	86.562	1.00	89.44	C
ATOM	9197	O	HIS	A1177	59.219	35.438	84.812	1.00	89.44	O
ATOM	9198	CG	HIS	A1177	55.938	33.438	87.062	1.00	89.44	C
ATOM	9199	CD2	HIS	A1177	54.906	32.781	86.500	1.00	89.44	C
ATOM	9200	ND1	HIS	A1177	55.562	33.625	88.375	1.00	89.44	N
ATOM	9201	CE1	HIS	A1177	54.375	33.094	88.562	1.00	89.44	C
ATOM	9202	NE2	HIS	A1177	53.938	32.594	87.438	1.00	89.44	N
ATOM	9203	N	LEU	A1178	59.219	36.750	86.562	1.00	91.19	N
ATOM	9204	CA	LEU	A1178	60.625	37.094	86.438	1.00	91.19	C
ATOM	9205	C	LEU	A1178	61.469	36.375	87.500	1.00	91.19	C
ATOM	9206	CB	LEU	A1178	60.844	38.594	86.500	1.00	91.19	C
ATOM	9207	O	LEU	A1178	61.281	36.625	88.688	1.00	91.19	O
ATOM	9208	CG	LEU	A1178	61.250	39.312	85.250	1.00	91.19	C
ATOM	9209	CD1	LEU	A1178	60.031	39.938	84.562	1.00	91.19	C
ATOM	9210	CD2	LEU	A1178	62.312	40.344	85.500	1.00	91.19	C
ATOM	9211	N	VAL	A1179	62.312	35.500	87.062	1.00	90.69	N
ATOM	9212	CA	VAL	A1179	63.062	34.656	88.000	1.00	90.69	C

ATOM	9213	C	VAL	A1179	64.562	34.844	87.812	1.00	90.69	C
ATOM	9214	CB	VAL	A1179	62.656	33.156	87.875	1.00	90.69	C
ATOM	9215	O	VAL	A1179	65.062	34.688	86.688	1.00	90.69	O
ATOM	9216	CG1	VAL	A1179	63.406	32.312	88.938	1.00	90.69	C
ATOM	9217	CG2	VAL	A1179	61.156	33.000	88.062	1.00	90.69	C
ATOM	9218	N	ILE	A1180	65.188	35.156	88.812	1.00	91.38	N
ATOM	9219	CA	ILE	A1180	66.688	35.125	88.812	1.00	91.38	C
ATOM	9220	C	ILE	A1180	67.125	33.875	89.500	1.00	91.38	C
ATOM	9221	CB	ILE	A1180	67.250	36.375	89.562	1.00	91.38	C
ATOM	9222	O	ILE	A1180	67.062	33.750	90.750	1.00	91.38	O
ATOM	9223	CG1	ILE	A1180	66.688	37.656	88.938	1.00	91.38	C
ATOM	9224	CG2	ILE	A1180	68.750	36.375	89.500	1.00	91.38	C
ATOM	9225	CD1	ILE	A1180	66.875	38.906	89.750	1.00	91.38	C
ATOM	9226	N	PRO	A1181	67.562	33.000	88.750	1.00	90.38	N
ATOM	9227	CA	PRO	A1181	67.875	31.719	89.375	1.00	90.38	C
ATOM	9228	C	PRO	A1181	69.062	31.797	90.312	1.00	90.38	C
ATOM	9229	CB	PRO	A1181	68.250	30.828	88.125	1.00	90.38	C
ATOM	9230	O	PRO	A1181	69.125	31.062	91.312	1.00	90.38	O
ATOM	9231	CG	PRO	A1181	67.875	31.656	86.938	1.00	90.38	C
ATOM	9232	CD	PRO	A1181	67.688	33.062	87.375	1.00	90.38	C
ATOM	9233	N	TYR	A1182	70.125	32.594	90.000	1.00	87.94	N
ATOM	9234	CA	TYR	A1182	71.312	32.625	90.812	1.00	87.94	C
ATOM	9235	C	TYR	A1182	72.062	33.969	90.750	1.00	87.94	C
ATOM	9236	CB	TYR	A1182	72.312	31.484	90.500	1.00	87.94	C
ATOM	9237	O	TYR	A1182	72.250	34.469	89.625	1.00	87.94	O
ATOM	9238	CG	TYR	A1182	73.562	31.484	91.312	1.00	87.94	C
ATOM	9239	CD1	TYR	A1182	74.750	31.891	90.750	1.00	87.94	C
ATOM	9240	CD2	TYR	A1182	73.562	31.062	92.625	1.00	87.94	C
ATOM	9241	CE1	TYR	A1182	75.938	31.891	91.500	1.00	87.94	C
ATOM	9242	CE2	TYR	A1182	74.688	31.047	93.375	1.00	87.94	C
ATOM	9243	OH	TYR	A1182	77.062	31.469	93.562	1.00	87.94	O
ATOM	9244	CZ	TYR	A1182	75.875	31.469	92.812	1.00	87.94	C
ATOM	9245	N	VAL	A1183	72.250	34.531	91.812	1.00	89.69	N
ATOM	9246	CA	VAL	A1183	73.188	35.656	91.938	1.00	89.69	C
ATOM	9247	C	VAL	A1183	74.188	35.375	93.062	1.00	89.69	C
ATOM	9248	CB	VAL	A1183	72.438	36.969	92.188	1.00	89.69	C
ATOM	9249	O	VAL	A1183	73.812	34.844	94.125	1.00	89.69	O
ATOM	9250	CG1	VAL	A1183	71.688	36.906	93.562	1.00	89.69	C
ATOM	9251	CG2	VAL	A1183	73.375	38.156	92.188	1.00	89.69	C
ATOM	9252	N	GLY	A1184	75.125	35.531	92.688	1.00	84.62	N
ATOM	9253	CA	GLY	A1184	76.062	35.094	93.688	1.00	84.62	C
ATOM	9254	C	GLY	A1184	77.375	35.844	93.688	1.00	84.62	C
ATOM	9255	O	GLY	A1184	77.750	36.438	92.625	1.00	84.62	O
ATOM	9256	N	ILE	A1185	78.375	35.656	94.938	1.00	87.44	N
ATOM	9257	CA	ILE	A1185	79.688	36.219	95.188	1.00	87.44	C
ATOM	9258	C	ILE	A1185	80.688	35.125	95.562	1.00	87.44	C
ATOM	9259	CB	ILE	A1185	79.625	37.312	96.250	1.00	87.44	C
ATOM	9260	O	ILE	A1185	80.312	34.188	96.250	1.00	87.44	O
ATOM	9261	CG1	ILE	A1185	78.688	38.438	95.875	1.00	87.44	C
ATOM	9262	CG2	ILE	A1185	81.062	37.844	96.562	1.00	87.44	C
ATOM	9263	CD1	ILE	A1185	78.438	39.469	96.938	1.00	87.44	C
ATOM	9264	N	HIS	A1186	81.688	35.125	94.875	1.00	84.69	N
ATOM	9265	CA	HIS	A1186	82.750	34.156	95.125	1.00	84.69	C
ATOM	9266	C	HIS	A1186	83.938	34.844	95.688	1.00	84.69	C
ATOM	9267	CB	HIS	A1186	83.062	33.375	93.875	1.00	84.69	C
ATOM	9268	O	HIS	A1186	84.562	35.688	95.062	1.00	84.69	O
ATOM	9269	CG	HIS	A1186	81.938	32.625	93.312	1.00	84.69	C
ATOM	9270	CD2	HIS	A1186	80.688	33.031	93.000	1.00	84.69	C
ATOM	9271	ND1	HIS	A1186	82.000	31.297	92.938	1.00	84.69	N
ATOM	9272	CE1	HIS	A1186	80.875	30.891	92.438	1.00	84.69	C
ATOM	9273	NE2	HIS	A1186	80.000	31.922	92.500	1.00	84.69	N
ATOM	9274	N	GLY	A1187	84.000	34.594	97.000	1.00	71.38	N
ATOM	9275	CA	GLY	A1187	85.125	35.125	97.688	1.00	71.38	C
ATOM	9276	C	GLY	A1187	86.375	34.188	97.625	1.00	71.38	C

ATOM	9277	O	GLY	A1187	86.188	32.969	97.562	1.00	71.38	O
ATOM	9278	N	TYR	A1188	87.562	34.406	97.438	1.00	74.31	N
ATOM	9279	CA	TYR	A1188	88.750	33.562	97.500	1.00	74.31	C
ATOM	9280	C	TYR	A1188	89.625	33.938	98.688	1.00	74.31	C
ATOM	9281	CB	TYR	A1188	89.562	33.594	96.250	1.00	74.31	C
ATOM	9282	O	TYR	A1188	89.562	35.094	99.188	1.00	74.31	O
ATOM	9283	CG	TYR	A1188	89.875	35.000	95.750	1.00	74.31	C
ATOM	9284	CD1	TYR	A1188	89.000	35.656	94.875	1.00	74.31	C
ATOM	9285	CD2	TYR	A1188	91.000	35.688	96.188	1.00	74.31	C
ATOM	9286	CE1	TYR	A1188	89.250	36.938	94.438	1.00	74.31	C
ATOM	9287	CE2	TYR	A1188	91.250	36.969	95.750	1.00	74.31	C
ATOM	9288	OH	TYR	A1188	90.625	38.875	94.438	1.00	74.31	O
ATOM	9289	CZ	TYR	A1188	90.375	37.594	94.875	1.00	74.31	C
ATOM	9290	N	THR	A1189	90.188	32.656	99.250	1.00	73.31	N
ATOM	9291	CA	THR	A1189	91.125	32.781	100.312	1.00	73.31	C
ATOM	9292	C	THR	A1189	92.438	33.438	99.875	1.00	73.31	C
ATOM	9293	CB	THR	A1189	91.438	31.391	100.938	1.00	73.31	C
ATOM	9294	O	THR	A1189	92.938	33.156	98.812	1.00	73.31	O
ATOM	9295	CG2	THR	A1189	91.062	31.375	102.438	1.00	73.31	C
ATOM	9296	OG1	THR	A1189	90.625	30.391	100.312	1.00	73.31	O
ATOM	9297	N	TYR	A1190	92.812	34.656	100.562	1.00	65.19	N
ATOM	9298	CA	TYR	A1190	93.938	35.438	100.188	1.00	65.19	C
ATOM	9299	C	TYR	A1190	94.938	35.500	101.375	1.00	65.19	C
ATOM	9300	CB	TYR	A1190	93.562	36.844	99.750	1.00	65.19	C
ATOM	9301	O	TYR	A1190	94.625	35.062	102.500	1.00	65.19	O
ATOM	9302	CG	TYR	A1190	92.938	37.656	100.875	1.00	65.19	C
ATOM	9303	CD1	TYR	A1190	91.625	37.469	101.250	1.00	65.19	C
ATOM	9304	CD2	TYR	A1190	93.688	38.625	101.562	1.00	65.19	C
ATOM	9305	CE1	TYR	A1190	91.062	38.250	102.250	1.00	65.19	C
ATOM	9306	CE2	TYR	A1190	93.125	39.375	102.562	1.00	65.19	C
ATOM	9307	OH	TYR	A1190	91.250	39.938	103.938	1.00	65.19	O
ATOM	9308	CZ	TYR	A1190	91.812	39.188	102.938	1.00	65.19	C
ATOM	9309	N	SER	A1191	96.250	35.375	101.562	1.00	64.19	N
ATOM	9310	CA	SER	A1191	97.375	35.219	102.438	1.00	64.19	C
ATOM	9311	C	SER	A1191	97.062	35.719	103.875	1.00	64.19	C
ATOM	9312	CB	SER	A1191	98.625	35.969	101.938	1.00	64.19	C
ATOM	9313	O	SER	A1191	96.625	36.844	104.000	1.00	64.19	O
ATOM	9314	OG	SER	A1191	99.625	36.094	102.938	1.00	64.19	O
ATOM	9315	N	GLY	A1192	96.625	34.781	104.812	1.00	69.69	N
ATOM	9316	CA	GLY	A1192	96.312	34.938	106.188	1.00	69.69	C
ATOM	9317	C	GLY	A1192	95.125	34.094	106.625	1.00	69.69	C
ATOM	9318	O	GLY	A1192	94.812	34.062	107.812	1.00	69.69	O
ATOM	9319	N	GLY	A1193	94.688	33.312	105.750	1.00	59.75	N
ATOM	9320	CA	GLY	A1193	93.562	32.469	106.062	1.00	59.75	C
ATOM	9321	C	GLY	A1193	92.250	33.219	106.125	1.00	59.75	C
ATOM	9322	O	GLY	A1193	91.250	32.656	106.562	1.00	59.75	O
ATOM	9323	N	THR	A1194	92.188	34.656	105.812	1.00	65.50	N
ATOM	9324	CA	THR	A1194	90.938	35.438	105.812	1.00	65.50	C
ATOM	9325	C	THR	A1194	90.312	35.438	104.438	1.00	65.50	C
ATOM	9326	CB	THR	A1194	91.125	36.875	106.250	1.00	65.50	C
ATOM	9327	O	THR	A1194	91.000	35.375	103.438	1.00	65.50	O
ATOM	9328	CG2	THR	A1194	90.938	37.031	107.750	1.00	65.50	C
ATOM	9329	OG1	THR	A1194	92.500	37.281	105.938	1.00	65.50	O
ATOM	9330	N	TRP	A1195	89.000	35.312	104.500	1.00	62.06	N
ATOM	9331	CA	TRP	A1195	88.188	35.094	103.250	1.00	62.06	C
ATOM	9332	C	TRP	A1195	87.625	36.406	102.750	1.00	62.06	C
ATOM	9333	CB	TRP	A1195	87.125	34.094	103.500	1.00	62.06	C
ATOM	9334	O	TRP	A1195	87.312	37.281	103.500	1.00	62.06	O
ATOM	9335	CG	TRP	A1195	87.562	32.750	104.000	1.00	62.06	C
ATOM	9336	CD1	TRP	A1195	88.000	32.500	105.312	1.00	62.06	C
ATOM	9337	CD2	TRP	A1195	87.688	31.531	103.312	1.00	62.06	C
ATOM	9338	CE2	TRP	A1195	88.125	30.562	104.188	1.00	62.06	C
ATOM	9339	CE3	TRP	A1195	87.375	31.156	102.000	1.00	62.06	C
ATOM	9340	NE1	TRP	A1195	88.375	31.172	105.438	1.00	62.06	N

ATOM	9341	CH2	TRP	A1195	88.062	28.891	102.500	1.00	62.06	C
ATOM	9342	CZ2	TRP	A1195	88.375	29.234	103.812	1.00	62.06	C
ATOM	9343	CZ3	TRP	A1195	87.562	29.828	101.625	1.00	62.06	C
ATOM	9344	N	GLY	A1196	87.875	36.594	101.438	1.00	77.81	N
ATOM	9345	CA	GLY	A1196	87.250	37.656	100.688	1.00	77.81	C
ATOM	9346	C	GLY	A1196	85.750	37.781	100.875	1.00	77.81	C
ATOM	9347	O	GLY	A1196	85.062	36.781	101.188	1.00	77.81	O
ATOM	9348	N	ARG	A1197	85.312	39.219	101.125	1.00	81.69	N
ATOM	9349	CA	ARG	A1197	83.938	39.594	101.438	1.00	81.69	C
ATOM	9350	C	ARG	A1197	83.375	40.562	100.375	1.00	81.69	C
ATOM	9351	CB	ARG	A1197	83.875	40.250	102.812	1.00	81.69	C
ATOM	9352	O	ARG	A1197	84.188	41.188	99.625	1.00	81.69	O
ATOM	9353	CG	ARG	A1197	84.688	41.531	102.938	1.00	81.69	C
ATOM	9354	CD	ARG	A1197	84.625	42.094	104.375	1.00	81.69	C
ATOM	9355	NE	ARG	A1197	85.438	43.281	104.500	1.00	81.69	N
ATOM	9356	NH1	ARG	A1197	84.938	43.656	106.750	1.00	81.69	N
ATOM	9357	NH2	ARG	A1197	86.312	45.062	105.625	1.00	81.69	N
ATOM	9358	CZ	ARG	A1197	85.562	44.000	105.625	1.00	81.69	C
ATOM	9359	N	GLY	A1198	82.062	40.531	100.000	1.00	86.50	N
ATOM	9360	CA	GLY	A1198	81.375	41.438	99.125	1.00	86.50	C
ATOM	9361	C	GLY	A1198	79.875	41.219	99.125	1.00	86.50	C
ATOM	9362	O	GLY	A1198	79.375	40.281	99.750	1.00	86.50	O
ATOM	9363	N	THR	A1199	79.250	42.344	98.625	1.00	88.25	N
ATOM	9364	CA	THR	A1199	77.812	42.281	98.438	1.00	88.25	C
ATOM	9365	C	THR	A1199	77.438	42.500	97.000	1.00	88.25	C
ATOM	9366	CB	THR	A1199	77.125	43.344	99.312	1.00	88.25	C
ATOM	9367	O	THR	A1199	78.000	43.344	96.312	1.00	88.25	O
ATOM	9368	CG2	THR	A1199	75.625	43.219	99.250	1.00	88.25	C
ATOM	9369	OG1	THR	A1199	77.562	43.188	100.688	1.00	88.25	O
ATOM	9370	N	VAL	A1200	76.562	41.750	96.312	1.00	90.62	N
ATOM	9371	CA	VAL	A1200	75.938	41.938	95.000	1.00	90.62	C
ATOM	9372	C	VAL	A1200	74.438	42.000	95.125	1.00	90.62	C
ATOM	9373	CB	VAL	A1200	76.375	40.844	94.000	1.00	90.62	C
ATOM	9374	O	VAL	A1200	73.875	41.375	96.000	1.00	90.62	O
ATOM	9375	CG1	VAL	A1200	77.875	40.938	93.750	1.00	90.62	C
ATOM	9376	CG2	VAL	A1200	76.000	39.469	94.562	1.00	90.62	C
ATOM	9377	N	TRP	A1201	73.812	42.906	94.312	1.00	92.12	N
ATOM	9378	CA	TRP	A1201	72.375	43.031	94.375	1.00	92.12	C
ATOM	9379	C	TRP	A1201	71.812	43.156	93.000	1.00	92.12	C
ATOM	9380	CB	TRP	A1201	72.000	44.281	95.188	1.00	92.12	C
ATOM	9381	O	TRP	A1201	72.500	43.438	92.000	1.00	92.12	O
ATOM	9382	CG	TRP	A1201	72.625	45.531	94.750	1.00	92.12	C
ATOM	9383	CD1	TRP	A1201	72.250	46.375	93.750	1.00	92.12	C
ATOM	9384	CD2	TRP	A1201	73.875	46.094	95.312	1.00	92.12	C
ATOM	9385	CE2	TRP	A1201	74.125	47.281	94.562	1.00	92.12	C
ATOM	9386	CE3	TRP	A1201	74.750	45.719	96.312	1.00	92.12	C
ATOM	9387	NE1	TRP	A1201	73.125	47.438	93.625	1.00	92.12	N
ATOM	9388	CH2	TRP	A1201	76.062	47.688	95.812	1.00	92.12	C
ATOM	9389	CZ2	TRP	A1201	75.250	48.094	94.812	1.00	92.12	C
ATOM	9390	CZ3	TRP	A1201	75.875	46.531	96.562	1.00	92.12	C
ATOM	9391	N	VAL	A1202	70.500	42.812	92.812	1.00	92.31	N
ATOM	9392	CA	VAL	A1202	69.688	43.031	91.625	1.00	92.31	C
ATOM	9393	C	VAL	A1202	68.438	43.906	92.000	1.00	92.31	C
ATOM	9394	CB	VAL	A1202	69.188	41.719	91.000	1.00	92.31	C
ATOM	9395	O	VAL	A1202	67.688	43.562	92.875	1.00	92.31	O
ATOM	9396	CG1	VAL	A1202	68.438	41.969	89.688	1.00	92.31	C
ATOM	9397	CG2	VAL	A1202	70.375	40.812	90.688	1.00	92.31	C
ATOM	9398	N	ASP	A1203	68.375	44.969	91.250	1.00	91.00	N
ATOM	9399	CA	ASP	A1203	67.312	45.875	91.438	1.00	91.00	C
ATOM	9400	C	ASP	A1203	66.312	45.875	90.250	1.00	91.00	C
ATOM	9401	CB	ASP	A1203	67.812	47.281	91.688	1.00	91.00	C
ATOM	9402	O	ASP	A1203	66.750	45.625	89.125	1.00	91.00	O
ATOM	9403	CG	ASP	A1203	68.500	47.469	93.062	1.00	91.00	C
ATOM	9404	OD1	ASP	A1203	67.938	46.969	94.062	1.00	91.00	O

ATOM	9405	OD2	ASP	A1203	69.562	48.094	93.125	1.00	91.00	O
ATOM	9406	N	SER	A1204	65.062	46.062	90.625	1.00	90.81	N
ATOM	9407	CA	SER	A1204	64.000	46.281	89.625	1.00	90.81	C
ATOM	9408	C	SER	A1204	63.281	47.594	89.875	1.00	90.81	C
ATOM	9409	CB	SER	A1204	63.000	45.125	89.625	1.00	90.81	C
ATOM	9410	O	SER	A1204	62.938	47.906	91.062	1.00	90.81	O
ATOM	9411	OG	SER	A1204	61.844	45.469	88.875	1.00	90.81	O
ATOM	9412	N	THR	A1205	63.094	48.406	88.750	1.00	90.06	N
ATOM	9413	CA	THR	A1205	62.344	49.625	88.875	1.00	90.06	C
ATOM	9414	C	THR	A1205	60.938	49.344	89.375	1.00	90.06	C
ATOM	9415	CB	THR	A1205	62.281	50.406	87.562	1.00	90.06	C
ATOM	9416	O	THR	A1205	60.281	50.250	89.938	1.00	90.06	O
ATOM	9417	CG2	THR	A1205	63.688	50.906	87.188	1.00	90.06	C
ATOM	9418	OG1	THR	A1205	61.812	49.531	86.562	1.00	90.06	O
ATOM	9419	N	TYR	A1206	60.375	48.094	89.125	1.00	88.44	N
ATOM	9420	CA	TYR	A1206	59.031	47.719	89.562	1.00	88.44	C
ATOM	9421	C	TYR	A1206	59.062	47.062	90.938	1.00	88.44	C
ATOM	9422	CB	TYR	A1206	58.406	46.719	88.562	1.00	88.44	C
ATOM	9423	O	TYR	A1206	58.250	47.406	91.812	1.00	88.44	O
ATOM	9424	CG	TYR	A1206	57.031	46.250	89.000	1.00	88.44	C
ATOM	9425	CD1	TYR	A1206	56.844	45.031	89.625	1.00	88.44	C
ATOM	9426	CD2	TYR	A1206	55.906	47.031	88.688	1.00	88.44	C
ATOM	9427	CE1	TYR	A1206	55.562	44.594	89.938	1.00	88.44	C
ATOM	9428	CE2	TYR	A1206	54.625	46.625	89.062	1.00	88.44	C
ATOM	9429	OH	TYR	A1206	53.219	44.969	90.062	1.00	88.44	O
ATOM	9430	CZ	TYR	A1206	54.469	45.406	89.688	1.00	88.44	C
ATOM	9431	N	GLY	A1207	60.031	46.250	91.312	1.00	82.81	N
ATOM	9432	CA	GLY	A1207	60.031	45.406	92.500	1.00	82.81	C
ATOM	9433	C	GLY	A1207	61.094	45.781	93.562	1.00	82.81	C
ATOM	9434	O	GLY	A1207	61.156	45.188	94.625	1.00	82.81	O
ATOM	9435	N	GLY	A1208	61.875	46.812	93.375	1.00	86.94	N
ATOM	9436	CA	GLY	A1208	62.969	47.188	94.250	1.00	86.94	C
ATOM	9437	C	GLY	A1208	64.062	46.188	94.250	1.00	86.94	C
ATOM	9438	O	GLY	A1208	64.375	45.594	93.188	1.00	86.94	O
ATOM	9439	N	ARG	A1209	64.875	46.031	95.438	1.00	90.12	N
ATOM	9440	CA	ARG	A1209	66.000	45.062	95.562	1.00	90.12	C
ATOM	9441	C	ARG	A1209	65.438	43.656	95.500	1.00	90.12	C
ATOM	9442	CB	ARG	A1209	66.812	45.312	96.812	1.00	90.12	C
ATOM	9443	O	ARG	A1209	64.750	43.219	96.438	1.00	90.12	O
ATOM	9444	CG	ARG	A1209	68.000	44.375	96.938	1.00	90.12	C
ATOM	9445	CD	ARG	A1209	69.188	44.875	96.125	1.00	90.12	C
ATOM	9446	NE	ARG	A1209	69.625	46.219	96.562	1.00	90.12	N
ATOM	9447	NH1	ARG	A1209	71.750	46.031	95.625	1.00	90.12	N
ATOM	9448	NH2	ARG	A1209	71.125	47.938	96.750	1.00	90.12	N
ATOM	9449	CZ	ARG	A1209	70.812	46.719	96.312	1.00	90.12	C
ATOM	9450	N	LEU	A1210	65.625	42.969	94.312	1.00	87.81	N
ATOM	9451	CA	LEU	A1210	65.062	41.625	94.125	1.00	87.81	C
ATOM	9452	C	LEU	A1210	66.000	40.594	94.812	1.00	87.81	C
ATOM	9453	CB	LEU	A1210	64.875	41.281	92.625	1.00	87.81	C
ATOM	9454	O	LEU	A1210	65.500	39.531	95.250	1.00	87.81	O
ATOM	9455	CG	LEU	A1210	63.969	42.219	91.875	1.00	87.81	C
ATOM	9456	CD1	LEU	A1210	63.875	41.781	90.438	1.00	87.81	C
ATOM	9457	CD2	LEU	A1210	62.594	42.281	92.500	1.00	87.81	C
ATOM	9458	N	ALA	A1211	67.250	40.781	94.812	1.00	87.88	N
ATOM	9459	CA	ALA	A1211	68.250	39.875	95.375	1.00	87.88	C
ATOM	9460	C	ALA	A1211	69.438	40.656	95.875	1.00	87.88	C
ATOM	9461	CB	ALA	A1211	68.688	38.844	94.375	1.00	87.88	C
ATOM	9462	O	ALA	A1211	69.875	41.656	95.250	1.00	87.88	O
ATOM	9463	N	ASN	A1212	69.875	40.281	97.062	1.00	88.31	N
ATOM	9464	CA	ASN	A1212	71.062	40.875	97.750	1.00	88.31	C
ATOM	9465	C	ASN	A1212	71.875	39.812	98.438	1.00	88.31	C
ATOM	9466	CB	ASN	A1212	70.625	41.938	98.750	1.00	88.31	C
ATOM	9467	O	ASN	A1212	71.438	39.062	99.250	1.00	88.31	O
ATOM	9468	CG	ASN	A1212	71.750	42.594	99.438	1.00	88.31	C

ATOM	9469	ND2	ASN	A1212	71.562	43.000	100.688	1.00	88.31	N
ATOM	9470	OD1	ASN	A1212	72.875	42.719	98.875	1.00	88.31	O
ATOM	9471	N	VAL	A1213	73.125	39.719	98.062	1.00	88.00	N
ATOM	9472	CA	VAL	A1213	74.000	38.688	98.625	1.00	88.00	C
ATOM	9473	C	VAL	A1213	75.250	39.344	99.250	1.00	88.00	C
ATOM	9474	CB	VAL	A1213	74.438	37.688	97.562	1.00	88.00	C
ATOM	9475	O	VAL	A1213	75.938	40.156	98.625	1.00	88.00	O
ATOM	9476	CG1	VAL	A1213	75.375	36.625	98.125	1.00	88.00	C
ATOM	9477	CG2	VAL	A1213	73.188	37.031	96.875	1.00	88.00	C
ATOM	9478	N	GLN	A1214	75.375	39.031	100.375	1.00	82.25	N
ATOM	9479	CA	GLN	A1214	76.562	39.500	101.125	1.00	82.25	C
ATOM	9480	C	GLN	A1214	77.438	38.312	101.562	1.00	82.25	C
ATOM	9481	CB	GLN	A1214	76.188	40.344	102.312	1.00	82.25	C
ATOM	9482	O	GLN	A1214	76.875	37.344	102.062	1.00	82.25	O
ATOM	9483	CG	GLN	A1214	76.312	41.844	102.062	1.00	82.25	C
ATOM	9484	CD	GLN	A1214	75.875	42.688	103.250	1.00	82.25	C
ATOM	9485	NE2	GLN	A1214	76.500	43.875	103.375	1.00	82.25	N
ATOM	9486	OE1	GLN	A1214	75.062	42.281	104.062	1.00	82.25	O
ATOM	9487	N	ALA	A1215	78.688	38.312	101.375	1.00	74.69	N
ATOM	9488	CA	ALA	A1215	79.562	37.312	102.000	1.00	74.69	C
ATOM	9489	C	ALA	A1215	80.688	37.938	102.875	1.00	74.69	C
ATOM	9490	CB	ALA	A1215	80.250	36.438	100.875	1.00	74.69	C
ATOM	9491	O	ALA	A1215	81.375	38.844	102.375	1.00	74.69	O
ATOM	9492	N	THR	A1216	80.625	37.344	104.062	1.00	69.50	N
ATOM	9493	CA	THR	A1216	81.750	37.688	104.875	1.00	69.50	C
ATOM	9494	C	THR	A1216	82.812	36.594	104.875	1.00	69.50	C
ATOM	9495	CB	THR	A1216	81.312	37.969	106.312	1.00	69.50	C
ATOM	9496	O	THR	A1216	83.938	36.812	105.312	1.00	69.50	O
ATOM	9497	CG2	THR	A1216	80.438	39.219	106.438	1.00	69.50	C
ATOM	9498	OG1	THR	A1216	80.562	36.844	106.812	1.00	69.50	O
ATOM	9499	N	ALA	A1217	82.562	35.219	104.000	1.00	61.44	N
ATOM	9500	CA	ALA	A1217	83.375	34.062	103.688	1.00	61.44	C
ATOM	9501	C	ALA	A1217	82.938	33.469	102.375	1.00	61.44	C
ATOM	9502	CB	ALA	A1217	83.250	33.031	104.812	1.00	61.44	C
ATOM	9503	O	ALA	A1217	81.938	33.812	101.812	1.00	61.44	O
ATOM	9504	N	MET	A1218	84.125	32.500	101.625	1.00	66.75	N
ATOM	9505	CA	MET	A1218	84.500	32.000	100.312	1.00	66.75	C
ATOM	9506	C	MET	A1218	83.312	32.062	99.375	1.00	66.75	C
ATOM	9507	CB	MET	A1218	85.000	30.562	100.438	1.00	66.75	C
ATOM	9508	O	MET	A1218	83.438	32.500	98.250	1.00	66.75	O
ATOM	9509	CG	MET	A1218	86.438	30.453	100.938	1.00	66.75	C
ATOM	9510	SD	MET	A1218	87.062	28.719	101.000	1.00	66.75	S
ATOM	9511	CE	MET	A1218	87.250	28.391	99.250	1.00	66.75	C
ATOM	9512	N	HIS	A1219	81.688	32.250	99.750	1.00	76.31	N
ATOM	9513	CA	HIS	A1219	80.688	32.188	98.750	1.00	76.31	C
ATOM	9514	C	HIS	A1219	79.250	32.531	99.312	1.00	76.31	C
ATOM	9515	CB	HIS	A1219	80.625	30.828	98.062	1.00	76.31	C
ATOM	9516	O	HIS	A1219	79.000	32.188	100.438	1.00	76.31	O
ATOM	9517	CG	HIS	A1219	81.938	30.438	97.438	1.00	76.31	C
ATOM	9518	CD2	HIS	A1219	82.875	29.500	97.812	1.00	76.31	C
ATOM	9519	ND1	HIS	A1219	82.500	31.031	96.312	1.00	76.31	N
ATOM	9520	CE1	HIS	A1219	83.625	30.484	96.062	1.00	76.31	C
ATOM	9521	NE2	HIS	A1219	83.875	29.547	96.938	1.00	76.31	N
ATOM	9522	N	GLY	A1220	78.750	33.344	98.688	1.00	81.25	N
ATOM	9523	CA	GLY	A1220	77.375	33.625	99.000	1.00	81.25	C
ATOM	9524	C	GLY	A1220	76.500	33.781	97.688	1.00	81.25	C
ATOM	9525	O	GLY	A1220	77.062	34.156	96.688	1.00	81.25	O
ATOM	9526	N	GLY	A1221	75.375	33.156	97.688	1.00	85.44	N
ATOM	9527	CA	GLY	A1221	74.438	33.219	96.562	1.00	85.44	C
ATOM	9528	C	GLY	A1221	73.000	33.406	97.000	1.00	85.44	C
ATOM	9529	O	GLY	A1221	72.688	33.094	98.125	1.00	85.44	O
ATOM	9530	N	SER	A1222	72.250	34.094	96.188	1.00	89.44	N
ATOM	9531	CA	SER	A1222	70.812	34.312	96.375	1.00	89.44	C
ATOM	9532	C	SER	A1222	70.062	34.188	95.000	1.00	89.44	C

ATOM	9533	CB	SER A1222	70.562	35.656	97.000	1.00	89.44	C
ATOM	9534	O	SER A1222	70.688	33.844	94.000	1.00	89.44	O
ATOM	9535	OG	SER A1222	69.188	35.781	97.375	1.00	89.44	O
ATOM	9536	N	SER A1223	68.812	34.156	95.062	1.00	90.31	N
ATOM	9537	CA	SER A1223	67.875	34.188	93.938	1.00	90.31	C
ATOM	9538	C	SER A1223	66.812	35.281	94.125	1.00	90.31	C
ATOM	9539	CB	SER A1223	67.250	32.844	93.688	1.00	90.31	C
ATOM	9540	O	SER A1223	66.688	35.812	95.250	1.00	90.31	O
ATOM	9541	OG	SER A1223	66.375	32.500	94.812	1.00	90.31	O
ATOM	9542	N	GLY A1224	66.188	35.625	93.000	1.00	89.00	N
ATOM	9543	CA	GLY A1224	65.125	36.625	93.062	1.00	89.00	C
ATOM	9544	C	GLY A1224	63.969	36.281	92.125	1.00	89.00	C
ATOM	9545	O	GLY A1224	64.125	35.469	91.250	1.00	89.00	O
ATOM	9546	N	TYR A1225	62.875	36.781	92.625	1.00	91.12	N
ATOM	9547	CA	TYR A1225	61.656	36.594	91.875	1.00	91.12	C
ATOM	9548	C	TYR A1225	60.750	37.844	91.938	1.00	91.12	C
ATOM	9549	CB	TYR A1225	60.875	35.375	92.438	1.00	91.12	C
ATOM	9550	O	TYR A1225	60.656	38.438	93.000	1.00	91.12	O
ATOM	9551	CG	TYR A1225	59.469	35.312	91.938	1.00	91.12	C
ATOM	9552	CD1	TYR A1225	58.406	35.781	92.750	1.00	91.12	C
ATOM	9553	CD2	TYR A1225	59.125	34.750	90.688	1.00	91.12	C
ATOM	9554	CE1	TYR A1225	57.094	35.719	92.312	1.00	91.12	C
ATOM	9555	CE2	TYR A1225	57.812	34.688	90.250	1.00	91.12	C
ATOM	9556	OH	TYR A1225	55.500	35.094	90.688	1.00	91.12	O
ATOM	9557	CZ	TYR A1225	56.812	35.156	91.062	1.00	91.12	C
ATOM	9558	N	VAL A1226	60.094	38.062	90.812	1.00	90.88	N
ATOM	9559	CA	VAL A1226	59.031	39.062	90.875	1.00	90.88	C
ATOM	9560	C	VAL A1226	57.969	38.750	89.812	1.00	90.88	C
ATOM	9561	CB	VAL A1226	59.625	40.500	90.562	1.00	90.88	C
ATOM	9562	O	VAL A1226	58.281	38.219	88.750	1.00	90.88	O
ATOM	9563	CG1	VAL A1226	60.062	40.625	89.125	1.00	90.88	C
ATOM	9564	CG2	VAL A1226	58.594	41.562	90.938	1.00	90.88	C
ATOM	9565	N	LEU A1227	56.750	38.812	90.250	1.00	90.69	N
ATOM	9566	CA	LEU A1227	55.625	38.750	89.312	1.00	90.69	C
ATOM	9567	C	LEU A1227	55.375	40.125	88.688	1.00	90.69	C
ATOM	9568	CB	LEU A1227	54.375	38.250	90.000	1.00	90.69	C
ATOM	9569	O	LEU A1227	55.094	41.094	89.375	1.00	90.69	O
ATOM	9570	CG	LEU A1227	53.156	38.000	89.062	1.00	90.69	C
ATOM	9571	CD1	LEU A1227	53.344	36.719	88.250	1.00	90.69	C
ATOM	9572	CD2	LEU A1227	51.875	37.906	89.938	1.00	90.69	C
ATOM	9573	N	LEU A1228	55.531	40.188	87.375	1.00	90.12	N
ATOM	9574	CA	LEU A1228	55.250	41.406	86.625	1.00	90.12	C
ATOM	9575	C	LEU A1228	53.844	41.344	86.062	1.00	90.12	C
ATOM	9576	CB	LEU A1228	56.281	41.594	85.562	1.00	90.12	C
ATOM	9577	O	LEU A1228	53.594	40.625	85.062	1.00	90.12	O
ATOM	9578	CG	LEU A1228	56.438	43.031	85.000	1.00	90.12	C
ATOM	9579	CD1	LEU A1228	57.281	43.844	86.000	1.00	90.12	C
ATOM	9580	CD2	LEU A1228	57.062	43.031	83.625	1.00	90.12	C
ATOM	9581	N	PRO A1229	52.875	42.000	86.688	1.00	90.19	N
ATOM	9582	CA	PRO A1229	51.531	41.969	86.125	1.00	90.19	C
ATOM	9583	C	PRO A1229	51.469	42.500	84.688	1.00	90.19	C
ATOM	9584	CB	PRO A1229	50.719	42.875	87.062	1.00	90.19	C
ATOM	9585	O	PRO A1229	52.312	43.250	84.250	1.00	90.19	O
ATOM	9586	CG	PRO A1229	51.656	43.125	88.250	1.00	90.19	C
ATOM	9587	CD	PRO A1229	53.031	42.750	87.812	1.00	90.19	C
ATOM	9588	N	ALA A1230	50.406	42.031	84.000	1.00	87.69	N
ATOM	9589	CA	ALA A1230	50.125	42.469	82.688	1.00	87.69	C
ATOM	9590	C	ALA A1230	50.156	43.969	82.562	1.00	87.69	C
ATOM	9591	CB	ALA A1230	48.781	41.906	82.188	1.00	87.69	C
ATOM	9592	O	ALA A1230	49.625	44.688	83.438	1.00	87.69	O
ATOM	9593	N	GLY A1231	50.875	44.531	81.625	1.00	88.75	N
ATOM	9594	CA	GLY A1231	50.875	45.938	81.312	1.00	88.75	C
ATOM	9595	C	GLY A1231	52.031	46.688	82.000	1.00	88.75	C
ATOM	9596	O	GLY A1231	52.156	47.906	81.750	1.00	88.75	O

ATOM	9597	N	ASN	A1232	52.844	46.125	82.750	1.00	90.12	N
ATOM	9598	CA	ASN	A1232	53.938	46.812	83.438	1.00	90.12	C
ATOM	9599	C	ASN	A1232	55.281	46.438	82.875	1.00	90.12	C
ATOM	9600	CB	ASN	A1232	53.875	46.469	84.938	1.00	90.12	C
ATOM	9601	O	ASN	A1232	55.500	45.312	82.438	1.00	90.12	O
ATOM	9602	CG	ASN	A1232	52.719	47.156	85.688	1.00	90.12	C
ATOM	9603	ND2	ASN	A1232	51.719	46.375	86.062	1.00	90.12	N
ATOM	9604	OD1	ASN	A1232	52.719	48.375	85.875	1.00	90.12	O
ATOM	9605	N	ALA	A1233	56.125	47.375	82.750	1.00	91.06	N
ATOM	9606	CA	ALA	A1233	57.531	47.219	82.375	1.00	91.06	C
ATOM	9607	C	ALA	A1233	58.438	47.344	83.562	1.00	91.06	C
ATOM	9608	CB	ALA	A1233	57.906	48.219	81.312	1.00	91.06	C
ATOM	9609	O	ALA	A1233	58.062	47.906	84.625	1.00	91.06	O
ATOM	9610	N	THR	A1234	59.656	46.656	83.562	1.00	91.56	N
ATOM	9611	CA	THR	A1234	60.625	46.812	84.688	1.00	91.56	C
ATOM	9612	C	THR	A1234	62.062	46.812	84.125	1.00	91.56	C
ATOM	9613	CB	THR	A1234	60.500	45.688	85.688	1.00	91.56	C
ATOM	9614	O	THR	A1234	62.281	46.250	83.000	1.00	91.56	O
ATOM	9615	CG2	THR	A1234	60.906	44.344	85.125	1.00	91.56	C
ATOM	9616	OG1	THR	A1234	61.312	46.000	86.812	1.00	91.56	O
ATOM	9617	N	THR	A1235	62.906	47.719	84.562	1.00	92.19	N
ATOM	9618	CA	THR	A1235	64.375	47.688	84.312	1.00	92.19	C
ATOM	9619	C	THR	A1235	65.125	46.969	85.438	1.00	92.19	C
ATOM	9620	CB	THR	A1235	64.938	49.094	84.125	1.00	92.19	C
ATOM	9621	O	THR	A1235	64.938	47.312	86.625	1.00	92.19	O
ATOM	9622	CG2	THR	A1235	66.375	49.062	83.812	1.00	92.19	C
ATOM	9623	OG1	THR	A1235	64.250	49.719	83.000	1.00	92.19	O
ATOM	9624	N	LEU	A1236	65.750	45.844	85.000	1.00	90.88	N
ATOM	9625	CA	LEU	A1236	66.625	45.125	86.000	1.00	90.88	C
ATOM	9626	C	LEU	A1236	68.062	45.656	85.938	1.00	90.88	C
ATOM	9627	CB	LEU	A1236	66.562	43.625	85.688	1.00	90.88	C
ATOM	9628	O	LEU	A1236	68.625	45.844	84.875	1.00	90.88	O
ATOM	9629	CG	LEU	A1236	65.188	42.938	85.812	1.00	90.88	C
ATOM	9630	CD1	LEU	A1236	65.312	41.500	85.250	1.00	90.88	C
ATOM	9631	CD2	LEU	A1236	64.750	42.938	87.250	1.00	90.88	C
ATOM	9632	N	SER	A1237	68.562	45.969	87.125	1.00	91.00	N
ATOM	9633	CA	SER	A1237	69.938	46.375	87.312	1.00	91.00	C
ATOM	9634	C	SER	A1237	70.688	45.438	88.250	1.00	91.00	C
ATOM	9635	CB	SER	A1237	70.062	47.812	87.812	1.00	91.00	C
ATOM	9636	O	SER	A1237	70.188	45.062	89.250	1.00	91.00	O
ATOM	9637	OG	SER	A1237	69.188	48.688	87.062	1.00	91.00	O
ATOM	9638	N	TYR	A1238	71.875	44.969	87.750	1.00	92.50	N
ATOM	9639	CA	TYR	A1238	72.750	44.062	88.500	1.00	92.50	C
ATOM	9640	C	TYR	A1238	74.062	44.750	88.938	1.00	92.50	C
ATOM	9641	CB	TYR	A1238	73.125	42.844	87.625	1.00	92.50	C
ATOM	9642	O	TYR	A1238	74.812	45.250	88.062	1.00	92.50	O
ATOM	9643	CG	TYR	A1238	74.000	41.844	88.312	1.00	92.50	C
ATOM	9644	CD1	TYR	A1238	74.938	41.094	87.562	1.00	92.50	C
ATOM	9645	CD2	TYR	A1238	73.938	41.625	89.625	1.00	92.50	C
ATOM	9646	CE1	TYR	A1238	75.750	40.156	88.188	1.00	92.50	C
ATOM	9647	CE2	TYR	A1238	74.750	40.688	90.312	1.00	92.50	C
ATOM	9648	OH	TYR	A1238	76.438	39.031	90.188	1.00	92.50	O
ATOM	9649	CZ	TYR	A1238	75.625	39.969	89.562	1.00	92.50	C
ATOM	9650	N	GLY	A1239	74.375	44.875	90.250	1.00	90.50	N
ATOM	9651	CA	GLY	A1239	75.500	45.562	90.750	1.00	90.50	C
ATOM	9652	C	GLY	A1239	76.062	44.969	92.062	1.00	90.50	C
ATOM	9653	O	GLY	A1239	75.438	44.094	92.625	1.00	90.50	O
ATOM	9654	N	GLY	A1240	77.125	45.438	92.500	1.00	88.50	N
ATOM	9655	CA	GLY	A1240	77.688	44.969	93.812	1.00	88.50	C
ATOM	9656	C	GLY	A1240	78.938	45.719	94.188	1.00	88.50	C
ATOM	9657	O	GLY	A1240	79.500	46.469	93.375	1.00	88.50	O
ATOM	9658	N	ASP	A1241	79.312	45.562	95.438	1.00	86.12	N
ATOM	9659	CA	ASP	A1241	80.625	45.969	96.000	1.00	86.12	C
ATOM	9660	C	ASP	A1241	81.500	44.750	96.312	1.00	86.12	C

ATOM	9661	CB	ASP	A1241	80.438	46.781	97.312	1.00	86.12	C
ATOM	9662	O	ASP	A1241	81.188	44.000	97.250	1.00	86.12	O
ATOM	9663	CG	ASP	A1241	81.250	48.031	97.312	1.00	86.12	C
ATOM	9664	OD1	ASP	A1241	82.312	48.062	96.688	1.00	86.12	O
ATOM	9665	OD2	ASP	A1241	80.875	49.000	98.062	1.00	86.12	O
ATOM	9666	N	LEU	A1242	82.812	44.656	95.812	1.00	85.75	N
ATOM	9667	CA	LEU	A1242	83.562	43.438	96.000	1.00	85.75	C
ATOM	9668	C	LEU	A1242	84.938	43.781	96.562	1.00	85.75	C
ATOM	9669	CB	LEU	A1242	83.688	42.688	94.688	1.00	85.75	C
ATOM	9670	O	LEU	A1242	85.625	44.656	96.000	1.00	85.75	O
ATOM	9671	CG	LEU	A1242	82.375	42.312	94.000	1.00	85.75	C
ATOM	9672	CD1	LEU	A1242	82.625	41.812	92.562	1.00	85.75	C
ATOM	9673	CD2	LEU	A1242	81.625	41.219	94.812	1.00	85.75	C
ATOM	9674	N	ASN	A1243	85.438	43.062	97.562	1.00	79.38	N
ATOM	9675	CA	ASN	A1243	86.750	43.125	98.125	1.00	79.38	C
ATOM	9676	C	ASN	A1243	87.375	41.719	98.125	1.00	79.38	C
ATOM	9677	CB	ASN	A1243	86.750	43.719	99.500	1.00	79.38	C
ATOM	9678	O	ASN	A1243	87.062	40.875	99.000	1.00	79.38	O
ATOM	9679	CG	ASN	A1243	88.188	43.969	100.062	1.00	79.38	C
ATOM	9680	ND2	ASN	A1243	88.250	44.688	101.188	1.00	79.38	N
ATOM	9681	OD1	ASN	A1243	89.125	43.500	99.438	1.00	79.38	O
ATOM	9682	N	HIS	A1244	88.188	41.594	97.250	1.00	83.38	N
ATOM	9683	CA	HIS	A1244	88.812	40.312	97.062	1.00	83.38	C
ATOM	9684	C	HIS	A1244	87.750	39.219	96.812	1.00	83.38	C
ATOM	9685	CB	HIS	A1244	89.688	39.938	98.250	1.00	83.38	C
ATOM	9686	O	HIS	A1244	87.875	38.125	97.375	1.00	83.38	O
ATOM	9687	CG	HIS	A1244	90.812	40.906	98.438	1.00	83.38	C
ATOM	9688	CD2	HIS	A1244	92.125	40.875	98.000	1.00	83.38	C
ATOM	9689	ND1	HIS	A1244	90.688	42.031	99.188	1.00	83.38	N
ATOM	9690	CE1	HIS	A1244	91.875	42.688	99.250	1.00	83.38	C
ATOM	9691	NE2	HIS	A1244	92.750	42.000	98.500	1.00	83.38	N
ATOM	9692	N	ALA	A1245	86.688	39.531	96.062	1.00	85.19	N
ATOM	9693	CA	ALA	A1245	85.562	38.625	95.688	1.00	85.19	C
ATOM	9694	C	ALA	A1245	85.125	38.906	94.250	1.00	85.19	C
ATOM	9695	CB	ALA	A1245	84.438	38.750	96.688	1.00	85.19	C
ATOM	9696	O	ALA	A1245	85.312	40.031	93.750	1.00	85.19	O
ATOM	9697	N	ASN	A1246	84.625	38.000	93.562	1.00	84.94	N
ATOM	9698	CA	ASN	A1246	84.000	38.156	92.250	1.00	84.94	C
ATOM	9699	C	ASN	A1246	82.500	37.844	92.250	1.00	84.94	C
ATOM	9700	CB	ASN	A1246	84.750	37.250	91.250	1.00	84.94	C
ATOM	9701	O	ASN	A1246	82.062	36.938	93.000	1.00	84.94	O
ATOM	9702	CG	ASN	A1246	86.188	37.656	91.000	1.00	84.94	C
ATOM	9703	ND2	ASN	A1246	87.062	36.656	90.875	1.00	84.94	N
ATOM	9704	OD1	ASN	A1246	86.500	38.844	90.938	1.00	84.94	O
ATOM	9705	N	ALA	A1247	81.750	38.781	91.562	1.00	89.56	N
ATOM	9706	CA	ALA	A1247	80.375	38.438	91.250	1.00	89.56	C
ATOM	9707	C	ALA	A1247	80.250	37.594	90.000	1.00	89.56	C
ATOM	9708	CB	ALA	A1247	79.500	39.719	91.125	1.00	89.56	C
ATOM	9709	O	ALA	A1247	81.125	37.625	89.125	1.00	89.56	O
ATOM	9710	N	VAL	A1248	79.188	36.906	90.000	1.00	85.81	N
ATOM	9711	CA	VAL	A1248	78.812	36.250	88.750	1.00	85.81	C
ATOM	9712	C	VAL	A1248	78.750	37.281	87.625	1.00	85.81	C
ATOM	9713	CB	VAL	A1248	77.500	35.438	88.812	1.00	85.81	C
ATOM	9714	O	VAL	A1248	78.000	38.281	87.688	1.00	85.81	O
ATOM	9715	CG1	VAL	A1248	77.312	34.531	87.625	1.00	85.81	C
ATOM	9716	CG2	VAL	A1248	77.500	34.656	90.125	1.00	85.81	C
ATOM	9717	N	PRO	A1249	79.625	37.156	86.688	1.00	86.75	N
ATOM	9718	CA	PRO	A1249	79.812	38.188	85.688	1.00	86.75	C
ATOM	9719	C	PRO	A1249	78.500	38.438	84.938	1.00	86.75	C
ATOM	9720	CB	PRO	A1249	80.875	37.625	84.812	1.00	86.75	C
ATOM	9721	O	PRO	A1249	78.250	39.562	84.438	1.00	86.75	O
ATOM	9722	CG	PRO	A1249	81.375	36.406	85.500	1.00	86.75	C
ATOM	9723	CD	PRO	A1249	80.438	36.094	86.625	1.00	86.75	C
ATOM	9724	N	ILE	A1250	77.562	37.375	84.625	1.00	90.12	N

ATOM	9725	CA	ILE	A1250	76.375	37.531	83.875	1.00	90.12	C
ATOM	9726	C	ILE	A1250	75.188	37.000	84.688	1.00	90.12	C
ATOM	9727	CB	ILE	A1250	76.438	36.781	82.500	1.00	90.12	C
ATOM	9728	O	ILE	A1250	75.188	35.875	85.125	1.00	90.12	O
ATOM	9729	CG1	ILE	A1250	77.562	37.344	81.688	1.00	90.12	C
ATOM	9730	CG2	ILE	A1250	75.125	36.844	81.750	1.00	90.12	C
ATOM	9731	CD1	ILE	A1250	77.812	36.625	80.312	1.00	90.12	C
ATOM	9732	N	LEU	A1251	74.250	37.906	84.812	1.00	89.88	N
ATOM	9733	CA	LEU	A1251	73.000	37.531	85.500	1.00	89.88	C
ATOM	9734	C	LEU	A1251	72.000	37.031	84.438	1.00	89.88	C
ATOM	9735	CB	LEU	A1251	72.438	38.688	86.250	1.00	89.88	C
ATOM	9736	O	LEU	A1251	71.750	37.656	83.438	1.00	89.88	O
ATOM	9737	CG	LEU	A1251	71.125	38.438	87.062	1.00	89.88	C
ATOM	9738	CD1	LEU	A1251	71.438	37.719	88.375	1.00	89.88	C
ATOM	9739	CD2	LEU	A1251	70.375	39.719	87.250	1.00	89.88	C
ATOM	9740	N	THR	A1252	71.438	35.938	84.750	1.00	91.38	N
ATOM	9741	CA	THR	A1252	70.375	35.406	83.875	1.00	91.38	C
ATOM	9742	C	THR	A1252	69.000	35.625	84.562	1.00	91.38	C
ATOM	9743	CB	THR	A1252	70.625	33.906	83.562	1.00	91.38	C
ATOM	9744	O	THR	A1252	68.812	35.312	85.688	1.00	91.38	O
ATOM	9745	CG2	THR	A1252	69.500	33.406	82.625	1.00	91.38	C
ATOM	9746	OG1	THR	A1252	71.875	33.719	83.000	1.00	91.38	O
ATOM	9747	N	VAL	A1253	68.188	36.188	83.750	1.00	89.62	N
ATOM	9748	CA	VAL	A1253	66.812	36.344	84.188	1.00	89.62	C
ATOM	9749	C	VAL	A1253	65.875	35.594	83.250	1.00	89.62	C
ATOM	9750	CB	VAL	A1253	66.375	37.844	84.250	1.00	89.62	C
ATOM	9751	O	VAL	A1253	66.000	35.719	82.000	1.00	89.62	O
ATOM	9752	CG1	VAL	A1253	64.938	38.000	84.750	1.00	89.62	C
ATOM	9753	CG2	VAL	A1253	67.375	38.625	85.125	1.00	89.62	C
ATOM	9754	N	LEU	A1254	65.125	34.906	83.938	1.00	90.06	N
ATOM	9755	CA	LEU	A1254	64.125	34.156	83.125	1.00	90.06	C
ATOM	9756	C	LEU	A1254	62.750	34.781	83.250	1.00	90.06	C
ATOM	9757	CB	LEU	A1254	64.062	32.688	83.625	1.00	90.06	C
ATOM	9758	O	LEU	A1254	62.250	35.094	84.312	1.00	90.06	O
ATOM	9759	CG	LEU	A1254	65.375	31.906	83.562	1.00	90.06	C
ATOM	9760	CD1	LEU	A1254	65.188	30.562	84.250	1.00	90.06	C
ATOM	9761	CD2	LEU	A1254	65.875	31.734	82.125	1.00	90.06	C
ATOM	9762	N	LEU	A1255	62.125	34.938	82.000	1.00	87.62	N
ATOM	9763	CA	LEU	A1255	60.781	35.438	81.938	1.00	87.62	C
ATOM	9764	C	LEU	A1255	59.812	34.344	81.562	1.00	87.62	C
ATOM	9765	CB	LEU	A1255	60.656	36.625	81.000	1.00	87.62	C
ATOM	9766	O	LEU	A1255	60.000	33.719	80.500	1.00	87.62	O
ATOM	9767	CG	LEU	A1255	61.531	37.844	81.312	1.00	87.62	C
ATOM	9768	CD1	LEU	A1255	62.125	38.438	80.000	1.00	87.62	C
ATOM	9769	CD2	LEU	A1255	60.750	38.875	82.062	1.00	87.62	C
ATOM	9770	N	PHE	A1256	58.906	34.219	82.500	1.00	86.62	N
ATOM	9771	CA	PHE	A1256	57.875	33.250	82.188	1.00	86.62	C
ATOM	9772	C	PHE	A1256	56.500	33.906	82.125	1.00	86.62	C
ATOM	9773	CB	PHE	A1256	57.906	32.094	83.188	1.00	86.62	C
ATOM	9774	O	PHE	A1256	56.188	34.750	83.000	1.00	86.62	O
ATOM	9775	CG	PHE	A1256	59.219	31.375	83.250	1.00	86.62	C
ATOM	9776	CD1	PHE	A1256	59.531	30.375	82.312	1.00	86.62	C
ATOM	9777	CD2	PHE	A1256	60.125	31.688	84.250	1.00	86.62	C
ATOM	9778	CE1	PHE	A1256	60.750	29.703	82.375	1.00	86.62	C
ATOM	9779	CE2	PHE	A1256	61.375	31.031	84.312	1.00	86.62	C
ATOM	9780	CZ	PHE	A1256	61.656	30.031	83.375	1.00	86.62	C
ATOM	9781	N	LYS	A1257	55.719	33.531	81.000	1.00	84.81	N
ATOM	9782	CA	LYS	A1257	54.312	33.906	81.125	1.00	84.81	C
ATOM	9783	C	LYS	A1257	53.656	33.250	82.312	1.00	84.81	C
ATOM	9784	CB	LYS	A1257	53.562	33.500	79.875	1.00	84.81	C
ATOM	9785	O	LYS	A1257	53.812	32.031	82.562	1.00	84.81	O
ATOM	9786	CG	LYS	A1257	52.125	34.000	79.812	1.00	84.81	C
ATOM	9787	CD	LYS	A1257	51.469	33.625	78.438	1.00	84.81	C
ATOM	9788	CE	LYS	A1257	50.031	34.156	78.375	1.00	84.81	C

```
ATOM   9789  NZ  LYS A1257      49.375  33.812  77.062  1.00 84.81           N
ATOM   9790  N   ALA A1258      53.062  34.031  83.375  1.00 77.50           N
ATOM   9791  CA  ALA A1258      52.375  33.469  84.500  1.00 77.50           C
ATOM   9792  C   ALA A1258      51.125  32.719  84.062  1.00 77.50           C
ATOM   9793  CB  ALA A1258      52.000  34.594  85.500  1.00 77.50           C
ATOM   9794  O   ALA A1258      50.500  33.062  83.062  1.00 77.50           O
TER    9795      ALA A1258
ENDMDL
END
```
