# Supplementary material for: The TolC and Lipopolysaccharide-Specific Escherichia coli Bacteriophage TLS—the Tlsvirus Archetype Virus
Source: Phage (New Rochelle). 2024 Sep 16;5(3):173–83. doi: 10.1089/phage.2023.0041 (PMC11447400; doi:10.1089/phage.2023.0041)
Supplement: Supplementary Data S2 [file phage.2023.0041_supp_datas2.pdf]

```
MODEL      1
HEADER    PHAGE PROTEIN                           15-NOV-23
TITLE     ALPHAFOLD2 PREDICTED STRUCTURE OF PHAGE T1 TAIL FIBRE

ATOM      1  N   MET A   1      23.141  28.188 -82.000  1.00 34.31           N
ATOM      2  CA  MET A   1      23.203  28.594 -80.625  1.00 34.31           C
ATOM      3  C   MET A   1      24.516  28.125 -80.000  1.00 34.31           C
ATOM      4  CB  MET A   1      22.031  28.016 -79.812  1.00 34.31           C
ATOM      5  O   MET A   1      24.781  26.938 -79.875  1.00 34.31           O
ATOM      6  CG  MET A   1      21.109  29.078 -79.250  1.00 34.31           C
ATOM      7  SD  MET A   1      19.781  28.359 -78.188  1.00 34.31           S
ATOM      8  CE  MET A   1      18.844  29.859 -77.812  1.00 34.31           C
ATOM      9  N   ILE A   2      25.656  28.656 -80.500  1.00 38.03           N
ATOM     10  CA  ILE A   2      27.094  28.812 -80.625  1.00 38.03           C
ATOM     11  C   ILE A   2      27.719  28.938 -79.188  1.00 38.03           C
ATOM     12  CB  ILE A   2      27.469  30.047 -81.500  1.00 38.03           C
ATOM     13  O   ILE A   2      27.312  29.797 -78.438  1.00 38.03           O
ATOM     14  CG1 ILE A   2      26.844  29.906 -82.875  1.00 38.03           C
ATOM     15  CG2 ILE A   2      28.984  30.203 -81.562  1.00 38.03           C
ATOM     16  CD1 ILE A   2      26.984  31.156 -83.750  1.00 38.03           C
ATOM     17  N   GLN A   3      28.375  27.797 -78.562  1.00 43.75           N
ATOM     18  CA  GLN A   3      29.578  28.156 -77.812  1.00 43.75           C
ATOM     19  C   GLN A   3      30.703  27.156 -78.062  1.00 43.75           C
ATOM     20  CB  GLN A   3      29.250  28.266 -76.312  1.00 43.75           C
ATOM     21  O   GLN A   3      30.500  25.953 -77.875  1.00 43.75           O
ATOM     22  CG  GLN A   3      29.391  29.672 -75.750  1.00 43.75           C
ATOM     23  CD  GLN A   3      29.125  29.734 -74.250  1.00 43.75           C
ATOM     24  NE2 GLN A   3      29.453  30.875 -73.625  1.00 43.75           N
ATOM     25  OE1 GLN A   3      28.641  28.766 -73.688  1.00 43.75           O
ATOM     26  N   LYS A   4      31.500  27.266 -79.125  1.00 41.59           N
ATOM     27  CA  LYS A   4      32.875  27.125 -79.562  1.00 41.59           C
ATOM     28  C   LYS A   4      33.406  25.719 -79.312  1.00 41.59           C
ATOM     29  CB  LYS A   4      33.781  28.156 -78.875  1.00 41.59           C
ATOM     30  O   LYS A   4      33.438  25.250 -78.188  1.00 41.59           O
ATOM     31  CG  LYS A   4      33.750  29.531 -79.562  1.00 41.59           C
ATOM     32  CD  LYS A   4      34.875  30.406 -79.000  1.00 41.59           C
ATOM     33  CE  LYS A   4      34.875  31.781 -79.688  1.00 41.59           C
ATOM     34  NZ  LYS A   4      35.969  32.656 -79.188  1.00 41.59           N
ATOM     35  N   VAL A   5      33.219  24.766 -80.188  1.00 47.31           N
ATOM     36  CA  VAL A   5      34.000  23.594 -80.625  1.00 47.31           C
ATOM     37  C   VAL A   5      35.469  23.938 -80.750  1.00 47.31           C
ATOM     38  CB  VAL A   5      33.469  23.000 -81.938  1.00 47.31           C
ATOM     39  O   VAL A   5      35.844  24.906 -81.438  1.00 47.31           O
ATOM     40  CG1 VAL A   5      34.250  21.750 -82.312  1.00 47.31           C
ATOM     41  CG2 VAL A   5      31.984  22.688 -81.875  1.00 47.31           C
ATOM     42  N   ILE A   6      36.219  23.812 -79.688  1.00 51.00           N
ATOM     43  CA  ILE A   6      37.688  23.766 -79.812  1.00 51.00           C
ATOM     44  C   ILE A   6      38.094  22.422 -80.438  1.00 51.00           C
ATOM     45  CB  ILE A   6      38.375  23.953 -78.438  1.00 51.00           C
ATOM     46  O   ILE A   6      37.688  21.359 -79.938  1.00 51.00           O
ATOM     47  CG1 ILE A   6      37.938  25.281 -77.812  1.00 51.00           C
ATOM     48  CG2 ILE A   6      39.906  23.891 -78.625  1.00 51.00           C
ATOM     49  CD1 ILE A   6      38.594  25.562 -76.438  1.00 51.00           C
ATOM     50  N   SER A   7      38.219  22.266 -81.750  1.00 43.75           N
ATOM     51  CA  SER A   7      38.969  21.406 -82.688  1.00 43.75           C
ATOM     52  C   SER A   7      40.406  21.891 -82.812  1.00 43.75           C
ATOM     53  CB  SER A   7      38.281  21.344 -84.062  1.00 43.75           C
ATOM     54  O   SER A   7      40.656  23.094 -82.938  1.00 43.75           O
ATOM     55  OG  SER A   7      38.312  22.625 -84.688  1.00 43.75           O
ATOM     56  N   GLY A   8      41.406  21.234 -82.188  1.00 42.06           N
ATOM     57  CA  GLY A   8      42.812  21.250 -82.500  1.00 42.06           C
ATOM     58  C   GLY A   8      43.312  19.906 -83.000  1.00 42.06           C
ATOM     59  O   GLY A   8      42.906  18.859 -82.500  1.00 42.06           O
ATOM     60  N   SER A   9      43.500  19.750 -84.375  1.00 37.34           N
```

ATOM	61	CA	SER	A	9	44.406	19.234	-85.375	1.00	37.34	C
ATOM	62	C	SER	A	9	45.844	19.141	-84.812	1.00	37.34	C
ATOM	63	CB	SER	A	9	44.406	20.125	-86.562	1.00	37.34	C
ATOM	64	O	SER	A	9	46.281	20.016	-84.062	1.00	37.34	O
ATOM	65	OG	SER	A	9	44.562	21.500	-86.250	1.00	37.34	O
ATOM	66	N	LYS	A	10	46.594	17.922	-84.688	1.00	32.50	N
ATOM	67	CA	LYS	A	10	47.844	17.516	-85.312	1.00	32.50	C
ATOM	68	C	LYS	A	10	48.969	17.562	-84.250	1.00	32.50	C
ATOM	69	CB	LYS	A	10	48.188	18.406	-86.500	1.00	32.50	C
ATOM	70	O	LYS	A	10	49.156	18.578	-83.562	1.00	32.50	O
ATOM	71	CG	LYS	A	10	48.000	17.719	-87.812	1.00	32.50	C
ATOM	72	CD	LYS	A	10	48.500	18.594	-89.000	1.00	32.50	C
ATOM	73	CE	LYS	A	10	48.344	17.891	-90.312	1.00	32.50	C
ATOM	74	NZ	LYS	A	10	48.844	18.750	-91.438	1.00	32.50	N
ATOM	75	N	GLY	A	11	49.562	16.328	-83.688	1.00	38.12	N
ATOM	76	CA	GLY	A	11	50.938	15.914	-83.750	1.00	38.12	C
ATOM	77	C	GLY	A	11	51.281	14.766	-82.812	1.00	38.12	C
ATOM	78	O	GLY	A	11	51.000	14.836	-81.625	1.00	38.12	O
ATOM	79	N	GLY	A	12	50.938	13.500	-83.000	1.00	44.03	N
ATOM	80	CA	GLY	A	12	51.469	12.164	-82.875	1.00	44.03	C
ATOM	81	C	GLY	A	12	52.219	11.953	-81.562	1.00	44.03	C
ATOM	82	O	GLY	A	12	53.281	12.547	-81.312	1.00	44.03	O
ATOM	83	N	SER	A	13	51.594	12.047	-80.312	1.00	44.69	N
ATOM	84	CA	SER	A	13	52.125	11.922	-78.938	1.00	44.69	C
ATOM	85	C	SER	A	13	51.531	10.719	-78.250	1.00	44.69	C
ATOM	86	CB	SER	A	13	51.875	13.195	-78.188	1.00	44.69	C
ATOM	87	O	SER	A	13	50.344	10.430	-78.375	1.00	44.69	O
ATOM	88	OG	SER	A	13	51.500	14.266	-79.000	1.00	44.69	O
ATOM	89	N	GLN	A	14	52.219	9.492	-78.188	1.00	51.31	N
ATOM	90	CA	GLN	A	14	52.281	8.352	-77.250	1.00	51.31	C
ATOM	91	C	GLN	A	14	51.281	8.500	-76.125	1.00	51.31	C
ATOM	92	CB	GLN	A	14	53.688	8.180	-76.688	1.00	51.31	C
ATOM	93	O	GLN	A	14	51.188	9.570	-75.562	1.00	51.31	O
ATOM	94	CG	GLN	A	14	54.625	7.387	-77.625	1.00	51.31	C
ATOM	95	CD	GLN	A	14	55.844	6.836	-76.875	1.00	51.31	C
ATOM	96	NE2	GLN	A	14	56.375	5.719	-77.375	1.00	51.31	N
ATOM	97	OE1	GLN	A	14	56.281	7.402	-75.875	1.00	51.31	O
ATOM	98	N	LYS	A	15	50.062	7.980	-76.250	1.00	54.88	N
ATOM	99	CA	LYS	A	15	49.125	7.828	-75.125	1.00	54.88	C
ATOM	100	C	LYS	A	15	49.906	7.668	-73.812	1.00	54.88	C
ATOM	101	CB	LYS	A	15	48.219	6.629	-75.312	1.00	54.88	C
ATOM	102	O	LYS	A	15	50.812	6.852	-73.688	1.00	54.88	O
ATOM	103	CG	LYS	A	15	47.031	6.926	-76.250	1.00	54.88	C
ATOM	104	CD	LYS	A	15	46.094	5.727	-76.312	1.00	54.88	C
ATOM	105	CE	LYS	A	15	44.938	5.996	-77.250	1.00	54.88	C
ATOM	106	NZ	LYS	A	15	43.938	4.875	-77.312	1.00	54.88	N
ATOM	107	N	PRO	A	16	50.188	8.664	-72.938	1.00	63.81	N
ATOM	108	CA	PRO	A	16	50.688	8.406	-71.562	1.00	63.81	C
ATOM	109	C	PRO	A	16	50.156	7.113	-70.938	1.00	63.81	C
ATOM	110	CB	PRO	A	16	50.188	9.625	-70.750	1.00	63.81	C
ATOM	111	O	PRO	A	16	49.000	6.727	-71.250	1.00	63.81	O
ATOM	112	CG	PRO	A	16	49.344	10.391	-71.750	1.00	63.81	C
ATOM	113	CD	PRO	A	16	49.344	9.664	-73.062	1.00	63.81	C
ATOM	114	N	HIS	A	17	51.094	5.984	-70.812	1.00	76.88	N
ATOM	115	CA	HIS	A	17	50.812	4.789	-70.062	1.00	76.88	C
ATOM	116	C	HIS	A	17	49.938	5.121	-68.812	1.00	76.88	C
ATOM	117	CB	HIS	A	17	52.094	4.109	-69.562	1.00	76.88	C
ATOM	118	O	HIS	A	17	50.250	6.031	-68.062	1.00	76.88	O
ATOM	119	CG	HIS	A	17	51.875	2.887	-68.750	1.00	76.88	C
ATOM	120	CD2	HIS	A	17	52.219	2.600	-67.438	1.00	76.88	C
ATOM	121	ND1	HIS	A	17	51.188	1.782	-69.188	1.00	76.88	N
ATOM	122	CE1	HIS	A	17	51.156	0.865	-68.250	1.00	76.88	C
ATOM	123	NE2	HIS	A	17	51.781	1.336	-67.188	1.00	76.88	N
ATOM	124	N	ASN	A	18	48.625	4.730	-68.812	1.00	78.69	N

```
ATOM    125  CA  ASN A  18      47.750   4.762 -67.688  1.00 78.69           C
ATOM    126  C   ASN A  18      48.125   3.699 -66.625  1.00 78.69           C
ATOM    127  CB  ASN A  18      46.281   4.582 -68.062  1.00 78.69           C
ATOM    128  O   ASN A  18      48.125   2.506 -66.938  1.00 78.69           O
ATOM    129  CG  ASN A  18      45.562   5.902 -68.312  1.00 78.69           C
ATOM    130  ND2 ASN A  18      44.312   5.840 -68.750  1.00 78.69           N
ATOM    131  OD1 ASN A  18      46.156   6.977 -68.125  1.00 78.69           O
ATOM    132  N   PRO A  19      48.938   4.039 -65.688  1.00 81.94           N
ATOM    133  CA  PRO A  19      49.281   3.074 -64.625  1.00 81.94           C
ATOM    134  C   PRO A  19      48.125   2.129 -64.312  1.00 81.94           C
ATOM    135  CB  PRO A  19      49.625   3.959 -63.438  1.00 81.94           C
ATOM    136  O   PRO A  19      46.969   2.555 -64.250  1.00 81.94           O
ATOM    137  CG  PRO A  19      49.812   5.324 -64.000  1.00 81.94           C
ATOM    138  CD  PRO A  19      49.094   5.398 -65.312  1.00 81.94           C
ATOM    139  N   VAL A  20      48.312   0.782 -64.375  1.00 84.25           N
ATOM    140  CA  VAL A  20      47.406  -0.282 -64.000  1.00 84.25           C
ATOM    141  C   VAL A  20      47.750  -0.802 -62.594  1.00 84.25           C
ATOM    142  CB  VAL A  20      47.406  -1.440 -65.000  1.00 84.25           C
ATOM    143  O   VAL A  20      48.938  -1.040 -62.312  1.00 84.25           O
ATOM    144  CG1 VAL A  20      46.438  -2.545 -64.625  1.00 84.25           C
ATOM    145  CG2 VAL A  20      47.062  -0.925 -66.438  1.00 84.25           C
ATOM    146  N   GLU A  21      46.750  -0.701 -61.688  1.00 91.12           N
ATOM    147  CA  GLU A  21      46.875  -1.209 -60.344  1.00 91.12           C
ATOM    148  C   GLU A  21      46.344  -2.633 -60.250  1.00 91.12           C
ATOM    149  CB  GLU A  21      46.125  -0.293 -59.375  1.00 91.12           C
ATOM    150  O   GLU A  21      45.156  -2.883 -60.531  1.00 91.12           O
ATOM    151  CG  GLU A  21      46.250  -0.729 -57.906  1.00 91.12           C
ATOM    152  CD  GLU A  21      45.562   0.229 -56.938  1.00 91.12           C
ATOM    153  OE1 GLU A  21      45.625  -0.008 -55.719  1.00 91.12           O
ATOM    154  OE2 GLU A  21      44.969   1.223 -57.406  1.00 91.12           O
ATOM    155  N   MET A  22      47.219  -3.719 -59.969  1.00 90.31           N
ATOM    156  CA  MET A  22      46.781  -5.078 -59.656  1.00 90.31           C
ATOM    157  C   MET A  22      45.750  -5.078 -58.531  1.00 90.31           C
ATOM    158  CB  MET A  22      47.969  -5.957 -59.312  1.00 90.31           C
ATOM    159  O   MET A  22      45.906  -4.332 -57.562  1.00 90.31           O
ATOM    160  CG  MET A  22      47.594  -7.414 -59.062  1.00 90.31           C
ATOM    161  SD  MET A  22      49.094  -8.461 -58.719  1.00 90.31           S
ATOM    162  CE  MET A  22      48.281 -10.078 -58.500  1.00 90.31           C
ATOM    163  N   GLU A  23      44.594  -5.621 -58.688  1.00 87.56           N
ATOM    164  CA  GLU A  23      43.625  -5.746 -57.625  1.00 87.56           C
ATOM    165  C   GLU A  23      44.219  -6.480 -56.406  1.00 87.56           C
ATOM    166  CB  GLU A  23      42.375  -6.469 -58.125  1.00 87.56           C
ATOM    167  O   GLU A  23      45.094  -7.328 -56.562  1.00 87.56           O
ATOM    168  CG  GLU A  23      41.562  -5.676 -59.125  1.00 87.56           C
ATOM    169  CD  GLU A  23      40.250  -6.340 -59.500  1.00 87.56           C
ATOM    170  OE1 GLU A  23      39.438  -5.730 -60.250  1.00 87.56           O
ATOM    171  OE2 GLU A  23      40.000  -7.473 -59.031  1.00 87.56           O
ATOM    172  N   ASP A  24      43.875  -6.016 -55.281  1.00 86.94           N
ATOM    173  CA  ASP A  24      44.281  -6.750 -54.094  1.00 86.94           C
ATOM    174  C   ASP A  24      43.906  -8.227 -54.188  1.00 86.94           C
ATOM    175  CB  ASP A  24      43.656  -6.133 -52.844  1.00 86.94           C
ATOM    176  O   ASP A  24      42.719  -8.555 -54.344  1.00 86.94           O
ATOM    177  CG  ASP A  24      44.406  -4.883 -52.375  1.00 86.94           C
ATOM    178  OD1 ASP A  24      45.469  -4.559 -52.938  1.00 86.94           O
ATOM    179  OD2 ASP A  24      43.906  -4.219 -51.438  1.00 86.94           O
ATOM    180  N   ASN A  25      44.875  -9.141 -54.312  1.00 88.19           N
ATOM    181  CA  ASN A  25      44.531 -10.555 -54.469  1.00 88.19           C
ATOM    182  C   ASN A  25      44.969 -11.352 -53.219  1.00 88.19           C
ATOM    183  CB  ASN A  25      45.188 -11.141 -55.719  1.00 88.19           C
ATOM    184  O   ASN A  25      44.844 -12.578 -53.188  1.00 88.19           O
ATOM    185  CG  ASN A  25      46.688 -11.062 -55.688  1.00 88.19           C
ATOM    186  ND2 ASN A  25      47.344 -11.797 -56.594  1.00 88.19           N
ATOM    187  OD1 ASN A  25      47.281 -10.344 -54.875  1.00 88.19           O
ATOM    188  N   LEU A  26      45.625 -10.664 -52.312  1.00 89.00           N
```

```
ATOM    189  CA  LEU A  26      45.844 -11.250 -50.969  1.00 89.00           C
ATOM    190  C   LEU A  26      44.750 -10.805 -50.000  1.00 89.00           C
ATOM    191  CB  LEU A  26      47.219 -10.859 -50.438  1.00 89.00           C
ATOM    192  O   LEU A  26      44.844  -9.711 -49.438  1.00 89.00           O
ATOM    193  CG  LEU A  26      48.438 -11.438 -51.188  1.00 89.00           C
ATOM    194  CD1 LEU A  26      49.719 -10.820 -50.656  1.00 89.00           C
ATOM    195  CD2 LEU A  26      48.469 -12.961 -51.031  1.00 89.00           C
ATOM    196  N   ILE A  27      43.688 -11.602 -49.969  1.00 80.44           N
ATOM    197  CA  ILE A  27      42.562 -11.297 -49.094  1.00 80.44           C
ATOM    198  C   ILE A  27      42.469 -12.297 -47.969  1.00 80.44           C
ATOM    199  CB  ILE A  27      41.219 -11.305 -49.906  1.00 80.44           C
ATOM    200  O   ILE A  27      42.469 -13.508 -48.188  1.00 80.44           O
ATOM    201  CG1 ILE A  27      41.312 -10.359 -51.125  1.00 80.44           C
ATOM    202  CG2 ILE A  27      40.062 -10.922 -49.000  1.00 80.44           C
ATOM    203  CD1 ILE A  27      40.125 -10.438 -52.062  1.00 80.44           C
ATOM    204  N   SER A  28      42.688 -11.781 -46.750  1.00 78.44           N
ATOM    205  CA  SER A  28      42.562 -12.648 -45.594  1.00 78.44           C
ATOM    206  C   SER A  28      41.094 -13.016 -45.312  1.00 78.44           C
ATOM    207  CB  SER A  28      43.156 -11.961 -44.344  1.00 78.44           C
ATOM    208  O   SER A  28      40.219 -12.156 -45.375  1.00 78.44           O
ATOM    209  OG  SER A  28      44.531 -11.727 -44.531  1.00 78.44           O
ATOM    210  N   ILE A  29      40.781 -14.352 -45.344  1.00 74.81           N
ATOM    211  CA  ILE A  29      39.469 -14.781 -44.844  1.00 74.81           C
ATOM    212  C   ILE A  29      39.594 -15.289 -43.406  1.00 74.81           C
ATOM    213  CB  ILE A  29      38.875 -15.867 -45.750  1.00 74.81           C
ATOM    214  O   ILE A  29      40.312 -16.250 -43.156  1.00 74.81           O
ATOM    215  CG1 ILE A  29      38.750 -15.367 -47.188  1.00 74.81           C
ATOM    216  CG2 ILE A  29      37.500 -16.312 -45.219  1.00 74.81           C
ATOM    217  CD1 ILE A  29      38.344 -16.438 -48.188  1.00 74.81           C
ATOM    218  N   ASN A  30      39.219 -14.508 -42.531  1.00 77.94           N
ATOM    219  CA  ASN A  30      39.219 -14.930 -41.156  1.00 77.94           C
ATOM    220  C   ASN A  30      37.844 -15.391 -40.719  1.00 77.94           C
ATOM    221  CB  ASN A  30      39.719 -13.805 -40.250  1.00 77.94           C
ATOM    222  O   ASN A  30      36.812 -14.766 -41.031  1.00 77.94           O
ATOM    223  CG  ASN A  30      41.188 -13.500 -40.469  1.00 77.94           C
ATOM    224  ND2 ASN A  30      41.562 -12.234 -40.281  1.00 77.94           N
ATOM    225  OD1 ASN A  30      41.969 -14.391 -40.781  1.00 77.94           O
ATOM    226  N   LYS A  31      37.781 -16.625 -40.250  1.00 81.44           N
ATOM    227  CA  LYS A  31      36.531 -17.141 -39.719  1.00 81.44           C
ATOM    228  C   LYS A  31      36.531 -17.109 -38.188  1.00 81.44           C
ATOM    229  CB  LYS A  31      36.312 -18.578 -40.188  1.00 81.44           C
ATOM    230  O   LYS A  31      37.594 -17.234 -37.562  1.00 81.44           O
ATOM    231  CG  LYS A  31      36.094 -18.703 -41.688  1.00 81.44           C
ATOM    232  CD  LYS A  31      35.750 -20.125 -42.094  1.00 81.44           C
ATOM    233  CE  LYS A  31      35.531 -20.250 -43.594  1.00 81.44           C
ATOM    234  NZ  LYS A  31      35.219 -21.641 -44.000  1.00 81.44           N
ATOM    235  N   ILE A  32      35.469 -16.734 -37.719  1.00 85.75           N
ATOM    236  CA  ILE A  32      35.281 -16.766 -36.281  1.00 85.75           C
ATOM    237  C   ILE A  32      34.594 -18.094 -35.906  1.00 85.75           C
ATOM    238  CB  ILE A  32      34.438 -15.570 -35.781  1.00 85.75           C
ATOM    239  O   ILE A  32      33.594 -18.469 -36.469  1.00 85.75           O
ATOM    240  CG1 ILE A  32      35.156 -14.258 -36.062  1.00 85.75           C
ATOM    241  CG2 ILE A  32      34.125 -15.719 -34.312  1.00 85.75           C
ATOM    242  CD1 ILE A  32      34.375 -13.016 -35.719  1.00 85.75           C
ATOM    243  N   LYS A  33      35.344 -18.859 -35.094  1.00 85.62           N
ATOM    244  CA  LYS A  33      34.781 -20.094 -34.531  1.00 85.62           C
ATOM    245  C   LYS A  33      34.562 -19.938 -33.031  1.00 85.62           C
ATOM    246  CB  LYS A  33      35.719 -21.266 -34.781  1.00 85.62           C
ATOM    247  O   LYS A  33      35.469 -19.641 -32.250  1.00 85.62           O
ATOM    248  CG  LYS A  33      36.000 -21.547 -36.250  1.00 85.62           C
ATOM    249  CD  LYS A  33      36.969 -22.703 -36.438  1.00 85.62           C
ATOM    250  CE  LYS A  33      37.281 -22.938 -37.906  1.00 85.62           C
ATOM    251  NZ  LYS A  33      38.219 -24.062 -38.094  1.00 85.62           N
ATOM    252  N   ILE A  34      33.250 -20.141 -32.594  1.00 89.31           N
```

ATOM	253	CA	ILE	A	34	32.875	-19.969	-31.188	1.00	89.31	C
ATOM	254	C	ILE	A	34	32.219	-21.234	-30.672	1.00	89.31	C
ATOM	255	CB	ILE	A	34	31.938	-18.750	-31.000	1.00	89.31	C
ATOM	256	O	ILE	A	34	31.328	-21.781	-31.328	1.00	89.31	O
ATOM	257	CG1	ILE	A	34	32.625	-17.469	-31.516	1.00	89.31	C
ATOM	258	CG2	ILE	A	34	31.516	-18.609	-29.547	1.00	89.31	C
ATOM	259	CD1	ILE	A	34	31.688	-16.266	-31.547	1.00	89.31	C
ATOM	260	N	LEU	A	35	32.781	-21.703	-29.609	1.00	88.50	N
ATOM	261	CA	LEU	A	35	32.188	-22.828	-28.891	1.00	88.50	C
ATOM	262	C	LEU	A	35	31.562	-22.359	-27.578	1.00	88.50	C
ATOM	263	CB	LEU	A	35	33.219	-23.922	-28.625	1.00	88.50	C
ATOM	264	O	LEU	A	35	32.281	-21.891	-26.688	1.00	88.50	O
ATOM	265	CG	LEU	A	35	32.688	-25.203	-27.984	1.00	88.50	C
ATOM	266	CD1	LEU	A	35	31.672	-25.891	-28.906	1.00	88.50	C
ATOM	267	CD2	LEU	A	35	33.844	-26.156	-27.656	1.00	88.50	C
ATOM	268	N	LEU	A	36	30.172	-22.500	-27.422	1.00	92.56	N
ATOM	269	CA	LEU	A	36	29.438	-22.031	-26.234	1.00	92.56	C
ATOM	270	C	LEU	A	36	28.922	-23.203	-25.422	1.00	92.56	C
ATOM	271	CB	LEU	A	36	28.281	-21.125	-26.656	1.00	92.56	C
ATOM	272	O	LEU	A	36	28.359	-24.141	-25.969	1.00	92.56	O
ATOM	273	CG	LEU	A	36	28.641	-19.844	-27.406	1.00	92.56	C
ATOM	274	CD1	LEU	A	36	27.406	-19.234	-28.078	1.00	92.56	C
ATOM	275	CD2	LEU	A	36	29.281	-18.828	-26.453	1.00	92.56	C
ATOM	276	N	ALA	A	37	29.297	-23.188	-24.094	1.00	92.31	N
ATOM	277	CA	ALA	A	37	28.562	-24.062	-23.172	1.00	92.31	C
ATOM	278	C	ALA	A	37	27.203	-23.469	-22.828	1.00	92.31	C
ATOM	279	CB	ALA	A	37	29.375	-24.297	-21.906	1.00	92.31	C
ATOM	280	O	ALA	A	37	27.125	-22.453	-22.156	1.00	92.31	O
ATOM	281	N	VAL	A	38	26.125	-24.125	-23.250	1.00	91.75	N
ATOM	282	CA	VAL	A	38	24.812	-23.500	-23.188	1.00	91.75	C
ATOM	283	C	VAL	A	38	23.984	-24.141	-22.078	1.00	91.75	C
ATOM	284	CB	VAL	A	38	24.062	-23.609	-24.531	1.00	91.75	C
ATOM	285	O	VAL	A	38	23.000	-23.562	-21.609	1.00	91.75	O
ATOM	286	CG1	VAL	A	38	24.734	-22.750	-25.594	1.00	91.75	C
ATOM	287	CG2	VAL	A	38	23.969	-25.062	-24.984	1.00	91.75	C
ATOM	288	N	SER	A	39	24.391	-25.297	-21.562	1.00	91.69	N
ATOM	289	CA	SER	A	39	23.625	-25.938	-20.516	1.00	91.69	C
ATOM	290	C	SER	A	39	24.453	-26.969	-19.766	1.00	91.69	C
ATOM	291	CB	SER	A	39	22.375	-26.609	-21.094	1.00	91.69	C
ATOM	292	O	SER	A	39	25.422	-27.516	-20.312	1.00	91.69	O
ATOM	293	OG	SER	A	39	21.641	-27.266	-20.078	1.00	91.69	O
ATOM	294	N	ASP	A	40	24.141	-27.172	-18.500	1.00	90.19	N
ATOM	295	CA	ASP	A	40	24.609	-28.297	-17.688	1.00	90.19	C
ATOM	296	C	ASP	A	40	23.594	-29.422	-17.656	1.00	90.19	C
ATOM	297	CB	ASP	A	40	24.922	-27.828	-16.266	1.00	90.19	C
ATOM	298	O	ASP	A	40	22.469	-29.250	-17.188	1.00	90.19	O
ATOM	299	CG	ASP	A	40	25.547	-28.922	-15.414	1.00	90.19	C
ATOM	300	OD1	ASP	A	40	26.781	-29.125	-15.484	1.00	90.19	O
ATOM	301	OD2	ASP	A	40	24.797	-29.578	-14.664	1.00	90.19	O
ATOM	302	N	GLY	A	41	23.844	-30.547	-18.328	1.00	89.06	N
ATOM	303	CA	GLY	A	41	22.922	-31.672	-18.422	1.00	89.06	C
ATOM	304	C	GLY	A	41	22.328	-31.828	-19.812	1.00	89.06	C
ATOM	305	O	GLY	A	41	22.531	-30.984	-20.688	1.00	89.06	O
ATOM	306	N	GLU	A	42	21.672	-33.000	-20.000	1.00	92.75	N
ATOM	307	CA	GLU	A	42	21.094	-33.312	-21.297	1.00	92.75	C
ATOM	308	C	GLU	A	42	19.922	-32.406	-21.625	1.00	92.75	C
ATOM	309	CB	GLU	A	42	20.641	-34.781	-21.328	1.00	92.75	C
ATOM	310	O	GLU	A	42	19.047	-32.156	-20.781	1.00	92.75	O
ATOM	311	CG	GLU	A	42	20.141	-35.250	-22.688	1.00	92.75	C
ATOM	312	CD	GLU	A	42	19.828	-36.750	-22.734	1.00	92.75	C
ATOM	313	OE1	GLU	A	42	19.203	-37.188	-23.719	1.00	92.75	O
ATOM	314	OE2	GLU	A	42	20.188	-37.438	-21.766	1.00	92.75	O
ATOM	315	N	ILE	A	43	19.891	-31.797	-22.812	1.00	93.62	N
ATOM	316	CA	ILE	A	43	18.812	-30.906	-23.266	1.00	93.62	C

```
ATOM    317  C   ILE A  43      18.188 -31.469 -24.531  1.00 93.62           C
ATOM    318  CB  ILE A  43      19.344 -29.484 -23.500  1.00 93.62           C
ATOM    319  O   ILE A  43      18.703 -32.406 -25.141  1.00 93.62           O
ATOM    320  CG1 ILE A  43      20.453 -29.484 -24.562  1.00 93.62           C
ATOM    321  CG2 ILE A  43      19.844 -28.859 -22.203  1.00 93.62           C
ATOM    322  CD1 ILE A  43      20.953 -28.109 -24.953  1.00 93.62           C
ATOM    323  N   ASP A  44      17.078 -30.922 -24.969  1.00 93.81           N
ATOM    324  CA  ASP A  44      16.375 -31.312 -26.188  1.00 93.81           C
ATOM    325  C   ASP A  44      17.297 -31.281 -27.391  1.00 93.81           C
ATOM    326  CB  ASP A  44      15.164 -30.391 -26.422  1.00 93.81           C
ATOM    327  O   ASP A  44      18.031 -30.312 -27.594  1.00 93.81           O
ATOM    328  CG  ASP A  44      14.359 -30.781 -27.641  1.00 93.81           C
ATOM    329  OD1 ASP A  44      13.680 -31.828 -27.625  1.00 93.81           O
ATOM    330  OD2 ASP A  44      14.398 -30.031 -28.641  1.00 93.81           O
ATOM    331  N   GLU A  45      17.312 -32.375 -28.250  1.00 91.25           N
ATOM    332  CA  GLU A  45      18.281 -32.531 -29.312  1.00 91.25           C
ATOM    333  C   GLU A  45      17.906 -31.734 -30.547  1.00 91.25           C
ATOM    334  CB  GLU A  45      18.422 -34.031 -29.672  1.00 91.25           C
ATOM    335  O   GLU A  45      18.734 -31.516 -31.438  1.00 91.25           O
ATOM    336  CG  GLU A  45      17.141 -34.656 -30.172  1.00 91.25           C
ATOM    337  CD  GLU A  45      17.281 -36.125 -30.484  1.00 91.25           C
ATOM    338  OE1 GLU A  45      16.297 -36.750 -30.969  1.00 91.25           O
ATOM    339  OE2 GLU A  45      18.375 -36.688 -30.266  1.00 91.25           O
ATOM    340  N   THR A  46      16.656 -31.219 -30.641  1.00 91.62           N
ATOM    341  CA  THR A  46      16.188 -30.484 -31.812  1.00 91.62           C
ATOM    342  C   THR A  46      16.906 -29.156 -31.953  1.00 91.62           C
ATOM    343  CB  THR A  46      14.664 -30.250 -31.734  1.00 91.62           C
ATOM    344  O   THR A  46      16.953 -28.359 -31.000  1.00 91.62           O
ATOM    345  CG2 THR A  46      14.133 -29.688 -33.062  1.00 91.62           C
ATOM    346  OG1 THR A  46      14.000 -31.484 -31.453  1.00 91.62           O
ATOM    347  N   PHE A  47      17.594 -28.922 -33.125  1.00 92.25           N
ATOM    348  CA  PHE A  47      18.328 -27.688 -33.406  1.00 92.25           C
ATOM    349  C   PHE A  47      18.328 -27.406 -34.906  1.00 92.25           C
ATOM    350  CB  PHE A  47      19.766 -27.781 -32.875  1.00 92.25           C
ATOM    351  O   PHE A  47      18.531 -28.328 -35.719  1.00 92.25           O
ATOM    352  CG  PHE A  47      20.609 -26.594 -33.250  1.00 92.25           C
ATOM    353  CD1 PHE A  47      21.547 -26.703 -34.281  1.00 92.25           C
ATOM    354  CD2 PHE A  47      20.484 -25.391 -32.594  1.00 92.25           C
ATOM    355  CE1 PHE A  47      22.344 -25.609 -34.656  1.00 92.25           C
ATOM    356  CE2 PHE A  47      21.281 -24.297 -32.938  1.00 92.25           C
ATOM    357  CZ  PHE A  47      22.203 -24.406 -33.969  1.00 92.25           C
ATOM    358  N   SER A  48      18.031 -26.188 -35.250  1.00 93.06           N
ATOM    359  CA  SER A  48      18.172 -25.719 -36.594  1.00 93.06           C
ATOM    360  C   SER A  48      18.672 -24.281 -36.656  1.00 93.06           C
ATOM    361  CB  SER A  48      16.828 -25.812 -37.344  1.00 93.06           C
ATOM    362  O   SER A  48      18.703 -23.594 -35.625  1.00 93.06           O
ATOM    363  OG  SER A  48      15.992 -24.719 -37.000  1.00 93.06           O
ATOM    364  N   LEU A  49      19.094 -23.828 -37.812  1.00 92.81           N
ATOM    365  CA  LEU A  49      19.609 -22.484 -37.969  1.00 92.81           C
ATOM    366  C   LEU A  49      18.500 -21.453 -37.781  1.00 92.81           C
ATOM    367  CB  LEU A  49      20.266 -22.297 -39.344  1.00 92.81           C
ATOM    368  O   LEU A  49      18.766 -20.266 -37.562  1.00 92.81           O
ATOM    369  CG  LEU A  49      21.609 -23.000 -39.531  1.00 92.81           C
ATOM    370  CD1 LEU A  49      22.109 -22.781 -40.969  1.00 92.81           C
ATOM    371  CD2 LEU A  49      22.625 -22.500 -38.531  1.00 92.81           C
ATOM    372  N   LYS A  50      17.234 -21.859 -37.781  1.00 92.81           N
ATOM    373  CA  LYS A  50      16.094 -20.984 -37.562  1.00 92.81           C
ATOM    374  C   LYS A  50      16.031 -20.531 -36.094  1.00 92.81           C
ATOM    375  CB  LYS A  50      14.797 -21.688 -37.938  1.00 92.81           C
ATOM    376  O   LYS A  50      15.422 -19.516 -35.781  1.00 92.81           O
ATOM    377  CG  LYS A  50      14.688 -22.016 -39.438  1.00 92.81           C
ATOM    378  CD  LYS A  50      13.414 -22.797 -39.750  1.00 92.81           C
ATOM    379  CE  LYS A  50      13.336 -23.188 -41.219  1.00 92.81           C
ATOM    380  NZ  LYS A  50      12.133 -24.031 -41.500  1.00 92.81           N
```

```
ATOM    381  N   GLN A  51      16.672 -21.266 -35.250  1.00 93.12           N
ATOM    382  CA  GLN A  51      16.672 -21.016 -33.812  1.00 93.12           C
ATOM    383  C   GLN A  51      17.859 -20.156 -33.406  1.00 93.12           C
ATOM    384  CB  GLN A  51      16.688 -22.344 -33.062  1.00 93.12           C
ATOM    385  O   GLN A  51      17.906 -19.656 -32.281  1.00 93.12           O
ATOM    386  CG  GLN A  51      15.461 -23.219 -33.281  1.00 93.12           C
ATOM    387  CD  GLN A  51      15.617 -24.609 -32.688  1.00 93.12           C
ATOM    388  NE2 GLN A  51      14.664 -25.000 -31.859  1.00 93.12           N
ATOM    389  OE1 GLN A  51      16.578 -25.312 -33.000  1.00 93.12           O
ATOM    390  N   LEU A  52      18.875 -19.953 -34.312  1.00 94.88           N
ATOM    391  CA  LEU A  52      20.109 -19.203 -34.031  1.00 94.88           C
ATOM    392  C   LEU A  52      19.984 -17.766 -34.500  1.00 94.88           C
ATOM    393  CB  LEU A  52      21.297 -19.875 -34.719  1.00 94.88           C
ATOM    394  O   LEU A  52      19.641 -17.516 -35.688  1.00 94.88           O
ATOM    395  CG  LEU A  52      22.656 -19.203 -34.531  1.00 94.88           C
ATOM    396  CD1 LEU A  52      23.047 -19.172 -33.062  1.00 94.88           C
ATOM    397  CD2 LEU A  52      23.734 -19.906 -35.375  1.00 94.88           C
ATOM    398  N   MET A  53      20.281 -16.812 -33.625  1.00 93.62           N
ATOM    399  CA  MET A  53      20.172 -15.398 -33.938  1.00 93.62           C
ATOM    400  C   MET A  53      21.484 -14.672 -33.719  1.00 93.62           C
ATOM    401  CB  MET A  53      19.047 -14.742 -33.125  1.00 93.62           C
ATOM    402  O   MET A  53      22.188 -14.953 -32.719  1.00 93.62           O
ATOM    403  CG  MET A  53      17.672 -15.320 -33.406  1.00 93.62           C
ATOM    404  SD  MET A  53      16.391 -14.664 -32.281  1.00 93.62           S
ATOM    405  CE  MET A  53      16.812 -15.570 -30.750  1.00 93.62           C
ATOM    406  N   PHE A  54      21.812 -13.742 -34.688  1.00 93.25           N
ATOM    407  CA  PHE A  54      22.891 -12.766 -34.531  1.00 93.25           C
ATOM    408  C   PHE A  54      22.312 -11.359 -34.344  1.00 93.25           C
ATOM    409  CB  PHE A  54      23.828 -12.797 -35.719  1.00 93.25           C
ATOM    410  O   PHE A  54      21.609 -10.852 -35.219  1.00 93.25           O
ATOM    411  CG  PHE A  54      24.594 -14.086 -35.875  1.00 93.25           C
ATOM    412  CD1 PHE A  54      24.734 -14.945 -34.781  1.00 93.25           C
ATOM    413  CD2 PHE A  54      25.156 -14.438 -37.094  1.00 93.25           C
ATOM    414  CE1 PHE A  54      25.438 -16.141 -34.906  1.00 93.25           C
ATOM    415  CE2 PHE A  54      25.875 -15.625 -37.219  1.00 93.25           C
ATOM    416  CZ  PHE A  54      26.000 -16.484 -36.125  1.00 93.25           C
ATOM    417  N   ASN A  55      22.594 -10.719 -33.125  1.00 90.56           N
ATOM    418  CA  ASN A  55      22.062  -9.391 -32.844  1.00 90.56           C
ATOM    419  C   ASN A  55      20.547  -9.328 -33.031  1.00 90.56           C
ATOM    420  CB  ASN A  55      22.750  -8.336 -33.719  1.00 90.56           C
ATOM    421  O   ASN A  55      20.031  -8.398 -33.625  1.00 90.56           O
ATOM    422  CG  ASN A  55      24.203  -8.109 -33.344  1.00 90.56           C
ATOM    423  ND2 ASN A  55      25.000  -7.652 -34.281  1.00 90.56           N
ATOM    424  OD1 ASN A  55      24.594  -8.352 -32.188  1.00 90.56           O
ATOM    425  N   SER A  56      19.891 -10.555 -32.656  1.00 90.06           N
ATOM    426  CA  SER A  56      18.438 -10.633 -32.562  1.00 90.06           C
ATOM    427  C   SER A  56      17.844 -10.844 -33.969  1.00 90.06           C
ATOM    428  CB  SER A  56      17.859  -9.367 -31.953  1.00 90.06           C
ATOM    429  O   SER A  56      16.656 -10.570 -34.188  1.00 90.06           O
ATOM    430  OG  SER A  56      18.344  -9.195 -30.625  1.00 90.06           O
ATOM    431  N   VAL A  57      18.656 -11.188 -35.000  1.00 91.88           N
ATOM    432  CA  VAL A  57      18.188 -11.547 -36.344  1.00 91.88           C
ATOM    433  C   VAL A  57      18.453 -13.023 -36.594  1.00 91.88           C
ATOM    434  CB  VAL A  57      18.844 -10.680 -37.438  1.00 91.88           C
ATOM    435  O   VAL A  57      19.594 -13.492 -36.500  1.00 91.88           O
ATOM    436  CG1 VAL A  57      18.344 -11.070 -38.812  1.00 91.88           C
ATOM    437  CG2 VAL A  57      18.594  -9.195 -37.156  1.00 91.88           C
ATOM    438  N   PRO A  58      17.312 -13.789 -36.812  1.00 93.38           N
ATOM    439  CA  PRO A  58      17.531 -15.211 -37.094  1.00 93.38           C
ATOM    440  C   PRO A  58      18.469 -15.438 -38.281  1.00 93.38           C
ATOM    441  CB  PRO A  58      16.125 -15.727 -37.406  1.00 93.38           C
ATOM    442  O   PRO A  58      18.406 -14.703 -39.281  1.00 93.38           O
ATOM    443  CG  PRO A  58      15.203 -14.688 -36.844  1.00 93.38           C
ATOM    444  CD  PRO A  58      15.945 -13.383 -36.781  1.00 93.38           C
```

```
ATOM    445  N   VAL A  59      19.391 -16.406 -38.156  1.00 94.12           N
ATOM    446  CA  VAL A  59      20.297 -16.766 -39.250  1.00 94.12           C
ATOM    447  C   VAL A  59      19.500 -17.250 -40.438  1.00 94.12           C
ATOM    448  CB  VAL A  59      21.312 -17.844 -38.781  1.00 94.12           C
ATOM    449  O   VAL A  59      19.781 -16.891 -41.594  1.00 94.12           O
ATOM    450  CG1 VAL A  59      22.109 -18.359 -40.000  1.00 94.12           C
ATOM    451  CG2 VAL A  59      22.250 -17.297 -37.719  1.00 94.12           C
ATOM    452  N   GLN A  60      18.500 -18.047 -40.188  1.00 94.75           N
ATOM    453  CA  GLN A  60      17.578 -18.547 -41.188  1.00 94.75           C
ATOM    454  C   GLN A  60      16.125 -18.234 -40.844  1.00 94.75           C
ATOM    455  CB  GLN A  60      17.750 -20.062 -41.375  1.00 94.75           C
ATOM    456  O   GLN A  60      15.688 -18.516 -39.719  1.00 94.75           O
ATOM    457  CG  GLN A  60      17.000 -20.641 -42.562  1.00 94.75           C
ATOM    458  CD  GLN A  60      17.172 -22.141 -42.719  1.00 94.75           C
ATOM    459  NE2 GLN A  60      16.844 -22.672 -43.875  1.00 94.75           N
ATOM    460  OE1 GLN A  60      17.625 -22.812 -41.781  1.00 94.75           O
ATOM    461  N   ASN A  61      15.383 -17.547 -41.719  1.00 92.19           N
ATOM    462  CA  ASN A  61      13.992 -17.188 -41.500  1.00 92.19           C
ATOM    463  C   ASN A  61      13.086 -18.422 -41.500  1.00 92.19           C
ATOM    464  CB  ASN A  61      13.508 -16.172 -42.531  1.00 92.19           C
ATOM    465  O   ASN A  61      13.500 -19.500 -41.938  1.00 92.19           O
ATOM    466  CG  ASN A  61      14.234 -14.852 -42.406  1.00 92.19           C
ATOM    467  ND2 ASN A  61      14.656 -14.312 -43.562  1.00 92.19           N
ATOM    468  OD1 ASN A  61      14.414 -14.312 -41.312  1.00 92.19           O
ATOM    469  N   GLU A  62      11.797 -18.250 -41.031  1.00 90.06           N
ATOM    470  CA  GLU A  62      10.820 -19.344 -41.000  1.00 90.06           C
ATOM    471  C   GLU A  62      10.500 -19.828 -42.406  1.00 90.06           C
ATOM    472  CB  GLU A  62       9.539 -18.891 -40.281  1.00 90.06           C
ATOM    473  O   GLU A  62      10.133 -21.000 -42.594  1.00 90.06           O
ATOM    474  CG  GLU A  62       9.688 -18.781 -38.781  1.00 90.06           C
ATOM    475  CD  GLU A  62       9.914 -20.109 -38.094  1.00 90.06           C
ATOM    476  OE1 GLU A  62      10.336 -20.125 -36.906  1.00 90.06           O
ATOM    477  OE2 GLU A  62       9.672 -21.156 -38.750  1.00 90.06           O
ATOM    478  N   ASP A  63      10.641 -18.906 -43.375  1.00 91.94           N
ATOM    479  CA  ASP A  63      10.336 -19.234 -44.781  1.00 91.94           C
ATOM    480  C   ASP A  63      11.516 -19.938 -45.438  1.00 91.94           C
ATOM    481  CB  ASP A  63       9.953 -17.984 -45.562  1.00 91.94           C
ATOM    482  O   ASP A  63      11.445 -20.297 -46.625  1.00 91.94           O
ATOM    483  CG  ASP A  63      11.094 -16.969 -45.656  1.00 91.94           C
ATOM    484  OD1 ASP A  63      12.172 -17.219 -45.062  1.00 91.94           O
ATOM    485  OD2 ASP A  63      10.906 -15.930 -46.312  1.00 91.94           O
ATOM    486  N   GLY A  64      12.719 -20.094 -44.719  1.00 89.88           N
ATOM    487  CA  GLY A  64      13.875 -20.812 -45.188  1.00 89.88           C
ATOM    488  C   GLY A  64      14.961 -19.906 -45.750  1.00 89.88           C
ATOM    489  O   GLY A  64      16.078 -20.344 -46.000  1.00 89.88           O
ATOM    490  N   SER A  65      14.758 -18.609 -46.000  1.00 93.12           N
ATOM    491  CA  SER A  65      15.734 -17.641 -46.500  1.00 93.12           C
ATOM    492  C   SER A  65      16.766 -17.297 -45.406  1.00 93.12           C
ATOM    493  CB  SER A  65      15.031 -16.359 -46.969  1.00 93.12           C
ATOM    494  O   SER A  65      16.453 -17.359 -44.219  1.00 93.12           O
ATOM    495  OG  SER A  65      14.375 -15.727 -45.875  1.00 93.12           O
ATOM    496  N   PHE A  66      18.078 -16.984 -45.906  1.00 93.38           N
ATOM    497  CA  PHE A  66      19.172 -16.719 -44.969  1.00 93.38           C
ATOM    498  C   PHE A  66      19.422 -15.227 -44.844  1.00 93.38           C
ATOM    499  CB  PHE A  66      20.453 -17.438 -45.438  1.00 93.38           C
ATOM    500  O   PHE A  66      19.516 -14.508 -45.844  1.00 93.38           O
ATOM    501  CG  PHE A  66      20.438 -18.922 -45.219  1.00 93.38           C
ATOM    502  CD1 PHE A  66      20.906 -19.453 -44.000  1.00 93.38           C
ATOM    503  CD2 PHE A  66      19.953 -19.781 -46.188  1.00 93.38           C
ATOM    504  CE1 PHE A  66      20.891 -20.828 -43.781  1.00 93.38           C
ATOM    505  CE2 PHE A  66      19.938 -21.156 -45.969  1.00 93.38           C
ATOM    506  CZ  PHE A  66      20.422 -21.672 -44.781  1.00 93.38           C
ATOM    507  N   ASN A  67      19.375 -14.766 -43.562  1.00 92.00           N
ATOM    508  CA  ASN A  67      19.797 -13.398 -43.281  1.00 92.00           C
```

ATOM	509	C	ASN	A	67	21.312	-13.266	-43.312	1.00	92.00	C
ATOM	510	CB	ASN	A	67	19.234	-12.930	-41.938	1.00	92.00	C
ATOM	511	O	ASN	A	67	21.844	-12.203	-43.625	1.00	92.00	O
ATOM	512	CG	ASN	A	67	17.734	-12.688	-41.969	1.00	92.00	C
ATOM	513	ND2	ASN	A	67	17.062	-13.055	-40.906	1.00	92.00	N
ATOM	514	OD1	ASN	A	67	17.203	-12.180	-42.969	1.00	92.00	O
ATOM	515	N	PHE	A	68	22.000	-14.383	-42.906	1.00	90.88	N
ATOM	516	CA	PHE	A	68	23.453	-14.477	-42.906	1.00	90.88	C
ATOM	517	C	PHE	A	68	23.922	-15.711	-43.688	1.00	90.88	C
ATOM	518	CB	PHE	A	68	24.000	-14.531	-41.469	1.00	90.88	C
ATOM	519	O	PHE	A	68	23.688	-16.844	-43.250	1.00	90.88	O
ATOM	520	CG	PHE	A	68	23.531	-13.391	-40.625	1.00	90.88	C
ATOM	521	CD1	PHE	A	68	24.172	-12.156	-40.656	1.00	90.88	C
ATOM	522	CD2	PHE	A	68	22.453	-13.547	-39.750	1.00	90.88	C
ATOM	523	CE1	PHE	A	68	23.750	-11.102	-39.875	1.00	90.88	C
ATOM	524	CE2	PHE	A	68	22.016	-12.492	-38.969	1.00	90.88	C
ATOM	525	CZ	PHE	A	68	22.672	-11.273	-39.000	1.00	90.88	C
ATOM	526	N	GLU	A	69	24.625	-15.406	-44.812	1.00	88.19	N
ATOM	527	CA	GLU	A	69	25.125	-16.516	-45.656	1.00	88.19	C
ATOM	528	C	GLU	A	69	26.484	-17.000	-45.156	1.00	88.19	C
ATOM	529	CB	GLU	A	69	25.219	-16.094	-47.094	1.00	88.19	C
ATOM	530	O	GLU	A	69	27.250	-16.219	-44.562	1.00	88.19	O
ATOM	531	CG	GLU	A	69	23.875	-15.805	-47.750	1.00	88.19	C
ATOM	532	CD	GLU	A	69	23.953	-15.688	-49.281	1.00	88.19	C
ATOM	533	OE1	GLU	A	69	22.906	-15.562	-49.938	1.00	88.19	O
ATOM	534	OE2	GLU	A	69	25.094	-15.719	-49.812	1.00	88.19	O
ATOM	535	N	GLY	A	70	26.781	-18.375	-45.156	1.00	86.88	N
ATOM	536	CA	GLY	A	70	28.078	-18.969	-44.844	1.00	86.88	C
ATOM	537	C	GLY	A	70	28.219	-19.297	-43.344	1.00	86.88	C
ATOM	538	O	GLY	A	70	29.344	-19.438	-42.844	1.00	86.88	O
ATOM	539	N	VAL	A	71	27.078	-19.266	-42.594	1.00	91.44	N
ATOM	540	CA	VAL	A	71	27.109	-19.609	-41.188	1.00	91.44	C
ATOM	541	C	VAL	A	71	27.016	-21.125	-41.031	1.00	91.44	C
ATOM	542	CB	VAL	A	71	25.953	-18.922	-40.438	1.00	91.44	C
ATOM	543	O	VAL	A	71	26.172	-21.766	-41.625	1.00	91.44	O
ATOM	544	CG1	VAL	A	71	25.922	-19.375	-38.969	1.00	91.44	C
ATOM	545	CG2	VAL	A	71	26.078	-17.391	-40.500	1.00	91.44	C
ATOM	546	N	LYS	A	72	28.031	-21.688	-40.344	1.00	88.56	N
ATOM	547	CA	LYS	A	72	28.000	-23.094	-39.906	1.00	88.56	C
ATOM	548	C	LYS	A	72	27.797	-23.219	-38.406	1.00	88.56	C
ATOM	549	CB	LYS	A	72	29.297	-23.797	-40.344	1.00	88.56	C
ATOM	550	O	LYS	A	72	28.469	-22.547	-37.625	1.00	88.56	O
ATOM	551	CG	LYS	A	72	29.281	-25.297	-40.125	1.00	88.56	C
ATOM	552	CD	LYS	A	72	30.594	-25.953	-40.562	1.00	88.56	C
ATOM	553	CE	LYS	A	72	30.625	-27.438	-40.219	1.00	88.56	C
ATOM	554	NZ	LYS	A	72	31.906	-28.062	-40.656	1.00	88.56	N
ATOM	555	N	ALA	A	73	26.797	-24.047	-37.938	1.00	92.12	N
ATOM	556	CA	ALA	A	73	26.516	-24.266	-36.531	1.00	92.12	C
ATOM	557	C	ALA	A	73	26.266	-25.750	-36.250	1.00	92.12	C
ATOM	558	CB	ALA	A	73	25.312	-23.438	-36.094	1.00	92.12	C
ATOM	559	O	ALA	A	73	25.609	-26.438	-37.031	1.00	92.12	O
ATOM	560	N	GLU	A	74	26.938	-26.297	-35.219	1.00	88.69	N
ATOM	561	CA	GLU	A	74	26.734	-27.656	-34.750	1.00	88.69	C
ATOM	562	C	GLU	A	74	26.297	-27.656	-33.281	1.00	88.69	C
ATOM	563	CB	GLU	A	74	28.016	-28.484	-34.906	1.00	88.69	C
ATOM	564	O	GLU	A	74	26.797	-26.875	-32.469	1.00	88.69	O
ATOM	565	CG	GLU	A	74	28.422	-28.703	-36.344	1.00	88.69	C
ATOM	566	CD	GLU	A	74	29.641	-29.609	-36.500	1.00	88.69	C
ATOM	567	OE1	GLU	A	74	30.375	-29.484	-37.500	1.00	88.69	O
ATOM	568	OE2	GLU	A	74	29.844	-30.469	-35.594	1.00	88.69	O
ATOM	569	N	PHE	A	75	25.375	-28.500	-32.938	1.00	92.19	N
ATOM	570	CA	PHE	A	75	24.797	-28.547	-31.594	1.00	92.19	C
ATOM	571	C	PHE	A	75	24.984	-29.922	-30.969	1.00	92.19	C
ATOM	572	CB	PHE	A	75	23.297	-28.203	-31.641	1.00	92.19	C

ATOM	573	O	PHE	A	75	24.781	-30.938	-31.609	1.00	92.19	O
ATOM	574	CG	PHE	A	75	22.656	-28.141	-30.281	1.00	92.19	C
ATOM	575	CD1	PHE	A	75	21.828	-29.156	-29.844	1.00	92.19	C
ATOM	576	CD2	PHE	A	75	22.859	-27.047	-29.453	1.00	92.19	C
ATOM	577	CE1	PHE	A	75	21.219	-29.109	-28.594	1.00	92.19	C
ATOM	578	CE2	PHE	A	75	22.266	-26.984	-28.203	1.00	92.19	C
ATOM	579	CZ	PHE	A	75	21.438	-28.016	-27.766	1.00	92.19	C
ATOM	580	N	ARG	A	76	25.547	-29.953	-29.750	1.00	92.56	N
ATOM	581	CA	ARG	A	76	25.594	-31.156	-28.922	1.00	92.56	C
ATOM	582	C	ARG	A	76	24.656	-31.047	-27.719	1.00	92.56	C
ATOM	583	CB	ARG	A	76	27.031	-31.422	-28.438	1.00	92.56	C
ATOM	584	O	ARG	A	76	24.750	-30.094	-26.953	1.00	92.56	O
ATOM	585	CG	ARG	A	76	28.016	-31.672	-29.562	1.00	92.56	C
ATOM	586	CD	ARG	A	76	27.734	-32.969	-30.297	1.00	92.56	C
ATOM	587	NE	ARG	A	76	28.719	-33.250	-31.344	1.00	92.56	N
ATOM	588	NH1	ARG	A	76	27.578	-32.125	-33.000	1.00	92.56	N
ATOM	589	NH2	ARG	A	76	29.578	-33.125	-33.469	1.00	92.56	N
ATOM	590	CZ	ARG	A	76	28.625	-32.844	-32.594	1.00	92.56	C
ATOM	591	N	PRO	A	77	23.750	-31.969	-27.562	1.00	93.31	N
ATOM	592	CA	PRO	A	77	22.688	-31.859	-26.562	1.00	93.31	C
ATOM	593	C	PRO	A	77	23.188	-32.156	-25.141	1.00	93.31	C
ATOM	594	CB	PRO	A	77	21.672	-32.906	-27.000	1.00	93.31	C
ATOM	595	O	PRO	A	77	22.453	-31.953	-24.172	1.00	93.31	O
ATOM	596	CG	PRO	A	77	22.469	-33.906	-27.766	1.00	93.31	C
ATOM	597	CD	PRO	A	77	23.609	-33.219	-28.438	1.00	93.31	C
ATOM	598	N	GLY	A	78	24.453	-32.562	-24.953	1.00	92.50	N
ATOM	599	CA	GLY	A	78	25.000	-32.781	-23.625	1.00	92.50	C
ATOM	600	C	GLY	A	78	24.750	-34.188	-23.094	1.00	92.50	C
ATOM	601	O	GLY	A	78	24.594	-34.375	-21.891	1.00	92.50	O
ATOM	602	N	THR	A	79	24.609	-35.250	-23.906	1.00	92.12	N
ATOM	603	CA	THR	A	79	24.516	-36.625	-23.484	1.00	92.12	C
ATOM	604	C	THR	A	79	25.844	-37.125	-22.938	1.00	92.12	C
ATOM	605	CB	THR	A	79	24.047	-37.531	-24.641	1.00	92.12	C
ATOM	606	O	THR	A	79	26.891	-36.531	-23.219	1.00	92.12	O
ATOM	607	CG2	THR	A	79	22.750	-37.031	-25.250	1.00	92.12	C
ATOM	608	OG1	THR	A	79	25.062	-37.562	-25.641	1.00	92.12	O
ATOM	609	N	GLN	A	80	25.703	-38.156	-22.094	1.00	89.81	N
ATOM	610	CA	GLN	A	80	26.906	-38.750	-21.531	1.00	89.81	C
ATOM	611	C	GLN	A	80	27.859	-39.219	-22.609	1.00	89.81	C
ATOM	612	CB	GLN	A	80	26.547	-39.875	-20.578	1.00	89.81	C
ATOM	613	O	GLN	A	80	29.078	-39.094	-22.500	1.00	89.81	O
ATOM	614	CG	GLN	A	80	27.750	-40.469	-19.844	1.00	89.81	C
ATOM	615	CD	GLN	A	80	28.328	-39.531	-18.812	1.00	89.81	C
ATOM	616	NE2	GLN	A	80	29.547	-39.781	-18.375	1.00	89.81	N
ATOM	617	OE1	GLN	A	80	27.672	-38.562	-18.391	1.00	89.81	O
ATOM	618	N	THR	A	81	27.312	-39.688	-23.750	1.00	88.81	N
ATOM	619	CA	THR	A	81	28.109	-40.312	-24.797	1.00	88.81	C
ATOM	620	C	THR	A	81	28.125	-39.469	-26.062	1.00	88.81	C
ATOM	621	CB	THR	A	81	27.578	-41.719	-25.125	1.00	88.81	C
ATOM	622	O	THR	A	81	28.297	-40.000	-27.172	1.00	88.81	O
ATOM	623	CG2	THR	A	81	27.734	-42.656	-23.938	1.00	88.81	C
ATOM	624	OG1	THR	A	81	26.188	-41.656	-25.469	1.00	88.81	O
ATOM	625	N	GLN	A	82	27.953	-38.062	-25.984	1.00	89.38	N
ATOM	626	CA	GLN	A	82	27.891	-37.219	-27.172	1.00	89.38	C
ATOM	627	C	GLN	A	82	29.266	-37.125	-27.844	1.00	89.38	C
ATOM	628	CB	GLN	A	82	27.391	-35.812	-26.812	1.00	89.38	C
ATOM	629	O	GLN	A	82	30.297	-37.250	-27.172	1.00	89.38	O
ATOM	630	CG	GLN	A	82	28.297	-35.062	-25.844	1.00	89.38	C
ATOM	631	CD	GLN	A	82	27.688	-33.750	-25.375	1.00	89.38	C
ATOM	632	NE2	GLN	A	82	28.188	-33.250	-24.250	1.00	89.38	N
ATOM	633	OE1	GLN	A	82	26.781	-33.219	-26.016	1.00	89.38	O
ATOM	634	N	GLU	A	83	29.312	-37.031	-29.234	1.00	81.94	N
ATOM	635	CA	GLU	A	83	30.547	-37.000	-30.016	1.00	81.94	C
ATOM	636	C	GLU	A	83	31.219	-35.625	-29.922	1.00	81.94	C

ATOM	637	CB	GLU	A	83	30.281	-37.344	-31.469	1.00	81.94	C
ATOM	638	O	GLU	A	83	30.547	-34.625	-29.594	1.00	81.94	O
ATOM	639	CG	GLU	A	83	29.844	-38.781	-31.688	1.00	81.94	C
ATOM	640	CD	GLU	A	83	29.812	-39.188	-33.156	1.00	81.94	C
ATOM	641	OE1	GLU	A	83	29.766	-40.406	-33.438	1.00	81.94	O
ATOM	642	OE2	GLU	A	83	29.828	-38.312	-34.000	1.00	81.94	O
ATOM	643	N	TYR	A	84	32.531	-35.531	-30.078	1.00	81.44	N
ATOM	644	CA	TYR	A	84	33.219	-34.250	-30.109	1.00	81.44	C
ATOM	645	C	TYR	A	84	32.844	-33.469	-31.359	1.00	81.44	C
ATOM	646	CB	TYR	A	84	34.750	-34.438	-30.016	1.00	81.44	C
ATOM	647	O	TYR	A	84	32.375	-34.062	-32.344	1.00	81.44	O
ATOM	648	CG	TYR	A	84	35.375	-34.844	-31.328	1.00	81.44	C
ATOM	649	CD1	TYR	A	84	35.344	-36.156	-31.766	1.00	81.44	C
ATOM	650	CD2	TYR	A	84	36.000	-33.906	-32.125	1.00	81.44	C
ATOM	651	CE1	TYR	A	84	35.938	-36.531	-32.969	1.00	81.44	C
ATOM	652	CE2	TYR	A	84	36.594	-34.250	-33.344	1.00	81.44	C
ATOM	653	OH	TYR	A	84	37.125	-35.938	-34.938	1.00	81.44	O
ATOM	654	CZ	TYR	A	84	36.531	-35.562	-33.750	1.00	81.44	C
ATOM	655	N	ILE	A	85	32.844	-32.156	-31.359	1.00	80.69	N
ATOM	656	CA	ILE	A	85	32.594	-31.297	-32.500	1.00	80.69	C
ATOM	657	C	ILE	A	85	33.781	-31.297	-33.438	1.00	80.69	C
ATOM	658	CB	ILE	A	85	32.250	-29.859	-32.031	1.00	80.69	C
ATOM	659	O	ILE	A	85	34.875	-30.922	-33.062	1.00	80.69	O
ATOM	660	CG1	ILE	A	85	30.953	-29.828	-31.250	1.00	80.69	C
ATOM	661	CG2	ILE	A	85	32.188	-28.906	-33.250	1.00	80.69	C
ATOM	662	CD1	ILE	A	85	30.562	-28.438	-30.750	1.00	80.69	C
ATOM	663	N	LYS	A	86	33.594	-31.797	-34.688	1.00	69.19	N
ATOM	664	CA	LYS	A	86	34.625	-31.891	-35.719	1.00	69.19	C
ATOM	665	C	LYS	A	86	35.031	-30.500	-36.219	1.00	69.19	C
ATOM	666	CB	LYS	A	86	34.094	-32.719	-36.875	1.00	69.19	C
ATOM	667	O	LYS	A	86	34.156	-29.625	-36.344	1.00	69.19	O
ATOM	668	CG	LYS	A	86	33.906	-34.219	-36.562	1.00	69.19	C
ATOM	669	CD	LYS	A	86	33.469	-35.000	-37.781	1.00	69.19	C
ATOM	670	CE	LYS	A	86	33.219	-36.469	-37.406	1.00	69.19	C
ATOM	671	NZ	LYS	A	86	32.750	-37.250	-38.625	1.00	69.19	N
ATOM	672	N	GLY	A	87	36.375	-30.094	-36.188	1.00	63.81	N
ATOM	673	CA	GLY	A	87	36.875	-28.766	-36.562	1.00	63.81	C
ATOM	674	C	GLY	A	87	37.469	-28.000	-35.406	1.00	63.81	C
ATOM	675	O	GLY	A	87	38.094	-26.969	-35.594	1.00	63.81	O
ATOM	676	N	MET	A	88	37.156	-28.453	-34.125	1.00	69.56	N
ATOM	677	CA	MET	A	88	37.781	-27.953	-32.906	1.00	69.56	C
ATOM	678	C	MET	A	88	38.625	-29.031	-32.250	1.00	69.56	C
ATOM	679	CB	MET	A	88	36.688	-27.484	-31.906	1.00	69.56	C
ATOM	680	O	MET	A	88	38.312	-29.531	-31.172	1.00	69.56	O
ATOM	681	CG	MET	A	88	36.062	-26.156	-32.281	1.00	69.56	C
ATOM	682	SD	MET	A	88	35.719	-25.109	-30.812	1.00	69.56	S
ATOM	683	CE	MET	A	88	35.062	-23.625	-31.625	1.00	69.56	C
ATOM	684	N	GLU	A	89	39.531	-29.578	-33.062	1.00	66.62	N
ATOM	685	CA	GLU	A	89	40.312	-30.734	-32.594	1.00	66.62	C
ATOM	686	C	GLU	A	89	41.469	-30.297	-31.719	1.00	66.62	C
ATOM	687	CB	GLU	A	89	40.844	-31.516	-33.812	1.00	66.62	C
ATOM	688	O	GLU	A	89	42.281	-31.125	-31.281	1.00	66.62	O
ATOM	689	CG	GLU	A	89	39.750	-32.062	-34.719	1.00	66.62	C
ATOM	690	CD	GLU	A	89	40.281	-32.750	-35.969	1.00	66.62	C
ATOM	691	OE1	GLU	A	89	39.469	-33.250	-36.781	1.00	66.62	O
ATOM	692	OE2	GLU	A	89	41.531	-32.750	-36.125	1.00	66.62	O
ATOM	693	N	ASP	A	90	41.438	-28.891	-31.422	1.00	75.50	N
ATOM	694	CA	ASP	A	90	42.562	-28.484	-30.594	1.00	75.50	C
ATOM	695	C	ASP	A	90	42.156	-27.406	-29.594	1.00	75.50	C
ATOM	696	CB	ASP	A	90	43.719	-27.969	-31.469	1.00	75.50	C
ATOM	697	O	ASP	A	90	41.062	-26.859	-29.688	1.00	75.50	O
ATOM	698	CG	ASP	A	90	43.344	-26.750	-32.281	1.00	75.50	C
ATOM	699	OD1	ASP	A	90	42.500	-25.953	-31.828	1.00	75.50	O
ATOM	700	OD2	ASP	A	90	43.906	-26.594	-33.406	1.00	75.50	O

ATOM	701	N	SER	A	91	42.594	-27.359	-28.516	1.00	78.75	N
ATOM	702	CA	SER	A	91	42.562	-26.266	-27.562	1.00	78.75	C
ATOM	703	C	SER	A	91	43.781	-25.359	-27.734	1.00	78.75	C
ATOM	704	CB	SER	A	91	42.531	-26.812	-26.125	1.00	78.75	C
ATOM	705	O	SER	A	91	44.906	-25.844	-27.781	1.00	78.75	O
ATOM	706	OG	SER	A	91	42.562	-25.750	-25.188	1.00	78.75	O
ATOM	707	N	SER	A	92	43.594	-24.031	-28.125	1.00	82.69	N
ATOM	708	CA	SER	A	92	44.719	-23.156	-28.375	1.00	82.69	C
ATOM	709	C	SER	A	92	44.625	-21.875	-27.562	1.00	82.69	C
ATOM	710	CB	SER	A	92	44.812	-22.812	-29.875	1.00	82.69	C
ATOM	711	O	SER	A	92	43.531	-21.469	-27.156	1.00	82.69	O
ATOM	712	OG	SER	A	92	43.719	-22.031	-30.266	1.00	82.69	O
ATOM	713	N	SER	A	93	45.625	-21.375	-27.078	1.00	84.62	N
ATOM	714	CA	SER	A	93	45.844	-20.094	-26.422	1.00	84.62	C
ATOM	715	C	SER	A	93	46.750	-19.188	-27.234	1.00	84.62	C
ATOM	716	CB	SER	A	93	46.438	-20.297	-25.031	1.00	84.62	C
ATOM	717	O	SER	A	93	47.906	-19.562	-27.531	1.00	84.62	O
ATOM	718	OG	SER	A	93	46.594	-19.062	-24.359	1.00	84.62	O
ATOM	719	N	GLU	A	94	46.250	-18.047	-27.703	1.00	89.00	N
ATOM	720	CA	GLU	A	94	47.000	-17.156	-28.594	1.00	89.00	C
ATOM	721	C	GLU	A	94	47.875	-16.172	-27.812	1.00	89.00	C
ATOM	722	CB	GLU	A	94	46.031	-16.391	-29.500	1.00	89.00	C
ATOM	723	O	GLU	A	94	47.438	-15.625	-26.797	1.00	89.00	O
ATOM	724	CG	GLU	A	94	46.688	-15.703	-30.688	1.00	89.00	C
ATOM	725	CD	GLU	A	94	45.688	-15.234	-31.734	1.00	89.00	C
ATOM	726	OE1	GLU	A	94	46.094	-15.055	-32.906	1.00	89.00	O
ATOM	727	OE2	GLU	A	94	44.500	-15.039	-31.391	1.00	89.00	O
ATOM	728	N	VAL	A	95	49.094	-15.930	-28.156	1.00	88.75	N
ATOM	729	CA	VAL	A	95	50.062	-14.938	-27.672	1.00	88.75	C
ATOM	730	C	VAL	A	95	50.312	-13.891	-28.750	1.00	88.75	C
ATOM	731	CB	VAL	A	95	51.375	-15.594	-27.219	1.00	88.75	C
ATOM	732	O	VAL	A	95	50.781	-14.227	-29.844	1.00	88.75	O
ATOM	733	CG1	VAL	A	95	52.344	-14.547	-26.703	1.00	88.75	C
ATOM	734	CG2	VAL	A	95	51.125	-16.672	-26.172	1.00	88.75	C
ATOM	735	N	THR	A	96	49.875	-12.547	-28.406	1.00	91.31	N
ATOM	736	CA	THR	A	96	50.062	-11.469	-29.359	1.00	91.31	C
ATOM	737	C	THR	A	96	51.531	-11.016	-29.375	1.00	91.31	C
ATOM	738	CB	THR	A	96	49.156	-10.266	-29.062	1.00	91.31	C
ATOM	739	O	THR	A	96	52.125	-10.773	-28.312	1.00	91.31	O
ATOM	740	CG2	THR	A	96	49.281	-9.188	-30.125	1.00	91.31	C
ATOM	741	OG1	THR	A	96	47.812	-10.711	-29.016	1.00	91.31	O
ATOM	742	N	VAL	A	97	52.250	-10.820	-30.516	1.00	90.00	N
ATOM	743	CA	VAL	A	97	53.656	-10.492	-30.641	1.00	90.00	C
ATOM	744	C	VAL	A	97	53.812	-9.148	-31.359	1.00	90.00	C
ATOM	745	CB	VAL	A	97	54.438	-11.594	-31.406	1.00	90.00	C
ATOM	746	O	VAL	A	97	54.188	-8.148	-30.734	1.00	90.00	O
ATOM	747	CG1	VAL	A	97	55.906	-11.258	-31.484	1.00	90.00	C
ATOM	748	CG2	VAL	A	97	54.219	-12.945	-30.719	1.00	90.00	C
ATOM	749	N	ASN	A	98	53.375	-9.023	-32.688	1.00	90.81	N
ATOM	750	CA	ASN	A	98	53.469	-7.867	-33.594	1.00	90.81	C
ATOM	751	C	ASN	A	98	54.938	-7.457	-33.812	1.00	90.81	C
ATOM	752	CB	ASN	A	98	52.656	-6.688	-33.031	1.00	90.81	C
ATOM	753	O	ASN	A	98	55.281	-6.281	-33.688	1.00	90.81	O
ATOM	754	CG	ASN	A	98	51.188	-6.910	-33.094	1.00	90.81	C
ATOM	755	ND2	ASN	A	98	50.438	-6.438	-32.094	1.00	90.81	N
ATOM	756	OD1	ASN	A	98	50.688	-7.504	-34.062	1.00	90.81	O
ATOM	757	N	ARG	A	99	55.875	-8.383	-34.094	1.00	93.69	N
ATOM	758	CA	ARG	A	99	57.281	-8.141	-34.312	1.00	93.69	C
ATOM	759	C	ARG	A	99	57.656	-8.328	-35.781	1.00	93.69	C
ATOM	760	CB	ARG	A	99	58.125	-9.062	-33.438	1.00	93.69	C
ATOM	761	O	ARG	A	99	57.312	-9.352	-36.375	1.00	93.69	O
ATOM	762	CG	ARG	A	99	58.656	-8.406	-32.156	1.00	93.69	C
ATOM	763	CD	ARG	A	99	59.625	-7.266	-32.469	1.00	93.69	C
ATOM	764	NE	ARG	A	99	60.438	-6.914	-31.297	1.00	93.69	N

```
ATOM    765  NH1 ARG A  99      61.000  -4.820 -32.094  1.00 93.69           N
ATOM    766  NH2 ARG A  99      61.781  -5.539 -30.047  1.00 93.69           N
ATOM    767  CZ  ARG A  99      61.062  -5.758 -31.141  1.00 93.69           C
ATOM    768  N   GLU A 100      58.281  -7.250 -36.344  1.00 93.56           N
ATOM    769  CA  GLU A 100      58.750  -7.348 -37.719  1.00 93.56           C
ATOM    770  C   GLU A 100      59.938  -8.312 -37.844  1.00 93.56           C
ATOM    771  CB  GLU A 100      59.156  -5.969 -38.250  1.00 93.56           C
ATOM    772  O   GLU A 100      60.812  -8.328 -36.969  1.00 93.56           O
ATOM    773  CG  GLU A 100      59.562  -5.965 -39.719  1.00 93.56           C
ATOM    774  CD  GLU A 100      59.906  -4.578 -40.250  1.00 93.56           C
ATOM    775  OE1 GLU A 100      60.375  -4.465 -41.406  1.00 93.56           O
ATOM    776  OE2 GLU A 100      59.688  -3.596 -39.500  1.00 93.56           O
ATOM    777  N   VAL A 101      59.844  -9.188 -38.781  1.00 94.56           N
ATOM    778  CA  VAL A 101      60.906 -10.141 -39.062  1.00 94.56           C
ATOM    779  C   VAL A 101      61.719  -9.664 -40.250  1.00 94.56           C
ATOM    780  CB  VAL A 101      60.344 -11.555 -39.312  1.00 94.56           C
ATOM    781  O   VAL A 101      61.188  -9.547 -41.375  1.00 94.56           O
ATOM    782  CG1 VAL A 101      61.469 -12.547 -39.562  1.00 94.56           C
ATOM    783  CG2 VAL A 101      59.469 -12.008 -38.156  1.00 94.56           C
ATOM    784  N   THR A 102      63.000  -9.227 -40.000  1.00 93.94           N
ATOM    785  CA  THR A 102      63.938  -8.852 -41.062  1.00 93.94           C
ATOM    786  C   THR A 102      65.000  -9.938 -41.281  1.00 93.94           C
ATOM    787  CB  THR A 102      64.625  -7.512 -40.750  1.00 93.94           C
ATOM    788  O   THR A 102      65.125 -10.867 -40.469  1.00 93.94           O
ATOM    789  CG2 THR A 102      63.625  -6.406 -40.531  1.00 93.94           C
ATOM    790  OG1 THR A 102      65.438  -7.660 -39.594  1.00 93.94           O
ATOM    791  N   THR A 103      65.625  -9.867 -42.375  1.00 91.00           N
ATOM    792  CA  THR A 103      66.688 -10.867 -42.688  1.00 91.00           C
ATOM    793  C   THR A 103      67.688 -10.953 -41.562  1.00 91.00           C
ATOM    794  CB  THR A 103      67.375 -10.539 -44.000  1.00 91.00           C
ATOM    795  O   THR A 103      68.188 -12.039 -41.250  1.00 91.00           O
ATOM    796  CG2 THR A 103      68.250 -11.711 -44.469  1.00 91.00           C
ATOM    797  OG1 THR A 103      66.438 -10.250 -45.000  1.00 91.00           O
ATOM    798  N   ASP A 104      67.938  -9.852 -40.719  1.00 91.88           N
ATOM    799  CA  ASP A 104      69.000  -9.805 -39.656  1.00 91.88           C
ATOM    800  C   ASP A 104      68.375  -9.984 -38.281  1.00 91.88           C
ATOM    801  CB  ASP A 104      69.750  -8.477 -39.719  1.00 91.88           C
ATOM    802  O   ASP A 104      69.062 -10.188 -37.281  1.00 91.88           O
ATOM    803  CG  ASP A 104      70.562  -8.336 -40.969  1.00 91.88           C
ATOM    804  OD1 ASP A 104      71.000  -9.359 -41.562  1.00 91.88           O
ATOM    805  OD2 ASP A 104      70.812  -7.180 -41.375  1.00 91.88           O
ATOM    806  N   ASN A 105      67.000  -9.969 -38.219  1.00 92.75           N
ATOM    807  CA  ASN A 105      66.312 -10.047 -36.906  1.00 92.75           C
ATOM    808  C   ASN A 105      65.250 -11.109 -36.906  1.00 92.75           C
ATOM    809  CB  ASN A 105      65.750  -8.688 -36.531  1.00 92.75           C
ATOM    810  O   ASN A 105      64.062 -10.797 -37.094  1.00 92.75           O
ATOM    811  CG  ASN A 105      66.812  -7.652 -36.250  1.00 92.75           C
ATOM    812  ND2 ASN A 105      66.625  -6.441 -36.781  1.00 92.75           N
ATOM    813  OD1 ASN A 105      67.812  -7.934 -35.594  1.00 92.75           O
ATOM    814  N   PRO A 106      65.625 -12.422 -36.719  1.00 94.62           N
ATOM    815  CA  PRO A 106      64.625 -13.469 -36.594  1.00 94.62           C
ATOM    816  C   PRO A 106      63.844 -13.359 -35.312  1.00 94.62           C
ATOM    817  CB  PRO A 106      65.438 -14.766 -36.656  1.00 94.62           C
ATOM    818  O   PRO A 106      64.312 -12.711 -34.344  1.00 94.62           O
ATOM    819  CG  PRO A 106      66.812 -14.375 -36.188  1.00 94.62           C
ATOM    820  CD  PRO A 106      67.000 -12.922 -36.469  1.00 94.62           C
ATOM    821  N   TYR A 107      62.594 -13.883 -35.250  1.00 95.06           N
ATOM    822  CA  TYR A 107      61.812 -13.930 -34.031  1.00 95.06           C
ATOM    823  C   TYR A 107      61.781 -15.336 -33.438  1.00 95.06           C
ATOM    824  CB  TYR A 107      60.375 -13.461 -34.312  1.00 95.06           C
ATOM    825  O   TYR A 107      61.500 -16.312 -34.125  1.00 95.06           O
ATOM    826  CG  TYR A 107      59.500 -13.383 -33.062  1.00 95.06           C
ATOM    827  CD1 TYR A 107      58.469 -14.305 -32.844  1.00 95.06           C
ATOM    828  CD2 TYR A 107      59.688 -12.383 -32.125  1.00 95.06           C
```

ATOM	829	CE1	TYR	A	107	57.688	-14.234	-31.719	1.00	95.06	C
ATOM	830	CE2	TYR	A	107	58.906	-12.305	-30.984	1.00	95.06	C
ATOM	831	OH	TYR	A	107	57.094	-13.156	-29.672	1.00	95.06	O
ATOM	832	CZ	TYR	A	107	57.906	-13.234	-30.797	1.00	95.06	C
ATOM	833	N	THR	A	108	62.062	-15.438	-32.062	1.00	94.62	N
ATOM	834	CA	THR	A	108	62.219	-16.719	-31.391	1.00	94.62	C
ATOM	835	C	THR	A	108	61.031	-16.984	-30.453	1.00	94.62	C
ATOM	836	CB	THR	A	108	63.531	-16.781	-30.594	1.00	94.62	C
ATOM	837	O	THR	A	108	60.625	-16.078	-29.703	1.00	94.62	O
ATOM	838	CG2	THR	A	108	63.656	-18.109	-29.844	1.00	94.62	C
ATOM	839	OG1	THR	A	108	64.625	-16.641	-31.500	1.00	94.62	O
ATOM	840	N	ILE	A	109	60.438	-18.125	-30.500	1.00	94.56	N
ATOM	841	CA	ILE	A	109	59.406	-18.625	-29.609	1.00	94.56	C
ATOM	842	C	ILE	A	109	59.938	-19.797	-28.797	1.00	94.56	C
ATOM	843	CB	ILE	A	109	58.125	-19.031	-30.375	1.00	94.56	C
ATOM	844	O	ILE	A	109	60.312	-20.828	-29.375	1.00	94.56	O
ATOM	845	CG1	ILE	A	109	57.625	-17.859	-31.250	1.00	94.56	C
ATOM	846	CG2	ILE	A	109	57.031	-19.500	-29.422	1.00	94.56	C
ATOM	847	CD1	ILE	A	109	56.656	-18.266	-32.344	1.00	94.56	C
ATOM	848	N	SER	A	110	59.938	-19.625	-27.422	1.00	93.19	N
ATOM	849	CA	SER	A	110	60.375	-20.703	-26.531	1.00	93.19	C
ATOM	850	C	SER	A	110	59.188	-21.453	-25.938	1.00	93.19	C
ATOM	851	CB	SER	A	110	61.281	-20.141	-25.422	1.00	93.19	C
ATOM	852	O	SER	A	110	58.312	-20.844	-25.312	1.00	93.19	O
ATOM	853	OG	SER	A	110	61.750	-21.188	-24.594	1.00	93.19	O
ATOM	854	N	VAL	A	111	59.188	-22.750	-26.156	1.00	92.00	N
ATOM	855	CA	VAL	A	111	58.125	-23.594	-25.641	1.00	92.00	C
ATOM	856	C	VAL	A	111	58.688	-24.578	-24.609	1.00	92.00	C
ATOM	857	CB	VAL	A	111	57.406	-24.359	-26.766	1.00	92.00	C
ATOM	858	O	VAL	A	111	59.531	-25.391	-24.938	1.00	92.00	O
ATOM	859	CG1	VAL	A	111	56.250	-25.203	-26.219	1.00	92.00	C
ATOM	860	CG2	VAL	A	111	56.906	-23.391	-27.844	1.00	92.00	C
ATOM	861	N	THR	A	112	58.094	-24.516	-23.281	1.00	90.88	N
ATOM	862	CA	THR	A	112	58.562	-25.422	-22.234	1.00	90.88	C
ATOM	863	C	THR	A	112	57.438	-26.375	-21.828	1.00	90.88	C
ATOM	864	CB	THR	A	112	59.062	-24.641	-21.000	1.00	90.88	C
ATOM	865	O	THR	A	112	57.688	-27.422	-21.203	1.00	90.88	O
ATOM	866	CG2	THR	A	112	60.312	-23.844	-21.328	1.00	90.88	C
ATOM	867	OG1	THR	A	112	58.031	-23.750	-20.562	1.00	90.88	O
ATOM	868	N	ASN	A	113	56.094	-26.094	-22.203	1.00	88.69	N
ATOM	869	CA	ASN	A	113	54.906	-26.875	-21.859	1.00	88.69	C
ATOM	870	C	ASN	A	113	54.812	-28.156	-22.703	1.00	88.69	C
ATOM	871	CB	ASN	A	113	53.625	-26.047	-22.000	1.00	88.69	C
ATOM	872	O	ASN	A	113	54.531	-28.078	-23.891	1.00	88.69	O
ATOM	873	CG	ASN	A	113	52.406	-26.766	-21.484	1.00	88.69	C
ATOM	874	ND2	ASN	A	113	51.344	-26.016	-21.266	1.00	88.69	N
ATOM	875	OD1	ASN	A	113	52.406	-27.984	-21.297	1.00	88.69	O
ATOM	876	N	LYS	A	114	55.031	-29.344	-22.109	1.00	82.81	N
ATOM	877	CA	LYS	A	114	55.062	-30.625	-22.812	1.00	82.81	C
ATOM	878	C	LYS	A	114	53.656	-31.062	-23.219	1.00	82.81	C
ATOM	879	CB	LYS	A	114	55.719	-31.688	-21.938	1.00	82.81	C
ATOM	880	O	LYS	A	114	53.500	-32.031	-23.969	1.00	82.81	O
ATOM	881	CG	LYS	A	114	57.219	-31.500	-21.766	1.00	82.81	C
ATOM	882	CD	LYS	A	114	57.844	-32.656	-20.969	1.00	82.81	C
ATOM	883	CE	LYS	A	114	59.344	-32.469	-20.766	1.00	82.81	C
ATOM	884	NZ	LYS	A	114	59.938	-33.594	-20.000	1.00	82.81	N
ATOM	885	N	THR	A	115	52.625	-30.422	-22.672	1.00	84.19	N
ATOM	886	CA	THR	A	115	51.250	-30.828	-22.969	1.00	84.19	C
ATOM	887	C	THR	A	115	50.812	-30.297	-24.328	1.00	84.19	C
ATOM	888	CB	THR	A	115	50.281	-30.328	-21.875	1.00	84.19	C
ATOM	889	O	THR	A	115	49.781	-30.734	-24.875	1.00	84.19	O
ATOM	890	CG2	THR	A	115	50.594	-30.938	-20.516	1.00	84.19	C
ATOM	891	OG1	THR	A	115	50.344	-28.906	-21.797	1.00	84.19	O
ATOM	892	N	LEU	A	116	51.656	-29.359	-24.922	1.00	86.69	N

ATOM	893	CA	LEU	A	116	51.312	-28.734	-26.203	1.00	86.69	C
ATOM	894	C	LEU	A	116	51.594	-29.672	-27.359	1.00	86.69	C
ATOM	895	CB	LEU	A	116	52.062	-27.422	-26.375	1.00	86.69	C
ATOM	896	O	LEU	A	116	52.562	-30.406	-27.344	1.00	86.69	O
ATOM	897	CG	LEU	A	116	51.781	-26.312	-25.359	1.00	86.69	C
ATOM	898	CD1	LEU	A	116	52.656	-25.094	-25.625	1.00	86.69	C
ATOM	899	CD2	LEU	A	116	50.312	-25.938	-25.375	1.00	86.69	C
ATOM	900	N	SER	A	117	50.688	-29.703	-28.328	1.00	86.19	N
ATOM	901	CA	SER	A	117	50.812	-30.531	-29.516	1.00	86.19	C
ATOM	902	C	SER	A	117	51.531	-29.781	-30.641	1.00	86.19	C
ATOM	903	CB	SER	A	117	49.438	-31.000	-30.000	1.00	86.19	C
ATOM	904	O	SER	A	117	52.250	-30.391	-31.422	1.00	86.19	O
ATOM	905	OG	SER	A	117	48.750	-31.703	-28.969	1.00	86.19	O
ATOM	906	N	ALA	A	118	51.375	-28.453	-30.734	1.00	90.12	N
ATOM	907	CA	ALA	A	118	51.875	-27.656	-31.859	1.00	90.12	C
ATOM	908	C	ALA	A	118	51.844	-26.172	-31.531	1.00	90.12	C
ATOM	909	CB	ALA	A	118	51.094	-27.938	-33.125	1.00	90.12	C
ATOM	910	O	ALA	A	118	51.281	-25.750	-30.516	1.00	90.12	O
ATOM	911	N	ILE	A	119	52.594	-25.406	-32.250	1.00	92.00	N
ATOM	912	CA	ILE	A	119	52.500	-23.953	-32.281	1.00	92.00	C
ATOM	913	C	ILE	A	119	51.969	-23.500	-33.625	1.00	92.00	C
ATOM	914	CB	ILE	A	119	53.875	-23.312	-32.000	1.00	92.00	C
ATOM	915	O	ILE	A	119	52.406	-24.000	-34.688	1.00	92.00	O
ATOM	916	CG1	ILE	A	119	54.375	-23.688	-30.609	1.00	92.00	C
ATOM	917	CG2	ILE	A	119	53.812	-21.797	-32.156	1.00	92.00	C
ATOM	918	CD1	ILE	A	119	53.688	-22.953	-29.484	1.00	92.00	C
ATOM	919	N	ARG	A	120	51.000	-22.672	-33.625	1.00	93.12	N
ATOM	920	CA	ARG	A	120	50.469	-22.047	-34.844	1.00	93.12	C
ATOM	921	C	ARG	A	120	50.906	-20.594	-34.969	1.00	93.12	C
ATOM	922	CB	ARG	A	120	48.938	-22.125	-34.875	1.00	93.12	C
ATOM	923	O	ARG	A	120	50.562	-19.766	-34.125	1.00	93.12	O
ATOM	924	CG	ARG	A	120	48.312	-21.672	-36.188	1.00	93.12	C
ATOM	925	CD	ARG	A	120	46.781	-21.797	-36.156	1.00	93.12	C
ATOM	926	NE	ARG	A	120	46.375	-23.188	-36.000	1.00	93.12	N
ATOM	927	NH1	ARG	A	120	44.219	-22.688	-35.312	1.00	93.12	N
ATOM	928	NH2	ARG	A	120	44.906	-24.875	-35.500	1.00	93.12	N
ATOM	929	CZ	ARG	A	120	45.156	-23.578	-35.594	1.00	93.12	C
ATOM	930	N	ILE	A	121	51.688	-20.297	-35.969	1.00	93.44	N
ATOM	931	CA	ILE	A	121	52.312	-19.000	-36.156	1.00	93.44	C
ATOM	932	C	ILE	A	121	51.500	-18.203	-37.188	1.00	93.44	C
ATOM	933	CB	ILE	A	121	53.781	-19.125	-36.625	1.00	93.44	C
ATOM	934	O	ILE	A	121	51.250	-18.672	-38.281	1.00	93.44	O
ATOM	935	CG1	ILE	A	121	54.562	-19.938	-35.594	1.00	93.44	C
ATOM	936	CG2	ILE	A	121	54.375	-17.750	-36.844	1.00	93.44	C
ATOM	937	CD1	ILE	A	121	55.938	-20.391	-36.094	1.00	93.44	C
ATOM	938	N	LYS	A	122	51.094	-17.016	-36.750	1.00	94.19	N
ATOM	939	CA	LYS	A	122	50.375	-16.109	-37.656	1.00	94.19	C
ATOM	940	C	LYS	A	122	51.281	-15.047	-38.219	1.00	94.19	C
ATOM	941	CB	LYS	A	122	49.188	-15.461	-36.906	1.00	94.19	C
ATOM	942	O	LYS	A	122	51.812	-14.211	-37.469	1.00	94.19	O
ATOM	943	CG	LYS	A	122	48.250	-14.672	-37.812	1.00	94.19	C
ATOM	944	CD	LYS	A	122	47.094	-14.078	-37.031	1.00	94.19	C
ATOM	945	CE	LYS	A	122	46.000	-15.125	-36.781	1.00	94.19	C
ATOM	946	NZ	LYS	A	122	44.875	-14.562	-35.969	1.00	94.19	N
ATOM	947	N	MET	A	123	51.500	-15.086	-39.531	1.00	94.56	N
ATOM	948	CA	MET	A	123	52.281	-14.086	-40.250	1.00	94.56	C
ATOM	949	C	MET	A	123	51.406	-12.953	-40.750	1.00	94.56	C
ATOM	950	CB	MET	A	123	53.031	-14.719	-41.406	1.00	94.56	C
ATOM	951	O	MET	A	123	50.250	-13.180	-41.156	1.00	94.56	O
ATOM	952	CG	MET	A	123	53.969	-15.852	-41.000	1.00	94.56	C
ATOM	953	SD	MET	A	123	55.250	-15.312	-39.812	1.00	94.56	S
ATOM	954	CE	MET	A	123	56.219	-14.172	-40.875	1.00	94.56	C
ATOM	955	N	PHE	A	124	51.875	-11.672	-40.656	1.00	94.75	N
ATOM	956	CA	PHE	A	124	51.125	-10.508	-41.094	1.00	94.75	C

ATOM	957	C	PHE	A	124	51.969	-9.625	-42.000	1.00	94.75	C
ATOM	958	CB	PHE	A	124	50.656	-9.695	-39.875	1.00	94.75	C
ATOM	959	O	PHE	A	124	53.125	-9.305	-41.688	1.00	94.75	O
ATOM	960	CG	PHE	A	124	49.844	-8.469	-40.250	1.00	94.75	C
ATOM	961	CD1	PHE	A	124	50.344	-7.199	-39.969	1.00	94.75	C
ATOM	962	CD2	PHE	A	124	48.625	-8.578	-40.875	1.00	94.75	C
ATOM	963	CE1	PHE	A	124	49.625	-6.059	-40.312	1.00	94.75	C
ATOM	964	CE2	PHE	A	124	47.906	-7.441	-41.219	1.00	94.75	C
ATOM	965	CZ	PHE	A	124	48.406	-6.184	-40.938	1.00	94.75	C
ATOM	966	N	MET	A	125	51.406	-9.336	-43.219	1.00	94.69	N
ATOM	967	CA	MET	A	125	51.938	-8.359	-44.156	1.00	94.69	C
ATOM	968	C	MET	A	125	51.062	-7.129	-44.250	1.00	94.69	C
ATOM	969	CB	MET	A	125	52.125	-8.977	-45.531	1.00	94.69	C
ATOM	970	O	MET	A	125	49.969	-7.184	-44.844	1.00	94.69	O
ATOM	971	CG	MET	A	125	52.719	-8.031	-46.562	1.00	94.69	C
ATOM	972	SD	MET	A	125	52.969	-8.820	-48.219	1.00	94.69	S
ATOM	973	CE	MET	A	125	54.500	-9.750	-47.906	1.00	94.69	C
ATOM	974	N	PRO	A	126	51.562	-6.035	-43.562	1.00	92.31	N
ATOM	975	CA	PRO	A	126	50.750	-4.828	-43.531	1.00	92.31	C
ATOM	976	C	PRO	A	126	50.469	-4.293	-44.938	1.00	92.31	C
ATOM	977	CB	PRO	A	126	51.594	-3.834	-42.750	1.00	92.31	C
ATOM	978	O	PRO	A	126	49.312	-3.893	-45.219	1.00	92.31	O
ATOM	979	CG	PRO	A	126	52.531	-4.684	-41.938	1.00	92.31	C
ATOM	980	CD	PRO	A	126	52.719	-5.977	-42.688	1.00	92.31	C
ATOM	981	N	ARG	A	127	51.469	-4.223	-45.781	1.00	91.94	N
ATOM	982	CA	ARG	A	127	51.375	-3.793	-47.188	1.00	91.94	C
ATOM	983	C	ARG	A	127	52.344	-4.574	-48.062	1.00	91.94	C
ATOM	984	CB	ARG	A	127	51.656	-2.293	-47.312	1.00	91.94	C
ATOM	985	O	ARG	A	127	53.469	-4.832	-47.688	1.00	91.94	O
ATOM	986	CG	ARG	A	127	50.594	-1.420	-46.656	1.00	91.94	C
ATOM	987	CD	ARG	A	127	50.875	0.062	-46.812	1.00	91.94	C
ATOM	988	NE	ARG	A	127	50.719	0.478	-48.219	1.00	91.94	N
ATOM	989	NH1	ARG	A	127	51.938	2.424	-48.000	1.00	91.94	N
ATOM	990	NH2	ARG	A	127	51.031	1.872	-50.031	1.00	91.94	N
ATOM	991	CZ	ARG	A	127	51.250	1.591	-48.750	1.00	91.94	C
ATOM	992	N	GLY	A	128	51.812	-5.062	-49.125	1.00	94.06	N
ATOM	993	CA	GLY	A	128	52.625	-5.703	-50.156	1.00	94.06	C
ATOM	994	C	GLY	A	128	52.406	-5.160	-51.531	1.00	94.06	C
ATOM	995	O	GLY	A	128	51.500	-5.594	-52.250	1.00	94.06	O
ATOM	996	N	VAL	A	129	53.344	-4.250	-51.938	1.00	95.44	N
ATOM	997	CA	VAL	A	129	53.125	-3.623	-53.250	1.00	95.44	C
ATOM	998	C	VAL	A	129	54.469	-3.195	-53.844	1.00	95.44	C
ATOM	999	CB	VAL	A	129	52.188	-2.410	-53.125	1.00	95.44	C
ATOM	1000	O	VAL	A	129	55.375	-2.748	-53.125	1.00	95.44	O
ATOM	1001	CG1	VAL	A	129	52.781	-1.343	-52.219	1.00	95.44	C
ATOM	1002	CG2	VAL	A	129	51.906	-1.828	-54.531	1.00	95.44	C
ATOM	1003	N	ARG	A	130	54.562	-3.432	-55.062	1.00	94.81	N
ATOM	1004	CA	ARG	A	130	55.688	-2.924	-55.844	1.00	94.81	C
ATOM	1005	C	ARG	A	130	55.219	-1.902	-56.875	1.00	94.81	C
ATOM	1006	CB	ARG	A	130	56.438	-4.070	-56.531	1.00	94.81	C
ATOM	1007	O	ARG	A	130	54.375	-2.203	-57.688	1.00	94.81	O
ATOM	1008	CG	ARG	A	130	57.594	-3.619	-57.406	1.00	94.81	C
ATOM	1009	CD	ARG	A	130	58.219	-4.785	-58.156	1.00	94.81	C
ATOM	1010	NE	ARG	A	130	59.031	-5.629	-57.281	1.00	94.81	N
ATOM	1011	NH1	ARG	A	130	61.062	-4.809	-58.000	1.00	94.81	N
ATOM	1012	NH2	ARG	A	130	60.969	-6.434	-56.375	1.00	94.81	N
ATOM	1013	CZ	ARG	A	130	60.344	-5.621	-57.219	1.00	94.81	C
ATOM	1014	N	ILE	A	131	55.781	-0.660	-56.781	1.00	93.25	N
ATOM	1015	CA	ILE	A	131	55.500	0.382	-57.750	1.00	93.25	C
ATOM	1016	C	ILE	A	131	56.531	0.362	-58.844	1.00	93.25	C
ATOM	1017	CB	ILE	A	131	55.438	1.778	-57.094	1.00	93.25	C
ATOM	1018	O	ILE	A	131	57.719	0.598	-58.594	1.00	93.25	O
ATOM	1019	CG1	ILE	A	131	54.438	1.784	-55.938	1.00	93.25	C
ATOM	1020	CG2	ILE	A	131	55.094	2.854	-58.125	1.00	93.25	C

ATOM	1021	CD1	ILE	A	131	54.500	3.023	-55.062	1.00	93.25	C
ATOM	1022	N	GLU	A	132	56.094	-0.037	-60.062	1.00	90.50	N
ATOM	1023	CA	GLU	A	132	57.000	-0.129	-61.219	1.00	90.50	C
ATOM	1024	C	GLU	A	132	57.344	1.255	-61.750	1.00	90.50	C
ATOM	1025	CB	GLU	A	132	56.375	-0.996	-62.312	1.00	90.50	C
ATOM	1026	O	GLU	A	132	56.656	2.236	-61.438	1.00	90.50	O
ATOM	1027	CG	GLU	A	132	56.031	-2.404	-61.844	1.00	90.50	C
ATOM	1028	CD	GLU	A	132	57.250	-3.303	-61.688	1.00	90.50	C
ATOM	1029	OE1	GLU	A	132	57.094	-4.438	-61.188	1.00	90.50	O
ATOM	1030	OE2	GLU	A	132	58.344	-2.867	-62.062	1.00	90.50	O
ATOM	1031	N	SER	A	133	58.406	1.431	-62.406	1.00	84.50	N
ATOM	1032	CA	SER	A	133	58.875	2.688	-63.000	1.00	84.50	C
ATOM	1033	C	SER	A	133	57.781	3.342	-63.844	1.00	84.50	C
ATOM	1034	CB	SER	A	133	60.125	2.459	-63.812	1.00	84.50	C
ATOM	1035	O	SER	A	133	57.750	4.566	-63.969	1.00	84.50	O
ATOM	1036	OG	SER	A	133	59.938	1.429	-64.750	1.00	84.50	O
ATOM	1037	N	ASN	A	134	56.781	2.547	-64.438	1.00	83.88	N
ATOM	1038	CA	ASN	A	134	55.719	3.064	-65.312	1.00	83.88	C
ATOM	1039	C	ASN	A	134	54.469	3.434	-64.438	1.00	83.88	C
ATOM	1040	CB	ASN	A	134	55.344	2.047	-66.375	1.00	83.88	C
ATOM	1041	O	ASN	A	134	53.438	3.764	-65.000	1.00	83.88	O
ATOM	1042	CG	ASN	A	134	54.844	0.738	-65.812	1.00	83.88	C
ATOM	1043	ND2	ASN	A	134	54.500	-0.212	-66.688	1.00	83.88	N
ATOM	1044	OD1	ASN	A	134	54.750	0.582	-64.562	1.00	83.88	O
ATOM	1045	N	GLY	A	135	54.625	3.451	-63.094	1.00	84.69	N
ATOM	1046	CA	GLY	A	135	53.531	3.805	-62.219	1.00	84.69	C
ATOM	1047	C	GLY	A	135	52.625	2.631	-61.875	1.00	84.69	C
ATOM	1048	O	GLY	A	135	51.750	2.746	-61.031	1.00	84.69	O
ATOM	1049	N	ASP	A	136	52.844	1.382	-62.531	1.00	89.44	N
ATOM	1050	CA	ASP	A	136	52.031	0.198	-62.219	1.00	89.44	C
ATOM	1051	C	ASP	A	136	52.281	-0.301	-60.812	1.00	89.44	C
ATOM	1052	CB	ASP	A	136	52.312	-0.914	-63.219	1.00	89.44	C
ATOM	1053	O	ASP	A	136	53.406	-0.240	-60.312	1.00	89.44	O
ATOM	1054	CG	ASP	A	136	51.781	-0.616	-64.625	1.00	89.44	C
ATOM	1055	OD1	ASP	A	136	51.062	0.393	-64.812	1.00	89.44	O
ATOM	1056	OD2	ASP	A	136	52.094	-1.398	-65.562	1.00	89.44	O
ATOM	1057	N	LYS	A	137	51.250	-0.652	-60.125	1.00	91.81	N
ATOM	1058	CA	LYS	A	137	51.312	-1.264	-58.781	1.00	91.81	C
ATOM	1059	C	LYS	A	137	51.094	-2.770	-58.875	1.00	91.81	C
ATOM	1060	CB	LYS	A	137	50.312	-0.622	-57.844	1.00	91.81	C
ATOM	1061	O	LYS	A	137	50.000	-3.215	-59.250	1.00	91.81	O
ATOM	1062	CG	LYS	A	137	50.625	0.832	-57.500	1.00	91.81	C
ATOM	1063	CD	LYS	A	137	49.625	1.389	-56.500	1.00	91.81	C
ATOM	1064	CE	LYS	A	137	49.875	2.859	-56.219	1.00	91.81	C
ATOM	1065	NZ	LYS	A	137	48.875	3.420	-55.250	1.00	91.81	N
ATOM	1066	N	ASN	A	138	52.156	-3.586	-58.594	1.00	93.44	N
ATOM	1067	CA	ASN	A	138	52.094	-5.043	-58.625	1.00	93.44	C
ATOM	1068	C	ASN	A	138	52.312	-5.648	-57.250	1.00	93.44	C
ATOM	1069	CB	ASN	A	138	53.125	-5.594	-59.625	1.00	93.44	C
ATOM	1070	O	ASN	A	138	52.625	-4.934	-56.312	1.00	93.44	O
ATOM	1071	CG	ASN	A	138	52.844	-5.172	-61.062	1.00	93.44	C
ATOM	1072	ND2	ASN	A	138	53.875	-4.871	-61.812	1.00	93.44	N
ATOM	1073	OD1	ASN	A	138	51.688	-5.117	-61.469	1.00	93.44	O
ATOM	1074	N	GLY	A	139	52.094	-6.926	-57.062	1.00	93.69	N
ATOM	1075	CA	GLY	A	139	52.281	-7.668	-55.844	1.00	93.69	C
ATOM	1076	C	GLY	A	139	53.719	-7.969	-55.531	1.00	93.69	C
ATOM	1077	O	GLY	A	139	54.594	-7.816	-56.406	1.00	93.69	O
ATOM	1078	N	VAL	A	140	54.125	-8.344	-54.344	1.00	94.81	N
ATOM	1079	CA	VAL	A	140	55.438	-8.719	-53.875	1.00	94.81	C
ATOM	1080	C	VAL	A	140	55.469	-10.211	-53.562	1.00	94.81	C
ATOM	1081	CB	VAL	A	140	55.875	-7.898	-52.625	1.00	94.81	C
ATOM	1082	O	VAL	A	140	54.438	-10.859	-53.469	1.00	94.81	O
ATOM	1083	CG1	VAL	A	140	56.000	-6.414	-52.969	1.00	94.81	C
ATOM	1084	CG2	VAL	A	140	54.875	-8.102	-51.500	1.00	94.81	C

```
ATOM   1085  N   ARG A 141      56.719 -10.742 -53.594  1.00 95.00           N
ATOM   1086  CA  ARG A 141      56.969 -12.117 -53.188  1.00 95.00           C
ATOM   1087  C   ARG A 141      57.969 -12.188 -52.031  1.00 95.00           C
ATOM   1088  CB  ARG A 141      57.438 -12.961 -54.344  1.00 95.00           C
ATOM   1089  O   ARG A 141      59.125 -11.773 -52.188  1.00 95.00           O
ATOM   1090  CG  ARG A 141      57.656 -14.430 -54.031  1.00 95.00           C
ATOM   1091  CD  ARG A 141      58.094 -15.219 -55.250  1.00 95.00           C
ATOM   1092  NE  ARG A 141      58.156 -16.656 -54.969  1.00 95.00           N
ATOM   1093  NH1 ARG A 141      60.281 -16.594 -54.031  1.00 95.00           N
ATOM   1094  NH2 ARG A 141      59.125 -18.578 -54.188  1.00 95.00           N
ATOM   1095  CZ  ARG A 141      59.188 -17.281 -54.406  1.00 95.00           C
ATOM   1096  N   VAL A 142      57.562 -12.555 -50.719  1.00 95.31           N
ATOM   1097  CA  VAL A 142      58.438 -12.664 -49.562  1.00 95.31           C
ATOM   1098  C   VAL A 142      58.469 -14.102 -49.062  1.00 95.31           C
ATOM   1099  CB  VAL A 142      58.000 -11.711 -48.438  1.00 95.31           C
ATOM   1100  O   VAL A 142      57.406 -14.680 -48.781  1.00 95.31           O
ATOM   1101  CG1 VAL A 142      58.938 -11.836 -47.219  1.00 95.31           C
ATOM   1102  CG2 VAL A 142      57.969 -10.266 -48.938  1.00 95.31           C
ATOM   1103  N   GLU A 143      59.625 -14.711 -49.062  1.00 96.62           N
ATOM   1104  CA  GLU A 143      59.844 -16.062 -48.562  1.00 96.62           C
ATOM   1105  C   GLU A 143      60.375 -16.047 -47.125  1.00 96.62           C
ATOM   1106  CB  GLU A 143      60.812 -16.828 -49.469  1.00 96.62           C
ATOM   1107  O   GLU A 143      61.250 -15.258 -46.812  1.00 96.62           O
ATOM   1108  CG  GLU A 143      60.938 -18.312 -49.125  1.00 96.62           C
ATOM   1109  CD  GLU A 143      61.812 -19.078 -50.094  1.00 96.62           C
ATOM   1110  OE1 GLU A 143      61.875 -20.328 -50.000  1.00 96.62           O
ATOM   1111  OE2 GLU A 143      62.438 -18.438 -50.969  1.00 96.62           O
ATOM   1112  N   TYR A 144      59.750 -16.828 -46.156  1.00 96.19           N
ATOM   1113  CA  TYR A 144      60.219 -16.984 -44.781  1.00 96.19           C
ATOM   1114  C   TYR A 144      60.219 -18.453 -44.375  1.00 96.19           C
ATOM   1115  CB  TYR A 144      59.312 -16.188 -43.844  1.00 96.19           C
ATOM   1116  O   TYR A 144      59.656 -19.297 -45.062  1.00 96.19           O
ATOM   1117  CG  TYR A 144      57.938 -16.781 -43.656  1.00 96.19           C
ATOM   1118  CD1 TYR A 144      56.906 -16.469 -44.562  1.00 96.19           C
ATOM   1119  CD2 TYR A 144      57.656 -17.641 -42.594  1.00 96.19           C
ATOM   1120  CE1 TYR A 144      55.656 -17.016 -44.406  1.00 96.19           C
ATOM   1121  CE2 TYR A 144      56.375 -18.188 -42.438  1.00 96.19           C
ATOM   1122  OH  TYR A 144      54.125 -18.406 -43.188  1.00 96.19           O
ATOM   1123  CZ  TYR A 144      55.375 -17.875 -43.344  1.00 96.19           C
ATOM   1124  N   GLU A 145      61.031 -18.781 -43.312  1.00 96.50           N
ATOM   1125  CA  GLU A 145      61.094 -20.141 -42.781  1.00 96.50           C
ATOM   1126  C   GLU A 145      60.906 -20.156 -41.281  1.00 96.50           C
ATOM   1127  CB  GLU A 145      62.438 -20.797 -43.156  1.00 96.50           C
ATOM   1128  O   GLU A 145      61.156 -19.141 -40.594  1.00 96.50           O
ATOM   1129  CG  GLU A 145      63.656 -20.109 -42.562  1.00 96.50           C
ATOM   1130  CD  GLU A 145      64.938 -20.734 -42.969  1.00 96.50           C
ATOM   1131  OE1 GLU A 145      66.000 -20.266 -42.531  1.00 96.50           O
ATOM   1132  OE2 GLU A 145      64.938 -21.703 -43.781  1.00 96.50           O
ATOM   1133  N   VAL A 146      60.375 -21.203 -40.781  1.00 95.94           N
ATOM   1134  CA  VAL A 146      60.312 -21.500 -39.344  1.00 95.94           C
ATOM   1135  C   VAL A 146      61.312 -22.609 -39.000  1.00 95.94           C
ATOM   1136  CB  VAL A 146      58.875 -21.875 -38.906  1.00 95.94           C
ATOM   1137  O   VAL A 146      61.250 -23.688 -39.594  1.00 95.94           O
ATOM   1138  CG1 VAL A 146      58.844 -22.109 -37.406  1.00 95.94           C
ATOM   1139  CG2 VAL A 146      57.906 -20.797 -39.312  1.00 95.94           C
ATOM   1140  N   GLN A 147      62.250 -22.281 -38.094  1.00 95.88           N
ATOM   1141  CA  GLN A 147      63.250 -23.250 -37.625  1.00 95.88           C
ATOM   1142  C   GLN A 147      62.969 -23.672 -36.188  1.00 95.88           C
ATOM   1143  CB  GLN A 147      64.625 -22.656 -37.750  1.00 95.88           C
ATOM   1144  O   GLN A 147      62.344 -22.953 -35.438  1.00 95.88           O
ATOM   1145  CG  GLN A 147      65.000 -22.250 -39.188  1.00 95.88           C
ATOM   1146  CD  GLN A 147      66.375 -21.562 -39.250  1.00 95.88           C
ATOM   1147  NE2 GLN A 147      67.000 -21.609 -40.438  1.00 95.88           N
ATOM   1148  OE1 GLN A 147      66.812 -20.984 -38.281  1.00 95.88           O
```

ATOM	1149	N	GLN	A	148	63.344	-24.812	-35.938	1.00	94.56	N
ATOM	1150	CA	GLN	A	148	63.125	-25.391	-34.625	1.00	94.56	C
ATOM	1151	C	GLN	A	148	64.438	-25.953	-34.062	1.00	94.56	C
ATOM	1152	CB	GLN	A	148	62.062	-26.484	-34.688	1.00	94.56	C
ATOM	1153	O	GLN	A	148	65.250	-26.547	-34.781	1.00	94.56	O
ATOM	1154	CG	GLN	A	148	62.031	-27.375	-33.438	1.00	94.56	C
ATOM	1155	CD	GLN	A	148	61.094	-28.562	-33.594	1.00	94.56	C
ATOM	1156	NE2	GLN	A	148	61.188	-29.531	-32.688	1.00	94.56	N
ATOM	1157	OE1	GLN	A	148	60.281	-28.625	-34.531	1.00	94.56	O
ATOM	1158	N	ALA	A	149	64.688	-25.703	-32.688	1.00	94.62	N
ATOM	1159	CA	ALA	A	149	65.750	-26.297	-31.906	1.00	94.62	C
ATOM	1160	C	ALA	A	149	65.188	-27.000	-30.672	1.00	94.62	C
ATOM	1161	CB	ALA	A	149	66.812	-25.250	-31.516	1.00	94.62	C
ATOM	1162	O	ALA	A	149	64.562	-26.391	-29.859	1.00	94.62	O
ATOM	1163	N	VAL	A	150	65.562	-28.234	-30.516	1.00	92.06	N
ATOM	1164	CA	VAL	A	150	65.062	-29.062	-29.438	1.00	92.06	C
ATOM	1165	C	VAL	A	150	66.125	-29.281	-28.375	1.00	92.06	C
ATOM	1166	CB	VAL	A	150	64.500	-30.422	-29.953	1.00	92.06	C
ATOM	1167	O	VAL	A	150	67.250	-29.719	-28.703	1.00	92.06	O
ATOM	1168	CG1	VAL	A	150	63.938	-31.250	-28.812	1.00	92.06	C
ATOM	1169	CG2	VAL	A	150	63.469	-30.219	-31.047	1.00	92.06	C
ATOM	1170	N	ASP	A	151	65.875	-28.797	-27.125	1.00	91.12	N
ATOM	1171	CA	ASP	A	151	66.750	-29.062	-25.969	1.00	91.12	C
ATOM	1172	C	ASP	A	151	68.188	-28.688	-26.234	1.00	91.12	C
ATOM	1173	CB	ASP	A	151	66.625	-30.531	-25.547	1.00	91.12	C
ATOM	1174	O	ASP	A	151	69.062	-29.484	-25.953	1.00	91.12	O
ATOM	1175	CG	ASP	A	151	65.250	-30.922	-25.062	1.00	91.12	C
ATOM	1176	OD1	ASP	A	151	64.562	-30.094	-24.375	1.00	91.12	O
ATOM	1177	OD2	ASP	A	151	64.812	-32.031	-25.359	1.00	91.12	O
ATOM	1178	N	GLY	A	152	68.375	-27.500	-26.844	1.00	85.88	N
ATOM	1179	CA	GLY	A	152	69.750	-26.969	-27.047	1.00	85.88	C
ATOM	1180	C	GLY	A	152	70.375	-27.453	-28.328	1.00	85.88	C
ATOM	1181	O	GLY	A	152	71.562	-27.203	-28.562	1.00	85.88	O
ATOM	1182	N	GLY	A	153	69.750	-28.219	-29.266	1.00	88.31	N
ATOM	1183	CA	GLY	A	153	70.250	-28.672	-30.547	1.00	88.31	C
ATOM	1184	C	GLY	A	153	70.375	-27.547	-31.578	1.00	88.31	C
ATOM	1185	O	GLY	A	153	70.125	-26.391	-31.266	1.00	88.31	O
ATOM	1186	N	SER	A	154	70.875	-27.859	-32.844	1.00	91.50	N
ATOM	1187	CA	SER	A	154	70.938	-26.922	-33.969	1.00	91.50	C
ATOM	1188	C	SER	A	154	69.562	-26.641	-34.531	1.00	91.50	C
ATOM	1189	CB	SER	A	154	71.875	-27.469	-35.062	1.00	91.50	C
ATOM	1190	O	SER	A	154	68.688	-27.469	-34.438	1.00	91.50	O
ATOM	1191	OG	SER	A	154	73.188	-27.688	-34.531	1.00	91.50	O
ATOM	1192	N	PHE	A	155	69.375	-25.391	-34.969	1.00	94.38	N
ATOM	1193	CA	PHE	A	155	68.125	-25.031	-35.625	1.00	94.38	C
ATOM	1194	C	PHE	A	155	68.000	-25.781	-36.938	1.00	94.38	C
ATOM	1195	CB	PHE	A	155	68.062	-23.516	-35.875	1.00	94.38	C
ATOM	1196	O	PHE	A	155	68.875	-25.812	-37.781	1.00	94.38	O
ATOM	1197	CG	PHE	A	155	67.750	-22.703	-34.656	1.00	94.38	C
ATOM	1198	CD1	PHE	A	155	66.438	-22.500	-34.281	1.00	94.38	C
ATOM	1199	CD2	PHE	A	155	68.750	-22.156	-33.875	1.00	94.38	C
ATOM	1200	CE1	PHE	A	155	66.125	-21.750	-33.156	1.00	94.38	C
ATOM	1201	CE2	PHE	A	155	68.500	-21.406	-32.750	1.00	94.38	C
ATOM	1202	CZ	PHE	A	155	67.188	-21.203	-32.375	1.00	94.38	C
ATOM	1203	N	GLU	A	156	66.812	-26.453	-37.125	1.00	93.81	N
ATOM	1204	CA	GLU	A	156	66.438	-27.125	-38.344	1.00	93.81	C
ATOM	1205	C	GLU	A	156	65.125	-26.516	-38.906	1.00	93.81	C
ATOM	1206	CB	GLU	A	156	66.188	-28.625	-38.125	1.00	93.81	C
ATOM	1207	O	GLU	A	156	64.250	-26.156	-38.156	1.00	93.81	O
ATOM	1208	CG	GLU	A	156	67.500	-29.344	-37.656	1.00	93.81	C
ATOM	1209	CD	GLU	A	156	67.312	-30.844	-37.500	1.00	93.81	C
ATOM	1210	OE1	GLU	A	156	68.312	-31.562	-37.281	1.00	93.81	O
ATOM	1211	OE2	GLU	A	156	66.125	-31.312	-37.656	1.00	93.81	O
ATOM	1212	N	THR	A	157	65.062	-26.344	-40.188	1.00	94.38	N

```
ATOM   1213  CA  THR A 157      63.906 -25.797 -40.844  1.00 94.38           C
ATOM   1214  C   THR A 157      62.719 -26.766 -40.750  1.00 94.38           C
ATOM   1215  CB  THR A 157      64.188 -25.453 -42.312  1.00 94.38           C
ATOM   1216  O   THR A 157      62.844 -27.922 -41.188  1.00 94.38           O
ATOM   1217  CG2 THR A 157      62.969 -24.875 -43.000  1.00 94.38           C
ATOM   1218  OG1 THR A 157      65.250 -24.500 -42.375  1.00 94.38           O
ATOM   1219  N   VAL A 158      61.625 -26.312 -40.219  1.00 92.62           N
ATOM   1220  CA  VAL A 158      60.469 -27.172 -40.094  1.00 92.62           C
ATOM   1221  C   VAL A 158      59.344 -26.719 -41.031  1.00 92.62           C
ATOM   1222  CB  VAL A 158      59.938 -27.188 -38.625  1.00 92.62           C
ATOM   1223  O   VAL A 158      58.438 -27.469 -41.312  1.00 92.62           O
ATOM   1224  CG1 VAL A 158      60.906 -27.938 -37.719  1.00 92.62           C
ATOM   1225  CG2 VAL A 158      59.719 -25.766 -38.125  1.00 92.62           C
ATOM   1226  N   LEU A 159      59.438 -25.438 -41.531  1.00 92.81           N
ATOM   1227  CA  LEU A 159      58.438 -24.891 -42.438  1.00 92.81           C
ATOM   1228  C   LEU A 159      59.000 -23.797 -43.312  1.00 92.81           C
ATOM   1229  CB  LEU A 159      57.219 -24.359 -41.656  1.00 92.81           C
ATOM   1230  O   LEU A 159      59.719 -22.906 -42.812  1.00 92.81           O
ATOM   1231  CG  LEU A 159      56.156 -23.594 -42.469  1.00 92.81           C
ATOM   1232  CD1 LEU A 159      55.250 -24.578 -43.188  1.00 92.81           C
ATOM   1233  CD2 LEU A 159      55.344 -22.672 -41.562  1.00 92.81           C
ATOM   1234  N   THR A 160      58.906 -23.922 -44.594  1.00 94.62           N
ATOM   1235  CA  THR A 160      59.156 -22.859 -45.562  1.00 94.62           C
ATOM   1236  C   THR A 160      57.875 -22.406 -46.219  1.00 94.62           C
ATOM   1237  CB  THR A 160      60.156 -23.312 -46.625  1.00 94.62           C
ATOM   1238  O   THR A 160      57.062 -23.234 -46.656  1.00 94.62           O
ATOM   1239  CG2 THR A 160      60.500 -22.172 -47.594  1.00 94.62           C
ATOM   1240  OG1 THR A 160      61.375 -23.750 -46.000  1.00 94.62           O
ATOM   1241  N   ASP A 161      57.656 -21.031 -46.219  1.00 94.25           N
ATOM   1242  CA  ASP A 161      56.438 -20.500 -46.812  1.00 94.25           C
ATOM   1243  C   ASP A 161      56.688 -19.203 -47.562  1.00 94.25           C
ATOM   1244  CB  ASP A 161      55.375 -20.281 -45.719  1.00 94.25           C
ATOM   1245  O   ASP A 161      57.719 -18.562 -47.375  1.00 94.25           O
ATOM   1246  CG  ASP A 161      53.969 -20.312 -46.250  1.00 94.25           C
ATOM   1247  OD1 ASP A 161      53.781 -20.469 -47.469  1.00 94.25           O
ATOM   1248  OD2 ASP A 161      53.031 -20.172 -45.438  1.00 94.25           O
ATOM   1249  N   VAL A 162      55.719 -18.906 -48.531  1.00 94.56           N
ATOM   1250  CA  VAL A 162      55.844 -17.719 -49.375  1.00 94.56           C
ATOM   1251  C   VAL A 162      54.531 -16.922 -49.344  1.00 94.56           C
ATOM   1252  CB  VAL A 162      56.219 -18.094 -50.812  1.00 94.56           C
ATOM   1253  O   VAL A 162      53.469 -17.500 -49.500  1.00 94.56           O
ATOM   1254  CG1 VAL A 162      56.312 -16.844 -51.688  1.00 94.56           C
ATOM   1255  CG2 VAL A 162      57.531 -18.875 -50.844  1.00 94.56           C
ATOM   1256  N   ILE A 163      54.625 -15.562 -49.000  1.00 93.94           N
ATOM   1257  CA  ILE A 163      53.531 -14.617 -49.219  1.00 93.94           C
ATOM   1258  C   ILE A 163      53.750 -13.859 -50.531  1.00 93.94           C
ATOM   1259  CB  ILE A 163      53.438 -13.617 -48.031  1.00 93.94           C
ATOM   1260  O   ILE A 163      54.688 -13.078 -50.656  1.00 93.94           O
ATOM   1261  CG1 ILE A 163      53.281 -14.375 -46.719  1.00 93.94           C
ATOM   1262  CG2 ILE A 163      52.281 -12.641 -48.250  1.00 93.94           C
ATOM   1263  CD1 ILE A 163      53.281 -13.477 -45.469  1.00 93.94           C
ATOM   1264  N   GLU A 164      52.812 -14.188 -51.625  1.00 93.88           N
ATOM   1265  CA  GLU A 164      52.969 -13.609 -52.969  1.00 93.88           C
ATOM   1266  C   GLU A 164      51.656 -12.961 -53.406  1.00 93.88           C
ATOM   1267  CB  GLU A 164      53.406 -14.680 -53.969  1.00 93.88           C
ATOM   1268  O   GLU A 164      50.594 -13.602 -53.406  1.00 93.88           O
ATOM   1269  CG  GLU A 164      53.625 -14.141 -55.375  1.00 93.88           C
ATOM   1270  CD  GLU A 164      54.094 -15.211 -56.344  1.00 93.88           C
ATOM   1271  OE1 GLU A 164      54.438 -14.859 -57.500  1.00 93.88           O
ATOM   1272  OE2 GLU A 164      54.125 -16.391 -55.969  1.00 93.88           O
ATOM   1273  N   GLY A 165      51.656 -11.758 -53.750  1.00 93.94           N
ATOM   1274  CA  GLY A 165      50.531 -11.000 -54.250  1.00 93.94           C
ATOM   1275  C   GLY A 165      50.531  -9.555 -53.812  1.00 93.94           C
ATOM   1276  O   GLY A 165      51.531  -9.047 -53.312  1.00 93.94           O
```

```
ATOM   1277  N   LYS A 166      49.438  -8.820 -54.094  1.00 93.88           N
ATOM   1278  CA  LYS A 166      49.312  -7.418 -53.719  1.00 93.88           C
ATOM   1279  C   LYS A 166      48.281  -7.250 -52.594  1.00 93.88           C
ATOM   1280  CB  LYS A 166      48.906  -6.566 -54.938  1.00 93.88           C
ATOM   1281  O   LYS A 166      47.188  -7.840 -52.656  1.00 93.88           O
ATOM   1282  CG  LYS A 166      48.812  -5.078 -54.625  1.00 93.88           C
ATOM   1283  CD  LYS A 166      48.219  -4.312 -55.812  1.00 93.88           C
ATOM   1284  CE  LYS A 166      47.875  -2.879 -55.438  1.00 93.88           C
ATOM   1285  NZ  LYS A 166      46.406  -2.715 -55.156  1.00 93.88           N
ATOM   1286  N   THR A 167      48.562  -6.492 -51.531  1.00 93.06           N
ATOM   1287  CA  THR A 167      47.656  -6.125 -50.469  1.00 93.06           C
ATOM   1288  C   THR A 167      47.938  -4.711 -49.969  1.00 93.06           C
ATOM   1289  CB  THR A 167      47.750  -7.113 -49.281  1.00 93.06           C
ATOM   1290  O   THR A 167      49.094  -4.340 -49.750  1.00 93.06           O
ATOM   1291  CG2 THR A 167      49.125  -7.082 -48.656  1.00 93.06           C
ATOM   1292  OG1 THR A 167      46.750  -6.758 -48.312  1.00 93.06           O
ATOM   1293  N   MET A 168      46.938  -3.838 -49.906  1.00 88.81           N
ATOM   1294  CA  MET A 168      47.062  -2.488 -49.344  1.00 88.81           C
ATOM   1295  C   MET A 168      46.375  -2.408 -47.969  1.00 88.81           C
ATOM   1296  CB  MET A 168      46.406  -1.472 -50.312  1.00 88.81           C
ATOM   1297  O   MET A 168      46.594  -1.444 -47.250  1.00 88.81           O
ATOM   1298  CG  MET A 168      47.125  -1.368 -51.656  1.00 88.81           C
ATOM   1299  SD  MET A 168      48.781  -0.628 -51.500  1.00 88.81           S
ATOM   1300  CE  MET A 168      48.344   1.075 -51.062  1.00 88.81           C
ATOM   1301  N   SER A 169      45.562  -3.457 -47.594  1.00 84.50           N
ATOM   1302  CA  SER A 169      44.750  -3.447 -46.406  1.00 84.50           C
ATOM   1303  C   SER A 169      45.312  -4.414 -45.344  1.00 84.50           C
ATOM   1304  CB  SER A 169      43.312  -3.807 -46.719  1.00 84.50           C
ATOM   1305  O   SER A 169      44.781  -4.520 -44.250  1.00 84.50           O
ATOM   1306  OG  SER A 169      43.219  -5.055 -47.375  1.00 84.50           O
ATOM   1307  N   GLY A 170      46.562  -5.047 -45.688  1.00 88.62           N
ATOM   1308  CA  GLY A 170      47.125  -6.012 -44.750  1.00 88.62           C
ATOM   1309  C   GLY A 170      46.688  -7.434 -45.000  1.00 88.62           C
ATOM   1310  O   GLY A 170      45.531  -7.664 -45.375  1.00 88.62           O
ATOM   1311  N   TYR A 171      47.531  -8.445 -44.969  1.00 91.56           N
ATOM   1312  CA  TYR A 171      47.312  -9.867 -45.219  1.00 91.56           C
ATOM   1313  C   TYR A 171      47.906 -10.703 -44.094  1.00 91.56           C
ATOM   1314  CB  TYR A 171      47.906 -10.281 -46.562  1.00 91.56           C
ATOM   1315  O   TYR A 171      49.094 -10.508 -43.719  1.00 91.56           O
ATOM   1316  CG  TYR A 171      47.844 -11.773 -46.812  1.00 91.56           C
ATOM   1317  CD1 TYR A 171      49.000 -12.547 -46.750  1.00 91.56           C
ATOM   1318  CD2 TYR A 171      46.656 -12.398 -47.125  1.00 91.56           C
ATOM   1319  CE1 TYR A 171      48.969 -13.922 -47.000  1.00 91.56           C
ATOM   1320  CE2 TYR A 171      46.594 -13.773 -47.344  1.00 91.56           C
ATOM   1321  OH  TYR A 171      47.719 -15.875 -47.531  1.00 91.56           O
ATOM   1322  CZ  TYR A 171      47.750 -14.523 -47.281  1.00 91.56           C
ATOM   1323  N   ASP A 172      47.094 -11.539 -43.438  1.00 91.31           N
ATOM   1324  CA  ASP A 172      47.625 -12.469 -42.438  1.00 91.31           C
ATOM   1325  C   ASP A 172      47.375 -13.914 -42.844  1.00 91.31           C
ATOM   1326  CB  ASP A 172      47.000 -12.195 -41.062  1.00 91.31           C
ATOM   1327  O   ASP A 172      46.438 -14.203 -43.594  1.00 91.31           O
ATOM   1328  CG  ASP A 172      45.500 -12.391 -41.031  1.00 91.31           C
ATOM   1329  OD1 ASP A 172      44.969 -13.055 -41.938  1.00 91.31           O
ATOM   1330  OD2 ASP A 172      44.875 -11.875 -40.094  1.00 91.31           O
ATOM   1331  N   ARG A 173      48.219 -14.805 -42.469  1.00 89.44           N
ATOM   1332  CA  ARG A 173      48.125 -16.234 -42.688  1.00 89.44           C
ATOM   1333  C   ARG A 173      48.688 -17.031 -41.531  1.00 89.44           C
ATOM   1334  CB  ARG A 173      48.812 -16.609 -44.000  1.00 89.44           C
ATOM   1335  O   ARG A 173      49.719 -16.641 -40.969  1.00 89.44           O
ATOM   1336  CG  ARG A 173      50.344 -16.453 -43.938  1.00 89.44           C
ATOM   1337  CD  ARG A 173      51.000 -16.875 -45.250  1.00 89.44           C
ATOM   1338  NE  ARG A 173      51.438 -18.266 -45.219  1.00 89.44           N
ATOM   1339  NH1 ARG A 173      52.188 -18.312 -47.406  1.00 89.44           N
ATOM   1340  NH2 ARG A 173      52.375 -20.172 -46.062  1.00 89.44           N
```

```
ATOM   1341  CZ  ARG A 173      52.000 -18.906 -46.219  1.00 89.44           C
ATOM   1342  N   SER A 174      48.062 -18.141 -41.094  1.00 90.56           N
ATOM   1343  CA  SER A 174      48.531 -18.953 -39.969  1.00 90.56           C
ATOM   1344  C   SER A 174      49.094 -20.281 -40.469  1.00 90.56           C
ATOM   1345  CB  SER A 174      47.375 -19.219 -39.000  1.00 90.56           C
ATOM   1346  O   SER A 174      48.531 -20.906 -41.375  1.00 90.56           O
ATOM   1347  OG  SER A 174      46.875 -18.000 -38.469  1.00 90.56           O
ATOM   1348  N   ARG A 175      50.250 -20.719 -39.875  1.00 91.19           N
ATOM   1349  CA  ARG A 175      50.875 -22.016 -40.156  1.00 91.19           C
ATOM   1350  C   ARG A 175      51.094 -22.797 -38.875  1.00 91.19           C
ATOM   1351  CB  ARG A 175      52.188 -21.828 -40.906  1.00 91.19           C
ATOM   1352  O   ARG A 175      51.625 -22.250 -37.906  1.00 91.19           O
ATOM   1353  CG  ARG A 175      52.062 -21.297 -42.312  1.00 91.19           C
ATOM   1354  CD  ARG A 175      51.406 -22.328 -43.219  1.00 91.19           C
ATOM   1355  NE  ARG A 175      51.344 -21.859 -44.625  1.00 91.19           N
ATOM   1356  NH1 ARG A 175      49.094 -21.359 -44.562  1.00 91.19           N
ATOM   1357  NH2 ARG A 175      50.312 -21.000 -46.469  1.00 91.19           N
ATOM   1358  CZ  ARG A 175      50.250 -21.406 -45.219  1.00 91.19           C
ATOM   1359  N   ARG A 176      50.625 -23.984 -38.844  1.00 90.94           N
ATOM   1360  CA  ARG A 176      50.812 -24.875 -37.688  1.00 90.94           C
ATOM   1361  C   ARG A 176      52.125 -25.641 -37.812  1.00 90.94           C
ATOM   1362  CB  ARG A 176      49.625 -25.859 -37.594  1.00 90.94           C
ATOM   1363  O   ARG A 176      52.438 -26.188 -38.875  1.00 90.94           O
ATOM   1364  CG  ARG A 176      49.781 -26.875 -36.469  1.00 90.94           C
ATOM   1365  CD  ARG A 176      48.594 -27.812 -36.375  1.00 90.94           C
ATOM   1366  NE  ARG A 176      48.812 -28.891 -35.406  1.00 90.94           N
ATOM   1367  NH1 ARG A 176      46.656 -29.719 -35.531  1.00 90.94           N
ATOM   1368  NH2 ARG A 176      48.188 -30.703 -34.156  1.00 90.94           N
ATOM   1369  CZ  ARG A 176      47.875 -29.766 -35.031  1.00 90.94           C
ATOM   1370  N   VAL A 177      52.969 -25.688 -36.719  1.00 90.62           N
ATOM   1371  CA  VAL A 177      54.188 -26.484 -36.594  1.00 90.62           C
ATOM   1372  C   VAL A 177      54.094 -27.422 -35.406  1.00 90.62           C
ATOM   1373  CB  VAL A 177      55.438 -25.594 -36.469  1.00 90.62           C
ATOM   1374  O   VAL A 177      53.906 -26.969 -34.281  1.00 90.62           O
ATOM   1375  CG1 VAL A 177      56.719 -26.422 -36.562  1.00 90.62           C
ATOM   1376  CG2 VAL A 177      55.438 -24.500 -37.562  1.00 90.62           C
ATOM   1377  N   ASN A 178      54.062 -28.750 -35.625  1.00 87.50           N
ATOM   1378  CA  ASN A 178      53.938 -29.750 -34.562  1.00 87.50           C
ATOM   1379  C   ASN A 178      55.219 -29.781 -33.719  1.00 87.50           C
ATOM   1380  CB  ASN A 178      53.656 -31.125 -35.156  1.00 87.50           C
ATOM   1381  O   ASN A 178      56.312 -29.656 -34.219  1.00 87.50           O
ATOM   1382  CG  ASN A 178      52.281 -31.234 -35.750  1.00 87.50           C
ATOM   1383  ND2 ASN A 178      52.094 -32.156 -36.719  1.00 87.50           N
ATOM   1384  OD1 ASN A 178      51.344 -30.516 -35.344  1.00 87.50           O
ATOM   1385  N   LEU A 179      54.906 -29.719 -32.344  1.00 86.38           N
ATOM   1386  CA  LEU A 179      56.031 -29.812 -31.422  1.00 86.38           C
ATOM   1387  C   LEU A 179      56.656 -31.203 -31.484  1.00 86.38           C
ATOM   1388  CB  LEU A 179      55.562 -29.516 -30.000  1.00 86.38           C
ATOM   1389  O   LEU A 179      55.969 -32.219 -31.562  1.00 86.38           O
ATOM   1390  CG  LEU A 179      55.281 -28.047 -29.672  1.00 86.38           C
ATOM   1391  CD1 LEU A 179      54.406 -27.953 -28.422  1.00 86.38           C
ATOM   1392  CD2 LEU A 179      56.594 -27.281 -29.484  1.00 86.38           C
ATOM   1393  N   PRO A 180      58.000 -31.391 -31.594  1.00 84.38           N
ATOM   1394  CA  PRO A 180      58.688 -32.688 -31.422  1.00 84.38           C
ATOM   1395  C   PRO A 180      58.656 -33.188 -29.984  1.00 84.38           C
ATOM   1396  CB  PRO A 180      60.094 -32.406 -31.891  1.00 84.38           C
ATOM   1397  O   PRO A 180      58.188 -32.500 -29.094  1.00 84.38           O
ATOM   1398  CG  PRO A 180      60.375 -30.984 -31.484  1.00 84.38           C
ATOM   1399  CD  PRO A 180      59.094 -30.219 -31.609  1.00 84.38           C
ATOM   1400  N   ASN A 181      58.969 -34.375 -29.812  1.00 81.88           N
ATOM   1401  CA  ASN A 181      59.250 -34.781 -28.438  1.00 81.88           C
ATOM   1402  C   ASN A 181      60.438 -34.031 -27.844  1.00 81.88           C
ATOM   1403  CB  ASN A 181      59.500 -36.281 -28.391  1.00 81.88           C
ATOM   1404  O   ASN A 181      61.438 -33.812 -28.516  1.00 81.88           O
```

```
ATOM   1405  CG  ASN A 181      58.250 -37.094 -28.594  1.00 81.88           C
ATOM   1406  ND2 ASN A 181      58.406 -38.375 -28.969  1.00 81.88           N
ATOM   1407  OD1 ASN A 181      57.125 -36.625 -28.391  1.00 81.88           O
ATOM   1408  N   PHE A 182      60.281 -33.344 -26.656  1.00 86.81           N
ATOM   1409  CA  PHE A 182      61.406 -32.625 -26.047  1.00 86.81           C
ATOM   1410  C   PHE A 182      61.375 -32.812 -24.531  1.00 86.81           C
ATOM   1411  CB  PHE A 182      61.344 -31.141 -26.406  1.00 86.81           C
ATOM   1412  O   PHE A 182      60.344 -33.094 -23.953  1.00 86.81           O
ATOM   1413  CG  PHE A 182      60.156 -30.422 -25.875  1.00 86.81           C
ATOM   1414  CD1 PHE A 182      58.906 -30.500 -26.531  1.00 86.81           C
ATOM   1415  CD2 PHE A 182      60.219 -29.672 -24.703  1.00 86.81           C
ATOM   1416  CE1 PHE A 182      57.812 -29.828 -26.047  1.00 86.81           C
ATOM   1417  CE2 PHE A 182      59.125 -29.000 -24.203  1.00 86.81           C
ATOM   1418  CZ  PHE A 182      57.906 -29.078 -24.875  1.00 86.81           C
ATOM   1419  N   ASN A 183      62.656 -32.656 -23.812  1.00 86.75           N
ATOM   1420  CA  ASN A 183      62.781 -32.812 -22.359  1.00 86.75           C
ATOM   1421  C   ASN A 183      62.625 -31.484 -21.641  1.00 86.75           C
ATOM   1422  CB  ASN A 183      64.125 -33.438 -22.031  1.00 86.75           C
ATOM   1423  O   ASN A 183      61.875 -31.391 -20.672  1.00 86.75           O
ATOM   1424  CG  ASN A 183      64.250 -34.875 -22.500  1.00 86.75           C
ATOM   1425  ND2 ASN A 183      65.438 -35.281 -22.953  1.00 86.75           N
ATOM   1426  OD1 ASN A 183      63.281 -35.625 -22.484  1.00 86.75           O
ATOM   1427  N   ASN A 184      63.312 -30.438 -22.109  1.00 89.69           N
ATOM   1428  CA  ASN A 184      63.375 -29.156 -21.406  1.00 89.69           C
ATOM   1429  C   ASN A 184      62.625 -28.062 -22.172  1.00 89.69           C
ATOM   1430  CB  ASN A 184      64.812 -28.750 -21.156  1.00 89.69           C
ATOM   1431  O   ASN A 184      61.656 -27.500 -21.656  1.00 89.69           O
ATOM   1432  CG  ASN A 184      65.562 -29.719 -20.266  1.00 89.69           C
ATOM   1433  ND2 ASN A 184      66.812 -29.984 -20.578  1.00 89.69           N
ATOM   1434  OD1 ASN A 184      65.000 -30.250 -19.297  1.00 89.69           O
ATOM   1435  N   GLN A 185      63.031 -27.875 -23.500  1.00 93.56           N
ATOM   1436  CA  GLN A 185      62.469 -26.766 -24.250  1.00 93.56           C
ATOM   1437  C   GLN A 185      62.625 -26.953 -25.750  1.00 93.56           C
ATOM   1438  CB  GLN A 185      63.094 -25.438 -23.812  1.00 93.56           C
ATOM   1439  O   GLN A 185      63.531 -27.672 -26.203  1.00 93.56           O
ATOM   1440  CG  GLN A 185      64.500 -25.234 -24.344  1.00 93.56           C
ATOM   1441  CD  GLN A 185      65.062 -23.859 -23.984  1.00 93.56           C
ATOM   1442  NE2 GLN A 185      66.062 -23.406 -24.750  1.00 93.56           N
ATOM   1443  OE1 GLN A 185      64.625 -23.219 -23.031  1.00 93.56           O
ATOM   1444  N   VAL A 186      61.656 -26.531 -26.531  1.00 93.38           N
ATOM   1445  CA  VAL A 186      61.750 -26.344 -27.969  1.00 93.38           C
ATOM   1446  C   VAL A 186      61.719 -24.859 -28.312  1.00 93.38           C
ATOM   1447  CB  VAL A 186      60.625 -27.094 -28.703  1.00 93.38           C
ATOM   1448  O   VAL A 186      60.906 -24.109 -27.766  1.00 93.38           O
ATOM   1449  CG1 VAL A 186      60.688 -26.859 -30.203  1.00 93.38           C
ATOM   1450  CG2 VAL A 186      60.656 -28.578 -28.375  1.00 93.38           C
ATOM   1451  N   ILE A 187      62.688 -24.406 -29.125  1.00 95.38           N
ATOM   1452  CA  ILE A 187      62.781 -23.016 -29.562  1.00 95.38           C
ATOM   1453  C   ILE A 187      62.438 -22.922 -31.047  1.00 95.38           C
ATOM   1454  CB  ILE A 187      64.188 -22.406 -29.281  1.00 95.38           C
ATOM   1455  O   ILE A 187      63.031 -23.609 -31.875  1.00 95.38           O
ATOM   1456  CG1 ILE A 187      64.500 -22.516 -27.797  1.00 95.38           C
ATOM   1457  CG2 ILE A 187      64.250 -20.969 -29.766  1.00 95.38           C
ATOM   1458  CD1 ILE A 187      65.938 -22.078 -27.453  1.00 95.38           C
ATOM   1459  N   PHE A 188      61.406 -22.203 -31.391  1.00 95.12           N
ATOM   1460  CA  PHE A 188      61.094 -21.875 -32.781  1.00 95.12           C
ATOM   1461  C   PHE A 188      61.625 -20.516 -33.156  1.00 95.12           C
ATOM   1462  CB  PHE A 188      59.594 -21.938 -33.000  1.00 95.12           C
ATOM   1463  O   PHE A 188      61.562 -19.562 -32.375  1.00 95.12           O
ATOM   1464  CG  PHE A 188      59.000 -23.328 -32.906  1.00 95.12           C
ATOM   1465  CD1 PHE A 188      59.062 -24.188 -34.000  1.00 95.12           C
ATOM   1466  CD2 PHE A 188      58.406 -23.766 -31.750  1.00 95.12           C
ATOM   1467  CE1 PHE A 188      58.531 -25.469 -33.906  1.00 95.12           C
ATOM   1468  CE2 PHE A 188      57.875 -25.047 -31.656  1.00 95.12           C
```

```
ATOM   1469  CZ  PHE A 188      57.938 -25.891 -32.750  1.00 95.12           C
ATOM   1470  N   ARG A 189      62.156 -20.484 -34.281  1.00 94.94           N
ATOM   1471  CA  ARG A 189      62.688 -19.250 -34.844  1.00 94.94           C
ATOM   1472  C   ARG A 189      62.094 -18.969 -36.219  1.00 94.94           C
ATOM   1473  CB  ARG A 189      64.188 -19.312 -34.938  1.00 94.94           C
ATOM   1474  O   ARG A 189      62.156 -19.812 -37.125  1.00 94.94           O
ATOM   1475  CG  ARG A 189      64.875 -18.000 -35.406  1.00 94.94           C
ATOM   1476  CD  ARG A 189      66.375 -18.109 -35.438  1.00 94.94           C
ATOM   1477  NE  ARG A 189      66.812 -19.344 -36.062  1.00 94.94           N
ATOM   1478  NH1 ARG A 189      69.062 -18.875 -35.812  1.00 94.94           N
ATOM   1479  NH2 ARG A 189      68.375 -20.828 -36.812  1.00 94.94           N
ATOM   1480  CZ  ARG A 189      68.125 -19.688 -36.219  1.00 94.94           C
ATOM   1481  N   VAL A 190      61.375 -17.781 -36.406  1.00 96.19           N
ATOM   1482  CA  VAL A 190      60.844 -17.344 -37.688  1.00 96.19           C
ATOM   1483  C   VAL A 190      61.875 -16.469 -38.406  1.00 96.19           C
ATOM   1484  CB  VAL A 190      59.531 -16.578 -37.531  1.00 96.19           C
ATOM   1485  O   VAL A 190      62.281 -15.414 -37.875  1.00 96.19           O
ATOM   1486  CG1 VAL A 190      58.938 -16.203 -38.875  1.00 96.19           C
ATOM   1487  CG2 VAL A 190      58.531 -17.406 -36.719  1.00 96.19           C
ATOM   1488  N   VAL A 191      62.250 -16.891 -39.594  1.00 95.75           N
ATOM   1489  CA  VAL A 191      63.344 -16.250 -40.344  1.00 95.75           C
ATOM   1490  C   VAL A 191      62.844 -15.805 -41.719  1.00 95.75           C
ATOM   1491  CB  VAL A 191      64.562 -17.172 -40.469  1.00 95.75           C
ATOM   1492  O   VAL A 191      62.250 -16.594 -42.438  1.00 95.75           O
ATOM   1493  CG1 VAL A 191      65.688 -16.469 -41.219  1.00 95.75           C
ATOM   1494  CG2 VAL A 191      65.000 -17.656 -39.094  1.00 95.75           C
ATOM   1495  N   ARG A 192      63.062 -14.500 -42.000  1.00 96.69           N
ATOM   1496  CA  ARG A 192      62.812 -14.023 -43.344  1.00 96.69           C
ATOM   1497  C   ARG A 192      63.969 -14.422 -44.281  1.00 96.69           C
ATOM   1498  CB  ARG A 192      62.656 -12.508 -43.375  1.00 96.69           C
ATOM   1499  O   ARG A 192      65.125 -14.125 -44.000  1.00 96.69           O
ATOM   1500  CG  ARG A 192      62.406 -11.930 -44.750  1.00 96.69           C
ATOM   1501  CD  ARG A 192      62.188 -10.422 -44.719  1.00 96.69           C
ATOM   1502  NE  ARG A 192      61.938  -9.875 -46.031  1.00 96.69           N
ATOM   1503  NH1 ARG A 192      59.938  -8.859 -45.469  1.00 96.69           N
ATOM   1504  NH2 ARG A 192      60.750  -8.695 -47.625  1.00 96.69           N
ATOM   1505  CZ  ARG A 192      60.875  -9.141 -46.375  1.00 96.69           C
ATOM   1506  N   LYS A 193      63.719 -15.094 -45.469  1.00 95.19           N
ATOM   1507  CA  LYS A 193      64.688 -15.531 -46.438  1.00 95.19           C
ATOM   1508  C   LYS A 193      64.875 -14.508 -47.562  1.00 95.19           C
ATOM   1509  CB  LYS A 193      64.312 -16.891 -47.031  1.00 95.19           C
ATOM   1510  O   LYS A 193      66.000 -14.336 -48.094  1.00 95.19           O
ATOM   1511  CG  LYS A 193      64.500 -18.047 -46.062  1.00 95.19           C
ATOM   1512  CD  LYS A 193      66.000 -18.266 -45.750  1.00 95.19           C
ATOM   1513  CE  LYS A 193      66.625 -19.250 -46.688  1.00 95.19           C
ATOM   1514  NZ  LYS A 193      68.000 -19.609 -46.250  1.00 95.19           N
ATOM   1515  N   THR A 194      63.781 -13.836 -47.938  1.00 94.62           N
ATOM   1516  CA  THR A 194      63.844 -12.797 -48.969  1.00 94.62           C
ATOM   1517  C   THR A 194      64.625 -11.570 -48.438  1.00 94.62           C
ATOM   1518  CB  THR A 194      62.438 -12.375 -49.438  1.00 94.62           C
ATOM   1519  O   THR A 194      64.312 -11.078 -47.344  1.00 94.62           O
ATOM   1520  CG2 THR A 194      62.531 -11.383 -50.594  1.00 94.62           C
ATOM   1521  OG1 THR A 194      61.719 -13.539 -49.844  1.00 94.62           O
ATOM   1522  N   PRO A 195      65.688 -11.070 -49.125  1.00 92.19           N
ATOM   1523  CA  PRO A 195      66.438  -9.898 -48.688  1.00 92.19           C
ATOM   1524  C   PRO A 195      65.562  -8.672 -48.469  1.00 92.19           C
ATOM   1525  CB  PRO A 195      67.438  -9.664 -49.812  1.00 92.19           C
ATOM   1526  O   PRO A 195      64.562  -8.469 -49.250  1.00 92.19           O
ATOM   1527  CG  PRO A 195      67.562 -10.992 -50.500  1.00 92.19           C
ATOM   1528  CD  PRO A 195      66.250 -11.719 -50.344  1.00 92.19           C
ATOM   1529  N   ASP A 196      65.750  -7.996 -47.281  1.00 91.44           N
ATOM   1530  CA  ASP A 196      65.000  -6.750 -47.031  1.00 91.44           C
ATOM   1531  C   ASP A 196      65.250  -5.770 -48.188  1.00 91.44           C
ATOM   1532  CB  ASP A 196      65.500  -6.113 -45.719  1.00 91.44           C
```

ATOM	1533	O	ASP	A	196	66.375	-5.629	-48.688	1.00	91.44	O
ATOM	1534	CG	ASP	A	196	65.188	-6.984	-44.500	1.00	91.44	C
ATOM	1535	OD1	ASP	A	196	64.062	-7.465	-44.375	1.00	91.44	O
ATOM	1536	OD2	ASP	A	196	66.125	-7.195	-43.688	1.00	91.44	O
ATOM	1537	N	SER	A	197	64.188	-5.219	-48.844	1.00	89.31	N
ATOM	1538	CA	SER	A	197	64.250	-4.293	-49.969	1.00	89.31	C
ATOM	1539	C	SER	A	197	64.688	-2.910	-49.500	1.00	89.31	C
ATOM	1540	CB	SER	A	197	62.938	-4.188	-50.688	1.00	89.31	C
ATOM	1541	O	SER	A	197	64.188	-2.393	-48.500	1.00	89.31	O
ATOM	1542	OG	SER	A	197	63.031	-3.248	-51.750	1.00	89.31	O
ATOM	1543	N	ASN	A	198	65.812	-2.234	-50.156	1.00	86.19	N
ATOM	1544	CA	ASN	A	198	66.188	-0.847	-49.938	1.00	86.19	C
ATOM	1545	C	ASN	A	198	65.500	0.102	-50.875	1.00	86.19	C
ATOM	1546	CB	ASN	A	198	67.688	-0.683	-50.062	1.00	86.19	C
ATOM	1547	O	ASN	A	198	65.688	1.312	-50.844	1.00	86.19	O
ATOM	1548	CG	ASN	A	198	68.500	-1.492	-49.031	1.00	86.19	C
ATOM	1549	ND2	ASN	A	198	69.688	-1.888	-49.375	1.00	86.19	N
ATOM	1550	OD1	ASN	A	198	68.000	-1.761	-47.938	1.00	86.19	O
ATOM	1551	N	ASP	A	199	64.562	-0.529	-51.750	1.00	88.12	N
ATOM	1552	CA	ASP	A	199	63.812	0.207	-52.781	1.00	88.12	C
ATOM	1553	C	ASP	A	199	62.500	0.769	-52.188	1.00	88.12	C
ATOM	1554	CB	ASP	A	199	63.500	-0.690	-53.969	1.00	88.12	C
ATOM	1555	O	ASP	A	199	61.656	0.015	-51.688	1.00	88.12	O
ATOM	1556	CG	ASP	A	199	62.875	0.070	-55.125	1.00	88.12	C
ATOM	1557	OD1	ASP	A	199	62.469	1.238	-54.938	1.00	88.12	O
ATOM	1558	OD2	ASP	A	199	62.781	-0.507	-56.250	1.00	88.12	O
ATOM	1559	N	SER	A	200	62.469	2.125	-52.125	1.00	88.94	N
ATOM	1560	CA	SER	A	200	61.281	2.775	-51.562	1.00	88.94	C
ATOM	1561	C	SER	A	200	60.031	2.398	-52.312	1.00	88.94	C
ATOM	1562	CB	SER	A	200	61.469	4.297	-51.562	1.00	88.94	C
ATOM	1563	O	SER	A	200	58.906	2.600	-51.844	1.00	88.94	O
ATOM	1564	OG	SER	A	200	61.688	4.770	-52.875	1.00	88.94	O
ATOM	1565	N	ASN	A	201	60.188	1.830	-53.656	1.00	90.75	N
ATOM	1566	CA	ASN	A	201	59.062	1.442	-54.500	1.00	90.75	C
ATOM	1567	C	ASN	A	201	58.594	0.022	-54.188	1.00	90.75	C
ATOM	1568	CB	ASN	A	201	59.406	1.572	-55.969	1.00	90.75	C
ATOM	1569	O	ASN	A	201	57.656	-0.477	-54.812	1.00	90.75	O
ATOM	1570	CG	ASN	A	201	59.531	3.016	-56.438	1.00	90.75	C
ATOM	1571	ND2	ASN	A	201	60.375	3.258	-57.438	1.00	90.75	N
ATOM	1572	OD1	ASN	A	201	58.875	3.906	-55.875	1.00	90.75	O
ATOM	1573	N	VAL	A	202	59.281	-0.699	-53.219	1.00	93.44	N
ATOM	1574	CA	VAL	A	202	58.875	-2.041	-52.781	1.00	93.44	C
ATOM	1575	C	VAL	A	202	58.531	-2.037	-51.312	1.00	93.44	C
ATOM	1576	CB	VAL	A	202	60.000	-3.070	-53.094	1.00	93.44	C
ATOM	1577	O	VAL	A	202	59.375	-1.676	-50.469	1.00	93.44	O
ATOM	1578	CG1	VAL	A	202	59.594	-4.465	-52.625	1.00	93.44	C
ATOM	1579	CG2	VAL	A	202	60.344	-3.076	-54.562	1.00	93.44	C
ATOM	1580	N	VAL	A	203	57.344	-2.295	-51.000	1.00	94.06	N
ATOM	1581	CA	VAL	A	203	56.875	-2.471	-49.625	1.00	94.06	C
ATOM	1582	C	VAL	A	203	56.531	-3.938	-49.375	1.00	94.06	C
ATOM	1583	CB	VAL	A	203	55.656	-1.575	-49.312	1.00	94.06	C
ATOM	1584	O	VAL	A	203	55.531	-4.441	-49.875	1.00	94.06	O
ATOM	1585	CG1	VAL	A	203	55.250	-1.688	-47.844	1.00	94.06	C
ATOM	1586	CG2	VAL	A	203	55.938	-0.122	-49.656	1.00	94.06	C
ATOM	1587	N	ASP	A	204	57.406	-4.676	-48.531	1.00	95.00	N
ATOM	1588	CA	ASP	A	204	57.188	-6.109	-48.375	1.00	95.00	C
ATOM	1589	C	ASP	A	204	57.531	-6.566	-46.938	1.00	95.00	C
ATOM	1590	CB	ASP	A	204	58.062	-6.887	-49.375	1.00	95.00	C
ATOM	1591	O	ASP	A	204	58.094	-7.648	-46.750	1.00	95.00	O
ATOM	1592	CG	ASP	A	204	59.531	-6.703	-49.156	1.00	95.00	C
ATOM	1593	OD1	ASP	A	204	59.938	-5.844	-48.344	1.00	95.00	O
ATOM	1594	OD2	ASP	A	204	60.344	-7.418	-49.812	1.00	95.00	O
ATOM	1595	N	ALA	A	205	57.312	-5.711	-45.969	1.00	94.06	N
ATOM	1596	CA	ALA	A	205	57.562	-6.078	-44.562	1.00	94.06	C

ATOM	1597	C	ALA	A	205	56.625	-7.191	-44.125	1.00	94.06	C
ATOM	1598	CB	ALA	A	205	57.438	-4.859	-43.656	1.00	94.06	C
ATOM	1599	O	ALA	A	205	55.438	-7.207	-44.469	1.00	94.06	O
ATOM	1600	N	ILE	A	206	57.219	-8.188	-43.406	1.00	95.06	N
ATOM	1601	CA	ILE	A	206	56.406	-9.219	-42.750	1.00	95.06	C
ATOM	1602	C	ILE	A	206	56.656	-9.219	-41.250	1.00	95.06	C
ATOM	1603	CB	ILE	A	206	56.688	-10.609	-43.344	1.00	95.06	C
ATOM	1604	O	ILE	A	206	57.750	-8.836	-40.812	1.00	95.06	O
ATOM	1605	CG1	ILE	A	206	58.188	-10.953	-43.219	1.00	95.06	C
ATOM	1606	CG2	ILE	A	206	56.250	-10.680	-44.812	1.00	95.06	C
ATOM	1607	CD1	ILE	A	206	58.531	-12.383	-43.625	1.00	95.06	C
ATOM	1608	N	GLN	A	207	55.656	-9.594	-40.562	1.00	94.94	N
ATOM	1609	CA	GLN	A	207	55.781	-9.672	-39.125	1.00	94.94	C
ATOM	1610	C	GLN	A	207	55.094	-10.930	-38.562	1.00	94.94	C
ATOM	1611	CB	GLN	A	207	55.156	-8.430	-38.438	1.00	94.94	C
ATOM	1612	O	GLN	A	207	54.156	-11.461	-39.188	1.00	94.94	O
ATOM	1613	CG	GLN	A	207	54.188	-7.680	-39.344	1.00	94.94	C
ATOM	1614	CD	GLN	A	207	53.500	-6.531	-38.625	1.00	94.94	C
ATOM	1615	NE2	GLN	A	207	52.875	-5.648	-39.375	1.00	94.94	N
ATOM	1616	OE1	GLN	A	207	53.562	-6.434	-37.375	1.00	94.94	O
ATOM	1617	N	VAL	A	208	55.656	-11.406	-37.500	1.00	95.62	N
ATOM	1618	CA	VAL	A	208	54.906	-12.352	-36.688	1.00	95.62	C
ATOM	1619	C	VAL	A	208	53.844	-11.602	-35.875	1.00	95.62	C
ATOM	1620	CB	VAL	A	208	55.844	-13.156	-35.750	1.00	95.62	C
ATOM	1621	O	VAL	A	208	54.188	-10.859	-34.969	1.00	95.62	O
ATOM	1622	CG1	VAL	A	208	55.031	-14.094	-34.875	1.00	95.62	C
ATOM	1623	CG2	VAL	A	208	56.875	-13.945	-36.562	1.00	95.62	C
ATOM	1624	N	LYS	A	209	52.594	-11.750	-36.219	1.00	93.50	N
ATOM	1625	CA	LYS	A	209	51.500	-11.062	-35.562	1.00	93.50	C
ATOM	1626	C	LYS	A	209	51.156	-11.711	-34.219	1.00	93.50	C
ATOM	1627	CB	LYS	A	209	50.250	-11.047	-36.438	1.00	93.50	C
ATOM	1628	O	LYS	A	209	51.062	-11.023	-33.188	1.00	93.50	O
ATOM	1629	CG	LYS	A	209	49.125	-10.172	-35.906	1.00	93.50	C
ATOM	1630	CD	LYS	A	209	47.969	-10.047	-36.938	1.00	93.50	C
ATOM	1631	CE	LYS	A	209	46.875	-9.117	-36.438	1.00	93.50	C
ATOM	1632	NZ	LYS	A	209	45.781	-8.961	-37.438	1.00	93.50	N
ATOM	1633	N	SER	A	210	51.062	-13.023	-34.188	1.00	94.00	N
ATOM	1634	CA	SER	A	210	50.750	-13.812	-33.000	1.00	94.00	C
ATOM	1635	C	SER	A	210	51.125	-15.281	-33.219	1.00	94.00	C
ATOM	1636	CB	SER	A	210	49.281	-13.680	-32.625	1.00	94.00	C
ATOM	1637	O	SER	A	210	51.406	-15.703	-34.344	1.00	94.00	O
ATOM	1638	OG	SER	A	210	48.469	-14.297	-33.594	1.00	94.00	O
ATOM	1639	N	TYR	A	211	51.281	-16.031	-32.156	1.00	93.19	N
ATOM	1640	CA	TYR	A	211	51.344	-17.484	-32.219	1.00	93.19	C
ATOM	1641	C	TYR	A	211	50.406	-18.109	-31.172	1.00	93.19	C
ATOM	1642	CB	TYR	A	211	52.750	-17.984	-31.984	1.00	93.19	C
ATOM	1643	O	TYR	A	211	50.062	-17.469	-30.156	1.00	93.19	O
ATOM	1644	CG	TYR	A	211	53.281	-17.719	-30.594	1.00	93.19	C
ATOM	1645	CD1	TYR	A	211	53.938	-16.531	-30.281	1.00	93.19	C
ATOM	1646	CD2	TYR	A	211	53.125	-18.672	-29.578	1.00	93.19	C
ATOM	1647	CE1	TYR	A	211	54.406	-16.297	-28.984	1.00	93.19	C
ATOM	1648	CE2	TYR	A	211	53.594	-18.453	-28.297	1.00	93.19	C
ATOM	1649	OH	TYR	A	211	54.719	-17.031	-26.734	1.00	93.19	O
ATOM	1650	CZ	TYR	A	211	54.250	-17.250	-28.016	1.00	93.19	C
ATOM	1651	N	ALA	A	212	49.812	-19.297	-31.484	1.00	91.38	N
ATOM	1652	CA	ALA	A	212	48.906	-20.016	-30.609	1.00	91.38	C
ATOM	1653	C	ALA	A	212	49.531	-21.328	-30.141	1.00	91.38	C
ATOM	1654	CB	ALA	A	212	47.562	-20.281	-31.297	1.00	91.38	C
ATOM	1655	O	ALA	A	212	50.125	-22.062	-30.938	1.00	91.38	O
ATOM	1656	N	GLU	A	213	49.500	-21.484	-28.844	1.00	90.56	N
ATOM	1657	CA	GLU	A	213	49.844	-22.781	-28.266	1.00	90.56	C
ATOM	1658	C	GLU	A	213	48.719	-23.781	-28.422	1.00	90.56	C
ATOM	1659	CB	GLU	A	213	50.219	-22.641	-26.797	1.00	90.56	C
ATOM	1660	O	GLU	A	213	47.594	-23.531	-27.922	1.00	90.56	O

```
ATOM   1661  CG  GLU A 213      51.469 -21.766 -26.562  1.00 90.56           C
ATOM   1662  CD  GLU A 213      51.750 -21.516 -25.078  1.00 90.56           C
ATOM   1663  OE1 GLU A 213      52.812 -20.984 -24.750  1.00 90.56           O
ATOM   1664  OE2 GLU A 213      50.875 -21.859 -24.250  1.00 90.56           O
ATOM   1665  N   VAL A 214      48.938 -24.906 -29.141  1.00 88.56           N
ATOM   1666  CA  VAL A 214      47.844 -25.797 -29.578  1.00 88.56           C
ATOM   1667  C   VAL A 214      47.969 -27.141 -28.859  1.00 88.56           C
ATOM   1668  CB  VAL A 214      47.875 -26.016 -31.109  1.00 88.56           C
ATOM   1669  O   VAL A 214      49.062 -27.719 -28.781  1.00 88.56           O
ATOM   1670  CG1 VAL A 214      46.781 -26.969 -31.547  1.00 88.56           C
ATOM   1671  CG2 VAL A 214      47.781 -24.672 -31.828  1.00 88.56           C
ATOM   1672  N   ILE A 215      46.938 -27.594 -28.219  1.00 85.19           N
ATOM   1673  CA  ILE A 215      46.781 -28.953 -27.703  1.00 85.19           C
ATOM   1674  C   ILE A 215      45.750 -29.703 -28.547  1.00 85.19           C
ATOM   1675  CB  ILE A 215      46.375 -28.969 -26.219  1.00 85.19           C
ATOM   1676  O   ILE A 215      44.594 -29.281 -28.641  1.00 85.19           O
ATOM   1677  CG1 ILE A 215      47.438 -28.266 -25.375  1.00 85.19           C
ATOM   1678  CG2 ILE A 215      46.156 -30.391 -25.734  1.00 85.19           C
ATOM   1679  CD1 ILE A 215      47.062 -28.094 -23.906  1.00 85.19           C
ATOM   1680  N   ASP A 216      46.312 -30.781 -29.281  1.00 76.75           N
ATOM   1681  CA  ASP A 216      45.344 -31.609 -30.016  1.00 76.75           C
ATOM   1682  C   ASP A 216      44.469 -32.438 -29.062  1.00 76.75           C
ATOM   1683  CB  ASP A 216      46.062 -32.531 -30.984  1.00 76.75           C
ATOM   1684  O   ASP A 216      45.000 -33.281 -28.344  1.00 76.75           O
ATOM   1685  CG  ASP A 216      46.750 -31.781 -32.125  1.00 76.75           C
ATOM   1686  OD1 ASP A 216      46.219 -30.750 -32.594  1.00 76.75           O
ATOM   1687  OD2 ASP A 216      47.844 -32.219 -32.562  1.00 76.75           O
ATOM   1688  N   ALA A 217      43.281 -31.953 -28.656  1.00 75.81           N
ATOM   1689  CA  ALA A 217      42.406 -32.688 -27.766  1.00 75.81           C
ATOM   1690  C   ALA A 217      40.938 -32.656 -28.281  1.00 75.81           C
ATOM   1691  CB  ALA A 217      42.469 -32.125 -26.359  1.00 75.81           C
ATOM   1692  O   ALA A 217      40.469 -31.578 -28.688  1.00 75.81           O
ATOM   1693  N   LYS A 218      40.406 -33.969 -28.484  1.00 77.25           N
ATOM   1694  CA  LYS A 218      38.969 -34.125 -28.812  1.00 77.25           C
ATOM   1695  C   LYS A 218      38.156 -34.250 -27.531  1.00 77.25           C
ATOM   1696  CB  LYS A 218      38.750 -35.344 -29.703  1.00 77.25           C
ATOM   1697  O   LYS A 218      38.250 -35.250 -26.828  1.00 77.25           O
ATOM   1698  CG  LYS A 218      39.500 -35.281 -31.031  1.00 77.25           C
ATOM   1699  CD  LYS A 218      39.312 -36.562 -31.828  1.00 77.25           C
ATOM   1700  CE  LYS A 218      40.094 -36.500 -33.125  1.00 77.25           C
ATOM   1701  NZ  LYS A 218      39.969 -37.781 -33.906  1.00 77.25           N
ATOM   1702  N   PHE A 219      37.531 -33.125 -27.156  1.00 80.94           N
ATOM   1703  CA  PHE A 219      36.719 -33.094 -25.938  1.00 80.94           C
ATOM   1704  C   PHE A 219      35.250 -33.344 -26.266  1.00 80.94           C
ATOM   1705  CB  PHE A 219      36.906 -31.766 -25.203  1.00 80.94           C
ATOM   1706  O   PHE A 219      34.688 -32.656 -27.125  1.00 80.94           O
ATOM   1707  CG  PHE A 219      38.281 -31.547 -24.656  1.00 80.94           C
ATOM   1708  CD1 PHE A 219      38.750 -32.312 -23.609  1.00 80.94           C
ATOM   1709  CD2 PHE A 219      39.125 -30.578 -25.203  1.00 80.94           C
ATOM   1710  CE1 PHE A 219      40.031 -32.125 -23.094  1.00 80.94           C
ATOM   1711  CE2 PHE A 219      40.406 -30.359 -24.703  1.00 80.94           C
ATOM   1712  CZ  PHE A 219      40.844 -31.141 -23.641  1.00 80.94           C
ATOM   1713  N   ARG A 220      34.719 -34.375 -25.703  1.00 84.69           N
ATOM   1714  CA  ARG A 220      33.312 -34.656 -25.891  1.00 84.69           C
ATOM   1715  C   ARG A 220      32.438 -33.781 -24.938  1.00 84.69           C
ATOM   1716  CB  ARG A 220      33.000 -36.125 -25.672  1.00 84.69           C
ATOM   1717  O   ARG A 220      31.281 -33.531 -25.219  1.00 84.69           O
ATOM   1718  CG  ARG A 220      33.219 -36.594 -24.234  1.00 84.69           C
ATOM   1719  CD  ARG A 220      32.969 -38.094 -24.078  1.00 84.69           C
ATOM   1720  NE  ARG A 220      33.062 -38.500 -22.688  1.00 84.69           N
ATOM   1721  NH1 ARG A 220      32.562 -40.719 -23.109  1.00 84.69           N
ATOM   1722  NH2 ARG A 220      33.000 -40.031 -20.969  1.00 84.69           N
ATOM   1723  CZ  ARG A 220      32.875 -39.750 -22.266  1.00 84.69           C
ATOM   1724  N   TYR A 221      32.969 -33.281 -23.797  1.00 89.00           N
```

```
ATOM   1725  CA  TYR A 221      32.250 -32.469 -22.828  1.00 89.00           C
ATOM   1726  C   TYR A 221      30.969 -33.125 -22.375  1.00 89.00           C
ATOM   1727  CB  TYR A 221      31.938 -31.078 -23.422  1.00 89.00           C
ATOM   1728  O   TYR A 221      29.875 -32.625 -22.609  1.00 89.00           O
ATOM   1729  CG  TYR A 221      33.156 -30.297 -23.828  1.00 89.00           C
ATOM   1730  CD1 TYR A 221      33.969 -29.672 -22.875  1.00 89.00           C
ATOM   1731  CD2 TYR A 221      33.531 -30.188 -25.172  1.00 89.00           C
ATOM   1732  CE1 TYR A 221      35.094 -28.953 -23.266  1.00 89.00           C
ATOM   1733  CE2 TYR A 221      34.656 -29.469 -25.562  1.00 89.00           C
ATOM   1734  OH  TYR A 221      36.531 -28.141 -24.969  1.00 89.00           O
ATOM   1735  CZ  TYR A 221      35.438 -28.859 -24.594  1.00 89.00           C
ATOM   1736  N   PRO A 222      31.188 -34.344 -21.625  1.00 91.12           N
ATOM   1737  CA  PRO A 222      30.000 -35.094 -21.203  1.00 91.12           C
ATOM   1738  C   PRO A 222      29.078 -34.250 -20.328  1.00 91.12           C
ATOM   1739  CB  PRO A 222      30.562 -36.250 -20.391  1.00 91.12           C
ATOM   1740  O   PRO A 222      29.547 -33.438 -19.516  1.00 91.12           O
ATOM   1741  CG  PRO A 222      31.906 -35.781 -19.938  1.00 91.12           C
ATOM   1742  CD  PRO A 222      32.438 -34.812 -20.953  1.00 91.12           C
ATOM   1743  N   LEU A 223      27.641 -34.281 -20.641  1.00 92.56           N
ATOM   1744  CA  LEU A 223      26.578 -33.656 -19.859  1.00 92.56           C
ATOM   1745  C   LEU A 223      26.656 -32.125 -19.953  1.00 92.56           C
ATOM   1746  CB  LEU A 223      26.656 -34.094 -18.391  1.00 92.56           C
ATOM   1747  O   LEU A 223      26.266 -31.422 -19.016  1.00 92.56           O
ATOM   1748  CG  LEU A 223      26.484 -35.594 -18.125  1.00 92.56           C
ATOM   1749  CD1 LEU A 223      26.688 -35.875 -16.641  1.00 92.56           C
ATOM   1750  CD2 LEU A 223      25.109 -36.062 -18.578  1.00 92.56           C
ATOM   1751  N   THR A 224      27.344 -31.641 -20.938  1.00 93.50           N
ATOM   1752  CA  THR A 224      27.406 -30.219 -21.250  1.00 93.50           C
ATOM   1753  C   THR A 224      26.844 -29.953 -22.641  1.00 93.50           C
ATOM   1754  CB  THR A 224      28.844 -29.672 -21.141  1.00 93.50           C
ATOM   1755  O   THR A 224      27.359 -30.469 -23.625  1.00 93.50           O
ATOM   1756  CG2 THR A 224      28.875 -28.172 -21.375  1.00 93.50           C
ATOM   1757  OG1 THR A 224      29.359 -29.953 -19.828  1.00 93.50           O
ATOM   1758  N   GLY A 225      25.703 -29.219 -22.734  1.00 92.50           N
ATOM   1759  CA  GLY A 225      25.219 -28.797 -24.031  1.00 92.50           C
ATOM   1760  C   GLY A 225      26.141 -27.781 -24.719  1.00 92.50           C
ATOM   1761  O   GLY A 225      26.578 -26.812 -24.094  1.00 92.50           O
ATOM   1762  N   LEU A 226      26.422 -28.047 -26.016  1.00 92.56           N
ATOM   1763  CA  LEU A 226      27.391 -27.219 -26.734  1.00 92.56           C
ATOM   1764  C   LEU A 226      26.781 -26.672 -28.016  1.00 92.56           C
ATOM   1765  CB  LEU A 226      28.641 -28.031 -27.062  1.00 92.56           C
ATOM   1766  O   LEU A 226      25.984 -27.344 -28.688  1.00 92.56           O
ATOM   1767  CG  LEU A 226      29.391 -28.625 -25.875  1.00 92.56           C
ATOM   1768  CD1 LEU A 226      30.453 -29.609 -26.344  1.00 92.56           C
ATOM   1769  CD2 LEU A 226      30.016 -27.516 -25.031  1.00 92.56           C
ATOM   1770  N   LEU A 227      27.062 -25.375 -28.297  1.00 93.62           N
ATOM   1771  CA  LEU A 227      26.750 -24.766 -29.578  1.00 93.62           C
ATOM   1772  C   LEU A 227      28.016 -24.266 -30.266  1.00 93.62           C
ATOM   1773  CB  LEU A 227      25.766 -23.609 -29.406  1.00 93.62           C
ATOM   1774  O   LEU A 227      28.734 -23.422 -29.703  1.00 93.62           O
ATOM   1775  CG  LEU A 227      25.453 -22.797 -30.672  1.00 93.62           C
ATOM   1776  CD1 LEU A 227      24.688 -23.641 -31.672  1.00 93.62           C
ATOM   1777  CD2 LEU A 227      24.641 -21.547 -30.312  1.00 93.62           C
ATOM   1778  N   PHE A 228      28.266 -24.828 -31.391  1.00 91.62           N
ATOM   1779  CA  PHE A 228      29.375 -24.391 -32.250  1.00 91.62           C
ATOM   1780  C   PHE A 228      28.875 -23.531 -33.375  1.00 91.62           C
ATOM   1781  CB  PHE A 228      30.125 -25.609 -32.781  1.00 91.62           C
ATOM   1782  O   PHE A 228      27.969 -23.922 -34.125  1.00 91.62           O
ATOM   1783  CG  PHE A 228      31.188 -25.250 -33.812  1.00 91.62           C
ATOM   1784  CD1 PHE A 228      30.984 -25.484 -35.156  1.00 91.62           C
ATOM   1785  CD2 PHE A 228      32.375 -24.688 -33.406  1.00 91.62           C
ATOM   1786  CE1 PHE A 228      31.969 -25.156 -36.094  1.00 91.62           C
ATOM   1787  CE2 PHE A 228      33.375 -24.359 -34.312  1.00 91.62           C
ATOM   1788  CZ  PHE A 228      33.156 -24.594 -35.656  1.00 91.62           C
```

```
ATOM   1789  N   VAL A 229      29.453 -22.328 -33.469  1.00 92.62           N
ATOM   1790  CA  VAL A 229      29.078 -21.391 -34.531  1.00 92.62           C
ATOM   1791  C   VAL A 229      30.312 -20.906 -35.281  1.00 92.62           C
ATOM   1792  CB  VAL A 229      28.281 -20.203 -34.000  1.00 92.62           C
ATOM   1793  O   VAL A 229      31.312 -20.547 -34.656  1.00 92.62           O
ATOM   1794  CG1 VAL A 229      27.859 -19.250 -35.125  1.00 92.62           C
ATOM   1795  CG2 VAL A 229      27.047 -20.688 -33.219  1.00 92.62           C
ATOM   1796  N   GLU A 230      30.266 -20.953 -36.625  1.00 89.94           N
ATOM   1797  CA  GLU A 230      31.344 -20.484 -37.500  1.00 89.94           C
ATOM   1798  C   GLU A 230      30.797 -19.547 -38.594  1.00 89.94           C
ATOM   1799  CB  GLU A 230      32.062 -21.672 -38.156  1.00 89.94           C
ATOM   1800  O   GLU A 230      29.781 -19.828 -39.219  1.00 89.94           O
ATOM   1801  CG  GLU A 230      33.156 -21.266 -39.125  1.00 89.94           C
ATOM   1802  CD  GLU A 230      33.812 -22.453 -39.812  1.00 89.94           C
ATOM   1803  OE1 GLU A 230      34.156 -22.328 -41.031  1.00 89.94           O
ATOM   1804  OE2 GLU A 230      33.969 -23.516 -39.188  1.00 89.94           O
ATOM   1805  N   PHE A 231      31.359 -18.344 -38.688  1.00 90.44           N
ATOM   1806  CA  PHE A 231      30.984 -17.391 -39.719  1.00 90.44           C
ATOM   1807  C   PHE A 231      32.188 -16.531 -40.125  1.00 90.44           C
ATOM   1808  CB  PHE A 231      29.828 -16.516 -39.250  1.00 90.44           C
ATOM   1809  O   PHE A 231      33.156 -16.453 -39.375  1.00 90.44           O
ATOM   1810  CG  PHE A 231      30.094 -15.789 -37.969  1.00 90.44           C
ATOM   1811  CD1 PHE A 231      29.781 -16.375 -36.750  1.00 90.44           C
ATOM   1812  CD2 PHE A 231      30.656 -14.523 -37.969  1.00 90.44           C
ATOM   1813  CE1 PHE A 231      30.031 -15.703 -35.531  1.00 90.44           C
ATOM   1814  CE2 PHE A 231      30.906 -13.844 -36.781  1.00 90.44           C
ATOM   1815  CZ  PHE A 231      30.594 -14.438 -35.562  1.00 90.44           C
ATOM   1816  N   ASP A 232      32.094 -15.844 -41.312  1.00 87.38           N
ATOM   1817  CA  ASP A 232      33.125 -14.953 -41.844  1.00 87.38           C
ATOM   1818  C   ASP A 232      33.219 -13.672 -41.000  1.00 87.38           C
ATOM   1819  CB  ASP A 232      32.844 -14.625 -43.312  1.00 87.38           C
ATOM   1820  O   ASP A 232      32.188 -13.047 -40.719  1.00 87.38           O
ATOM   1821  CG  ASP A 232      34.031 -13.984 -44.000  1.00 87.38           C
ATOM   1822  OD1 ASP A 232      34.781 -13.234 -43.344  1.00 87.38           O
ATOM   1823  OD2 ASP A 232      34.219 -14.227 -45.219  1.00 87.38           O
ATOM   1824  N   SER A 233      34.406 -13.312 -40.562  1.00 80.62           N
ATOM   1825  CA  SER A 233      34.625 -12.109 -39.750  1.00 80.62           C
ATOM   1826  C   SER A 233      34.125 -10.867 -40.500  1.00 80.62           C
ATOM   1827  CB  SER A 233      36.094 -11.953 -39.375  1.00 80.62           C
ATOM   1828  O   SER A 233      33.812  -9.852 -39.844  1.00 80.62           O
ATOM   1829  OG  SER A 233      36.875 -11.766 -40.531  1.00 80.62           O
ATOM   1830  N   LYS A 234      34.031 -10.867 -41.906  1.00 81.31           N
ATOM   1831  CA  LYS A 234      33.594  -9.727 -42.719  1.00 81.31           C
ATOM   1832  C   LYS A 234      32.156  -9.359 -42.375  1.00 81.31           C
ATOM   1833  CB  LYS A 234      33.719 -10.031 -44.188  1.00 81.31           C
ATOM   1834  O   LYS A 234      31.734  -8.219 -42.594  1.00 81.31           O
ATOM   1835  CG  LYS A 234      33.531  -8.812 -45.094  1.00 81.31           C
ATOM   1836  CD  LYS A 234      33.719  -9.172 -46.562  1.00 81.31           C
ATOM   1837  CE  LYS A 234      33.469  -7.977 -47.469  1.00 81.31           C
ATOM   1838  NZ  LYS A 234      33.625  -8.328 -48.906  1.00 81.31           N
ATOM   1839  N   MET A 235      31.359 -10.359 -41.844  1.00 84.50           N
ATOM   1840  CA  MET A 235      29.938 -10.141 -41.562  1.00 84.50           C
ATOM   1841  C   MET A 235      29.766  -9.164 -40.406  1.00 84.50           C
ATOM   1842  CB  MET A 235      29.250 -11.469 -41.219  1.00 84.50           C
ATOM   1843  O   MET A 235      28.781  -8.414 -40.375  1.00 84.50           O
ATOM   1844  CG  MET A 235      29.125 -12.406 -42.438  1.00 84.50           C
ATOM   1845  SD  MET A 235      28.328 -13.992 -42.000  1.00 84.50           S
ATOM   1846  CE  MET A 235      28.516 -14.891 -43.562  1.00 84.50           C
ATOM   1847  N   PHE A 236      30.703  -9.242 -39.406  1.00 84.94           N
ATOM   1848  CA  PHE A 236      30.688  -8.367 -38.250  1.00 84.94           C
ATOM   1849  C   PHE A 236      32.062  -7.773 -37.969  1.00 84.94           C
ATOM   1850  CB  PHE A 236      30.188  -9.133 -37.000  1.00 84.94           C
ATOM   1851  O   PHE A 236      32.781  -8.250 -37.094  1.00 84.94           O
ATOM   1852  CG  PHE A 236      28.844  -9.781 -37.188  1.00 84.94           C
```

```
ATOM   1853  CD1 PHE A 236      27.672  -9.023 -37.094  1.00 84.94           C
ATOM   1854  CD2 PHE A 236      28.734 -11.133 -37.469  1.00 84.94           C
ATOM   1855  CE1 PHE A 236      26.422  -9.617 -37.250  1.00 84.94           C
ATOM   1856  CE2 PHE A 236      27.500 -11.734 -37.625  1.00 84.94           C
ATOM   1857  CZ  PHE A 236      26.344 -10.977 -37.531  1.00 84.94           C
ATOM   1858  N   PRO A 237      32.531  -6.629 -38.750  1.00 76.69           N
ATOM   1859  CA  PRO A 237      33.906  -6.094 -38.750  1.00 76.69           C
ATOM   1860  C   PRO A 237      34.281  -5.434 -37.438  1.00 76.69           C
ATOM   1861  CB  PRO A 237      33.875  -5.066 -39.875  1.00 76.69           C
ATOM   1862  O   PRO A 237      35.438  -5.461 -37.031  1.00 76.69           O
ATOM   1863  CG  PRO A 237      32.406  -4.848 -40.156  1.00 76.69           C
ATOM   1864  CD  PRO A 237      31.641  -6.012 -39.625  1.00 76.69           C
ATOM   1865  N   ASN A 238      33.375  -4.914 -36.625  1.00 80.19           N
ATOM   1866  CA  ASN A 238      33.719  -4.035 -35.500  1.00 80.19           C
ATOM   1867  C   ASN A 238      33.469  -4.711 -34.156  1.00 80.19           C
ATOM   1868  CB  ASN A 238      32.938  -2.723 -35.594  1.00 80.19           C
ATOM   1869  O   ASN A 238      34.000  -4.285 -33.156  1.00 80.19           O
ATOM   1870  CG  ASN A 238      33.281  -1.922 -36.844  1.00 80.19           C
ATOM   1871  ND2 ASN A 238      32.312  -1.150 -37.344  1.00 80.19           N
ATOM   1872  OD1 ASN A 238      34.406  -1.995 -37.344  1.00 80.19           O
ATOM   1873  N   GLN A 239      32.625  -5.785 -34.125  1.00 84.19           N
ATOM   1874  CA  GLN A 239      32.312  -6.426 -32.875  1.00 84.19           C
ATOM   1875  C   GLN A 239      31.625  -7.773 -33.094  1.00 84.19           C
ATOM   1876  CB  GLN A 239      31.422  -5.516 -32.031  1.00 84.19           C
ATOM   1877  O   GLN A 239      30.984  -7.984 -34.094  1.00 84.19           O
ATOM   1878  CG  GLN A 239      30.047  -5.258 -32.625  1.00 84.19           C
ATOM   1879  CD  GLN A 239      29.219  -4.289 -31.812  1.00 84.19           C
ATOM   1880  NE2 GLN A 239      28.234  -3.652 -32.469  1.00 84.19           N
ATOM   1881  OE1 GLN A 239      29.438  -4.109 -30.609  1.00 84.19           O
ATOM   1882  N   LEU A 240      32.000  -8.695 -32.219  1.00 86.88           N
ATOM   1883  CA  LEU A 240      31.297  -9.977 -32.250  1.00 86.88           C
ATOM   1884  C   LEU A 240      29.812  -9.781 -31.938  1.00 86.88           C
ATOM   1885  CB  LEU A 240      31.906 -10.938 -31.219  1.00 86.88           C
ATOM   1886  O   LEU A 240      29.453  -9.039 -31.031  1.00 86.88           O
ATOM   1887  CG  LEU A 240      33.281 -11.539 -31.594  1.00 86.88           C
ATOM   1888  CD1 LEU A 240      33.844 -12.289 -30.406  1.00 86.88           C
ATOM   1889  CD2 LEU A 240      33.125 -12.461 -32.812  1.00 86.88           C
ATOM   1890  N   PRO A 241      28.969 -10.289 -32.844  1.00 91.38           N
ATOM   1891  CA  PRO A 241      27.531 -10.172 -32.594  1.00 91.38           C
ATOM   1892  C   PRO A 241      27.078 -10.938 -31.344  1.00 91.38           C
ATOM   1893  CB  PRO A 241      26.906 -10.781 -33.844  1.00 91.38           C
ATOM   1894  O   PRO A 241      27.750 -11.867 -30.906  1.00 91.38           O
ATOM   1895  CG  PRO A 241      27.875 -11.820 -34.281  1.00 91.38           C
ATOM   1896  CD  PRO A 241      29.266 -11.328 -34.031  1.00 91.38           C
ATOM   1897  N   THR A 242      26.047 -10.445 -30.672  1.00 91.38           N
ATOM   1898  CA  THR A 242      25.391 -11.242 -29.641  1.00 91.38           C
ATOM   1899  C   THR A 242      24.766 -12.500 -30.234  1.00 91.38           C
ATOM   1900  CB  THR A 242      24.312 -10.430 -28.906  1.00 91.38           C
ATOM   1901  O   THR A 242      24.000 -12.422 -31.203  1.00 91.38           O
ATOM   1902  CG2 THR A 242      23.688 -11.242 -27.766  1.00 91.38           C
ATOM   1903  OG1 THR A 242      24.906  -9.242 -28.359  1.00 91.38           O
ATOM   1904  N   ILE A 243      25.266 -13.656 -29.750  1.00 92.50           N
ATOM   1905  CA  ILE A 243      24.750 -14.945 -30.219  1.00 92.50           C
ATOM   1906  C   ILE A 243      23.656 -15.430 -29.266  1.00 92.50           C
ATOM   1907  CB  ILE A 243      25.875 -15.992 -30.328  1.00 92.50           C
ATOM   1908  O   ILE A 243      23.906 -15.641 -28.078  1.00 92.50           O
ATOM   1909  CG1 ILE A 243      26.969 -15.523 -31.297  1.00 92.50           C
ATOM   1910  CG2 ILE A 243      25.312 -17.359 -30.750  1.00 92.50           C
ATOM   1911  CD1 ILE A 243      28.188 -16.422 -31.359  1.00 92.50           C
ATOM   1912  N   SER A 244      22.469 -15.555 -29.781  1.00 92.19           N
ATOM   1913  CA  SER A 244      21.328 -16.031 -28.984  1.00 92.19           C
ATOM   1914  C   SER A 244      20.656 -17.219 -29.656  1.00 92.19           C
ATOM   1915  CB  SER A 244      20.328 -14.906 -28.766  1.00 92.19           C
ATOM   1916  O   SER A 244      20.625 -17.328 -30.891  1.00 92.19           O
```

```
ATOM   1917  OG  SER A 244      20.953 -13.734 -28.281  1.00 92.19           O
ATOM   1918  N   ILE A 245      20.156 -18.156 -28.844  1.00 91.69           N
ATOM   1919  CA  ILE A 245      19.469 -19.344 -29.328  1.00 91.69           C
ATOM   1920  C   ILE A 245      18.109 -19.484 -28.641  1.00 91.69           C
ATOM   1921  CB  ILE A 245      20.328 -20.609 -29.078  1.00 91.69           C
ATOM   1922  O   ILE A 245      18.000 -19.297 -27.422  1.00 91.69           O
ATOM   1923  CG1 ILE A 245      19.656 -21.844 -29.719  1.00 91.69           C
ATOM   1924  CG2 ILE A 245      20.531 -20.844 -27.578  1.00 91.69           C
ATOM   1925  CD1 ILE A 245      20.547 -23.062 -29.781  1.00 91.69           C
ATOM   1926  N   ARG A 246      17.125 -19.656 -29.469  1.00 90.25           N
ATOM   1927  CA  ARG A 246      15.781 -20.000 -29.000  1.00 90.25           C
ATOM   1928  C   ARG A 246      15.523 -21.500 -29.125  1.00 90.25           C
ATOM   1929  CB  ARG A 246      14.719 -19.219 -29.766  1.00 90.25           C
ATOM   1930  O   ARG A 246      15.414 -22.031 -30.234  1.00 90.25           O
ATOM   1931  CG  ARG A 246      13.305 -19.438 -29.266  1.00 90.25           C
ATOM   1932  CD  ARG A 246      12.281 -18.688 -30.109  1.00 90.25           C
ATOM   1933  NE  ARG A 246      10.914 -18.969 -29.688  1.00 90.25           N
ATOM   1934  NH1 ARG A 246       9.922 -17.609 -31.250  1.00 90.25           N
ATOM   1935  NH2 ARG A 246       8.633 -18.781 -29.766  1.00 90.25           N
ATOM   1936  CZ  ARG A 246       9.828 -18.453 -30.234  1.00 90.25           C
ATOM   1937  N   LYS A 247      15.398 -22.203 -27.938  1.00 90.38           N
ATOM   1938  CA  LYS A 247      15.297 -23.656 -27.984  1.00 90.38           C
ATOM   1939  C   LYS A 247      14.633 -24.203 -26.719  1.00 90.38           C
ATOM   1940  CB  LYS A 247      16.688 -24.297 -28.156  1.00 90.38           C
ATOM   1941  O   LYS A 247      14.570 -23.516 -25.703  1.00 90.38           O
ATOM   1942  CG  LYS A 247      16.672 -25.812 -28.266  1.00 90.38           C
ATOM   1943  CD  LYS A 247      18.062 -26.375 -28.500  1.00 90.38           C
ATOM   1944  CE  LYS A 247      18.031 -27.875 -28.719  1.00 90.38           C
ATOM   1945  NZ  LYS A 247      17.125 -28.250 -29.844  1.00 90.38           N
ATOM   1946  N   ARG A 248      14.195 -25.453 -26.922  1.00 91.31           N
ATOM   1947  CA  ARG A 248      13.828 -26.250 -25.750  1.00 91.31           C
ATOM   1948  C   ARG A 248      15.062 -26.781 -25.047  1.00 91.31           C
ATOM   1949  CB  ARG A 248      12.914 -27.406 -26.172  1.00 91.31           C
ATOM   1950  O   ARG A 248      15.930 -27.391 -25.672  1.00 91.31           O
ATOM   1951  CG  ARG A 248      11.570 -26.969 -26.734  1.00 91.31           C
ATOM   1952  CD  ARG A 248      10.742 -28.141 -27.203  1.00 91.31           C
ATOM   1953  NE  ARG A 248       9.469 -27.719 -27.781  1.00 91.31           N
ATOM   1954  NH1 ARG A 248       8.766 -29.844 -28.344  1.00 91.31           N
ATOM   1955  NH2 ARG A 248       7.438 -28.031 -28.812  1.00 91.31           N
ATOM   1956  CZ  ARG A 248       8.562 -28.531 -28.312  1.00 91.31           C
ATOM   1957  N   TRP A 249      15.078 -26.656 -23.766  1.00 92.06           N
ATOM   1958  CA  TRP A 249      16.359 -26.875 -23.078  1.00 92.06           C
ATOM   1959  C   TRP A 249      16.344 -28.203 -22.344  1.00 92.06           C
ATOM   1960  CB  TRP A 249      16.656 -25.734 -22.109  1.00 92.06           C
ATOM   1961  O   TRP A 249      16.375 -29.281 -22.969  1.00 92.06           O
ATOM   1962  CG  TRP A 249      16.953 -24.422 -22.781  1.00 92.06           C
ATOM   1963  CD1 TRP A 249      16.094 -23.375 -22.938  1.00 92.06           C
ATOM   1964  CD2 TRP A 249      18.188 -24.031 -23.391  1.00 92.06           C
ATOM   1965  CE2 TRP A 249      18.000 -22.719 -23.891  1.00 92.06           C
ATOM   1966  CE3 TRP A 249      19.422 -24.656 -23.562  1.00 92.06           C
ATOM   1967  NE1 TRP A 249      16.719 -22.344 -23.594  1.00 92.06           N
ATOM   1968  CH2 TRP A 249      20.219 -22.672 -24.703  1.00 92.06           C
ATOM   1969  CZ2 TRP A 249      19.016 -22.031 -24.547  1.00 92.06           C
ATOM   1970  CZ3 TRP A 249      20.438 -23.969 -24.219  1.00 92.06           C
ATOM   1971  N   LYS A 250      16.250 -28.281 -21.094  1.00 92.62           N
ATOM   1972  CA  LYS A 250      16.594 -29.438 -20.266  1.00 92.62           C
ATOM   1973  C   LYS A 250      15.523 -30.531 -20.391  1.00 92.62           C
ATOM   1974  CB  LYS A 250      16.750 -29.031 -18.812  1.00 92.62           C
ATOM   1975  O   LYS A 250      14.328 -30.234 -20.438  1.00 92.62           O
ATOM   1976  CG  LYS A 250      18.078 -28.344 -18.500  1.00 92.62           C
ATOM   1977  CD  LYS A 250      18.312 -28.250 -17.000  1.00 92.62           C
ATOM   1978  CE  LYS A 250      19.172 -29.391 -16.484  1.00 92.62           C
ATOM   1979  NZ  LYS A 250      19.828 -29.062 -15.180  1.00 92.62           N
ATOM   1980  N   ILE A 251      16.000 -31.766 -20.484  1.00 94.06           N
```

```
ATOM   1981  CA  ILE A 251      15.227 -33.000 -20.312  1.00 94.06           C
ATOM   1982  C   ILE A 251      15.266 -33.438 -18.844  1.00 94.06           C
ATOM   1983  CB  ILE A 251      15.758 -34.125 -21.219  1.00 94.06           C
ATOM   1984  O   ILE A 251      16.344 -33.562 -18.250  1.00 94.06           O
ATOM   1985  CG1 ILE A 251      15.664 -33.719 -22.688  1.00 94.06           C
ATOM   1986  CG2 ILE A 251      15.000 -35.438 -20.953  1.00 94.06           C
ATOM   1987  CD1 ILE A 251      16.312 -34.719 -23.641  1.00 94.06           C
ATOM   1988  N   VAL A 252      14.016 -33.562 -18.266  1.00 94.44           N
ATOM   1989  CA  VAL A 252      13.898 -33.844 -16.844  1.00 94.44           C
ATOM   1990  C   VAL A 252      13.000 -35.062 -16.609  1.00 94.44           C
ATOM   1991  CB  VAL A 252      13.344 -32.625 -16.062  1.00 94.44           C
ATOM   1992  O   VAL A 252      12.422 -35.594 -17.562  1.00 94.44           O
ATOM   1993  CG1 VAL A 252      14.266 -31.406 -16.250  1.00 94.44           C
ATOM   1994  CG2 VAL A 252      11.930 -32.281 -16.531  1.00 94.44           C
ATOM   1995  N   ASN A 253      12.961 -35.500 -15.367  1.00 94.56           N
ATOM   1996  CA  ASN A 253      12.062 -36.594 -15.000  1.00 94.56           C
ATOM   1997  C   ASN A 253      10.609 -36.125 -14.953  1.00 94.56           C
ATOM   1998  CB  ASN A 253      12.469 -37.188 -13.648  1.00 94.56           C
ATOM   1999  O   ASN A 253      10.289 -35.125 -14.289  1.00 94.56           O
ATOM   2000  CG  ASN A 253      13.758 -37.969 -13.727  1.00 94.56           C
ATOM   2001  ND2 ASN A 253      14.562 -37.906 -12.664  1.00 94.56           N
ATOM   2002  OD1 ASN A 253      14.039 -38.656 -14.727  1.00 94.56           O
ATOM   2003  N   VAL A 254       9.797 -36.812 -15.758  1.00 96.31           N
ATOM   2004  CA  VAL A 254       8.359 -36.562 -15.734  1.00 96.31           C
ATOM   2005  C   VAL A 254       7.609 -37.844 -15.492  1.00 96.31           C
ATOM   2006  CB  VAL A 254       7.879 -35.906 -17.047  1.00 96.31           C
ATOM   2007  O   VAL A 254       8.141 -38.938 -15.742  1.00 96.31           O
ATOM   2008  CG1 VAL A 254       8.578 -34.562 -17.266  1.00 96.31           C
ATOM   2009  CG2 VAL A 254       8.125 -36.812 -18.234  1.00 96.31           C
ATOM   2010  N   PRO A 255       6.363 -37.812 -14.953  1.00 97.62           N
ATOM   2011  CA  PRO A 255       5.582 -39.031 -14.758  1.00 97.62           C
ATOM   2012  C   PRO A 255       5.426 -39.844 -16.047  1.00 97.62           C
ATOM   2013  CB  PRO A 255       4.227 -38.531 -14.273  1.00 97.62           C
ATOM   2014  O   PRO A 255       5.273 -39.281 -17.125  1.00 97.62           O
ATOM   2015  CG  PRO A 255       4.531 -37.219 -13.625  1.00 97.62           C
ATOM   2016  CD  PRO A 255       5.645 -36.562 -14.383  1.00 97.62           C
ATOM   2017  N   SER A 256       5.445 -41.125 -15.914  1.00 96.56           N
ATOM   2018  CA  SER A 256       5.402 -42.031 -17.062  1.00 96.56           C
ATOM   2019  C   SER A 256       4.082 -41.875 -17.812  1.00 96.56           C
ATOM   2020  CB  SER A 256       5.602 -43.469 -16.625  1.00 96.56           C
ATOM   2021  O   SER A 256       4.012 -42.188 -19.016  1.00 96.56           O
ATOM   2022  OG  SER A 256       4.633 -43.844 -15.656  1.00 96.56           O
ATOM   2023  N   ASN A 257       2.992 -41.438 -17.172  1.00 96.69           N
ATOM   2024  CA  ASN A 257       1.684 -41.312 -17.797  1.00 96.69           C
ATOM   2025  C   ASN A 257       1.443 -39.906 -18.312  1.00 96.69           C
ATOM   2026  CB  ASN A 257       0.576 -41.719 -16.828  1.00 96.69           C
ATOM   2027  O   ASN A 257       0.332 -39.562 -18.734  1.00 96.69           O
ATOM   2028  CG  ASN A 257       0.435 -40.750 -15.664  1.00 96.69           C
ATOM   2029  ND2 ASN A 257      -0.731 -40.750 -15.031  1.00 96.69           N
ATOM   2030  OD1 ASN A 257       1.367 -40.000 -15.344  1.00 96.69           O
ATOM   2031  N   TYR A 258       2.408 -38.969 -18.219  1.00 97.44           N
ATOM   2032  CA  TYR A 258       2.314 -37.562 -18.594  1.00 97.44           C
ATOM   2033  C   TYR A 258       2.932 -37.312 -19.969  1.00 97.44           C
ATOM   2034  CB  TYR A 258       3.006 -36.688 -17.547  1.00 97.44           C
ATOM   2035  O   TYR A 258       4.059 -37.750 -20.234  1.00 97.44           O
ATOM   2036  CG  TYR A 258       3.092 -35.219 -17.938  1.00 97.44           C
ATOM   2037  CD1 TYR A 258       4.301 -34.531 -17.875  1.00 97.44           C
ATOM   2038  CD2 TYR A 258       1.965 -34.531 -18.359  1.00 97.44           C
ATOM   2039  CE1 TYR A 258       4.387 -33.219 -18.219  1.00 97.44           C
ATOM   2040  CE2 TYR A 258       2.037 -33.188 -18.719  1.00 97.44           C
ATOM   2041  OH  TYR A 258       3.328 -31.219 -19.000  1.00 97.44           O
ATOM   2042  CZ  TYR A 258       3.250 -32.531 -18.656  1.00 97.44           C
ATOM   2043  N   ASP A 259       2.145 -36.656 -20.859  1.00 95.94           N
ATOM   2044  CA  ASP A 259       2.639 -36.156 -22.125  1.00 95.94           C
```

ATOM	2045	C	ASP	A	259	2.844	-34.625	-22.078	1.00	95.94	C
ATOM	2046	CB	ASP	A	259	1.672	-36.500	-23.266	1.00	95.94	C
ATOM	2047	O	ASP	A	259	1.879	-33.875	-22.125	1.00	95.94	O
ATOM	2048	CG	ASP	A	259	2.170	-36.094	-24.625	1.00	95.94	C
ATOM	2049	OD1	ASP	A	259	3.166	-35.344	-24.703	1.00	95.94	O
ATOM	2050	OD2	ASP	A	259	1.560	-36.500	-25.641	1.00	95.94	O
ATOM	2051	N	PRO	A	260	4.148	-34.219	-22.109	1.00	94.62	N
ATOM	2052	CA	PRO	A	260	4.461	-32.781	-21.891	1.00	94.62	C
ATOM	2053	C	PRO	A	260	4.051	-31.906	-23.062	1.00	94.62	C
ATOM	2054	CB	PRO	A	260	5.984	-32.781	-21.703	1.00	94.62	C
ATOM	2055	O	PRO	A	260	3.869	-30.703	-22.891	1.00	94.62	O
ATOM	2056	CG	PRO	A	260	6.449	-34.031	-22.391	1.00	94.62	C
ATOM	2057	CD	PRO	A	260	5.375	-35.094	-22.266	1.00	94.62	C
ATOM	2058	N	GLU	A	261	3.904	-32.531	-24.281	1.00	92.81	N
ATOM	2059	CA	GLU	A	261	3.531	-31.766	-25.453	1.00	92.81	C
ATOM	2060	C	GLU	A	261	2.039	-31.453	-25.453	1.00	92.81	C
ATOM	2061	CB	GLU	A	261	3.912	-32.531	-26.734	1.00	92.81	C
ATOM	2062	O	GLU	A	261	1.647	-30.297	-25.625	1.00	92.81	O
ATOM	2063	CG	GLU	A	261	5.410	-32.688	-26.938	1.00	92.81	C
ATOM	2064	CD	GLU	A	261	6.117	-31.359	-27.219	1.00	92.81	C
ATOM	2065	OE1	GLU	A	261	7.336	-31.250	-26.938	1.00	92.81	O
ATOM	2066	OE2	GLU	A	261	5.449	-30.438	-27.734	1.00	92.81	O
ATOM	2067	N	SER	A	262	1.283	-32.500	-25.297	1.00	94.06	N
ATOM	2068	CA	SER	A	262	-0.164	-32.312	-25.312	1.00	94.06	C
ATOM	2069	C	SER	A	262	-0.682	-31.922	-23.922	1.00	94.06	C
ATOM	2070	CB	SER	A	262	-0.866	-33.562	-25.812	1.00	94.06	C
ATOM	2071	O	SER	A	262	-1.846	-31.547	-23.781	1.00	94.06	O
ATOM	2072	OG	SER	A	262	-0.603	-34.656	-24.938	1.00	94.06	O
ATOM	2073	N	ARG	A	263	0.159	-32.156	-22.859	1.00	94.81	N
ATOM	2074	CA	ARG	A	263	-0.147	-31.844	-21.469	1.00	94.81	C
ATOM	2075	C	ARG	A	263	-1.305	-32.688	-20.938	1.00	94.81	C
ATOM	2076	CB	ARG	A	263	-0.479	-30.344	-21.328	1.00	94.81	C
ATOM	2077	O	ARG	A	263	-2.209	-32.156	-20.281	1.00	94.81	O
ATOM	2078	CG	ARG	A	263	0.621	-29.422	-21.828	1.00	94.81	C
ATOM	2079	CD	ARG	A	263	0.316	-27.969	-21.500	1.00	94.81	C
ATOM	2080	NE	ARG	A	263	1.435	-27.094	-21.859	1.00	94.81	N
ATOM	2081	NH1	ARG	A	263	2.482	-27.266	-19.797	1.00	94.81	N
ATOM	2082	NH2	ARG	A	263	3.404	-25.984	-21.453	1.00	94.81	N
ATOM	2083	CZ	ARG	A	263	2.438	-26.781	-21.031	1.00	94.81	C
ATOM	2084	N	THR	A	264	-1.375	-33.875	-21.281	1.00	94.94	N
ATOM	2085	CA	THR	A	264	-2.412	-34.844	-20.922	1.00	94.94	C
ATOM	2086	C	THR	A	264	-1.841	-35.938	-20.031	1.00	94.94	C
ATOM	2087	CB	THR	A	264	-3.064	-35.469	-22.156	1.00	94.94	C
ATOM	2088	O	THR	A	264	-0.661	-36.281	-20.125	1.00	94.94	O
ATOM	2089	CG2	THR	A	264	-3.783	-34.406	-23.000	1.00	94.94	C
ATOM	2090	OG1	THR	A	264	-2.053	-36.094	-22.969	1.00	94.94	O
ATOM	2091	N	TYR	A	265	-2.662	-36.438	-19.094	1.00	95.94	N
ATOM	2092	CA	TYR	A	265	-2.332	-37.531	-18.172	1.00	95.94	C
ATOM	2093	C	TYR	A	265	-3.188	-38.781	-18.453	1.00	95.94	C
ATOM	2094	CB	TYR	A	265	-2.521	-37.094	-16.719	1.00	95.94	C
ATOM	2095	O	TYR	A	265	-4.410	-38.719	-18.312	1.00	95.94	O
ATOM	2096	CG	TYR	A	265	-1.663	-35.906	-16.328	1.00	95.94	C
ATOM	2097	CD1	TYR	A	265	-0.518	-36.094	-15.555	1.00	95.94	C
ATOM	2098	CD2	TYR	A	265	-1.995	-34.625	-16.734	1.00	95.94	C
ATOM	2099	CE1	TYR	A	265	0.275	-35.000	-15.188	1.00	95.94	C
ATOM	2100	CE2	TYR	A	265	-1.210	-33.531	-16.375	1.00	95.94	C
ATOM	2101	OH	TYR	A	265	0.703	-32.656	-15.242	1.00	95.94	O
ATOM	2102	CZ	TYR	A	265	-0.079	-33.750	-15.602	1.00	95.94	C
ATOM	2103	N	ASN	A	266	-2.592	-39.812	-18.906	1.00	93.56	N
ATOM	2104	CA	ASN	A	266	-3.342	-41.031	-19.281	1.00	93.56	C
ATOM	2105	C	ASN	A	266	-3.357	-42.062	-18.172	1.00	93.56	C
ATOM	2106	CB	ASN	A	266	-2.768	-41.625	-20.562	1.00	93.56	C
ATOM	2107	O	ASN	A	266	-2.359	-42.750	-17.938	1.00	93.56	O
ATOM	2108	CG	ASN	A	266	-2.936	-40.719	-21.766	1.00	93.56	C

```
ATOM   2109  ND2 ASN A 266      -1.932 -40.688 -22.641  1.00 93.56           N
ATOM   2110  OD1 ASN A 266      -3.957 -40.031 -21.906  1.00 93.56           O
ATOM   2111  N   GLY A 267      -4.531 -42.250 -17.500  1.00 92.56           N
ATOM   2112  CA  GLY A 267      -4.668 -43.188 -16.406  1.00 92.56           C
ATOM   2113  C   GLY A 267      -4.059 -42.688 -15.102  1.00 92.56           C
ATOM   2114  O   GLY A 267      -3.676 -41.500 -15.000  1.00 92.56           O
ATOM   2115  N   ASN A 268      -4.180 -43.469 -14.055  1.00 92.31           N
ATOM   2116  CA  ASN A 268      -3.553 -43.156 -12.766  1.00 92.31           C
ATOM   2117  C   ASN A 268      -2.037 -43.344 -12.828  1.00 92.31           C
ATOM   2118  CB  ASN A 268      -4.141 -44.062 -11.664  1.00 92.31           C
ATOM   2119  O   ASN A 268      -1.538 -44.250 -13.492  1.00 92.31           O
ATOM   2120  CG  ASN A 268      -5.617 -43.781 -11.438  1.00 92.31           C
ATOM   2121  ND2 ASN A 268      -6.289 -44.750 -10.781  1.00 92.31           N
ATOM   2122  OD1 ASN A 268      -6.148 -42.750 -11.844  1.00 92.31           O
ATOM   2123  N   TRP A 269      -1.271 -42.438 -12.422  1.00 95.19           N
ATOM   2124  CA  TRP A 269       0.187 -42.500 -12.383  1.00 95.19           C
ATOM   2125  C   TRP A 269       0.662 -43.594 -11.406  1.00 95.19           C
ATOM   2126  CB  TRP A 269       0.768 -41.156 -11.992  1.00 95.19           C
ATOM   2127  O   TRP A 269       0.229 -43.625 -10.250  1.00 95.19           O
ATOM   2128  CG  TRP A 269       2.266 -41.094 -11.961  1.00 95.19           C
ATOM   2129  CD1 TRP A 269       3.121 -41.719 -12.844  1.00 95.19           C
ATOM   2130  CD2 TRP A 269       3.086 -40.438 -11.008  1.00 95.19           C
ATOM   2131  CE2 TRP A 269       4.434 -40.656 -11.367  1.00 95.19           C
ATOM   2132  CE3 TRP A 269       2.814 -39.656  -9.875  1.00 95.19           C
ATOM   2133  NE1 TRP A 269       4.426 -41.438 -12.492  1.00 95.19           N
ATOM   2134  CH2 TRP A 269       5.211 -39.375  -9.539  1.00 95.19           C
ATOM   2135  CZ2 TRP A 269       5.504 -40.156 -10.633  1.00 95.19           C
ATOM   2136  CZ3 TRP A 269       3.883 -39.125  -9.148  1.00 95.19           C
ATOM   2137  N   ASP A 270       1.588 -44.438 -11.664  1.00 93.31           N
ATOM   2138  CA  ASP A 270       2.068 -45.562 -10.875  1.00 93.31           C
ATOM   2139  C   ASP A 270       3.287 -45.188 -10.047  1.00 93.31           C
ATOM   2140  CB  ASP A 270       2.412 -46.750 -11.789  1.00 93.31           C
ATOM   2141  O   ASP A 270       3.895 -46.031  -9.391  1.00 93.31           O
ATOM   2142  CG  ASP A 270       3.523 -46.438 -12.773  1.00 93.31           C
ATOM   2143  OD1 ASP A 270       4.066 -45.312 -12.742  1.00 93.31           O
ATOM   2144  OD2 ASP A 270       3.855 -47.312 -13.594  1.00 93.31           O
ATOM   2145  N   GLY A 271       3.738 -43.938 -10.133  1.00 93.25           N
ATOM   2146  CA  GLY A 271       4.840 -43.438  -9.328  1.00 93.25           C
ATOM   2147  C   GLY A 271       6.176 -43.469 -10.047  1.00 93.25           C
ATOM   2148  O   GLY A 271       7.191 -43.031  -9.508  1.00 93.25           O
ATOM   2149  N   THR A 272       6.281 -44.000 -11.305  1.00 95.31           N
ATOM   2150  CA  THR A 272       7.520 -44.125 -12.070  1.00 95.31           C
ATOM   2151  C   THR A 272       7.703 -42.906 -12.984  1.00 95.31           C
ATOM   2152  CB  THR A 272       7.543 -45.406 -12.906  1.00 95.31           C
ATOM   2153  O   THR A 272       6.730 -42.219 -13.328  1.00 95.31           O
ATOM   2154  CG2 THR A 272       7.402 -46.656 -12.016  1.00 95.31           C
ATOM   2155  OG1 THR A 272       6.461 -45.375 -13.844  1.00 95.31           O
ATOM   2156  N   PHE A 273       9.008 -42.625 -13.383  1.00 96.12           N
ATOM   2157  CA  PHE A 273       9.336 -41.438 -14.172  1.00 96.12           C
ATOM   2158  C   PHE A 273       9.898 -41.844 -15.531  1.00 96.12           C
ATOM   2159  CB  PHE A 273      10.344 -40.562 -13.430  1.00 96.12           C
ATOM   2160  O   PHE A 273      10.398 -42.969 -15.695  1.00 96.12           O
ATOM   2161  CG  PHE A 273       9.750 -39.812 -12.266  1.00 96.12           C
ATOM   2162  CD1 PHE A 273       9.141 -38.562 -12.453  1.00 96.12           C
ATOM   2163  CD2 PHE A 273       9.805 -40.344 -10.984  1.00 96.12           C
ATOM   2164  CE1 PHE A 273       8.594 -37.875 -11.375  1.00 96.12           C
ATOM   2165  CE2 PHE A 273       9.250 -39.656  -9.906  1.00 96.12           C
ATOM   2166  CZ  PHE A 273       8.648 -38.406 -10.102  1.00 96.12           C
ATOM   2167  N   LYS A 274       9.734 -41.031 -16.562  1.00 95.69           N
ATOM   2168  CA  LYS A 274      10.438 -41.031 -17.844  1.00 95.69           C
ATOM   2169  C   LYS A 274      11.141 -39.719 -18.109  1.00 95.69           C
ATOM   2170  CB  LYS A 274       9.461 -41.344 -18.984  1.00 95.69           C
ATOM   2171  O   LYS A 274      10.852 -38.719 -17.453  1.00 95.69           O
ATOM   2172  CG  LYS A 274       8.438 -40.250 -19.234  1.00 95.69           C
```

```
ATOM   2173  CD  LYS A 274       7.512 -40.625 -20.391  1.00 95.69           C
ATOM   2174  CE  LYS A 274       6.484 -39.531 -20.641  1.00 95.69           C
ATOM   2175  NZ  LYS A 274       5.551 -39.875 -21.750  1.00 95.69           N
ATOM   2176  N   LYS A 275      12.148 -39.625 -19.000  1.00 93.69           N
ATOM   2177  CA  LYS A 275      12.859 -38.406 -19.328  1.00 93.69           C
ATOM   2178  C   LYS A 275      12.203 -37.688 -20.516  1.00 93.69           C
ATOM   2179  CB  LYS A 275      14.328 -38.688 -19.641  1.00 93.69           C
ATOM   2180  O   LYS A 275      11.969 -38.312 -21.562  1.00 93.69           O
ATOM   2181  CG  LYS A 275      15.125 -39.219 -18.453  1.00 93.69           C
ATOM   2182  CD  LYS A 275      15.555 -38.062 -17.547  1.00 93.69           C
ATOM   2183  CE  LYS A 275      16.594 -38.531 -16.516  1.00 93.69           C
ATOM   2184  NZ  LYS A 275      17.047 -37.406 -15.656  1.00 93.69           N
ATOM   2185  N   ALA A 276      11.867 -36.438 -20.312  1.00 95.88           N
ATOM   2186  CA  ALA A 276      11.250 -35.656 -21.375  1.00 95.88           C
ATOM   2187  C   ALA A 276      11.406 -34.156 -21.109  1.00 95.88           C
ATOM   2188  CB  ALA A 276       9.773 -36.031 -21.516  1.00 95.88           C
ATOM   2189  O   ALA A 276      11.602 -33.750 -19.953  1.00 95.88           O
ATOM   2190  N   TRP A 277      11.383 -33.438 -22.219  1.00 94.75           N
ATOM   2191  CA  TRP A 277      11.336 -32.000 -22.062  1.00 94.75           C
ATOM   2192  C   TRP A 277       9.984 -31.547 -21.531  1.00 94.75           C
ATOM   2193  CB  TRP A 277      11.625 -31.312 -23.406  1.00 94.75           C
ATOM   2194  O   TRP A 277       8.945 -32.094 -21.938  1.00 94.75           O
ATOM   2195  CG  TRP A 277      11.500 -29.812 -23.359  1.00 94.75           C
ATOM   2196  CD1 TRP A 277      12.477 -28.922 -23.047  1.00 94.75           C
ATOM   2197  CD2 TRP A 277      10.320 -29.047 -23.641  1.00 94.75           C
ATOM   2198  CE2 TRP A 277      10.664 -27.688 -23.469  1.00 94.75           C
ATOM   2199  CE3 TRP A 277       9.016 -29.391 -24.000  1.00 94.75           C
ATOM   2200  NE1 TRP A 277      11.984 -27.641 -23.109  1.00 94.75           N
ATOM   2201  CH2 TRP A 277       8.461 -27.016 -24.031  1.00 94.75           C
ATOM   2202  CZ2 TRP A 277       9.734 -26.672 -23.672  1.00 94.75           C
ATOM   2203  CZ3 TRP A 277       8.094 -28.359 -24.203  1.00 94.75           C
ATOM   2204  N   THR A 278      10.039 -30.609 -20.594  1.00 95.81           N
ATOM   2205  CA  THR A 278       8.797 -30.031 -20.094  1.00 95.81           C
ATOM   2206  C   THR A 278       9.023 -28.609 -19.594  1.00 95.81           C
ATOM   2207  CB  THR A 278       8.180 -30.891 -18.969  1.00 95.81           C
ATOM   2208  O   THR A 278      10.125 -28.281 -19.141  1.00 95.81           O
ATOM   2209  CG2 THR A 278       9.086 -30.922 -17.750  1.00 95.81           C
ATOM   2210  OG1 THR A 278       6.910 -30.328 -18.594  1.00 95.81           O
ATOM   2211  N   ASN A 279       8.141 -27.781 -19.781  1.00 95.38           N
ATOM   2212  CA  ASN A 279       8.180 -26.469 -19.156  1.00 95.38           C
ATOM   2213  C   ASN A 279       7.094 -26.328 -18.094  1.00 95.38           C
ATOM   2214  CB  ASN A 279       8.055 -25.375 -20.203  1.00 95.38           C
ATOM   2215  O   ASN A 279       6.598 -25.219 -17.859  1.00 95.38           O
ATOM   2216  CG  ASN A 279       6.750 -25.438 -20.969  1.00 95.38           C
ATOM   2217  ND2 ASN A 279       6.543 -24.484 -21.875  1.00 95.38           N
ATOM   2218  OD1 ASN A 279       5.934 -26.344 -20.750  1.00 95.38           O
ATOM   2219  N   ASN A 280       6.633 -27.422 -17.609  1.00 96.50           N
ATOM   2220  CA  ASN A 280       5.777 -27.453 -16.422  1.00 96.50           C
ATOM   2221  C   ASN A 280       6.570 -27.188 -15.148  1.00 96.50           C
ATOM   2222  CB  ASN A 280       5.066 -28.812 -16.328  1.00 96.50           C
ATOM   2223  O   ASN A 280       7.500 -27.922 -14.820  1.00 96.50           O
ATOM   2224  CG  ASN A 280       4.012 -28.828 -15.234  1.00 96.50           C
ATOM   2225  ND2 ASN A 280       2.822 -29.328 -15.570  1.00 96.50           N
ATOM   2226  OD1 ASN A 280       4.262 -28.406 -14.102  1.00 96.50           O
ATOM   2227  N   PRO A 281       6.211 -26.078 -14.477  1.00 96.19           N
ATOM   2228  CA  PRO A 281       7.008 -25.625 -13.328  1.00 96.19           C
ATOM   2229  C   PRO A 281       7.117 -26.672 -12.234  1.00 96.19           C
ATOM   2230  CB  PRO A 281       6.242 -24.391 -12.828  1.00 96.19           C
ATOM   2231  O   PRO A 281       8.102 -26.703 -11.484  1.00 96.19           O
ATOM   2232  CG  PRO A 281       4.859 -24.547 -13.375  1.00 96.19           C
ATOM   2233  CD  PRO A 281       4.945 -25.312 -14.672  1.00 96.19           C
ATOM   2234  N   ALA A 282       6.141 -27.609 -12.023  1.00 96.38           N
ATOM   2235  CA  ALA A 282       6.168 -28.625 -10.992  1.00 96.38           C
ATOM   2236  C   ALA A 282       7.309 -29.625 -11.227  1.00 96.38           C
```

```
ATOM   2237  CB  ALA A 282       4.832 -29.375 -10.938  1.00 96.38           C
ATOM   2238  O   ALA A 282       8.070 -29.938 -10.305  1.00 96.38           O
ATOM   2239  N   TRP A 283       7.516 -29.969 -12.422  1.00 96.00           N
ATOM   2240  CA  TRP A 283       8.531 -30.969 -12.719  1.00 96.00           C
ATOM   2241  C   TRP A 283       9.906 -30.328 -12.859  1.00 96.00           C
ATOM   2242  CB  TRP A 283       8.180 -31.734 -13.992  1.00 96.00           C
ATOM   2243  O   TRP A 283      10.930 -30.984 -12.664  1.00 96.00           O
ATOM   2244  CG  TRP A 283       6.867 -32.469 -13.930  1.00 96.00           C
ATOM   2245  CD1 TRP A 283       5.801 -32.312 -14.773  1.00 96.00           C
ATOM   2246  CD2 TRP A 283       6.484 -33.438 -12.953  1.00 96.00           C
ATOM   2247  CE2 TRP A 283       5.172 -33.844 -13.273  1.00 96.00           C
ATOM   2248  CE3 TRP A 283       7.125 -34.031 -11.852  1.00 96.00           C
ATOM   2249  NE1 TRP A 283       4.777 -33.156 -14.383  1.00 96.00           N
ATOM   2250  CH2 TRP A 283       5.133 -35.344 -11.445  1.00 96.00           C
ATOM   2251  CZ2 TRP A 283       4.484 -34.812 -12.523  1.00 96.00           C
ATOM   2252  CZ3 TRP A 283       6.438 -34.969 -11.102  1.00 96.00           C
ATOM   2253  N   VAL A 284       9.891 -29.047 -13.227  1.00 95.12           N
ATOM   2254  CA  VAL A 284      11.133 -28.297 -13.164  1.00 95.12           C
ATOM   2255  C   VAL A 284      11.594 -28.188 -11.711  1.00 95.12           C
ATOM   2256  CB  VAL A 284      10.984 -26.891 -13.781  1.00 95.12           C
ATOM   2257  O   VAL A 284      12.781 -28.359 -11.414  1.00 95.12           O
ATOM   2258  CG1 VAL A 284      12.258 -26.078 -13.578  1.00 95.12           C
ATOM   2259  CG2 VAL A 284      10.648 -27.000 -15.266  1.00 95.12           C
ATOM   2260  N   LEU A 285      10.664 -27.922 -10.812  1.00 94.75           N
ATOM   2261  CA  LEU A 285      10.945 -27.859  -9.383  1.00 94.75           C
ATOM   2262  C   LEU A 285      11.469 -29.203  -8.875  1.00 94.75           C
ATOM   2263  CB  LEU A 285       9.688 -27.469  -8.609  1.00 94.75           C
ATOM   2264  O   LEU A 285      12.453 -29.250  -8.141  1.00 94.75           O
ATOM   2265  CG  LEU A 285       9.828 -27.344  -7.090  1.00 94.75           C
ATOM   2266  CD1 LEU A 285      10.867 -26.281  -6.742  1.00 94.75           C
ATOM   2267  CD2 LEU A 285       8.484 -27.016  -6.445  1.00 94.75           C
ATOM   2268  N   TYR A 286      10.812 -30.312  -9.250  1.00 94.19           N
ATOM   2269  CA  TYR A 286      11.242 -31.641  -8.859  1.00 94.19           C
ATOM   2270  C   TYR A 286      12.680 -31.906  -9.297  1.00 94.19           C
ATOM   2271  CB  TYR A 286      10.320 -32.719  -9.477  1.00 94.19           C
ATOM   2272  O   TYR A 286      13.484 -32.406  -8.516  1.00 94.19           O
ATOM   2273  CG  TYR A 286      10.695 -34.125  -9.117  1.00 94.19           C
ATOM   2274  CD1 TYR A 286      11.305 -34.969 -10.047  1.00 94.19           C
ATOM   2275  CD2 TYR A 286      10.438 -34.625  -7.844  1.00 94.19           C
ATOM   2276  CE1 TYR A 286      11.656 -36.250  -9.719  1.00 94.19           C
ATOM   2277  CE2 TYR A 286      10.781 -35.938  -7.508  1.00 94.19           C
ATOM   2278  OH  TYR A 286      11.734 -38.031  -8.125  1.00 94.19           O
ATOM   2279  CZ  TYR A 286      11.391 -36.750  -8.453  1.00 94.19           C
ATOM   2280  N   ASP A 287      13.016 -31.516 -10.461  1.00 93.19           N
ATOM   2281  CA  ASP A 287      14.352 -31.719 -11.000  1.00 93.19           C
ATOM   2282  C   ASP A 287      15.398 -30.969 -10.195  1.00 93.19           C
ATOM   2283  CB  ASP A 287      14.414 -31.297 -12.469  1.00 93.19           C
ATOM   2284  O   ASP A 287      16.469 -31.500  -9.891  1.00 93.19           O
ATOM   2285  CG  ASP A 287      15.727 -31.656 -13.141  1.00 93.19           C
ATOM   2286  OD1 ASP A 287      16.078 -32.844 -13.172  1.00 93.19           O
ATOM   2287  OD2 ASP A 287      16.406 -30.734 -13.641  1.00 93.19           O
ATOM   2288  N   LEU A 288      15.109 -29.750  -9.805  1.00 91.56           N
ATOM   2289  CA  LEU A 288      16.031 -28.922  -9.031  1.00 91.56           C
ATOM   2290  C   LEU A 288      16.234 -29.500  -7.633  1.00 91.56           C
ATOM   2291  CB  LEU A 288      15.492 -27.484  -8.930  1.00 91.56           C
ATOM   2292  O   LEU A 288      17.328 -29.391  -7.070  1.00 91.56           O
ATOM   2293  CG  LEU A 288      15.617 -26.625 -10.180  1.00 91.56           C
ATOM   2294  CD1 LEU A 288      14.844 -25.328 -10.016  1.00 91.56           C
ATOM   2295  CD2 LEU A 288      17.078 -26.344 -10.492  1.00 91.56           C
ATOM   2296  N   MET A 289      15.250 -30.156  -7.105  1.00 90.56           N
ATOM   2297  CA  MET A 289      15.305 -30.703  -5.750  1.00 90.56           C
ATOM   2298  C   MET A 289      16.109 -32.000  -5.715  1.00 90.56           C
ATOM   2299  CB  MET A 289      13.891 -30.953  -5.211  1.00 90.56           C
ATOM   2300  O   MET A 289      16.859 -32.250  -4.770  1.00 90.56           O
```

```
ATOM   2301  CG  MET A 289      13.148 -29.672  -4.840  1.00 90.56           C
ATOM   2302  SD  MET A 289      11.531 -30.016  -4.047  1.00 90.56           S
ATOM   2303  CE  MET A 289      10.422 -29.812  -5.473  1.00 90.56           C
ATOM   2304  N   ILE A 290      16.078 -32.781  -6.801  1.00 89.50           N
ATOM   2305  CA  ILE A 290      16.641 -34.125  -6.715  1.00 89.50           C
ATOM   2306  C   ILE A 290      17.984 -34.188  -7.449  1.00 89.50           C
ATOM   2307  CB  ILE A 290      15.672 -35.188  -7.297  1.00 89.50           C
ATOM   2308  O   ILE A 290      18.797 -35.062  -7.207  1.00 89.50           O
ATOM   2309  CG1 ILE A 290      15.359 -34.875  -8.766  1.00 89.50           C
ATOM   2310  CG2 ILE A 290      14.391 -35.250  -6.461  1.00 89.50           C
ATOM   2311  CD1 ILE A 290      14.797 -36.031  -9.539  1.00 89.50           C
ATOM   2312  N   ASN A 291      18.281 -33.219  -8.344  1.00 87.62           N
ATOM   2313  CA  ASN A 291      19.469 -33.312  -9.172  1.00 87.62           C
ATOM   2314  C   ASN A 291      20.734 -33.031  -8.359  1.00 87.62           C
ATOM   2315  CB  ASN A 291      19.391 -32.344 -10.352  1.00 87.62           C
ATOM   2316  O   ASN A 291      20.828 -32.031  -7.668  1.00 87.62           O
ATOM   2317  CG  ASN A 291      20.422 -32.625 -11.422  1.00 87.62           C
ATOM   2318  ND2 ASN A 291      19.969 -33.094 -12.578  1.00 87.62           N
ATOM   2319  OD1 ASN A 291      21.625 -32.438 -11.203  1.00 87.62           O
ATOM   2320  N   GLN A 292      21.812 -33.781  -8.516  1.00 84.38           N
ATOM   2321  CA  GLN A 292      23.047 -33.750  -7.742  1.00 84.38           C
ATOM   2322  C   GLN A 292      24.031 -32.719  -8.312  1.00 84.38           C
ATOM   2323  CB  GLN A 292      23.703 -35.125  -7.715  1.00 84.38           C
ATOM   2324  O   GLN A 292      24.891 -32.219  -7.586  1.00 84.38           O
ATOM   2325  CG  GLN A 292      22.938 -36.156  -6.879  1.00 84.38           C
ATOM   2326  CD  GLN A 292      23.578 -37.531  -6.883  1.00 84.38           C
ATOM   2327  NE2 GLN A 292      23.094 -38.406  -6.023  1.00 84.38           N
ATOM   2328  OE1 GLN A 292      24.516 -37.781  -7.648  1.00 84.38           O
ATOM   2329  N   ARG A 293      23.875 -32.250  -9.523  1.00 83.00           N
ATOM   2330  CA  ARG A 293      24.875 -31.453 -10.203  1.00 83.00           C
ATOM   2331  C   ARG A 293      24.609 -29.969 -10.023  1.00 83.00           C
ATOM   2332  CB  ARG A 293      24.938 -31.812 -11.695  1.00 83.00           C
ATOM   2333  O   ARG A 293      25.484 -29.219  -9.602  1.00 83.00           O
ATOM   2334  CG  ARG A 293      25.688 -33.094 -12.000  1.00 83.00           C
ATOM   2335  CD  ARG A 293      25.734 -33.375 -13.492  1.00 83.00           C
ATOM   2336  NE  ARG A 293      26.859 -32.688 -14.125  1.00 83.00           N
ATOM   2337  NH1 ARG A 293      26.031 -32.875 -16.266  1.00 83.00           N
ATOM   2338  NH2 ARG A 293      28.031 -31.844 -15.906  1.00 83.00           N
ATOM   2339  CZ  ARG A 293      26.969 -32.469 -15.438  1.00 83.00           C
ATOM   2340  N   TYR A 294      23.344 -29.672 -10.102  1.00 83.88           N
ATOM   2341  CA  TYR A 294      23.062 -28.250 -10.070  1.00 83.88           C
ATOM   2342  C   TYR A 294      21.953 -27.938  -9.070  1.00 83.88           C
ATOM   2343  CB  TYR A 294      22.672 -27.734 -11.461  1.00 83.88           C
ATOM   2344  O   TYR A 294      21.625 -26.766  -8.852  1.00 83.88           O
ATOM   2345  CG  TYR A 294      21.594 -28.562 -12.125  1.00 83.88           C
ATOM   2346  CD1 TYR A 294      21.922 -29.531 -13.070  1.00 83.88           C
ATOM   2347  CD2 TYR A 294      20.250 -28.375 -11.812  1.00 83.88           C
ATOM   2348  CE1 TYR A 294      20.922 -30.281 -13.688  1.00 83.88           C
ATOM   2349  CE2 TYR A 294      19.250 -29.125 -12.422  1.00 83.88           C
ATOM   2350  OH  TYR A 294      18.625 -30.828 -13.969  1.00 83.88           O
ATOM   2351  CZ  TYR A 294      19.609 -30.078 -13.359  1.00 83.88           C
ATOM   2352  N   GLY A 295      21.328 -28.906  -8.531  1.00 82.31           N
ATOM   2353  CA  GLY A 295      20.203 -28.734  -7.613  1.00 82.31           C
ATOM   2354  C   GLY A 295      20.578 -28.984  -6.164  1.00 82.31           C
ATOM   2355  O   GLY A 295      21.750 -28.875  -5.797  1.00 82.31           O
ATOM   2356  N   LEU A 296      19.594 -29.328  -5.242  1.00 82.62           N
ATOM   2357  CA  LEU A 296      19.750 -29.438  -3.797  1.00 82.62           C
ATOM   2358  C   LEU A 296      20.297 -30.812  -3.414  1.00 82.62           C
ATOM   2359  CB  LEU A 296      18.406 -29.203  -3.098  1.00 82.62           C
ATOM   2360  O   LEU A 296      20.891 -30.969  -2.346  1.00 82.62           O
ATOM   2361  CG  LEU A 296      17.891 -27.766  -3.064  1.00 82.62           C
ATOM   2362  CD1 LEU A 296      16.484 -27.703  -2.473  1.00 82.62           C
ATOM   2363  CD2 LEU A 296      18.859 -26.875  -2.271  1.00 82.62           C
ATOM   2364  N   ASP A 297      20.281 -31.766  -4.289  1.00 79.75           N
```

```
ATOM   2365  CA  ASP A 297      20.750 -33.125  -4.039  1.00 79.75           C
ATOM   2366  C   ASP A 297      20.078 -33.719  -2.809  1.00 79.75           C
ATOM   2367  CB  ASP A 297      22.266 -33.156  -3.867  1.00 79.75           C
ATOM   2368  O   ASP A 297      20.734 -34.344  -1.971  1.00 79.75           O
ATOM   2369  CG  ASP A 297      22.859 -34.531  -3.980  1.00 79.75           C
ATOM   2370  OD1 ASP A 297      22.172 -35.469  -4.480  1.00 79.75           O
ATOM   2371  OD2 ASP A 297      24.031 -34.719  -3.562  1.00 79.75           O
ATOM   2372  N   GLN A 298      18.719 -33.469  -2.619  1.00 69.19           N
ATOM   2373  CA  GLN A 298      17.984 -33.875  -1.423  1.00 69.19           C
ATOM   2374  C   GLN A 298      18.047 -35.406  -1.241  1.00 69.19           C
ATOM   2375  CB  GLN A 298      16.531 -33.406  -1.489  1.00 69.19           C
ATOM   2376  O   GLN A 298      18.016 -35.906  -0.114  1.00 69.19           O
ATOM   2377  CG  GLN A 298      16.375 -31.906  -1.289  1.00 69.19           C
ATOM   2378  CD  GLN A 298      16.625 -31.469   0.142  1.00 69.19           C
ATOM   2379  NE2 GLN A 298      17.000 -30.219   0.321  1.00 69.19           N
ATOM   2380  OE1 GLN A 298      16.484 -32.281   1.079  1.00 69.19           O
ATOM   2381  N   LYS A 299      18.078 -36.156  -2.346  1.00 64.06           N
ATOM   2382  CA  LYS A 299      18.156 -37.594  -2.223  1.00 64.06           C
ATOM   2383  C   LYS A 299      19.391 -38.031  -1.444  1.00 64.06           C
ATOM   2384  CB  LYS A 299      18.141 -38.250  -3.605  1.00 64.06           C
ATOM   2385  O   LYS A 299      19.328 -38.938  -0.621  1.00 64.06           O
ATOM   2386  CG  LYS A 299      17.844 -39.750  -3.582  1.00 64.06           C
ATOM   2387  CD  LYS A 299      17.812 -40.312  -4.988  1.00 64.06           C
ATOM   2388  CE  LYS A 299      17.625 -41.844  -4.961  1.00 64.06           C
ATOM   2389  NZ  LYS A 299      17.562 -42.406  -6.340  1.00 64.06           N
ATOM   2390  N   GLU A 300      20.453 -37.281  -1.552  1.00 61.22           N
ATOM   2391  CA  GLU A 300      21.688 -37.594  -0.843  1.00 61.22           C
ATOM   2392  C   GLU A 300      21.578 -37.250   0.640  1.00 61.22           C
ATOM   2393  CB  GLU A 300      22.859 -36.844  -1.466  1.00 61.22           C
ATOM   2394  O   GLU A 300      22.203 -37.906   1.480  1.00 61.22           O
ATOM   2395  CG  GLU A 300      23.391 -37.469  -2.740  1.00 61.22           C
ATOM   2396  CD  GLU A 300      24.078 -38.812  -2.506  1.00 61.22           C
ATOM   2397  OE1 GLU A 300      24.141 -39.625  -3.447  1.00 61.22           O
ATOM   2398  OE2 GLU A 300      24.531 -39.062  -1.367  1.00 61.22           O
ATOM   2399  N   LEU A 301      20.594 -36.312   0.963  1.00 63.91           N
ATOM   2400  CA  LEU A 301      20.406 -35.906   2.354  1.00 63.91           C
ATOM   2401  C   LEU A 301      19.344 -36.750   3.033  1.00 63.91           C
ATOM   2402  CB  LEU A 301      20.016 -34.438   2.439  1.00 63.91           C
ATOM   2403  O   LEU A 301      19.078 -36.594   4.227  1.00 63.91           O
ATOM   2404  CG  LEU A 301      21.094 -33.406   2.031  1.00 63.91           C
ATOM   2405  CD1 LEU A 301      20.516 -32.000   1.949  1.00 63.91           C
ATOM   2406  CD2 LEU A 301      22.266 -33.438   3.014  1.00 63.91           C
ATOM   2407  N   GLY A 302      18.750 -37.688   2.270  1.00 70.12           N
ATOM   2408  CA  GLY A 302      17.719 -38.594   2.816  1.00 70.12           C
ATOM   2409  C   GLY A 302      16.391 -37.906   3.008  1.00 70.12           C
ATOM   2410  O   GLY A 302      15.602 -38.281   3.881  1.00 70.12           O
ATOM   2411  N   ILE A 303      16.234 -36.750   2.445  1.00 77.56           N
ATOM   2412  CA  ILE A 303      14.977 -36.000   2.578  1.00 77.56           C
ATOM   2413  C   ILE A 303      14.086 -36.312   1.373  1.00 77.56           C
ATOM   2414  CB  ILE A 303      15.219 -34.500   2.721  1.00 77.56           C
ATOM   2415  O   ILE A 303      14.445 -35.969   0.237  1.00 77.56           O
ATOM   2416  CG1 ILE A 303      16.109 -34.219   3.932  1.00 77.56           C
ATOM   2417  CG2 ILE A 303      13.883 -33.750   2.832  1.00 77.56           C
ATOM   2418  CD1 ILE A 303      16.531 -32.750   4.055  1.00 77.56           C
ATOM   2419  N   ALA A 304      12.992 -36.969   1.555  1.00 85.94           N
ATOM   2420  CA  ALA A 304      12.078 -37.344   0.480  1.00 85.94           C
ATOM   2421  C   ALA A 304      11.266 -36.125   0.001  1.00 85.94           C
ATOM   2422  CB  ALA A 304      11.148 -38.469   0.944  1.00 85.94           C
ATOM   2423  O   ALA A 304      11.031 -35.188   0.763  1.00 85.94           O
ATOM   2424  N   VAL A 305      11.078 -36.062  -1.355  1.00 89.88           N
ATOM   2425  CA  VAL A 305      10.195 -35.062  -1.974  1.00 89.88           C
ATOM   2426  C   VAL A 305       8.836 -35.719  -2.246  1.00 89.88           C
ATOM   2427  CB  VAL A 305      10.797 -34.531  -3.283  1.00 89.88           C
ATOM   2428  O   VAL A 305       8.750 -36.812  -2.787  1.00 89.88           O
```

```
ATOM   2429  CG1 VAL A 305       9.836 -33.531  -3.941  1.00 89.88           C
ATOM   2430  CG2 VAL A 305      12.148 -33.844  -3.010  1.00 89.88           C
ATOM   2431  N   ASP A 306       7.727 -35.125  -1.739  1.00 91.69           N
ATOM   2432  CA  ASP A 306       6.367 -35.594  -1.967  1.00 91.69           C
ATOM   2433  C   ASP A 306       5.977 -35.469  -3.438  1.00 91.69           C
ATOM   2434  CB  ASP A 306       5.375 -34.812  -1.090  1.00 91.69           C
ATOM   2435  O   ASP A 306       5.488 -34.406  -3.865  1.00 91.69           O
ATOM   2436  CG  ASP A 306       3.967 -35.406  -1.153  1.00 91.69           C
ATOM   2437  OD1 ASP A 306       3.703 -36.281  -1.994  1.00 91.69           O
ATOM   2438  OD2 ASP A 306       3.113 -34.938  -0.358  1.00 91.69           O
ATOM   2439  N   LYS A 307       6.211 -36.469  -4.230  1.00 92.75           N
ATOM   2440  CA  LYS A 307       5.984 -36.438  -5.672  1.00 92.75           C
ATOM   2441  C   LYS A 307       4.492 -36.406  -5.996  1.00 92.75           C
ATOM   2442  CB  LYS A 307       6.645 -37.656  -6.348  1.00 92.75           C
ATOM   2443  O   LYS A 307       4.086 -35.938  -7.059  1.00 92.75           O
ATOM   2444  CG  LYS A 307       6.133 -39.000  -5.852  1.00 92.75           C
ATOM   2445  CD  LYS A 307       6.879 -40.156  -6.504  1.00 92.75           C
ATOM   2446  CE  LYS A 307       6.398 -41.500  -5.977  1.00 92.75           C
ATOM   2447  NZ  LYS A 307       7.184 -42.625  -6.551  1.00 92.75           N
ATOM   2448  N   TRP A 308       3.691 -36.875  -5.094  1.00 93.38           N
ATOM   2449  CA  TRP A 308       2.250 -36.938  -5.320  1.00 93.38           C
ATOM   2450  C   TRP A 308       1.650 -35.531  -5.281  1.00 93.38           C
ATOM   2451  CB  TRP A 308       1.569 -37.812  -4.281  1.00 93.38           C
ATOM   2452  O   TRP A 308       0.781 -35.188  -6.090  1.00 93.38           O
ATOM   2453  CG  TRP A 308       2.100 -39.219  -4.242  1.00 93.38           C
ATOM   2454  CD1 TRP A 308       2.834 -39.812  -3.242  1.00 93.38           C
ATOM   2455  CD2 TRP A 308       1.937 -40.219  -5.246  1.00 93.38           C
ATOM   2456  CE2 TRP A 308       2.600 -41.406  -4.793  1.00 93.38           C
ATOM   2457  CE3 TRP A 308       1.292 -40.250  -6.492  1.00 93.38           C
ATOM   2458  NE1 TRP A 308       3.139 -41.094  -3.568  1.00 93.38           N
ATOM   2459  CH2 TRP A 308       2.000 -42.562  -6.754  1.00 93.38           C
ATOM   2460  CZ2 TRP A 308       2.639 -42.562  -5.539  1.00 93.38           C
ATOM   2461  CZ3 TRP A 308       1.331 -41.438  -7.234  1.00 93.38           C
ATOM   2462  N   ALA A 309       2.094 -34.719  -4.309  1.00 92.31           N
ATOM   2463  CA  ALA A 309       1.654 -33.344  -4.234  1.00 92.31           C
ATOM   2464  C   ALA A 309       2.092 -32.562  -5.469  1.00 92.31           C
ATOM   2465  CB  ALA A 309       2.195 -32.656  -2.969  1.00 92.31           C
ATOM   2466  O   ALA A 309       1.339 -31.719  -5.988  1.00 92.31           O
ATOM   2467  N   LEU A 310       3.293 -32.812  -5.988  1.00 94.56           N
ATOM   2468  CA  LEU A 310       3.789 -32.156  -7.191  1.00 94.56           C
ATOM   2469  C   LEU A 310       2.980 -32.562  -8.414  1.00 94.56           C
ATOM   2470  CB  LEU A 310       5.270 -32.469  -7.410  1.00 94.56           C
ATOM   2471  O   LEU A 310       2.756 -31.766  -9.320  1.00 94.56           O
ATOM   2472  CG  LEU A 310       6.270 -31.672  -6.574  1.00 94.56           C
ATOM   2473  CD1 LEU A 310       7.656 -32.312  -6.656  1.00 94.56           C
ATOM   2474  CD2 LEU A 310       6.309 -30.219  -7.039  1.00 94.56           C
ATOM   2475  N   TYR A 311       2.611 -33.875  -8.422  1.00 95.81           N
ATOM   2476  CA  TYR A 311       1.771 -34.375  -9.492  1.00 95.81           C
ATOM   2477  C   TYR A 311       0.457 -33.625  -9.578  1.00 95.81           C
ATOM   2478  CB  TYR A 311       1.491 -35.875  -9.289  1.00 95.81           C
ATOM   2479  O   TYR A 311       0.037 -33.219 -10.664  1.00 95.81           O
ATOM   2480  CG  TYR A 311       0.716 -36.500 -10.414  1.00 95.81           C
ATOM   2481  CD1 TYR A 311      -0.598 -36.938 -10.234  1.00 95.81           C
ATOM   2482  CD2 TYR A 311       1.297 -36.688 -11.664  1.00 95.81           C
ATOM   2483  CE1 TYR A 311      -1.315 -37.531 -11.266  1.00 95.81           C
ATOM   2484  CE2 TYR A 311       0.589 -37.281 -12.711  1.00 95.81           C
ATOM   2485  OH  TYR A 311      -1.420 -38.281 -13.531  1.00 95.81           O
ATOM   2486  CZ  TYR A 311      -0.714 -37.688 -12.500  1.00 95.81           C
ATOM   2487  N   GLU A 312      -0.186 -33.375  -8.438  1.00 94.06           N
ATOM   2488  CA  GLU A 312      -1.424 -32.594  -8.391  1.00 94.06           C
ATOM   2489  C   GLU A 312      -1.197 -31.156  -8.852  1.00 94.06           C
ATOM   2490  CB  GLU A 312      -2.008 -32.594  -6.977  1.00 94.06           C
ATOM   2491  O   GLU A 312      -2.008 -30.609  -9.602  1.00 94.06           O
ATOM   2492  CG  GLU A 312      -2.592 -33.938  -6.562  1.00 94.06           C
```

ATOM	2493	CD	GLU	A	312	-3.141	-33.969	-5.145	1.00	94.06	C
ATOM	2494	OE1	GLU	A	312	-3.568	-35.031	-4.664	1.00	94.06	O
ATOM	2495	OE2	GLU	A	312	-3.146	-32.875	-4.512	1.00	94.06	O
ATOM	2496	N	ALA	A	313	-0.091	-30.578	-8.398	1.00	95.31	N
ATOM	2497	CA	ALA	A	313	0.275	-29.234	-8.820	1.00	95.31	C
ATOM	2498	C	ALA	A	313	0.505	-29.172	-10.328	1.00	95.31	C
ATOM	2499	CB	ALA	A	313	1.521	-28.750	-8.078	1.00	95.31	C
ATOM	2500	O	ALA	A	313	0.084	-28.219	-10.992	1.00	95.31	O
ATOM	2501	N	ALA	A	314	1.120	-30.219	-10.859	1.00	96.19	N
ATOM	2502	CA	ALA	A	314	1.399	-30.281	-12.289	1.00	96.19	C
ATOM	2503	C	ALA	A	314	0.107	-30.344	-13.102	1.00	96.19	C
ATOM	2504	CB	ALA	A	314	2.271	-31.500	-12.609	1.00	96.19	C
ATOM	2505	O	ALA	A	314	-0.013	-29.672	-14.133	1.00	96.19	O
ATOM	2506	N	GLN	A	315	-0.833	-31.094	-12.672	1.00	95.56	N
ATOM	2507	CA	GLN	A	315	-2.121	-31.156	-13.359	1.00	95.56	C
ATOM	2508	C	GLN	A	315	-2.811	-29.797	-13.367	1.00	95.56	C
ATOM	2509	CB	GLN	A	315	-3.023	-32.219	-12.703	1.00	95.56	C
ATOM	2510	O	GLN	A	315	-3.398	-29.406	-14.375	1.00	95.56	O
ATOM	2511	CG	GLN	A	315	-2.584	-33.656	-12.945	1.00	95.56	C
ATOM	2512	CD	GLN	A	315	-3.490	-34.656	-12.273	1.00	95.56	C
ATOM	2513	NE2	GLN	A	315	-4.164	-35.469	-13.078	1.00	95.56	N
ATOM	2514	OE1	GLN	A	315	-3.584	-34.719	-11.039	1.00	95.56	O
ATOM	2515	N	TYR	A	316	-2.674	-29.141	-12.234	1.00	95.44	N
ATOM	2516	CA	TYR	A	316	-3.246	-27.812	-12.117	1.00	95.44	C
ATOM	2517	C	TYR	A	316	-2.574	-26.844	-13.086	1.00	95.44	C
ATOM	2518	CB	TYR	A	316	-3.111	-27.281	-10.688	1.00	95.44	C
ATOM	2519	O	TYR	A	316	-3.244	-26.031	-13.734	1.00	95.44	O
ATOM	2520	CG	TYR	A	316	-3.828	-25.984	-10.438	1.00	95.44	C
ATOM	2521	CD1	TYR	A	316	-3.123	-24.828	-10.102	1.00	95.44	C
ATOM	2522	CD2	TYR	A	316	-5.211	-25.891	-10.555	1.00	95.44	C
ATOM	2523	CE1	TYR	A	316	-3.781	-23.625	-9.867	1.00	95.44	C
ATOM	2524	CE2	TYR	A	316	-5.879	-24.703	-10.328	1.00	95.44	C
ATOM	2525	OH	TYR	A	316	-5.812	-22.375	-9.766	1.00	95.44	O
ATOM	2526	CZ	TYR	A	316	-5.156	-23.562	-9.984	1.00	95.44	C
ATOM	2527	N	CYS	A	317	-1.272	-26.828	-13.172	1.00	95.75	N
ATOM	2528	CA	CYS	A	317	-0.509	-25.984	-14.086	1.00	95.75	C
ATOM	2529	C	CYS	A	317	-0.906	-26.250	-15.531	1.00	95.75	C
ATOM	2530	CB	CYS	A	317	0.990	-26.219	-13.906	1.00	95.75	C
ATOM	2531	O	CYS	A	317	-0.948	-25.328	-16.344	1.00	95.75	O
ATOM	2532	SG	CYS	A	317	1.638	-25.641	-12.320	1.00	95.75	S
ATOM	2533	N	ASP	A	318	-1.350	-27.453	-15.875	1.00	96.88	N
ATOM	2534	CA	ASP	A	318	-1.626	-27.859	-17.250	1.00	96.88	C
ATOM	2535	C	ASP	A	318	-3.098	-27.656	-17.594	1.00	96.88	C
ATOM	2536	CB	ASP	A	318	-1.239	-29.328	-17.469	1.00	96.88	C
ATOM	2537	O	ASP	A	318	-3.516	-27.906	-18.734	1.00	96.88	O
ATOM	2538	CG	ASP	A	318	0.248	-29.516	-17.703	1.00	96.88	C
ATOM	2539	OD1	ASP	A	318	0.917	-28.578	-18.172	1.00	96.88	O
ATOM	2540	OD2	ASP	A	318	0.756	-30.625	-17.406	1.00	96.88	O
ATOM	2541	N	GLN	A	319	-3.898	-27.172	-16.734	1.00	95.19	N
ATOM	2542	CA	GLN	A	319	-5.293	-26.875	-17.062	1.00	95.19	C
ATOM	2543	C	GLN	A	319	-5.395	-25.719	-18.047	1.00	95.19	C
ATOM	2544	CB	GLN	A	319	-6.074	-26.547	-15.781	1.00	95.19	C
ATOM	2545	O	GLN	A	319	-4.742	-24.688	-17.875	1.00	95.19	O
ATOM	2546	CG	GLN	A	319	-6.344	-27.750	-14.898	1.00	95.19	C
ATOM	2547	CD	GLN	A	319	-7.094	-27.406	-13.633	1.00	95.19	C
ATOM	2548	NE2	GLN	A	319	-7.582	-28.422	-12.922	1.00	95.19	N
ATOM	2549	OE1	GLN	A	319	-7.238	-26.219	-13.289	1.00	95.19	O
ATOM	2550	N	MET	A	320	-6.191	-25.922	-19.125	1.00	94.75	N
ATOM	2551	CA	MET	A	320	-6.344	-24.891	-20.156	1.00	94.75	C
ATOM	2552	C	MET	A	320	-7.199	-23.734	-19.641	1.00	94.75	C
ATOM	2553	CB	MET	A	320	-6.965	-25.484	-21.422	1.00	94.75	C
ATOM	2554	O	MET	A	320	-8.312	-23.953	-19.156	1.00	94.75	O
ATOM	2555	CG	MET	A	320	-6.113	-26.547	-22.078	1.00	94.75	C
ATOM	2556	SD	MET	A	320	-4.449	-25.938	-22.547	1.00	94.75	S

ATOM	2557	CE	MET	A	320	-4.836	-25.125	-24.109	1.00	94.75	C
ATOM	2558	N	VAL	A	321	-6.637	-22.516	-19.688	1.00	95.19	N
ATOM	2559	CA	VAL	A	321	-7.344	-21.297	-19.297	1.00	95.19	C
ATOM	2560	C	VAL	A	321	-7.363	-20.328	-20.469	1.00	95.19	C
ATOM	2561	CB	VAL	A	321	-6.695	-20.641	-18.062	1.00	95.19	C
ATOM	2562	O	VAL	A	321	-6.543	-20.422	-21.391	1.00	95.19	O
ATOM	2563	CG1	VAL	A	321	-6.777	-21.562	-16.844	1.00	95.19	C
ATOM	2564	CG2	VAL	A	321	-5.246	-20.266	-18.359	1.00	95.19	C
ATOM	2565	N	PRO	A	322	-8.367	-19.422	-20.547	1.00	94.44	N
ATOM	2566	CA	PRO	A	322	-8.461	-18.469	-21.656	1.00	94.44	C
ATOM	2567	C	PRO	A	322	-7.223	-17.578	-21.766	1.00	94.44	C
ATOM	2568	CB	PRO	A	322	-9.695	-17.641	-21.281	1.00	94.44	C
ATOM	2569	O	PRO	A	322	-6.684	-17.125	-20.766	1.00	94.44	O
ATOM	2570	CG	PRO	A	322	-10.523	-18.531	-20.422	1.00	94.44	C
ATOM	2571	CD	PRO	A	322	-9.594	-19.391	-19.609	1.00	94.44	C
ATOM	2572	N	ASP	A	323	-6.652	-17.391	-23.031	1.00	92.06	N
ATOM	2573	CA	ASP	A	323	-5.445	-16.609	-23.266	1.00	92.06	C
ATOM	2574	C	ASP	A	323	-5.773	-15.125	-23.422	1.00	92.06	C
ATOM	2575	CB	ASP	A	323	-4.703	-17.109	-24.500	1.00	92.06	C
ATOM	2576	O	ASP	A	323	-4.879	-14.297	-23.641	1.00	92.06	O
ATOM	2577	CG	ASP	A	323	-5.496	-16.938	-25.781	1.00	92.06	C
ATOM	2578	OD1	ASP	A	323	-6.629	-16.406	-25.734	1.00	92.06	O
ATOM	2579	OD2	ASP	A	323	-4.984	-17.328	-26.859	1.00	92.06	O
ATOM	2580	N	GLY	A	324	-7.078	-14.688	-23.375	1.00	86.19	N
ATOM	2581	CA	GLY	A	324	-7.488	-13.305	-23.516	1.00	86.19	C
ATOM	2582	C	GLY	A	324	-7.625	-12.859	-24.953	1.00	86.19	C
ATOM	2583	O	GLY	A	324	-8.008	-11.719	-25.234	1.00	86.19	O
ATOM	2584	N	LYS	A	325	-7.297	-13.742	-25.938	1.00	86.88	N
ATOM	2585	CA	LYS	A	325	-7.387	-13.438	-27.359	1.00	86.88	C
ATOM	2586	C	LYS	A	325	-8.367	-14.383	-28.062	1.00	86.88	C
ATOM	2587	CB	LYS	A	325	-6.008	-13.531	-28.016	1.00	86.88	C
ATOM	2588	O	LYS	A	325	-8.281	-14.570	-29.281	1.00	86.88	O
ATOM	2589	CG	LYS	A	325	-4.977	-12.586	-27.422	1.00	86.88	C
ATOM	2590	CD	LYS	A	325	-3.598	-12.805	-28.031	1.00	86.88	C
ATOM	2591	CE	LYS	A	325	-2.543	-11.938	-27.375	1.00	86.88	C
ATOM	2592	NZ	LYS	A	325	-1.177	-12.211	-27.906	1.00	86.88	N
ATOM	2593	N	GLY	A	326	-9.242	-15.062	-27.266	1.00	85.62	N
ATOM	2594	CA	GLY	A	326	-10.219	-15.969	-27.844	1.00	85.62	C
ATOM	2595	C	GLY	A	326	-9.758	-17.406	-27.875	1.00	85.62	C
ATOM	2596	O	GLY	A	326	-10.500	-18.297	-28.312	1.00	85.62	O
ATOM	2597	N	GLY	A	327	-8.602	-17.844	-27.391	1.00	93.88	N
ATOM	2598	CA	GLY	A	327	-8.062	-19.188	-27.281	1.00	93.88	C
ATOM	2599	C	GLY	A	327	-7.770	-19.594	-25.844	1.00	93.88	C
ATOM	2600	O	GLY	A	327	-8.227	-18.938	-24.906	1.00	93.88	O
ATOM	2601	N	THR	A	328	-7.160	-20.797	-25.688	1.00	94.94	N
ATOM	2602	CA	THR	A	328	-6.789	-21.297	-24.359	1.00	94.94	C
ATOM	2603	C	THR	A	328	-5.285	-21.531	-24.281	1.00	94.94	C
ATOM	2604	CB	THR	A	328	-7.539	-22.594	-24.016	1.00	94.94	C
ATOM	2605	O	THR	A	328	-4.605	-21.625	-25.297	1.00	94.94	O
ATOM	2606	CG2	THR	A	328	-9.047	-22.375	-24.031	1.00	94.94	C
ATOM	2607	OG1	THR	A	328	-7.207	-23.594	-24.984	1.00	94.94	O
ATOM	2608	N	GLU	A	329	-4.754	-21.469	-23.172	1.00	94.88	N
ATOM	2609	CA	GLU	A	329	-3.357	-21.766	-22.859	1.00	94.88	C
ATOM	2610	C	GLU	A	329	-3.223	-22.469	-21.516	1.00	94.88	C
ATOM	2611	CB	GLU	A	329	-2.525	-20.484	-22.875	1.00	94.88	C
ATOM	2612	O	GLU	A	329	-4.113	-22.375	-20.672	1.00	94.88	O
ATOM	2613	CG	GLU	A	329	-2.914	-19.484	-21.781	1.00	94.88	C
ATOM	2614	CD	GLU	A	329	-2.129	-18.188	-21.844	1.00	94.88	C
ATOM	2615	OE1	GLU	A	329	-2.398	-17.281	-21.031	1.00	94.88	O
ATOM	2616	OE2	GLU	A	329	-1.236	-18.078	-22.719	1.00	94.88	O
ATOM	2617	N	PRO	A	330	-2.139	-23.266	-21.359	1.00	95.75	N
ATOM	2618	CA	PRO	A	330	-1.921	-23.812	-20.016	1.00	95.75	C
ATOM	2619	C	PRO	A	330	-1.797	-22.719	-18.953	1.00	95.75	C
ATOM	2620	CB	PRO	A	330	-0.609	-24.594	-20.156	1.00	95.75	C

```
ATOM   2621  O   PRO A 330      -1.371 -21.609 -19.250  1.00 95.75           O
ATOM   2622  CG  PRO A 330      -0.452 -24.812 -21.625  1.00 95.75           C
ATOM   2623  CD  PRO A 330      -1.157 -23.703 -22.359  1.00 95.75           C
ATOM   2624  N   ARG A 331      -2.137 -23.031 -17.828  1.00 95.44           N
ATOM   2625  CA  ARG A 331      -2.262 -22.031 -16.766  1.00 95.44           C
ATOM   2626  C   ARG A 331      -0.904 -21.438 -16.406  1.00 95.44           C
ATOM   2627  CB  ARG A 331      -2.896 -22.656 -15.516  1.00 95.44           C
ATOM   2628  O   ARG A 331      -0.767 -20.219 -16.312  1.00 95.44           O
ATOM   2629  CG  ARG A 331      -3.266 -21.641 -14.445  1.00 95.44           C
ATOM   2630  CD  ARG A 331      -4.023 -22.297 -13.297  1.00 95.44           C
ATOM   2631  NE  ARG A 331      -5.355 -22.734 -13.703  1.00 95.44           N
ATOM   2632  NH1 ARG A 331      -6.469 -21.469 -12.109  1.00 95.44           N
ATOM   2633  NH2 ARG A 331      -7.648 -22.781 -13.578  1.00 95.44           N
ATOM   2634  CZ  ARG A 331      -6.488 -22.328 -13.133  1.00 95.44           C
ATOM   2635  N   TYR A 332       0.128 -22.344 -16.094  1.00 96.00           N
ATOM   2636  CA  TYR A 332       1.455 -21.875 -15.719  1.00 96.00           C
ATOM   2637  C   TYR A 332       2.535 -22.547 -16.547  1.00 96.00           C
ATOM   2638  CB  TYR A 332       1.713 -22.125 -14.227  1.00 96.00           C
ATOM   2639  O   TYR A 332       2.465 -23.766 -16.797  1.00 96.00           O
ATOM   2640  CG  TYR A 332       0.758 -21.391 -13.312  1.00 96.00           C
ATOM   2641  CD1 TYR A 332       0.827 -20.000 -13.172  1.00 96.00           C
ATOM   2642  CD2 TYR A 332      -0.215 -22.078 -12.602  1.00 96.00           C
ATOM   2643  CE1 TYR A 332      -0.050 -19.328 -12.328  1.00 96.00           C
ATOM   2644  CE2 TYR A 332      -1.097 -21.406 -11.758  1.00 96.00           C
ATOM   2645  OH  TYR A 332      -1.878 -19.359 -10.797  1.00 96.00           O
ATOM   2646  CZ  TYR A 332      -1.007 -20.031 -11.633  1.00 96.00           C
ATOM   2647  N   LEU A 333       3.551 -21.766 -16.953  1.00 94.94           N
ATOM   2648  CA  LEU A 333       4.754 -22.281 -17.594  1.00 94.94           C
ATOM   2649  C   LEU A 333       6.008 -21.812 -16.859  1.00 94.94           C
ATOM   2650  CB  LEU A 333       4.812 -21.812 -19.062  1.00 94.94           C
ATOM   2651  O   LEU A 333       5.969 -20.828 -16.125  1.00 94.94           O
ATOM   2652  CG  LEU A 333       3.660 -22.266 -19.953  1.00 94.94           C
ATOM   2653  CD1 LEU A 333       3.830 -21.703 -21.359  1.00 94.94           C
ATOM   2654  CD2 LEU A 333       3.574 -23.797 -19.984  1.00 94.94           C
ATOM   2655  N   CYS A 334       7.086 -22.516 -17.031  1.00 95.25           N
ATOM   2656  CA  CYS A 334       8.375 -22.125 -16.469  1.00 95.25           C
ATOM   2657  C   CYS A 334       9.438 -22.031 -17.547  1.00 95.25           C
ATOM   2658  CB  CYS A 334       8.812 -23.125 -15.398  1.00 95.25           C
ATOM   2659  O   CYS A 334       9.984 -23.047 -17.984  1.00 95.25           O
ATOM   2660  SG  CYS A 334      10.367 -22.688 -14.586  1.00 95.25           S
ATOM   2661  N   ASP A 335       9.727 -20.812 -17.969  1.00 94.00           N
ATOM   2662  CA  ASP A 335      10.789 -20.531 -18.922  1.00 94.00           C
ATOM   2663  C   ASP A 335      11.891 -19.688 -18.297  1.00 94.00           C
ATOM   2664  CB  ASP A 335      10.219 -19.828 -20.156  1.00 94.00           C
ATOM   2665  O   ASP A 335      11.898 -18.469 -18.453  1.00 94.00           O
ATOM   2666  CG  ASP A 335       9.250 -20.688 -20.938  1.00 94.00           C
ATOM   2667  OD1 ASP A 335       9.500 -21.906 -21.094  1.00 94.00           O
ATOM   2668  OD2 ASP A 335       8.227 -20.156 -21.422  1.00 94.00           O
ATOM   2669  N   VAL A 336      12.734 -20.391 -17.625  1.00 91.69           N
ATOM   2670  CA  VAL A 336      13.742 -19.672 -16.844  1.00 91.69           C
ATOM   2671  C   VAL A 336      15.141 -20.109 -17.297  1.00 91.69           C
ATOM   2672  CB  VAL A 336      13.570 -19.922 -15.328  1.00 91.69           C
ATOM   2673  O   VAL A 336      15.344 -21.250 -17.703  1.00 91.69           O
ATOM   2674  CG1 VAL A 336      13.875 -21.375 -14.977  1.00 91.69           C
ATOM   2675  CG2 VAL A 336      14.461 -18.969 -14.531  1.00 91.69           C
ATOM   2676  N   ILE A 337      16.047 -19.109 -17.250  1.00 89.50           N
ATOM   2677  CA  ILE A 337      17.484 -19.359 -17.422  1.00 89.50           C
ATOM   2678  C   ILE A 337      18.234 -18.938 -16.156  1.00 89.50           C
ATOM   2679  CB  ILE A 337      18.047 -18.625 -18.656  1.00 89.50           C
ATOM   2680  O   ILE A 337      18.172 -17.781 -15.750  1.00 89.50           O
ATOM   2681  CG1 ILE A 337      17.328 -19.094 -19.922  1.00 89.50           C
ATOM   2682  CG2 ILE A 337      19.562 -18.844 -18.766  1.00 89.50           C
ATOM   2683  CD1 ILE A 337      17.719 -18.328 -21.172  1.00 89.50           C
ATOM   2684  N   ILE A 338      18.844 -19.875 -15.516  1.00 89.06           N
```

ATOM	2685	CA	ILE	A	338	19.641	-19.594	-14.328	1.00	89.06	C
ATOM	2686	C	ILE	A	338	21.109	-19.406	-14.734	1.00	89.06	C
ATOM	2687	CB	ILE	A	338	19.516	-20.703	-13.273	1.00	89.06	C
ATOM	2688	O	ILE	A	338	21.812	-20.391	-15.008	1.00	89.06	O
ATOM	2689	CG1	ILE	A	338	18.031	-20.938	-12.922	1.00	89.06	C
ATOM	2690	CG2	ILE	A	338	20.328	-20.375	-12.023	1.00	89.06	C
ATOM	2691	CD1	ILE	A	338	17.781	-22.188	-12.078	1.00	89.06	C
ATOM	2692	N	GLN	A	339	21.656	-18.156	-14.742	1.00	85.75	N
ATOM	2693	CA	GLN	A	339	22.938	-17.812	-15.328	1.00	85.75	C
ATOM	2694	C	GLN	A	339	24.016	-17.656	-14.250	1.00	85.75	C
ATOM	2695	CB	GLN	A	339	22.844	-16.531	-16.156	1.00	85.75	C
ATOM	2696	O	GLN	A	339	25.172	-18.031	-14.453	1.00	85.75	O
ATOM	2697	CG	GLN	A	339	22.062	-16.703	-17.453	1.00	85.75	C
ATOM	2698	CD	GLN	A	339	22.047	-15.445	-18.297	1.00	85.75	C
ATOM	2699	NE2	GLN	A	339	21.312	-15.477	-19.406	1.00	85.75	N
ATOM	2700	OE1	GLN	A	339	22.703	-14.453	-17.969	1.00	85.75	O
ATOM	2701	N	SER	A	340	23.562	-17.078	-13.094	1.00	87.06	N
ATOM	2702	CA	SER	A	340	24.547	-16.812	-12.047	1.00	87.06	C
ATOM	2703	C	SER	A	340	24.250	-17.641	-10.805	1.00	87.06	C
ATOM	2704	CB	SER	A	340	24.547	-15.320	-11.688	1.00	87.06	C
ATOM	2705	O	SER	A	340	23.125	-18.078	-10.586	1.00	87.06	O
ATOM	2706	OG	SER	A	340	23.250	-14.891	-11.297	1.00	87.06	O
ATOM	2707	N	GLN	A	341	25.266	-17.953	-10.047	1.00	84.06	N
ATOM	2708	CA	GLN	A	341	25.156	-18.719	-8.805	1.00	84.06	C
ATOM	2709	C	GLN	A	341	24.297	-17.984	-7.785	1.00	84.06	C
ATOM	2710	CB	GLN	A	341	26.547	-18.969	-8.219	1.00	84.06	C
ATOM	2711	O	GLN	A	341	24.469	-16.781	-7.555	1.00	84.06	O
ATOM	2712	CG	GLN	A	341	26.547	-19.922	-7.031	1.00	84.06	C
ATOM	2713	CD	GLN	A	341	27.953	-20.219	-6.520	1.00	84.06	C
ATOM	2714	NE2	GLN	A	341	28.078	-21.281	-5.719	1.00	84.06	N
ATOM	2715	OE1	GLN	A	341	28.906	-19.516	-6.844	1.00	84.06	O
ATOM	2716	N	THR	A	342	23.125	-18.641	-7.355	1.00	85.50	N
ATOM	2717	CA	THR	A	342	22.219	-18.125	-6.332	1.00	85.50	C
ATOM	2718	C	THR	A	342	21.906	-19.203	-5.301	1.00	85.50	C
ATOM	2719	CB	THR	A	342	20.922	-17.609	-6.957	1.00	85.50	C
ATOM	2720	O	THR	A	342	22.094	-20.391	-5.562	1.00	85.50	O
ATOM	2721	CG2	THR	A	342	19.797	-17.531	-5.922	1.00	85.50	C
ATOM	2722	OG1	THR	A	342	21.141	-16.297	-7.492	1.00	85.50	O
ATOM	2723	N	ASP	A	343	21.531	-18.734	-4.066	1.00	87.06	N
ATOM	2724	CA	ASP	A	343	21.141	-19.672	-3.023	1.00	87.06	C
ATOM	2725	C	ASP	A	343	20.062	-20.641	-3.527	1.00	87.06	C
ATOM	2726	CB	ASP	A	343	20.641	-18.922	-1.786	1.00	87.06	C
ATOM	2727	O	ASP	A	343	19.047	-20.203	-4.086	1.00	87.06	O
ATOM	2728	CG	ASP	A	343	20.438	-19.828	-0.583	1.00	87.06	C
ATOM	2729	OD1	ASP	A	343	20.531	-21.078	-0.732	1.00	87.06	O
ATOM	2730	OD2	ASP	A	343	20.188	-19.312	0.522	1.00	87.06	O
ATOM	2731	N	ALA	A	344	20.391	-21.969	-3.426	1.00	82.38	N
ATOM	2732	CA	ALA	A	344	19.531	-23.031	-3.961	1.00	82.38	C
ATOM	2733	C	ALA	A	344	18.109	-22.906	-3.418	1.00	82.38	C
ATOM	2734	CB	ALA	A	344	20.109	-24.406	-3.631	1.00	82.38	C
ATOM	2735	O	ALA	A	344	17.141	-23.062	-4.164	1.00	82.38	O
ATOM	2736	N	TYR	A	345	17.984	-22.641	-2.107	1.00	82.56	N
ATOM	2737	CA	TYR	A	345	16.656	-22.531	-1.499	1.00	82.56	C
ATOM	2738	C	TYR	A	345	15.875	-21.375	-2.098	1.00	82.56	C
ATOM	2739	CB	TYR	A	345	16.781	-22.359	0.018	1.00	82.56	C
ATOM	2740	O	TYR	A	345	14.664	-21.469	-2.309	1.00	82.56	O
ATOM	2741	CG	TYR	A	345	15.453	-22.391	0.739	1.00	82.56	C
ATOM	2742	CD1	TYR	A	345	14.883	-21.234	1.257	1.00	82.56	C
ATOM	2743	CD2	TYR	A	345	14.766	-23.594	0.906	1.00	82.56	C
ATOM	2744	CE1	TYR	A	345	13.664	-21.266	1.924	1.00	82.56	C
ATOM	2745	CE2	TYR	A	345	13.547	-23.625	1.571	1.00	82.56	C
ATOM	2746	OH	TYR	A	345	11.797	-22.500	2.736	1.00	82.56	O
ATOM	2747	CZ	TYR	A	345	13.000	-22.469	2.076	1.00	82.56	C
ATOM	2748	N	LYS	A	346	16.531	-20.266	-2.371	1.00	89.00	N

ATOM	2749	CA	LYS	A	346	15.875	-19.125	-3.020	1.00	89.00	C
ATOM	2750	C	LYS	A	346	15.328	-19.516	-4.391	1.00	89.00	C
ATOM	2751	CB	LYS	A	346	16.859	-17.953	-3.158	1.00	89.00	C
ATOM	2752	O	LYS	A	346	14.203	-19.156	-4.738	1.00	89.00	O
ATOM	2753	CG	LYS	A	346	16.234	-16.703	-3.738	1.00	89.00	C
ATOM	2754	CD	LYS	A	346	17.250	-15.562	-3.818	1.00	89.00	C
ATOM	2755	CE	LYS	A	346	16.641	-14.312	-4.441	1.00	89.00	C
ATOM	2756	NZ	LYS	A	346	17.625	-13.195	-4.520	1.00	89.00	N
ATOM	2757	N	VAL	A	347	16.125	-20.281	-5.203	1.00	90.38	N
ATOM	2758	CA	VAL	A	347	15.703	-20.734	-6.523	1.00	90.38	C
ATOM	2759	C	VAL	A	347	14.477	-21.641	-6.398	1.00	90.38	C
ATOM	2760	CB	VAL	A	347	16.844	-21.484	-7.262	1.00	90.38	C
ATOM	2761	O	VAL	A	347	13.516	-21.500	-7.160	1.00	90.38	O
ATOM	2762	CG1	VAL	A	347	16.344	-22.031	-8.594	1.00	90.38	C
ATOM	2763	CG2	VAL	A	347	18.031	-20.547	-7.484	1.00	90.38	C
ATOM	2764	N	ILE	A	348	14.484	-22.484	-5.410	1.00	90.00	N
ATOM	2765	CA	ILE	A	348	13.367	-23.391	-5.168	1.00	90.00	C
ATOM	2766	C	ILE	A	348	12.117	-22.594	-4.824	1.00	90.00	C
ATOM	2767	CB	ILE	A	348	13.695	-24.406	-4.039	1.00	90.00	C
ATOM	2768	O	ILE	A	348	11.039	-22.844	-5.383	1.00	90.00	O
ATOM	2769	CG1	ILE	A	348	14.805	-25.359	-4.480	1.00	90.00	C
ATOM	2770	CG2	ILE	A	348	12.438	-25.172	-3.625	1.00	90.00	C
ATOM	2771	CD1	ILE	A	348	14.438	-26.219	-5.676	1.00	90.00	C
ATOM	2772	N	ARG	A	349	12.242	-21.609	-3.977	1.00	89.94	N
ATOM	2773	CA	ARG	A	349	11.109	-20.766	-3.596	1.00	89.94	C
ATOM	2774	C	ARG	A	349	10.586	-19.969	-4.789	1.00	89.94	C
ATOM	2775	CB	ARG	A	349	11.500	-19.812	-2.465	1.00	89.94	C
ATOM	2776	O	ARG	A	349	9.375	-19.844	-4.977	1.00	89.94	O
ATOM	2777	CG	ARG	A	349	10.320	-19.078	-1.845	1.00	89.94	C
ATOM	2778	CD	ARG	A	349	10.773	-18.109	-0.764	1.00	89.94	C
ATOM	2779	NE	ARG	A	349	9.797	-17.031	-0.563	1.00	89.94	N
ATOM	2780	NH1	ARG	A	349	11.039	-15.922	1.037	1.00	89.94	N
ATOM	2781	NH2	ARG	A	349	9.000	-15.109	0.390	1.00	89.94	N
ATOM	2782	CZ	ARG	A	349	9.945	-16.031	0.287	1.00	89.94	C
ATOM	2783	N	ASP	A	350	11.523	-19.500	-5.637	1.00	92.44	N
ATOM	2784	CA	ASP	A	350	11.148	-18.688	-6.797	1.00	92.44	C
ATOM	2785	C	ASP	A	350	10.383	-19.531	-7.820	1.00	92.44	C
ATOM	2786	CB	ASP	A	350	12.391	-18.078	-7.449	1.00	92.44	C
ATOM	2787	O	ASP	A	350	9.359	-19.078	-8.352	1.00	92.44	O
ATOM	2788	CG	ASP	A	350	12.945	-16.891	-6.676	1.00	92.44	C
ATOM	2789	OD1	ASP	A	350	12.258	-16.391	-5.758	1.00	92.44	O
ATOM	2790	OD2	ASP	A	350	14.070	-16.469	-6.984	1.00	92.44	O
ATOM	2791	N	ILE	A	351	10.867	-20.781	-8.023	1.00	93.81	N
ATOM	2792	CA	ILE	A	351	10.203	-21.656	-8.992	1.00	93.81	C
ATOM	2793	C	ILE	A	351	8.844	-22.078	-8.445	1.00	93.81	C
ATOM	2794	CB	ILE	A	351	11.062	-22.891	-9.320	1.00	93.81	C
ATOM	2795	O	ILE	A	351	7.863	-22.156	-9.195	1.00	93.81	O
ATOM	2796	CG1	ILE	A	351	12.383	-22.469	-9.984	1.00	93.81	C
ATOM	2797	CG2	ILE	A	351	10.289	-23.859	-10.211	1.00	93.81	C
ATOM	2798	CD1	ILE	A	351	12.195	-21.703	-11.289	1.00	93.81	C
ATOM	2799	N	CYS	A	352	8.781	-22.359	-7.129	1.00	92.88	N
ATOM	2800	CA	CYS	A	352	7.520	-22.734	-6.500	1.00	92.88	C
ATOM	2801	C	CYS	A	352	6.500	-21.609	-6.602	1.00	92.88	C
ATOM	2802	CB	CYS	A	352	7.742	-23.109	-5.035	1.00	92.88	C
ATOM	2803	O	CYS	A	352	5.309	-21.859	-6.781	1.00	92.88	O
ATOM	2804	SG	CYS	A	352	6.367	-24.031	-4.301	1.00	92.88	S
ATOM	2805	N	SER	A	353	6.910	-20.328	-6.559	1.00	93.38	N
ATOM	2806	CA	SER	A	353	6.043	-19.156	-6.602	1.00	93.38	C
ATOM	2807	C	SER	A	353	5.371	-19.016	-7.961	1.00	93.38	C
ATOM	2808	CB	SER	A	353	6.840	-17.891	-6.281	1.00	93.38	C
ATOM	2809	O	SER	A	353	4.328	-18.359	-8.078	1.00	93.38	O
ATOM	2810	OG	SER	A	353	7.727	-17.578	-7.340	1.00	93.38	O
ATOM	2811	N	ILE	A	354	5.945	-19.672	-9.094	1.00	94.75	N
ATOM	2812	CA	ILE	A	354	5.395	-19.594	-10.438	1.00	94.75	C

```
ATOM   2813  C   ILE A 354       3.959 -20.109 -10.438  1.00 94.75           C
ATOM   2814  CB  ILE A 354       6.254 -20.375 -11.453  1.00 94.75           C
ATOM   2815  O   ILE A 354       3.082 -19.547 -11.086  1.00 94.75           O
ATOM   2816  CG1 ILE A 354       7.605 -19.688 -11.664  1.00 94.75           C
ATOM   2817  CG2 ILE A 354       5.512 -20.547 -12.781  1.00 94.75           C
ATOM   2818  CD1 ILE A 354       8.586 -20.484 -12.500  1.00 94.75           C
ATOM   2819  N   PHE A 355       3.654 -21.156  -9.703  1.00 93.12           N
ATOM   2820  CA  PHE A 355       2.311 -21.719  -9.711  1.00 93.12           C
ATOM   2821  C   PHE A 355       1.610 -21.484  -8.383  1.00 93.12           C
ATOM   2822  CB  PHE A 355       2.363 -23.234 -10.008  1.00 93.12           C
ATOM   2823  O   PHE A 355       0.845 -22.328  -7.910  1.00 93.12           O
ATOM   2824  CG  PHE A 355       3.434 -23.969  -9.250  1.00 93.12           C
ATOM   2825  CD1 PHE A 355       4.707 -24.109  -9.781  1.00 93.12           C
ATOM   2826  CD2 PHE A 355       3.164 -24.500  -7.996  1.00 93.12           C
ATOM   2827  CE1 PHE A 355       5.699 -24.781  -9.078  1.00 93.12           C
ATOM   2828  CE2 PHE A 355       4.152 -25.188  -7.289  1.00 93.12           C
ATOM   2829  CZ  PHE A 355       5.418 -25.328  -7.828  1.00 93.12           C
ATOM   2830  N   ARG A 356       1.954 -20.328  -7.723  1.00 91.38           N
ATOM   2831  CA  ARG A 356       1.342 -19.844  -6.488  1.00 91.38           C
ATOM   2832  C   ARG A 356       1.553 -20.844  -5.348  1.00 91.38           C
ATOM   2833  CB  ARG A 356      -0.153 -19.594  -6.691  1.00 91.38           C
ATOM   2834  O   ARG A 356       0.651 -21.062  -4.535  1.00 91.38           O
ATOM   2835  CG  ARG A 356      -0.465 -18.625  -7.816  1.00 91.38           C
ATOM   2836  CD  ARG A 356      -1.954 -18.578  -8.133  1.00 91.38           C
ATOM   2837  NE  ARG A 356      -2.248 -17.672  -9.234  1.00 91.38           N
ATOM   2838  NH1 ARG A 356      -4.379 -18.484  -9.609  1.00 91.38           N
ATOM   2839  NH2 ARG A 356      -3.553 -16.781 -10.906  1.00 91.38           N
ATOM   2840  CZ  ARG A 356      -3.393 -17.641  -9.914  1.00 91.38           C
ATOM   2841  N   GLY A 357       2.652 -21.484  -5.324  1.00 88.88           N
ATOM   2842  CA  GLY A 357       2.900 -22.516  -4.332  1.00 88.88           C
ATOM   2843  C   GLY A 357       3.963 -22.125  -3.322  1.00 88.88           C
ATOM   2844  O   GLY A 357       4.629 -21.109  -3.477  1.00 88.88           O
ATOM   2845  N   MET A 358       3.936 -22.781  -2.295  1.00 88.00           N
ATOM   2846  CA  MET A 358       4.938 -22.703  -1.238  1.00 88.00           C
ATOM   2847  C   MET A 358       5.477 -24.078  -0.885  1.00 88.00           C
ATOM   2848  CB  MET A 358       4.352 -22.031   0.008  1.00 88.00           C
ATOM   2849  O   MET A 358       4.715 -25.047  -0.810  1.00 88.00           O
ATOM   2850  CG  MET A 358       3.916 -20.594  -0.214  1.00 88.00           C
ATOM   2851  SD  MET A 358       3.273 -19.812   1.315  1.00 88.00           S
ATOM   2852  CE  MET A 358       2.879 -21.281   2.291  1.00 88.00           C
ATOM   2853  N   SER A 359       6.844 -24.125  -0.861  1.00 86.62           N
ATOM   2854  CA  SER A 359       7.461 -25.375  -0.410  1.00 86.62           C
ATOM   2855  C   SER A 359       7.637 -25.391   1.104  1.00 86.62           C
ATOM   2856  CB  SER A 359       8.820 -25.578  -1.089  1.00 86.62           C
ATOM   2857  O   SER A 359       7.828 -24.328   1.723  1.00 86.62           O
ATOM   2858  OG  SER A 359       9.750 -24.594  -0.653  1.00 86.62           O
ATOM   2859  N   PHE A 360       7.336 -26.453   1.772  1.00 83.69           N
ATOM   2860  CA  PHE A 360       7.508 -26.594   3.213  1.00 83.69           C
ATOM   2861  C   PHE A 360       7.902 -28.016   3.574  1.00 83.69           C
ATOM   2862  CB  PHE A 360       6.223 -26.188   3.949  1.00 83.69           C
ATOM   2863  O   PHE A 360       7.754 -28.922   2.762  1.00 83.69           O
ATOM   2864  CG  PHE A 360       5.078 -27.141   3.723  1.00 83.69           C
ATOM   2865  CD1 PHE A 360       4.285 -27.047   2.586  1.00 83.69           C
ATOM   2866  CD2 PHE A 360       4.793 -28.141   4.648  1.00 83.69           C
ATOM   2867  CE1 PHE A 360       3.227 -27.922   2.375  1.00 83.69           C
ATOM   2868  CE2 PHE A 360       3.738 -29.031   4.441  1.00 83.69           C
ATOM   2869  CZ  PHE A 360       2.953 -28.906   3.307  1.00 83.69           C
ATOM   2870  N   TRP A 361       8.555 -28.141   4.824  1.00 81.31           N
ATOM   2871  CA  TRP A 361       8.875 -29.438   5.402  1.00 81.31           C
ATOM   2872  C   TRP A 361       7.734 -29.953   6.270  1.00 81.31           C
ATOM   2873  CB  TRP A 361      10.164 -29.359   6.230  1.00 81.31           C
ATOM   2874  O   TRP A 361       7.363 -29.312   7.262  1.00 81.31           O
ATOM   2875  CG  TRP A 361      10.539 -30.641   6.906  1.00 81.31           C
ATOM   2876  CD1 TRP A 361      10.531 -30.906   8.250  1.00 81.31           C
```

ATOM	2877	CD2	TRP	A	361	11.000	-31.828	6.270	1.00	81.31	C
ATOM	2878	CE2	TRP	A	361	11.242	-32.781	7.285	1.00	81.31	C
ATOM	2879	CE3	TRP	A	361	11.219	-32.188	4.934	1.00	81.31	C
ATOM	2880	NE1	TRP	A	361	10.945	-32.188	8.484	1.00	81.31	N
ATOM	2881	CH2	TRP	A	361	11.906	-34.406	5.695	1.00	81.31	C
ATOM	2882	CZ2	TRP	A	361	11.695	-34.062	7.008	1.00	81.31	C
ATOM	2883	CZ3	TRP	A	361	11.680	-33.469	4.660	1.00	81.31	C
ATOM	2884	N	ASN	A	362	7.059	-31.062	5.883	1.00	75.50	N
ATOM	2885	CA	ASN	A	362	5.891	-31.562	6.605	1.00	75.50	C
ATOM	2886	C	ASN	A	362	6.285	-32.562	7.691	1.00	75.50	C
ATOM	2887	CB	ASN	A	362	4.887	-32.188	5.641	1.00	75.50	C
ATOM	2888	O	ASN	A	362	5.418	-33.156	8.328	1.00	75.50	O
ATOM	2889	CG	ASN	A	362	5.418	-33.438	4.984	1.00	75.50	C
ATOM	2890	ND2	ASN	A	362	4.660	-34.000	4.039	1.00	75.50	N
ATOM	2891	OD1	ASN	A	362	6.500	-33.938	5.328	1.00	75.50	O
ATOM	2892	N	GLY	A	363	7.566	-32.719	7.996	1.00	76.88	N
ATOM	2893	CA	GLY	A	363	8.078	-33.625	8.992	1.00	76.88	C
ATOM	2894	C	GLY	A	363	8.695	-34.875	8.391	1.00	76.88	C
ATOM	2895	O	GLY	A	363	9.594	-35.469	8.977	1.00	76.88	O
ATOM	2896	N	GLU	A	364	8.297	-35.219	7.176	1.00	83.12	N
ATOM	2897	CA	GLU	A	364	8.781	-36.406	6.504	1.00	83.12	C
ATOM	2898	C	GLU	A	364	9.398	-36.094	5.148	1.00	83.12	C
ATOM	2899	CB	GLU	A	364	7.641	-37.438	6.332	1.00	83.12	C
ATOM	2900	O	GLU	A	364	10.422	-36.656	4.773	1.00	83.12	O
ATOM	2901	CG	GLU	A	364	7.168	-38.062	7.637	1.00	83.12	C
ATOM	2902	CD	GLU	A	364	6.020	-39.031	7.453	1.00	83.12	C
ATOM	2903	OE1	GLU	A	364	5.559	-39.625	8.461	1.00	83.12	O
ATOM	2904	OE2	GLU	A	364	5.582	-39.219	6.297	1.00	83.12	O
ATOM	2905	N	SER	A	365	8.867	-35.219	4.441	1.00	86.19	N
ATOM	2906	CA	SER	A	365	9.281	-34.906	3.082	1.00	86.19	C
ATOM	2907	C	SER	A	365	9.102	-33.406	2.795	1.00	86.19	C
ATOM	2908	CB	SER	A	365	8.484	-35.719	2.066	1.00	86.19	C
ATOM	2909	O	SER	A	365	8.469	-32.688	3.576	1.00	86.19	O
ATOM	2910	OG	SER	A	365	7.102	-35.406	2.146	1.00	86.19	O
ATOM	2911	N	ILE	A	366	9.883	-32.969	1.891	1.00	86.06	N
ATOM	2912	CA	ILE	A	366	9.617	-31.625	1.351	1.00	86.06	C
ATOM	2913	C	ILE	A	366	8.352	-31.672	0.498	1.00	86.06	C
ATOM	2914	CB	ILE	A	366	10.805	-31.109	0.518	1.00	86.06	C
ATOM	2915	O	ILE	A	366	8.258	-32.438	-0.457	1.00	86.06	O
ATOM	2916	CG1	ILE	A	366	12.062	-31.000	1.390	1.00	86.06	C
ATOM	2917	CG2	ILE	A	366	10.469	-29.750	-0.118	1.00	86.06	C
ATOM	2918	CD1	ILE	A	366	13.320	-30.641	0.616	1.00	86.06	C
ATOM	2919	N	SER	A	367	7.320	-30.953	0.851	1.00	87.75	N
ATOM	2920	CA	SER	A	367	6.039	-30.953	0.153	1.00	87.75	C
ATOM	2921	C	SER	A	367	5.703	-29.562	-0.375	1.00	87.75	C
ATOM	2922	CB	SER	A	367	4.922	-31.438	1.077	1.00	87.75	C
ATOM	2923	O	SER	A	367	6.422	-28.609	-0.107	1.00	87.75	O
ATOM	2924	OG	SER	A	367	3.750	-31.734	0.338	1.00	87.75	O
ATOM	2925	N	VAL	A	368	4.754	-29.484	-1.319	1.00	88.50	N
ATOM	2926	CA	VAL	A	368	4.312	-28.219	-1.890	1.00	88.50	C
ATOM	2927	C	VAL	A	368	2.805	-28.062	-1.694	1.00	88.50	C
ATOM	2928	CB	VAL	A	368	4.668	-28.109	-3.391	1.00	88.50	C
ATOM	2929	O	VAL	A	368	2.068	-29.047	-1.684	1.00	88.50	O
ATOM	2930	CG1	VAL	A	368	6.184	-28.062	-3.584	1.00	88.50	C
ATOM	2931	CG2	VAL	A	368	4.059	-29.281	-4.164	1.00	88.50	C
ATOM	2932	N	ILE	A	369	2.455	-26.844	-1.449	1.00	87.12	N
ATOM	2933	CA	ILE	A	369	1.036	-26.516	-1.398	1.00	87.12	C
ATOM	2934	C	ILE	A	369	0.744	-25.359	-2.359	1.00	87.12	C
ATOM	2935	CB	ILE	A	369	0.595	-26.141	0.034	1.00	87.12	C
ATOM	2936	O	ILE	A	369	1.545	-24.438	-2.490	1.00	87.12	O
ATOM	2937	CG1	ILE	A	369	-0.930	-25.984	0.101	1.00	87.12	C
ATOM	2938	CG2	ILE	A	369	1.292	-24.859	0.498	1.00	87.12	C
ATOM	2939	CD1	ILE	A	369	-1.490	-26.000	1.517	1.00	87.12	C
ATOM	2940	N	ILE	A	370	-0.328	-25.531	-3.156	1.00	89.88	N

```
ATOM   2941  CA  ILE A 370      -0.654 -24.516  -4.152  1.00 89.88           C
ATOM   2942  C   ILE A 370      -1.985 -23.859  -3.799  1.00 89.88           C
ATOM   2943  CB  ILE A 370      -0.715 -25.125  -5.574  1.00 89.88           C
ATOM   2944  O   ILE A 370      -2.818 -24.453  -3.111  1.00 89.88           O
ATOM   2945  CG1 ILE A 370      -1.790 -26.203  -5.648  1.00 89.88           C
ATOM   2946  CG2 ILE A 370       0.654 -25.672  -5.984  1.00 89.88           C
ATOM   2947  CD1 ILE A 370      -2.029 -26.750  -7.051  1.00 89.88           C
ATOM   2948  N   ASP A 371      -2.123 -22.531  -4.184  1.00 90.00           N
ATOM   2949  CA  ASP A 371      -3.359 -21.766  -4.020  1.00 90.00           C
ATOM   2950  C   ASP A 371      -4.379 -22.156  -5.094  1.00 90.00           C
ATOM   2951  CB  ASP A 371      -3.080 -20.266  -4.070  1.00 90.00           C
ATOM   2952  O   ASP A 371      -4.348 -21.609  -6.203  1.00 90.00           O
ATOM   2953  CG  ASP A 371      -4.289 -19.422  -3.697  1.00 90.00           C
ATOM   2954  OD1 ASP A 371      -5.258 -19.969  -3.125  1.00 90.00           O
ATOM   2955  OD2 ASP A 371      -4.273 -18.219  -3.980  1.00 90.00           O
ATOM   2956  N   ARG A 372      -5.281 -23.016  -4.809  1.00 89.81           N
ATOM   2957  CA  ARG A 372      -6.332 -23.531  -5.688  1.00 89.81           C
ATOM   2958  C   ARG A 372      -7.652 -23.672  -4.934  1.00 89.81           C
ATOM   2959  CB  ARG A 372      -5.926 -24.875  -6.289  1.00 89.81           C
ATOM   2960  O   ARG A 372      -7.680 -23.609  -3.703  1.00 89.81           O
ATOM   2961  CG  ARG A 372      -5.762 -25.984  -5.266  1.00 89.81           C
ATOM   2962  CD  ARG A 372      -5.406 -27.312  -5.922  1.00 89.81           C
ATOM   2963  NE  ARG A 372      -5.141 -28.344  -4.934  1.00 89.81           N
ATOM   2964  NH1 ARG A 372      -7.316 -29.125  -4.859  1.00 89.81           N
ATOM   2965  NH2 ARG A 372      -5.703 -30.094  -3.551  1.00 89.81           N
ATOM   2966  CZ  ARG A 372      -6.055 -29.188  -4.449  1.00 89.81           C
ATOM   2967  N   PRO A 373      -8.742 -23.766  -5.719  1.00 89.88           N
ATOM   2968  CA  PRO A 373     -10.008 -23.953  -5.012  1.00 89.88           C
ATOM   2969  C   PRO A 373      -9.977 -25.141  -4.051  1.00 89.88           C
ATOM   2970  CB  PRO A 373     -11.008 -24.188  -6.141  1.00 89.88           C
ATOM   2971  O   PRO A 373      -9.555 -26.234  -4.434  1.00 89.88           O
ATOM   2972  CG  PRO A 373     -10.414 -23.500  -7.328  1.00 89.88           C
ATOM   2973  CD  PRO A 373      -8.922 -23.625  -7.258  1.00 89.88           C
ATOM   2974  N   ARG A 374     -10.328 -24.797  -2.768  1.00 89.06           N
ATOM   2975  CA  ARG A 374     -10.328 -25.828  -1.723  1.00 89.06           C
ATOM   2976  C   ARG A 374     -11.648 -25.828  -0.958  1.00 89.06           C
ATOM   2977  CB  ARG A 374      -9.164 -25.594  -0.756  1.00 89.06           C
ATOM   2978  O   ARG A 374     -12.305 -24.797  -0.840  1.00 89.06           O
ATOM   2979  CG  ARG A 374      -7.789 -25.734  -1.400  1.00 89.06           C
ATOM   2980  CD  ARG A 374      -6.672 -25.391  -0.422  1.00 89.06           C
ATOM   2981  NE  ARG A 374      -6.516 -23.953  -0.250  1.00 89.06           N
ATOM   2982  NH1 ARG A 374      -4.848 -24.109   1.341  1.00 89.06           N
ATOM   2983  NH2 ARG A 374      -5.598 -22.062   0.661  1.00 89.06           N
ATOM   2984  CZ  ARG A 374      -5.656 -23.375   0.583  1.00 89.06           C
ATOM   2985  N   GLU A 375     -11.914 -26.984  -0.468  1.00 89.44           N
ATOM   2986  CA  GLU A 375     -13.070 -27.109   0.411  1.00 89.44           C
ATOM   2987  C   GLU A 375     -12.664 -27.047   1.879  1.00 89.44           C
ATOM   2988  CB  GLU A 375     -13.820 -28.422   0.132  1.00 89.44           C
ATOM   2989  O   GLU A 375     -11.508 -27.312   2.221  1.00 89.44           O
ATOM   2990  CG  GLU A 375     -14.391 -28.516  -1.275  1.00 89.44           C
ATOM   2991  CD  GLU A 375     -15.523 -27.531  -1.529  1.00 89.44           C
ATOM   2992  OE1 GLU A 375     -15.625 -27.016  -2.662  1.00 89.44           O
ATOM   2993  OE2 GLU A 375     -16.312 -27.281  -0.590  1.00 89.44           O
ATOM   2994  N   PRO A 376     -13.539 -26.484   2.701  1.00 92.25           N
ATOM   2995  CA  PRO A 376     -13.203 -26.375   4.121  1.00 92.25           C
ATOM   2996  C   PRO A 376     -12.859 -27.719   4.754  1.00 92.25           C
ATOM   2997  CB  PRO A 376     -14.477 -25.781   4.742  1.00 92.25           C
ATOM   2998  O   PRO A 376     -13.523 -28.719   4.480  1.00 92.25           O
ATOM   2999  CG  PRO A 376     -15.180 -25.109   3.607  1.00 92.25           C
ATOM   3000  CD  PRO A 376     -14.859 -25.859   2.342  1.00 92.25           C
ATOM   3001  N   ALA A 377     -11.797 -27.734   5.562  1.00 92.81           N
ATOM   3002  CA  ALA A 377     -11.328 -28.938   6.250  1.00 92.81           C
ATOM   3003  C   ALA A 377     -12.078 -29.141   7.562  1.00 92.81           C
ATOM   3004  CB  ALA A 377      -9.828 -28.844   6.512  1.00 92.81           C
```

```
ATOM   3005  O   ALA A 377     -12.156 -30.266   8.070  1.00 92.81           O
ATOM   3006  N   TYR A 378     -12.562 -28.047   8.117  1.00 94.44           N
ATOM   3007  CA  TYR A 378     -13.242 -28.125   9.398  1.00 94.44           C
ATOM   3008  C   TYR A 378     -14.086 -26.875   9.641  1.00 94.44           C
ATOM   3009  CB  TYR A 378     -12.227 -28.281  10.539  1.00 94.44           C
ATOM   3010  O   TYR A 378     -13.797 -25.797   9.102  1.00 94.44           O
ATOM   3011  CG  TYR A 378     -12.805 -28.938  11.773  1.00 94.44           C
ATOM   3012  CD1 TYR A 378     -13.102 -28.188  12.906  1.00 94.44           C
ATOM   3013  CD2 TYR A 378     -13.039 -30.312  11.805  1.00 94.44           C
ATOM   3014  CE1 TYR A 378     -13.633 -28.797  14.047  1.00 94.44           C
ATOM   3015  CE2 TYR A 378     -13.570 -30.922  12.938  1.00 94.44           C
ATOM   3016  OH  TYR A 378     -14.375 -30.750  15.180  1.00 94.44           O
ATOM   3017  CZ  TYR A 378     -13.859 -30.156  14.055  1.00 94.44           C
ATOM   3018  N   ILE A 379     -15.141 -27.000  10.445  1.00 94.75           N
ATOM   3019  CA  ILE A 379     -16.016 -25.875  10.781  1.00 94.75           C
ATOM   3020  C   ILE A 379     -15.781 -25.453  12.227  1.00 94.75           C
ATOM   3021  CB  ILE A 379     -17.500 -26.234  10.555  1.00 94.75           C
ATOM   3022  O   ILE A 379     -15.742 -26.297  13.125  1.00 94.75           O
ATOM   3023  CG1 ILE A 379     -17.734 -26.641   9.094  1.00 94.75           C
ATOM   3024  CG2 ILE A 379     -18.391 -25.047  10.930  1.00 94.75           C
ATOM   3025  CD1 ILE A 379     -19.031 -27.406   8.875  1.00 94.75           C
ATOM   3026  N   PHE A 380     -15.570 -24.172  12.469  1.00 95.62           N
ATOM   3027  CA  PHE A 380     -15.469 -23.594  13.805  1.00 95.62           C
ATOM   3028  C   PHE A 380     -16.656 -22.688  14.102  1.00 95.62           C
ATOM   3029  CB  PHE A 380     -14.164 -22.812  13.953  1.00 95.62           C
ATOM   3030  O   PHE A 380     -17.016 -21.828  13.281  1.00 95.62           O
ATOM   3031  CG  PHE A 380     -12.930 -23.672  13.992  1.00 95.62           C
ATOM   3032  CD1 PHE A 380     -12.578 -24.359  15.148  1.00 95.62           C
ATOM   3033  CD2 PHE A 380     -12.133 -23.812  12.867  1.00 95.62           C
ATOM   3034  CE1 PHE A 380     -11.438 -25.156  15.188  1.00 95.62           C
ATOM   3035  CE2 PHE A 380     -11.000 -24.625  12.891  1.00 95.62           C
ATOM   3036  CZ  PHE A 380     -10.648 -25.281  14.055  1.00 95.62           C
ATOM   3037  N   THR A 381     -17.281 -22.906  15.250  1.00 95.25           N
ATOM   3038  CA  THR A 381     -18.406 -22.109  15.742  1.00 95.25           C
ATOM   3039  C   THR A 381     -18.109 -21.531  17.125  1.00 95.25           C
ATOM   3040  CB  THR A 381     -19.688 -22.953  15.805  1.00 95.25           C
ATOM   3041  O   THR A 381     -17.047 -21.812  17.703  1.00 95.25           O
ATOM   3042  CG2 THR A 381     -19.922 -23.703  14.492  1.00 95.25           C
ATOM   3043  OG1 THR A 381     -19.594 -23.891  16.875  1.00 95.25           O
ATOM   3044  N   ASN A 382     -19.000 -20.609  17.594  1.00 95.12           N
ATOM   3045  CA  ASN A 382     -18.812 -20.031  18.922  1.00 95.12           C
ATOM   3046  C   ASN A 382     -18.781 -21.125  20.000  1.00 95.12           C
ATOM   3047  CB  ASN A 382     -19.906 -19.016  19.234  1.00 95.12           C
ATOM   3048  O   ASN A 382     -18.250 -20.906  21.094  1.00 95.12           O
ATOM   3049  CG  ASN A 382     -19.875 -17.828  18.281  1.00 95.12           C
ATOM   3050  ND2 ASN A 382     -19.172 -16.766  18.672  1.00 95.12           N
ATOM   3051  OD1 ASN A 382     -20.469 -17.859  17.203  1.00 95.12           O
ATOM   3052  N   ASP A 383     -19.219 -22.453  19.781  1.00 93.19           N
ATOM   3053  CA  ASP A 383     -19.359 -23.516  20.766  1.00 93.19           C
ATOM   3054  C   ASP A 383     -18.078 -24.344  20.859  1.00 93.19           C
ATOM   3055  CB  ASP A 383     -20.547 -24.422  20.422  1.00 93.19           C
ATOM   3056  O   ASP A 383     -17.844 -25.016  21.875  1.00 93.19           O
ATOM   3057  CG  ASP A 383     -21.859 -23.906  20.953  1.00 93.19           C
ATOM   3058  OD1 ASP A 383     -21.891 -22.797  21.531  1.00 93.19           O
ATOM   3059  OD2 ASP A 383     -22.875 -24.625  20.812  1.00 93.19           O
ATOM   3060  N   ASN A 384     -17.250 -24.328  19.766  1.00 93.62           N
ATOM   3061  CA  ASN A 384     -16.031 -25.109  19.844  1.00 93.62           C
ATOM   3062  C   ASN A 384     -14.797 -24.203  19.875  1.00 93.62           C
ATOM   3063  CB  ASN A 384     -15.945 -26.078  18.672  1.00 93.62           C
ATOM   3064  O   ASN A 384     -13.680 -24.672  19.609  1.00 93.62           O
ATOM   3065  CG  ASN A 384     -15.812 -25.375  17.328  1.00 93.62           C
ATOM   3066  ND2 ASN A 384     -15.375 -26.109  16.312  1.00 93.62           N
ATOM   3067  OD1 ASN A 384     -16.109 -24.188  17.219  1.00 93.62           O
ATOM   3068  N   VAL A 385     -15.000 -22.984  20.109  1.00 95.50           N
```

ATOM	3069	CA	VAL	A	385	-13.930	-22.016	20.312	1.00	95.50	C
ATOM	3070	C	VAL	A	385	-13.914	-21.578	21.781	1.00	95.50	C
ATOM	3071	CB	VAL	A	385	-14.094	-20.781	19.391	1.00	95.50	C
ATOM	3072	O	VAL	A	385	-14.969	-21.391	22.391	1.00	95.50	O
ATOM	3073	CG1	VAL	A	385	-13.172	-19.656	19.828	1.00	95.50	C
ATOM	3074	CG2	VAL	A	385	-13.828	-21.172	17.938	1.00	95.50	C
ATOM	3075	N	VAL	A	386	-12.758	-21.516	22.391	1.00	93.31	N
ATOM	3076	CA	VAL	A	386	-12.594	-21.172	23.797	1.00	93.31	C
ATOM	3077	C	VAL	A	386	-13.297	-19.844	24.078	1.00	93.31	C
ATOM	3078	CB	VAL	A	386	-11.102	-21.078	24.188	1.00	93.31	C
ATOM	3079	O	VAL	A	386	-13.023	-18.828	23.422	1.00	93.31	O
ATOM	3080	CG1	VAL	A	386	-10.953	-20.609	25.625	1.00	93.31	C
ATOM	3081	CG2	VAL	A	386	-10.406	-22.422	23.969	1.00	93.31	C
ATOM	3082	N	ASN	A	387	-14.352	-19.781	24.906	1.00	92.25	N
ATOM	3083	CA	ASN	A	387	-15.156	-18.625	25.312	1.00	92.25	C
ATOM	3084	C	ASN	A	387	-16.016	-18.125	24.172	1.00	92.25	C
ATOM	3085	CB	ASN	A	387	-14.258	-17.516	25.844	1.00	92.25	C
ATOM	3086	O	ASN	A	387	-16.547	-17.000	24.219	1.00	92.25	O
ATOM	3087	CG	ASN	A	387	-13.547	-17.891	27.125	1.00	92.25	C
ATOM	3088	ND2	ASN	A	387	-12.344	-17.359	27.312	1.00	92.25	N
ATOM	3089	OD1	ASN	A	387	-14.070	-18.656	27.938	1.00	92.25	O
ATOM	3090	N	GLY	A	388	-15.922	-18.812	23.000	1.00	92.12	N
ATOM	3091	CA	GLY	A	388	-16.703	-18.406	21.828	1.00	92.12	C
ATOM	3092	C	GLY	A	388	-16.188	-17.141	21.172	1.00	92.12	C
ATOM	3093	O	GLY	A	388	-16.938	-16.438	20.500	1.00	92.12	O
ATOM	3094	N	ASP	A	389	-14.859	-16.859	21.328	1.00	94.44	N
ATOM	3095	CA	ASP	A	389	-14.281	-15.570	20.953	1.00	94.44	C
ATOM	3096	C	ASP	A	389	-13.469	-15.688	19.656	1.00	94.44	C
ATOM	3097	CB	ASP	A	389	-13.398	-15.023	22.078	1.00	94.44	C
ATOM	3098	O	ASP	A	389	-12.508	-16.453	19.594	1.00	94.44	O
ATOM	3099	CG	ASP	A	389	-14.195	-14.570	23.281	1.00	94.44	C
ATOM	3100	OD1	ASP	A	389	-15.422	-14.359	23.172	1.00	94.44	O
ATOM	3101	OD2	ASP	A	389	-13.594	-14.414	24.375	1.00	94.44	O
ATOM	3102	N	PHE	A	390	-13.914	-14.945	18.719	1.00	96.94	N
ATOM	3103	CA	PHE	A	390	-13.148	-14.711	17.500	1.00	96.94	C
ATOM	3104	C	PHE	A	390	-12.609	-13.289	17.453	1.00	96.94	C
ATOM	3105	CB	PHE	A	390	-14.008	-14.984	16.250	1.00	96.94	C
ATOM	3106	O	PHE	A	390	-13.352	-12.336	17.703	1.00	96.94	O
ATOM	3107	CG	PHE	A	390	-14.375	-16.422	16.078	1.00	96.94	C
ATOM	3108	CD1	PHE	A	390	-13.484	-17.328	15.500	1.00	96.94	C
ATOM	3109	CD2	PHE	A	390	-15.617	-16.891	16.484	1.00	96.94	C
ATOM	3110	CE1	PHE	A	390	-13.828	-18.672	15.328	1.00	96.94	C
ATOM	3111	CE2	PHE	A	390	-15.969	-18.234	16.328	1.00	96.94	C
ATOM	3112	CZ	PHE	A	390	-15.070	-19.109	15.750	1.00	96.94	C
ATOM	3113	N	SER	A	391	-11.375	-13.141	17.188	1.00	96.38	N
ATOM	3114	CA	SER	A	391	-10.781	-11.812	17.062	1.00	96.38	C
ATOM	3115	C	SER	A	391	-10.359	-11.531	15.625	1.00	96.38	C
ATOM	3116	CB	SER	A	391	-9.570	-11.680	17.984	1.00	96.38	C
ATOM	3117	O	SER	A	391	-9.594	-12.297	15.031	1.00	96.38	O
ATOM	3118	OG	SER	A	391	-8.969	-10.406	17.844	1.00	96.38	O
ATOM	3119	N	TYR	A	392	-10.875	-10.398	15.055	1.00	95.50	N
ATOM	3120	CA	TYR	A	392	-10.617	-10.070	13.656	1.00	95.50	C
ATOM	3121	C	TYR	A	392	-9.680	-8.867	13.539	1.00	95.50	C
ATOM	3122	CB	TYR	A	392	-11.930	-9.773	12.922	1.00	95.50	C
ATOM	3123	O	TYR	A	392	-9.805	-7.902	14.305	1.00	95.50	O
ATOM	3124	CG	TYR	A	392	-12.891	-10.938	12.922	1.00	95.50	C
ATOM	3125	CD1	TYR	A	392	-12.812	-11.938	11.953	1.00	95.50	C
ATOM	3126	CD2	TYR	A	392	-13.891	-11.039	13.883	1.00	95.50	C
ATOM	3127	CE1	TYR	A	392	-13.695	-13.008	11.945	1.00	95.50	C
ATOM	3128	CE2	TYR	A	392	-14.781	-12.109	13.883	1.00	95.50	C
ATOM	3129	OH	TYR	A	392	-15.555	-14.141	12.906	1.00	95.50	O
ATOM	3130	CZ	TYR	A	392	-14.680	-13.086	12.914	1.00	95.50	C
ATOM	3131	N	THR	A	393	-8.773	-8.984	12.641	1.00	94.19	N
ATOM	3132	CA	THR	A	393	-7.848	-7.910	12.305	1.00	94.19	C

ATOM	3133	C	THR	A	393	-7.961	-7.539	10.828	1.00	94.19	C
ATOM	3134	CB	THR	A	393	-6.391	-8.305	12.625	1.00	94.19	C
ATOM	3135	O	THR	A	393	-7.898	-8.414	9.953	1.00	94.19	O
ATOM	3136	CG2	THR	A	393	-5.445	-7.125	12.422	1.00	94.19	C
ATOM	3137	OG1	THR	A	393	-6.312	-8.734	13.992	1.00	94.19	O
ATOM	3138	N	PHE	A	394	-8.234	-6.250	10.578	1.00	90.25	N
ATOM	3139	CA	PHE	A	394	-8.398	-5.777	9.211	1.00	90.25	C
ATOM	3140	C	PHE	A	394	-7.172	-5.000	8.758	1.00	90.25	C
ATOM	3141	CB	PHE	A	394	-9.648	-4.895	9.102	1.00	90.25	C
ATOM	3142	O	PHE	A	394	-6.500	-4.359	9.562	1.00	90.25	O
ATOM	3143	CG	PHE	A	394	-10.914	-5.562	9.578	1.00	90.25	C
ATOM	3144	CD1	PHE	A	394	-11.680	-6.336	8.711	1.00	90.25	C
ATOM	3145	CD2	PHE	A	394	-11.328	-5.422	10.891	1.00	90.25	C
ATOM	3146	CE1	PHE	A	394	-12.844	-6.953	9.148	1.00	90.25	C
ATOM	3147	CE2	PHE	A	394	-12.500	-6.039	11.336	1.00	90.25	C
ATOM	3148	CZ	PHE	A	394	-13.250	-6.805	10.461	1.00	90.25	C
ATOM	3149	N	ALA	A	395	-6.879	-5.250	7.445	1.00	87.69	N
ATOM	3150	CA	ALA	A	395	-5.727	-4.555	6.875	1.00	87.69	C
ATOM	3151	C	ALA	A	395	-6.023	-3.074	6.664	1.00	87.69	C
ATOM	3152	CB	ALA	A	395	-5.309	-5.203	5.559	1.00	87.69	C
ATOM	3153	O	ALA	A	395	-7.188	-2.682	6.535	1.00	87.69	O
ATOM	3154	N	SER	A	396	-5.008	-2.254	6.719	1.00	88.38	N
ATOM	3155	CA	SER	A	396	-5.137	-0.820	6.477	1.00	88.38	C
ATOM	3156	C	SER	A	396	-5.555	-0.540	5.035	1.00	88.38	C
ATOM	3157	CB	SER	A	396	-3.818	-0.105	6.777	1.00	88.38	C
ATOM	3158	O	SER	A	396	-5.207	-1.291	4.125	1.00	88.38	O
ATOM	3159	OG	SER	A	396	-3.867	1.243	6.344	1.00	88.38	O
ATOM	3160	N	GLU	A	397	-6.383	0.464	4.867	1.00	85.94	N
ATOM	3161	CA	GLU	A	397	-6.824	0.877	3.535	1.00	85.94	C
ATOM	3162	C	GLU	A	397	-5.633	1.121	2.613	1.00	85.94	C
ATOM	3163	CB	GLU	A	397	-7.688	2.137	3.621	1.00	85.94	C
ATOM	3164	O	GLU	A	397	-5.691	0.812	1.422	1.00	85.94	O
ATOM	3165	CG	GLU	A	397	-8.344	2.521	2.305	1.00	85.94	C
ATOM	3166	CD	GLU	A	397	-9.234	3.754	2.412	1.00	85.94	C
ATOM	3167	OE1	GLU	A	397	-9.750	4.223	1.374	1.00	85.94	O
ATOM	3168	OE2	GLU	A	397	-9.406	4.254	3.547	1.00	85.94	O
ATOM	3169	N	LYS	A	398	-4.559	1.503	2.994	1.00	85.94	N
ATOM	3170	CA	LYS	A	398	-3.371	1.812	2.201	1.00	85.94	C
ATOM	3171	C	LYS	A	398	-2.680	0.537	1.727	1.00	85.94	C
ATOM	3172	CB	LYS	A	398	-2.395	2.668	3.010	1.00	85.94	C
ATOM	3173	O	LYS	A	398	-1.945	0.555	0.736	1.00	85.94	O
ATOM	3174	CG	LYS	A	398	-2.877	4.086	3.262	1.00	85.94	C
ATOM	3175	CD	LYS	A	398	-1.797	4.938	3.916	1.00	85.94	C
ATOM	3176	CE	LYS	A	398	-2.275	6.363	4.156	1.00	85.94	C
ATOM	3177	NZ	LYS	A	398	-1.220	7.199	4.801	1.00	85.94	N
ATOM	3178	N	SER	A	399	-2.865	-0.506	2.447	1.00	88.31	N
ATOM	3179	CA	SER	A	399	-2.238	-1.771	2.082	1.00	88.31	C
ATOM	3180	C	SER	A	399	-3.121	-2.574	1.133	1.00	88.31	C
ATOM	3181	CB	SER	A	399	-1.935	-2.600	3.332	1.00	88.31	C
ATOM	3182	O	SER	A	399	-2.744	-3.664	0.698	1.00	88.31	O
ATOM	3183	OG	SER	A	399	-3.119	-2.850	4.070	1.00	88.31	O
ATOM	3184	N	MET	A	400	-4.301	-1.938	0.873	1.00	91.69	N
ATOM	3185	CA	MET	A	400	-5.168	-2.582	-0.109	1.00	91.69	C
ATOM	3186	C	MET	A	400	-4.801	-2.152	-1.525	1.00	91.69	C
ATOM	3187	CB	MET	A	400	-6.633	-2.254	0.172	1.00	91.69	C
ATOM	3188	O	MET	A	400	-5.262	-1.115	-2.004	1.00	91.69	O
ATOM	3189	CG	MET	A	400	-7.113	-2.717	1.538	1.00	91.69	C
ATOM	3190	SD	MET	A	400	-7.082	-4.543	1.712	1.00	91.69	S
ATOM	3191	CE	MET	A	400	-8.859	-4.910	1.581	1.00	91.69	C
ATOM	3192	N	TYR	A	401	-3.996	-2.971	-2.227	1.00	95.19	N
ATOM	3193	CA	TYR	A	401	-3.471	-2.654	-3.551	1.00	95.19	C
ATOM	3194	C	TYR	A	401	-4.570	-2.719	-4.602	1.00	95.19	C
ATOM	3195	CB	TYR	A	401	-2.334	-3.611	-3.922	1.00	95.19	C
ATOM	3196	O	TYR	A	401	-5.383	-3.646	-4.605	1.00	95.19	O

ATOM	3197	CG	TYR	A	401	-1.174	-3.580	-2.957	1.00	95.19	C
ATOM	3198	CD1	TYR	A	401	-0.526	-2.385	-2.654	1.00	95.19	C
ATOM	3199	CD2	TYR	A	401	-0.722	-4.746	-2.348	1.00	95.19	C
ATOM	3200	CE1	TYR	A	401	0.545	-2.352	-1.768	1.00	95.19	C
ATOM	3201	CE2	TYR	A	401	0.348	-4.727	-1.459	1.00	95.19	C
ATOM	3202	OH	TYR	A	401	2.033	-3.498	-0.297	1.00	95.19	O
ATOM	3203	CZ	TYR	A	401	0.974	-3.525	-1.176	1.00	95.19	C
ATOM	3204	N	THR	A	402	-4.574	-1.743	-5.461	1.00	95.31	N
ATOM	3205	CA	THR	A	402	-5.609	-1.625	-6.484	1.00	95.31	C
ATOM	3206	C	THR	A	402	-5.012	-1.794	-7.879	1.00	95.31	C
ATOM	3207	CB	THR	A	402	-6.332	-0.270	-6.395	1.00	95.31	C
ATOM	3208	O	THR	A	402	-5.734	-2.047	-8.844	1.00	95.31	O
ATOM	3209	CG2	THR	A	402	-7.078	-0.132	-5.070	1.00	95.31	C
ATOM	3210	OG1	THR	A	402	-5.371	0.788	-6.500	1.00	95.31	O
ATOM	3211	N	THR	A	403	-3.717	-1.629	-7.918	1.00	96.38	N
ATOM	3212	CA	THR	A	403	-2.988	-1.790	-9.172	1.00	96.38	C
ATOM	3213	C	THR	A	403	-1.720	-2.611	-8.953	1.00	96.38	C
ATOM	3214	CB	THR	A	403	-2.623	-0.426	-9.781	1.00	96.38	C
ATOM	3215	O	THR	A	403	-1.032	-2.447	-7.945	1.00	96.38	O
ATOM	3216	CG2	THR	A	403	-1.927	-0.597	-11.133	1.00	96.38	C
ATOM	3217	OG1	THR	A	403	-3.818	0.342	-9.969	1.00	96.38	O
ATOM	3218	N	CYS	A	404	-1.388	-3.566	-9.898	1.00	97.31	N
ATOM	3219	CA	CYS	A	404	-0.162	-4.355	-9.844	1.00	97.31	C
ATOM	3220	C	CYS	A	404	0.574	-4.309	-11.180	1.00	97.31	C
ATOM	3221	CB	CYS	A	404	-0.471	-5.805	-9.477	1.00	97.31	C
ATOM	3222	O	CYS	A	404	0.021	-4.695	-12.211	1.00	97.31	O
ATOM	3223	SG	CYS	A	404	0.999	-6.836	-9.273	1.00	97.31	S
ATOM	3224	N	ASN	A	405	1.826	-3.832	-11.133	1.00	97.88	N
ATOM	3225	CA	ASN	A	405	2.709	-3.893	-12.297	1.00	97.88	C
ATOM	3226	C	ASN	A	405	3.520	-5.184	-12.312	1.00	97.88	C
ATOM	3227	CB	ASN	A	405	3.641	-2.680	-12.320	1.00	97.88	C
ATOM	3228	O	ASN	A	405	4.312	-5.441	-11.406	1.00	97.88	O
ATOM	3229	CG	ASN	A	405	2.895	-1.374	-12.516	1.00	97.88	C
ATOM	3230	ND2	ASN	A	405	3.414	-0.303	-11.922	1.00	97.88	N
ATOM	3231	OD1	ASN	A	405	1.859	-1.328	-13.180	1.00	97.88	O
ATOM	3232	N	VAL	A	406	3.246	-6.047	-13.336	1.00	97.88	N
ATOM	3233	CA	VAL	A	406	3.891	-7.352	-13.406	1.00	97.88	C
ATOM	3234	C	VAL	A	406	4.934	-7.352	-14.523	1.00	97.88	C
ATOM	3235	CB	VAL	A	406	2.861	-8.484	-13.641	1.00	97.88	C
ATOM	3236	O	VAL	A	406	4.594	-7.250	-15.703	1.00	97.88	O
ATOM	3237	CG1	VAL	A	406	3.535	-9.852	-13.562	1.00	97.88	C
ATOM	3238	CG2	VAL	A	406	1.722	-8.383	-12.625	1.00	97.88	C
ATOM	3239	N	MET	A	407	6.219	-7.434	-14.102	1.00	97.75	N
ATOM	3240	CA	MET	A	407	7.305	-7.523	-15.070	1.00	97.75	C
ATOM	3241	C	MET	A	407	7.453	-8.953	-15.586	1.00	97.75	C
ATOM	3242	CB	MET	A	407	8.625	-7.055	-14.453	1.00	97.75	C
ATOM	3243	O	MET	A	407	7.410	-9.906	-14.812	1.00	97.75	O
ATOM	3244	CG	MET	A	407	8.773	-5.543	-14.414	1.00	97.75	C
ATOM	3245	SD	MET	A	407	10.414	-5.016	-13.773	1.00	97.75	S
ATOM	3246	CE	MET	A	407	10.188	-3.217	-13.750	1.00	97.75	C
ATOM	3247	N	PHE	A	408	7.496	-9.164	-16.922	1.00	96.88	N
ATOM	3248	CA	PHE	A	408	7.672	-10.453	-17.562	1.00	96.88	C
ATOM	3249	C	PHE	A	408	8.594	-10.336	-18.781	1.00	96.88	C
ATOM	3250	CB	PHE	A	408	6.316	-11.031	-18.000	1.00	96.88	C
ATOM	3251	O	PHE	A	408	8.906	-9.227	-19.219	1.00	96.88	O
ATOM	3252	CG	PHE	A	408	5.719	-10.352	-19.203	1.00	96.88	C
ATOM	3253	CD1	PHE	A	408	5.234	-9.055	-19.109	1.00	96.88	C
ATOM	3254	CD2	PHE	A	408	5.645	-11.016	-20.422	1.00	96.88	C
ATOM	3255	CE1	PHE	A	408	4.684	-8.430	-20.234	1.00	96.88	C
ATOM	3256	CE2	PHE	A	408	5.094	-10.398	-21.531	1.00	96.88	C
ATOM	3257	CZ	PHE	A	408	4.613	-9.102	-21.438	1.00	96.88	C
ATOM	3258	N	ASP	A	409	9.094	-11.430	-19.219	1.00	95.75	N
ATOM	3259	CA	ASP	A	409	9.867	-11.469	-20.469	1.00	95.75	C
ATOM	3260	C	ASP	A	409	8.953	-11.672	-21.672	1.00	95.75	C

ATOM	3261	CB	ASP	A	409	10.922	-12.578	-20.406	1.00	95.75	C
ATOM	3262	O	ASP	A	409	8.305	-12.719	-21.797	1.00	95.75	O
ATOM	3263	CG	ASP	A	409	12.000	-12.320	-19.375	1.00	95.75	C
ATOM	3264	OD1	ASP	A	409	12.242	-11.141	-19.016	1.00	95.75	O
ATOM	3265	OD2	ASP	A	409	12.609	-13.305	-18.891	1.00	95.75	O
ATOM	3266	N	ASP	A	410	8.906	-10.609	-22.500	1.00	94.75	N
ATOM	3267	CA	ASP	A	410	7.953	-10.547	-23.609	1.00	94.75	C
ATOM	3268	C	ASP	A	410	8.484	-11.305	-24.828	1.00	94.75	C
ATOM	3269	CB	ASP	A	410	7.652	-9.094	-23.969	1.00	94.75	C
ATOM	3270	O	ASP	A	410	9.445	-10.867	-25.469	1.00	94.75	O
ATOM	3271	CG	ASP	A	410	6.523	-8.961	-24.984	1.00	94.75	C
ATOM	3272	OD1	ASP	A	410	6.035	-9.992	-25.500	1.00	94.75	O
ATOM	3273	OD2	ASP	A	410	6.117	-7.812	-25.266	1.00	94.75	O
ATOM	3274	N	GLU	A	411	7.824	-12.383	-25.078	1.00	89.88	N
ATOM	3275	CA	GLU	A	411	8.227	-13.211	-26.219	1.00	89.88	C
ATOM	3276	C	GLU	A	411	8.070	-12.453	-27.531	1.00	89.88	C
ATOM	3277	CB	GLU	A	411	7.402	-14.500	-26.250	1.00	89.88	C
ATOM	3278	O	GLU	A	411	8.867	-12.648	-28.453	1.00	89.88	O
ATOM	3279	CG	GLU	A	411	7.754	-15.422	-27.406	1.00	89.88	C
ATOM	3280	CD	GLU	A	411	6.844	-16.641	-27.516	1.00	89.88	C
ATOM	3281	OE1	GLU	A	411	7.113	-17.531	-28.344	1.00	89.88	O
ATOM	3282	OE2	GLU	A	411	5.855	-16.703	-26.750	1.00	89.88	O
ATOM	3283	N	GLN	A	412	7.121	-11.562	-27.672	1.00	89.19	N
ATOM	3284	CA	GLN	A	412	6.859	-10.797	-28.891	1.00	89.19	C
ATOM	3285	C	GLN	A	412	7.922	-9.719	-29.094	1.00	89.19	C
ATOM	3286	CB	GLN	A	412	5.469	-10.156	-28.828	1.00	89.19	C
ATOM	3287	O	GLN	A	412	8.070	-9.195	-30.203	1.00	89.19	O
ATOM	3288	CG	GLN	A	412	4.328	-11.164	-28.859	1.00	89.19	C
ATOM	3289	CD	GLN	A	412	2.963	-10.508	-28.781	1.00	89.19	C
ATOM	3290	NE2	GLN	A	412	1.944	-11.195	-29.281	1.00	89.19	N
ATOM	3291	OE1	GLN	A	412	2.826	-9.391	-28.266	1.00	89.19	O
ATOM	3292	N	ASN	A	413	8.641	-9.438	-27.969	1.00	90.69	N
ATOM	3293	CA	ASN	A	413	9.719	-8.461	-28.016	1.00	90.69	C
ATOM	3294	C	ASN	A	413	11.070	-9.102	-27.719	1.00	90.69	C
ATOM	3295	CB	ASN	A	413	9.453	-7.316	-27.031	1.00	90.69	C
ATOM	3296	O	ASN	A	413	11.883	-8.547	-26.969	1.00	90.69	O
ATOM	3297	CG	ASN	A	413	10.336	-6.109	-27.281	1.00	90.69	C
ATOM	3298	ND2	ASN	A	413	10.500	-5.270	-26.266	1.00	90.69	N
ATOM	3299	OD1	ASN	A	413	10.867	-5.934	-28.391	1.00	90.69	O
ATOM	3300	N	MET	A	414	11.266	-10.305	-28.219	1.00	88.62	N
ATOM	3301	CA	MET	A	414	12.516	-11.047	-28.156	1.00	88.62	C
ATOM	3302	C	MET	A	414	12.938	-11.281	-26.703	1.00	88.62	C
ATOM	3303	CB	MET	A	414	13.617	-10.312	-28.906	1.00	88.62	C
ATOM	3304	O	MET	A	414	14.117	-11.141	-26.359	1.00	88.62	O
ATOM	3305	CG	MET	A	414	13.367	-10.203	-30.406	1.00	88.62	C
ATOM	3306	SD	MET	A	414	13.227	-11.844	-31.219	1.00	88.62	S
ATOM	3307	CE	MET	A	414	12.938	-11.320	-32.938	1.00	88.62	C
ATOM	3308	N	TYR	A	415	11.945	-11.398	-25.766	1.00	90.62	N
ATOM	3309	CA	TYR	A	415	12.125	-11.773	-24.359	1.00	90.62	C
ATOM	3310	C	TYR	A	415	12.766	-10.641	-23.578	1.00	90.62	C
ATOM	3311	CB	TYR	A	415	12.984	-13.039	-24.250	1.00	90.62	C
ATOM	3312	O	TYR	A	415	13.469	-10.883	-22.594	1.00	90.62	O
ATOM	3313	CG	TYR	A	415	12.336	-14.266	-24.844	1.00	90.62	C
ATOM	3314	CD1	TYR	A	415	11.430	-15.023	-24.094	1.00	90.62	C
ATOM	3315	CD2	TYR	A	415	12.625	-14.672	-26.141	1.00	90.62	C
ATOM	3316	CE1	TYR	A	415	10.828	-16.156	-24.641	1.00	90.62	C
ATOM	3317	CE2	TYR	A	415	12.031	-15.805	-26.688	1.00	90.62	C
ATOM	3318	OH	TYR	A	415	10.547	-17.656	-26.469	1.00	90.62	O
ATOM	3319	CZ	TYR	A	415	11.133	-16.547	-25.938	1.00	90.62	C
ATOM	3320	N	GLN	A	416	12.688	-9.414	-24.094	1.00	92.31	N
ATOM	3321	CA	GLN	A	416	13.039	-8.234	-23.312	1.00	92.31	C
ATOM	3322	C	GLN	A	416	12.016	-8.000	-22.188	1.00	92.31	C
ATOM	3323	CB	GLN	A	416	13.133	-6.996	-24.203	1.00	92.31	C
ATOM	3324	O	GLN	A	416	10.844	-8.336	-22.344	1.00	92.31	O

```
ATOM   3325  CG  GLN A 416      14.344  -6.996 -25.125  1.00 92.31           C
ATOM   3326  CD  GLN A 416      14.453  -5.730 -25.953  1.00 92.31           C
ATOM   3327  NE2 GLN A 416      15.539  -5.605 -26.703  1.00 92.31           N
ATOM   3328  OE1 GLN A 416      13.570  -4.871 -25.906  1.00 92.31           O
ATOM   3329  N   GLN A 417      12.508  -7.449 -21.031  1.00 94.56           N
ATOM   3330  CA  GLN A 417      11.625  -7.234 -19.906  1.00 94.56           C
ATOM   3331  C   GLN A 417      10.555  -6.191 -20.219  1.00 94.56           C
ATOM   3332  CB  GLN A 417      12.422  -6.805 -18.672  1.00 94.56           C
ATOM   3333  O   GLN A 417      10.859  -5.141 -20.797  1.00 94.56           O
ATOM   3334  CG  GLN A 417      13.305  -7.902 -18.094  1.00 94.56           C
ATOM   3335  CD  GLN A 417      14.031  -7.473 -16.844  1.00 94.56           C
ATOM   3336  NE2 GLN A 417      14.570  -8.438 -16.094  1.00 94.56           N
ATOM   3337  OE1 GLN A 417      14.117  -6.277 -16.531  1.00 94.56           O
ATOM   3338  N   ASP A 418       9.312  -6.516 -20.094  1.00 97.44           N
ATOM   3339  CA  ASP A 418       8.148  -5.652 -20.250  1.00 97.44           C
ATOM   3340  C   ASP A 418       7.246  -5.719 -19.016  1.00 97.44           C
ATOM   3341  CB  ASP A 418       7.359  -6.035 -21.500  1.00 97.44           C
ATOM   3342  O   ASP A 418       7.367  -6.629 -18.203  1.00 97.44           O
ATOM   3343  CG  ASP A 418       6.453  -4.922 -22.000  1.00 97.44           C
ATOM   3344  OD1 ASP A 418       6.469  -3.818 -21.406  1.00 97.44           O
ATOM   3345  OD2 ASP A 418       5.715  -5.152 -22.984  1.00 97.44           O
ATOM   3346  N   VAL A 419       6.418  -4.691 -18.828  1.00 97.50           N
ATOM   3347  CA  VAL A 419       5.551  -4.617 -17.656  1.00 97.50           C
ATOM   3348  C   VAL A 419       4.090  -4.672 -18.094  1.00 97.50           C
ATOM   3349  CB  VAL A 419       5.816  -3.334 -16.844  1.00 97.50           C
ATOM   3350  O   VAL A 419       3.678  -3.945 -19.000  1.00 97.50           O
ATOM   3351  CG1 VAL A 419       4.867  -3.254 -15.648  1.00 97.50           C
ATOM   3352  CG2 VAL A 419       7.270  -3.287 -16.375  1.00 97.50           C
ATOM   3353  N   GLU A 420       3.303  -5.641 -17.484  1.00 96.56           N
ATOM   3354  CA  GLU A 420       1.854  -5.719 -17.656  1.00 96.56           C
ATOM   3355  C   GLU A 420       1.124  -5.086 -16.484  1.00 96.56           C
ATOM   3356  CB  GLU A 420       1.412  -7.176 -17.828  1.00 96.56           C
ATOM   3357  O   GLU A 420       1.134  -5.629 -15.367  1.00 96.56           O
ATOM   3358  CG  GLU A 420      -0.096  -7.352 -17.922  1.00 96.56           C
ATOM   3359  CD  GLU A 420      -0.718  -6.637 -19.109  1.00 96.56           C
ATOM   3360  OE1 GLU A 420      -1.937  -6.348 -19.078  1.00 96.56           O
ATOM   3361  OE2 GLU A 420       0.018  -6.363 -20.078  1.00 96.56           O
ATOM   3362  N   PRO A 421       0.499  -3.869 -16.688  1.00 96.88           N
ATOM   3363  CA  PRO A 421      -0.274  -3.246 -15.617  1.00 96.88           C
ATOM   3364  C   PRO A 421      -1.648  -3.883 -15.430  1.00 96.88           C
ATOM   3365  CB  PRO A 421      -0.405  -1.791 -16.078  1.00 96.88           C
ATOM   3366  O   PRO A 421      -2.387  -4.062 -16.406  1.00 96.88           O
ATOM   3367  CG  PRO A 421      -0.348  -1.854 -17.562  1.00 96.88           C
ATOM   3368  CD  PRO A 421       0.483  -3.039 -17.969  1.00 96.88           C
ATOM   3369  N   VAL A 422      -1.972  -4.320 -14.242  1.00 95.81           N
ATOM   3370  CA  VAL A 422      -3.248  -4.941 -13.914  1.00 95.81           C
ATOM   3371  C   VAL A 422      -3.996  -4.086 -12.898  1.00 95.81           C
ATOM   3372  CB  VAL A 422      -3.057  -6.375 -13.367  1.00 95.81           C
ATOM   3373  O   VAL A 422      -3.436  -3.697 -11.875  1.00 95.81           O
ATOM   3374  CG1 VAL A 422      -4.406  -7.023 -13.062  1.00 95.81           C
ATOM   3375  CG2 VAL A 422      -2.264  -7.223 -14.359  1.00 95.81           C
ATOM   3376  N   PHE A 423      -5.305  -3.768 -13.195  1.00 93.69           N
ATOM   3377  CA  PHE A 423      -6.102  -2.912 -12.328  1.00 93.69           C
ATOM   3378  C   PHE A 423      -7.367  -3.633 -11.875  1.00 93.69           C
ATOM   3379  CB  PHE A 423      -6.469  -1.607 -13.039  1.00 93.69           C
ATOM   3380  O   PHE A 423      -7.922  -4.449 -12.609  1.00 93.69           O
ATOM   3381  CG  PHE A 423      -5.293  -0.899 -13.656  1.00 93.69           C
ATOM   3382  CD1 PHE A 423      -4.570   0.037 -12.930  1.00 93.69           C
ATOM   3383  CD2 PHE A 423      -4.910  -1.170 -14.961  1.00 93.69           C
ATOM   3384  CE1 PHE A 423      -3.480   0.695 -13.492  1.00 93.69           C
ATOM   3385  CE2 PHE A 423      -3.822  -0.518 -15.539  1.00 93.69           C
ATOM   3386  CZ  PHE A 423      -3.109   0.415 -14.797  1.00 93.69           C
ATOM   3387  N   ASP A 424      -7.746  -3.371 -10.625  1.00 92.50           N
ATOM   3388  CA  ASP A 424      -9.055  -3.768 -10.109  1.00 92.50           C
```

ATOM	3389	C	ASP	A	424	-9.961	-2.555	-9.914	1.00	92.50	C
ATOM	3390	CB	ASP	A	424	-8.898	-4.527	-8.789	1.00	92.50	C
ATOM	3391	O	ASP	A	424	-9.789	-1.787	-8.969	1.00	92.50	O
ATOM	3392	CG	ASP	A	424	-10.188	-5.191	-8.336	1.00	92.50	C
ATOM	3393	OD1	ASP	A	424	-11.219	-4.496	-8.188	1.00	92.50	O
ATOM	3394	OD2	ASP	A	424	-10.172	-6.422	-8.117	1.00	92.50	O
ATOM	3395	N	ARG	A	425	-10.898	-2.387	-10.766	1.00	90.38	N
ATOM	3396	CA	ARG	A	425	-11.742	-1.196	-10.820	1.00	90.38	C
ATOM	3397	C	ARG	A	425	-12.547	-1.032	-9.539	1.00	90.38	C
ATOM	3398	CB	ARG	A	425	-12.688	-1.263	-12.023	1.00	90.38	C
ATOM	3399	O	ARG	A	425	-12.633	0.069	-8.992	1.00	90.38	O
ATOM	3400	CG	ARG	A	425	-13.555	-0.029	-12.195	1.00	90.38	C
ATOM	3401	CD	ARG	A	425	-14.422	-0.118	-13.438	1.00	90.38	C
ATOM	3402	NE	ARG	A	425	-15.844	-0.036	-13.109	1.00	90.38	N
ATOM	3403	NH1	ARG	A	425	-16.547	0.250	-15.297	1.00	90.38	N
ATOM	3404	NH2	ARG	A	425	-18.078	0.197	-13.594	1.00	90.38	N
ATOM	3405	CZ	ARG	A	425	-16.812	0.137	-14.000	1.00	90.38	C
ATOM	3406	N	GLU	A	426	-13.156	-2.137	-9.094	1.00	86.12	N
ATOM	3407	CA	GLU	A	426	-13.977	-2.092	-7.887	1.00	86.12	C
ATOM	3408	C	GLU	A	426	-13.156	-1.669	-6.676	1.00	86.12	C
ATOM	3409	CB	GLU	A	426	-14.633	-3.451	-7.633	1.00	86.12	C
ATOM	3410	O	GLU	A	426	-13.594	-0.841	-5.875	1.00	86.12	O
ATOM	3411	CG	GLU	A	426	-15.617	-3.451	-6.477	1.00	86.12	C
ATOM	3412	CD	GLU	A	426	-16.281	-4.801	-6.246	1.00	86.12	C
ATOM	3413	OE1	GLU	A	426	-17.094	-4.930	-5.305	1.00	86.12	O
ATOM	3414	OE2	GLU	A	426	-15.977	-5.742	-7.012	1.00	86.12	O
ATOM	3415	N	ALA	A	427	-12.023	-2.172	-6.574	1.00	89.75	N
ATOM	3416	CA	ALA	A	427	-11.133	-1.831	-5.469	1.00	89.75	C
ATOM	3417	C	ALA	A	427	-10.617	-0.399	-5.602	1.00	89.75	C
ATOM	3418	CB	ALA	A	427	-9.961	-2.809	-5.410	1.00	89.75	C
ATOM	3419	O	ALA	A	427	-10.469	0.308	-4.602	1.00	89.75	O
ATOM	3420	N	THR	A	428	-10.344	0.026	-6.809	1.00	92.50	N
ATOM	3421	CA	THR	A	428	-9.867	1.381	-7.070	1.00	92.50	C
ATOM	3422	C	THR	A	428	-10.914	2.410	-6.648	1.00	92.50	C
ATOM	3423	CB	THR	A	428	-9.523	1.575	-8.555	1.00	92.50	C
ATOM	3424	O	THR	A	428	-10.578	3.434	-6.051	1.00	92.50	O
ATOM	3425	CG2	THR	A	428	-8.992	2.982	-8.812	1.00	92.50	C
ATOM	3426	OG1	THR	A	428	-8.523	0.622	-8.930	1.00	92.50	O
ATOM	3427	N	LEU	A	429	-12.188	2.166	-6.938	1.00	91.75	N
ATOM	3428	CA	LEU	A	429	-13.281	3.062	-6.562	1.00	91.75	C
ATOM	3429	C	LEU	A	429	-13.391	3.176	-5.043	1.00	91.75	C
ATOM	3430	CB	LEU	A	429	-14.602	2.570	-7.148	1.00	91.75	C
ATOM	3431	O	LEU	A	429	-13.672	4.254	-4.520	1.00	91.75	O
ATOM	3432	CG	LEU	A	429	-14.789	2.762	-8.656	1.00	91.75	C
ATOM	3433	CD1	LEU	A	429	-16.031	2.020	-9.133	1.00	91.75	C
ATOM	3434	CD2	LEU	A	429	-14.883	4.246	-9.000	1.00	91.75	C
ATOM	3435	N	ARG	A	430	-13.102	2.076	-4.387	1.00	88.75	N
ATOM	3436	CA	ARG	A	430	-13.258	2.008	-2.938	1.00	88.75	C
ATOM	3437	C	ARG	A	430	-12.031	2.564	-2.227	1.00	88.75	C
ATOM	3438	CB	ARG	A	430	-13.516	0.567	-2.490	1.00	88.75	C
ATOM	3439	O	ARG	A	430	-12.156	3.332	-1.269	1.00	88.75	O
ATOM	3440	CG	ARG	A	430	-13.781	0.422	-1.002	1.00	88.75	C
ATOM	3441	CD	ARG	A	430	-14.172	-1.004	-0.635	1.00	88.75	C
ATOM	3442	NE	ARG	A	430	-14.359	-1.158	0.805	1.00	88.75	N
ATOM	3443	NH1	ARG	A	430	-14.969	-3.385	0.696	1.00	88.75	N
ATOM	3444	NH2	ARG	A	430	-14.875	-2.314	2.721	1.00	88.75	N
ATOM	3445	CZ	ARG	A	430	-14.734	-2.285	1.404	1.00	88.75	C
ATOM	3446	N	PHE	A	431	-10.805	2.225	-2.656	1.00	91.50	N
ATOM	3447	CA	PHE	A	431	-9.609	2.488	-1.869	1.00	91.50	C
ATOM	3448	C	PHE	A	431	-8.711	3.506	-2.566	1.00	91.50	C
ATOM	3449	CB	PHE	A	431	-8.828	1.190	-1.620	1.00	91.50	C
ATOM	3450	O	PHE	A	431	-7.695	3.930	-2.014	1.00	91.50	O
ATOM	3451	CG	PHE	A	431	-9.633	0.124	-0.929	1.00	91.50	C
ATOM	3452	CD1	PHE	A	431	-10.109	0.318	0.363	1.00	91.50	C

```
ATOM   3453  CD2 PHE A 431      -9.922  -1.074  -1.572  1.00 91.50           C
ATOM   3454  CE1 PHE A 431     -10.859  -0.667   1.005  1.00 91.50           C
ATOM   3455  CE2 PHE A 431     -10.664  -2.062  -0.937  1.00 91.50           C
ATOM   3456  CZ  PHE A 431     -11.133  -1.858   0.352  1.00 91.50           C
ATOM   3457  N   GLY A 432      -9.094   4.008  -3.715  1.00 91.81           N
ATOM   3458  CA  GLY A 432      -8.195   4.844  -4.492  1.00 91.81           C
ATOM   3459  C   GLY A 432      -7.098   4.055  -5.184  1.00 91.81           C
ATOM   3460  O   GLY A 432      -7.207   2.836  -5.340  1.00 91.81           O
ATOM   3461  N   ASN A 433      -6.047   4.793  -5.637  1.00 92.44           N
ATOM   3462  CA  ASN A 433      -4.969   4.168  -6.395  1.00 92.44           C
ATOM   3463  C   ASN A 433      -3.805   3.770  -5.492  1.00 92.44           C
ATOM   3464  CB  ASN A 433      -4.484   5.098  -7.508  1.00 92.44           C
ATOM   3465  O   ASN A 433      -3.076   4.633  -4.996  1.00 92.44           O
ATOM   3466  CG  ASN A 433      -3.646   4.383  -8.547  1.00 92.44           C
ATOM   3467  ND2 ASN A 433      -3.002   5.145  -9.422  1.00 92.44           N
ATOM   3468  OD1 ASN A 433      -3.580   3.148  -8.570  1.00 92.44           O
ATOM   3469  N   ASN A 434      -3.668   2.496  -5.145  1.00 94.25           N
ATOM   3470  CA  ASN A 434      -2.557   1.910  -4.402  1.00 94.25           C
ATOM   3471  C   ASN A 434      -1.823   0.856  -5.227  1.00 94.25           C
ATOM   3472  CB  ASN A 434      -3.051   1.305  -3.086  1.00 94.25           C
ATOM   3473  O   ASN A 434      -2.359  -0.224  -5.480  1.00 94.25           O
ATOM   3474  CG  ASN A 434      -3.596   2.350  -2.131  1.00 94.25           C
ATOM   3475  ND2 ASN A 434      -4.398   1.911  -1.169  1.00 94.25           N
ATOM   3476  OD1 ASN A 434      -3.297   3.539  -2.258  1.00 94.25           O
ATOM   3477  N   VAL A 435      -0.529   1.110  -5.633  1.00 95.81           N
ATOM   3478  CA  VAL A 435       0.179   0.331  -6.645  1.00 95.81           C
ATOM   3479  C   VAL A 435       1.174  -0.609  -5.969  1.00 95.81           C
ATOM   3480  CB  VAL A 435       0.909   1.243  -7.656  1.00 95.81           C
ATOM   3481  O   VAL A 435       1.818  -0.239  -4.984  1.00 95.81           O
ATOM   3482  CG1 VAL A 435       1.619   0.408  -8.719  1.00 95.81           C
ATOM   3483  CG2 VAL A 435      -0.075   2.217  -8.305  1.00 95.81           C
ATOM   3484  N   THR A 436       1.284  -1.860  -6.477  1.00 96.06           N
ATOM   3485  CA  THR A 436       2.336  -2.816  -6.145  1.00 96.06           C
ATOM   3486  C   THR A 436       2.957  -3.395  -7.414  1.00 96.06           C
ATOM   3487  CB  THR A 436       1.797  -3.959  -5.266  1.00 96.06           C
ATOM   3488  O   THR A 436       2.367  -3.312  -8.492  1.00 96.06           O
ATOM   3489  CG2 THR A 436       0.868  -4.871  -6.062  1.00 96.06           C
ATOM   3490  OG1 THR A 436       2.896  -4.730  -4.770  1.00 96.06           O
ATOM   3491  N   SER A 437       4.230  -3.832  -7.293  1.00 97.19           N
ATOM   3492  CA  SER A 437       4.926  -4.379  -8.453  1.00 97.19           C
ATOM   3493  C   SER A 437       5.547  -5.734  -8.141  1.00 97.19           C
ATOM   3494  CB  SER A 437       6.004  -3.410  -8.938  1.00 97.19           C
ATOM   3495  O   SER A 437       6.031  -5.957  -7.031  1.00 97.19           O
ATOM   3496  OG  SER A 437       5.438  -2.162  -9.305  1.00 97.19           O
ATOM   3497  N   ILE A 438       5.418  -6.695  -9.109  1.00 95.50           N
ATOM   3498  CA  ILE A 438       6.086  -7.984  -8.992  1.00 95.50           C
ATOM   3499  C   ILE A 438       6.797  -8.320 -10.305  1.00 95.50           C
ATOM   3500  CB  ILE A 438       5.090  -9.109  -8.625  1.00 95.50           C
ATOM   3501  O   ILE A 438       6.449  -7.781 -11.359  1.00 95.50           O
ATOM   3502  CG1 ILE A 438       4.059  -9.297  -9.742  1.00 95.50           C
ATOM   3503  CG2 ILE A 438       4.402  -8.797  -7.289  1.00 95.50           C
ATOM   3504  CD1 ILE A 438       3.275 -10.602  -9.641  1.00 95.50           C
ATOM   3505  N   THR A 439       7.887  -9.117 -10.125  1.00 95.06           N
ATOM   3506  CA  THR A 439       8.539  -9.750 -11.258  1.00 95.06           C
ATOM   3507  C   THR A 439       8.117 -11.203 -11.391  1.00 95.06           C
ATOM   3508  CB  THR A 439      10.078  -9.672 -11.133  1.00 95.06           C
ATOM   3509  O   THR A 439       8.391 -12.023 -10.500  1.00 95.06           O
ATOM   3510  CG2 THR A 439      10.758 -10.320 -12.328  1.00 95.06           C
ATOM   3511  OG1 THR A 439      10.469  -8.297 -11.062  1.00 95.06           O
ATOM   3512  N   ALA A 440       7.379 -11.445 -12.438  1.00 95.31           N
ATOM   3513  CA  ALA A 440       6.898 -12.797 -12.680  1.00 95.31           C
ATOM   3514  C   ALA A 440       8.016 -13.688 -13.227  1.00 95.31           C
ATOM   3515  CB  ALA A 440       5.719 -12.781 -13.648  1.00 95.31           C
ATOM   3516  O   ALA A 440       8.289 -13.680 -14.422  1.00 95.31           O
```

```
ATOM   3517  N   ILE A 441       8.594 -14.461 -12.344  1.00 93.06           N
ATOM   3518  CA  ILE A 441       9.711 -15.328 -12.688  1.00 93.06           C
ATOM   3519  C   ILE A 441       9.258 -16.375 -13.703  1.00 93.06           C
ATOM   3520  CB  ILE A 441      10.312 -16.016 -11.438  1.00 93.06           C
ATOM   3521  O   ILE A 441       8.211 -17.000 -13.531  1.00 93.06           O
ATOM   3522  CG1 ILE A 441      10.859 -14.961 -10.469  1.00 93.06           C
ATOM   3523  CG2 ILE A 441      11.398 -17.016 -11.836  1.00 93.06           C
ATOM   3524  CD1 ILE A 441      11.266 -15.523  -9.109  1.00 93.06           C
ATOM   3525  N   GLY A 442      10.039 -16.625 -14.742  1.00 93.00           N
ATOM   3526  CA  GLY A 442       9.797 -17.641 -15.750  1.00 93.00           C
ATOM   3527  C   GLY A 442       8.578 -17.359 -16.609  1.00 93.00           C
ATOM   3528  O   GLY A 442       8.102 -18.234 -17.328  1.00 93.00           O
ATOM   3529  N   CYS A 443       7.969 -16.125 -16.531  1.00 95.31           N
ATOM   3530  CA  CYS A 443       6.762 -15.766 -17.281  1.00 95.31           C
ATOM   3531  C   CYS A 443       7.117 -15.117 -18.609  1.00 95.31           C
ATOM   3532  CB  CYS A 443       5.891 -14.820 -16.453  1.00 95.31           C
ATOM   3533  O   CYS A 443       7.812 -14.102 -18.641  1.00 95.31           O
ATOM   3534  SG  CYS A 443       4.379 -14.305 -17.297  1.00 95.31           S
ATOM   3535  N   THR A 444       6.738 -15.625 -19.734  1.00 94.88           N
ATOM   3536  CA  THR A 444       7.043 -15.117 -21.078  1.00 94.88           C
ATOM   3537  C   THR A 444       5.766 -14.727 -21.812  1.00 94.88           C
ATOM   3538  CB  THR A 444       7.820 -16.156 -21.906  1.00 94.88           C
ATOM   3539  O   THR A 444       5.812 -14.297 -22.969  1.00 94.88           O
ATOM   3540  CG2 THR A 444       9.203 -16.406 -21.312  1.00 94.88           C
ATOM   3541  OG1 THR A 444       7.090 -17.391 -21.922  1.00 94.88           O
ATOM   3542  N   ARG A 445       4.598 -14.953 -21.109  1.00 94.25           N
ATOM   3543  CA  ARG A 445       3.311 -14.625 -21.719  1.00 94.25           C
ATOM   3544  C   ARG A 445       2.635 -13.477 -20.969  1.00 94.25           C
ATOM   3545  CB  ARG A 445       2.395 -15.852 -21.734  1.00 94.25           C
ATOM   3546  O   ARG A 445       2.578 -13.477 -19.734  1.00 94.25           O
ATOM   3547  CG  ARG A 445       2.957 -17.031 -22.500  1.00 94.25           C
ATOM   3548  CD  ARG A 445       2.059 -18.250 -22.406  1.00 94.25           C
ATOM   3549  NE  ARG A 445       1.967 -18.734 -21.031  1.00 94.25           N
ATOM   3550  NH1 ARG A 445       0.384 -20.359 -21.484  1.00 94.25           N
ATOM   3551  NH2 ARG A 445       1.169 -20.094 -19.344  1.00 94.25           N
ATOM   3552  CZ  ARG A 445       1.174 -19.719 -20.625  1.00 94.25           C
ATOM   3553  N   ARG A 446       2.160 -12.492 -21.734  1.00 94.62           N
ATOM   3554  CA  ARG A 446       1.500 -11.328 -21.156  1.00 94.62           C
ATOM   3555  C   ARG A 446       0.228 -11.727 -20.406  1.00 94.62           C
ATOM   3556  CB  ARG A 446       1.173 -10.297 -22.234  1.00 94.62           C
ATOM   3557  O   ARG A 446      -0.077 -11.188 -19.344  1.00 94.62           O
ATOM   3558  CG  ARG A 446       0.594  -9.000 -21.703  1.00 94.62           C
ATOM   3559  CD  ARG A 446       0.341  -7.988 -22.812  1.00 94.62           C
ATOM   3560  NE  ARG A 446       1.579  -7.355 -23.250  1.00 94.62           N
ATOM   3561  NH1 ARG A 446       0.576  -6.074 -24.891  1.00 94.62           N
ATOM   3562  NH2 ARG A 446       2.840  -5.949 -24.562  1.00 94.62           N
ATOM   3563  CZ  ARG A 446       1.662  -6.461 -24.234  1.00 94.62           C
ATOM   3564  N   SER A 447      -0.589 -12.648 -20.938  1.00 94.50           N
ATOM   3565  CA  SER A 447      -1.836 -13.117 -20.344  1.00 94.50           C
ATOM   3566  C   SER A 447      -1.592 -13.766 -18.984  1.00 94.50           C
ATOM   3567  CB  SER A 447      -2.531 -14.109 -21.281  1.00 94.50           C
ATOM   3568  O   SER A 447      -2.361 -13.555 -18.047  1.00 94.50           O
ATOM   3569  OG  SER A 447      -1.672 -15.195 -21.594  1.00 94.50           O
ATOM   3570  N   GLU A 448      -0.537 -14.609 -18.875  1.00 95.19           N
ATOM   3571  CA  GLU A 448      -0.174 -15.219 -17.609  1.00 95.19           C
ATOM   3572  C   GLU A 448       0.240 -14.156 -16.594  1.00 95.19           C
ATOM   3573  CB  GLU A 448       0.956 -16.234 -17.797  1.00 95.19           C
ATOM   3574  O   GLU A 448      -0.122 -14.234 -15.414  1.00 95.19           O
ATOM   3575  CG  GLU A 448       1.381 -16.922 -16.500  1.00 95.19           C
ATOM   3576  CD  GLU A 448       2.477 -17.953 -16.703  1.00 95.19           C
ATOM   3577  OE1 GLU A 448       3.148 -18.328 -15.727  1.00 95.19           O
ATOM   3578  OE2 GLU A 448       2.662 -18.391 -17.859  1.00 95.19           O
ATOM   3579  N   ALA A 449       1.062 -13.062 -17.078  1.00 95.69           N
ATOM   3580  CA  ALA A 449       1.449 -11.930 -16.234  1.00 95.69           C
```

```
ATOM   3581  C   ALA A 449       0.221 -11.203 -15.695  1.00 95.69           C
ATOM   3582  CB  ALA A 449       2.338 -10.961 -17.016  1.00 95.69           C
ATOM   3583  O   ALA A 449       0.187 -10.812 -14.523  1.00 95.69           O
ATOM   3584  N   ASN A 450      -0.803 -11.016 -16.516  1.00 95.06           N
ATOM   3585  CA  ASN A 450      -2.061 -10.398 -16.109  1.00 95.06           C
ATOM   3586  C   ASN A 450      -2.762 -11.211 -15.023  1.00 95.06           C
ATOM   3587  CB  ASN A 450      -2.982 -10.219 -17.312  1.00 95.06           C
ATOM   3588  O   ASN A 450      -3.242 -10.648 -14.039  1.00 95.06           O
ATOM   3589  CG  ASN A 450      -4.246  -9.453 -16.984  1.00 95.06           C
ATOM   3590  ND2 ASN A 450      -4.188  -8.133 -17.094  1.00 95.06           N
ATOM   3591  OD1 ASN A 450      -5.270 -10.047 -16.625  1.00 95.06           O
ATOM   3592  N   ARG A 451      -2.855 -12.586 -15.180  1.00 95.25           N
ATOM   3593  CA  ARG A 451      -3.482 -13.453 -14.180  1.00 95.25           C
ATOM   3594  C   ARG A 451      -2.729 -13.398 -12.859  1.00 95.25           C
ATOM   3595  CB  ARG A 451      -3.555 -14.891 -14.688  1.00 95.25           C
ATOM   3596  O   ARG A 451      -3.340 -13.422 -11.789  1.00 95.25           O
ATOM   3597  CG  ARG A 451      -4.613 -15.117 -15.758  1.00 95.25           C
ATOM   3598  CD  ARG A 451      -4.738 -16.594 -16.125  1.00 95.25           C
ATOM   3599  NE  ARG A 451      -3.551 -17.078 -16.828  1.00 95.25           N
ATOM   3600  NH1 ARG A 451      -3.521 -19.141 -15.773  1.00 95.25           N
ATOM   3601  NH2 ARG A 451      -1.920 -18.609 -17.328  1.00 95.25           N
ATOM   3602  CZ  ARG A 451      -3.000 -18.281 -16.641  1.00 95.25           C
ATOM   3603  N   ARG A 452      -1.399 -13.305 -12.914  1.00 94.88           N
ATOM   3604  CA  ARG A 452      -0.604 -13.188 -11.695  1.00 94.88           C
ATOM   3605  C   ARG A 452      -0.896 -11.867 -10.984  1.00 94.88           C
ATOM   3606  CB  ARG A 452       0.889 -13.289 -12.016  1.00 94.88           C
ATOM   3607  O   ARG A 452      -1.001 -11.828  -9.758  1.00 94.88           O
ATOM   3608  CG  ARG A 452       1.335 -14.680 -12.430  1.00 94.88           C
ATOM   3609  CD  ARG A 452       2.852 -14.789 -12.508  1.00 94.88           C
ATOM   3610  NE  ARG A 452       3.277 -16.125 -12.930  1.00 94.88           N
ATOM   3611  NH1 ARG A 452       5.340 -16.016 -11.898  1.00 94.88           N
ATOM   3612  NH2 ARG A 452       4.734 -17.891 -13.062  1.00 94.88           N
ATOM   3613  CZ  ARG A 452       4.449 -16.672 -12.633  1.00 94.88           C
ATOM   3614  N   GLY A 453      -1.003 -10.750 -11.789  1.00 95.56           N
ATOM   3615  CA  GLY A 453      -1.385  -9.461 -11.227  1.00 95.56           C
ATOM   3616  C   GLY A 453      -2.756  -9.484 -10.578  1.00 95.56           C
ATOM   3617  O   GLY A 453      -2.930  -8.969  -9.469  1.00 95.56           O
ATOM   3618  N   ARG A 454      -3.721 -10.109 -11.211  1.00 94.38           N
ATOM   3619  CA  ARG A 454      -5.074 -10.234 -10.680  1.00 94.38           C
ATOM   3620  C   ARG A 454      -5.094 -11.062  -9.406  1.00 94.38           C
ATOM   3621  CB  ARG A 454      -6.008 -10.852 -11.727  1.00 94.38           C
ATOM   3622  O   ARG A 454      -5.812 -10.734  -8.453  1.00 94.38           O
ATOM   3623  CG  ARG A 454      -6.383  -9.914 -12.859  1.00 94.38           C
ATOM   3624  CD  ARG A 454      -7.328 -10.570 -13.852  1.00 94.38           C
ATOM   3625  NE  ARG A 454      -7.652  -9.680 -14.961  1.00 94.38           N
ATOM   3626  NH1 ARG A 454      -8.992 -11.203 -16.062  1.00 94.38           N
ATOM   3627  NH2 ARG A 454      -8.664  -9.117 -16.938  1.00 94.38           N
ATOM   3628  CZ  ARG A 454      -8.438 -10.000 -15.984  1.00 94.38           C
ATOM   3629  N   TRP A 455      -4.254 -12.086  -9.453  1.00 94.19           N
ATOM   3630  CA  TRP A 455      -4.156 -12.938  -8.273  1.00 94.19           C
ATOM   3631  C   TRP A 455      -3.674 -12.148  -7.062  1.00 94.19           C
ATOM   3632  CB  TRP A 455      -3.211 -14.117  -8.539  1.00 94.19           C
ATOM   3633  O   TRP A 455      -4.242 -12.258  -5.977  1.00 94.19           O
ATOM   3634  CG  TRP A 455      -2.912 -14.945  -7.324  1.00 94.19           C
ATOM   3635  CD1 TRP A 455      -3.756 -15.812  -6.688  1.00 94.19           C
ATOM   3636  CD2 TRP A 455      -1.682 -14.969  -6.594  1.00 94.19           C
ATOM   3637  CE2 TRP A 455      -1.851 -15.883  -5.527  1.00 94.19           C
ATOM   3638  CE3 TRP A 455      -0.451 -14.320  -6.738  1.00 94.19           C
ATOM   3639  NE1 TRP A 455      -3.123 -16.375  -5.605  1.00 94.19           N
ATOM   3640  CH2 TRP A 455       0.360 -15.500  -4.773  1.00 94.19           C
ATOM   3641  CZ2 TRP A 455      -0.833 -16.156  -4.609  1.00 94.19           C
ATOM   3642  CZ3 TRP A 455       0.560 -14.586  -5.824  1.00 94.19           C
ATOM   3643  N   ILE A 456      -2.725 -11.359  -7.211  1.00 94.12           N
ATOM   3644  CA  ILE A 456      -2.168 -10.555  -6.125  1.00 94.12           C
```

ATOM	3645	C	ILE	A	456	-3.227	-9.578	-5.613	1.00	94.12	C
ATOM	3646	CB	ILE	A	456	-0.907	-9.789	-6.582	1.00	94.12	C
ATOM	3647	O	ILE	A	456	-3.406	-9.430	-4.402	1.00	94.12	O
ATOM	3648	CG1	ILE	A	456	0.240	-10.766	-6.863	1.00	94.12	C
ATOM	3649	CG2	ILE	A	456	-0.498	-8.750	-5.531	1.00	94.12	C
ATOM	3650	CD1	ILE	A	456	0.738	-11.508	-5.633	1.00	94.12	C
ATOM	3651	N	LEU	A	457	-3.900	-8.945	-6.523	1.00	93.62	N
ATOM	3652	CA	LEU	A	457	-4.895	-7.945	-6.160	1.00	93.62	C
ATOM	3653	C	LEU	A	457	-6.082	-8.586	-5.453	1.00	93.62	C
ATOM	3654	CB	LEU	A	457	-5.375	-7.191	-7.402	1.00	93.62	C
ATOM	3655	O	LEU	A	457	-6.523	-8.102	-4.406	1.00	93.62	O
ATOM	3656	CG	LEU	A	457	-4.332	-6.332	-8.117	1.00	93.62	C
ATOM	3657	CD1	LEU	A	457	-4.949	-5.660	-9.344	1.00	93.62	C
ATOM	3658	CD2	LEU	A	457	-3.750	-5.293	-7.168	1.00	93.62	C
ATOM	3659	N	LYS	A	458	-6.582	-9.727	-5.992	1.00	92.06	N
ATOM	3660	CA	LYS	A	458	-7.766	-10.375	-5.434	1.00	92.06	C
ATOM	3661	C	LYS	A	458	-7.453	-11.031	-4.094	1.00	92.06	C
ATOM	3662	CB	LYS	A	458	-8.320	-11.414	-6.410	1.00	92.06	C
ATOM	3663	O	LYS	A	458	-8.281	-11.023	-3.180	1.00	92.06	O
ATOM	3664	CG	LYS	A	458	-8.898	-10.820	-7.684	1.00	92.06	C
ATOM	3665	CD	LYS	A	458	-10.133	-9.984	-7.398	1.00	92.06	C
ATOM	3666	CE	LYS	A	458	-10.766	-9.453	-8.680	1.00	92.06	C
ATOM	3667	NZ	LYS	A	458	-11.906	-8.539	-8.398	1.00	92.06	N
ATOM	3668	N	THR	A	459	-6.273	-11.594	-4.000	1.00	91.50	N
ATOM	3669	CA	THR	A	459	-5.844	-12.180	-2.736	1.00	91.50	C
ATOM	3670	C	THR	A	459	-5.664	-11.102	-1.673	1.00	91.50	C
ATOM	3671	CB	THR	A	459	-4.531	-12.961	-2.902	1.00	91.50	C
ATOM	3672	O	THR	A	459	-6.043	-11.289	-0.516	1.00	91.50	O
ATOM	3673	CG2	THR	A	459	-4.078	-13.570	-1.576	1.00	91.50	C
ATOM	3674	OG1	THR	A	459	-4.730	-14.023	-3.852	1.00	91.50	O
ATOM	3675	N	ASN	A	460	-5.160	-9.914	-2.100	1.00	90.94	N
ATOM	3676	CA	ASN	A	460	-4.906	-8.789	-1.206	1.00	90.94	C
ATOM	3677	C	ASN	A	460	-6.203	-8.211	-0.651	1.00	90.94	C
ATOM	3678	CB	ASN	A	460	-4.102	-7.703	-1.926	1.00	90.94	C
ATOM	3679	O	ASN	A	460	-6.250	-7.781	0.504	1.00	90.94	O
ATOM	3680	CG	ASN	A	460	-3.828	-6.500	-1.047	1.00	90.94	C
ATOM	3681	ND2	ASN	A	460	-2.906	-6.652	-0.104	1.00	90.94	N
ATOM	3682	OD1	ASN	A	460	-4.441	-5.441	-1.215	1.00	90.94	O
ATOM	3683	N	LEU	A	461	-7.258	-8.219	-1.448	1.00	87.69	N
ATOM	3684	CA	LEU	A	461	-8.539	-7.641	-1.059	1.00	87.69	C
ATOM	3685	C	LEU	A	461	-9.211	-8.492	0.015	1.00	87.69	C
ATOM	3686	CB	LEU	A	461	-9.461	-7.512	-2.273	1.00	87.69	C
ATOM	3687	O	LEU	A	461	-10.133	-8.023	0.695	1.00	87.69	O
ATOM	3688	CG	LEU	A	461	-9.070	-6.457	-3.311	1.00	87.69	C
ATOM	3689	CD1	LEU	A	461	-10.016	-6.512	-4.508	1.00	87.69	C
ATOM	3690	CD2	LEU	A	461	-9.070	-5.066	-2.686	1.00	87.69	C
ATOM	3691	N	ARG	A	462	-8.672	-9.695	0.223	1.00	87.94	N
ATOM	3692	CA	ARG	A	462	-9.188	-10.617	1.229	1.00	87.94	C
ATOM	3693	C	ARG	A	462	-8.148	-10.859	2.322	1.00	87.94	C
ATOM	3694	CB	ARG	A	462	-9.586	-11.945	0.583	1.00	87.94	C
ATOM	3695	O	ARG	A	462	-7.633	-11.977	2.461	1.00	87.94	O
ATOM	3696	CG	ARG	A	462	-10.578	-11.805	-0.559	1.00	87.94	C
ATOM	3697	CD	ARG	A	462	-11.875	-11.148	-0.100	1.00	87.94	C
ATOM	3698	NE	ARG	A	462	-12.375	-10.188	-1.084	1.00	87.94	N
ATOM	3699	NH1	ARG	A	462	-14.133	-9.453	0.221	1.00	87.94	N
ATOM	3700	NH2	ARG	A	462	-13.797	-8.570	-1.870	1.00	87.94	N
ATOM	3701	CZ	ARG	A	462	-13.430	-9.406	-0.909	1.00	87.94	C
ATOM	3702	N	SER	A	463	-7.828	-9.805	3.188	1.00	87.56	N
ATOM	3703	CA	SER	A	463	-6.691	-9.820	4.102	1.00	87.56	C
ATOM	3704	C	SER	A	463	-7.148	-9.852	5.555	1.00	87.56	C
ATOM	3705	CB	SER	A	463	-5.801	-8.594	3.869	1.00	87.56	C
ATOM	3706	O	SER	A	463	-6.344	-9.680	6.473	1.00	87.56	O
ATOM	3707	OG	SER	A	463	-6.559	-7.402	3.934	1.00	87.56	O
ATOM	3708	N	THR	A	464	-8.453	-10.008	5.734	1.00	93.50	N

```
ATOM   3709  CA  THR A 464      -8.945 -10.086   7.105  1.00 93.50           C
ATOM   3710  C   THR A 464      -8.375 -11.305   7.816  1.00 93.50           C
ATOM   3711  CB  THR A 464     -10.484 -10.133   7.145  1.00 93.50           C
ATOM   3712  O   THR A 464      -8.445 -12.422   7.297  1.00 93.50           O
ATOM   3713  CG2 THR A 464     -11.000 -10.133   8.578  1.00 93.50           C
ATOM   3714  OG1 THR A 464     -11.008  -8.984   6.465  1.00 93.50           O
ATOM   3715  N   THR A 465      -7.762 -11.062   8.961  1.00 95.94           N
ATOM   3716  CA  THR A 465      -7.191 -12.133   9.773  1.00 95.94           C
ATOM   3717  C   THR A 465      -8.086 -12.445  10.961  1.00 95.94           C
ATOM   3718  CB  THR A 465      -5.781 -11.758  10.273  1.00 95.94           C
ATOM   3719  O   THR A 465      -8.617 -11.531  11.609  1.00 95.94           O
ATOM   3720  CG2 THR A 465      -5.184 -12.875  11.117  1.00 95.94           C
ATOM   3721  OG1 THR A 465      -4.930 -11.516   9.148  1.00 95.94           O
ATOM   3722  N   VAL A 466      -8.328 -13.742  11.188  1.00 97.19           N
ATOM   3723  CA  VAL A 466      -9.102 -14.180  12.344  1.00 97.19           C
ATOM   3724  C   VAL A 466      -8.203 -14.977  13.297  1.00 97.19           C
ATOM   3725  CB  VAL A 466     -10.320 -15.031  11.930  1.00 97.19           C
ATOM   3726  O   VAL A 466      -7.406 -15.805  12.859  1.00 97.19           O
ATOM   3727  CG1 VAL A 466      -9.875 -16.297  11.203  1.00 97.19           C
ATOM   3728  CG2 VAL A 466     -11.172 -15.375  13.148  1.00 97.19           C
ATOM   3729  N   ASN A 467      -8.281 -14.602  14.594  1.00 97.00           N
ATOM   3730  CA  ASN A 467      -7.578 -15.305  15.664  1.00 97.00           C
ATOM   3731  C   ASN A 467      -8.555 -15.906  16.672  1.00 97.00           C
ATOM   3732  CB  ASN A 467      -6.598 -14.359  16.359  1.00 97.00           C
ATOM   3733  O   ASN A 467      -9.469 -15.227  17.141  1.00 97.00           O
ATOM   3734  CG  ASN A 467      -5.566 -13.773  15.422  1.00 97.00           C
ATOM   3735  ND2 ASN A 467      -5.695 -12.484  15.133  1.00 97.00           N
ATOM   3736  OD1 ASN A 467      -4.664 -14.477  14.953  1.00 97.00           O
ATOM   3737  N   PHE A 468      -8.383 -17.188  16.953  1.00 97.06           N
ATOM   3738  CA  PHE A 468      -9.211 -17.844  17.953  1.00 97.06           C
ATOM   3739  C   PHE A 468      -8.492 -19.031  18.562  1.00 97.06           C
ATOM   3740  CB  PHE A 468     -10.547 -18.297  17.344  1.00 97.06           C
ATOM   3741  O   PHE A 468      -7.500 -19.516  18.000  1.00 97.06           O
ATOM   3742  CG  PHE A 468     -10.391 -19.312  16.234  1.00 97.06           C
ATOM   3743  CD1 PHE A 468     -10.164 -18.906  14.930  1.00 97.06           C
ATOM   3744  CD2 PHE A 468     -10.484 -20.672  16.516  1.00 97.06           C
ATOM   3745  CE1 PHE A 468     -10.023 -19.844  13.906  1.00 97.06           C
ATOM   3746  CE2 PHE A 468     -10.344 -21.609  15.500  1.00 97.06           C
ATOM   3747  CZ  PHE A 468     -10.117 -21.203  14.203  1.00 97.06           C
ATOM   3748  N   ALA A 469      -8.898 -19.438  19.781  1.00 96.19           N
ATOM   3749  CA  ALA A 469      -8.375 -20.594  20.484  1.00 96.19           C
ATOM   3750  C   ALA A 469      -9.438 -21.688  20.609  1.00 96.19           C
ATOM   3751  CB  ALA A 469      -7.855 -20.203  21.859  1.00 96.19           C
ATOM   3752  O   ALA A 469     -10.609 -21.406  20.859  1.00 96.19           O
ATOM   3753  N   THR A 470      -8.984 -22.938  20.281  1.00 94.88           N
ATOM   3754  CA  THR A 470      -9.883 -24.078  20.359  1.00 94.88           C
ATOM   3755  C   THR A 470      -9.242 -25.219  21.141  1.00 94.88           C
ATOM   3756  CB  THR A 470     -10.297 -24.562  18.953  1.00 94.88           C
ATOM   3757  O   THR A 470      -8.078 -25.141  21.531  1.00 94.88           O
ATOM   3758  CG2 THR A 470      -9.102 -25.172  18.219  1.00 94.88           C
ATOM   3759  OG1 THR A 470     -11.312 -25.562  19.094  1.00 94.88           O
ATOM   3760  N   GLY A 471     -10.062 -26.188  21.531  1.00 93.38           N
ATOM   3761  CA  GLY A 471      -9.547 -27.359  22.234  1.00 93.38           C
ATOM   3762  C   GLY A 471      -8.805 -28.312  21.312  1.00 93.38           C
ATOM   3763  O   GLY A 471      -8.031 -27.875  20.453  1.00 93.38           O
ATOM   3764  N   LEU A 472      -8.953 -29.562  21.438  1.00 90.44           N
ATOM   3765  CA  LEU A 472      -8.227 -30.609  20.734  1.00 90.44           C
ATOM   3766  C   LEU A 472      -8.578 -30.609  19.250  1.00 90.44           C
ATOM   3767  CB  LEU A 472      -8.531 -31.984  21.328  1.00 90.44           C
ATOM   3768  O   LEU A 472      -7.848 -31.172  18.422  1.00 90.44           O
ATOM   3769  CG  LEU A 472      -7.930 -32.281  22.719  1.00 90.44           C
ATOM   3770  CD1 LEU A 472      -8.461 -33.594  23.266  1.00 90.44           C
ATOM   3771  CD2 LEU A 472      -6.406 -32.281  22.641  1.00 90.44           C
ATOM   3772  N   GLU A 473      -9.656 -29.891  18.969  1.00 89.88           N
```

```
ATOM   3773  CA  GLU A 473     -10.062 -29.797  17.578  1.00 89.88           C
ATOM   3774  C   GLU A 473      -9.039 -29.016  16.750  1.00 89.88           C
ATOM   3775  CB  GLU A 473     -11.438 -29.141  17.453  1.00 89.88           C
ATOM   3776  O   GLU A 473      -9.008 -29.109  15.531  1.00 89.88           O
ATOM   3777  CG  GLU A 473     -12.562 -29.953  18.078  1.00 89.88           C
ATOM   3778  CD  GLU A 473     -12.812 -29.609  19.547  1.00 89.88           C
ATOM   3779  OE1 GLU A 473     -13.828 -30.062  20.109  1.00 89.88           O
ATOM   3780  OE2 GLU A 473     -11.984 -28.859  20.125  1.00 89.88           O
ATOM   3781  N   GLY A 474      -8.227 -28.188  17.422  1.00 91.00           N
ATOM   3782  CA  GLY A 474      -7.184 -27.406  16.766  1.00 91.00           C
ATOM   3783  C   GLY A 474      -6.121 -28.266  16.109  1.00 91.00           C
ATOM   3784  O   GLY A 474      -5.328 -27.766  15.305  1.00 91.00           O
ATOM   3785  N   MET A 475      -6.117 -29.641  16.359  1.00 88.69           N
ATOM   3786  CA  MET A 475      -5.133 -30.562  15.805  1.00 88.69           C
ATOM   3787  C   MET A 475      -5.574 -31.078  14.438  1.00 88.69           C
ATOM   3788  CB  MET A 475      -4.906 -31.750  16.750  1.00 88.69           C
ATOM   3789  O   MET A 475      -4.793 -31.719  13.727  1.00 88.69           O
ATOM   3790  CG  MET A 475      -4.215 -31.359  18.047  1.00 88.69           C
ATOM   3791  SD  MET A 475      -4.082 -32.781  19.219  1.00 88.69           S
ATOM   3792  CE  MET A 475      -2.828 -33.781  18.375  1.00 88.69           C
ATOM   3793  N   ILE A 476      -6.766 -30.766  14.008  1.00 90.31           N
ATOM   3794  CA  ILE A 476      -7.336 -31.328  12.789  1.00 90.31           C
ATOM   3795  C   ILE A 476      -6.859 -30.516  11.578  1.00 90.31           C
ATOM   3796  CB  ILE A 476      -8.883 -31.344  12.844  1.00 90.31           C
ATOM   3797  O   ILE A 476      -6.207 -31.062  10.680  1.00 90.31           O
ATOM   3798  CG1 ILE A 476      -9.359 -32.250  13.984  1.00 90.31           C
ATOM   3799  CG2 ILE A 476      -9.461 -31.797  11.500  1.00 90.31           C
ATOM   3800  CD1 ILE A 476     -10.867 -32.219  14.203  1.00 90.31           C
ATOM   3801  N   PRO A 477      -7.098 -29.125  11.672  1.00 90.62           N
ATOM   3802  CA  PRO A 477      -6.590 -28.375  10.516  1.00 90.62           C
ATOM   3803  C   PRO A 477      -5.074 -28.219  10.539  1.00 90.62           C
ATOM   3804  CB  PRO A 477      -7.289 -27.031  10.641  1.00 90.62           C
ATOM   3805  O   PRO A 477      -4.469 -28.203  11.617  1.00 90.62           O
ATOM   3806  CG  PRO A 477      -7.566 -26.859  12.102  1.00 90.62           C
ATOM   3807  CD  PRO A 477      -7.781 -28.234  12.695  1.00 90.62           C
ATOM   3808  N   THR A 478      -4.457 -28.250   9.406  1.00 87.50           N
ATOM   3809  CA  THR A 478      -3.039 -27.938   9.242  1.00 87.50           C
ATOM   3810  C   THR A 478      -2.857 -26.594   8.539  1.00 87.50           C
ATOM   3811  CB  THR A 478      -2.307 -29.047   8.461  1.00 87.50           C
ATOM   3812  O   THR A 478      -3.820 -26.031   8.023  1.00 87.50           O
ATOM   3813  CG2 THR A 478      -2.438 -30.391   9.156  1.00 87.50           C
ATOM   3814  OG1 THR A 478      -2.871 -29.141   7.148  1.00 87.50           O
ATOM   3815  N   ILE A 479      -1.655 -26.000   8.664  1.00 86.62           N
ATOM   3816  CA  ILE A 479      -1.353 -24.766   7.969  1.00 86.62           C
ATOM   3817  C   ILE A 479      -1.632 -24.922   6.477  1.00 86.62           C
ATOM   3818  CB  ILE A 479       0.113 -24.328   8.195  1.00 86.62           C
ATOM   3819  O   ILE A 479      -1.251 -25.922   5.871  1.00 86.62           O
ATOM   3820  CG1 ILE A 479       0.354 -24.016   9.680  1.00 86.62           C
ATOM   3821  CG2 ILE A 479       0.464 -23.125   7.316  1.00 86.62           C
ATOM   3822  CD1 ILE A 479       1.807 -23.719  10.016  1.00 86.62           C
ATOM   3823  N   GLY A 480      -2.488 -24.031   5.906  1.00 84.94           N
ATOM   3824  CA  GLY A 480      -2.859 -24.078   4.500  1.00 84.94           C
ATOM   3825  C   GLY A 480      -4.281 -24.562   4.273  1.00 84.94           C
ATOM   3826  O   GLY A 480      -4.863 -24.312   3.217  1.00 84.94           O
ATOM   3827  N   ASP A 481      -4.883 -25.203   5.281  1.00 90.12           N
ATOM   3828  CA  ASP A 481      -6.258 -25.688   5.176  1.00 90.12           C
ATOM   3829  C   ASP A 481      -7.250 -24.516   5.246  1.00 90.12           C
ATOM   3830  CB  ASP A 481      -6.559 -26.703   6.281  1.00 90.12           C
ATOM   3831  O   ASP A 481      -7.000 -23.531   5.930  1.00 90.12           O
ATOM   3832  CG  ASP A 481      -5.984 -28.078   5.996  1.00 90.12           C
ATOM   3833  OD1 ASP A 481      -5.500 -28.312   4.867  1.00 90.12           O
ATOM   3834  OD2 ASP A 481      -6.020 -28.938   6.902  1.00 90.12           O
ATOM   3835  N   VAL A 482      -8.312 -24.656   4.453  1.00 93.62           N
ATOM   3836  CA  VAL A 482      -9.430 -23.719   4.547  1.00 93.62           C
```

ATOM	3837	C	VAL	A	482	-10.406	-24.188	5.621	1.00	93.62	C
ATOM	3838	CB	VAL	A	482	-10.156	-23.562	3.193	1.00	93.62	C
ATOM	3839	O	VAL	A	482	-10.773	-25.359	5.664	1.00	93.62	O
ATOM	3840	CG1	VAL	A	482	-11.352	-22.625	3.330	1.00	93.62	C
ATOM	3841	CG2	VAL	A	482	-9.188	-23.047	2.125	1.00	93.62	C
ATOM	3842	N	VAL	A	483	-10.711	-23.297	6.570	1.00	95.06	N
ATOM	3843	CA	VAL	A	483	-11.695	-23.609	7.602	1.00	95.06	C
ATOM	3844	C	VAL	A	483	-12.922	-22.703	7.438	1.00	95.06	C
ATOM	3845	CB	VAL	A	483	-11.109	-23.453	9.016	1.00	95.06	C
ATOM	3846	O	VAL	A	483	-12.812	-21.578	6.938	1.00	95.06	O
ATOM	3847	CG1	VAL	A	483	-9.977	-24.453	9.242	1.00	95.06	C
ATOM	3848	CG2	VAL	A	483	-10.609	-22.031	9.234	1.00	95.06	C
ATOM	3849	N	ALA	A	484	-14.023	-23.234	7.727	1.00	95.38	N
ATOM	3850	CA	ALA	A	484	-15.258	-22.453	7.730	1.00	95.38	C
ATOM	3851	C	ALA	A	484	-15.594	-21.969	9.141	1.00	95.38	C
ATOM	3852	CB	ALA	A	484	-16.406	-23.281	7.164	1.00	95.38	C
ATOM	3853	O	ALA	A	484	-15.562	-22.750	10.094	1.00	95.38	O
ATOM	3854	N	ILE	A	485	-15.867	-20.703	9.258	1.00	95.81	N
ATOM	3855	CA	ILE	A	485	-16.141	-20.094	10.562	1.00	95.81	C
ATOM	3856	C	ILE	A	485	-17.578	-19.609	10.617	1.00	95.81	C
ATOM	3857	CB	ILE	A	485	-15.164	-18.938	10.867	1.00	95.81	C
ATOM	3858	O	ILE	A	485	-18.000	-18.812	9.766	1.00	95.81	O
ATOM	3859	CG1	ILE	A	485	-13.734	-19.469	11.008	1.00	95.81	C
ATOM	3860	CG2	ILE	A	485	-15.594	-18.188	12.133	1.00	95.81	C
ATOM	3861	CD1	ILE	A	485	-12.695	-18.391	11.266	1.00	95.81	C
ATOM	3862	N	ALA	A	486	-18.297	-20.109	11.523	1.00	95.44	N
ATOM	3863	CA	ALA	A	486	-19.656	-19.641	11.797	1.00	95.44	C
ATOM	3864	C	ALA	A	486	-19.703	-18.844	13.094	1.00	95.44	C
ATOM	3865	CB	ALA	A	486	-20.609	-20.828	11.867	1.00	95.44	C
ATOM	3866	O	ALA	A	486	-19.938	-19.406	14.172	1.00	95.44	O
ATOM	3867	N	ASP	A	487	-19.453	-17.594	13.023	1.00	95.56	N
ATOM	3868	CA	ASP	A	487	-19.469	-16.672	14.148	1.00	95.56	C
ATOM	3869	C	ASP	A	487	-20.797	-15.906	14.211	1.00	95.56	C
ATOM	3870	CB	ASP	A	487	-18.297	-15.680	14.062	1.00	95.56	C
ATOM	3871	O	ASP	A	487	-21.016	-14.977	13.430	1.00	95.56	O
ATOM	3872	CG	ASP	A	487	-18.219	-14.750	15.250	1.00	95.56	C
ATOM	3873	OD1	ASP	A	487	-19.031	-14.883	16.188	1.00	95.56	O
ATOM	3874	OD2	ASP	A	487	-17.328	-13.867	15.258	1.00	95.56	O
ATOM	3875	N	ASN	A	488	-21.641	-16.250	15.188	1.00	91.81	N
ATOM	3876	CA	ASN	A	488	-22.984	-15.664	15.289	1.00	91.81	C
ATOM	3877	C	ASN	A	488	-22.922	-14.188	15.672	1.00	91.81	C
ATOM	3878	CB	ASN	A	488	-23.828	-16.438	16.297	1.00	91.81	C
ATOM	3879	O	ASN	A	488	-23.828	-13.422	15.352	1.00	91.81	O
ATOM	3880	CG	ASN	A	488	-24.219	-17.812	15.781	1.00	91.81	C
ATOM	3881	ND2	ASN	A	488	-24.453	-18.750	16.703	1.00	91.81	N
ATOM	3882	OD1	ASN	A	488	-24.328	-18.031	14.578	1.00	91.81	O
ATOM	3883	N	PHE	A	489	-21.828	-13.789	16.312	1.00	91.25	N
ATOM	3884	CA	PHE	A	489	-21.641	-12.375	16.656	1.00	91.25	C
ATOM	3885	C	PHE	A	489	-21.422	-11.555	15.391	1.00	91.25	C
ATOM	3886	CB	PHE	A	489	-20.469	-12.195	17.609	1.00	91.25	C
ATOM	3887	O	PHE	A	489	-21.953	-10.453	15.258	1.00	91.25	O
ATOM	3888	CG	PHE	A	489	-20.766	-12.664	19.016	1.00	91.25	C
ATOM	3889	CD1	PHE	A	489	-21.422	-11.828	19.922	1.00	91.25	C
ATOM	3890	CD2	PHE	A	489	-20.375	-13.930	19.438	1.00	91.25	C
ATOM	3891	CE1	PHE	A	489	-21.688	-12.258	21.219	1.00	91.25	C
ATOM	3892	CE2	PHE	A	489	-20.641	-14.359	20.734	1.00	91.25	C
ATOM	3893	CZ	PHE	A	489	-21.297	-13.523	21.625	1.00	91.25	C
ATOM	3894	N	TRP	A	490	-20.656	-12.102	14.430	1.00	91.12	N
ATOM	3895	CA	TRP	A	490	-20.328	-11.445	13.172	1.00	91.12	C
ATOM	3896	C	TRP	A	490	-21.516	-11.516	12.203	1.00	91.12	C
ATOM	3897	CB	TRP	A	490	-19.094	-12.086	12.531	1.00	91.12	C
ATOM	3898	O	TRP	A	490	-21.922	-10.492	11.648	1.00	91.12	O
ATOM	3899	CG	TRP	A	490	-18.672	-11.438	11.258	1.00	91.12	C
ATOM	3900	CD1	TRP	A	490	-19.109	-11.727	9.992	1.00	91.12	C

ATOM	3901	CD2	TRP	A	490	-17.703	-10.391	11.109	1.00	91.12	C
ATOM	3902	CE2	TRP	A	490	-17.609	-10.094	9.734	1.00	91.12	C
ATOM	3903	CE3	TRP	A	490	-16.906	-9.672	12.016	1.00	91.12	C
ATOM	3904	NE1	TRP	A	490	-18.469	-10.922	9.070	1.00	91.12	N
ATOM	3905	CH2	TRP	A	490	-15.984	-8.422	10.148	1.00	91.12	C
ATOM	3906	CZ2	TRP	A	490	-16.750	-9.109	9.242	1.00	91.12	C
ATOM	3907	CZ3	TRP	A	490	-16.062	-8.695	11.523	1.00	91.12	C
ATOM	3908	N	SER	A	491	-22.047	-12.688	12.000	1.00	92.38	N
ATOM	3909	CA	SER	A	491	-23.016	-12.938	10.930	1.00	92.38	C
ATOM	3910	C	SER	A	491	-24.312	-12.195	11.180	1.00	92.38	C
ATOM	3911	CB	SER	A	491	-23.281	-14.430	10.789	1.00	92.38	C
ATOM	3912	O	SER	A	491	-24.938	-11.664	10.250	1.00	92.38	O
ATOM	3913	OG	SER	A	491	-23.844	-14.953	11.984	1.00	92.38	O
ATOM	3914	N	SER	A	492	-24.734	-12.070	12.547	1.00	90.62	N
ATOM	3915	CA	SER	A	492	-26.078	-11.570	12.797	1.00	90.62	C
ATOM	3916	C	SER	A	492	-26.141	-10.758	14.094	1.00	90.62	C
ATOM	3917	CB	SER	A	492	-27.078	-12.719	12.859	1.00	90.62	C
ATOM	3918	O	SER	A	492	-27.203	-10.273	14.477	1.00	90.62	O
ATOM	3919	OG	SER	A	492	-26.844	-13.523	14.000	1.00	90.62	O
ATOM	3920	N	ASN	A	493	-25.016	-10.656	14.789	1.00	87.31	N
ATOM	3921	CA	ASN	A	493	-25.000	-10.016	16.094	1.00	87.31	C
ATOM	3922	C	ASN	A	493	-25.922	-10.734	17.078	1.00	87.31	C
ATOM	3923	CB	ASN	A	493	-25.406	-8.539	15.977	1.00	87.31	C
ATOM	3924	O	ASN	A	493	-26.703	-10.094	17.797	1.00	87.31	O
ATOM	3925	CG	ASN	A	493	-25.047	-7.738	17.219	1.00	87.31	C
ATOM	3926	ND2	ASN	A	493	-25.781	-6.656	17.453	1.00	87.31	N
ATOM	3927	OD1	ASN	A	493	-24.125	-8.086	17.953	1.00	87.31	O
ATOM	3928	N	LEU	A	494	-26.062	-12.055	16.891	1.00	87.81	N
ATOM	3929	CA	LEU	A	494	-26.750	-13.000	17.781	1.00	87.81	C
ATOM	3930	C	LEU	A	494	-28.250	-12.938	17.578	1.00	87.81	C
ATOM	3931	CB	LEU	A	494	-26.406	-12.703	19.234	1.00	87.81	C
ATOM	3932	O	LEU	A	494	-29.016	-13.305	18.469	1.00	87.81	O
ATOM	3933	CG	LEU	A	494	-24.922	-12.812	19.625	1.00	87.81	C
ATOM	3934	CD1	LEU	A	494	-24.719	-12.398	21.078	1.00	87.81	C
ATOM	3935	CD2	LEU	A	494	-24.406	-14.234	19.391	1.00	87.81	C
ATOM	3936	N	THR	A	495	-28.766	-12.281	16.469	1.00	87.56	N
ATOM	3937	CA	THR	A	495	-30.188	-12.242	16.188	1.00	87.56	C
ATOM	3938	C	THR	A	495	-30.641	-13.555	15.539	1.00	87.56	C
ATOM	3939	CB	THR	A	495	-30.547	-11.062	15.258	1.00	87.56	C
ATOM	3940	O	THR	A	495	-31.828	-13.898	15.586	1.00	87.56	O
ATOM	3941	CG2	THR	A	495	-30.266	-9.727	15.945	1.00	87.56	C
ATOM	3942	OG1	THR	A	495	-29.750	-11.141	14.070	1.00	87.56	O
ATOM	3943	N	MET	A	496	-29.641	-14.266	14.992	1.00	90.44	N
ATOM	3944	CA	MET	A	496	-29.906	-15.578	14.406	1.00	90.44	C
ATOM	3945	C	MET	A	496	-29.031	-16.656	15.055	1.00	90.44	C
ATOM	3946	CB	MET	A	496	-29.672	-15.555	12.898	1.00	90.44	C
ATOM	3947	O	MET	A	496	-27.875	-16.406	15.352	1.00	90.44	O
ATOM	3948	CG	MET	A	496	-30.625	-14.641	12.141	1.00	90.44	C
ATOM	3949	SD	MET	A	496	-30.219	-14.516	10.359	1.00	90.44	S
ATOM	3950	CE	MET	A	496	-31.312	-15.812	9.688	1.00	90.44	C
ATOM	3951	N	ASN	A	497	-29.656	-17.844	15.289	1.00	92.62	N
ATOM	3952	CA	ASN	A	497	-28.922	-19.016	15.750	1.00	92.62	C
ATOM	3953	C	ASN	A	497	-28.625	-19.969	14.602	1.00	92.62	C
ATOM	3954	CB	ASN	A	497	-29.688	-19.734	16.859	1.00	92.62	C
ATOM	3955	O	ASN	A	497	-29.469	-20.797	14.242	1.00	92.62	O
ATOM	3956	CG	ASN	A	497	-29.781	-18.906	18.125	1.00	92.62	C
ATOM	3957	ND2	ASN	A	497	-30.875	-19.078	18.859	1.00	92.62	N
ATOM	3958	OD1	ASN	A	497	-28.875	-18.141	18.453	1.00	92.62	O
ATOM	3959	N	LEU	A	498	-27.406	-19.922	14.031	1.00	94.31	N
ATOM	3960	CA	LEU	A	498	-27.109	-20.672	12.820	1.00	94.31	C
ATOM	3961	C	LEU	A	498	-26.000	-21.703	13.070	1.00	94.31	C
ATOM	3962	CB	LEU	A	498	-26.672	-19.734	11.688	1.00	94.31	C
ATOM	3963	O	LEU	A	498	-25.578	-22.406	12.156	1.00	94.31	O
ATOM	3964	CG	LEU	A	498	-27.719	-18.719	11.227	1.00	94.31	C

```
ATOM   3965  CD1 LEU A 498     -27.125 -17.781  10.180  1.00 94.31           C
ATOM   3966  CD2 LEU A 498     -28.953 -19.422  10.672  1.00 94.31           C
ATOM   3967  N   SER A 499     -25.562 -21.828  14.211  1.00 95.25           N
ATOM   3968  CA  SER A 499     -24.531 -22.812  14.539  1.00 95.25           C
ATOM   3969  C   SER A 499     -24.656 -23.281  15.984  1.00 95.25           C
ATOM   3970  CB  SER A 499     -23.141 -22.234  14.289  1.00 95.25           C
ATOM   3971  O   SER A 499     -25.312 -22.625  16.797  1.00 95.25           O
ATOM   3972  OG  SER A 499     -22.875 -21.156  15.180  1.00 95.25           O
ATOM   3973  N   GLY A 500     -24.078 -24.469  16.266  1.00 94.06           N
ATOM   3974  CA  GLY A 500     -24.078 -25.047  17.609  1.00 94.06           C
ATOM   3975  C   GLY A 500     -23.734 -26.531  17.609  1.00 94.06           C
ATOM   3976  O   GLY A 500     -23.031 -27.016  16.703  1.00 94.06           O
ATOM   3977  N   ARG A 501     -24.156 -27.109  18.688  1.00 94.00           N
ATOM   3978  CA  ARG A 501     -23.969 -28.547  18.797  1.00 94.00           C
ATOM   3979  C   ARG A 501     -25.312 -29.281  18.766  1.00 94.00           C
ATOM   3980  CB  ARG A 501     -23.219 -28.906  20.078  1.00 94.00           C
ATOM   3981  O   ARG A 501     -26.281 -28.828  19.375  1.00 94.00           O
ATOM   3982  CG  ARG A 501     -21.781 -28.422  20.109  1.00 94.00           C
ATOM   3983  CD  ARG A 501     -21.062 -28.844  21.375  1.00 94.00           C
ATOM   3984  NE  ARG A 501     -19.672 -28.359  21.391  1.00 94.00           N
ATOM   3985  NH1 ARG A 501     -18.828 -30.141  20.203  1.00 94.00           N
ATOM   3986  NH2 ARG A 501     -17.438 -28.469  20.922  1.00 94.00           N
ATOM   3987  CZ  ARG A 501     -18.641 -29.000  20.844  1.00 94.00           C
ATOM   3988  N   LEU A 502     -25.234 -30.391  17.953  1.00 94.50           N
ATOM   3989  CA  LEU A 502     -26.484 -31.141  17.844  1.00 94.50           C
ATOM   3990  C   LEU A 502     -26.656 -32.062  19.031  1.00 94.50           C
ATOM   3991  CB  LEU A 502     -26.516 -31.922  16.531  1.00 94.50           C
ATOM   3992  O   LEU A 502     -25.688 -32.406  19.719  1.00 94.50           O
ATOM   3993  CG  LEU A 502     -25.359 -32.906  16.297  1.00 94.50           C
ATOM   3994  CD1 LEU A 502     -25.594 -34.219  17.031  1.00 94.50           C
ATOM   3995  CD2 LEU A 502     -25.156 -33.125  14.797  1.00 94.50           C
ATOM   3996  N   LEU A 503     -27.938 -32.500  19.406  1.00 94.88           N
ATOM   3997  CA  LEU A 503     -28.234 -33.500  20.438  1.00 94.88           C
ATOM   3998  C   LEU A 503     -28.000 -34.906  19.922  1.00 94.88           C
ATOM   3999  CB  LEU A 503     -29.688 -33.344  20.922  1.00 94.88           C
ATOM   4000  O   LEU A 503     -27.219 -35.656  20.500  1.00 94.88           O
ATOM   4001  CG  LEU A 503     -29.984 -32.125  21.812  1.00 94.88           C
ATOM   4002  CD1 LEU A 503     -31.484 -32.031  22.062  1.00 94.88           C
ATOM   4003  CD2 LEU A 503     -29.219 -32.250  23.125  1.00 94.88           C
ATOM   4004  N   GLU A 504     -28.562 -35.156  18.766  1.00 94.00           N
ATOM   4005  CA  GLU A 504     -28.469 -36.469  18.125  1.00 94.00           C
ATOM   4006  C   GLU A 504     -29.047 -36.438  16.719  1.00 94.00           C
ATOM   4007  CB  GLU A 504     -29.172 -37.531  18.969  1.00 94.00           C
ATOM   4008  O   GLU A 504     -29.812 -35.531  16.375  1.00 94.00           O
ATOM   4009  CG  GLU A 504     -29.172 -38.906  18.344  1.00 94.00           C
ATOM   4010  CD  GLU A 504     -29.969 -39.938  19.141  1.00 94.00           C
ATOM   4011  OE1 GLU A 504     -30.078 -41.094  18.688  1.00 94.00           O
ATOM   4012  OE2 GLU A 504     -30.500 -39.562  20.203  1.00 94.00           O
ATOM   4013  N   VAL A 505     -28.609 -37.344  15.883  1.00 93.44           N
ATOM   4014  CA  VAL A 505     -29.156 -37.562  14.547  1.00 93.44           C
ATOM   4015  C   VAL A 505     -29.812 -38.938  14.453  1.00 93.44           C
ATOM   4016  CB  VAL A 505     -28.062 -37.406  13.461  1.00 93.44           C
ATOM   4017  O   VAL A 505     -29.188 -39.938  14.812  1.00 93.44           O
ATOM   4018  CG1 VAL A 505     -28.641 -37.688  12.070  1.00 93.44           C
ATOM   4019  CG2 VAL A 505     -27.453 -36.031  13.508  1.00 93.44           C
ATOM   4020  N   SER A 506     -31.141 -39.000  14.164  1.00 91.44           N
ATOM   4021  CA  SER A 506     -31.875 -40.219  13.906  1.00 91.44           C
ATOM   4022  C   SER A 506     -32.438 -40.250  12.492  1.00 91.44           C
ATOM   4023  CB  SER A 506     -33.000 -40.406  14.914  1.00 91.44           C
ATOM   4024  O   SER A 506     -33.406 -39.562  12.188  1.00 91.44           O
ATOM   4025  OG  SER A 506     -33.688 -41.625  14.719  1.00 91.44           O
ATOM   4026  N   GLY A 507     -31.797 -41.125  11.625  1.00 87.69           N
ATOM   4027  CA  GLY A 507     -32.188 -41.156  10.227  1.00 87.69           C
ATOM   4028  C   GLY A 507     -31.891 -39.844   9.500  1.00 87.69           C
```

ATOM	4029	O	GLY	A	507	-30.734	-39.406	9.469	1.00	87.69	O
ATOM	4030	N	SER	A	508	-32.875	-39.156	9.008	1.00	91.31	N
ATOM	4031	CA	SER	A	508	-32.750	-37.906	8.273	1.00	91.31	C
ATOM	4032	C	SER	A	508	-33.125	-36.719	9.148	1.00	91.31	C
ATOM	4033	CB	SER	A	508	-33.625	-37.906	7.020	1.00	91.31	C
ATOM	4034	O	SER	A	508	-33.219	-35.594	8.656	1.00	91.31	O
ATOM	4035	OG	SER	A	508	-33.219	-38.938	6.117	1.00	91.31	O
ATOM	4036	N	GLN	A	509	-33.406	-37.094	10.438	1.00	94.38	N
ATOM	4037	CA	GLN	A	509	-33.812	-36.062	11.383	1.00	94.38	C
ATOM	4038	C	GLN	A	509	-32.656	-35.656	12.305	1.00	94.38	C
ATOM	4039	CB	GLN	A	509	-35.000	-36.500	12.211	1.00	94.38	C
ATOM	4040	O	GLN	A	509	-31.984	-36.531	12.867	1.00	94.38	O
ATOM	4041	CG	GLN	A	509	-36.281	-36.688	11.391	1.00	94.38	C
ATOM	4042	CD	GLN	A	509	-37.469	-37.031	12.250	1.00	94.38	C
ATOM	4043	NE2	GLN	A	509	-38.656	-36.531	11.867	1.00	94.38	N
ATOM	4044	OE1	GLN	A	509	-37.344	-37.750	13.250	1.00	94.38	O
ATOM	4045	N	ILE	A	510	-32.438	-34.469	12.445	1.00	96.44	N
ATOM	4046	CA	ILE	A	510	-31.422	-33.906	13.336	1.00	96.44	C
ATOM	4047	C	ILE	A	510	-32.094	-33.188	14.500	1.00	96.44	C
ATOM	4048	CB	ILE	A	510	-30.469	-32.938	12.578	1.00	96.44	C
ATOM	4049	O	ILE	A	510	-32.875	-32.250	14.289	1.00	96.44	O
ATOM	4050	CG1	ILE	A	510	-29.859	-33.656	11.367	1.00	96.44	C
ATOM	4051	CG2	ILE	A	510	-29.391	-32.406	13.508	1.00	96.44	C
ATOM	4052	CD1	ILE	A	510	-29.312	-32.719	10.297	1.00	96.44	C
ATOM	4053	N	PHE	A	511	-31.766	-33.531	15.727	1.00	96.31	N
ATOM	4054	CA	PHE	A	511	-32.344	-32.938	16.922	1.00	96.31	C
ATOM	4055	C	PHE	A	511	-31.375	-31.938	17.547	1.00	96.31	C
ATOM	4056	CB	PHE	A	511	-32.719	-34.031	17.938	1.00	96.31	C
ATOM	4057	O	PHE	A	511	-30.203	-32.281	17.797	1.00	96.31	O
ATOM	4058	CG	PHE	A	511	-33.750	-35.000	17.438	1.00	96.31	C
ATOM	4059	CD1	PHE	A	511	-35.094	-34.750	17.594	1.00	96.31	C
ATOM	4060	CD2	PHE	A	511	-33.344	-36.188	16.812	1.00	96.31	C
ATOM	4061	CE1	PHE	A	511	-36.062	-35.656	17.141	1.00	96.31	C
ATOM	4062	CE2	PHE	A	511	-34.312	-37.094	16.344	1.00	96.31	C
ATOM	4063	CZ	PHE	A	511	-35.656	-36.812	16.516	1.00	96.31	C
ATOM	4064	N	LEU	A	512	-31.875	-30.734	17.828	1.00	96.12	N
ATOM	4065	CA	LEU	A	512	-31.062	-29.641	18.359	1.00	96.12	C
ATOM	4066	C	LEU	A	512	-31.578	-29.203	19.734	1.00	96.12	C
ATOM	4067	CB	LEU	A	512	-31.078	-28.453	17.406	1.00	96.12	C
ATOM	4068	O	LEU	A	512	-32.781	-29.312	20.016	1.00	96.12	O
ATOM	4069	CG	LEU	A	512	-30.656	-28.734	15.953	1.00	96.12	C
ATOM	4070	CD1	LEU	A	512	-30.938	-27.516	15.070	1.00	96.12	C
ATOM	4071	CD2	LEU	A	512	-29.188	-29.141	15.891	1.00	96.12	C
ATOM	4072	N	PRO	A	513	-30.766	-28.688	20.656	1.00	94.12	N
ATOM	4073	CA	PRO	A	513	-31.172	-28.297	22.016	1.00	94.12	C
ATOM	4074	C	PRO	A	513	-31.844	-26.938	22.062	1.00	94.12	C
ATOM	4075	CB	PRO	A	513	-29.844	-28.281	22.781	1.00	94.12	C
ATOM	4076	O	PRO	A	513	-32.406	-26.562	23.094	1.00	94.12	O
ATOM	4077	CG	PRO	A	513	-28.812	-27.969	21.750	1.00	94.12	C
ATOM	4078	CD	PRO	A	513	-29.234	-28.594	20.438	1.00	94.12	C
ATOM	4079	N	PHE	A	514	-31.766	-26.188	20.984	1.00	92.88	N
ATOM	4080	CA	PHE	A	514	-32.344	-24.844	20.922	1.00	92.88	C
ATOM	4081	C	PHE	A	514	-32.906	-24.547	19.531	1.00	92.88	C
ATOM	4082	CB	PHE	A	514	-31.281	-23.797	21.297	1.00	92.88	C
ATOM	4083	O	PHE	A	514	-32.594	-25.266	18.578	1.00	92.88	O
ATOM	4084	CG	PHE	A	514	-30.016	-23.906	20.516	1.00	92.88	C
ATOM	4085	CD1	PHE	A	514	-28.969	-24.703	20.984	1.00	92.88	C
ATOM	4086	CD2	PHE	A	514	-29.859	-23.234	19.312	1.00	92.88	C
ATOM	4087	CE1	PHE	A	514	-27.781	-24.812	20.266	1.00	92.88	C
ATOM	4088	CE2	PHE	A	514	-28.672	-23.344	18.578	1.00	92.88	C
ATOM	4089	CZ	PHE	A	514	-27.641	-24.125	19.062	1.00	92.88	C
ATOM	4090	N	ARG	A	515	-33.750	-23.562	19.438	1.00	94.50	N
ATOM	4091	CA	ARG	A	515	-34.312	-23.172	18.156	1.00	94.50	C
ATOM	4092	C	ARG	A	515	-33.250	-22.625	17.219	1.00	94.50	C

```
ATOM   4093  CB  ARG A 515     -35.438 -22.141  18.359  1.00 94.50           C
ATOM   4094  O   ARG A 515     -32.438 -21.781  17.625  1.00 94.50           O
ATOM   4095  CG  ARG A 515     -36.000 -21.562  17.062  1.00 94.50           C
ATOM   4096  CD  ARG A 515     -37.125 -20.578  17.312  1.00 94.50           C
ATOM   4097  NE  ARG A 515     -37.750 -20.141  16.078  1.00 94.50           N
ATOM   4098  NH1 ARG A 515     -37.469 -17.891  16.484  1.00 94.50           N
ATOM   4099  NH2 ARG A 515     -38.500 -18.594  14.555  1.00 94.50           N
ATOM   4100  CZ  ARG A 515     -37.906 -18.875  15.703  1.00 94.50           C
ATOM   4101  N   VAL A 516     -33.188 -23.141  15.891  1.00 94.25           N
ATOM   4102  CA  VAL A 516     -32.281 -22.641  14.875  1.00 94.25           C
ATOM   4103  C   VAL A 516     -33.031 -21.891  13.797  1.00 94.25           C
ATOM   4104  CB  VAL A 516     -31.453 -23.797  14.242  1.00 94.25           C
ATOM   4105  O   VAL A 516     -34.188 -22.203  13.523  1.00 94.25           O
ATOM   4106  CG1 VAL A 516     -30.516 -24.422  15.281  1.00 94.25           C
ATOM   4107  CG2 VAL A 516     -32.375 -24.844  13.648  1.00 94.25           C
ATOM   4108  N   ASP A 517     -32.500 -20.828  13.344  1.00 93.94           N
ATOM   4109  CA  ASP A 517     -33.125 -19.984  12.320  1.00 93.94           C
ATOM   4110  C   ASP A 517     -32.812 -20.516  10.914  1.00 93.94           C
ATOM   4111  CB  ASP A 517     -32.625 -18.547  12.453  1.00 93.94           C
ATOM   4112  O   ASP A 517     -32.375 -19.766  10.039  1.00 93.94           O
ATOM   4113  CG  ASP A 517     -33.031 -17.906  13.766  1.00 93.94           C
ATOM   4114  OD1 ASP A 517     -34.250 -17.703  14.008  1.00 93.94           O
ATOM   4115  OD2 ASP A 517     -32.125 -17.594  14.570  1.00 93.94           O
ATOM   4116  N   ALA A 518     -32.938 -21.781  10.578  1.00 94.06           N
ATOM   4117  CA  ALA A 518     -32.750 -22.438   9.289  1.00 94.06           C
ATOM   4118  C   ALA A 518     -34.062 -22.531   8.531  1.00 94.06           C
ATOM   4119  CB  ALA A 518     -32.125 -23.828   9.477  1.00 94.06           C
ATOM   4120  O   ALA A 518     -35.156 -22.516   9.141  1.00 94.06           O
ATOM   4121  N   ARG A 519     -34.031 -22.594   7.191  1.00 94.50           N
ATOM   4122  CA  ARG A 519     -35.188 -22.750   6.328  1.00 94.50           C
ATOM   4123  C   ARG A 519     -35.031 -23.922   5.371  1.00 94.50           C
ATOM   4124  CB  ARG A 519     -35.469 -21.469   5.535  1.00 94.50           C
ATOM   4125  O   ARG A 519     -33.875 -24.328   5.086  1.00 94.50           O
ATOM   4126  CG  ARG A 519     -35.625 -20.234   6.406  1.00 94.50           C
ATOM   4127  CD  ARG A 519     -35.531 -18.953   5.582  1.00 94.50           C
ATOM   4128  NE  ARG A 519     -35.438 -17.766   6.434  1.00 94.50           N
ATOM   4129  NH1 ARG A 519     -35.031 -16.297   4.699  1.00 94.50           N
ATOM   4130  NH2 ARG A 519     -35.125 -15.539   6.855  1.00 94.50           N
ATOM   4131  CZ  ARG A 519     -35.188 -16.547   5.996  1.00 94.50           C
ATOM   4132  N   ALA A 520     -36.125 -24.438   4.934  1.00 94.06           N
ATOM   4133  CA  ALA A 520     -36.062 -25.422   3.865  1.00 94.06           C
ATOM   4134  C   ALA A 520     -35.250 -24.891   2.678  1.00 94.06           C
ATOM   4135  CB  ALA A 520     -37.438 -25.844   3.420  1.00 94.06           C
ATOM   4136  O   ALA A 520     -35.438 -23.734   2.275  1.00 94.06           O
ATOM   4137  N   GLY A 521     -34.250 -25.688   2.127  1.00 92.75           N
ATOM   4138  CA  GLY A 521     -33.406 -25.266   1.024  1.00 92.75           C
ATOM   4139  C   GLY A 521     -32.000 -24.844   1.470  1.00 92.75           C
ATOM   4140  O   GLY A 521     -31.062 -24.875   0.683  1.00 92.75           O
ATOM   4141  N   ASP A 522     -31.922 -24.453   2.789  1.00 95.50           N
ATOM   4142  CA  ASP A 522     -30.609 -24.141   3.369  1.00 95.50           C
ATOM   4143  C   ASP A 522     -29.766 -25.391   3.537  1.00 95.50           C
ATOM   4144  CB  ASP A 522     -30.781 -23.438   4.719  1.00 95.50           C
ATOM   4145  O   ASP A 522     -30.234 -26.500   3.316  1.00 95.50           O
ATOM   4146  CG  ASP A 522     -31.422 -22.062   4.598  1.00 95.50           C
ATOM   4147  OD1 ASP A 522     -31.438 -21.484   3.486  1.00 95.50           O
ATOM   4148  OD2 ASP A 522     -31.922 -21.547   5.621  1.00 95.50           O
ATOM   4149  N   PHE A 523     -28.500 -25.203   3.895  1.00 94.88           N
ATOM   4150  CA  PHE A 523     -27.609 -26.344   4.094  1.00 94.88           C
ATOM   4151  C   PHE A 523     -27.203 -26.453   5.555  1.00 94.88           C
ATOM   4152  CB  PHE A 523     -26.375 -26.219   3.201  1.00 94.88           C
ATOM   4153  O   PHE A 523     -26.984 -25.438   6.227  1.00 94.88           O
ATOM   4154  CG  PHE A 523     -26.688 -26.219   1.729  1.00 94.88           C
ATOM   4155  CD1 PHE A 523     -26.953 -27.406   1.061  1.00 94.88           C
ATOM   4156  CD2 PHE A 523     -26.703 -25.031   1.013  1.00 94.88           C
```

ATOM	4157	CE1	PHE	A	523	-27.250	-27.406	-0.302	1.00	94.88	C
ATOM	4158	CE2	PHE	A	523	-27.000	-25.031	-0.349	1.00	94.88	C
ATOM	4159	CZ	PHE	A	523	-27.266	-26.219	-1.005	1.00	94.88	C
ATOM	4160	N	ILE	A	524	-27.141	-27.719	6.043	1.00	95.38	N
ATOM	4161	CA	ILE	A	524	-26.516	-28.000	7.332	1.00	95.38	C
ATOM	4162	C	ILE	A	524	-25.203	-28.734	7.113	1.00	95.38	C
ATOM	4163	CB	ILE	A	524	-27.453	-28.828	8.242	1.00	95.38	C
ATOM	4164	O	ILE	A	524	-25.125	-29.656	6.293	1.00	95.38	O
ATOM	4165	CG1	ILE	A	524	-26.812	-29.031	9.617	1.00	95.38	C
ATOM	4166	CG2	ILE	A	524	-27.797	-30.172	7.590	1.00	95.38	C
ATOM	4167	CD1	ILE	A	524	-27.781	-29.562	10.672	1.00	95.38	C
ATOM	4168	N	ILE	A	525	-24.156	-28.266	7.773	1.00	95.19	N
ATOM	4169	CA	ILE	A	525	-22.828	-28.844	7.668	1.00	95.19	C
ATOM	4170	C	ILE	A	525	-22.422	-29.438	9.016	1.00	95.19	C
ATOM	4171	CB	ILE	A	525	-21.797	-27.812	7.188	1.00	95.19	C
ATOM	4172	O	ILE	A	525	-22.500	-28.766	10.047	1.00	95.19	O
ATOM	4173	CG1	ILE	A	525	-22.297	-27.094	5.926	1.00	95.19	C
ATOM	4174	CG2	ILE	A	525	-20.438	-28.484	6.938	1.00	95.19	C
ATOM	4175	CD1	ILE	A	525	-21.438	-25.906	5.512	1.00	95.19	C
ATOM	4176	N	VAL	A	526	-21.938	-30.750	8.977	1.00	94.25	N
ATOM	4177	CA	VAL	A	526	-21.469	-31.406	10.188	1.00	94.25	C
ATOM	4178	C	VAL	A	526	-20.062	-31.969	9.945	1.00	94.25	C
ATOM	4179	CB	VAL	A	526	-22.422	-32.531	10.641	1.00	94.25	C
ATOM	4180	O	VAL	A	526	-19.734	-32.344	8.820	1.00	94.25	O
ATOM	4181	CG1	VAL	A	526	-23.797	-31.938	11.008	1.00	94.25	C
ATOM	4182	CG2	VAL	A	526	-22.562	-33.594	9.547	1.00	94.25	C
ATOM	4183	N	ASN	A	527	-19.281	-31.984	11.023	1.00	90.50	N
ATOM	4184	CA	ASN	A	527	-17.953	-32.562	10.938	1.00	90.50	C
ATOM	4185	C	ASN	A	527	-17.984	-34.094	11.062	1.00	90.50	C
ATOM	4186	CB	ASN	A	527	-17.031	-31.969	12.008	1.00	90.50	C
ATOM	4187	O	ASN	A	527	-18.578	-34.625	12.000	1.00	90.50	O
ATOM	4188	CG	ASN	A	527	-16.703	-30.516	11.742	1.00	90.50	C
ATOM	4189	ND2	ASN	A	527	-16.422	-29.766	12.805	1.00	90.50	N
ATOM	4190	OD1	ASN	A	527	-16.688	-30.062	10.594	1.00	90.50	O
ATOM	4191	N	LYS	A	528	-17.359	-34.781	10.062	1.00	86.88	N
ATOM	4192	CA	LYS	A	528	-17.141	-36.219	10.172	1.00	86.88	C
ATOM	4193	C	LYS	A	528	-16.125	-36.531	11.266	1.00	86.88	C
ATOM	4194	CB	LYS	A	528	-16.672	-36.812	8.836	1.00	86.88	C
ATOM	4195	O	LYS	A	528	-15.344	-35.688	11.664	1.00	86.88	O
ATOM	4196	CG	LYS	A	528	-17.734	-36.812	7.754	1.00	86.88	C
ATOM	4197	CD	LYS	A	528	-17.234	-37.469	6.469	1.00	86.88	C
ATOM	4198	CE	LYS	A	528	-18.250	-37.344	5.340	1.00	86.88	C
ATOM	4199	NZ	LYS	A	528	-17.766	-37.969	4.082	1.00	86.88	N
ATOM	4200	N	PRO	A	529	-16.125	-37.781	11.766	1.00	80.44	N
ATOM	4201	CA	PRO	A	529	-15.141	-38.156	12.781	1.00	80.44	C
ATOM	4202	C	PRO	A	529	-13.695	-37.938	12.328	1.00	80.44	C
ATOM	4203	CB	PRO	A	529	-15.406	-39.656	12.984	1.00	80.44	C
ATOM	4204	O	PRO	A	529	-12.820	-37.688	13.156	1.00	80.44	O
ATOM	4205	CG	PRO	A	529	-16.859	-39.812	12.648	1.00	80.44	C
ATOM	4206	CD	PRO	A	529	-17.219	-38.844	11.555	1.00	80.44	C
ATOM	4207	N	ASP	A	530	-13.469	-37.875	11.000	1.00	81.19	N
ATOM	4208	CA	ASP	A	530	-12.117	-37.625	10.492	1.00	81.19	C
ATOM	4209	C	ASP	A	530	-11.836	-36.156	10.367	1.00	81.19	C
ATOM	4210	CB	ASP	A	530	-11.922	-38.344	9.141	1.00	81.19	C
ATOM	4211	O	ASP	A	530	-10.727	-35.750	10.000	1.00	81.19	O
ATOM	4212	CG	ASP	A	530	-12.836	-37.781	8.055	1.00	81.19	C
ATOM	4213	OD1	ASP	A	530	-13.633	-36.875	8.344	1.00	81.19	O
ATOM	4214	OD2	ASP	A	530	-12.750	-38.281	6.910	1.00	81.19	O
ATOM	4215	N	GLY	A	531	-12.867	-35.312	10.695	1.00	78.44	N
ATOM	4216	CA	GLY	A	531	-12.664	-33.875	10.711	1.00	78.44	C
ATOM	4217	C	GLY	A	531	-13.219	-33.188	9.484	1.00	78.44	C
ATOM	4218	O	GLY	A	531	-13.586	-32.000	9.547	1.00	78.44	O
ATOM	4219	N	LYS	A	532	-13.484	-33.906	8.344	1.00	87.12	N
ATOM	4220	CA	LYS	A	532	-13.969	-33.312	7.109	1.00	87.12	C

```
ATOM   4221  C   LYS A 532     -15.461 -32.969   7.203  1.00 87.12           C
ATOM   4222  CB  LYS A 532     -13.703 -34.219   5.914  1.00 87.12           C
ATOM   4223  O   LYS A 532     -16.266 -33.844   7.590  1.00 87.12           O
ATOM   4224  CG  LYS A 532     -12.242 -34.312   5.523  1.00 87.12           C
ATOM   4225  CD  LYS A 532     -12.055 -35.125   4.254  1.00 87.12           C
ATOM   4226  CE  LYS A 532     -10.578 -35.281   3.895  1.00 87.12           C
ATOM   4227  NZ  LYS A 532     -10.391 -36.094   2.654  1.00 87.12           N
ATOM   4228  N   PRO A 533     -15.859 -31.766   6.832  1.00 92.00           N
ATOM   4229  CA  PRO A 533     -17.266 -31.375   6.922  1.00 92.00           C
ATOM   4230  C   PRO A 533     -18.125 -32.000   5.832  1.00 92.00           C
ATOM   4231  CB  PRO A 533     -17.219 -29.844   6.758  1.00 92.00           C
ATOM   4232  O   PRO A 533     -17.641 -32.250   4.723  1.00 92.00           O
ATOM   4233  CG  PRO A 533     -15.953 -29.578   6.004  1.00 92.00           C
ATOM   4234  CD  PRO A 533     -14.977 -30.672   6.320  1.00 92.00           C
ATOM   4235  N   VAL A 534     -19.297 -32.469   6.129  1.00 93.31           N
ATOM   4236  CA  VAL A 534     -20.266 -32.969   5.172  1.00 93.31           C
ATOM   4237  C   VAL A 534     -21.484 -32.062   5.121  1.00 93.31           C
ATOM   4238  CB  VAL A 534     -20.688 -34.438   5.527  1.00 93.31           C
ATOM   4239  O   VAL A 534     -22.094 -31.766   6.156  1.00 93.31           O
ATOM   4240  CG1 VAL A 534     -21.719 -34.938   4.523  1.00 93.31           C
ATOM   4241  CG2 VAL A 534     -19.469 -35.344   5.570  1.00 93.31           C
ATOM   4242  N   LYS A 535     -21.812 -31.625   3.887  1.00 93.31           N
ATOM   4243  CA  LYS A 535     -22.891 -30.688   3.617  1.00 93.31           C
ATOM   4244  C   LYS A 535     -24.141 -31.406   3.133  1.00 93.31           C
ATOM   4245  CB  LYS A 535     -22.438 -29.656   2.582  1.00 93.31           C
ATOM   4246  O   LYS A 535     -24.062 -32.281   2.258  1.00 93.31           O
ATOM   4247  CG  LYS A 535     -23.453 -28.531   2.359  1.00 93.31           C
ATOM   4248  CD  LYS A 535     -22.922 -27.500   1.368  1.00 93.31           C
ATOM   4249  CE  LYS A 535     -23.922 -26.359   1.169  1.00 93.31           C
ATOM   4250  NZ  LYS A 535     -23.375 -25.297   0.288  1.00 93.31           N
ATOM   4251  N   ARG A 536     -25.328 -31.062   3.715  1.00 94.69           N
ATOM   4252  CA  ARG A 536     -26.625 -31.625   3.305  1.00 94.69           C
ATOM   4253  C   ARG A 536     -27.672 -30.531   3.156  1.00 94.69           C
ATOM   4254  CB  ARG A 536     -27.094 -32.688   4.309  1.00 94.69           C
ATOM   4255  O   ARG A 536     -27.672 -29.562   3.914  1.00 94.69           O
ATOM   4256  CG  ARG A 536     -26.219 -33.938   4.355  1.00 94.69           C
ATOM   4257  CD  ARG A 536     -26.328 -34.719   3.068  1.00 94.69           C
ATOM   4258  NE  ARG A 536     -25.516 -35.938   3.127  1.00 94.69           N
ATOM   4259  NH1 ARG A 536     -23.719 -35.062   1.984  1.00 94.69           N
ATOM   4260  NH2 ARG A 536     -23.656 -37.219   2.721  1.00 94.69           N
ATOM   4261  CZ  ARG A 536     -24.297 -36.062   2.609  1.00 94.69           C
ATOM   4262  N   THR A 537     -28.594 -30.750   2.203  1.00 95.75           N
ATOM   4263  CA  THR A 537     -29.688 -29.812   1.985  1.00 95.75           C
ATOM   4264  C   THR A 537     -30.812 -30.047   2.986  1.00 95.75           C
ATOM   4265  CB  THR A 537     -30.234 -29.922   0.552  1.00 95.75           C
ATOM   4266  O   THR A 537     -31.219 -31.188   3.197  1.00 95.75           O
ATOM   4267  CG2 THR A 537     -31.312 -28.875   0.297  1.00 95.75           C
ATOM   4268  OG1 THR A 537     -29.172 -29.719  -0.382  1.00 95.75           O
ATOM   4269  N   ILE A 538     -31.344 -29.016   3.680  1.00 96.56           N
ATOM   4270  CA  ILE A 538     -32.438 -29.094   4.648  1.00 96.56           C
ATOM   4271  C   ILE A 538     -33.750 -29.203   3.922  1.00 96.56           C
ATOM   4272  CB  ILE A 538     -32.438 -27.875   5.594  1.00 96.56           C
ATOM   4273  O   ILE A 538     -34.094 -28.359   3.080  1.00 96.56           O
ATOM   4274  CG1 ILE A 538     -31.141 -27.844   6.422  1.00 96.56           C
ATOM   4275  CG2 ILE A 538     -33.656 -27.891   6.500  1.00 96.56           C
ATOM   4276  CD1 ILE A 538     -30.969 -26.562   7.238  1.00 96.56           C
ATOM   4277  N   SER A 539     -34.469 -30.219   4.156  1.00 96.38           N
ATOM   4278  CA  SER A 539     -35.750 -30.438   3.541  1.00 96.38           C
ATOM   4279  C   SER A 539     -36.844 -29.688   4.293  1.00 96.38           C
ATOM   4280  CB  SER A 539     -36.094 -31.922   3.490  1.00 96.38           C
ATOM   4281  O   SER A 539     -37.750 -29.094   3.682  1.00 96.38           O
ATOM   4282  OG  SER A 539     -36.219 -32.469   4.797  1.00 96.38           O
ATOM   4283  N   SER A 540     -36.875 -29.750   5.566  1.00 96.19           N
ATOM   4284  CA  SER A 540     -37.875 -29.047   6.383  1.00 96.19           C
```

```
ATOM   4285  C   SER A 540     -37.312 -28.766   7.785  1.00 96.19           C
ATOM   4286  CB  SER A 540     -39.156 -29.844   6.473  1.00 96.19           C
ATOM   4287  O   SER A 540     -36.375 -29.438   8.234  1.00 96.19           O
ATOM   4288  OG  SER A 540     -38.938 -31.062   7.160  1.00 96.19           O
ATOM   4289  N   VAL A 541     -37.906 -27.750   8.477  1.00 96.06           N
ATOM   4290  CA  VAL A 541     -37.594 -27.406   9.867  1.00 96.06           C
ATOM   4291  C   VAL A 541     -38.906 -27.438  10.688  1.00 96.06           C
ATOM   4292  CB  VAL A 541     -36.938 -26.031   9.977  1.00 96.06           C
ATOM   4293  O   VAL A 541     -39.938 -26.922  10.258  1.00 96.06           O
ATOM   4294  CG1 VAL A 541     -36.562 -25.719  11.430  1.00 96.06           C
ATOM   4295  CG2 VAL A 541     -35.688 -25.969   9.094  1.00 96.06           C
ATOM   4296  N   SER A 542     -38.938 -28.156  11.875  1.00 95.38           N
ATOM   4297  CA  SER A 542     -40.125 -28.219  12.727  1.00 95.38           C
ATOM   4298  C   SER A 542     -40.594 -26.828  13.133  1.00 95.38           C
ATOM   4299  CB  SER A 542     -39.844 -29.062  13.977  1.00 95.38           C
ATOM   4300  O   SER A 542     -39.812 -25.875  13.070  1.00 95.38           O
ATOM   4301  OG  SER A 542     -38.875 -28.422  14.805  1.00 95.38           O
ATOM   4302  N   ALA A 543     -41.812 -26.641  13.570  1.00 91.44           N
ATOM   4303  CA  ALA A 543     -42.438 -25.375  13.953  1.00 91.44           C
ATOM   4304  C   ALA A 543     -41.656 -24.734  15.109  1.00 91.44           C
ATOM   4305  CB  ALA A 543     -43.906 -25.594  14.344  1.00 91.44           C
ATOM   4306  O   ALA A 543     -41.531 -23.500  15.148  1.00 91.44           O
ATOM   4307  N   ASP A 544     -41.156 -25.562  16.078  1.00 92.62           N
ATOM   4308  CA  ASP A 544     -40.438 -25.016  17.219  1.00 92.62           C
ATOM   4309  C   ASP A 544     -39.000 -24.672  16.875  1.00 92.62           C
ATOM   4310  CB  ASP A 544     -40.500 -25.984  18.406  1.00 92.62           C
ATOM   4311  O   ASP A 544     -38.281 -24.078  17.672  1.00 92.62           O
ATOM   4312  CG  ASP A 544     -39.875 -27.328  18.078  1.00 92.62           C
ATOM   4313  OD1 ASP A 544     -39.312 -27.500  16.969  1.00 92.62           O
ATOM   4314  OD2 ASP A 544     -39.938 -28.234  18.938  1.00 92.62           O
ATOM   4315  N   GLY A 545     -38.500 -25.125  15.594  1.00 93.50           N
ATOM   4316  CA  GLY A 545     -37.156 -24.812  15.109  1.00 93.50           C
ATOM   4317  C   GLY A 545     -36.062 -25.688  15.734  1.00 93.50           C
ATOM   4318  O   GLY A 545     -34.875 -25.406  15.586  1.00 93.50           O
ATOM   4319  N   LYS A 546     -36.375 -26.797  16.375  1.00 95.31           N
ATOM   4320  CA  LYS A 546     -35.438 -27.625  17.125  1.00 95.31           C
ATOM   4321  C   LYS A 546     -35.188 -28.953  16.422  1.00 95.31           C
ATOM   4322  CB  LYS A 546     -35.969 -27.875  18.547  1.00 95.31           C
ATOM   4323  O   LYS A 546     -34.344 -29.734  16.859  1.00 95.31           O
ATOM   4324  CG  LYS A 546     -35.969 -26.625  19.422  1.00 95.31           C
ATOM   4325  CD  LYS A 546     -36.438 -26.953  20.844  1.00 95.31           C
ATOM   4326  CE  LYS A 546     -36.438 -25.719  21.719  1.00 95.31           C
ATOM   4327  NZ  LYS A 546     -36.875 -26.031  23.125  1.00 95.31           N
ATOM   4328  N   THR A 547     -35.906 -29.266  15.344  1.00 96.56           N
ATOM   4329  CA  THR A 547     -35.719 -30.469  14.547  1.00 96.56           C
ATOM   4330  C   THR A 547     -35.562 -30.125  13.070  1.00 96.56           C
ATOM   4331  CB  THR A 547     -36.906 -31.453  14.742  1.00 96.56           C
ATOM   4332  O   THR A 547     -36.438 -29.438  12.500  1.00 96.56           O
ATOM   4333  CG2 THR A 547     -36.625 -32.781  14.031  1.00 96.56           C
ATOM   4334  OG1 THR A 547     -37.062 -31.703  16.141  1.00 96.56           O
ATOM   4335  N   ILE A 548     -34.438 -30.578  12.500  1.00 96.88           N
ATOM   4336  CA  ILE A 548     -34.188 -30.391  11.078  1.00 96.88           C
ATOM   4337  C   ILE A 548     -34.281 -31.734  10.352  1.00 96.88           C
ATOM   4338  CB  ILE A 548     -32.781 -29.766  10.836  1.00 96.88           C
ATOM   4339  O   ILE A 548     -33.781 -32.750  10.844  1.00 96.88           O
ATOM   4340  CG1 ILE A 548     -32.750 -28.328  11.391  1.00 96.88           C
ATOM   4341  CG2 ILE A 548     -32.438 -29.781   9.352  1.00 96.88           C
ATOM   4342  CD1 ILE A 548     -31.391 -27.672  11.297  1.00 96.88           C
ATOM   4343  N   GLU A 549     -34.938 -31.734   9.188  1.00 96.12           N
ATOM   4344  CA  GLU A 549     -34.969 -32.906   8.297  1.00 96.12           C
ATOM   4345  C   GLU A 549     -34.156 -32.656   7.031  1.00 96.12           C
ATOM   4346  CB  GLU A 549     -36.406 -33.250   7.934  1.00 96.12           C
ATOM   4347  O   GLU A 549     -34.312 -31.625   6.367  1.00 96.12           O
ATOM   4348  CG  GLU A 549     -36.562 -34.625   7.309  1.00 96.12           C
```

ATOM	4349	CD	GLU	A	549	-38.031	-35.062	7.219	1.00	96.12	C
ATOM	4350	OE1	GLU	A	549	-38.281	-36.219	6.770	1.00	96.12	O
ATOM	4351	OE2	GLU	A	549	-38.906	-34.281	7.594	1.00	96.12	O
ATOM	4352	N	VAL	A	550	-33.219	-33.594	6.625	1.00	95.50	N
ATOM	4353	CA	VAL	A	550	-32.375	-33.438	5.438	1.00	95.50	C
ATOM	4354	C	VAL	A	550	-32.875	-34.375	4.336	1.00	95.50	C
ATOM	4355	CB	VAL	A	550	-30.891	-33.719	5.750	1.00	95.50	C
ATOM	4356	O	VAL	A	550	-33.562	-35.344	4.613	1.00	95.50	O
ATOM	4357	CG1	VAL	A	550	-30.344	-32.688	6.727	1.00	95.50	C
ATOM	4358	CG2	VAL	A	550	-30.734	-35.125	6.312	1.00	95.50	C
ATOM	4359	N	ASN	A	551	-32.562	-34.062	3.049	1.00	90.00	N
ATOM	4360	CA	ASN	A	551	-33.000	-34.812	1.877	1.00	90.00	C
ATOM	4361	C	ASN	A	551	-32.281	-36.156	1.789	1.00	90.00	C
ATOM	4362	CB	ASN	A	551	-32.750	-34.000	0.600	1.00	90.00	C
ATOM	4363	O	ASN	A	551	-32.875	-37.156	1.350	1.00	90.00	O
ATOM	4364	CG	ASN	A	551	-33.719	-32.844	0.459	1.00	90.00	C
ATOM	4365	ND2	ASN	A	551	-33.281	-31.797	-0.259	1.00	90.00	N
ATOM	4366	OD1	ASN	A	551	-34.812	-32.844	0.993	1.00	90.00	O
ATOM	4367	N	ILE	A	552	-30.953	-36.125	2.154	1.00	89.75	N
ATOM	4368	CA	ILE	A	552	-30.094	-37.312	2.119	1.00	89.75	C
ATOM	4369	C	ILE	A	552	-29.312	-37.406	3.426	1.00	89.75	C
ATOM	4370	CB	ILE	A	552	-29.125	-37.250	0.917	1.00	89.75	C
ATOM	4371	O	ILE	A	552	-28.750	-36.406	3.904	1.00	89.75	O
ATOM	4372	CG1	ILE	A	552	-29.906	-37.156	-0.398	1.00	89.75	C
ATOM	4373	CG2	ILE	A	552	-28.203	-38.469	0.915	1.00	89.75	C
ATOM	4374	CD1	ILE	A	552	-29.031	-36.875	-1.615	1.00	89.75	C
ATOM	4375	N	GLY	A	553	-29.422	-38.688	4.109	1.00	87.31	N
ATOM	4376	CA	GLY	A	553	-28.641	-38.875	5.324	1.00	87.31	C
ATOM	4377	C	GLY	A	553	-27.172	-38.594	5.137	1.00	87.31	C
ATOM	4378	O	GLY	A	553	-26.688	-38.438	4.004	1.00	87.31	O
ATOM	4379	N	PHE	A	554	-26.344	-38.469	6.176	1.00	88.94	N
ATOM	4380	CA	PHE	A	554	-24.938	-38.094	6.141	1.00	88.94	C
ATOM	4381	C	PHE	A	554	-24.078	-39.281	5.699	1.00	88.94	C
ATOM	4382	CB	PHE	A	554	-24.469	-37.594	7.512	1.00	88.94	C
ATOM	4383	O	PHE	A	554	-22.953	-39.094	5.234	1.00	88.94	O
ATOM	4384	CG	PHE	A	554	-24.969	-36.219	7.844	1.00	88.94	C
ATOM	4385	CD1	PHE	A	554	-24.375	-35.094	7.270	1.00	88.94	C
ATOM	4386	CD2	PHE	A	554	-26.016	-36.031	8.742	1.00	88.94	C
ATOM	4387	CE1	PHE	A	554	-24.828	-33.812	7.578	1.00	88.94	C
ATOM	4388	CE2	PHE	A	554	-26.469	-34.750	9.055	1.00	88.94	C
ATOM	4389	CZ	PHE	A	554	-25.875	-33.656	8.469	1.00	88.94	C
ATOM	4390	N	GLY	A	555	-24.609	-40.531	5.656	1.00	87.25	N
ATOM	4391	CA	GLY	A	555	-23.875	-41.719	5.215	1.00	87.25	C
ATOM	4392	C	GLY	A	555	-22.938	-42.250	6.266	1.00	87.25	C
ATOM	4393	O	GLY	A	555	-22.062	-43.062	5.961	1.00	87.25	O
ATOM	4394	N	PHE	A	556	-22.906	-41.719	7.539	1.00	88.88	N
ATOM	4395	CA	PHE	A	556	-22.172	-42.188	8.695	1.00	88.88	C
ATOM	4396	C	PHE	A	556	-22.891	-41.875	9.992	1.00	88.88	C
ATOM	4397	CB	PHE	A	556	-20.766	-41.594	8.719	1.00	88.88	C
ATOM	4398	O	PHE	A	556	-23.750	-40.969	10.016	1.00	88.88	O
ATOM	4399	CG	PHE	A	556	-20.734	-40.094	8.852	1.00	88.88	C
ATOM	4400	CD1	PHE	A	556	-20.812	-39.281	7.730	1.00	88.88	C
ATOM	4401	CD2	PHE	A	556	-20.656	-39.500	10.109	1.00	88.88	C
ATOM	4402	CE1	PHE	A	556	-20.797	-37.875	7.859	1.00	88.88	C
ATOM	4403	CE2	PHE	A	556	-20.641	-38.125	10.242	1.00	88.88	C
ATOM	4404	CZ	PHE	A	556	-20.703	-37.312	9.117	1.00	88.88	C
ATOM	4405	N	PRO	A	557	-22.672	-42.656	11.055	1.00	89.88	N
ATOM	4406	CA	PRO	A	557	-23.297	-42.344	12.344	1.00	89.88	C
ATOM	4407	C	PRO	A	557	-22.766	-41.062	12.977	1.00	89.88	C
ATOM	4408	CB	PRO	A	557	-22.953	-43.562	13.195	1.00	89.88	C
ATOM	4409	O	PRO	A	557	-21.625	-41.031	13.445	1.00	89.88	O
ATOM	4410	CG	PRO	A	557	-21.703	-44.125	12.578	1.00	89.88	C
ATOM	4411	CD	PRO	A	557	-21.688	-43.750	11.125	1.00	89.88	C
ATOM	4412	N	VAL	A	558	-23.547	-39.906	12.938	1.00	92.44	N

```
ATOM   4413  CA  VAL A 558     -23.172 -38.625  13.531  1.00 92.44           C
ATOM   4414  C   VAL A 558     -23.250 -38.719  15.047  1.00 92.44           C
ATOM   4415  CB  VAL A 558     -24.078 -37.469  13.016  1.00 92.44           C
ATOM   4416  O   VAL A 558     -24.281 -39.125  15.602  1.00 92.44           O
ATOM   4417  CG1 VAL A 558     -23.641 -36.156  13.633  1.00 92.44           C
ATOM   4418  CG2 VAL A 558     -24.031 -37.406  11.492  1.00 92.44           C
ATOM   4419  N   LYS A 559     -22.141 -38.438  15.805  1.00 91.69           N
ATOM   4420  CA  LYS A 559     -22.094 -38.531  17.266  1.00 91.69           C
ATOM   4421  C   LYS A 559     -22.812 -37.344  17.922  1.00 91.69           C
ATOM   4422  CB  LYS A 559     -20.641 -38.594  17.750  1.00 91.69           C
ATOM   4423  O   LYS A 559     -22.859 -36.250  17.359  1.00 91.69           O
ATOM   4424  CG  LYS A 559     -19.875 -39.812  17.250  1.00 91.69           C
ATOM   4425  CD  LYS A 559     -20.359 -41.094  17.938  1.00 91.69           C
ATOM   4426  CE  LYS A 559     -19.531 -42.281  17.516  1.00 91.69           C
ATOM   4427  NZ  LYS A 559     -20.062 -43.562  18.094  1.00 91.69           N
ATOM   4428  N   PRO A 560     -23.484 -37.688  19.141  1.00 92.06           N
ATOM   4429  CA  PRO A 560     -24.094 -36.562  19.859  1.00 92.06           C
ATOM   4430  C   PRO A 560     -23.109 -35.469  20.188  1.00 92.06           C
ATOM   4431  CB  PRO A 560     -24.609 -37.219  21.141  1.00 92.06           C
ATOM   4432  O   PRO A 560     -21.906 -35.719  20.344  1.00 92.06           O
ATOM   4433  CG  PRO A 560     -24.828 -38.656  20.781  1.00 92.06           C
ATOM   4434  CD  PRO A 560     -23.797 -39.062  19.750  1.00 92.06           C
ATOM   4435  N   ASN A 561     -23.484 -34.156  20.125  1.00 91.38           N
ATOM   4436  CA  ASN A 561     -22.734 -32.969  20.500  1.00 91.38           C
ATOM   4437  C   ASN A 561     -21.750 -32.562  19.391  1.00 91.38           C
ATOM   4438  CB  ASN A 561     -21.984 -33.188  21.812  1.00 91.38           C
ATOM   4439  O   ASN A 561     -20.828 -31.781  19.641  1.00 91.38           O
ATOM   4440  CG  ASN A 561     -22.922 -33.188  23.016  1.00 91.38           C
ATOM   4441  ND2 ASN A 561     -22.609 -34.031  24.000  1.00 91.38           N
ATOM   4442  OD1 ASN A 561     -23.922 -32.469  23.047  1.00 91.38           O
ATOM   4443  N   THR A 562     -21.938 -33.125  18.234  1.00 92.81           N
ATOM   4444  CA  THR A 562     -21.141 -32.719  17.078  1.00 92.81           C
ATOM   4445  C   THR A 562     -21.453 -31.281  16.688  1.00 92.81           C
ATOM   4446  CB  THR A 562     -21.422 -33.656  15.867  1.00 92.81           C
ATOM   4447  O   THR A 562     -22.609 -30.844  16.734  1.00 92.81           O
ATOM   4448  CG2 THR A 562     -20.609 -33.219  14.648  1.00 92.81           C
ATOM   4449  OG1 THR A 562     -21.047 -35.000  16.219  1.00 92.81           O
ATOM   4450  N   VAL A 563     -20.484 -30.609  16.375  1.00 93.12           N
ATOM   4451  CA  VAL A 563     -20.641 -29.219  15.992  1.00 93.12           C
ATOM   4452  C   VAL A 563     -21.297 -29.141  14.617  1.00 93.12           C
ATOM   4453  CB  VAL A 563     -19.281 -28.484  15.977  1.00 93.12           C
ATOM   4454  O   VAL A 563     -21.000 -29.938  13.727  1.00 93.12           O
ATOM   4455  CG1 VAL A 563     -19.453 -27.062  15.430  1.00 93.12           C
ATOM   4456  CG2 VAL A 563     -18.672 -28.453  17.375  1.00 93.12           C
ATOM   4457  N   PHE A 564     -22.219 -28.172  14.477  1.00 94.75           N
ATOM   4458  CA  PHE A 564     -22.859 -27.969  13.180  1.00 94.75           C
ATOM   4459  C   PHE A 564     -22.891 -26.484  12.828  1.00 94.75           C
ATOM   4460  CB  PHE A 564     -24.297 -28.531  13.188  1.00 94.75           C
ATOM   4461  O   PHE A 564     -22.750 -25.625  13.703  1.00 94.75           O
ATOM   4462  CG  PHE A 564     -25.281 -27.656  13.914  1.00 94.75           C
ATOM   4463  CD1 PHE A 564     -25.500 -27.828  15.273  1.00 94.75           C
ATOM   4464  CD2 PHE A 564     -25.984 -26.672  13.234  1.00 94.75           C
ATOM   4465  CE1 PHE A 564     -26.422 -27.016  15.953  1.00 94.75           C
ATOM   4466  CE2 PHE A 564     -26.906 -25.859  13.906  1.00 94.75           C
ATOM   4467  CZ  PHE A 564     -27.125 -26.047  15.266  1.00 94.75           C
ATOM   4468  N   ALA A 565     -23.031 -26.172  11.562  1.00 96.38           N
ATOM   4469  CA  ALA A 565     -23.250 -24.828  11.031  1.00 96.38           C
ATOM   4470  C   ALA A 565     -24.328 -24.828   9.938  1.00 96.38           C
ATOM   4471  CB  ALA A 565     -21.953 -24.250  10.492  1.00 96.38           C
ATOM   4472  O   ALA A 565     -24.406 -25.766   9.148  1.00 96.38           O
ATOM   4473  N   ILE A 566     -25.109 -23.844   9.938  1.00 95.62           N
ATOM   4474  CA  ILE A 566     -26.156 -23.703   8.922  1.00 95.62           C
ATOM   4475  C   ILE A 566     -25.703 -22.672   7.883  1.00 95.62           C
ATOM   4476  CB  ILE A 566     -27.500 -23.266   9.555  1.00 95.62           C
```

```
ATOM   4477  O   ILE A 566     -25.375 -21.531   8.227  1.00 95.62           O
ATOM   4478  CG1 ILE A 566     -27.984 -24.328  10.555  1.00 95.62           C
ATOM   4479  CG2 ILE A 566     -28.547 -23.031   8.469  1.00 95.62           C
ATOM   4480  CD1 ILE A 566     -28.359 -25.656   9.914  1.00 95.62           C
ATOM   4481  N   ASP A 567     -25.594 -23.062   6.617  1.00 94.38           N
ATOM   4482  CA  ASP A 567     -25.297 -22.219   5.465  1.00 94.38           C
ATOM   4483  C   ASP A 567     -26.578 -21.812   4.738  1.00 94.38           C
ATOM   4484  CB  ASP A 567     -24.359 -22.953   4.500  1.00 94.38           C
ATOM   4485  O   ASP A 567     -27.188 -22.625   4.043  1.00 94.38           O
ATOM   4486  CG  ASP A 567     -23.781 -22.031   3.438  1.00 94.38           C
ATOM   4487  OD1 ASP A 567     -24.141 -20.844   3.391  1.00 94.38           O
ATOM   4488  OD2 ASP A 567     -22.938 -22.500   2.637  1.00 94.38           O
ATOM   4489  N   ARG A 568     -26.984 -20.625   4.871  1.00 92.06           N
ATOM   4490  CA  ARG A 568     -28.219 -20.109   4.293  1.00 92.06           C
ATOM   4491  C   ARG A 568     -27.984 -19.562   2.887  1.00 92.06           C
ATOM   4492  CB  ARG A 568     -28.812 -19.016   5.180  1.00 92.06           C
ATOM   4493  O   ARG A 568     -26.828 -19.391   2.467  1.00 92.06           O
ATOM   4494  CG  ARG A 568     -29.375 -19.531   6.500  1.00 92.06           C
ATOM   4495  CD  ARG A 568     -30.250 -18.500   7.184  1.00 92.06           C
ATOM   4496  NE  ARG A 568     -31.438 -18.188   6.395  1.00 92.06           N
ATOM   4497  NH1 ARG A 568     -32.219 -16.547   7.820  1.00 92.06           N
ATOM   4498  NH2 ARG A 568     -33.406 -17.078   5.922  1.00 92.06           N
ATOM   4499  CZ  ARG A 568     -32.344 -17.266   6.715  1.00 92.06           C
ATOM   4500  N   THR A 569     -29.062 -19.312   2.154  1.00 86.44           N
ATOM   4501  CA  THR A 569     -29.000 -18.812   0.784  1.00 86.44           C
ATOM   4502  C   THR A 569     -28.719 -17.312   0.766  1.00 86.44           C
ATOM   4503  CB  THR A 569     -30.297 -19.109   0.017  1.00 86.44           C
ATOM   4504  O   THR A 569     -28.281 -16.766  -0.249  1.00 86.44           O
ATOM   4505  CG2 THR A 569     -30.469 -20.609  -0.204  1.00 86.44           C
ATOM   4506  OG1 THR A 569     -31.406 -18.625   0.769  1.00 86.44           O
ATOM   4507  N   ASP A 570     -28.906 -16.578   1.912  1.00 87.56           N
ATOM   4508  CA  ASP A 570     -28.719 -15.125   1.956  1.00 87.56           C
ATOM   4509  C   ASP A 570     -27.531 -14.750   2.826  1.00 87.56           C
ATOM   4510  CB  ASP A 570     -29.984 -14.445   2.477  1.00 87.56           C
ATOM   4511  O   ASP A 570     -27.172 -13.570   2.934  1.00 87.56           O
ATOM   4512  CG  ASP A 570     -30.469 -15.016   3.799  1.00 87.56           C
ATOM   4513  OD1 ASP A 570     -29.953 -16.078   4.230  1.00 87.56           O
ATOM   4514  OD2 ASP A 570     -31.375 -14.406   4.414  1.00 87.56           O
ATOM   4515  N   ILE A 571     -26.875 -15.711   3.555  1.00 91.38           N
ATOM   4516  CA  ILE A 571     -25.703 -15.469   4.391  1.00 91.38           C
ATOM   4517  C   ILE A 571     -24.719 -16.625   4.250  1.00 91.38           C
ATOM   4518  CB  ILE A 571     -26.094 -15.273   5.871  1.00 91.38           C
ATOM   4519  O   ILE A 571     -25.078 -17.781   4.508  1.00 91.38           O
ATOM   4520  CG1 ILE A 571     -27.047 -14.078   6.020  1.00 91.38           C
ATOM   4521  CG2 ILE A 571     -24.844 -15.094   6.742  1.00 91.38           C
ATOM   4522  CD1 ILE A 571     -27.672 -13.961   7.402  1.00 91.38           C
ATOM   4523  N   ALA A 572     -23.500 -16.281   3.842  1.00 92.62           N
ATOM   4524  CA  ALA A 572     -22.484 -17.312   3.629  1.00 92.62           C
ATOM   4525  C   ALA A 572     -21.594 -17.469   4.859  1.00 92.62           C
ATOM   4526  CB  ALA A 572     -21.641 -16.969   2.406  1.00 92.62           C
ATOM   4527  O   ALA A 572     -21.391 -16.516   5.609  1.00 92.62           O
ATOM   4528  N   LEU A 573     -21.094 -18.703   5.078  1.00 93.81           N
ATOM   4529  CA  LEU A 573     -20.078 -18.938   6.102  1.00 93.81           C
ATOM   4530  C   LEU A 573     -18.750 -18.281   5.707  1.00 93.81           C
ATOM   4531  CB  LEU A 573     -19.875 -20.438   6.336  1.00 93.81           C
ATOM   4532  O   LEU A 573     -18.391 -18.266   4.531  1.00 93.81           O
ATOM   4533  CG  LEU A 573     -21.078 -21.203   6.898  1.00 93.81           C
ATOM   4534  CD1 LEU A 573     -20.766 -22.688   6.957  1.00 93.81           C
ATOM   4535  CD2 LEU A 573     -21.453 -20.672   8.273  1.00 93.81           C
ATOM   4536  N   GLN A 574     -18.094 -17.719   6.789  1.00 94.44           N
ATOM   4537  CA  GLN A 574     -16.750 -17.172   6.535  1.00 94.44           C
ATOM   4538  C   GLN A 574     -15.742 -18.297   6.309  1.00 94.44           C
ATOM   4539  CB  GLN A 574     -16.312 -16.281   7.691  1.00 94.44           C
ATOM   4540  O   GLN A 574     -15.734 -19.281   7.035  1.00 94.44           O
```

ATOM	4541	CG	GLN	A	574	-17.219	-15.094	7.938	1.00	94.44	C
ATOM	4542	CD	GLN	A	574	-16.844	-14.297	9.172	1.00	94.44	C
ATOM	4543	NE2	GLN	A	574	-17.438	-14.641	10.312	1.00	94.44	N
ATOM	4544	OE1	GLN	A	574	-16.016	-13.391	9.109	1.00	94.44	O
ATOM	4545	N	GLN	A	575	-14.969	-18.156	5.242	1.00	94.88	N
ATOM	4546	CA	GLN	A	575	-13.898	-19.109	4.977	1.00	94.88	C
ATOM	4547	C	GLN	A	575	-12.523	-18.453	5.133	1.00	94.88	C
ATOM	4548	CB	GLN	A	575	-14.039	-19.703	3.572	1.00	94.88	C
ATOM	4549	O	GLN	A	575	-12.305	-17.344	4.652	1.00	94.88	O
ATOM	4550	CG	GLN	A	575	-15.320	-20.500	3.371	1.00	94.88	C
ATOM	4551	CD	GLN	A	575	-15.336	-21.266	2.055	1.00	94.88	C
ATOM	4552	NE2	GLN	A	575	-16.516	-21.359	1.442	1.00	94.88	N
ATOM	4553	OE1	GLN	A	575	-14.305	-21.750	1.592	1.00	94.88	O
ATOM	4554	N	TYR	A	576	-11.641	-19.172	5.871	1.00	96.06	N
ATOM	4555	CA	TYR	A	576	-10.297	-18.672	6.160	1.00	96.06	C
ATOM	4556	C	TYR	A	576	-9.250	-19.766	5.914	1.00	96.06	C
ATOM	4557	CB	TYR	A	576	-10.203	-18.188	7.609	1.00	96.06	C
ATOM	4558	O	TYR	A	576	-9.531	-20.953	6.066	1.00	96.06	O
ATOM	4559	CG	TYR	A	576	-10.891	-16.859	7.848	1.00	96.06	C
ATOM	4560	CD1	TYR	A	576	-10.164	-15.672	7.844	1.00	96.06	C
ATOM	4561	CD2	TYR	A	576	-12.258	-16.797	8.078	1.00	96.06	C
ATOM	4562	CE1	TYR	A	576	-10.789	-14.453	8.062	1.00	96.06	C
ATOM	4563	CE2	TYR	A	576	-12.891	-15.586	8.305	1.00	96.06	C
ATOM	4564	OH	TYR	A	576	-12.773	-13.211	8.508	1.00	96.06	O
ATOM	4565	CZ	TYR	A	576	-12.156	-14.414	8.289	1.00	96.06	C
ATOM	4566	N	VAL	A	577	-8.078	-19.312	5.410	1.00	93.94	N
ATOM	4567	CA	VAL	A	577	-6.941	-20.219	5.254	1.00	93.94	C
ATOM	4568	C	VAL	A	577	-6.023	-20.109	6.465	1.00	93.94	C
ATOM	4569	CB	VAL	A	577	-6.156	-19.922	3.957	1.00	93.94	C
ATOM	4570	O	VAL	A	577	-5.590	-19.016	6.824	1.00	93.94	O
ATOM	4571	CG1	VAL	A	577	-4.910	-20.812	3.869	1.00	93.94	C
ATOM	4572	CG2	VAL	A	577	-7.047	-20.141	2.732	1.00	93.94	C
ATOM	4573	N	VAL	A	578	-5.727	-21.203	7.094	1.00	93.06	N
ATOM	4574	CA	VAL	A	578	-4.922	-21.266	8.312	1.00	93.06	C
ATOM	4575	C	VAL	A	578	-3.471	-20.922	7.984	1.00	93.06	C
ATOM	4576	CB	VAL	A	578	-5.008	-22.656	8.977	1.00	93.06	C
ATOM	4577	O	VAL	A	578	-2.865	-21.531	7.098	1.00	93.06	O
ATOM	4578	CG1	VAL	A	578	-4.098	-22.719	10.203	1.00	93.06	C
ATOM	4579	CG2	VAL	A	578	-6.449	-22.969	9.367	1.00	93.06	C
ATOM	4580	N	THR	A	579	-2.895	-19.953	8.711	1.00	91.50	N
ATOM	4581	CA	THR	A	579	-1.524	-19.531	8.445	1.00	91.50	C
ATOM	4582	C	THR	A	579	-0.619	-19.875	9.625	1.00	91.50	C
ATOM	4583	CB	THR	A	579	-1.455	-18.016	8.156	1.00	91.50	C
ATOM	4584	O	THR	A	579	0.600	-19.984	9.469	1.00	91.50	O
ATOM	4585	CG2	THR	A	579	-2.176	-17.672	6.859	1.00	91.50	C
ATOM	4586	OG1	THR	A	579	-2.068	-17.312	9.234	1.00	91.50	O
ATOM	4587	N	LYS	A	580	-1.277	-20.031	10.766	1.00	92.75	N
ATOM	4588	CA	LYS	A	580	-0.471	-20.297	11.953	1.00	92.75	C
ATOM	4589	C	LYS	A	580	-1.269	-21.078	13.000	1.00	92.75	C
ATOM	4590	CB	LYS	A	580	0.045	-18.984	12.562	1.00	92.75	C
ATOM	4591	O	LYS	A	580	-2.441	-20.781	13.242	1.00	92.75	O
ATOM	4592	CG	LYS	A	580	1.059	-19.172	13.680	1.00	92.75	C
ATOM	4593	CD	LYS	A	580	1.604	-17.844	14.172	1.00	92.75	C
ATOM	4594	CE	LYS	A	580	2.594	-18.031	15.312	1.00	92.75	C
ATOM	4595	NZ	LYS	A	580	3.162	-16.734	15.773	1.00	92.75	N
ATOM	4596	N	ILE	A	581	-0.613	-22.125	13.609	1.00	92.56	N
ATOM	4597	CA	ILE	A	581	-1.187	-22.875	14.719	1.00	92.56	C
ATOM	4598	C	ILE	A	581	-0.181	-22.953	15.859	1.00	92.56	C
ATOM	4599	CB	ILE	A	581	-1.606	-24.297	14.273	1.00	92.56	C
ATOM	4600	O	ILE	A	581	0.948	-23.422	15.672	1.00	92.56	O
ATOM	4601	CG1	ILE	A	581	-2.604	-24.219	13.109	1.00	92.56	C
ATOM	4602	CG2	ILE	A	581	-2.197	-25.078	15.453	1.00	92.56	C
ATOM	4603	CD1	ILE	A	581	-2.922	-25.578	12.477	1.00	92.56	C
ATOM	4604	N	ASP	A	582	-0.613	-22.453	17.047	1.00	93.06	N

ATOM	4605	CA	ASP	A	582	0.227	-22.500	18.250	1.00	93.06	C
ATOM	4606	C	ASP	A	582	-0.428	-23.328	19.344	1.00	93.06	C
ATOM	4607	CB	ASP	A	582	0.514	-21.094	18.750	1.00	93.06	C
ATOM	4608	O	ASP	A	582	-1.582	-23.094	19.703	1.00	93.06	O
ATOM	4609	CG	ASP	A	582	1.448	-20.312	17.844	1.00	93.06	C
ATOM	4610	OD1	ASP	A	582	2.525	-20.828	17.484	1.00	93.06	O
ATOM	4611	OD2	ASP	A	582	1.105	-19.156	17.484	1.00	93.06	O
ATOM	4612	N	LYS	A	583	0.396	-24.359	19.812	1.00	91.88	N
ATOM	4613	CA	LYS	A	583	-0.070	-25.125	20.953	1.00	91.88	C
ATOM	4614	C	LYS	A	583	0.276	-24.422	22.266	1.00	91.88	C
ATOM	4615	CB	LYS	A	583	0.534	-26.531	20.938	1.00	91.88	C
ATOM	4616	O	LYS	A	583	1.394	-23.938	22.438	1.00	91.88	O
ATOM	4617	CG	LYS	A	583	0.026	-27.438	22.062	1.00	91.88	C
ATOM	4618	CD	LYS	A	583	0.699	-28.797	22.016	1.00	91.88	C
ATOM	4619	CE	LYS	A	583	0.265	-29.672	23.188	1.00	91.88	C
ATOM	4620	NZ	LYS	A	583	0.941	-31.000	23.172	1.00	91.88	N
ATOM	4621	N	GLY	A	584	-0.793	-24.188	23.141	1.00	87.00	N
ATOM	4622	CA	GLY	A	584	-0.549	-23.562	24.422	1.00	87.00	C
ATOM	4623	C	GLY	A	584	0.379	-24.359	25.312	1.00	87.00	C
ATOM	4624	O	GLY	A	584	0.629	-25.531	25.062	1.00	87.00	O
ATOM	4625	N	ASP	A	585	1.113	-23.766	26.188	1.00	83.81	N
ATOM	4626	CA	ASP	A	585	1.993	-24.375	27.188	1.00	83.81	C
ATOM	4627	C	ASP	A	585	1.309	-24.453	28.547	1.00	83.81	C
ATOM	4628	CB	ASP	A	585	3.299	-23.578	27.297	1.00	83.81	C
ATOM	4629	O	ASP	A	585	0.283	-23.812	28.766	1.00	83.81	O
ATOM	4630	CG	ASP	A	585	4.133	-23.625	26.031	1.00	83.81	C
ATOM	4631	OD1	ASP	A	585	3.988	-24.594	25.234	1.00	83.81	O
ATOM	4632	OD2	ASP	A	585	4.941	-22.703	25.812	1.00	83.81	O
ATOM	4633	N	ASP	A	586	1.662	-25.453	29.375	1.00	86.81	N
ATOM	4634	CA	ASP	A	586	1.284	-25.609	30.766	1.00	86.81	C
ATOM	4635	C	ASP	A	586	-0.220	-25.828	30.906	1.00	86.81	C
ATOM	4636	CB	ASP	A	586	1.717	-24.391	31.578	1.00	86.81	C
ATOM	4637	O	ASP	A	586	-0.751	-26.844	30.453	1.00	86.81	O
ATOM	4638	CG	ASP	A	586	3.223	-24.188	31.594	1.00	86.81	C
ATOM	4639	OD1	ASP	A	586	3.971	-25.188	31.594	1.00	86.81	O
ATOM	4640	OD2	ASP	A	586	3.664	-23.016	31.609	1.00	86.81	O
ATOM	4641	N	ASP	A	587	-0.951	-24.766	31.500	1.00	79.81	N
ATOM	4642	CA	ASP	A	587	-2.367	-24.875	31.828	1.00	79.81	C
ATOM	4643	C	ASP	A	587	-3.238	-24.812	30.578	1.00	79.81	C
ATOM	4644	CB	ASP	A	587	-2.770	-23.766	32.812	1.00	79.81	C
ATOM	4645	O	ASP	A	587	-4.422	-25.156	30.625	1.00	79.81	O
ATOM	4646	CG	ASP	A	587	-2.102	-23.906	34.156	1.00	79.81	C
ATOM	4647	OD1	ASP	A	587	-1.711	-25.031	34.531	1.00	79.81	O
ATOM	4648	OD2	ASP	A	587	-1.966	-22.875	34.875	1.00	79.81	O
ATOM	4649	N	GLU	A	588	-2.684	-24.484	29.375	1.00	83.19	N
ATOM	4650	CA	GLU	A	588	-3.393	-24.391	28.094	1.00	83.19	C
ATOM	4651	C	GLU	A	588	-2.906	-25.453	27.125	1.00	83.19	C
ATOM	4652	CB	GLU	A	588	-3.229	-23.000	27.484	1.00	83.19	C
ATOM	4653	O	GLU	A	588	-2.855	-25.219	25.906	1.00	83.19	O
ATOM	4654	CG	GLU	A	588	-3.898	-21.891	28.281	1.00	83.19	C
ATOM	4655	CD	GLU	A	588	-3.867	-20.547	27.594	1.00	83.19	C
ATOM	4656	OE1	GLU	A	588	-4.480	-19.578	28.109	1.00	83.19	O
ATOM	4657	OE2	GLU	A	588	-3.227	-20.438	26.516	1.00	83.19	O
ATOM	4658	N	GLU	A	589	-2.322	-26.656	27.781	1.00	85.94	N
ATOM	4659	CA	GLU	A	589	-1.722	-27.703	26.969	1.00	85.94	C
ATOM	4660	C	GLU	A	589	-2.711	-28.219	25.922	1.00	85.94	C
ATOM	4661	CB	GLU	A	589	-1.216	-28.844	27.844	1.00	85.94	C
ATOM	4662	O	GLU	A	589	-2.312	-28.688	24.859	1.00	85.94	O
ATOM	4663	CG	GLU	A	589	-0.519	-29.953	27.062	1.00	85.94	C
ATOM	4664	CD	GLU	A	589	-0.027	-31.094	27.938	1.00	85.94	C
ATOM	4665	OE1	GLU	A	589	0.657	-32.000	27.422	1.00	85.94	O
ATOM	4666	OE2	GLU	A	589	-0.329	-31.062	29.156	1.00	85.94	O
ATOM	4667	N	PHE	A	590	-4.039	-28.141	26.125	1.00	88.81	N
ATOM	4668	CA	PHE	A	590	-5.023	-28.672	25.188	1.00	88.81	C

```
ATOM   4669  C   PHE A 590      -5.707 -27.547  24.422  1.00 88.81           C
ATOM   4670  CB  PHE A 590      -6.070 -29.516  25.938  1.00 88.81           C
ATOM   4671  O   PHE A 590      -6.781 -27.734  23.859  1.00 88.81           O
ATOM   4672  CG  PHE A 590      -5.496 -30.719  26.625  1.00 88.81           C
ATOM   4673  CD1 PHE A 590      -4.914 -31.750  25.906  1.00 88.81           C
ATOM   4674  CD2 PHE A 590      -5.543 -30.812  28.016  1.00 88.81           C
ATOM   4675  CE1 PHE A 590      -4.383 -32.875  26.547  1.00 88.81           C
ATOM   4676  CE2 PHE A 590      -5.012 -31.922  28.672  1.00 88.81           C
ATOM   4677  CZ  PHE A 590      -4.438 -32.938  27.938  1.00 88.81           C
ATOM   4678  N   THR A 591      -5.109 -26.391  24.469  1.00 94.12           N
ATOM   4679  CA  THR A 591      -5.605 -25.234  23.734  1.00 94.12           C
ATOM   4680  C   THR A 591      -4.691 -24.906  22.547  1.00 94.12           C
ATOM   4681  CB  THR A 591      -5.719 -24.000  24.641  1.00 94.12           C
ATOM   4682  O   THR A 591      -3.467 -24.844  22.703  1.00 94.12           O
ATOM   4683  CG2 THR A 591      -6.297 -22.797  23.891  1.00 94.12           C
ATOM   4684  OG1 THR A 591      -6.578 -24.312  25.750  1.00 94.12           O
ATOM   4685  N   TYR A 592      -5.320 -24.750  21.484  1.00 95.31           N
ATOM   4686  CA  TYR A 592      -4.605 -24.406  20.250  1.00 95.31           C
ATOM   4687  C   TYR A 592      -5.055 -23.062  19.719  1.00 95.31           C
ATOM   4688  CB  TYR A 592      -4.812 -25.484  19.188  1.00 95.31           C
ATOM   4689  O   TYR A 592      -6.250 -22.828  19.500  1.00 95.31           O
ATOM   4690  CG  TYR A 592      -4.230 -26.828  19.562  1.00 95.31           C
ATOM   4691  CD1 TYR A 592      -3.004 -27.250  19.047  1.00 95.31           C
ATOM   4692  CD2 TYR A 592      -4.898 -27.688  20.438  1.00 95.31           C
ATOM   4693  CE1 TYR A 592      -2.461 -28.484  19.391  1.00 95.31           C
ATOM   4694  CE2 TYR A 592      -4.367 -28.922  20.781  1.00 95.31           C
ATOM   4695  OH  TYR A 592      -2.617 -30.531  20.594  1.00 95.31           O
ATOM   4696  CZ  TYR A 592      -3.148 -29.312  20.266  1.00 95.31           C
ATOM   4697  N   LYS A 593      -4.141 -22.172  19.531  1.00 95.50           N
ATOM   4698  CA  LYS A 593      -4.414 -20.859  18.953  1.00 95.50           C
ATOM   4699  C   LYS A 593      -4.219 -20.875  17.438  1.00 95.50           C
ATOM   4700  CB  LYS A 593      -3.518 -19.797  19.594  1.00 95.50           C
ATOM   4701  O   LYS A 593      -3.143 -21.219  16.953  1.00 95.50           O
ATOM   4702  CG  LYS A 593      -3.770 -19.578  21.078  1.00 95.50           C
ATOM   4703  CD  LYS A 593      -2.805 -18.562  21.656  1.00 95.50           C
ATOM   4704  CE  LYS A 593      -2.965 -18.438  23.172  1.00 95.50           C
ATOM   4705  NZ  LYS A 593      -1.955 -17.516  23.766  1.00 95.50           N
ATOM   4706  N   ILE A 594      -5.305 -20.516  16.734  1.00 96.19           N
ATOM   4707  CA  ILE A 594      -5.285 -20.578  15.281  1.00 96.19           C
ATOM   4708  C   ILE A 594      -5.359 -19.156  14.703  1.00 96.19           C
ATOM   4709  CB  ILE A 594      -6.449 -21.438  14.742  1.00 96.19           C
ATOM   4710  O   ILE A 594      -6.191 -18.359  15.117  1.00 96.19           O
ATOM   4711  CG1 ILE A 594      -6.305 -22.891  15.219  1.00 96.19           C
ATOM   4712  CG2 ILE A 594      -6.504 -21.375  13.211  1.00 96.19           C
ATOM   4713  CD1 ILE A 594      -7.438 -23.797  14.773  1.00 96.19           C
ATOM   4714  N   THR A 595      -4.480 -18.844  13.805  1.00 96.56           N
ATOM   4715  CA  THR A 595      -4.496 -17.625  12.984  1.00 96.56           C
ATOM   4716  C   THR A 595      -4.762 -17.969  11.523  1.00 96.56           C
ATOM   4717  CB  THR A 595      -3.172 -16.859  13.102  1.00 96.56           C
ATOM   4718  O   THR A 595      -4.117 -18.859  10.961  1.00 96.56           O
ATOM   4719  CG2 THR A 595      -3.221 -15.555  12.305  1.00 96.56           C
ATOM   4720  OG1 THR A 595      -2.926 -16.547  14.484  1.00 96.56           O
ATOM   4721  N   ALA A 596      -5.781 -17.281  10.992  1.00 96.19           N
ATOM   4722  CA  ALA A 596      -6.156 -17.578   9.609  1.00 96.19           C
ATOM   4723  C   ALA A 596      -6.531 -16.312   8.852  1.00 96.19           C
ATOM   4724  CB  ALA A 596      -7.312 -18.578   9.578  1.00 96.19           C
ATOM   4725  O   ALA A 596      -6.930 -15.312   9.461  1.00 96.19           O
ATOM   4726  N   VAL A 597      -6.352 -16.328   7.488  1.00 95.06           N
ATOM   4727  CA  VAL A 597      -6.637 -15.172   6.633  1.00 95.06           C
ATOM   4728  C   VAL A 597      -7.816 -15.492   5.715  1.00 95.06           C
ATOM   4729  CB  VAL A 597      -5.406 -14.766   5.797  1.00 95.06           C
ATOM   4730  O   VAL A 597      -8.008 -16.641   5.320  1.00 95.06           O
ATOM   4731  CG1 VAL A 597      -5.758 -13.641   4.832  1.00 95.06           C
ATOM   4732  CG2 VAL A 597      -4.254 -14.352   6.715  1.00 95.06           C
```

```
ATOM   4733  N   GLU A 598      -8.617 -14.508   5.480  1.00 95.25           N
ATOM   4734  CA  GLU A 598      -9.844 -14.656   4.699  1.00 95.25           C
ATOM   4735  C   GLU A 598      -9.570 -15.336   3.363  1.00 95.25           C
ATOM   4736  CB  GLU A 598     -10.500 -13.289   4.469  1.00 95.25           C
ATOM   4737  O   GLU A 598      -8.586 -15.023   2.688  1.00 95.25           O
ATOM   4738  CG  GLU A 598     -11.883 -13.375   3.842  1.00 95.25           C
ATOM   4739  CD  GLU A 598     -12.578 -12.023   3.734  1.00 95.25           C
ATOM   4740  OE1 GLU A 598     -13.781 -11.984   3.383  1.00 95.25           O
ATOM   4741  OE2 GLU A 598     -11.914 -11.000   4.004  1.00 95.25           O
ATOM   4742  N   TYR A 599     -10.406 -16.312   3.023  1.00 93.94           N
ATOM   4743  CA  TYR A 599     -10.328 -17.094   1.802  1.00 93.94           C
ATOM   4744  C   TYR A 599     -11.547 -16.875   0.920  1.00 93.94           C
ATOM   4745  CB  TYR A 599     -10.195 -18.594   2.135  1.00 93.94           C
ATOM   4746  O   TYR A 599     -12.688 -16.922   1.397  1.00 93.94           O
ATOM   4747  CG  TYR A 599     -10.219 -19.484   0.922  1.00 93.94           C
ATOM   4748  CD1 TYR A 599     -11.320 -20.297   0.648  1.00 93.94           C
ATOM   4749  CD2 TYR A 599      -9.141 -19.531   0.047  1.00 93.94           C
ATOM   4750  CE1 TYR A 599     -11.344 -21.125  -0.467  1.00 93.94           C
ATOM   4751  CE2 TYR A 599      -9.148 -20.359  -1.072  1.00 93.94           C
ATOM   4752  OH  TYR A 599     -10.273 -21.969  -2.428  1.00 93.94           O
ATOM   4753  CZ  TYR A 599     -10.258 -21.141  -1.321  1.00 93.94           C
ATOM   4754  N   ASP A 600     -11.320 -16.578  -0.343  1.00 91.44           N
ATOM   4755  CA  ASP A 600     -12.383 -16.422  -1.327  1.00 91.44           C
ATOM   4756  C   ASP A 600     -12.328 -17.531  -2.377  1.00 91.44           C
ATOM   4757  CB  ASP A 600     -12.297 -15.047  -2.002  1.00 91.44           C
ATOM   4758  O   ASP A 600     -11.461 -17.500  -3.260  1.00 91.44           O
ATOM   4759  CG  ASP A 600     -13.430 -14.797  -2.984  1.00 91.44           C
ATOM   4760  OD1 ASP A 600     -14.336 -15.648  -3.102  1.00 91.44           O
ATOM   4761  OD2 ASP A 600     -13.414 -13.742  -3.648  1.00 91.44           O
ATOM   4762  N   PRO A 601     -13.266 -18.422  -2.338  1.00 89.75           N
ATOM   4763  CA  PRO A 601     -13.242 -19.531  -3.285  1.00 89.75           C
ATOM   4764  C   PRO A 601     -13.562 -19.109  -4.715  1.00 89.75           C
ATOM   4765  CB  PRO A 601     -14.328 -20.484  -2.746  1.00 89.75           C
ATOM   4766  O   PRO A 601     -13.211 -19.797  -5.668  1.00 89.75           O
ATOM   4767  CG  PRO A 601     -15.234 -19.594  -1.949  1.00 89.75           C
ATOM   4768  CD  PRO A 601     -14.414 -18.500  -1.328  1.00 89.75           C
ATOM   4769  N   ASN A 602     -14.211 -17.969  -4.887  1.00 89.56           N
ATOM   4770  CA  ASN A 602     -14.648 -17.500  -6.199  1.00 89.56           C
ATOM   4771  C   ASN A 602     -13.547 -16.734  -6.918  1.00 89.56           C
ATOM   4772  CB  ASN A 602     -15.906 -16.641  -6.074  1.00 89.56           C
ATOM   4773  O   ASN A 602     -13.688 -16.391  -8.094  1.00 89.56           O
ATOM   4774  CG  ASN A 602     -17.125 -17.438  -5.629  1.00 89.56           C
ATOM   4775  ND2 ASN A 602     -18.016 -16.781  -4.906  1.00 89.56           N
ATOM   4776  OD1 ASN A 602     -17.250 -18.625  -5.934  1.00 89.56           O
ATOM   4777  N   LYS A 603     -12.438 -16.391  -6.234  1.00 91.38           N
ATOM   4778  CA  LYS A 603     -11.383 -15.555  -6.809  1.00 91.38           C
ATOM   4779  C   LYS A 603     -10.727 -16.234  -8.000  1.00 91.38           C
ATOM   4780  CB  LYS A 603     -10.328 -15.211  -5.754  1.00 91.38           C
ATOM   4781  O   LYS A 603     -10.266 -15.570  -8.930  1.00 91.38           O
ATOM   4782  CG  LYS A 603      -9.500 -16.406  -5.305  1.00 91.38           C
ATOM   4783  CD  LYS A 603      -8.453 -15.992  -4.277  1.00 91.38           C
ATOM   4784  CE  LYS A 603      -7.668 -17.203  -3.768  1.00 91.38           C
ATOM   4785  NZ  LYS A 603      -6.738 -16.828  -2.658  1.00 91.38           N
ATOM   4786  N   TYR A 604     -10.656 -17.547  -8.031  1.00 92.06           N
ATOM   4787  CA  TYR A 604      -9.961 -18.297  -9.070  1.00 92.06           C
ATOM   4788  C   TYR A 604     -10.656 -18.125 -10.414  1.00 92.06           C
ATOM   4789  CB  TYR A 604      -9.883 -19.781  -8.695  1.00 92.06           C
ATOM   4790  O   TYR A 604     -10.000 -17.922 -11.438  1.00 92.06           O
ATOM   4791  CG  TYR A 604      -9.227 -20.031  -7.363  1.00 92.06           C
ATOM   4792  CD1 TYR A 604      -7.844 -19.953  -7.215  1.00 92.06           C
ATOM   4793  CD2 TYR A 604      -9.984 -20.359  -6.246  1.00 92.06           C
ATOM   4794  CE1 TYR A 604      -7.234 -20.188  -5.988  1.00 92.06           C
ATOM   4795  CE2 TYR A 604      -9.391 -20.609  -5.016  1.00 92.06           C
ATOM   4796  OH  TYR A 604      -7.418 -20.750  -3.676  1.00 92.06           O
```

ATOM	4797	CZ	TYR	A	604	-8.016	-20.516	-4.895	1.00	92.06	C
ATOM	4798	N	ASP	A	605	-12.000	-18.156	-10.445	1.00	90.56	N
ATOM	4799	CA	ASP	A	605	-12.734	-17.906	-11.680	1.00	90.56	C
ATOM	4800	C	ASP	A	605	-12.578	-16.453	-12.133	1.00	90.56	C
ATOM	4801	CB	ASP	A	605	-14.219	-18.234	-11.492	1.00	90.56	C
ATOM	4802	O	ASP	A	605	-12.500	-16.188	-13.336	1.00	90.56	O
ATOM	4803	CG	ASP	A	605	-14.492	-19.734	-11.477	1.00	90.56	C
ATOM	4804	OD1	ASP	A	605	-13.609	-20.516	-11.867	1.00	90.56	O
ATOM	4805	OD2	ASP	A	605	-15.609	-20.125	-11.062	1.00	90.56	O
ATOM	4806	N	GLU	A	606	-12.469	-15.602	-11.172	1.00	89.88	N
ATOM	4807	CA	GLU	A	606	-12.250	-14.195	-11.492	1.00	89.88	C
ATOM	4808	C	GLU	A	606	-10.867	-13.984	-12.109	1.00	89.88	C
ATOM	4809	CB	GLU	A	606	-12.406	-13.328	-10.242	1.00	89.88	C
ATOM	4810	O	GLU	A	606	-10.719	-13.195	-13.047	1.00	89.88	O
ATOM	4811	CG	GLU	A	606	-13.828	-13.258	-9.711	1.00	89.88	C
ATOM	4812	CD	GLU	A	606	-13.977	-12.383	-8.477	1.00	89.88	C
ATOM	4813	OE1	GLU	A	606	-15.102	-12.242	-7.961	1.00	89.88	O
ATOM	4814	OE2	GLU	A	606	-12.945	-11.828	-8.031	1.00	89.88	O
ATOM	4815	N	ILE	A	607	-9.930	-14.672	-11.602	1.00	92.69	N
ATOM	4816	CA	ILE	A	607	-8.555	-14.562	-12.070	1.00	92.69	C
ATOM	4817	C	ILE	A	607	-8.414	-15.219	-13.445	1.00	92.69	C
ATOM	4818	CB	ILE	A	607	-7.566	-15.211	-11.070	1.00	92.69	C
ATOM	4819	O	ILE	A	607	-7.902	-14.602	-14.383	1.00	92.69	O
ATOM	4820	CG1	ILE	A	607	-7.539	-14.422	-9.758	1.00	92.69	C
ATOM	4821	CG2	ILE	A	607	-6.168	-15.312	-11.680	1.00	92.69	C
ATOM	4822	CD1	ILE	A	607	-6.902	-15.164	-8.594	1.00	92.69	C
ATOM	4823	N	ASP	A	608	-8.922	-16.406	-13.625	1.00	91.38	N
ATOM	4824	CA	ASP	A	608	-8.695	-17.203	-14.828	1.00	91.38	C
ATOM	4825	C	ASP	A	608	-9.656	-16.797	-15.945	1.00	91.38	C
ATOM	4826	CB	ASP	A	608	-8.852	-18.688	-14.516	1.00	91.38	C
ATOM	4827	O	ASP	A	608	-9.258	-16.703	-17.109	1.00	91.38	O
ATOM	4828	CG	ASP	A	608	-7.699	-19.250	-13.695	1.00	91.38	C
ATOM	4829	OD1	ASP	A	608	-6.633	-18.594	-13.617	1.00	91.38	O
ATOM	4830	OD2	ASP	A	608	-7.855	-20.344	-13.125	1.00	91.38	O
ATOM	4831	N	TYR	A	609	-10.930	-16.391	-15.602	1.00	88.88	N
ATOM	4832	CA	TYR	A	609	-11.961	-16.234	-16.609	1.00	88.88	C
ATOM	4833	C	TYR	A	609	-12.500	-14.812	-16.641	1.00	88.88	C
ATOM	4834	CB	TYR	A	609	-13.117	-17.219	-16.359	1.00	88.88	C
ATOM	4835	O	TYR	A	609	-13.273	-14.453	-17.531	1.00	88.88	O
ATOM	4836	CG	TYR	A	609	-12.680	-18.672	-16.297	1.00	88.88	C
ATOM	4837	CD1	TYR	A	609	-12.367	-19.359	-17.469	1.00	88.88	C
ATOM	4838	CD2	TYR	A	609	-12.578	-19.328	-15.086	1.00	88.88	C
ATOM	4839	CE1	TYR	A	609	-11.969	-20.703	-17.422	1.00	88.88	C
ATOM	4840	CE2	TYR	A	609	-12.188	-20.672	-15.031	1.00	88.88	C
ATOM	4841	OH	TYR	A	609	-11.484	-22.656	-16.141	1.00	88.88	O
ATOM	4842	CZ	TYR	A	609	-11.875	-21.344	-16.203	1.00	88.88	C
ATOM	4843	N	GLY	A	610	-12.016	-14.016	-15.672	1.00	81.56	N
ATOM	4844	CA	GLY	A	610	-12.445	-12.625	-15.633	1.00	81.56	C
ATOM	4845	C	GLY	A	610	-13.914	-12.461	-15.305	1.00	81.56	C
ATOM	4846	O	GLY	A	610	-14.562	-11.516	-15.758	1.00	81.56	O
ATOM	4847	N	VAL	A	611	-14.531	-13.531	-14.625	1.00	82.25	N
ATOM	4848	CA	VAL	A	611	-15.938	-13.531	-14.258	1.00	82.25	C
ATOM	4849	C	VAL	A	611	-16.141	-12.773	-12.945	1.00	82.25	C
ATOM	4850	CB	VAL	A	611	-16.484	-14.977	-14.117	1.00	82.25	C
ATOM	4851	O	VAL	A	611	-15.258	-12.773	-12.078	1.00	82.25	O
ATOM	4852	CG1	VAL	A	611	-17.969	-14.961	-13.773	1.00	82.25	C
ATOM	4853	CG2	VAL	A	611	-16.234	-15.766	-15.406	1.00	82.25	C
ATOM	4854	N	ASN	A	612	-17.094	-11.922	-12.938	1.00	78.25	N
ATOM	4855	CA	ASN	A	612	-17.500	-11.242	-11.711	1.00	78.25	C
ATOM	4856	C	ASN	A	612	-18.641	-11.984	-11.023	1.00	78.25	C
ATOM	4857	CB	ASN	A	612	-17.906	-9.797	-12.008	1.00	78.25	C
ATOM	4858	O	ASN	A	612	-19.750	-12.094	-11.570	1.00	78.25	O
ATOM	4859	CG	ASN	A	612	-18.031	-8.953	-10.758	1.00	78.25	C
ATOM	4860	ND2	ASN	A	612	-18.281	-7.664	-10.930	1.00	78.25	N

```
ATOM   4861  OD1 ASN A 612     -17.875  -9.453  -9.641  1.00 78.25           O
ATOM   4862  N   ILE A 613     -18.312 -12.617  -9.883  1.00 81.06           N
ATOM   4863  CA  ILE A 613     -19.297 -13.383  -9.125  1.00 81.06           C
ATOM   4864  C   ILE A 613     -19.703 -12.609  -7.871  1.00 81.06           C
ATOM   4865  CB  ILE A 613     -18.750 -14.773  -8.734  1.00 81.06           C
ATOM   4866  O   ILE A 613     -18.844 -12.180  -7.094  1.00 81.06           O
ATOM   4867  CG1 ILE A 613     -18.328 -15.547  -9.992  1.00 81.06           C
ATOM   4868  CG2 ILE A 613     -19.797 -15.562  -7.938  1.00 81.06           C
ATOM   4869  CD1 ILE A 613     -17.594 -16.844  -9.695  1.00 81.06           C
ATOM   4870  N   ASP A 614     -20.969 -12.273  -7.809  1.00 80.69           N
ATOM   4871  CA  ASP A 614     -21.484 -11.625  -6.609  1.00 80.69           C
ATOM   4872  C   ASP A 614     -21.484 -12.594  -5.426  1.00 80.69           C
ATOM   4873  CB  ASP A 614     -22.891 -11.086  -6.855  1.00 80.69           C
ATOM   4874  O   ASP A 614     -22.094 -13.672  -5.500  1.00 80.69           O
ATOM   4875  CG  ASP A 614     -22.922  -9.906  -7.812  1.00 80.69           C
ATOM   4876  OD1 ASP A 614     -21.906  -9.172  -7.902  1.00 80.69           O
ATOM   4877  OD2 ASP A 614     -23.953  -9.711  -8.477  1.00 80.69           O
ATOM   4878  N   ASP A 615     -20.641 -12.211  -4.414  1.00 83.94           N
ATOM   4879  CA  ASP A 615     -20.562 -13.070  -3.234  1.00 83.94           C
ATOM   4880  C   ASP A 615     -21.766 -12.867  -2.320  1.00 83.94           C
ATOM   4881  CB  ASP A 615     -19.266 -12.805  -2.465  1.00 83.94           C
ATOM   4882  O   ASP A 615     -22.312 -11.766  -2.248  1.00 83.94           O
ATOM   4883  CG  ASP A 615     -18.016 -13.188  -3.248  1.00 83.94           C
ATOM   4884  OD1 ASP A 615     -18.000 -14.266  -3.883  1.00 83.94           O
ATOM   4885  OD2 ASP A 615     -17.047 -12.406  -3.227  1.00 83.94           O
ATOM   4886  N   ARG A 616     -22.250 -14.031  -1.710  1.00 88.06           N
ATOM   4887  CA  ARG A 616     -23.219 -13.914  -0.628  1.00 88.06           C
ATOM   4888  C   ARG A 616     -22.609 -13.203   0.576  1.00 88.06           C
ATOM   4889  CB  ARG A 616     -23.734 -15.297  -0.215  1.00 88.06           C
ATOM   4890  O   ARG A 616     -21.453 -13.422   0.922  1.00 88.06           O
ATOM   4891  CG  ARG A 616     -24.484 -16.031  -1.313  1.00 88.06           C
ATOM   4892  CD  ARG A 616     -24.953 -17.406  -0.856  1.00 88.06           C
ATOM   4893  NE  ARG A 616     -23.828 -18.281  -0.561  1.00 88.06           N
ATOM   4894  NH1 ARG A 616     -25.141 -20.062   0.128  1.00 88.06           N
ATOM   4895  NH2 ARG A 616     -22.859 -20.250   0.134  1.00 88.06           N
ATOM   4896  CZ  ARG A 616     -23.938 -19.531  -0.101  1.00 88.06           C
ATOM   4897  N   PRO A 617     -23.312 -12.172   1.085  1.00 91.12           N
ATOM   4898  CA  PRO A 617     -22.734 -11.492   2.246  1.00 91.12           C
ATOM   4899  C   PRO A 617     -22.453 -12.438   3.412  1.00 91.12           C
ATOM   4900  CB  PRO A 617     -23.797 -10.461   2.615  1.00 91.12           C
ATOM   4901  O   PRO A 617     -23.125 -13.461   3.549  1.00 91.12           O
ATOM   4902  CG  PRO A 617     -25.078 -11.047   2.121  1.00 91.12           C
ATOM   4903  CD  PRO A 617     -24.797 -11.852   0.887  1.00 91.12           C
ATOM   4904  N   THR A 618     -21.375 -12.172   4.203  1.00 91.62           N
ATOM   4905  CA  THR A 618     -21.078 -12.984   5.375  1.00 91.62           C
ATOM   4906  C   THR A 618     -21.797 -12.453   6.605  1.00 91.62           C
ATOM   4907  CB  THR A 618     -19.562 -13.039   5.656  1.00 91.62           C
ATOM   4908  O   THR A 618     -21.828 -13.102   7.652  1.00 91.62           O
ATOM   4909  CG2 THR A 618     -18.812 -13.727   4.520  1.00 91.62           C
ATOM   4910  OG1 THR A 618     -19.062 -11.703   5.805  1.00 91.62           O
ATOM   4911  N   SER A 619     -22.359 -11.180   6.457  1.00 90.56           N
ATOM   4912  CA  SER A 619     -23.078 -10.586   7.586  1.00 90.56           C
ATOM   4913  C   SER A 619     -24.344  -9.867   7.125  1.00 90.56           C
ATOM   4914  CB  SER A 619     -22.172  -9.602   8.336  1.00 90.56           C
ATOM   4915  O   SER A 619     -24.344  -9.219   6.078  1.00 90.56           O
ATOM   4916  OG  SER A 619     -22.891  -8.945   9.359  1.00 90.56           O
ATOM   4917  N   ILE A 620     -25.438 -10.039   7.797  1.00 86.94           N
ATOM   4918  CA  ILE A 620     -26.656  -9.305   7.500  1.00 86.94           C
ATOM   4919  C   ILE A 620     -26.719  -8.031   8.352  1.00 86.94           C
ATOM   4920  CB  ILE A 620     -27.906 -10.164   7.750  1.00 86.94           C
ATOM   4921  O   ILE A 620     -27.703  -7.293   8.312  1.00 86.94           O
ATOM   4922  CG1 ILE A 620     -27.953 -10.648   9.203  1.00 86.94           C
ATOM   4923  CG2 ILE A 620     -27.953 -11.352   6.781  1.00 86.94           C
ATOM   4924  CD1 ILE A 620     -29.297 -11.219   9.633  1.00 86.94           C
```

ATOM	4925	N	VAL	A	621	-25.500	-7.906	9.078	1.00	81.38	N
ATOM	4926	CA	VAL	A	621	-25.406	-6.738	9.945	1.00	81.38	C
ATOM	4927	C	VAL	A	621	-25.016	-5.512	9.117	1.00	81.38	C
ATOM	4928	CB	VAL	A	621	-24.375	-6.957	11.086	1.00	81.38	C
ATOM	4929	O	VAL	A	621	-24.000	-5.527	8.422	1.00	81.38	O
ATOM	4930	CG1	VAL	A	621	-24.297	-5.723	11.984	1.00	81.38	C
ATOM	4931	CG2	VAL	A	621	-24.750	-8.188	11.906	1.00	81.38	C
ATOM	4932	N	GLU	A	622	-25.781	-4.594	8.891	1.00	73.50	N
ATOM	4933	CA	GLU	A	622	-25.547	-3.363	8.133	1.00	73.50	C
ATOM	4934	C	GLU	A	622	-25.516	-2.148	9.055	1.00	73.50	C
ATOM	4935	CB	GLU	A	622	-26.625	-3.182	7.066	1.00	73.50	C
ATOM	4936	O	GLU	A	622	-26.484	-1.403	9.148	1.00	73.50	O
ATOM	4937	CG	GLU	A	622	-26.594	-4.238	5.973	1.00	73.50	C
ATOM	4938	CD	GLU	A	622	-27.688	-4.055	4.930	1.00	73.50	C
ATOM	4939	OE1	GLU	A	622	-27.719	-4.828	3.943	1.00	73.50	O
ATOM	4940	OE2	GLU	A	622	-28.516	-3.135	5.102	1.00	73.50	O
ATOM	4941	N	PRO	A	623	-24.312	-1.935	9.695	1.00	65.31	N
ATOM	4942	CA	PRO	A	623	-24.328	-0.812	10.633	1.00	65.31	C
ATOM	4943	C	PRO	A	623	-24.531	0.534	9.945	1.00	65.31	C
ATOM	4944	CB	PRO	A	623	-22.938	-0.882	11.289	1.00	65.31	C
ATOM	4945	O	PRO	A	623	-24.984	1.490	10.570	1.00	65.31	O
ATOM	4946	CG	PRO	A	623	-22.141	-1.777	10.391	1.00	65.31	C
ATOM	4947	CD	PRO	A	623	-23.094	-2.605	9.578	1.00	65.31	C
ATOM	4948	N	ASP	A	624	-24.188	0.535	8.555	1.00	66.19	N
ATOM	4949	CA	ASP	A	624	-24.266	1.830	7.887	1.00	66.19	C
ATOM	4950	C	ASP	A	624	-25.703	2.180	7.523	1.00	66.19	C
ATOM	4951	CB	ASP	A	624	-23.391	1.835	6.629	1.00	66.19	C
ATOM	4952	O	ASP	A	624	-26.016	3.342	7.250	1.00	66.19	O
ATOM	4953	CG	ASP	A	624	-21.906	1.650	6.934	1.00	66.19	C
ATOM	4954	OD1	ASP	A	624	-21.469	2.039	8.031	1.00	66.19	O
ATOM	4955	OD2	ASP	A	624	-21.188	1.113	6.066	1.00	66.19	O
ATOM	4956	N	GLN	A	625	-26.547	1.153	7.445	1.00	71.19	N
ATOM	4957	CA	GLN	A	625	-27.953	1.381	7.152	1.00	71.19	C
ATOM	4958	C	GLN	A	625	-28.812	1.206	8.406	1.00	71.19	C
ATOM	4959	CB	GLN	A	625	-28.438	0.434	6.051	1.00	71.19	C
ATOM	4960	O	GLN	A	625	-29.062	0.080	8.836	1.00	71.19	O
ATOM	4961	CG	GLN	A	625	-27.781	0.679	4.699	1.00	71.19	C
ATOM	4962	CD	GLN	A	625	-28.781	0.771	3.566	1.00	71.19	C
ATOM	4963	NE2	GLN	A	625	-28.375	1.374	2.457	1.00	71.19	N
ATOM	4964	OE1	GLN	A	625	-29.922	0.303	3.688	1.00	71.19	O
ATOM	4965	N	ILE	A	626	-28.828	2.240	9.195	1.00	81.31	N
ATOM	4966	CA	ILE	A	626	-29.609	2.197	10.430	1.00	81.31	C
ATOM	4967	C	ILE	A	626	-31.078	2.469	10.125	1.00	81.31	C
ATOM	4968	CB	ILE	A	626	-29.078	3.215	11.461	1.00	81.31	C
ATOM	4969	O	ILE	A	626	-31.406	3.457	9.461	1.00	81.31	O
ATOM	4970	CG1	ILE	A	626	-29.547	2.828	12.875	1.00	81.31	C
ATOM	4971	CG2	ILE	A	626	-29.547	4.633	11.109	1.00	81.31	C
ATOM	4972	CD1	ILE	A	626	-28.703	3.451	13.984	1.00	81.31	C
ATOM	4973	N	PRO	A	627	-31.953	1.392	10.375	1.00	86.50	N
ATOM	4974	CA	PRO	A	627	-33.375	1.579	10.117	1.00	86.50	C
ATOM	4975	C	PRO	A	627	-33.969	2.725	10.930	1.00	86.50	C
ATOM	4976	CB	PRO	A	627	-34.000	0.239	10.531	1.00	86.50	C
ATOM	4977	O	PRO	A	627	-33.438	3.111	11.961	1.00	86.50	O
ATOM	4978	CG	PRO	A	627	-32.812	-0.657	10.773	1.00	86.50	C
ATOM	4979	CD	PRO	A	627	-31.594	0.194	10.914	1.00	86.50	C
ATOM	4980	N	ARG	A	628	-34.938	3.307	10.336	1.00	90.12	N
ATOM	4981	CA	ARG	A	628	-35.688	4.320	11.078	1.00	90.12	C
ATOM	4982	C	ARG	A	628	-36.531	3.688	12.188	1.00	90.12	C
ATOM	4983	CB	ARG	A	628	-36.594	5.133	10.141	1.00	90.12	C
ATOM	4984	O	ARG	A	628	-36.969	2.539	12.062	1.00	90.12	O
ATOM	4985	CG	ARG	A	628	-37.625	4.301	9.406	1.00	90.12	C
ATOM	4986	CD	ARG	A	628	-38.469	5.152	8.461	1.00	90.12	C
ATOM	4987	NE	ARG	A	628	-39.031	4.352	7.387	1.00	90.12	N
ATOM	4988	NH1	ARG	A	628	-39.719	6.168	6.133	1.00	90.12	N

ATOM	4989	NH2	ARG	A	628	-40.125	4.035	5.387	1.00	90.12	N
ATOM	4990	CZ	ARG	A	628	-39.625	4.855	6.305	1.00	90.12	C
ATOM	4991	N	PRO	A	629	-36.625	4.250	13.352	1.00	93.88	N
ATOM	4992	CA	PRO	A	629	-37.469	3.723	14.438	1.00	93.88	C
ATOM	4993	C	PRO	A	629	-38.906	3.410	14.000	1.00	93.88	C
ATOM	4994	CB	PRO	A	629	-37.438	4.855	15.469	1.00	93.88	C
ATOM	4995	O	PRO	A	629	-39.469	4.137	13.180	1.00	93.88	O
ATOM	4996	CG	PRO	A	629	-36.188	5.625	15.172	1.00	93.88	C
ATOM	4997	CD	PRO	A	629	-35.938	5.562	13.695	1.00	93.88	C
ATOM	4998	N	LYS	A	630	-39.438	2.166	14.305	1.00	91.56	N
ATOM	4999	CA	LYS	A	630	-40.812	1.772	14.031	1.00	91.56	C
ATOM	5000	C	LYS	A	630	-41.688	1.943	15.266	1.00	91.56	C
ATOM	5001	CB	LYS	A	630	-40.875	0.323	13.547	1.00	91.56	C
ATOM	5002	O	LYS	A	630	-41.188	2.059	16.391	1.00	91.56	O
ATOM	5003	CG	LYS	A	630	-40.188	0.096	12.195	1.00	91.56	C
ATOM	5004	CD	LYS	A	630	-40.312	-1.356	11.758	1.00	91.56	C
ATOM	5005	CE	LYS	A	630	-39.594	-1.593	10.422	1.00	91.56	C
ATOM	5006	NZ	LYS	A	630	-39.656	-3.027	10.016	1.00	91.56	N
ATOM	5007	N	ASN	A	631	-43.000	2.154	15.070	1.00	90.75	N
ATOM	5008	CA	ASN	A	631	-44.031	2.221	16.109	1.00	90.75	C
ATOM	5009	C	ASN	A	631	-43.812	3.438	17.000	1.00	90.75	C
ATOM	5010	CB	ASN	A	631	-44.031	0.938	16.938	1.00	90.75	C
ATOM	5011	O	ASN	A	631	-43.906	3.328	18.234	1.00	90.75	O
ATOM	5012	CG	ASN	A	631	-44.375	-0.288	16.125	1.00	90.75	C
ATOM	5013	ND2	ASN	A	631	-43.688	-1.396	16.375	1.00	90.75	N
ATOM	5014	OD1	ASN	A	631	-45.281	-0.239	15.273	1.00	90.75	O
ATOM	5015	N	VAL	A	632	-43.375	4.488	16.266	1.00	93.31	N
ATOM	5016	CA	VAL	A	632	-43.344	5.730	17.031	1.00	93.31	C
ATOM	5017	C	VAL	A	632	-44.750	6.055	17.562	1.00	93.31	C
ATOM	5018	CB	VAL	A	632	-42.812	6.906	16.172	1.00	93.31	C
ATOM	5019	O	VAL	A	632	-45.719	6.086	16.812	1.00	93.31	O
ATOM	5020	CG1	VAL	A	632	-42.781	8.195	17.000	1.00	93.31	C
ATOM	5021	CG2	VAL	A	632	-41.438	6.574	15.602	1.00	93.31	C
ATOM	5022	N	GLN	A	633	-44.844	6.043	18.844	1.00	93.31	N
ATOM	5023	CA	GLN	A	633	-46.125	6.324	19.484	1.00	93.31	C
ATOM	5024	C	GLN	A	633	-46.062	7.625	20.281	1.00	93.31	C
ATOM	5025	CB	GLN	A	633	-46.531	5.168	20.391	1.00	93.31	C
ATOM	5026	O	GLN	A	633	-45.062	7.906	20.938	1.00	93.31	O
ATOM	5027	CG	GLN	A	633	-46.719	3.846	19.656	1.00	93.31	C
ATOM	5028	CD	GLN	A	633	-47.406	2.791	20.516	1.00	93.31	C
ATOM	5029	NE2	GLN	A	633	-48.062	1.834	19.875	1.00	93.31	N
ATOM	5030	OE1	GLN	A	633	-47.344	2.834	21.750	1.00	93.31	O
ATOM	5031	N	VAL	A	634	-47.094	8.359	20.062	1.00	92.31	N
ATOM	5032	CA	VAL	A	634	-47.188	9.625	20.781	1.00	92.31	C
ATOM	5033	C	VAL	A	634	-48.406	9.594	21.703	1.00	92.31	C
ATOM	5034	CB	VAL	A	634	-47.281	10.828	19.812	1.00	92.31	C
ATOM	5035	O	VAL	A	634	-49.531	9.328	21.250	1.00	92.31	O
ATOM	5036	CG1	VAL	A	634	-47.344	12.141	20.594	1.00	92.31	C
ATOM	5037	CG2	VAL	A	634	-46.125	10.812	18.828	1.00	92.31	C
ATOM	5038	N	SER	A	635	-48.250	9.523	22.984	1.00	89.81	N
ATOM	5039	CA	SER	A	635	-49.312	9.609	23.984	1.00	89.81	C
ATOM	5040	C	SER	A	635	-49.000	10.703	25.016	1.00	89.81	C
ATOM	5041	CB	SER	A	635	-49.469	8.266	24.703	1.00	89.81	C
ATOM	5042	O	SER	A	635	-47.969	11.359	24.938	1.00	89.81	O
ATOM	5043	OG	SER	A	635	-48.312	7.930	25.422	1.00	89.81	O
ATOM	5044	N	SER	A	636	-49.938	10.930	25.812	1.00	86.56	N
ATOM	5045	CA	SER	A	636	-49.719	11.930	26.859	1.00	86.56	C
ATOM	5046	C	SER	A	636	-50.250	11.430	28.203	1.00	86.56	C
ATOM	5047	CB	SER	A	636	-50.438	13.242	26.484	1.00	86.56	C
ATOM	5048	O	SER	A	636	-51.125	10.555	28.250	1.00	86.56	O
ATOM	5049	OG	SER	A	636	-51.844	13.086	26.516	1.00	86.56	O
ATOM	5050	N	GLU	A	637	-49.688	11.703	29.234	1.00	82.81	N
ATOM	5051	CA	GLU	A	637	-50.156	11.477	30.609	1.00	82.81	C
ATOM	5052	C	GLU	A	637	-50.125	12.773	31.422	1.00	82.81	C

ATOM	5053	CB	GLU	A	637	-49.312	10.406	31.297	1.00	82.81	C
ATOM	5054	O	GLU	A	637	-49.250	13.633	31.188	1.00	82.81	O
ATOM	5055	CG	GLU	A	637	-48.875	9.273	30.375	1.00	82.81	C
ATOM	5056	CD	GLU	A	637	-48.031	8.211	31.078	1.00	82.81	C
ATOM	5057	OE1	GLU	A	637	-47.656	7.207	30.422	1.00	82.81	O
ATOM	5058	OE2	GLU	A	637	-47.781	8.383	32.281	1.00	82.81	O
ATOM	5059	N	SER	A	638	-51.188	12.945	32.062	1.00	70.69	N
ATOM	5060	CA	SER	A	638	-51.344	14.141	32.875	1.00	70.69	C
ATOM	5061	C	SER	A	638	-51.188	13.828	34.375	1.00	70.69	C
ATOM	5062	CB	SER	A	638	-52.719	14.797	32.656	1.00	70.69	C
ATOM	5063	O	SER	A	638	-51.562	12.750	34.812	1.00	70.69	O
ATOM	5064	OG	SER	A	638	-53.750	13.945	33.062	1.00	70.69	O
ATOM	5065	N	ARG	A	639	-50.219	14.336	34.969	1.00	68.94	N
ATOM	5066	CA	ARG	A	639	-50.031	14.219	36.406	1.00	68.94	C
ATOM	5067	C	ARG	A	639	-50.094	15.594	37.094	1.00	68.94	C
ATOM	5068	CB	ARG	A	639	-48.719	13.531	36.750	1.00	68.94	C
ATOM	5069	O	ARG	A	639	-49.875	16.609	36.438	1.00	68.94	O
ATOM	5070	CG	ARG	A	639	-47.500	14.367	36.406	1.00	68.94	C
ATOM	5071	CD	ARG	A	639	-46.188	13.633	36.750	1.00	68.94	C
ATOM	5072	NE	ARG	A	639	-45.031	14.453	36.500	1.00	68.94	N
ATOM	5073	NH1	ARG	A	639	-43.531	12.688	36.438	1.00	68.94	N
ATOM	5074	NH2	ARG	A	639	-42.781	14.836	36.125	1.00	68.94	N
ATOM	5075	CZ	ARG	A	639	-43.781	13.992	36.375	1.00	68.94	C
ATOM	5076	N	ILE	A	640	-50.750	15.648	38.250	1.00	59.84	N
ATOM	5077	CA	ILE	A	640	-50.844	16.859	39.062	1.00	59.84	C
ATOM	5078	C	ILE	A	640	-49.562	17.078	39.844	1.00	59.84	C
ATOM	5079	CB	ILE	A	640	-52.062	16.812	40.000	1.00	59.84	C
ATOM	5080	O	ILE	A	640	-49.188	16.203	40.625	1.00	59.84	O
ATOM	5081	CG1	ILE	A	640	-53.375	16.688	39.188	1.00	59.84	C
ATOM	5082	CG2	ILE	A	640	-52.094	18.031	40.906	1.00	59.84	C
ATOM	5083	CD1	ILE	A	640	-54.594	16.438	40.031	1.00	59.84	C
ATOM	5084	N	VAL	A	641	-48.688	17.797	39.281	1.00	50.53	N
ATOM	5085	CA	VAL	A	641	-47.469	18.125	40.031	1.00	50.53	C
ATOM	5086	C	VAL	A	641	-47.625	19.453	40.750	1.00	50.53	C
ATOM	5087	CB	VAL	A	641	-46.250	18.172	39.094	1.00	50.53	C
ATOM	5088	O	VAL	A	641	-47.781	20.500	40.094	1.00	50.53	O
ATOM	5089	CG1	VAL	A	641	-44.969	18.516	39.875	1.00	50.53	C
ATOM	5090	CG2	VAL	A	641	-46.062	16.844	38.344	1.00	50.53	C
ATOM	5091	N	GLN	A	642	-47.844	19.438	42.094	1.00	44.31	N
ATOM	5092	CA	GLN	A	642	-48.031	20.594	42.969	1.00	44.31	C
ATOM	5093	C	GLN	A	642	-49.250	21.422	42.594	1.00	44.31	C
ATOM	5094	CB	GLN	A	642	-46.750	21.469	42.969	1.00	44.31	C
ATOM	5095	O	GLN	A	642	-49.156	22.641	42.438	1.00	44.31	O
ATOM	5096	CG	GLN	A	642	-45.531	20.797	43.531	1.00	44.31	C
ATOM	5097	CD	GLN	A	642	-44.344	21.750	43.688	1.00	44.31	C
ATOM	5098	NE2	GLN	A	642	-43.469	21.453	44.656	1.00	44.31	N
ATOM	5099	OE1	GLN	A	642	-44.250	22.734	42.969	1.00	44.31	O
ATOM	5100	N	GLY	A	643	-50.406	20.812	42.250	1.00	49.44	N
ATOM	5101	CA	GLY	A	643	-51.688	21.516	42.125	1.00	49.44	C
ATOM	5102	C	GLY	A	643	-52.000	21.938	40.719	1.00	49.44	C
ATOM	5103	O	GLY	A	643	-53.062	22.484	40.438	1.00	49.44	O
ATOM	5104	N	MET	A	644	-51.156	21.781	39.594	1.00	50.44	N
ATOM	5105	CA	MET	A	644	-51.312	22.062	38.156	1.00	50.44	C
ATOM	5106	C	MET	A	644	-51.188	20.797	37.344	1.00	50.44	C
ATOM	5107	CB	MET	A	644	-50.312	23.125	37.719	1.00	50.44	C
ATOM	5108	O	MET	A	644	-50.312	19.969	37.625	1.00	50.44	O
ATOM	5109	CG	MET	A	644	-50.500	24.484	38.344	1.00	50.44	C
ATOM	5110	SD	MET	A	644	-49.438	25.766	37.625	1.00	50.44	S
ATOM	5111	CE	MET	A	644	-47.781	25.078	37.969	1.00	50.44	C
ATOM	5112	N	SER	A	645	-52.312	20.453	36.531	1.00	61.22	N
ATOM	5113	CA	SER	A	645	-52.312	19.328	35.625	1.00	61.22	C
ATOM	5114	C	SER	A	645	-51.281	19.547	34.500	1.00	61.22	C
ATOM	5115	CB	SER	A	645	-53.688	19.141	35.000	1.00	61.22	C
ATOM	5116	O	SER	A	645	-51.312	20.531	33.781	1.00	61.22	O

ATOM	5117	OG	SER	A	645	-53.688	18.094	34.062	1.00	61.22	O
ATOM	5118	N	VAL	A	646	-50.000	18.969	34.594	1.00	67.12	N
ATOM	5119	CA	VAL	A	646	-48.969	19.016	33.594	1.00	67.12	C
ATOM	5120	C	VAL	A	646	-49.094	17.797	32.656	1.00	67.12	C
ATOM	5121	CB	VAL	A	646	-47.562	19.047	34.219	1.00	67.12	C
ATOM	5122	O	VAL	A	646	-49.062	16.656	33.125	1.00	67.12	O
ATOM	5123	CG1	VAL	A	646	-46.469	19.109	33.125	1.00	67.12	C
ATOM	5124	CG2	VAL	A	646	-47.438	20.234	35.156	1.00	67.12	C
ATOM	5125	N	GLU	A	647	-49.562	18.219	31.578	1.00	77.31	N
ATOM	5126	CA	GLU	A	647	-49.688	17.188	30.547	1.00	77.31	C
ATOM	5127	C	GLU	A	647	-48.344	16.953	29.859	1.00	77.31	C
ATOM	5128	CB	GLU	A	647	-50.750	17.562	29.516	1.00	77.31	C
ATOM	5129	O	GLU	A	647	-47.688	17.891	29.406	1.00	77.31	O
ATOM	5130	CG	GLU	A	647	-51.125	16.422	28.578	1.00	77.31	C
ATOM	5131	CD	GLU	A	647	-51.906	15.320	29.250	1.00	77.31	C
ATOM	5132	OE1	GLU	A	647	-51.875	14.156	28.781	1.00	77.31	O
ATOM	5133	OE2	GLU	A	647	-52.594	15.617	30.266	1.00	77.31	O
ATOM	5134	N	THR	A	648	-47.688	15.727	29.875	1.00	85.69	N
ATOM	5135	CA	THR	A	648	-46.406	15.281	29.328	1.00	85.69	C
ATOM	5136	C	THR	A	648	-46.625	14.375	28.109	1.00	85.69	C
ATOM	5137	CB	THR	A	648	-45.594	14.539	30.391	1.00	85.69	C
ATOM	5138	O	THR	A	648	-47.375	13.383	28.203	1.00	85.69	O
ATOM	5139	CG2	THR	A	648	-44.219	14.156	29.828	1.00	85.69	C
ATOM	5140	OG1	THR	A	648	-45.406	15.383	31.531	1.00	85.69	O
ATOM	5141	N	MET	A	649	-46.156	14.938	27.047	1.00	90.25	N
ATOM	5142	CA	MET	A	649	-46.156	14.102	25.859	1.00	90.25	C
ATOM	5143	C	MET	A	649	-45.094	13.023	25.938	1.00	90.25	C
ATOM	5144	CB	MET	A	649	-45.969	14.953	24.594	1.00	90.25	C
ATOM	5145	O	MET	A	649	-43.938	13.320	26.219	1.00	90.25	O
ATOM	5146	CG	MET	A	649	-46.000	14.164	23.297	1.00	90.25	C
ATOM	5147	SD	MET	A	649	-45.812	15.227	21.828	1.00	90.25	S
ATOM	5148	CE	MET	A	649	-44.062	15.641	21.953	1.00	90.25	C
ATOM	5149	N	ILE	A	650	-45.500	11.867	25.734	1.00	92.81	N
ATOM	5150	CA	ILE	A	650	-44.594	10.727	25.719	1.00	92.81	C
ATOM	5151	C	ILE	A	650	-44.438	10.195	24.297	1.00	92.81	C
ATOM	5152	CB	ILE	A	650	-45.062	9.609	26.672	1.00	92.81	C
ATOM	5153	O	ILE	A	650	-45.438	9.805	23.672	1.00	92.81	O
ATOM	5154	CG1	ILE	A	650	-45.281	10.148	28.094	1.00	92.81	C
ATOM	5155	CG2	ILE	A	650	-44.094	8.430	26.656	1.00	92.81	C
ATOM	5156	CD1	ILE	A	650	-45.969	9.180	29.031	1.00	92.81	C
ATOM	5157	N	VAL	A	651	-43.250	10.328	23.859	1.00	94.00	N
ATOM	5158	CA	VAL	A	651	-42.906	9.766	22.562	1.00	94.00	C
ATOM	5159	C	VAL	A	651	-42.062	8.508	22.734	1.00	94.00	C
ATOM	5160	CB	VAL	A	651	-42.156	10.789	21.672	1.00	94.00	C
ATOM	5161	O	VAL	A	651	-41.000	8.562	23.359	1.00	94.00	O
ATOM	5162	CG1	VAL	A	651	-41.969	10.242	20.266	1.00	94.00	C
ATOM	5163	CG2	VAL	A	651	-42.906	12.117	21.641	1.00	94.00	C
ATOM	5164	N	SER	A	652	-42.625	7.449	22.266	1.00	94.62	N
ATOM	5165	CA	SER	A	652	-41.906	6.184	22.422	1.00	94.62	C
ATOM	5166	C	SER	A	652	-41.812	5.434	21.094	1.00	94.62	C
ATOM	5167	CB	SER	A	652	-42.625	5.309	23.469	1.00	94.62	C
ATOM	5168	O	SER	A	652	-42.656	5.629	20.203	1.00	94.62	O
ATOM	5169	OG	SER	A	652	-43.906	4.965	23.047	1.00	94.62	O
ATOM	5170	N	TRP	A	653	-40.812	4.742	21.016	1.00	94.88	N
ATOM	5171	CA	TRP	A	653	-40.562	3.936	19.828	1.00	94.88	C
ATOM	5172	C	TRP	A	653	-39.844	2.639	20.188	1.00	94.88	C
ATOM	5173	CB	TRP	A	653	-39.750	4.719	18.797	1.00	94.88	C
ATOM	5174	O	TRP	A	653	-39.469	2.441	21.344	1.00	94.88	O
ATOM	5175	CG	TRP	A	653	-38.438	5.246	19.344	1.00	94.88	C
ATOM	5176	CD1	TRP	A	653	-37.250	4.621	19.312	1.00	94.88	C
ATOM	5177	CD2	TRP	A	653	-38.250	6.500	19.984	1.00	94.88	C
ATOM	5178	CE2	TRP	A	653	-36.875	6.574	20.328	1.00	94.88	C
ATOM	5179	CE3	TRP	A	653	-39.094	7.574	20.312	1.00	94.88	C
ATOM	5180	NE1	TRP	A	653	-36.281	5.414	19.906	1.00	94.88	N

ATOM	5181	CH2	TRP	A	653	-37.156	8.711	21.297	1.00	94.88	C
ATOM	5182	CZ2	TRP	A	653	-36.312	7.680	20.984	1.00	94.88	C
ATOM	5183	CZ3	TRP	A	653	-38.531	8.672	20.969	1.00	94.88	C
ATOM	5184	N	ASP	A	654	-39.844	1.750	19.234	1.00	92.75	N
ATOM	5185	CA	ASP	A	654	-39.156	0.476	19.422	1.00	92.75	C
ATOM	5186	C	ASP	A	654	-37.625	0.645	19.312	1.00	92.75	C
ATOM	5187	CB	ASP	A	654	-39.625	-0.559	18.406	1.00	92.75	C
ATOM	5188	O	ASP	A	654	-37.156	1.523	18.578	1.00	92.75	O
ATOM	5189	CG	ASP	A	654	-41.062	-0.980	18.641	1.00	92.75	C
ATOM	5190	OD1	ASP	A	654	-41.625	-0.666	19.719	1.00	92.75	O
ATOM	5191	OD2	ASP	A	654	-41.656	-1.637	17.750	1.00	92.75	O
ATOM	5192	N	LYS	A	655	-36.875	-0.119	20.062	1.00	88.44	N
ATOM	5193	CA	LYS	A	655	-35.406	-0.175	19.922	1.00	88.44	C
ATOM	5194	C	LYS	A	655	-35.031	-0.560	18.484	1.00	88.44	C
ATOM	5195	CB	LYS	A	655	-34.812	-1.166	20.906	1.00	88.44	C
ATOM	5196	O	LYS	A	655	-35.594	-1.494	17.922	1.00	88.44	O
ATOM	5197	CG	LYS	A	655	-33.281	-1.272	20.828	1.00	88.44	C
ATOM	5198	CD	LYS	A	655	-32.750	-2.223	21.891	1.00	88.44	C
ATOM	5199	CE	LYS	A	655	-31.234	-2.324	21.828	1.00	88.44	C
ATOM	5200	NZ	LYS	A	655	-30.688	-3.205	22.891	1.00	88.44	N
ATOM	5201	N	VAL	A	656	-34.250	0.426	17.969	1.00	88.44	N
ATOM	5202	CA	VAL	A	656	-33.750	0.146	16.625	1.00	88.44	C
ATOM	5203	C	VAL	A	656	-32.406	-0.566	16.719	1.00	88.44	C
ATOM	5204	CB	VAL	A	656	-33.594	1.438	15.797	1.00	88.44	C
ATOM	5205	O	VAL	A	656	-31.500	-0.120	17.453	1.00	88.44	O
ATOM	5206	CG1	VAL	A	656	-33.031	1.126	14.406	1.00	88.44	C
ATOM	5207	CG2	VAL	A	656	-34.969	2.148	15.680	1.00	88.44	C
ATOM	5208	N	PRO	A	657	-32.250	-1.682	16.109	1.00	81.38	N
ATOM	5209	CA	PRO	A	657	-30.984	-2.385	16.109	1.00	81.38	C
ATOM	5210	C	PRO	A	657	-29.812	-1.490	15.680	1.00	81.38	C
ATOM	5211	CB	PRO	A	657	-31.188	-3.512	15.094	1.00	81.38	C
ATOM	5212	O	PRO	A	657	-29.938	-0.728	14.719	1.00	81.38	O
ATOM	5213	CG	PRO	A	657	-32.688	-3.730	15.070	1.00	81.38	C
ATOM	5214	CD	PRO	A	657	-33.344	-2.422	15.352	1.00	81.38	C
ATOM	5215	N	TYR	A	658	-28.672	-1.427	16.453	1.00	78.50	N
ATOM	5216	CA	TYR	A	658	-27.406	-0.756	16.172	1.00	78.50	C
ATOM	5217	C	TYR	A	658	-27.469	0.706	16.609	1.00	78.50	C
ATOM	5218	CB	TYR	A	658	-27.062	-0.856	14.695	1.00	78.50	C
ATOM	5219	O	TYR	A	658	-26.469	1.428	16.500	1.00	78.50	O
ATOM	5220	CG	TYR	A	658	-27.047	-2.270	14.164	1.00	78.50	C
ATOM	5221	CD1	TYR	A	658	-26.203	-3.230	14.695	1.00	78.50	C
ATOM	5222	CD2	TYR	A	658	-27.891	-2.646	13.125	1.00	78.50	C
ATOM	5223	CE1	TYR	A	658	-26.188	-4.535	14.211	1.00	78.50	C
ATOM	5224	CE2	TYR	A	658	-27.891	-3.947	12.633	1.00	78.50	C
ATOM	5225	OH	TYR	A	658	-27.031	-6.172	12.695	1.00	78.50	O
ATOM	5226	CZ	TYR	A	658	-27.031	-4.883	13.180	1.00	78.50	C
ATOM	5227	N	ALA	A	659	-28.750	1.209	16.984	1.00	87.25	N
ATOM	5228	CA	ALA	A	659	-28.875	2.594	17.422	1.00	87.25	C
ATOM	5229	C	ALA	A	659	-28.391	2.754	18.859	1.00	87.25	C
ATOM	5230	CB	ALA	A	659	-30.312	3.066	17.297	1.00	87.25	C
ATOM	5231	O	ALA	A	659	-28.781	1.991	19.750	1.00	87.25	O
ATOM	5232	N	VAL	A	660	-27.312	3.586	19.016	1.00	87.25	N
ATOM	5233	CA	VAL	A	660	-26.812	3.906	20.359	1.00	87.25	C
ATOM	5234	C	VAL	A	660	-27.578	5.102	20.922	1.00	87.25	C
ATOM	5235	CB	VAL	A	660	-25.297	4.203	20.344	1.00	87.25	C
ATOM	5236	O	VAL	A	660	-27.875	5.152	22.109	1.00	87.25	O
ATOM	5237	CG1	VAL	A	660	-24.812	4.574	21.734	1.00	87.25	C
ATOM	5238	CG2	VAL	A	660	-24.516	3.000	19.812	1.00	87.25	C
ATOM	5239	N	PHE	A	661	-27.969	6.043	20.000	1.00	93.25	N
ATOM	5240	CA	PHE	A	661	-28.703	7.242	20.375	1.00	93.25	C
ATOM	5241	C	PHE	A	661	-29.891	7.465	19.438	1.00	93.25	C
ATOM	5242	CB	PHE	A	661	-27.781	8.469	20.359	1.00	93.25	C
ATOM	5243	O	PHE	A	661	-29.969	6.852	18.375	1.00	93.25	O
ATOM	5244	CG	PHE	A	661	-26.609	8.352	21.281	1.00	93.25	C

ATOM	5245	CD1	PHE	A	661	-26.766	8.445	22.656	1.00	93.25	C
ATOM	5246	CD2	PHE	A	661	-25.328	8.148	20.766	1.00	93.25	C
ATOM	5247	CE1	PHE	A	661	-25.672	8.336	23.516	1.00	93.25	C
ATOM	5248	CE2	PHE	A	661	-24.234	8.047	21.609	1.00	93.25	C
ATOM	5249	CZ	PHE	A	661	-24.406	8.133	22.984	1.00	93.25	C
ATOM	5250	N	TYR	A	662	-30.828	8.367	19.891	1.00	95.00	N
ATOM	5251	CA	TYR	A	662	-31.969	8.758	19.047	1.00	95.00	C
ATOM	5252	C	TYR	A	662	-32.125	10.273	19.047	1.00	95.00	C
ATOM	5253	CB	TYR	A	662	-33.250	8.102	19.547	1.00	95.00	C
ATOM	5254	O	TYR	A	662	-31.953	10.930	20.078	1.00	95.00	O
ATOM	5255	CG	TYR	A	662	-33.219	6.590	19.516	1.00	95.00	C
ATOM	5256	CD1	TYR	A	662	-33.594	5.891	18.375	1.00	95.00	C
ATOM	5257	CD2	TYR	A	662	-32.781	5.863	20.625	1.00	95.00	C
ATOM	5258	CE1	TYR	A	662	-33.562	4.500	18.344	1.00	95.00	C
ATOM	5259	CE2	TYR	A	662	-32.750	4.473	20.594	1.00	95.00	C
ATOM	5260	OH	TYR	A	662	-33.094	2.426	19.422	1.00	95.00	O
ATOM	5261	CZ	TYR	A	662	-33.125	3.801	19.453	1.00	95.00	C
ATOM	5262	N	ASP	A	663	-32.281	10.703	17.812	1.00	95.06	N
ATOM	5263	CA	ASP	A	663	-32.688	12.094	17.656	1.00	95.06	C
ATOM	5264	C	ASP	A	663	-34.219	12.203	17.516	1.00	95.06	C
ATOM	5265	CB	ASP	A	663	-32.000	12.727	16.438	1.00	95.06	C
ATOM	5266	O	ASP	A	663	-34.812	11.484	16.719	1.00	95.06	O
ATOM	5267	CG	ASP	A	663	-30.516	12.945	16.656	1.00	95.06	C
ATOM	5268	OD1	ASP	A	663	-30.094	13.133	17.812	1.00	95.06	O
ATOM	5269	OD2	ASP	A	663	-29.766	12.930	15.648	1.00	95.06	O
ATOM	5270	N	VAL	A	664	-34.875	13.055	18.297	1.00	95.38	N
ATOM	5271	CA	VAL	A	664	-36.312	13.203	18.266	1.00	95.38	C
ATOM	5272	C	VAL	A	664	-36.688	14.656	17.953	1.00	95.38	C
ATOM	5273	CB	VAL	A	664	-36.969	12.773	19.609	1.00	95.38	C
ATOM	5274	O	VAL	A	664	-36.094	15.586	18.500	1.00	95.38	O
ATOM	5275	CG1	VAL	A	664	-38.469	12.891	19.547	1.00	95.38	C
ATOM	5276	CG2	VAL	A	664	-36.531	11.344	19.953	1.00	95.38	C
ATOM	5277	N	GLN	A	665	-37.688	14.773	16.969	1.00	95.56	N
ATOM	5278	CA	GLN	A	665	-38.219	16.094	16.688	1.00	95.56	C
ATOM	5279	C	GLN	A	665	-39.750	16.078	16.812	1.00	95.56	C
ATOM	5280	CB	GLN	A	665	-37.812	16.547	15.281	1.00	95.56	C
ATOM	5281	O	GLN	A	665	-40.406	15.055	16.578	1.00	95.56	O
ATOM	5282	CG	GLN	A	665	-36.344	16.875	15.148	1.00	95.56	C
ATOM	5283	CD	GLN	A	665	-35.969	17.422	13.781	1.00	95.56	C
ATOM	5284	NE2	GLN	A	665	-34.875	18.141	13.703	1.00	95.56	N
ATOM	5285	OE1	GLN	A	665	-36.719	17.203	12.805	1.00	95.56	O
ATOM	5286	N	TRP	A	666	-40.281	17.094	17.266	1.00	93.31	N
ATOM	5287	CA	TRP	A	666	-41.719	17.234	17.312	1.00	93.31	C
ATOM	5288	C	TRP	A	666	-42.156	18.625	16.875	1.00	93.31	C
ATOM	5289	CB	TRP	A	666	-42.250	16.938	18.719	1.00	93.31	C
ATOM	5290	O	TRP	A	666	-41.344	19.562	16.906	1.00	93.31	O
ATOM	5291	CG	TRP	A	666	-41.750	17.891	19.766	1.00	93.31	C
ATOM	5292	CD1	TRP	A	666	-40.594	17.797	20.484	1.00	93.31	C
ATOM	5293	CD2	TRP	A	666	-42.406	19.078	20.219	1.00	93.31	C
ATOM	5294	CE2	TRP	A	666	-41.594	19.656	21.203	1.00	93.31	C
ATOM	5295	CE3	TRP	A	666	-43.625	19.703	19.875	1.00	93.31	C
ATOM	5296	NE1	TRP	A	666	-40.500	18.859	21.359	1.00	93.31	N
ATOM	5297	CH2	TRP	A	666	-43.125	21.438	21.516	1.00	93.31	C
ATOM	5298	CZ2	TRP	A	666	-41.938	20.844	21.859	1.00	93.31	C
ATOM	5299	CZ3	TRP	A	666	-43.969	20.891	20.531	1.00	93.31	C
ATOM	5300	N	ARG	A	667	-43.312	18.703	16.328	1.00	90.88	N
ATOM	5301	CA	ARG	A	667	-43.906	20.000	16.016	1.00	90.88	C
ATOM	5302	C	ARG	A	667	-45.406	20.000	16.312	1.00	90.88	C
ATOM	5303	CB	ARG	A	667	-43.656	20.375	14.562	1.00	90.88	C
ATOM	5304	O	ARG	A	667	-46.062	18.953	16.281	1.00	90.88	O
ATOM	5305	CG	ARG	A	667	-44.375	19.500	13.562	1.00	90.88	C
ATOM	5306	CD	ARG	A	667	-44.125	19.922	12.125	1.00	90.88	C
ATOM	5307	NE	ARG	A	667	-44.812	19.078	11.172	1.00	90.88	N
ATOM	5308	NH1	ARG	A	667	-43.938	20.109	9.297	1.00	90.88	N

ATOM	5309	NH2	ARG	A	667	-45.406	18.359	9.070	1.00	90.88	N
ATOM	5310	CZ	ARG	A	667	-44.719	19.188	9.844	1.00	90.88	C
ATOM	5311	N	LYS	A	668	-46.062	20.953	16.641	1.00	87.00	N
ATOM	5312	CA	LYS	A	668	-47.469	21.156	16.938	1.00	87.00	C
ATOM	5313	C	LYS	A	668	-48.156	21.969	15.828	1.00	87.00	C
ATOM	5314	CB	LYS	A	668	-47.656	21.859	18.281	1.00	87.00	C
ATOM	5315	O	LYS	A	668	-47.625	23.016	15.414	1.00	87.00	O
ATOM	5316	CG	LYS	A	668	-49.125	22.094	18.672	1.00	87.00	C
ATOM	5317	CD	LYS	A	668	-49.219	22.812	20.000	1.00	87.00	C
ATOM	5318	CE	LYS	A	668	-50.656	23.141	20.344	1.00	87.00	C
ATOM	5319	NZ	LYS	A	668	-51.125	24.391	19.656	1.00	87.00	N
ATOM	5320	N	ASP	A	669	-49.344	21.547	15.211	1.00	84.38	N
ATOM	5321	CA	ASP	A	669	-50.219	22.203	14.242	1.00	84.38	C
ATOM	5322	C	ASP	A	669	-49.406	22.688	13.031	1.00	84.38	C
ATOM	5323	CB	ASP	A	669	-50.938	23.375	14.898	1.00	84.38	C
ATOM	5324	O	ASP	A	669	-49.531	23.859	12.641	1.00	84.38	O
ATOM	5325	CG	ASP	A	669	-51.906	22.938	16.000	1.00	84.38	C
ATOM	5326	OD1	ASP	A	669	-52.531	21.859	15.875	1.00	84.38	O
ATOM	5327	OD2	ASP	A	669	-52.031	23.672	17.000	1.00	84.38	O
ATOM	5328	N	ASN	A	670	-48.438	21.859	12.516	1.00	80.31	N
ATOM	5329	CA	ASN	A	670	-47.656	22.109	11.297	1.00	80.31	C
ATOM	5330	C	ASN	A	670	-46.688	23.250	11.484	1.00	80.31	C
ATOM	5331	CB	ASN	A	670	-48.594	22.406	10.125	1.00	80.31	C
ATOM	5332	O	ASN	A	670	-46.406	23.984	10.539	1.00	80.31	O
ATOM	5333	CG	ASN	A	670	-49.344	21.172	9.656	1.00	80.31	C
ATOM	5334	ND2	ASN	A	670	-50.594	21.359	9.211	1.00	80.31	N
ATOM	5335	OD1	ASN	A	670	-48.812	20.047	9.688	1.00	80.31	O
ATOM	5336	N	GLY	A	671	-46.188	23.531	12.727	1.00	80.88	N
ATOM	5337	CA	GLY	A	671	-45.156	24.516	13.039	1.00	80.88	C
ATOM	5338	C	GLY	A	671	-43.750	24.016	12.742	1.00	80.88	C
ATOM	5339	O	GLY	A	671	-43.562	23.078	11.961	1.00	80.88	O
ATOM	5340	N	ASN	A	672	-42.719	24.703	13.227	1.00	85.06	N
ATOM	5341	CA	ASN	A	672	-41.312	24.312	13.039	1.00	85.06	C
ATOM	5342	C	ASN	A	672	-40.969	23.078	13.852	1.00	85.06	C
ATOM	5343	CB	ASN	A	672	-40.406	25.469	13.406	1.00	85.06	C
ATOM	5344	O	ASN	A	672	-41.500	22.859	14.945	1.00	85.06	O
ATOM	5345	CG	ASN	A	672	-40.500	26.625	12.430	1.00	85.06	C
ATOM	5346	ND2	ASN	A	672	-40.188	27.828	12.906	1.00	85.06	N
ATOM	5347	OD1	ASN	A	672	-40.844	26.453	11.258	1.00	85.06	O
ATOM	5348	N	TRP	A	673	-40.125	22.234	13.273	1.00	88.44	N
ATOM	5349	CA	TRP	A	673	-39.625	21.047	13.977	1.00	88.44	C
ATOM	5350	C	TRP	A	673	-38.781	21.438	15.164	1.00	88.44	C
ATOM	5351	CB	TRP	A	673	-38.844	20.156	13.023	1.00	88.44	C
ATOM	5352	O	TRP	A	673	-37.906	22.312	15.055	1.00	88.44	O
ATOM	5353	CG	TRP	A	673	-39.656	19.453	11.992	1.00	88.44	C
ATOM	5354	CD1	TRP	A	673	-39.844	19.812	10.688	1.00	88.44	C
ATOM	5355	CD2	TRP	A	673	-40.469	18.281	12.195	1.00	88.44	C
ATOM	5356	CE2	TRP	A	673	-41.062	17.969	10.961	1.00	88.44	C
ATOM	5357	CE3	TRP	A	673	-40.688	17.453	13.305	1.00	88.44	C
ATOM	5358	NE1	TRP	A	673	-40.688	18.922	10.062	1.00	88.44	N
ATOM	5359	CH2	TRP	A	673	-42.156	16.078	11.906	1.00	88.44	C
ATOM	5360	CZ2	TRP	A	673	-41.938	16.875	10.805	1.00	88.44	C
ATOM	5361	CZ3	TRP	A	673	-41.531	16.359	13.141	1.00	88.44	C
ATOM	5362	N	GLN	A	674	-39.031	20.906	16.344	1.00	90.44	N
ATOM	5363	CA	GLN	A	674	-38.250	21.094	17.562	1.00	90.44	C
ATOM	5364	C	GLN	A	674	-37.438	19.828	17.906	1.00	90.44	C
ATOM	5365	CB	GLN	A	674	-39.156	21.469	18.734	1.00	90.44	C
ATOM	5366	O	GLN	A	674	-38.000	18.719	17.875	1.00	90.44	O
ATOM	5367	CG	GLN	A	674	-39.938	22.750	18.516	1.00	90.44	C
ATOM	5368	CD	GLN	A	674	-39.062	24.000	18.609	1.00	90.44	C
ATOM	5369	NE2	GLN	A	674	-39.531	25.094	18.016	1.00	90.44	N
ATOM	5370	OE1	GLN	A	674	-38.000	23.969	19.203	1.00	90.44	O
ATOM	5371	N	ASN	A	675	-36.031	20.078	18.234	1.00	91.19	N
ATOM	5372	CA	ASN	A	675	-35.156	18.953	18.531	1.00	91.19	C

ATOM	5373	C	ASN	A	675	-35.156	18.594	20.016	1.00	91.19	C
ATOM	5374	CB	ASN	A	675	-33.750	19.234	18.047	1.00	91.19	C
ATOM	5375	O	ASN	A	675	-35.094	19.484	20.859	1.00	91.19	O
ATOM	5376	CG	ASN	A	675	-33.656	19.328	16.531	1.00	91.19	C
ATOM	5377	ND2	ASN	A	675	-32.938	20.344	16.062	1.00	91.19	N
ATOM	5378	OD1	ASN	A	675	-34.219	18.516	15.805	1.00	91.19	O
ATOM	5379	N	VAL	A	676	-35.375	17.406	20.359	1.00	91.31	N
ATOM	5380	CA	VAL	A	676	-35.125	16.891	21.703	1.00	91.31	C
ATOM	5381	C	VAL	A	676	-33.688	16.438	21.828	1.00	91.31	C
ATOM	5382	CB	VAL	A	676	-36.094	15.711	22.031	1.00	91.31	C
ATOM	5383	O	VAL	A	676	-33.094	15.953	20.875	1.00	91.31	O
ATOM	5384	CG1	VAL	A	676	-35.844	15.219	23.453	1.00	91.31	C
ATOM	5385	CG2	VAL	A	676	-37.531	16.141	21.844	1.00	91.31	C
ATOM	5386	N	PRO	A	677	-32.938	16.750	23.000	1.00	89.75	N
ATOM	5387	CA	PRO	A	677	-31.578	16.250	23.188	1.00	89.75	C
ATOM	5388	C	PRO	A	677	-31.453	14.766	22.844	1.00	89.75	C
ATOM	5389	CB	PRO	A	677	-31.312	16.500	24.672	1.00	89.75	C
ATOM	5390	O	PRO	A	677	-32.375	13.984	23.141	1.00	89.75	O
ATOM	5391	CG	PRO	A	677	-32.250	17.609	25.047	1.00	89.75	C
ATOM	5392	CD	PRO	A	677	-33.469	17.500	24.203	1.00	89.75	C
ATOM	5393	N	GLN	A	678	-30.438	14.492	22.172	1.00	91.25	N
ATOM	5394	CA	GLN	A	678	-30.156	13.109	21.781	1.00	91.25	C
ATOM	5395	C	GLN	A	678	-30.234	12.188	23.000	1.00	91.25	C
ATOM	5396	CB	GLN	A	678	-28.781	13.008	21.125	1.00	91.25	C
ATOM	5397	O	GLN	A	678	-29.781	12.547	24.094	1.00	91.25	O
ATOM	5398	CG	GLN	A	678	-28.500	11.648	20.500	1.00	91.25	C
ATOM	5399	CD	GLN	A	678	-27.172	11.594	19.766	1.00	91.25	C
ATOM	5400	NE2	GLN	A	678	-27.203	11.133	18.531	1.00	91.25	N
ATOM	5401	OE1	GLN	A	678	-26.141	11.977	20.328	1.00	91.25	O
ATOM	5402	N	THR	A	679	-30.891	11.094	22.906	1.00	92.69	N
ATOM	5403	CA	THR	A	679	-31.094	10.195	24.031	1.00	92.69	C
ATOM	5404	C	THR	A	679	-30.766	8.758	23.641	1.00	92.69	C
ATOM	5405	CB	THR	A	679	-32.562	10.258	24.547	1.00	92.69	C
ATOM	5406	O	THR	A	679	-30.922	8.367	22.484	1.00	92.69	O
ATOM	5407	CG2	THR	A	679	-33.531	9.820	23.469	1.00	92.69	C
ATOM	5408	OG1	THR	A	679	-32.688	9.398	25.688	1.00	92.69	O
ATOM	5409	N	ALA	A	680	-30.172	7.969	24.562	1.00	91.19	N
ATOM	5410	CA	ALA	A	680	-29.922	6.543	24.375	1.00	91.19	C
ATOM	5411	C	ALA	A	680	-31.156	5.711	24.719	1.00	91.19	C
ATOM	5412	CB	ALA	A	680	-28.734	6.102	25.234	1.00	91.19	C
ATOM	5413	O	ALA	A	680	-31.188	4.504	24.484	1.00	91.19	O
ATOM	5414	N	ASN	A	681	-32.219	6.449	25.312	1.00	93.00	N
ATOM	5415	CA	ASN	A	681	-33.438	5.766	25.719	1.00	93.00	C
ATOM	5416	C	ASN	A	681	-34.406	5.660	24.562	1.00	93.00	C
ATOM	5417	CB	ASN	A	681	-34.062	6.480	26.906	1.00	93.00	C
ATOM	5418	O	ASN	A	681	-34.281	6.348	23.547	1.00	93.00	O
ATOM	5419	CG	ASN	A	681	-33.219	6.398	28.156	1.00	93.00	C
ATOM	5420	ND2	ASN	A	681	-33.312	7.434	28.984	1.00	93.00	N
ATOM	5421	OD1	ASN	A	681	-32.531	5.418	28.391	1.00	93.00	O
ATOM	5422	N	LYS	A	682	-35.500	4.676	24.625	1.00	92.06	N
ATOM	5423	CA	LYS	A	682	-36.500	4.430	23.594	1.00	92.06	C
ATOM	5424	C	LYS	A	682	-37.719	5.297	23.812	1.00	92.06	C
ATOM	5425	CB	LYS	A	682	-36.875	2.951	23.578	1.00	92.06	C
ATOM	5426	O	LYS	A	682	-38.781	5.039	23.234	1.00	92.06	O
ATOM	5427	CG	LYS	A	682	-35.719	2.002	23.344	1.00	92.06	C
ATOM	5428	CD	LYS	A	682	-36.188	0.546	23.375	1.00	92.06	C
ATOM	5429	CE	LYS	A	682	-36.438	0.071	24.797	1.00	92.06	C
ATOM	5430	NZ	LYS	A	682	-36.750	-1.389	24.828	1.00	92.06	N
ATOM	5431	N	GLU	A	683	-37.594	6.285	24.625	1.00	93.62	N
ATOM	5432	CA	GLU	A	683	-38.688	7.227	24.891	1.00	93.62	C
ATOM	5433	C	GLU	A	683	-38.156	8.578	25.344	1.00	93.62	C
ATOM	5434	CB	GLU	A	683	-39.656	6.664	25.922	1.00	93.62	C
ATOM	5435	O	GLU	A	683	-37.062	8.656	25.922	1.00	93.62	O
ATOM	5436	CG	GLU	A	683	-39.000	6.402	27.281	1.00	93.62	C

```
ATOM   5437  CD  GLU A 683     -39.938   5.730  28.266  1.00 93.62           C
ATOM   5438  OE1 GLU A 683     -39.625   5.652  29.469  1.00 93.62           O
ATOM   5439  OE2 GLU A 683     -41.031   5.277  27.844  1.00 93.62           O
ATOM   5440  N   VAL A 684     -38.906   9.539  24.984  1.00 92.81           N
ATOM   5441  CA  VAL A 684     -38.594  10.875  25.500  1.00 92.81           C
ATOM   5442  C   VAL A 684     -39.875  11.516  26.047  1.00 92.81           C
ATOM   5443  CB  VAL A 684     -38.000  11.773  24.391  1.00 92.81           C
ATOM   5444  O   VAL A 684     -40.969  11.203  25.609  1.00 92.81           O
ATOM   5445  CG1 VAL A 684     -36.625  11.258  23.969  1.00 92.81           C
ATOM   5446  CG2 VAL A 684     -38.938  11.844  23.188  1.00 92.81           C
ATOM   5447  N   TYR A 685     -39.750  12.164  27.078  1.00 90.88           N
ATOM   5448  CA  TYR A 685     -40.844  12.859  27.719  1.00 90.88           C
ATOM   5449  C   TYR A 685     -40.750  14.359  27.516  1.00 90.88           C
ATOM   5450  CB  TYR A 685     -40.906  12.531  29.203  1.00 90.88           C
ATOM   5451  O   TYR A 685     -39.750  14.977  27.875  1.00 90.88           O
ATOM   5452  CG  TYR A 685     -41.062  11.055  29.500  1.00 90.88           C
ATOM   5453  CD1 TYR A 685     -42.312  10.492  29.750  1.00 90.88           C
ATOM   5454  CD2 TYR A 685     -39.938  10.227  29.547  1.00 90.88           C
ATOM   5455  CE1 TYR A 685     -42.438   9.133  30.016  1.00 90.88           C
ATOM   5456  CE2 TYR A 685     -40.062   8.875  29.828  1.00 90.88           C
ATOM   5457  OH  TYR A 685     -41.438   6.988  30.328  1.00 90.88           O
ATOM   5458  CZ  TYR A 685     -41.312   8.336  30.047  1.00 90.88           C
ATOM   5459  N   VAL A 686     -41.781  14.797  26.797  1.00 87.88           N
ATOM   5460  CA  VAL A 686     -41.875  16.234  26.609  1.00 87.88           C
ATOM   5461  C   VAL A 686     -43.000  16.797  27.484  1.00 87.88           C
ATOM   5462  CB  VAL A 686     -42.156  16.609  25.125  1.00 87.88           C
ATOM   5463  O   VAL A 686     -44.188  16.594  27.203  1.00 87.88           O
ATOM   5464  CG1 VAL A 686     -42.188  18.125  24.938  1.00 87.88           C
ATOM   5465  CG2 VAL A 686     -41.094  15.969  24.219  1.00 87.88           C
ATOM   5466  N   GLU A 687     -42.750  17.250  28.594  1.00 82.81           N
ATOM   5467  CA  GLU A 687     -43.656  17.719  29.625  1.00 82.81           C
ATOM   5468  C   GLU A 687     -44.250  19.078  29.266  1.00 82.81           C
ATOM   5469  CB  GLU A 687     -42.938  17.781  30.984  1.00 82.81           C
ATOM   5470  O   GLU A 687     -43.594  19.891  28.609  1.00 82.81           O
ATOM   5471  CG  GLU A 687     -42.625  16.422  31.578  1.00 82.81           C
ATOM   5472  CD  GLU A 687     -42.094  16.500  33.000  1.00 82.81           C
ATOM   5473  OE1 GLU A 687     -41.844  15.445  33.594  1.00 82.81           O
ATOM   5474  OE2 GLU A 687     -41.906  17.641  33.500  1.00 82.81           O
ATOM   5475  N   GLY A 688     -45.531  19.250  29.656  1.00 73.75           N
ATOM   5476  CA  GLY A 688     -46.156  20.562  29.578  1.00 73.75           C
ATOM   5477  C   GLY A 688     -46.625  20.906  28.188  1.00 73.75           C
ATOM   5478  O   GLY A 688     -46.469  22.047  27.734  1.00 73.75           O
ATOM   5479  N   ILE A 689     -47.219  19.906  27.484  1.00 74.50           N
ATOM   5480  CA  ILE A 689     -47.594  20.141  26.094  1.00 74.50           C
ATOM   5481  C   ILE A 689     -48.969  20.812  26.047  1.00 74.50           C
ATOM   5482  CB  ILE A 689     -47.625  18.828  25.281  1.00 74.50           C
ATOM   5483  O   ILE A 689     -49.781  20.625  26.953  1.00 74.50           O
ATOM   5484  CG1 ILE A 689     -48.656  17.844  25.891  1.00 74.50           C
ATOM   5485  CG2 ILE A 689     -46.219  18.188  25.234  1.00 74.50           C
ATOM   5486  CD1 ILE A 689     -49.062  16.734  24.953  1.00 74.50           C
ATOM   5487  N   TYR A 690     -49.344  21.797  25.219  1.00 69.62           N
ATOM   5488  CA  TYR A 690     -50.625  22.453  24.984  1.00 69.62           C
ATOM   5489  C   TYR A 690     -51.500  21.609  24.078  1.00 69.62           C
ATOM   5490  CB  TYR A 690     -50.406  23.828  24.344  1.00 69.62           C
ATOM   5491  O   TYR A 690     -51.031  20.719  23.375  1.00 69.62           O
ATOM   5492  CG  TYR A 690     -49.844  24.859  25.312  1.00 69.62           C
ATOM   5493  CD1 TYR A 690     -50.688  25.672  26.062  1.00 69.62           C
ATOM   5494  CD2 TYR A 690     -48.469  25.031  25.453  1.00 69.62           C
ATOM   5495  CE1 TYR A 690     -50.188  26.609  26.953  1.00 69.62           C
ATOM   5496  CE2 TYR A 690     -47.969  25.969  26.328  1.00 69.62           C
ATOM   5497  OH  TYR A 690     -48.312  27.703  27.938  1.00 69.62           O
ATOM   5498  CZ  TYR A 690     -48.812  26.766  27.062  1.00 69.62           C
ATOM   5499  N   ALA A 691     -52.781  21.906  24.234  1.00 76.12           N
ATOM   5500  CA  ALA A 691     -53.719  21.156  23.438  1.00 76.12           C
```

ATOM	5501	C	ALA	A	691	-53.531	21.406	21.938	1.00	76.12	C
ATOM	5502	CB	ALA	A	691	-55.156	21.516	23.828	1.00	76.12	C
ATOM	5503	O	ALA	A	691	-53.312	22.547	21.531	1.00	76.12	O
ATOM	5504	N	GLY	A	692	-53.188	20.578	21.188	1.00	80.56	N
ATOM	5505	CA	GLY	A	692	-53.094	20.656	19.734	1.00	80.56	C
ATOM	5506	C	GLY	A	692	-52.656	19.359	19.078	1.00	80.56	C
ATOM	5507	O	GLY	A	692	-52.625	18.312	19.734	1.00	80.56	O
ATOM	5508	N	ASN	A	693	-52.562	19.438	17.688	1.00	87.56	N
ATOM	5509	CA	ASN	A	693	-52.125	18.297	16.875	1.00	87.56	C
ATOM	5510	C	ASN	A	693	-50.594	18.219	16.781	1.00	87.56	C
ATOM	5511	CB	ASN	A	693	-52.719	18.375	15.477	1.00	87.56	C
ATOM	5512	O	ASN	A	693	-49.938	19.141	16.266	1.00	87.56	O
ATOM	5513	CG	ASN	A	693	-52.500	17.094	14.680	1.00	87.56	C
ATOM	5514	ND2	ASN	A	693	-52.531	17.219	13.352	1.00	87.56	N
ATOM	5515	OD1	ASN	A	693	-52.281	16.016	15.242	1.00	87.56	O
ATOM	5516	N	TYR	A	694	-49.969	17.250	17.469	1.00	90.62	N
ATOM	5517	CA	TYR	A	694	-48.500	17.078	17.484	1.00	90.62	C
ATOM	5518	C	TYR	A	694	-48.062	16.047	16.453	1.00	90.62	C
ATOM	5519	CB	TYR	A	694	-48.031	16.656	18.875	1.00	90.62	C
ATOM	5520	O	TYR	A	694	-48.688	15.000	16.312	1.00	90.62	O
ATOM	5521	CG	TYR	A	694	-48.125	17.766	19.906	1.00	90.62	C
ATOM	5522	CD1	TYR	A	694	-47.000	18.531	20.219	1.00	90.62	C
ATOM	5523	CD2	TYR	A	694	-49.312	18.047	20.562	1.00	90.62	C
ATOM	5524	CE1	TYR	A	694	-47.062	19.547	21.156	1.00	90.62	C
ATOM	5525	CE2	TYR	A	694	-49.375	19.062	21.500	1.00	90.62	C
ATOM	5526	OH	TYR	A	694	-48.312	20.812	22.734	1.00	90.62	O
ATOM	5527	CZ	TYR	A	694	-48.250	19.812	21.797	1.00	90.62	C
ATOM	5528	N	GLN	A	695	-47.000	16.531	15.727	1.00	93.94	N
ATOM	5529	CA	GLN	A	695	-46.312	15.633	14.805	1.00	93.94	C
ATOM	5530	C	GLN	A	695	-44.906	15.328	15.289	1.00	93.94	C
ATOM	5531	CB	GLN	A	695	-46.281	16.234	13.398	1.00	93.94	C
ATOM	5532	O	GLN	A	695	-44.188	16.234	15.742	1.00	93.94	O
ATOM	5533	CG	GLN	A	695	-47.656	16.484	12.797	1.00	93.94	C
ATOM	5534	CD	GLN	A	695	-47.594	17.219	11.477	1.00	93.94	C
ATOM	5535	NE2	GLN	A	695	-48.594	17.031	10.633	1.00	93.94	N
ATOM	5536	OE1	GLN	A	695	-46.656	17.953	11.203	1.00	93.94	O
ATOM	5537	N	VAL	A	696	-44.594	14.117	15.336	1.00	95.31	N
ATOM	5538	CA	VAL	A	696	-43.312	13.672	15.867	1.00	95.31	C
ATOM	5539	C	VAL	A	696	-42.594	12.773	14.844	1.00	95.31	C
ATOM	5540	CB	VAL	A	696	-43.469	12.922	17.203	1.00	95.31	C
ATOM	5541	O	VAL	A	696	-43.250	12.000	14.141	1.00	95.31	O
ATOM	5542	CG1	VAL	A	696	-42.125	12.391	17.703	1.00	95.31	C
ATOM	5543	CG2	VAL	A	696	-44.125	13.836	18.250	1.00	95.31	C
ATOM	5544	N	ARG	A	697	-41.312	12.938	14.812	1.00	95.12	N
ATOM	5545	CA	ARG	A	697	-40.531	11.977	14.055	1.00	95.12	C
ATOM	5546	C	ARG	A	697	-39.188	11.703	14.766	1.00	95.12	C
ATOM	5547	CB	ARG	A	697	-40.250	12.492	12.641	1.00	95.12	C
ATOM	5548	O	ARG	A	697	-38.688	12.562	15.477	1.00	95.12	O
ATOM	5549	CG	ARG	A	697	-39.469	13.789	12.594	1.00	95.12	C
ATOM	5550	CD	ARG	A	697	-39.281	14.281	11.164	1.00	95.12	C
ATOM	5551	NE	ARG	A	697	-38.469	15.508	11.117	1.00	95.12	N
ATOM	5552	NH1	ARG	A	697	-38.562	15.711	8.820	1.00	95.12	N
ATOM	5553	NH2	ARG	A	697	-37.438	17.266	10.078	1.00	95.12	N
ATOM	5554	CZ	ARG	A	697	-38.156	16.156	10.000	1.00	95.12	C
ATOM	5555	N	VAL	A	698	-38.719	10.508	14.656	1.00	96.25	N
ATOM	5556	CA	VAL	A	698	-37.531	10.016	15.359	1.00	96.25	C
ATOM	5557	C	VAL	A	698	-36.562	9.367	14.359	1.00	96.25	C
ATOM	5558	CB	VAL	A	698	-37.906	9.000	16.469	1.00	96.25	C
ATOM	5559	O	VAL	A	698	-37.000	8.711	13.414	1.00	96.25	O
ATOM	5560	CG1	VAL	A	698	-36.688	8.609	17.281	1.00	96.25	C
ATOM	5561	CG2	VAL	A	698	-39.000	9.578	17.375	1.00	96.25	C
ATOM	5562	N	ARG	A	699	-35.344	9.586	14.578	1.00	95.25	N
ATOM	5563	CA	ARG	A	699	-34.344	8.867	13.781	1.00	95.25	C
ATOM	5564	C	ARG	A	699	-33.281	8.250	14.672	1.00	95.25	C

ATOM	5565	CB	ARG	A	699	-33.719	9.797	12.750	1.00	95.25	C
ATOM	5566	O	ARG	A	699	-33.062	8.711	15.789	1.00	95.25	O
ATOM	5567	CG	ARG	A	699	-32.844	10.883	13.367	1.00	95.25	C
ATOM	5568	CD	ARG	A	699	-32.719	10.727	14.875	1.00	95.25	C
ATOM	5569	NE	ARG	A	699	-31.766	11.672	15.438	1.00	95.25	N
ATOM	5570	NH1	ARG	A	699	-32.062	10.977	17.625	1.00	95.25	N
ATOM	5571	NH2	ARG	A	699	-30.594	12.672	17.141	1.00	95.25	N
ATOM	5572	CZ	ARG	A	699	-31.469	11.773	16.734	1.00	95.25	C
ATOM	5573	N	SER	A	700	-32.688	7.262	14.219	1.00	93.50	N
ATOM	5574	CA	SER	A	700	-31.672	6.496	14.945	1.00	93.50	C
ATOM	5575	C	SER	A	700	-30.266	6.992	14.633	1.00	93.50	C
ATOM	5576	CB	SER	A	700	-31.781	5.008	14.602	1.00	93.50	C
ATOM	5577	O	SER	A	700	-30.000	7.426	13.508	1.00	93.50	O
ATOM	5578	OG	SER	A	700	-33.125	4.551	14.773	1.00	93.50	O
ATOM	5579	N	VAL	A	701	-29.391	6.992	15.680	1.00	90.38	N
ATOM	5580	CA	VAL	A	701	-28.000	7.387	15.531	1.00	90.38	C
ATOM	5581	C	VAL	A	701	-27.094	6.250	15.984	1.00	90.38	C
ATOM	5582	CB	VAL	A	701	-27.688	8.672	16.312	1.00	90.38	C
ATOM	5583	O	VAL	A	701	-27.234	5.738	17.094	1.00	90.38	O
ATOM	5584	CG1	VAL	A	701	-26.250	9.125	16.062	1.00	90.38	C
ATOM	5585	CG2	VAL	A	701	-28.672	9.781	15.945	1.00	90.38	C
ATOM	5586	N	ALA	A	702	-26.156	5.922	15.070	1.00	83.88	N
ATOM	5587	CA	ALA	A	702	-25.203	4.871	15.398	1.00	83.88	C
ATOM	5588	C	ALA	A	702	-24.016	5.426	16.188	1.00	83.88	C
ATOM	5589	CB	ALA	A	702	-24.703	4.180	14.133	1.00	83.88	C
ATOM	5590	O	ALA	A	702	-23.844	6.645	16.281	1.00	83.88	O
ATOM	5591	N	GLY	A	703	-23.188	4.676	16.859	1.00	76.00	N
ATOM	5592	CA	GLY	A	703	-21.984	5.062	17.578	1.00	76.00	C
ATOM	5593	C	GLY	A	703	-21.000	5.848	16.734	1.00	76.00	C
ATOM	5594	O	GLY	A	703	-20.281	6.703	17.250	1.00	76.00	O
ATOM	5595	N	SER	A	704	-20.969	5.594	15.359	1.00	74.81	N
ATOM	5596	CA	SER	A	704	-20.062	6.270	14.438	1.00	74.81	C
ATOM	5597	C	SER	A	704	-20.594	7.645	14.047	1.00	74.81	C
ATOM	5598	CB	SER	A	704	-19.844	5.426	13.180	1.00	74.81	C
ATOM	5599	O	SER	A	704	-19.891	8.438	13.422	1.00	74.81	O
ATOM	5600	OG	SER	A	704	-21.078	5.160	12.531	1.00	74.81	O
ATOM	5601	N	GLY	A	705	-21.906	7.941	14.469	1.00	77.56	N
ATOM	5602	CA	GLY	A	705	-22.516	9.211	14.117	1.00	77.56	C
ATOM	5603	C	GLY	A	705	-23.422	9.117	12.898	1.00	77.56	C
ATOM	5604	O	GLY	A	705	-24.062	10.102	12.523	1.00	77.56	O
ATOM	5605	N	THR	A	706	-23.516	7.965	12.133	1.00	81.94	N
ATOM	5606	CA	THR	A	706	-24.422	7.746	11.008	1.00	81.94	C
ATOM	5607	C	THR	A	706	-25.875	7.754	11.477	1.00	81.94	C
ATOM	5608	CB	THR	A	706	-24.109	6.418	10.297	1.00	81.94	C
ATOM	5609	O	THR	A	706	-26.203	7.176	12.516	1.00	81.94	O
ATOM	5610	CG2	THR	A	706	-24.875	6.312	8.977	1.00	81.94	C
ATOM	5611	OG1	THR	A	706	-22.703	6.336	10.031	1.00	81.94	O
ATOM	5612	N	THR	A	707	-26.781	8.508	10.750	1.00	89.12	N
ATOM	5613	CA	THR	A	707	-28.172	8.641	11.141	1.00	89.12	C
ATOM	5614	C	THR	A	707	-29.094	7.973	10.125	1.00	89.12	C
ATOM	5615	CB	THR	A	707	-28.578	10.117	11.305	1.00	89.12	C
ATOM	5616	O	THR	A	707	-28.750	7.871	8.945	1.00	89.12	O
ATOM	5617	CG2	THR	A	707	-27.719	10.805	12.367	1.00	89.12	C
ATOM	5618	OG1	THR	A	707	-28.391	10.797	10.055	1.00	89.12	O
ATOM	5619	N	SER	A	708	-30.156	7.355	10.562	1.00	90.75	N
ATOM	5620	CA	SER	A	708	-31.203	6.855	9.672	1.00	90.75	C
ATOM	5621	C	SER	A	708	-32.031	7.996	9.109	1.00	90.75	C
ATOM	5622	CB	SER	A	708	-32.094	5.871	10.422	1.00	90.75	C
ATOM	5623	O	SER	A	708	-31.875	9.148	9.516	1.00	90.75	O
ATOM	5624	OG	SER	A	708	-33.000	6.555	11.289	1.00	90.75	O
ATOM	5625	N	GLY	A	709	-32.781	7.695	8.078	1.00	89.12	N
ATOM	5626	CA	GLY	A	709	-33.844	8.625	7.770	1.00	89.12	C
ATOM	5627	C	GLY	A	709	-34.812	8.836	8.930	1.00	89.12	C
ATOM	5628	O	GLY	A	709	-34.750	8.102	9.922	1.00	89.12	O

ATOM	5629	N	TRP	A	710	-35.656	9.859	8.906	1.00	93.94	N
ATOM	5630	CA	TRP	A	710	-36.656	10.117	9.953	1.00	93.94	C
ATOM	5631	C	TRP	A	710	-37.781	9.109	9.883	1.00	93.94	C
ATOM	5632	CB	TRP	A	710	-37.188	11.539	9.820	1.00	93.94	C
ATOM	5633	O	TRP	A	710	-38.125	8.625	8.805	1.00	93.94	O
ATOM	5634	CG	TRP	A	710	-36.188	12.617	10.117	1.00	93.94	C
ATOM	5635	CD1	TRP	A	710	-35.469	13.328	9.211	1.00	93.94	C
ATOM	5636	CD2	TRP	A	710	-35.812	13.094	11.406	1.00	93.94	C
ATOM	5637	CE2	TRP	A	710	-34.844	14.102	11.203	1.00	93.94	C
ATOM	5638	CE3	TRP	A	710	-36.188	12.773	12.719	1.00	93.94	C
ATOM	5639	NE1	TRP	A	710	-34.656	14.227	9.859	1.00	93.94	N
ATOM	5640	CH2	TRP	A	710	-34.625	14.445	13.539	1.00	93.94	C
ATOM	5641	CZ2	TRP	A	710	-34.250	14.781	12.266	1.00	93.94	C
ATOM	5642	CZ3	TRP	A	710	-35.594	13.453	13.773	1.00	93.94	C
ATOM	5643	N	SER	A	711	-38.344	8.656	10.977	1.00	93.44	N
ATOM	5644	CA	SER	A	711	-39.562	7.852	11.000	1.00	93.44	C
ATOM	5645	C	SER	A	711	-40.719	8.578	10.320	1.00	93.44	C
ATOM	5646	CB	SER	A	711	-39.938	7.496	12.438	1.00	93.44	C
ATOM	5647	O	SER	A	711	-40.594	9.766	10.000	1.00	93.44	O
ATOM	5648	OG	SER	A	711	-40.469	8.633	13.117	1.00	93.44	O
ATOM	5649	N	ASN	A	712	-41.719	7.750	9.969	1.00	91.25	N
ATOM	5650	CA	ASN	A	712	-42.938	8.414	9.523	1.00	91.25	C
ATOM	5651	C	ASN	A	712	-43.438	9.422	10.555	1.00	91.25	C
ATOM	5652	CB	ASN	A	712	-44.031	7.379	9.234	1.00	91.25	C
ATOM	5653	O	ASN	A	712	-43.250	9.219	11.758	1.00	91.25	O
ATOM	5654	CG	ASN	A	712	-43.719	6.531	8.016	1.00	91.25	C
ATOM	5655	ND2	ASN	A	712	-44.219	5.305	8.008	1.00	91.25	N
ATOM	5656	OD1	ASN	A	712	-43.031	6.969	7.098	1.00	91.25	O
ATOM	5657	N	ILE	A	713	-43.875	10.562	10.086	1.00	92.25	N
ATOM	5658	CA	ILE	A	713	-44.438	11.570	10.984	1.00	92.25	C
ATOM	5659	C	ILE	A	713	-45.688	11.016	11.656	1.00	92.25	C
ATOM	5660	CB	ILE	A	713	-44.750	12.883	10.234	1.00	92.25	C
ATOM	5661	O	ILE	A	713	-46.594	10.516	10.984	1.00	92.25	O
ATOM	5662	CG1	ILE	A	713	-43.469	13.500	9.672	1.00	92.25	C
ATOM	5663	CG2	ILE	A	713	-45.500	13.859	11.156	1.00	92.25	C
ATOM	5664	CD1	ILE	A	713	-43.719	14.688	8.742	1.00	92.25	C
ATOM	5665	N	VAL	A	714	-45.750	10.852	12.977	1.00	92.50	N
ATOM	5666	CA	VAL	A	714	-46.938	10.453	13.758	1.00	92.50	C
ATOM	5667	C	VAL	A	714	-47.562	11.680	14.383	1.00	92.50	C
ATOM	5668	CB	VAL	A	714	-46.531	9.438	14.859	1.00	92.50	C
ATOM	5669	O	VAL	A	714	-46.906	12.477	15.039	1.00	92.50	O
ATOM	5670	CG1	VAL	A	714	-47.781	9.047	15.680	1.00	92.50	C
ATOM	5671	CG2	VAL	A	714	-45.906	8.195	14.234	1.00	92.50	C
ATOM	5672	N	ALA	A	715	-48.812	11.805	13.969	1.00	90.75	N
ATOM	5673	CA	ALA	A	715	-49.562	12.953	14.484	1.00	90.75	C
ATOM	5674	C	ALA	A	715	-50.562	12.516	15.555	1.00	90.75	C
ATOM	5675	CB	ALA	A	715	-50.281	13.664	13.344	1.00	90.75	C
ATOM	5676	O	ALA	A	715	-51.156	11.453	15.453	1.00	90.75	O
ATOM	5677	N	ALA	A	716	-50.594	13.008	16.641	1.00	89.25	N
ATOM	5678	CA	ALA	A	716	-51.562	12.789	17.719	1.00	89.25	C
ATOM	5679	C	ALA	A	716	-52.125	14.109	18.234	1.00	89.25	C
ATOM	5680	CB	ALA	A	716	-50.906	11.992	18.844	1.00	89.25	C
ATOM	5681	O	ALA	A	716	-51.375	15.070	18.438	1.00	89.25	O
ATOM	5682	N	THR	A	717	-53.500	14.133	18.219	1.00	85.38	N
ATOM	5683	CA	THR	A	717	-54.156	15.266	18.859	1.00	85.38	C
ATOM	5684	C	THR	A	717	-54.156	15.086	20.375	1.00	85.38	C
ATOM	5685	CB	THR	A	717	-55.594	15.453	18.344	1.00	85.38	C
ATOM	5686	O	THR	A	717	-54.750	14.117	20.891	1.00	85.38	O
ATOM	5687	CG2	THR	A	717	-56.219	16.719	18.922	1.00	85.38	C
ATOM	5688	OG1	THR	A	717	-55.562	15.547	16.922	1.00	85.38	O
ATOM	5689	N	LEU	A	718	-53.375	15.727	21.062	1.00	80.81	N
ATOM	5690	CA	LEU	A	718	-53.219	15.594	22.500	1.00	80.81	C
ATOM	5691	C	LEU	A	718	-53.906	16.750	23.234	1.00	80.81	C
ATOM	5692	CB	LEU	A	718	-51.719	15.539	22.891	1.00	80.81	C

ATOM	5693	O	LEU	A	718	-53.938	17.859	22.719	1.00	80.81	O
ATOM	5694	CG	LEU	A	718	-50.906	14.469	22.188	1.00	80.81	C
ATOM	5695	CD1	LEU	A	718	-49.438	14.578	22.609	1.00	80.81	C
ATOM	5696	CD2	LEU	A	718	-51.438	13.078	22.484	1.00	80.81	C
ATOM	5697	N	THR	A	719	-54.781	16.344	24.219	1.00	67.56	N
ATOM	5698	CA	THR	A	719	-55.500	17.266	25.094	1.00	67.56	C
ATOM	5699	C	THR	A	719	-54.562	17.875	26.125	1.00	67.56	C
ATOM	5700	CB	THR	A	719	-56.688	16.578	25.797	1.00	67.56	C
ATOM	5701	O	THR	A	719	-53.625	17.203	26.578	1.00	67.56	O
ATOM	5702	CG2	THR	A	719	-57.781	16.188	24.812	1.00	67.56	C
ATOM	5703	OG1	THR	A	719	-56.188	15.391	26.453	1.00	67.56	O
ATOM	5704	N	GLY	A	720	-54.188	19.141	25.906	1.00	59.06	N
ATOM	5705	CA	GLY	A	720	-53.250	19.859	26.750	1.00	59.06	C
ATOM	5706	C	GLY	A	720	-53.844	21.094	27.375	1.00	59.06	C
ATOM	5707	O	GLY	A	720	-55.062	21.234	27.484	1.00	59.06	O
ATOM	5708	N	LYS	A	721	-53.406	21.859	28.094	1.00	53.00	N
ATOM	5709	CA	LYS	A	721	-53.719	23.047	28.875	1.00	53.00	C
ATOM	5710	C	LYS	A	721	-54.438	24.094	28.016	1.00	53.00	C
ATOM	5711	CB	LYS	A	721	-52.469	23.641	29.500	1.00	53.00	C
ATOM	5712	O	LYS	A	721	-54.000	24.375	26.891	1.00	53.00	O
ATOM	5713	CG	LYS	A	721	-52.750	24.625	30.641	1.00	53.00	C
ATOM	5714	CD	LYS	A	721	-51.469	25.109	31.281	1.00	53.00	C
ATOM	5715	CE	LYS	A	721	-51.719	26.141	32.375	1.00	53.00	C
ATOM	5716	NZ	LYS	A	721	-50.469	26.594	33.031	1.00	53.00	N
ATOM	5717	N	GLN	A	722	-55.844	24.562	28.094	1.00	49.41	N
ATOM	5718	CA	GLN	A	722	-56.750	25.594	27.594	1.00	49.41	C
ATOM	5719	C	GLN	A	722	-56.750	26.797	28.516	1.00	49.41	C
ATOM	5720	CB	GLN	A	722	-58.156	25.031	27.453	1.00	49.41	C
ATOM	5721	O	GLN	A	722	-56.812	26.656	29.734	1.00	49.41	O
ATOM	5722	CG	GLN	A	722	-58.344	24.141	26.234	1.00	49.41	C
ATOM	5723	CD	GLN	A	722	-59.812	23.797	25.969	1.00	49.41	C
ATOM	5724	NE2	GLN	A	722	-60.062	23.172	24.828	1.00	49.41	N
ATOM	5725	OE1	GLN	A	722	-60.688	24.094	26.781	1.00	49.41	O
ATOM	5726	N	GLY	A	723	-56.250	28.125	27.953	1.00	58.53	N
ATOM	5727	CA	GLY	A	723	-56.156	29.375	28.672	1.00	58.53	C
ATOM	5728	C	GLY	A	723	-54.844	30.109	28.422	1.00	58.53	C
ATOM	5729	O	GLY	A	723	-53.969	29.594	27.750	1.00	58.53	O
ATOM	5730	N	GLU	A	724	-54.938	31.641	28.422	1.00	59.62	N
ATOM	5731	CA	GLU	A	724	-53.781	32.500	28.250	1.00	59.62	C
ATOM	5732	C	GLU	A	724	-52.688	32.188	29.266	1.00	59.62	C
ATOM	5733	CB	GLU	A	724	-54.188	33.969	28.391	1.00	59.62	C
ATOM	5734	O	GLU	A	724	-52.969	31.891	30.422	1.00	59.62	O
ATOM	5735	CG	GLU	A	724	-55.031	34.500	27.234	1.00	59.62	C
ATOM	5736	CD	GLU	A	724	-55.438	35.969	27.375	1.00	59.62	C
ATOM	5737	OE1	GLU	A	724	-56.031	36.531	26.438	1.00	59.62	O
ATOM	5738	OE2	GLU	A	724	-55.125	36.562	28.438	1.00	59.62	O
ATOM	5739	N	PRO	A	725	-51.531	31.781	28.734	1.00	70.50	N
ATOM	5740	CA	PRO	A	725	-50.406	31.391	29.594	1.00	70.50	C
ATOM	5741	C	PRO	A	725	-50.031	32.469	30.594	1.00	70.50	C
ATOM	5742	CB	PRO	A	725	-49.281	31.156	28.594	1.00	70.50	C
ATOM	5743	O	PRO	A	725	-50.312	33.656	30.375	1.00	70.50	O
ATOM	5744	CG	PRO	A	725	-49.719	31.781	27.328	1.00	70.50	C
ATOM	5745	CD	PRO	A	725	-51.219	32.031	27.438	1.00	70.50	C
ATOM	5746	N	GLY	A	726	-49.906	32.125	31.859	1.00	79.44	N
ATOM	5747	CA	GLY	A	726	-49.469	33.031	32.906	1.00	79.44	C
ATOM	5748	C	GLY	A	726	-48.219	33.844	32.500	1.00	79.44	C
ATOM	5749	O	GLY	A	726	-47.375	33.344	31.766	1.00	79.44	O
ATOM	5750	N	ARG	A	727	-48.250	35.188	32.750	1.00	86.94	N
ATOM	5751	CA	ARG	A	727	-47.094	36.031	32.469	1.00	86.94	C
ATOM	5752	C	ARG	A	727	-45.875	35.562	33.219	1.00	86.94	C
ATOM	5753	CB	ARG	A	727	-47.406	37.500	32.844	1.00	86.94	C
ATOM	5754	O	ARG	A	727	-45.969	35.062	34.344	1.00	86.94	O
ATOM	5755	CG	ARG	A	727	-47.281	37.781	34.312	1.00	86.94	C
ATOM	5756	CD	ARG	A	727	-47.406	39.281	34.594	1.00	86.94	C

ATOM	5757	NE	ARG	A	727	-47.625	39.562	36.031	1.00	86.94	N
ATOM	5758	NH1	ARG	A	727	-48.219	41.781	35.750	1.00	86.94	N
ATOM	5759	NH2	ARG	A	727	-48.188	40.844	37.844	1.00	86.94	N
ATOM	5760	CZ	ARG	A	727	-48.000	40.719	36.531	1.00	86.94	C
ATOM	5761	N	PRO	A	728	-44.625	35.438	32.531	1.00	90.69	N
ATOM	5762	CA	PRO	A	728	-43.375	35.094	33.250	1.00	90.69	C
ATOM	5763	C	PRO	A	728	-43.188	35.875	34.531	1.00	90.69	C
ATOM	5764	CB	PRO	A	728	-42.281	35.406	32.250	1.00	90.69	C
ATOM	5765	O	PRO	A	728	-43.688	37.000	34.656	1.00	90.69	O
ATOM	5766	CG	PRO	A	728	-42.969	35.344	30.906	1.00	90.69	C
ATOM	5767	CD	PRO	A	728	-44.406	35.781	31.078	1.00	90.69	C
ATOM	5768	N	ILE	A	729	-42.688	35.219	35.656	1.00	90.25	N
ATOM	5769	CA	ILE	A	729	-42.438	35.844	36.938	1.00	90.25	C
ATOM	5770	C	ILE	A	729	-40.969	35.875	37.250	1.00	90.25	C
ATOM	5771	CB	ILE	A	729	-43.219	35.125	38.062	1.00	90.25	C
ATOM	5772	O	ILE	A	729	-40.188	35.125	36.656	1.00	90.25	O
ATOM	5773	CG1	ILE	A	729	-42.844	33.625	38.094	1.00	90.25	C
ATOM	5774	CG2	ILE	A	729	-44.719	35.312	37.906	1.00	90.25	C
ATOM	5775	CD1	ILE	A	729	-43.406	32.875	39.312	1.00	90.25	C
ATOM	5776	N	ASN	A	730	-40.469	36.812	37.875	1.00	90.88	N
ATOM	5777	CA	ASN	A	730	-39.125	36.969	38.406	1.00	90.88	C
ATOM	5778	C	ASN	A	730	-38.094	37.219	37.281	1.00	90.88	C
ATOM	5779	CB	ASN	A	730	-38.719	35.750	39.219	1.00	90.88	C
ATOM	5780	O	ASN	A	730	-37.000	36.656	37.312	1.00	90.88	O
ATOM	5781	CG	ASN	A	730	-39.562	35.594	40.469	1.00	90.88	C
ATOM	5782	ND2	ASN	A	730	-39.812	34.344	40.875	1.00	90.88	N
ATOM	5783	OD1	ASN	A	730	-40.031	36.594	41.062	1.00	90.88	O
ATOM	5784	N	LEU	A	731	-38.562	37.906	36.188	1.00	93.69	N
ATOM	5785	CA	LEU	A	731	-37.562	38.250	35.156	1.00	93.69	C
ATOM	5786	C	LEU	A	731	-36.438	39.062	35.750	1.00	93.69	C
ATOM	5787	CB	LEU	A	731	-38.250	39.031	34.031	1.00	93.69	C
ATOM	5788	O	LEU	A	731	-36.656	40.062	36.406	1.00	93.69	O
ATOM	5789	CG	LEU	A	731	-37.312	39.562	32.938	1.00	93.69	C
ATOM	5790	CD1	LEU	A	731	-36.688	38.406	32.156	1.00	93.69	C
ATOM	5791	CD2	LEU	A	731	-38.062	40.500	31.969	1.00	93.69	C
ATOM	5792	N	THR	A	732	-35.219	38.469	35.594	1.00	93.06	N
ATOM	5793	CA	THR	A	732	-34.031	39.188	36.000	1.00	93.06	C
ATOM	5794	C	THR	A	732	-33.031	39.312	34.844	1.00	93.06	C
ATOM	5795	CB	THR	A	732	-33.344	38.500	37.188	1.00	93.06	C
ATOM	5796	O	THR	A	732	-33.031	38.469	33.938	1.00	93.06	O
ATOM	5797	CG2	THR	A	732	-34.250	38.469	38.406	1.00	93.06	C
ATOM	5798	OG1	THR	A	732	-33.000	37.156	36.812	1.00	93.06	O
ATOM	5799	N	ALA	A	733	-32.406	40.312	34.719	1.00	93.31	N
ATOM	5800	CA	ALA	A	733	-31.281	40.531	33.844	1.00	93.31	C
ATOM	5801	C	ALA	A	733	-30.000	40.844	34.625	1.00	93.31	C
ATOM	5802	CB	ALA	A	733	-31.594	41.688	32.875	1.00	93.31	C
ATOM	5803	O	ALA	A	733	-30.031	41.625	35.562	1.00	93.31	O
ATOM	5804	N	THR	A	734	-28.969	40.062	34.250	1.00	91.25	N
ATOM	5805	CA	THR	A	734	-27.734	40.188	35.031	1.00	91.25	C
ATOM	5806	C	THR	A	734	-27.047	41.500	34.781	1.00	91.25	C
ATOM	5807	CB	THR	A	734	-26.766	39.031	34.719	1.00	91.25	C
ATOM	5808	O	THR	A	734	-27.328	42.156	33.750	1.00	91.25	O
ATOM	5809	CG2	THR	A	734	-27.359	37.688	35.094	1.00	91.25	C
ATOM	5810	OG1	THR	A	734	-26.484	39.031	33.312	1.00	91.25	O
ATOM	5811	N	ASP	A	735	-26.328	41.906	35.688	1.00	86.88	N
ATOM	5812	CA	ASP	A	735	-25.438	43.062	35.531	1.00	86.88	C
ATOM	5813	C	ASP	A	735	-23.984	42.656	35.750	1.00	86.88	C
ATOM	5814	CB	ASP	A	735	-25.828	44.156	36.500	1.00	86.88	C
ATOM	5815	O	ASP	A	735	-23.125	43.531	35.906	1.00	86.88	O
ATOM	5816	CG	ASP	A	735	-25.703	43.781	37.938	1.00	86.88	C
ATOM	5817	OD1	ASP	A	735	-25.328	42.625	38.219	1.00	86.88	O
ATOM	5818	OD2	ASP	A	735	-25.969	44.625	38.812	1.00	86.88	O
ATOM	5819	N	ASP	A	736	-23.703	41.281	35.719	1.00	83.94	N
ATOM	5820	CA	ASP	A	736	-22.344	40.875	36.094	1.00	83.94	C

ATOM	5821	C	ASP	A	736	-21.719	40.000	35.031	1.00	83.94	C
ATOM	5822	CB	ASP	A	736	-22.359	40.156	37.438	1.00	83.94	C
ATOM	5823	O	ASP	A	736	-20.625	39.469	35.219	1.00	83.94	O
ATOM	5824	CG	ASP	A	736	-23.234	38.938	37.469	1.00	83.94	C
ATOM	5825	OD1	ASP	A	736	-24.047	38.750	36.500	1.00	83.94	O
ATOM	5826	OD2	ASP	A	736	-23.125	38.125	38.406	1.00	83.94	O
ATOM	5827	N	VAL	A	737	-22.406	39.781	33.906	1.00	87.44	N
ATOM	5828	CA	VAL	A	737	-21.922	38.875	32.844	1.00	87.44	C
ATOM	5829	C	VAL	A	737	-21.000	39.656	31.906	1.00	87.44	C
ATOM	5830	CB	VAL	A	737	-23.094	38.250	32.062	1.00	87.44	C
ATOM	5831	O	VAL	A	737	-21.359	40.719	31.406	1.00	87.44	O
ATOM	5832	CG1	VAL	A	737	-22.562	37.375	30.938	1.00	87.44	C
ATOM	5833	CG2	VAL	A	737	-23.984	37.438	33.000	1.00	87.44	C
ATOM	5834	N	VAL	A	738	-19.766	39.125	31.703	1.00	84.44	N
ATOM	5835	CA	VAL	A	738	-18.750	39.781	30.891	1.00	84.44	C
ATOM	5836	C	VAL	A	738	-19.172	39.750	29.422	1.00	84.44	C
ATOM	5837	CB	VAL	A	738	-17.359	39.125	31.078	1.00	84.44	C
ATOM	5838	O	VAL	A	738	-19.531	38.688	28.906	1.00	84.44	O
ATOM	5839	CG1	VAL	A	738	-16.344	39.750	30.141	1.00	84.44	C
ATOM	5840	CG2	VAL	A	738	-16.906	39.250	32.531	1.00	84.44	C
ATOM	5841	N	PHE	A	739	-19.250	40.875	28.719	1.00	86.62	N
ATOM	5842	CA	PHE	A	739	-19.562	41.062	27.312	1.00	86.62	C
ATOM	5843	C	PHE	A	739	-20.938	40.500	26.969	1.00	86.62	C
ATOM	5844	CB	PHE	A	739	-18.500	40.438	26.406	1.00	86.62	C
ATOM	5845	O	PHE	A	739	-21.188	40.062	25.828	1.00	86.62	O
ATOM	5846	CG	PHE	A	739	-17.125	41.031	26.594	1.00	86.62	C
ATOM	5847	CD1	PHE	A	739	-16.969	42.406	26.641	1.00	86.62	C
ATOM	5848	CD2	PHE	A	739	-16.016	40.219	26.703	1.00	86.62	C
ATOM	5849	CE1	PHE	A	739	-15.695	42.969	26.797	1.00	86.62	C
ATOM	5850	CE2	PHE	A	739	-14.742	40.781	26.859	1.00	86.62	C
ATOM	5851	CZ	PHE	A	739	-14.586	42.156	26.906	1.00	86.62	C
ATOM	5852	N	GLY	A	740	-21.766	40.438	28.062	1.00	91.75	N
ATOM	5853	CA	GLY	A	740	-23.094	39.938	27.797	1.00	91.75	C
ATOM	5854	C	GLY	A	740	-24.062	40.156	28.938	1.00	91.75	C
ATOM	5855	O	GLY	A	740	-23.672	40.625	30.016	1.00	91.75	O
ATOM	5856	N	ILE	A	741	-25.281	39.969	28.781	1.00	92.75	N
ATOM	5857	CA	ILE	A	741	-26.375	40.031	29.750	1.00	92.75	C
ATOM	5858	C	ILE	A	741	-27.172	38.719	29.719	1.00	92.75	C
ATOM	5859	CB	ILE	A	741	-27.312	41.219	29.453	1.00	92.75	C
ATOM	5860	O	ILE	A	741	-27.531	38.219	28.656	1.00	92.75	O
ATOM	5861	CG1	ILE	A	741	-26.547	42.531	29.562	1.00	92.75	C
ATOM	5862	CG2	ILE	A	741	-28.516	41.219	30.406	1.00	92.75	C
ATOM	5863	CD1	ILE	A	741	-27.312	43.750	29.031	1.00	92.75	C
ATOM	5864	N	ARG	A	742	-27.250	38.156	30.906	1.00	93.19	N
ATOM	5865	CA	ARG	A	742	-28.062	36.969	31.047	1.00	93.19	C
ATOM	5866	C	ARG	A	742	-29.422	37.281	31.656	1.00	93.19	C
ATOM	5867	CB	ARG	A	742	-27.359	35.906	31.906	1.00	93.19	C
ATOM	5868	O	ARG	A	742	-29.500	37.906	32.719	1.00	93.19	O
ATOM	5869	CG	ARG	A	742	-28.062	34.562	31.953	1.00	93.19	C
ATOM	5870	CD	ARG	A	742	-27.328	33.562	32.844	1.00	93.19	C
ATOM	5871	NE	ARG	A	742	-26.219	32.938	32.125	1.00	93.19	N
ATOM	5872	NH1	ARG	A	742	-25.641	31.531	33.875	1.00	93.19	N
ATOM	5873	NH2	ARG	A	742	-24.469	31.469	31.891	1.00	93.19	N
ATOM	5874	CZ	ARG	A	742	-25.438	31.984	32.625	1.00	93.19	C
ATOM	5875	N	THR	A	743	-30.453	37.031	30.922	1.00	94.06	N
ATOM	5876	CA	THR	A	743	-31.828	37.188	31.391	1.00	94.06	C
ATOM	5877	C	THR	A	743	-32.438	35.844	31.797	1.00	94.06	C
ATOM	5878	CB	THR	A	743	-32.719	37.844	30.312	1.00	94.06	C
ATOM	5879	O	THR	A	743	-32.188	34.844	31.141	1.00	94.06	O
ATOM	5880	CG2	THR	A	743	-32.156	39.188	29.891	1.00	94.06	C
ATOM	5881	OG1	THR	A	743	-32.750	36.969	29.156	1.00	94.06	O
ATOM	5882	N	LYS	A	744	-33.000	35.938	33.062	1.00	93.81	N
ATOM	5883	CA	LYS	A	744	-33.656	34.750	33.594	1.00	93.81	C
ATOM	5884	C	LYS	A	744	-35.094	35.094	34.062	1.00	93.81	C

ATOM	5885	CB	LYS	A	744	-32.875	34.188	34.750	1.00	93.81	C
ATOM	5886	O	LYS	A	744	-35.312	36.188	34.594	1.00	93.81	O
ATOM	5887	CG	LYS	A	744	-31.516	33.594	34.344	1.00	93.81	C
ATOM	5888	CD	LYS	A	744	-30.797	32.969	35.531	1.00	93.81	C
ATOM	5889	CE	LYS	A	744	-29.438	32.406	35.125	1.00	93.81	C
ATOM	5890	NZ	LYS	A	744	-28.734	31.781	36.281	1.00	93.81	N
ATOM	5891	N	TRP	A	745	-35.969	34.312	33.812	1.00	93.88	N
ATOM	5892	CA	TRP	A	745	-37.344	34.469	34.281	1.00	93.88	C
ATOM	5893	C	TRP	A	745	-37.938	33.156	34.781	1.00	93.88	C
ATOM	5894	CB	TRP	A	745	-38.219	35.031	33.125	1.00	93.88	C
ATOM	5895	O	TRP	A	745	-37.344	32.094	34.562	1.00	93.88	O
ATOM	5896	CG	TRP	A	745	-38.312	34.125	31.953	1.00	93.88	C
ATOM	5897	CD1	TRP	A	745	-39.281	33.188	31.734	1.00	93.88	C
ATOM	5898	CD2	TRP	A	745	-37.438	34.094	30.828	1.00	93.88	C
ATOM	5899	CE2	TRP	A	745	-37.938	33.062	29.953	1.00	93.88	C
ATOM	5900	CE3	TRP	A	745	-36.312	34.781	30.453	1.00	93.88	C
ATOM	5901	NE1	TRP	A	745	-39.062	32.531	30.531	1.00	93.88	N
ATOM	5902	CH2	TRP	A	745	-36.188	33.469	28.422	1.00	93.88	C
ATOM	5903	CZ2	TRP	A	745	-37.312	32.750	28.750	1.00	93.88	C
ATOM	5904	CZ3	TRP	A	745	-35.688	34.469	29.250	1.00	93.88	C
ATOM	5905	N	GLY	A	746	-38.844	33.312	35.594	1.00	89.62	N
ATOM	5906	CA	GLY	A	746	-39.625	32.156	36.062	1.00	89.62	C
ATOM	5907	C	GLY	A	746	-40.969	32.062	35.406	1.00	89.62	C
ATOM	5908	O	GLY	A	746	-41.469	33.031	34.781	1.00	89.62	O
ATOM	5909	N	PHE	A	747	-41.500	30.922	35.406	1.00	88.19	N
ATOM	5910	CA	PHE	A	747	-42.844	30.688	34.875	1.00	88.19	C
ATOM	5911	C	PHE	A	747	-43.875	30.594	35.969	1.00	88.19	C
ATOM	5912	CB	PHE	A	747	-42.875	29.406	34.031	1.00	88.19	C
ATOM	5913	O	PHE	A	747	-43.594	30.016	37.031	1.00	88.19	O
ATOM	5914	CG	PHE	A	747	-42.000	29.484	32.781	1.00	88.19	C
ATOM	5915	CD1	PHE	A	747	-42.438	30.203	31.672	1.00	88.19	C
ATOM	5916	CD2	PHE	A	747	-40.781	28.828	32.750	1.00	88.19	C
ATOM	5917	CE1	PHE	A	747	-41.625	30.266	30.531	1.00	88.19	C
ATOM	5918	CE2	PHE	A	747	-39.969	28.891	31.609	1.00	88.19	C
ATOM	5919	CZ	PHE	A	747	-40.406	29.609	30.500	1.00	88.19	C
ATOM	5920	N	SER	A	748	-44.969	31.375	35.938	1.00	81.25	N
ATOM	5921	CA	SER	A	748	-46.062	31.281	36.875	1.00	81.25	C
ATOM	5922	C	SER	A	748	-46.594	29.844	36.969	1.00	81.25	C
ATOM	5923	CB	SER	A	748	-47.188	32.219	36.500	1.00	81.25	C
ATOM	5924	O	SER	A	748	-46.375	29.047	36.031	1.00	81.25	O
ATOM	5925	OG	SER	A	748	-47.531	32.094	35.125	1.00	81.25	O
ATOM	5926	N	ASP	A	749	-47.031	29.516	38.094	1.00	70.88	N
ATOM	5927	CA	ASP	A	749	-47.688	28.234	38.344	1.00	70.88	C
ATOM	5928	C	ASP	A	749	-48.781	28.000	37.312	1.00	70.88	C
ATOM	5929	CB	ASP	A	749	-48.250	28.172	39.750	1.00	70.88	C
ATOM	5930	O	ASP	A	749	-49.562	28.891	37.000	1.00	70.88	O
ATOM	5931	CG	ASP	A	749	-47.250	27.703	40.781	1.00	70.88	C
ATOM	5932	OD1	ASP	A	749	-46.125	27.344	40.406	1.00	70.88	O
ATOM	5933	OD2	ASP	A	749	-47.625	27.672	41.969	1.00	70.88	O
ATOM	5934	N	GLY	A	750	-48.656	26.938	36.469	1.00	67.19	N
ATOM	5935	CA	GLY	A	750	-49.594	26.547	35.438	1.00	67.19	C
ATOM	5936	C	GLY	A	750	-49.188	26.969	34.062	1.00	67.19	C
ATOM	5937	O	GLY	A	750	-50.000	26.906	33.125	1.00	67.19	O
ATOM	5938	N	SER	A	751	-47.938	27.641	33.875	1.00	71.94	N
ATOM	5939	CA	SER	A	751	-47.469	28.141	32.594	1.00	71.94	C
ATOM	5940	C	SER	A	751	-46.719	27.047	31.812	1.00	71.94	C
ATOM	5941	CB	SER	A	751	-46.594	29.359	32.750	1.00	71.94	C
ATOM	5942	O	SER	A	751	-46.000	27.344	30.875	1.00	71.94	O
ATOM	5943	OG	SER	A	751	-46.250	29.547	34.125	1.00	71.94	O
ATOM	5944	N	GLY	A	752	-46.781	25.828	32.219	1.00	70.69	N
ATOM	5945	CA	GLY	A	752	-46.125	24.688	31.594	1.00	70.69	C
ATOM	5946	C	GLY	A	752	-46.500	24.531	30.125	1.00	70.69	C
ATOM	5947	O	GLY	A	752	-45.781	23.891	29.359	1.00	70.69	O
ATOM	5948	N	ASP	A	753	-47.625	25.250	29.703	1.00	70.31	N

ATOM	5949	CA	ASP	A	753	-48.062	25.141	28.312	1.00	70.31	C
ATOM	5950	C	ASP	A	753	-47.375	26.172	27.438	1.00	70.31	C
ATOM	5951	CB	ASP	A	753	-49.594	25.297	28.219	1.00	70.31	C
ATOM	5952	O	ASP	A	753	-47.719	26.328	26.266	1.00	70.31	O
ATOM	5953	CG	ASP	A	753	-50.094	26.609	28.812	1.00	70.31	C
ATOM	5954	OD1	ASP	A	753	-49.375	27.219	29.641	1.00	70.31	O
ATOM	5955	OD2	ASP	A	753	-51.188	27.047	28.438	1.00	70.31	O
ATOM	5956	N	THR	A	754	-46.406	26.969	27.969	1.00	82.06	N
ATOM	5957	CA	THR	A	754	-45.688	28.000	27.219	1.00	82.06	C
ATOM	5958	C	THR	A	754	-44.844	27.375	26.094	1.00	82.06	C
ATOM	5959	CB	THR	A	754	-44.750	28.812	28.141	1.00	82.06	C
ATOM	5960	O	THR	A	754	-44.156	26.375	26.328	1.00	82.06	O
ATOM	5961	CG2	THR	A	754	-44.000	29.906	27.359	1.00	82.06	C
ATOM	5962	OG1	THR	A	754	-45.531	29.438	29.156	1.00	82.06	O
ATOM	5963	N	ALA	A	755	-45.156	27.641	24.734	1.00	84.00	N
ATOM	5964	CA	ALA	A	755	-44.344	27.203	23.609	1.00	84.00	C
ATOM	5965	C	ALA	A	755	-43.000	27.891	23.625	1.00	84.00	C
ATOM	5966	CB	ALA	A	755	-45.062	27.469	22.281	1.00	84.00	C
ATOM	5967	O	ALA	A	755	-41.938	27.234	23.594	1.00	84.00	O
ATOM	5968	N	TYR	A	756	-43.000	29.125	23.734	1.00	88.44	N
ATOM	5969	CA	TYR	A	756	-41.781	29.906	23.812	1.00	88.44	C
ATOM	5970	C	TYR	A	756	-42.000	31.234	24.531	1.00	88.44	C
ATOM	5971	CB	TYR	A	756	-41.219	30.172	22.406	1.00	88.44	C
ATOM	5972	O	TYR	A	756	-43.156	31.625	24.719	1.00	88.44	O
ATOM	5973	CG	TYR	A	756	-42.188	30.797	21.469	1.00	88.44	C
ATOM	5974	CD1	TYR	A	756	-42.938	30.000	20.594	1.00	88.44	C
ATOM	5975	CD2	TYR	A	756	-42.406	32.156	21.438	1.00	88.44	C
ATOM	5976	CE1	TYR	A	756	-43.875	30.578	19.719	1.00	88.44	C
ATOM	5977	CE2	TYR	A	756	-43.312	32.750	20.562	1.00	88.44	C
ATOM	5978	OH	TYR	A	756	-44.938	32.531	18.844	1.00	88.44	O
ATOM	5979	CZ	TYR	A	756	-44.031	31.953	19.703	1.00	88.44	C
ATOM	5980	N	THR	A	757	-41.156	31.781	25.141	1.00	93.06	N
ATOM	5981	CA	THR	A	757	-41.125	33.125	25.719	1.00	93.06	C
ATOM	5982	C	THR	A	757	-40.594	34.156	24.734	1.00	93.06	C
ATOM	5983	CB	THR	A	757	-40.312	33.156	27.016	1.00	93.06	C
ATOM	5984	O	THR	A	757	-39.531	33.906	24.125	1.00	93.06	O
ATOM	5985	CG2	THR	A	757	-40.375	34.562	27.672	1.00	93.06	C
ATOM	5986	OG1	THR	A	757	-40.844	32.188	27.938	1.00	93.06	O
ATOM	5987	N	GLU	A	758	-41.406	35.062	24.375	1.00	94.50	N
ATOM	5988	CA	GLU	A	758	-40.969	36.188	23.547	1.00	94.50	C
ATOM	5989	C	GLU	A	758	-40.188	37.188	24.375	1.00	94.50	C
ATOM	5990	CB	GLU	A	758	-42.188	36.844	22.891	1.00	94.50	C
ATOM	5991	O	GLU	A	758	-40.719	37.781	25.312	1.00	94.50	O
ATOM	5992	CG	GLU	A	758	-41.812	37.938	21.891	1.00	94.50	C
ATOM	5993	CD	GLU	A	758	-43.000	38.531	21.172	1.00	94.50	C
ATOM	5994	OE1	GLU	A	758	-42.875	39.625	20.594	1.00	94.50	O
ATOM	5995	OE2	GLU	A	758	-44.062	37.875	21.172	1.00	94.50	O
ATOM	5996	N	LEU	A	759	-38.875	37.344	24.078	1.00	95.44	N
ATOM	5997	CA	LEU	A	759	-37.969	38.281	24.750	1.00	95.44	C
ATOM	5998	C	LEU	A	759	-37.719	39.531	23.891	1.00	95.44	C
ATOM	5999	CB	LEU	A	759	-36.625	37.594	25.047	1.00	95.44	C
ATOM	6000	O	LEU	A	759	-37.312	39.406	22.734	1.00	95.44	O
ATOM	6001	CG	LEU	A	759	-35.562	38.469	25.703	1.00	95.44	C
ATOM	6002	CD1	LEU	A	759	-36.031	38.938	27.078	1.00	95.44	C
ATOM	6003	CD2	LEU	A	759	-34.250	37.688	25.828	1.00	95.44	C
ATOM	6004	N	GLN	A	760	-38.000	40.594	24.438	1.00	95.75	N
ATOM	6005	CA	GLN	A	760	-37.781	41.875	23.750	1.00	95.75	C
ATOM	6006	C	GLN	A	760	-36.656	42.656	24.453	1.00	95.75	C
ATOM	6007	CB	GLN	A	760	-39.062	42.719	23.688	1.00	95.75	C
ATOM	6008	O	GLN	A	760	-36.438	42.500	25.641	1.00	95.75	O
ATOM	6009	CG	GLN	A	760	-40.031	42.219	22.625	1.00	95.75	C
ATOM	6010	CD	GLN	A	760	-41.188	43.188	22.406	1.00	95.75	C
ATOM	6011	NE2	GLN	A	760	-41.938	43.000	21.344	1.00	95.75	N
ATOM	6012	OE1	GLN	A	760	-41.375	44.125	23.203	1.00	95.75	O

ATOM	6013	N	GLN	A	761	-36.000	43.375	23.594	1.00	94.31	N
ATOM	6014	CA	GLN	A	761	-35.000	44.312	24.109	1.00	94.31	C
ATOM	6015	C	GLN	A	761	-35.375	45.750	23.797	1.00	94.31	C
ATOM	6016	CB	GLN	A	761	-33.594	44.000	23.547	1.00	94.31	C
ATOM	6017	O	GLN	A	761	-36.094	46.031	22.828	1.00	94.31	O
ATOM	6018	CG	GLN	A	761	-33.469	44.250	22.047	1.00	94.31	C
ATOM	6019	CD	GLN	A	761	-32.062	44.031	21.531	1.00	94.31	C
ATOM	6020	NE2	GLN	A	761	-31.938	43.969	20.219	1.00	94.31	N
ATOM	6021	OE1	GLN	A	761	-31.125	43.938	22.312	1.00	94.31	O
ATOM	6022	N	SER	A	762	-34.969	46.594	24.703	1.00	93.62	N
ATOM	6023	CA	SER	A	762	-35.156	48.031	24.547	1.00	93.62	C
ATOM	6024	C	SER	A	762	-33.969	48.844	25.062	1.00	93.62	C
ATOM	6025	CB	SER	A	762	-36.438	48.500	25.281	1.00	93.62	C
ATOM	6026	O	SER	A	762	-33.500	48.594	26.188	1.00	93.62	O
ATOM	6027	OG	SER	A	762	-36.594	49.906	25.188	1.00	93.62	O
ATOM	6028	N	PRO	A	763	-33.469	49.781	24.219	1.00	88.00	N
ATOM	6029	CA	PRO	A	763	-32.312	50.562	24.656	1.00	88.00	C
ATOM	6030	C	PRO	A	763	-32.656	51.500	25.828	1.00	88.00	C
ATOM	6031	CB	PRO	A	763	-31.953	51.375	23.422	1.00	88.00	C
ATOM	6032	O	PRO	A	763	-31.797	51.844	26.641	1.00	88.00	O
ATOM	6033	CG	PRO	A	763	-33.062	51.125	22.453	1.00	88.00	C
ATOM	6034	CD	PRO	A	763	-33.938	50.031	23.000	1.00	88.00	C
ATOM	6035	N	ASP	A	764	-34.000	51.969	26.031	1.00	88.19	N
ATOM	6036	CA	ASP	A	764	-34.344	53.000	27.031	1.00	88.19	C
ATOM	6037	C	ASP	A	764	-35.469	52.500	27.938	1.00	88.19	C
ATOM	6038	CB	ASP	A	764	-34.750	54.281	26.328	1.00	88.19	C
ATOM	6039	O	ASP	A	764	-36.000	53.281	28.734	1.00	88.19	O
ATOM	6040	CG	ASP	A	764	-35.875	54.125	25.344	1.00	88.19	C
ATOM	6041	OD1	ASP	A	764	-36.375	52.969	25.203	1.00	88.19	O
ATOM	6042	OD2	ASP	A	764	-36.281	55.094	24.703	1.00	88.19	O
ATOM	6043	N	GLY	A	765	-35.875	51.156	27.844	1.00	88.81	N
ATOM	6044	CA	GLY	A	765	-36.906	50.562	28.703	1.00	88.81	C
ATOM	6045	C	GLY	A	765	-38.312	51.000	28.359	1.00	88.81	C
ATOM	6046	O	GLY	A	765	-39.219	50.938	29.203	1.00	88.81	O
ATOM	6047	N	THR	A	766	-38.500	51.656	27.078	1.00	90.69	N
ATOM	6048	CA	THR	A	766	-39.844	52.094	26.641	1.00	90.69	C
ATOM	6049	C	THR	A	766	-40.375	51.156	25.578	1.00	90.69	C
ATOM	6050	CB	THR	A	766	-39.812	53.531	26.078	1.00	90.69	C
ATOM	6051	O	THR	A	766	-39.625	50.625	24.766	1.00	90.69	O
ATOM	6052	CG2	THR	A	766	-39.312	54.531	27.141	1.00	90.69	C
ATOM	6053	OG1	THR	A	766	-38.906	53.594	24.969	1.00	90.69	O
ATOM	6054	N	VAL	A	767	-41.656	50.906	25.578	1.00	88.88	N
ATOM	6055	CA	VAL	A	767	-42.344	49.938	24.703	1.00	88.88	C
ATOM	6056	C	VAL	A	767	-42.156	50.375	23.250	1.00	88.88	C
ATOM	6057	CB	VAL	A	767	-43.844	49.844	25.031	1.00	88.88	C
ATOM	6058	O	VAL	A	767	-42.000	49.500	22.359	1.00	88.88	O
ATOM	6059	CG1	VAL	A	767	-44.562	48.938	24.031	1.00	88.88	C
ATOM	6060	CG2	VAL	A	767	-44.031	49.312	26.469	1.00	88.88	C
ATOM	6061	N	ASP	A	768	-42.062	51.750	22.891	1.00	88.12	N
ATOM	6062	CA	ASP	A	768	-42.000	52.250	21.547	1.00	88.12	C
ATOM	6063	C	ASP	A	768	-40.688	51.875	20.875	1.00	88.12	C
ATOM	6064	CB	ASP	A	768	-42.156	53.781	21.547	1.00	88.12	C
ATOM	6065	O	ASP	A	768	-40.625	51.688	19.656	1.00	88.12	O
ATOM	6066	CG	ASP	A	768	-43.562	54.219	21.906	1.00	88.12	C
ATOM	6067	OD1	ASP	A	768	-44.531	53.406	21.766	1.00	88.12	O
ATOM	6068	OD2	ASP	A	768	-43.750	55.375	22.328	1.00	88.12	O
ATOM	6069	N	ASN	A	769	-39.656	51.625	21.734	1.00	88.44	N
ATOM	6070	CA	ASN	A	769	-38.344	51.312	21.172	1.00	88.44	C
ATOM	6071	C	ASN	A	769	-37.969	49.844	21.406	1.00	88.44	C
ATOM	6072	CB	ASN	A	769	-37.281	52.250	21.781	1.00	88.44	C
ATOM	6073	O	ASN	A	769	-36.812	49.469	21.141	1.00	88.44	O
ATOM	6074	CG	ASN	A	769	-37.469	53.688	21.359	1.00	88.44	C
ATOM	6075	ND2	ASN	A	769	-37.031	54.625	22.203	1.00	88.44	N
ATOM	6076	OD1	ASN	A	769	-38.000	53.969	20.281	1.00	88.44	O

```
ATOM   6077  N   ALA A 770     -39.000  49.094  21.906  1.00 92.62           N
ATOM   6078  CA  ALA A 770     -38.750  47.656  22.156  1.00 92.62           C
ATOM   6079  C   ALA A 770     -38.812  46.875  20.859  1.00 92.62           C
ATOM   6080  CB  ALA A 770     -39.781  47.125  23.156  1.00 92.62           C
ATOM   6081  O   ALA A 770     -39.625  47.156  19.984  1.00 92.62           O
ATOM   6082  N   SER A 771     -37.812  46.062  20.625  1.00 92.94           N
ATOM   6083  CA  SER A 771     -37.781  45.188  19.469  1.00 92.94           C
ATOM   6084  C   SER A 771     -37.531  43.750  19.891  1.00 92.94           C
ATOM   6085  CB  SER A 771     -36.656  45.625  18.500  1.00 92.94           C
ATOM   6086  O   SER A 771     -37.000  43.469  20.969  1.00 92.94           O
ATOM   6087  OG  SER A 771     -35.406  45.562  19.125  1.00 92.94           O
ATOM   6088  N   LEU A 772     -38.031  42.844  19.078  1.00 94.94           N
ATOM   6089  CA  LEU A 772     -37.875  41.406  19.359  1.00 94.94           C
ATOM   6090  C   LEU A 772     -36.406  41.031  19.344  1.00 94.94           C
ATOM   6091  CB  LEU A 772     -38.625  40.594  18.328  1.00 94.94           C
ATOM   6092  O   LEU A 772     -35.688  41.281  18.375  1.00 94.94           O
ATOM   6093  CG  LEU A 772     -38.500  39.062  18.453  1.00 94.94           C
ATOM   6094  CD1 LEU A 772     -39.188  38.594  19.734  1.00 94.94           C
ATOM   6095  CD2 LEU A 772     -39.094  38.375  17.234  1.00 94.94           C
ATOM   6096  N   LEU A 773     -35.906  40.531  20.547  1.00 92.38           N
ATOM   6097  CA  LEU A 773     -34.531  40.000  20.625  1.00 92.38           C
ATOM   6098  C   LEU A 773     -34.500  38.562  20.172  1.00 92.38           C
ATOM   6099  CB  LEU A 773     -34.000  40.125  22.047  1.00 92.38           C
ATOM   6100  O   LEU A 773     -33.719  38.188  19.281  1.00 92.38           O
ATOM   6101  CG  LEU A 773     -32.562  39.688  22.266  1.00 92.38           C
ATOM   6102  CD1 LEU A 773     -31.594  40.469  21.391  1.00 92.38           C
ATOM   6103  CD2 LEU A 773     -32.156  39.812  23.734  1.00 92.38           C
ATOM   6104  N   SER A 774     -35.344  37.719  20.828  1.00 93.88           N
ATOM   6105  CA  SER A 774     -35.406  36.281  20.500  1.00 93.88           C
ATOM   6106  C   SER A 774     -36.688  35.625  21.016  1.00 93.88           C
ATOM   6107  CB  SER A 774     -34.188  35.562  21.109  1.00 93.88           C
ATOM   6108  O   SER A 774     -37.281  36.125  21.953  1.00 93.88           O
ATOM   6109  OG  SER A 774     -34.094  34.250  20.625  1.00 93.88           O
ATOM   6110  N   LEU A 775     -37.062  34.594  20.328  1.00 93.00           N
ATOM   6111  CA  LEU A 775     -38.125  33.719  20.812  1.00 93.00           C
ATOM   6112  C   LEU A 775     -37.500  32.469  21.484  1.00 93.00           C
ATOM   6113  CB  LEU A 775     -39.031  33.281  19.672  1.00 93.00           C
ATOM   6114  O   LEU A 775     -36.844  31.672  20.812  1.00 93.00           O
ATOM   6115  CG  LEU A 775     -39.781  34.406  18.938  1.00 93.00           C
ATOM   6116  CD1 LEU A 775     -40.562  33.844  17.766  1.00 93.00           C
ATOM   6117  CD2 LEU A 775     -40.688  35.156  19.891  1.00 93.00           C
ATOM   6118  N   ILE A 776     -37.688  32.312  22.828  1.00 92.06           N
ATOM   6119  CA  ILE A 776     -37.000  31.297  23.625  1.00 92.06           C
ATOM   6120  C   ILE A 776     -37.969  30.188  23.969  1.00 92.06           C
ATOM   6121  CB  ILE A 776     -36.375  31.906  24.906  1.00 92.06           C
ATOM   6122  O   ILE A 776     -38.969  30.422  24.641  1.00 92.06           O
ATOM   6123  CG1 ILE A 776     -35.469  33.094  24.547  1.00 92.06           C
ATOM   6124  CG2 ILE A 776     -35.625  30.828  25.688  1.00 92.06           C
ATOM   6125  CD1 ILE A 776     -35.625  34.281  25.453  1.00 92.06           C
ATOM   6126  N   PRO A 777     -37.688  28.953  23.406  1.00 89.19           N
ATOM   6127  CA  PRO A 777     -38.594  27.828  23.594  1.00 89.19           C
ATOM   6128  C   PRO A 777     -38.719  27.406  25.047  1.00 89.19           C
ATOM   6129  CB  PRO A 777     -37.969  26.719  22.734  1.00 89.19           C
ATOM   6130  O   PRO A 777     -37.719  27.438  25.781  1.00 89.19           O
ATOM   6131  CG  PRO A 777     -36.562  27.156  22.500  1.00 89.19           C
ATOM   6132  CD  PRO A 777     -36.500  28.641  22.719  1.00 89.19           C
ATOM   6133  N   TYR A 778     -39.812  27.359  25.734  1.00 83.12           N
ATOM   6134  CA  TYR A 778     -40.062  26.766  27.031  1.00 83.12           C
ATOM   6135  C   TYR A 778     -39.344  25.438  27.188  1.00 83.12           C
ATOM   6136  CB  TYR A 778     -41.562  26.562  27.250  1.00 83.12           C
ATOM   6137  O   TYR A 778     -39.281  24.656  26.250  1.00 83.12           O
ATOM   6138  CG  TYR A 778     -41.906  25.953  28.594  1.00 83.12           C
ATOM   6139  CD1 TYR A 778     -42.031  24.578  28.734  1.00 83.12           C
ATOM   6140  CD2 TYR A 778     -42.125  26.750  29.719  1.00 83.12           C
```

ATOM	6141	CE1	TYR	A	778	-42.344	24.000	29.969	1.00	83.12	C
ATOM	6142	CE2	TYR	A	778	-42.438	26.188	30.953	1.00	83.12	C
ATOM	6143	OH	TYR	A	778	-42.844	24.250	32.281	1.00	83.12	O
ATOM	6144	CZ	TYR	A	778	-42.531	24.812	31.062	1.00	83.12	C
ATOM	6145	N	PRO	A	779	-38.875	24.859	28.250	1.00	82.62	N
ATOM	6146	CA	PRO	A	779	-38.969	25.578	29.531	1.00	82.62	C
ATOM	6147	C	PRO	A	779	-37.750	26.469	29.781	1.00	82.62	C
ATOM	6148	CB	PRO	A	779	-39.031	24.453	30.562	1.00	82.62	C
ATOM	6149	O	PRO	A	779	-37.469	26.828	30.938	1.00	82.62	O
ATOM	6150	CG	PRO	A	779	-38.219	23.328	29.969	1.00	82.62	C
ATOM	6151	CD	PRO	A	779	-38.375	23.391	28.484	1.00	82.62	C
ATOM	6152	N	GLN	A	780	-37.031	26.734	28.500	1.00	88.88	N
ATOM	6153	CA	GLN	A	780	-35.875	27.594	28.672	1.00	88.88	C
ATOM	6154	C	GLN	A	780	-36.281	28.922	29.312	1.00	88.88	C
ATOM	6155	CB	GLN	A	780	-35.188	27.844	27.328	1.00	88.88	C
ATOM	6156	O	GLN	A	780	-37.281	29.516	28.938	1.00	88.88	O
ATOM	6157	CG	GLN	A	780	-34.438	26.625	26.797	1.00	88.88	C
ATOM	6158	CD	GLN	A	780	-33.656	26.938	25.516	1.00	88.88	C
ATOM	6159	NE2	GLN	A	780	-33.062	25.906	24.938	1.00	88.88	N
ATOM	6160	OE1	GLN	A	780	-33.625	28.078	25.062	1.00	88.88	O
ATOM	6161	N	HIS	A	781	-35.750	29.219	30.375	1.00	89.25	N
ATOM	6162	CA	HIS	A	781	-36.156	30.391	31.141	1.00	89.25	C
ATOM	6163	C	HIS	A	781	-35.000	31.359	31.312	1.00	89.25	C
ATOM	6164	CB	HIS	A	781	-36.719	29.984	32.500	1.00	89.25	C
ATOM	6165	O	HIS	A	781	-35.000	32.156	32.250	1.00	89.25	O
ATOM	6166	CG	HIS	A	781	-35.750	29.203	33.344	1.00	89.25	C
ATOM	6167	CD2	HIS	A	781	-34.594	29.578	33.938	1.00	89.25	C
ATOM	6168	ND1	HIS	A	781	-35.906	27.875	33.625	1.00	89.25	N
ATOM	6169	CE1	HIS	A	781	-34.906	27.453	34.375	1.00	89.25	C
ATOM	6170	NE2	HIS	A	781	-34.094	28.484	34.594	1.00	89.25	N
ATOM	6171	N	GLU	A	782	-34.000	31.172	30.469	1.00	92.25	N
ATOM	6172	CA	GLU	A	782	-32.875	32.094	30.500	1.00	92.25	C
ATOM	6173	C	GLU	A	782	-32.344	32.344	29.094	1.00	92.25	C
ATOM	6174	CB	GLU	A	782	-31.781	31.562	31.406	1.00	92.25	C
ATOM	6175	O	GLU	A	782	-32.500	31.516	28.203	1.00	92.25	O
ATOM	6176	CG	GLU	A	782	-31.094	30.312	30.875	1.00	92.25	C
ATOM	6177	CD	GLU	A	782	-29.953	29.828	31.750	1.00	92.25	C
ATOM	6178	OE1	GLU	A	782	-29.359	28.766	31.453	1.00	92.25	O
ATOM	6179	OE2	GLU	A	782	-29.656	30.516	32.750	1.00	92.25	O
ATOM	6180	N	TYR	A	783	-31.844	33.469	28.750	1.00	93.44	N
ATOM	6181	CA	TYR	A	783	-31.250	33.844	27.484	1.00	93.44	C
ATOM	6182	C	TYR	A	783	-29.984	34.656	27.703	1.00	93.44	C
ATOM	6183	CB	TYR	A	783	-32.250	34.656	26.641	1.00	93.44	C
ATOM	6184	O	TYR	A	783	-29.953	35.594	28.516	1.00	93.44	O
ATOM	6185	CG	TYR	A	783	-31.719	35.031	25.281	1.00	93.44	C
ATOM	6186	CD1	TYR	A	783	-31.188	36.312	25.031	1.00	93.44	C
ATOM	6187	CD2	TYR	A	783	-31.734	34.125	24.234	1.00	93.44	C
ATOM	6188	CE1	TYR	A	783	-30.703	36.656	23.766	1.00	93.44	C
ATOM	6189	CE2	TYR	A	783	-31.250	34.438	22.969	1.00	93.44	C
ATOM	6190	OH	TYR	A	783	-30.266	36.062	21.500	1.00	93.44	O
ATOM	6191	CZ	TYR	A	783	-30.750	35.719	22.750	1.00	93.44	C
ATOM	6192	N	TYR	A	784	-28.859	34.219	26.953	1.00	92.00	N
ATOM	6193	CA	TYR	A	784	-27.578	34.906	27.031	1.00	92.00	C
ATOM	6194	C	TYR	A	784	-27.406	35.875	25.859	1.00	92.00	C
ATOM	6195	CB	TYR	A	784	-26.422	33.906	27.062	1.00	92.00	C
ATOM	6196	O	TYR	A	784	-27.219	35.438	24.719	1.00	92.00	O
ATOM	6197	CG	TYR	A	784	-26.125	33.375	28.438	1.00	92.00	C
ATOM	6198	CD1	TYR	A	784	-25.219	34.031	29.281	1.00	92.00	C
ATOM	6199	CD2	TYR	A	784	-26.734	32.219	28.891	1.00	92.00	C
ATOM	6200	CE1	TYR	A	784	-24.938	33.531	30.547	1.00	92.00	C
ATOM	6201	CE2	TYR	A	784	-26.453	31.719	30.172	1.00	92.00	C
ATOM	6202	OH	TYR	A	784	-25.281	31.891	32.250	1.00	92.00	O
ATOM	6203	CZ	TYR	A	784	-25.562	32.375	30.984	1.00	92.00	C
ATOM	6204	N	HIS	A	785	-27.531	37.094	25.984	1.00	92.69	N

ATOM	6205	CA	HIS	A	785	-27.406	38.156	24.984	1.00	92.69	C
ATOM	6206	C	HIS	A	785	-25.984	38.688	24.906	1.00	92.69	C
ATOM	6207	CB	HIS	A	785	-28.391	39.281	25.281	1.00	92.69	C
ATOM	6208	O	HIS	A	785	-25.547	39.406	25.797	1.00	92.69	O
ATOM	6209	CG	HIS	A	785	-28.547	40.250	24.141	1.00	92.69	C
ATOM	6210	CD2	HIS	A	785	-27.969	40.281	22.922	1.00	92.69	C
ATOM	6211	ND1	HIS	A	785	-29.375	41.344	24.219	1.00	92.69	N
ATOM	6212	CE1	HIS	A	785	-29.312	42.031	23.078	1.00	92.69	C
ATOM	6213	NE2	HIS	A	785	-28.469	41.375	22.266	1.00	92.69	N
ATOM	6214	N	SER	A	786	-25.109	38.125	23.953	1.00	88.25	N
ATOM	6215	CA	SER	A	786	-23.719	38.438	23.641	1.00	88.25	C
ATOM	6216	C	SER	A	786	-23.438	38.312	22.141	1.00	88.25	C
ATOM	6217	CB	SER	A	786	-22.781	37.531	24.438	1.00	88.25	C
ATOM	6218	O	SER	A	786	-24.141	37.594	21.438	1.00	88.25	O
ATOM	6219	OG	SER	A	786	-21.438	37.750	24.047	1.00	88.25	O
ATOM	6220	N	PRO	A	787	-22.219	39.094	21.578	1.00	83.38	N
ATOM	6221	CA	PRO	A	787	-21.281	39.906	22.344	1.00	83.38	C
ATOM	6222	C	PRO	A	787	-21.719	41.344	22.484	1.00	83.38	C
ATOM	6223	CB	PRO	A	787	-19.984	39.781	21.531	1.00	83.38	C
ATOM	6224	O	PRO	A	787	-22.453	41.844	21.625	1.00	83.38	O
ATOM	6225	CG	PRO	A	787	-20.438	39.562	20.125	1.00	83.38	C
ATOM	6226	CD	PRO	A	787	-21.766	38.875	20.156	1.00	83.38	C
ATOM	6227	N	MET	A	788	-21.688	41.938	23.703	1.00	85.81	N
ATOM	6228	CA	MET	A	788	-21.953	43.375	23.891	1.00	85.81	C
ATOM	6229	C	MET	A	788	-20.828	44.000	24.703	1.00	85.81	C
ATOM	6230	CB	MET	A	788	-23.297	43.594	24.578	1.00	85.81	C
ATOM	6231	O	MET	A	788	-20.125	43.344	25.453	1.00	85.81	O
ATOM	6232	CG	MET	A	788	-23.375	42.938	25.969	1.00	85.81	C
ATOM	6233	SD	MET	A	788	-25.078	42.969	26.641	1.00	85.81	S
ATOM	6234	CE	MET	A	788	-25.859	41.719	25.594	1.00	85.81	C
ATOM	6235	N	PRO	A	789	-20.578	45.312	24.359	1.00	82.12	N
ATOM	6236	CA	PRO	A	789	-19.516	46.031	25.109	1.00	82.12	C
ATOM	6237	C	PRO	A	789	-19.812	46.094	26.609	1.00	82.12	C
ATOM	6238	CB	PRO	A	789	-19.500	47.406	24.469	1.00	82.12	C
ATOM	6239	O	PRO	A	789	-20.969	46.062	27.016	1.00	82.12	O
ATOM	6240	CG	PRO	A	789	-20.328	47.281	23.234	1.00	82.12	C
ATOM	6241	CD	PRO	A	789	-21.188	46.062	23.359	1.00	82.12	C
ATOM	6242	N	GLY	A	790	-18.844	46.031	27.438	1.00	83.00	N
ATOM	6243	CA	GLY	A	790	-19.000	46.219	28.859	1.00	83.00	C
ATOM	6244	C	GLY	A	790	-19.750	47.469	29.234	1.00	83.00	C
ATOM	6245	O	GLY	A	790	-19.594	48.500	28.578	1.00	83.00	O
ATOM	6246	N	GLY	A	791	-20.719	47.438	30.078	1.00	84.06	N
ATOM	6247	CA	GLY	A	791	-21.516	48.562	30.594	1.00	84.06	C
ATOM	6248	C	GLY	A	791	-22.766	48.812	29.781	1.00	84.06	C
ATOM	6249	O	GLY	A	791	-23.547	49.719	30.109	1.00	84.06	O
ATOM	6250	N	ASN	A	792	-22.828	48.125	28.625	1.00	87.50	N
ATOM	6251	CA	ASN	A	792	-24.016	48.312	27.797	1.00	87.50	C
ATOM	6252	C	ASN	A	792	-25.297	47.906	28.547	1.00	87.50	C
ATOM	6253	CB	ASN	A	792	-23.906	47.500	26.500	1.00	87.50	C
ATOM	6254	O	ASN	A	792	-25.375	46.844	29.125	1.00	87.50	O
ATOM	6255	CG	ASN	A	792	-24.828	48.000	25.406	1.00	87.50	C
ATOM	6256	ND2	ASN	A	792	-24.266	48.469	24.312	1.00	87.50	N
ATOM	6257	OD1	ASN	A	792	-26.062	47.969	25.562	1.00	87.50	O
ATOM	6258	N	ILE	A	793	-26.188	48.906	28.672	1.00	89.88	N
ATOM	6259	CA	ILE	A	793	-27.438	48.719	29.375	1.00	89.88	C
ATOM	6260	C	ILE	A	793	-28.562	48.438	28.375	1.00	89.88	C
ATOM	6261	CB	ILE	A	793	-27.797	49.969	30.219	1.00	89.88	C
ATOM	6262	O	ILE	A	793	-28.719	49.156	27.391	1.00	89.88	O
ATOM	6263	CG1	ILE	A	793	-26.750	50.188	31.312	1.00	89.88	C
ATOM	6264	CG2	ILE	A	793	-29.203	49.812	30.844	1.00	89.88	C
ATOM	6265	CD1	ILE	A	793	-26.906	51.500	32.062	1.00	89.88	C
ATOM	6266	N	VAL	A	794	-29.203	47.406	28.531	1.00	93.81	N
ATOM	6267	CA	VAL	A	794	-30.359	47.000	27.750	1.00	93.81	C
ATOM	6268	C	VAL	A	794	-31.500	46.594	28.688	1.00	93.81	C

ATOM	6269	CB	VAL	A	794	-30.016	45.812	26.812	1.00	93.81	C
ATOM	6270	O	VAL	A	794	-31.266	46.000	29.750	1.00	93.81	O
ATOM	6271	CG1	VAL	A	794	-31.234	45.438	25.953	1.00	93.81	C
ATOM	6272	CG2	VAL	A	794	-28.828	46.188	25.922	1.00	93.81	C
ATOM	6273	N	TRP	A	795	-32.656	47.000	28.406	1.00	94.88	N
ATOM	6274	CA	TRP	A	795	-33.844	46.656	29.156	1.00	94.88	C
ATOM	6275	C	TRP	A	795	-34.594	45.500	28.469	1.00	94.88	C
ATOM	6276	CB	TRP	A	795	-34.781	47.844	29.328	1.00	94.88	C
ATOM	6277	O	TRP	A	795	-34.688	45.469	27.250	1.00	94.88	O
ATOM	6278	CG	TRP	A	795	-34.156	49.000	30.078	1.00	94.88	C
ATOM	6279	CD1	TRP	A	795	-33.250	49.906	29.594	1.00	94.88	C
ATOM	6280	CD2	TRP	A	795	-34.375	49.344	31.453	1.00	94.88	C
ATOM	6281	CE2	TRP	A	795	-33.562	50.469	31.734	1.00	94.88	C
ATOM	6282	CE3	TRP	A	795	-35.156	48.812	32.469	1.00	94.88	C
ATOM	6283	NE1	TRP	A	795	-32.906	50.812	30.578	1.00	94.88	N
ATOM	6284	CH2	TRP	A	795	-34.312	50.531	33.969	1.00	94.88	C
ATOM	6285	CZ2	TRP	A	795	-33.531	51.094	33.000	1.00	94.88	C
ATOM	6286	CZ3	TRP	A	795	-35.125	49.406	33.719	1.00	94.88	C
ATOM	6287	N	TYR	A	796	-35.031	44.531	29.266	1.00	96.00	N
ATOM	6288	CA	TYR	A	796	-35.688	43.344	28.719	1.00	96.00	C
ATOM	6289	C	TYR	A	796	-37.094	43.188	29.281	1.00	96.00	C
ATOM	6290	CB	TYR	A	796	-34.844	42.094	29.000	1.00	96.00	C
ATOM	6291	O	TYR	A	796	-37.344	43.562	30.422	1.00	96.00	O
ATOM	6292	CG	TYR	A	796	-33.469	42.125	28.375	1.00	96.00	C
ATOM	6293	CD1	TYR	A	796	-33.281	41.688	27.062	1.00	96.00	C
ATOM	6294	CD2	TYR	A	796	-32.375	42.594	29.094	1.00	96.00	C
ATOM	6295	CE1	TYR	A	796	-32.000	41.719	26.484	1.00	96.00	C
ATOM	6296	CE2	TYR	A	796	-31.109	42.594	28.516	1.00	96.00	C
ATOM	6297	OH	TYR	A	796	-29.688	42.188	26.641	1.00	96.00	O
ATOM	6298	CZ	TYR	A	796	-30.938	42.188	27.219	1.00	96.00	C
ATOM	6299	N	ARG	A	797	-37.938	42.750	28.547	1.00	95.44	N
ATOM	6300	CA	ARG	A	797	-39.250	42.250	28.984	1.00	95.44	C
ATOM	6301	C	ARG	A	797	-39.625	40.969	28.250	1.00	95.44	C
ATOM	6302	CB	ARG	A	797	-40.344	43.281	28.781	1.00	95.44	C
ATOM	6303	O	ARG	A	797	-39.125	40.688	27.156	1.00	95.44	O
ATOM	6304	CG	ARG	A	797	-40.625	43.594	27.312	1.00	95.44	C
ATOM	6305	CD	ARG	A	797	-41.719	44.625	27.156	1.00	95.44	C
ATOM	6306	NE	ARG	A	797	-42.062	44.844	25.766	1.00	95.44	N
ATOM	6307	NH1	ARG	A	797	-43.781	46.344	26.188	1.00	95.44	N
ATOM	6308	NH2	ARG	A	797	-43.250	45.812	24.031	1.00	95.44	N
ATOM	6309	CZ	ARG	A	797	-43.031	45.656	25.328	1.00	95.44	C
ATOM	6310	N	VAL	A	798	-40.344	40.188	28.891	1.00	95.25	N
ATOM	6311	CA	VAL	A	798	-40.656	38.906	28.281	1.00	95.25	C
ATOM	6312	C	VAL	A	798	-42.156	38.625	28.422	1.00	95.25	C
ATOM	6313	CB	VAL	A	798	-39.844	37.750	28.906	1.00	95.25	C
ATOM	6314	O	VAL	A	798	-42.812	39.219	29.297	1.00	95.25	O
ATOM	6315	CG1	VAL	A	798	-38.344	37.969	28.672	1.00	95.25	C
ATOM	6316	CG2	VAL	A	798	-40.156	37.656	30.406	1.00	95.25	C
ATOM	6317	N	ARG	A	799	-42.719	37.875	27.641	1.00	93.75	N
ATOM	6318	CA	ARG	A	799	-44.062	37.250	27.750	1.00	93.75	C
ATOM	6319	C	ARG	A	799	-44.062	35.844	27.188	1.00	93.75	C
ATOM	6320	CB	ARG	A	799	-45.094	38.125	27.016	1.00	93.75	C
ATOM	6321	O	ARG	A	799	-43.250	35.500	26.344	1.00	93.75	O
ATOM	6322	CG	ARG	A	799	-44.969	38.094	25.500	1.00	93.75	C
ATOM	6323	CD	ARG	A	799	-46.062	38.906	24.828	1.00	93.75	C
ATOM	6324	NE	ARG	A	799	-45.906	38.906	23.375	1.00	93.75	N
ATOM	6325	NH1	ARG	A	799	-47.656	40.375	22.984	1.00	93.75	N
ATOM	6326	NH2	ARG	A	799	-46.469	39.500	21.234	1.00	93.75	N
ATOM	6327	CZ	ARG	A	799	-46.688	39.594	22.531	1.00	93.75	C
ATOM	6328	N	THR	A	800	-44.875	35.094	27.672	1.00	90.25	N
ATOM	6329	CA	THR	A	800	-44.969	33.688	27.250	1.00	90.25	C
ATOM	6330	C	THR	A	800	-46.062	33.531	26.203	1.00	90.25	C
ATOM	6331	CB	THR	A	800	-45.281	32.781	28.453	1.00	90.25	C
ATOM	6332	O	THR	A	800	-47.094	34.219	26.234	1.00	90.25	O

ATOM	6333	CG2	THR	A	800	-44.125	32.750	29.438	1.00	90.25	C
ATOM	6334	OG1	THR	A	800	-46.469	33.250	29.125	1.00	90.25	O
ATOM	6335	N	VAL	A	801	-45.750	32.781	25.234	1.00	86.44	N
ATOM	6336	CA	VAL	A	801	-46.688	32.375	24.188	1.00	86.44	C
ATOM	6337	C	VAL	A	801	-46.906	30.875	24.250	1.00	86.44	C
ATOM	6338	CB	VAL	A	801	-46.156	32.781	22.781	1.00	86.44	C
ATOM	6339	O	VAL	A	801	-45.969	30.094	24.266	1.00	86.44	O
ATOM	6340	CG1	VAL	A	801	-47.188	32.438	21.703	1.00	86.44	C
ATOM	6341	CG2	VAL	A	801	-45.812	34.281	22.734	1.00	86.44	C
ATOM	6342	N	ASP	A	802	-48.062	30.453	24.531	1.00	78.12	N
ATOM	6343	CA	ASP	A	802	-48.344	29.031	24.703	1.00	78.12	C
ATOM	6344	C	ASP	A	802	-48.375	28.328	23.344	1.00	78.12	C
ATOM	6345	CB	ASP	A	802	-49.688	28.844	25.422	1.00	78.12	C
ATOM	6346	O	ASP	A	802	-48.250	28.953	22.297	1.00	78.12	O
ATOM	6347	CG	ASP	A	802	-50.875	29.281	24.594	1.00	78.12	C
ATOM	6348	OD1	ASP	A	802	-50.750	29.438	23.359	1.00	78.12	O
ATOM	6349	OD2	ASP	A	802	-51.969	29.484	25.172	1.00	78.12	O
ATOM	6350	N	ARG	A	803	-48.219	27.172	23.281	1.00	69.12	N
ATOM	6351	CA	ARG	A	803	-48.000	26.266	22.156	1.00	69.12	C
ATOM	6352	C	ARG	A	803	-49.125	26.391	21.125	1.00	69.12	C
ATOM	6353	CB	ARG	A	803	-47.875	24.828	22.656	1.00	69.12	C
ATOM	6354	O	ARG	A	803	-48.938	25.969	19.984	1.00	69.12	O
ATOM	6355	CG	ARG	A	803	-46.625	24.547	23.469	1.00	69.12	C
ATOM	6356	CD	ARG	A	803	-46.531	23.078	23.844	1.00	69.12	C
ATOM	6357	NE	ARG	A	803	-45.344	22.812	24.672	1.00	69.12	N
ATOM	6358	NH1	ARG	A	803	-45.875	20.609	25.094	1.00	69.12	N
ATOM	6359	NH2	ARG	A	803	-43.969	21.516	25.969	1.00	69.12	N
ATOM	6360	CZ	ARG	A	803	-45.062	21.641	25.250	1.00	69.12	C
ATOM	6361	N	ILE	A	804	-50.281	27.078	21.406	1.00	66.31	N
ATOM	6362	CA	ILE	A	804	-51.312	27.234	20.406	1.00	66.31	C
ATOM	6363	C	ILE	A	804	-51.438	28.719	20.016	1.00	66.31	C
ATOM	6364	CB	ILE	A	804	-52.688	26.719	20.938	1.00	66.31	C
ATOM	6365	O	ILE	A	804	-52.375	29.094	19.297	1.00	66.31	O
ATOM	6366	CG1	ILE	A	804	-53.031	27.359	22.281	1.00	66.31	C
ATOM	6367	CG2	ILE	A	804	-52.656	25.188	21.047	1.00	66.31	C
ATOM	6368	CD1	ILE	A	804	-54.438	27.094	22.750	1.00	66.31	C
ATOM	6369	N	GLY	A	805	-50.438	29.547	20.422	1.00	66.69	N
ATOM	6370	CA	GLY	A	805	-50.312	30.906	19.938	1.00	66.69	C
ATOM	6371	C	GLY	A	805	-50.906	31.938	20.875	1.00	66.69	C
ATOM	6372	O	GLY	A	805	-50.875	33.125	20.594	1.00	66.69	O
ATOM	6373	N	ASN	A	806	-51.562	31.562	22.047	1.00	71.12	N
ATOM	6374	CA	ASN	A	806	-52.062	32.531	23.031	1.00	71.12	C
ATOM	6375	C	ASN	A	806	-50.906	33.188	23.781	1.00	71.12	C
ATOM	6376	CB	ASN	A	806	-53.000	31.844	24.016	1.00	71.12	C
ATOM	6377	O	ASN	A	806	-49.938	32.531	24.125	1.00	71.12	O
ATOM	6378	CG	ASN	A	806	-54.219	31.219	23.344	1.00	71.12	C
ATOM	6379	ND2	ASN	A	806	-54.562	30.016	23.750	1.00	71.12	N
ATOM	6380	OD1	ASN	A	806	-54.812	31.844	22.469	1.00	71.12	O
ATOM	6381	N	VAL	A	807	-50.906	34.469	23.828	1.00	83.50	N
ATOM	6382	CA	VAL	A	807	-49.812	35.250	24.375	1.00	83.50	C
ATOM	6383	C	VAL	A	807	-50.188	35.781	25.766	1.00	83.50	C
ATOM	6384	CB	VAL	A	807	-49.438	36.438	23.453	1.00	83.50	C
ATOM	6385	O	VAL	A	807	-51.344	36.156	25.984	1.00	83.50	O
ATOM	6386	CG1	VAL	A	807	-48.844	35.906	22.125	1.00	83.50	C
ATOM	6387	CG2	VAL	A	807	-50.625	37.344	23.203	1.00	83.50	C
ATOM	6388	N	SER	A	808	-49.375	35.656	26.766	1.00	87.88	N
ATOM	6389	CA	SER	A	808	-49.562	36.219	28.078	1.00	87.88	C
ATOM	6390	C	SER	A	808	-49.406	37.750	28.047	1.00	87.88	C
ATOM	6391	CB	SER	A	808	-48.594	35.625	29.078	1.00	87.88	C
ATOM	6392	O	SER	A	808	-48.969	38.312	27.031	1.00	87.88	O
ATOM	6393	OG	SER	A	808	-47.281	36.125	28.875	1.00	87.88	O
ATOM	6394	N	GLN	A	809	-49.656	38.531	29.219	1.00	89.00	N
ATOM	6395	CA	GLN	A	809	-49.250	39.906	29.438	1.00	89.00	C
ATOM	6396	C	GLN	A	809	-47.750	40.031	29.516	1.00	89.00	C

ATOM	6397	CB	GLN	A	809	-49.906	40.469	30.719	1.00	89.00	C
ATOM	6398	O	GLN	A	809	-47.062	39.062	29.891	1.00	89.00	O
ATOM	6399	CG	GLN	A	809	-51.406	40.688	30.609	1.00	89.00	C
ATOM	6400	CD	GLN	A	809	-52.000	41.344	31.828	1.00	89.00	C
ATOM	6401	NE2	GLN	A	809	-53.281	41.656	31.781	1.00	89.00	N
ATOM	6402	OE1	GLN	A	809	-51.281	41.562	32.812	1.00	89.00	O
ATOM	6403	N	TRP	A	810	-47.188	41.219	29.078	1.00	92.62	N
ATOM	6404	CA	TRP	A	810	-45.750	41.469	29.188	1.00	92.62	C
ATOM	6405	C	TRP	A	810	-45.344	41.656	30.641	1.00	92.62	C
ATOM	6406	CB	TRP	A	810	-45.375	42.719	28.375	1.00	92.62	C
ATOM	6407	O	TRP	A	810	-46.094	42.156	31.453	1.00	92.62	O
ATOM	6408	CG	TRP	A	810	-45.375	42.500	26.891	1.00	92.62	C
ATOM	6409	CD1	TRP	A	810	-46.344	42.906	26.016	1.00	92.62	C
ATOM	6410	CD2	TRP	A	810	-44.375	41.844	26.109	1.00	92.62	C
ATOM	6411	CE2	TRP	A	810	-44.812	41.875	24.766	1.00	92.62	C
ATOM	6412	CE3	TRP	A	810	-43.156	41.219	26.422	1.00	92.62	C
ATOM	6413	NE1	TRP	A	810	-46.031	42.531	24.734	1.00	92.62	N
ATOM	6414	CH2	TRP	A	810	-42.875	40.719	24.047	1.00	92.62	C
ATOM	6415	CZ2	TRP	A	810	-44.062	41.312	23.734	1.00	92.62	C
ATOM	6416	CZ3	TRP	A	810	-42.438	40.656	25.375	1.00	92.62	C
ATOM	6417	N	THR	A	811	-44.156	41.188	30.984	1.00	93.31	N
ATOM	6418	CA	THR	A	811	-43.531	41.625	32.219	1.00	93.31	C
ATOM	6419	C	THR	A	811	-43.125	43.094	32.125	1.00	93.31	C
ATOM	6420	CB	THR	A	811	-42.312	40.750	32.562	1.00	93.31	C
ATOM	6421	O	THR	A	811	-43.062	43.656	31.047	1.00	93.31	O
ATOM	6422	CG2	THR	A	811	-42.688	39.281	32.594	1.00	93.31	C
ATOM	6423	OG1	THR	A	811	-41.312	40.969	31.578	1.00	93.31	O
ATOM	6424	N	ASP	A	812	-42.812	43.625	33.344	1.00	91.94	N
ATOM	6425	CA	ASP	A	812	-42.156	44.906	33.375	1.00	91.94	C
ATOM	6426	C	ASP	A	812	-40.719	44.844	32.844	1.00	91.94	C
ATOM	6427	CB	ASP	A	812	-42.156	45.500	34.781	1.00	91.94	C
ATOM	6428	O	ASP	A	812	-40.094	43.781	32.906	1.00	91.94	O
ATOM	6429	CG	ASP	A	812	-43.562	45.781	35.312	1.00	91.94	C
ATOM	6430	OD1	ASP	A	812	-44.469	46.125	34.500	1.00	91.94	O
ATOM	6431	OD2	ASP	A	812	-43.781	45.656	36.531	1.00	91.94	O
ATOM	6432	N	PHE	A	813	-40.250	45.875	32.188	1.00	94.25	N
ATOM	6433	CA	PHE	A	813	-38.875	45.906	31.734	1.00	94.25	C
ATOM	6434	C	PHE	A	813	-37.906	45.750	32.906	1.00	94.25	C
ATOM	6435	CB	PHE	A	813	-38.562	47.250	31.016	1.00	94.25	C
ATOM	6436	O	PHE	A	813	-38.156	46.250	34.000	1.00	94.25	O
ATOM	6437	CG	PHE	A	813	-39.031	47.250	29.578	1.00	94.25	C
ATOM	6438	CD1	PHE	A	813	-38.281	46.625	28.594	1.00	94.25	C
ATOM	6439	CD2	PHE	A	813	-40.188	47.938	29.219	1.00	94.25	C
ATOM	6440	CE1	PHE	A	813	-38.719	46.625	27.266	1.00	94.25	C
ATOM	6441	CE2	PHE	A	813	-40.594	47.969	27.891	1.00	94.25	C
ATOM	6442	CZ	PHE	A	813	-39.875	47.312	26.922	1.00	94.25	C
ATOM	6443	N	VAL	A	814	-36.938	44.844	32.719	1.00	92.81	N
ATOM	6444	CA	VAL	A	814	-35.875	44.719	33.688	1.00	92.81	C
ATOM	6445	C	VAL	A	814	-34.562	45.156	33.062	1.00	92.81	C
ATOM	6446	CB	VAL	A	814	-35.750	43.281	34.250	1.00	92.81	C
ATOM	6447	O	VAL	A	814	-34.312	44.906	31.875	1.00	92.81	O
ATOM	6448	CG1	VAL	A	814	-37.000	42.938	35.062	1.00	92.81	C
ATOM	6449	CG2	VAL	A	814	-35.531	42.312	33.094	1.00	92.81	C
ATOM	6450	N	ARG	A	815	-33.781	45.938	33.750	1.00	93.38	N
ATOM	6451	CA	ARG	A	815	-32.531	46.531	33.281	1.00	93.38	C
ATOM	6452	C	ARG	A	815	-31.375	45.562	33.406	1.00	93.38	C
ATOM	6453	CB	ARG	A	815	-32.219	47.812	34.094	1.00	93.38	C
ATOM	6454	O	ARG	A	815	-31.188	44.938	34.469	1.00	93.38	O
ATOM	6455	CG	ARG	A	815	-30.969	48.531	33.656	1.00	93.38	C
ATOM	6456	CD	ARG	A	815	-30.688	49.750	34.500	1.00	93.38	C
ATOM	6457	NE	ARG	A	815	-29.500	50.469	34.062	1.00	93.38	N
ATOM	6458	NH1	ARG	A	815	-28.000	49.219	35.281	1.00	93.38	N
ATOM	6459	NH2	ARG	A	815	-27.250	50.938	33.938	1.00	93.38	N
ATOM	6460	CZ	ARG	A	815	-28.250	50.219	34.438	1.00	93.38	C

```
ATOM   6461  N   GLY A 816     -30.734  45.156  32.281  1.00 92.62           N
ATOM   6462  CA  GLY A 816     -29.500  44.406  32.281  1.00 92.62           C
ATOM   6463  C   GLY A 816     -28.312  45.250  31.812  1.00 92.62           C
ATOM   6464  O   GLY A 816     -28.469  46.188  31.031  1.00 92.62           O
ATOM   6465  N   MET A 817     -27.266  44.875  32.375  1.00 91.06           N
ATOM   6466  CA  MET A 817     -26.047  45.594  32.031  1.00 91.06           C
ATOM   6467  C   MET A 817     -24.859  44.625  31.938  1.00 91.06           C
ATOM   6468  CB  MET A 817     -25.750  46.688  33.031  1.00 91.06           C
ATOM   6469  O   MET A 817     -24.672  43.781  32.812  1.00 91.06           O
ATOM   6470  CG  MET A 817     -24.531  47.531  32.688  1.00 91.06           C
ATOM   6471  SD  MET A 817     -24.250  48.906  33.906  1.00 91.06           S
ATOM   6472  CE  MET A 817     -23.625  47.938  35.312  1.00 91.06           C
ATOM   6473  N   ALA A 818     -24.266  44.594  30.797  1.00 89.12           N
ATOM   6474  CA  ALA A 818     -23.016  43.844  30.688  1.00 89.12           C
ATOM   6475  C   ALA A 818     -22.047  44.281  31.797  1.00 89.12           C
ATOM   6476  CB  ALA A 818     -22.391  44.031  29.312  1.00 89.12           C
ATOM   6477  O   ALA A 818     -22.000  45.438  32.156  1.00 89.12           O
ATOM   6478  N   SER A 819     -21.422  43.281  32.406  1.00 87.62           N
ATOM   6479  CA  SER A 819     -20.562  43.469  33.562  1.00 87.62           C
ATOM   6480  C   SER A 819     -19.609  44.656  33.375  1.00 87.62           C
ATOM   6481  CB  SER A 819     -19.766  42.219  33.875  1.00 87.62           C
ATOM   6482  O   SER A 819     -19.062  44.844  32.312  1.00 87.62           O
ATOM   6483  OG  SER A 819     -18.875  42.406  34.969  1.00 87.62           O
ATOM   6484  N   THR A 820     -19.547  45.594  34.281  1.00 82.31           N
ATOM   6485  CA  THR A 820     -18.531  46.656  34.281  1.00 82.31           C
ATOM   6486  C   THR A 820     -17.391  46.281  35.250  1.00 82.31           C
ATOM   6487  CB  THR A 820     -19.141  48.000  34.719  1.00 82.31           C
ATOM   6488  O   THR A 820     -16.516  47.125  35.500  1.00 82.31           O
ATOM   6489  CG2 THR A 820     -20.141  48.500  33.656  1.00 82.31           C
ATOM   6490  OG1 THR A 820     -19.828  47.844  35.969  1.00 82.31           O
ATOM   6491  N   ASN A 821     -17.578  44.875  35.812  1.00 82.12           N
ATOM   6492  CA  ASN A 821     -16.516  44.438  36.719  1.00 82.12           C
ATOM   6493  C   ASN A 821     -15.203  44.188  35.969  1.00 82.12           C
ATOM   6494  CB  ASN A 821     -16.953  43.125  37.438  1.00 82.12           C
ATOM   6495  O   ASN A 821     -15.094  43.281  35.156  1.00 82.12           O
ATOM   6496  CG  ASN A 821     -15.969  42.719  38.500  1.00 82.12           C
ATOM   6497  ND2 ASN A 821     -16.484  42.344  39.656  1.00 82.12           N
ATOM   6498  OD1 ASN A 821     -14.750  42.719  38.281  1.00 82.12           O
ATOM   6499  N   VAL A 822     -14.281  45.031  36.125  1.00 77.94           N
ATOM   6500  CA  VAL A 822     -13.016  45.094  35.406  1.00 77.94           C
ATOM   6501  C   VAL A 822     -12.266  43.781  35.562  1.00 77.94           C
ATOM   6502  CB  VAL A 822     -12.141  46.250  35.875  1.00 77.94           C
ATOM   6503  O   VAL A 822     -11.711  43.250  34.594  1.00 77.94           O
ATOM   6504  CG1 VAL A 822     -10.758  46.219  35.219  1.00 77.94           C
ATOM   6505  CG2 VAL A 822     -12.820  47.594  35.562  1.00 77.94           C
ATOM   6506  N   ASP A 823     -12.297  43.062  36.812  1.00 79.94           N
ATOM   6507  CA  ASP A 823     -11.570  41.812  37.062  1.00 79.94           C
ATOM   6508  C   ASP A 823     -12.102  40.688  36.188  1.00 79.94           C
ATOM   6509  CB  ASP A 823     -11.656  41.438  38.531  1.00 79.94           C
ATOM   6510  O   ASP A 823     -11.320  39.906  35.625  1.00 79.94           O
ATOM   6511  CG  ASP A 823     -10.891  42.375  39.438  1.00 79.94           C
ATOM   6512  OD1 ASP A 823      -9.891  42.969  39.000  1.00 79.94           O
ATOM   6513  OD2 ASP A 823     -11.289  42.531  40.625  1.00 79.94           O
ATOM   6514  N   ASP A 824     -13.406  40.562  36.000  1.00 80.38           N
ATOM   6515  CA  ASP A 824     -14.039  39.500  35.219  1.00 80.38           C
ATOM   6516  C   ASP A 824     -13.766  39.719  33.719  1.00 80.38           C
ATOM   6517  CB  ASP A 824     -15.547  39.469  35.469  1.00 80.38           C
ATOM   6518  O   ASP A 824     -13.500  38.750  33.000  1.00 80.38           O
ATOM   6519  CG  ASP A 824     -15.898  39.000  36.875  1.00 80.38           C
ATOM   6520  OD1 ASP A 824     -15.023  38.438  37.562  1.00 80.38           O
ATOM   6521  OD2 ASP A 824     -17.062  39.188  37.281  1.00 80.38           O
ATOM   6522  N   ILE A 825     -13.844  41.000  33.281  1.00 82.94           N
ATOM   6523  CA  ILE A 825     -13.609  41.312  31.859  1.00 82.94           C
ATOM   6524  C   ILE A 825     -12.164  40.969  31.500  1.00 82.94           C
```

ATOM	6525	CB	ILE	A	825	-13.922	42.781	31.562	1.00	82.94	C
ATOM	6526	O	ILE	A	825	-11.906	40.312	30.484	1.00	82.94	O
ATOM	6527	CG1	ILE	A	825	-15.430	43.062	31.688	1.00	82.94	C
ATOM	6528	CG2	ILE	A	825	-13.414	43.156	30.172	1.00	82.94	C
ATOM	6529	CD1	ILE	A	825	-15.805	44.531	31.578	1.00	82.94	C
ATOM	6530	N	ILE	A	826	-11.266	41.406	32.406	1.00	81.38	N
ATOM	6531	CA	ILE	A	826	-9.852	41.125	32.156	1.00	81.38	C
ATOM	6532	C	ILE	A	826	-9.617	39.625	32.156	1.00	81.38	C
ATOM	6533	CB	ILE	A	826	-8.961	41.812	33.219	1.00	81.38	C
ATOM	6534	O	ILE	A	826	-8.852	39.125	31.328	1.00	81.38	O
ATOM	6535	CG1	ILE	A	826	-8.961	43.312	33.031	1.00	81.38	C
ATOM	6536	CG2	ILE	A	826	-7.539	41.250	33.188	1.00	81.38	C
ATOM	6537	CD1	ILE	A	826	-8.188	44.094	34.125	1.00	81.38	C
ATOM	6538	N	GLY	A	827	-10.242	38.875	33.031	1.00	81.88	N
ATOM	6539	CA	GLY	A	827	-10.102	37.438	33.094	1.00	81.88	C
ATOM	6540	C	GLY	A	827	-10.484	36.719	31.812	1.00	81.88	C
ATOM	6541	O	GLY	A	827	-9.773	35.812	31.359	1.00	81.88	O
ATOM	6542	N	GLU	A	828	-11.641	37.031	31.125	1.00	81.62	N
ATOM	6543	CA	GLU	A	828	-12.078	36.406	29.875	1.00	81.62	C
ATOM	6544	C	GLU	A	828	-11.125	36.750	28.734	1.00	81.62	C
ATOM	6545	CB	GLU	A	828	-13.500	36.875	29.531	1.00	81.62	C
ATOM	6546	O	GLU	A	828	-10.797	35.906	27.922	1.00	81.62	O
ATOM	6547	CG	GLU	A	828	-14.211	35.969	28.531	1.00	81.62	C
ATOM	6548	CD	GLU	A	828	-14.516	34.594	29.094	1.00	81.62	C
ATOM	6549	OE1	GLU	A	828	-14.969	33.719	28.312	1.00	81.62	O
ATOM	6550	OE2	GLU	A	828	-14.305	34.375	30.297	1.00	81.62	O
ATOM	6551	N	ILE	A	829	-10.742	38.062	28.656	1.00	82.94	N
ATOM	6552	CA	ILE	A	829	-9.789	38.500	27.641	1.00	82.94	C
ATOM	6553	C	ILE	A	829	-8.461	37.781	27.828	1.00	82.94	C
ATOM	6554	CB	ILE	A	829	-9.586	40.031	27.656	1.00	82.94	C
ATOM	6555	O	ILE	A	829	-7.820	37.344	26.859	1.00	82.94	O
ATOM	6556	CG1	ILE	A	829	-10.867	40.750	27.219	1.00	82.94	C
ATOM	6557	CG2	ILE	A	829	-8.406	40.406	26.750	1.00	82.94	C
ATOM	6558	CD1	ILE	A	829	-10.812	42.250	27.375	1.00	82.94	C
ATOM	6559	N	SER	A	830	-8.086	37.531	29.156	1.00	83.94	N
ATOM	6560	CA	SER	A	830	-6.836	36.844	29.484	1.00	83.94	C
ATOM	6561	C	SER	A	830	-6.824	35.406	28.969	1.00	83.94	C
ATOM	6562	CB	SER	A	830	-6.613	36.844	31.000	1.00	83.94	C
ATOM	6563	O	SER	A	830	-5.809	34.938	28.438	1.00	83.94	O
ATOM	6564	OG	SER	A	830	-5.355	36.281	31.328	1.00	83.94	O
ATOM	6565	N	VAL	A	831	-7.945	34.625	29.141	1.00	83.56	N
ATOM	6566	CA	VAL	A	831	-8.023	33.250	28.688	1.00	83.56	C
ATOM	6567	C	VAL	A	831	-7.789	33.188	27.188	1.00	83.56	C
ATOM	6568	CB	VAL	A	831	-9.383	32.594	29.047	1.00	83.56	C
ATOM	6569	O	VAL	A	831	-7.043	32.344	26.688	1.00	83.56	O
ATOM	6570	CG1	VAL	A	831	-9.531	31.250	28.359	1.00	83.56	C
ATOM	6571	CG2	VAL	A	831	-9.523	32.469	30.562	1.00	83.56	C
ATOM	6572	N	ASP	A	832	-8.469	34.062	26.469	1.00	81.88	N
ATOM	6573	CA	ASP	A	832	-8.328	34.125	25.016	1.00	81.88	C
ATOM	6574	C	ASP	A	832	-6.891	34.469	24.609	1.00	81.88	C
ATOM	6575	CB	ASP	A	832	-9.289	35.156	24.422	1.00	81.88	C
ATOM	6576	O	ASP	A	832	-6.328	33.844	23.703	1.00	81.88	O
ATOM	6577	CG	ASP	A	832	-9.484	35.000	22.922	1.00	81.88	C
ATOM	6578	OD1	ASP	A	832	-9.250	33.875	22.391	1.00	81.88	O
ATOM	6579	OD2	ASP	A	832	-9.883	35.969	22.266	1.00	81.88	O
ATOM	6580	N	ILE	A	833	-6.250	35.469	25.250	1.00	85.06	N
ATOM	6581	CA	ILE	A	833	-4.871	35.875	25.000	1.00	85.06	C
ATOM	6582	C	ILE	A	833	-3.918	34.719	25.344	1.00	85.06	C
ATOM	6583	CB	ILE	A	833	-4.504	37.125	25.797	1.00	85.06	C
ATOM	6584	O	ILE	A	833	-3.008	34.406	24.578	1.00	85.06	O
ATOM	6585	CG1	ILE	A	833	-5.242	38.344	25.234	1.00	85.06	C
ATOM	6586	CG2	ILE	A	833	-2.990	37.375	25.781	1.00	85.06	C
ATOM	6587	CD1	ILE	A	833	-5.059	39.625	26.047	1.00	85.06	C
ATOM	6588	N	GLU	A	834	-4.172	34.031	26.500	1.00	86.69	N

ATOM	6589	CA	GLU	A	834	-3.281	32.969	26.984	1.00	86.69	C
ATOM	6590	C	GLU	A	834	-3.277	31.781	26.047	1.00	86.69	C
ATOM	6591	CB	GLU	A	834	-3.684	32.531	28.391	1.00	86.69	C
ATOM	6592	O	GLU	A	834	-2.293	31.047	25.969	1.00	86.69	O
ATOM	6593	CG	GLU	A	834	-3.369	33.562	29.469	1.00	86.69	C
ATOM	6594	CD	GLU	A	834	-3.855	33.156	30.859	1.00	86.69	C
ATOM	6595	OE1	GLU	A	834	-3.617	33.906	31.828	1.00	86.69	O
ATOM	6596	OE2	GLU	A	834	-4.477	32.062	30.969	1.00	86.69	O
ATOM	6597	N	ASN	A	835	-4.359	31.531	25.281	1.00	83.25	N
ATOM	6598	CA	ASN	A	835	-4.453	30.406	24.359	1.00	83.25	C
ATOM	6599	C	ASN	A	835	-3.961	30.766	22.969	1.00	83.25	C
ATOM	6600	CB	ASN	A	835	-5.891	29.875	24.297	1.00	83.25	C
ATOM	6601	O	ASN	A	835	-3.910	29.922	22.078	1.00	83.25	O
ATOM	6602	CG	ASN	A	835	-6.305	29.172	25.578	1.00	83.25	C
ATOM	6603	ND2	ASN	A	835	-7.605	29.156	25.844	1.00	83.25	N
ATOM	6604	OD1	ASN	A	835	-5.469	28.625	26.297	1.00	83.25	O
ATOM	6605	N	SER	A	836	-3.488	32.062	22.688	1.00	86.06	N
ATOM	6606	CA	SER	A	836	-2.996	32.500	21.375	1.00	86.06	C
ATOM	6607	C	SER	A	836	-1.529	32.156	21.188	1.00	86.06	C
ATOM	6608	CB	SER	A	836	-3.178	34.031	21.219	1.00	86.06	C
ATOM	6609	O	SER	A	836	-0.779	32.031	22.156	1.00	86.06	O
ATOM	6610	OG	SER	A	836	-2.266	34.719	22.047	1.00	86.06	O
ATOM	6611	N	PRO	A	837	-1.179	31.734	19.938	1.00	83.31	N
ATOM	6612	CA	PRO	A	837	0.215	31.406	19.641	1.00	83.31	C
ATOM	6613	C	PRO	A	837	1.193	32.500	20.078	1.00	83.31	C
ATOM	6614	CB	PRO	A	837	0.224	31.234	18.109	1.00	83.31	C
ATOM	6615	O	PRO	A	837	2.279	32.188	20.578	1.00	83.31	O
ATOM	6616	CG	PRO	A	837	-1.198	30.953	17.750	1.00	83.31	C
ATOM	6617	CD	PRO	A	837	-2.088	31.578	18.797	1.00	83.31	C
ATOM	6618	N	GLY	A	838	0.822	33.781	19.938	1.00	85.81	N
ATOM	6619	CA	GLY	A	838	1.675	34.875	20.344	1.00	85.81	C
ATOM	6620	C	GLY	A	838	1.964	34.875	21.828	1.00	85.81	C
ATOM	6621	O	GLY	A	838	3.104	35.094	22.250	1.00	85.81	O
ATOM	6622	N	TYR	A	839	0.933	34.562	22.656	1.00	88.19	N
ATOM	6623	CA	TYR	A	839	1.097	34.531	24.109	1.00	88.19	C
ATOM	6624	C	TYR	A	839	1.994	33.375	24.531	1.00	88.19	C
ATOM	6625	CB	TYR	A	839	-0.264	34.438	24.797	1.00	88.19	C
ATOM	6626	O	TYR	A	839	2.852	33.531	25.406	1.00	88.19	O
ATOM	6627	CG	TYR	A	839	-0.178	34.406	26.312	1.00	88.19	C
ATOM	6628	CD1	TYR	A	839	-0.209	33.188	27.016	1.00	88.19	C
ATOM	6629	CD2	TYR	A	839	-0.066	35.594	27.031	1.00	88.19	C
ATOM	6630	CE1	TYR	A	839	-0.132	33.188	28.406	1.00	88.19	C
ATOM	6631	CE2	TYR	A	839	0.012	35.562	28.422	1.00	88.19	C
ATOM	6632	OH	TYR	A	839	0.055	34.344	30.469	1.00	88.19	O
ATOM	6633	CZ	TYR	A	839	-0.021	34.375	29.094	1.00	88.19	C
ATOM	6634	N	GLU	A	840	1.728	32.188	23.922	1.00	86.38	N
ATOM	6635	CA	GLU	A	840	2.525	31.016	24.219	1.00	86.38	C
ATOM	6636	C	GLU	A	840	4.012	31.281	24.000	1.00	86.38	C
ATOM	6637	CB	GLU	A	840	2.076	29.828	23.375	1.00	86.38	C
ATOM	6638	O	GLU	A	840	4.848	30.859	24.812	1.00	86.38	O
ATOM	6639	CG	GLU	A	840	0.786	29.188	23.844	1.00	86.38	C
ATOM	6640	CD	GLU	A	840	0.364	27.984	23.000	1.00	86.38	C
ATOM	6641	OE1	GLU	A	840	-0.678	27.359	23.312	1.00	86.38	O
ATOM	6642	OE2	GLU	A	840	1.081	27.672	22.031	1.00	86.38	O
ATOM	6643	N	TRP	A	841	4.316	32.031	22.906	1.00	87.75	N
ATOM	6644	CA	TRP	A	841	5.707	32.344	22.609	1.00	87.75	C
ATOM	6645	C	TRP	A	841	6.242	33.375	23.609	1.00	87.75	C
ATOM	6646	CB	TRP	A	841	5.844	32.875	21.188	1.00	87.75	C
ATOM	6647	O	TRP	A	841	7.371	33.250	24.078	1.00	87.75	O
ATOM	6648	CG	TRP	A	841	7.246	33.250	20.797	1.00	87.75	C
ATOM	6649	CD1	TRP	A	841	8.234	32.406	20.391	1.00	87.75	C
ATOM	6650	CD2	TRP	A	841	7.812	34.562	20.797	1.00	87.75	C
ATOM	6651	CE2	TRP	A	841	9.156	34.438	20.375	1.00	87.75	C
ATOM	6652	CE3	TRP	A	841	7.316	35.844	21.109	1.00	87.75	C

ATOM	6653	NE1	TRP	A	841	9.391	33.125	20.141	1.00	87.75	N
ATOM	6654	CH2	TRP	A	841	9.492	36.781	20.578	1.00	87.75	C
ATOM	6655	CZ2	TRP	A	841	10.008	35.531	20.266	1.00	87.75	C
ATOM	6656	CZ3	TRP	A	841	8.164	36.938	21.000	1.00	87.75	C
ATOM	6657	N	LEU	A	842	5.434	34.375	23.922	1.00	87.38	N
ATOM	6658	CA	LEU	A	842	5.844	35.500	24.750	1.00	87.38	C
ATOM	6659	C	LEU	A	842	6.141	35.062	26.172	1.00	87.38	C
ATOM	6660	CB	LEU	A	842	4.758	36.594	24.781	1.00	87.38	C
ATOM	6661	O	LEU	A	842	7.039	35.594	26.828	1.00	87.38	O
ATOM	6662	CG	LEU	A	842	5.141	37.938	25.406	1.00	87.38	C
ATOM	6663	CD1	LEU	A	842	6.254	38.594	24.609	1.00	87.38	C
ATOM	6664	CD2	LEU	A	842	3.924	38.844	25.500	1.00	87.38	C
ATOM	6665	N	VAL	A	843	5.516	33.906	26.703	1.00	85.75	N
ATOM	6666	CA	VAL	A	843	5.672	33.500	28.094	1.00	85.75	C
ATOM	6667	C	VAL	A	843	6.922	32.625	28.219	1.00	85.75	C
ATOM	6668	CB	VAL	A	843	4.430	32.719	28.594	1.00	85.75	C
ATOM	6669	O	VAL	A	843	7.441	32.438	29.328	1.00	85.75	O
ATOM	6670	CG1	VAL	A	843	4.664	32.188	30.000	1.00	85.75	C
ATOM	6671	CG2	VAL	A	843	3.197	33.625	28.562	1.00	85.75	C
ATOM	6672	N	ASP	A	844	7.387	32.125	27.062	1.00	84.44	N
ATOM	6673	CA	ASP	A	844	8.617	31.344	27.062	1.00	84.44	C
ATOM	6674	C	ASP	A	844	9.828	32.219	27.391	1.00	84.44	C
ATOM	6675	CB	ASP	A	844	8.812	30.672	25.703	1.00	84.44	C
ATOM	6676	O	ASP	A	844	9.922	33.344	26.922	1.00	84.44	O
ATOM	6677	CG	ASP	A	844	7.938	29.438	25.516	1.00	84.44	C
ATOM	6678	OD1	ASP	A	844	7.297	29.000	26.500	1.00	84.44	O
ATOM	6679	OD2	ASP	A	844	7.887	28.906	24.391	1.00	84.44	O
ATOM	6680	N	ASN	A	845	10.641	31.719	28.281	1.00	82.44	N
ATOM	6681	CA	ASN	A	845	11.828	32.438	28.734	1.00	82.44	C
ATOM	6682	C	ASN	A	845	12.867	32.594	27.625	1.00	82.44	C
ATOM	6683	CB	ASN	A	845	12.445	31.719	29.938	1.00	82.44	C
ATOM	6684	O	ASN	A	845	13.180	31.625	26.938	1.00	82.44	O
ATOM	6685	CG	ASN	A	845	13.602	32.469	30.547	1.00	82.44	C
ATOM	6686	ND2	ASN	A	845	13.453	32.875	31.812	1.00	82.44	N
ATOM	6687	OD1	ASN	A	845	14.625	32.719	29.891	1.00	82.44	O
ATOM	6688	N	ALA	A	846	13.289	33.875	27.281	1.00	86.12	N
ATOM	6689	CA	ALA	A	846	14.234	34.125	26.203	1.00	86.12	C
ATOM	6690	C	ALA	A	846	15.539	33.375	26.422	1.00	86.12	C
ATOM	6691	CB	ALA	A	846	14.500	35.625	26.109	1.00	86.12	C
ATOM	6692	O	ALA	A	846	16.156	32.906	25.453	1.00	86.12	O
ATOM	6693	N	THR	A	847	16.031	33.219	27.703	1.00	83.88	N
ATOM	6694	CA	THR	A	847	17.250	32.500	28.000	1.00	83.88	C
ATOM	6695	C	THR	A	847	17.078	31.016	27.688	1.00	83.88	C
ATOM	6696	CB	THR	A	847	17.656	32.656	29.484	1.00	83.88	C
ATOM	6697	O	THR	A	847	18.000	30.391	27.141	1.00	83.88	O
ATOM	6698	CG2	THR	A	847	18.188	34.062	29.750	1.00	83.88	C
ATOM	6699	OG1	THR	A	847	16.500	32.438	30.312	1.00	83.88	O
ATOM	6700	N	ASP	A	848	15.938	30.438	28.000	1.00	85.44	N
ATOM	6701	CA	ASP	A	848	15.617	29.047	27.688	1.00	85.44	C
ATOM	6702	C	ASP	A	848	15.523	28.844	26.172	1.00	85.44	C
ATOM	6703	CB	ASP	A	848	14.312	28.625	28.359	1.00	85.44	C
ATOM	6704	O	ASP	A	848	16.000	27.844	25.641	1.00	85.44	O
ATOM	6705	CG	ASP	A	848	14.438	28.484	29.859	1.00	85.44	C
ATOM	6706	OD1	ASP	A	848	15.578	28.469	30.375	1.00	85.44	O
ATOM	6707	OD2	ASP	A	848	13.391	28.406	30.547	1.00	85.44	O
ATOM	6708	N	ASN	A	849	14.828	29.906	25.578	1.00	85.75	N
ATOM	6709	CA	ASN	A	849	14.695	29.844	24.125	1.00	85.75	C
ATOM	6710	C	ASN	A	849	16.062	29.859	23.438	1.00	85.75	C
ATOM	6711	CB	ASN	A	849	13.828	30.984	23.625	1.00	85.75	C
ATOM	6712	O	ASN	A	849	16.297	29.109	22.500	1.00	85.75	O
ATOM	6713	CG	ASN	A	849	12.367	30.609	23.484	1.00	85.75	C
ATOM	6714	ND2	ASN	A	849	11.477	31.547	23.781	1.00	85.75	N
ATOM	6715	OD1	ASN	A	849	12.039	29.469	23.141	1.00	85.75	O
ATOM	6716	N	ALA	A	850	16.906	30.688	23.859	1.00	85.12	N

```
ATOM   6717  CA  ALA A 850      18.250  30.781  23.312  1.00 85.12           C
ATOM   6718  C   ALA A 850      19.016  29.469  23.516  1.00 85.12           C
ATOM   6719  CB  ALA A 850      19.016  31.922  23.984  1.00 85.12           C
ATOM   6720  O   ALA A 850      19.703  29.000  22.609  1.00 85.12           O
ATOM   6721  N   ALA A 851      18.859  28.828  24.688  1.00 84.31           N
ATOM   6722  CA  ALA A 851      19.500  27.562  24.984  1.00 84.31           C
ATOM   6723  C   ALA A 851      18.969  26.453  24.094  1.00 84.31           C
ATOM   6724  CB  ALA A 851      19.312  27.203  26.453  1.00 84.31           C
ATOM   6725  O   ALA A 851      19.734  25.625  23.578  1.00 84.31           O
ATOM   6726  N   GLN A 852      17.656  26.422  23.984  1.00 85.62           N
ATOM   6727  CA  GLN A 852      17.000  25.406  23.156  1.00 85.62           C
ATOM   6728  C   GLN A 852      17.406  25.547  21.703  1.00 85.62           C
ATOM   6729  CB  GLN A 852      15.477  25.516  23.297  1.00 85.62           C
ATOM   6730  O   GLN A 852      17.703  24.547  21.031  1.00 85.62           O
ATOM   6731  CG  GLN A 852      14.836  24.297  23.953  1.00 85.62           C
ATOM   6732  CD  GLN A 852      13.336  24.453  24.141  1.00 85.62           C
ATOM   6733  NE2 GLN A 852      12.680  23.375  24.578  1.00 85.62           N
ATOM   6734  OE1 GLN A 852      12.773  25.531  23.906  1.00 85.62           O
ATOM   6735  N   ASN A 853      17.391  26.828  21.141  1.00 86.12           N
ATOM   6736  CA  ASN A 853      17.781  27.078  19.750  1.00 86.12           C
ATOM   6737  C   ASN A 853      19.234  26.719  19.500  1.00 86.12           C
ATOM   6738  CB  ASN A 853      17.516  28.547  19.375  1.00 86.12           C
ATOM   6739  O   ASN A 853      19.578  26.188  18.438  1.00 86.12           O
ATOM   6740  CG  ASN A 853      16.047  28.828  19.141  1.00 86.12           C
ATOM   6741  ND2 ASN A 853      15.711  30.109  19.000  1.00 86.12           N
ATOM   6742  OD1 ASN A 853      15.227  27.922  19.094  1.00 86.12           O
ATOM   6743  N   SER A 854      20.047  27.000  20.500  1.00 82.88           N
ATOM   6744  CA  SER A 854      21.453  26.609  20.438  1.00 82.88           C
ATOM   6745  C   SER A 854      21.609  25.094  20.406  1.00 82.88           C
ATOM   6746  CB  SER A 854      22.234  27.203  21.609  1.00 82.88           C
ATOM   6747  O   SER A 854      22.391  24.562  19.625  1.00 82.88           O
ATOM   6748  OG  SER A 854      23.609  26.922  21.484  1.00 82.88           O
ATOM   6749  N   ALA A 855      20.906  24.406  21.250  1.00 83.19           N
ATOM   6750  CA  ALA A 855      20.953  22.953  21.328  1.00 83.19           C
ATOM   6751  C   ALA A 855      20.453  22.312  20.031  1.00 83.19           C
ATOM   6752  CB  ALA A 855      20.125  22.453  22.516  1.00 83.19           C
ATOM   6753  O   ALA A 855      21.047  21.344  19.547  1.00 83.19           O
ATOM   6754  N   ASN A 856      19.328  22.859  19.516  1.00 81.56           N
ATOM   6755  CA  ASN A 856      18.781  22.375  18.266  1.00 81.56           C
ATOM   6756  C   ASN A 856      19.766  22.562  17.109  1.00 81.56           C
ATOM   6757  CB  ASN A 856      17.453  23.062  17.938  1.00 81.56           C
ATOM   6758  O   ASN A 856      19.922  21.672  16.281  1.00 81.56           O
ATOM   6759  CG  ASN A 856      16.312  22.594  18.844  1.00 81.56           C
ATOM   6760  ND2 ASN A 856      15.180  23.281  18.766  1.00 81.56           N
ATOM   6761  OD1 ASN A 856      16.469  21.641  19.594  1.00 81.56           O
ATOM   6762  N   ALA A 857      20.375  23.719  17.031  1.00 80.44           N
ATOM   6763  CA  ALA A 857      21.375  23.984  16.000  1.00 80.44           C
ATOM   6764  C   ALA A 857      22.578  23.031  16.141  1.00 80.44           C
ATOM   6765  CB  ALA A 857      21.844  25.438  16.094  1.00 80.44           C
ATOM   6766  O   ALA A 857      23.062  22.500  15.156  1.00 80.44           O
ATOM   6767  N   GLU A 858      22.984  22.766  17.453  1.00 81.75           N
ATOM   6768  CA  GLU A 858      24.125  21.891  17.719  1.00 81.75           C
ATOM   6769  C   GLU A 858      23.812  20.453  17.328  1.00 81.75           C
ATOM   6770  CB  GLU A 858      24.531  21.969  19.188  1.00 81.75           C
ATOM   6771  O   GLU A 858      24.672  19.750  16.766  1.00 81.75           O
ATOM   6772  CG  GLU A 858      25.453  23.141  19.516  1.00 81.75           C
ATOM   6773  CD  GLU A 858      25.734  23.281  21.000  1.00 81.75           C
ATOM   6774  OE1 GLU A 858      26.359  24.281  21.406  1.00 81.75           O
ATOM   6775  OE2 GLU A 858      25.328  22.391  21.766  1.00 81.75           O
ATOM   6776  N   ALA A 859      22.688  20.000  17.719  1.00 80.25           N
ATOM   6777  CA  ALA A 859      22.250  18.641  17.375  1.00 80.25           C
ATOM   6778  C   ALA A 859      22.188  18.453  15.867  1.00 80.25           C
ATOM   6779  CB  ALA A 859      20.875  18.359  18.000  1.00 80.25           C
ATOM   6780  O   ALA A 859      22.609  17.422  15.344  1.00 80.25           O
```

ATOM	6781	N	ALA	A	860	21.609	19.453	15.156	1.00	75.94	N
ATOM	6782	CA	ALA	A	860	21.547	19.391	13.703	1.00	75.94	C
ATOM	6783	C	ALA	A	860	22.938	19.406	13.086	1.00	75.94	C
ATOM	6784	CB	ALA	A	860	20.719	20.562	13.164	1.00	75.94	C
ATOM	6785	O	ALA	A	860	23.188	18.703	12.102	1.00	75.94	O
ATOM	6786	N	ILE	A	861	23.859	20.234	13.609	1.00	75.81	N
ATOM	6787	CA	ILE	A	861	25.250	20.297	13.172	1.00	75.81	C
ATOM	6788	C	ILE	A	861	25.922	18.938	13.375	1.00	75.81	C
ATOM	6789	CB	ILE	A	861	26.031	21.391	13.922	1.00	75.81	C
ATOM	6790	O	ILE	A	861	26.609	18.438	12.484	1.00	75.81	O
ATOM	6791	CG1	ILE	A	861	25.516	22.781	13.516	1.00	75.81	C
ATOM	6792	CG2	ILE	A	861	27.531	21.266	13.664	1.00	75.81	C
ATOM	6793	CD1	ILE	A	861	26.047	23.922	14.383	1.00	75.81	C
ATOM	6794	N	GLU	A	862	25.688	18.344	14.617	1.00	80.25	N
ATOM	6795	CA	GLU	A	862	26.281	17.047	14.945	1.00	80.25	C
ATOM	6796	C	GLU	A	862	25.781	15.961	14.000	1.00	80.25	C
ATOM	6797	CB	GLU	A	862	25.969	16.672	16.391	1.00	80.25	C
ATOM	6798	O	GLU	A	862	26.562	15.141	13.523	1.00	80.25	O
ATOM	6799	CG	GLU	A	862	26.859	15.578	16.953	1.00	80.25	C
ATOM	6800	CD	GLU	A	862	26.625	15.305	18.438	1.00	80.25	C
ATOM	6801	OE1	GLU	A	862	27.281	14.398	18.984	1.00	80.25	O
ATOM	6802	OE2	GLU	A	862	25.797	16.016	19.031	1.00	80.25	O
ATOM	6803	N	ASN	A	863	24.469	15.922	13.742	1.00	74.62	N
ATOM	6804	CA	ASN	A	863	23.875	14.953	12.836	1.00	74.62	C
ATOM	6805	C	ASN	A	863	24.391	15.133	11.406	1.00	74.62	C
ATOM	6806	CB	ASN	A	863	22.344	15.062	12.867	1.00	74.62	C
ATOM	6807	O	ASN	A	863	24.656	14.156	10.719	1.00	74.62	O
ATOM	6808	CG	ASN	A	863	21.719	14.133	13.883	1.00	74.62	C
ATOM	6809	ND2	ASN	A	863	20.438	14.359	14.188	1.00	74.62	N
ATOM	6810	OD1	ASN	A	863	22.359	13.219	14.391	1.00	74.62	O
ATOM	6811	N	ALA	A	864	24.469	16.391	10.867	1.00	70.88	N
ATOM	6812	CA	ALA	A	864	25.000	16.672	9.531	1.00	70.88	C
ATOM	6813	C	ALA	A	864	26.453	16.234	9.406	1.00	70.88	C
ATOM	6814	CB	ALA	A	864	24.859	18.156	9.203	1.00	70.88	C
ATOM	6815	O	ALA	A	864	26.859	15.703	8.367	1.00	70.88	O
ATOM	6816	N	LEU	A	865	27.281	16.344	10.484	1.00	70.69	N
ATOM	6817	CA	LEU	A	865	28.703	15.992	10.484	1.00	70.69	C
ATOM	6818	C	LEU	A	865	28.891	14.484	10.523	1.00	70.69	C
ATOM	6819	CB	LEU	A	865	29.406	16.641	11.672	1.00	70.69	C
ATOM	6820	O	LEU	A	865	29.844	13.953	9.945	1.00	70.69	O
ATOM	6821	CG	LEU	A	865	29.672	18.141	11.570	1.00	70.69	C
ATOM	6822	CD1	LEU	A	865	30.000	18.719	12.945	1.00	70.69	C
ATOM	6823	CD2	LEU	A	865	30.812	18.422	10.586	1.00	70.69	C
ATOM	6824	N	ALA	A	866	28.016	13.727	11.242	1.00	70.06	N
ATOM	6825	CA	ALA	A	866	28.156	12.281	11.398	1.00	70.06	C
ATOM	6826	C	ALA	A	866	27.938	11.562	10.070	1.00	70.06	C
ATOM	6827	CB	ALA	A	866	27.172	11.766	12.445	1.00	70.06	C
ATOM	6828	O	ALA	A	866	28.594	10.562	9.789	1.00	70.06	O
ATOM	6829	N	ASN	A	867	27.250	12.211	9.062	1.00	61.25	N
ATOM	6830	CA	ASN	A	867	26.875	11.453	7.879	1.00	61.25	C
ATOM	6831	C	ASN	A	867	27.422	12.086	6.605	1.00	61.25	C
ATOM	6832	CB	ASN	A	867	25.344	11.312	7.793	1.00	61.25	C
ATOM	6833	O	ASN	A	867	27.391	11.469	5.539	1.00	61.25	O
ATOM	6834	CG	ASN	A	867	24.781	10.398	8.859	1.00	61.25	C
ATOM	6835	ND2	ASN	A	867	23.531	10.648	9.258	1.00	61.25	N
ATOM	6836	OD1	ASN	A	867	25.453	9.484	9.336	1.00	61.25	O
ATOM	6837	N	ASP	A	868	28.047	13.281	6.684	1.00	64.31	N
ATOM	6838	CA	ASP	A	868	28.406	14.023	5.480	1.00	64.31	C
ATOM	6839	C	ASP	A	868	29.922	13.969	5.246	1.00	64.31	C
ATOM	6840	CB	ASP	A	868	27.938	15.477	5.586	1.00	64.31	C
ATOM	6841	O	ASP	A	868	30.688	14.516	6.035	1.00	64.31	O
ATOM	6842	CG	ASP	A	868	28.062	16.234	4.273	1.00	64.31	C
ATOM	6843	OD1	ASP	A	868	28.547	15.656	3.277	1.00	64.31	O
ATOM	6844	OD2	ASP	A	868	27.672	17.422	4.238	1.00	64.31	O

ATOM	6845	N	LYS	A	869	30.484	13.117	4.328	1.00	64.19	N
ATOM	6846	CA	LYS	A	869	31.891	12.992	4.000	1.00	64.19	C
ATOM	6847	C	LYS	A	869	32.438	14.289	3.412	1.00	64.19	C
ATOM	6848	CB	LYS	A	869	32.125	11.844	3.021	1.00	64.19	C
ATOM	6849	O	LYS	A	869	33.656	14.539	3.453	1.00	64.19	O
ATOM	6850	CG	LYS	A	869	31.844	10.469	3.613	1.00	64.19	C
ATOM	6851	CD	LYS	A	869	32.312	9.352	2.674	1.00	64.19	C
ATOM	6852	CE	LYS	A	869	31.969	7.977	3.240	1.00	64.19	C
ATOM	6853	NZ	LYS	A	869	32.438	6.883	2.328	1.00	64.19	N
ATOM	6854	N	ASP	A	870	31.516	15.312	3.113	1.00	69.88	N
ATOM	6855	CA	ASP	A	870	31.984	16.531	2.441	1.00	69.88	C
ATOM	6856	C	ASP	A	870	31.984	17.719	3.393	1.00	69.88	C
ATOM	6857	CB	ASP	A	870	31.109	16.828	1.219	1.00	69.88	C
ATOM	6858	O	ASP	A	870	32.188	18.859	2.969	1.00	69.88	O
ATOM	6859	CG	ASP	A	870	31.250	15.781	0.124	1.00	69.88	C
ATOM	6860	OD1	ASP	A	870	32.344	15.172	-0.009	1.00	69.88	O
ATOM	6861	OD2	ASP	A	870	30.266	15.570	-0.613	1.00	69.88	O
ATOM	6862	N	ALA	A	871	31.641	17.469	4.715	1.00	74.00	N
ATOM	6863	CA	ALA	A	871	31.656	18.562	5.684	1.00	74.00	C
ATOM	6864	C	ALA	A	871	33.031	19.203	5.754	1.00	74.00	C
ATOM	6865	CB	ALA	A	871	31.234	18.062	7.062	1.00	74.00	C
ATOM	6866	O	ALA	A	871	34.062	18.516	5.637	1.00	74.00	O
ATOM	6867	N	ILE	A	872	33.156	20.578	5.555	1.00	80.69	N
ATOM	6868	CA	ILE	A	872	34.406	21.328	5.695	1.00	80.69	C
ATOM	6869	C	ILE	A	872	34.594	21.703	7.160	1.00	80.69	C
ATOM	6870	CB	ILE	A	872	34.406	22.594	4.812	1.00	80.69	C
ATOM	6871	O	ILE	A	872	33.750	22.312	7.781	1.00	80.69	O
ATOM	6872	CG1	ILE	A	872	34.219	22.219	3.338	1.00	80.69	C
ATOM	6873	CG2	ILE	A	872	35.688	23.391	5.020	1.00	80.69	C
ATOM	6874	CD1	ILE	A	872	34.000	23.406	2.416	1.00	80.69	C
ATOM	6875	N	TYR	A	873	35.625	21.031	7.738	1.00	82.75	N
ATOM	6876	CA	TYR	A	873	36.031	21.328	9.109	1.00	82.75	C
ATOM	6877	C	TYR	A	873	37.406	21.984	9.133	1.00	82.75	C
ATOM	6878	CB	TYR	A	873	36.031	20.062	9.961	1.00	82.75	C
ATOM	6879	O	TYR	A	873	38.375	21.422	8.633	1.00	82.75	O
ATOM	6880	CG	TYR	A	873	36.281	20.312	11.430	1.00	82.75	C
ATOM	6881	CD1	TYR	A	873	37.531	20.094	11.992	1.00	82.75	C
ATOM	6882	CD2	TYR	A	873	35.250	20.766	12.250	1.00	82.75	C
ATOM	6883	CE1	TYR	A	873	37.781	20.328	13.344	1.00	82.75	C
ATOM	6884	CE2	TYR	A	873	35.469	21.000	13.602	1.00	82.75	C
ATOM	6885	OH	TYR	A	873	36.969	21.016	15.484	1.00	82.75	O
ATOM	6886	CZ	TYR	A	873	36.750	20.781	14.141	1.00	82.75	C
ATOM	6887	N	MET	A	874	37.531	23.281	9.492	1.00	83.62	N
ATOM	6888	CA	MET	A	874	38.781	24.000	9.648	1.00	83.62	C
ATOM	6889	C	MET	A	874	39.031	24.344	11.109	1.00	83.62	C
ATOM	6890	CB	MET	A	874	38.812	25.266	8.789	1.00	83.62	C
ATOM	6891	O	MET	A	874	38.156	24.906	11.781	1.00	83.62	O
ATOM	6892	CG	MET	A	874	38.625	25.000	7.305	1.00	83.62	C
ATOM	6893	SD	MET	A	874	38.719	26.531	6.305	1.00	83.62	S
ATOM	6894	CE	MET	A	874	38.125	25.906	4.703	1.00	83.62	C
ATOM	6895	N	LYS	A	875	40.094	23.734	11.594	1.00	88.44	N
ATOM	6896	CA	LYS	A	875	40.469	23.969	12.984	1.00	88.44	C
ATOM	6897	C	LYS	A	875	41.906	24.500	13.109	1.00	88.44	C
ATOM	6898	CB	LYS	A	875	40.312	22.688	13.797	1.00	88.44	C
ATOM	6899	O	LYS	A	875	42.812	24.031	12.406	1.00	88.44	O
ATOM	6900	CG	LYS	A	875	40.656	22.828	15.273	1.00	88.44	C
ATOM	6901	CD	LYS	A	875	40.531	21.516	16.016	1.00	88.44	C
ATOM	6902	CE	LYS	A	875	41.031	21.625	17.453	1.00	88.44	C
ATOM	6903	NZ	LYS	A	875	40.906	20.328	18.188	1.00	88.44	N
ATOM	6904	N	LYS	A	876	42.094	25.562	13.844	1.00	83.19	N
ATOM	6905	CA	LYS	A	876	43.375	26.094	14.250	1.00	83.19	C
ATOM	6906	C	LYS	A	876	43.500	26.156	15.766	1.00	83.19	C
ATOM	6907	CB	LYS	A	876	43.625	27.469	13.648	1.00	83.19	C
ATOM	6908	O	LYS	A	876	42.625	26.719	16.438	1.00	83.19	O

ATOM	6909	CG	LYS	A	876	45.062	27.938	13.672	1.00	83.19	C
ATOM	6910	CD	LYS	A	876	45.281	29.125	12.750	1.00	83.19	C
ATOM	6911	CE	LYS	A	876	46.750	29.562	12.758	1.00	83.19	C
ATOM	6912	NZ	LYS	A	876	47.000	30.688	11.805	1.00	83.19	N
ATOM	6913	N	GLU	A	877	44.531	25.406	16.234	1.00	83.81	N
ATOM	6914	CA	GLU	A	877	44.719	25.297	17.688	1.00	83.81	C
ATOM	6915	C	GLU	A	877	46.094	25.797	18.094	1.00	83.81	C
ATOM	6916	CB	GLU	A	877	44.531	23.859	18.141	1.00	83.81	C
ATOM	6917	O	GLU	A	877	47.094	25.469	17.453	1.00	83.81	O
ATOM	6918	CG	GLU	A	877	44.531	23.688	19.656	1.00	83.81	C
ATOM	6919	CD	GLU	A	877	44.219	22.266	20.109	1.00	83.81	C
ATOM	6920	OE1	GLU	A	877	44.344	21.969	21.312	1.00	83.81	O
ATOM	6921	OE2	GLU	A	877	43.875	21.438	19.234	1.00	83.81	O
ATOM	6922	N	ASN	A	878	46.250	26.734	19.078	1.00	75.06	N
ATOM	6923	CA	ASN	A	878	47.438	27.188	19.766	1.00	75.06	C
ATOM	6924	C	ASN	A	878	47.250	27.172	21.281	1.00	75.06	C
ATOM	6925	CB	ASN	A	878	47.812	28.594	19.312	1.00	75.06	C
ATOM	6926	O	ASN	A	878	46.688	28.094	21.859	1.00	75.06	O
ATOM	6927	CG	ASN	A	878	49.156	29.047	19.859	1.00	75.06	C
ATOM	6928	ND2	ASN	A	878	49.469	30.328	19.719	1.00	75.06	N
ATOM	6929	OD1	ASN	A	878	49.906	28.250	20.422	1.00	75.06	O
ATOM	6930	N	GLY	A	879	47.688	26.062	21.953	1.00	78.38	N
ATOM	6931	CA	GLY	A	879	47.500	25.938	23.391	1.00	78.38	C
ATOM	6932	C	GLY	A	879	46.031	25.781	23.766	1.00	78.38	C
ATOM	6933	O	GLY	A	879	45.344	24.859	23.297	1.00	78.38	O
ATOM	6934	N	LYS	A	880	45.406	26.641	24.672	1.00	77.25	N
ATOM	6935	CA	LYS	A	880	44.031	26.641	25.172	1.00	77.25	C
ATOM	6936	C	LYS	A	880	43.125	27.391	24.203	1.00	77.25	C
ATOM	6937	CB	LYS	A	880	43.969	27.281	26.547	1.00	77.25	C
ATOM	6938	O	LYS	A	880	41.906	27.406	24.391	1.00	77.25	O
ATOM	6939	CG	LYS	A	880	44.656	26.469	27.641	1.00	77.25	C
ATOM	6940	CD	LYS	A	880	44.469	27.094	29.016	1.00	77.25	C
ATOM	6941	CE	LYS	A	880	45.156	26.281	30.094	1.00	77.25	C
ATOM	6942	NZ	LYS	A	880	44.969	26.891	31.453	1.00	77.25	N
ATOM	6943	N	ARG	A	881	43.719	28.000	23.062	1.00	82.62	N
ATOM	6944	CA	ARG	A	881	43.000	28.797	22.078	1.00	82.62	C
ATOM	6945	C	ARG	A	881	42.594	27.953	20.875	1.00	82.62	C
ATOM	6946	CB	ARG	A	881	43.875	29.969	21.609	1.00	82.62	C
ATOM	6947	O	ARG	A	881	43.406	27.266	20.266	1.00	82.62	O
ATOM	6948	CG	ARG	A	881	44.344	30.875	22.734	1.00	82.62	C
ATOM	6949	CD	ARG	A	881	45.375	31.875	22.250	1.00	82.62	C
ATOM	6950	NE	ARG	A	881	45.906	32.688	23.359	1.00	82.62	N
ATOM	6951	NH1	ARG	A	881	47.875	31.516	23.531	1.00	82.62	N
ATOM	6952	NH2	ARG	A	881	47.469	33.281	24.938	1.00	82.62	N
ATOM	6953	CZ	ARG	A	881	47.094	32.500	23.938	1.00	82.62	C
ATOM	6954	N	LYS	A	882	41.219	27.891	20.719	1.00	87.69	N
ATOM	6955	CA	LYS	A	882	40.688	27.109	19.594	1.00	87.69	C
ATOM	6956	C	LYS	A	882	39.750	27.953	18.719	1.00	87.69	C
ATOM	6957	CB	LYS	A	882	39.938	25.875	20.109	1.00	87.69	C
ATOM	6958	O	LYS	A	882	38.906	28.688	19.234	1.00	87.69	O
ATOM	6959	CG	LYS	A	882	40.844	24.828	20.734	1.00	87.69	C
ATOM	6960	CD	LYS	A	882	40.062	23.609	21.203	1.00	87.69	C
ATOM	6961	CE	LYS	A	882	40.938	22.656	22.016	1.00	87.69	C
ATOM	6962	NZ	LYS	A	882	40.156	21.516	22.547	1.00	87.69	N
ATOM	6963	N	ALA	A	883	40.125	27.984	17.312	1.00	82.19	N
ATOM	6964	CA	ALA	A	883	39.188	28.547	16.328	1.00	82.19	C
ATOM	6965	C	ALA	A	883	38.688	27.469	15.383	1.00	82.19	C
ATOM	6966	CB	ALA	A	883	39.875	29.656	15.531	1.00	82.19	C
ATOM	6967	O	ALA	A	883	39.469	26.734	14.781	1.00	82.19	O
ATOM	6968	N	GLU	A	884	37.344	27.406	15.320	1.00	89.25	N
ATOM	6969	CA	GLU	A	884	36.719	26.344	14.516	1.00	89.25	C
ATOM	6970	C	GLU	A	884	35.719	26.922	13.508	1.00	89.25	C
ATOM	6971	CB	GLU	A	884	36.031	25.312	15.414	1.00	89.25	C
ATOM	6972	O	GLU	A	884	35.000	27.859	13.820	1.00	89.25	O

ATOM	6973	CG	GLU	A	884	36.969	24.609	16.375	1.00	89.25	C
ATOM	6974	CD	GLU	A	884	36.250	23.719	17.375	1.00	89.25	C
ATOM	6975	OE1	GLU	A	884	35.969	22.531	17.047	1.00	89.25	O
ATOM	6976	OE2	GLU	A	884	35.938	24.203	18.484	1.00	89.25	O
ATOM	6977	N	TYR	A	885	35.938	26.516	12.281	1.00	86.50	N
ATOM	6978	CA	TYR	A	885	34.969	26.797	11.227	1.00	86.50	C
ATOM	6979	C	TYR	A	885	34.375	25.500	10.664	1.00	86.50	C
ATOM	6980	CB	TYR	A	885	35.625	27.609	10.102	1.00	86.50	C
ATOM	6981	O	TYR	A	885	35.125	24.609	10.258	1.00	86.50	O
ATOM	6982	CG	TYR	A	885	34.719	27.797	8.891	1.00	86.50	C
ATOM	6983	CD1	TYR	A	885	35.000	27.125	7.699	1.00	86.50	C
ATOM	6984	CD2	TYR	A	885	33.625	28.625	8.938	1.00	86.50	C
ATOM	6985	CE1	TYR	A	885	34.188	27.281	6.582	1.00	86.50	C
ATOM	6986	CE2	TYR	A	885	32.812	28.797	7.824	1.00	86.50	C
ATOM	6987	OH	TYR	A	885	32.281	28.297	5.551	1.00	86.50	O
ATOM	6988	CZ	TYR	A	885	33.094	28.125	6.652	1.00	86.50	C
ATOM	6989	N	THR	A	886	33.000	25.406	10.758	1.00	85.12	N
ATOM	6990	CA	THR	A	886	32.312	24.203	10.258	1.00	85.12	C
ATOM	6991	C	THR	A	886	31.234	24.578	9.258	1.00	85.12	C
ATOM	6992	CB	THR	A	886	31.703	23.391	11.414	1.00	85.12	C
ATOM	6993	O	THR	A	886	30.438	25.484	9.508	1.00	85.12	O
ATOM	6994	CG2	THR	A	886	31.156	22.062	10.914	1.00	85.12	C
ATOM	6995	OG1	THR	A	886	32.719	23.141	12.398	1.00	85.12	O
ATOM	6996	N	LYS	A	887	31.375	24.078	7.992	1.00	84.75	N
ATOM	6997	CA	LYS	A	887	30.375	24.234	6.945	1.00	84.75	C
ATOM	6998	C	LYS	A	887	29.906	22.891	6.418	1.00	84.75	C
ATOM	6999	CB	LYS	A	887	30.922	25.094	5.797	1.00	84.75	C
ATOM	7000	O	LYS	A	887	30.719	22.031	6.090	1.00	84.75	O
ATOM	7001	CG	LYS	A	887	29.906	25.422	4.719	1.00	84.75	C
ATOM	7002	CD	LYS	A	887	30.500	26.297	3.631	1.00	84.75	C
ATOM	7003	CE	LYS	A	887	29.469	26.656	2.572	1.00	84.75	C
ATOM	7004	NZ	LYS	A	887	30.031	27.562	1.525	1.00	84.75	N
ATOM	7005	N	SER	A	888	28.594	22.719	6.480	1.00	83.50	N
ATOM	7006	CA	SER	A	888	28.031	21.453	6.008	1.00	83.50	C
ATOM	7007	C	SER	A	888	26.797	21.688	5.156	1.00	83.50	C
ATOM	7008	CB	SER	A	888	27.688	20.547	7.184	1.00	83.50	C
ATOM	7009	O	SER	A	888	26.000	22.578	5.441	1.00	83.50	O
ATOM	7010	OG	SER	A	888	27.078	19.344	6.734	1.00	83.50	O
ATOM	7011	N	LEU	A	889	26.734	21.078	3.963	1.00	81.31	N
ATOM	7012	CA	LEU	A	889	25.562	21.047	3.084	1.00	81.31	C
ATOM	7013	C	LEU	A	889	25.062	19.609	2.910	1.00	81.31	C
ATOM	7014	CB	LEU	A	889	25.906	21.641	1.718	1.00	81.31	C
ATOM	7015	O	LEU	A	889	25.781	18.766	2.373	1.00	81.31	O
ATOM	7016	CG	LEU	A	889	24.719	21.906	0.780	1.00	81.31	C
ATOM	7017	CD1	LEU	A	889	23.875	23.062	1.292	1.00	81.31	C
ATOM	7018	CD2	LEU	A	889	25.219	22.172	-0.638	1.00	81.31	C
ATOM	7019	N	LYS	A	890	23.906	19.359	3.525	1.00	78.81	N
ATOM	7020	CA	LYS	A	890	23.312	18.031	3.434	1.00	78.81	C
ATOM	7021	C	LYS	A	890	22.078	18.047	2.535	1.00	78.81	C
ATOM	7022	CB	LYS	A	890	22.938	17.516	4.824	1.00	78.81	C
ATOM	7023	O	LYS	A	890	21.141	18.812	2.768	1.00	78.81	O
ATOM	7024	CG	LYS	A	890	22.469	16.078	4.840	1.00	78.81	C
ATOM	7025	CD	LYS	A	890	22.172	15.594	6.258	1.00	78.81	C
ATOM	7026	CE	LYS	A	890	21.703	14.148	6.273	1.00	78.81	C
ATOM	7027	NZ	LYS	A	890	21.391	13.680	7.656	1.00	78.81	N
ATOM	7028	N	LEU	A	891	22.219	17.297	1.433	1.00	75.56	N
ATOM	7029	CA	LEU	A	891	21.141	17.156	0.461	1.00	75.56	C
ATOM	7030	C	LEU	A	891	20.500	15.766	0.557	1.00	75.56	C
ATOM	7031	CB	LEU	A	891	21.672	17.391	-0.958	1.00	75.56	C
ATOM	7032	O	LEU	A	891	21.188	14.750	0.402	1.00	75.56	O
ATOM	7033	CG	LEU	A	891	22.328	18.734	-1.222	1.00	75.56	C
ATOM	7034	CD1	LEU	A	891	23.375	18.609	-2.322	1.00	75.56	C
ATOM	7035	CD2	LEU	A	891	21.281	19.781	-1.593	1.00	75.56	C
ATOM	7036	N	ILE	A	892	19.328	15.766	1.116	1.00	71.25	N

ATOM	7037	CA	ILE	A	892	18.578	14.508	1.220	1.00	71.25	C
ATOM	7038	C	ILE	A	892	17.422	14.516	0.238	1.00	71.25	C
ATOM	7039	CB	ILE	A	892	18.078	14.273	2.658	1.00	71.25	C
ATOM	7040	O	ILE	A	892	16.578	15.430	0.257	1.00	71.25	O
ATOM	7041	CG1	ILE	A	892	19.250	14.195	3.637	1.00	71.25	C
ATOM	7042	CG2	ILE	A	892	17.219	13.000	2.727	1.00	71.25	C
ATOM	7043	CD1	ILE	A	892	18.844	14.070	5.098	1.00	71.25	C
ATOM	7044	N	ALA	A	893	17.594	13.664	-0.751	1.00	66.56	N
ATOM	7045	CA	ALA	A	893	16.500	13.445	-1.690	1.00	66.56	C
ATOM	7046	C	ALA	A	893	16.125	11.961	-1.762	1.00	66.56	C
ATOM	7047	CB	ALA	A	893	16.875	13.961	-3.076	1.00	66.56	C
ATOM	7048	O	ALA	A	893	16.828	11.172	-2.408	1.00	66.56	O
ATOM	7049	N	ASP	A	894	15.188	11.742	-0.846	1.00	67.00	N
ATOM	7050	CA	ASP	A	894	14.750	10.352	-0.840	1.00	67.00	C
ATOM	7051	C	ASP	A	894	13.266	10.242	-1.162	1.00	67.00	C
ATOM	7052	CB	ASP	A	894	15.047	9.703	0.514	1.00	67.00	C
ATOM	7053	O	ASP	A	894	12.648	11.203	-1.631	1.00	67.00	O
ATOM	7054	CG	ASP	A	894	14.312	10.375	1.664	1.00	67.00	C
ATOM	7055	OD1	ASP	A	894	13.219	10.938	1.443	1.00	67.00	O
ATOM	7056	OD2	ASP	A	894	14.828	10.336	2.801	1.00	67.00	O
ATOM	7057	N	GLU	A	895	12.695	8.961	-1.047	1.00	58.50	N
ATOM	7058	CA	GLU	A	895	11.344	8.672	-1.520	1.00	58.50	C
ATOM	7059	C	GLU	A	895	10.305	9.523	-0.789	1.00	58.50	C
ATOM	7060	CB	GLU	A	895	11.023	7.184	-1.346	1.00	58.50	C
ATOM	7061	O	GLU	A	895	9.203	9.734	-1.293	1.00	58.50	O
ATOM	7062	CG	GLU	A	895	11.938	6.262	-2.145	1.00	58.50	C
ATOM	7063	CD	GLU	A	895	11.664	4.789	-1.887	1.00	58.50	C
ATOM	7064	OE1	GLU	A	895	12.336	3.932	-2.512	1.00	58.50	O
ATOM	7065	OE2	GLU	A	895	10.781	4.484	-1.054	1.00	58.50	O
ATOM	7066	N	THR	A	896	10.641	10.195	0.348	1.00	54.75	N
ATOM	7067	CA	THR	A	896	9.625	10.867	1.153	1.00	54.75	C
ATOM	7068	C	THR	A	896	9.844	12.375	1.140	1.00	54.75	C
ATOM	7069	CB	THR	A	896	9.641	10.359	2.605	1.00	54.75	C
ATOM	7070	O	THR	A	896	8.891	13.148	1.323	1.00	54.75	O
ATOM	7071	CG2	THR	A	896	9.266	8.883	2.674	1.00	54.75	C
ATOM	7072	OG1	THR	A	896	10.945	10.539	3.158	1.00	54.75	O
ATOM	7073	N	GLN	A	897	11.141	12.797	0.837	1.00	69.19	N
ATOM	7074	CA	GLN	A	897	11.398	14.211	1.063	1.00	69.19	C
ATOM	7075	C	GLN	A	897	12.602	14.688	0.253	1.00	69.19	C
ATOM	7076	CB	GLN	A	897	11.625	14.484	2.551	1.00	69.19	C
ATOM	7077	O	GLN	A	897	13.438	13.883	-0.158	1.00	69.19	O
ATOM	7078	CG	GLN	A	897	12.883	13.836	3.109	1.00	69.19	C
ATOM	7079	CD	GLN	A	897	13.070	14.094	4.594	1.00	69.19	C
ATOM	7080	NE2	GLN	A	897	14.000	13.375	5.211	1.00	69.19	N
ATOM	7081	OE1	GLN	A	897	12.383	14.938	5.180	1.00	69.19	O
ATOM	7082	N	ALA	A	898	12.492	15.836	-0.122	1.00	72.38	N
ATOM	7083	CA	ALA	A	898	13.648	16.609	-0.554	1.00	72.38	C
ATOM	7084	C	ALA	A	898	14.023	17.672	0.489	1.00	72.38	C
ATOM	7085	CB	ALA	A	898	13.367	17.281	-1.899	1.00	72.38	C
ATOM	7086	O	ALA	A	898	13.203	18.516	0.840	1.00	72.38	O
ATOM	7087	N	ARG	A	899	15.117	17.344	1.144	1.00	81.38	N
ATOM	7088	CA	ARG	A	899	15.547	18.188	2.256	1.00	81.38	C
ATOM	7089	C	ARG	A	899	16.969	18.719	2.027	1.00	81.38	C
ATOM	7090	CB	ARG	A	899	15.484	17.422	3.572	1.00	81.38	C
ATOM	7091	O	ARG	A	899	17.859	17.969	1.624	1.00	81.38	O
ATOM	7092	CG	ARG	A	899	15.906	18.234	4.785	1.00	81.38	C
ATOM	7093	CD	ARG	A	899	16.078	17.359	6.020	1.00	81.38	C
ATOM	7094	NE	ARG	A	899	14.812	16.766	6.441	1.00	81.38	N
ATOM	7095	NH1	ARG	A	899	14.406	18.312	8.102	1.00	81.38	N
ATOM	7096	NH2	ARG	A	899	12.914	16.625	7.727	1.00	81.38	N
ATOM	7097	CZ	ARG	A	899	14.047	17.234	7.422	1.00	81.38	C
ATOM	7098	N	VAL	A	900	17.109	20.000	2.090	1.00	82.00	N
ATOM	7099	CA	VAL	A	900	18.422	20.641	2.061	1.00	82.00	C
ATOM	7100	C	VAL	A	900	18.703	21.328	3.402	1.00	82.00	C

ATOM	7101	CB	VAL	A	900	18.531	21.672	0.915	1.00	82.00	C
ATOM	7102	O	VAL	A	900	17.891	22.109	3.885	1.00	82.00	O
ATOM	7103	CG1	VAL	A	900	19.906	22.328	0.886	1.00	82.00	C
ATOM	7104	CG2	VAL	A	900	18.203	21.016	-0.426	1.00	82.00	C
ATOM	7105	N	THR	A	901	19.734	20.828	4.012	1.00	82.00	N
ATOM	7106	CA	THR	A	901	20.172	21.453	5.258	1.00	82.00	C
ATOM	7107	C	THR	A	901	21.562	22.062	5.098	1.00	82.00	C
ATOM	7108	CB	THR	A	901	20.188	20.438	6.418	1.00	82.00	C
ATOM	7109	O	THR	A	901	22.500	21.375	4.688	1.00	82.00	O
ATOM	7110	CG2	THR	A	901	20.469	21.125	7.746	1.00	82.00	C
ATOM	7111	OG1	THR	A	901	18.906	19.797	6.500	1.00	82.00	O
ATOM	7112	N	ALA	A	902	21.688	23.359	5.188	1.00	83.94	N
ATOM	7113	CA	ALA	A	902	22.953	24.094	5.148	1.00	83.94	C
ATOM	7114	C	ALA	A	902	23.328	24.641	6.527	1.00	83.94	C
ATOM	7115	CB	ALA	A	902	22.891	25.234	4.129	1.00	83.94	C
ATOM	7116	O	ALA	A	902	22.500	25.297	7.176	1.00	83.94	O
ATOM	7117	N	ILE	A	903	24.516	24.281	6.922	1.00	84.25	N
ATOM	7118	CA	ILE	A	903	25.016	24.734	8.227	1.00	84.25	C
ATOM	7119	C	ILE	A	903	26.344	25.438	8.062	1.00	84.25	C
ATOM	7120	CB	ILE	A	903	25.141	23.547	9.203	1.00	84.25	C
ATOM	7121	O	ILE	A	903	27.234	24.969	7.336	1.00	84.25	O
ATOM	7122	CG1	ILE	A	903	23.797	22.859	9.422	1.00	84.25	C
ATOM	7123	CG2	ILE	A	903	25.734	24.016	10.539	1.00	84.25	C
ATOM	7124	CD1	ILE	A	903	23.875	21.484	10.055	1.00	84.25	C
ATOM	7125	N	GLU	A	904	26.500	26.594	8.719	1.00	87.00	N
ATOM	7126	CA	GLU	A	904	27.750	27.328	8.875	1.00	87.00	C
ATOM	7127	C	GLU	A	904	27.969	27.750	10.320	1.00	87.00	C
ATOM	7128	CB	GLU	A	904	27.766	28.562	7.961	1.00	87.00	C
ATOM	7129	O	GLU	A	904	27.094	28.359	10.938	1.00	87.00	O
ATOM	7130	CG	GLU	A	904	29.156	29.172	7.777	1.00	87.00	C
ATOM	7131	CD	GLU	A	904	29.203	30.250	6.703	1.00	87.00	C
ATOM	7132	OE1	GLU	A	904	30.312	30.703	6.336	1.00	87.00	O
ATOM	7133	OE2	GLU	A	904	28.109	30.641	6.227	1.00	87.00	O
ATOM	7134	N	GLN	A	905	29.016	27.266	10.914	1.00	89.06	N
ATOM	7135	CA	GLN	A	905	29.281	27.562	12.320	1.00	89.06	C
ATOM	7136	C	GLN	A	905	30.703	28.078	12.500	1.00	89.06	C
ATOM	7137	CB	GLN	A	905	29.062	26.328	13.188	1.00	89.06	C
ATOM	7138	O	GLN	A	905	31.656	27.516	11.945	1.00	89.06	O
ATOM	7139	CG	GLN	A	905	29.203	26.594	14.680	1.00	89.06	C
ATOM	7140	CD	GLN	A	905	28.984	25.344	15.523	1.00	89.06	C
ATOM	7141	NE2	GLN	A	905	28.266	25.500	16.641	1.00	89.06	N
ATOM	7142	OE1	GLN	A	905	29.453	24.266	15.180	1.00	89.06	O
ATOM	7143	N	LEU	A	906	30.922	29.188	13.203	1.00	85.31	N
ATOM	7144	CA	LEU	A	906	32.188	29.734	13.680	1.00	85.31	C
ATOM	7145	C	LEU	A	906	32.281	29.609	15.203	1.00	85.31	C
ATOM	7146	CB	LEU	A	906	32.344	31.188	13.258	1.00	85.31	C
ATOM	7147	O	LEU	A	906	31.375	30.016	15.922	1.00	85.31	O
ATOM	7148	CG	LEU	A	906	32.188	31.484	11.766	1.00	85.31	C
ATOM	7149	CD1	LEU	A	906	32.031	33.000	11.531	1.00	85.31	C
ATOM	7150	CD2	LEU	A	906	33.375	30.938	10.992	1.00	85.31	C
ATOM	7151	N	LYS	A	907	33.281	28.938	15.609	1.00	87.62	N
ATOM	7152	CA	LYS	A	907	33.500	28.797	17.047	1.00	87.62	C
ATOM	7153	C	LYS	A	907	34.938	29.219	17.422	1.00	87.62	C
ATOM	7154	CB	LYS	A	907	33.250	27.359	17.484	1.00	87.62	C
ATOM	7155	O	LYS	A	907	35.875	28.875	16.734	1.00	87.62	O
ATOM	7156	CG	LYS	A	907	33.188	27.172	18.984	1.00	87.62	C
ATOM	7157	CD	LYS	A	907	32.812	25.734	19.359	1.00	87.62	C
ATOM	7158	CE	LYS	A	907	32.812	25.547	20.875	1.00	87.62	C
ATOM	7159	NZ	LYS	A	907	32.438	24.141	21.250	1.00	87.62	N
ATOM	7160	N	ALA	A	908	35.000	30.031	18.406	1.00	82.88	N
ATOM	7161	CA	ALA	A	908	36.281	30.438	19.000	1.00	82.88	C
ATOM	7162	C	ALA	A	908	36.250	30.188	20.516	1.00	82.88	C
ATOM	7163	CB	ALA	A	908	36.562	31.906	18.703	1.00	82.88	C
ATOM	7164	O	ALA	A	908	35.312	30.562	21.203	1.00	82.88	O

ATOM	7165	N	SER	A	909	37.312	29.469	21.000	1.00	85.50	N
ATOM	7166	CA	SER	A	909	37.312	29.156	22.422	1.00	85.50	C
ATOM	7167	C	SER	A	909	38.719	29.297	23.016	1.00	85.50	C
ATOM	7168	CB	SER	A	909	36.812	27.734	22.656	1.00	85.50	C
ATOM	7169	O	SER	A	909	39.719	29.172	22.297	1.00	85.50	O
ATOM	7170	OG	SER	A	909	37.625	26.781	22.000	1.00	85.50	O
ATOM	7171	N	PHE	A	910	38.906	29.703	24.266	1.00	75.00	N
ATOM	7172	CA	PHE	A	910	40.094	29.672	25.141	1.00	75.00	C
ATOM	7173	C	PHE	A	910	39.875	28.703	26.297	1.00	75.00	C
ATOM	7174	CB	PHE	A	910	40.406	31.062	25.672	1.00	75.00	C
ATOM	7175	O	PHE	A	910	39.125	29.016	27.234	1.00	75.00	O
ATOM	7176	CG	PHE	A	910	41.750	31.172	26.328	1.00	75.00	C
ATOM	7177	CD1	PHE	A	910	41.906	31.016	27.703	1.00	75.00	C
ATOM	7178	CD2	PHE	A	910	42.906	31.406	25.562	1.00	75.00	C
ATOM	7179	CE1	PHE	A	910	43.156	31.109	28.297	1.00	75.00	C
ATOM	7180	CE2	PHE	A	910	44.156	31.500	26.156	1.00	75.00	C
ATOM	7181	CZ	PHE	A	910	44.281	31.359	27.531	1.00	75.00	C
ATOM	7182	N	GLY	A	911	40.375	27.438	26.125	1.00	69.12	N
ATOM	7183	CA	GLY	A	911	40.000	26.469	27.141	1.00	69.12	C
ATOM	7184	C	GLY	A	911	38.500	26.172	27.141	1.00	69.12	C
ATOM	7185	O	GLY	A	911	37.812	26.453	26.172	1.00	69.12	O
ATOM	7186	N	ASP	A	912	37.906	25.391	28.016	1.00	70.62	N
ATOM	7187	CA	ASP	A	912	36.500	24.984	28.062	1.00	70.62	C
ATOM	7188	C	ASP	A	912	35.625	26.062	28.719	1.00	70.62	C
ATOM	7189	CB	ASP	A	912	36.344	23.656	28.797	1.00	70.62	C
ATOM	7190	O	ASP	A	912	34.406	26.047	28.594	1.00	70.62	O
ATOM	7191	CG	ASP	A	912	37.000	22.484	28.062	1.00	70.62	C
ATOM	7192	OD1	ASP	A	912	37.219	22.594	26.844	1.00	70.62	O
ATOM	7193	OD2	ASP	A	912	37.250	21.453	28.719	1.00	70.62	O
ATOM	7194	N	GLN	A	913	36.188	27.109	29.172	1.00	62.03	N
ATOM	7195	CA	GLN	A	913	35.500	28.031	30.062	1.00	62.03	C
ATOM	7196	C	GLN	A	913	35.062	29.297	29.312	1.00	62.03	C
ATOM	7197	CB	GLN	A	913	36.375	28.406	31.250	1.00	62.03	C
ATOM	7198	O	GLN	A	913	34.094	29.938	29.688	1.00	62.03	O
ATOM	7199	CG	GLN	A	913	36.625	27.234	32.219	1.00	62.03	C
ATOM	7200	CD	GLN	A	913	37.406	27.656	33.438	1.00	62.03	C
ATOM	7201	NE2	GLN	A	913	36.938	27.266	34.625	1.00	62.03	N
ATOM	7202	OE1	GLN	A	913	38.469	28.312	33.344	1.00	62.03	O
ATOM	7203	N	ILE	A	914	35.781	29.734	28.156	1.00	77.44	N
ATOM	7204	CA	ILE	A	914	35.500	30.922	27.359	1.00	77.44	C
ATOM	7205	C	ILE	A	914	35.219	30.531	25.906	1.00	77.44	C
ATOM	7206	CB	ILE	A	914	36.656	31.953	27.438	1.00	77.44	C
ATOM	7207	O	ILE	A	914	36.125	30.016	25.234	1.00	77.44	O
ATOM	7208	CG1	ILE	A	914	36.906	32.344	28.891	1.00	77.44	C
ATOM	7209	CG2	ILE	A	914	36.312	33.188	26.594	1.00	77.44	C
ATOM	7210	CD1	ILE	A	914	38.125	33.250	29.078	1.00	77.44	C
ATOM	7211	N	SER	A	915	33.875	30.703	25.484	1.00	83.81	N
ATOM	7212	CA	SER	A	915	33.562	30.266	24.141	1.00	83.81	C
ATOM	7213	C	SER	A	915	32.562	31.234	23.484	1.00	83.81	C
ATOM	7214	CB	SER	A	915	32.969	28.859	24.141	1.00	83.81	C
ATOM	7215	O	SER	A	915	31.828	31.938	24.172	1.00	83.81	O
ATOM	7216	OG	SER	A	915	31.750	28.828	24.875	1.00	83.81	O
ATOM	7217	N	ALA	A	916	32.781	31.469	22.156	1.00	80.81	N
ATOM	7218	CA	ALA	A	916	31.844	32.188	21.297	1.00	80.81	C
ATOM	7219	C	ALA	A	916	31.469	31.359	20.078	1.00	80.81	C
ATOM	7220	CB	ALA	A	916	32.438	33.531	20.859	1.00	80.81	C
ATOM	7221	O	ALA	A	916	32.312	30.672	19.500	1.00	80.81	O
ATOM	7222	N	SER	A	917	30.266	31.391	19.859	1.00	86.12	N
ATOM	7223	CA	SER	A	917	29.812	30.625	18.703	1.00	86.12	C
ATOM	7224	C	SER	A	917	28.797	31.391	17.875	1.00	86.12	C
ATOM	7225	CB	SER	A	917	29.219	29.281	19.141	1.00	86.12	C
ATOM	7226	O	SER	A	917	28.047	32.219	18.422	1.00	86.12	O
ATOM	7227	OG	SER	A	917	28.016	29.484	19.875	1.00	86.12	O
ATOM	7228	N	ASN	A	918	28.906	31.375	16.562	1.00	84.06	N

ATOM	7229	CA	ASN	A	918	27.953	31.859	15.578	1.00	84.06	C
ATOM	7230	C	ASN	A	918	27.484	30.750	14.641	1.00	84.06	C
ATOM	7231	CB	ASN	A	918	28.547	33.000	14.773	1.00	84.06	C
ATOM	7232	O	ASN	A	918	28.281	30.188	13.883	1.00	84.06	O
ATOM	7233	CG	ASN	A	918	27.516	33.688	13.891	1.00	84.06	C
ATOM	7234	ND2	ASN	A	918	27.922	34.781	13.250	1.00	84.06	N
ATOM	7235	OD1	ASN	A	918	26.375	33.250	13.789	1.00	84.06	O
ATOM	7236	N	SER	A	919	26.219	30.406	14.820	1.00	85.25	N
ATOM	7237	CA	SER	A	919	25.703	29.297	14.039	1.00	85.25	C
ATOM	7238	C	SER	A	919	24.516	29.719	13.180	1.00	85.25	C
ATOM	7239	CB	SER	A	919	25.297	28.141	14.953	1.00	85.25	C
ATOM	7240	O	SER	A	919	23.656	30.469	13.641	1.00	85.25	O
ATOM	7241	OG	SER	A	919	26.391	27.688	15.719	1.00	85.25	O
ATOM	7242	N	GLU	A	920	24.609	29.500	11.852	1.00	87.81	N
ATOM	7243	CA	GLU	A	920	23.500	29.672	10.922	1.00	87.81	C
ATOM	7244	C	GLU	A	920	23.031	28.328	10.359	1.00	87.81	C
ATOM	7245	CB	GLU	A	920	23.906	30.609	9.773	1.00	87.81	C
ATOM	7246	O	GLU	A	920	23.844	27.562	9.836	1.00	87.81	O
ATOM	7247	CG	GLU	A	920	22.750	31.000	8.875	1.00	87.81	C
ATOM	7248	CD	GLU	A	920	23.156	31.953	7.754	1.00	87.81	C
ATOM	7249	OE1	GLU	A	920	22.266	32.438	7.016	1.00	87.81	O
ATOM	7250	OE2	GLU	A	920	24.375	32.219	7.617	1.00	87.81	O
ATOM	7251	N	LEU	A	921	21.750	28.047	10.555	1.00	85.44	N
ATOM	7252	CA	LEU	A	921	21.125	26.828	10.031	1.00	85.44	C
ATOM	7253	C	LEU	A	921	19.984	27.172	9.078	1.00	85.44	C
ATOM	7254	CB	LEU	A	921	20.609	25.953	11.180	1.00	85.44	C
ATOM	7255	O	LEU	A	921	19.094	27.938	9.430	1.00	85.44	O
ATOM	7256	CG	LEU	A	921	20.016	24.609	10.789	1.00	85.44	C
ATOM	7257	CD1	LEU	A	921	20.531	23.516	11.727	1.00	85.44	C
ATOM	7258	CD2	LEU	A	921	18.500	24.656	10.797	1.00	85.44	C
ATOM	7259	N	ARG	A	922	20.109	26.750	7.785	1.00	84.19	N
ATOM	7260	CA	ARG	A	922	19.062	26.891	6.785	1.00	84.19	C
ATOM	7261	C	ARG	A	922	18.578	25.531	6.305	1.00	84.19	C
ATOM	7262	CB	ARG	A	922	19.562	27.719	5.598	1.00	84.19	C
ATOM	7263	O	ARG	A	922	19.391	24.656	5.965	1.00	84.19	O
ATOM	7264	CG	ARG	A	922	19.922	29.156	5.957	1.00	84.19	C
ATOM	7265	CD	ARG	A	922	20.469	29.906	4.754	1.00	84.19	C
ATOM	7266	NE	ARG	A	922	21.000	31.219	5.137	1.00	84.19	N
ATOM	7267	NH1	ARG	A	922	19.703	32.406	3.668	1.00	84.19	N
ATOM	7268	NH2	ARG	A	922	21.188	33.500	5.035	1.00	84.19	N
ATOM	7269	CZ	ARG	A	922	20.625	32.375	4.613	1.00	84.19	C
ATOM	7270	N	GLU	A	923	17.266	25.438	6.441	1.00	83.75	N
ATOM	7271	CA	GLU	A	923	16.656	24.172	6.055	1.00	83.75	C
ATOM	7272	C	GLU	A	923	15.438	24.375	5.164	1.00	83.75	C
ATOM	7273	CB	GLU	A	923	16.281	23.344	7.293	1.00	83.75	C
ATOM	7274	O	GLU	A	923	14.594	25.219	5.461	1.00	83.75	O
ATOM	7275	CG	GLU	A	923	15.828	21.938	6.980	1.00	83.75	C
ATOM	7276	CD	GLU	A	923	15.531	21.109	8.227	1.00	83.75	C
ATOM	7277	OE1	GLU	A	923	15.617	19.859	8.164	1.00	83.75	O
ATOM	7278	OE2	GLU	A	923	15.211	21.719	9.266	1.00	83.75	O
ATOM	7279	N	VAL	A	924	15.430	23.625	4.059	1.00	77.12	N
ATOM	7280	CA	VAL	A	924	14.273	23.609	3.168	1.00	77.12	C
ATOM	7281	C	VAL	A	924	13.781	22.172	2.980	1.00	77.12	C
ATOM	7282	CB	VAL	A	924	14.602	24.234	1.798	1.00	77.12	C
ATOM	7283	O	VAL	A	924	14.570	21.281	2.672	1.00	77.12	O
ATOM	7284	CG1	VAL	A	924	13.367	24.234	0.891	1.00	77.12	C
ATOM	7285	CG2	VAL	A	924	15.117	25.672	1.982	1.00	77.12	C
ATOM	7286	N	ILE	A	925	12.570	22.016	3.289	1.00	79.50	N
ATOM	7287	CA	ILE	A	925	12.016	20.672	3.250	1.00	79.50	C
ATOM	7288	C	ILE	A	925	10.773	20.656	2.357	1.00	79.50	C
ATOM	7289	CB	ILE	A	925	11.664	20.172	4.668	1.00	79.50	C
ATOM	7290	O	ILE	A	925	9.875	21.484	2.514	1.00	79.50	O
ATOM	7291	CG1	ILE	A	925	12.906	20.188	5.562	1.00	79.50	C
ATOM	7292	CG2	ILE	A	925	11.055	18.766	4.602	1.00	79.50	C

```
ATOM   7293  CD1 ILE A 925      12.609  19.969   7.039  1.00 79.50           C
ATOM   7294  N   ALA A 926      10.812  19.828   1.295  1.00 69.94           N
ATOM   7295  CA  ALA A 926       9.672  19.531   0.435  1.00 69.94           C
ATOM   7296  C   ALA A 926       9.242  18.078   0.561  1.00 69.94           C
ATOM   7297  CB  ALA A 926      10.000  19.859  -1.019  1.00 69.94           C
ATOM   7298  O   ALA A 926      10.055  17.172   0.365  1.00 69.94           O
ATOM   7299  N   THR A 927       8.164  17.875   1.111  1.00 61.34           N
ATOM   7300  CA  THR A 927       7.719  16.531   1.440  1.00 61.34           C
ATOM   7301  C   THR A 927       6.496  16.156   0.613  1.00 61.34           C
ATOM   7302  CB  THR A 927       7.391  16.406   2.939  1.00 61.34           C
ATOM   7303  O   THR A 927       5.617  16.984   0.375  1.00 61.34           O
ATOM   7304  CG2 THR A 927       8.273  15.359   3.609  1.00 61.34           C
ATOM   7305  OG1 THR A 927       7.594  17.672   3.578  1.00 61.34           O
ATOM   7306  N   GLU A 928       6.516  14.992  -0.162  1.00 55.56           N
ATOM   7307  CA  GLU A 928       5.445  14.398  -0.956  1.00 55.56           C
ATOM   7308  C   GLU A 928       4.711  13.320  -0.168  1.00 55.56           C
ATOM   7309  CB  GLU A 928       6.000  13.820  -2.258  1.00 55.56           C
ATOM   7310  O   GLU A 928       5.336  12.391   0.354  1.00 55.56           O
ATOM   7311  CG  GLU A 928       5.109  14.055  -3.467  1.00 55.56           C
ATOM   7312  CD  GLU A 928       5.703  13.531  -4.766  1.00 55.56           C
ATOM   7313  OE1 GLU A 928       5.062  13.688  -5.832  1.00 55.56           O
ATOM   7314  OE2 GLU A 928       6.816  12.969  -4.715  1.00 55.56           O
ATOM   7315  N   THR A 929       3.609  13.625   0.590  1.00 56.88           N
ATOM   7316  CA  THR A 929       2.973  12.789   1.604  1.00 56.88           C
ATOM   7317  C   THR A 929       1.890  11.914   0.982  1.00 56.88           C
ATOM   7318  CB  THR A 929       2.357  13.648   2.727  1.00 56.88           C
ATOM   7319  O   THR A 929       1.719  10.758   1.377  1.00 56.88           O
ATOM   7320  CG2 THR A 929       2.336  12.891   4.051  1.00 56.88           C
ATOM   7321  OG1 THR A 929       3.133  14.844   2.885  1.00 56.88           O
ATOM   7322  N   GLU A 930       1.584  11.844  -0.412  1.00 46.00           N
ATOM   7323  CA  GLU A 930       0.484  10.891  -0.533  1.00 46.00           C
ATOM   7324  C   GLU A 930       0.840   9.750  -1.487  1.00 46.00           C
ATOM   7325  CB  GLU A 930      -0.789  11.594  -1.009  1.00 46.00           C
ATOM   7326  O   GLU A 930       0.467   8.602  -1.256  1.00 46.00           O
ATOM   7327  CG  GLU A 930      -1.674  12.094   0.124  1.00 46.00           C
ATOM   7328  CD  GLU A 930      -2.912  12.828  -0.363  1.00 46.00           C
ATOM   7329  OE1 GLU A 930      -3.717  13.289   0.480  1.00 46.00           O
ATOM   7330  OE2 GLU A 930      -3.078  12.953  -1.597  1.00 46.00           O
ATOM   7331  N   ALA A 931       2.109   9.477  -1.903  1.00 47.47           N
ATOM   7332  CA  ALA A 931       2.336   8.258  -2.668  1.00 47.47           C
ATOM   7333  C   ALA A 931       3.381   7.371  -1.995  1.00 47.47           C
ATOM   7334  CB  ALA A 931       2.766   8.594  -4.094  1.00 47.47           C
ATOM   7335  O   ALA A 931       3.230   6.148  -1.946  1.00 47.47           O
ATOM   7336  N   LEU A 932       4.094   7.707  -0.948  1.00 44.66           N
ATOM   7337  CA  LEU A 932       5.215   7.027  -0.306  1.00 44.66           C
ATOM   7338  C   LEU A 932       4.863   6.637   1.127  1.00 44.66           C
ATOM   7339  CB  LEU A 932       6.457   7.918  -0.316  1.00 44.66           C
ATOM   7340  O   LEU A 932       5.320   5.605   1.624  1.00 44.66           O
ATOM   7341  CG  LEU A 932       7.789   7.227  -0.018  1.00 44.66           C
ATOM   7342  CD1 LEU A 932       8.211   6.359  -1.199  1.00 44.66           C
ATOM   7343  CD2 LEU A 932       8.867   8.258   0.308  1.00 44.66           C
ATOM   7344  N   SER A 933       3.908   7.480   1.955  1.00 46.75           N
ATOM   7345  CA  SER A 933       3.412   7.090   3.271  1.00 46.75           C
ATOM   7346  C   SER A 933       2.701   5.742   3.215  1.00 46.75           C
ATOM   7347  CB  SER A 933       2.463   8.156   3.824  1.00 46.75           C
ATOM   7348  O   SER A 933       2.834   4.926   4.129  1.00 46.75           O
ATOM   7349  OG  SER A 933       2.674   8.344   5.215  1.00 46.75           O
ATOM   7350  N   ARG A 934       2.256   5.293   1.978  1.00 51.25           N
ATOM   7351  CA  ARG A 934       1.629   3.990   1.791  1.00 51.25           C
ATOM   7352  C   ARG A 934       2.678   2.904   1.574  1.00 51.25           C
ATOM   7353  CB  ARG A 934       0.658   4.023   0.609  1.00 51.25           C
ATOM   7354  O   ARG A 934       2.557   1.802   2.113  1.00 51.25           O
ATOM   7355  CG  ARG A 934      -0.709   4.594   0.950  1.00 51.25           C
ATOM   7356  CD  ARG A 934      -1.690   4.438  -0.203  1.00 51.25           C
```

ATOM	7357	NE	ARG	A	934	-2.766	5.422	-0.134	1.00	51.25	N
ATOM	7358	NH1	ARG	A	934	-2.889	5.754	-2.418	1.00	51.25	N
ATOM	7359	NH2	ARG	A	934	-4.281	6.906	-1.011	1.00	51.25	N
ATOM	7360	CZ	ARG	A	934	-3.311	6.027	-1.188	1.00	51.25	C
ATOM	7361	N	GLU	A	935	3.877	3.215	1.153	1.00	50.97	N
ATOM	7362	CA	GLU	A	935	4.906	2.215	0.887	1.00	50.97	C
ATOM	7363	C	GLU	A	935	5.801	2.006	2.105	1.00	50.97	C
ATOM	7364	CB	GLU	A	935	5.750	2.619	-0.324	1.00	50.97	C
ATOM	7365	O	GLU	A	935	6.180	0.875	2.418	1.00	50.97	O
ATOM	7366	CG	GLU	A	935	5.934	1.506	-1.345	1.00	50.97	C
ATOM	7367	CD	GLU	A	935	6.762	1.928	-2.549	1.00	50.97	C
ATOM	7368	OE1	GLU	A	935	6.969	1.097	-3.463	1.00	50.97	O
ATOM	7369	OE2	GLU	A	935	7.203	3.098	-2.578	1.00	50.97	O
ATOM	7370	N	ILE	A	936	6.078	3.072	3.006	1.00	49.94	N
ATOM	7371	CA	ILE	A	936	6.973	3.088	4.160	1.00	49.94	C
ATOM	7372	C	ILE	A	936	6.262	2.482	5.371	1.00	49.94	C
ATOM	7373	CB	ILE	A	936	7.453	4.520	4.484	1.00	49.94	C
ATOM	7374	O	ILE	A	936	6.863	1.726	6.137	1.00	49.94	O
ATOM	7375	CG1	ILE	A	936	8.461	4.996	3.432	1.00	49.94	C
ATOM	7376	CG2	ILE	A	936	8.055	4.582	5.891	1.00	49.94	C
ATOM	7377	CD1	ILE	A	936	8.883	6.449	3.588	1.00	49.94	C
ATOM	7378	N	ASP	A	937	4.941	2.680	5.539	1.00	53.75	N
ATOM	7379	CA	ASP	A	937	4.168	1.997	6.574	1.00	53.75	C
ATOM	7380	C	ASP	A	937	4.121	0.492	6.320	1.00	53.75	C
ATOM	7381	CB	ASP	A	937	2.748	2.562	6.645	1.00	53.75	C
ATOM	7382	O	ASP	A	937	4.188	-0.302	7.262	1.00	53.75	O
ATOM	7383	CG	ASP	A	937	2.686	3.924	7.312	1.00	53.75	C
ATOM	7384	OD1	ASP	A	937	3.633	4.289	8.039	1.00	53.75	O
ATOM	7385	OD2	ASP	A	937	1.678	4.637	7.113	1.00	53.75	O
ATOM	7386	N	GLN	A	938	4.336	0.059	5.117	1.00	53.75	N
ATOM	7387	CA	GLN	A	938	4.363	-1.352	4.742	1.00	53.75	C
ATOM	7388	C	GLN	A	938	5.727	-1.971	5.031	1.00	53.75	C
ATOM	7389	CB	GLN	A	938	4.012	-1.523	3.264	1.00	53.75	C
ATOM	7390	O	GLN	A	938	5.812	-3.098	5.523	1.00	53.75	O
ATOM	7391	CG	GLN	A	938	2.559	-1.897	3.014	1.00	53.75	C
ATOM	7392	CD	GLN	A	938	2.238	-2.061	1.540	1.00	53.75	C
ATOM	7393	NE2	GLN	A	938	1.018	-2.494	1.243	1.00	53.75	N
ATOM	7394	OE1	GLN	A	938	3.080	-1.798	0.675	1.00	53.75	O
ATOM	7395	N	LEU	A	939	6.777	-1.237	4.941	1.00	55.66	N
ATOM	7396	CA	LEU	A	939	8.125	-1.732	5.188	1.00	55.66	C
ATOM	7397	C	LEU	A	939	8.469	-1.669	6.672	1.00	55.66	C
ATOM	7398	CB	LEU	A	939	9.148	-0.926	4.383	1.00	55.66	C
ATOM	7399	O	LEU	A	939	9.133	-2.562	7.199	1.00	55.66	O
ATOM	7400	CG	LEU	A	939	9.906	-1.688	3.295	1.00	55.66	C
ATOM	7401	CD1	LEU	A	939	9.758	-0.988	1.949	1.00	55.66	C
ATOM	7402	CD2	LEU	A	939	11.383	-1.822	3.668	1.00	55.66	C
ATOM	7403	N	LYS	A	940	7.918	-0.583	7.480	1.00	53.84	N
ATOM	7404	CA	LYS	A	940	8.102	-0.390	8.914	1.00	53.84	C
ATOM	7405	C	LYS	A	940	7.418	-1.496	9.711	1.00	53.84	C
ATOM	7406	CB	LYS	A	940	7.570	0.976	9.344	1.00	53.84	C
ATOM	7407	O	LYS	A	940	7.934	-1.941	10.734	1.00	53.84	O
ATOM	7408	CG	LYS	A	940	8.586	2.104	9.227	1.00	53.84	C
ATOM	7409	CD	LYS	A	940	8.031	3.416	9.766	1.00	53.84	C
ATOM	7410	CE	LYS	A	940	9.016	4.559	9.586	1.00	53.84	C
ATOM	7411	NZ	LYS	A	940	8.500	5.836	10.164	1.00	53.84	N
ATOM	7412	N	ALA	A	941	6.418	-2.109	9.148	1.00	53.56	N
ATOM	7413	CA	ALA	A	941	5.766	-3.275	9.734	1.00	53.56	C
ATOM	7414	C	ALA	A	941	6.574	-4.543	9.484	1.00	53.56	C
ATOM	7415	CB	ALA	A	941	4.352	-3.432	9.180	1.00	53.56	C
ATOM	7416	O	ALA	A	941	6.684	-5.406	10.359	1.00	53.56	O
ATOM	7417	N	GLN	A	942	7.484	-4.512	8.555	1.00	56.41	N
ATOM	7418	CA	GLN	A	942	8.320	-5.668	8.266	1.00	56.41	C
ATOM	7419	C	GLN	A	942	9.617	-5.621	9.070	1.00	56.41	C
ATOM	7420	CB	GLN	A	942	8.641	-5.750	6.773	1.00	56.41	C

ATOM	7421	O	GLN	A	942	10.062	-6.641	9.602	1.00	56.41	O
ATOM	7422	CG	GLN	A	942	9.227	-7.086	6.340	1.00	56.41	C
ATOM	7423	CD	GLN	A	942	8.180	-8.070	5.863	1.00	56.41	C
ATOM	7424	NE2	GLN	A	942	8.492	-9.359	5.945	1.00	56.41	N
ATOM	7425	OE1	GLN	A	942	7.094	-7.672	5.426	1.00	56.41	O
ATOM	7426	N	ILE	A	943	10.148	-4.566	9.539	1.00	59.25	N
ATOM	7427	CA	ILE	A	943	11.469	-4.430	10.148	1.00	59.25	C
ATOM	7428	C	ILE	A	943	11.328	-4.262	11.656	1.00	59.25	C
ATOM	7429	CB	ILE	A	943	12.250	-3.238	9.547	1.00	59.25	C
ATOM	7430	O	ILE	A	943	12.211	-4.668	12.422	1.00	59.25	O
ATOM	7431	CG1	ILE	A	943	12.633	-3.529	8.094	1.00	59.25	C
ATOM	7432	CG2	ILE	A	943	13.484	-2.922	10.391	1.00	59.25	C
ATOM	7433	CD1	ILE	A	943	13.211	-2.330	7.355	1.00	59.25	C
ATOM	7434	N	GLY	A	944	10.133	-3.900	12.258	1.00	53.22	N
ATOM	7435	CA	GLY	A	944	9.898	-3.559	13.648	1.00	53.22	C
ATOM	7436	C	GLY	A	944	9.969	-4.754	14.578	1.00	53.22	C
ATOM	7437	O	GLY	A	944	10.492	-4.656	15.688	1.00	53.22	O
ATOM	7438	N	ASP	A	945	9.945	-5.980	14.117	1.00	55.34	N
ATOM	7439	CA	ASP	A	945	9.914	-7.086	15.070	1.00	55.34	C
ATOM	7440	C	ASP	A	945	11.305	-7.672	15.281	1.00	55.34	C
ATOM	7441	CB	ASP	A	945	8.953	-8.180	14.594	1.00	55.34	C
ATOM	7442	O	ASP	A	945	11.664	-8.039	16.406	1.00	55.34	O
ATOM	7443	CG	ASP	A	945	7.492	-7.797	14.766	1.00	55.34	C
ATOM	7444	OD1	ASP	A	945	7.195	-6.820	15.484	1.00	55.34	O
ATOM	7445	OD2	ASP	A	945	6.629	-8.484	14.172	1.00	55.34	O
ATOM	7446	N	ASP	A	946	12.250	-7.363	14.422	1.00	56.81	N
ATOM	7447	CA	ASP	A	946	13.602	-7.898	14.578	1.00	56.81	C
ATOM	7448	C	ASP	A	946	14.500	-6.910	15.320	1.00	56.81	C
ATOM	7449	CB	ASP	A	946	14.211	-8.242	13.219	1.00	56.81	C
ATOM	7450	O	ASP	A	946	15.320	-7.305	16.156	1.00	56.81	O
ATOM	7451	CG	ASP	A	946	13.594	-9.484	12.594	1.00	56.81	C
ATOM	7452	OD1	ASP	A	946	12.883	-10.234	13.297	1.00	56.81	O
ATOM	7453	OD2	ASP	A	946	13.828	-9.719	11.391	1.00	56.81	O
ATOM	7454	N	ILE	A	947	14.141	-5.582	15.320	1.00	59.41	N
ATOM	7455	CA	ILE	A	947	14.992	-4.562	15.930	1.00	59.41	C
ATOM	7456	C	ILE	A	947	14.586	-4.355	17.391	1.00	59.41	C
ATOM	7457	CB	ILE	A	947	14.914	-3.227	15.156	1.00	59.41	C
ATOM	7458	O	ILE	A	947	15.438	-4.191	18.266	1.00	59.41	O
ATOM	7459	CG1	ILE	A	947	15.602	-3.357	13.789	1.00	59.41	C
ATOM	7460	CG2	ILE	A	947	15.539	-2.092	15.977	1.00	59.41	C
ATOM	7461	CD1	ILE	A	947	15.445	-2.135	12.898	1.00	59.41	C
ATOM	7462	N	GLN	A	948	13.305	-4.676	17.812	1.00	56.25	N
ATOM	7463	CA	GLN	A	948	12.836	-4.457	19.172	1.00	56.25	C
ATOM	7464	C	GLN	A	948	13.344	-5.551	20.109	1.00	56.25	C
ATOM	7465	CB	GLN	A	948	11.305	-4.398	19.219	1.00	56.25	C
ATOM	7466	O	GLN	A	948	13.680	-5.277	21.266	1.00	56.25	O
ATOM	7467	CG	GLN	A	948	10.750	-2.982	19.266	1.00	56.25	C
ATOM	7468	CD	GLN	A	948	11.117	-2.246	20.531	1.00	56.25	C
ATOM	7469	NE2	GLN	A	948	11.281	-0.931	20.438	1.00	56.25	N
ATOM	7470	OE1	GLN	A	948	11.242	-2.854	21.594	1.00	56.25	O
ATOM	7471	N	ALA	A	949	13.758	-6.707	19.578	1.00	55.56	N
ATOM	7472	CA	ALA	A	949	14.297	-7.770	20.422	1.00	55.56	C
ATOM	7473	C	ALA	A	949	15.797	-7.598	20.641	1.00	55.56	C
ATOM	7474	CB	ALA	A	949	14.000	-9.141	19.812	1.00	55.56	C
ATOM	7475	O	ALA	A	949	16.312	-7.824	21.734	1.00	55.56	O
ATOM	7476	N	SER	A	950	16.469	-6.879	19.719	1.00	56.91	N
ATOM	7477	CA	SER	A	950	17.891	-6.625	19.891	1.00	56.91	C
ATOM	7478	C	SER	A	950	18.141	-5.328	20.656	1.00	56.91	C
ATOM	7479	CB	SER	A	950	18.594	-6.566	18.531	1.00	56.91	C
ATOM	7480	O	SER	A	950	19.094	-5.230	21.438	1.00	56.91	O
ATOM	7481	OG	SER	A	950	18.734	-7.863	17.984	1.00	56.91	O
ATOM	7482	N	LEU	A	951	17.141	-4.355	20.719	1.00	56.75	N
ATOM	7483	CA	LEU	A	951	17.297	-3.070	21.391	1.00	56.75	C
ATOM	7484	C	LEU	A	951	16.969	-3.193	22.891	1.00	56.75	C

ATOM	7485	CB	LEU	A	951	16.375	-2.018	20.750	1.00	56.75	C
ATOM	7486	O	LEU	A	951	17.578	-2.504	23.703	1.00	56.75	O
ATOM	7487	CG	LEU	A	951	17.062	-0.748	20.250	1.00	56.75	C
ATOM	7488	CD1	LEU	A	951	16.922	-0.636	18.734	1.00	56.75	C
ATOM	7489	CD2	LEU	A	951	16.484	0.484	20.938	1.00	56.75	C
ATOM	7490	N	THR	A	952	16.219	-4.234	23.297	1.00	57.78	N
ATOM	7491	CA	THR	A	952	15.891	-4.395	24.703	1.00	57.78	C
ATOM	7492	C	THR	A	952	17.078	-4.977	25.469	1.00	57.78	C
ATOM	7493	CB	THR	A	952	14.656	-5.297	24.906	1.00	57.78	C
ATOM	7494	O	THR	A	952	17.375	-4.547	26.594	1.00	57.78	O
ATOM	7495	CG2	THR	A	952	14.141	-5.223	26.328	1.00	57.78	C
ATOM	7496	OG1	THR	A	952	13.617	-4.871	24.016	1.00	57.78	O
ATOM	7497	N	ASP	A	953	17.969	-5.676	24.828	1.00	60.88	N
ATOM	7498	CA	ASP	A	953	19.125	-6.242	25.500	1.00	60.88	C
ATOM	7499	C	ASP	A	953	20.281	-5.234	25.562	1.00	60.88	C
ATOM	7500	CB	ASP	A	953	19.594	-7.516	24.797	1.00	60.88	C
ATOM	7501	O	ASP	A	953	20.953	-5.125	26.594	1.00	60.88	O
ATOM	7502	CG	ASP	A	953	18.766	-8.734	25.156	1.00	60.88	C
ATOM	7503	OD1	ASP	A	953	17.969	-8.672	26.125	1.00	60.88	O
ATOM	7504	OD2	ASP	A	953	18.891	-9.766	24.469	1.00	60.88	O
ATOM	7505	N	ILE	A	954	20.281	-4.246	24.562	1.00	63.25	N
ATOM	7506	CA	ILE	A	954	21.344	-3.248	24.547	1.00	63.25	C
ATOM	7507	C	ILE	A	954	21.000	-2.107	25.500	1.00	63.25	C
ATOM	7508	CB	ILE	A	954	21.578	-2.699	23.125	1.00	63.25	C
ATOM	7509	O	ILE	A	954	21.875	-1.584	26.203	1.00	63.25	O
ATOM	7510	CG1	ILE	A	954	22.234	-3.766	22.234	1.00	63.25	C
ATOM	7511	CG2	ILE	A	954	22.422	-1.423	23.156	1.00	63.25	C
ATOM	7512	CD1	ILE	A	954	22.297	-3.395	20.766	1.00	63.25	C
ATOM	7513	N	ARG	A	955	19.625	-1.843	25.797	1.00	58.78	N
ATOM	7514	CA	ARG	A	955	19.156	-0.740	26.641	1.00	58.78	C
ATOM	7515	C	ARG	A	955	19.406	-1.027	28.109	1.00	58.78	C
ATOM	7516	CB	ARG	A	955	17.672	-0.475	26.406	1.00	58.78	C
ATOM	7517	O	ARG	A	955	19.750	-0.122	28.875	1.00	58.78	O
ATOM	7518	CG	ARG	A	955	17.391	0.588	25.344	1.00	58.78	C
ATOM	7519	CD	ARG	A	955	15.906	0.917	25.266	1.00	58.78	C
ATOM	7520	NE	ARG	A	955	15.523	1.354	23.922	1.00	58.78	N
ATOM	7521	NH1	ARG	A	955	13.258	1.392	24.344	1.00	58.78	N
ATOM	7522	NH2	ARG	A	955	14.047	1.967	22.281	1.00	58.78	N
ATOM	7523	CZ	ARG	A	955	14.273	1.570	23.516	1.00	58.78	C
ATOM	7524	N	GLU	A	956	19.531	-2.326	28.453	1.00	59.94	N
ATOM	7525	CA	GLU	A	956	19.766	-2.670	29.844	1.00	59.94	C
ATOM	7526	C	GLU	A	956	21.266	-2.635	30.172	1.00	59.94	C
ATOM	7527	CB	GLU	A	956	19.188	-4.051	30.172	1.00	59.94	C
ATOM	7528	O	GLU	A	956	21.656	-2.170	31.250	1.00	59.94	O
ATOM	7529	CG	GLU	A	956	18.859	-4.250	31.641	1.00	59.94	C
ATOM	7530	CD	GLU	A	956	18.062	-5.520	31.906	1.00	59.94	C
ATOM	7531	OE1	GLU	A	956	17.656	-5.746	33.062	1.00	59.94	O
ATOM	7532	OE2	GLU	A	956	17.859	-6.297	30.953	1.00	59.94	O
ATOM	7533	N	VAL	A	957	22.125	-2.857	29.141	1.00	66.31	N
ATOM	7534	CA	VAL	A	957	23.547	-2.799	29.391	1.00	66.31	C
ATOM	7535	C	VAL	A	957	24.016	-1.344	29.391	1.00	66.31	C
ATOM	7536	CB	VAL	A	957	24.344	-3.605	28.344	1.00	66.31	C
ATOM	7537	O	VAL	A	957	24.844	-0.951	30.234	1.00	66.31	O
ATOM	7538	CG1	VAL	A	957	25.844	-3.361	28.484	1.00	66.31	C
ATOM	7539	CG2	VAL	A	957	24.031	-5.094	28.453	1.00	66.31	C
ATOM	7540	N	ILE	A	958	23.359	-0.372	28.547	1.00	63.78	N
ATOM	7541	CA	ILE	A	958	23.766	1.028	28.453	1.00	63.78	C
ATOM	7542	C	ILE	A	958	23.297	1.784	29.703	1.00	63.78	C
ATOM	7543	CB	ILE	A	958	23.188	1.691	27.188	1.00	63.78	C
ATOM	7544	O	ILE	A	958	24.016	2.648	30.203	1.00	63.78	O
ATOM	7545	CG1	ILE	A	958	23.875	1.134	25.938	1.00	63.78	C
ATOM	7546	CG2	ILE	A	958	23.328	3.215	27.250	1.00	63.78	C
ATOM	7547	CD1	ILE	A	958	23.250	1.600	24.625	1.00	63.78	C
ATOM	7548	N	ALA	A	959	22.141	1.361	30.312	1.00	60.66	N

ATOM	7549	CA	ALA	A	959	21.609	2.059	31.484	1.00	60.66	C
ATOM	7550	C	ALA	A	959	22.531	1.861	32.688	1.00	60.66	C
ATOM	7551	CB	ALA	A	959	20.203	1.574	31.812	1.00	60.66	C
ATOM	7552	O	ALA	A	959	22.766	2.797	33.469	1.00	60.66	O
ATOM	7553	N	THR	A	960	23.234	0.672	32.781	1.00	63.78	N
ATOM	7554	CA	THR	A	960	24.078	0.411	33.938	1.00	63.78	C
ATOM	7555	C	THR	A	960	25.422	1.114	33.781	1.00	63.78	C
ATOM	7556	CB	THR	A	960	24.297	-1.100	34.125	1.00	63.78	C
ATOM	7557	O	THR	A	960	25.938	1.687	34.750	1.00	63.78	O
ATOM	7558	CG2	THR	A	960	24.078	-1.503	35.594	1.00	63.78	C
ATOM	7559	OG1	THR	A	960	23.391	-1.826	33.312	1.00	63.78	O
ATOM	7560	N	GLU	A	961	25.875	1.338	32.500	1.00	64.06	N
ATOM	7561	CA	GLU	A	961	27.156	2.014	32.281	1.00	64.06	C
ATOM	7562	C	GLU	A	961	26.984	3.529	32.281	1.00	64.06	C
ATOM	7563	CB	GLU	A	961	27.781	1.561	30.953	1.00	64.06	C
ATOM	7564	O	GLU	A	961	27.875	4.258	32.750	1.00	64.06	O
ATOM	7565	CG	GLU	A	961	28.734	0.385	31.109	1.00	64.06	C
ATOM	7566	CD	GLU	A	961	29.344	-0.050	29.781	1.00	64.06	C
ATOM	7567	OE1	GLU	A	961	30.172	-0.991	29.781	1.00	64.06	O
ATOM	7568	OE2	GLU	A	961	29.000	0.555	28.734	1.00	64.06	O
ATOM	7569	N	THR	A	962	25.750	4.098	31.859	1.00	63.31	N
ATOM	7570	CA	THR	A	962	25.406	5.516	31.844	1.00	63.31	C
ATOM	7571	C	THR	A	962	25.266	6.043	33.281	1.00	63.31	C
ATOM	7572	CB	THR	A	962	24.109	5.773	31.062	1.00	63.31	C
ATOM	7573	O	THR	A	962	25.719	7.148	33.594	1.00	63.31	O
ATOM	7574	CG2	THR	A	962	23.891	7.266	30.844	1.00	63.31	C
ATOM	7575	OG1	THR	A	962	24.188	5.121	29.797	1.00	63.31	O
ATOM	7576	N	GLU	A	963	24.812	5.176	34.156	1.00	64.69	N
ATOM	7577	CA	GLU	A	963	24.625	5.648	35.531	1.00	64.69	C
ATOM	7578	C	GLU	A	963	25.969	5.762	36.250	1.00	64.69	C
ATOM	7579	CB	GLU	A	963	23.688	4.719	36.312	1.00	64.69	C
ATOM	7580	O	GLU	A	963	26.203	6.711	37.000	1.00	64.69	O
ATOM	7581	CG	GLU	A	963	22.219	5.062	36.156	1.00	64.69	C
ATOM	7582	CD	GLU	A	963	21.297	4.078	36.844	1.00	64.69	C
ATOM	7583	OE1	GLU	A	963	20.062	4.254	36.781	1.00	64.69	O
ATOM	7584	OE2	GLU	A	963	21.812	3.121	37.469	1.00	64.69	O
ATOM	7585	N	ALA	A	964	26.922	4.836	35.875	1.00	67.44	N
ATOM	7586	CA	ALA	A	964	28.250	4.863	36.500	1.00	67.44	C
ATOM	7587	C	ALA	A	964	29.094	6.020	35.969	1.00	67.44	C
ATOM	7588	CB	ALA	A	964	28.969	3.537	36.281	1.00	67.44	C
ATOM	7589	O	ALA	A	964	29.797	6.688	36.719	1.00	67.44	O
ATOM	7590	N	LEU	A	965	28.922	6.473	34.656	1.00	69.88	N
ATOM	7591	CA	LEU	A	965	29.609	7.590	34.031	1.00	69.88	C
ATOM	7592	C	LEU	A	965	29.016	8.922	34.469	1.00	69.88	C
ATOM	7593	CB	LEU	A	965	29.547	7.477	32.500	1.00	69.88	C
ATOM	7594	O	LEU	A	965	29.734	9.891	34.688	1.00	69.88	O
ATOM	7595	CG	LEU	A	965	30.703	8.109	31.734	1.00	69.88	C
ATOM	7596	CD1	LEU	A	965	31.859	7.137	31.609	1.00	69.88	C
ATOM	7597	CD2	LEU	A	965	30.234	8.555	30.344	1.00	69.88	C
ATOM	7598	N	SER	A	966	27.656	9.008	34.719	1.00	64.81	N
ATOM	7599	CA	SER	A	966	26.969	10.195	35.219	1.00	64.81	C
ATOM	7600	C	SER	A	966	27.453	10.562	36.625	1.00	64.81	C
ATOM	7601	CB	SER	A	966	25.453	9.969	35.219	1.00	64.81	C
ATOM	7602	O	SER	A	966	27.609	11.742	36.938	1.00	64.81	O
ATOM	7603	OG	SER	A	966	24.766	11.195	35.031	1.00	64.81	O
ATOM	7604	N	ARG	A	967	27.859	9.516	37.500	1.00	61.50	N
ATOM	7605	CA	ARG	A	967	28.328	9.797	38.844	1.00	61.50	C
ATOM	7606	C	ARG	A	967	29.766	10.312	38.812	1.00	61.50	C
ATOM	7607	CB	ARG	A	967	28.234	8.539	39.719	1.00	61.50	C
ATOM	7608	O	ARG	A	967	30.109	11.227	39.594	1.00	61.50	O
ATOM	7609	CG	ARG	A	967	26.859	8.305	40.312	1.00	61.50	C
ATOM	7610	CD	ARG	A	967	26.859	7.152	41.281	1.00	61.50	C
ATOM	7611	NE	ARG	A	967	25.531	6.559	41.438	1.00	61.50	N
ATOM	7612	NH1	ARG	A	967	26.266	4.699	42.594	1.00	61.50	N

ATOM	7613	NH2	ARG	A	967	24.031	4.953	42.094	1.00	61.50	N
ATOM	7614	CZ	ARG	A	967	25.281	5.402	42.031	1.00	61.50	C
ATOM	7615	N	GLU	A	968	30.578	9.922	37.781	1.00	59.12	N
ATOM	7616	CA	GLU	A	968	31.953	10.398	37.656	1.00	59.12	C
ATOM	7617	C	GLU	A	968	32.000	11.797	37.031	1.00	59.12	C
ATOM	7618	CB	GLU	A	968	32.781	9.438	36.781	1.00	59.12	C
ATOM	7619	O	GLU	A	968	32.812	12.633	37.469	1.00	59.12	O
ATOM	7620	CG	GLU	A	968	33.969	8.828	37.531	1.00	59.12	C
ATOM	7621	CD	GLU	A	968	34.844	7.949	36.625	1.00	59.12	C
ATOM	7622	OE1	GLU	A	968	35.844	7.410	37.125	1.00	59.12	O
ATOM	7623	OE2	GLU	A	968	34.500	7.797	35.438	1.00	59.12	O
ATOM	7624	N	ILE	A	969	30.953	12.219	36.125	1.00	60.69	N
ATOM	7625	CA	ILE	A	969	30.828	13.508	35.469	1.00	60.69	C
ATOM	7626	C	ILE	A	969	30.312	14.555	36.438	1.00	60.69	C
ATOM	7627	CB	ILE	A	969	29.906	13.430	34.219	1.00	60.69	C
ATOM	7628	O	ILE	A	969	30.781	15.688	36.469	1.00	60.69	O
ATOM	7629	CG1	ILE	A	969	30.594	12.648	33.094	1.00	60.69	C
ATOM	7630	CG2	ILE	A	969	29.500	14.828	33.750	1.00	60.69	C
ATOM	7631	CD1	ILE	A	969	29.703	12.398	31.891	1.00	60.69	C
ATOM	7632	N	ASP	A	970	29.438	14.148	37.438	1.00	60.06	N
ATOM	7633	CA	ASP	A	970	28.875	15.070	38.406	1.00	60.06	C
ATOM	7634	C	ASP	A	970	29.938	15.500	39.438	1.00	60.06	C
ATOM	7635	CB	ASP	A	970	27.688	14.438	39.125	1.00	60.06	C
ATOM	7636	O	ASP	A	970	29.953	16.656	39.875	1.00	60.06	O
ATOM	7637	CG	ASP	A	970	26.438	14.383	38.281	1.00	60.06	C
ATOM	7638	OD1	ASP	A	970	26.344	15.148	37.281	1.00	60.06	O
ATOM	7639	OD2	ASP	A	970	25.531	13.578	38.594	1.00	60.06	O
ATOM	7640	N	GLN	A	971	31.031	14.680	39.656	1.00	54.72	N
ATOM	7641	CA	GLN	A	971	32.094	14.984	40.594	1.00	54.72	C
ATOM	7642	C	GLN	A	971	33.156	15.906	40.000	1.00	54.72	C
ATOM	7643	CB	GLN	A	971	32.750	13.695	41.094	1.00	54.72	C
ATOM	7644	O	GLN	A	971	33.688	16.781	40.688	1.00	54.72	O
ATOM	7645	CG	GLN	A	971	32.156	13.164	42.406	1.00	54.72	C
ATOM	7646	CD	GLN	A	971	32.844	11.922	42.906	1.00	54.72	C
ATOM	7647	NE2	GLN	A	971	32.531	11.508	44.125	1.00	54.72	N
ATOM	7648	OE1	GLN	A	971	33.688	11.336	42.219	1.00	54.72	O
ATOM	7649	N	LEU	A	972	33.312	15.984	38.594	1.00	55.31	N
ATOM	7650	CA	LEU	A	972	34.250	16.828	37.875	1.00	55.31	C
ATOM	7651	C	LEU	A	972	33.656	18.219	37.625	1.00	55.31	C
ATOM	7652	CB	LEU	A	972	34.656	16.188	36.562	1.00	55.31	C
ATOM	7653	O	LEU	A	972	34.375	19.219	37.656	1.00	55.31	O
ATOM	7654	CG	LEU	A	972	36.094	15.680	36.469	1.00	55.31	C
ATOM	7655	CD1	LEU	A	972	36.125	14.219	36.000	1.00	55.31	C
ATOM	7656	CD2	LEU	A	972	36.906	16.547	35.500	1.00	55.31	C
ATOM	7657	N	LYS	A	973	32.219	18.453	37.594	1.00	51.75	N
ATOM	7658	CA	LYS	A	973	31.500	19.688	37.344	1.00	51.75	C
ATOM	7659	C	LYS	A	973	31.531	20.609	38.562	1.00	51.75	C
ATOM	7660	CB	LYS	A	973	30.047	19.391	36.938	1.00	51.75	C
ATOM	7661	O	LYS	A	973	31.547	21.828	38.438	1.00	51.75	O
ATOM	7662	CG	LYS	A	973	29.859	19.109	35.469	1.00	51.75	C
ATOM	7663	CD	LYS	A	973	28.406	18.906	35.094	1.00	51.75	C
ATOM	7664	CE	LYS	A	973	28.219	18.516	33.656	1.00	51.75	C
ATOM	7665	NZ	LYS	A	973	26.781	18.375	33.281	1.00	51.75	N
ATOM	7666	N	ALA	A	974	31.969	20.109	39.781	1.00	49.97	N
ATOM	7667	CA	ALA	A	974	31.953	20.906	41.000	1.00	49.97	C
ATOM	7668	C	ALA	A	974	33.281	21.609	41.219	1.00	49.97	C
ATOM	7669	CB	ALA	A	974	31.625	20.031	42.219	1.00	49.97	C
ATOM	7670	O	ALA	A	974	33.344	22.672	41.844	1.00	49.97	O
ATOM	7671	N	GLN	A	975	34.375	21.562	40.250	1.00	43.28	N
ATOM	7672	CA	GLN	A	975	35.656	22.234	40.500	1.00	43.28	C
ATOM	7673	C	GLN	A	975	35.969	23.203	39.375	1.00	43.28	C
ATOM	7674	CB	GLN	A	975	36.781	21.203	40.625	1.00	43.28	C
ATOM	7675	O	GLN	A	975	36.938	23.984	39.469	1.00	43.28	O
ATOM	7676	CG	GLN	A	975	36.969	20.719	42.062	1.00	43.28	C

ATOM	7677	CD	GLN	A	975	38.188	19.797	42.188	1.00	43.28	C
ATOM	7678	NE2	GLN	A	975	38.625	19.609	43.438	1.00	43.28	N
ATOM	7679	OE1	GLN	A	975	38.688	19.250	41.219	1.00	43.28	O
ATOM	7680	N	ILE	A	976	35.031	24.078	38.625	1.00	46.62	N
ATOM	7681	CA	ILE	A	976	35.500	24.906	37.500	1.00	46.62	C
ATOM	7682	C	ILE	A	976	34.781	26.250	37.531	1.00	46.62	C
ATOM	7683	CB	ILE	A	976	35.250	24.203	36.156	1.00	46.62	C
ATOM	7684	O	ILE	A	976	33.562	26.312	37.781	1.00	46.62	O
ATOM	7685	CG1	ILE	A	976	36.062	22.906	36.094	1.00	46.62	C
ATOM	7686	CG2	ILE	A	976	35.625	25.141	35.000	1.00	46.62	C
ATOM	7687	CD1	ILE	A	976	35.781	22.062	34.844	1.00	46.62	C
ATOM	7688	N	GLY	A	977	35.375	27.359	38.125	1.00	51.47	N
ATOM	7689	CA	GLY	A	977	35.344	28.781	37.844	1.00	51.47	C
ATOM	7690	C	GLY	A	977	34.312	29.156	36.781	1.00	51.47	C
ATOM	7691	O	GLY	A	977	33.750	28.297	36.125	1.00	51.47	O
ATOM	7692	N	ASP	A	978	33.500	30.172	36.844	1.00	54.62	N
ATOM	7693	CA	ASP	A	978	32.344	30.750	36.156	1.00	54.62	C
ATOM	7694	C	ASP	A	978	32.562	30.781	34.656	1.00	54.62	C
ATOM	7695	CB	ASP	A	978	32.062	32.156	36.688	1.00	54.62	C
ATOM	7696	O	ASP	A	978	33.656	31.125	34.188	1.00	54.62	O
ATOM	7697	CG	ASP	A	978	31.500	32.156	38.094	1.00	54.62	C
ATOM	7698	OD1	ASP	A	978	30.984	31.125	38.562	1.00	54.62	O
ATOM	7699	OD2	ASP	A	978	31.578	33.219	38.750	1.00	54.62	O
ATOM	7700	N	ASP	A	979	31.875	30.062	33.719	1.00	65.81	N
ATOM	7701	CA	ASP	A	979	31.844	29.953	32.281	1.00	65.81	C
ATOM	7702	C	ASP	A	979	31.391	31.266	31.625	1.00	65.81	C
ATOM	7703	CB	ASP	A	979	30.906	28.812	31.844	1.00	65.81	C
ATOM	7704	O	ASP	A	979	30.516	31.953	32.156	1.00	65.81	O
ATOM	7705	CG	ASP	A	979	31.516	27.438	32.031	1.00	65.81	C
ATOM	7706	OD1	ASP	A	979	32.750	27.344	32.250	1.00	65.81	O
ATOM	7707	OD2	ASP	A	979	30.781	26.438	31.906	1.00	65.81	O
ATOM	7708	N	ILE	A	980	32.219	31.875	30.703	1.00	78.44	N
ATOM	7709	CA	ILE	A	980	31.906	33.031	29.859	1.00	78.44	C
ATOM	7710	C	ILE	A	980	31.562	32.562	28.438	1.00	78.44	C
ATOM	7711	CB	ILE	A	980	33.094	34.031	29.812	1.00	78.44	C
ATOM	7712	O	ILE	A	980	32.375	31.922	27.781	1.00	78.44	O
ATOM	7713	CG1	ILE	A	980	33.469	34.469	31.234	1.00	78.44	C
ATOM	7714	CG2	ILE	A	980	32.719	35.219	28.938	1.00	78.44	C
ATOM	7715	CD1	ILE	A	980	34.750	35.281	31.297	1.00	78.44	C
ATOM	7716	N	GLN	A	981	30.219	32.719	27.984	1.00	82.50	N
ATOM	7717	CA	GLN	A	981	29.734	32.219	26.703	1.00	82.50	C
ATOM	7718	C	GLN	A	981	28.938	33.281	25.953	1.00	82.50	C
ATOM	7719	CB	GLN	A	981	28.859	30.969	26.906	1.00	82.50	C
ATOM	7720	O	GLN	A	981	28.141	34.000	26.547	1.00	82.50	O
ATOM	7721	CG	GLN	A	981	29.625	29.750	27.406	1.00	82.50	C
ATOM	7722	CD	GLN	A	981	28.734	28.547	27.625	1.00	82.50	C
ATOM	7723	NE2	GLN	A	981	29.344	27.438	28.047	1.00	82.50	N
ATOM	7724	OE1	GLN	A	981	27.531	28.594	27.406	1.00	82.50	O
ATOM	7725	N	ALA	A	982	29.344	33.500	24.688	1.00	76.50	N
ATOM	7726	CA	ALA	A	982	28.562	34.281	23.734	1.00	76.50	C
ATOM	7727	C	ALA	A	982	28.109	33.438	22.562	1.00	76.50	C
ATOM	7728	CB	ALA	A	982	29.375	35.500	23.250	1.00	76.50	C
ATOM	7729	O	ALA	A	982	28.906	32.688	21.984	1.00	76.50	O
ATOM	7730	N	SER	A	983	26.750	33.500	22.266	1.00	84.06	N
ATOM	7731	CA	SER	A	983	26.188	32.656	21.219	1.00	84.06	C
ATOM	7732	C	SER	A	983	25.188	33.438	20.359	1.00	84.06	C
ATOM	7733	CB	SER	A	983	25.516	31.422	21.812	1.00	84.06	C
ATOM	7734	O	SER	A	983	24.375	34.188	20.891	1.00	84.06	O
ATOM	7735	OG	SER	A	983	24.938	30.609	20.812	1.00	84.06	O
ATOM	7736	N	LEU	A	984	25.406	33.438	19.109	1.00	86.00	N
ATOM	7737	CA	LEU	A	984	24.453	33.906	18.109	1.00	86.00	C
ATOM	7738	C	LEU	A	984	23.953	32.719	17.266	1.00	86.00	C
ATOM	7739	CB	LEU	A	984	25.094	34.969	17.219	1.00	86.00	C
ATOM	7740	O	LEU	A	984	24.750	32.031	16.625	1.00	86.00	O

ATOM	7741	CG	LEU	A	984	24.234	35.500	16.062	1.00	86.00	C
ATOM	7742	CD1	LEU	A	984	23.141	36.406	16.609	1.00	86.00	C
ATOM	7743	CD2	LEU	A	984	25.094	36.250	15.055	1.00	86.00	C
ATOM	7744	N	THR	A	985	22.578	32.438	17.344	1.00	87.00	N
ATOM	7745	CA	THR	A	985	21.984	31.344	16.594	1.00	87.00	C
ATOM	7746	C	THR	A	985	20.875	31.859	15.680	1.00	87.00	C
ATOM	7747	CB	THR	A	985	21.422	30.281	17.531	1.00	87.00	C
ATOM	7748	O	THR	A	985	19.953	32.562	16.125	1.00	87.00	O
ATOM	7749	CG2	THR	A	985	20.875	29.078	16.750	1.00	87.00	C
ATOM	7750	OG1	THR	A	985	22.453	29.828	18.422	1.00	87.00	O
ATOM	7751	N	ASP	A	986	21.031	31.672	14.359	1.00	90.00	N
ATOM	7752	CA	ASP	A	986	20.062	32.000	13.320	1.00	90.00	C
ATOM	7753	C	ASP	A	986	19.531	30.750	12.641	1.00	90.00	C
ATOM	7754	CB	ASP	A	986	20.703	32.938	12.281	1.00	90.00	C
ATOM	7755	O	ASP	A	986	20.266	30.062	11.922	1.00	90.00	O
ATOM	7756	CG	ASP	A	986	19.688	33.500	11.289	1.00	90.00	C
ATOM	7757	OD1	ASP	A	986	18.500	33.125	11.359	1.00	90.00	O
ATOM	7758	OD2	ASP	A	986	20.078	34.312	10.430	1.00	90.00	O
ATOM	7759	N	ILE	A	987	18.219	30.391	12.883	1.00	84.88	N
ATOM	7760	CA	ILE	A	987	17.609	29.188	12.320	1.00	84.88	C
ATOM	7761	C	ILE	A	987	16.500	29.594	11.352	1.00	84.88	C
ATOM	7762	CB	ILE	A	987	17.047	28.266	13.422	1.00	84.88	C
ATOM	7763	O	ILE	A	987	15.547	30.281	11.742	1.00	84.88	O
ATOM	7764	CG1	ILE	A	987	18.172	27.828	14.367	1.00	84.88	C
ATOM	7765	CG2	ILE	A	987	16.344	27.047	12.805	1.00	84.88	C
ATOM	7766	CD1	ILE	A	987	17.672	27.078	15.602	1.00	84.88	C
ATOM	7767	N	ARG	A	988	16.688	29.266	10.086	1.00	87.06	N
ATOM	7768	CA	ARG	A	988	15.680	29.531	9.062	1.00	87.06	C
ATOM	7769	C	ARG	A	988	15.203	28.234	8.422	1.00	87.06	C
ATOM	7770	CB	ARG	A	988	16.234	30.469	7.988	1.00	87.06	C
ATOM	7771	O	ARG	A	988	16.016	27.406	8.008	1.00	87.06	O
ATOM	7772	CG	ARG	A	988	16.594	31.844	8.508	1.00	87.06	C
ATOM	7773	CD	ARG	A	988	17.047	32.781	7.379	1.00	87.06	C
ATOM	7774	NE	ARG	A	988	17.453	34.094	7.883	1.00	87.06	N
ATOM	7775	NH1	ARG	A	988	18.172	34.875	5.836	1.00	87.06	N
ATOM	7776	NH2	ARG	A	988	18.297	36.219	7.699	1.00	87.06	N
ATOM	7777	CZ	ARG	A	988	17.969	35.062	7.141	1.00	87.06	C
ATOM	7778	N	GLU	A	989	13.797	28.094	8.445	1.00	86.81	N
ATOM	7779	CA	GLU	A	989	13.188	26.875	7.914	1.00	86.81	C
ATOM	7780	C	GLU	A	989	12.008	27.203	7.000	1.00	86.81	C
ATOM	7781	CB	GLU	A	989	12.734	25.953	9.047	1.00	86.81	C
ATOM	7782	O	GLU	A	989	11.195	28.078	7.324	1.00	86.81	O
ATOM	7783	CG	GLU	A	989	12.320	24.562	8.586	1.00	86.81	C
ATOM	7784	CD	GLU	A	989	11.906	23.656	9.734	1.00	86.81	C
ATOM	7785	OE1	GLU	A	989	11.977	22.422	9.578	1.00	86.81	O
ATOM	7786	OE2	GLU	A	989	11.500	24.188	10.789	1.00	86.81	O
ATOM	7787	N	ALA	A	990	12.023	26.500	5.895	1.00	80.62	N
ATOM	7788	CA	ALA	A	990	10.898	26.547	4.965	1.00	80.62	C
ATOM	7789	C	ALA	A	990	10.391	25.141	4.648	1.00	80.62	C
ATOM	7790	CB	ALA	A	990	11.297	27.266	3.682	1.00	80.62	C
ATOM	7791	O	ALA	A	990	11.164	24.281	4.215	1.00	80.62	O
ATOM	7792	N	ILE	A	991	9.109	24.953	4.984	1.00	80.12	N
ATOM	7793	CA	ILE	A	991	8.523	23.625	4.801	1.00	80.12	C
ATOM	7794	C	ILE	A	991	7.344	23.719	3.838	1.00	80.12	C
ATOM	7795	CB	ILE	A	991	8.078	23.016	6.145	1.00	80.12	C
ATOM	7796	O	ILE	A	991	6.477	24.578	3.986	1.00	80.12	O
ATOM	7797	CG1	ILE	A	991	9.273	22.906	7.102	1.00	80.12	C
ATOM	7798	CG2	ILE	A	991	7.418	21.656	5.926	1.00	80.12	C
ATOM	7799	CD1	ILE	A	991	8.883	22.547	8.531	1.00	80.12	C
ATOM	7800	N	ALA	A	992	7.324	22.859	2.852	1.00	71.56	N
ATOM	7801	CA	ALA	A	992	6.203	22.719	1.928	1.00	71.56	C
ATOM	7802	C	ALA	A	992	5.824	21.266	1.739	1.00	71.56	C
ATOM	7803	CB	ALA	A	992	6.539	23.359	0.581	1.00	71.56	C
ATOM	7804	O	ALA	A	992	6.645	20.453	1.289	1.00	71.56	O

ATOM	7805	N	ASN	A	993	4.578	21.047	2.316	1.00	67.12	N
ATOM	7806	CA	ASN	A	993	4.066	19.703	2.064	1.00	67.12	C
ATOM	7807	C	ASN	A	993	2.602	19.734	1.640	1.00	67.12	C
ATOM	7808	CB	ASN	A	993	4.242	18.812	3.303	1.00	67.12	C
ATOM	7809	O	ASN	A	993	2.053	20.797	1.359	1.00	67.12	O
ATOM	7810	CG	ASN	A	993	3.521	19.359	4.520	1.00	67.12	C
ATOM	7811	ND2	ASN	A	993	4.211	19.391	5.652	1.00	67.12	N
ATOM	7812	OD1	ASN	A	993	2.355	19.766	4.438	1.00	67.12	O
ATOM	7813	N	GLU	A	994	1.791	18.516	1.622	1.00	60.69	N
ATOM	7814	CA	GLU	A	994	0.443	18.453	1.064	1.00	60.69	C
ATOM	7815	C	GLU	A	994	-0.558	19.172	1.966	1.00	60.69	C
ATOM	7816	CB	GLU	A	994	0.011	17.000	0.852	1.00	60.69	C
ATOM	7817	O	GLU	A	994	-1.582	19.672	1.493	1.00	60.69	O
ATOM	7818	CG	GLU	A	994	0.710	16.312	-0.314	1.00	60.69	C
ATOM	7819	CD	GLU	A	994	0.175	14.922	-0.600	1.00	60.69	C
ATOM	7820	OE1	GLU	A	994	0.653	14.273	-1.557	1.00	60.69	O
ATOM	7821	OE2	GLU	A	994	-0.731	14.477	0.140	1.00	60.69	O
ATOM	7822	N	THR	A	995	-0.209	19.438	3.172	1.00	63.25	N
ATOM	7823	CA	THR	A	995	-1.207	19.953	4.109	1.00	63.25	C
ATOM	7824	C	THR	A	995	-0.757	21.266	4.715	1.00	63.25	C
ATOM	7825	CB	THR	A	995	-1.484	18.938	5.230	1.00	63.25	C
ATOM	7826	O	THR	A	995	-1.550	21.969	5.352	1.00	63.25	O
ATOM	7827	CG2	THR	A	995	-2.088	17.641	4.676	1.00	63.25	C
ATOM	7828	OG1	THR	A	995	-0.256	18.625	5.898	1.00	63.25	O
ATOM	7829	N	GLU	A	996	0.633	21.656	4.422	1.00	77.25	N
ATOM	7830	CA	GLU	A	996	1.104	22.828	5.145	1.00	77.25	C
ATOM	7831	C	GLU	A	996	2.227	23.531	4.387	1.00	77.25	C
ATOM	7832	CB	GLU	A	996	1.575	22.453	6.551	1.00	77.25	C
ATOM	7833	O	GLU	A	996	3.012	22.891	3.691	1.00	77.25	O
ATOM	7834	CG	GLU	A	996	2.098	23.625	7.363	1.00	77.25	C
ATOM	7835	CD	GLU	A	996	2.641	23.219	8.727	1.00	77.25	C
ATOM	7836	OE1	GLU	A	996	2.939	24.109	9.555	1.00	77.25	O
ATOM	7837	OE2	GLU	A	996	2.771	22.000	8.969	1.00	77.25	O
ATOM	7838	N	ALA	A	997	2.156	24.797	4.391	1.00	76.62	N
ATOM	7839	CA	ALA	A	997	3.236	25.703	4.004	1.00	76.62	C
ATOM	7840	C	ALA	A	997	3.621	26.625	5.160	1.00	76.62	C
ATOM	7841	CB	ALA	A	997	2.830	26.531	2.787	1.00	76.62	C
ATOM	7842	O	ALA	A	997	2.777	27.344	5.695	1.00	76.62	O
ATOM	7843	N	ARG	A	998	4.855	26.484	5.539	1.00	87.50	N
ATOM	7844	CA	ARG	A	998	5.254	27.250	6.723	1.00	87.50	C
ATOM	7845	C	ARG	A	998	6.684	27.766	6.586	1.00	87.50	C
ATOM	7846	CB	ARG	A	998	5.121	26.391	7.980	1.00	87.50	C
ATOM	7847	O	ARG	A	998	7.566	27.031	6.121	1.00	87.50	O
ATOM	7848	CG	ARG	A	998	5.375	27.141	9.273	1.00	87.50	C
ATOM	7849	CD	ARG	A	998	5.004	26.312	10.500	1.00	87.50	C
ATOM	7850	NE	ARG	A	998	5.125	27.094	11.727	1.00	87.50	N
ATOM	7851	NH1	ARG	A	998	4.555	25.344	13.125	1.00	87.50	N
ATOM	7852	NH2	ARG	A	998	5.047	27.406	14.000	1.00	87.50	N
ATOM	7853	CZ	ARG	A	998	4.906	26.609	12.953	1.00	87.50	C
ATOM	7854	N	THR	A	999	6.969	28.969	6.836	1.00	86.12	N
ATOM	7855	CA	THR	A	999	8.289	29.594	6.922	1.00	86.12	C
ATOM	7856	C	THR	A	999	8.539	30.141	8.320	1.00	86.12	C
ATOM	7857	CB	THR	A	999	8.453	30.719	5.887	1.00	86.12	C
ATOM	7858	O	THR	A	999	7.652	30.766	8.914	1.00	86.12	O
ATOM	7859	CG2	THR	A	999	8.336	30.172	4.465	1.00	86.12	C
ATOM	7860	OG1	THR	A	999	7.434	31.703	6.098	1.00	86.12	O
ATOM	7861	N	GLN	A	1000	9.695	29.734	8.836	1.00	88.88	N
ATOM	7862	CA	GLN	A	1000	10.031	30.156	10.188	1.00	88.88	C
ATOM	7863	C	GLN	A	1000	11.469	30.656	10.266	1.00	88.88	C
ATOM	7864	CB	GLN	A	1000	9.812	29.016	11.180	1.00	88.88	C
ATOM	7865	O	GLN	A	1000	12.359	30.094	9.625	1.00	88.88	O
ATOM	7866	CG	GLN	A	1000	10.078	29.406	12.633	1.00	88.88	C
ATOM	7867	CD	GLN	A	1000	9.828	28.250	13.594	1.00	88.88	C
ATOM	7868	NE2	GLN	A	1000	9.977	28.531	14.891	1.00	88.88	N

ATOM	7869	OE1	GLN	A1000	9.500	27.141	13.180	1.00	88.88	O
ATOM	7870	N	ALA	A1001	11.656	31.781	11.016	1.00	88.81	N
ATOM	7871	CA	ALA	A1001	12.984	32.312	11.312	1.00	88.81	C
ATOM	7872	C	ALA	A1001	13.125	32.656	12.789	1.00	88.81	C
ATOM	7873	CB	ALA	A1001	13.281	33.531	10.445	1.00	88.81	C
ATOM	7874	O	ALA	A1001	12.320	33.406	13.328	1.00	88.81	O
ATOM	7875	N	ASP	A1002	14.125	32.031	13.453	1.00	88.06	N
ATOM	7876	CA	ASP	A1002	14.422	32.281	14.852	1.00	88.06	C
ATOM	7877	C	ASP	A1002	15.828	32.875	15.016	1.00	88.06	C
ATOM	7878	CB	ASP	A1002	14.289	31.000	15.680	1.00	88.06	C
ATOM	7879	O	ASP	A1002	16.812	32.250	14.586	1.00	88.06	O
ATOM	7880	CG	ASP	A1002	12.859	30.484	15.750	1.00	88.06	C
ATOM	7881	OD1	ASP	A1002	11.922	31.297	15.742	1.00	88.06	O
ATOM	7882	OD2	ASP	A1002	12.680	29.250	15.828	1.00	88.06	O
ATOM	7883	N	LEU	A1003	15.961	34.000	15.469	1.00	90.62	N
ATOM	7884	CA	LEU	A1003	17.234	34.656	15.805	1.00	90.62	C
ATOM	7885	C	LEU	A1003	17.375	34.781	17.312	1.00	90.62	C
ATOM	7886	CB	LEU	A1003	17.344	36.031	15.148	1.00	90.62	C
ATOM	7887	O	LEU	A1003	16.516	35.344	17.984	1.00	90.62	O
ATOM	7888	CG	LEU	A1003	18.609	36.812	15.445	1.00	90.62	C
ATOM	7889	CD1	LEU	A1003	19.828	36.094	14.859	1.00	90.62	C
ATOM	7890	CD2	LEU	A1003	18.500	38.219	14.891	1.00	90.62	C
ATOM	7891	N	THR	A1004	18.406	34.125	17.875	1.00	87.50	N
ATOM	7892	CA	THR	A1004	18.641	34.188	19.312	1.00	87.50	C
ATOM	7893	C	THR	A1004	20.062	34.656	19.609	1.00	87.50	C
ATOM	7894	CB	THR	A1004	18.391	32.812	19.984	1.00	87.50	C
ATOM	7895	O	THR	A1004	21.000	34.250	18.938	1.00	87.50	O
ATOM	7896	CG2	THR	A1004	16.922	32.438	19.922	1.00	87.50	C
ATOM	7897	OG1	THR	A1004	19.172	31.828	19.328	1.00	87.50	O
ATOM	7898	N	LEU	A1005	20.219	35.594	20.484	1.00	88.06	N
ATOM	7899	CA	LEU	A1005	21.484	36.094	21.016	1.00	88.06	C
ATOM	7900	C	LEU	A1005	21.578	35.812	22.516	1.00	88.06	C
ATOM	7901	CB	LEU	A1005	21.609	37.594	20.750	1.00	88.06	C
ATOM	7902	O	LEU	A1005	20.625	36.031	23.250	1.00	88.06	O
ATOM	7903	CG	LEU	A1005	21.734	38.000	19.281	1.00	88.06	C
ATOM	7904	CD1	LEU	A1005	20.375	38.000	18.609	1.00	88.06	C
ATOM	7905	CD2	LEU	A1005	22.406	39.375	19.172	1.00	88.06	C
ATOM	7906	N	SER	A1006	22.688	35.188	22.922	1.00	89.81	N
ATOM	7907	CA	SER	A1006	22.891	34.938	24.344	1.00	89.81	C
ATOM	7908	C	SER	A1006	24.312	35.312	24.766	1.00	89.81	C
ATOM	7909	CB	SER	A1006	22.578	33.500	24.703	1.00	89.81	C
ATOM	7910	O	SER	A1006	25.281	34.938	24.094	1.00	89.81	O
ATOM	7911	OG	SER	A1006	22.891	33.219	26.047	1.00	89.81	O
ATOM	7912	N	ALA	A1007	24.484	36.062	25.734	1.00	84.50	N
ATOM	7913	CA	ALA	A1007	25.750	36.375	26.406	1.00	84.50	C
ATOM	7914	C	ALA	A1007	25.703	36.000	27.875	1.00	84.50	C
ATOM	7915	CB	ALA	A1007	26.047	37.875	26.234	1.00	84.50	C
ATOM	7916	O	ALA	A1007	24.891	36.531	28.641	1.00	84.50	O
ATOM	7917	N	ARG	A1008	26.547	35.031	28.328	1.00	84.62	N
ATOM	7918	CA	ARG	A1008	26.500	34.469	29.672	1.00	84.62	C
ATOM	7919	C	ARG	A1008	27.844	34.594	30.375	1.00	84.62	C
ATOM	7920	CB	ARG	A1008	26.047	33.000	29.656	1.00	84.62	C
ATOM	7921	O	ARG	A1008	28.875	34.281	29.797	1.00	84.62	O
ATOM	7922	CG	ARG	A1008	25.875	32.406	31.031	1.00	84.62	C
ATOM	7923	CD	ARG	A1008	25.406	30.953	30.953	1.00	84.62	C
ATOM	7924	NE	ARG	A1008	25.391	30.312	32.250	1.00	84.62	N
ATOM	7925	NH1	ARG	A1008	24.484	28.328	31.516	1.00	84.62	N
ATOM	7926	NH2	ARG	A1008	24.969	28.609	33.750	1.00	84.62	N
ATOM	7927	CZ	ARG	A1008	24.953	29.078	32.500	1.00	84.62	C
ATOM	7928	N	LEU	A1009	27.906	35.031	31.516	1.00	79.12	N
ATOM	7929	CA	LEU	A1009	29.047	35.062	32.438	1.00	79.12	C
ATOM	7930	C	LEU	A1009	28.672	34.500	33.781	1.00	79.12	C
ATOM	7931	CB	LEU	A1009	29.547	36.500	32.594	1.00	79.12	C
ATOM	7932	O	LEU	A1009	27.953	35.125	34.562	1.00	79.12	O

ATOM	7933	CG	LEU	A1009	30.828	36.688	33.438	1.00	79.12	C
ATOM	7934	CD1	LEU	A1009	32.062	36.281	32.625	1.00	79.12	C
ATOM	7935	CD2	LEU	A1009	30.953	38.125	33.938	1.00	79.12	C
ATOM	7936	N	GLY	A1010	29.078	33.219	34.031	1.00	75.31	N
ATOM	7937	CA	GLY	A1010	28.672	32.594	35.281	1.00	75.31	C
ATOM	7938	C	GLY	A1010	27.172	32.406	35.406	1.00	75.31	C
ATOM	7939	O	GLY	A1010	26.547	31.750	34.562	1.00	75.31	O
ATOM	7940	N	ASN	A1011	26.438	33.031	36.406	1.00	75.94	N
ATOM	7941	CA	ASN	A1011	25.000	32.938	36.625	1.00	75.94	C
ATOM	7942	C	ASN	A1011	24.250	34.094	36.000	1.00	75.94	C
ATOM	7943	CB	ASN	A1011	24.703	32.875	38.125	1.00	75.94	C
ATOM	7944	O	ASN	A1011	23.016	34.125	36.031	1.00	75.94	O
ATOM	7945	CG	ASN	A1011	25.188	31.609	38.781	1.00	75.94	C
ATOM	7946	ND2	ASN	A1011	25.312	31.625	40.125	1.00	75.94	N
ATOM	7947	OD1	ASN	A1011	25.469	30.609	38.125	1.00	75.94	O
ATOM	7948	N	ASN	A1012	24.969	35.062	35.312	1.00	81.44	N
ATOM	7949	CA	ASN	A1012	24.344	36.219	34.656	1.00	81.44	C
ATOM	7950	C	ASN	A1012	24.234	35.969	33.156	1.00	81.44	C
ATOM	7951	CB	ASN	A1012	25.141	37.469	34.969	1.00	81.44	C
ATOM	7952	O	ASN	A1012	25.219	35.656	32.500	1.00	81.44	O
ATOM	7953	CG	ASN	A1012	25.188	37.812	36.438	1.00	81.44	C
ATOM	7954	ND2	ASN	A1012	26.281	38.438	36.844	1.00	81.44	N
ATOM	7955	OD1	ASN	A1012	24.266	37.500	37.188	1.00	81.44	O
ATOM	7956	N	GLU	A1013	23.000	36.062	32.562	1.00	89.56	N
ATOM	7957	CA	GLU	A1013	22.750	35.781	31.141	1.00	89.56	C
ATOM	7958	C	GLU	A1013	21.766	36.781	30.547	1.00	89.56	C
ATOM	7959	CB	GLU	A1013	22.234	34.344	30.969	1.00	89.56	C
ATOM	7960	O	GLU	A1013	20.734	37.094	31.141	1.00	89.56	O
ATOM	7961	CG	GLU	A1013	22.047	33.938	29.531	1.00	89.56	C
ATOM	7962	CD	GLU	A1013	21.594	32.500	29.375	1.00	89.56	C
ATOM	7963	OE1	GLU	A1013	21.469	32.031	28.219	1.00	89.56	O
ATOM	7964	OE2	GLU	A1013	21.359	31.844	30.406	1.00	89.56	O
ATOM	7965	N	ALA	A1014	22.203	37.438	29.516	1.00	87.81	N
ATOM	7966	CA	ALA	A1014	21.328	38.281	28.688	1.00	87.81	C
ATOM	7967	C	ALA	A1014	21.000	37.594	27.375	1.00	87.81	C
ATOM	7968	CB	ALA	A1014	21.969	39.625	28.422	1.00	87.81	C
ATOM	7969	O	ALA	A1014	21.891	37.062	26.703	1.00	87.81	O
ATOM	7970	N	ALA	A1015	19.766	37.500	27.078	1.00	90.75	N
ATOM	7971	CA	ALA	A1015	19.359	36.812	25.859	1.00	90.75	C
ATOM	7972	C	ALA	A1015	18.312	37.625	25.094	1.00	90.75	C
ATOM	7973	CB	ALA	A1015	18.797	35.406	26.203	1.00	90.75	C
ATOM	7974	O	ALA	A1015	17.531	38.375	25.703	1.00	90.75	O
ATOM	7975	N	LEU	A1016	18.438	37.719	23.844	1.00	91.00	N
ATOM	7976	CA	LEU	A1016	17.438	38.250	22.922	1.00	91.00	C
ATOM	7977	C	LEU	A1016	16.938	37.156	21.969	1.00	91.00	C
ATOM	7978	CB	LEU	A1016	18.000	39.406	22.125	1.00	91.00	C
ATOM	7979	O	LEU	A1016	17.750	36.438	21.391	1.00	91.00	O
ATOM	7980	CG	LEU	A1016	17.047	40.094	21.141	1.00	91.00	C
ATOM	7981	CD1	LEU	A1016	17.328	41.594	21.094	1.00	91.00	C
ATOM	7982	CD2	LEU	A1016	17.172	39.500	19.750	1.00	91.00	C
ATOM	7983	N	ALA	A1017	15.625	37.062	21.875	1.00	90.00	N
ATOM	7984	CA	ALA	A1017	15.016	36.094	20.984	1.00	90.00	C
ATOM	7985	C	ALA	A1017	14.000	36.750	20.047	1.00	90.00	C
ATOM	7986	CB	ALA	A1017	14.344	34.969	21.766	1.00	90.00	C
ATOM	7987	O	ALA	A1017	13.102	37.469	20.516	1.00	90.00	O
ATOM	7988	N	GLN	A1018	14.234	36.625	18.781	1.00	92.25	N
ATOM	7989	CA	GLN	A1018	13.336	37.125	17.750	1.00	92.25	C
ATOM	7990	C	GLN	A1018	12.742	36.000	16.938	1.00	92.25	C
ATOM	7991	CB	GLN	A1018	14.062	38.094	16.828	1.00	92.25	C
ATOM	7992	O	GLN	A1018	13.461	35.062	16.531	1.00	92.25	O
ATOM	7993	CG	GLN	A1018	13.172	38.750	15.773	1.00	92.25	C
ATOM	7994	CD	GLN	A1018	13.922	39.719	14.875	1.00	92.25	C
ATOM	7995	NE2	GLN	A1018	13.172	40.531	14.133	1.00	92.25	N
ATOM	7996	OE1	GLN	A1018	15.156	39.750	14.852	1.00	92.25	O

ATOM	7997	N	LYS	A1019	11.359	36.062	16.656	1.00	90.81	N
ATOM	7998	CA	LYS	A1019	10.695	35.000	15.922	1.00	90.81	C
ATOM	7999	C	LYS	A1019	9.820	35.562	14.805	1.00	90.81	C
ATOM	8000	CB	LYS	A1019	9.852	34.125	16.859	1.00	90.81	C
ATOM	8001	O	LYS	A1019	9.031	36.469	15.031	1.00	90.81	O
ATOM	8002	CG	LYS	A1019	9.242	32.906	16.203	1.00	90.81	C
ATOM	8003	CD	LYS	A1019	8.664	31.938	17.234	1.00	90.81	C
ATOM	8004	CE	LYS	A1019	8.164	30.656	16.594	1.00	90.81	C
ATOM	8005	NZ	LYS	A1019	8.031	29.547	17.594	1.00	90.81	N
ATOM	8006	N	LEU	A1020	10.062	35.031	13.586	1.00	91.25	N
ATOM	8007	CA	LEU	A1020	9.188	35.219	12.438	1.00	91.25	C
ATOM	8008	C	LEU	A1020	8.555	33.906	12.000	1.00	91.25	C
ATOM	8009	CB	LEU	A1020	9.961	35.844	11.273	1.00	91.25	C
ATOM	8010	O	LEU	A1020	9.266	32.906	11.789	1.00	91.25	O
ATOM	8011	CG	LEU	A1020	10.664	37.156	11.555	1.00	91.25	C
ATOM	8012	CD1	LEU	A1020	11.758	37.438	10.523	1.00	91.25	C
ATOM	8013	CD2	LEU	A1020	9.664	38.312	11.570	1.00	91.25	C
ATOM	8014	N	ASP	A1021	7.273	33.875	11.914	1.00	89.88	N
ATOM	8015	CA	ASP	A1021	6.555	32.656	11.578	1.00	89.88	C
ATOM	8016	C	ASP	A1021	5.344	32.969	10.688	1.00	89.88	C
ATOM	8017	CB	ASP	A1021	6.109	31.922	12.844	1.00	89.88	C
ATOM	8018	O	ASP	A1021	4.551	33.844	11.000	1.00	89.88	O
ATOM	8019	CG	ASP	A1021	5.785	30.469	12.602	1.00	89.88	C
ATOM	8020	OD1	ASP	A1021	6.055	29.953	11.492	1.00	89.88	O
ATOM	8021	OD2	ASP	A1021	5.254	29.812	13.531	1.00	89.88	O
ATOM	8022	N	SER	A1022	5.344	32.375	9.516	1.00	88.38	N
ATOM	8023	CA	SER	A1022	4.191	32.406	8.617	1.00	88.38	C
ATOM	8024	C	SER	A1022	3.752	31.000	8.234	1.00	88.38	C
ATOM	8025	CB	SER	A1022	4.516	33.219	7.359	1.00	88.38	C
ATOM	8026	O	SER	A1022	4.582	30.141	7.914	1.00	88.38	O
ATOM	8027	OG	SER	A1022	5.605	32.625	6.660	1.00	88.38	O
ATOM	8028	N	TRP	A1023	2.410	30.750	8.406	1.00	90.12	N
ATOM	8029	CA	TRP	A1023	1.970	29.406	8.102	1.00	90.12	C
ATOM	8030	C	TRP	A1023	0.591	29.406	7.449	1.00	90.12	C
ATOM	8031	CB	TRP	A1023	1.944	28.547	9.367	1.00	90.12	C
ATOM	8032	O	TRP	A1023	-0.151	30.391	7.566	1.00	90.12	O
ATOM	8033	CG	TRP	A1023	0.862	28.922	10.336	1.00	90.12	C
ATOM	8034	CD1	TRP	A1023	-0.380	28.359	10.438	1.00	90.12	C
ATOM	8035	CD2	TRP	A1023	0.925	29.938	11.328	1.00	90.12	C
ATOM	8036	CE2	TRP	A1023	-0.317	29.938	12.008	1.00	90.12	C
ATOM	8037	CE3	TRP	A1023	1.911	30.859	11.719	1.00	90.12	C
ATOM	8038	NE1	TRP	A1023	-1.094	28.969	11.445	1.00	90.12	N
ATOM	8039	CH2	TRP	A1023	0.385	31.719	13.414	1.00	90.12	C
ATOM	8040	CZ2	TRP	A1023	-0.597	30.828	13.047	1.00	90.12	C
ATOM	8041	CZ3	TRP	A1023	1.630	31.734	12.758	1.00	90.12	C
ATOM	8042	N	SER	A1024	0.295	28.406	6.598	1.00	84.19	N
ATOM	8043	CA	SER	A1024	-1.016	28.125	6.023	1.00	84.19	C
ATOM	8044	C	SER	A1024	-1.288	26.625	5.988	1.00	84.19	C
ATOM	8045	CB	SER	A1024	-1.122	28.703	4.609	1.00	84.19	C
ATOM	8046	O	SER	A1024	-0.532	25.859	5.379	1.00	84.19	O
ATOM	8047	OG	SER	A1024	-2.424	28.500	4.086	1.00	84.19	O
ATOM	8048	N	ASN	A1025	-2.309	26.250	6.723	1.00	82.31	N
ATOM	8049	CA	ASN	A1025	-2.701	24.844	6.707	1.00	82.31	C
ATOM	8050	C	ASN	A1025	-4.219	24.688	6.660	1.00	82.31	C
ATOM	8051	CB	ASN	A1025	-2.129	24.109	7.922	1.00	82.31	C
ATOM	8052	O	ASN	A1025	-4.941	25.656	6.430	1.00	82.31	O
ATOM	8053	CG	ASN	A1025	-2.594	24.703	9.234	1.00	82.31	C
ATOM	8054	ND2	ASN	A1025	-1.679	24.828	10.188	1.00	82.31	N
ATOM	8055	OD1	ASN	A1025	-3.768	25.062	9.391	1.00	82.31	O
ATOM	8056	N	ALA	A1026	-4.809	23.469	6.824	1.00	75.94	N
ATOM	8057	CA	ALA	A1026	-6.230	23.188	6.656	1.00	75.94	C
ATOM	8058	C	ALA	A1026	-7.066	23.891	7.727	1.00	75.94	C
ATOM	8059	CB	ALA	A1026	-6.477	21.688	6.695	1.00	75.94	C
ATOM	8060	O	ALA	A1026	-8.211	24.266	7.477	1.00	75.94	O

```
ATOM   8061  N   ASP A1027      -6.367  24.234   8.820  1.00 78.56           N
ATOM   8062  CA  ASP A1027      -7.105  24.688   9.992  1.00 78.56           C
ATOM   8063  C   ASP A1027      -6.984  26.203  10.164  1.00 78.56           C
ATOM   8064  CB  ASP A1027      -6.605  23.984  11.250  1.00 78.56           C
ATOM   8065  O   ASP A1027      -7.906  26.844  10.664  1.00 78.56           O
ATOM   8066  CG  ASP A1027      -6.840  22.484  11.219  1.00 78.56           C
ATOM   8067  OD1 ASP A1027      -7.887  22.031  10.703  1.00 78.56           O
ATOM   8068  OD2 ASP A1027      -5.969  21.734  11.719  1.00 78.56           O
ATOM   8069  N   SER A1028      -5.762  26.812   9.672  1.00 84.19           N
ATOM   8070  CA  SER A1028      -5.516  28.219   9.992  1.00 84.19           C
ATOM   8071  C   SER A1028      -4.445  28.812   9.078  1.00 84.19           C
ATOM   8072  CB  SER A1028      -5.098  28.375  11.453  1.00 84.19           C
ATOM   8073  O   SER A1028      -3.688  28.078   8.445  1.00 84.19           O
ATOM   8074  OG  SER A1028      -3.811  27.812  11.672  1.00 84.19           O
ATOM   8075  N   THR A1029      -4.555  29.984   8.867  1.00 86.69           N
ATOM   8076  CA  THR A1029      -3.543  30.797   8.203  1.00 86.69           C
ATOM   8077  C   THR A1029      -3.127  31.969   9.094  1.00 86.69           C
ATOM   8078  CB  THR A1029      -4.051  31.344   6.852  1.00 86.69           C
ATOM   8079  O   THR A1029      -3.979  32.656   9.648  1.00 86.69           O
ATOM   8080  CG2 THR A1029      -2.963  32.125   6.121  1.00 86.69           C
ATOM   8081  OG1 THR A1029      -4.469  30.234   6.035  1.00 86.69           O
ATOM   8082  N   GLY A1030      -1.729  32.062   9.266  1.00 88.62           N
ATOM   8083  CA  GLY A1030      -1.364  33.125  10.172  1.00 88.62           C
ATOM   8084  C   GLY A1030       0.066  33.625   9.992  1.00 88.62           C
ATOM   8085  O   GLY A1030       0.847  32.969   9.273  1.00 88.62           O
ATOM   8086  N   ALA A1031       0.340  34.812  10.422  1.00 88.88           N
ATOM   8087  CA  ALA A1031       1.671  35.406  10.484  1.00 88.88           C
ATOM   8088  C   ALA A1031       1.952  35.969  11.883  1.00 88.88           C
ATOM   8089  CB  ALA A1031       1.813  36.500   9.438  1.00 88.88           C
ATOM   8090  O   ALA A1031       1.056  36.500  12.531  1.00 88.88           O
ATOM   8091  N   MET A1032       3.188  35.719  12.320  1.00 91.56           N
ATOM   8092  CA  MET A1032       3.549  36.188  13.664  1.00 91.56           C
ATOM   8093  C   MET A1032       4.941  36.812  13.672  1.00 91.56           C
ATOM   8094  CB  MET A1032       3.494  35.000  14.656  1.00 91.56           C
ATOM   8095  O   MET A1032       5.820  36.375  12.914  1.00 91.56           O
ATOM   8096  CG  MET A1032       4.000  35.375  16.047  1.00 91.56           C
ATOM   8097  SD  MET A1032       4.090  33.906  17.156  1.00 91.56           S
ATOM   8098  CE  MET A1032       5.836  33.469  16.938  1.00 91.56           C
ATOM   8099  N   TYR A1033       5.066  37.906  14.445  1.00 92.25           N
ATOM   8100  CA  TYR A1033       6.328  38.562  14.734  1.00 92.25           C
ATOM   8101  C   TYR A1033       6.504  38.781  16.234  1.00 92.25           C
ATOM   8102  CB  TYR A1033       6.410  39.906  14.016  1.00 92.25           C
ATOM   8103  O   TYR A1033       5.602  39.281  16.906  1.00 92.25           O
ATOM   8104  CG  TYR A1033       7.613  40.750  14.398  1.00 92.25           C
ATOM   8105  CD1 TYR A1033       7.473  41.906  15.156  1.00 92.25           C
ATOM   8106  CD2 TYR A1033       8.891  40.375  14.000  1.00 92.25           C
ATOM   8107  CE1 TYR A1033       8.578  42.656  15.516  1.00 92.25           C
ATOM   8108  CE2 TYR A1033      10.000  41.125  14.352  1.00 92.25           C
ATOM   8109  OH  TYR A1033      10.938  43.031  15.453  1.00 92.25           O
ATOM   8110  CZ  TYR A1033       9.836  42.281  15.109  1.00 92.25           C
ATOM   8111  N   GLY A1034       7.621  38.281  16.812  1.00 92.00           N
ATOM   8112  CA  GLY A1034       7.855  38.469  18.234  1.00 92.00           C
ATOM   8113  C   GLY A1034       9.297  38.781  18.562  1.00 92.00           C
ATOM   8114  O   GLY A1034      10.219  38.312  17.906  1.00 92.00           O
ATOM   8115  N   VAL A1035       9.492  39.750  19.531  1.00 92.38           N
ATOM   8116  CA  VAL A1035      10.805  40.062  20.094  1.00 92.38           C
ATOM   8117  C   VAL A1035      10.758  39.969  21.609  1.00 92.38           C
ATOM   8118  CB  VAL A1035      11.297  41.469  19.656  1.00 92.38           C
ATOM   8119  O   VAL A1035       9.820  40.469  22.250  1.00 92.38           O
ATOM   8120  CG1 VAL A1035      10.422  42.562  20.281  1.00 92.38           C
ATOM   8121  CG2 VAL A1035      12.758  41.656  20.062  1.00 92.38           C
ATOM   8122  N   LYS A1036      11.742  39.219  22.156  1.00 92.94           N
ATOM   8123  CA  LYS A1036      11.766  39.031  23.609  1.00 92.94           C
ATOM   8124  C   LYS A1036      13.164  39.250  24.172  1.00 92.94           C
```

ATOM	8125	CB	LYS	A1036	11.258	37.625	23.984	1.00	92.94	C
ATOM	8126	O	LYS	A1036	14.141	38.719	23.625	1.00	92.94	O
ATOM	8127	CG	LYS	A1036	11.016	37.438	25.469	1.00	92.94	C
ATOM	8128	CD	LYS	A1036	9.898	36.438	25.734	1.00	92.94	C
ATOM	8129	CE	LYS	A1036	9.641	36.250	27.219	1.00	92.94	C
ATOM	8130	NZ	LYS	A1036	8.477	35.344	27.484	1.00	92.94	N
ATOM	8131	N	LEU	A1037	13.242	40.000	25.172	1.00	90.75	N
ATOM	8132	CA	LEU	A1037	14.477	40.219	25.938	1.00	90.75	C
ATOM	8133	C	LEU	A1037	14.438	39.500	27.266	1.00	90.75	C
ATOM	8134	CB	LEU	A1037	14.688	41.719	26.172	1.00	90.75	C
ATOM	8135	O	LEU	A1037	13.430	39.531	27.969	1.00	90.75	O
ATOM	8136	CG	LEU	A1037	14.594	42.625	24.938	1.00	90.75	C
ATOM	8137	CD1	LEU	A1037	14.625	44.094	25.344	1.00	90.75	C
ATOM	8138	CD2	LEU	A1037	15.719	42.312	23.953	1.00	90.75	C
ATOM	8139	N	GLY	A1038	15.469	38.688	27.578	1.00	90.50	N
ATOM	8140	CA	GLY	A1038	15.578	37.938	28.844	1.00	90.50	C
ATOM	8141	C	GLY	A1038	16.875	38.250	29.578	1.00	90.50	C
ATOM	8142	O	GLY	A1038	17.922	38.375	28.969	1.00	90.50	O
ATOM	8143	N	LEU	A1039	16.781	38.500	30.844	1.00	88.94	N
ATOM	8144	CA	LEU	A1039	17.922	38.750	31.703	1.00	88.94	C
ATOM	8145	C	LEU	A1039	17.875	37.875	32.969	1.00	88.94	C
ATOM	8146	CB	LEU	A1039	17.969	40.219	32.094	1.00	88.94	C
ATOM	8147	O	LEU	A1039	16.859	37.844	33.656	1.00	88.94	O
ATOM	8148	CG	LEU	A1039	19.172	40.656	32.969	1.00	88.94	C
ATOM	8149	CD1	LEU	A1039	20.438	40.625	32.125	1.00	88.94	C
ATOM	8150	CD2	LEU	A1039	18.938	42.031	33.562	1.00	88.94	C
ATOM	8151	N	LYS	A1040	18.828	36.906	33.062	1.00	86.00	N
ATOM	8152	CA	LYS	A1040	19.078	36.188	34.312	1.00	86.00	C
ATOM	8153	C	LYS	A1040	20.203	36.844	35.094	1.00	86.00	C
ATOM	8154	CB	LYS	A1040	19.406	34.719	34.031	1.00	86.00	C
ATOM	8155	O	LYS	A1040	21.359	36.844	34.656	1.00	86.00	O
ATOM	8156	CG	LYS	A1040	19.234	33.812	35.219	1.00	86.00	C
ATOM	8157	CD	LYS	A1040	19.391	32.344	34.812	1.00	86.00	C
ATOM	8158	CE	LYS	A1040	19.266	31.422	36.031	1.00	86.00	C
ATOM	8159	NZ	LYS	A1040	19.406	29.984	35.656	1.00	86.00	N
ATOM	8160	N	TYR	A1041	19.922	37.594	36.094	1.00	83.19	N
ATOM	8161	CA	TYR	A1041	20.859	38.344	36.906	1.00	83.19	C
ATOM	8162	C	TYR	A1041	20.766	37.906	38.375	1.00	83.19	C
ATOM	8163	CB	TYR	A1041	20.562	39.844	36.812	1.00	83.19	C
ATOM	8164	O	TYR	A1041	19.719	38.031	39.000	1.00	83.19	O
ATOM	8165	CG	TYR	A1041	21.562	40.719	37.531	1.00	83.19	C
ATOM	8166	CD1	TYR	A1041	21.234	41.375	38.688	1.00	83.19	C
ATOM	8167	CD2	TYR	A1041	22.859	40.844	37.031	1.00	83.19	C
ATOM	8168	CE1	TYR	A1041	22.156	42.188	39.375	1.00	83.19	C
ATOM	8169	CE2	TYR	A1041	23.797	41.656	37.688	1.00	83.19	C
ATOM	8170	OH	TYR	A1041	24.344	43.094	39.500	1.00	83.19	O
ATOM	8171	CZ	TYR	A1041	23.438	42.312	38.844	1.00	83.19	C
ATOM	8172	N	ASN	A1042	21.797	37.188	38.875	1.00	78.00	N
ATOM	8173	CA	ASN	A1042	21.922	36.719	40.250	1.00	78.00	C
ATOM	8174	C	ASN	A1042	20.812	35.688	40.594	1.00	78.00	C
ATOM	8175	CB	ASN	A1042	21.875	37.875	41.219	1.00	78.00	C
ATOM	8176	O	ASN	A1042	20.234	35.750	41.656	1.00	78.00	O
ATOM	8177	CG	ASN	A1042	23.156	38.688	41.219	1.00	78.00	C
ATOM	8178	ND2	ASN	A1042	23.078	39.938	41.688	1.00	78.00	N
ATOM	8179	OD1	ASN	A1042	24.219	38.219	40.781	1.00	78.00	O
ATOM	8180	N	GLY	A1043	20.422	34.938	39.625	1.00	75.25	N
ATOM	8181	CA	GLY	A1043	19.500	33.844	39.875	1.00	75.25	C
ATOM	8182	C	GLY	A1043	18.062	34.219	39.594	1.00	75.25	C
ATOM	8183	O	GLY	A1043	17.188	33.344	39.594	1.00	75.25	O
ATOM	8184	N	GLN	A1044	17.781	35.531	39.406	1.00	80.56	N
ATOM	8185	CA	GLN	A1044	16.422	36.000	39.156	1.00	80.56	C
ATOM	8186	C	GLN	A1044	16.219	36.312	37.688	1.00	80.56	C
ATOM	8187	CB	GLN	A1044	16.125	37.250	40.000	1.00	80.56	C
ATOM	8188	O	GLN	A1044	17.094	36.875	37.031	1.00	80.56	O

ATOM	8189	CG	GLN	A1044	15.734	36.938	41.438	1.00	80.56	C
ATOM	8190	CD	GLN	A1044	15.297	38.188	42.188	1.00	80.56	C
ATOM	8191	NE2	GLN	A1044	14.727	37.969	43.375	1.00	80.56	N
ATOM	8192	OE1	GLN	A1044	15.469	39.312	41.719	1.00	80.56	O
ATOM	8193	N	GLU	A1045	15.117	36.031	37.062	1.00	85.56	N
ATOM	8194	CA	GLU	A1045	14.836	36.125	35.625	1.00	85.56	C
ATOM	8195	C	GLU	A1045	13.961	37.344	35.344	1.00	85.56	C
ATOM	8196	CB	GLU	A1045	14.164	34.875	35.094	1.00	85.56	C
ATOM	8197	O	GLU	A1045	12.977	37.594	36.031	1.00	85.56	O
ATOM	8198	CG	GLU	A1045	15.078	33.656	35.094	1.00	85.56	C
ATOM	8199	CD	GLU	A1045	14.406	32.406	34.531	1.00	85.56	C
ATOM	8200	OE1	GLU	A1045	15.078	31.359	34.406	1.00	85.56	O
ATOM	8201	OE2	GLU	A1045	13.203	32.469	34.219	1.00	85.56	O
ATOM	8202	N	TYR	A1046	14.367	38.188	34.469	1.00	86.69	N
ATOM	8203	CA	TYR	A1046	13.609	39.344	34.000	1.00	86.69	C
ATOM	8204	C	TYR	A1046	13.320	39.250	32.500	1.00	86.69	C
ATOM	8205	CB	TYR	A1046	14.375	40.656	34.281	1.00	86.69	C
ATOM	8206	O	TYR	A1046	14.125	38.688	31.750	1.00	86.69	O
ATOM	8207	CG	TYR	A1046	14.711	40.844	35.750	1.00	86.69	C
ATOM	8208	CD1	TYR	A1046	13.859	41.562	36.594	1.00	86.69	C
ATOM	8209	CD2	TYR	A1046	15.875	40.312	36.281	1.00	86.69	C
ATOM	8210	CE1	TYR	A1046	14.164	41.750	37.938	1.00	86.69	C
ATOM	8211	CE2	TYR	A1046	16.188	40.500	37.625	1.00	86.69	C
ATOM	8212	OH	TYR	A1046	15.633	41.406	39.781	1.00	86.69	O
ATOM	8213	CZ	TYR	A1046	15.328	41.219	38.438	1.00	86.69	C
ATOM	8214	N	SER	A1047	12.117	39.750	32.094	1.00	89.50	N
ATOM	8215	CA	SER	A1047	11.789	39.625	30.672	1.00	89.50	C
ATOM	8216	C	SER	A1047	11.008	40.844	30.188	1.00	89.50	C
ATOM	8217	CB	SER	A1047	10.992	38.344	30.406	1.00	89.50	C
ATOM	8218	O	SER	A1047	10.328	41.500	30.969	1.00	89.50	O
ATOM	8219	OG	SER	A1047	9.758	38.375	31.094	1.00	89.50	O
ATOM	8220	N	ALA	A1048	11.266	41.281	29.047	1.00	89.25	N
ATOM	8221	CA	ALA	A1048	10.477	42.250	28.281	1.00	89.25	C
ATOM	8222	C	ALA	A1048	10.328	41.812	26.828	1.00	89.25	C
ATOM	8223	CB	ALA	A1048	11.109	43.625	28.359	1.00	89.25	C
ATOM	8224	O	ALA	A1048	11.281	41.344	26.219	1.00	89.25	O
ATOM	8225	N	GLY	A1049	9.086	41.906	26.297	1.00	91.75	N
ATOM	8226	CA	GLY	A1049	8.945	41.438	24.922	1.00	91.75	C
ATOM	8227	C	GLY	A1049	7.648	41.906	24.281	1.00	91.75	C
ATOM	8228	O	GLY	A1049	6.785	42.469	24.938	1.00	91.75	O
ATOM	8229	N	MET	A1050	7.578	41.812	22.953	1.00	93.25	N
ATOM	8230	CA	MET	A1050	6.398	42.094	22.156	1.00	93.25	C
ATOM	8231	C	MET	A1050	6.102	40.969	21.188	1.00	93.25	C
ATOM	8232	CB	MET	A1050	6.594	43.406	21.375	1.00	93.25	C
ATOM	8233	O	MET	A1050	7.016	40.375	20.594	1.00	93.25	O
ATOM	8234	CG	MET	A1050	5.449	43.750	20.422	1.00	93.25	C
ATOM	8235	SD	MET	A1050	5.746	45.281	19.469	1.00	93.25	S
ATOM	8236	CE	MET	A1050	6.934	44.656	18.234	1.00	93.25	C
ATOM	8237	N	ALA	A1051	4.816	40.625	21.109	1.00	92.25	N
ATOM	8238	CA	ALA	A1051	4.355	39.656	20.141	1.00	92.25	C
ATOM	8239	C	ALA	A1051	3.180	40.188	19.328	1.00	92.25	C
ATOM	8240	CB	ALA	A1051	3.963	38.344	20.859	1.00	92.25	C
ATOM	8241	O	ALA	A1051	2.184	40.625	19.891	1.00	92.25	O
ATOM	8242	N	MET	A1052	3.402	40.156	17.969	1.00	92.00	N
ATOM	8243	CA	MET	A1052	2.359	40.531	17.016	1.00	92.00	C
ATOM	8244	C	MET	A1052	1.880	39.312	16.219	1.00	92.00	C
ATOM	8245	CB	MET	A1052	2.867	41.625	16.078	1.00	92.00	C
ATOM	8246	O	MET	A1052	2.689	38.594	15.664	1.00	92.00	O
ATOM	8247	CG	MET	A1052	1.770	42.281	15.266	1.00	92.00	C
ATOM	8248	SD	MET	A1052	2.365	43.781	14.359	1.00	92.00	S
ATOM	8249	CE	MET	A1052	3.531	43.000	13.203	1.00	92.00	C
ATOM	8250	N	SER	A1053	0.582	39.062	16.219	1.00	91.50	N
ATOM	8251	CA	SER	A1053	0.069	37.906	15.484	1.00	91.50	C
ATOM	8252	C	SER	A1053	-1.231	38.250	14.758	1.00	91.50	C

ATOM	8253	CB	SER	A1053	-0.156	36.750	16.438	1.00	91.50	C
ATOM	8254	O	SER	A1053	-2.033	39.062	15.250	1.00	91.50	O
ATOM	8255	OG	SER	A1053	-1.164	37.031	17.375	1.00	91.50	O
ATOM	8256	N	LEU	A1054	-1.437	37.750	13.461	1.00	89.62	N
ATOM	8257	CA	LEU	A1054	-2.637	37.750	12.633	1.00	89.62	C
ATOM	8258	C	LEU	A1054	-3.027	36.344	12.219	1.00	89.62	C
ATOM	8259	CB	LEU	A1054	-2.418	38.625	11.383	1.00	89.62	C
ATOM	8260	O	LEU	A1054	-2.340	35.719	11.414	1.00	89.62	O
ATOM	8261	CG	LEU	A1054	-3.678	39.062	10.625	1.00	89.62	C
ATOM	8262	CD1	LEU	A1054	-4.477	40.062	11.453	1.00	89.62	C
ATOM	8263	CD2	LEU	A1054	-3.311	39.656	9.273	1.00	89.62	C
ATOM	8264	N	VAL	A1055	-4.012	35.844	12.844	1.00	88.00	N
ATOM	8265	CA	VAL	A1055	-4.344	34.438	12.656	1.00	88.00	C
ATOM	8266	C	VAL	A1055	-5.805	34.281	12.242	1.00	88.00	C
ATOM	8267	CB	VAL	A1055	-4.074	33.594	13.945	1.00	88.00	C
ATOM	8268	O	VAL	A1055	-6.699	34.812	12.906	1.00	88.00	O
ATOM	8269	CG1	VAL	A1055	-4.379	32.125	13.711	1.00	88.00	C
ATOM	8270	CG2	VAL	A1055	-2.631	33.781	14.398	1.00	88.00	C
ATOM	8271	N	GLY	A1056	-6.055	33.625	11.078	1.00	82.62	N
ATOM	8272	CA	GLY	A1056	-7.398	33.281	10.633	1.00	82.62	C
ATOM	8273	C	GLY	A1056	-7.746	31.812	10.836	1.00	82.62	C
ATOM	8274	O	GLY	A1056	-6.973	30.938	10.461	1.00	82.62	O
ATOM	8275	N	SER	A1057	-8.711	31.562	11.578	1.00	74.25	N
ATOM	8276	CA	SER	A1057	-9.266	30.234	11.750	1.00	74.25	C
ATOM	8277	C	SER	A1057	-10.789	30.234	11.625	1.00	74.25	C
ATOM	8278	CB	SER	A1057	-8.859	29.656	13.109	1.00	74.25	C
ATOM	8279	O	SER	A1057	-11.469	31.000	12.320	1.00	74.25	O
ATOM	8280	OG	SER	A1057	-9.547	28.438	13.375	1.00	74.25	O
ATOM	8281	N	GLY	A1058	-11.414	29.484	10.609	1.00	70.00	N
ATOM	8282	CA	GLY	A1058	-12.836	29.547	10.328	1.00	70.00	C
ATOM	8283	C	GLY	A1058	-13.266	30.891	9.766	1.00	70.00	C
ATOM	8284	O	GLY	A1058	-12.703	31.375	8.773	1.00	70.00	O
ATOM	8285	N	ALA	A1059	-14.344	31.562	10.242	1.00	71.00	N
ATOM	8286	CA	ALA	A1059	-14.836	32.844	9.758	1.00	71.00	C
ATOM	8287	C	ALA	A1059	-14.203	34.000	10.531	1.00	71.00	C
ATOM	8288	CB	ALA	A1059	-16.359	32.906	9.875	1.00	71.00	C
ATOM	8289	O	ALA	A1059	-14.305	35.156	10.125	1.00	71.00	O
ATOM	8290	N	ALA	A1060	-13.250	33.812	11.508	1.00	75.44	N
ATOM	8291	CA	ALA	A1060	-12.781	34.906	12.375	1.00	75.44	C
ATOM	8292	C	ALA	A1060	-11.281	35.125	12.227	1.00	75.44	C
ATOM	8293	CB	ALA	A1060	-13.141	34.625	13.828	1.00	75.44	C
ATOM	8294	O	ALA	A1060	-10.516	34.156	12.133	1.00	75.44	O
ATOM	8295	N	VAL	A1061	-10.836	36.344	11.930	1.00	82.69	N
ATOM	8296	CA	VAL	A1061	-9.453	36.812	11.898	1.00	82.69	C
ATOM	8297	C	VAL	A1061	-9.148	37.594	13.164	1.00	82.69	C
ATOM	8298	CB	VAL	A1061	-9.164	37.656	10.641	1.00	82.69	C
ATOM	8299	O	VAL	A1061	-9.906	38.500	13.531	1.00	82.69	O
ATOM	8300	CG1	VAL	A1061	-7.711	38.125	10.625	1.00	82.69	C
ATOM	8301	CG2	VAL	A1061	-9.484	36.844	9.375	1.00	82.69	C
ATOM	8302	N	LYS	A1062	-8.117	37.156	13.859	1.00	85.94	N
ATOM	8303	CA	LYS	A1062	-7.715	37.906	15.062	1.00	85.94	C
ATOM	8304	C	LYS	A1062	-6.348	38.531	14.883	1.00	85.94	C
ATOM	8305	CB	LYS	A1062	-7.699	36.969	16.266	1.00	85.94	C
ATOM	8306	O	LYS	A1062	-5.367	37.875	14.570	1.00	85.94	O
ATOM	8307	CG	LYS	A1062	-9.078	36.469	16.703	1.00	85.94	C
ATOM	8308	CD	LYS	A1062	-9.008	35.656	17.984	1.00	85.94	C
ATOM	8309	CE	LYS	A1062	-10.383	35.156	18.406	1.00	85.94	C
ATOM	8310	NZ	LYS	A1062	-10.312	34.312	19.641	1.00	85.94	N
ATOM	8311	N	ALA	A1063	-6.332	39.875	14.969	1.00	86.31	N
ATOM	8312	CA	ALA	A1063	-5.105	40.688	14.984	1.00	86.31	C
ATOM	8313	C	ALA	A1063	-4.738	41.094	16.406	1.00	86.31	C
ATOM	8314	CB	ALA	A1063	-5.250	41.906	14.078	1.00	86.31	C
ATOM	8315	O	ALA	A1063	-5.539	41.750	17.078	1.00	86.31	O
ATOM	8316	N	GLN	A1064	-3.512	40.594	16.922	1.00	88.69	N

```
ATOM   8317  CA  GLN A1064      -3.156  40.844  18.312  1.00 88.69           C
ATOM   8318  C   GLN A1064      -1.714  41.344  18.438  1.00 88.69           C
ATOM   8319  CB  GLN A1064      -3.355  39.562  19.141  1.00 88.69           C
ATOM   8320  O   GLN A1064      -0.816  40.812  17.781  1.00 88.69           O
ATOM   8321  CG  GLN A1064      -4.816  39.188  19.312  1.00 88.69           C
ATOM   8322  CD  GLN A1064      -4.988  37.938  20.156  1.00 88.69           C
ATOM   8323  NE2 GLN A1064      -6.191  37.719  20.672  1.00 88.69           N
ATOM   8324  OE1 GLN A1064      -4.047  37.156  20.328  1.00 88.69           O
ATOM   8325  N   ILE A1065      -1.528  42.406  19.266  1.00 91.00           N
ATOM   8326  CA  ILE A1065      -0.200  42.844  19.688  1.00 91.00           C
ATOM   8327  C   ILE A1065      -0.081  42.781  21.203  1.00 91.00           C
ATOM   8328  CB  ILE A1065       0.100  44.281  19.188  1.00 91.00           C
ATOM   8329  O   ILE A1065      -0.844  43.438  21.922  1.00 91.00           O
ATOM   8330  CG1 ILE A1065      -0.139  44.406  17.672  1.00 91.00           C
ATOM   8331  CG2 ILE A1065       1.533  44.688  19.547  1.00 91.00           C
ATOM   8332  CD1 ILE A1065       0.045  45.781  17.109  1.00 91.00           C
ATOM   8333  N   LEU A1066       0.839  41.969  21.750  1.00 91.12           N
ATOM   8334  CA  LEU A1066       0.997  41.750  23.188  1.00 91.12           C
ATOM   8335  C   LEU A1066       2.336  42.312  23.672  1.00 91.12           C
ATOM   8336  CB  LEU A1066       0.886  40.281  23.531  1.00 91.12           C
ATOM   8337  O   LEU A1066       3.363  42.094  23.031  1.00 91.12           O
ATOM   8338  CG  LEU A1066      -0.377  39.562  23.047  1.00 91.12           C
ATOM   8339  CD1 LEU A1066      -0.237  38.062  23.219  1.00 91.12           C
ATOM   8340  CD2 LEU A1066      -1.604  40.062  23.781  1.00 91.12           C
ATOM   8341  N   PHE A1067       2.316  43.062  24.781  1.00 90.50           N
ATOM   8342  CA  PHE A1067       3.527  43.594  25.391  1.00 90.50           C
ATOM   8343  C   PHE A1067       3.721  43.000  26.781  1.00 90.50           C
ATOM   8344  CB  PHE A1067       3.473  45.125  25.484  1.00 90.50           C
ATOM   8345  O   PHE A1067       2.760  42.844  27.547  1.00 90.50           O
ATOM   8346  CG  PHE A1067       3.439  45.781  24.141  1.00 90.50           C
ATOM   8347  CD1 PHE A1067       4.621  46.094  23.469  1.00 90.50           C
ATOM   8348  CD2 PHE A1067       2.229  46.125  23.547  1.00 90.50           C
ATOM   8349  CE1 PHE A1067       4.594  46.719  22.234  1.00 90.50           C
ATOM   8350  CE2 PHE A1067       2.195  46.750  22.312  1.00 90.50           C
ATOM   8351  CZ  PHE A1067       3.377  47.062  21.656  1.00 90.50           C
ATOM   8352  N   GLU A1068       4.934  42.469  27.062  1.00 89.62           N
ATOM   8353  CA  GLU A1068       5.383  42.125  28.406  1.00 89.62           C
ATOM   8354  C   GLU A1068       6.504  43.062  28.875  1.00 89.62           C
ATOM   8355  CB  GLU A1068       5.848  40.688  28.469  1.00 89.62           C
ATOM   8356  O   GLU A1068       7.570  43.094  28.266  1.00 89.62           O
ATOM   8357  CG  GLU A1068       6.273  40.219  29.844  1.00 89.62           C
ATOM   8358  CD  GLU A1068       6.922  38.844  29.844  1.00 89.62           C
ATOM   8359  OE1 GLU A1068       7.145  38.281  30.938  1.00 89.62           O
ATOM   8360  OE2 GLU A1068       7.207  38.312  28.750  1.00 89.62           O
ATOM   8361  N   ALA A1069       6.211  43.844  29.844  1.00 86.94           N
ATOM   8362  CA  ALA A1069       7.215  44.750  30.375  1.00 86.94           C
ATOM   8363  C   ALA A1069       6.812  45.250  31.766  1.00 86.94           C
ATOM   8364  CB  ALA A1069       7.422  45.938  29.438  1.00 86.94           C
ATOM   8365  O   ALA A1069       5.625  45.344  32.094  1.00 86.94           O
ATOM   8366  N   SER A1070       7.805  45.438  32.688  1.00 84.19           N
ATOM   8367  CA  SER A1070       7.543  46.031  33.969  1.00 84.19           C
ATOM   8368  C   SER A1070       7.051  47.469  33.844  1.00 84.19           C
ATOM   8369  CB  SER A1070       8.805  46.000  34.844  1.00 84.19           C
ATOM   8370  O   SER A1070       6.266  47.938  34.688  1.00 84.19           O
ATOM   8371  OG  SER A1070       9.219  44.656  35.094  1.00 84.19           O
ATOM   8372  N   ARG A1071       7.383  48.219  32.812  1.00 87.50           N
ATOM   8373  CA  ARG A1071       6.992  49.594  32.500  1.00 87.50           C
ATOM   8374  C   ARG A1071       6.805  49.750  30.984  1.00 87.50           C
ATOM   8375  CB  ARG A1071       8.039  50.594  33.000  1.00 87.50           C
ATOM   8376  O   ARG A1071       7.680  49.406  30.188  1.00 87.50           O
ATOM   8377  CG  ARG A1071       7.668  52.031  32.781  1.00 87.50           C
ATOM   8378  CD  ARG A1071       8.766  52.969  33.219  1.00 87.50           C
ATOM   8379  NE  ARG A1071       8.930  52.938  34.688  1.00 87.50           N
ATOM   8380  NH1 ARG A1071      10.711  54.406  34.719  1.00 87.50           N
```

ATOM	8381	NH2	ARG	A1071	9.914	53.500	36.688	1.00	87.50	N
ATOM	8382	CZ	ARG	A1071	9.852	53.625	35.375	1.00	87.50	C
ATOM	8383	N	PHE	A1072	5.637	50.094	30.625	1.00	91.31	N
ATOM	8384	CA	PHE	A1072	5.328	50.438	29.250	1.00	91.31	C
ATOM	8385	C	PHE	A1072	4.828	51.875	29.141	1.00	91.31	C
ATOM	8386	CB	PHE	A1072	4.281	49.469	28.672	1.00	91.31	C
ATOM	8387	O	PHE	A1072	3.947	52.281	29.891	1.00	91.31	O
ATOM	8388	CG	PHE	A1072	3.836	49.812	27.281	1.00	91.31	C
ATOM	8389	CD1	PHE	A1072	2.656	50.531	27.062	1.00	91.31	C
ATOM	8390	CD2	PHE	A1072	4.602	49.438	26.188	1.00	91.31	C
ATOM	8391	CE1	PHE	A1072	2.242	50.844	25.766	1.00	91.31	C
ATOM	8392	CE2	PHE	A1072	4.195	49.750	24.891	1.00	91.31	C
ATOM	8393	CZ	PHE	A1072	3.014	50.469	24.688	1.00	91.31	C
ATOM	8394	N	ALA	A1073	5.461	52.719	28.203	1.00	88.88	N
ATOM	8395	CA	ALA	A1073	5.059	54.125	28.078	1.00	88.88	C
ATOM	8396	C	ALA	A1073	5.023	54.562	26.609	1.00	88.88	C
ATOM	8397	CB	ALA	A1073	6.004	55.031	28.875	1.00	88.88	C
ATOM	8398	O	ALA	A1073	5.801	54.062	25.797	1.00	88.88	O
ATOM	8399	N	ILE	A1074	3.975	55.219	26.234	1.00	87.81	N
ATOM	8400	CA	ILE	A1074	3.941	55.938	24.969	1.00	87.81	C
ATOM	8401	C	ILE	A1074	4.398	57.375	25.188	1.00	87.81	C
ATOM	8402	CB	ILE	A1074	2.529	55.906	24.344	1.00	87.81	C
ATOM	8403	O	ILE	A1074	3.879	58.062	26.078	1.00	87.81	O
ATOM	8404	CG1	ILE	A1074	2.086	54.469	24.094	1.00	87.81	C
ATOM	8405	CG2	ILE	A1074	2.500	56.719	23.047	1.00	87.81	C
ATOM	8406	CD1	ILE	A1074	0.676	54.344	23.531	1.00	87.81	C
ATOM	8407	N	MET	A1075	5.500	57.844	24.500	1.00	84.69	N
ATOM	8408	CA	MET	A1075	6.098	59.156	24.703	1.00	84.69	C
ATOM	8409	C	MET	A1075	6.012	60.000	23.438	1.00	84.69	C
ATOM	8410	CB	MET	A1075	7.559	59.000	25.141	1.00	84.69	C
ATOM	8411	O	MET	A1075	6.035	59.438	22.328	1.00	84.69	O
ATOM	8412	CG	MET	A1075	8.453	58.375	24.094	1.00	84.69	C
ATOM	8413	SD	MET	A1075	10.188	58.188	24.672	1.00	84.69	S
ATOM	8414	CE	MET	A1075	10.891	59.750	24.062	1.00	84.69	C
ATOM	8415	N	THR	A1076	5.621	61.156	23.516	1.00	80.62	N
ATOM	8416	CA	THR	A1076	5.680	62.094	22.406	1.00	80.62	C
ATOM	8417	C	THR	A1076	6.656	63.219	22.703	1.00	80.62	C
ATOM	8418	CB	THR	A1076	4.289	62.688	22.094	1.00	80.62	C
ATOM	8419	O	THR	A1076	6.754	63.688	23.844	1.00	80.62	O
ATOM	8420	CG2	THR	A1076	3.186	61.656	22.391	1.00	80.62	C
ATOM	8421	OG1	THR	A1076	4.082	63.844	22.891	1.00	80.62	O
ATOM	8422	N	GLY	A1077	7.570	63.406	21.844	1.00	70.56	N
ATOM	8423	CA	GLY	A1077	8.562	64.438	21.953	1.00	70.56	C
ATOM	8424	C	GLY	A1077	8.266	65.625	21.062	1.00	70.56	C
ATOM	8425	O	GLY	A1077	8.102	65.500	19.844	1.00	70.56	O
ATOM	8426	N	MET	A1078	7.414	66.562	21.359	1.00	64.69	N
ATOM	8427	CA	MET	A1078	7.316	67.812	20.594	1.00	64.69	C
ATOM	8428	C	MET	A1078	7.996	69.000	21.328	1.00	64.69	C
ATOM	8429	CB	MET	A1078	5.852	68.188	20.312	1.00	64.69	C
ATOM	8430	O	MET	A1078	7.785	69.188	22.531	1.00	64.69	O
ATOM	8431	CG	MET	A1078	5.297	67.500	19.047	1.00	64.69	C
ATOM	8432	SD	MET	A1078	3.514	67.875	18.812	1.00	64.69	S
ATOM	8433	CE	MET	A1078	3.570	69.688	18.594	1.00	64.69	C
ATOM	8434	N	ASN	A1079	9.094	69.688	20.734	1.00	63.69	N
ATOM	8435	CA	ASN	A1079	9.805	70.875	21.156	1.00	63.69	C
ATOM	8436	C	ASN	A1079	10.648	70.625	22.406	1.00	63.69	C
ATOM	8437	CB	ASN	A1079	8.820	72.000	21.391	1.00	63.69	C
ATOM	8438	O	ASN	A1079	10.656	71.438	23.328	1.00	63.69	O
ATOM	8439	CG	ASN	A1079	8.242	72.562	20.109	1.00	63.69	C
ATOM	8440	ND2	ASN	A1079	7.125	73.312	20.219	1.00	63.69	N
ATOM	8441	OD1	ASN	A1079	8.797	72.375	19.016	1.00	63.69	O
ATOM	8442	N	GLY	A1080	11.258	69.438	22.688	1.00	74.44	N
ATOM	8443	CA	GLY	A1080	12.188	69.250	23.781	1.00	74.44	C
ATOM	8444	C	GLY	A1080	11.508	68.812	25.062	1.00	74.44	C

ATOM	8445	O	GLY	A1080	12.172	68.562	26.078	1.00	74.44	O
ATOM	8446	N	GLN	A1081	10.078	68.812	25.094	1.00	76.69	N
ATOM	8447	CA	GLN	A1081	9.352	68.375	26.266	1.00	76.69	C
ATOM	8448	C	GLN	A1081	8.719	67.000	26.047	1.00	76.69	C
ATOM	8449	CB	GLN	A1081	8.273	69.375	26.656	1.00	76.69	C
ATOM	8450	O	GLN	A1081	8.016	66.812	25.047	1.00	76.69	O
ATOM	8451	CG	GLN	A1081	8.828	70.750	27.047	1.00	76.69	C
ATOM	8452	CD	GLN	A1081	9.500	70.750	28.406	1.00	76.69	C
ATOM	8453	NE2	GLN	A1081	10.539	71.562	28.547	1.00	76.69	N
ATOM	8454	OE1	GLN	A1081	9.094	70.000	29.312	1.00	76.69	O
ATOM	8455	N	THR	A1082	9.195	66.000	26.734	1.00	81.75	N
ATOM	8456	CA	THR	A1082	8.680	64.625	26.688	1.00	81.75	C
ATOM	8457	C	THR	A1082	7.387	64.500	27.484	1.00	81.75	C
ATOM	8458	CB	THR	A1082	9.711	63.625	27.234	1.00	81.75	C
ATOM	8459	O	THR	A1082	7.316	64.938	28.625	1.00	81.75	O
ATOM	8460	CG2	THR	A1082	9.242	62.188	27.016	1.00	81.75	C
ATOM	8461	OG1	THR	A1082	10.961	63.812	26.547	1.00	81.75	O
ATOM	8462	N	GLN	A1083	6.348	64.250	26.797	1.00	84.00	N
ATOM	8463	CA	GLN	A1083	5.070	63.938	27.438	1.00	84.00	C
ATOM	8464	C	GLN	A1083	4.730	62.469	27.375	1.00	84.00	C
ATOM	8465	CB	GLN	A1083	3.951	64.750	26.797	1.00	84.00	C
ATOM	8466	O	GLN	A1083	5.055	61.781	26.406	1.00	84.00	O
ATOM	8467	CG	GLN	A1083	4.109	66.250	26.984	1.00	84.00	C
ATOM	8468	CD	GLN	A1083	3.006	67.062	26.297	1.00	84.00	C
ATOM	8469	NE2	GLN	A1083	3.371	67.812	25.281	1.00	84.00	N
ATOM	8470	OE1	GLN	A1083	1.836	67.000	26.688	1.00	84.00	O
ATOM	8471	N	TYR	A1084	4.148	61.875	28.453	1.00	86.12	N
ATOM	8472	CA	TYR	A1084	3.707	60.500	28.547	1.00	86.12	C
ATOM	8473	C	TYR	A1084	2.188	60.406	28.625	1.00	86.12	C
ATOM	8474	CB	TYR	A1084	4.332	59.812	29.766	1.00	86.12	C
ATOM	8475	O	TYR	A1084	1.618	60.312	29.703	1.00	86.12	O
ATOM	8476	CG	TYR	A1084	5.840	59.781	29.734	1.00	86.12	C
ATOM	8477	CD1	TYR	A1084	6.508	58.781	29.016	1.00	86.12	C
ATOM	8478	CD2	TYR	A1084	6.602	60.719	30.406	1.00	86.12	C
ATOM	8479	CE1	TYR	A1084	7.895	58.719	28.969	1.00	86.12	C
ATOM	8480	CE2	TYR	A1084	7.992	60.656	30.391	1.00	86.12	C
ATOM	8481	OH	TYR	A1084	10.008	59.625	29.641	1.00	86.12	O
ATOM	8482	CZ	TYR	A1084	8.625	59.688	29.672	1.00	86.12	C
ATOM	8483	N	PRO	A1085	1.690	60.531	27.531	1.00	85.00	N
ATOM	8484	CA	PRO	A1085	0.230	60.469	27.594	1.00	85.00	C
ATOM	8485	C	PRO	A1085	-0.257	59.125	28.172	1.00	85.00	C
ATOM	8486	CB	PRO	A1085	-0.204	60.625	26.141	1.00	85.00	C
ATOM	8487	O	PRO	A1085	-1.330	59.062	28.781	1.00	85.00	O
ATOM	8488	CG	PRO	A1085	1.031	60.344	25.344	1.00	85.00	C
ATOM	8489	CD	PRO	A1085	2.229	60.625	26.203	1.00	85.00	C
ATOM	8490	N	PHE	A1086	0.574	57.938	28.094	1.00	90.00	N
ATOM	8491	CA	PHE	A1086	0.189	56.594	28.516	1.00	90.00	C
ATOM	8492	C	PHE	A1086	1.372	55.875	29.125	1.00	90.00	C
ATOM	8493	CB	PHE	A1086	-0.363	55.812	27.328	1.00	90.00	C
ATOM	8494	O	PHE	A1086	2.414	55.719	28.484	1.00	90.00	O
ATOM	8495	CG	PHE	A1086	-0.887	54.438	27.703	1.00	90.00	C
ATOM	8496	CD1	PHE	A1086	-0.032	53.344	27.781	1.00	90.00	C
ATOM	8497	CD2	PHE	A1086	-2.236	54.250	27.969	1.00	90.00	C
ATOM	8498	CE1	PHE	A1086	-0.515	52.094	28.125	1.00	90.00	C
ATOM	8499	CE2	PHE	A1086	-2.727	53.000	28.312	1.00	90.00	C
ATOM	8500	CZ	PHE	A1086	-1.863	51.906	28.391	1.00	90.00	C
ATOM	8501	N	VAL	A1087	1.253	55.594	30.422	1.00	91.06	N
ATOM	8502	CA	VAL	A1087	2.279	54.844	31.125	1.00	91.06	C
ATOM	8503	C	VAL	A1087	1.626	53.719	31.922	1.00	91.06	C
ATOM	8504	CB	VAL	A1087	3.129	55.719	32.062	1.00	91.06	C
ATOM	8505	O	VAL	A1087	0.633	53.938	32.625	1.00	91.06	O
ATOM	8506	CG1	VAL	A1087	4.180	54.906	32.781	1.00	91.06	C
ATOM	8507	CG2	VAL	A1087	3.793	56.844	31.250	1.00	91.06	C
ATOM	8508	N	VAL	A1088	2.111	52.469	31.672	1.00	90.06	N

ATOM	8509	CA	VAL	A1088	1.774	51.375	32.562	1.00	90.06	C
ATOM	8510	C	VAL	A1088	2.959	51.062	33.469	1.00	90.06	C
ATOM	8511	CB	VAL	A1088	1.367	50.094	31.781	1.00	90.06	C
ATOM	8512	O	VAL	A1088	4.059	50.781	33.000	1.00	90.06	O
ATOM	8513	CG1	VAL	A1088	0.988	48.969	32.719	1.00	90.06	C
ATOM	8514	CG2	VAL	A1088	0.212	50.406	30.828	1.00	90.06	C
ATOM	8515	N	GLU	A1089	2.795	51.281	34.688	1.00	88.69	N
ATOM	8516	CA	GLU	A1089	3.834	51.062	35.688	1.00	88.69	C
ATOM	8517	C	GLU	A1089	3.242	50.500	36.969	1.00	88.69	C
ATOM	8518	CB	GLU	A1089	4.605	52.344	35.938	1.00	88.69	C
ATOM	8519	O	GLU	A1089	2.287	51.094	37.500	1.00	88.69	O
ATOM	8520	CG	GLU	A1089	5.793	52.156	36.875	1.00	88.69	C
ATOM	8521	CD	GLU	A1089	6.668	53.406	36.969	1.00	88.69	C
ATOM	8522	OE1	GLU	A1089	7.852	53.281	37.375	1.00	88.69	O
ATOM	8523	OE2	GLU	A1089	6.172	54.500	36.656	1.00	88.69	O
ATOM	8524	N	ASN	A1090	3.883	49.406	37.500	1.00	81.62	N
ATOM	8525	CA	ASN	A1090	3.490	48.750	38.719	1.00	81.62	C
ATOM	8526	C	ASN	A1090	2.020	48.344	38.719	1.00	81.62	C
ATOM	8527	CB	ASN	A1090	3.771	49.656	39.938	1.00	81.62	C
ATOM	8528	O	ASN	A1090	1.303	48.531	39.688	1.00	81.62	O
ATOM	8529	CG	ASN	A1090	5.250	49.781	40.219	1.00	81.62	C
ATOM	8530	ND2	ASN	A1090	5.629	50.875	40.875	1.00	81.62	N
ATOM	8531	OD1	ASN	A1090	6.047	48.906	39.875	1.00	81.62	O
ATOM	8532	N	GLY	A1091	1.567	47.969	37.438	1.00	80.00	N
ATOM	8533	CA	GLY	A1091	0.202	47.469	37.344	1.00	80.00	C
ATOM	8534	C	GLY	A1091	-0.822	48.594	37.219	1.00	80.00	C
ATOM	8535	O	GLY	A1091	-2.027	48.344	37.219	1.00	80.00	O
ATOM	8536	N	GLN	A1092	-0.338	49.844	37.219	1.00	85.38	N
ATOM	8537	CA	GLN	A1092	-1.210	51.031	37.125	1.00	85.38	C
ATOM	8538	C	GLN	A1092	-1.055	51.719	35.781	1.00	85.38	C
ATOM	8539	CB	GLN	A1092	-0.917	52.000	38.250	1.00	85.38	C
ATOM	8540	O	GLN	A1092	0.061	51.875	35.281	1.00	85.38	O
ATOM	8541	CG	GLN	A1092	-1.322	51.500	39.625	1.00	85.38	C
ATOM	8542	CD	GLN	A1092	-1.138	52.531	40.719	1.00	85.38	C
ATOM	8543	NE2	GLN	A1092	-1.859	52.344	41.812	1.00	85.38	N
ATOM	8544	OE1	GLN	A1092	-0.353	53.469	40.562	1.00	85.38	O
ATOM	8545	N	VAL	A1093	-2.168	51.969	35.156	1.00	89.25	N
ATOM	8546	CA	VAL	A1093	-2.154	52.812	33.969	1.00	89.25	C
ATOM	8547	C	VAL	A1093	-2.199	54.281	34.344	1.00	89.25	C
ATOM	8548	CB	VAL	A1093	-3.336	52.469	33.031	1.00	89.25	C
ATOM	8549	O	VAL	A1093	-3.086	54.719	35.094	1.00	89.25	O
ATOM	8550	CG1	VAL	A1093	-3.328	53.406	31.797	1.00	89.25	C
ATOM	8551	CG2	VAL	A1093	-3.279	51.000	32.594	1.00	89.25	C
ATOM	8552	N	ILE	A1094	-1.165	55.062	33.969	1.00	88.88	N
ATOM	8553	CA	ILE	A1094	-1.086	56.500	34.250	1.00	88.88	C
ATOM	8554	C	ILE	A1094	-1.370	57.281	32.969	1.00	88.88	C
ATOM	8555	CB	ILE	A1094	0.294	56.875	34.844	1.00	88.88	C
ATOM	8556	O	ILE	A1094	-0.654	57.156	31.984	1.00	88.88	O
ATOM	8557	CG1	ILE	A1094	0.600	56.062	36.094	1.00	88.88	C
ATOM	8558	CG2	ILE	A1094	0.351	58.375	35.125	1.00	88.88	C
ATOM	8559	CD1	ILE	A1094	2.057	56.125	36.531	1.00	88.88	C
ATOM	8560	N	LEU	A1095	-2.529	58.031	32.906	1.00	88.88	N
ATOM	8561	CA	LEU	A1095	-2.910	58.875	31.781	1.00	88.88	C
ATOM	8562	C	LEU	A1095	-2.793	60.344	32.125	1.00	88.88	C
ATOM	8563	CB	LEU	A1095	-4.340	58.531	31.328	1.00	88.88	C
ATOM	8564	O	LEU	A1095	-3.324	60.781	33.156	1.00	88.88	O
ATOM	8565	CG	LEU	A1095	-4.582	57.125	30.812	1.00	88.88	C
ATOM	8566	CD1	LEU	A1095	-6.055	56.750	30.938	1.00	88.88	C
ATOM	8567	CD2	LEU	A1095	-4.125	57.031	29.359	1.00	88.88	C
ATOM	8568	N	SER	A1096	-1.990	61.219	31.328	1.00	84.31	N
ATOM	8569	CA	SER	A1096	-1.858	62.688	31.531	1.00	84.31	C
ATOM	8570	C	SER	A1096	-3.146	63.406	31.172	1.00	84.31	C
ATOM	8571	CB	SER	A1096	-0.696	63.219	30.703	1.00	84.31	C
ATOM	8572	O	SER	A1096	-3.506	64.375	31.797	1.00	84.31	O

ATOM	8573	OG	SER	A1096	-0.866	62.938	29.328	1.00	84.31	O
ATOM	8574	N	SER	A1097	-3.900	63.000	30.141	1.00	82.81	N
ATOM	8575	CA	SER	A1097	-5.164	63.531	29.625	1.00	82.81	C
ATOM	8576	C	SER	A1097	-5.945	62.469	28.875	1.00	82.81	C
ATOM	8577	CB	SER	A1097	-4.918	64.750	28.719	1.00	82.81	C
ATOM	8578	O	SER	A1097	-5.359	61.656	28.156	1.00	82.81	O
ATOM	8579	OG	SER	A1097	-6.133	65.188	28.156	1.00	82.81	O
ATOM	8580	N	ALA	A1098	-7.254	62.312	29.156	1.00	82.38	N
ATOM	8581	CA	ALA	A1098	-8.086	61.312	28.484	1.00	82.38	C
ATOM	8582	C	ALA	A1098	-9.422	61.938	28.062	1.00	82.38	C
ATOM	8583	CB	ALA	A1098	-8.320	60.125	29.375	1.00	82.38	C
ATOM	8584	O	ALA	A1098	-9.977	62.781	28.766	1.00	82.38	O
ATOM	8585	N	ILE	A1099	-9.812	61.844	26.781	1.00	81.25	N
ATOM	8586	CA	ILE	A1099	-11.180	62.094	26.312	1.00	81.25	C
ATOM	8587	C	ILE	A1099	-11.984	60.781	26.406	1.00	81.25	C
ATOM	8588	CB	ILE	A1099	-11.203	62.625	24.859	1.00	81.25	C
ATOM	8589	O	ILE	A1099	-11.664	59.812	25.750	1.00	81.25	O
ATOM	8590	CG1	ILE	A1099	-10.430	63.938	24.766	1.00	81.25	C
ATOM	8591	CG2	ILE	A1099	-12.641	62.781	24.375	1.00	81.25	C
ATOM	8592	CD1	ILE	A1099	-10.289	64.500	23.359	1.00	81.25	C
ATOM	8593	N	ILE	A1100	-12.891	60.750	27.438	1.00	83.62	N
ATOM	8594	CA	ILE	A1100	-13.680	59.531	27.688	1.00	83.62	C
ATOM	8595	C	ILE	A1100	-15.148	59.812	27.344	1.00	83.62	C
ATOM	8596	CB	ILE	A1100	-13.539	59.062	29.141	1.00	83.62	C
ATOM	8597	O	ILE	A1100	-15.789	60.656	27.984	1.00	83.62	O
ATOM	8598	CG1	ILE	A1100	-12.062	58.812	29.484	1.00	83.62	C
ATOM	8599	CG2	ILE	A1100	-14.367	57.781	29.375	1.00	83.62	C
ATOM	8600	CD1	ILE	A1100	-11.805	58.594	30.969	1.00	83.62	C
ATOM	8601	N	LYS	A1101	-15.547	59.312	26.109	1.00	80.69	N
ATOM	8602	CA	LYS	A1101	-16.953	59.469	25.719	1.00	80.69	C
ATOM	8603	C	LYS	A1101	-17.875	58.875	26.781	1.00	80.69	C
ATOM	8604	CB	LYS	A1101	-17.219	58.781	24.375	1.00	80.69	C
ATOM	8605	O	LYS	A1101	-18.719	59.594	27.328	1.00	80.69	O
ATOM	8606	CG	LYS	A1101	-18.609	59.031	23.812	1.00	80.69	C
ATOM	8607	CD	LYS	A1101	-18.766	58.438	22.422	1.00	80.69	C
ATOM	8608	CE	LYS	A1101	-20.172	58.688	21.875	1.00	80.69	C
ATOM	8609	NZ	LYS	A1101	-20.328	58.094	20.500	1.00	80.69	N
ATOM	8610	N	ASN	A1102	-17.719	57.469	27.125	1.00	78.81	N
ATOM	8611	CA	ASN	A1102	-18.438	56.781	28.172	1.00	78.81	C
ATOM	8612	C	ASN	A1102	-17.500	56.094	29.156	1.00	78.81	C
ATOM	8613	CB	ASN	A1102	-19.406	55.750	27.562	1.00	78.81	C
ATOM	8614	O	ASN	A1102	-16.641	55.312	28.750	1.00	78.81	O
ATOM	8615	CG	ASN	A1102	-20.453	56.375	26.688	1.00	78.81	C
ATOM	8616	ND2	ASN	A1102	-20.766	55.750	25.562	1.00	78.81	N
ATOM	8617	OD1	ASN	A1102	-20.984	57.469	27.000	1.00	78.81	O
ATOM	8618	N	GLY	A1103	-17.438	56.531	30.531	1.00	80.00	N
ATOM	8619	CA	GLY	A1103	-16.531	55.938	31.500	1.00	80.00	C
ATOM	8620	C	GLY	A1103	-17.250	55.188	32.594	1.00	80.00	C
ATOM	8621	O	GLY	A1103	-18.312	55.594	33.062	1.00	80.00	O
ATOM	8622	N	PHE	A1104	-16.844	53.844	32.844	1.00	79.50	N
ATOM	8623	CA	PHE	A1104	-17.266	53.094	34.031	1.00	79.50	C
ATOM	8624	C	PHE	A1104	-16.203	53.188	35.125	1.00	79.50	C
ATOM	8625	CB	PHE	A1104	-17.531	51.625	33.688	1.00	79.50	C
ATOM	8626	O	PHE	A1104	-15.125	52.594	35.000	1.00	79.50	O
ATOM	8627	CG	PHE	A1104	-18.656	51.438	32.688	1.00	79.50	C
ATOM	8628	CD1	PHE	A1104	-19.984	51.438	33.125	1.00	79.50	C
ATOM	8629	CD2	PHE	A1104	-18.391	51.250	31.344	1.00	79.50	C
ATOM	8630	CE1	PHE	A1104	-21.016	51.250	32.219	1.00	79.50	C
ATOM	8631	CE2	PHE	A1104	-19.422	51.062	30.438	1.00	79.50	C
ATOM	8632	CZ	PHE	A1104	-20.734	51.062	30.875	1.00	79.50	C
ATOM	8633	N	ILE	A1105	-16.375	54.031	36.188	1.00	83.56	N
ATOM	8634	CA	ILE	A1105	-15.383	54.344	37.219	1.00	83.56	C
ATOM	8635	C	ILE	A1105	-15.875	53.812	38.562	1.00	83.56	C
ATOM	8636	CB	ILE	A1105	-15.086	55.844	37.312	1.00	83.56	C

ATOM	8637	O	ILE	A1105	-16.938	54.219	39.031	1.00	83.56	O
ATOM	8638	CG1	ILE	A1105	-14.625	56.375	35.938	1.00	83.56	C
ATOM	8639	CG2	ILE	A1105	-14.039	56.125	38.375	1.00	83.56	C
ATOM	8640	CD1	ILE	A1105	-14.570	57.906	35.844	1.00	83.56	C
ATOM	8641	N	THR	A1106	-15.266	52.656	39.031	1.00	82.38	N
ATOM	8642	CA	THR	A1106	-15.617	52.094	40.344	1.00	82.38	C
ATOM	8643	C	THR	A1106	-15.391	53.094	41.438	1.00	82.38	C
ATOM	8644	CB	THR	A1106	-14.797	50.812	40.625	1.00	82.38	C
ATOM	8645	O	THR	A1106	-16.266	53.312	42.281	1.00	82.38	O
ATOM	8646	CG2	THR	A1106	-15.203	50.188	41.969	1.00	82.38	C
ATOM	8647	OG1	THR	A1106	-15.031	49.844	39.594	1.00	82.38	O
ATOM	8648	N	ASN	A1107	-14.188	53.719	41.531	1.00	80.44	N
ATOM	8649	CA	ASN	A1107	-13.820	54.812	42.438	1.00	80.44	C
ATOM	8650	C	ASN	A1107	-13.148	55.969	41.719	1.00	80.44	C
ATOM	8651	CB	ASN	A1107	-12.922	54.281	43.562	1.00	80.44	C
ATOM	8652	O	ASN	A1107	-12.297	55.750	40.844	1.00	80.44	O
ATOM	8653	CG	ASN	A1107	-13.586	53.219	44.406	1.00	80.44	C
ATOM	8654	ND2	ASN	A1107	-13.008	52.000	44.406	1.00	80.44	N
ATOM	8655	OD1	ASN	A1107	-14.625	53.438	45.031	1.00	80.44	O
ATOM	8656	N	ALA	A1108	-13.805	57.156	41.781	1.00	83.19	N
ATOM	8657	CA	ALA	A1108	-13.156	58.344	41.188	1.00	83.19	C
ATOM	8658	C	ALA	A1108	-12.727	59.344	42.281	1.00	83.19	C
ATOM	8659	CB	ALA	A1108	-14.094	59.000	40.188	1.00	83.19	C
ATOM	8660	O	ALA	A1108	-13.508	59.656	43.156	1.00	83.19	O
ATOM	8661	N	MET	A1109	-11.445	59.531	42.344	1.00	83.50	N
ATOM	8662	CA	MET	A1109	-10.969	60.688	43.156	1.00	83.50	C
ATOM	8663	C	MET	A1109	-10.961	61.969	42.312	1.00	83.50	C
ATOM	8664	CB	MET	A1109	-9.570	60.406	43.688	1.00	83.50	C
ATOM	8665	O	MET	A1109	-10.352	62.000	41.250	1.00	83.50	O
ATOM	8666	CG	MET	A1109	-9.516	59.219	44.656	1.00	83.50	C
ATOM	8667	SD	MET	A1109	-7.828	58.938	45.344	1.00	83.50	S
ATOM	8668	CE	MET	A1109	-7.773	60.219	46.625	1.00	83.50	C
ATOM	8669	N	ILE	A1110	-11.820	62.906	42.656	1.00	82.19	N
ATOM	8670	CA	ILE	A1110	-11.961	64.188	41.938	1.00	82.19	C
ATOM	8671	C	ILE	A1110	-10.922	65.188	42.406	1.00	82.19	C
ATOM	8672	CB	ILE	A1110	-13.383	64.750	42.062	1.00	82.19	C
ATOM	8673	O	ILE	A1110	-10.906	65.562	43.594	1.00	82.19	O
ATOM	8674	CG1	ILE	A1110	-14.438	63.688	41.750	1.00	82.19	C
ATOM	8675	CG2	ILE	A1110	-13.555	66.000	41.188	1.00	82.19	C
ATOM	8676	CD1	ILE	A1110	-14.398	63.188	40.312	1.00	82.19	C
ATOM	8677	N	GLY	A1111	-9.914	65.500	41.500	1.00	82.06	N
ATOM	8678	CA	GLY	A1111	-8.891	66.438	41.812	1.00	82.06	C
ATOM	8679	C	GLY	A1111	-9.430	67.875	42.000	1.00	82.06	C
ATOM	8680	O	GLY	A1111	-9.266	68.500	43.062	1.00	82.06	O
ATOM	8681	N	ASN	A1112	-10.148	68.375	41.062	1.00	88.50	N
ATOM	8682	CA	ASN	A1112	-10.711	69.750	41.125	1.00	88.50	C
ATOM	8683	C	ASN	A1112	-12.234	69.688	41.125	1.00	88.50	C
ATOM	8684	CB	ASN	A1112	-10.188	70.625	39.969	1.00	88.50	C
ATOM	8685	O	ASN	A1112	-12.867	70.000	42.125	1.00	88.50	O
ATOM	8686	CG	ASN	A1112	-8.711	70.875	40.094	1.00	88.50	C
ATOM	8687	ND2	ASN	A1112	-8.039	71.062	38.969	1.00	88.50	N
ATOM	8688	OD1	ASN	A1112	-8.164	71.000	41.188	1.00	88.50	O
ATOM	8689	N	PHE	A1113	-12.922	69.312	40.125	1.00	89.94	N
ATOM	8690	CA	PHE	A1113	-14.375	69.250	40.000	1.00	89.94	C
ATOM	8691	C	PHE	A1113	-14.797	68.188	38.969	1.00	89.94	C
ATOM	8692	CB	PHE	A1113	-14.938	70.625	39.594	1.00	89.94	C
ATOM	8693	O	PHE	A1113	-13.977	67.750	38.156	1.00	89.94	O
ATOM	8694	CG	PHE	A1113	-14.328	71.188	38.344	1.00	89.94	C
ATOM	8695	CD1	PHE	A1113	-13.289	72.062	38.406	1.00	89.94	C
ATOM	8696	CD2	PHE	A1113	-14.797	70.812	37.094	1.00	89.94	C
ATOM	8697	CE1	PHE	A1113	-12.727	72.625	37.250	1.00	89.94	C
ATOM	8698	CE2	PHE	A1113	-14.242	71.312	35.938	1.00	89.94	C
ATOM	8699	CZ	PHE	A1113	-13.203	72.188	36.031	1.00	89.94	C
ATOM	8700	N	ILE	A1114	-15.969	67.562	39.188	1.00	92.19	N

ATOM	8701	CA	ILE	A1114	-16.703	66.875	38.125	1.00	92.19	C
ATOM	8702	C	ILE	A1114	-17.938	67.688	37.750	1.00	92.19	C
ATOM	8703	CB	ILE	A1114	-17.125	65.438	38.562	1.00	92.19	C
ATOM	8704	O	ILE	A1114	-18.641	68.188	38.594	1.00	92.19	O
ATOM	8705	CG1	ILE	A1114	-17.719	64.688	37.375	1.00	92.19	C
ATOM	8706	CG2	ILE	A1114	-18.109	65.500	39.750	1.00	92.19	C
ATOM	8707	CD1	ILE	A1114	-17.875	63.156	37.625	1.00	92.19	C
ATOM	8708	N	GLN	A1115	-18.047	67.938	36.406	1.00	91.50	N
ATOM	8709	CA	GLN	A1115	-19.156	68.812	36.000	1.00	91.50	C
ATOM	8710	C	GLN	A1115	-19.766	68.312	34.688	1.00	91.50	C
ATOM	8711	CB	GLN	A1115	-18.688	70.250	35.844	1.00	91.50	C
ATOM	8712	O	GLN	A1115	-19.156	67.562	33.969	1.00	91.50	O
ATOM	8713	CG	GLN	A1115	-17.656	70.438	34.750	1.00	91.50	C
ATOM	8714	CD	GLN	A1115	-17.156	71.875	34.656	1.00	91.50	C
ATOM	8715	NE2	GLN	A1115	-16.344	72.188	33.625	1.00	91.50	N
ATOM	8716	OE1	GLN	A1115	-17.500	72.750	35.469	1.00	91.50	O
ATOM	8717	N	SER	A1116	-21.109	68.625	34.531	1.00	89.25	N
ATOM	8718	CA	SER	A1116	-21.781	68.375	33.281	1.00	89.25	C
ATOM	8719	C	SER	A1116	-21.125	69.188	32.156	1.00	89.25	C
ATOM	8720	CB	SER	A1116	-23.266	68.750	33.375	1.00	89.25	C
ATOM	8721	O	SER	A1116	-20.500	70.250	32.406	1.00	89.25	O
ATOM	8722	OG	SER	A1116	-23.406	70.125	33.656	1.00	89.25	O
ATOM	8723	N	ASN	A1117	-21.156	68.688	30.859	1.00	87.00	N
ATOM	8724	CA	ASN	A1117	-20.562	69.312	29.672	1.00	87.00	C
ATOM	8725	C	ASN	A1117	-21.156	70.750	29.422	1.00	87.00	C
ATOM	8726	CB	ASN	A1117	-20.734	68.438	28.453	1.00	87.00	C
ATOM	8727	O	ASN	A1117	-20.516	71.562	28.812	1.00	87.00	O
ATOM	8728	CG	ASN	A1117	-22.188	68.188	28.125	1.00	87.00	C
ATOM	8729	ND2	ASN	A1117	-22.438	67.438	27.031	1.00	87.00	N
ATOM	8730	OD1	ASN	A1117	-23.094	68.625	28.828	1.00	87.00	O
ATOM	8731	N	ASN	A1118	-22.469	71.062	29.891	1.00	87.88	N
ATOM	8732	CA	ASN	A1118	-23.141	72.312	29.641	1.00	87.88	C
ATOM	8733	C	ASN	A1118	-23.078	73.250	30.859	1.00	87.88	C
ATOM	8734	CB	ASN	A1118	-24.594	72.062	29.234	1.00	87.88	C
ATOM	8735	O	ASN	A1118	-23.812	74.250	30.938	1.00	87.88	O
ATOM	8736	CG	ASN	A1118	-25.422	71.438	30.344	1.00	87.88	C
ATOM	8737	ND2	ASN	A1118	-26.734	71.625	30.266	1.00	87.88	N
ATOM	8738	OD1	ASN	A1118	-24.875	70.812	31.250	1.00	87.88	O
ATOM	8739	N	TYR	A1119	-22.109	72.812	31.938	1.00	88.31	N
ATOM	8740	CA	TYR	A1119	-22.094	73.625	33.156	1.00	88.31	C
ATOM	8741	C	TYR	A1119	-21.656	75.062	32.906	1.00	88.31	C
ATOM	8742	CB	TYR	A1119	-21.156	73.000	34.188	1.00	88.31	C
ATOM	8743	O	TYR	A1119	-20.641	75.312	32.250	1.00	88.31	O
ATOM	8744	CG	TYR	A1119	-20.953	73.812	35.438	1.00	88.31	C
ATOM	8745	CD1	TYR	A1119	-19.797	74.562	35.625	1.00	88.31	C
ATOM	8746	CD2	TYR	A1119	-21.906	73.812	36.438	1.00	88.31	C
ATOM	8747	CE1	TYR	A1119	-19.609	75.312	36.750	1.00	88.31	C
ATOM	8748	CE2	TYR	A1119	-21.734	74.625	37.594	1.00	88.31	C
ATOM	8749	OH	TYR	A1119	-20.391	76.125	38.875	1.00	88.31	O
ATOM	8750	CZ	TYR	A1119	-20.578	75.375	37.719	1.00	88.31	C
ATOM	8751	N	VAL	A1120	-22.406	75.875	33.062	1.00	86.62	N
ATOM	8752	CA	VAL	A1120	-22.172	77.312	33.125	1.00	86.62	C
ATOM	8753	C	VAL	A1120	-22.609	77.875	34.469	1.00	86.62	C
ATOM	8754	CB	VAL	A1120	-22.906	78.062	31.984	1.00	86.62	C
ATOM	8755	O	VAL	A1120	-23.766	77.688	34.875	1.00	86.62	O
ATOM	8756	CG1	VAL	A1120	-22.625	79.562	32.031	1.00	86.62	C
ATOM	8757	CG2	VAL	A1120	-22.516	77.500	30.625	1.00	86.62	C
ATOM	8758	N	PHE	A1121	-21.656	78.312	35.312	1.00	87.12	N
ATOM	8759	CA	PHE	A1121	-21.922	78.812	36.656	1.00	87.12	C
ATOM	8760	C	PHE	A1121	-23.203	79.625	36.688	1.00	87.12	C
ATOM	8761	CB	PHE	A1121	-20.734	79.688	37.156	1.00	87.12	C
ATOM	8762	O	PHE	A1121	-23.422	80.500	35.844	1.00	87.12	O
ATOM	8763	CG	PHE	A1121	-20.922	80.188	38.562	1.00	87.12	C
ATOM	8764	CD1	PHE	A1121	-21.203	81.562	38.750	1.00	87.12	C

```
ATOM   8765  CD2 PHE A1121     -20.812  79.375  39.656  1.00 87.12           C
ATOM   8766  CE1 PHE A1121     -21.359  82.062  40.031  1.00 87.12           C
ATOM   8767  CE2 PHE A1121     -20.969  79.875  40.938  1.00 87.12           C
ATOM   8768  CZ  PHE A1121     -21.250  81.188  41.125  1.00 87.12           C
ATOM   8769  N   ASN A1122     -24.141  79.312  37.594  1.00 83.31           N
ATOM   8770  CA  ASN A1122     -25.375  80.062  37.875  1.00 83.31           C
ATOM   8771  C   ASN A1122     -26.312  80.062  36.688  1.00 83.31           C
ATOM   8772  CB  ASN A1122     -25.047  81.500  38.281  1.00 83.31           C
ATOM   8773  O   ASN A1122     -27.281  80.812  36.656  1.00 83.31           O
ATOM   8774  CG  ASN A1122     -25.984  82.000  39.344  1.00 83.31           C
ATOM   8775  ND2 ASN A1122     -26.000  83.312  39.531  1.00 83.31           N
ATOM   8776  OD1 ASN A1122     -26.703  81.250  40.000  1.00 83.31           O
ATOM   8777  N   GLN A1123     -26.141  79.250  35.625  1.00 84.62           N
ATOM   8778  CA  GLN A1123     -26.969  79.312  34.438  1.00 84.62           C
ATOM   8779  C   GLN A1123     -27.484  77.875  34.094  1.00 84.62           C
ATOM   8780  CB  GLN A1123     -26.203  79.875  33.250  1.00 84.62           C
ATOM   8781  O   GLN A1123     -28.688  77.688  33.969  1.00 84.62           O
ATOM   8782  CG  GLN A1123     -25.984  81.375  33.312  1.00 84.62           C
ATOM   8783  CD  GLN A1123     -25.281  81.938  32.094  1.00 84.62           C
ATOM   8784  NE2 GLN A1123     -24.547  83.000  32.312  1.00 84.62           N
ATOM   8785  OE1 GLN A1123     -25.391  81.375  31.000  1.00 84.62           O
ATOM   8786  N   SER A1124     -26.578  77.000  33.844  1.00 86.94           N
ATOM   8787  CA  SER A1124     -26.938  75.625  33.375  1.00 86.94           C
ATOM   8788  C   SER A1124     -25.906  74.625  33.812  1.00 86.94           C
ATOM   8789  CB  SER A1124     -27.094  75.625  31.859  1.00 86.94           C
ATOM   8790  O   SER A1124     -24.766  74.938  34.125  1.00 86.94           O
ATOM   8791  OG  SER A1124     -25.859  76.000  31.234  1.00 86.94           O
ATOM   8792  N   GLY A1125     -26.375  73.438  34.062  1.00 89.75           N
ATOM   8793  CA  GLY A1125     -25.484  72.312  34.312  1.00 89.75           C
ATOM   8794  C   GLY A1125     -25.250  72.125  35.812  1.00 89.75           C
ATOM   8795  O   GLY A1125     -25.875  72.750  36.656  1.00 89.75           O
ATOM   8796  N   TRP A1126     -24.578  71.062  36.156  1.00 91.38           N
ATOM   8797  CA  TRP A1126     -24.266  70.750  37.531  1.00 91.38           C
ATOM   8798  C   TRP A1126     -22.750  70.625  37.719  1.00 91.38           C
ATOM   8799  CB  TRP A1126     -24.969  69.438  38.000  1.00 91.38           C
ATOM   8800  O   TRP A1126     -22.031  70.312  36.781  1.00 91.38           O
ATOM   8801  CG  TRP A1126     -24.625  68.250  37.188  1.00 91.38           C
ATOM   8802  CD1 TRP A1126     -25.266  67.812  36.062  1.00 91.38           C
ATOM   8803  CD2 TRP A1126     -23.531  67.375  37.406  1.00 91.38           C
ATOM   8804  CE2 TRP A1126     -23.594  66.375  36.406  1.00 91.38           C
ATOM   8805  CE3 TRP A1126     -22.531  67.312  38.375  1.00 91.38           C
ATOM   8806  NE1 TRP A1126     -24.656  66.688  35.594  1.00 91.38           N
ATOM   8807  CH2 TRP A1126     -21.688  65.250  37.281  1.00 91.38           C
ATOM   8808  CZ2 TRP A1126     -22.672  65.312  36.344  1.00 91.38           C
ATOM   8809  CZ3 TRP A1126     -21.609  66.250  38.312  1.00 91.38           C
ATOM   8810  N   ARG A1127     -22.234  71.000  38.781  1.00 93.12           N
ATOM   8811  CA  ARG A1127     -20.844  70.875  39.188  1.00 93.12           C
ATOM   8812  C   ARG A1127     -20.703  70.438  40.625  1.00 93.12           C
ATOM   8813  CB  ARG A1127     -20.109  72.250  38.969  1.00 93.12           C
ATOM   8814  O   ARG A1127     -21.391  71.000  41.500  1.00 93.12           O
ATOM   8815  CG  ARG A1127     -18.656  72.188  39.406  1.00 93.12           C
ATOM   8816  CD  ARG A1127     -17.984  73.562  39.188  1.00 93.12           C
ATOM   8817  NE  ARG A1127     -16.578  73.562  39.625  1.00 93.12           N
ATOM   8818  NH1 ARG A1127     -16.109  75.688  38.844  1.00 93.12           N
ATOM   8819  NH2 ARG A1127     -14.477  74.438  39.875  1.00 93.12           N
ATOM   8820  CZ  ARG A1127     -15.719  74.562  39.438  1.00 93.12           C
ATOM   8821  N   LEU A1128     -19.875  69.438  40.844  1.00 91.88           N
ATOM   8822  CA  LEU A1128     -19.453  69.000  42.188  1.00 91.88           C
ATOM   8823  C   LEU A1128     -17.969  69.312  42.406  1.00 91.88           C
ATOM   8824  CB  LEU A1128     -19.734  67.562  42.438  1.00 91.88           C
ATOM   8825  O   LEU A1128     -17.125  68.688  41.750  1.00 91.88           O
ATOM   8826  CG  LEU A1128     -19.312  67.000  43.781  1.00 91.88           C
ATOM   8827  CD1 LEU A1128     -20.062  67.688  44.906  1.00 91.88           C
ATOM   8828  CD2 LEU A1128     -19.547  65.500  43.844  1.00 91.88           C
```

```
ATOM   8829  N   ASP A1129     -17.719  70.188  43.219  1.00 90.19           N
ATOM   8830  CA  ASP A1129     -16.359  70.625  43.500  1.00 90.19           C
ATOM   8831  C   ASP A1129     -15.719  69.750  44.594  1.00 90.19           C
ATOM   8832  CB  ASP A1129     -16.328  72.062  43.969  1.00 90.19           C
ATOM   8833  O   ASP A1129     -16.422  69.188  45.438  1.00 90.19           O
ATOM   8834  CG  ASP A1129     -14.930  72.625  44.125  1.00 90.19           C
ATOM   8835  OD1 ASP A1129     -14.367  72.562  45.219  1.00 90.19           O
ATOM   8836  OD2 ASP A1129     -14.383  73.125  43.094  1.00 90.19           O
ATOM   8837  N   LYS A1130     -14.398  69.625  44.531  1.00 89.31           N
ATOM   8838  CA  LYS A1130     -13.680  68.812  45.500  1.00 89.31           C
ATOM   8839  C   LYS A1130     -13.953  69.312  46.938  1.00 89.31           C
ATOM   8840  CB  LYS A1130     -12.180  68.812  45.219  1.00 89.31           C
ATOM   8841  O   LYS A1130     -13.812  68.562  47.906  1.00 89.31           O
ATOM   8842  CG  LYS A1130     -11.523  70.188  45.469  1.00 89.31           C
ATOM   8843  CD  LYS A1130     -10.016  70.125  45.250  1.00 89.31           C
ATOM   8844  CE  LYS A1130      -9.359  71.500  45.500  1.00 89.31           C
ATOM   8845  NZ  LYS A1130      -7.871  71.375  45.406  1.00 89.31           N
ATOM   8846  N   GLY A1131     -14.391  70.625  47.125  1.00 88.12           N
ATOM   8847  CA  GLY A1131     -14.656  71.250  48.406  1.00 88.12           C
ATOM   8848  C   GLY A1131     -16.016  70.938  48.969  1.00 88.12           C
ATOM   8849  O   GLY A1131     -16.344  71.250  50.094  1.00 88.12           O
ATOM   8850  N   GLY A1132     -16.828  70.062  48.250  1.00 84.81           N
ATOM   8851  CA  GLY A1132     -18.094  69.562  48.781  1.00 84.81           C
ATOM   8852  C   GLY A1132     -19.281  70.375  48.312  1.00 84.81           C
ATOM   8853  O   GLY A1132     -20.422  70.125  48.719  1.00 84.81           O
ATOM   8854  N   THR A1133     -19.078  71.375  47.500  1.00 91.44           N
ATOM   8855  CA  THR A1133     -20.188  72.188  47.000  1.00 91.44           C
ATOM   8856  C   THR A1133     -20.781  71.562  45.750  1.00 91.44           C
ATOM   8857  CB  THR A1133     -19.719  73.625  46.656  1.00 91.44           C
ATOM   8858  O   THR A1133     -20.078  71.312  44.781  1.00 91.44           O
ATOM   8859  CG2 THR A1133     -20.891  74.500  46.188  1.00 91.44           C
ATOM   8860  OG1 THR A1133     -19.156  74.250  47.844  1.00 91.44           O
ATOM   8861  N   PHE A1134     -22.031  71.188  45.906  1.00 93.06           N
ATOM   8862  CA  PHE A1134     -22.797  70.750  44.750  1.00 93.06           C
ATOM   8863  C   PHE A1134     -23.750  71.812  44.250  1.00 93.06           C
ATOM   8864  CB  PHE A1134     -23.578  69.438  45.094  1.00 93.06           C
ATOM   8865  O   PHE A1134     -24.594  72.312  45.000  1.00 93.06           O
ATOM   8866  CG  PHE A1134     -24.391  68.938  43.969  1.00 93.06           C
ATOM   8867  CD1 PHE A1134     -25.766  68.750  44.094  1.00 93.06           C
ATOM   8868  CD2 PHE A1134     -23.781  68.562  42.750  1.00 93.06           C
ATOM   8869  CE1 PHE A1134     -26.516  68.250  43.031  1.00 93.06           C
ATOM   8870  CE2 PHE A1134     -24.531  68.062  41.719  1.00 93.06           C
ATOM   8871  CZ  PHE A1134     -25.891  67.875  41.844  1.00 93.06           C
ATOM   8872  N   GLU A1135     -23.469  72.188  43.000  1.00 91.12           N
ATOM   8873  CA  GLU A1135     -24.328  73.188  42.344  1.00 91.12           C
ATOM   8874  C   GLU A1135     -25.047  72.562  41.125  1.00 91.12           C
ATOM   8875  CB  GLU A1135     -23.531  74.375  41.906  1.00 91.12           C
ATOM   8876  O   GLU A1135     -24.422  71.875  40.312  1.00 91.12           O
ATOM   8877  CG  GLU A1135     -22.734  75.062  43.000  1.00 91.12           C
ATOM   8878  CD  GLU A1135     -21.812  76.125  42.531  1.00 91.12           C
ATOM   8879  OE1 GLU A1135     -21.281  76.875  43.375  1.00 91.12           O
ATOM   8880  OE2 GLU A1135     -21.609  76.250  41.312  1.00 91.12           O
ATOM   8881  N   ASN A1136     -26.406  72.750  41.188  1.00 91.62           N
ATOM   8882  CA  ASN A1136     -27.297  72.250  40.125  1.00 91.62           C
ATOM   8883  C   ASN A1136     -28.219  73.375  39.625  1.00 91.62           C
ATOM   8884  CB  ASN A1136     -28.109  71.062  40.594  1.00 91.62           C
ATOM   8885  O   ASN A1136     -29.141  73.750  40.312  1.00 91.62           O
ATOM   8886  CG  ASN A1136     -28.875  70.375  39.500  1.00 91.62           C
ATOM   8887  ND2 ASN A1136     -29.266  69.125  39.719  1.00 91.62           N
ATOM   8888  OD1 ASN A1136     -29.094  71.000  38.438  1.00 91.62           O
ATOM   8889  N   TYR A1137     -27.938  73.750  38.438  1.00 86.88           N
ATOM   8890  CA  TYR A1137     -28.688  74.812  37.781  1.00 86.88           C
ATOM   8891  C   TYR A1137     -29.609  74.312  36.688  1.00 86.88           C
ATOM   8892  CB  TYR A1137     -27.734  75.875  37.188  1.00 86.88           C
```

ATOM	8893	O	TYR	A1137	-29.188	73.500	35.875	1.00	86.88	O
ATOM	8894	CG	TYR	A1137	-26.781	76.438	38.219	1.00	86.88	C
ATOM	8895	CD1	TYR	A1137	-27.219	77.312	39.219	1.00	86.88	C
ATOM	8896	CD2	TYR	A1137	-25.422	76.125	38.156	1.00	86.88	C
ATOM	8897	CE1	TYR	A1137	-26.328	77.812	40.156	1.00	86.88	C
ATOM	8898	CE2	TYR	A1137	-24.516	76.688	39.062	1.00	86.88	C
ATOM	8899	OH	TYR	A1137	-24.109	78.062	40.969	1.00	86.88	O
ATOM	8900	CZ	TYR	A1137	-24.984	77.500	40.062	1.00	86.88	C
ATOM	8901	N	GLY	A1138	-30.969	74.062	37.062	1.00	72.31	N
ATOM	8902	CA	GLY	A1138	-31.984	73.562	36.156	1.00	72.31	C
ATOM	8903	C	GLY	A1138	-31.781	74.000	34.719	1.00	72.31	C
ATOM	8904	O	GLY	A1138	-31.203	75.000	34.469	1.00	72.31	O
ATOM	8905	N	SER	A1139	-31.812	72.938	33.844	1.00	66.12	N
ATOM	8906	CA	SER	A1139	-31.641	73.062	32.406	1.00	66.12	C
ATOM	8907	C	SER	A1139	-32.719	73.938	31.781	1.00	66.12	C
ATOM	8908	CB	SER	A1139	-31.672	71.688	31.750	1.00	66.12	C
ATOM	8909	O	SER	A1139	-32.469	74.625	30.766	1.00	66.12	O
ATOM	8910	OG	SER	A1139	-32.500	70.812	32.469	1.00	66.12	O
ATOM	8911	N	ASP	A1140	-33.844	74.375	32.562	1.00	66.38	N
ATOM	8912	CA	ASP	A1140	-34.938	75.062	31.922	1.00	66.38	C
ATOM	8913	C	ASP	A1140	-35.281	76.375	32.688	1.00	66.38	C
ATOM	8914	CB	ASP	A1140	-36.188	74.188	31.797	1.00	66.38	C
ATOM	8915	O	ASP	A1140	-36.406	76.875	32.594	1.00	66.38	O
ATOM	8916	CG	ASP	A1140	-35.969	73.000	30.922	1.00	66.38	C
ATOM	8917	OD1	ASP	A1140	-35.062	73.000	30.047	1.00	66.38	O
ATOM	8918	OD2	ASP	A1140	-36.688	72.000	31.094	1.00	66.38	O
ATOM	8919	N	GLY	A1141	-34.344	76.875	33.469	1.00	66.50	N
ATOM	8920	CA	GLY	A1141	-34.625	78.125	34.094	1.00	66.50	C
ATOM	8921	C	GLY	A1141	-35.688	78.000	35.188	1.00	66.50	C
ATOM	8922	O	GLY	A1141	-36.312	79.000	35.562	1.00	66.50	O
ATOM	8923	N	GLU	A1142	-36.031	76.812	35.656	1.00	73.44	N
ATOM	8924	CA	GLU	A1142	-37.156	76.562	36.562	1.00	73.44	C
ATOM	8925	C	GLU	A1142	-36.688	76.625	38.000	1.00	73.44	C
ATOM	8926	CB	GLU	A1142	-37.812	75.250	36.250	1.00	73.44	C
ATOM	8927	O	GLU	A1142	-37.500	76.625	38.938	1.00	73.44	O
ATOM	8928	CG	GLU	A1142	-38.594	75.188	34.938	1.00	73.44	C
ATOM	8929	CD	GLU	A1142	-39.375	73.938	34.719	1.00	73.44	C
ATOM	8930	OE1	GLU	A1142	-40.250	73.875	33.844	1.00	73.44	O
ATOM	8931	OE2	GLU	A1142	-39.125	72.938	35.469	1.00	73.44	O
ATOM	8932	N	GLY	A1143	-35.344	76.812	38.250	1.00	81.62	N
ATOM	8933	CA	GLY	A1143	-34.812	76.812	39.594	1.00	81.62	C
ATOM	8934	C	GLY	A1143	-33.469	76.188	39.750	1.00	81.62	C
ATOM	8935	O	GLY	A1143	-32.906	75.688	38.750	1.00	81.62	O
ATOM	8936	N	ALA	A1144	-32.750	76.500	40.812	1.00	89.25	N
ATOM	8937	CA	ALA	A1144	-31.406	76.000	41.094	1.00	89.25	C
ATOM	8938	C	ALA	A1144	-31.297	75.500	42.500	1.00	89.25	C
ATOM	8939	CB	ALA	A1144	-30.391	77.125	40.875	1.00	89.25	C
ATOM	8940	O	ALA	A1144	-32.094	75.875	43.375	1.00	89.25	O
ATOM	8941	N	MET	A1145	-30.453	74.562	42.656	1.00	90.44	N
ATOM	8942	CA	MET	A1145	-30.125	74.000	43.969	1.00	90.44	C
ATOM	8943	C	MET	A1145	-28.625	74.125	44.219	1.00	90.44	C
ATOM	8944	CB	MET	A1145	-30.609	72.562	44.094	1.00	90.44	C
ATOM	8945	O	MET	A1145	-27.812	73.875	43.344	1.00	90.44	O
ATOM	8946	CG	MET	A1145	-30.219	71.938	45.438	1.00	90.44	C
ATOM	8947	SD	MET	A1145	-30.656	70.125	45.469	1.00	90.44	S
ATOM	8948	CE	MET	A1145	-30.031	69.688	47.125	1.00	90.44	C
ATOM	8949	N	LYS	A1146	-28.281	74.562	45.312	1.00	90.19	N
ATOM	8950	CA	LYS	A1146	-26.891	74.625	45.781	1.00	90.19	C
ATOM	8951	C	LYS	A1146	-26.734	73.938	47.125	1.00	90.19	C
ATOM	8952	CB	LYS	A1146	-26.422	76.062	45.844	1.00	90.19	C
ATOM	8953	O	LYS	A1146	-27.531	74.188	48.031	1.00	90.19	O
ATOM	8954	CG	LYS	A1146	-24.969	76.250	46.250	1.00	90.19	C
ATOM	8955	CD	LYS	A1146	-24.562	77.688	46.312	1.00	90.19	C
ATOM	8956	CE	LYS	A1146	-23.156	77.875	46.875	1.00	90.19	C

ATOM	8957	NZ	LYS	A1146	-22.750	79.312	46.969	1.00	90.19	N
ATOM	8958	N	GLN	A1147	-25.781	73.000	47.188	1.00	91.62	N
ATOM	8959	CA	GLN	A1147	-25.438	72.375	48.469	1.00	91.62	C
ATOM	8960	C	GLN	A1147	-23.984	72.625	48.844	1.00	91.62	C
ATOM	8961	CB	GLN	A1147	-25.688	70.875	48.406	1.00	91.62	C
ATOM	8962	O	GLN	A1147	-23.078	72.375	48.062	1.00	91.62	O
ATOM	8963	CG	GLN	A1147	-25.312	70.125	49.656	1.00	91.62	C
ATOM	8964	CD	GLN	A1147	-25.547	68.625	49.562	1.00	91.62	C
ATOM	8965	NE2	GLN	A1147	-24.750	67.875	50.281	1.00	91.62	N
ATOM	8966	OE1	GLN	A1147	-26.438	68.188	48.844	1.00	91.62	O
ATOM	8967	N	THR	A1148	-23.828	73.250	49.875	1.00	89.25	N
ATOM	8968	CA	THR	A1148	-22.516	73.438	50.438	1.00	89.25	C
ATOM	8969	C	THR	A1148	-22.312	72.562	51.656	1.00	89.25	C
ATOM	8970	CB	THR	A1148	-22.281	74.938	50.875	1.00	89.25	C
ATOM	8971	O	THR	A1148	-23.156	71.688	51.906	1.00	89.25	O
ATOM	8972	CG2	THR	A1148	-22.688	75.875	49.781	1.00	89.25	C
ATOM	8973	OG1	THR	A1148	-23.078	75.188	52.062	1.00	89.25	O
ATOM	8974	N	ASN	A1149	-21.266	72.688	52.250	1.00	86.69	N
ATOM	8975	CA	ASN	A1149	-21.000	71.875	53.438	1.00	86.69	C
ATOM	8976	C	ASN	A1149	-21.906	72.250	54.594	1.00	86.69	C
ATOM	8977	CB	ASN	A1149	-19.531	72.000	53.844	1.00	86.69	C
ATOM	8978	O	ASN	A1149	-22.047	71.562	55.562	1.00	86.69	O
ATOM	8979	CG	ASN	A1149	-18.594	71.312	52.875	1.00	86.69	C
ATOM	8980	ND2	ASN	A1149	-17.359	71.812	52.812	1.00	86.69	N
ATOM	8981	OD1	ASN	A1149	-18.969	70.375	52.219	1.00	86.69	O
ATOM	8982	N	THR	A1150	-22.641	73.375	54.469	1.00	88.81	N
ATOM	8983	CA	THR	A1150	-23.406	73.938	55.562	1.00	88.81	C
ATOM	8984	C	THR	A1150	-24.875	74.062	55.219	1.00	88.81	C
ATOM	8985	CB	THR	A1150	-22.844	75.312	56.031	1.00	88.81	C
ATOM	8986	O	THR	A1150	-25.750	74.062	56.062	1.00	88.81	O
ATOM	8987	CG2	THR	A1150	-21.422	75.125	56.562	1.00	88.81	C
ATOM	8988	OG1	THR	A1150	-22.828	76.188	54.906	1.00	88.81	O
ATOM	8989	N	THR	A1151	-25.109	74.250	53.844	1.00	90.38	N
ATOM	8990	CA	THR	A1151	-26.484	74.625	53.500	1.00	90.38	C
ATOM	8991	C	THR	A1151	-26.922	73.875	52.250	1.00	90.38	C
ATOM	8992	CB	THR	A1151	-26.625	76.125	53.312	1.00	90.38	C
ATOM	8993	O	THR	A1151	-26.109	73.562	51.406	1.00	90.38	O
ATOM	8994	CG2	THR	A1151	-26.328	76.875	54.594	1.00	90.38	C
ATOM	8995	OG1	THR	A1151	-25.734	76.562	52.281	1.00	90.38	O
ATOM	8996	N	ILE	A1152	-28.234	73.500	52.250	1.00	92.19	N
ATOM	8997	CA	ILE	A1152	-28.938	73.188	51.031	1.00	92.19	C
ATOM	8998	C	ILE	A1152	-29.953	74.250	50.688	1.00	92.19	C
ATOM	8999	CB	ILE	A1152	-29.641	71.812	51.188	1.00	92.19	C
ATOM	9000	O	ILE	A1152	-30.844	74.562	51.469	1.00	92.19	O
ATOM	9001	CG1	ILE	A1152	-28.609	70.688	51.562	1.00	92.19	C
ATOM	9002	CG2	ILE	A1152	-30.359	71.438	49.875	1.00	92.19	C
ATOM	9003	CD1	ILE	A1152	-29.234	69.375	51.906	1.00	92.19	C
ATOM	9004	N	SER	A1153	-29.734	74.812	49.500	1.00	92.19	N
ATOM	9005	CA	SER	A1153	-30.609	75.938	49.094	1.00	92.19	C
ATOM	9006	C	SER	A1153	-31.234	75.625	47.750	1.00	92.19	C
ATOM	9007	CB	SER	A1153	-29.828	77.250	49.062	1.00	92.19	C
ATOM	9008	O	SER	A1153	-30.594	75.125	46.844	1.00	92.19	O
ATOM	9009	OG	SER	A1153	-29.250	77.500	50.344	1.00	92.19	O
ATOM	9010	N	VAL	A1154	-32.625	75.938	47.656	1.00	91.88	N
ATOM	9011	CA	VAL	A1154	-33.375	75.812	46.438	1.00	91.88	C
ATOM	9012	C	VAL	A1154	-34.031	77.125	46.062	1.00	91.88	C
ATOM	9013	CB	VAL	A1154	-34.438	74.688	46.531	1.00	91.88	C
ATOM	9014	O	VAL	A1154	-34.688	77.750	46.906	1.00	91.88	O
ATOM	9015	CG1	VAL	A1154	-35.219	74.562	45.250	1.00	91.88	C
ATOM	9016	CG2	VAL	A1154	-33.781	73.375	46.938	1.00	91.88	C
ATOM	9017	N	ARG	A1155	-33.625	77.625	44.875	1.00	88.50	N
ATOM	9018	CA	ARG	A1155	-34.344	78.812	44.344	1.00	88.50	C
ATOM	9019	C	ARG	A1155	-35.219	78.438	43.156	1.00	88.50	C
ATOM	9020	CB	ARG	A1155	-33.375	79.875	44.000	1.00	88.50	C

ATOM	9021	O	ARG	A1155	-34.938	77.438	42.438	1.00	88.50	O
ATOM	9022	CG	ARG	A1155	-32.500	79.562	42.781	1.00	88.50	C
ATOM	9023	CD	ARG	A1155	-31.625	80.750	42.375	1.00	88.50	C
ATOM	9024	NE	ARG	A1155	-30.781	80.438	41.250	1.00	88.50	N
ATOM	9025	NH1	ARG	A1155	-30.719	82.625	40.438	1.00	88.50	N
ATOM	9026	NH2	ARG	A1155	-29.594	80.938	39.344	1.00	88.50	N
ATOM	9027	CZ	ARG	A1155	-30.359	81.312	40.344	1.00	88.50	C
ATOM	9028	N	ASP	A1156	-36.406	79.062	43.094	1.00	86.88	N
ATOM	9029	CA	ASP	A1156	-37.281	78.812	41.969	1.00	86.88	C
ATOM	9030	C	ASP	A1156	-36.812	79.625	40.750	1.00	86.88	C
ATOM	9031	CB	ASP	A1156	-38.719	79.250	42.344	1.00	86.88	C
ATOM	9032	O	ASP	A1156	-35.750	80.188	40.750	1.00	86.88	O
ATOM	9033	CG	ASP	A1156	-38.875	80.750	42.594	1.00	86.88	C
ATOM	9034	OD1	ASP	A1156	-38.000	81.500	42.156	1.00	86.88	O
ATOM	9035	OD2	ASP	A1156	-39.875	81.125	43.219	1.00	86.88	O
ATOM	9036	N	ALA	A1157	-37.625	79.625	39.688	1.00	83.56	N
ATOM	9037	CA	ALA	A1157	-37.312	80.188	38.375	1.00	83.56	C
ATOM	9038	C	ALA	A1157	-37.188	81.688	38.469	1.00	83.56	C
ATOM	9039	CB	ALA	A1157	-38.406	79.812	37.375	1.00	83.56	C
ATOM	9040	O	ALA	A1157	-36.562	82.312	37.625	1.00	83.56	O
ATOM	9041	N	SER	A1158	-37.844	82.312	39.500	1.00	83.75	N
ATOM	9042	CA	SER	A1158	-37.812	83.750	39.688	1.00	83.75	C
ATOM	9043	C	SER	A1158	-36.625	84.188	40.531	1.00	83.75	C
ATOM	9044	CB	SER	A1158	-39.094	84.250	40.312	1.00	83.75	C
ATOM	9045	O	SER	A1158	-36.469	85.375	40.875	1.00	83.75	O
ATOM	9046	OG	SER	A1158	-40.219	83.938	39.500	1.00	83.75	O
ATOM	9047	N	GLY	A1159	-35.688	83.188	40.844	1.00	82.75	N
ATOM	9048	CA	GLY	A1159	-34.531	83.500	41.688	1.00	82.75	C
ATOM	9049	C	GLY	A1159	-34.844	83.562	43.156	1.00	82.75	C
ATOM	9050	O	GLY	A1159	-34.000	83.938	43.969	1.00	82.75	O
ATOM	9051	N	ARG	A1160	-36.125	83.250	43.562	1.00	85.38	N
ATOM	9052	CA	ARG	A1160	-36.562	83.312	44.938	1.00	85.38	C
ATOM	9053	C	ARG	A1160	-36.219	82.000	45.688	1.00	85.38	C
ATOM	9054	CB	ARG	A1160	-38.062	83.562	45.031	1.00	85.38	C
ATOM	9055	O	ARG	A1160	-36.531	80.938	45.219	1.00	85.38	O
ATOM	9056	CG	ARG	A1160	-38.469	85.000	44.625	1.00	85.38	C
ATOM	9057	CD	ARG	A1160	-39.969	85.250	44.781	1.00	85.38	C
ATOM	9058	NE	ARG	A1160	-40.344	86.625	44.375	1.00	85.38	N
ATOM	9059	NH1	ARG	A1160	-42.594	86.188	44.375	1.00	85.38	N
ATOM	9060	NH2	ARG	A1160	-41.812	88.250	43.844	1.00	85.38	N
ATOM	9061	CZ	ARG	A1160	-41.562	87.000	44.219	1.00	85.38	C
ATOM	9062	N	LEU	A1161	-35.500	82.188	46.875	1.00	89.88	N
ATOM	9063	CA	LEU	A1161	-35.188	81.125	47.750	1.00	89.88	C
ATOM	9064	C	LEU	A1161	-36.469	80.438	48.250	1.00	89.88	C
ATOM	9065	CB	LEU	A1161	-34.344	81.562	48.938	1.00	89.88	C
ATOM	9066	O	LEU	A1161	-37.312	81.125	48.844	1.00	89.88	O
ATOM	9067	CG	LEU	A1161	-33.875	80.438	49.906	1.00	89.88	C
ATOM	9068	CD1	LEU	A1161	-32.812	79.562	49.219	1.00	89.88	C
ATOM	9069	CD2	LEU	A1161	-33.281	81.062	51.156	1.00	89.88	C
ATOM	9070	N	ARG	A1162	-36.688	79.125	47.938	1.00	89.88	N
ATOM	9071	CA	ARG	A1162	-37.906	78.438	48.344	1.00	89.88	C
ATOM	9072	C	ARG	A1162	-37.625	77.500	49.531	1.00	89.88	C
ATOM	9073	CB	ARG	A1162	-38.469	77.625	47.156	1.00	89.88	C
ATOM	9074	O	ARG	A1162	-38.500	77.312	50.375	1.00	89.88	O
ATOM	9075	CG	ARG	A1162	-38.812	78.500	45.938	1.00	89.88	C
ATOM	9076	CD	ARG	A1162	-39.906	79.500	46.250	1.00	89.88	C
ATOM	9077	NE	ARG	A1162	-41.250	78.875	46.219	1.00	89.88	N
ATOM	9078	NH1	ARG	A1162	-42.375	80.875	46.000	1.00	89.88	N
ATOM	9079	NH2	ARG	A1162	-43.531	78.938	46.062	1.00	89.88	N
ATOM	9080	CZ	ARG	A1162	-42.375	79.562	46.094	1.00	89.88	C
ATOM	9081	N	VAL	A1163	-36.438	77.062	49.531	1.00	89.38	N
ATOM	9082	CA	VAL	A1163	-36.094	76.125	50.562	1.00	89.38	C
ATOM	9083	C	VAL	A1163	-34.625	76.250	50.938	1.00	89.38	C
ATOM	9084	CB	VAL	A1163	-36.375	74.688	50.125	1.00	89.38	C

ATOM	9085	O	VAL	A1163	-33.750	76.375	50.094	1.00	89.38	O
ATOM	9086	CG1	VAL	A1163	-35.938	73.688	51.219	1.00	89.38	C
ATOM	9087	CG2	VAL	A1163	-37.875	74.500	49.812	1.00	89.38	C
ATOM	9088	N	GLN	A1164	-34.312	76.500	52.188	1.00	92.12	N
ATOM	9089	CA	GLN	A1164	-32.969	76.438	52.719	1.00	92.12	C
ATOM	9090	C	GLN	A1164	-32.938	75.562	53.969	1.00	92.12	C
ATOM	9091	CB	GLN	A1164	-32.469	77.875	53.094	1.00	92.12	C
ATOM	9092	O	GLN	A1164	-33.688	75.750	54.906	1.00	92.12	O
ATOM	9093	CG	GLN	A1164	-30.953	77.938	53.312	1.00	92.12	C
ATOM	9094	CD	GLN	A1164	-30.453	79.375	53.469	1.00	92.12	C
ATOM	9095	NE2	GLN	A1164	-29.188	79.562	53.125	1.00	92.12	N
ATOM	9096	OE1	GLN	A1164	-31.188	80.250	53.938	1.00	92.12	O
ATOM	9097	N	ILE	A1165	-32.156	74.500	53.812	1.00	88.06	N
ATOM	9098	CA	ILE	A1165	-31.922	73.500	54.875	1.00	88.06	C
ATOM	9099	C	ILE	A1165	-30.484	73.625	55.344	1.00	88.06	C
ATOM	9100	CB	ILE	A1165	-32.250	72.125	54.375	1.00	88.06	C
ATOM	9101	O	ILE	A1165	-29.547	73.688	54.562	1.00	88.06	O
ATOM	9102	CG1	ILE	A1165	-33.656	72.062	53.750	1.00	88.06	C
ATOM	9103	CG2	ILE	A1165	-32.094	71.062	55.531	1.00	88.06	C
ATOM	9104	CD1	ILE	A1165	-33.969	70.688	53.125	1.00	88.06	C
ATOM	9105	N	GLY	A1166	-30.297	73.688	56.781	1.00	88.50	N
ATOM	9106	CA	GLY	A1166	-29.031	74.000	57.469	1.00	88.50	C
ATOM	9107	C	GLY	A1166	-28.969	75.375	58.031	1.00	88.50	C
ATOM	9108	O	GLY	A1166	-29.891	75.812	58.750	1.00	88.50	O
ATOM	9109	N	ARG	A1167	-27.859	76.000	57.844	1.00	85.31	N
ATOM	9110	CA	ARG	A1167	-27.734	77.438	58.219	1.00	85.31	C
ATOM	9111	C	ARG	A1167	-28.594	78.312	57.344	1.00	85.31	C
ATOM	9112	CB	ARG	A1167	-26.266	77.875	58.156	1.00	85.31	C
ATOM	9113	O	ARG	A1167	-28.375	78.438	56.156	1.00	85.31	O
ATOM	9114	CG	ARG	A1167	-26.016	79.312	58.656	1.00	85.31	C
ATOM	9115	CD	ARG	A1167	-24.547	79.625	58.656	1.00	85.31	C
ATOM	9116	NE	ARG	A1167	-24.312	81.000	59.062	1.00	85.31	N
ATOM	9117	NH1	ARG	A1167	-22.016	80.875	58.969	1.00	85.31	N
ATOM	9118	NH2	ARG	A1167	-23.031	82.812	59.594	1.00	85.31	N
ATOM	9119	CZ	ARG	A1167	-23.109	81.562	59.219	1.00	85.31	C
ATOM	9120	N	LEU	A1168	-29.672	78.812	57.969	1.00	85.38	N
ATOM	9121	CA	LEU	A1168	-30.672	79.625	57.250	1.00	85.38	C
ATOM	9122	C	LEU	A1168	-30.188	81.062	57.094	1.00	85.38	C
ATOM	9123	CB	LEU	A1168	-32.000	79.562	57.969	1.00	85.38	C
ATOM	9124	O	LEU	A1168	-30.109	81.812	58.062	1.00	85.38	O
ATOM	9125	CG	LEU	A1168	-32.625	78.188	58.188	1.00	85.38	C
ATOM	9126	CD1	LEU	A1168	-33.906	78.312	59.031	1.00	85.38	C
ATOM	9127	CD2	LEU	A1168	-32.938	77.500	56.844	1.00	85.38	C
ATOM	9128	N	THR	A1169	-29.703	81.375	55.938	1.00	84.62	N
ATOM	9129	CA	THR	A1169	-29.203	82.688	55.656	1.00	84.62	C
ATOM	9130	C	THR	A1169	-30.281	83.562	55.000	1.00	84.62	C
ATOM	9131	CB	THR	A1169	-27.953	82.688	54.781	1.00	84.62	C
ATOM	9132	O	THR	A1169	-30.203	84.812	55.031	1.00	84.62	O
ATOM	9133	CG2	THR	A1169	-26.828	81.875	55.438	1.00	84.62	C
ATOM	9134	OG1	THR	A1169	-28.266	82.062	53.531	1.00	84.62	O
ATOM	9135	N	GLY	A1170	-31.234	82.875	54.406	1.00	80.94	N
ATOM	9136	CA	GLY	A1170	-32.281	83.562	53.688	1.00	80.94	C
ATOM	9137	C	GLY	A1170	-31.859	84.062	52.312	1.00	80.94	C
ATOM	9138	O	GLY	A1170	-32.625	84.750	51.625	1.00	80.94	O
ATOM	9139	N	SER	A1171	-30.594	83.750	51.906	1.00	78.75	N
ATOM	9140	CA	SER	A1171	-30.094	84.188	50.594	1.00	78.75	C
ATOM	9141	C	SER	A1171	-29.547	83.000	49.812	1.00	78.75	C
ATOM	9142	CB	SER	A1171	-29.031	85.250	50.750	1.00	78.75	C
ATOM	9143	O	SER	A1171	-28.938	82.125	50.375	1.00	78.75	O
ATOM	9144	OG	SER	A1171	-27.953	84.812	51.562	1.00	78.75	O
ATOM	9145	N	TRP	A1172	-30.141	82.875	48.500	1.00	77.31	N
ATOM	9146	CA	TRP	A1172	-29.469	82.000	47.562	1.00	77.31	C
ATOM	9147	C	TRP	A1172	-27.969	82.312	47.500	1.00	77.31	C
ATOM	9148	CB	TRP	A1172	-30.078	82.062	46.188	1.00	77.31	C

```
ATOM   9149  O   TRP A1172     -27.578  83.438  47.438  1.00 77.31           O
ATOM   9150  CG  TRP A1172     -29.406  81.188  45.156  1.00 77.31           C
ATOM   9151  CD1 TRP A1172     -28.547  81.625  44.156  1.00 77.31           C
ATOM   9152  CD2 TRP A1172     -29.516  79.750  45.031  1.00 77.31           C
ATOM   9153  CE2 TRP A1172     -28.703  79.375  43.938  1.00 77.31           C
ATOM   9154  CE3 TRP A1172     -30.219  78.812  45.750  1.00 77.31           C
ATOM   9155  NE1 TRP A1172     -28.141  80.500  43.438  1.00 77.31           N
ATOM   9156  CH2 TRP A1172     -29.281  77.125  44.250  1.00 77.31           C
ATOM   9157  CZ2 TRP A1172     -28.594  78.062  43.531  1.00 77.31           C
ATOM   9158  CZ3 TRP A1172     -30.094  77.500  45.344  1.00 77.31           C
TER    9159      TRP A1172
ENDMDL
END
```
